# Hippocampal Transcriptomic Profiles: Subfield Vulnerability to Age and Cognitive Impairment

**DOI:** 10.3389/fnagi.2017.00383

**Published:** 2017-12-08

**Authors:** Lara Ianov, Matt De Both, Monica K. Chawla, Asha Rani, Andrew J. Kennedy, Ignazio Piras, Jeremy J. Day, Ashley Siniard, Ashok Kumar, J. David Sweatt, Carol A. Barnes, Matthew J. Huentelman, Thomas C. Foster

**Affiliations:** ^1^Departments of Neuroscience and Genetics and Genomics Program, Evelyn F. and William L. McKnight Brain Institute, University of Florida, Gainesville, FL, United States; ^2^Civitan International Research Center, University of Alabama at Birmingham, Birmingham, AL, United States; ^3^Neurogenomics Division, Translational Genomics Research Institute, Phoenix, AZ, United States; ^4^Evelyn F. McKnight Brain Institute, University of Arizona, Tucson, AZ, United States; ^5^Evelyn F. McKnight Brain Institute, University of Alabama, Birmingham, AL, United States; ^6^Department of Pharmacology, Vanderbilt University, Nashville, TN, United States; ^7^Departments of Psychology, Neurology and Neuroscience, University of Arizona, Tucson, AZ, United States

**Keywords:** aging, hippocampus, cognitive function, transcription, gene expression, Illumina HiSeq, Ion proton

## Abstract

The current study employed next-generation RNA sequencing to examine gene expression differences related to brain aging, cognitive decline, and hippocampal subfields. Young and aged rats were trained on a spatial episodic memory task. Hippocampal regions CA1, CA3, and the dentate gyrus were isolated. Poly-A mRNA was examined using two different sequencing platforms, Illumina, and Ion Proton. The Illumina platform was used to generate seed lists of genes that were statistically differentially expressed across regions, ages, or in association with cognitive function. The gene lists were then retested using the data from the Ion Proton platform. The results indicate hippocampal subfield differences in gene expression and point to regional differences in vulnerability to aging. Aging was associated with increased expression of immune response-related genes, particularly in the dentate gyrus. For the memory task, impaired performance of aged animals was linked to the regulation of Ca^2+^ and synaptic function in region CA1. Finally, we provide a transcriptomic characterization of the three subfields regardless of age or cognitive status, highlighting and confirming a correspondence between cytoarchitectural boundaries and molecular profiling.

## Introduction

Regional variation in brain aging may explain differential susceptibility to impairment in specific cognitive domains over the course of aging. Deficits in hippocampal-dependent episodic memory emerges in middle-age, and the degree or propensity for impairment increases with advancing age in humans and animal models (Uttl and Graf, 1993; Ronnlund et al., 2005; Cansino, 2009; Foster, 2012). There has been a notable increase in recent efforts to accurately identify boundaries that define distinct hippocampal subfields in humans (Yushkevich et al., 2015; Wisse et al., 2016; Berron et al., 2017) and to link behavioral impairments in older individuals to subfield-specific hippocampal volume or activity changes (Mueller and Weiner, 2009; Yassa et al., 2011; Daugherty et al., 2016). Computational approaches, as well as research from brain damaged humans and animal models, suggest that the different subfields of the hippocampus (CA1, CA3, and dentate gyrus-DG) contribute to various aspects of episodic memory (Rolls and Kesner, 2006; Goodrich-Hunsaker et al., 2008; Maass et al., 2014; Wang and Diana, 2016). This includes pattern separation and completion mechanisms, rapid acquisition, and retention across different time scales. In addition, each region may employ different mechanisms to modulate the strength of their synaptic communication to alter connectivity during the formation of episodic memories. Furthermore, the three regions exhibit differential vulnerability to the aging process (Jackson et al., 2009; Wang et al., 2009; Zeier et al., 2011; Chapman et al., 2012). To address the complexity of the molecular mechanisms linked to age-related changes in hippocampal-dependent cognition, it is necessary to consider the variability in region-specific responses to aging and memory mechanisms.

The current study investigated changes in gene expression within each of the three hippocampal regions in relation to both aging and to cognitive decline in aging. Previous work suggests that brain aging is associated with altered genes and proteins linked to biological processes for cell maintenance and repair, reactions to oxidative stress, neuroinflammation, and neuron-specific signaling, including cell excitability and synaptic plasticity (Blalock et al., 2003; Lu et al., 2004; Fraser et al., 2005; Wang et al., 2009; Burger, 2010; Zeier et al., 2011; Cribbs et al., 2012; Haberman et al., 2013; Masser et al., 2014). It is unclear; however, which biomarkers may represent causative factors for cognitive decline, compensation to maintain cognitive function, or epiphenomenon associated with the aging process (Gray and Barnes, 2015). For example, studies that examine gene expression soon after behavioral testing indicate a decrease in expression of neural activity-related genes; however, it is unclear if altered expression represents the cause of the age-related deficit, or whether the genes are not expressed because learning did not occur (Rowe et al., 2007; Penner et al., 2011; Chawla et al., 2013). Similarly, brain aging can be characterized by an increase in molecular markers of oxidative stress and neuroinflammation (Foster, 2006; Droge and Schipper, 2007; Craft et al., 2012) and it is unclear whether such changes represent precursors to cognitive decline, age-related factors independent of cognition, neuroprotective elements in cognitively unimpaired individuals, or compensation due to changes in afferent connections.

Next-generation sequencing technology is a powerful tool for examining complex processes by monitoring the parallel expression of tens of thousands of genes. Along with the advantage of examining large numbers of transcripts, comes the necessity for validation of the differentially expressed genes. Normally this would occur using a different technique, such as qRT-PCR. We have taken advantage of two different next-generation platforms to confirm differential expression. Our study was designed to increase experimental robustness by utilizing two different laboratories to perform the behavioral characterization of the animals and by using two different next-generation sequencing chemistries on the same RNA samples to minimize false positives and negatives in a staged discovery/validation approach. In this case, transcriptome analysis of the same tissue was performed using Illumina fluorescent labeling and Ion Proton semiconductor measures of proton release during sequencing-by-synthesis. Each technology has pros and cons (Jessri and Farah, 2014); however, previous reports indicate a good concordance in the accuracy of expression profiling between the different platforms (Kusko et al., 2014; Li et al., 2015; Reuter et al., 2015). This study was designed to lead to a high probability of independent validation in other laboratories.

## Methods

### Animals

Procedures involving animal subjects have been reviewed and approved by the Institutional Animal Care and Use Committee at the University of Arizona (AZ) and University of Florida (FL) and were in accordance with guidelines established by the U.S. Public Health Service Policy on Humane Care and Use of Laboratory. Male Fischer 344 rats of two ages, young (5–6 months, total *n* = 10; *n* = 5 AZ, *n* = 5 FL) and aged (17–22 months, total *n* = 24; *n* = 11 AZ, *n* = 13 FL) were obtained from National Institute on Aging's colonies (Taconic, FL; Charles River, AZ). Animals were maintained on a 12:12 h light/dark schedule, and provided *ad libitum* access to food and water. Animal characterization was performed at two sites—University of Arizona and University of Florida—and the resulting data were combined and analyzed as a single cohort.

### Morris water maze

Procedures for the water maze have previously been published (Foster et al., 1991; Bean et al., 2015). Animals were trained in a tank, 1.7 m (FL), 1.8 m (AZ) in diameter, positioned in room that provided cues for the animals. Water (27 ± 2°C) was maintained at a level approximately 8 cm below the surface of the tank. For cue and spatial tasks, training consisted of five blocks with three trials per block and training on each task was massed into a single day. Inter-trial intervals were 20 s and inter-block intervals were ~15 min. Rats remained on the platform between trials and in home cages under the heat lamp after each block. Behavioral data were acquired with either Noldus EthoVision computer tracking software (Noldus Information Technology, (Leesburg, VA) in FL or AnyMaze (Wood Dale, IL) in AZ and included path-length and time in the goal and opposite quadrants.

Rats were first trained on the cue discrimination version of the water escape task. The escape platform was extended approximately 1 cm above the water level and its location was signaled by a visual cue. For each trial, the platform position and start location were randomized. If an animal did not escape the water maze within 60 s, the rat was gently guided to the platform. Three days following cue training, animals were trained on the spatial discrimination task. For spatial discrimination, the escape platform was hidden approximately 1.5 cm beneath the water level and remained in the same location relative to the distal cues in the room for the duration of the initial spatial training. Fifteen minutes following the end of training on block 5, a free-swim probe trial was administered as a measure of learning. For the probe trial, the platform was removed and the animal placed in the tank for 60 s. A spatial discrimination index was computed according to the formula (G − O)/(G + O) where G and O represent the percent of time spent in the goal quadrant and quadrant opposite the goal, respectively.

### Statistical analysis of behavior

The mean distance to find the platform during the first training block was used as a baseline for the water maze cue and spatial tasks and the mean percent change from the baseline was calculated for each subsequent block. Repeated measures analyses of variance (ANOVAs) were used to examine age and training effects over blocks of trials. One-way ANOVAs were used to examine age effects for the water maze probe trial discrimination index. Fisher's protected least significant difference comparisons, with the *p*-value set at 0.05, were used as *post hoc* tests to localize differences. Pearson's correlations were calculated between the distance measures for the last block of cue and spatial discrimination trials in order to determine if differences in sensory-motor function or procedural learning contributed to spatial learning. Correlations were limited to within each age group.

### Tissue collection

Two weeks following water maze testing, rats were briefly anesthetized using isoflurane (Piramal Healthcare) and then quickly decapitated. The brains were rapidly removed, briefly rinsed in sterile saline, and the hippocampus was dissected out using a microspatula, razor blade, and surgical scissors. Methods for dissection of hippocampal subfields was adapted from previous work, which enabled isolation of CA1, CA3, and the dentate gyrus (DG) along the rostral and ventral hippocampus (Lein et al., 2004; Zeier et al., 2011). The hippocampus was placed ventral side up on a microdissection tray on ice, the hippocampal fissure was identified, and the dentate gyrus (DG) was dissected free of the CA and subicular fields by gently teasing it away along the hippocampal fissure. Further dissection along the margin of the free blade produced a block consisting of subiculum/cortex, CA1 and most of CA3. The subiculum was separated from CA regions, which was then further cut into CA1 and CA3 subfields. The tissues (CA1, CA3, and DG) were quickly frozen in liquid nitrogen then stored at −80°C. The tissue for one hippocampus from each animal was used for RNA isolation. Due to the dissection along the length of the hippocampus, other subfields including region CA2 and the subiculum were not isolated.

### RNA isolation

The tissue was sent to University of Alabama at Birmingham for RNA isolation. Total RNA was extracted (Qiagen, miRNeasy #217004), DNase-treated (Qiagen), and quality was assessed (Bioanalyzer, Agilent), which indicated a RNA integrity of 8.83 ± 0.04 (mean ± standard error) for the cohort.

### Ion proton sequencing and analysis

Sequencing and analysis by using Ion Proton was performed at the University of Florida. Poly-A selection for the Ion Proton sequencer was performed with 250 ng of total RNA using the Dynabeads mRNA DIRECT Micro kit (Thermo Fisher, catalog number 61021) followed by library preparations with the Ion Total RNA-seq Kit v2 (Thermo Fisher, catalog number 4475936) with the addition of the Ion Xpress barcodes for multiplex sequencing (Thermo Fisher, catalog number 4475485). In brief, poly-A selection was performed by the base pairing of the poly-A tail of mRNA to the oligo (dT)_25_ sequence of the magnetic Dynabeads. Further, the mRNA was enzymatically fragmented by RNAse III, purified, ligated to the Ion adaptor mix, and reverse transcribed. The cDNA was uniquely barcoded per biological replicate and amplified with 16 cycles of PCR. The concentration of the libraries was quantified by the Qubit dsDNA HS Assay (Thermo Fisher, catalog number Q32851), and size distribution was evaluated with the High Sensitivity D1000 ScreenTape in the 2200 Tapestation system (Agilent Technologies).

Template preparation was performed in the Ion Chef system and sequencing was completed in the Ion Proton (Thermo Fisher). Low quality reads were removed from the FASTQ files resulting in reads containing an average length of 134 bp. The Ion Proton data were aligned to the *rattus norvegicus* (rn5) genome using the two step alignment method with TopHat2 and Bowtie2 in the Partek Flow servers (Partek, Inc.). Aligned reads were summarized as gene-level counts (featureCounts 1.4.4). The FASTQ files from the Ion Proton have been submitted to NCBI's Gene Expression Omnibus (GEO) under the accession number: GSE97608.

### Illumina HiSeq sequencing and analysis

Sequencing and analysis by using Illumina HiSeq was performed at the Translational Genomics Research Institute, Arizona. Sequencing libraries were prepared with 250 ng of total RNA using Illumina's Truseq RNA Sample Preparation Kit v2 (Illumina, Inc.) following the manufacturer's protocol. In brief, poly-A containing mRNA molecules were purified using poly-T oligo attached magnetic beads. The mRNA was then thermally fragmented and converted to double-stranded cDNA. The cDNA fragments were end-repaired, a single “A” nucleotide was incorporated, sequencing adapters were ligated, and fragments were enriched with 15 cycles of PCR. Final PCR-enriched fragments were validated on a 2200 TapeStation (Agilent Technologies) and quantitated via qPCR using Kapa's Library Quantification Kit (Kapa Biosystems) on the QuantStudio 6 Flex Instrument (ThermoFisher). The final library was sequenced by 50 bp paired-end sequencing on a HiSeq 2500 (Illumina). Illumina BCL files were converted and demultiplexed (bcl2fastq 2.17). FASTQ files were trimmed of adapter sequences (CutAdapt 1.8.3) and aligned to rn5 (STAR 2.5). Aligned reads were summarized as gene-level counts (featureCounts 1.4.4). Sequencing and quality control reports were generated (FastQC 0.11.4 and Qualimap 2.1.3). The FASTQ files from the Illumina platform have been separately submitted to GEO under the accession number: GSE101798.

### Analysis

For poly-A mRNA gene expression sequenced by Illumina and Ion Proton, pairwise differential expression analysis was conducted with the R package *DESeq2* (1.10.1) to identify transcriptional changes due to age, cognitive performance, or subfield. For the transcriptional changes due to age and cognitive performance, a significant cut-off was set at *p* < 0.01. The resulting data sets for each region represent seed lists of differentially expressed genes. Validation of expression was performed using poly-A mRNA sequenced on the Ion Proton. For Ion Proton mRNA, a significance cut-off was set at *p* < 0.05 for one tailed-tests, with the tail specified by the direction of fold change (FC) determined by the Illumina seed list. Thus, the adjusted *p*-value for the combined tests was *p* < 0.0005. The following formula was used to calculate the false discovery rate (FDR) = (number of genes identified by Illumina ^*^ 0.0005/the number genes from this list identified by Ion Proton). Heat maps were generated in R with “gplots” (3.0.1) and “ComplexHeatmap” (1.14.0) using counts which were standardized to *z*-scores from genes validated with the Ion Proton, and the box plots were generated with “ggplot2” (2.2.1).

To be labeled subfield-specific in CA1, CA3, or DG, gene counts had to be significantly different (Benjamini-Hochberg adj- *p* < 0.05) from both of the other two subfields with a concordant direction of fold change and validated by Ion Proton sequencing at the same significance threshold. Finally, pathway analysis was conducted with the ToppGene web portal against KEGG, PantherDB, and Reactome databases. An additional analysis was conducted using DAVID (version 6.8), considering Gene Ontology for biological processes and cellular components in the “Direct” and “FAT” categories with the Benjamini FDR set at *p* < 0.05 as a cut-off for cluster selection.

## Results

### Behavior

#### Cue discrimination

**Figure 1:**
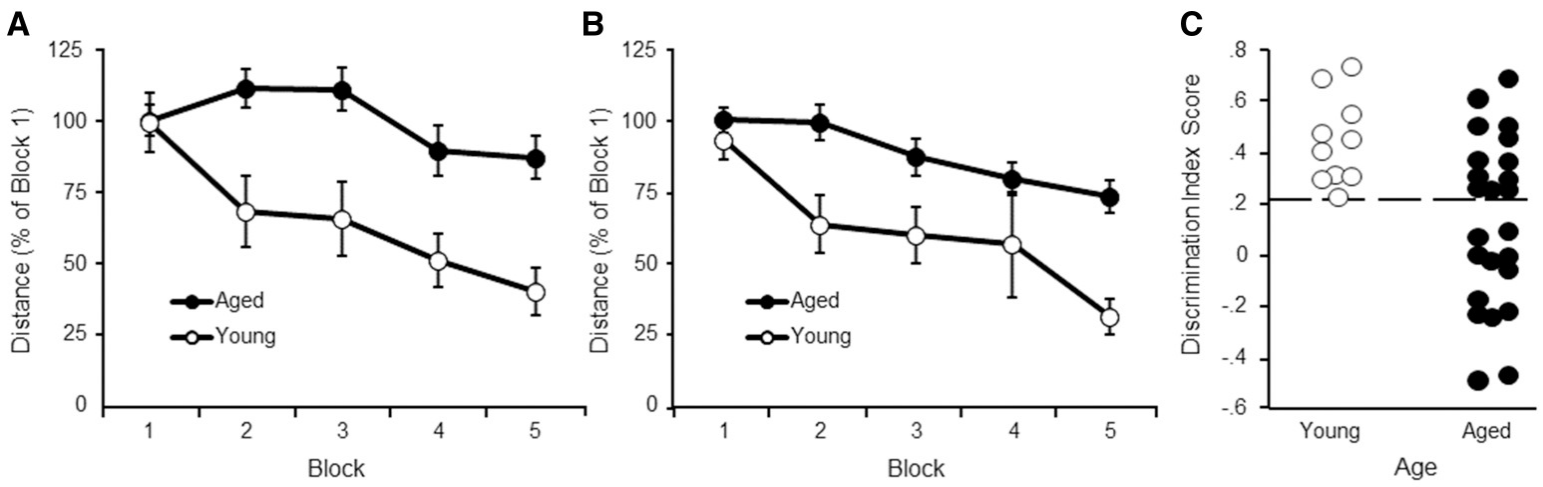
Performance on the water maze task. Symbols indicate the mean (±SEM) escape path length to the escape platform during 5 training blocks on the **(A)** cue and **(B)** spatial discrimination tasks for young (open symbols) and aged (filled symbols) animals. **(C)** Acquisition of a spatial search strategy. Individual discrimination index scores for young (open symbols) and aged (filled symbols) animals. The dashed line indicates the cut-off for behavioral classification of aged-impaired (AI) and aged-unimpaired (AU) animals.

A repeated measures ANOVA indicated an effect of training [*F*_(4, 128)_ = 5.81, *p* < 0.0005] and age [*F*_(1, 32)_ = 11.57, *p* < 0.005], and an interaction [*F*_(4, 128)_ = 2.46, *p* < 0.05] (Figure 1A). Despite the age difference, all animals were able to locate the platform by the end of training and an ANOVA in each age group confirmed decreased path length with training (*p* < 0.05). *Post hoc* analysis indicated that young rats exhibited a rapid rate of learning observed as a decrease in escape path length for blocks 2–5 relative to block 1. For aged animals, learning was slower, such that escape path length decreased on blocks 4–5 relative to blocks 2–3.

#### Spatial discrimination

A repeated measures ANOVA on performance in the spatial task indicated an effect of training [*F*_(4, 128)_ = 11.28, *p* < 0.0001] and age [*F*_(1, 32)_ = 16.81, *p* < 0.001], in the absence of an interaction (Figure 1B). An ANOVA in each age group confirmed decreased path length with training (*p* < 0.005), indicating that both groups exhibited improved performance with training. *Post hoc* analysis indicated that young rats exhibited a rapid rate of learning observed as a decrease in escape path length for blocks 2–5 relative to block 1. For aged animals, learning was observed as a decrease in escape path length for blocks 4–5 relative to blocks 1–2.

Regression analysis performed within each age group indicated no relationship between performance on the cue task (block 5 distance) and the spatial task (block 5 distance) (young: *r* = 0.43, *p* = 0.22; aged: *r* = 0.078, *p* = 0.72). The results indicate that age differences in sensory-motor function or acquisition of the procedural aspects of the task did not mediate impairments in acquisition of a spatial search strategy.

#### Probe trial discrimination index (DI) scores

An ANOVA on the discrimination index scores using age and testing site as factors indicated an age difference [*F*_(1, 30)_ = 8.41, *p* < 0.01] in the absence of a training site difference or interaction (Figure 1C). As expected, from the age range of 17–22 months in this strain of rat, there was considerable variability for aged animals, with some animals performing as well as young.

The observed lowest DI score for young animals was 0.24. This value was used as cut-off to classify aged animals as aged-impaired (AI) (DI < 0.24) or aged-unimpaired (AU; DI > 0.24). A repeated measures ANOVA was computed comparing AI and AU groups for the cue task. No group difference was observed for distance to escape to the platform on the cue task (*p* = 0.82). Thus, the difference for the DI scores is not likely due to sensory-motor function or learning the procedural aspects of the task.

### Gene expression across regions: comparison of illumina and ion proton technologies

#### Sequence quality metrics

We sequenced a total of 17.1 and 18.9 million reads per sample, the total mapping rate was 94.1 and 96.8%, with exonic mapping 63.8 and 58.0%, intronic 15.2 and 13.1% and the percentage of intergenic reads was 21.0 and 28.9% on the Illumina and Ion Proton platforms, respectively.

**Figure 2:**
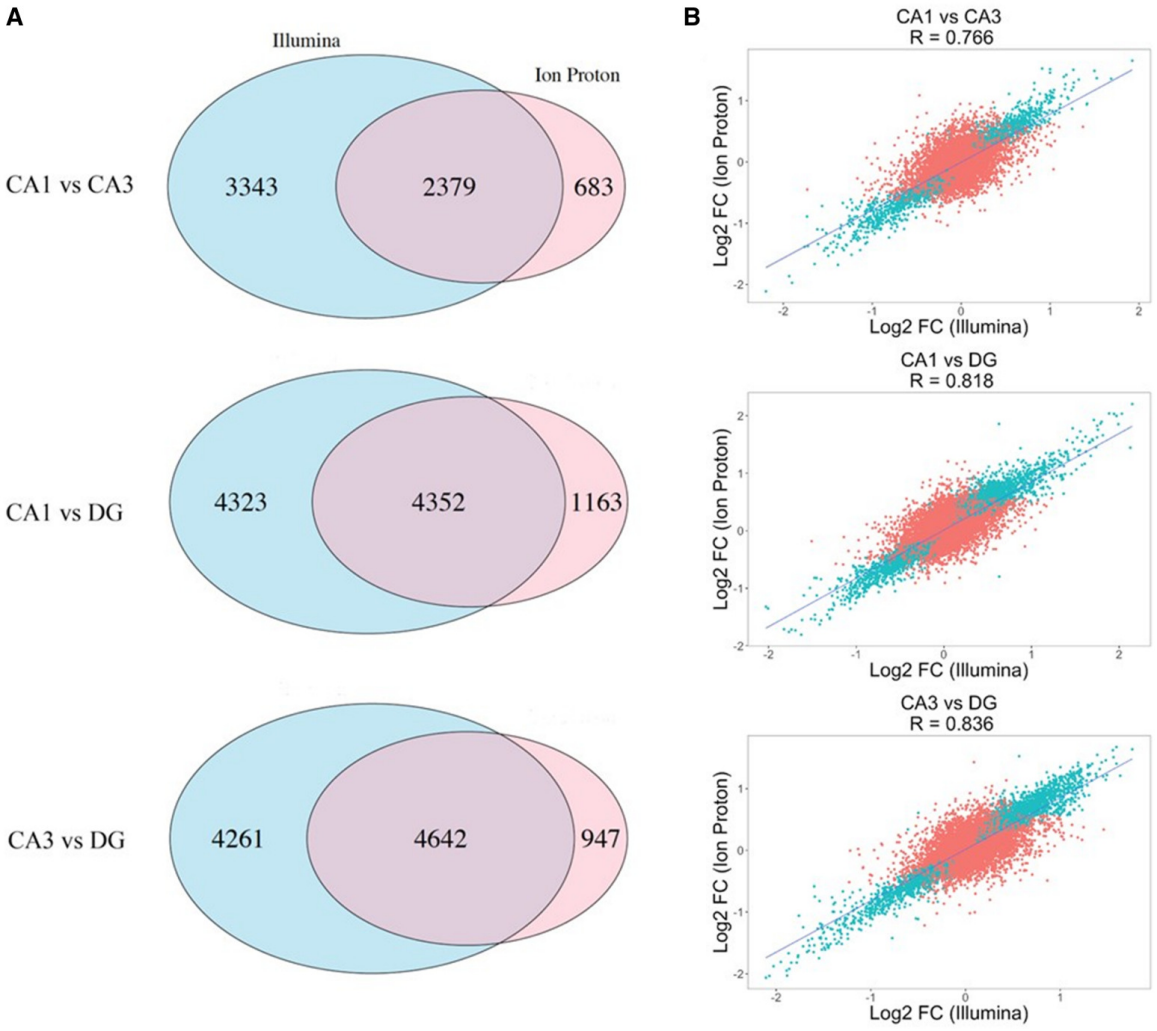
Differential expression across the Illumina and Ion Proton platforms. **(A)** Proportional Venn diagrams showing the concordance for genes that were significant after *p*-value adjustment for the Illumina and Ion Proton in each pairwise comparison indicated (CA1 vs. CA3; CA1 vs. DG; CA3 vs. DG). For example, total number of significant genes for CA1 vs. CA3 were 5722 and 3062 for Illumina and Ion Proton, respectively, with an overlap of 2379. **(B)** Scatterplot of the fold changes for all detected genes across both platforms. Blue points are the genes differentially expressed in Illumina and Ion Proton (adj-*p* < 0.05).

The correlation of number of counts for the common genes is shown in Figure S1. We conducted pairwise differential expression analysis between the three subfields regardless of age or cognitive status. For each pairwise comparison, we retained the differentially expressed genes (DEGs) that were statistically significant with both platforms (adj-*p* < 0.05) and concordant for FC direction. A summary of the number of genes detected in all the pairwise comparisons in both platforms is reported in Table 1. A total of 24 DEGs were discordant for FC between the two platforms in the 3 comparisons (specifically: 5 for CA1 vs. CA3, 14 for CA1 vs. DG and 5 for CA3 vs. DG). Finally, the significant genes showing concordant FC in both platforms were: 2,379 (CA1 vs. CA3), 4,352 (CA1 vs. DG), and 4,642 (CA3 vs. DG). The complete results of the differential analysis for the genes detected with both platforms are reported in Tables S1–S3, and the overlap is represented in the proportional Venn diagram in Figure 2A.

**Table 1:** Number of genes that were statistically significant (adj-*p* < 0.05) and detected in both platforms.

**Test**	**Illumina**		**Ion Proton**		**Cross-Platform Comparison**		
	**Total Genes Detected**	**adj-*p* < 0.05**	**Total Genes Detected**	**adj-*p* < 0.05**	**Mutual Found**	**adj-*p* < 0.05**	**adj-*p* < 0.05 and FC Concordant**
*CA1* vs. *CA3*	18,987	5,722	19,506	3,062	18,263	2,384	2,379
*CA1* vs. *DG*	19,427	8,675	19,506	5,515	18,436	4,366	4,352
*CA3* vs. *DG*	19,870	8,903	19,972	5,589	18,929	4,647	4,642

*Total Genes Detected are the number of genes detected that were not filtered out by DESeq2 and for which an adj-p-value was computed*.

We computed the correlation coefficient for the Log2 FC between the two platforms in each pairwise comparison for all detected genes. The Pearson coefficients demonstrated a strong positive correlation for all of the comparisons (*p* < 2.2e-16), ranging from *R* = 0.776 (CA1 vs. CA3) to R = 0.836 (CA3 vs. DG). In Figure 2B, we illustrate the corresponding scatterplots from these comparisons with the blue points indicating genes that were significantly differentially expressed across both platforms (adj-*p* < 0.05).

### Gene expression across regions: subfield specific transcriptome profiling

We looked for subfield specific transcriptomic signatures for genes that met the criteria of significantly differentially expressed on both platforms and concordant for fold change across platforms. We detected a total of 908 (CA1), 1,063 (CA3), and 2,431 (DG) specific genes. The results for all three regions are reported in Tables S4–S6. The heatmap including the specific genes for each region is reported in Figure S2.

In the CA1 region, 898 of the DEGs were protein coding, whereas the remaining 10 were pseudogenes or processed transcripts. A total of 359 genes (39.5%) were overexpressed and 549 (60.5%) underexpressed with respect to the CA3 and DG regions. The boxplots for all of the specific genes are illustrated in Figure S3, and in Figure S4 we show the Allen Brain Atlas data from mouse for *Wfs1, Nov, Ndst4*, and *Gpr161*. The first 5 genes ranked by absolute average fold change were: *Vgll3, Mx1, Gnrhr* (underexpressed), and *Wnt3, Ackr2* (overexpressed). Pathway analysis conducted with ToppGene using genes with average FC > |0.50| (*n* = 379) reveals one significant pathway after Bonferroni correction (KEGG: neuroactive ligand-receptor interaction; adj-p = 7.40E-04) that included 20 genes (Table S7). The enrichment analysis conducted with DAVID did not identify any significant results after Benjamini correction.

In the CA3 region a total of 1,051 of the differentially expressed genes were protein coding, whereas the remaining 12 were processed transcripts or pseudogenes. A total of 551 genes (51.8%) were overexpressed, whereas 512 were underexpressed (48.2%) with respect to the CA1 and DG (Table S5). The boxplots for all of the specific genes are illustrated in Figure S5, and in Figure S6 we show the Allen Brain Atlas data from mouse for *Col6a6* and *Nnat*. The first 5 genes ranked by absolute average fold change were *Lnp1, RGD1562638, Plk5* (underexpressed), and *Mmp3, Mx1* (overexpressed). Pathway and enrichment analysis conducted using genes with average FC > |0.50| (*n* = 535) revealed 11 significant pathways after Bonferroni correction. Top results were: KEGG calcium signaling (adj-*p* = 4.27E-04; 17 genes) and REACTOME potassium channel (adj-*p* = 1.73E-03) (Table S7). The analysis conducted with DAVID allowed us to detect 3 significant GO biological processes after Benjamini correction: cilium movement, outer dynein arm, and potassium ion transmembrane transport (Table S7). The last process was also detected using ToppGene.

In the DG region 2,403 of the differentially expressed genes were protein coding, whereas the remaining 28 were pseudogenes, miRNA, processed transcripts or lincRNA. 975 genes (40.1%) were overexpressed and 1,456 underexpressed (59.9%) with respect to CA1 and CA3 regions (Table S6). The first 5 genes ranked by absolute FC were: *Bhlhe23, Met, Abca12, RGD1562638*, and *Ptpru*. The boxplots for all the specific genes are illustrated in Figure S7; in Figure S8 we show the Allen Brain Atlas data from mouse for genes *Pdin, Plk5, C1ql2*, and *Dsp*. The enrichment analysis conducted with ToppGene using genes with absolute average FC > |0.50| (*n* = 927) revealed 7 significant pathways after Bonferroni correction. The most significant pathways were: KEGG axon guidance (adj-*p* = 4.70E-05; 23 genes) and REACTOME adherens junctions interactions (adj-*p* = 6.20E-04; 10 genes). The remaining significant pathways are reported in Table S7. In this same subfield we also detected the KEGG pathway neuroactive ligand-receptor interaction (adj-*p* = 4.92E-03; 32 genes), the only one shared with CA1 and CA3. In contrast, REACTOME gastrin-CREB signaling pathway via PKC and MAPK and REACTOME neuronal system were detected in CA3 region. The analysis conducted with DAVID revealed enrichment for 8 biological processes: neural crest cell migration (FDR adj-*p* = 1.77E-03; 12 genes), semaphorin-plexin signaling (FDR adj-*p* = 2.11E-03) and myosin complex (FDR adj-*p* = 7.65E-03). All the remaining processes (FDR adj-*p* < 0.05) are reported in Table S7.

### Gene expression related to aging

The data sets for differential expression using the cross platform validation method described above were used to investigate age-related changes in gene expression. The CA1 Illumina poly-A mRNA analysis produced 228 DEGs that passed the statistical filter. From this seed list the number of genes that was detected for Ion Proton poly-A mRNA was 104 (FDR = 1.1^−3^). All 104 of these genes were in the NIH DAVID database. Among these, 90 genes were up regulated and 14 genes were down regulated in aged rats. Enrichment analysis indicated clusters related to immune function including the lysosome (GO:0005764, 14 genes, FDR adj-*p* = 1.3^−.3^), regulation of leukocyte mediated immunity (GO:0002443, 14 genes, FDR adj-*p* = 1.6^−5^), immune response (GO:0006955, 28 genes, FDR adj-*p* = 1.8^−7^), and neutrophil activation (GO:0042119, 4 genes, FDR adj-*p* = 9.8^−3^). In addition, clusters related to cell adhesion (GO:0007155, 25 genes FDR adj-*p* = 2.9^−4^) and regulation of ERK1 and ERK2 cascade (GO:0070372, 8 genes, FDR adj-*p* = 2.2^−2^) were also present. Furthermore, a KEGG pathway for the lysosome (rno04142, 8 genes, FDR adj-*p* = 5.2^−3^) was identified. Enrichment analysis using the ToppGene web tool confirmed the enrichment of pathways related to immune function (i.e.: Lysosome, B cell receptor signaling pathway and adaptive immune system). All genes under the indicated immune related functional clusters were up regulated genes (Figure S9).

For region CA3, analysis of Illumina poly-A mRNA indicated 189 DEGs in the seed list and the number validated with the Ion Proton poly-A mRNA was 81 (FDR adj-*p* = 1.2^−3^). All 81 genes were in the NIH DAVID database, with 61 genes up regulated and 20 down regulated genes in aged rats. As with area CA1, clustering analysis was related to immune function including leukocyte activation (GO:0045321, 17 genes, FDR adj-*p* = 4.9^−5^) immune response (GO:0006955, 20 genes, FDR adj-*p* = 8.8^−5^), cytokine production (GO:0001816, 12 genes, FDR adj-*p* = 2.4^−3^), antigen processing and presentation of peptide antigen via MHC class II (GO:0002495, 7 genes, FDR adj-*p* = 4.4^−7^), and interleukin-10 production (GO:0032613, 5 genes, FDR adj-*p* = 3.0^−3^). Additionally, a cluster for biological adhesion (GO:0022610, 21 genes, FDR adj-*p* = 4.4^−4^) and a KEGG pathway for cell adhesion molecules (rno04514, 7 genes, FDR adj-*p* = 2.3^−3^) were found. Enrichment analysis conducted with ToppGene confirmed the presence of pathways correlated with the immune system (i.e., immunoregulatory interactions between a lymphoid and a non-lymphoid cell, adaptive immune system, and phagosome). All genes under the indicated immune related functional clusters were up regulated (Figure S10).

The DG region exhibited similar aging patterns in relation to the CA1 and CA3, with 280 DEGs in the Illumina poly-A mRNA data set. From this list, 162 genes were validated by the Ion Proton (FDR = 8.6^−4^), and all 161 genes were in the DAVID database. From the validated gene list, 134 genes were up regulated and 27 genes decreased with aging. Enriched clusters included immune response (GO:0006955, 41 genes, FDR adj-*p* = 2.9^−11^), defense response (GO:0006952, 40 genes, FDR adj-*p* = 5.8^−9^), inflammatory response (GO:0006954, 26 genes, FDR adj-*p* = 1.1^−8^), response to wounding (GO:0009611, 15 genes, FDR adj-*p* = 9.3^−3^), response to steroid hormone (GO:0048545, 14 genes, FDR adj-*p* = 1.3^−2^) and cell-cell adhesion (GO:0098609, 30 genes, FDR adj-*p* = 2.7^−6^). KEGG pathways were identified for lysosome (rno04142, 10 genes, FDR adj-*p* = 3.2^−4^), antigen processing and presentation (rno04612, 11 genes, FDR adj-*p* = 5.2^−6^), and cell adhesion molecules (rno04514, 10 genes, FDR adj-*p* = 2.8^−3^). All genes under the indicated immune related functional clusters were up regulated genes (Figure S11). Again, the analysis conducted with ToppGene confirmed the immunologic processes detected with DAVID. In addition, we detected 3 pathways involving the complement system: initial triggering of complement, complement and coagulation cascades, complement cascade, and activation of C3 and C5. Beside the immunological pathways, we detected the cadherin signaling and Wnt signaling.

**Figure 3:**
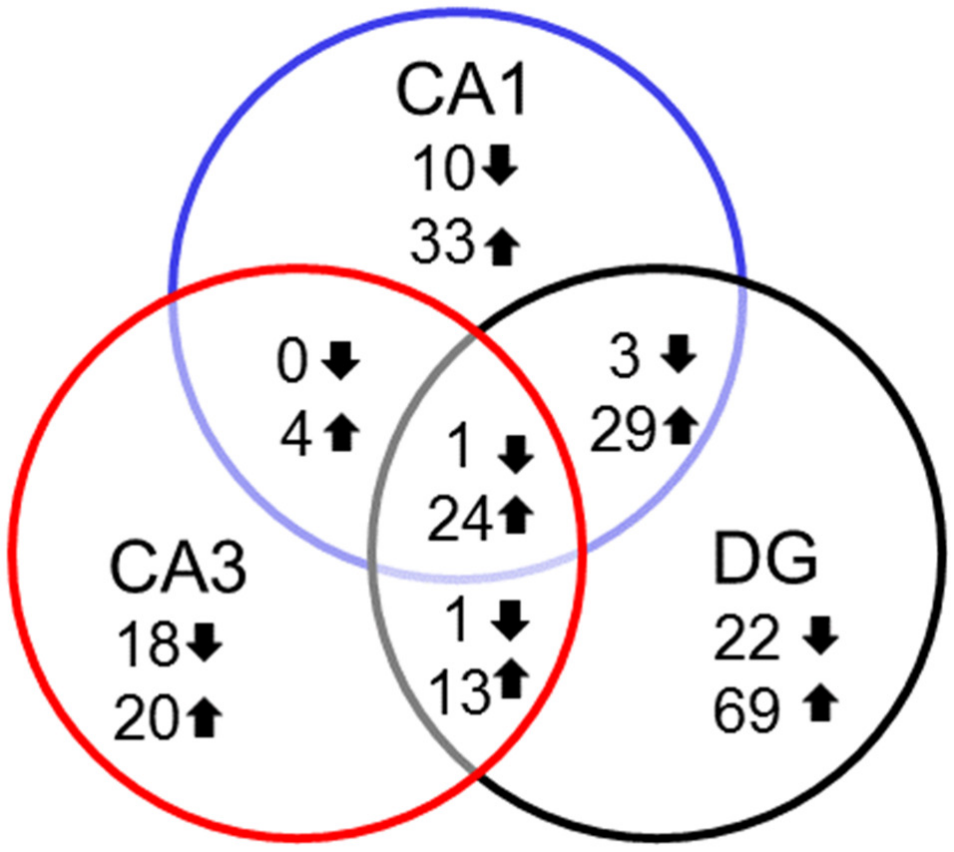
Number of genes altered during aging across hippocampal regions. Summary of the total number of genes across the hippocampal subfields whose expression increased (up arrow) or decreased (down arrow) using Illumina DEGs with *p* < 0.01 validated with Ion Proton mRNA with *p* < 0.05.

In examining genes that were differentially expressed with age across regions it is clear that each region contains a number of age-related genes that are unique relative to the other hippocampal regions. For the most part, more genes were up regulated in older rats within each region and several up regulated genes overlapped across regions. Among the genes that overlapped regions, several were related to immune function (Table 2). Only one gene, *Col4a5*, exhibited a decrease across all three regions. Interestingly, the DG region showed the highest number of gene changes related to the age of the animal (162), including the greatest number of distinct genes (91 genes) (Figure 3). Among the 69 genes that were uniquely up regulated in the DG, 68 matched DAVID for functional annotation clustering analysis with the top clusters involved in immune function. Together, the results indicate that the DG region may be more sensitive to aging effects.

**Table 2:** Genes, which significantly increased expression across all three regions in aged animals from both the Illumina and Ion Proton data.

**Gene symbol**	**Gene Name**	**Immune related**
*C3*	Complement component 3	x
*Card11*	Caspase recruitment domain family, member 11	x
*Cd4*	Cd4 molecule	x
*Cd74*	Cd74 molecule, major histocompatibility complex, class II invariant chain	x
*Cdh1*	Cadherin 1	
*Cdh23*	Cadherin 23 (otocadherin)	
*Csf1r*	Colony stimulating factor 1 receptor	x
*Ctss*	Cathepsin S	x
*Ctsz*	Cathepsin Z	
*Cx3cr1*	Chemokine (C-X3-C motif) receptor 1	x
*Fcgr2b*	Fc fragment of IgG, low affinity IIb, receptor (CD32)	x
*Gfap*	Glial fibrillary acidic protein	x
*Gpr183*	G protein-coupled receptor 183	x
*Gpr84*	G protein-coupled receptor 84	x
*Itgb2*	Integrin beta 2	x
*Ncf1*	Neutrophil cytosolic factor 1	x
*Nckap1l*	NCK associated protein 1 like	x
*Npc2*	Niemann-Pick disease, type C2	
*Pcdhb4*	protocadherin beta 4	
*Pld4*	Phospholipase D family, member 4	
*Slc14a1*	Solute carrier family 14 (urea transporter), member 1	
*Trem2*	Triggering receptor expressed on myeloid cells 2	x
*Bpifb4*	BPI Fold Containing Family B, Member 4	
*Wdfy4*	WDFY Family Member 4	

*Genes that are related to immune function are indicated*.

### Gene expression related to behavior

**Figure 4:**
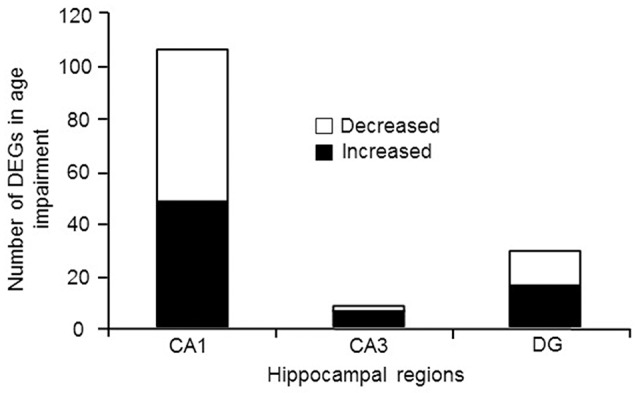
Number of genes differentially expressed in regions CA1, CA3, and DG according to cognitive function. In each case, the number of genes down regulated (open portion) and up regulated (filled portion) in aged-cognitively impaired animals are represented.

For examination of genes linked to cognitive status, only aged animals were included in order to avoid age as a confound. Aged animals were subdivided into AI and AU according to the water maze DI score. Figure 4 illustrates the number of genes that were differentially expressed in CA1, CA3, and the DG according to cognitive status, and were annotated in DAVID. The Illumina analysis of CA1 poly-A mRNA produced 235 sequences that passed the statistical filter. From the seed list, the number of genes that was detected for Ion Proton mRNA was 107 (FDR = 1.1^−3^) (Table S8). For the 107 genes, 106 were in the NIH DAVID database, with 48 up regulated and 58 down regulated genes in aged impaired animals. The CA3 Illumina mRNA analysis contained 32 sequences in the seed list and the number validated with the Ion Proton poly-A mRNA was 8 (FDR = 2.0^−3^) (Table S8). All 8 genes were annotated in DAVID (increased: *Prl, Pcdh19, Pcdh17, Gabrb1, Gda, Als2*; decreased: *Msrb2, Wnt6*). The DG Illumina poly-A mRNA analysis contained 97 sequences in the seed list and the number validated with the Ion Proton mRNA was 30 (FDR = 1.6^−3^) (Table S8), which included 29 genes that were annotated in DAVID (16 up regulated and 13 down regulated). Only one gene, *Prl*, was increased across all regions.

Up and down regulated genes were combined within each region and submitted for cluster analysis. The results indicated that only CA1 exhibited significant enrichment. Clustering was observed for cellular components with CA1 genes linked to synapse (GO:0045202, 20 genes, FDR adj-*p* = 2.6^−5^), cell junction (GO:0030054, 18 genes, FDR adj-*p* = 1.2^−2^), neuron part (GO:0097458, 22 genes, FDR adj-*p* = 5.6^−3^), postsynapse (GO:0098794, 10 genes, FDR adj-*p* = 1.3^−2^), ion channel complex (GO:0034702, 9 genes, FDR adj-*p* = 5.9^−3^) and plasma membrane region (GO:0098590, 16 genes, FDR adj-*p* = 6.1^−3^) (Table 3). In addition, enrichment for biological processes included regulation of signaling (GO:0023051, 34 genes, FDR adj-*p* = 4.7^−3^), the KEGG pathways for calcium signaling (rno04020, 7 genes, FDR adj-*p* = 1.7^−2^) and long-term potentiation (rno04720, 4 genes, FDR adj-*p* = 3.5^−2^). ToppGene identified five significant pathways after Bonferroni correction: glutamatergic synapse, neuronal system, heterotrimeric G-protein signaling pathway-Gq alpha and Go alpha mediated pathway, calcium signaling, and phosphatidylinositol signaling system. The results confirmed the presence of neuronal genes, but pointed to more specific processes, including the glutamatergic synapse.

**Table 3:** Genes of age impaired and age unimpaired animals, which were significantly differentially expressed in region CA1 in both the Illumina and Ion Proton data.

**Gene symbol**	**Gene Name**	**Ca^2+^ binding**	**Synaptic function**
*Adra1d*	Adrenergic, alpha-1D-, receptor	x	x
*Akap13*	A kinase (PRKA) anchor protein 13		x
*Arpc5*	Actin related protein 2/3 complex, subunit 5		x
*Dclk2*	Doublecortin-like kinase 2		x
*Dgkz*	Diacylglycerol kinase zeta		x
*Gabra5*	Gamma-aminobutyric acid (GABA) A receptor, alpha 5		x
*Homer3*	Homer homolog 3 (Drosophila)		x
*Itpr1*	Inositol 1,4,5-triphosphate receptor, type 1		x
*Kcnab2*	Potassium voltage-gated channel, shaker-related subfamily, beta member 2		x
*Ksr1*	Kinase suppressor of ras 1		x
*Mpp3*	Membrane protein, palmitoylated 3 (MAGUK p55 subfamily member 3)		x
*Neto1*	Neuropilin (NRP) and tolloid (TLL)-like 1	x	x
*Nptxr*	Chromobox homolog 6; neuronal pentraxin receptor	x	x
*Pragmin*	Pragma of Rnd2		x
*Prickle2*	Prickle homolog 2 (Drosophila)	x	x
*Rem2*	RAS (RAD and GEM) like GTP binding 2	x	x
*Slc17a8*	Solute carrier family 17, member 8		x
*Vav2*	vav 2 guanine nucleotide exchange factor		x
*Cacnb2*	Calcium channel, voltage-dependent, beta 2 subunit	x	x
*Dgka*	Diacylglycerol kinase, alpha	x	
*Hpca*	hippocalcin	x	
*Itpr1*	Inositol 1,4,5-triphosphate receptor, type 1	x	
*Prkca*	Protein kinase C, alpha	x	
*Scube2*	Signal peptide, CUB domain, EGF-like 2	x	
*Sulf2*	Sulfatase 2	x	

*Genes that are related to Ca^2+^ binding and synaptic function are indicated*.

**Figure 5:**
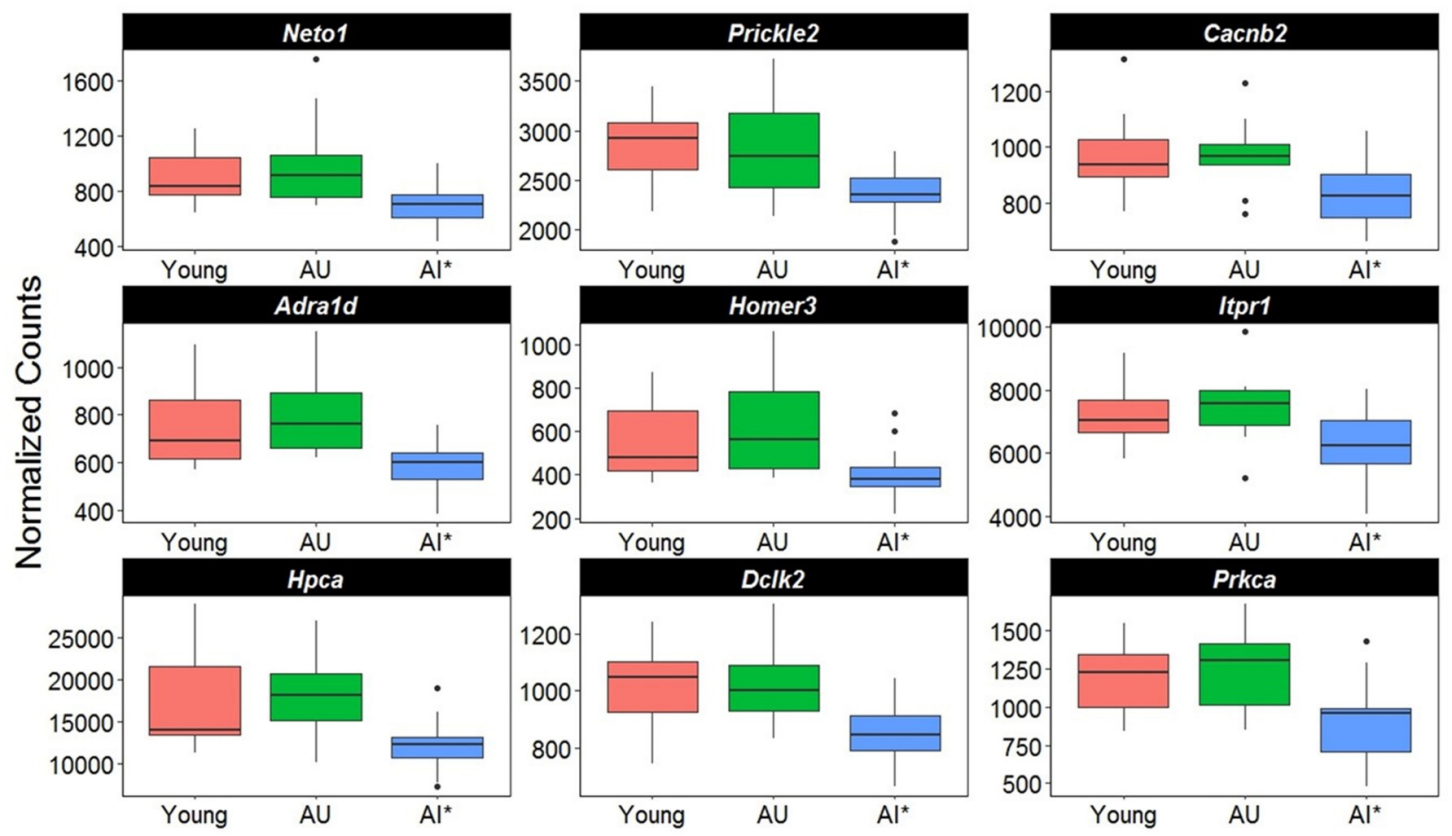
Altered transcription of genes linked to calcium regulation. The y-axis represents Illumina normalized counts and the asterisk in the x-axis represent differential expression of AI rats relative to AU in the Illumina platform (*p* < 0.01) validated with the Ion Proton (*p* < 0.05).

A closer examination of the direction of gene changes suggested that there was decreased expression of genes that regulated Ca^2+^ entry by modulating the function of NMDA receptors (*Neto1, Prickle2*), or voltage-gated Ca^2+^ channels (*Cacnb2*) (Figure 5). Decreased expression was also observed for genes linked to receptor mediated release of Ca^2+^ from intracellular Ca^2+^ stores (*Adra1d, Homer3, Itpr1*). A decline in protein expression for these genes might be expected to decrease intracellular Ca^2+^. Similarly, increased expression was observed for the membrane Ca^2+^ ATPase pump (*Atp2b4*), which would remove intracellular Ca^2+^. Moreover, decreased expression was observed for genes linked to downstream Ca^2+^ signaling (*Hpca, Dclk2, Prkca*). Together, the results suggest that in impaired animals, transcription is altered in an attempt to limit intracellular Ca^2+^ signaling (Figure 5).

**Figure 6:**
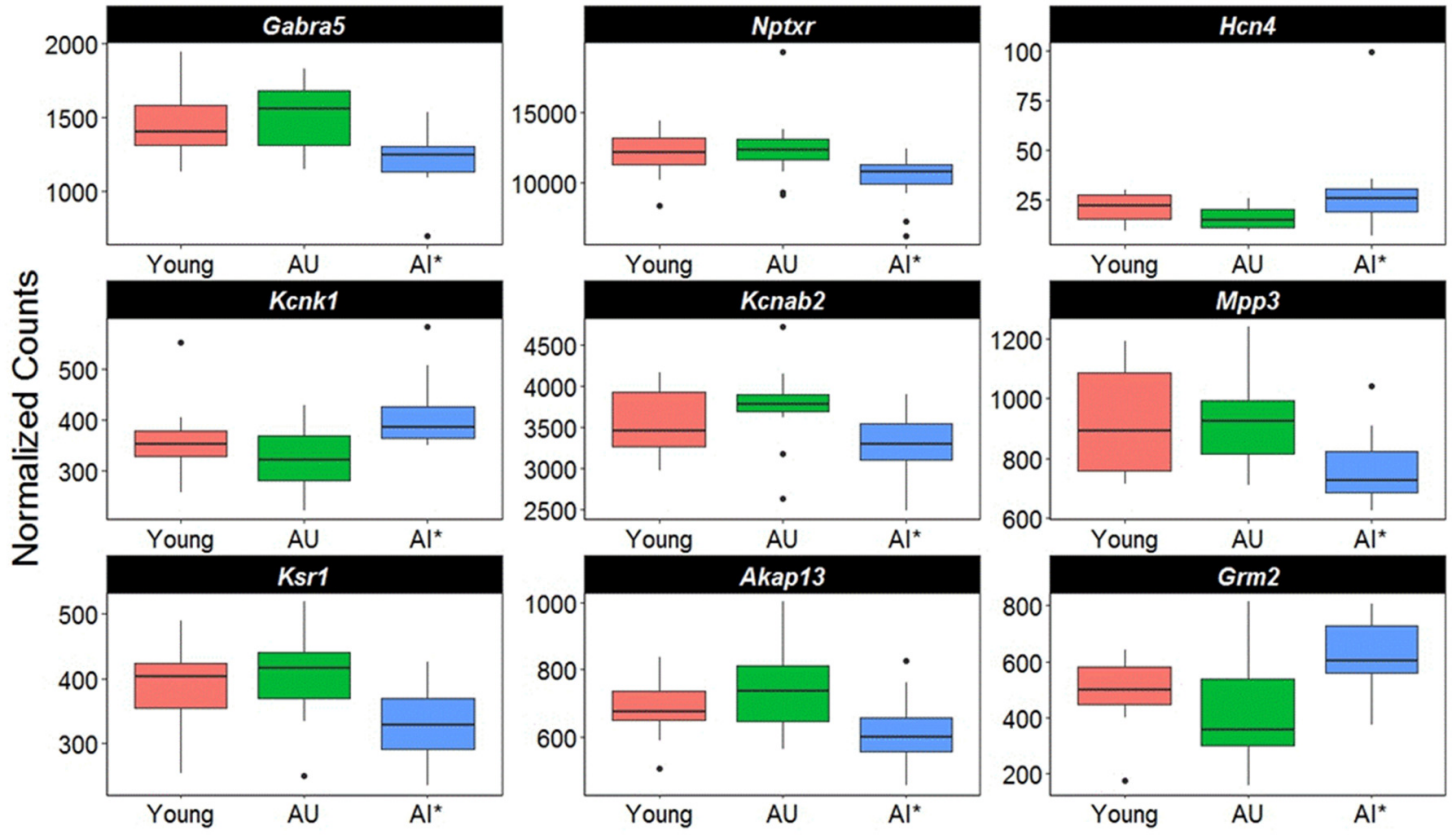
Altered transcription of genes linked to cell excitability. The y-axis represents Illumina normalized counts and the asterisk in the x-axis represent differential expression of AI rats relative to AU in the Illumina platform (*p* < 0.01) validated with the Ion Proton (*p* < 0.05).

Closer examination of the direction of change for genes linked to synaptic activity suggests differential expression in genes for regulating synaptic receptors and K^+^ channels (Figure 6). For example, there was a decrease in the GABA receptor subunit (*Gabra5*) and *Nptxr*, which are involved in clustering of AMPA receptors at the synapse. Increased expression was observed for two K^+^ channels, *Hcn4* and *Kcnk1*, and *Kcnab2*, a cytoplasmic potassium channel subunit that modulates K^+^ channel activity was down regulated. Finally, there was down regulation of genes for proteins that are downstream of G-protein coupled receptor activation (*Mpp3, Ksr1, Akap13*) and an increase expression of the glutamate metabotropic receptor (*Grm2*).

Finally, we examined the specificity of gene changes. In this case, aged animals were separated by performance on the cue task, using a mean split for block 5. Relative to gene expression associated with spatial discrimination performance, fewer genes were associated with performance on the cue task. Analysis of the CA1 region poly-A mRNA using Illumina produced 135 sequences that differed according to cue discrimination performance. From this seed list the number of genes that was detected for Ion Proton poly-A mRNA was 24 (FDR = 2.8^−3^). For region CA3, 38 differentially expressed genes were detected by Illumina and 3 genes were confirmed by the Ion Proton (FDR = 6.3^−3^). For the dentate gyrus, Illumina detected 71 sequences and 4 were confirmed by the Ion Proton (FDR = 8.8^−3^). No gene clusters were observed for any region.

## Discussion

### Gene expression across subfields: subfield specific transcriptome profiling

We report the results of the transcriptional differences among hippocampus brain subfields (CA1, CA3, and DG) regardless of the animal's age or cognitive status. Our approach was based on retaining for each subfield, genes significant and concordant in FC in both pairwise comparisons. We were able to detect a high number of specific genes for each subfield, specifically: 908 (CA1), 1,063 (CA3), and 2,431 (DG) genes. These lists include genes highly or lowly expressed in each subfield with respect to the others. In CA1, the top gene with high values of expression compared to the other subfields was *Wnt3* (Wnt Family Member 3), which is considered to play a central role in synaptogenesis and adult neurogenesis (Varela-Nallar and Inestrosa, 2013). In area CA1, Wnt signaling has been linked to synaptic plasticity and associated hippocampal-dependent memory processes (Xu et al., 2015; Ivanova et al., 2016). At the pathway level, those genes with higher expression in CA1 exhibited enrichment for Neuroactive ligand-receptor interaction, including *Nts* (neurotensin), *Htr1b* (5-hydroxytryptamine receptor 1B) and *Gpr161* (G Protein-Coupled Receptor 161).

For subfield CA3, we observed increased expression of genes linked to processes involving potassium channel activity in both ToppGene (Potassium channels, Voltage gated potassium channels) and DAVID enrichment analysis (GO:0071805 Potassium ion transmembrane transport). Potassium channels are central to intrinsic excitability in region CA3 of the hippocampus, which is highly vulnerable to cell death due to seizure activity (Ben-Ari, 1985; Cooper, 2012). Indeed, many of the top overexpressed genes (*Mmp3, Pagl1Zac1, Galr1, Glra1*, and *Nk3r*) are regulated by neural activity and linked to epiletogenesis of the hippocampus (Elmslie and Gardiner, 1995; Valente et al., 2004; Mazarati et al., 2006; McColl et al., 2006; Dubey et al., 2017).

Enrichment analysis of genes that are highly expressed in the DG relative to other subfields indicated that the most significant processes were “Axon guidance” (KEGG) and “Neural crest cell migration” (GO:0001755). In general, the genes and the biological processes detected as enriched in the DG may be due to the known neurogenesis activity in this subfield. In the DG, the top genes overexpressed with respect the other regions included *Bhlhe23* (basic helix-loop-helix family member E23) and *Msx3* (msh like homeobox 3), which are involved in neurogenesis and specification of neuronal subtype (Bramblett et al., 2004; Liu et al., 2004; Hesse et al., 2011). *EphA8* (EPH receptor A8), a member of the ephrin receptor subfamily and *C1ql2* (complement C1q like 2) are implicated in neurite growth and establishing synaptic connections (Buchser et al., 2010; Iijima et al., 2010). *Plk5* (polo like kinase 5) modulates the formation of neuritic processes upon stimulation of the brain-derived neurotrophic factor (BDNF)/nerve growth factor (NGF)-Ras pathway in neurons (de Carcer et al., 2011). Interestingly, we also found *Bdnf* specifically overexpressed in this subfield (average FC = 0.948).

### Subfield vulnerability to aging

It is likely that regional differences in transcription during aging are associated with differences in connectivity and molecular make-up of each subfield. For example, relative to CA3 pyramidal neurons, CA1 pyramidal neurons are more sensitive to metabolic perturbations (Jackson et al., 2009). Blalock and associates have suggested that metabolic changes precede neuroinflammation and have linked increased vulnerability of gene changes in CA1, relative to CA3, to biomarkers of metabolic syndrome in nonhuman primates (Blalock et al., 2010). In considering the increase in age-related shift in transcription in the DG, it is important to note that neurogenesis in the DG exhibits a robust decline between adult and middle-age (Kuhn et al., 1996; Lemaire et al., 2000; Driscoll et al., 2006). Similarly, the rat DG exhibits a loss of perforant path input (Geinisman, 1979; Barnes and McNaughton, 1980), and loss of neuronal connections underlie the altered expression of genes related to cell-cell adhesion or axon guidance and increased markers of inflammation (Ianov et al., 2016). Interestingly, older memory-impaired humans also show reduced white matter volume in the vicinity of the major axon input pathway to the hippocampus, the perforant pathway (Rogalski et al., 2012). Thus, while we observed that the DG exhibits robust changes in transcription during aging, it remains to be discovered how these changes specifically relate to DG function and to anatomical or physiological changes associated with aging. The subfield-specific changes observed in activity of the human dentate gyrus/CA3 region, however, does suggest that these alterations occur across species, and may contribute to memory deficits during aging in humans and other animals (Yassa et al., 2011).

Consistent with a number of previous studies, we observed that brain aging was associated with an increase in markers of neuroinflammation including expression of immune response genes (Rowe et al., 2007; Kadish et al., 2009; Blalock et al., 2010; Zeier et al., 2011; Cribbs et al., 2012; Ianov et al., 2016). Typical genes include *Gfap*, complement genes (*C3, C4b*), and genes associated with antigen processing through the lysosome (*Cd74, Ctsd, Ctsz, Laptm5, Dnase2*) or phagosome (*Fcgr2b, Ncf1*). In the current study, the DG region showed the highest number of gene changes related to the age of the animal. This is in contrast to previous microarray studies, which suggest that for BNxF344 rats examined between middle-age (12–18) and advanced age (26–28 months), region CA1 is more vulnerable than DG (Zeier et al., 2011; Masser et al., 2014). In this case the differences likely relate to the animal model, age of onset for gene changes, and differences in the age range examined (Ianov et al., 2016). In F344 rats, for ages similar to those employed in the current study (5–6 and 17–22 months), the CA1 region of older animals exhibited relatively smaller transcriptional changes compared to the prefrontal cortex and white matter (Ianov et al., 2016). Similarly, a decline in interneuron markers occurs in all hippocampal regions from adult to middle-age in F344 rats and the decline is observed in CA1 but not the DG when analysis is limited to middle-age and aged BNxF344 rats (Shi et al., 2004; Stanley and Shetty, 2004). Thus, one possibility is that changes in the DG emerge earlier than in CA1, minimizing age-related changes between middle-age and older animals. Alternatively, we have observed that the majority of gene changes in region CA1 of F344 rats occurs between adult and middle-age (Blalock et al., 2010), suggesting possible species differences.

The increase in immune response genes suggests activation of astrocytes and microglia. However, it is important to note that the age-related increase in expression of inflammation genes does not necessarily predict memory impairment or the stage of neurodegeneration (Blalock et al., 2003; Cribbs et al., 2012). In the case of neurodegeneration, neuroinflammation may initially have beneficial and neuroprotective effects (Chakrabarty et al., 2010; Streit et al., 2014; Sochocka et al., 2017). Glial activation during aging is associated with a decline in synaptic density (Rozovsky et al., 2005) and the ability to maintain or form new synapses may underlie resiliency in the face of aging or neurodegenerative disease (Arnold et al., 2013; Ianov et al., 2016, 2017).

### Genes associated with impaired episodic memory

The identification and specificity of genes related to cognition is a function of the brain region sampled and the type of cognitive process examined (Ianov et al., 2016). In the current study, within each region, more genes were correlated with episodic memory scores relative to sensory-motor performance on the cue discrimination task, suggesting specificity of gene changes. While the hippocampus is required for spatial memory, different hippocampal-dependent processes depend on specific regional and molecular mechanisms. For example, the DG is involved in spatial pattern separation and region CA1 with memory for topological relationships in space (Kesner et al., 2004; Goodrich-Hunsaker et al., 2008; Kesner and Rolls, 2015). Thus, if the task had focused on spatial pattern separation we would expect a greater correspondence between behavior and transcription in the DG. Regardless, aging of the DG likely contributes to impaired spatial episodic memory. While no functional gene clusters were observed, several of the DG genes that were down regulated in impaired animals (*Bcl6, Crim1, Dapk1, Mtss1, Oas1a*) have been linked to cell growth/apoptosis and neurogenesis or neuronal development.

The results on CA1 transcriptional changes associated with cognitive decline are in confirmation of previous studies employing microarray technology, which indicate that impaired cognition is associated with altered expression of synaptic genes, genes involved in Ca^2+^ regulation, and glutamatergic synapses (Toescu et al., 2004; Burger, 2010; Uddin and Singh, 2013; Volk et al., 2015). However, rarely are the same genes observed to change in the same direction across studies. The difference may be due to different cognitive processes examined including reference memory and episodic memory, differences in ages, and the time after behavioral training in which transcription is examined. Previous work has established that Ca^2+^ dysregulation and impaired Ca^2+^-dependent synaptic plasticity in region CA1 is associated with poor performance on episodic spatial memory (Landfield, 1988; Foster and Norris, 1997; Foster, 1999, 2007; Burke and Barnes, 2010; Oh and Disterhoft, 2010) and these processes are influenced by behavioral training. We examined transcription 2 weeks after testing. Thus, the differences observed here are not likely due to differential response to training and may represent an underlying and chronic shift in transcription for this region. In our experiments, the changes in expression of genes involved in the glutamatergic synapses in aging are concordant with the evidence that P301L tau expression increased hippocampal glutamate release and decreased glutamate uptake, and these alterations in glutamate signaling correlated with cognitive deficits in the hippocampal-dependent Barnes maze task (Hunsberger et al., 2015).

Finally, it is important to point out the advantages or disadvantages of the experimental design. The current study was conducted across multiple testing sites and employed two different methods for transcriptional analysis. The advantage of multiple testing sites includes the ability to pool data across sites. However, there is the challenge of equating data due to differences in hardware or protocols. Episodic memory is sensitive to age-related cognitive decline across species; however, there is considerable variability in cognition with advancing age. Furthermore, aged animals can exhibit sensory-motor impairment and acquire the procedural aspects of the task much more slowly than young animals, which could influence the validity of cognitive measures. Thus, in the current study possible sensory-motor and procedural learning contributions to behavior on the spatial task were controlled by first testing the animals on the cue discrimination task. Characterization of spatial episodic memory was based on a DI score, which would tend to standardize scores for animals tested on mazes of different sizes. The testing procedure and use of a DI score likely contributed to the good correspondence of behavioral results across the different testing sites, such that all young animals performed above chance (DI score = 0) and approximately half the aged animals at each site exhibited impaired performance. Finally, behavior on the spatial task was not related to behavior on the cue discrimination task.

Due to the large numbers of transcripts examined, the possibility of false positives or false negatives should be controlled. In order to reduce the instance of false discovery we were able to increase the power of the analysis by pooling a relatively large number of animals that were tested across the two sites (young = 10; aged unimpaired = 12; aged impaired = 12). In addition, it is advised that transcriptional profiling studies should use another method to confirm suspected changes. Each method has its benefits and limitations and there are major differences in the nature of the approaches. In the current study, we have taken advantage of two different next-generation platforms, Illumina fluorescent labeling and Ion Proton semiconductor measures of proton release, to confirm differential expression of thousands of genes (Kusko et al., 2014; Li et al., 2015; Reuter et al., 2015). Consistent with previous reports, we observed a good concordance in the gene counts between the different platforms (R = 0.909), increasing the confidence in the observed transcriptional changes. We now expand this idea by showing considerable concordance between the two platforms in detecting differences in expression. Thus, coefficients for the Log2 FC between the two platforms across regions was highly significant (Figure 2B), with the correlation coefficients ranging from 0.776 (CA1 vs. CA3) to 0.836 (CA3 vs DG). Indeed, for genes that changed in relation to age or cognitive function, the probability of false discovery across the platforms was generally <2.0^−3^.

## Conclusion

The two next-generation sequencing platforms were able to validate variability in gene expression associated with several variables. Due to the high number of specific genes detected in each subfield, our findings confirm previous studies conducted on the same hippocampal subfields in other animal models with the microarray technology, demonstrating a correspondence between cytoarchitectural boundaries, transcriptome distribution, and underlying differences in physiology and vulnerability to insults (Datson et al., 2004; Lein et al., 2004; Newrzella et al., 2007). The results confirm that brain aging is associated with increased expression of genes linked to the immune response; however, we noted regional differences with more robust changes observed in the DG. Finally, the literature suggests several physiological and transcriptional processes that may underlie cognitive decline; however, there is considerable diversity in the literature concerning which specific genes are altered. Our results confirm that cognitive decline is associated with differential expression of CA1 genes linked to Ca^2+^ homeostasis and synaptic plasticity. Furthermore, due to the time between testing and examination of transcription, we would suggest that these changes represent an underlying and chronic shift in transcription.

## Author contributions

LI performed experiments, analyzed data, wrote the manuscript, and constructed figures; AR, MC, AS, and AJK, performed experiments; AK and IP performed experiments, contributed in writing the manuscript and figures. MD analyzed data and constructed figures. MH and TF designed the experiments, analyzed the data, constructed figures, wrote the manuscript. JD, CB, and JS designed the experiments, contributed to the manuscript.

### Conflict of interest statement

The authors declare that the research was conducted in the absence of any commercial or financial relationships that could be construed as a potential conflict of interest.

## Supplementary material

Supplementary Figure 1 Correlation (*R*^2^ = 0.938) of gene counts across sequencing platforms, Illumina and Ion Proton.

Supplementary Figure 2 Heatmap of hippocampal subfield changes in gene expression. Plotted for each hippocampal subfield, are genes found with both sequencing platforms to be differentially expressed compared to the other two subfields at a significance of adj-*p* < 0.05. Each row represents the z-scores from the DEGs, and the columns are the biological replicates for each region.

Supplementary Figure 3 Boxplots for the CA1 specific genes reported in Table S4. The normalized counts are from the Illumina experiment, and the genes are ranked according the average Fold Change computed from the pairwise comparisons CA1 vs. CA3 and CA1 vs. DG.

Supplementary Figure 4 Expression of genes *Wsf1, Nov, Ndst4*, and *Gpr161*, (specific to CA1 in our study) as reported in the Allen Brain Atlas (Mouse).

Supplementary Figure 5 Boxplots for the CA3 specific genes reported in Table S5. The normalized counts are from the Illumina experiment, and the genes are ranked according the average Fold Change computed from the pairwise comparisons CA3 vs. CA1 and CA3 vs. DG.

Supplementary Figure 6 Expression of genes *Col6a6* and *Nnat* (specific to CA3 in our study) as reported in the Allen Brain Atlas (Mouse).

Supplementary Figure 7 Boxplots for the DG specific genes reported in Table S6. The normalized counts are from the Illumina experiment, and the genes are ranked according the average Fold Change computed from the pairwise comparisons DG vs. CA1 and DG vs. CA3.

Supplementary Figure 8 Expression of genes *Pdin, Plk5, C1ql2*, and *Dsp* (specific to DG in our study) as reported in the Allen Brain Atlas (Mouse).

Supplementary Figure 9 Heatmap of age-related changes in gene expression in the CA1 region. Each row represents a DEG (Illumina *p* < 0.01; Ion Proton *p* < 0.05) associated with aging. Gene-level counts were standardized to z-scores and the color represents the standard deviation increasing (yellow) or decreasing (blue) relative to the mean (black). The age-related gene enrichment clusters (FDR adj-*p* < 0.05) in the CA1 region included immune related GOs, neutrophil activation, cell adhesion and regulation of ERK1 and ERK2 cascade.

Supplementary Figure 10 Heatmap of age-related changes in gene expression in the CA3 region. Each row represents a DEG (Illumina *p* < 0.01; Ion Proton *p* < 0.05) associated with aging. Gene-level counts were standardized to z-scores and the color represents the standard deviation increasing (yellow) or decreasing (blue) relative to the mean (black). The age-related gene enrichment clusters (FDR adj-*p* < 0.05) in the CA3 region included immune related GOs such as leukocyte activation, cytokine production, and antigen processing and presentation.

Supplementary Figure 11 Heatmap of age-related changes in gene expression in the DG region. Each row represents a DEG (Illumina *p* < 0.01; Ion Proton *p* < 0.05) associated with aging. Gene-level counts were standardized to z-scores and the color represents the standard deviation increasing (yellow) or decreasing (blue) relative to the mean (black). The age-related gene enrichment clusters (FDR adj-*p* < 0.05) in the DG region included immune related GOs such as defense response, and inflammatory response. Cell-cell adhesion GO was also detected.

## References

[B1] Arnold S. E. Louneva N. Cao K. Wang L. S. Han L. Y. Wolk D. A.. (2013). Cellular, synaptic, and biochemical features of resilient cognition in Alzheimer's disease. Neurobiol. Aging 34, 157–168. 10.1016/j.neurobiolaging.2012.03.004 22554416 PMC3478410

[B2] Barnes C. A. McNaughton B. L. (1980). Physiological compensation for loss of afferent synapses in rat hippocampal granule cells during senescence. J. Physiol. 309, 473–485. 10.1113/jphysiol.1980.sp013521 7252877 PMC1274597

[B3] Bean L. A. Kumar A. Rani A. Guidi M. Rosario A. M. Cruz P. E.. (2015). Re-opening the critical window for estrogen therapy. J. Neurosci. 35, 16077–16093. 10.1523/JNEUROSCI.1890-15.2015 26658861 PMC4682778

[B4] Ben-Ari Y. (1985). Limbic seizure and brain damage produced by kainic acid: mechanisms and relevance to human temporal lobe epilepsy. Neuroscience 14, 375–403. 10.1016/0306-4522(85)90299-4 2859548

[B5] Berron D. Vieweg P. Hochkeppler A. Pluta J. B. Ding S. L. Maass A.. (2017). A protocol for manual segmentation of medial temporal lobe subregions in 7 Tesla MRI. Neuroimage Clin. 15, 466–482. 10.1016/j.nicl.2017.05.022 28652965 PMC5476466

[B6] Blalock E. M. Chen K. C. Sharrow K. Herman J. P. Porter N. M. Foster T. C.. (2003). Gene microarrays in hippocampal aging: statistical profiling identifies novel processes correlated with cognitive impairment. J. Neurosci. 23, 3807–3819. 12736351 10.1523/JNEUROSCI.23-09-03807.2003 PMC6742177

[B7] Blalock E. M. Grondin R. Chen K. C. Thibault O. Thibault V. Pandya J. D.. (2010). Aging-related gene expression in hippocampus proper compared with dentate gyrus is selectively associated with metabolic syndrome variables in rhesus monkeys. J. Neurosci. 30, 6058–6071. 10.1523/JNEUROSCI.3956-09.2010 20427664 PMC3155249

[B8] Bramblett D. E. Pennesi M. E. Wu S. M. Tsai M. J. (2004). The transcription factor Bhlhb4 is required for rod bipolar cell maturation. Neuron 43, 779–793. 10.1016/j.neuron.2004.08.032 15363390

[B9] Buchser W. J. Slepak T. I. Gutierrez-Arenas O. Bixby J. L. Lemmon V. P. (2010). Kinase/phosphatase overexpression reveals pathways regulating hippocampal neuron morphology. Mol. Syst. Biol. 6:391. 10.1038/msb.2010.52 20664637 PMC2925531

[B10] Burger C. (2010). Region-specific genetic alterations in the aging hippocampus: implications for cognitive aging. Front. Aging Neurosci. 2:140. 10.3389/fnagi.2010.00140 21048902 PMC2967426

[B11] Burke S. N. Barnes C. A. (2010). Senescent synapses and hippocampal circuit dynamics. Trends Neurosci. 33, 153–161. 10.1016/j.tins.2009.12.003 20071039 PMC3076741

[B12] Cansino S. (2009). Episodic memory decay along the adult lifespan: a review of behavioral and neurophysiological evidence. Int. J. Psychophysiol. 71, 64–69. 10.1016/j.ijpsycho.2008.07.005 18725253

[B13] Chakrabarty P. Jansen-West K. Beccard A. Ceballos-Diaz C. Levites Y. Verbeeck C.. (2010). Massive gliosis induced by interleukin-6 suppresses Abeta deposition *in vivo*: evidence against inflammation as a driving force for amyloid deposition. FASEB J. 24, 548–559. 10.1096/fj.09-141754 19825975 PMC3083918

[B14] Chapman T. R. Barrientos R. M. Ahrendsen J. T. Hoover J. M. Maier S. F. Patterson S. L. (2012). Aging and infection reduce expression of specific brain-derived neurotrophic factor mRNAs in hippocampus. Neurobiol. Aging 33:832 e1–14. 10.1016/j.neurobiolaging.2011.07.015 21907460 PMC3237944

[B15] Chawla M. K. Penner M. R. Olson K. M. Sutherland V. L. Mittelman-Smith M. A. Barnes C. A. (2013). Spatial behavior and seizure-induced changes in c-fos mRNA expression in young and old rats. Neurobiol. Aging 34, 1184–1198. 10.1016/j.neurobiolaging.2012.10.017 23158763 PMC3665155

[B16] Cooper E. C. (2012). Potassium Channels (including KCNQ) and Epilepsy, in Jasper's Basic Mechanisms of the Epilepsies, eds Noebels J. L. Avoli M. Rogawski M. Olsen R. Delgado-Escueta A. (Bethesda, MD: National Center for Biotechnology Information (US)). 22787644

[B17] Craft S. Foster T. C. Landfield P. W. Maier S. F. Resnick S. M. Yaffe K. (2012). Session III: mechanisms of age-related cognitive change and targets for intervention: inflammatory, oxidative, and metabolic processes. J. Gerontol. A Biol. Sci. Med. Sci. 67, 754–759. 10.1093/gerona/gls112 22570133 PMC3536547

[B18] Cribbs D. H. Berchtold N. C. Perreau V. Coleman P. D. Rogers J. Tenner A. J.. (2012). Extensive innate immune gene activation accompanies brain aging, increasing vulnerability to cognitive decline and neurodegeneration: a microarray study. J. Neuroinflammation 9:179. 10.1186/1742-2094-9-179 22824372 PMC3419089

[B19] Datson N. A. Meijer L. Steenbergen P. J. Morsink M. C. van der Laan S. Meijer O. C.. (2004). Expression profiling in laser-microdissected hippocampal subregions in rat brain reveals large subregion-specific differences in expression. Eur. J. Neurosci. 20, 2541–2554. 10.1111/j.1460-9568.2004.03738.x 15548198

[B20] Daugherty A. M. Bender A. R. Yuan P. Raz N. (2016). Changes in search path complexity and length during learning of a virtual water maze: age differences and differential associations with hippocampal subfield volumes. Cereb. Cortex 26, 2391–2401. 10.1093/cercor/bhv061 25838036 PMC4869801

[B21] de Cárcer G. Escobar B. Higuero A. M. García L. Ansón A. Pérez G.. (2011). Plk5, a polo box domain-only protein with specific roles in neuron differentiation and glioblastoma suppression. Mol. Cell. Biol. 31, 1225–1239. 10.1128/MCB.00607-10 21245385 PMC3067912

[B22] Driscoll I. Howard S. R. Stone J. C. Monfils M. H. Tomanek B. Brooks W. M.. (2006). The aging hippocampus: a multi-level analysis in the rat. Neuroscience 139, 1173–1185. 10.1016/j.neuroscience.2006.01.040 16564634

[B23] Dröge W. Schipper H. M. (2007). Oxidative stress and aberrant signaling in aging and cognitive decline. Aging Cell. 6, 361–370. 10.1111/j.1474-9726.2007.00294.x 17517043 PMC1974775

[B24] Dubey D. McRae P. A. Rankin-Gee E. K. Baranov E. Wandrey L. Rogers S.. (2017). Increased metalloproteinase activity in the hippocampus following status epilepticus. Epilepsy Res. 132, 50–58. 10.1016/j.eplepsyres.2017.02.021 28292736 PMC6690398

[B25] Elmslie F. Gardiner M. (1995). Genetics of the epilepsies. Curr. Opin. Neurol. 8, 126–129. 10.1097/00019052-199504000-00007 7620586

[B26] Foster T. C. (1999). Involvement of hippocampal synaptic plasticity in age-related memory decline. Brain Res. Brain Res. Rev. 30, 236–249. 10.1016/S0165-0173(99)00017-X 10567726

[B27] Foster T. C. (2006). Biological markers of age-related memory deficits: treatment of senescent physiology. CNS Drugs 20, 153–166. 10.2165/00023210-200620020-00006 16478290

[B28] Foster T. C. (2007). Calcium homeostasis and modulation of synaptic plasticity in the aged brain. Aging Cell 6, 319–325. 10.1111/j.1474-9726.2007.00283.x 17517041

[B29] Foster T. C. (2012). Dissecting the age-related decline on spatial learning and memory tasks in rodent models: N-methyl-D-aspartate receptors and voltage-dependent Ca^2+^ channels in senescent synaptic plasticity. Prog. Neurobiol. 96, 283–303. 10.1016/j.pneurobio.2012.01.007 22307057 PMC3307831

[B30] Foster T. C. Barnes C. A. Rao G. McNaughton B. L. (1991). Increase in perforant path quantal size in aged F-344 rats. Neurobiol. Aging 12, 441–448. 10.1016/0197-4580(91)90071-Q 1770978

[B31] Foster T. C. Norris C. M. (1997). Age-associated changes in Ca(2+)-dependent processes: relation to hippocampal synaptic plasticity. Hippocampus 7, 602–612. 10.1002/(SICI)1098-1063(1997)7:6<602::AID-HIPO3>3.0.CO2-G 9443057

[B32] Fraser H. B. Khaitovich P. Plotkin J. B. Pääbo S. Eisen M. B. (2005). Aging and gene expression in the primate brain. PLoS Biol. 3:e274. 10.1371/journal.pbio.0030274 16048372 PMC1181540

[B33] Geinisman Y. (1979). Loss of axosomatic synapses in the dentate gyrus of aged rats. Brain Res. 168, 485–492. 10.1016/0006-8993(79)90304-4 435979

[B34] Goodrich-Hunsaker N. J. Hunsaker M. R. Kesner R. P. (2008). The interactions and dissociations of the dorsal hippocampus subregions: how the dentate gyrus, CA3, and CA1 process spatial information. Behav. Neurosci. 122, 16–26. 10.1037/0735-7044.122.1.16 18298245

[B35] Gray D. T. Barnes C. A. (2015). Distinguishing adaptive plasticity from vulnerability in the aging hippocampus. Neuroscience 309, 17–28. 10.1016/j.neuroscience.2015.08.001 26255677 PMC4630086

[B36] Haberman R. P. Colantuoni C. Koh M. T. Gallagher M. (2013). Behaviorally activated mRNA expression profiles produce signatures of learning and enhanced inhibition in aged rats with preserved memory. PLoS ONE 8:e83674. 10.1371/journal.pone.0083674 24349543 PMC3862806

[B37] Hesse K. Vaupel K. Kurt S. Buettner R. Kirfel J. Moser M. (2011). AP-2delta is a crucial transcriptional regulator of the posterior midbrain. PLoS ONE 6:e23483. 10.1371/journal.pone.0023483 21858141 PMC3153493

[B38] Hunsberger H. C. Rudy C. C. Batten S. R. Gerhardt G. A. Reed M. N. (2015). P301L tau expression affects glutamate release and clearance in the hippocampal trisynaptic pathway. J. Neurochem. 132, 169–182. 10.1111/jnc.12967 25319522 PMC4302046

[B39] Ianov L. Rani A. Beas B. S. Kumar A. Foster T. C. (2016). Transcription profile of aging and cognition-related genes in the medial prefrontal cortex. Front. Aging Neurosci. 8:113. 10.3389/fnagi.2016.00113 27242522 PMC4868850

[B40] Ianov L. Riva A. Kumar A. Foster T. C. (2017). DNA methylation of synaptic genes in the prefrontal cortex is associated with aging and age-related cognitive impairment. Front. Aging Neurosci. 9:249. 10.3389/fnagi.2017.00249 28824413 PMC5539085

[B41] Iijima T. Miura E. Watanabe M. Yuzaki M. (2010). Distinct expression of C1q-like family mRNAs in mouse brain and biochemical characterization of their encoded proteins. Eur. J. Neurosci. 31, 1606–1615. 10.1111/j.1460-9568.2010.07202.x 20525073

[B42] Ivanova O. Y. Dobryakova Y. V. Salozhin S. V. Aniol V. A. Onufriev M. V. Gulyaeva N. V.. (2016). Lentiviral modulation of Wnt/beta-catenin signaling affects *in vivo* LTP. Cell Mol. Neurobiol. 37, 1227–1241. 10.1007/s10571-016-0455-z 28012021 PMC11482074

[B43] Jackson T. C. Rani A. Kumar A. Foster T. C. (2009). Regional hippocampal differences in AKT survival signaling across the lifespan: implications for CA1 vulnerability with aging. Cell Death Differ. 16, 439–448. 10.1038/cdd.2008.171 19039330 PMC2680608

[B44] Jessri M. Farah C. S. (2014). Next generation sequencing and its application in deciphering head and neck cancer. Oral Oncol. 50, 247–253. 10.1016/j.oraloncology.2013.12.017 24440145

[B45] Kadish I. Thibault O. Blalock E. M. Chen K. C. Gant J. C. Porter N. M.. (2009). Hippocampal and cognitive aging across the lifespan: a bioenergetic shift precedes and increased cholesterol trafficking parallels memory impairment. J. Neurosci. 29, 1805–1816. 10.1523/JNEUROSCI.4599-08.2009 19211887 PMC2661568

[B46] Kesner R. P. Lee I. Gilbert P. (2004). A behavioral assessment of hippocampal function based on a subregional analysis. Rev. Neurosci. 15, 333–351. 10.1515/REVNEURO.2004.15.5.333 15575490

[B47] Kesner R. P. Rolls E. T. (2015). A computational theory of hippocampal function, and tests of the theory: new developments. Neurosci. Biobehav. Rev. 48, 92–147. 10.1016/j.neubiorev.2014.11.009 25446947

[B48] Kuhn H. G. Dickinson-Anson H. Gage F. H. (1996). Neurogenesis in the dentate gyrus of the adult rat: age-related decrease of neuronal progenitor proliferation. J. Neurosci. 16, 2027–2033. 8604047 10.1523/JNEUROSCI.16-06-02027.1996 PMC6578509

[B49] Kusko R. LeClerc A. Swahn S. Lenburg M. Spira A. (2014). Comparison of illumina and ion torrent RNA-sequencing and microarray-based approaches for profiling the transcriptome. J. Biomol. Tech. 25, S14.

[B50] Landfield P. W. (1988). Hippocampal neurobiological mechanisms of age-related memory dysfunction. Neurobiol. Aging 9, 571–579. 10.1016/S0197-4580(88)80116-7 3062468

[B51] Lein E. S. Zhao X. Gage F. H. (2004). Defining a molecular atlas of the hippocampus using DNA microarrays and high-throughput *in situ* hybridization. J. Neurosci. 24, 3879–3889. 10.1523/JNEUROSCI.4710-03.2004 15084669 PMC6729356

[B52] Lemaire V. Koehl M. Le Moal M. Abrous D. N. (2000). Prenatal stress produces learning deficits associated with an inhibition of neurogenesis in the hippocampus. Proc. Natl. Acad. Sci. U.S.A. 97, 11032–11037. 10.1073/pnas.97.20.11032 11005874 PMC27143

[B53] Li W. Turner A. Aggarwal P. Matter A. Storvick E. Arnett D. K.. (2015). Comprehensive evaluation of AmpliSeq transcriptome, a novel targeted whole transcriptome RNA sequencing methodology for global gene expression analysis. BMC Genomics 16:1069. 10.1186/s12864-015-2270-1 26673413 PMC4681149

[B54] Liu Y. Helms A. W. Johnson J. E. (2004). Distinct activities of Msx1 and Msx3 in dorsal neural tube development. Development 131, 1017–1028. 10.1242/dev.00994 14973289

[B55] Lu T. Pan Y. Kao S. Y. Li C. Kohane I. Chan J.. (2004). Gene regulation and DNA damage in the ageing human brain. Nature 429, 883–891. 10.1038/nature02661 15190254

[B56] Maass A. Schütze H. Speck O. Yonelinas A. Tempelmann C. Heinze H. J.. (2014). Laminar activity in the hippocampus and entorhinal cortex related to novelty and episodic encoding. Nat. Commun. 5, 5547. 10.1038/ncomms6547 25424131 PMC4263140

[B57] Masser D. R. Bixler G. V. Brucklacher R. M. Yan H. Giles C. B. Wren J. D.. (2014). Hippocampal subregions exhibit both distinct and shared transcriptomic responses to aging and nonneurodegenerative cognitive decline. J. Gerontol. A Biol. Sci. Med. Sci. 69, 1311–1324. 10.1093/gerona/glu091 24994846 PMC4271093

[B58] Mazarati A. Lundström L. Sollenberg U. Shin D. Langel U. Sankar R. (2006). Regulation of kindling epileptogenesis by hippocampal galanin type 1 and type 2 receptors: the effects of subtype-selective agonists and the role of G-protein-mediated signaling. J. Pharmacol. Exp. Ther. 318, 700–708. 10.1124/jpet.106.104703 16699066

[B59] McColl C. D. Jacoby A. S. Shine J. Iismaa T. P. Bekkers J. M. (2006). Galanin receptor-1 knockout mice exhibit spontaneous epilepsy, abnormal EEGs and altered inhibition in the hippocampus. Neuropharmacology 50, 209–218. 10.1016/j.neuropharm.2005.09.001 16243364

[B60] Mueller S. G. Weiner M. W. (2009). Selective effect of age, Apo e4, and Alzheimer's disease on hippocampal subfields. Hippocampus 19, 558–564. 10.1002/hipo.20614 19405132 PMC2802542

[B61] Newrzella D. Pahlavan P. S. Krüger C. Boehm C. Sorgenfrei O. Schröck H.. (2007). The functional genome of CA1 and CA3 neurons under native conditions and in response to ischemia. BMC Genomics 8:370. 10.1186/1471-2164-8-370 17937787 PMC2194787

[B62] Oh M. M. Disterhoft J. F. (2010). Cellular mechanisms for altered learning in aging. Future Neurol. 5, 147–155. 10.2217/fnl.09.74 23525658 PMC3606028

[B63] Penner M. R. Roth T. L. Chawla M. K. Hoang L. T. Roth E. D. Lubin F. D.. (2011). Age-related changes in Arc transcription and DNA methylation within the hippocampus. Neurobiol. Aging 32, 2198–2210. 10.1016/j.neurobiolaging.2010.01.009 20189687 PMC2888808

[B64] Reuter J. A. Spacek D. V. Snyder M. P. (2015). High-throughput sequencing technologies. Mol. Cell 58, 586–597. 10.1016/j.molcel.2015.05.004 26000844 PMC4494749

[B65] Rogalski E. Stebbins G. T. Barnes C. A. Murphy C. M. Stoub T. R. George S.. (2012). Age-related changes in parahippocampal white matter integrity: a diffusion tensor imaging study. Neuropsychologia 50, 1759–1765. 10.1016/j.neuropsychologia.2012.03.033 22561887 PMC3576131

[B66] Rolls E. T. Kesner R. P. (2006). A computational theory of hippocampal function, and empirical tests of the theory. Prog. Neurobiol. 79, 1–48. 10.1016/j.pneurobio.2006.04.005 16781044

[B67] Rönnlund M. Nyberg L. Bäckman L. Nilsson L. G. (2005). Stability, growth, and decline in adult life span development of declarative memory: cross-sectional and longitudinal data from a population-based study. Psychol. Aging 20, 3–18. 10.1037/0882-7974.20.1.3 15769210

[B68] Rowe W. B. Blalock E. M. Chen K. C. Kadish I. Wang D. Barrett J. E.. (2007). Hippocampal expression analyses reveal selective association of immediate-early, neuroenergetic, and myelinogenic pathways with cognitive impairment in aged rats. J. Neurosci. 27, 3098–3110. 10.1523/JNEUROSCI.4163-06.2007 17376971 PMC6672456

[B69] Rozovsky I. Wei M. Morgan T. E. Finch C. E. (2005). Reversible age impairments in neurite outgrowth by manipulations of astrocytic GFAP. Neurobiol. Aging 26, 705–715. 10.1016/j.neurobiolaging.2004.06.009 15708446

[B70] Shi L. Argenta A. E. Winseck A. K. Brunso-Bechtold J. K. (2004). Stereological quantification of GAD-67-immunoreactive neurons and boutons in the hippocampus of middle-aged and old Fischer 344 x Brown Norway rats. J. Comp. Neurol. 478, 282–291. 10.1002/cne.20303 15368530

[B71] Sochocka M. Diniz B. S. Leszek J. (2017). Inflammatory response in the CNS: friend or foe? Mol. Neurobiol. 54, 8071–8089. 10.1007/s12035-016-0297-1 27889895 PMC5684251

[B72] Stanley D. P. Shetty A. K. (2004). Aging in the rat hippocampus is associated with widespread reductions in the number of glutamate decarboxylase-67 positive interneurons but not interneuron degeneration. J. Neurochem. 89, 204–216. 10.1111/j.1471-4159.2004.02318.x 15030405

[B73] Streit W. J. Xue Q. S. Braak H. del Tredici K. (2014). Presence of severe neuroinflammation does not intensify neurofibrillary degeneration in human brain. Glia 62, 96–105. 10.1002/glia.22589 24272705

[B74] Toescu E. C. Verkhratsky A. Landfield P. W. (2004). Ca^2+^ regulation and gene expression in normal brain aging. Trends Neurosci. 27, 614–620. 10.1016/j.tins.2004.07.010 15374673

[B75] Uddin R. K. Singh S. M. (2013). Hippocampal gene expression meta-analysis identifies aging and age-associated spatial learning impairment (ASLI) genes and pathways. PLoS ONE 8:e69768. 10.1371/journal.pone.0069768 23874995 PMC3715497

[B76] Uttl B. Graf P. (1993). Episodic spatial memory in adulthood. Psychol. Aging 8, 257–273. 10.1037/0882-7974.8.2.257 8323729

[B77] Valente T. Domínguez M. I. Bellmann A. Journot L. Ferrer I. Auladell C. (2004). Zac1 is up-regulated in neural cells of the limbic system of mouse brain following seizures that provoke strong cell activation. Neuroscience 128, 323–336. 10.1016/j.neuroscience.2004.06.056 15350644

[B78] Varela-Nallar L. Inestrosa N. C. (2013). Wnt signaling in the regulation of adult hippocampal neurogenesis. Front. Cell. Neurosci. 7:100. 10.3389/fncel.2013.00100 23805076 PMC3693081

[B79] Volk L. Chiu S. L. Sharma K. Huganir R. L. (2015). Glutamate synapses in human cognitive disorders. Annu. Rev. Neurosci. 38, 127–149. 10.1146/annurev-neuro-071714-033821 25897873

[B80] Wang F. Diana R. A. (2016). Temporal context processing within hippocampal subfields. Neuroimage 134, 261–269. 10.1016/j.neuroimage.2016.03.048 27039142 PMC4912848

[B81] Wang X. Zaidi A. Pal R. Garrett A. S. Braceras R. Chen X. W.. (2009). Genomic and biochemical approaches in the discovery of mechanisms for selective neuronal vulnerability to oxidative stress. BMC Neurosci. 10:12. 10.1186/1471-2202-10-12 19228403 PMC2677396

[B82] Wisse L. E. Adler D. H. Ittyerah R. Pluta J. B. Robinson J. L. Schuck T.. (2016). Comparison of *in vivo* and *ex vivo* MRI of the human hippocampal formation in the same subjects. Cereb. Cortex 27, 5185–5196. 10.1093/cercor/bhw299 27664967 PMC6075210

[B83] Xu N. Zhou W. J. Wang Y. Huang S. H. Li X. Chen Z. Y. (2015). Hippocampal Wnt3a is necessary and sufficient for contextual fear memory acquisition and consolidation. Cereb. Cortex 25, 4062–4075. 10.1093/cercor/bhu121 24904070 PMC4626827

[B84] Yassa M. A. Mattfeld A. T. Stark S. M. Stark C. E. (2011). Age-related memory deficits linked to circuit-specific disruptions in the hippocampus. Proc. Natl. Acad. Sci. U.S.A. 108, 8873–8878. 10.1073/pnas.1101567108 21555581 PMC3102362

[B85] Yushkevich P. A. Amaral R. S. Augustinack J. C. Bender A. R. Bernstein J. D. Boccardi M.. (2015). Quantitative comparison of 21 protocols for labeling hippocampal subfields and parahippocampal subregions in *in vivo* MRI: towards a harmonized segmentation protocol. Neuroimage 111, 526–541. 10.1016/j.neuroimage.2015.01.004 25596463 PMC4387011

[B86] Zeier Z. Madorsky I. Xu Y. Ogle W. O. Notterpek L. Foster T. C. (2011). Gene expression in the hippocampus: regionally specific effects of aging and caloric restriction. Mech. Ageing Dev. 132, 8–19. 10.1016/j.mad.2010.10.006 21055414 PMC3032012

