# Supplementary figures and images for: Hippocampal Transcriptomic Profiles: Subfield Vulnerability to Age and Cognitive Impairment
Source: Front Aging Neurosci. 2017 Dec 8;9:383. doi: 10.3389/fnagi.2017.00383 (PMC5727020; doi:10.3389/fnagi.2017.00383)

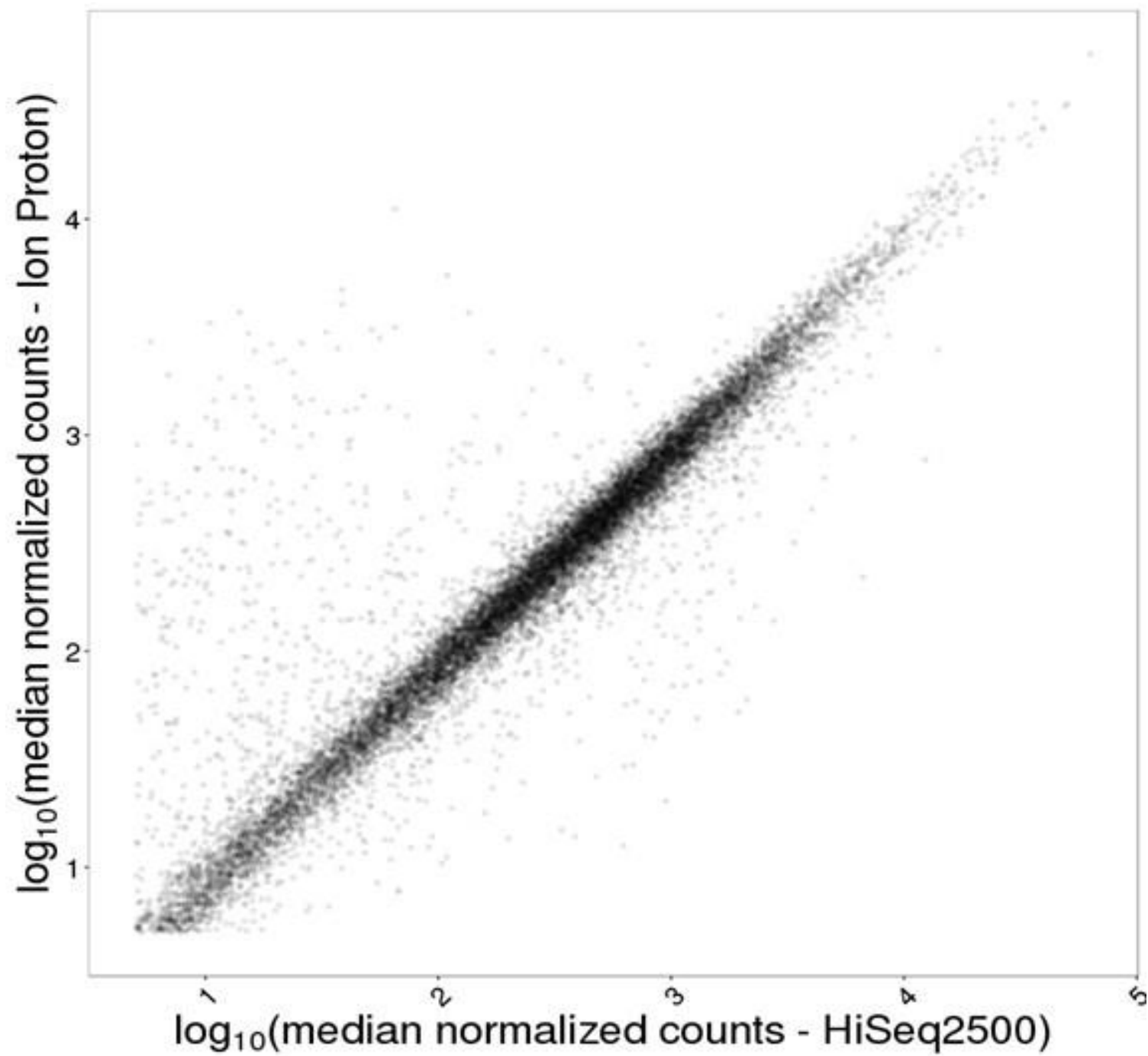

Supplement: Supplementary Figure 1 — Correlation (R2 = 0.938) of gene counts across sequencing platforms, Illumina and Ion Proton. [file Image1.PDF]

# Subregion specific transcriptomic profile

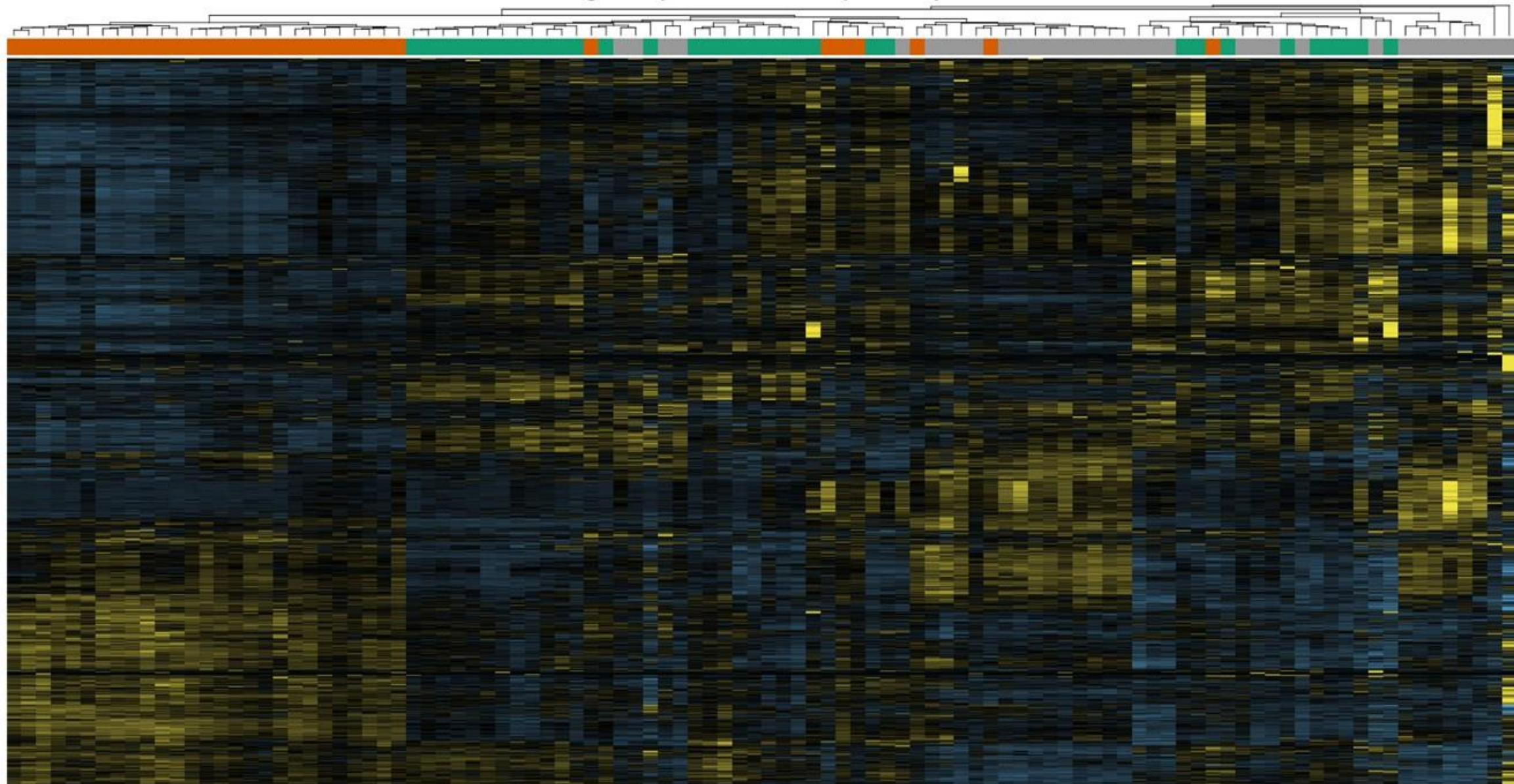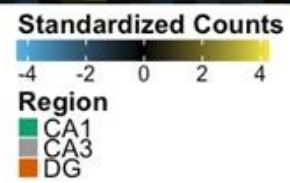

Supplement: Supplementary Figure 2 — Heatmap of hippocampal subfield changes in gene expression. Plotted for each hippocampal subfield, are genes found with both sequencing platforms to be differentially expressed compared to the other two subfields at a significance of adj-p < 0.05. Each row represents the z-scores from the DEGs, and the columns are the biological replicates for each region. [file Image2.PDF]

*Wfs1*

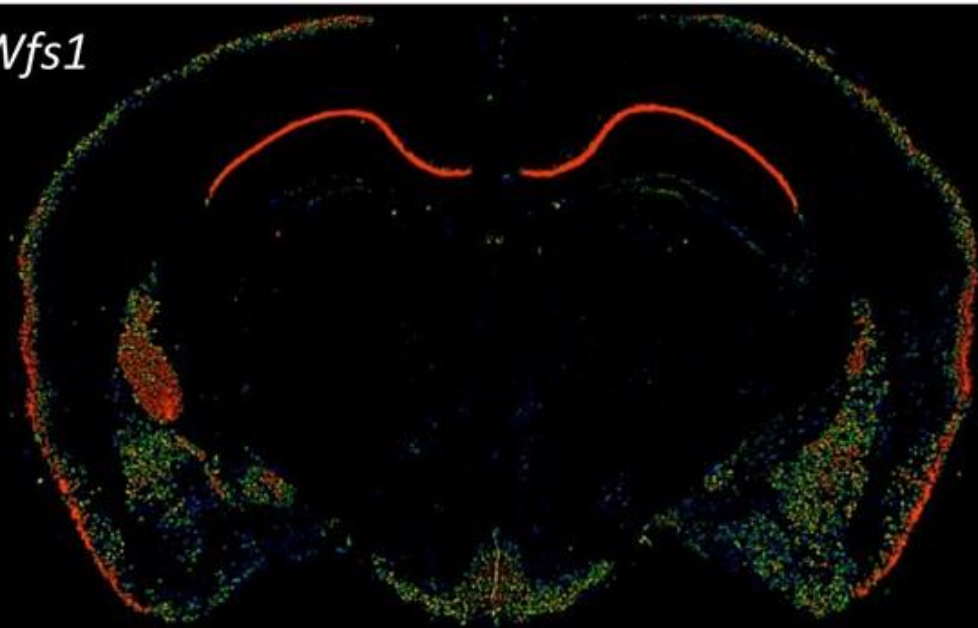

*Nov*

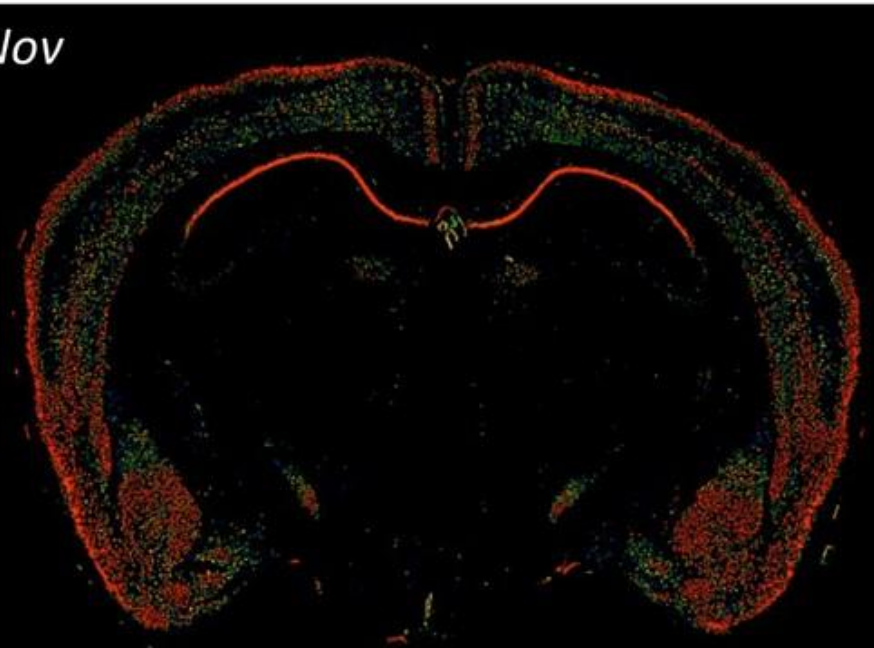

*Ndst4*

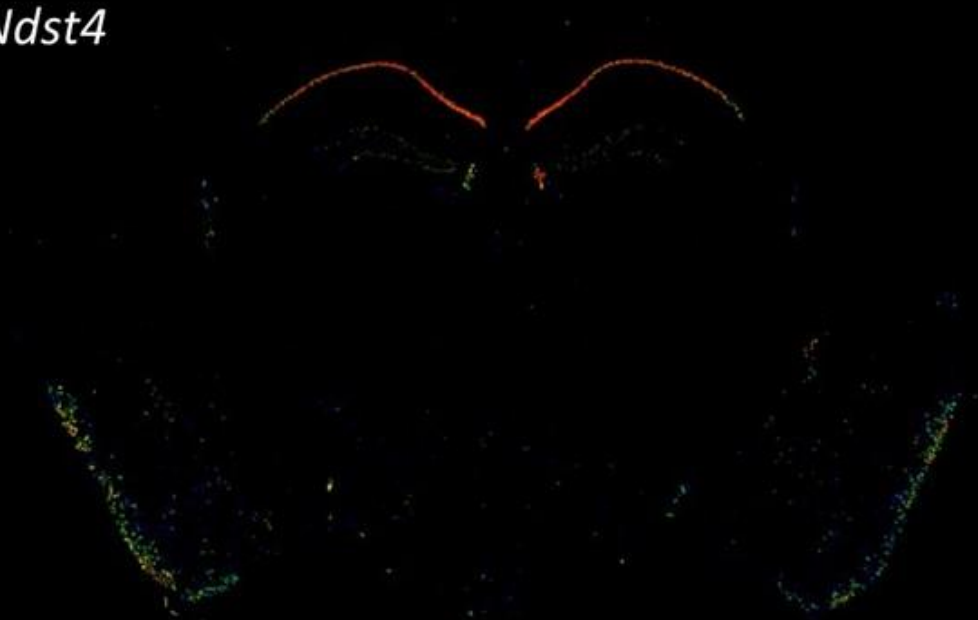

*Gpr161*

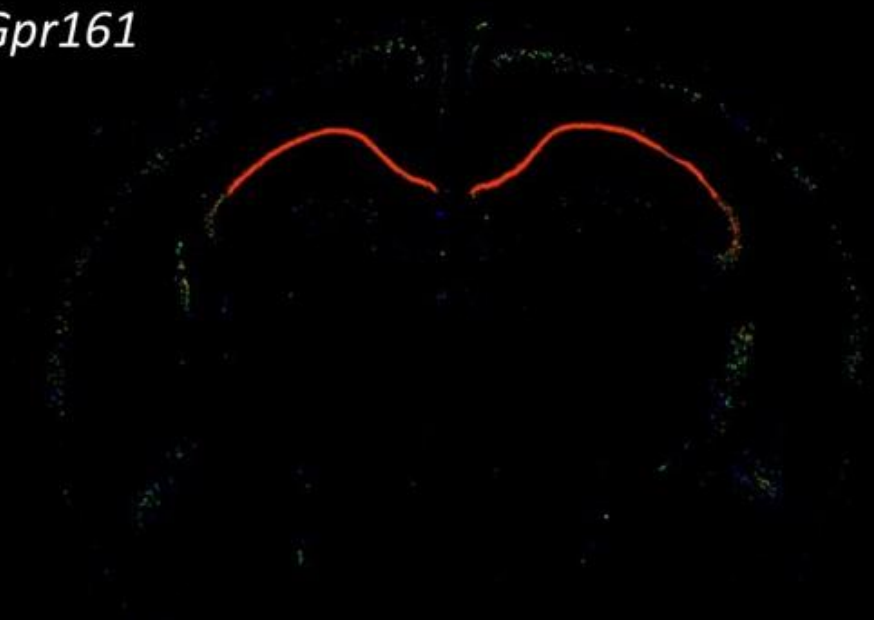

Supplement: Supplementary Figure 4 — Expression of genes Wsf1, Nov, Ndst4, and Gpr161, (specific to CA1 in our study) as reported in the Allen Brain Atlas (Mouse). [file Image4.PDF]

*Col6a6*

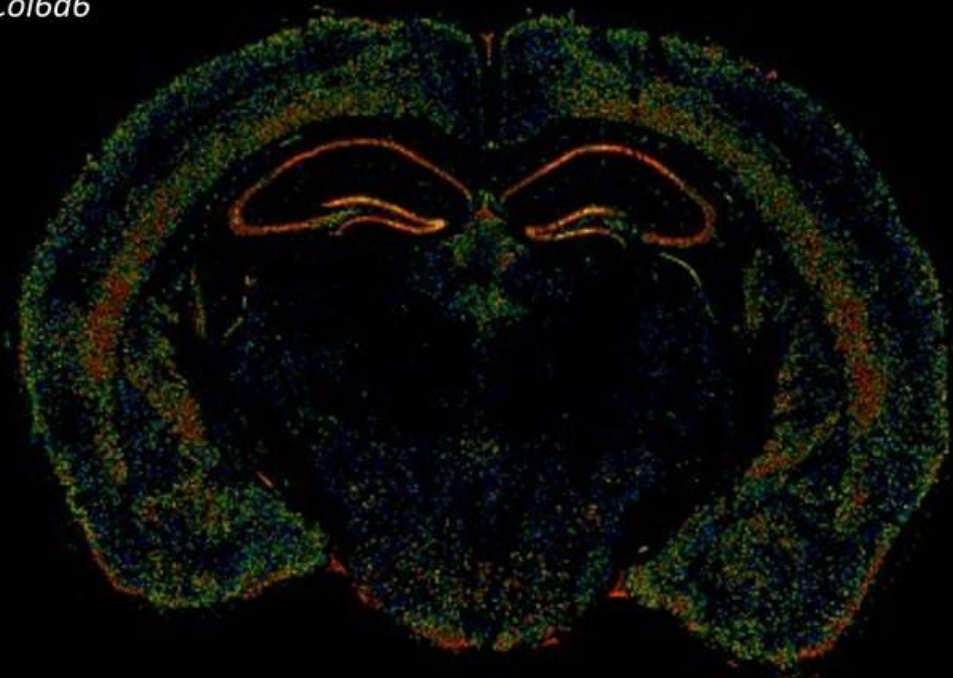

*Nnat*

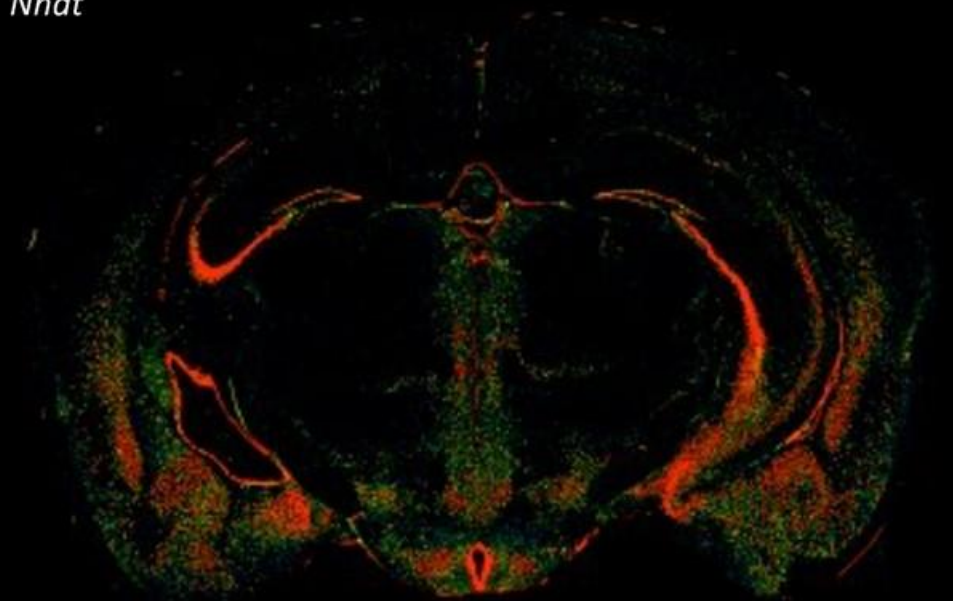

Supplement: Supplementary Figure 6 — Expression of genes Col6a6 and Nnat (specific to CA3 in our study) as reported in the Allen Brain Atlas (Mouse). [file Image6.PDF]

*Pdin*

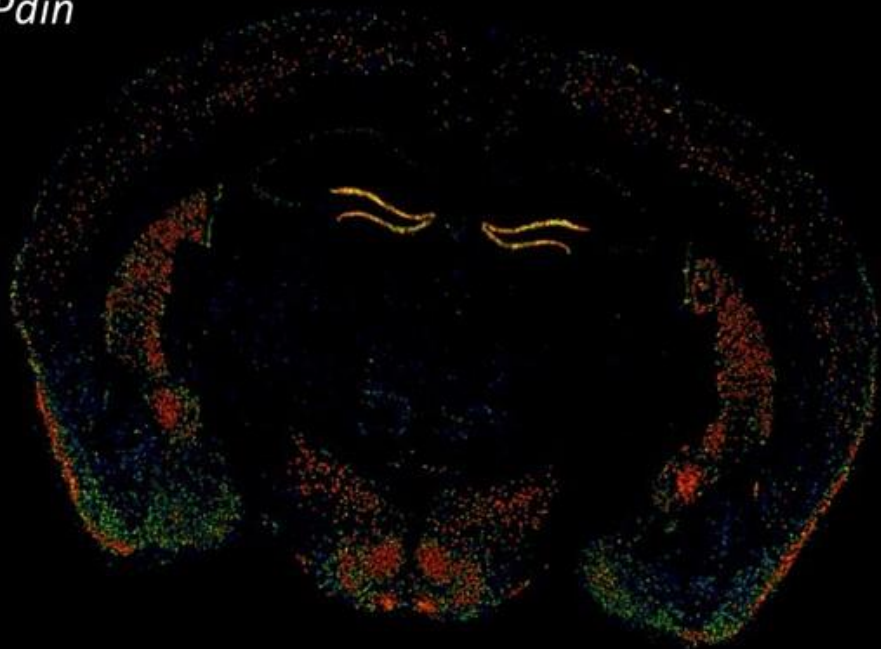

*Plk5*

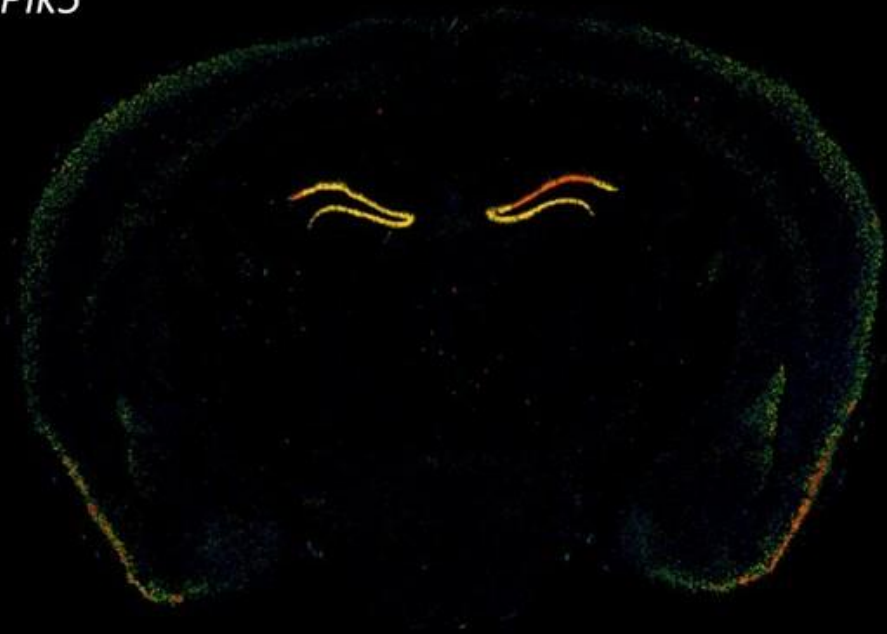

*C1ql2*

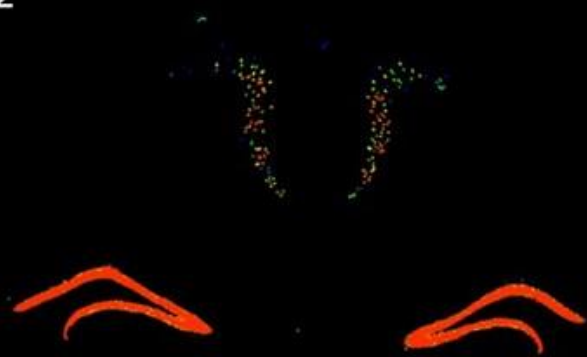

*Dsp*

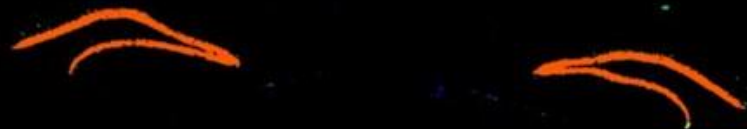

Supplement: Supplementary Figure 8 — Expression of genes Pdin, Plk5, C1ql2, and Dsp (specific to DG in our study) as reported in the Allen Brain Atlas (Mouse). [file Image8.PDF]

# Transcriptomic profile of the aging CA1

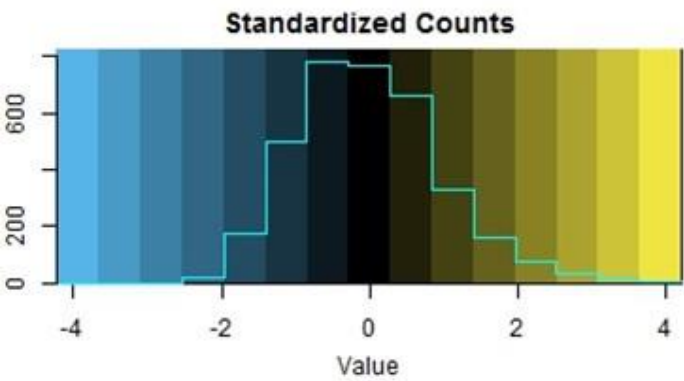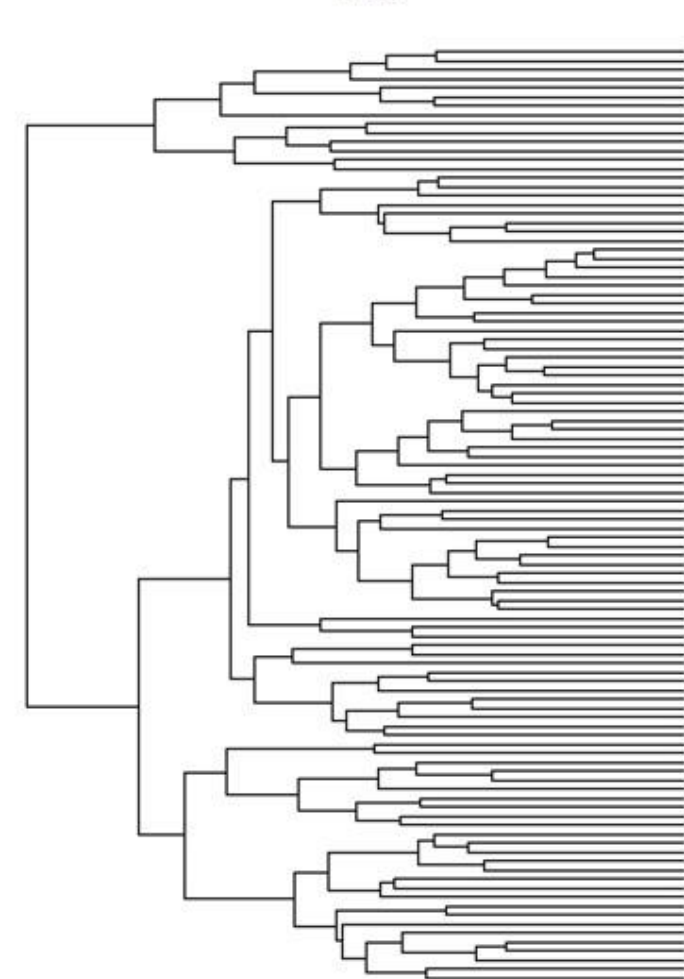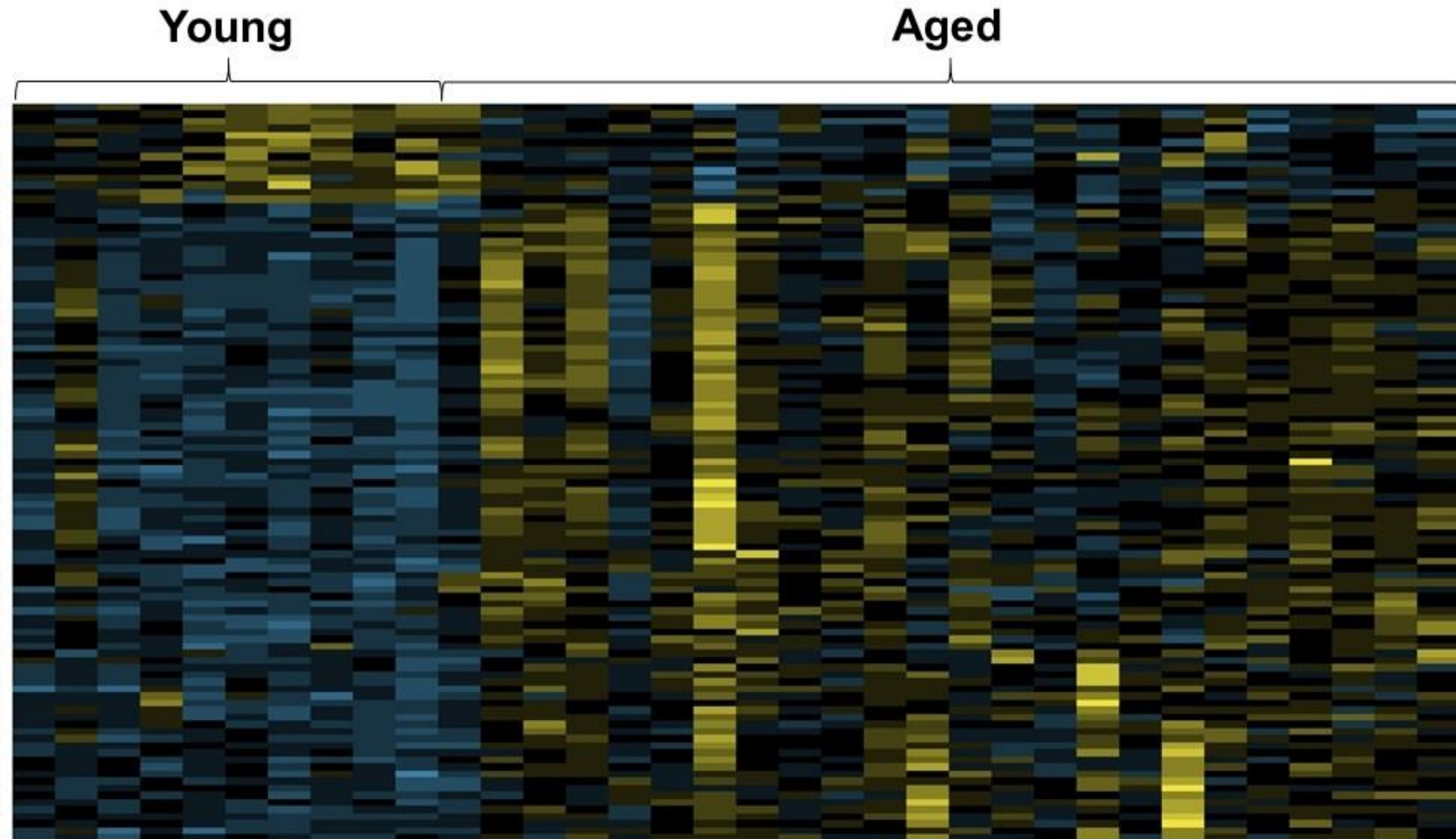

Supplement: Supplementary Figure 9 — Heatmap of age-related changes in gene expression in the CA1 region. Each row represents a DEG (Illumina p < 0.01; Ion Proton p < 0.05) associated with aging. Gene-level counts were standardized to z-scores and the color represents the standard deviation increasing (yellow) or decreasing (blue) relative to the mean (black). The age-related gene enrichment clusters (FDR adj-p < 0.05) in the CA1 region included immune related GOs, neutrophil activation, cell adhesion and regulation of ERK1 and ERK2 cascade. [file Image9.PDF]

# Transcriptomic profile of the aging CA3

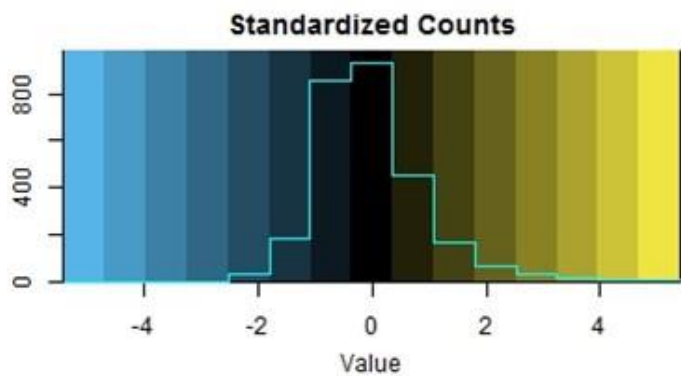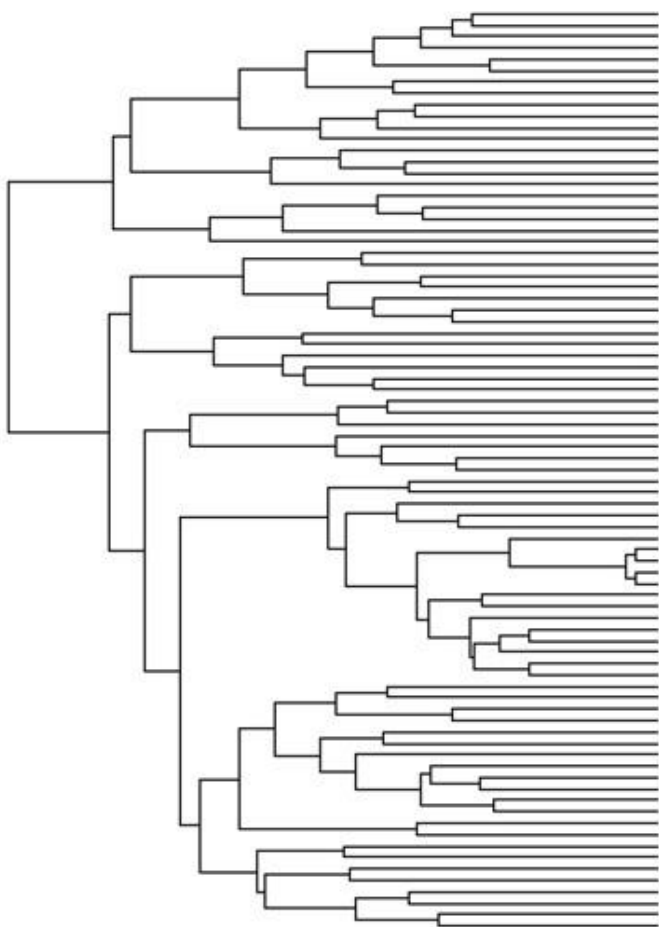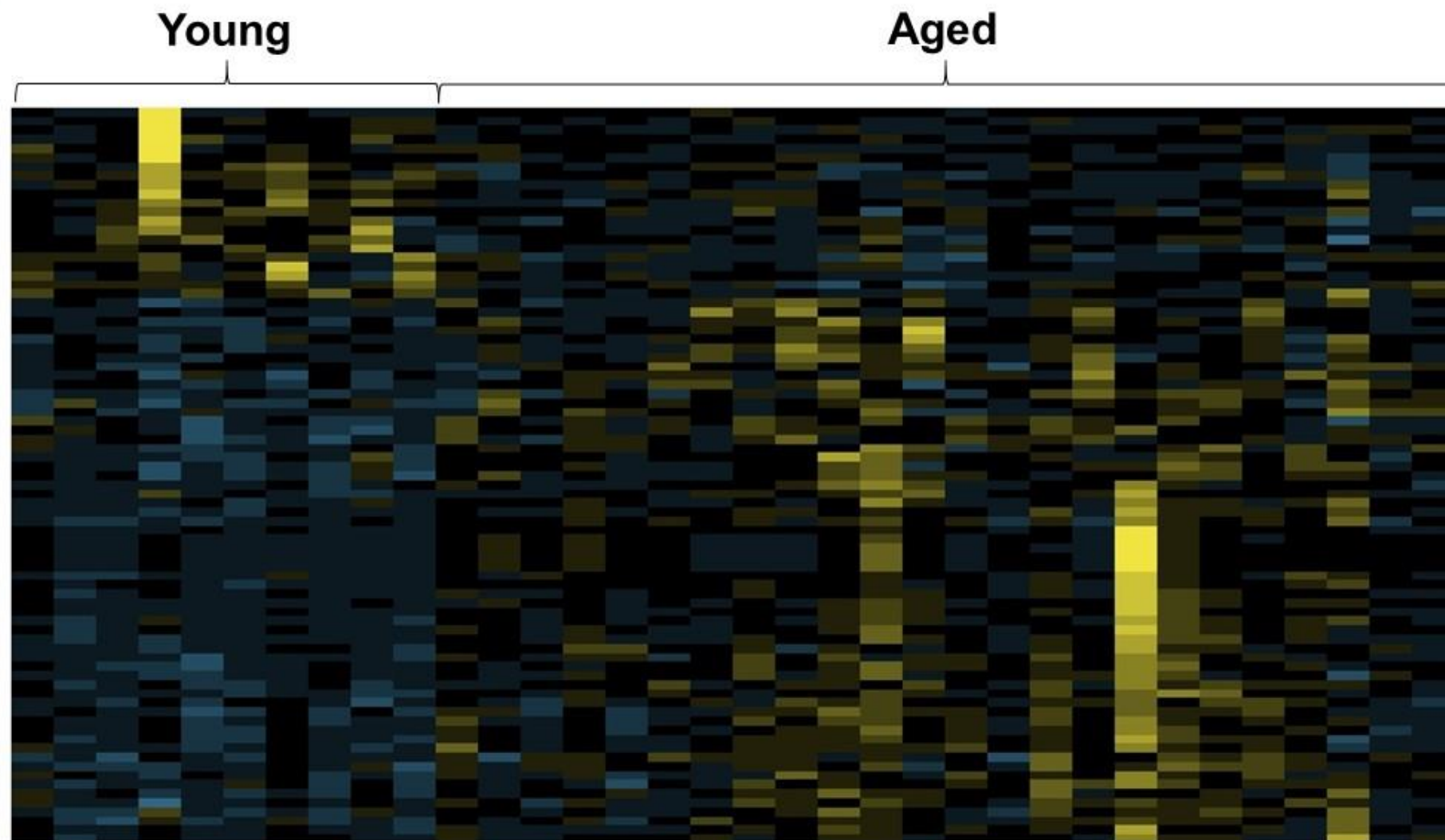

Supplement: Supplementary Figure 10 — Heatmap of age-related changes in gene expression in the CA3 region. Each row represents a DEG (Illumina p < 0.01; Ion Proton p < 0.05) associated with aging. Gene-level counts were standardized to z-scores and the color represents the standard deviation increasing (yellow) or decreasing (blue) relative to the mean (black). The age-related gene enrichment clusters (FDR adj-p < 0.05) in the CA3 region included immune related GOs such as leukocyte activation, cytokine production, and antigen processing and presentation. [file Image10.PDF]

# Transcriptomic profile of the aging DG

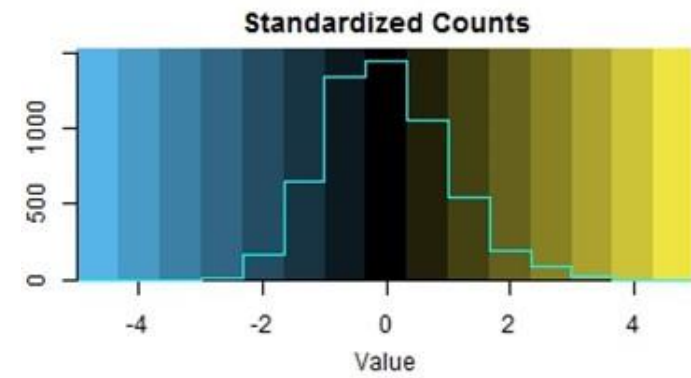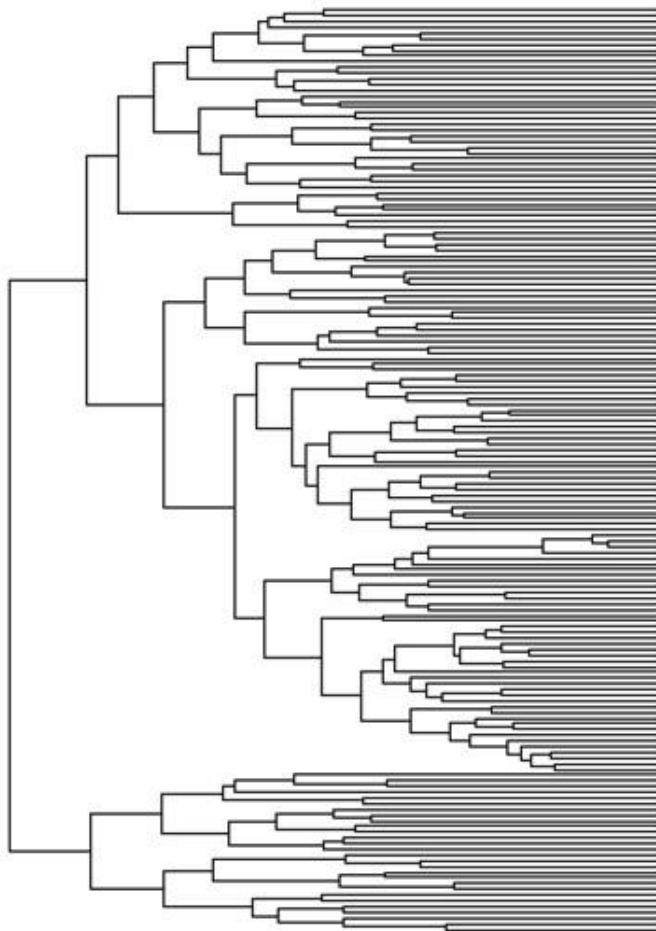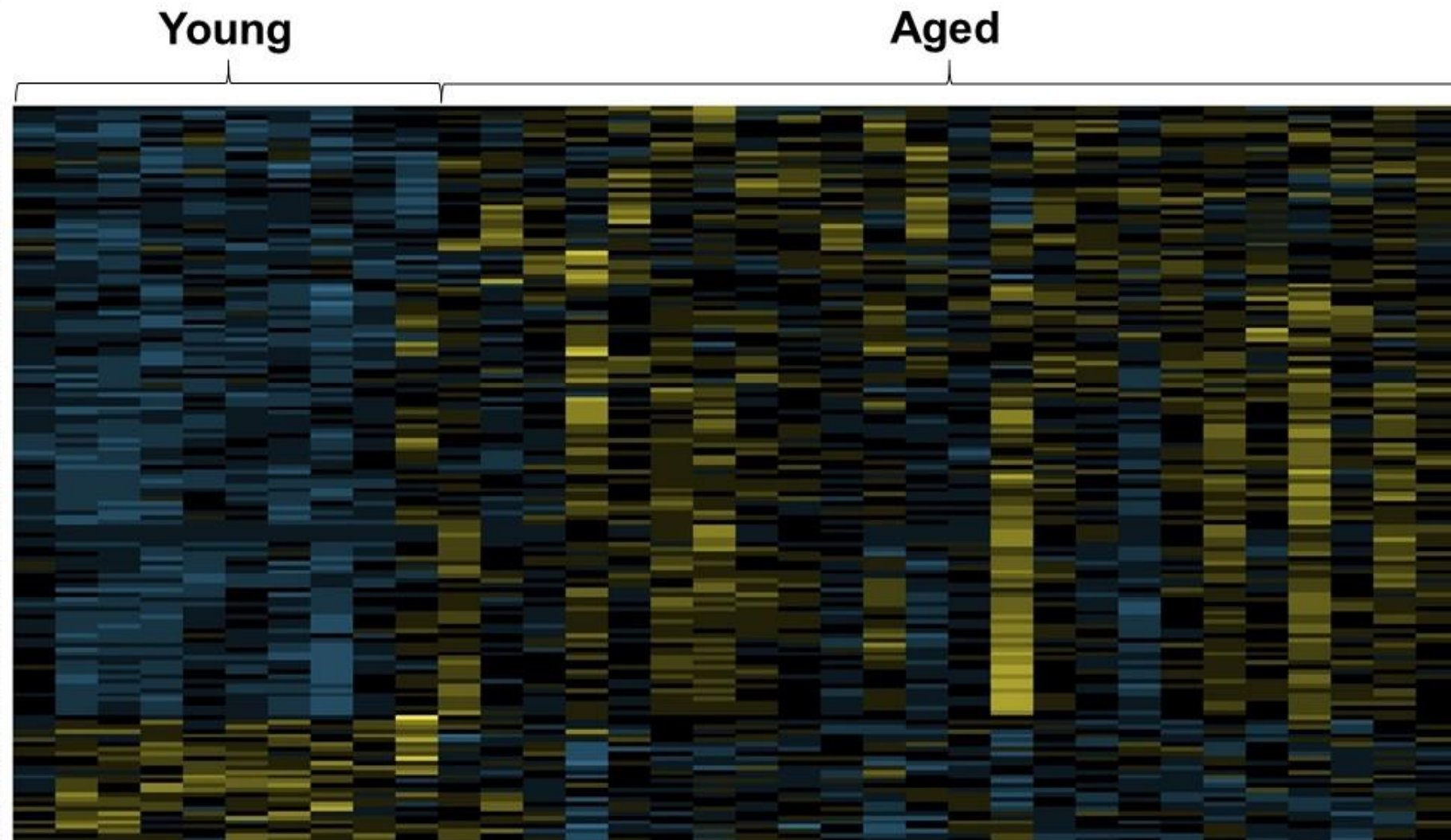

Supplement: Supplementary Figure 11 — Heatmap of age-related changes in gene expression in the DG region. Each row represents a DEG (Illumina p < 0.01; Ion Proton p < 0.05) associated with aging. Gene-level counts were standardized to z-scores and the color represents the standard deviation increasing (yellow) or decreasing (blue) relative to the mean (black). The age-related gene enrichment clusters (FDR adj-p < 0.05) in the DG region included immune related GOs such as defense response, and inflammatory response. Cell-cell adhesion GO was also detected. [file Image11.PDF]
