# Supplementary material for: Hippocampal Transcriptomic Profiles: Subfield Vulnerability to Age and Cognitive Impairment
Source: Front Aging Neurosci. 2017 Dec 8;9:383. doi: 10.3389/fnagi.2017.00383 (PMC5727020; doi:10.3389/fnagi.2017.00383)
Supplement: Supplementary Figure 3 — Boxplots for the CA1 specific genes reported in Table S4. The normalized counts are from the Illumina experiment, and the genes are ranked according the average Fold Change computed from the pairwise comparisons CA1 vs. CA3 and CA1 vs. DG. [file Image3.PDF]

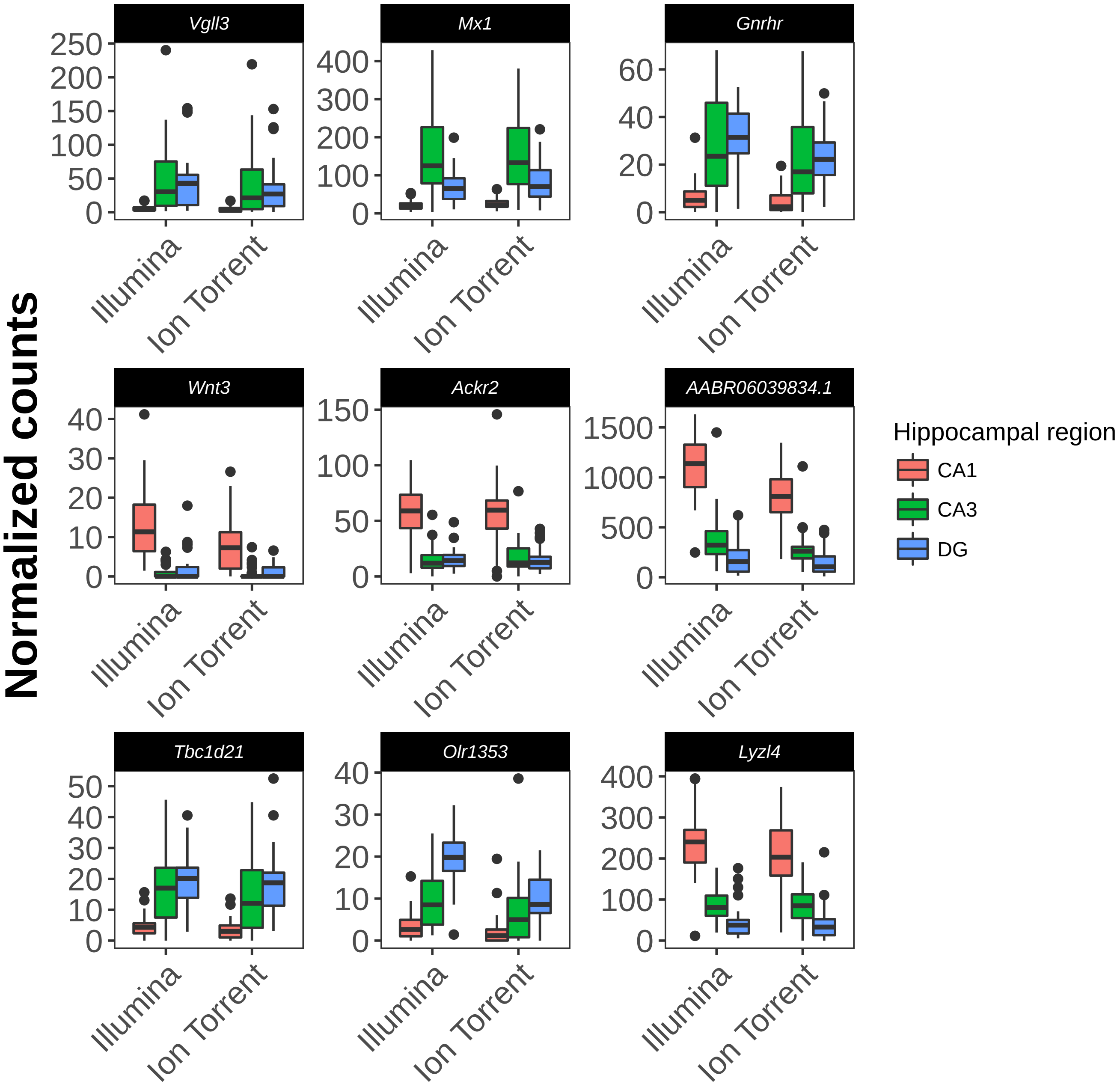
Normalized counts
Vgll3
Mx1
Gnrhr
Wnt3
Ackr2
AABR06039834.1
Tbc1d21
Olr1353
Lyzl4
Illumina
Ion Torrent
Hippocampal region
CA1
CA3
DG

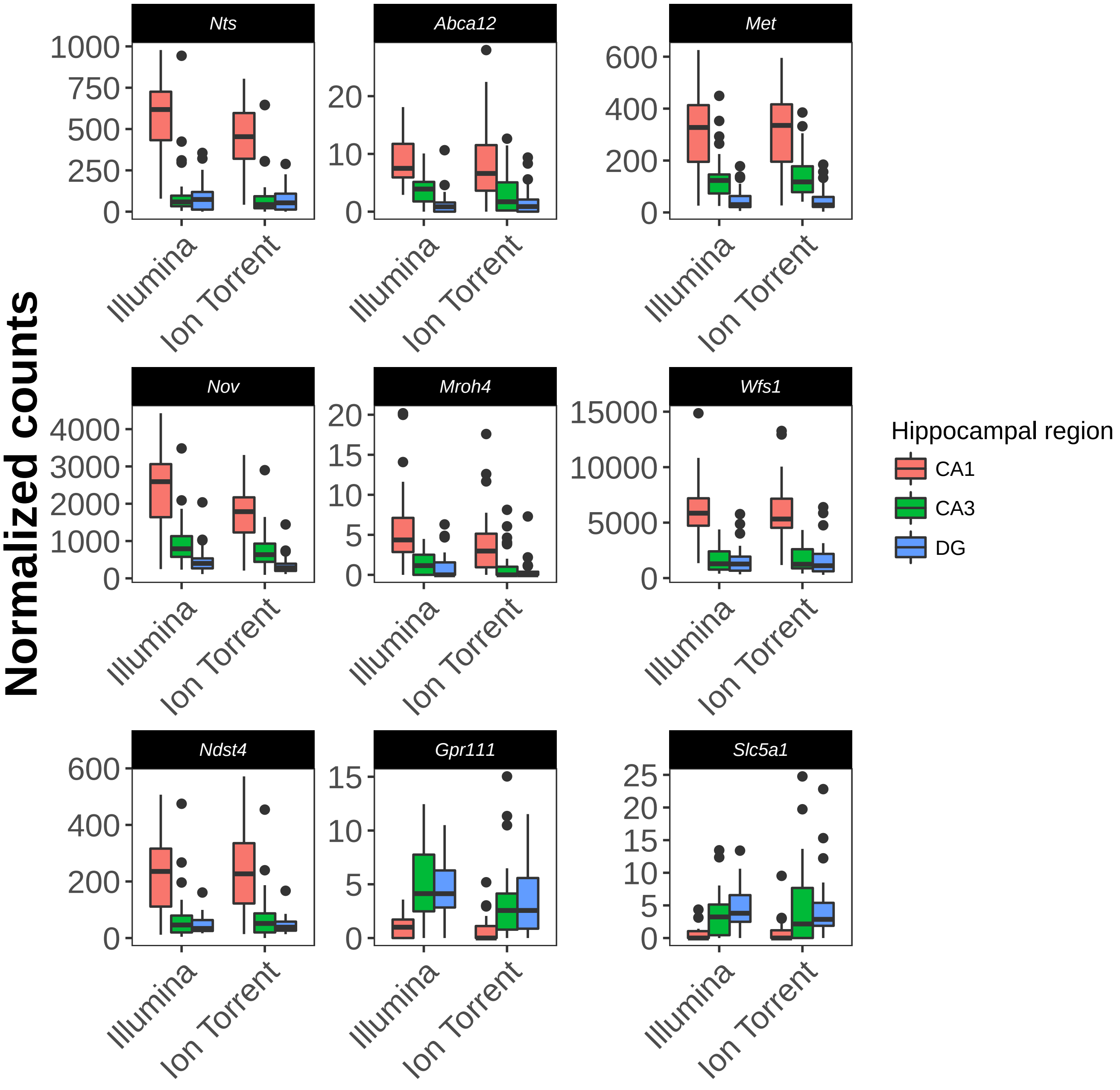
Normalized counts
Nts
Abca12
Met
Nov
Mroh4
Wfs1
Ndst4
Gpr111
Slc5a1
Illumina
Ion Torrent
Hippocampal region
CA1
CA3
DG

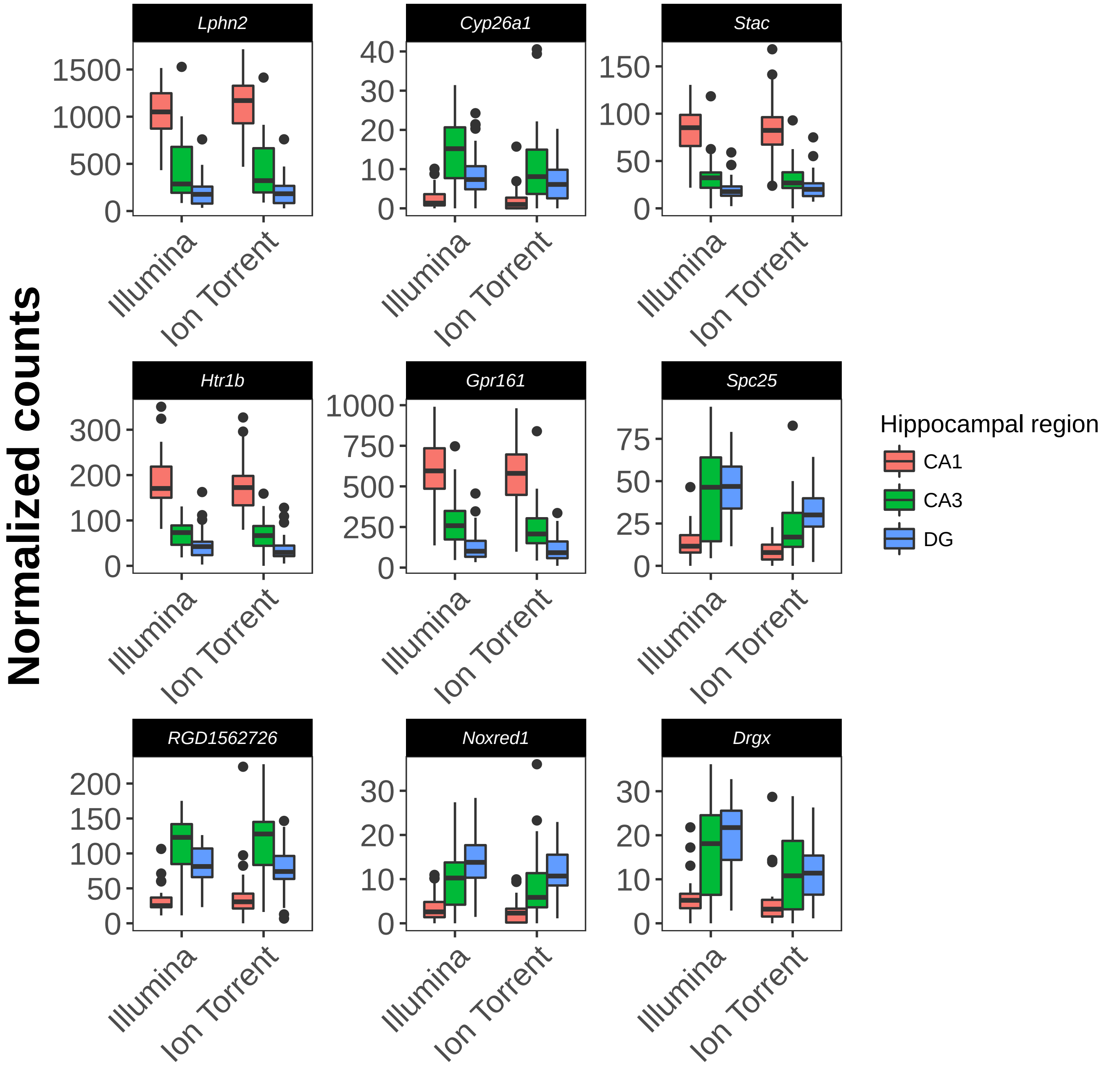
Normalized counts
Lphn2
Cyp26a1
Stac
Htr1b
Gpr161
Spc25
RGD1562726
Noxred1
Drgx
Illumina
Ion Torrent
Hippocampal region
CA1
CA3
DG

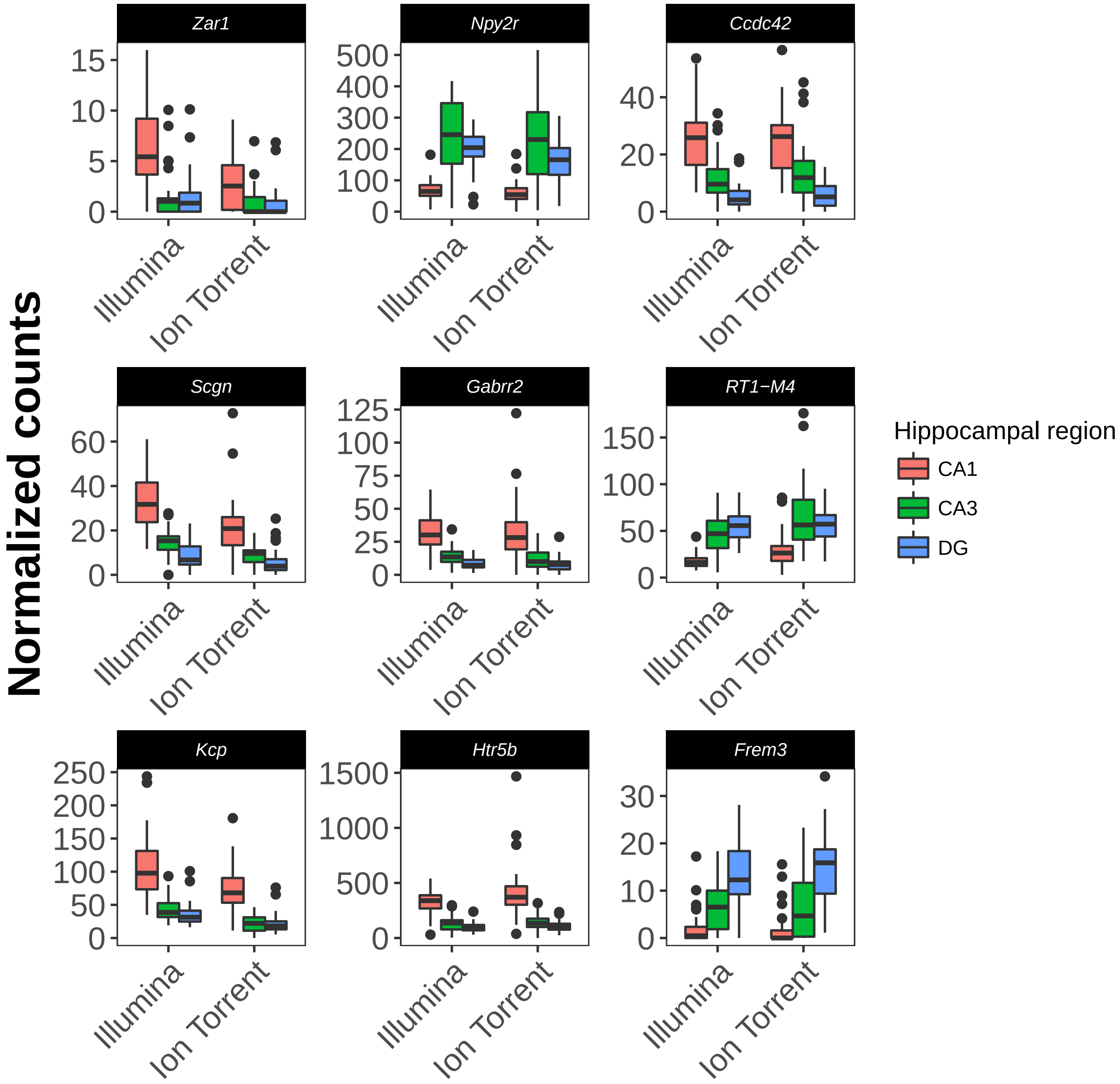

Normalized counts
Zar1
Npy2r
Ccdc42
Scgn
Gabrr2
RT1−M4
Kcp
Htr5b
Frem3
Illumina
Ion Torrent
Hippocampal region
CA1
CA3
DG

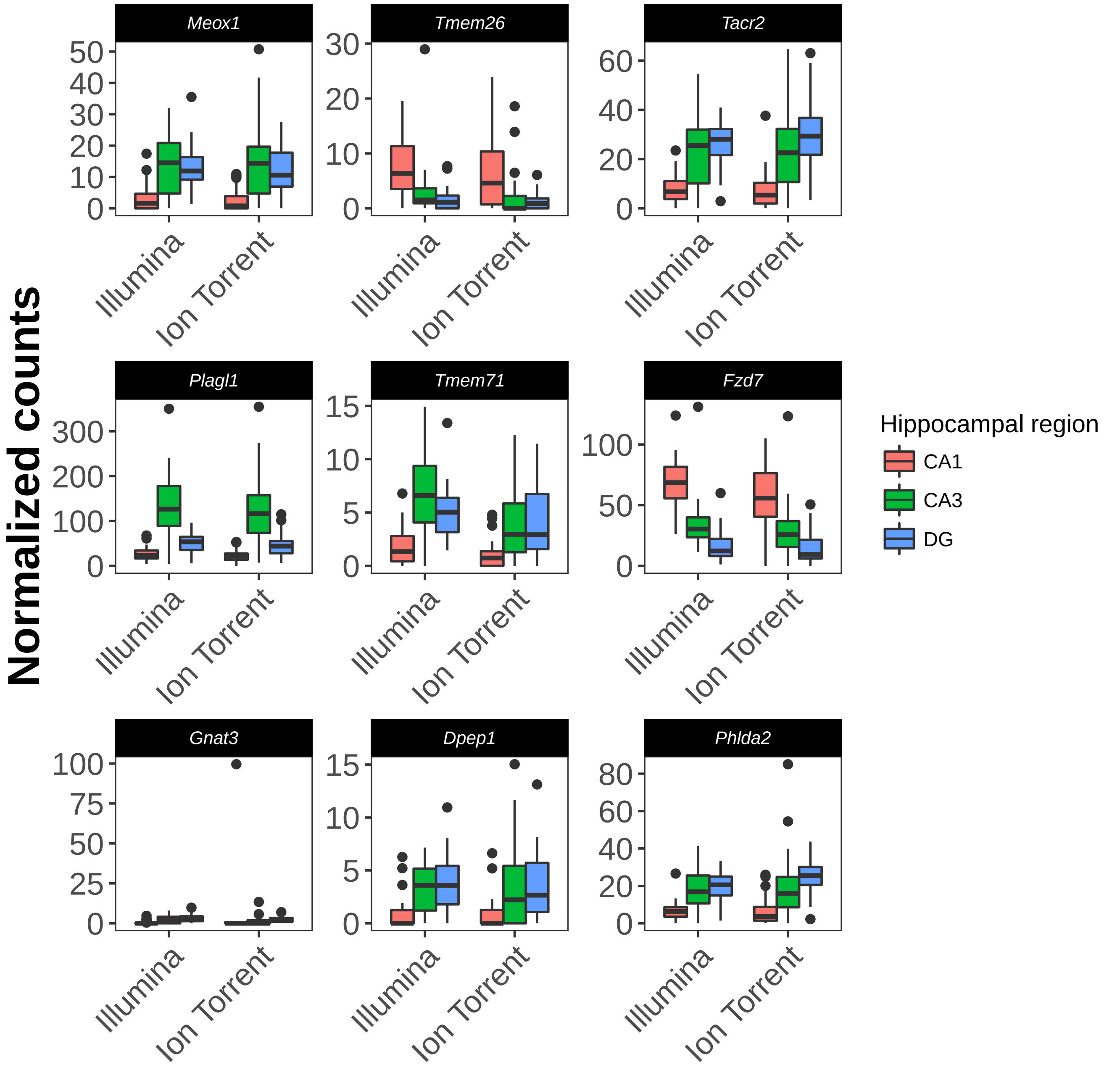

Normalized counts
Meox1
Tmem26
Tacr2
Plagl1
Tmem71
Fzd7
Gnat3
Dpep1
Phlda2
Illumina
Ion Torrent
Hippocampal region
CA1
CA3
DG

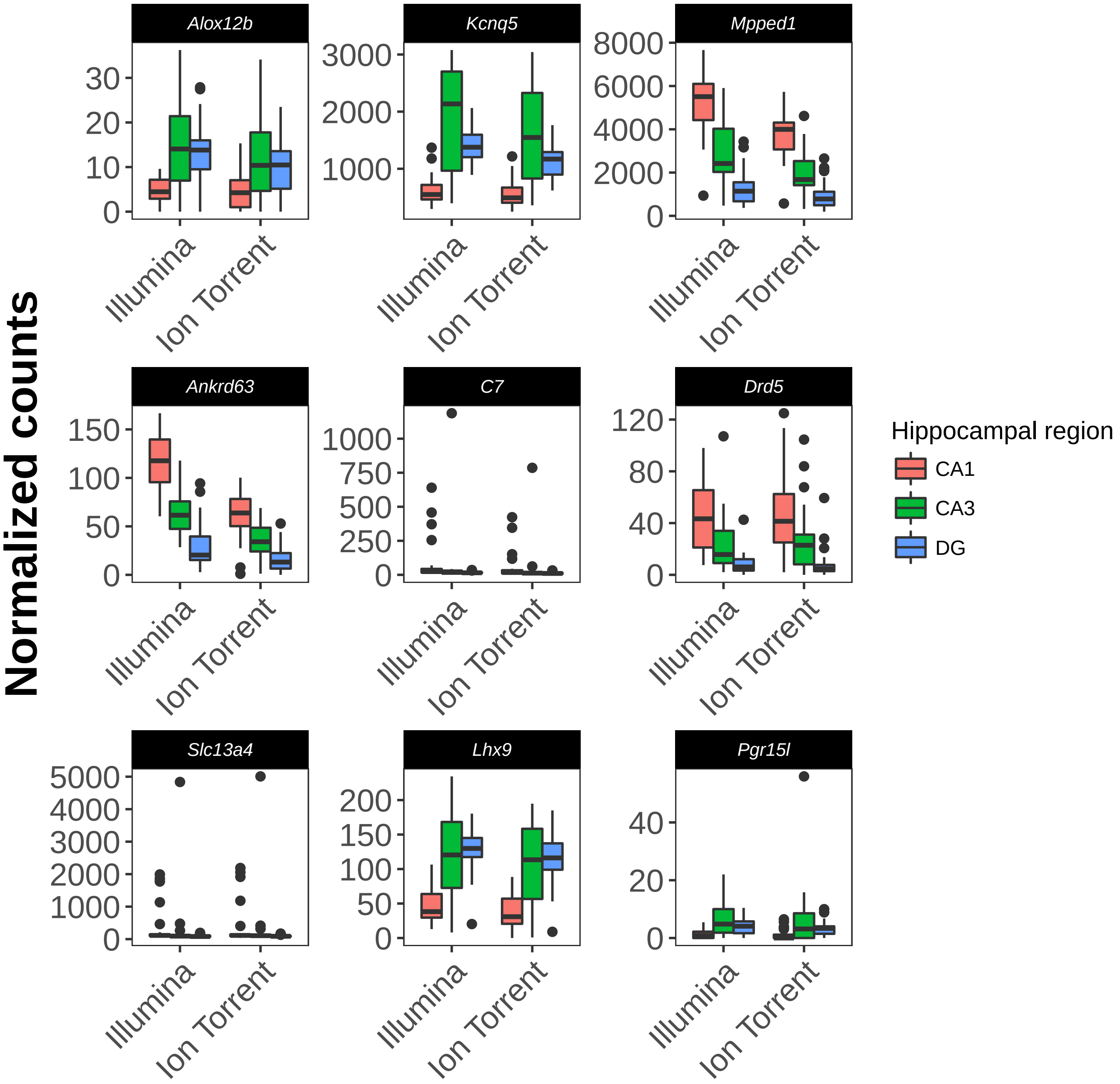
Normalized counts
Alox12b
Kcnq5
Mpped1
Ankrd63
C7
Drd5
Slc13a4
Lhx9
Pgr15l
Illumina
Ion Torrent
Hippocampal region
CA1
CA3
DG

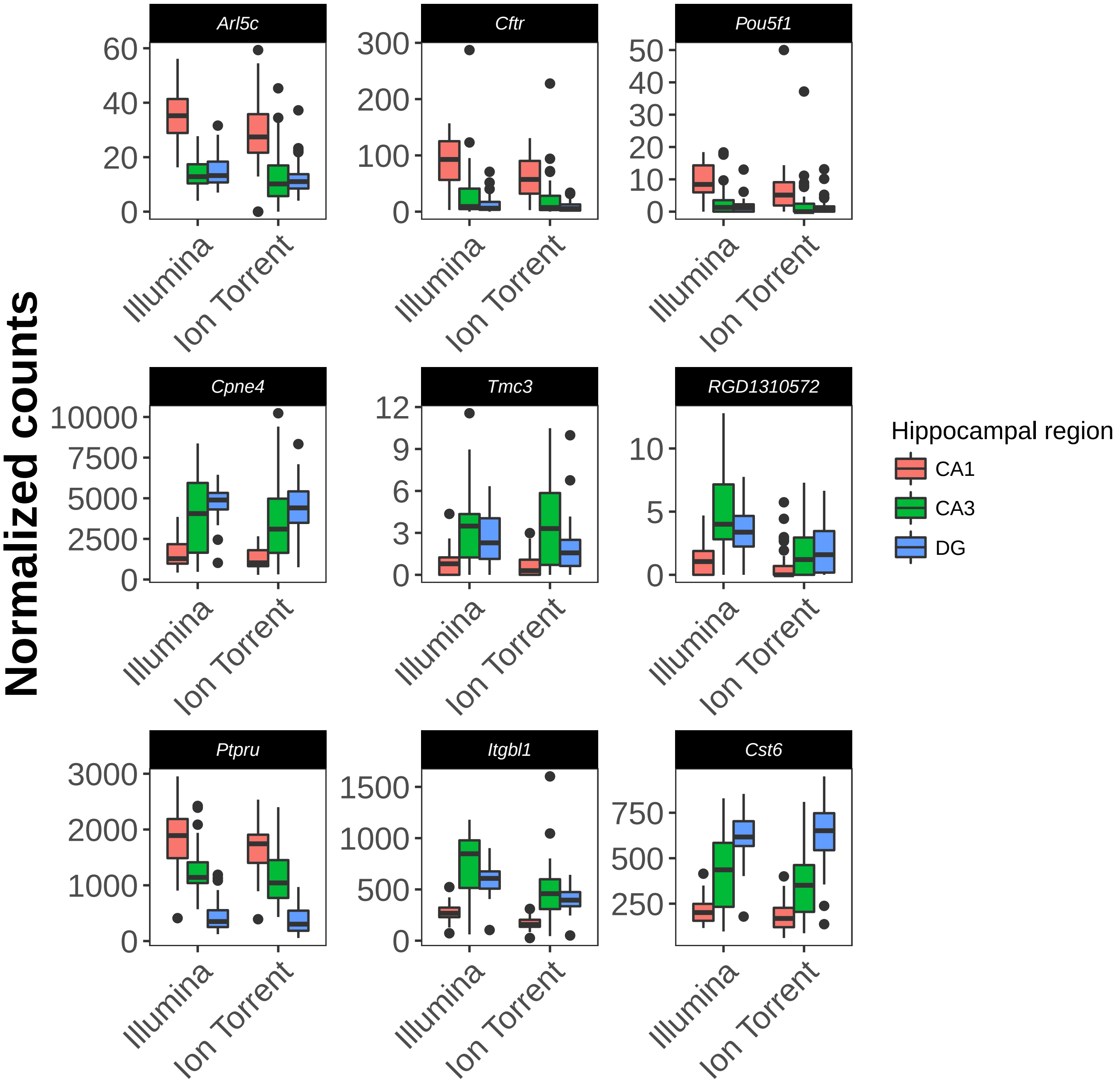
Arl5c
Cftr
Pou5f1
Cpne4
Tmc3
RGD1310572
Ptpru
Itgbl1
Cst6
Normalized counts
Illumina
Ion Torrent
Hippocampal region
CA1
CA3
DG

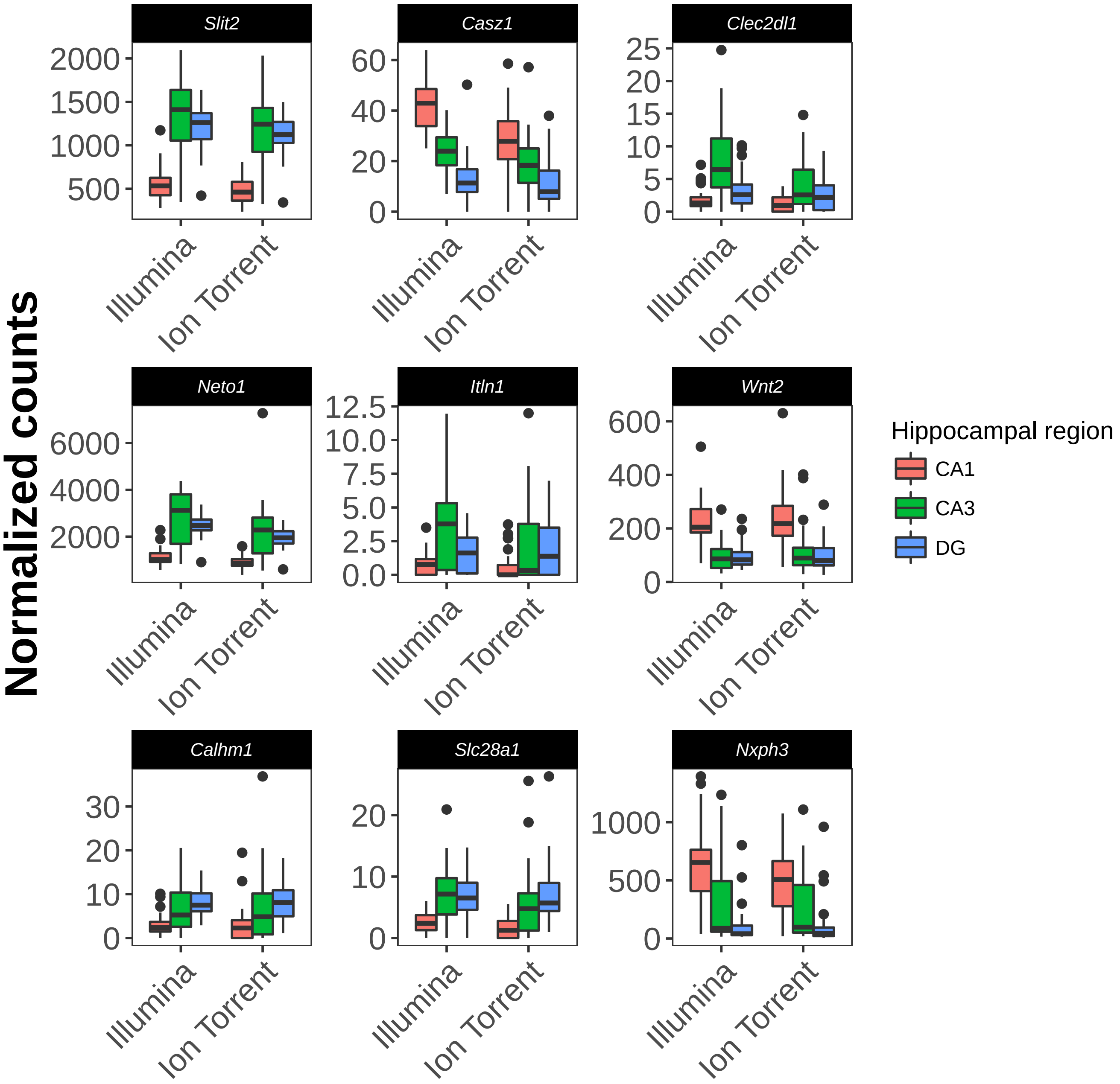

Normalized counts
Slit2
Casz1
Clec2dl1
Neto1
Itln1
Wnt2
Calhm1
Slc28a1
Nxph3
Illumina
Ion Torrent
Hippocampal region
CA1
CA3
DG

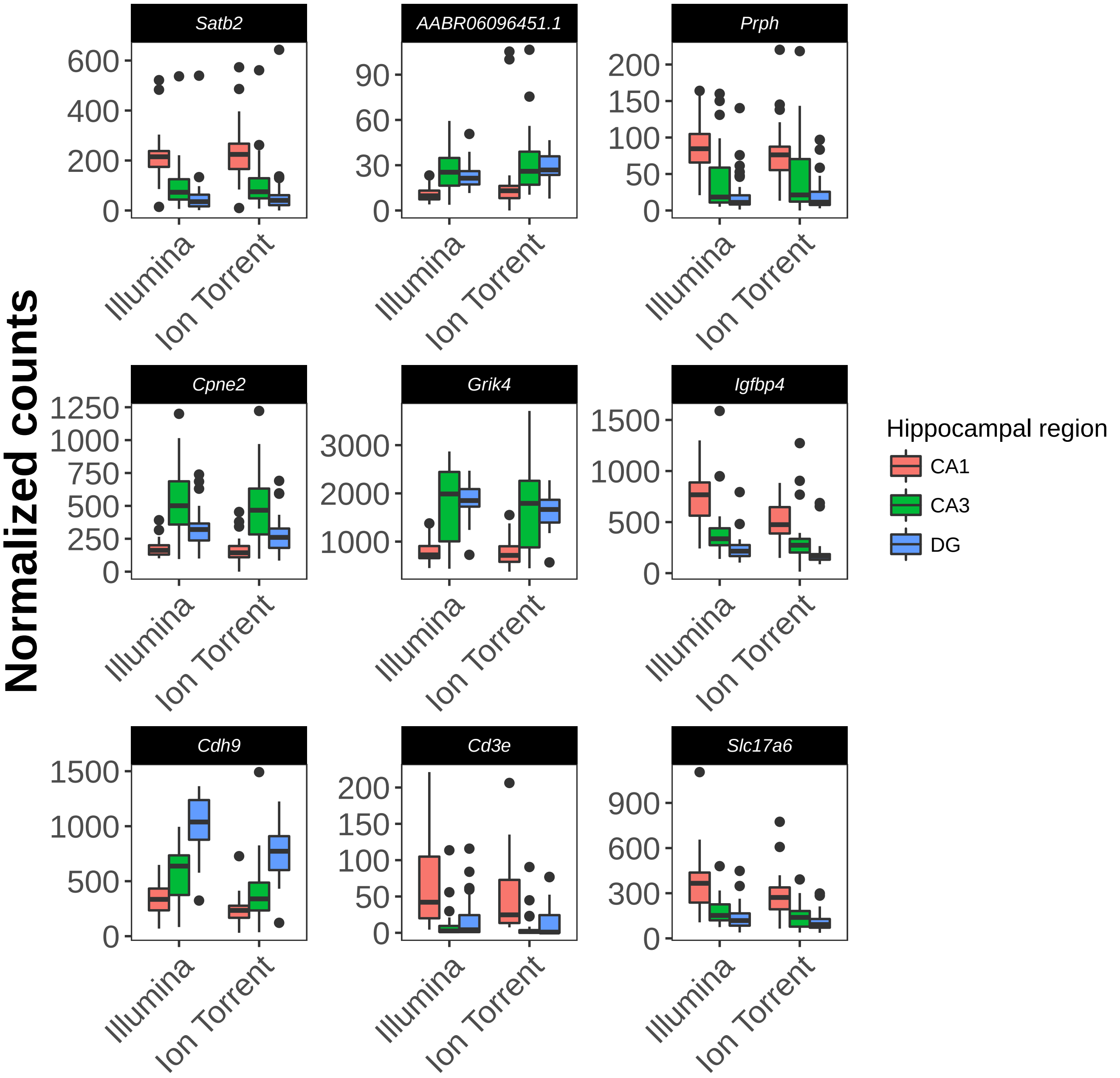
Normalized counts
Satb2
AABR06096451.1
Prph
Cpne2
Grik4
Igfbp4
Cdh9
Cd3e
Slc17a6
Illumina
Ion Torrent
Hippocampal region
CA1
CA3
DG

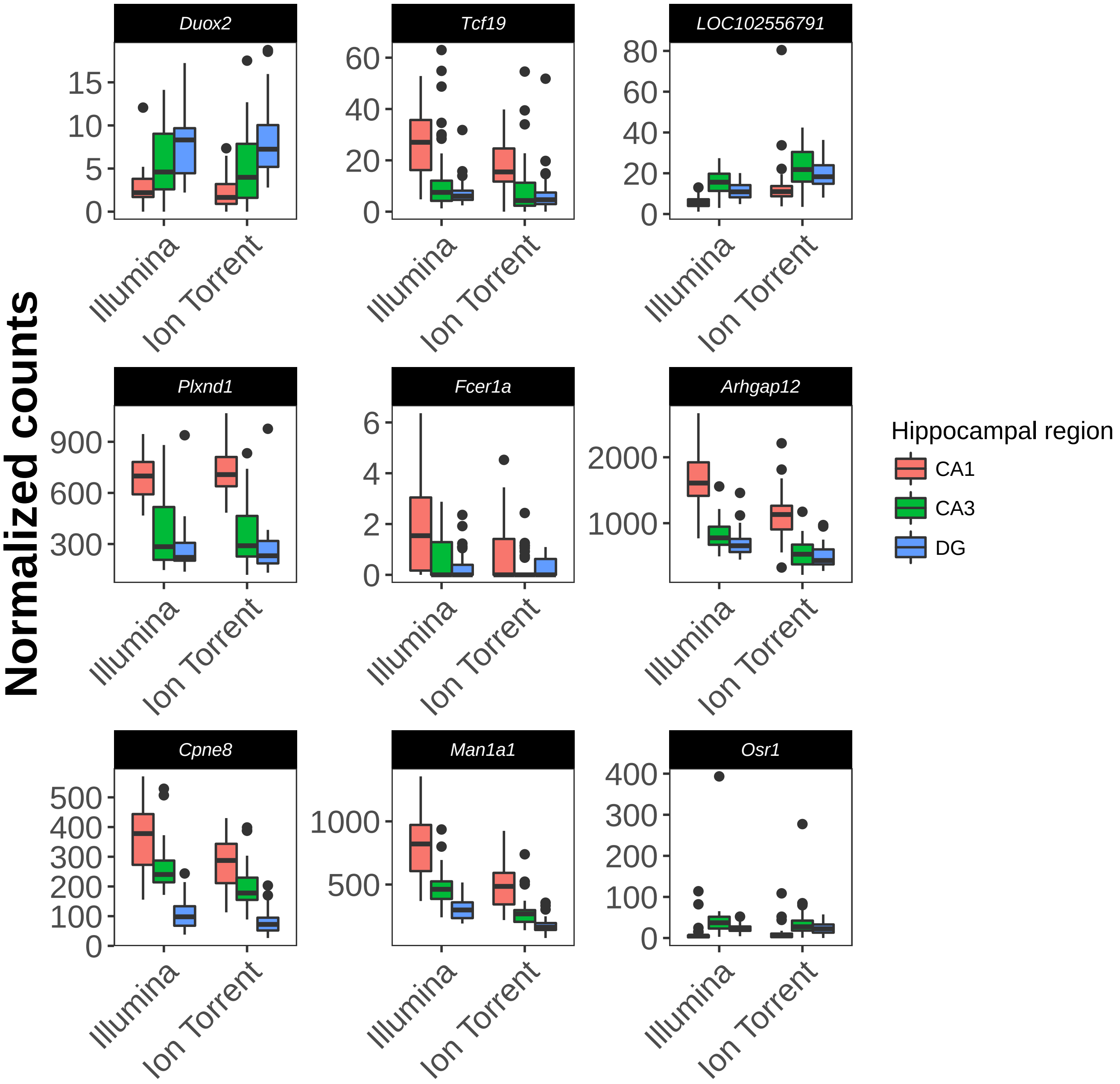

Normalized counts
Duox2
Tcf19
LOC102556791
Plxnd1
Fcer1a
Arhgap12
Cpne8
Man1a1
Osr1
Illumina
Ion Torrent
Hippocampal region
CA1
CA3
DG

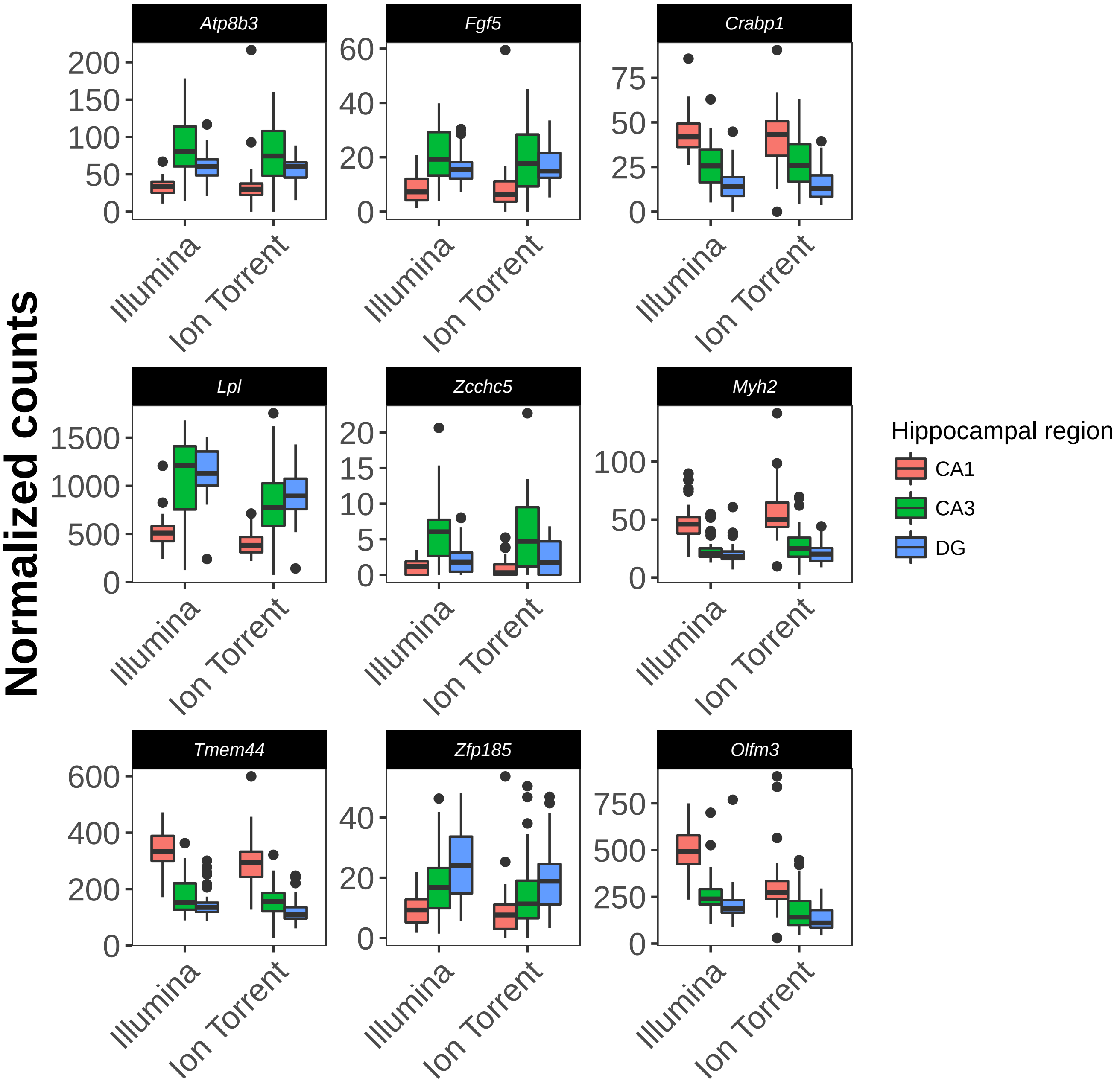
Normalized counts
Atp8b3
Fgf5
Crabp1
Lpl
Zcchc5
Myh2
Tmem44
Zfp185
Olfm3
Illumina
Ion Torrent
Hippocampal region
CA1
CA3
DG

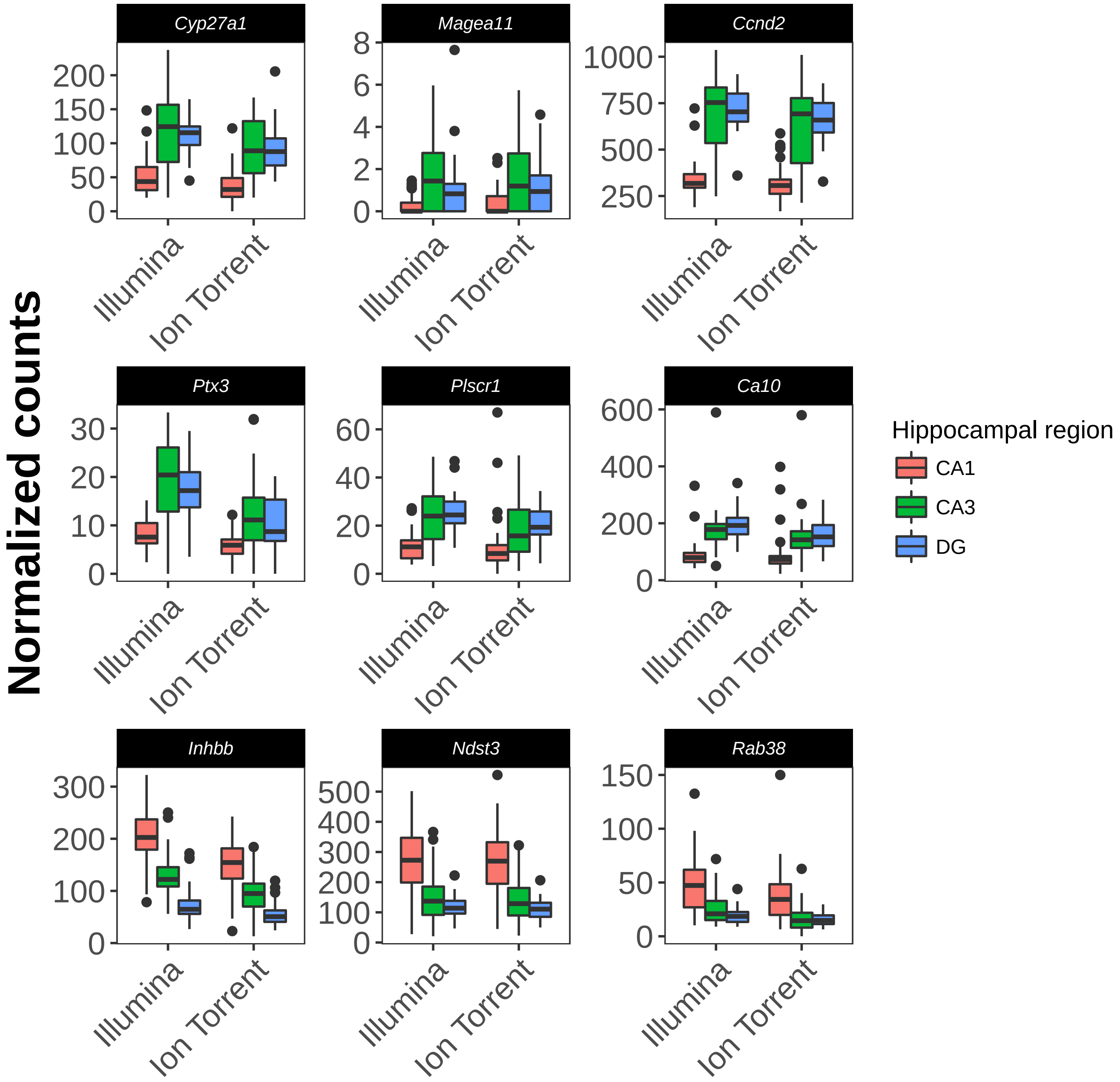
Normalized counts
Cyp27a1
Magea11
Ccnd2
Ptx3
Plscr1
Ca10
Inhbb
Ndst3
Rab38
Illumina
Ion Torrent
Hippocampal region
CA1
CA3
DG

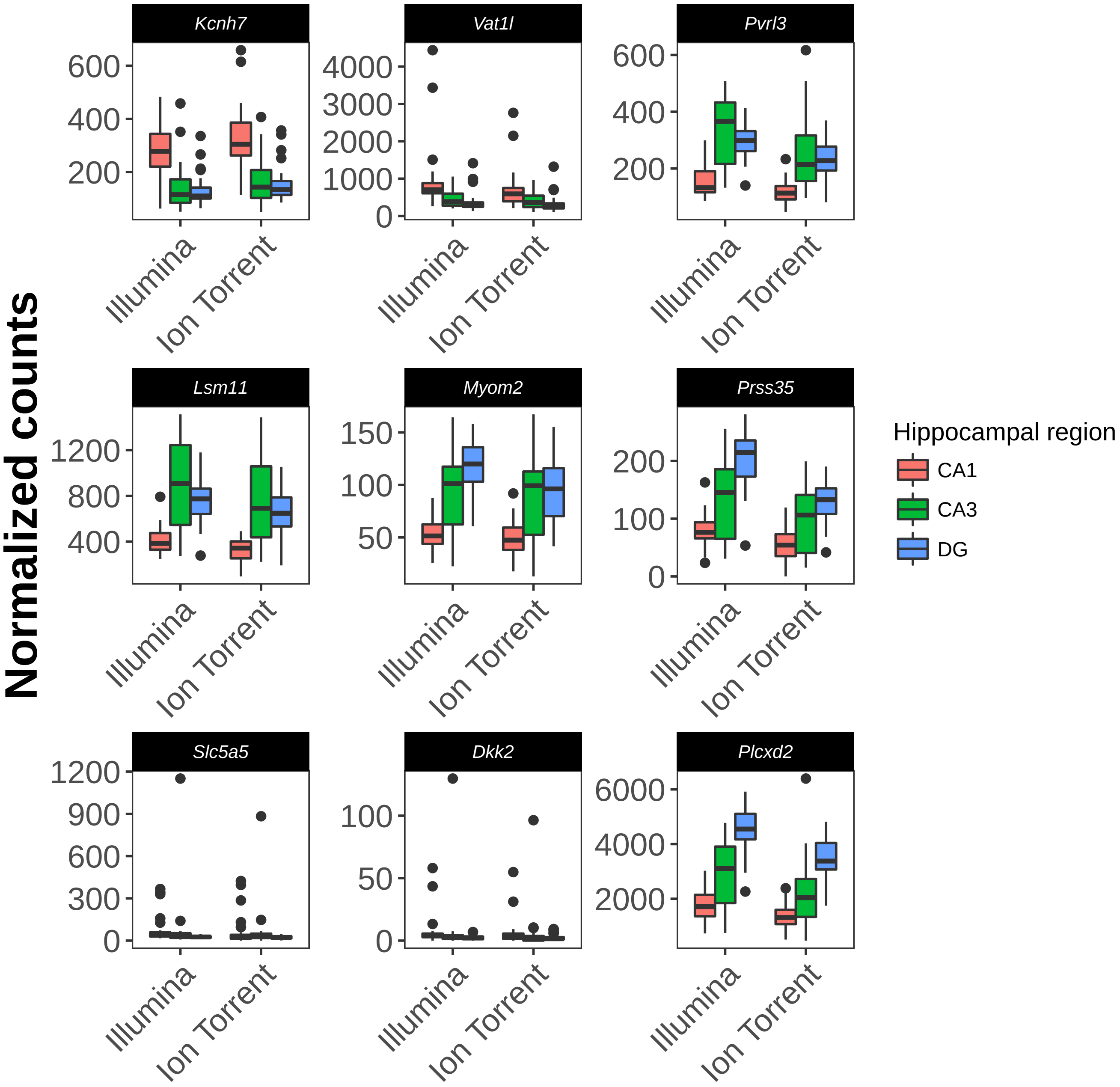
Normalized counts
Kcnh7
Vat1l
Pvrl3
Lsm11
Myom2
Prss35
Slc5a5
Dkk2
Plcxd2
Illumina
Ion Torrent
Hippocampal region
CA1
CA3
DG

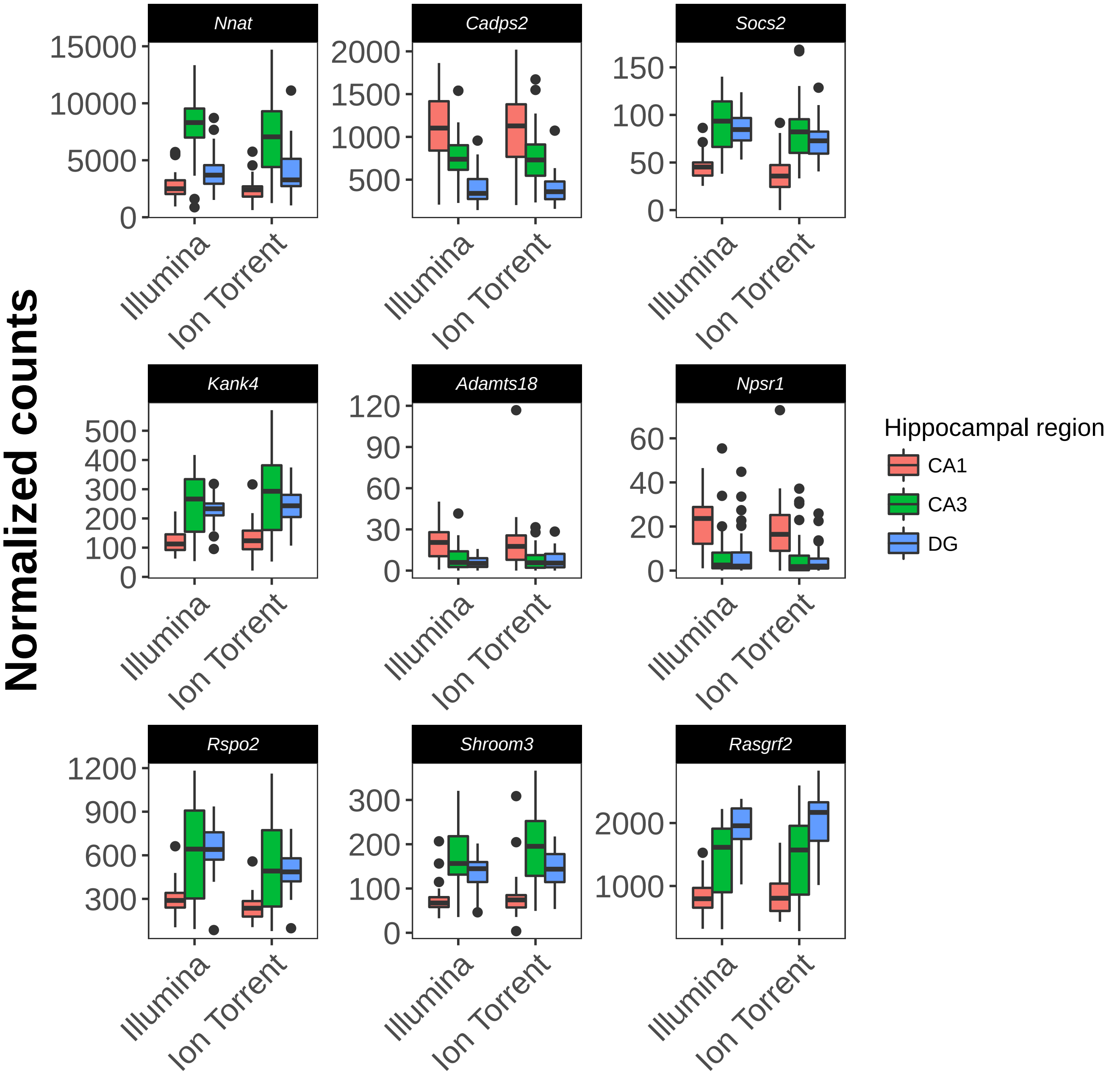

Normalized counts
Nnat
Cadps2
Socs2
Kank4
Adamts18
Npsr1
Rspo2
Shroom3
Rasgrf2
Illumina
Ion Torrent
Hippocampal region
CA1
CA3
DG

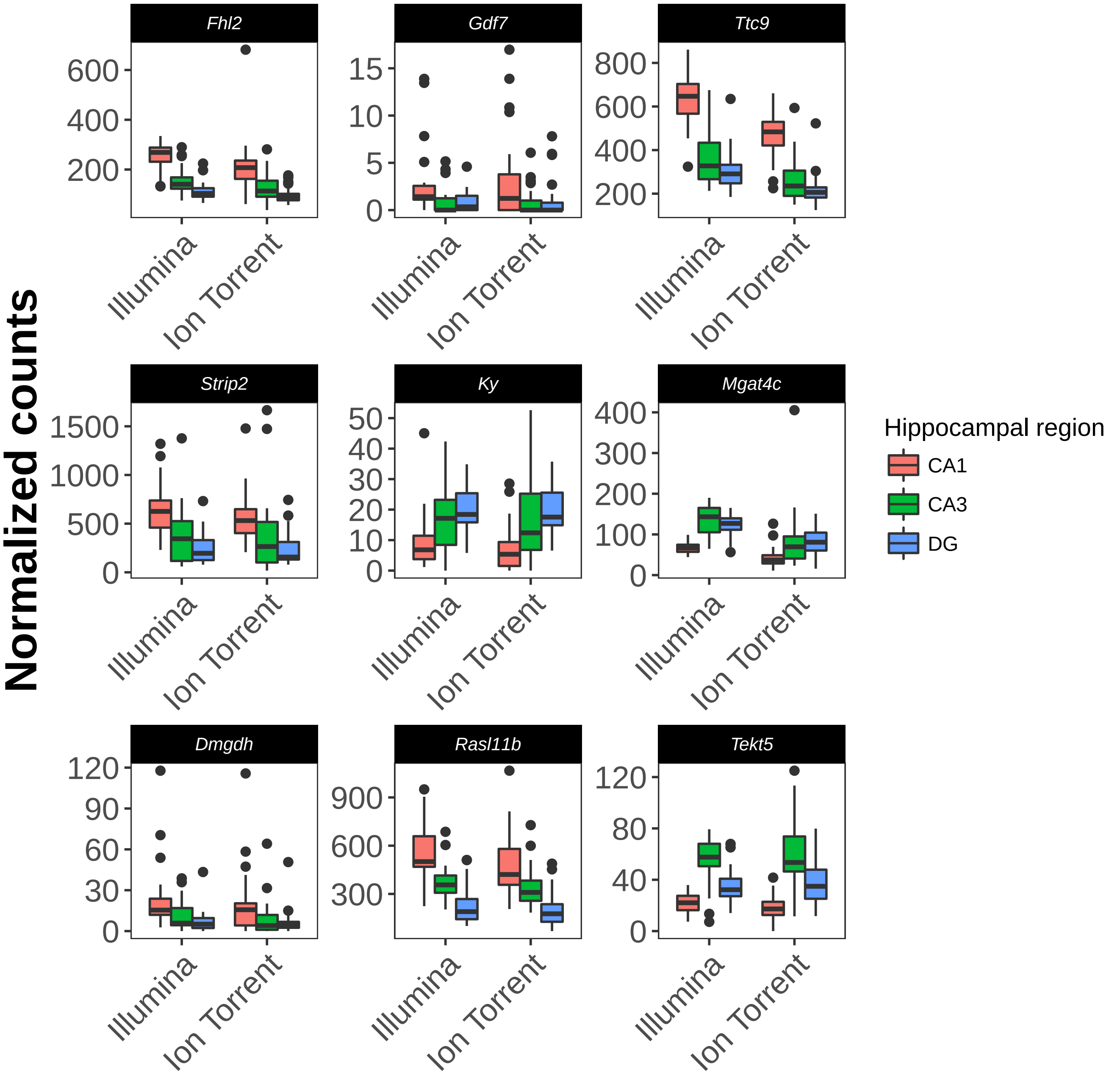

Normalized counts
Fhl2
Gdf7
Ttc9
Strip2
Ky
Mgat4c
Dmgdh
Rasl11b
Tekt5
Illumina
Ion Torrent
Hippocampal region
CA1
CA3
DG

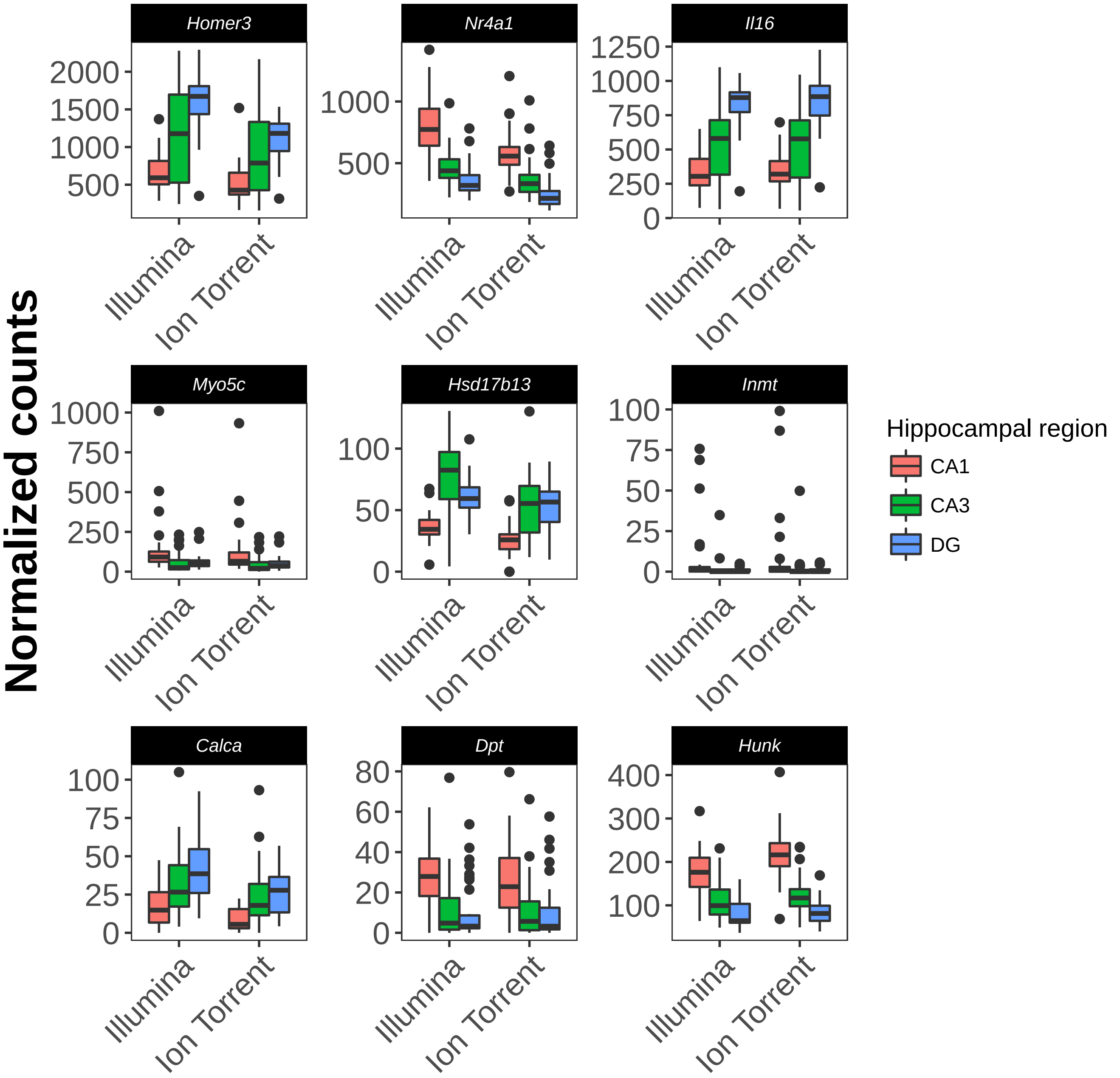
Normalized counts
Homer3
Nr4a1
Il16
Myo5c
Hsd17b13
Inmt
Calca
Dpt
Hunk
Illumina
Ion Torrent
Hippocampal region
CA1
CA3
DG

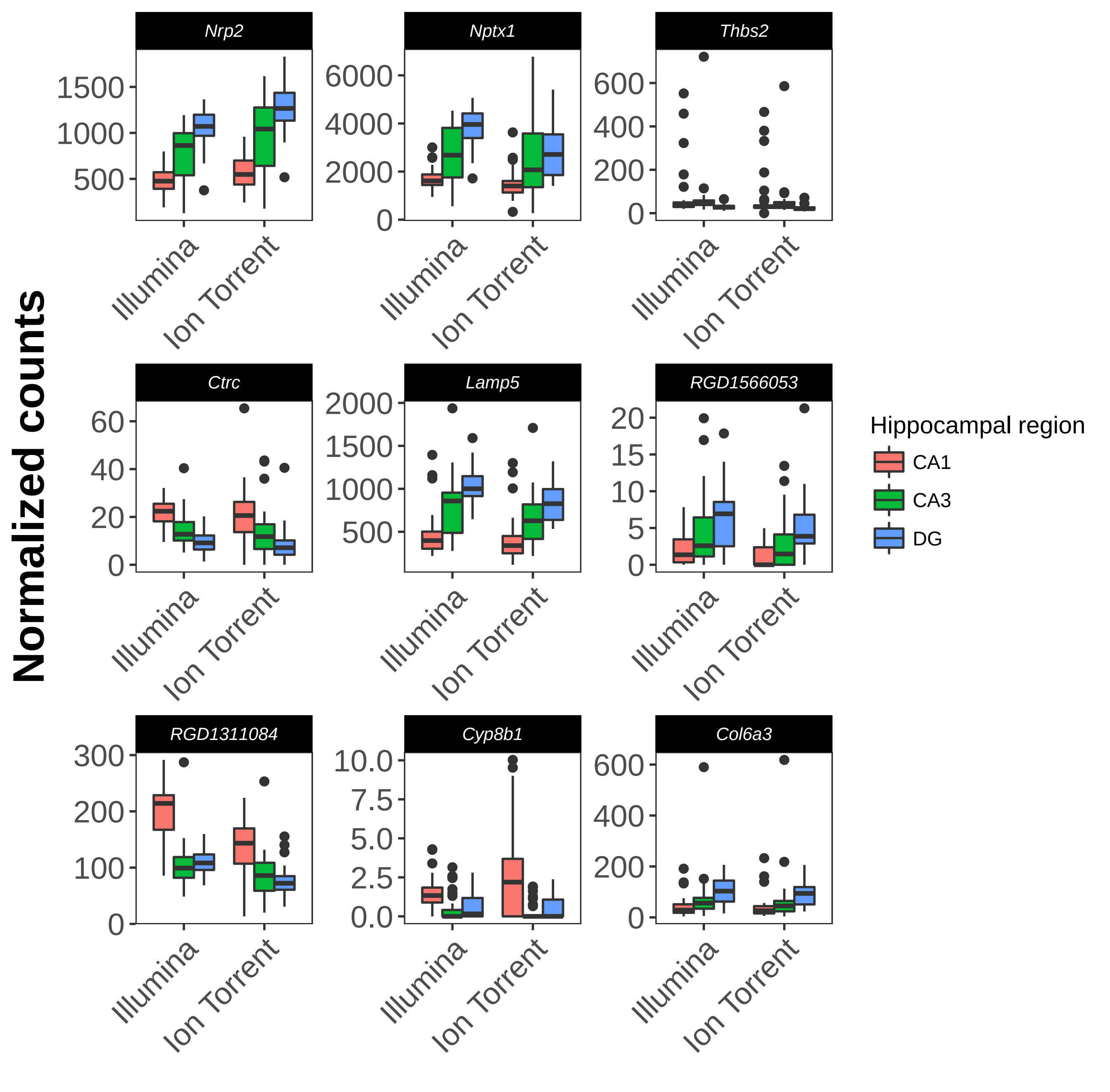
Normalized counts
Nrp2
Nptx1
Thbs2
Ctrc
Lamp5
RGD1566053
RGD1311084
Cyp8b1
Col6a3
Illumina
Ion Torrent
Hippocampal region
CA1
CA3
DG

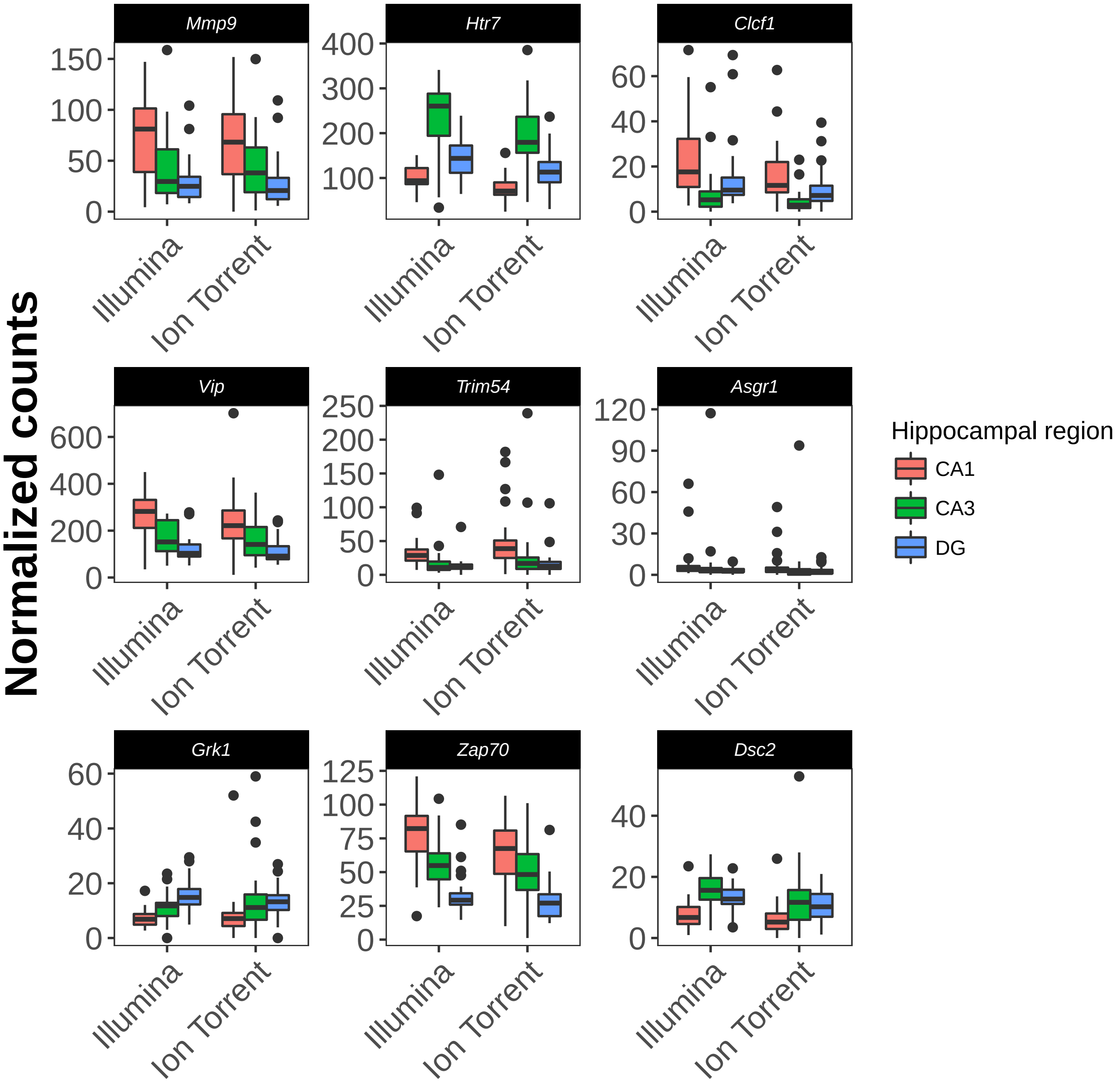

Normalized counts
Mmp9
Htr7
Clcf1
Vip
Trim54
Asgr1
Grk1
Zap70
Dsc2
Illumina
Ion Torrent
Hippocampal region
CA1
CA3
DG

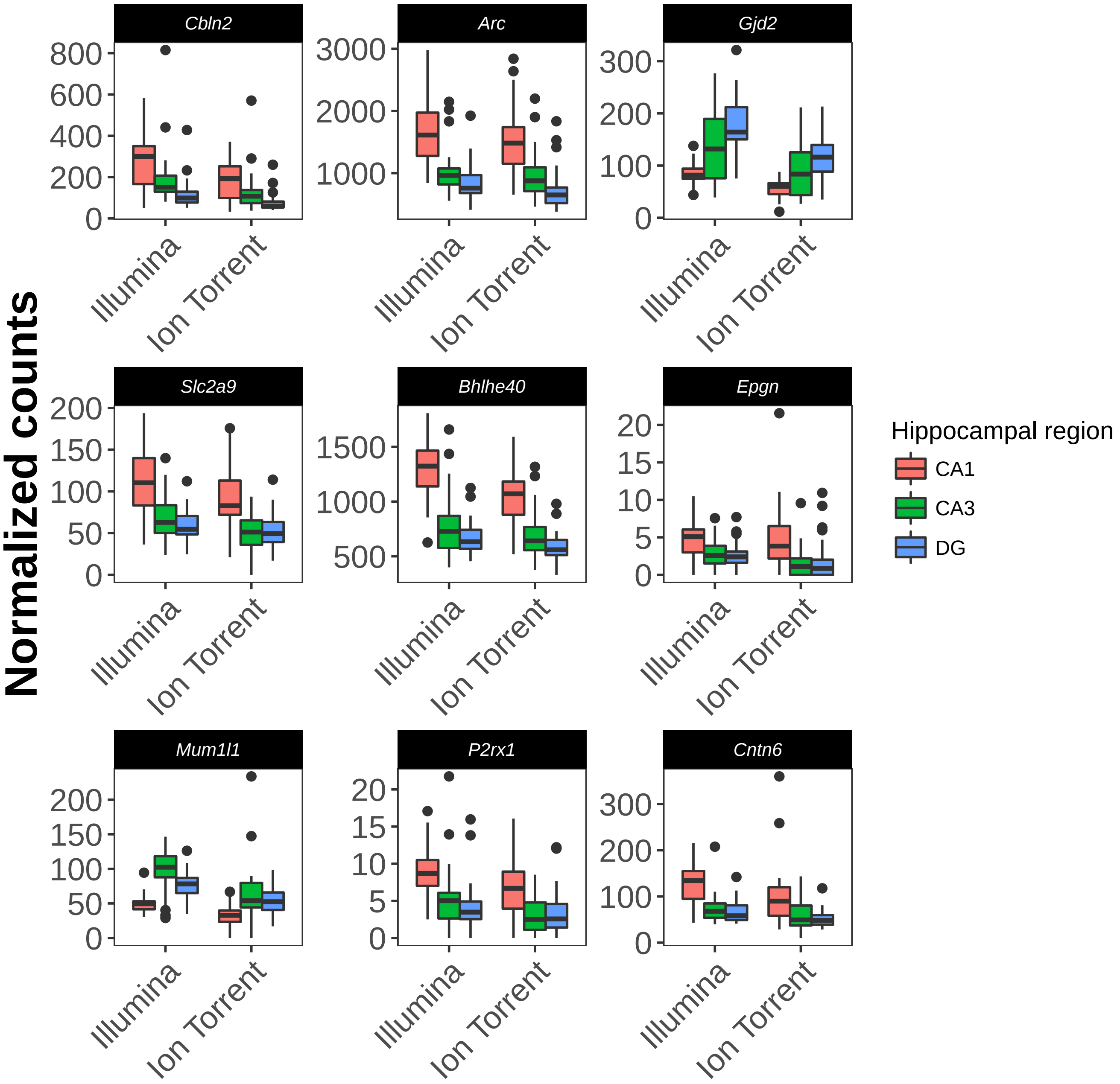
Normalized counts
Cbln2
Arc
Gjd2
Slc2a9
Bhlhe40
Epgn
Mum1l1
P2rx1
Cntn6
Illumina
Ion Torrent
Hippocampal region
CA1
CA3
DG

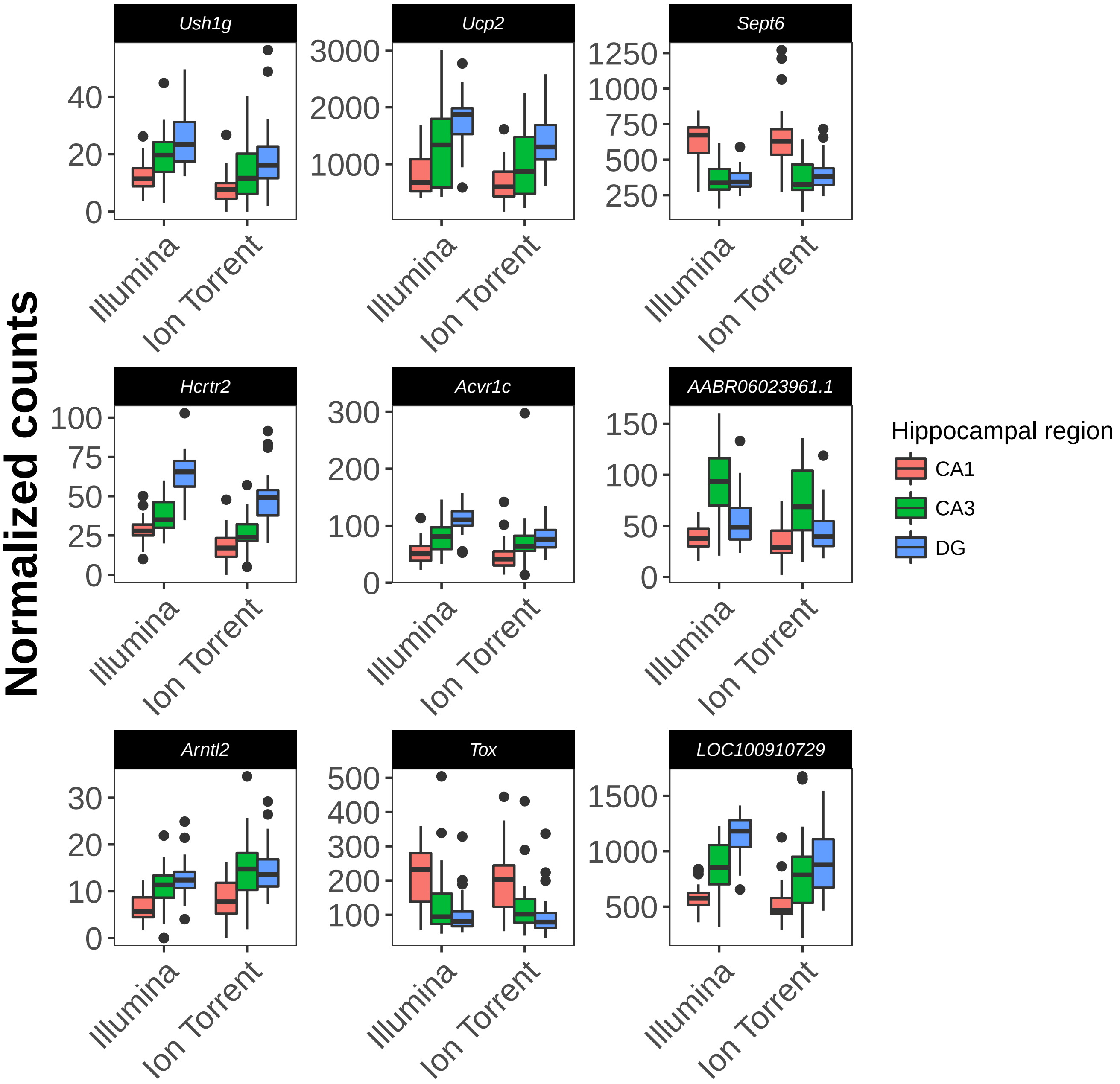

Normalized counts
Ush1g
Ucp2
Sept6
Hcrtr2
Acvr1c
AABR06023961.1
Arntl2
Tox
LOC100910729
Illumina
Ion Torrent
Hippocampal region
CA1
CA3
DG

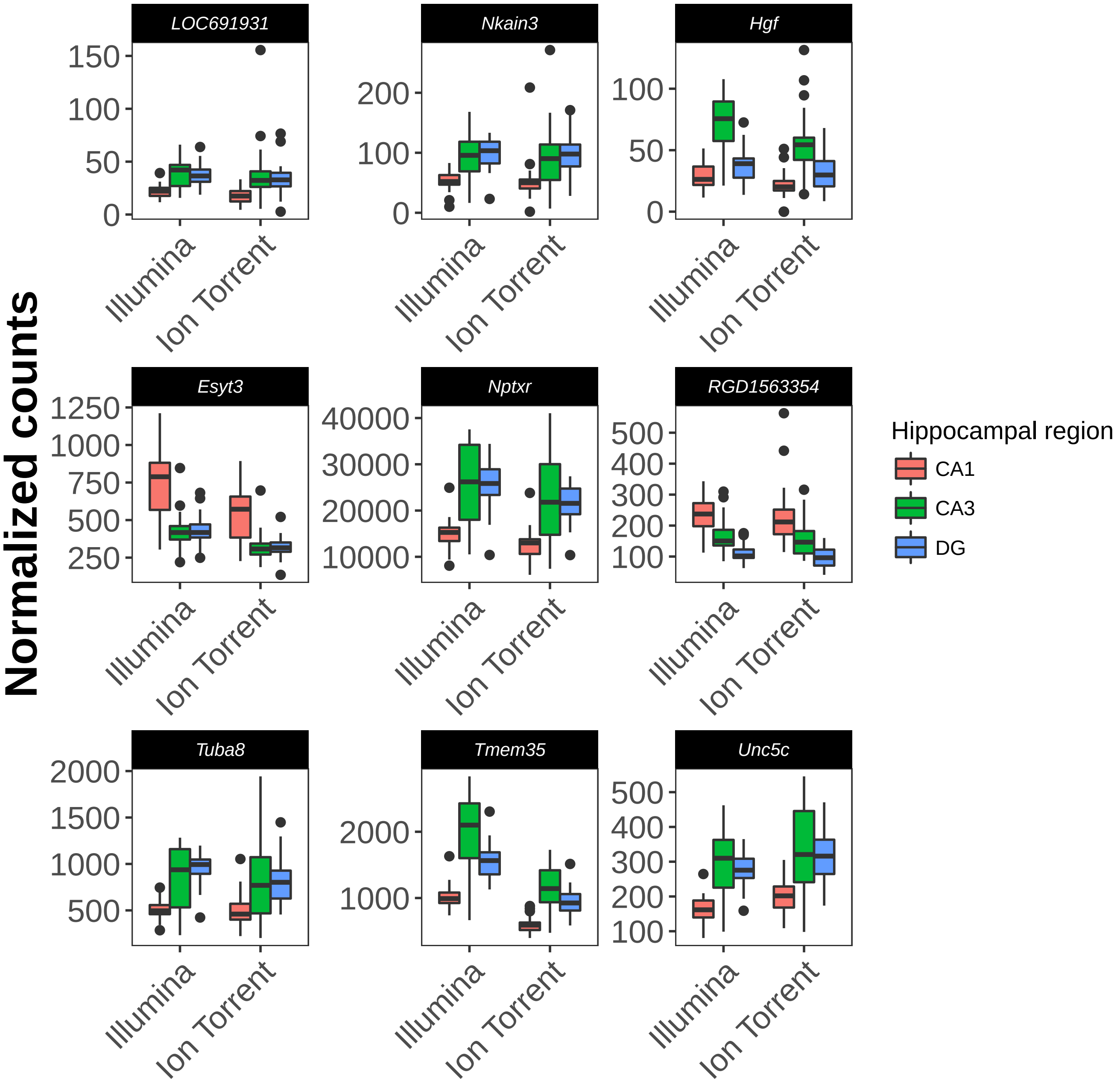
LOC691931
Nkain3
Hgf
Esyt3
Nptxr
RGD1563354
Tuba8
Tmem35
Unc5c
Normalized counts
Illumina
Ion Torrent
Hippocampal region
CA1
CA3
DG

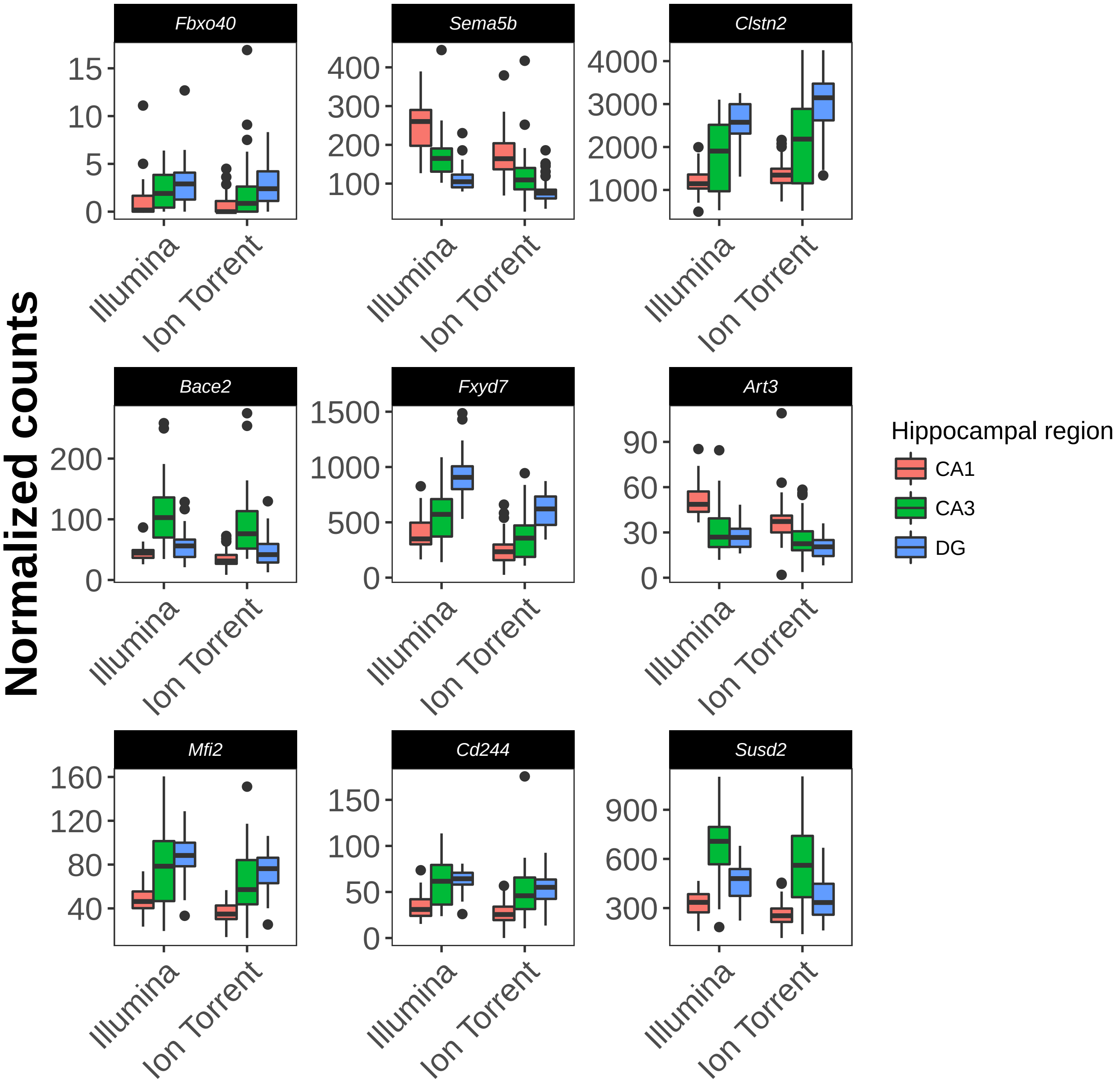

Fbxo40
Sema5b
Clstn2
Bace2
Fxyd7
Art3
Mfi2
Cd244
Susd2
Normalized counts
Illumina
Ion Torrent
Hippocampal region
CA1
CA3
DG

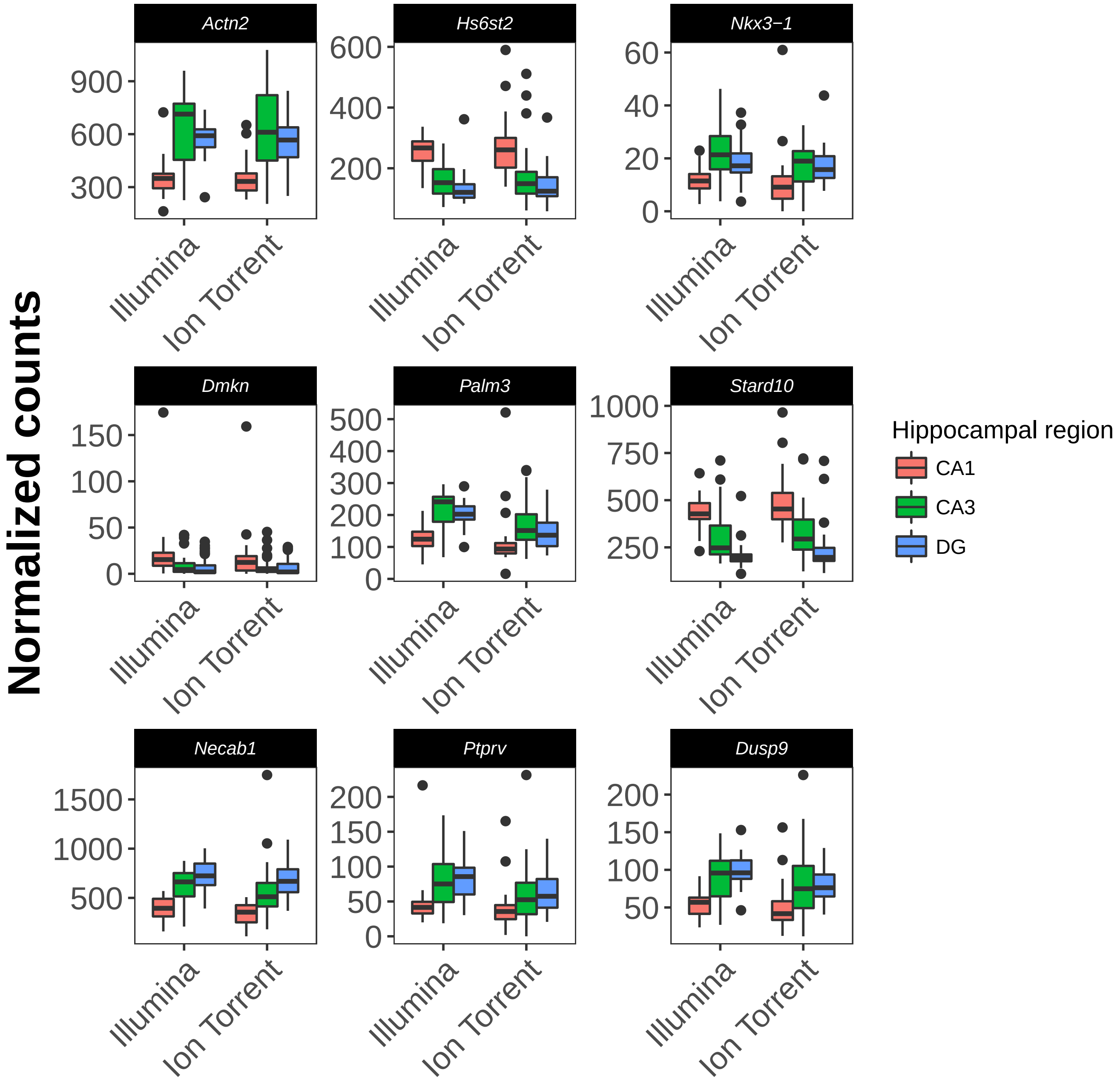
Normalized counts
Actn2
Hs6st2
Nkx3−1
Dmkn
Palm3
Stard10
Necab1
Ptprv
Dusp9
Illumina
Ion Torrent
Hippocampal region
CA1
CA3
DG

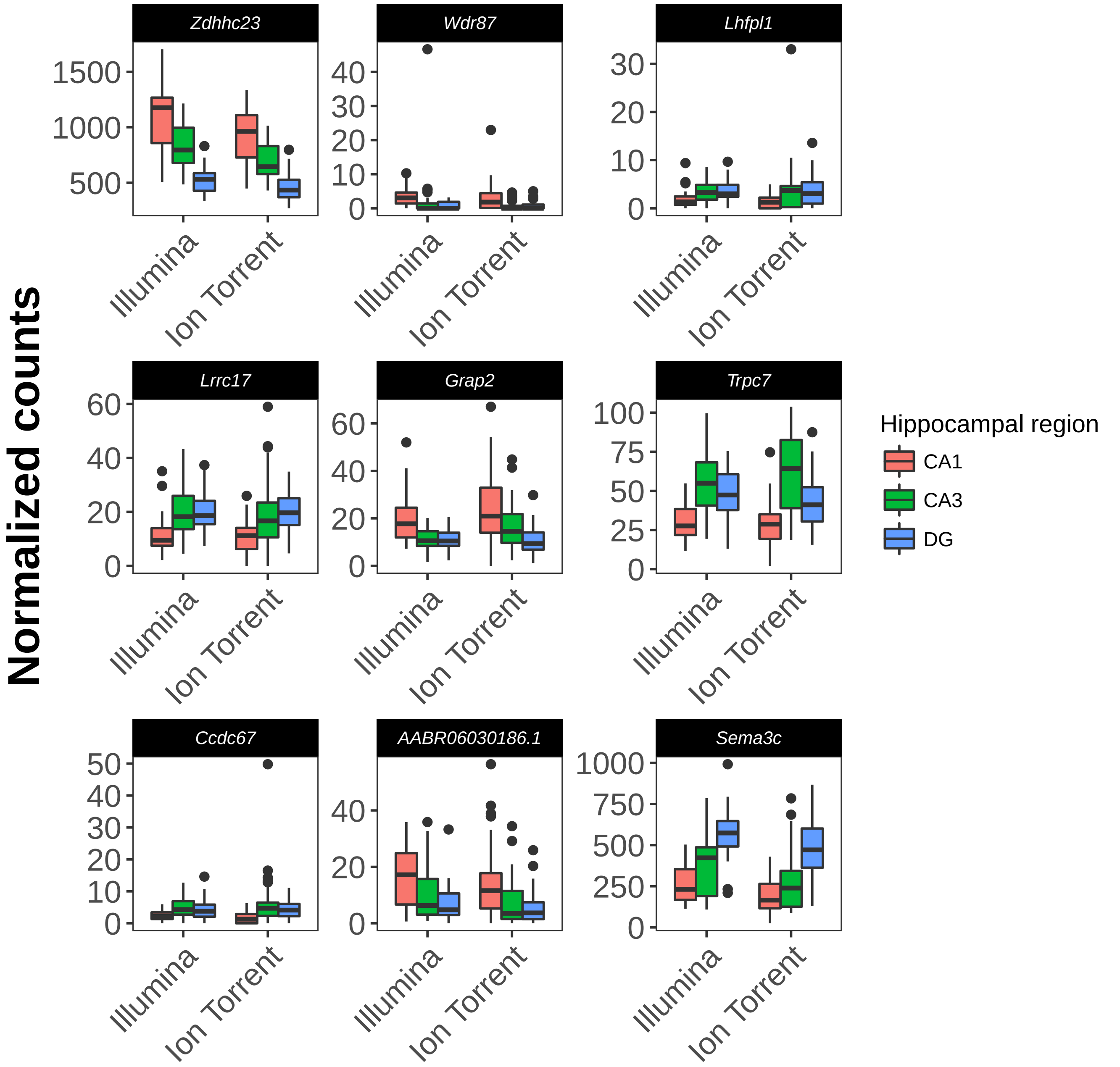
Normalized counts
Zdhhc23
Wdr87
Lhfpl1
Lrrc17
Grap2
Trpc7
Ccdc67
AABR06030186.1
Sema3c
Illumina
Ion Torrent
Hippocampal region
CA1
CA3
DG

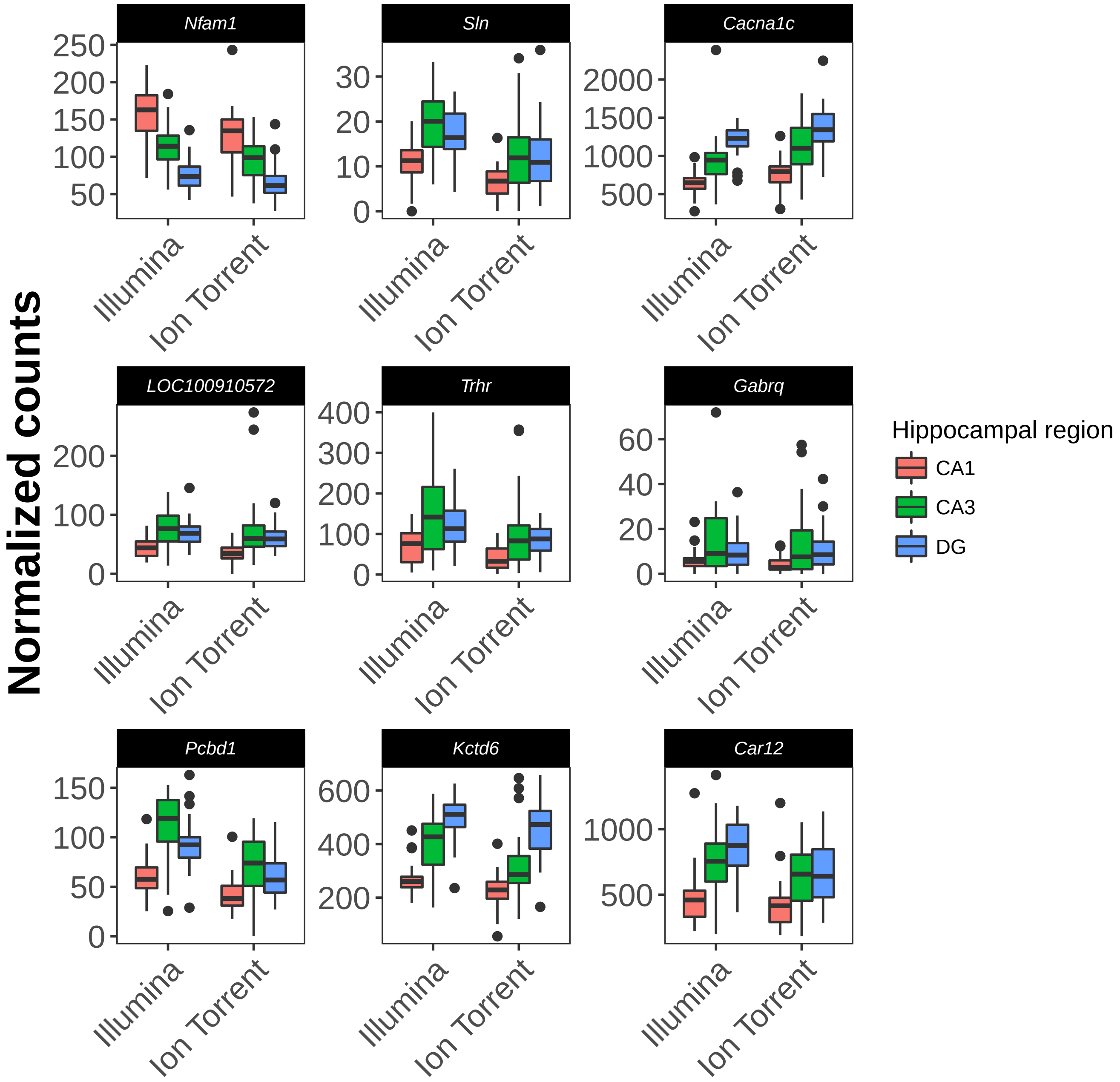

Normalized counts
Nfam1
Sln
Cacna1c
LOC100910572
Trhr
Gabrq
Pcbd1
Kctd6
Car12
Illumina
Ion Torrent
Hippocampal region
CA1
CA3
DG

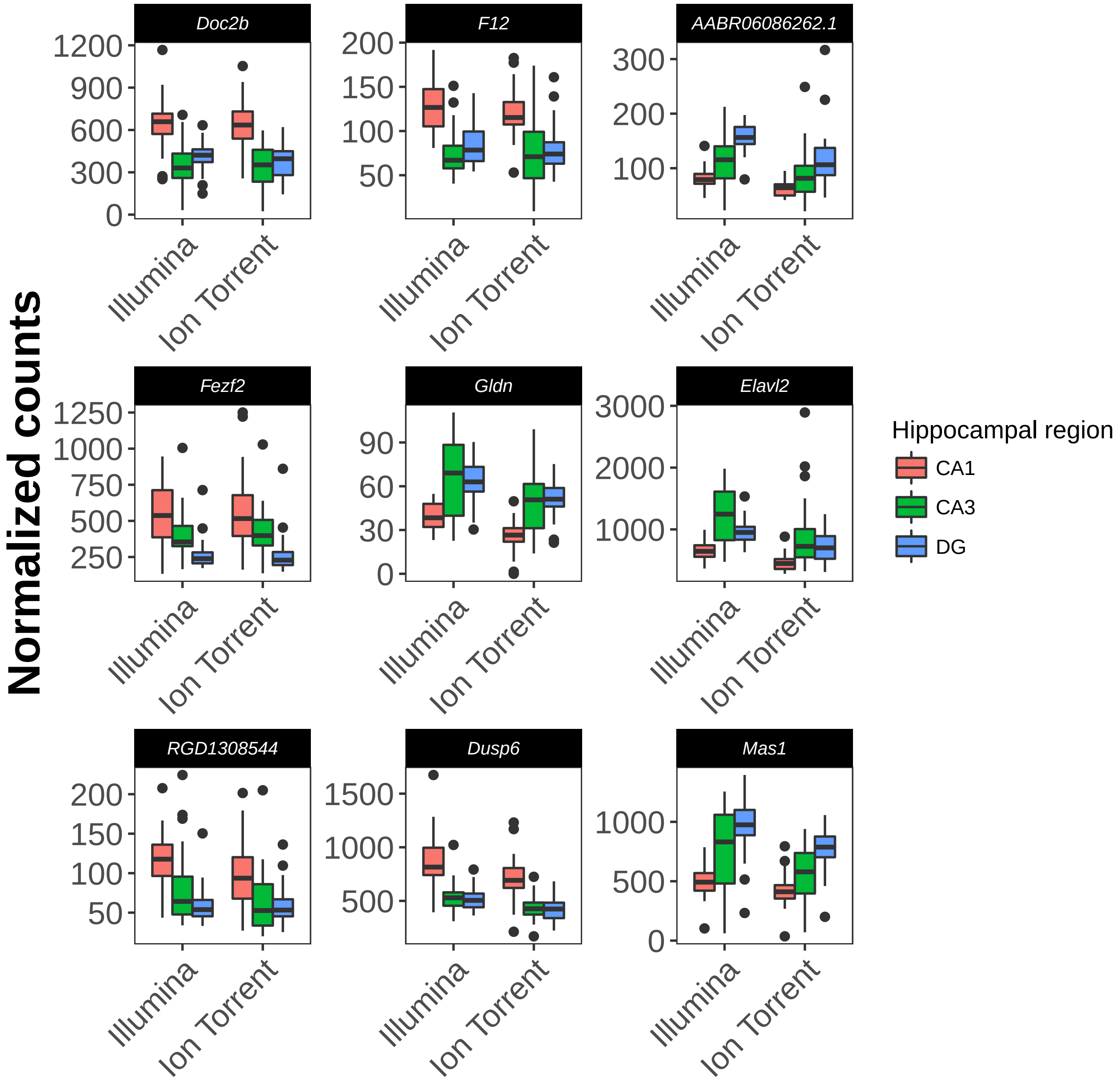

Normalized counts
Doc2b
F12
AABR06086262.1
Fezf2
Gldn
Elavl2
RGD1308544
Dusp6
Mas1
Illumina
Ion Torrent
Hippocampal region
CA1
CA3
DG

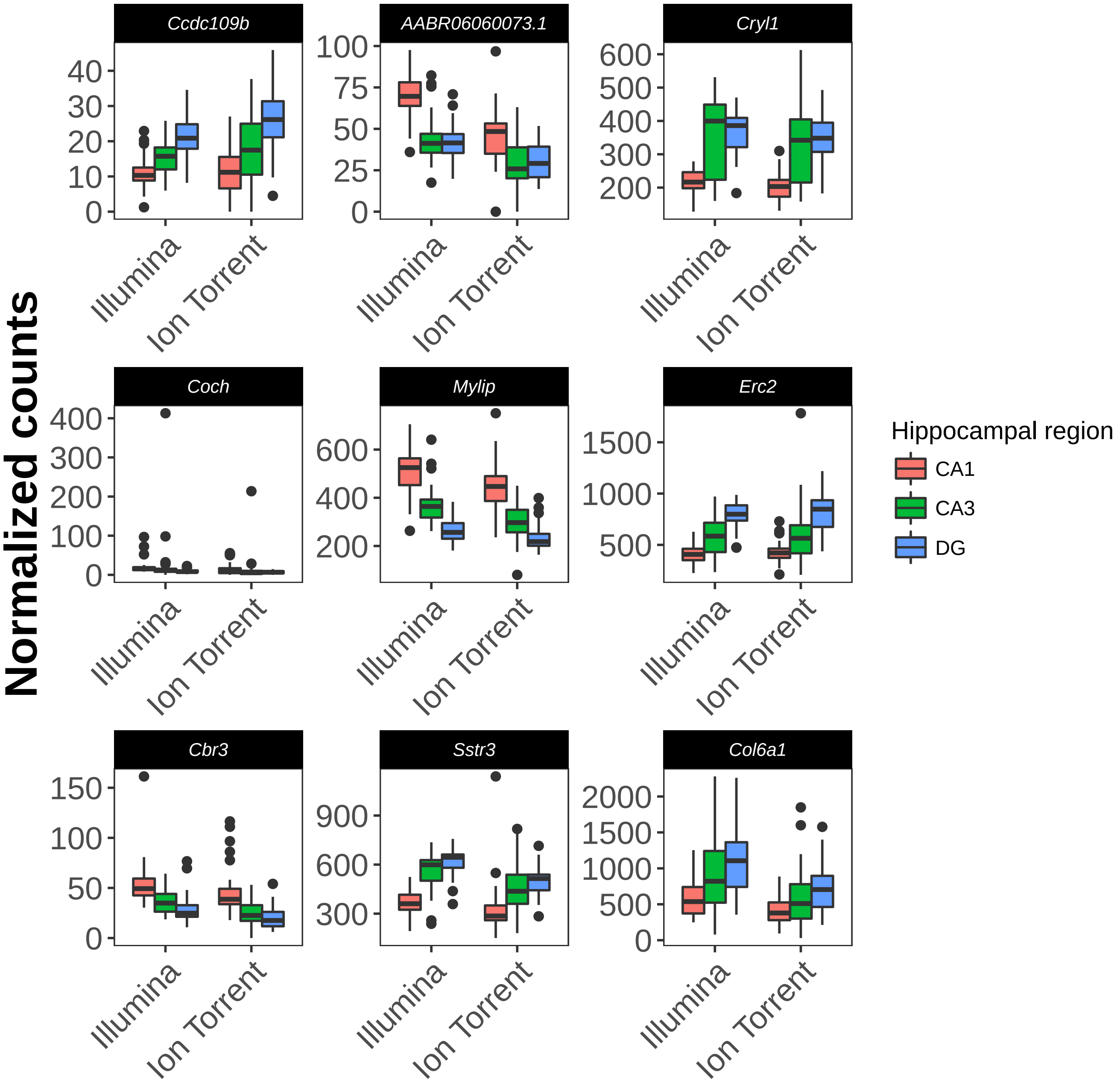

Normalized counts
Ccdc109b
AABR06060073.1
Cryl1
Coch
Mylip
Erc2
Cbr3
Sstr3
Col6a1
Illumina
Ion Torrent
Hippocampal region
CA1
CA3
DG

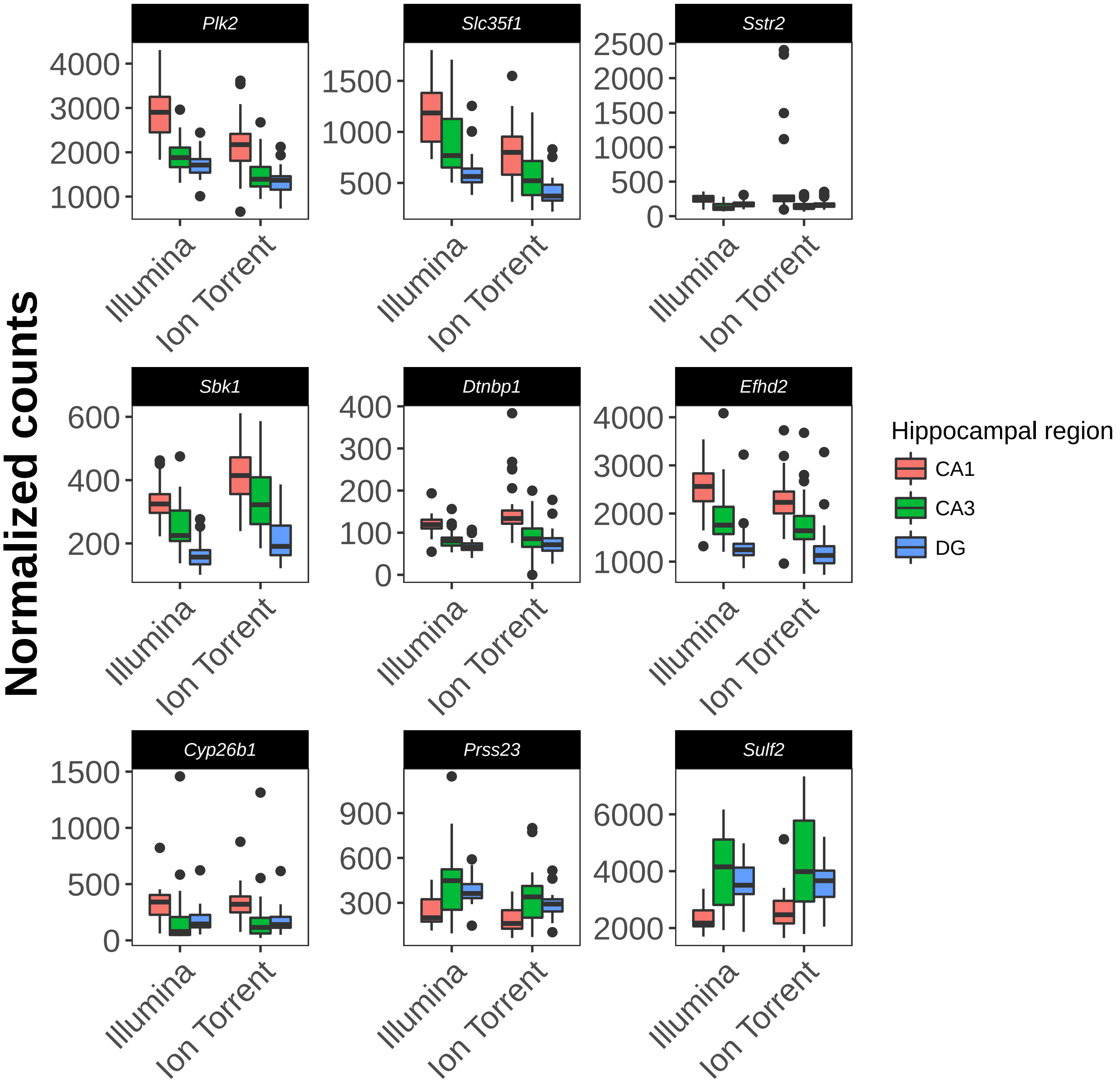

Normalized counts
Plk2
Slc35f1
Sstr2
Sbk1
Dtnbp1
Efhd2
Cyp26b1
Prss23
Sulf2
Illumina
Ion Torrent
Hippocampal region
CA1
CA3
DG

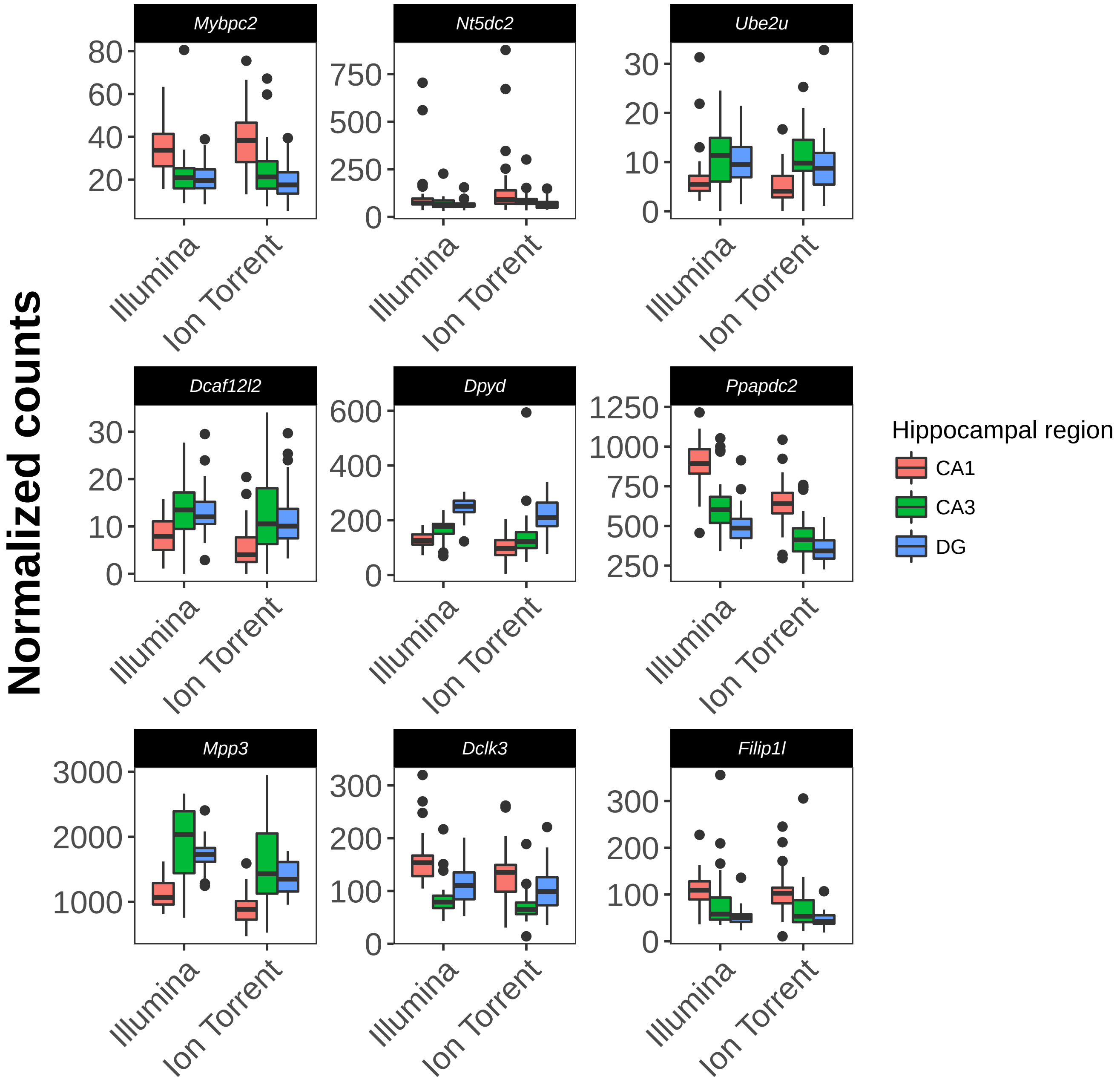
Normalized counts
Mybpc2
Nt5dc2
Ube2u
Dcaf12l2
Dpyd
Ppapdc2
Mpp3
Dclk3
Filip1l
Illumina
Ion Torrent
Hippocampal region
CA1
CA3
DG

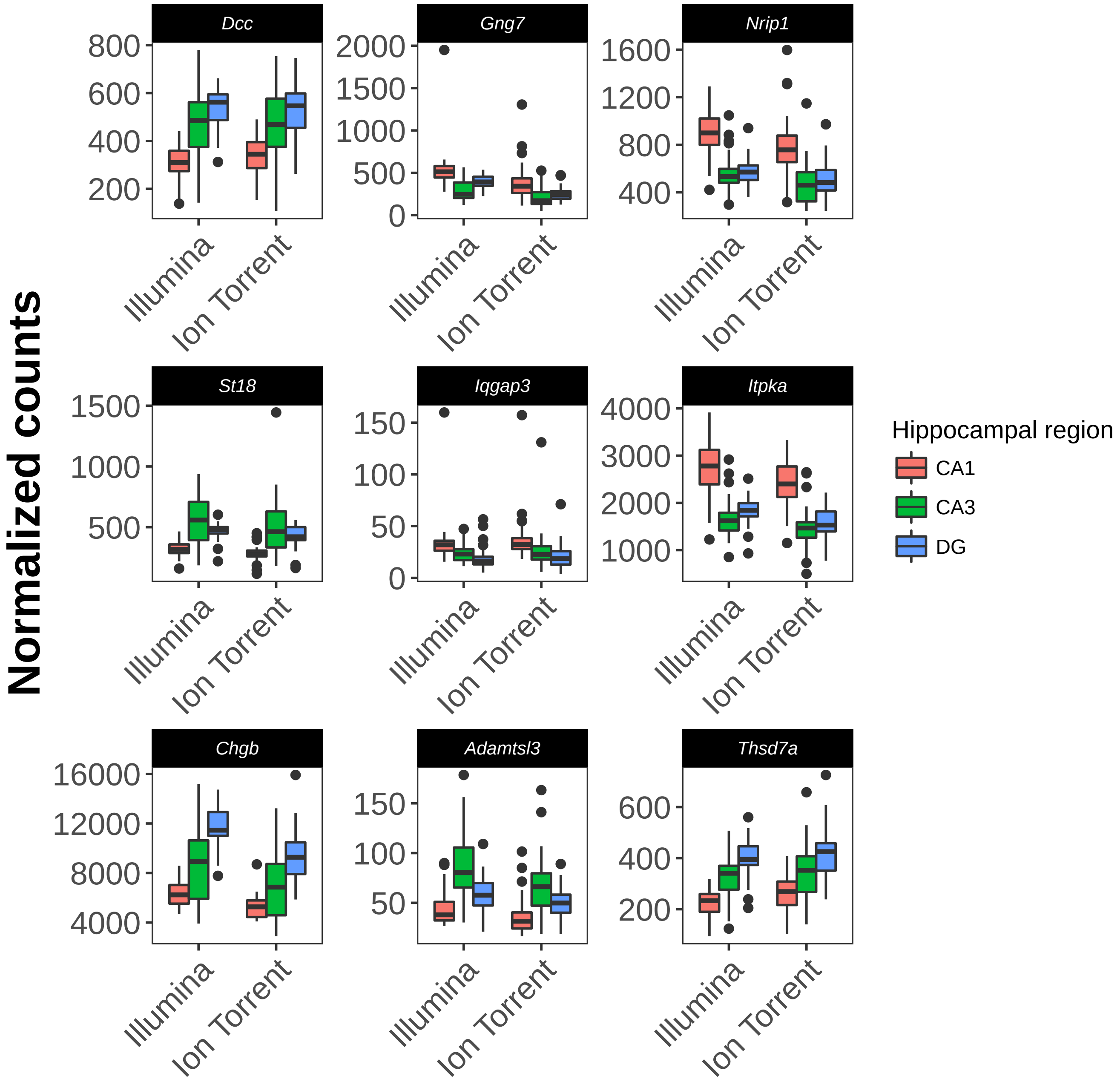
Normalized counts
Dcc
Gng7
Nrip1
St18
Iqgap3
Itpka
Chgb
Adamtsl3
Thsd7a
Illumina
Ion Torrent
Hippocampal region
CA1
CA3
DG

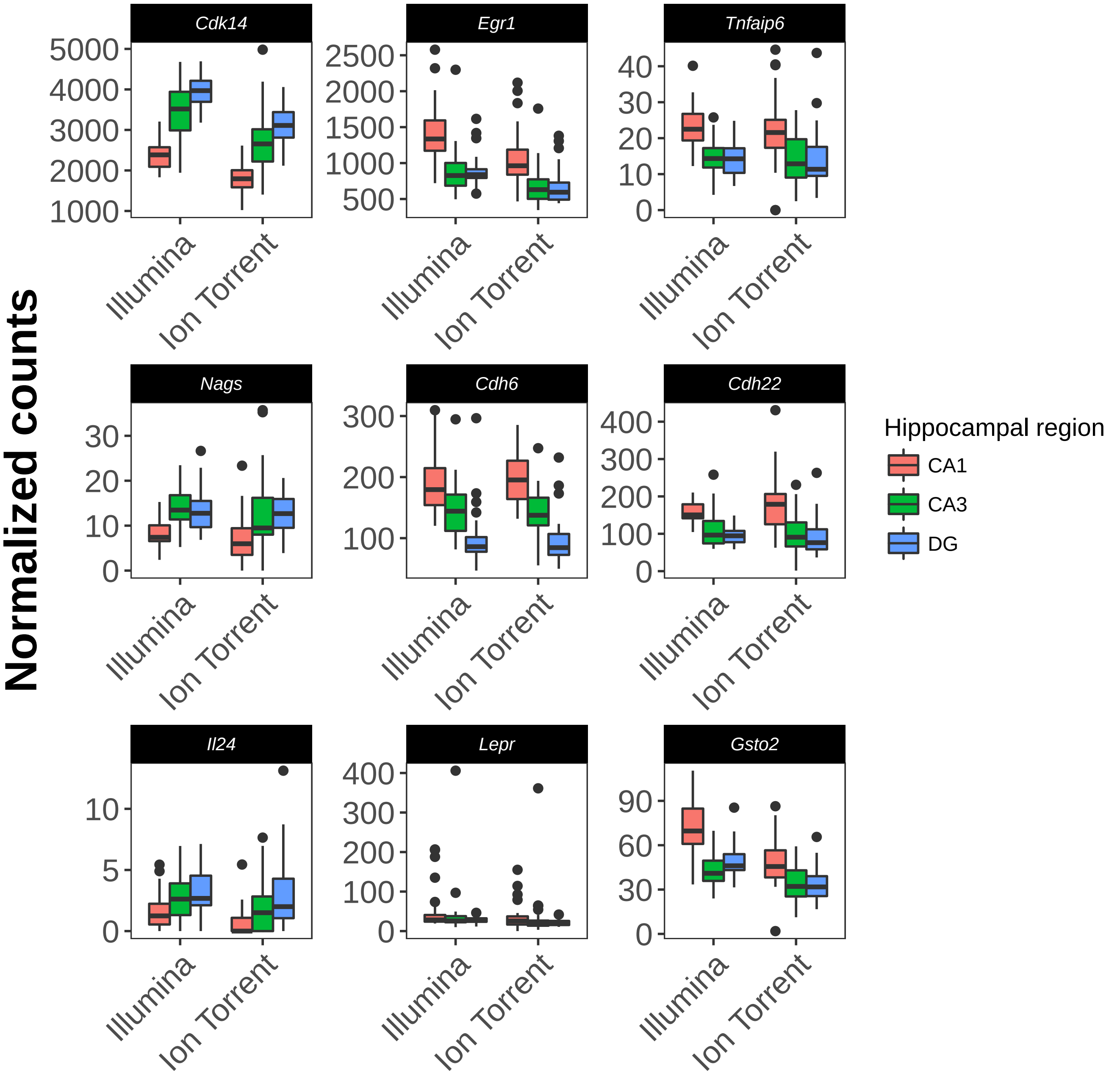

Normalized counts
Cdk14
Egr1
Tnfaip6
Nags
Cdh6
Cdh22
Il24
Lepr
Gsto2
Illumina
Ion Torrent
Hippocampal region
CA1
CA3
DG

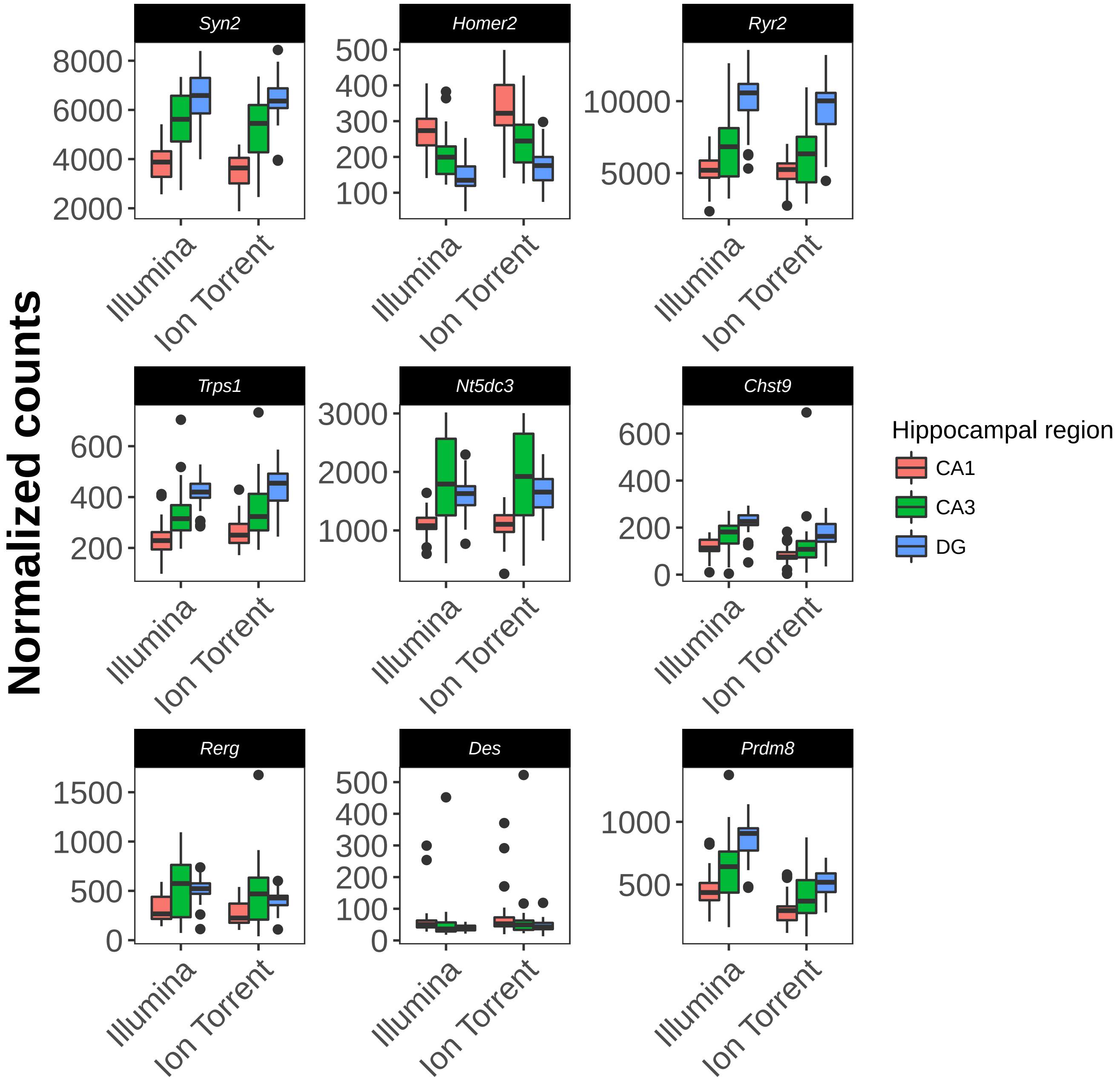

Normalized counts
Syn2
Homer2
Ryr2
Trps1
Nt5dc3
Chst9
Rerg
Des
Prdm8
Illumina
Ion Torrent
Hippocampal region
CA1
CA3
DG

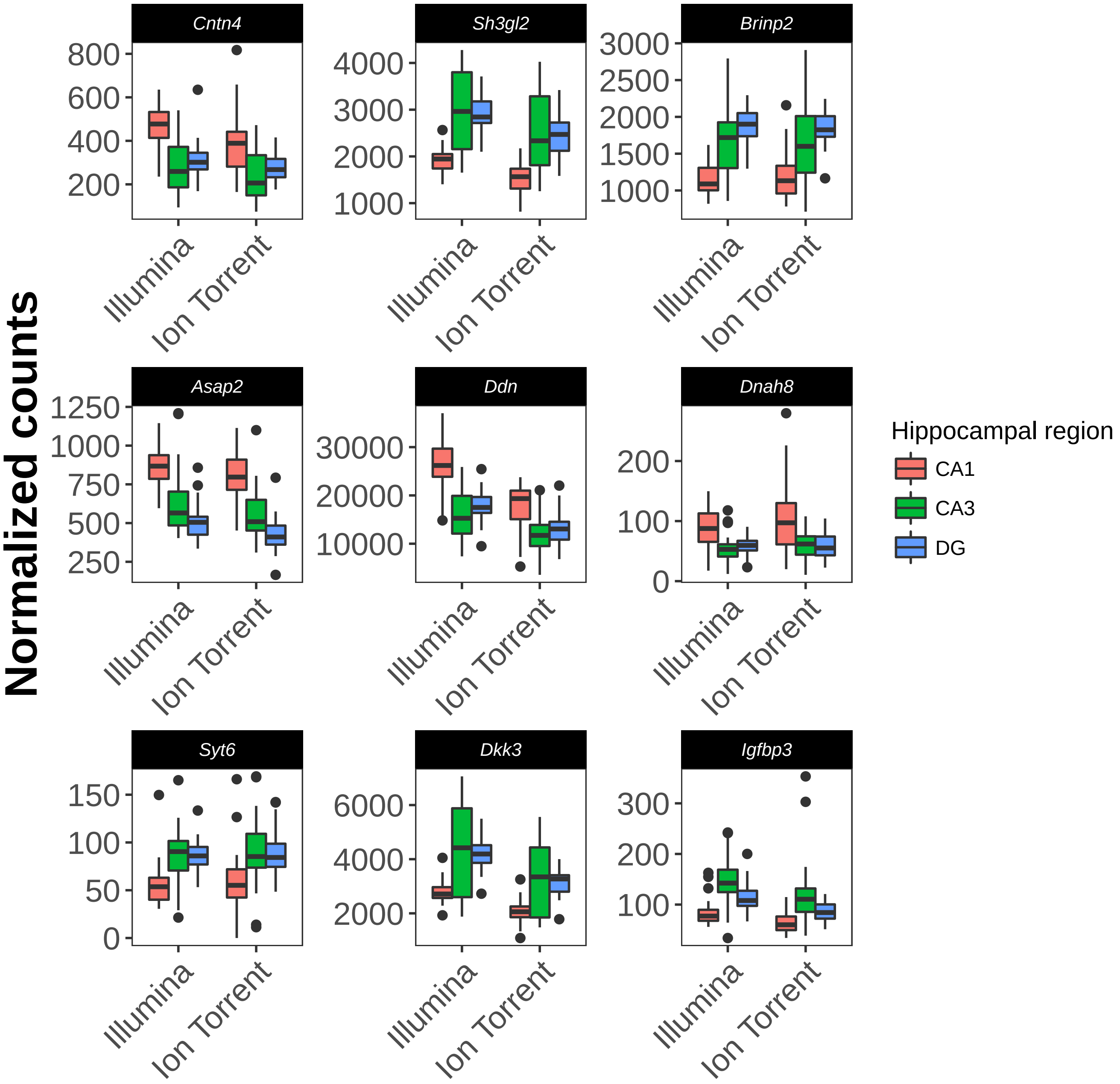
Normalized counts
Cntn4
Sh3gl2
Brinp2
Asap2
Ddn
Dnah8
Syt6
Dkk3
Igfbp3
Illumina
Ion Torrent
Hippocampal region
CA1
CA3
DG

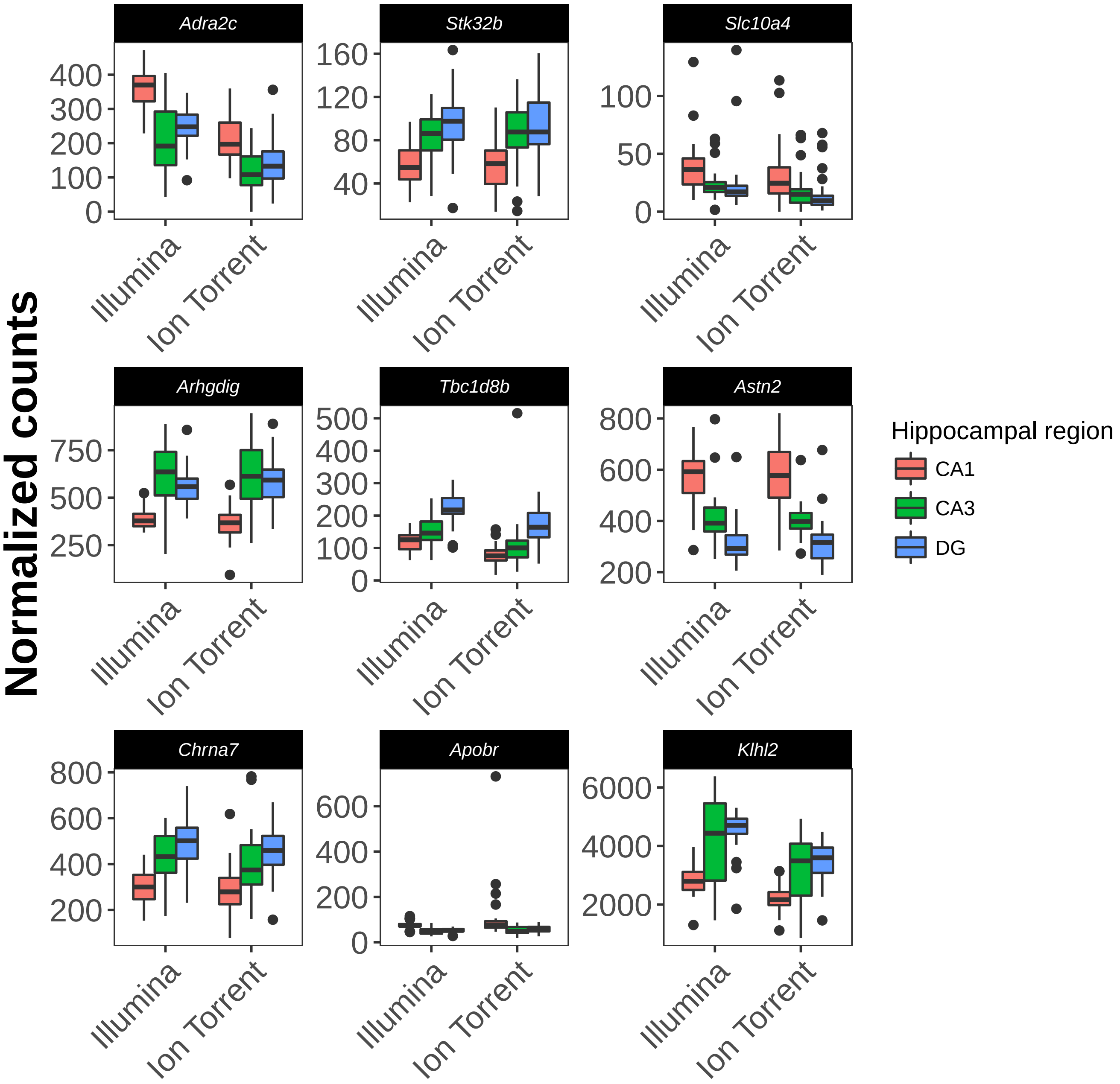
Normalized counts
Adra2c
Stk32b
Slc10a4
Arhgdig
Tbc1d8b
Astn2
Chrna7
Apobr
Klhl2
Illumina
Ion Torrent
Hippocampal region
CA1
CA3
DG

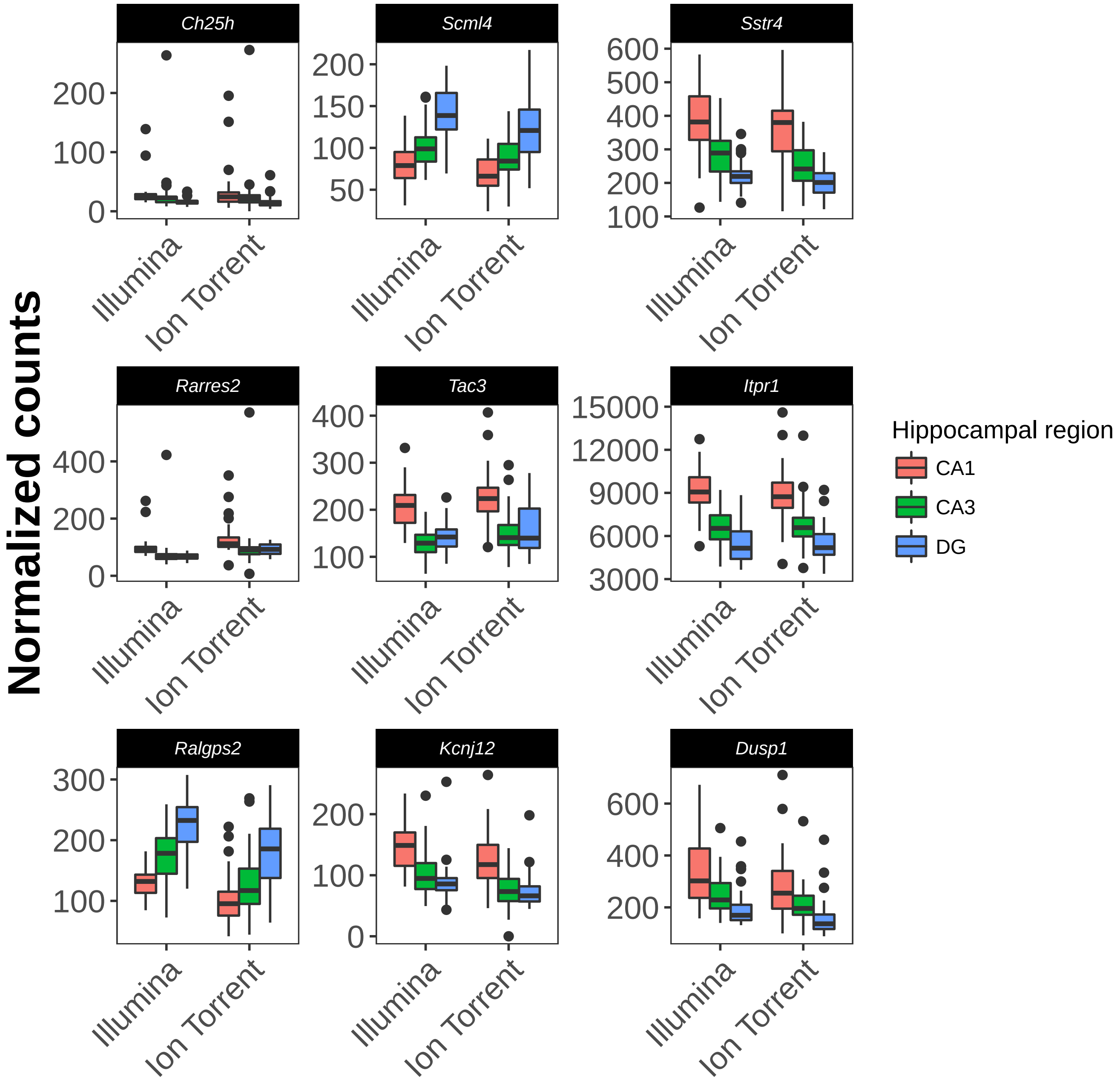
Normalized counts
Ch25h
Scml4
Sstr4
Rarres2
Tac3
Itpr1
Ralgps2
Kcnj12
Dusp1
Illumina
Ion Torrent
Hippocampal region
CA1
CA3
DG

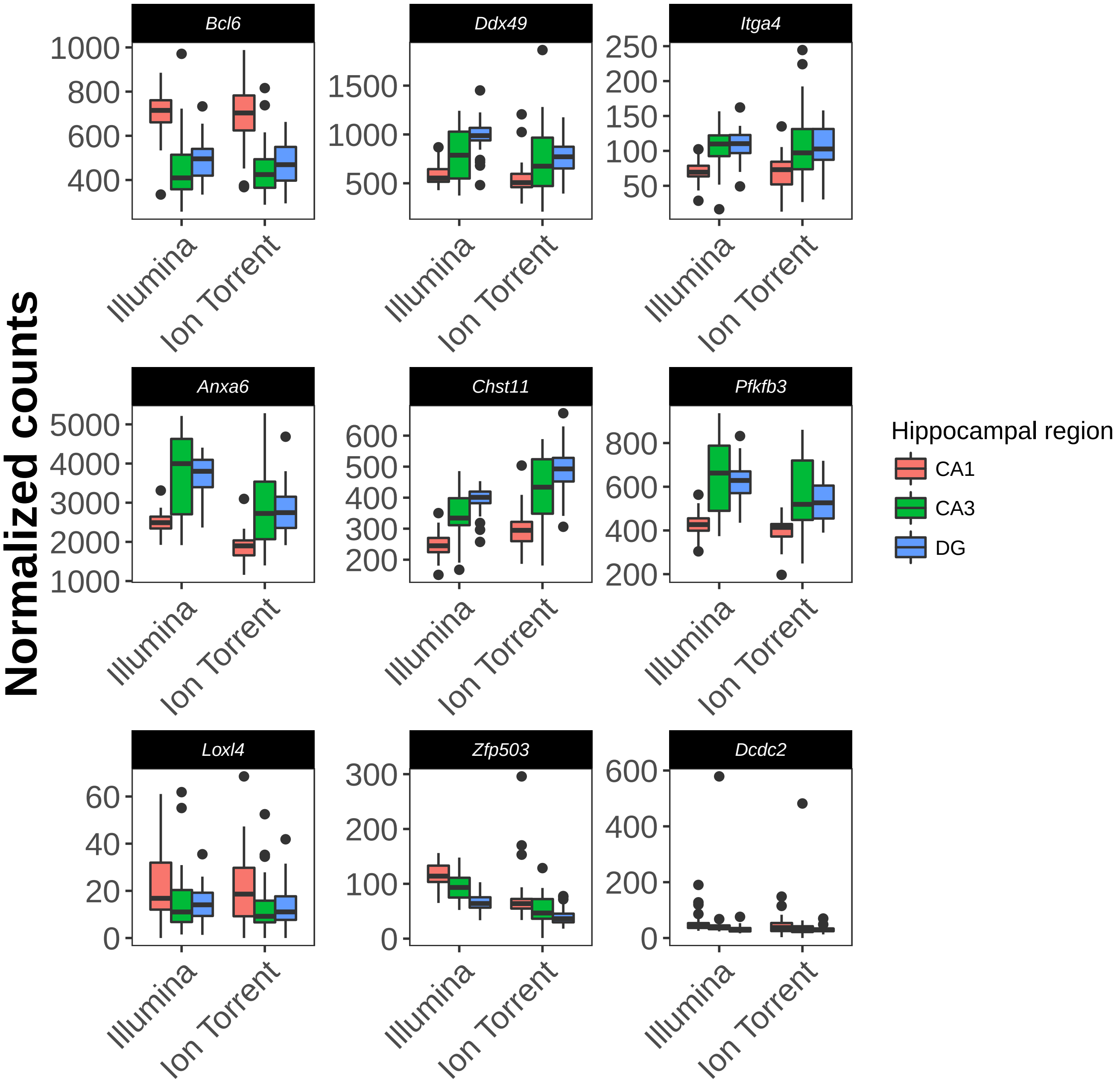

Normalized counts
Bcl6
Ddx49
Itga4
Anxa6
Chst11
Pfkfb3
Loxl4
Zfp503
Dcdc2
Illumina
Ion Torrent
Hippocampal region
CA1
CA3
DG

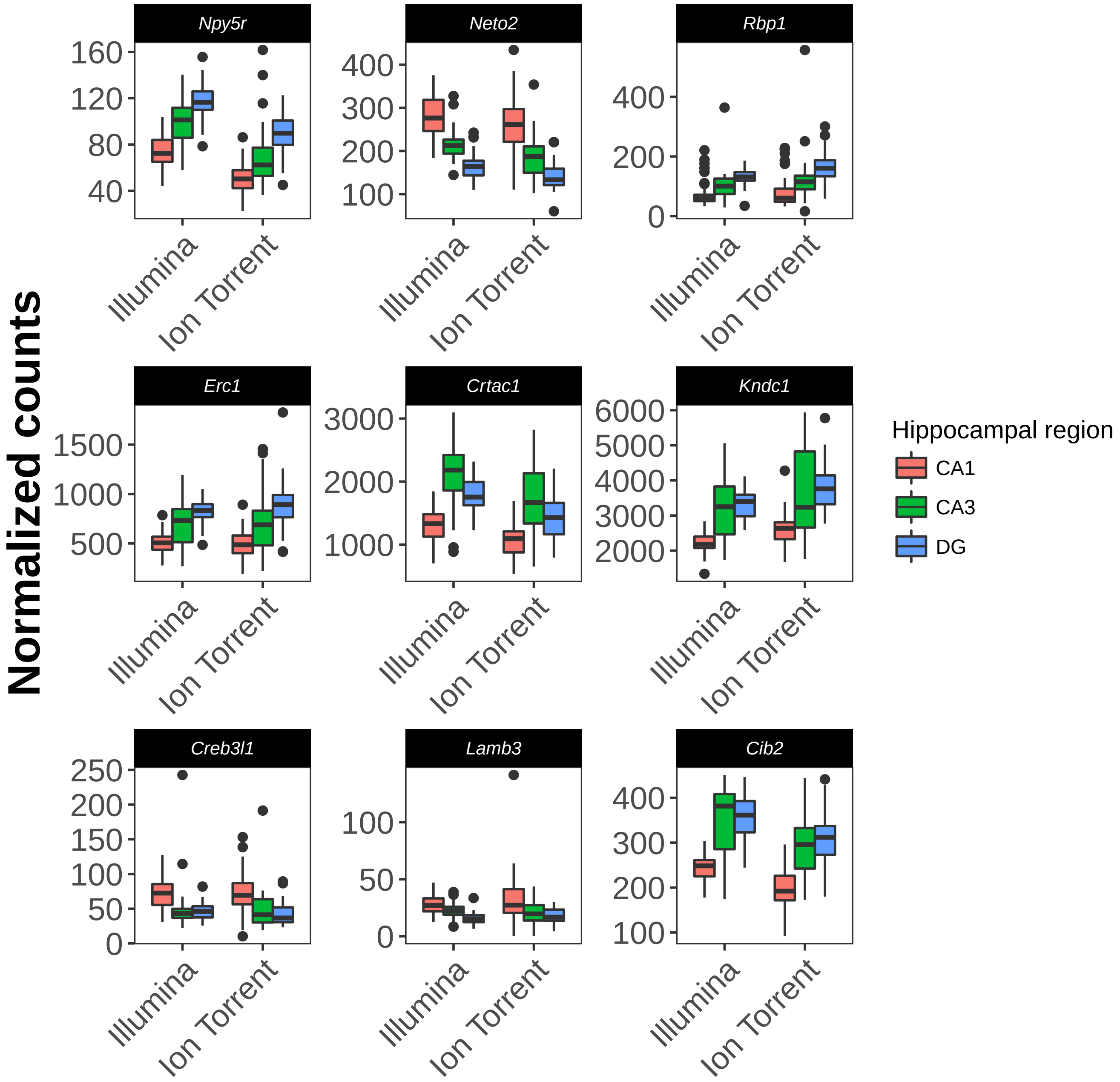
Normalized counts
Npy5r
Neto2
Rbp1
Erc1
Crtac1
Kndc1
Creb3l1
Lamb3
Cib2
Illumina
Ion Torrent
Hippocampal region
CA1
CA3
DG

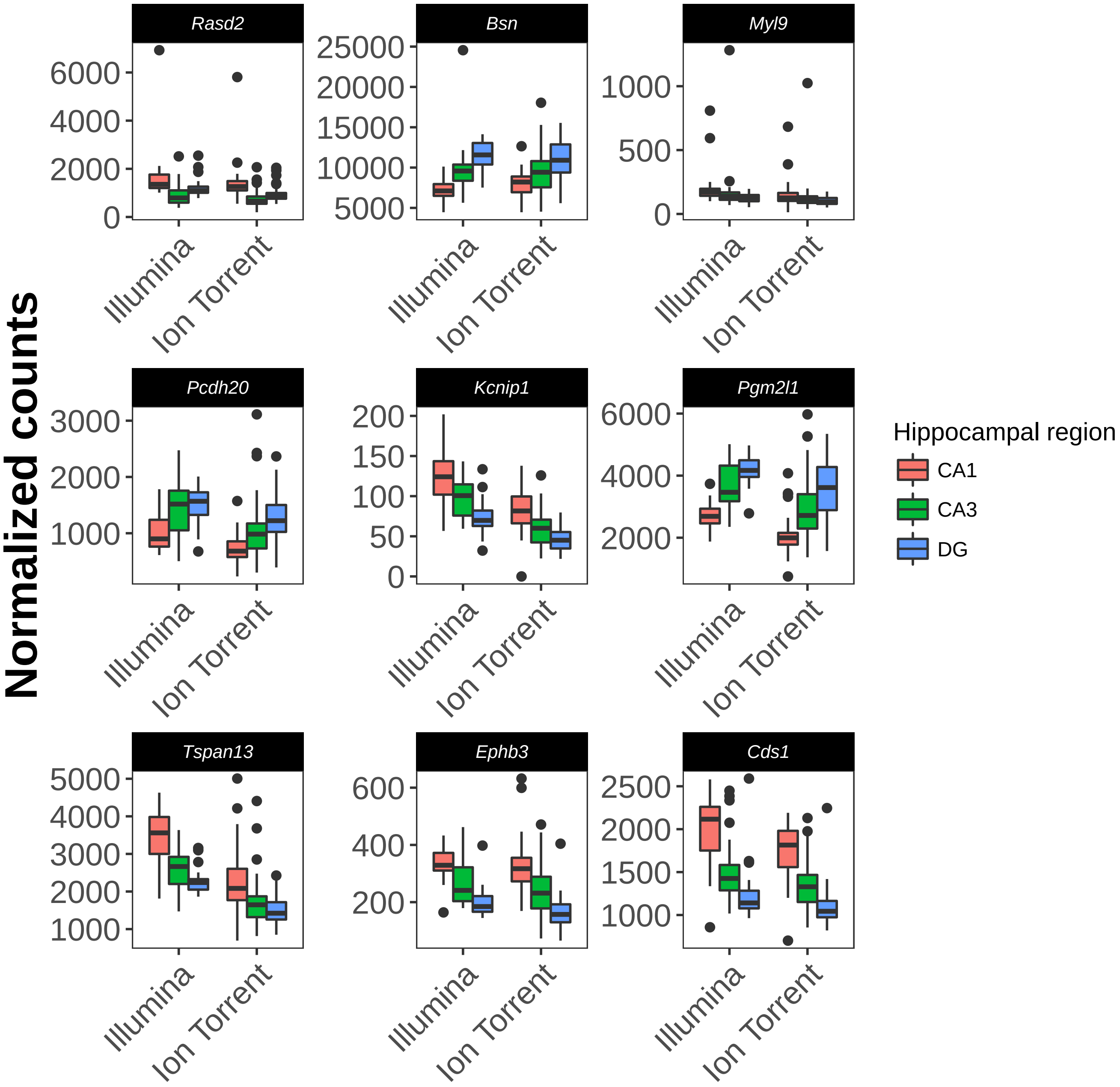
Normalized counts
Rasd2
Bsn
Myl9
Pcdh20
Kcnip1
Pgm2l1
Tspan13
Ephb3
Cds1
Illumina
Ion Torrent
Hippocampal region
CA1
CA3
DG

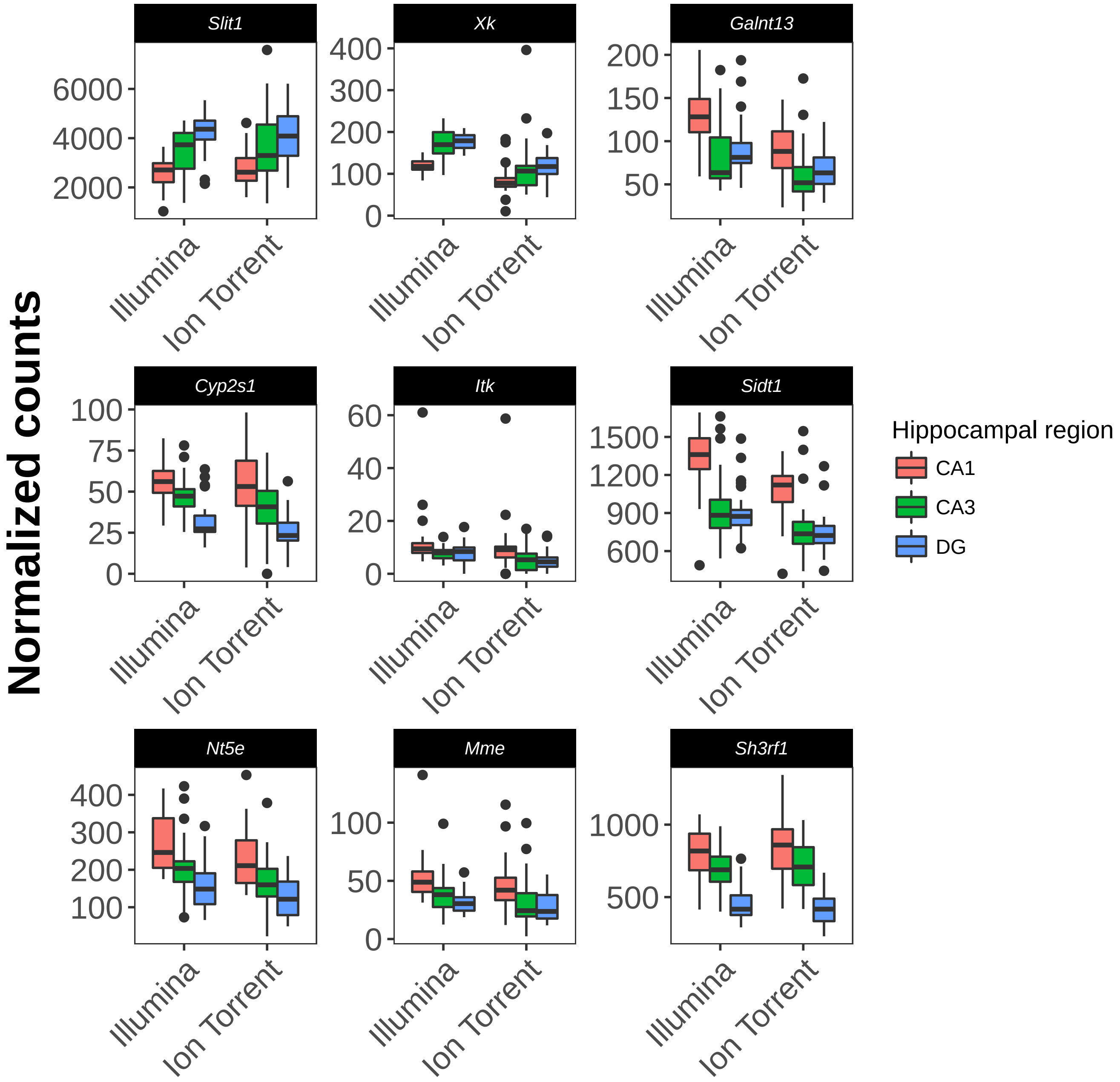
Normalized counts
Slit1
Xk
Galnt13
Cyp2s1
Itk
Sidt1
Nt5e
Mme
Sh3rf1
Illumina
Ion Torrent
Hippocampal region
CA1
CA3
DG

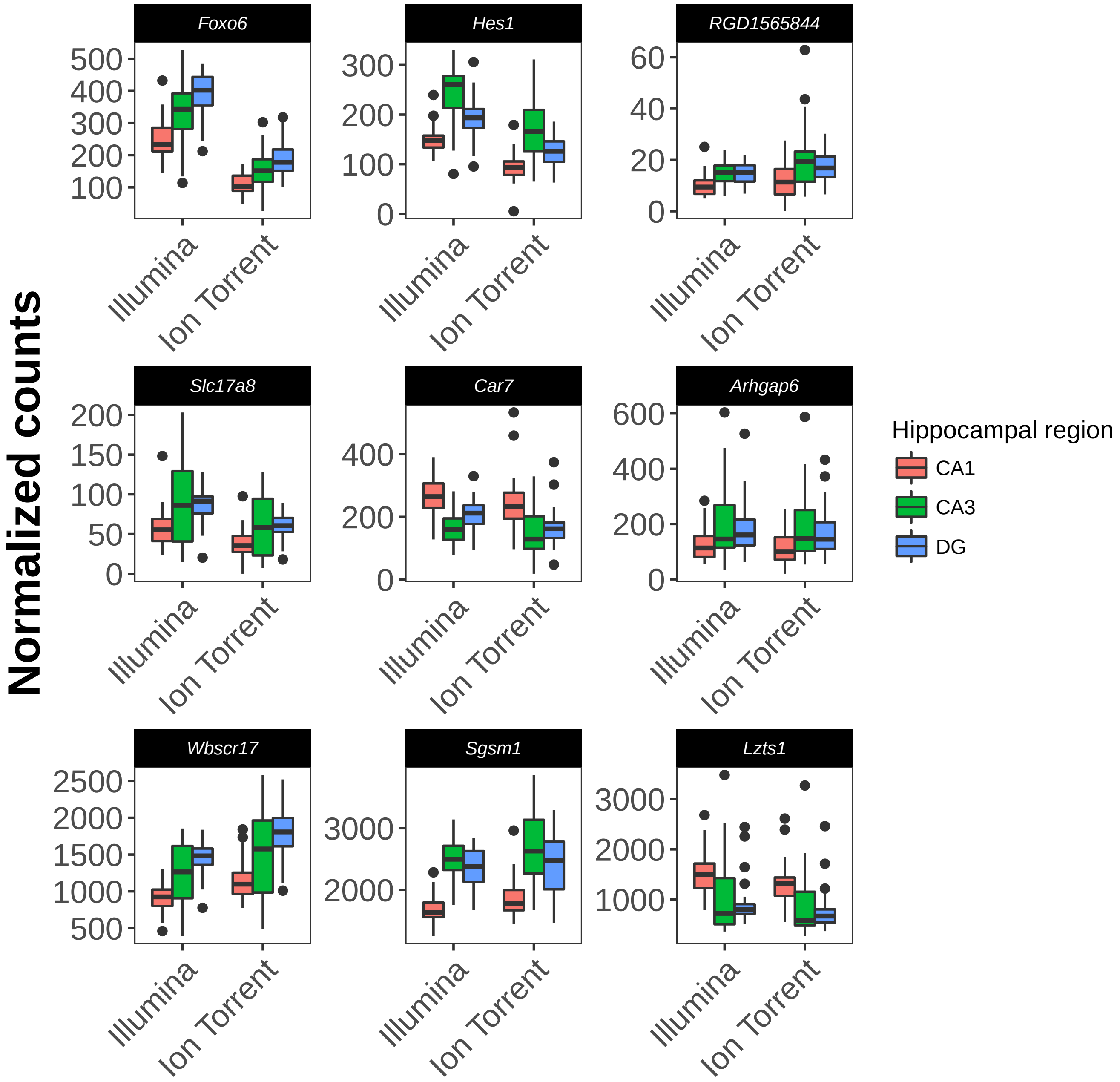

Normalized counts
Foxo6
Hes1
RGD1565844
Slc17a8
Car7
Arhgap6
Wbscr17
Sgsm1
Lzts1
Illumina
Ion Torrent
Hippocampal region
CA1
CA3
DG

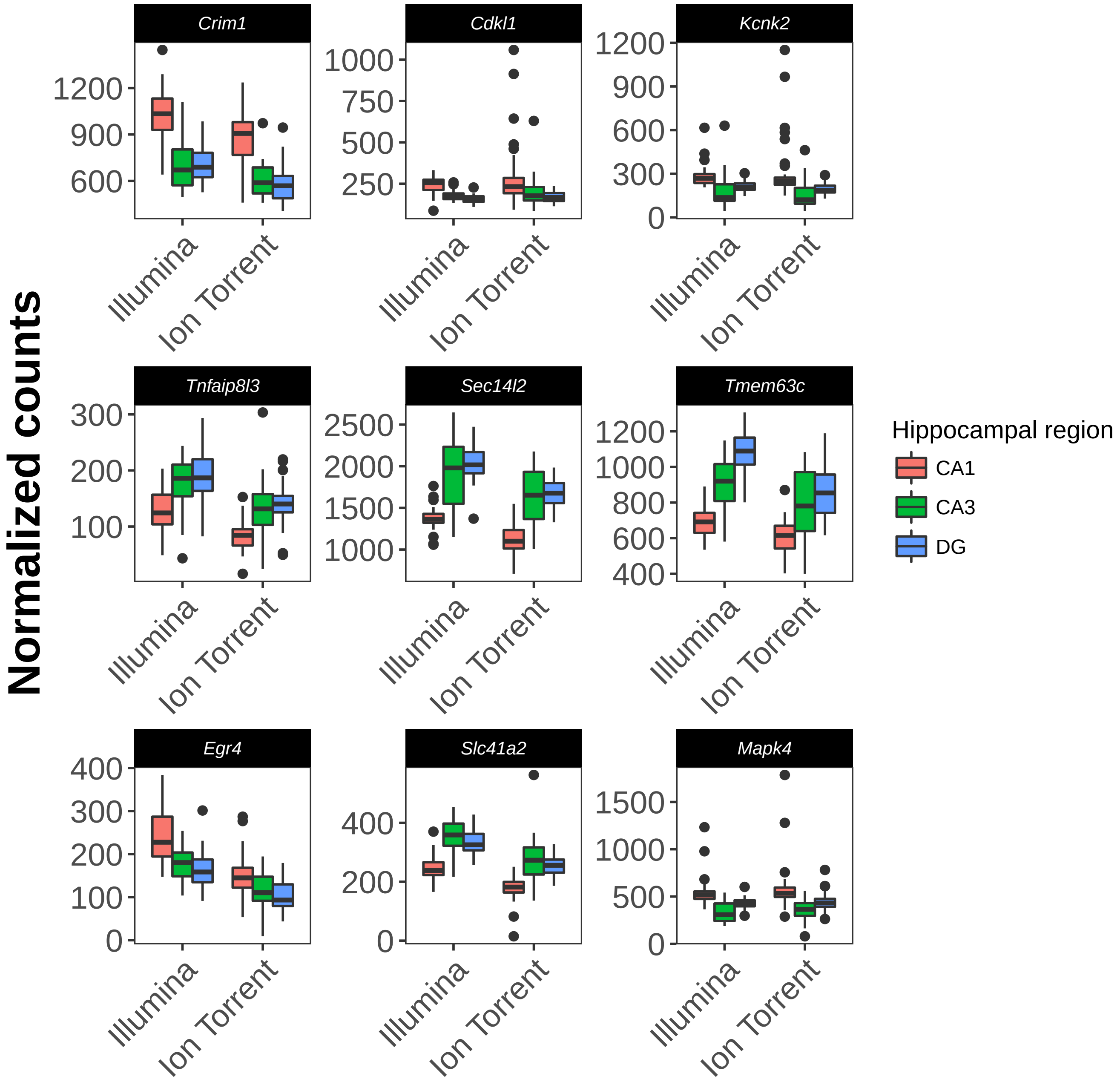
Normalized counts
Crim1
Cdkl1
Kcnk2
Tnfaip8l3
Sec14l2
Tmem63c
Egr4
Slc41a2
Mapk4
Illumina
Ion Torrent
Hippocampal region
CA1
CA3
DG

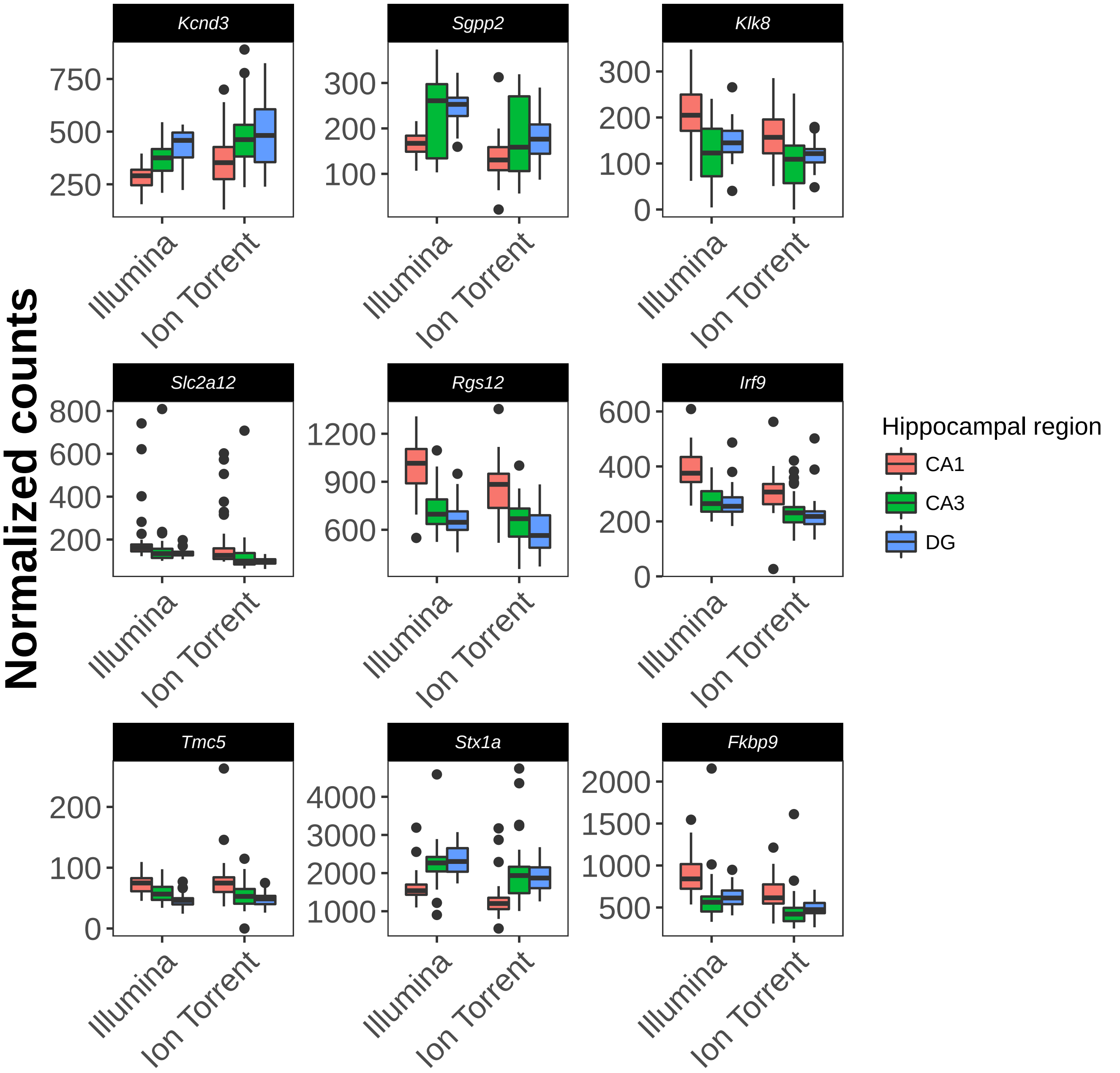

Normalized counts
Kcnd3
Sgpp2
Klk8
Slc2a12
Rgs12
Irf9
Tmc5
Stx1a
Fkbp9
Illumina
Ion Torrent
Hippocampal region
CA1
CA3
DG

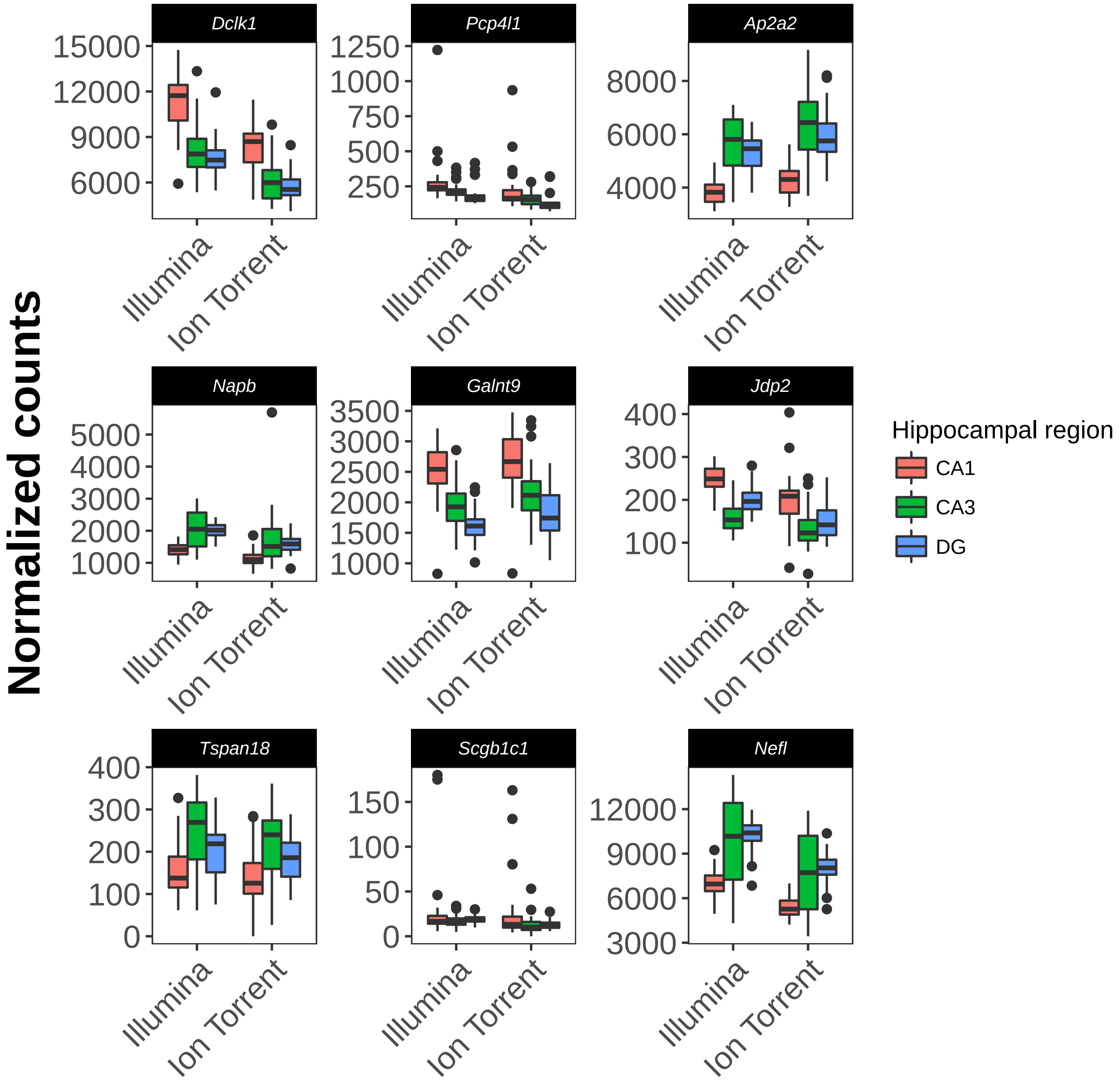

Normalized counts
Dclk1
Pcp4l1
Ap2a2
Napb
Galnt9
Jdp2
Tspan18
Scgb1c1
Nefl
Illumina
Ion Torrent
Hippocampal region
CA1
CA3
DG

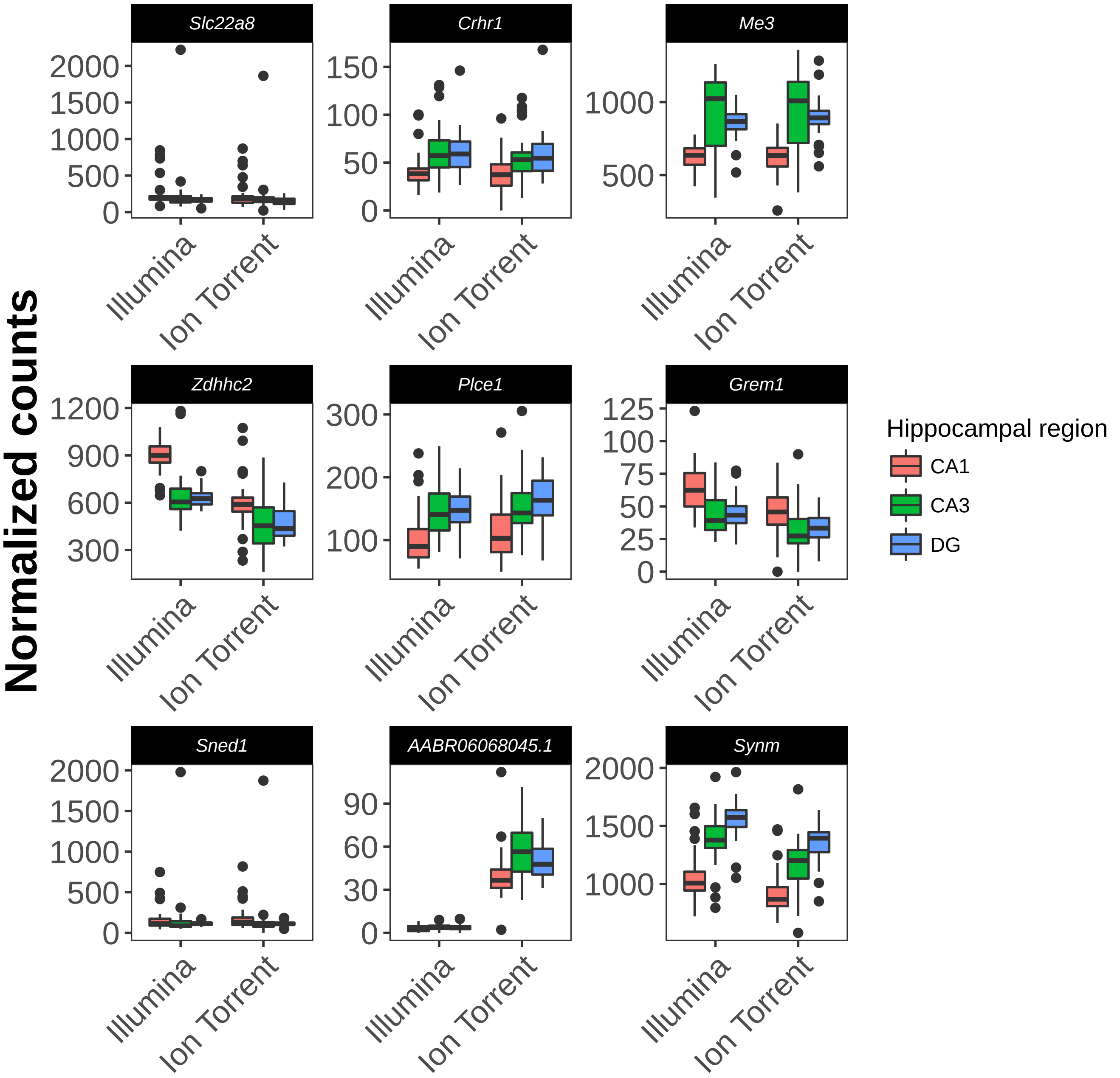
Slc22a8
Crhr1
Me3
Zdhhc2
Plce1
Grem1
Sned1
AABR06068045.1
Synm
Illumina
Ion Torrent
Normalized counts
Hippocampal region
CA1
CA3
DG

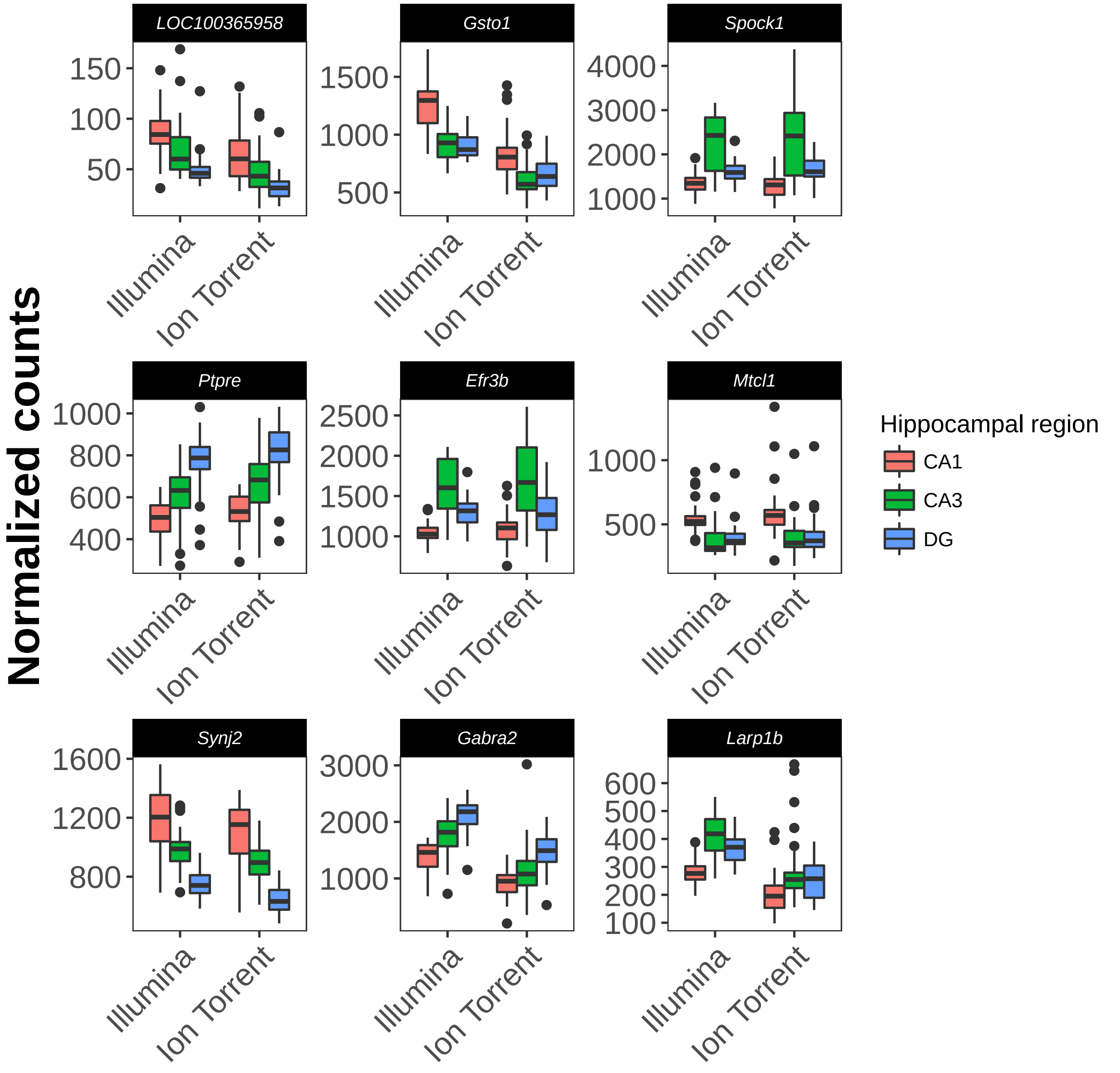

Normalized counts
LOC100365958
Gsto1
Spock1
Ptpre
Efr3b
Mtcl1
Synj2
Gabra2
Larp1b
Illumina
Ion Torrent
Hippocampal region
CA1
CA3
DG

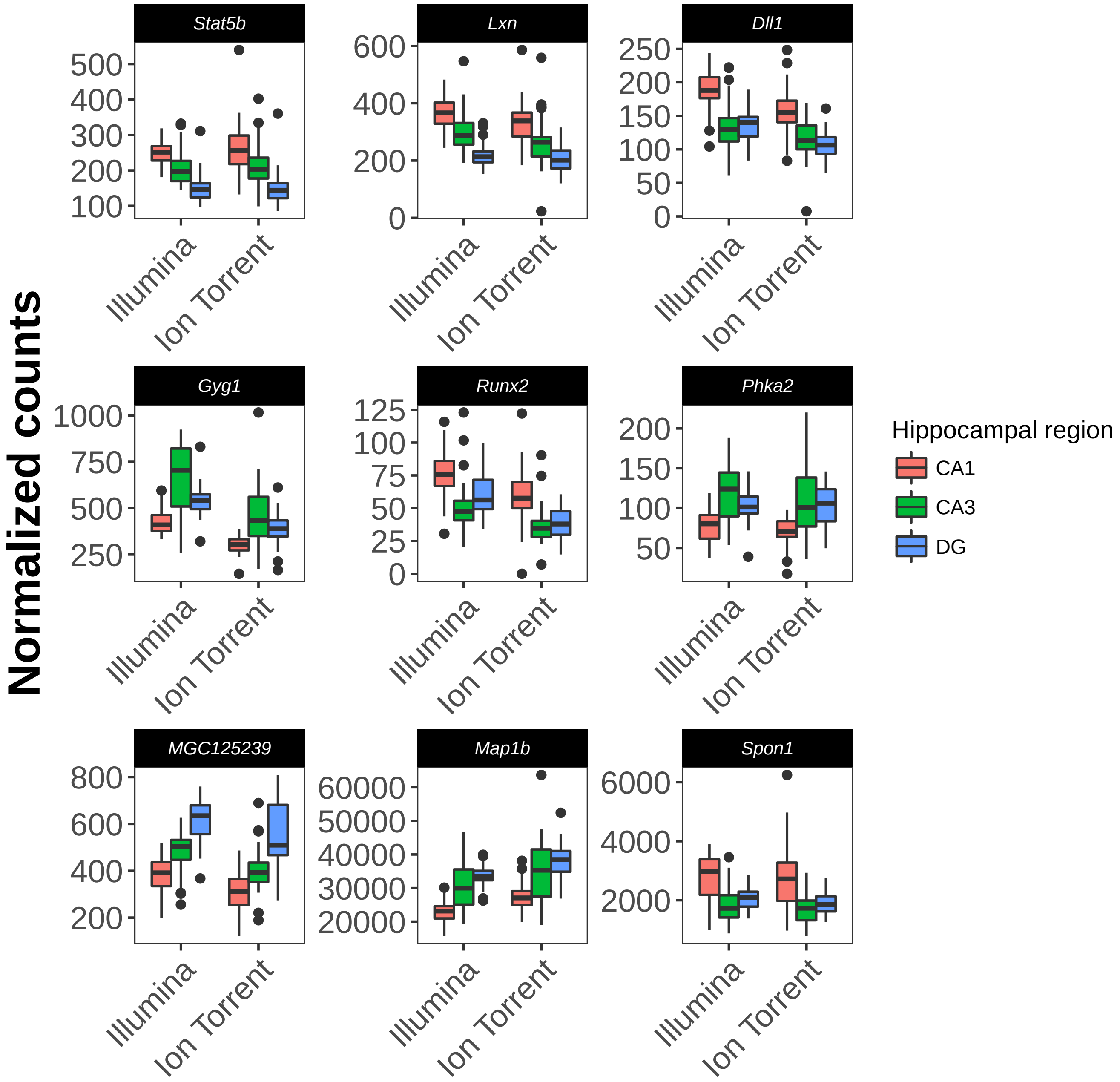
Normalized counts
Stat5b
Lxn
Dll1
Gyg1
Runx2
Phka2
MGC125239
Map1b
Spon1
Illumina
Ion Torrent
Hippocampal region
CA1
CA3
DG

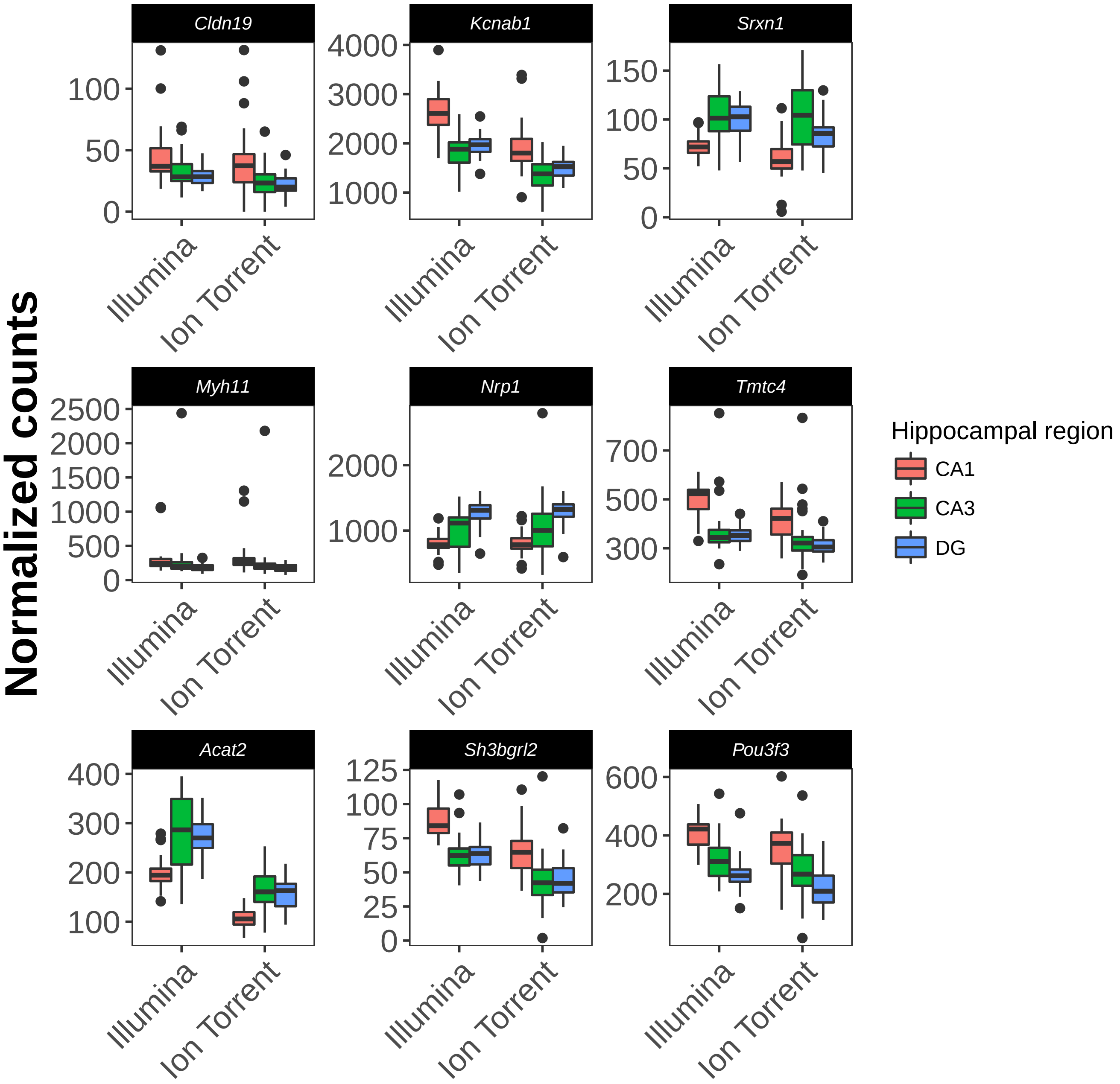
Normalized counts
Cldn19
Kcnab1
Srxn1
Myh11
Nrp1
Tmtc4
Acat2
Sh3bgrl2
Pou3f3
Illumina
Ion Torrent
Hippocampal region
CA1
CA3
DG

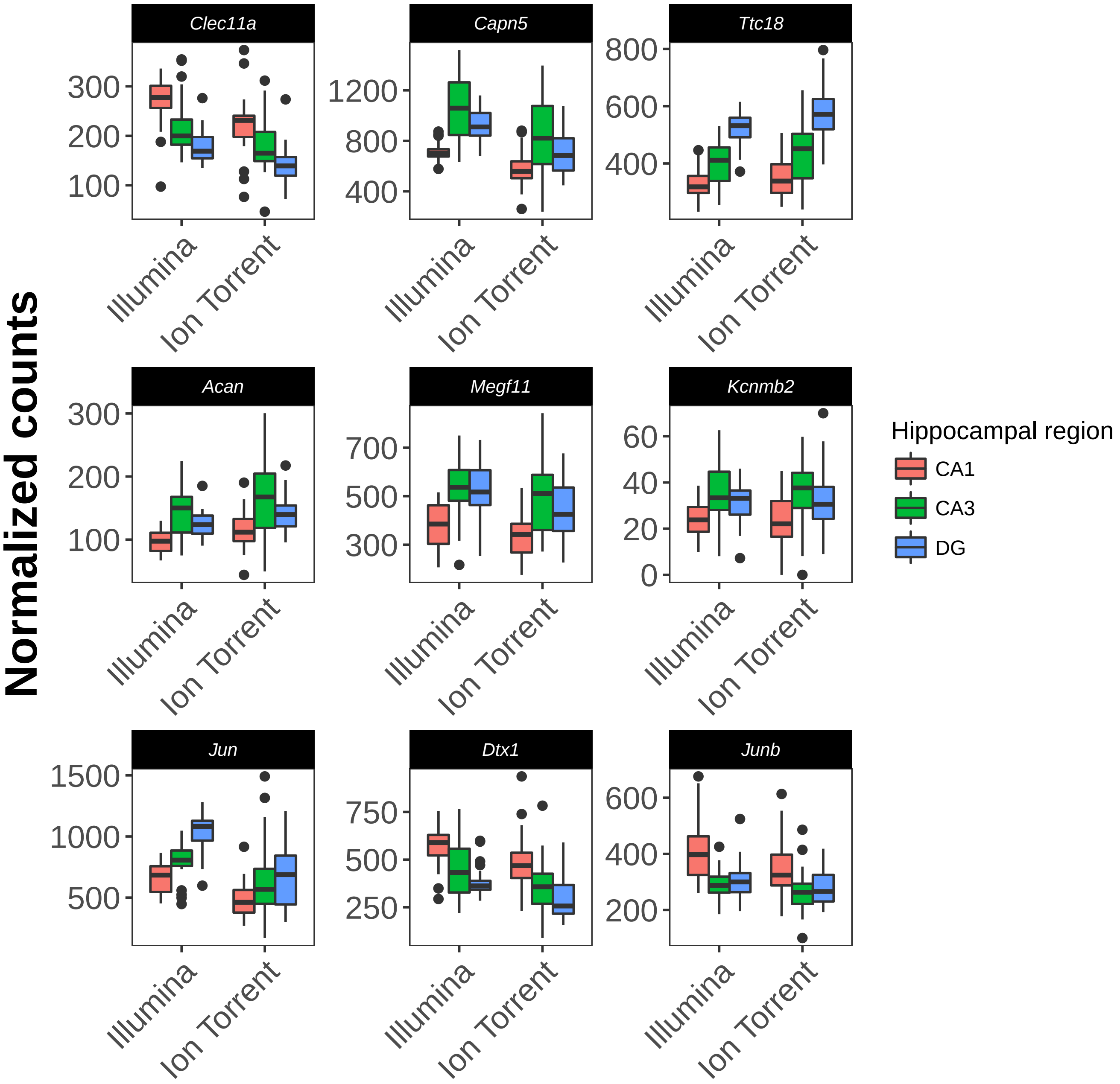
Normalized counts
Clec11a
Capn5
Ttc18
Acan
Megf11
Kcnmb2
Jun
Dtx1
Junb
Illumina
Ion Torrent
Hippocampal region
CA1
CA3
DG

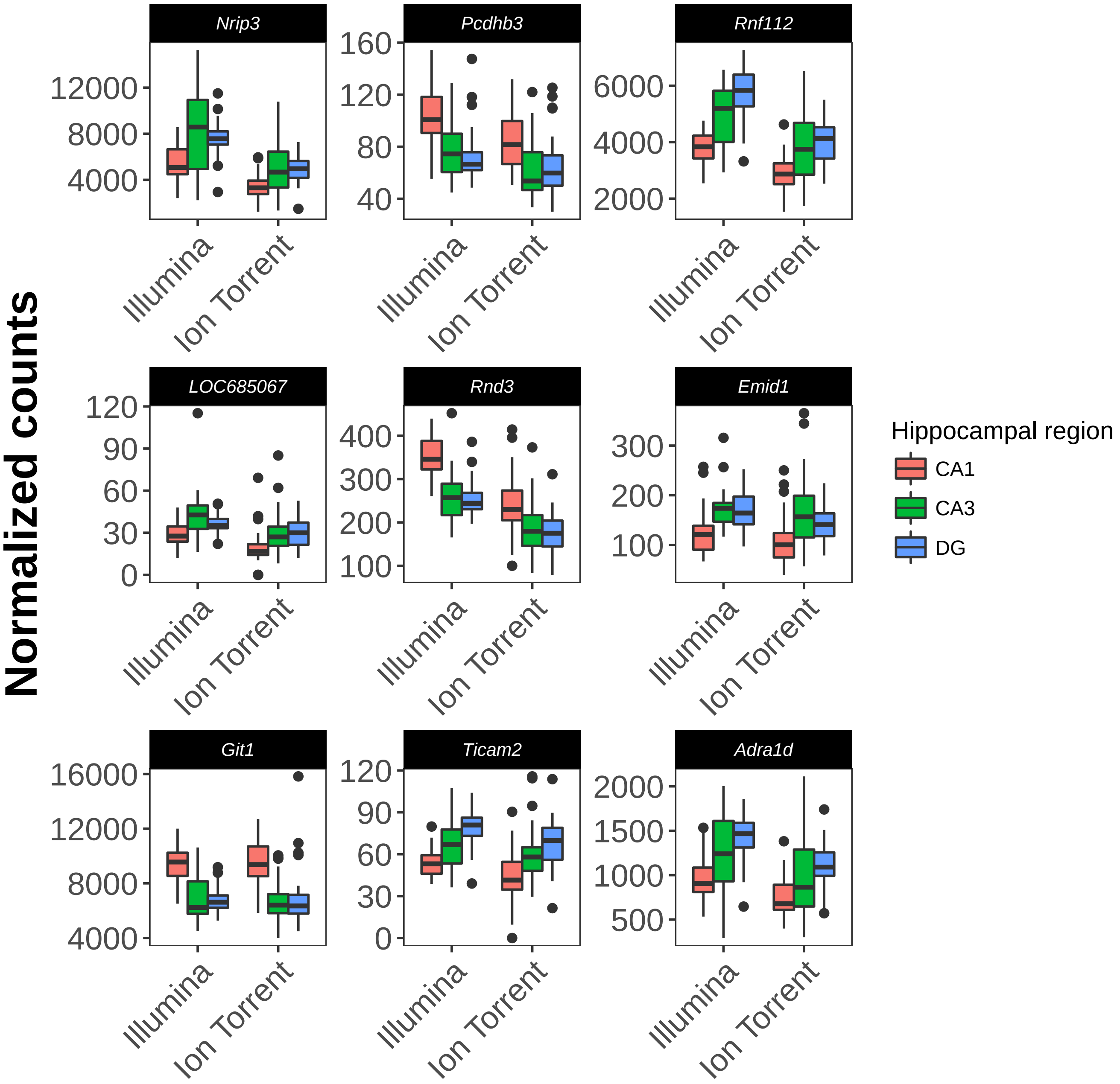
Normalized counts
Nrip3
Pcdhb3
Rnf112
LOC685067
Rnd3
Emid1
Git1
Ticam2
Adra1d
Illumina
Ion Torrent
Hippocampal region
CA1
CA3
DG

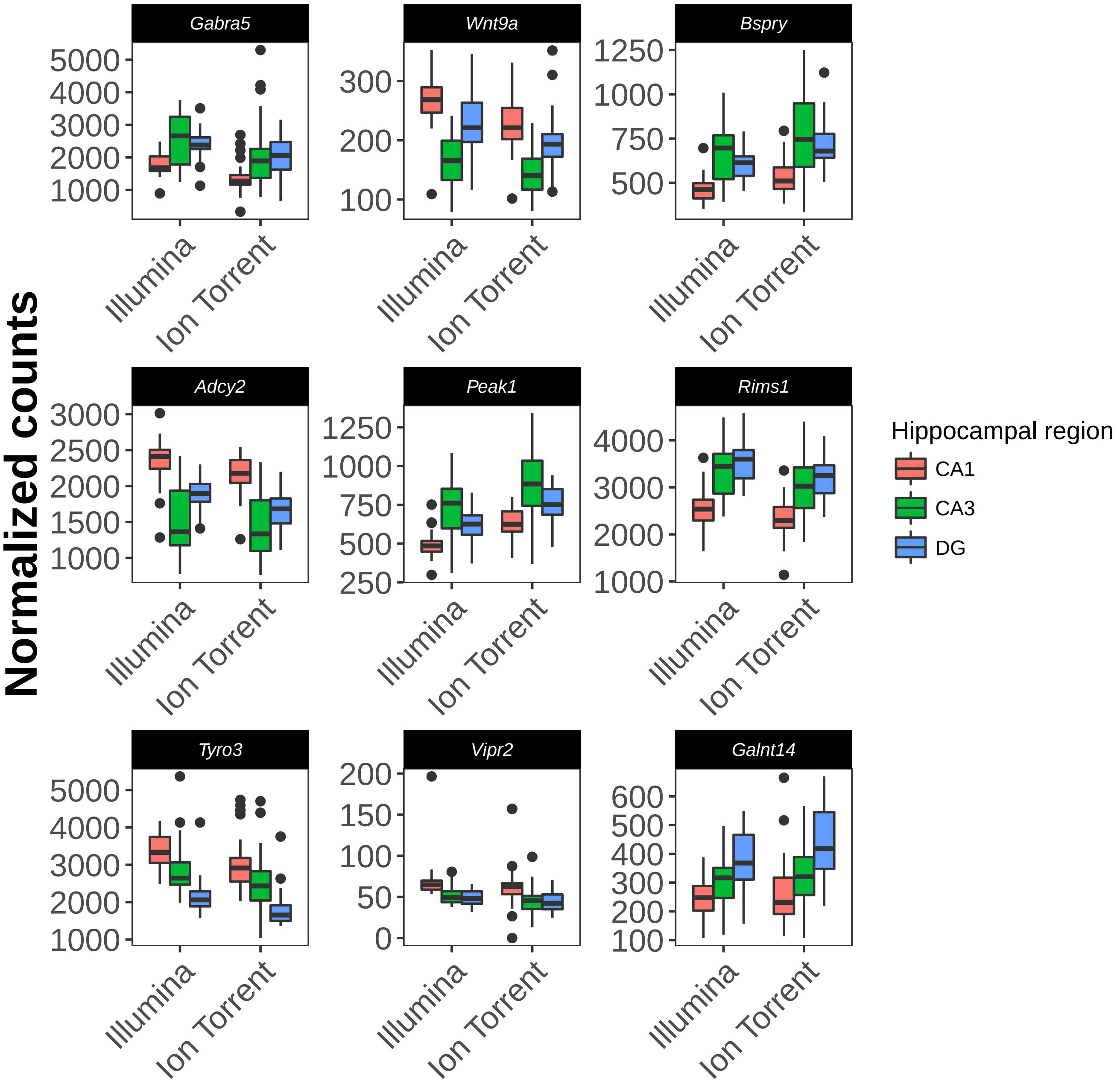
Normalized counts
Gabra5
Wnt9a
Bspry
Adcy2
Peak1
Rims1
Tyro3
Vipr2
Galnt14
Illumina
Ion Torrent
Hippocampal region
CA1
CA3
DG

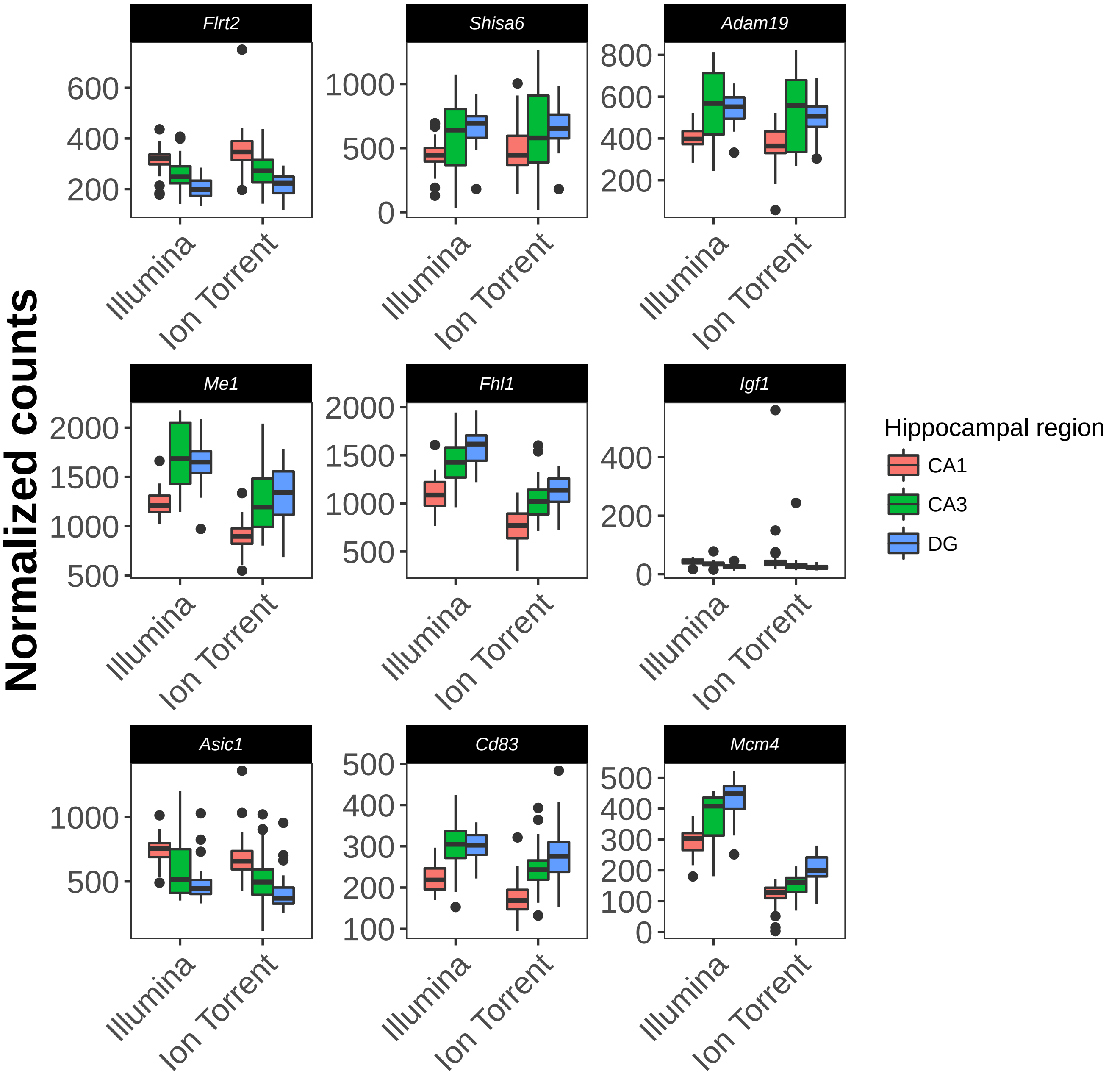

Normalized counts
Flrt2
Shisa6
Adam19
Me1
Fhl1
Igf1
Asic1
Cd83
Mcm4
Illumina
Ion Torrent
Hippocampal region
CA1
CA3
DG

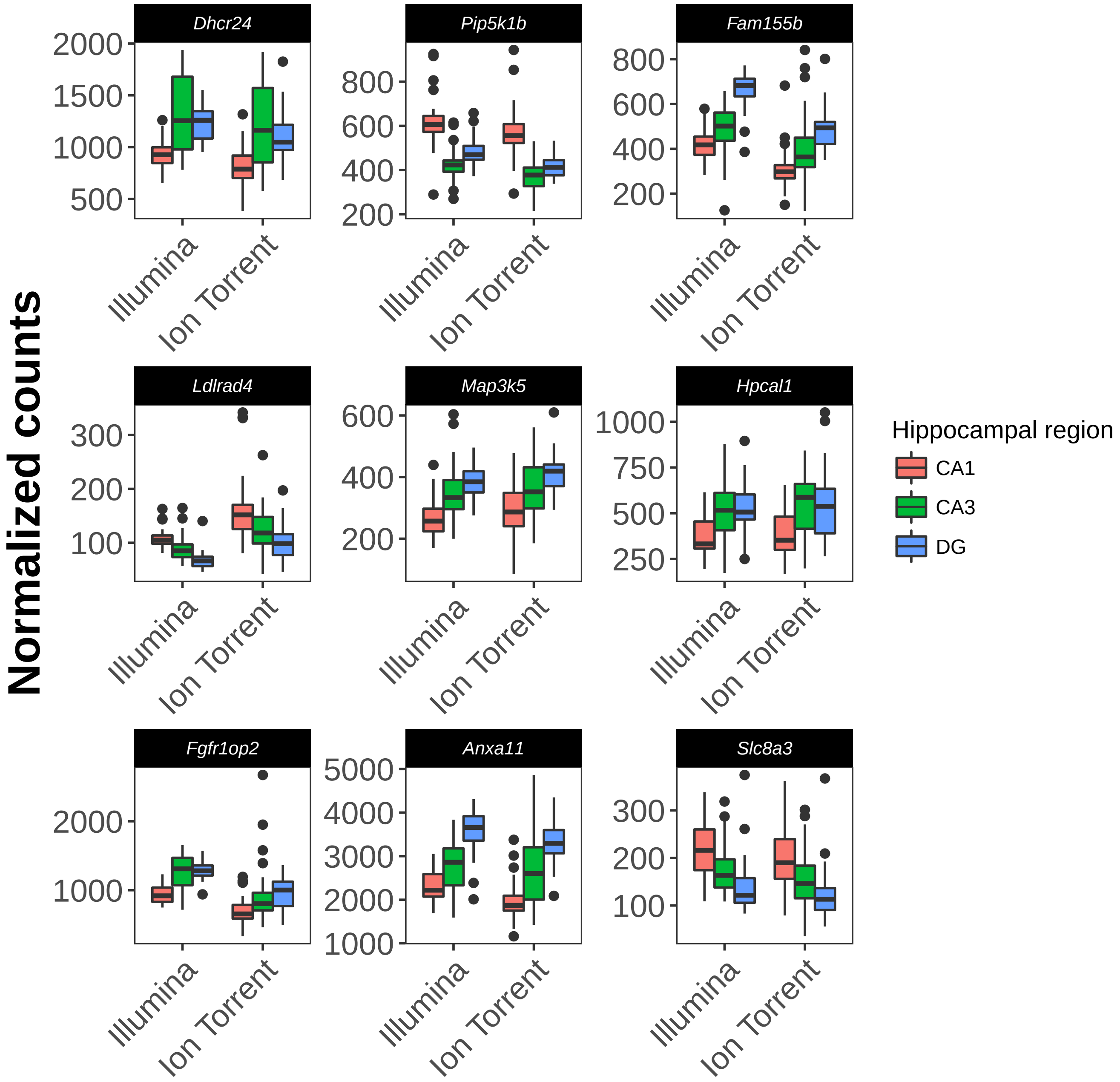
Normalized counts
Dhcr24
Pip5k1b
Fam155b
Ldlrad4
Map3k5
Hpcal1
Fgfr1op2
Anxa11
Slc8a3
Illumina
Ion Torrent
Hippocampal region
CA1
CA3
DG

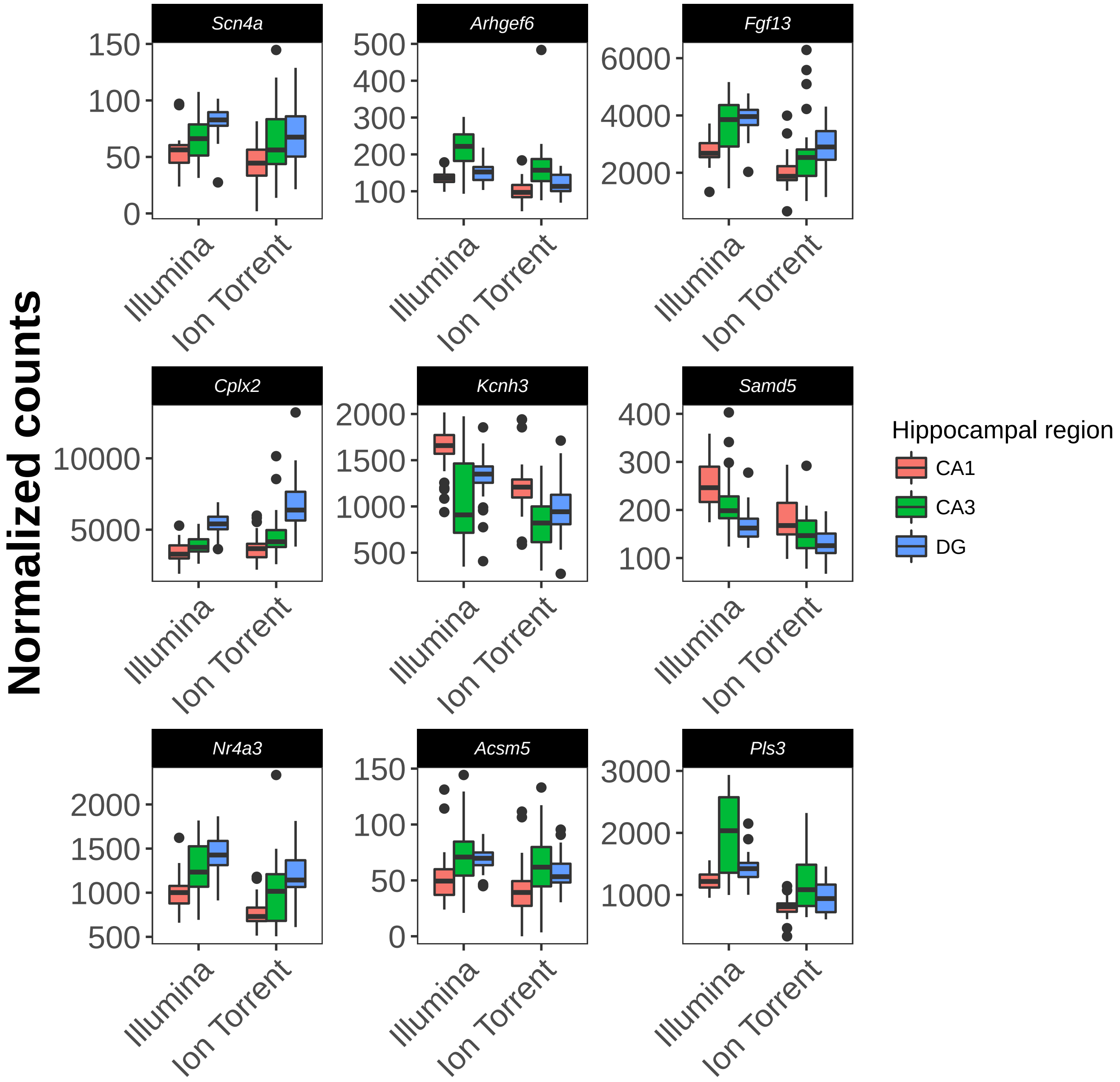
Normalized counts
Scn4a
Arhgef6
Fgf13
Cplx2
Kcnh3
Samd5
Nr4a3
Acsm5
Pls3
Illumina
Ion Torrent
Hippocampal region
CA1
CA3
DG

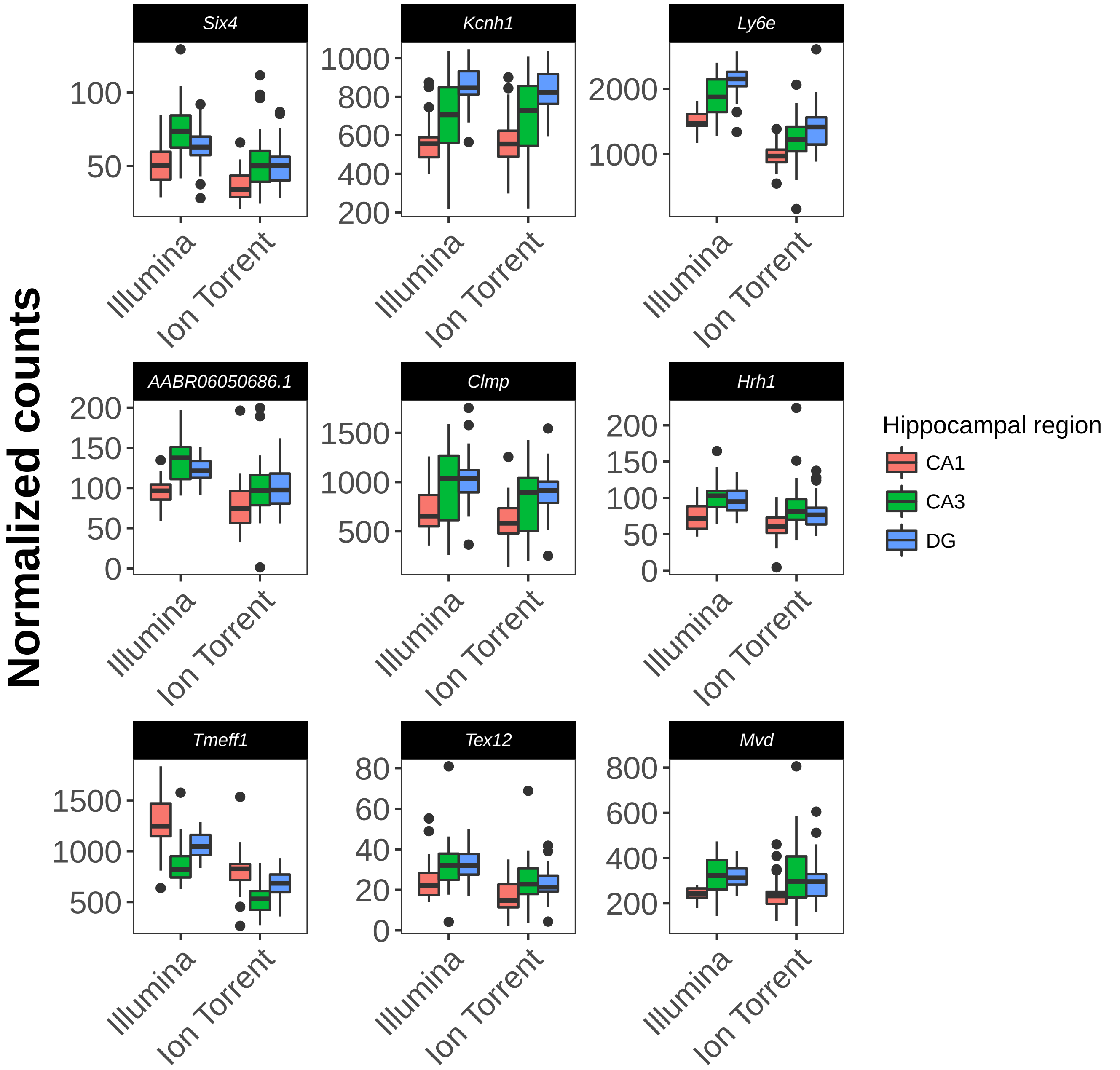
Normalized counts
Six4
Kcnh1
Ly6e
AABR06050686.1
Clmp
Hrh1
Tmeff1
Tex12
Mvd
Illumina
Ion Torrent
Hippocampal region
CA1
CA3
DG

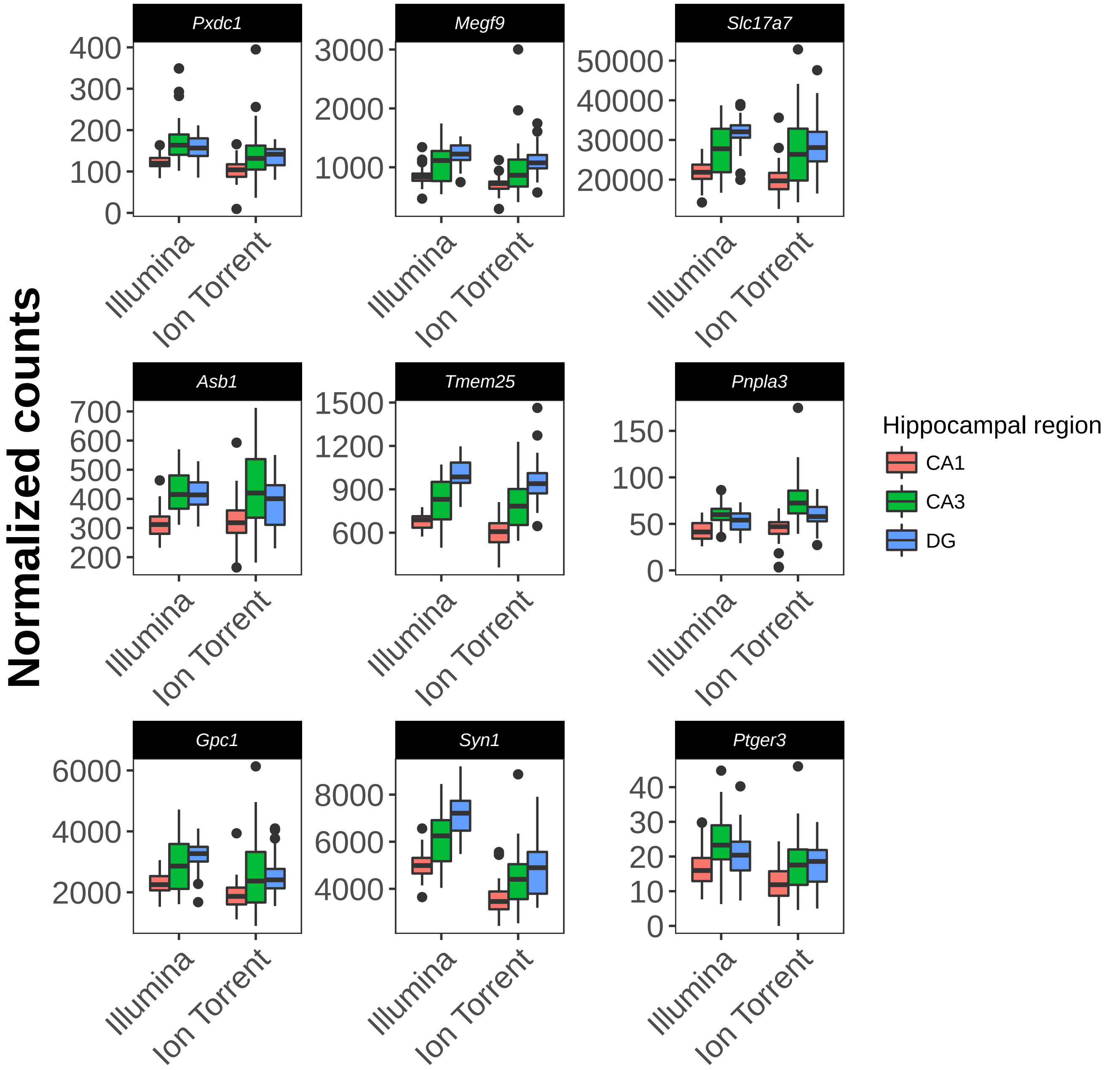
Normalized counts
Pxdc1
Megf9
Slc17a7
Asb1
Tmem25
Pnpla3
Gpc1
Syn1
Ptger3
Illumina
Ion Torrent
Hippocampal region
CA1
CA3
DG

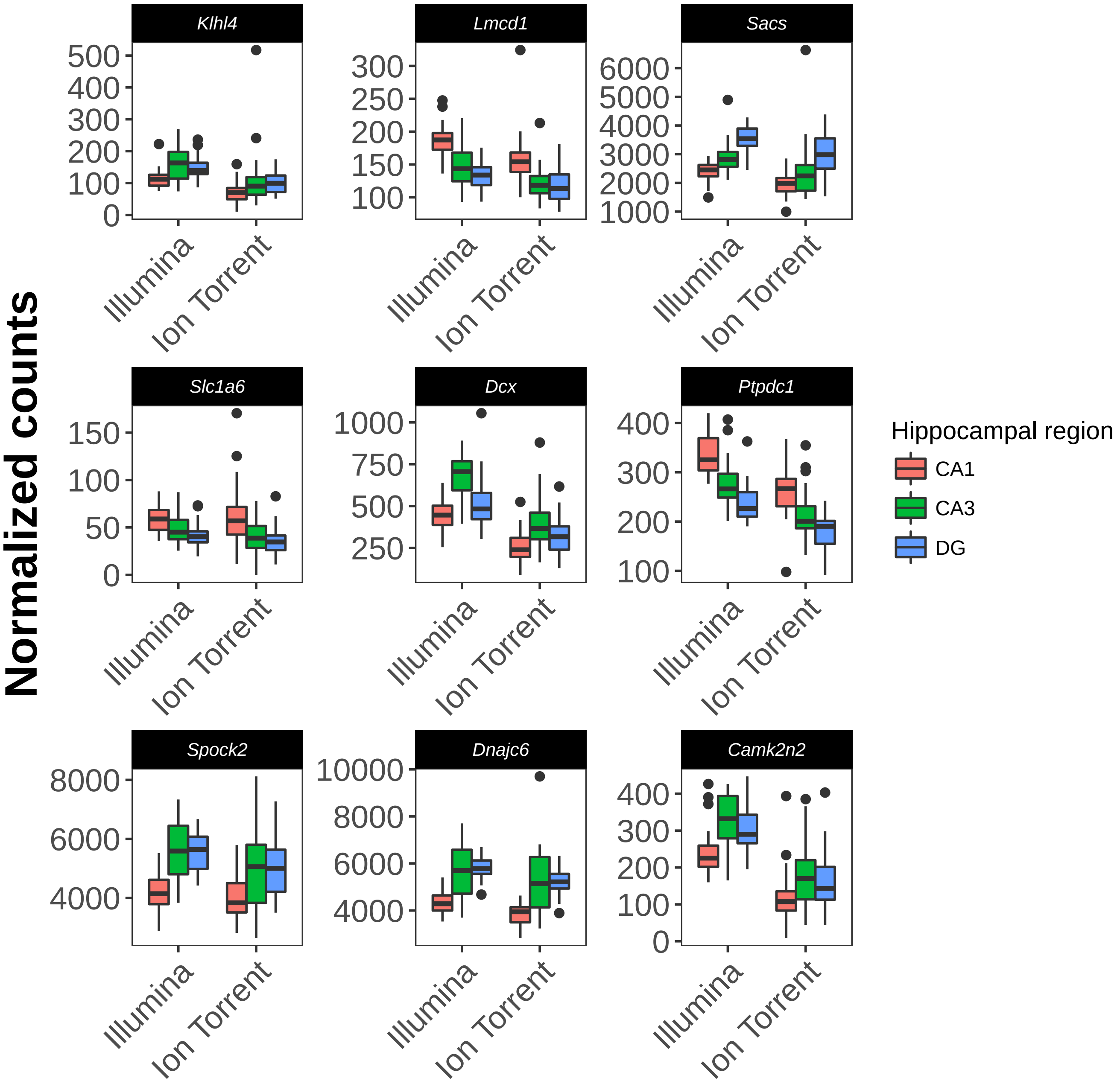

Normalized counts
Klhl4
Lmcd1
Sacs
Slc1a6
Dcx
Ptpdc1
Spock2
Dnajc6
Camk2n2
Illumina
Ion Torrent
Hippocampal region
CA1
CA3
DG

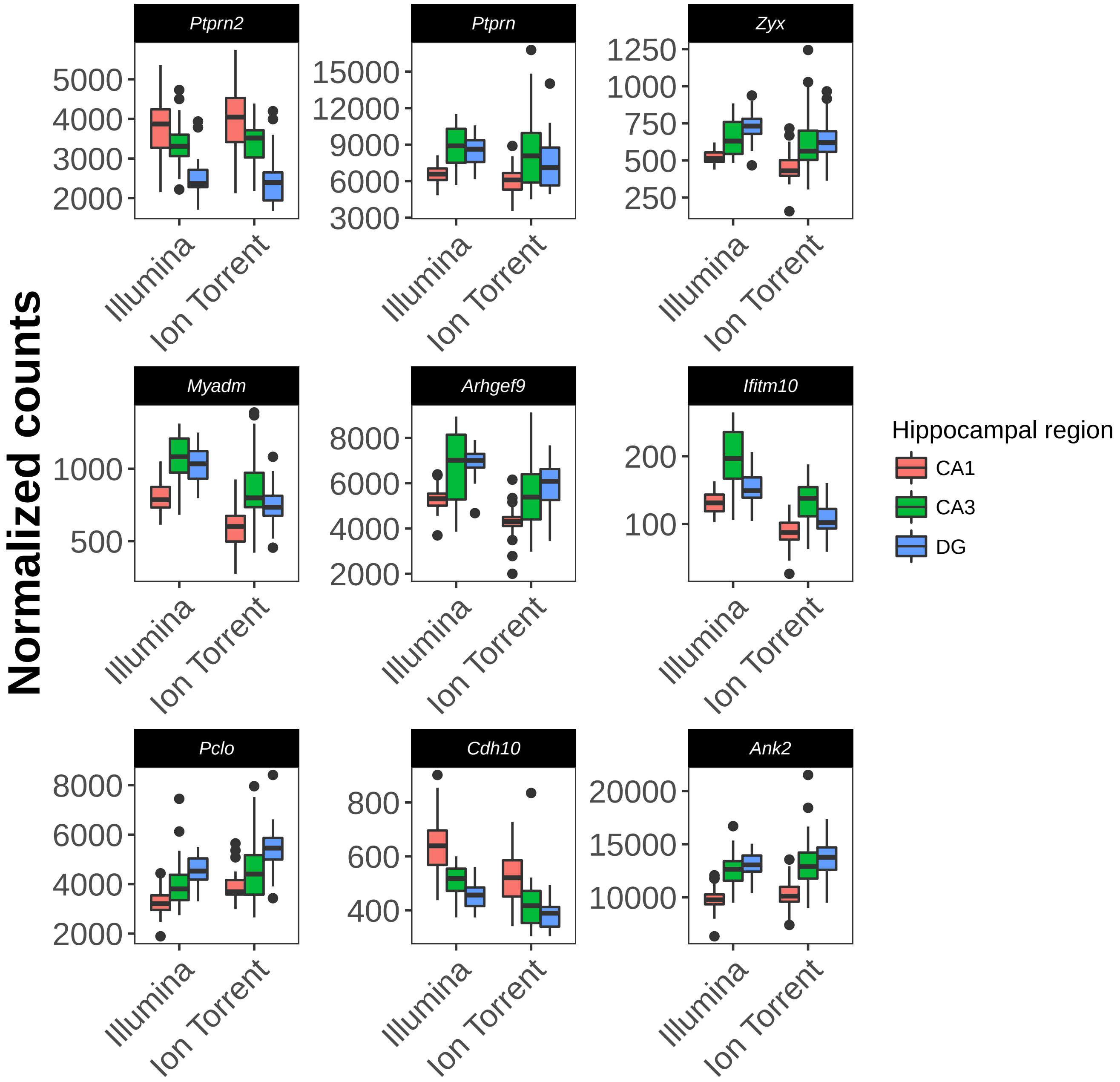
Normalized counts
Ptprn2
5000
4000
3000
2000
Ptprn
15000
12000
9000
6000
3000
Zyx
1250
1000
750
500
250
Myadm
1000
500
Arhgef9
8000
6000
4000
2000
Ifitm10
200
100
Pclo
8000
6000
4000
2000
Cdh10
800
600
400
Ank2
20000
15000
10000
Illumina
Ion Torrent
Hippocampal region
CA1
CA3
DG

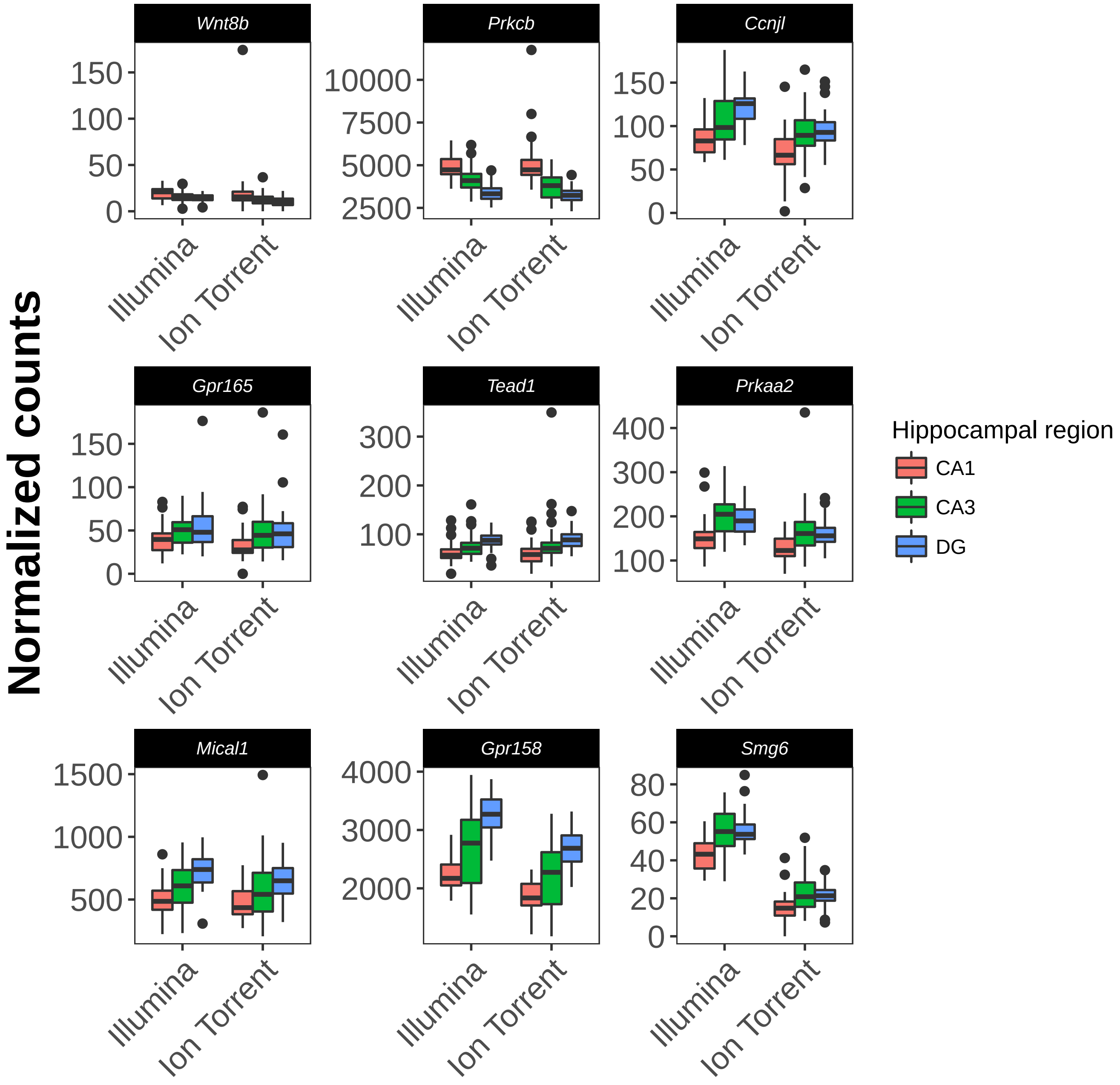

Normalized counts
Wnt8b
Prkcb
Ccnjl
Gpr165
Tead1
Prkaa2
Mical1
Gpr158
Smg6
Illumina
Ion Torrent
Hippocampal region
CA1
CA3
DG

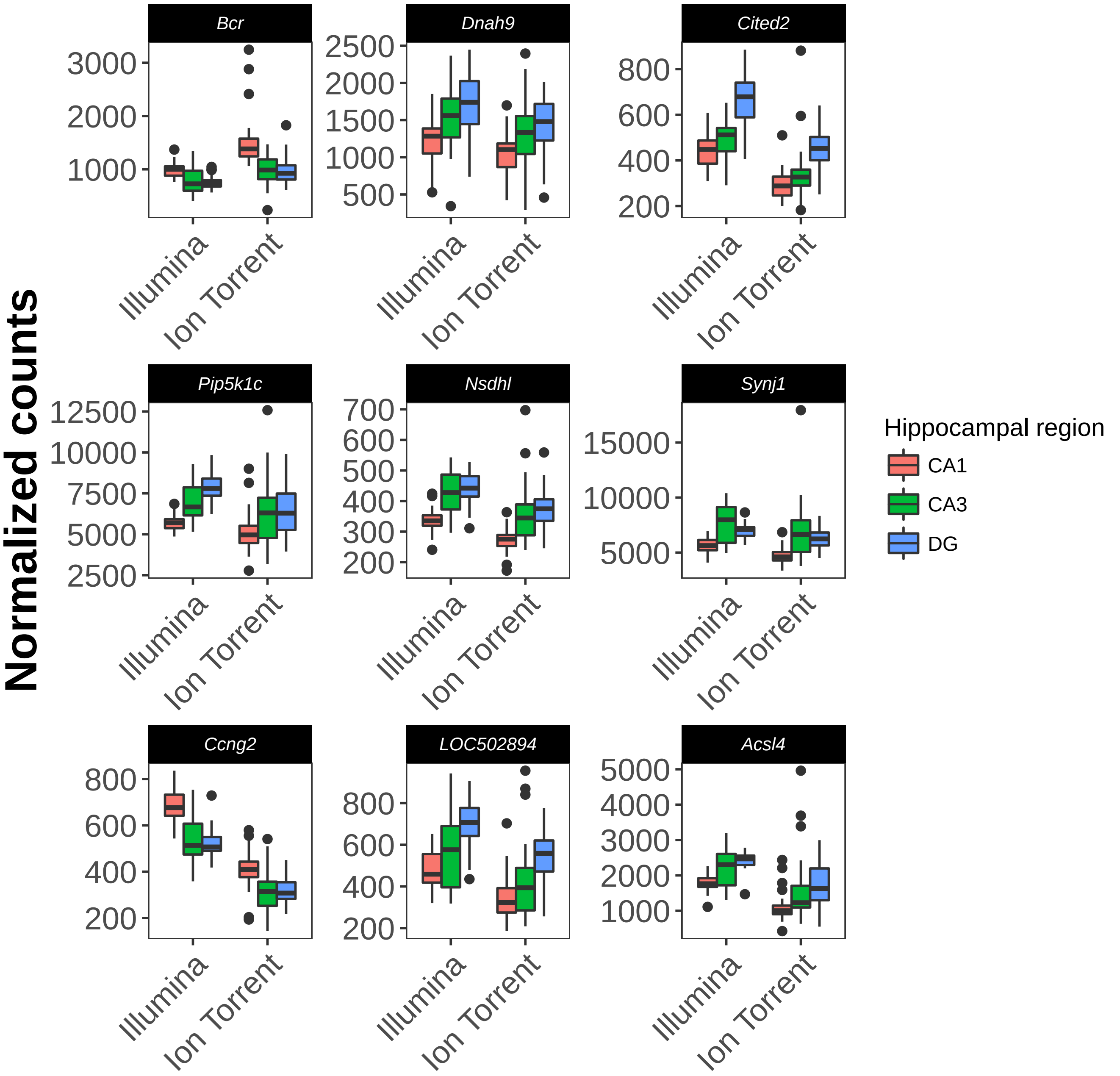

Normalized counts
Bcr
Dnah9
Cited2
Pip5k1c
Nsdhl
Synj1
Ccng2
LOC502894
Acsl4
Illumina
Ion Torrent
Hippocampal region
CA1
CA3
DG

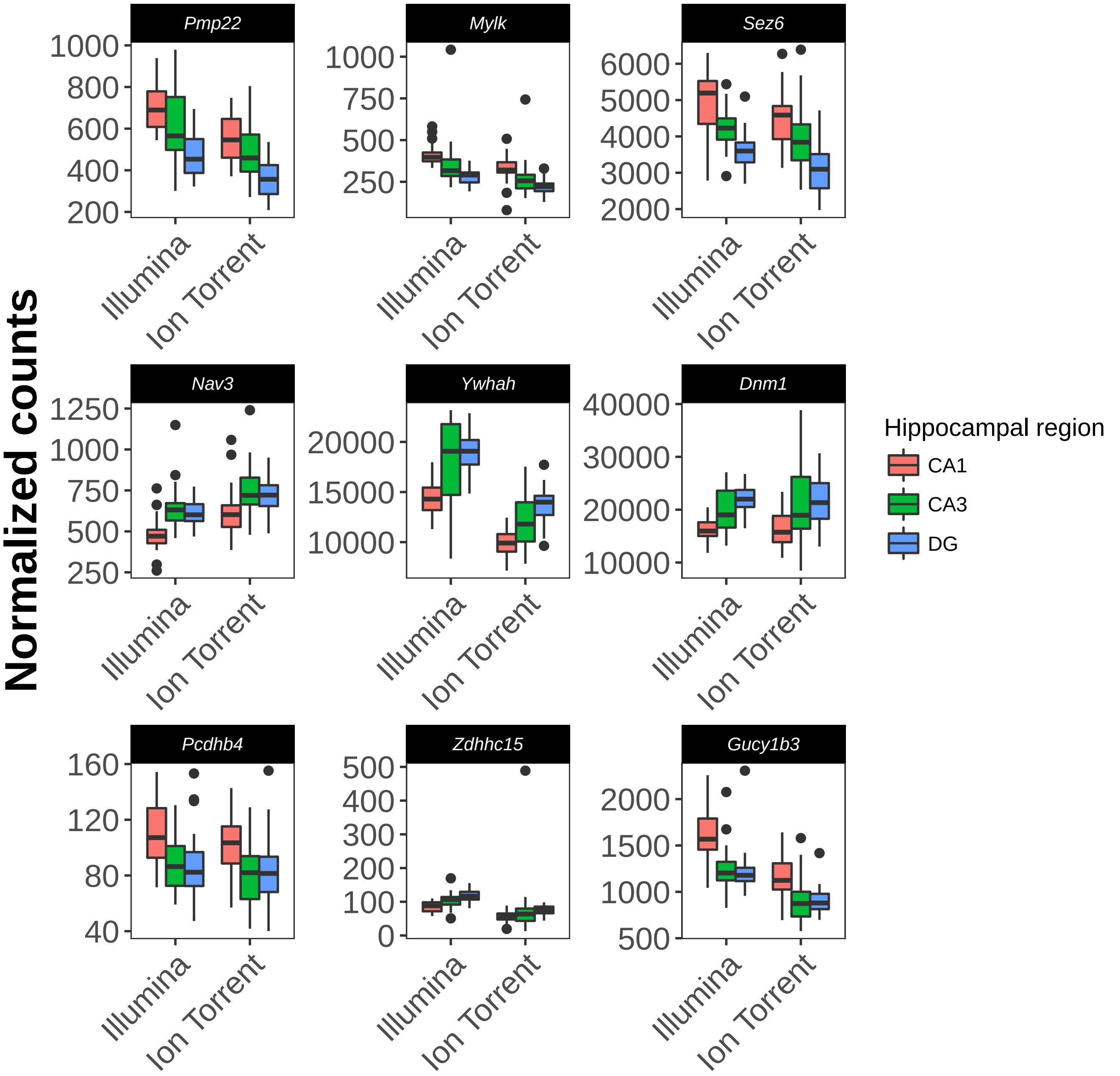
Normalized counts
Pmp22
Mylk
Sez6
Nav3
Ywhah
Dnm1
Pcdhb4
Zdhhc15
Gucy1b3
Illumina
Ion Torrent
Hippocampal region
CA1
CA3
DG

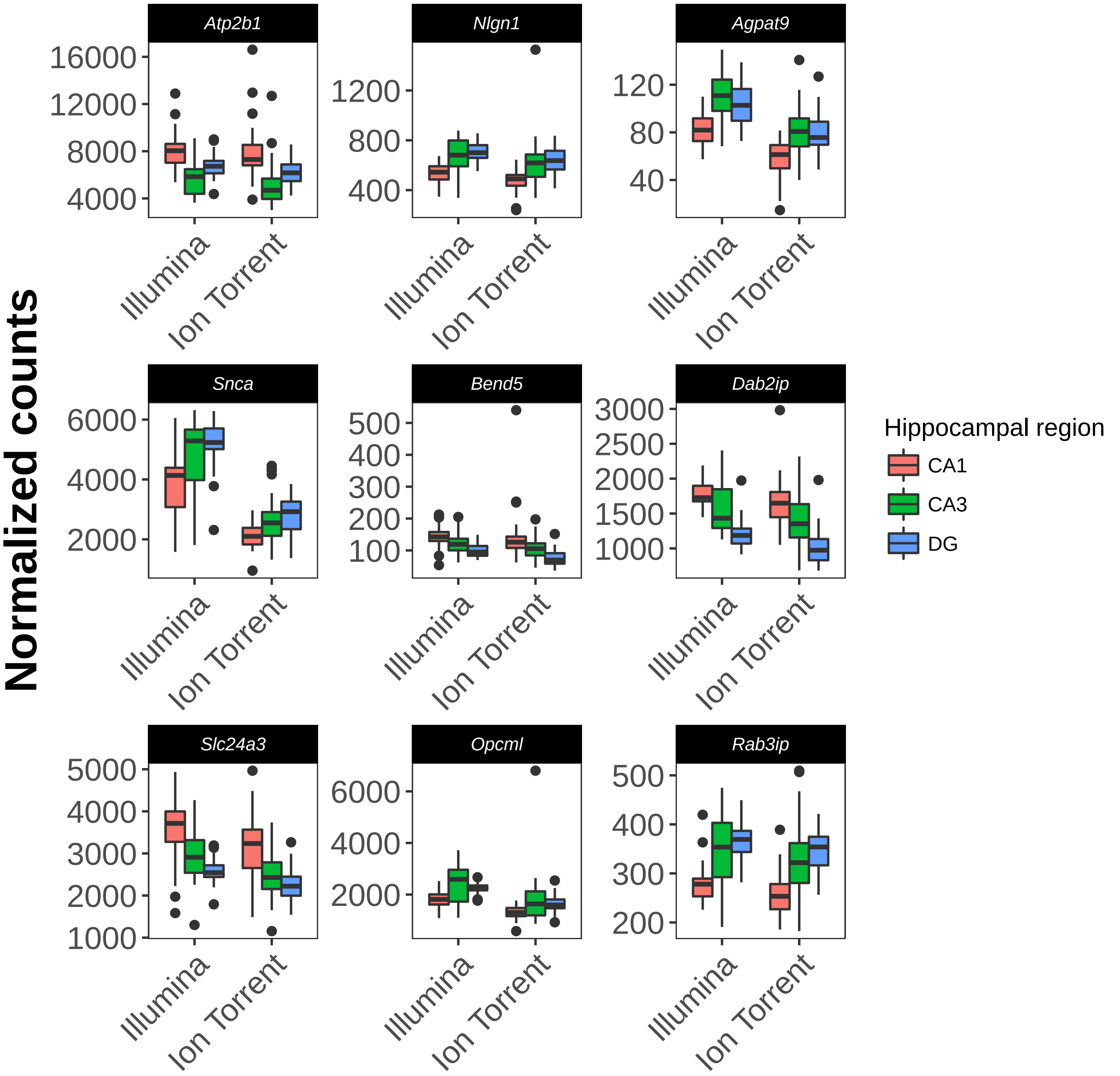

Normalized counts
Atp2b1
Nlgn1
Agpat9
Snca
Bend5
Dab2ip
Slc24a3
Opcml
Rab3ip
Illumina
Ion Torrent
Hippocampal region
CA1
CA3
DG

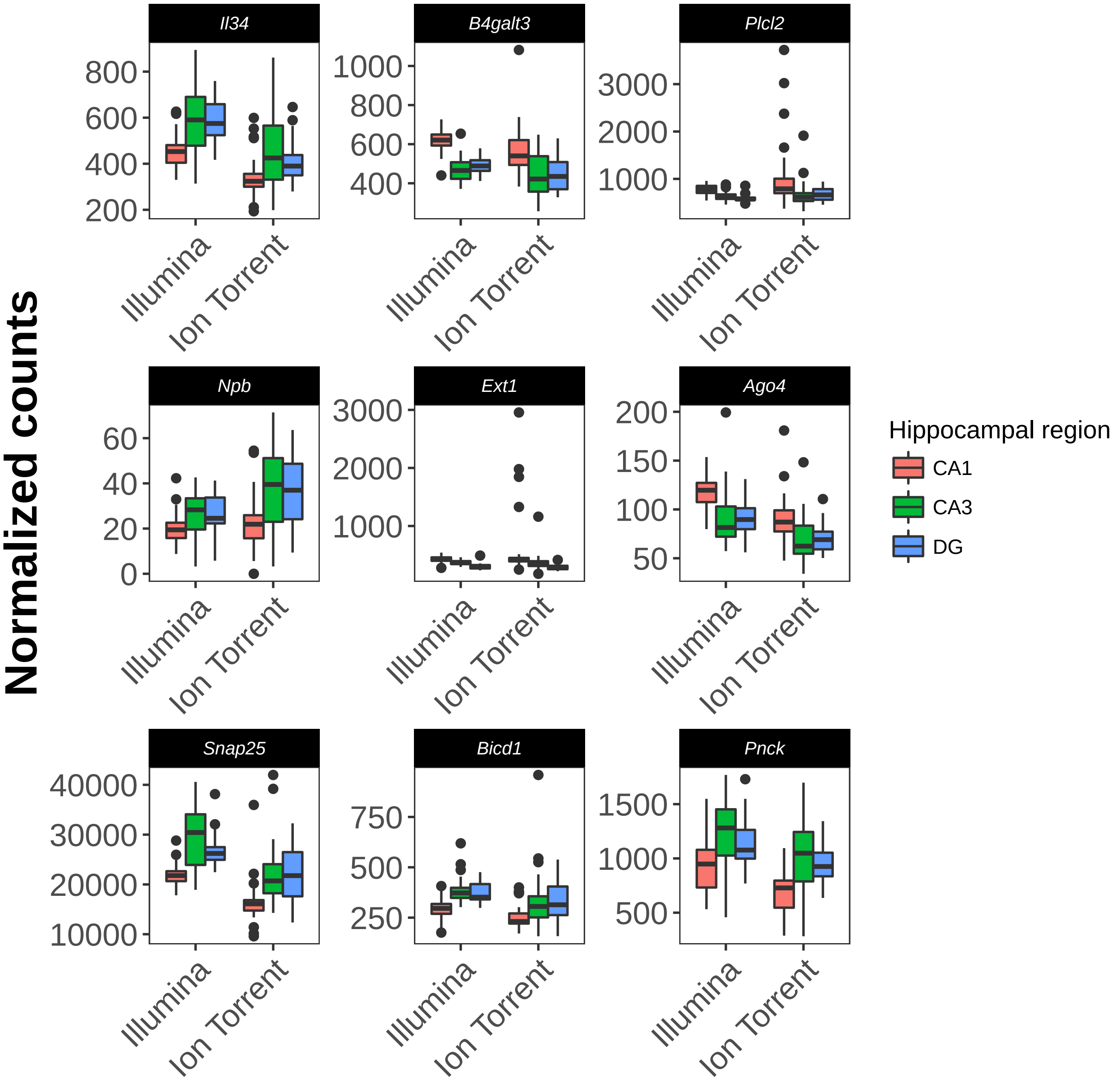
Normalized counts
Il34
B4galt3
Plcl2
Npb
Ext1
Ago4
Snap25
Bicd1
Pnck
Illumina
Ion Torrent
Hippocampal region
CA1
CA3
DG

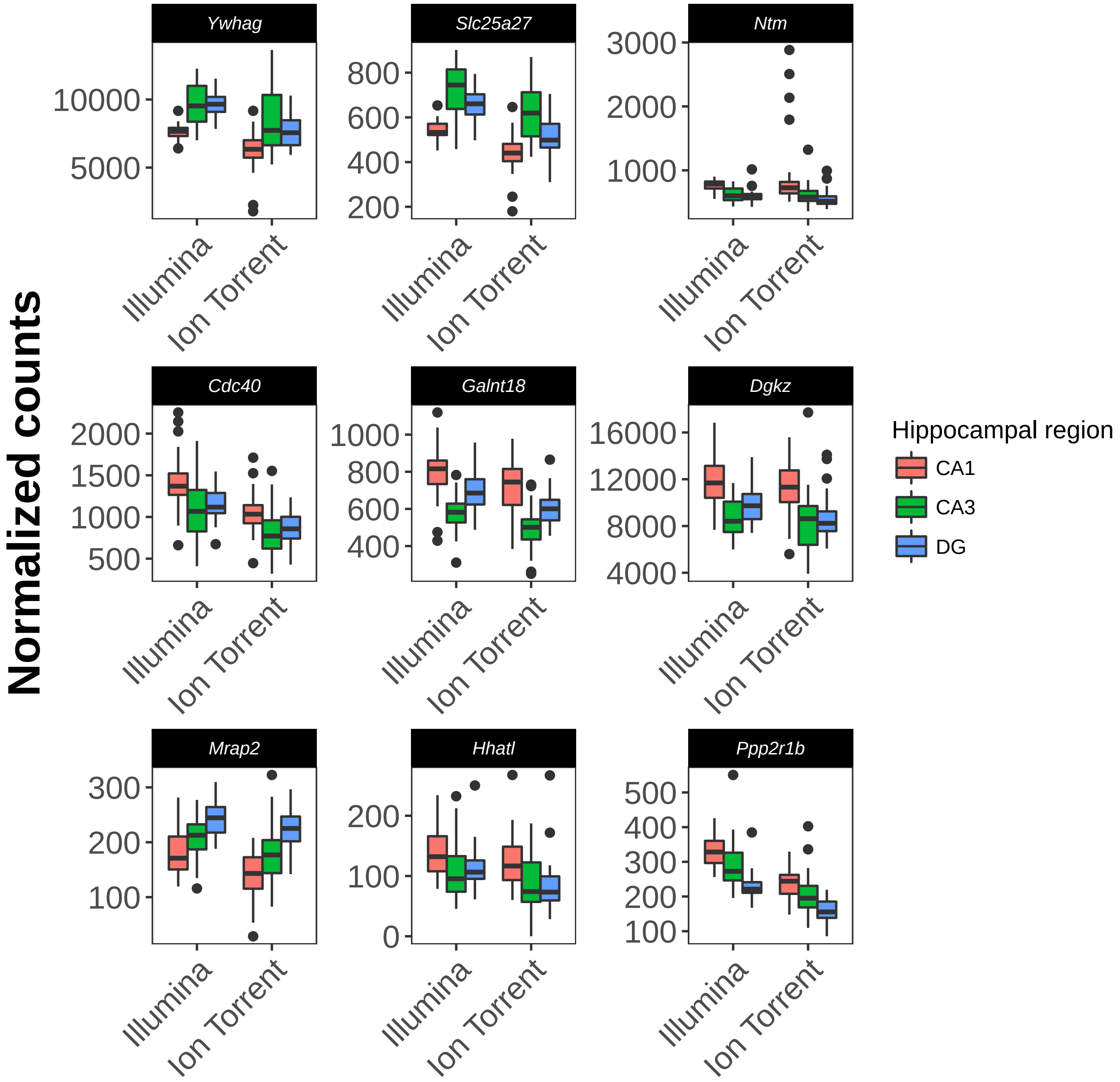
Normalized counts
Ywhag
Slc25a27
Ntm
Cdc40
Galnt18
Dgkz
Mrap2
Hhatl
Ppp2r1b
Illumina
Ion Torrent
Hippocampal region
CA1
CA3
DG

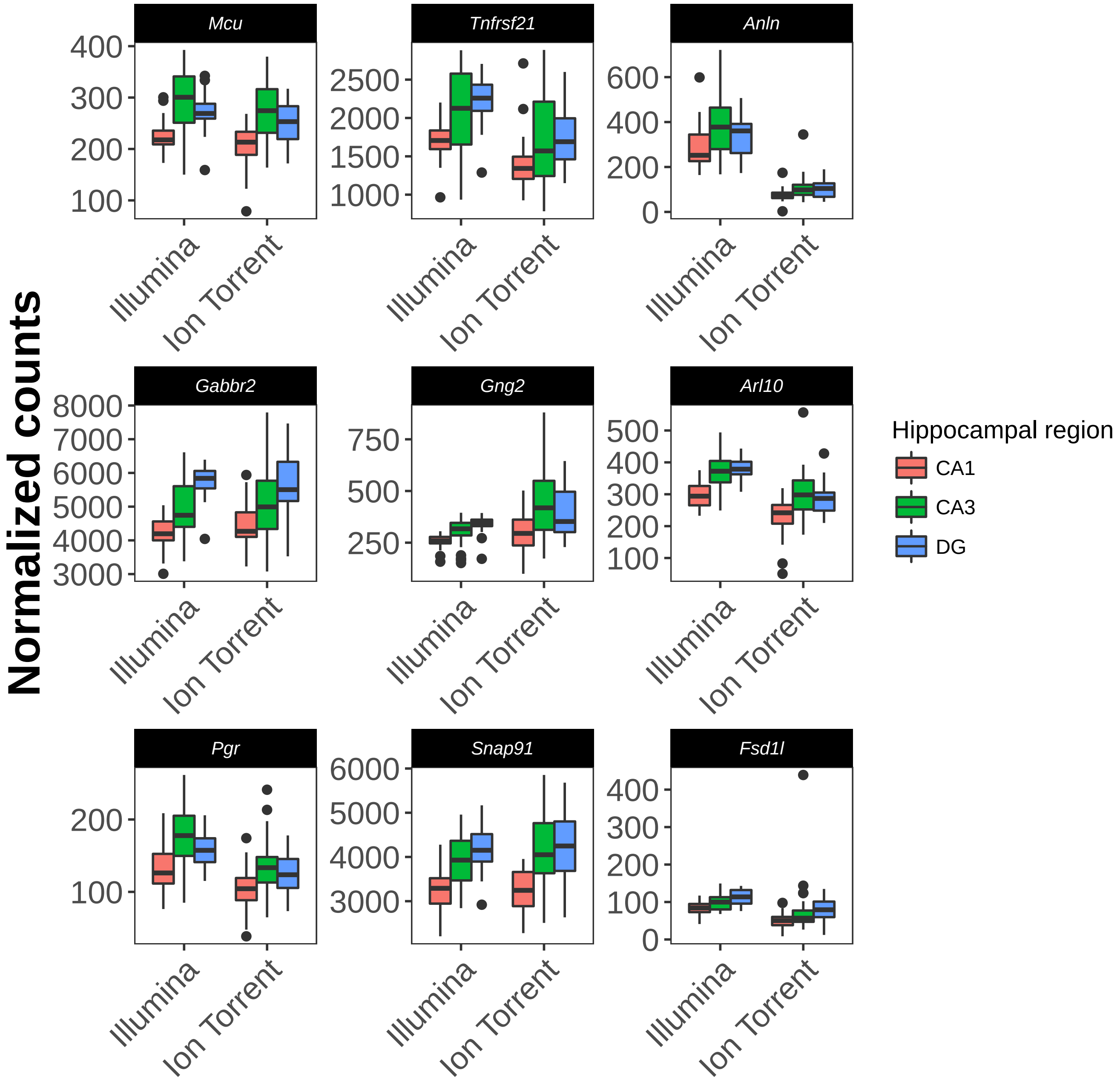

Normalized counts
Mcu
Tnfrsf21
Anln
Gabbr2
Gng2
Arl10
Pgr
Snap91
Fsd1l
Illumina
Ion Torrent
Hippocampal region
CA1
CA3
DG

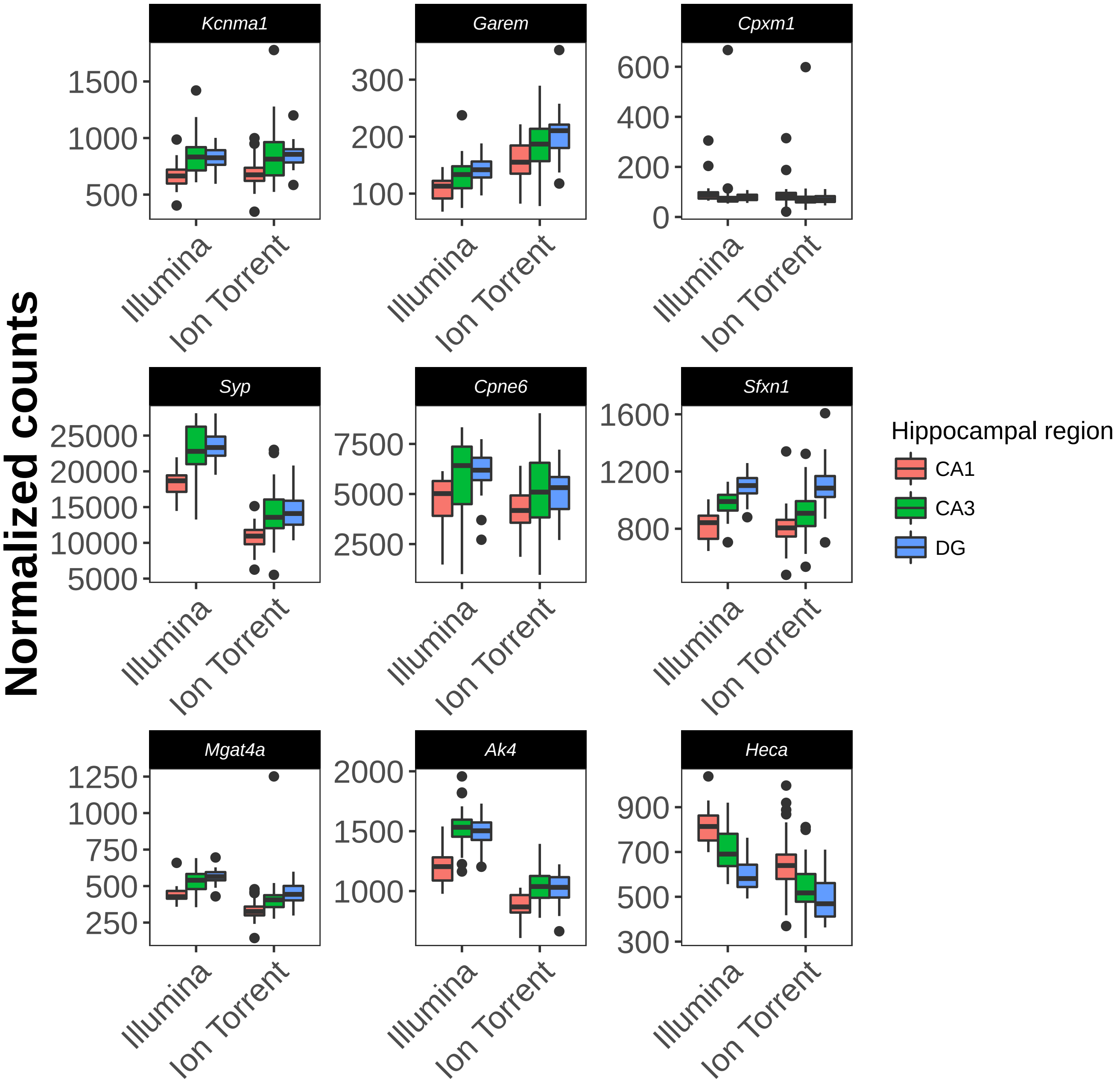
Normalized counts
Kcnma1
Garem
Cpxm1
Syp
Cpne6
Sfxn1
Mgat4a
Ak4
Heca
Illumina
Ion Torrent
Hippocampal region
CA1
CA3
DG

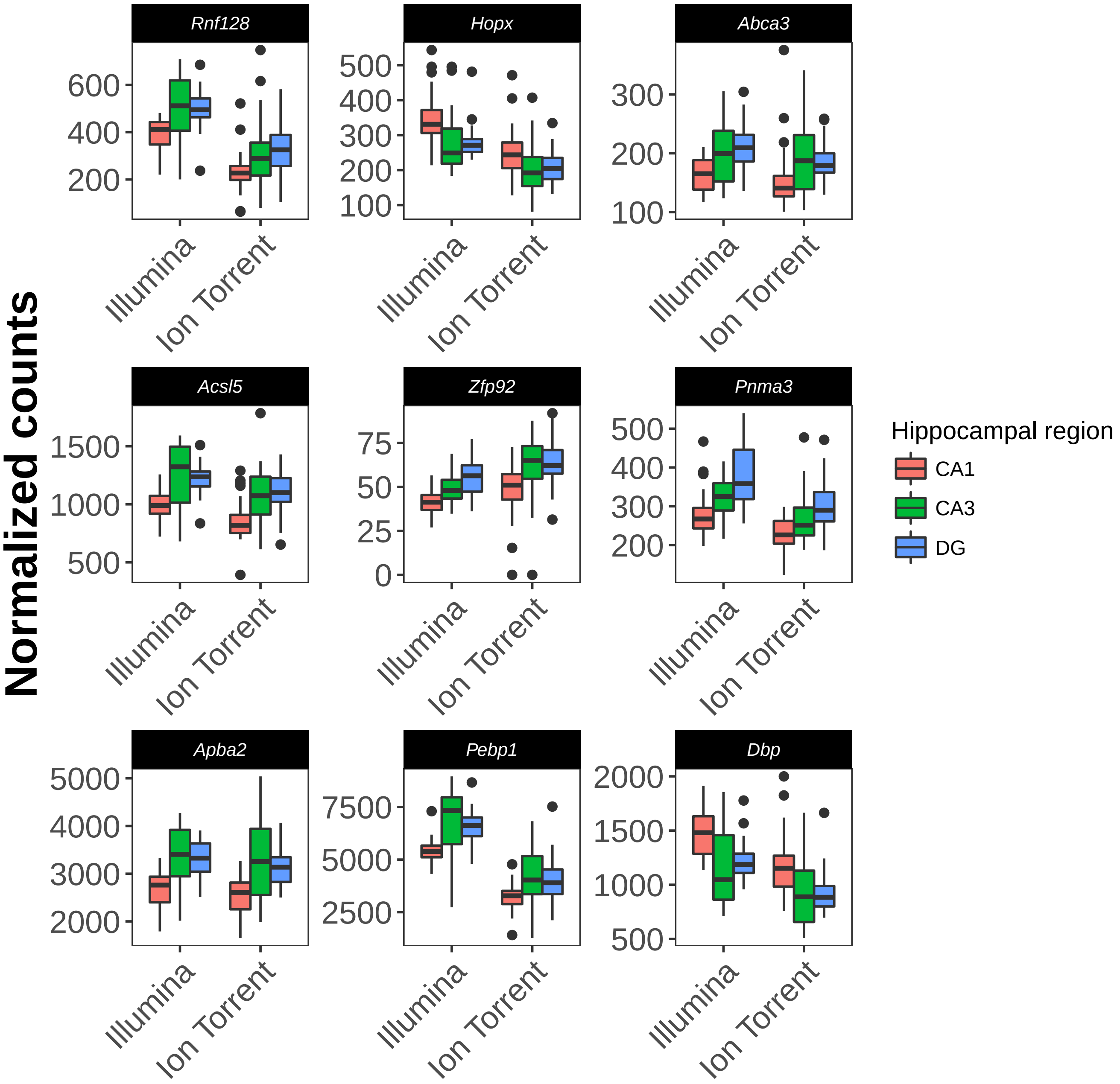
Normalized counts
Rnf128
Hopx
Abca3
Acsl5
Zfp92
Pnma3
Apba2
Pebp1
Dbp
Illumina
Ion Torrent
Hippocampal region
CA1
CA3
DG

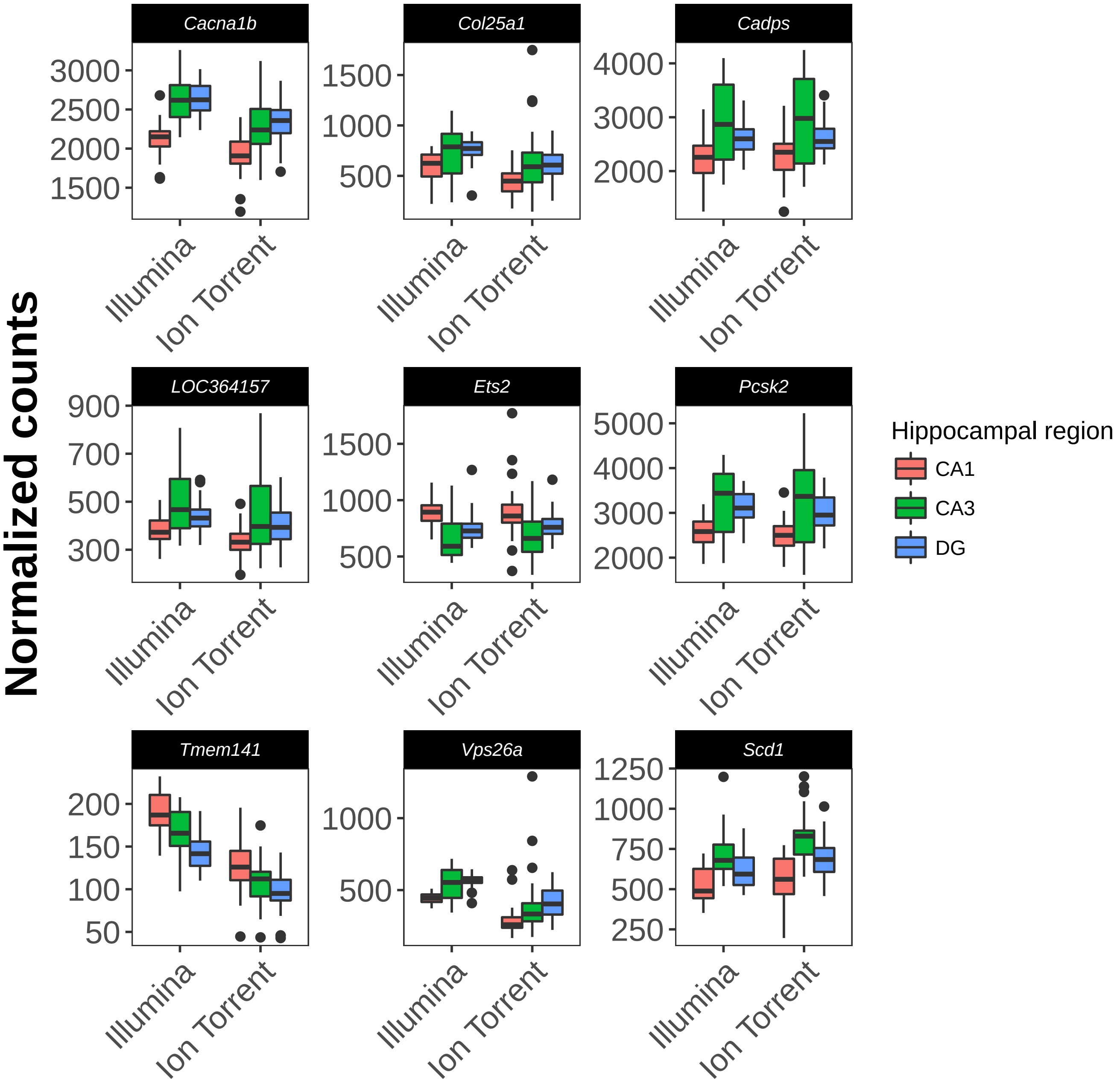
Normalized counts
Cacna1b
Col25a1
Cadps
LOC364157
Ets2
Pcsk2
Tmem141
Vps26a
Scd1
Illumina
Ion Torrent
Hippocampal region
CA1
CA3
DG

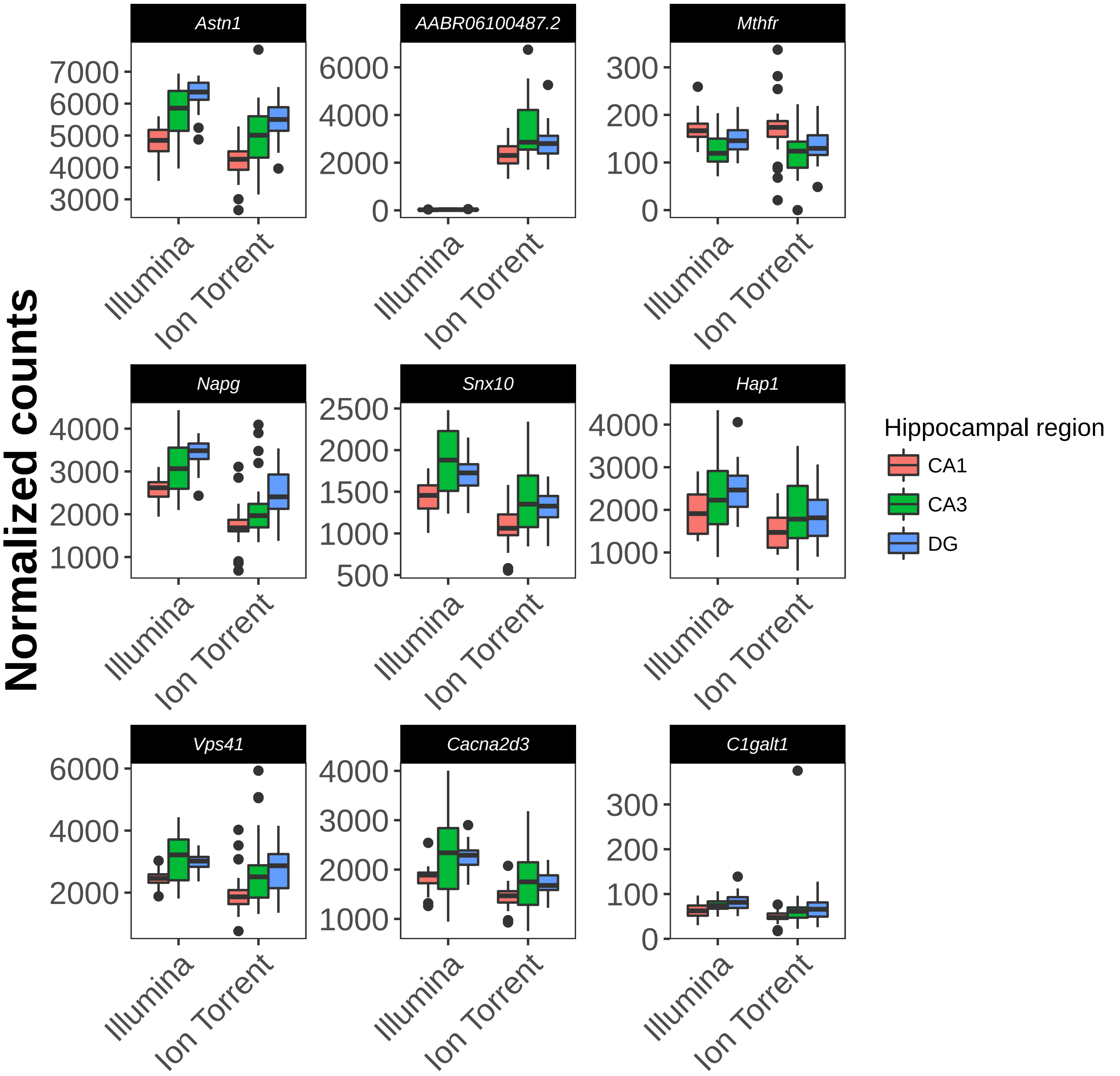
Normalized counts
Astn1
AABR06100487.2
Mthfr
Napg
Snx10
Hap1
Vps41
Cacna2d3
C1galt1
Illumina
Ion Torrent
Hippocampal region
CA1
CA3
DG

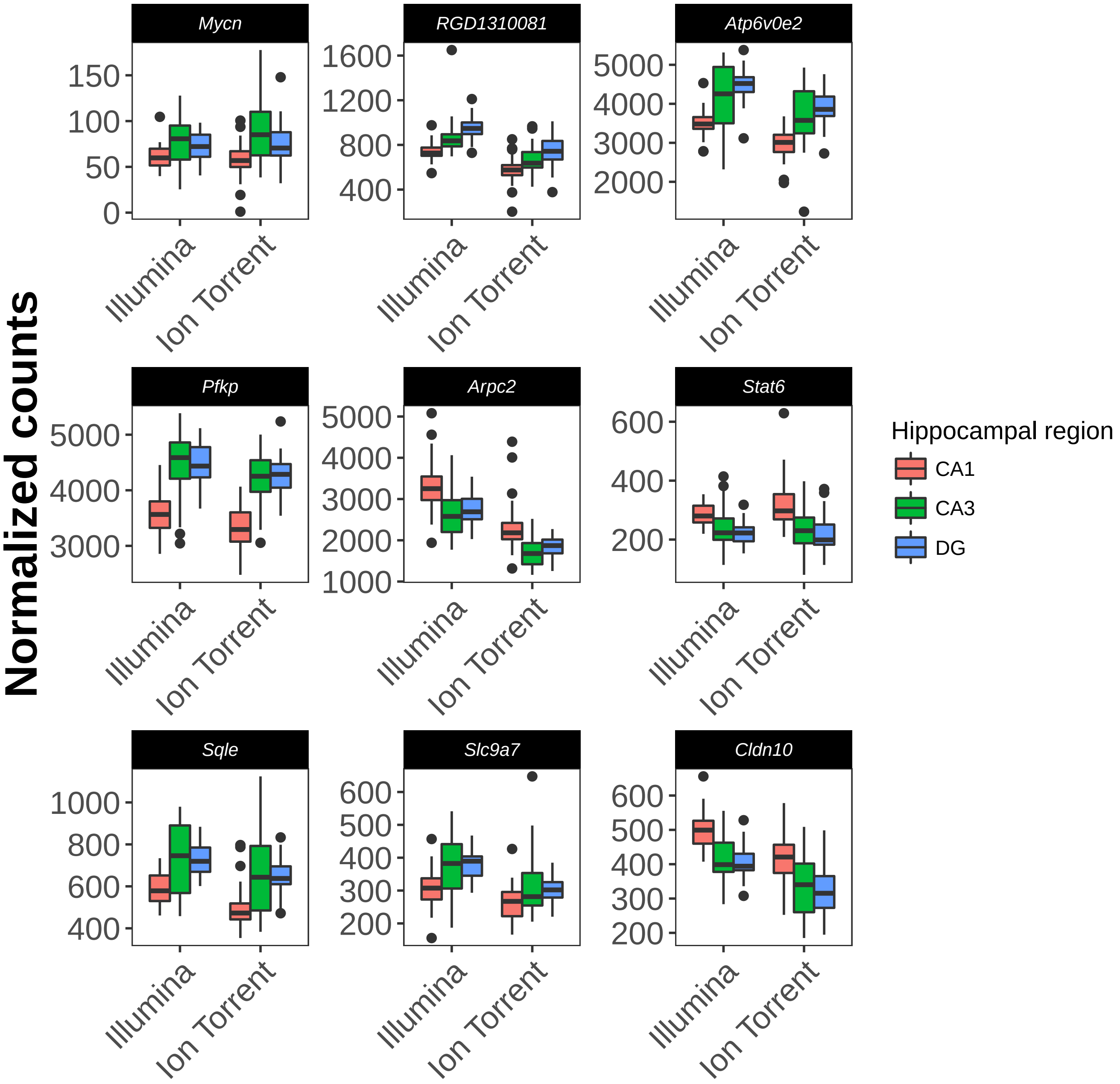

Normalized counts
Mycn
RGD1310081
Atp6v0e2
Pfkp
Arpc2
Stat6
Sqle
Slc9a7
Cldn10
Illumina
Ion Torrent
Hippocampal region
CA1
CA3
DG

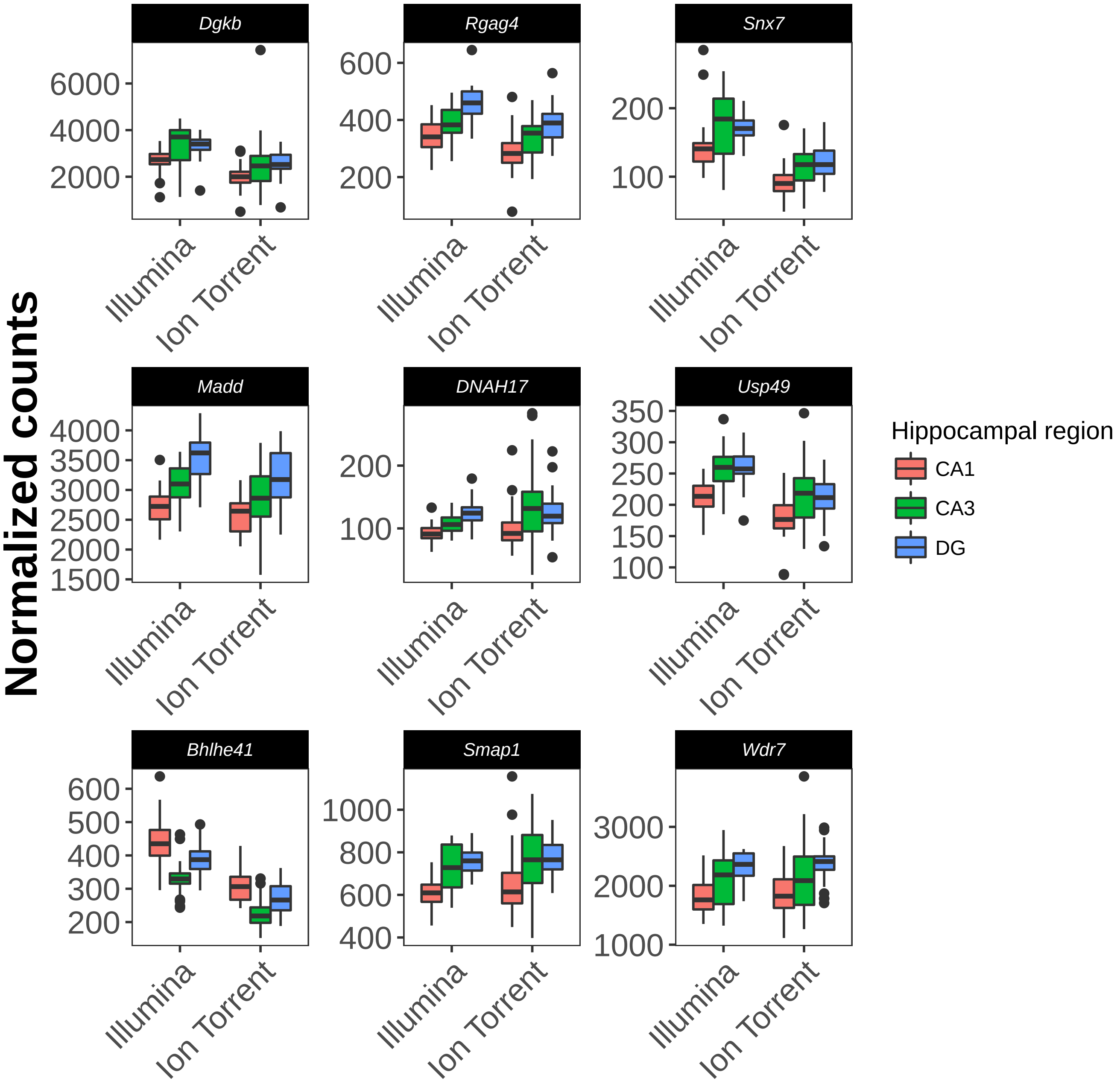

Normalized counts
Dgkb
Rgag4
Snx7
Madd
DNAH17
Usp49
Bhlhe41
Smap1
Wdr7
Illumina
Ion Torrent
Hippocampal region
CA1
CA3
DG

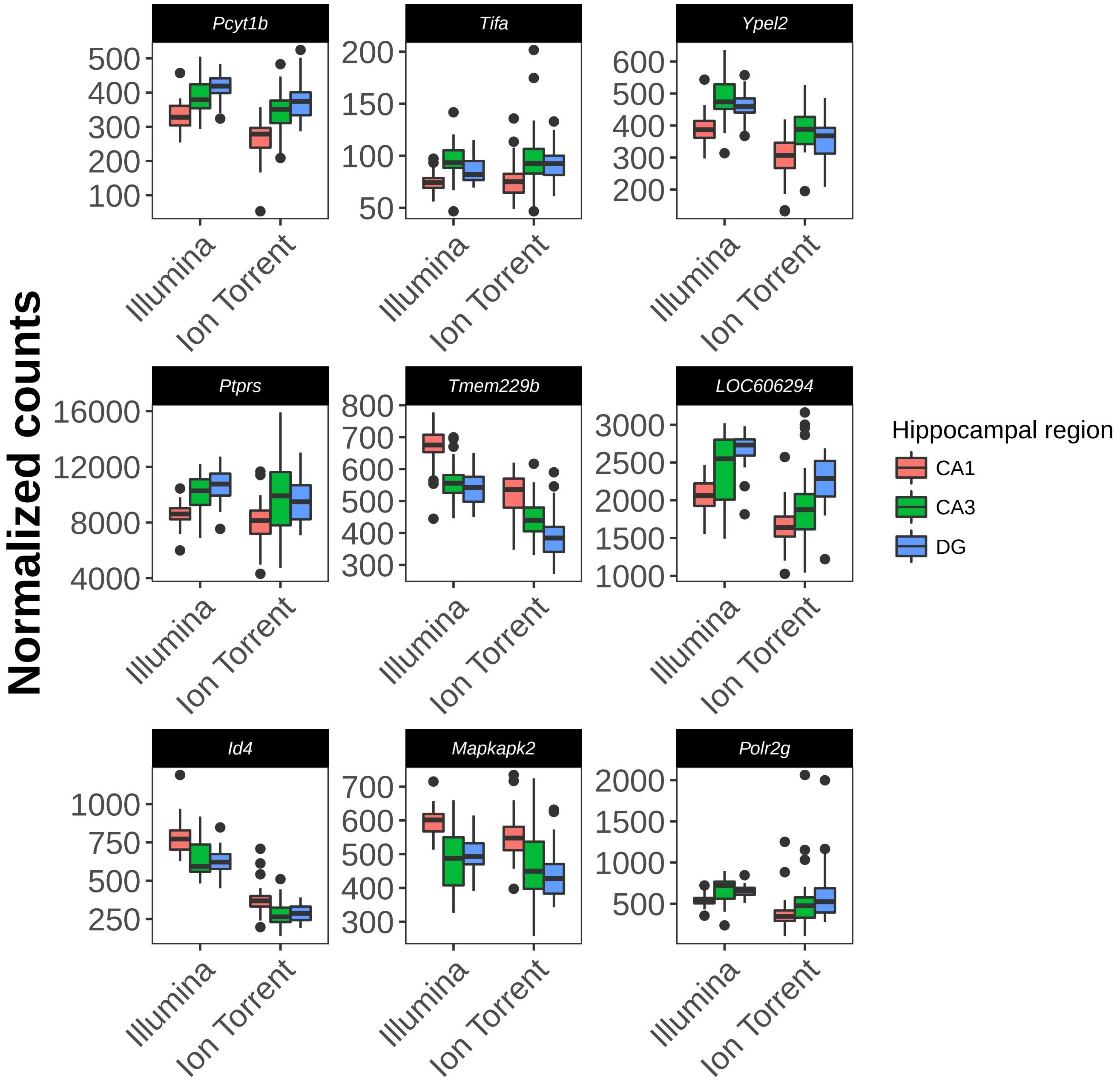
Normalized counts
Pcyt1b
Tifa
Ypel2
Ptprs
Tmem229b
LOC606294
Id4
Mapkapk2
Polr2g
Illumina
Ion Torrent
Hippocampal region
CA1
CA3
DG

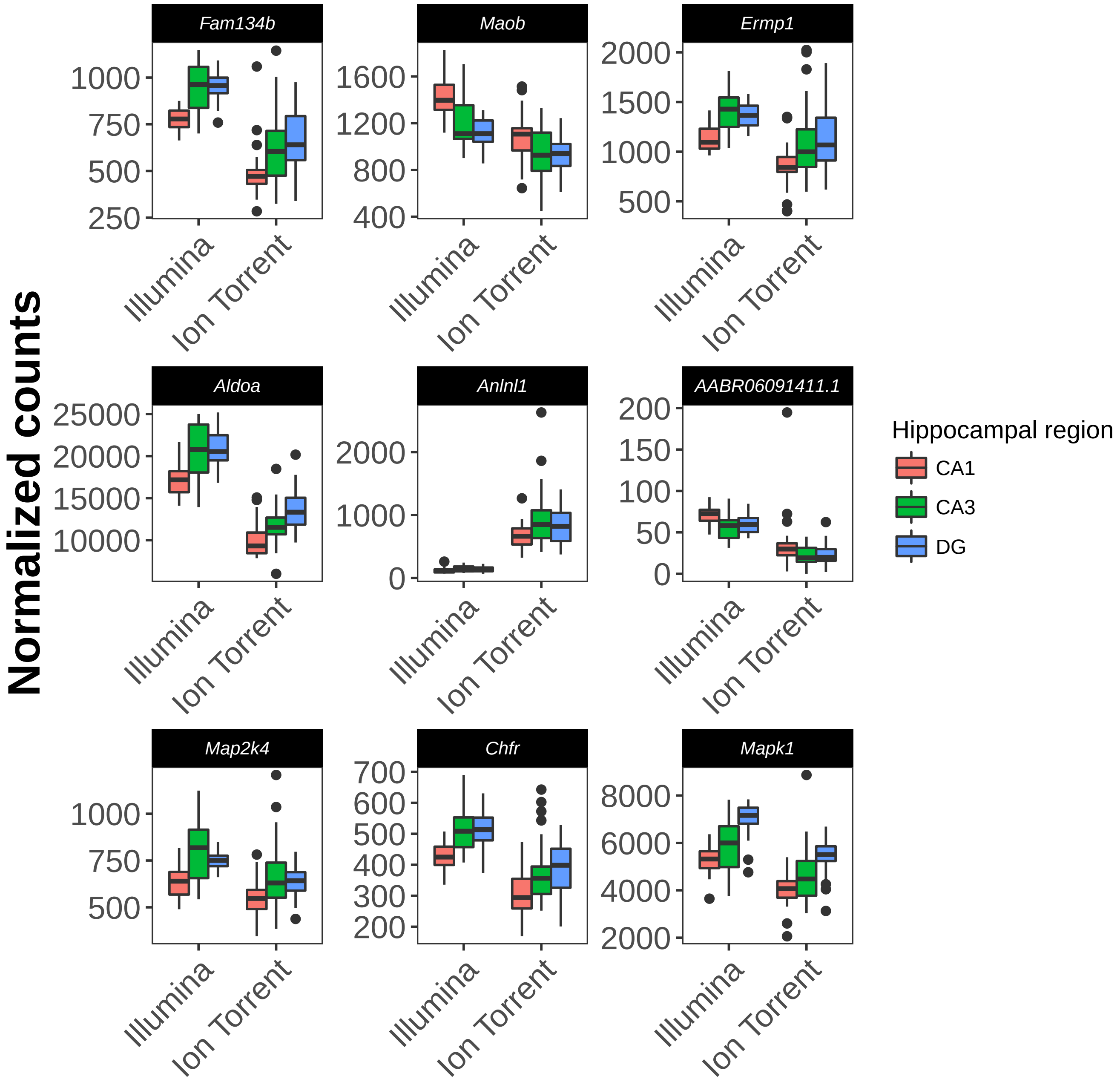

Normalized counts
Fam134b
Maob
Ermp1
Aldoa
Anlnl1
AABR06091411.1
Map2k4
Chfr
Mapk1
Illumina
Ion Torrent
Hippocampal region
CA1
CA3
DG

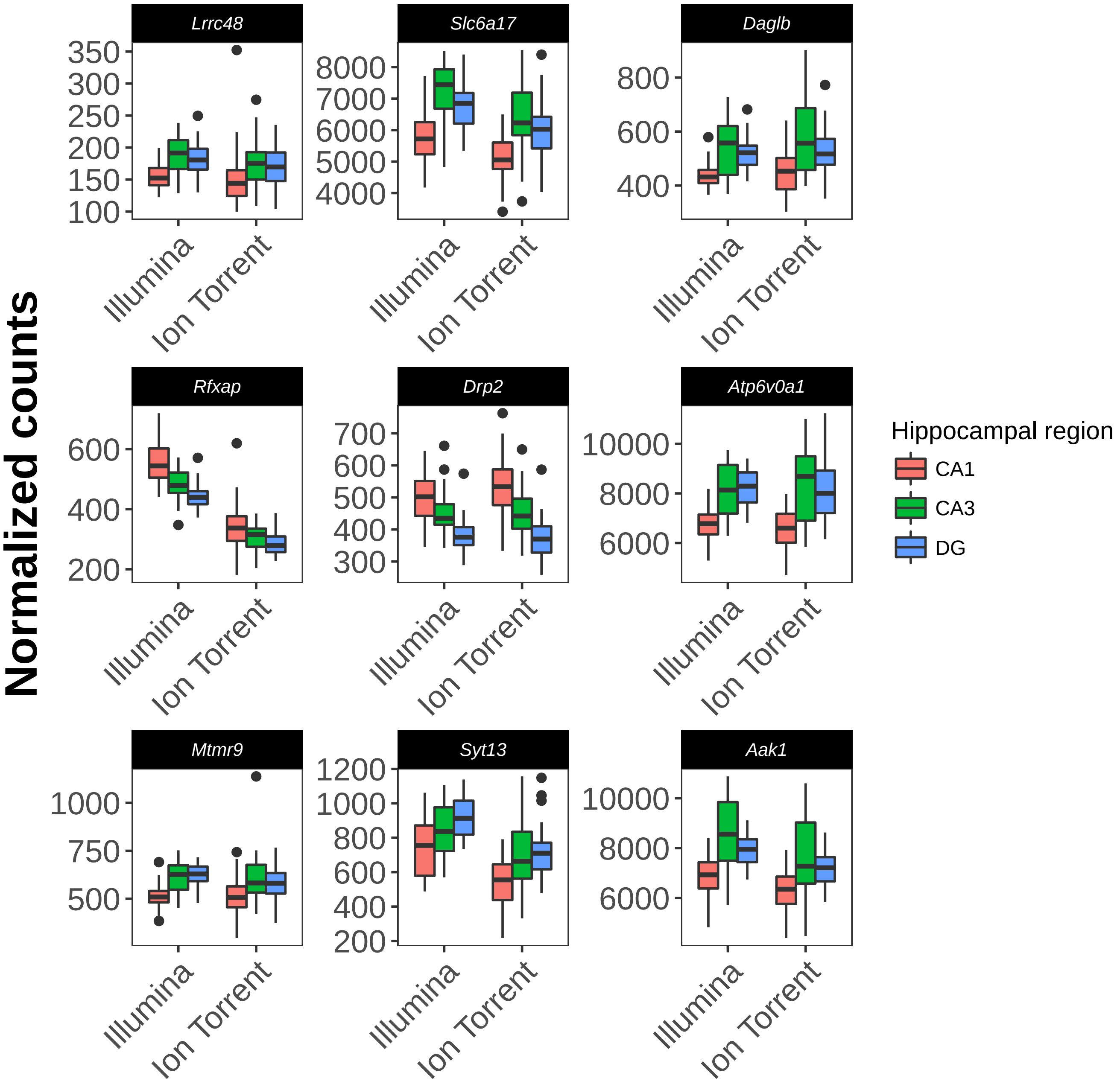
Normalized counts
Lrrc48
Slc6a17
Daglb
Rfxap
Drp2
Atp6v0a1
Mtmr9
Syt13
Aak1
Illumina
Ion Torrent
Hippocampal region
CA1
CA3
DG

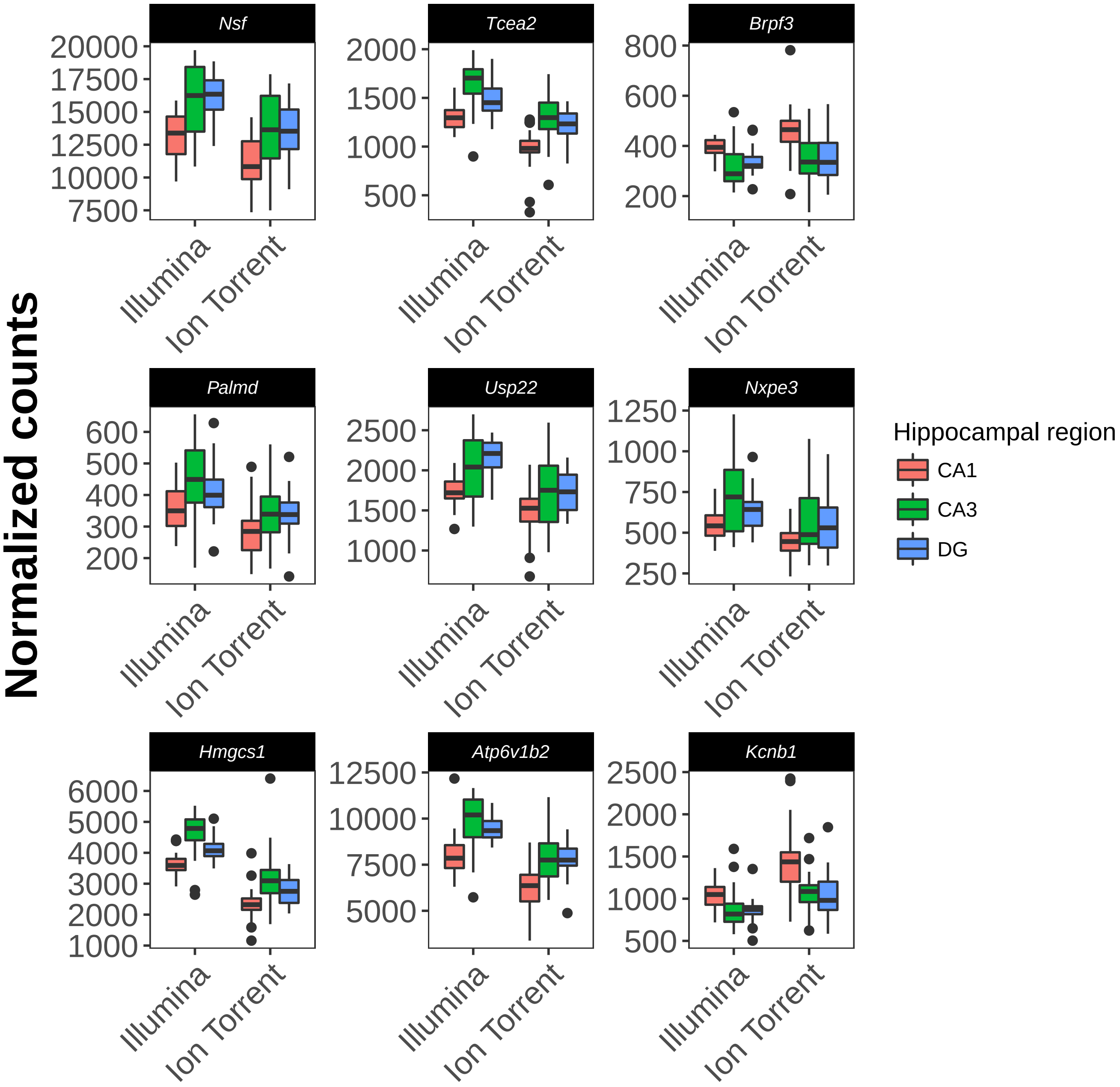
Normalized counts
Nsf
Tcea2
Brpf3
Palmd
Usp22
Nxpe3
Hmgcs1
Atp6v1b2
Kcnb1
Illumina
Ion Torrent
Hippocampal region
CA1
CA3
DG

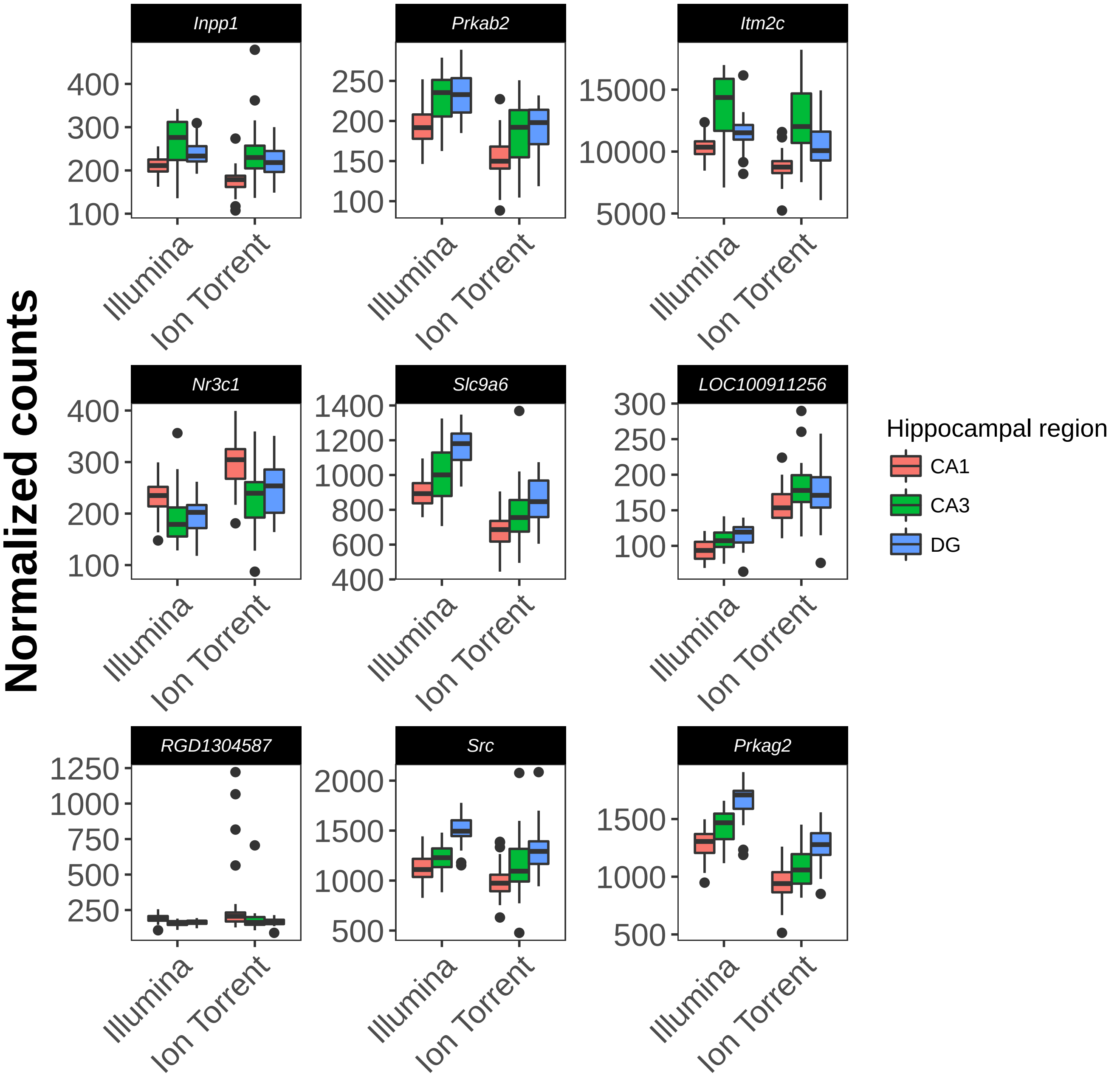
Normalized counts
Inpp1
Prkab2
Itm2c
Nr3c1
Slc9a6
LOC100911256
RGD1304587
Src
Prkag2
Illumina
Ion Torrent
Hippocampal region
CA1
CA3
DG

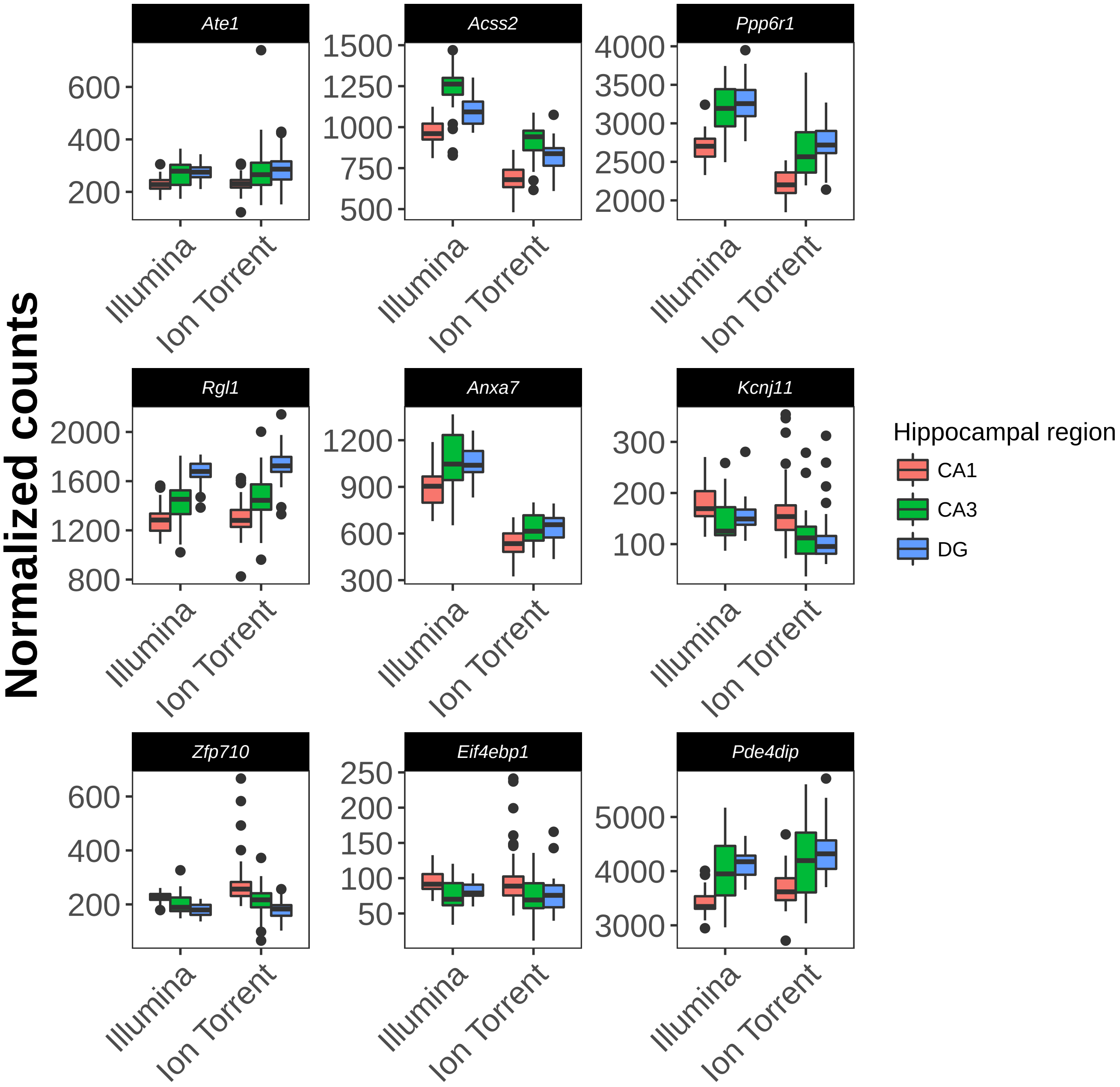
Normalized counts
Ate1
Acss2
Ppp6r1
Rgl1
Anxa7
Kcnj11
Zfp710
Eif4ebp1
Pde4dip
Illumina
Ion Torrent
Hippocampal region
CA1
CA3
DG

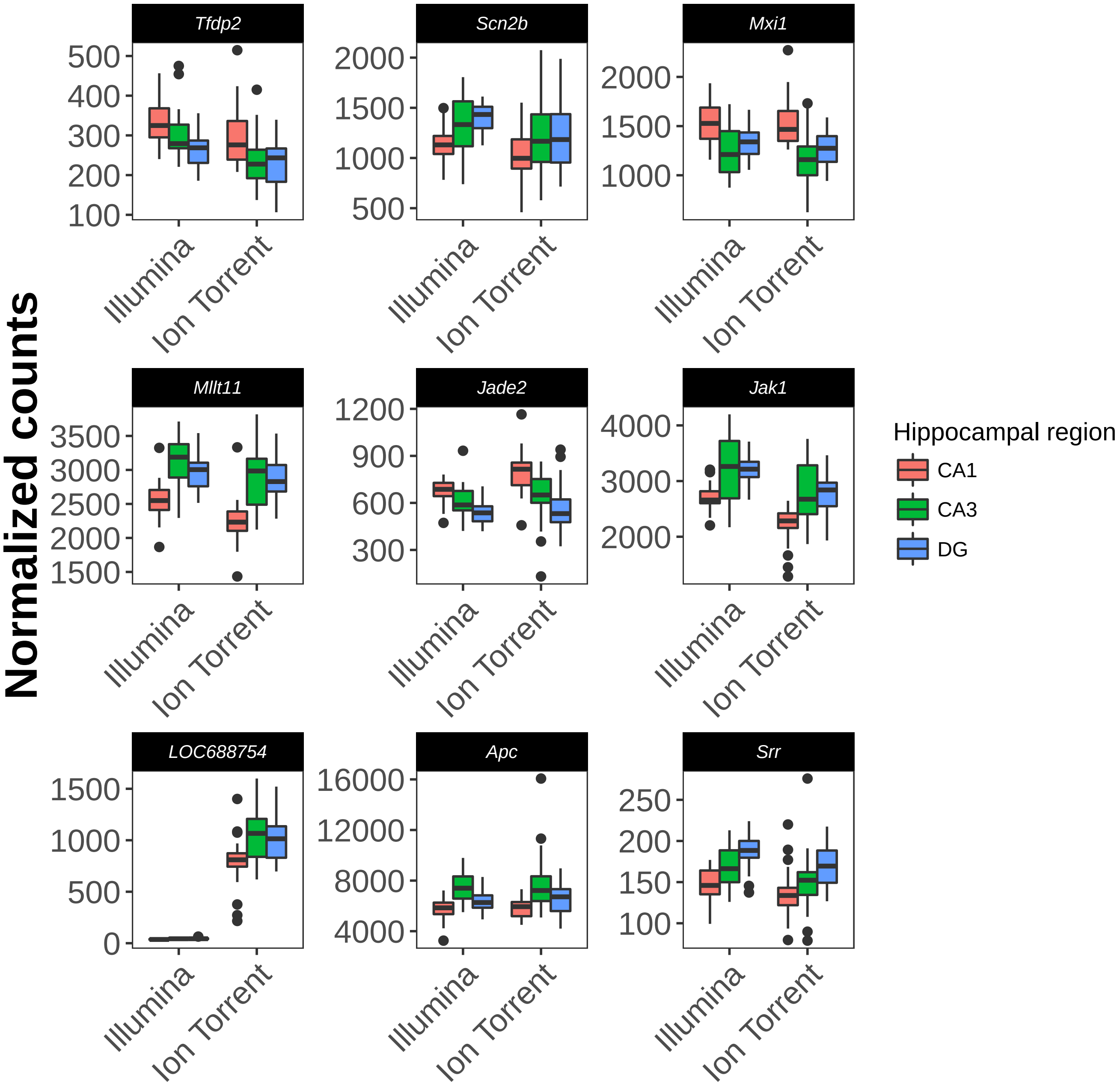
Normalized counts
Tfdp2
Scn2b
Mxi1
Mllt11
Jade2
Jak1
LOC688754
Apc
Srr
Illumina
Ion Torrent
Hippocampal region
CA1
CA3
DG

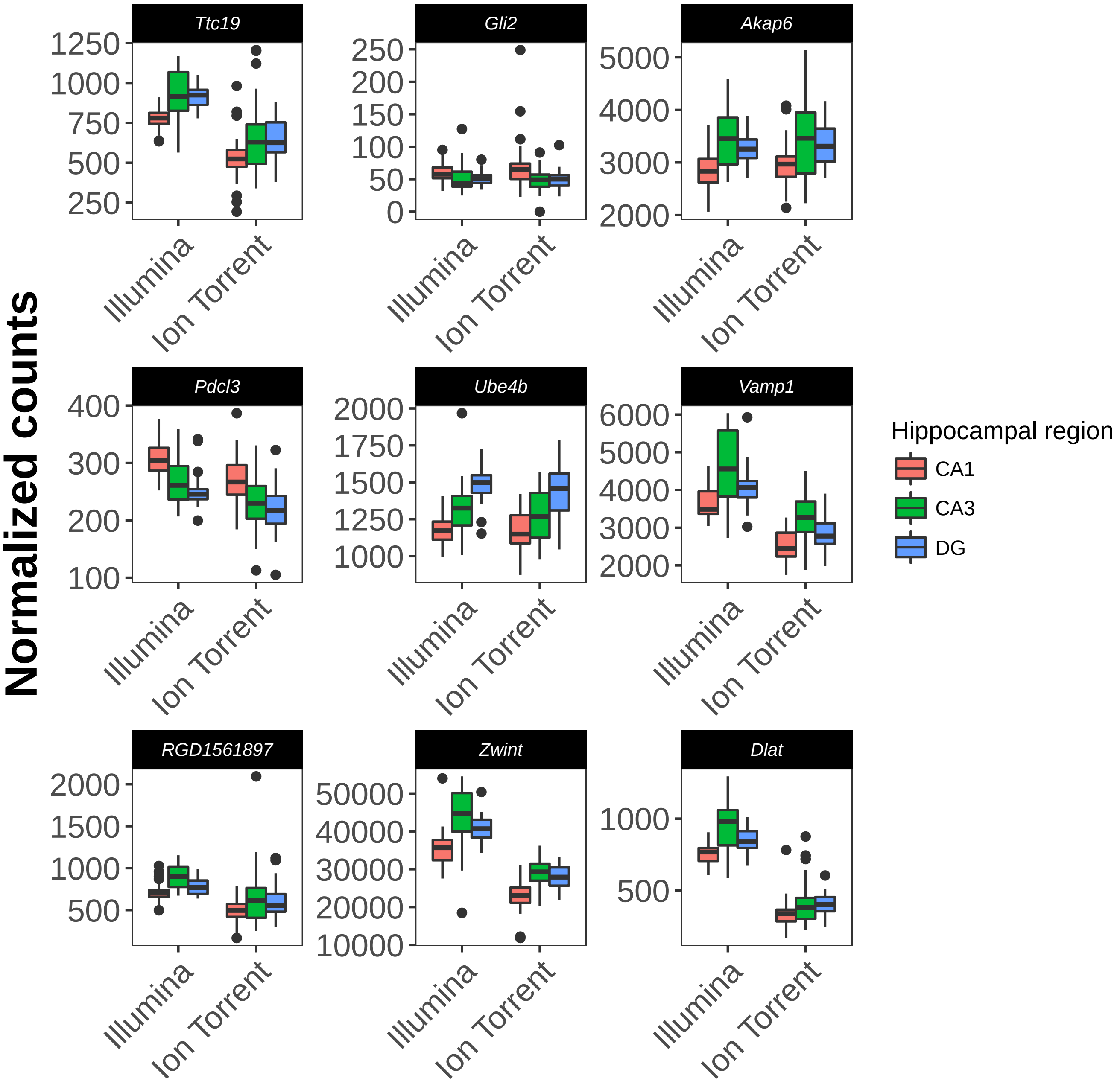
Normalized counts
Ttc19
Gli2
Akap6
Pdcl3
Ube4b
Vamp1
RGD1561897
Zwint
Dlat
Illumina
Ion Torrent
Hippocampal region
CA1
CA3
DG

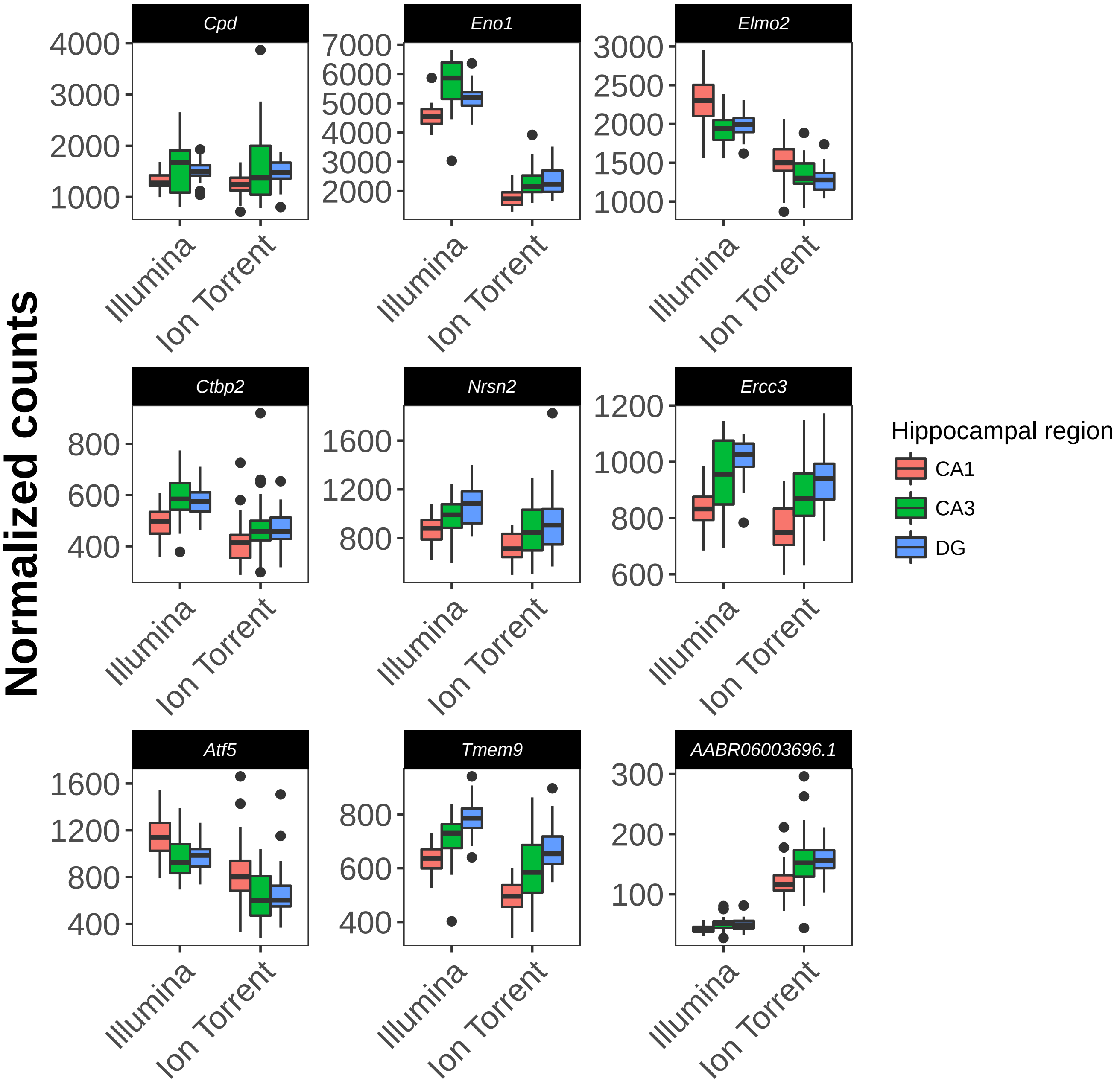
Normalized counts
Cpd
Eno1
Elmo2
Ctbp2
Nrsn2
Ercc3
Atf5
Tmem9
AABR06003696.1
Illumina
Ion Torrent
Hippocampal region
CA1
CA3
DG

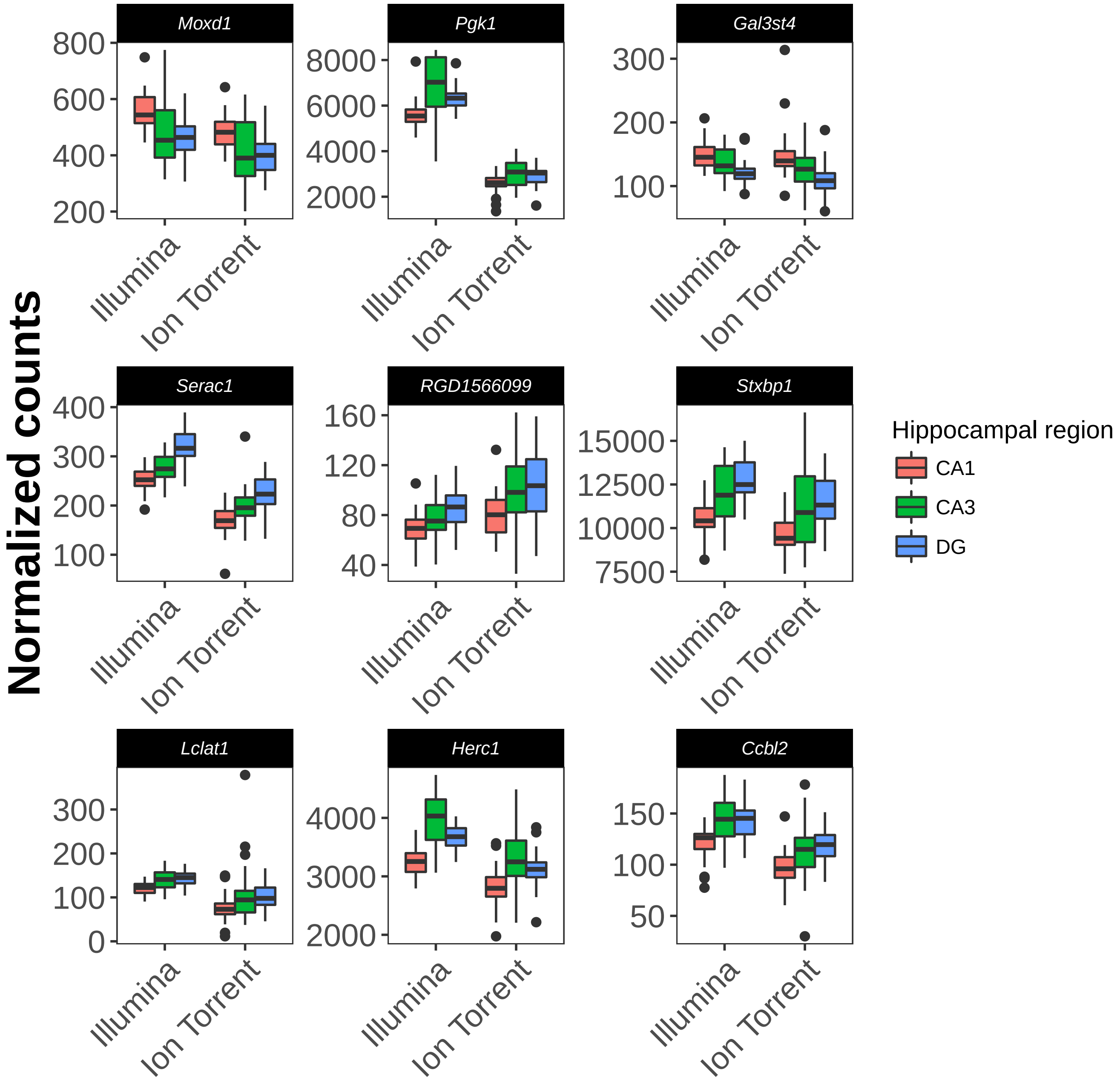
Normalized counts
Moxd1
Pgk1
Gal3st4
Serac1
RGD1566099
Stxbp1
Lclat1
Herc1
Ccbl2
Illumina
Ion Torrent
Hippocampal region
CA1
CA3
DG
200
400
600
800
2000
4000
6000
8000
100
200
300
400
40
80
120
160
7500
10000
12500
15000
0
3000
50
150

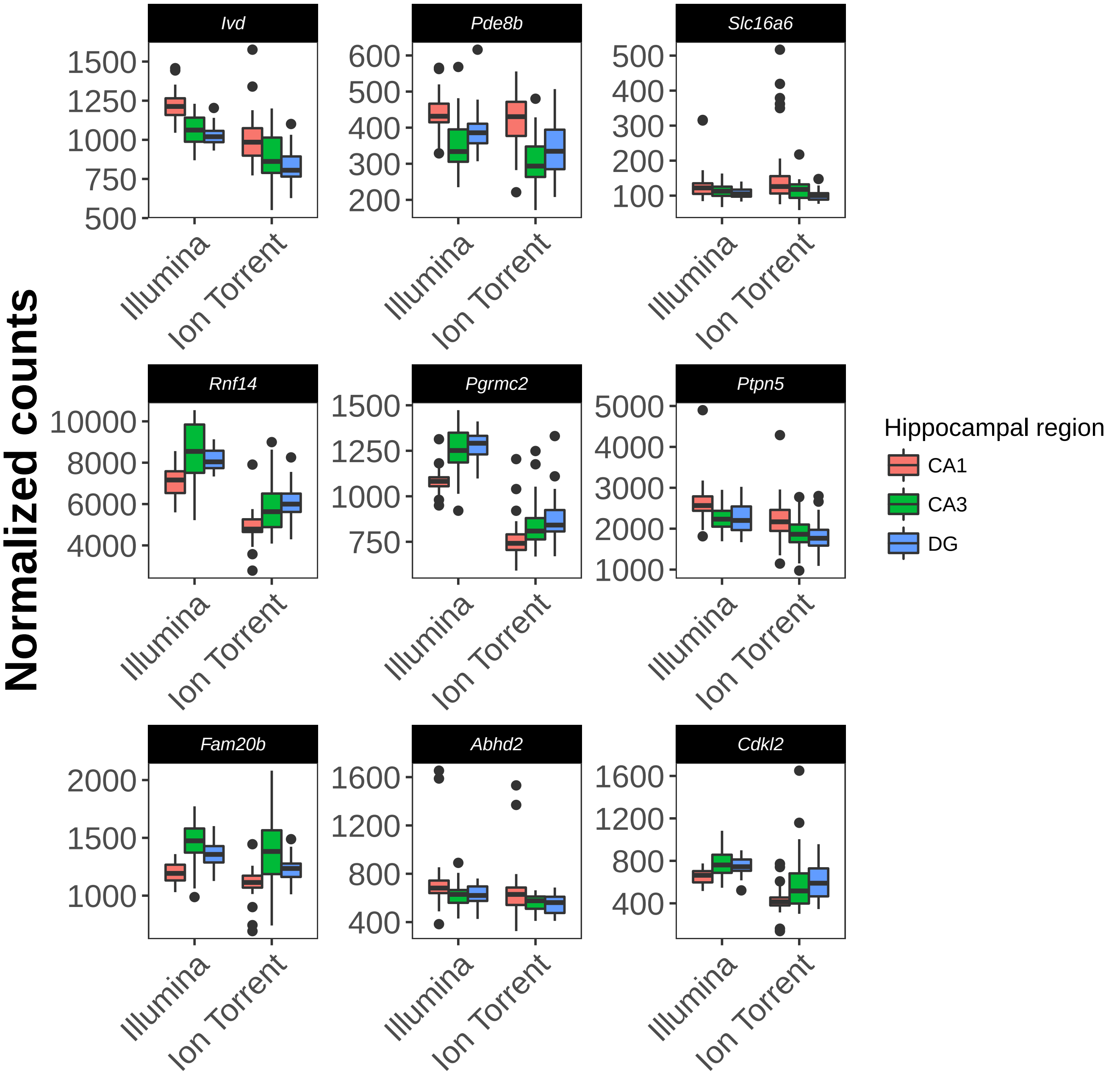
Normalized counts
Ivd
Pde8b
Slc16a6
Rnf14
Pgrmc2
Ptpn5
Fam20b
Abhd2
Cdkl2
Illumina
Ion Torrent
Hippocampal region
CA1
CA3
DG

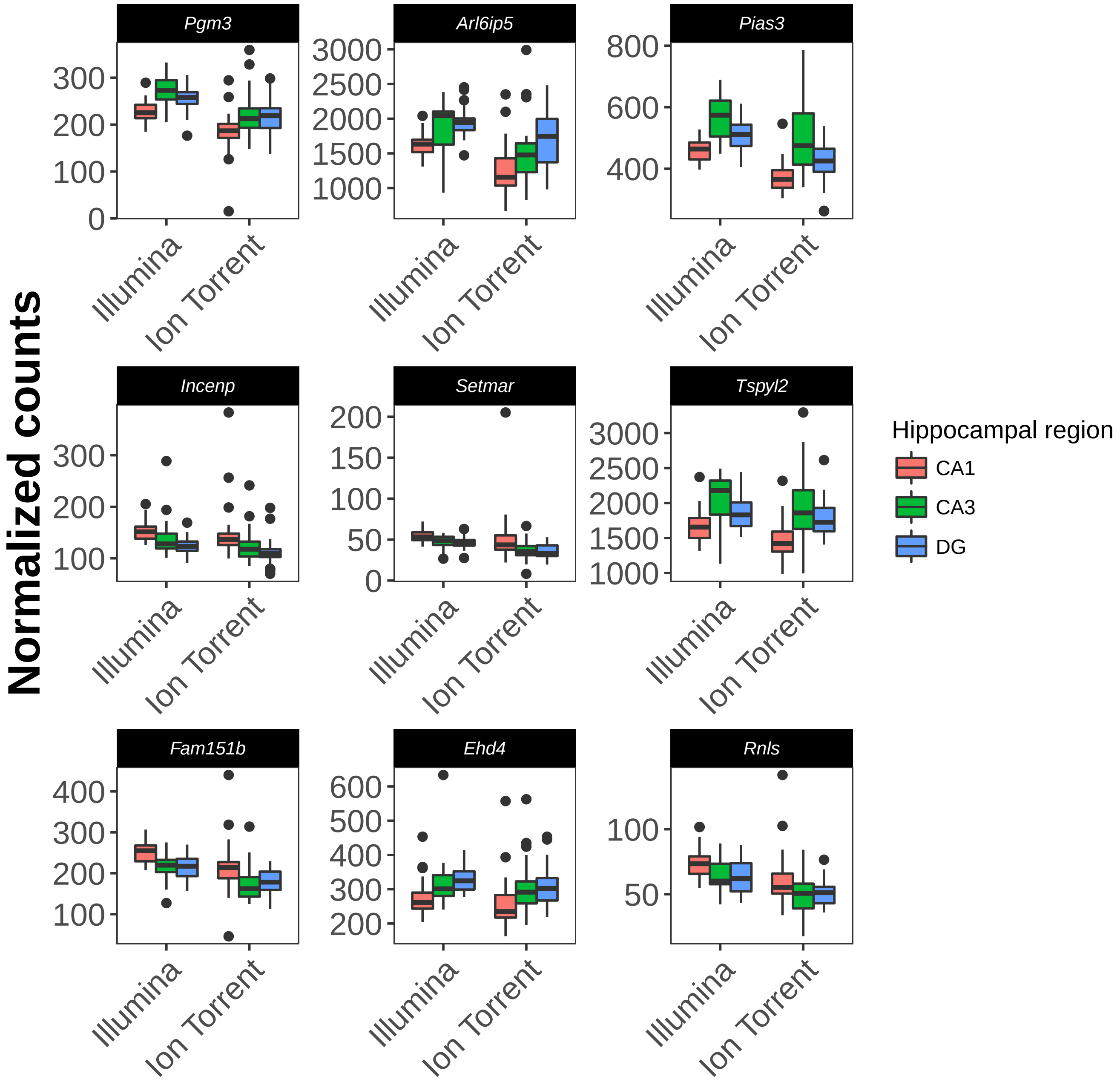
Normalized counts
Pgm3
Arl6ip5
Pias3
Incenp
Setmar
Tspyl2
Fam151b
Ehd4
Rnls
Illumina
Ion Torrent
Hippocampal region
CA1
CA3
DG

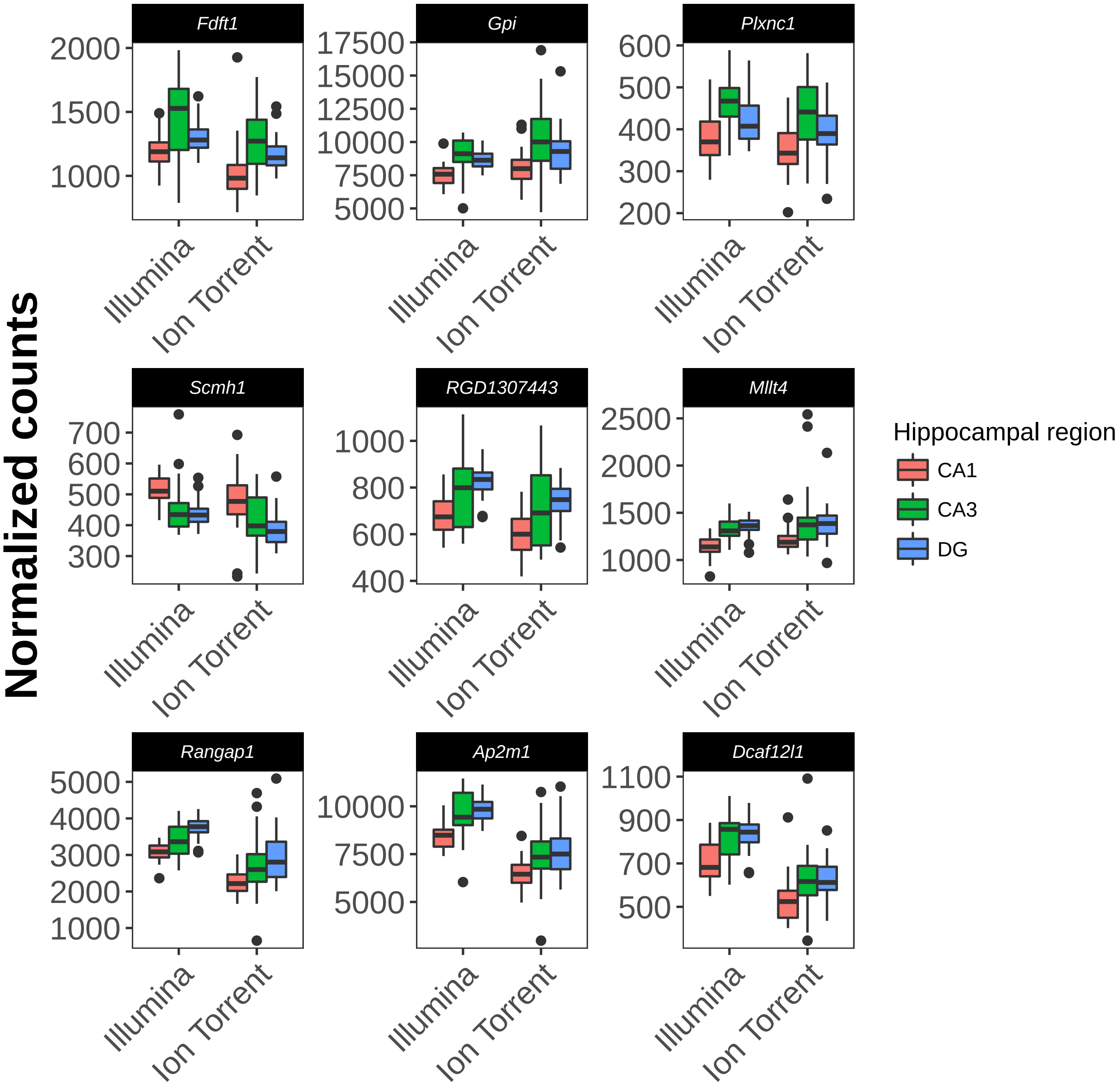

Normalized counts
Fdft1
Gpi
Plxnc1
Scmh1
RGD1307443
Mllt4
Rangap1
Ap2m1
Dcaf12l1
Illumina
Ion Torrent
Hippocampal region
CA1
CA3
DG

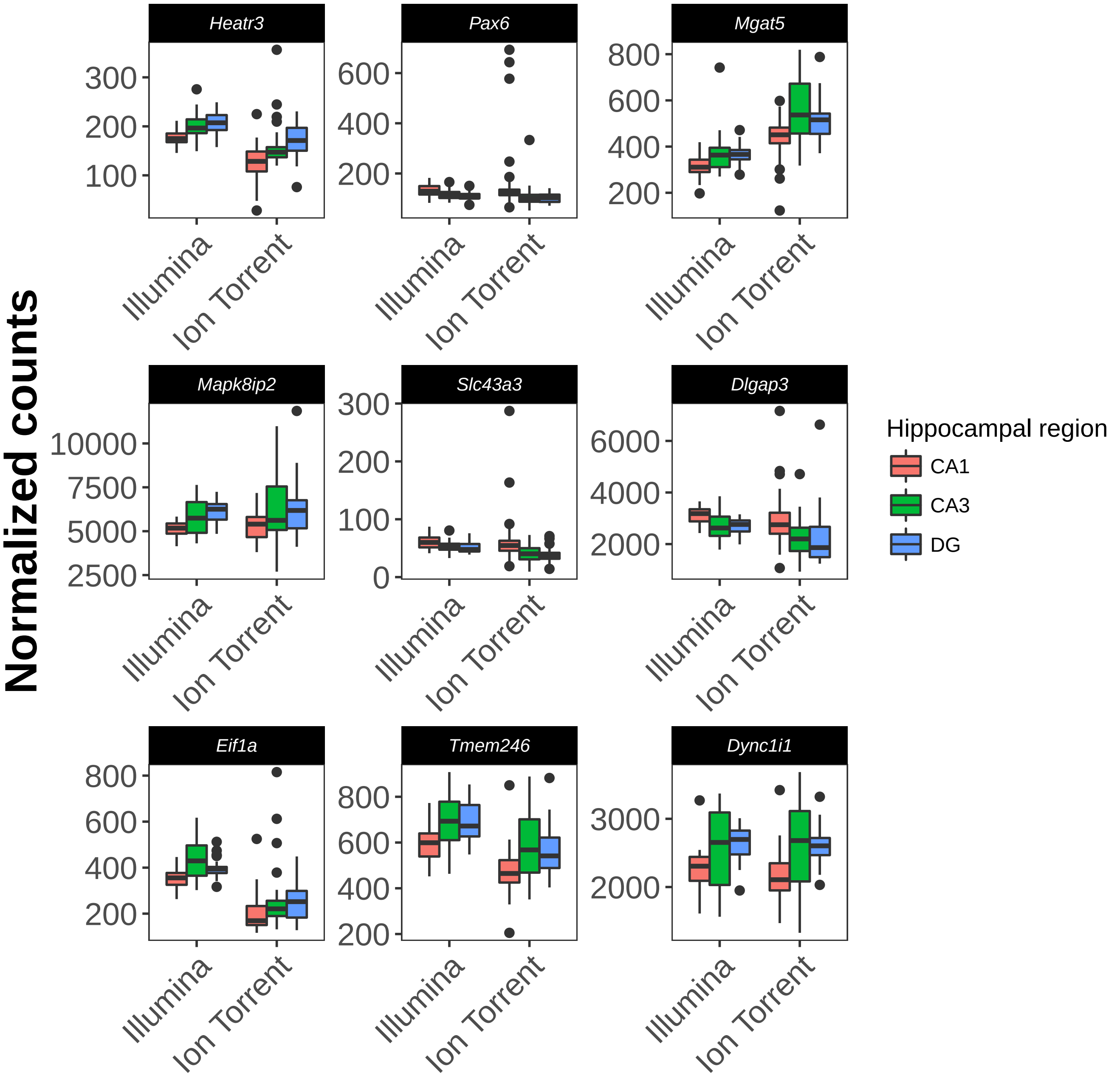

Normalized counts
Heatr3
Pax6
Mgat5
Mapk8ip2
Slc43a3
Dlgap3
Eif1a
Tmem246
Dync1i1
Illumina
Ion Torrent
Hippocampal region
CA1
CA3
DG

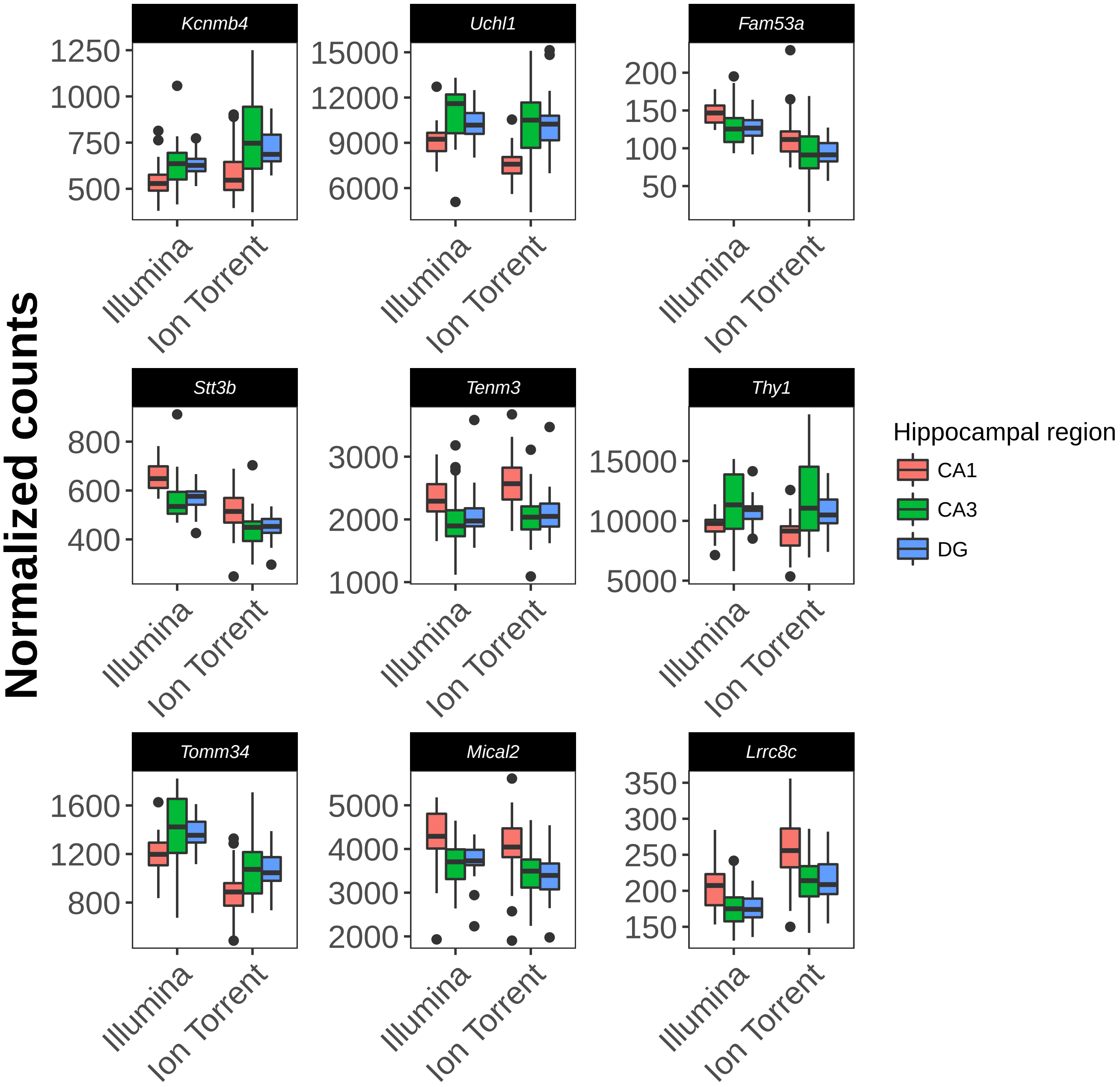

Normalized counts
Kcnmb4
Uchl1
Fam53a
Stt3b
Tenm3
Thy1
Tomm34
Mical2
Lrrc8c
Illumina
Ion Torrent
Hippocampal region
CA1
CA3
DG

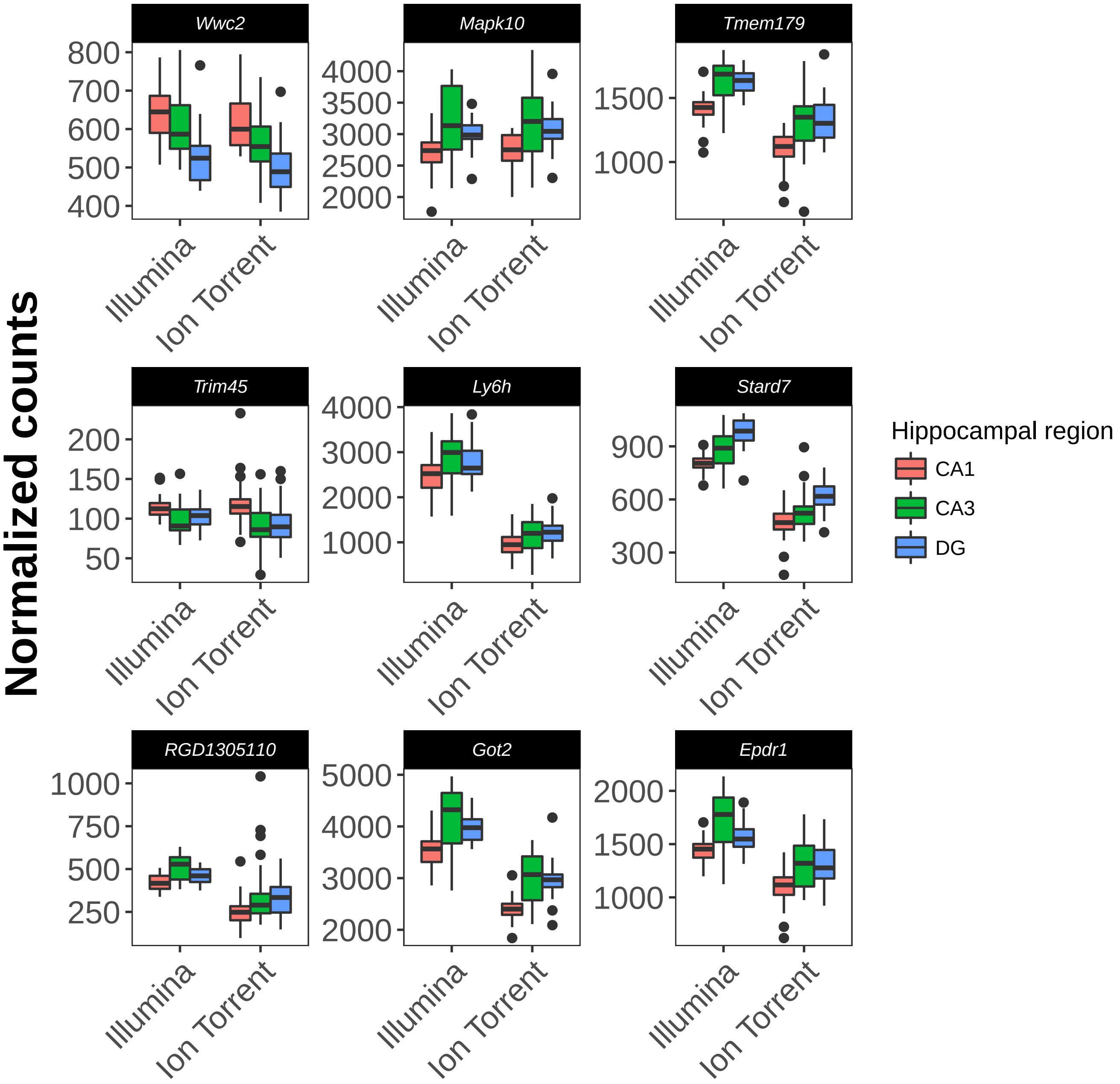
Normalized counts
Wwc2
Mapk10
Tmem179
Trim45
Ly6h
Stard7
RGD1305110
Got2
Epdr1
Illumina
Ion Torrent
Hippocampal region
CA1
CA3
DG

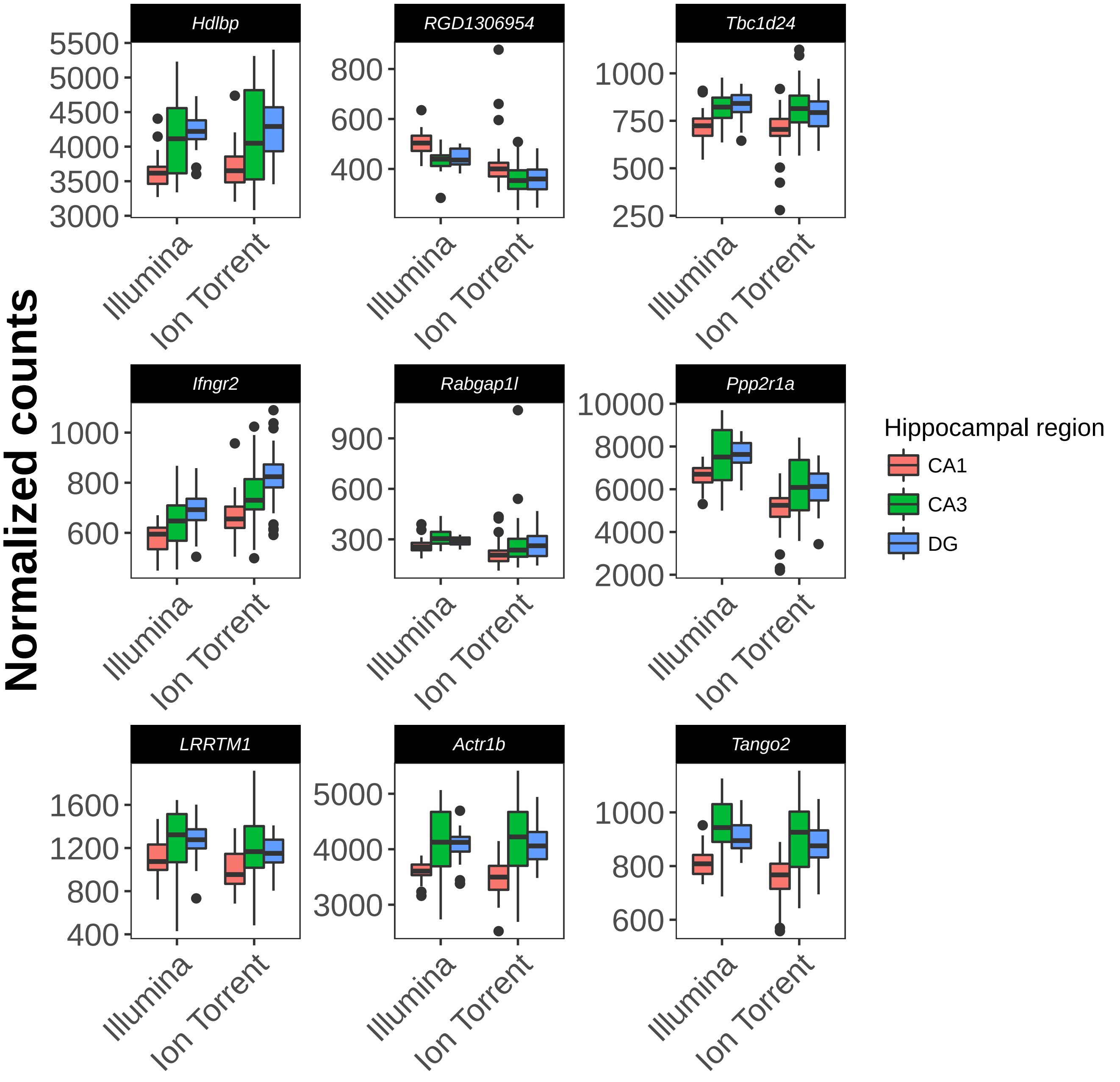

Normalized counts
Hdlbp
RGD1306954
Tbc1d24
Ifngr2
Rabgap1l
Ppp2r1a
LRRTM1
Actr1b
Tango2
Illumina
Ion Torrent
Hippocampal region
CA1
CA3
DG

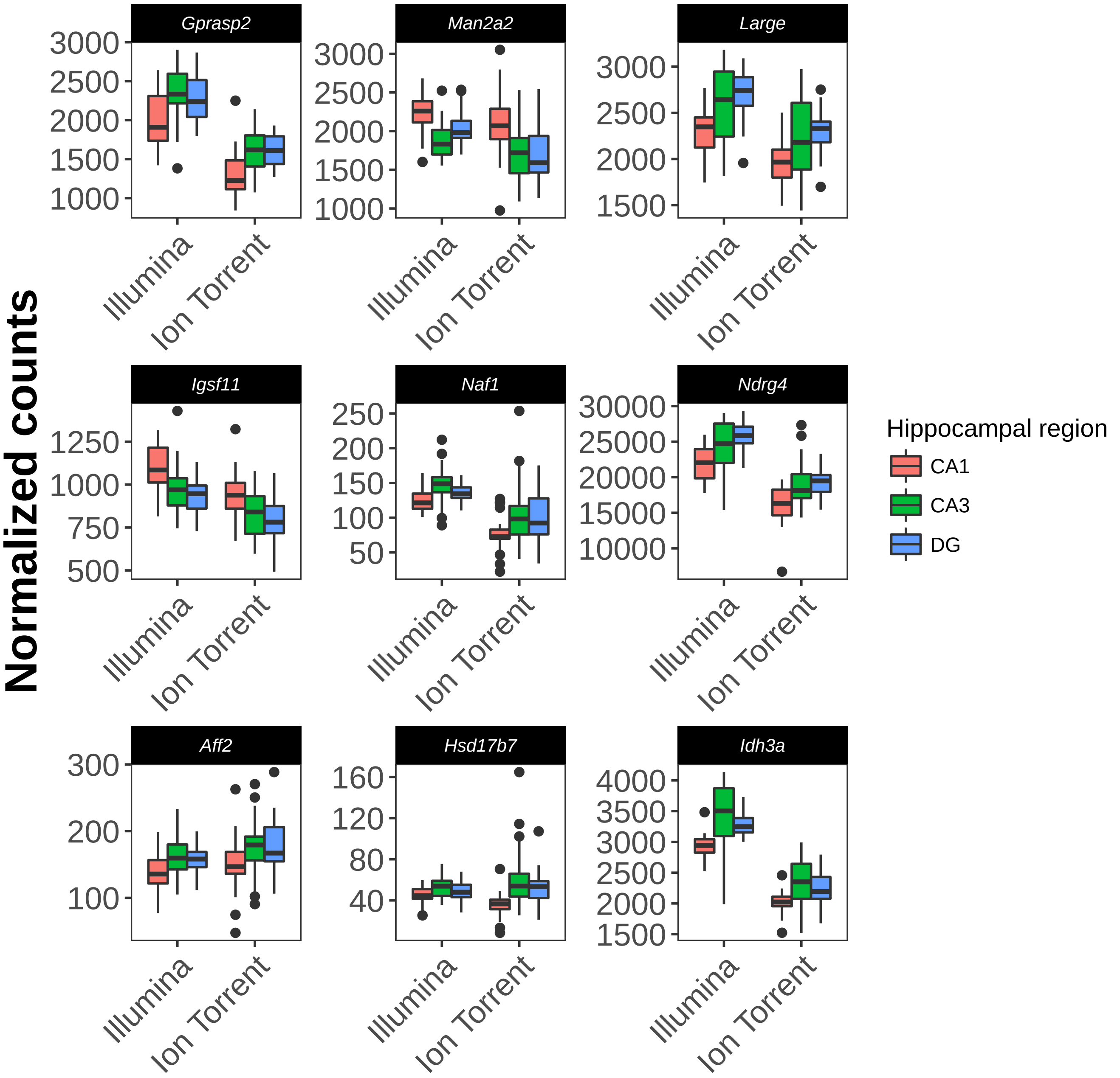
Normalized counts
Gprasp2
Man2a2
Large
Igsf11
Naf1
Ndrg4
Aff2
Hsd17b7
Idh3a
Illumina
Ion Torrent
Hippocampal region
CA1
CA3
DG

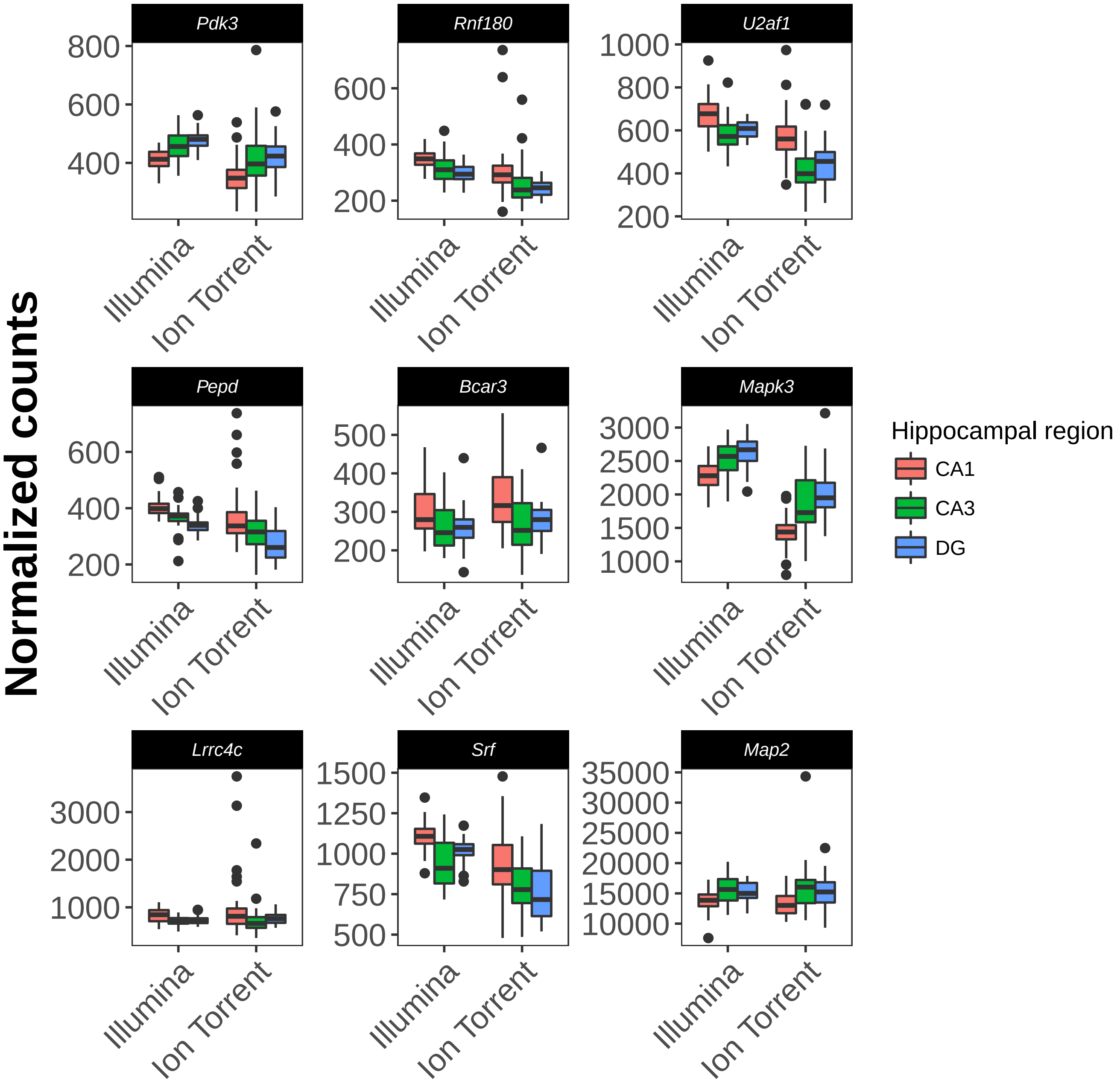

Normalized counts
Pdk3
Rnf180
U2af1
Pepd
Bcar3
Mapk3
Lrrc4c
Srf
Map2
Illumina
Ion Torrent
Hippocampal region
CA1
CA3
DG

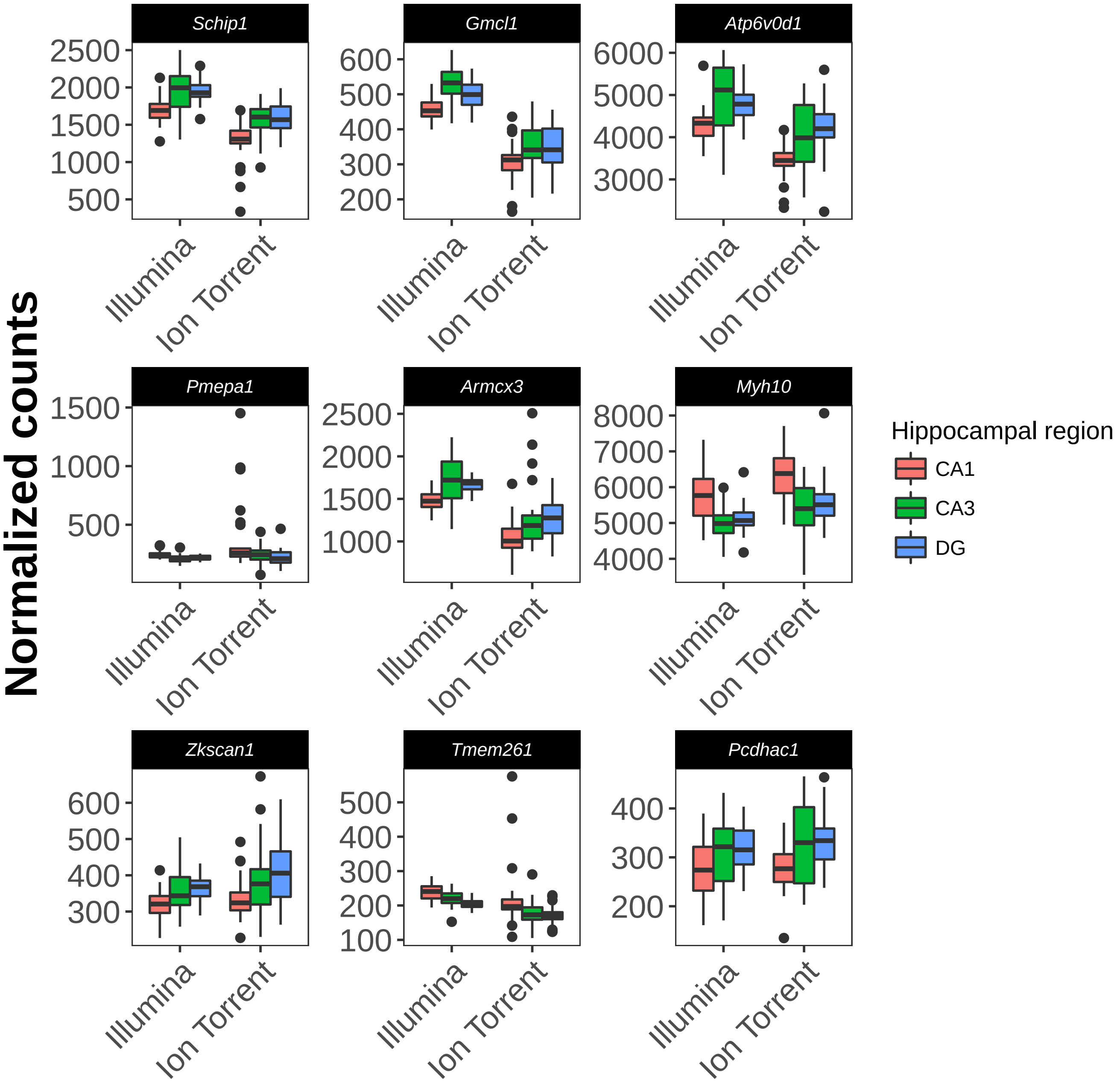

Normalized counts
Schip1
Gmcl1
Atp6v0d1
Pmepa1
Armcx3
Myh10
Zkscan1
Tmem261
Pcdhac1
Illumina
Ion Torrent
Hippocampal region
CA1
CA3
DG

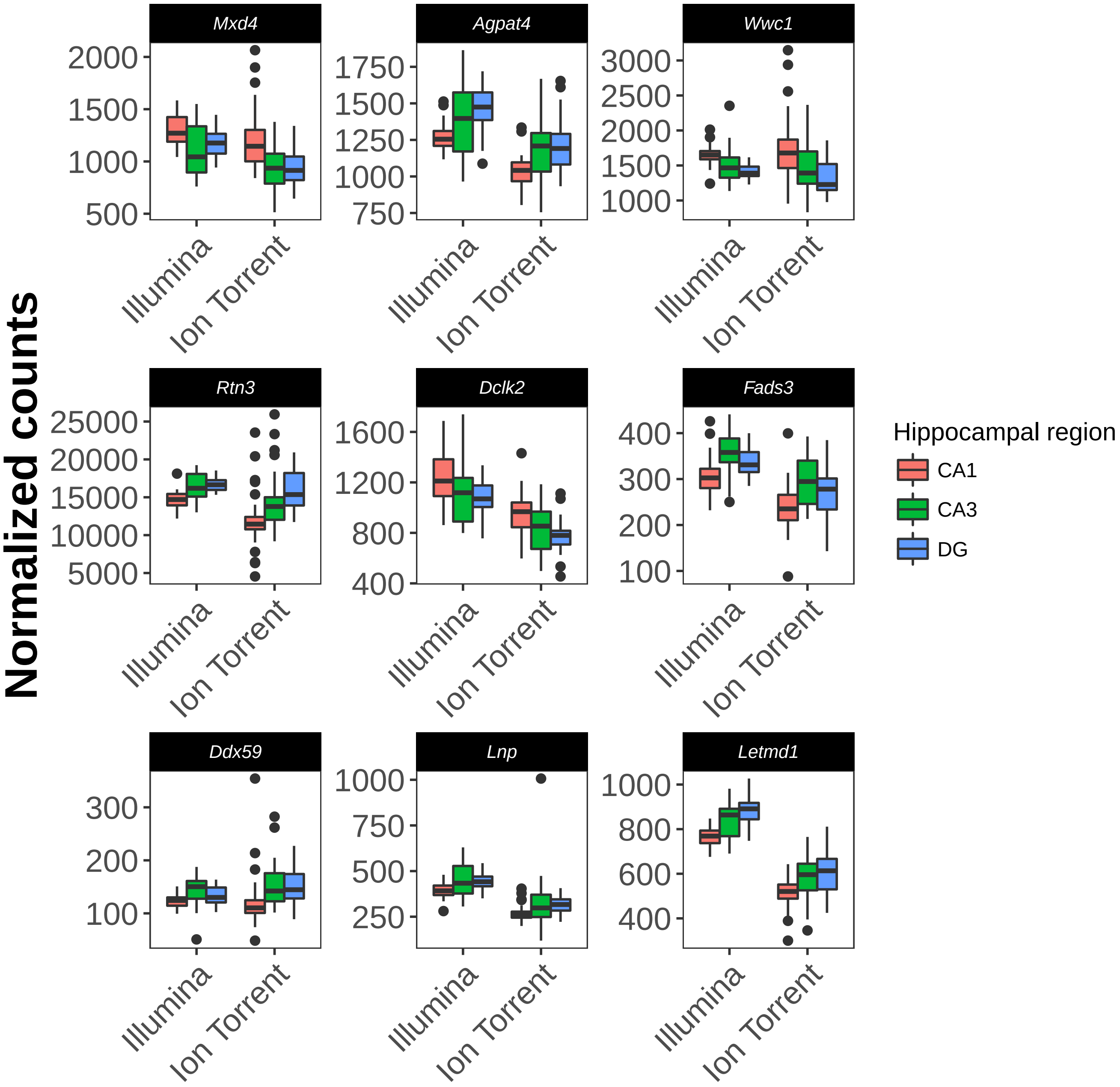

Normalized counts
Mxd4
Agpat4
Wwc1
Rtn3
Dclk2
Fads3
Ddx59
Lnp
Letmd1
Illumina
Ion Torrent
Hippocampal region
CA1
CA3
DG

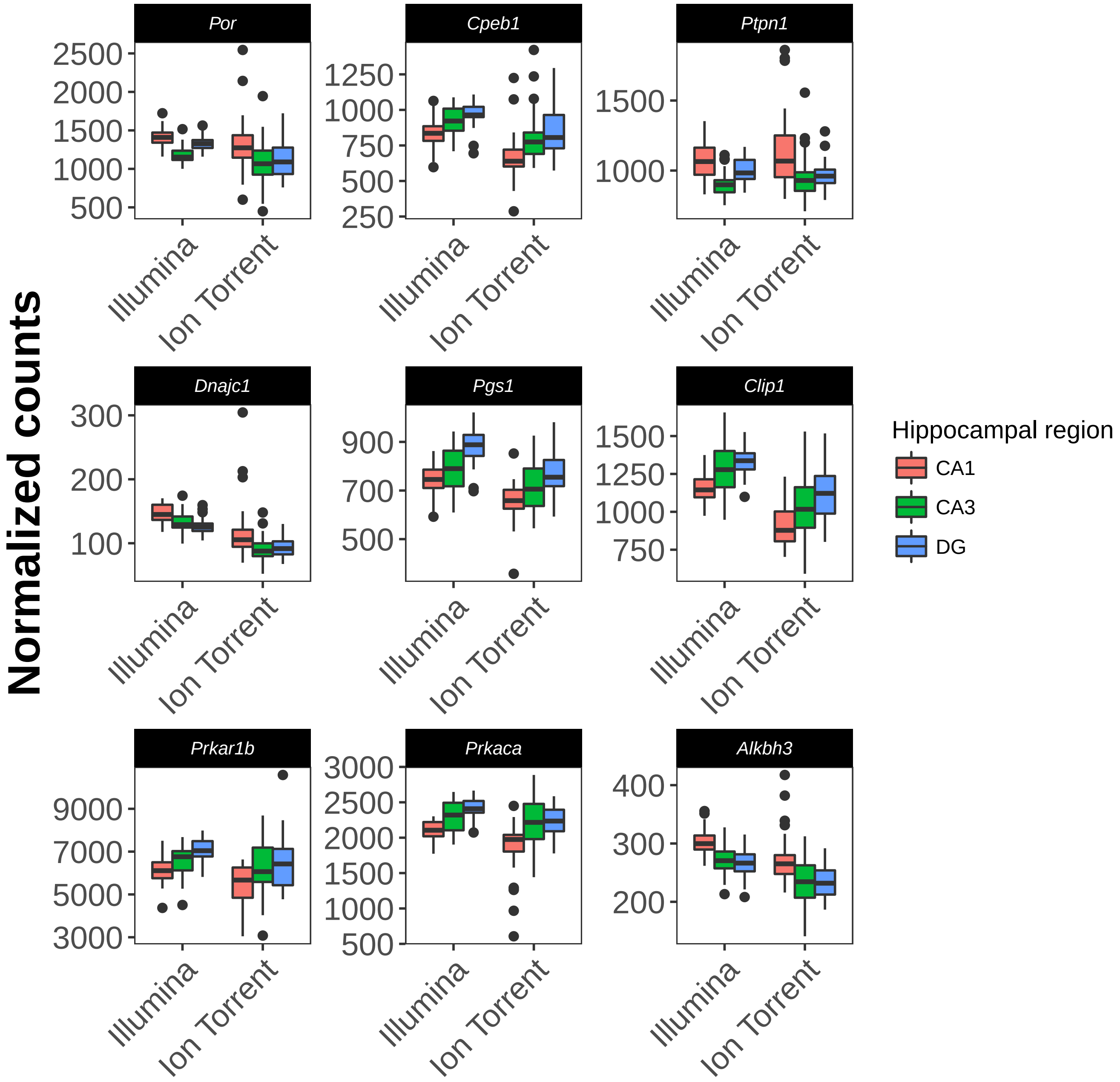
Por
Cpeb1
Ptpn1
Dnajc1
Pgs1
Clip1
Prkar1b
Prkaca
Alkbh3
Illumina
Ion Torrent
Normalized counts
Hippocampal region
CA1
CA3
DG

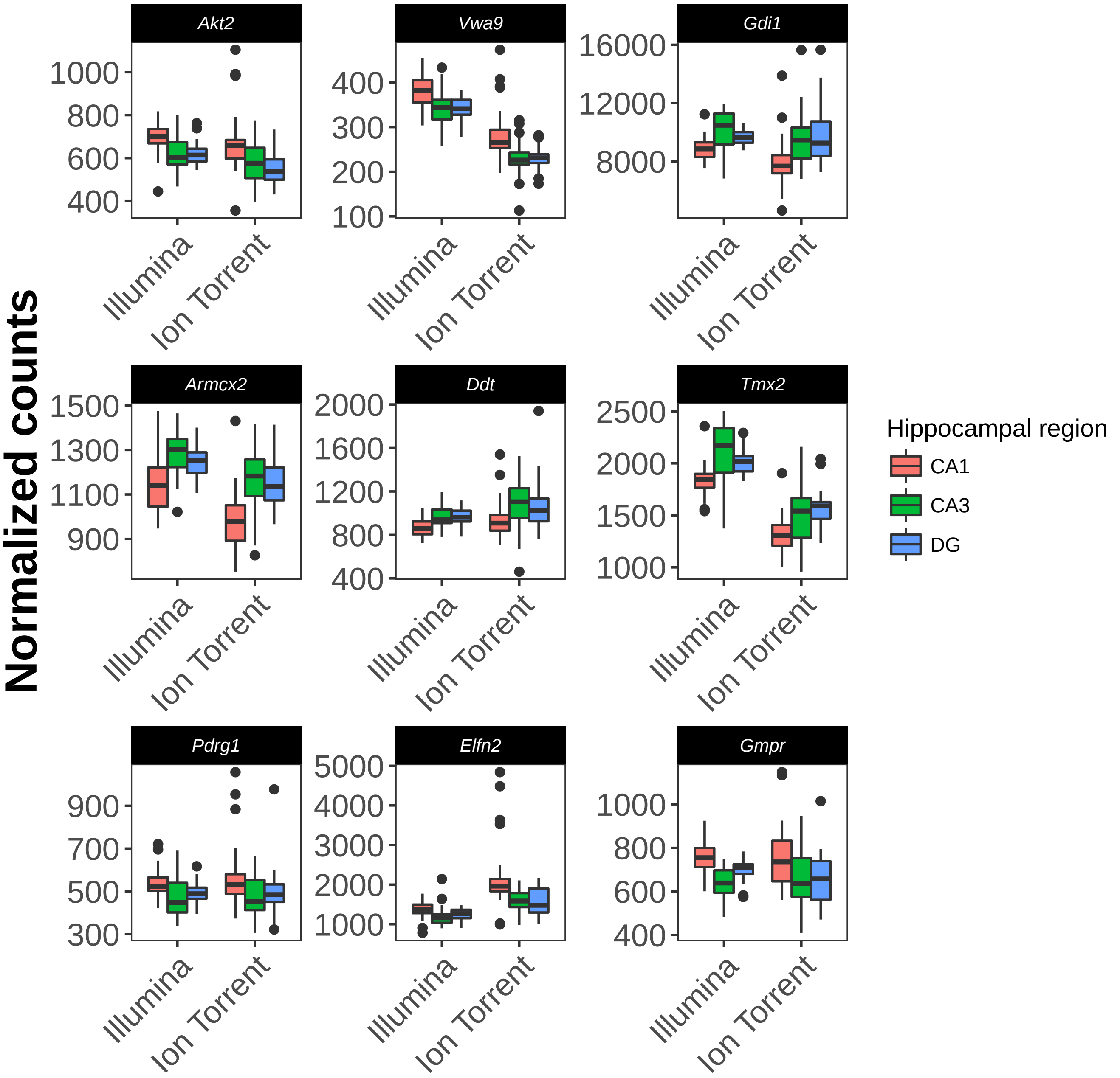

Normalized counts
Akt2
Vwa9
Gdi1
Armcx2
Ddt
Tmx2
Pdrg1
Elfn2
Gmpr
Illumina
Ion Torrent
Hippocampal region
CA1
CA3
DG

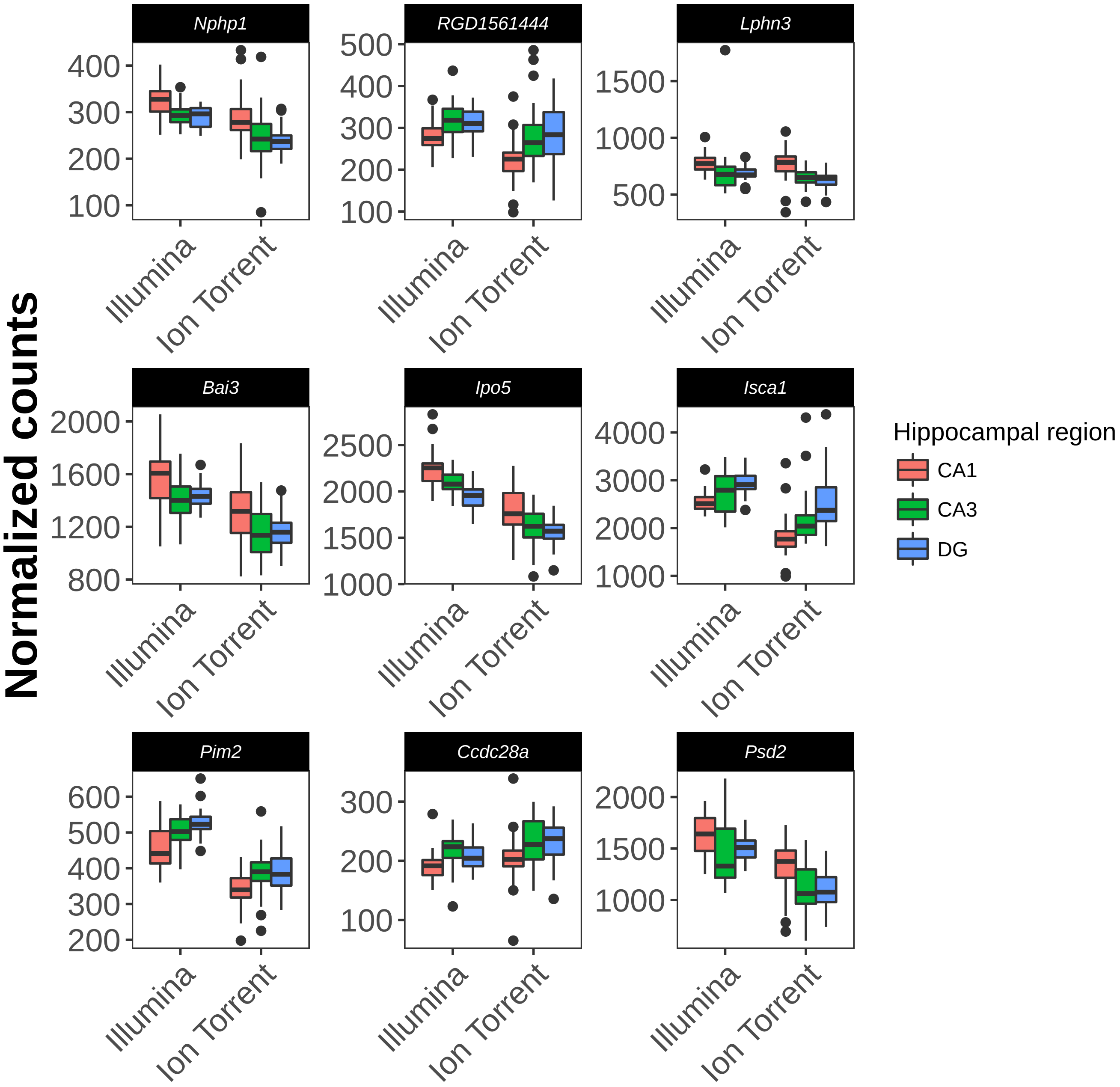

Normalized counts
Nphp1
RGD1561444
Lphn3
Bai3
Ipo5
Isca1
Pim2
Ccdc28a
Psd2
Illumina
Ion Torrent
Hippocampal region
CA1
CA3
DG

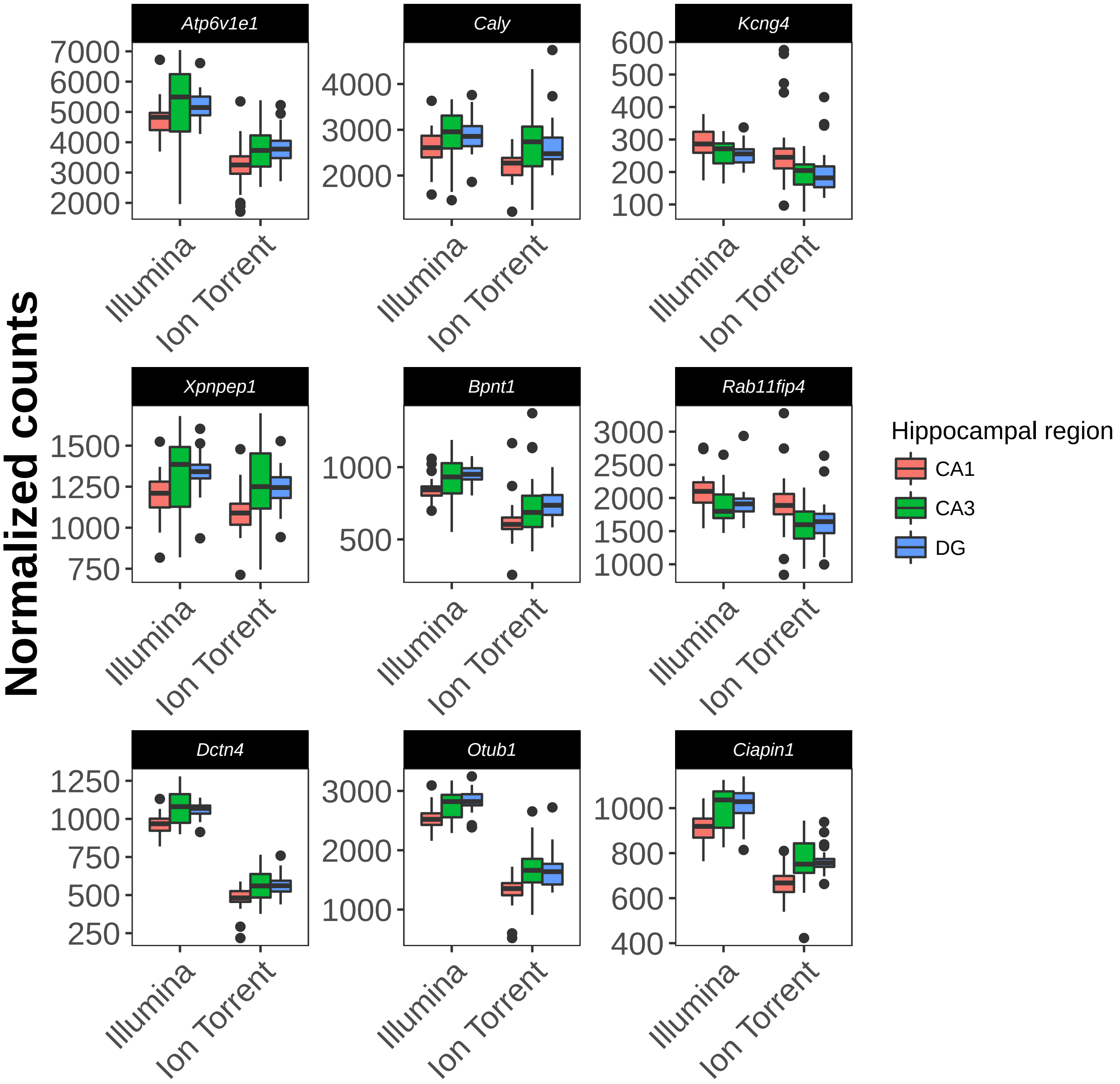
Normalized counts
Atp6v1e1
Caly
Kcng4
Xpnpep1
Bpnt1
Rab11fip4
Dctn4
Otub1
Ciapin1
Illumina
Ion Torrent
Hippocampal region
CA1
CA3
DG

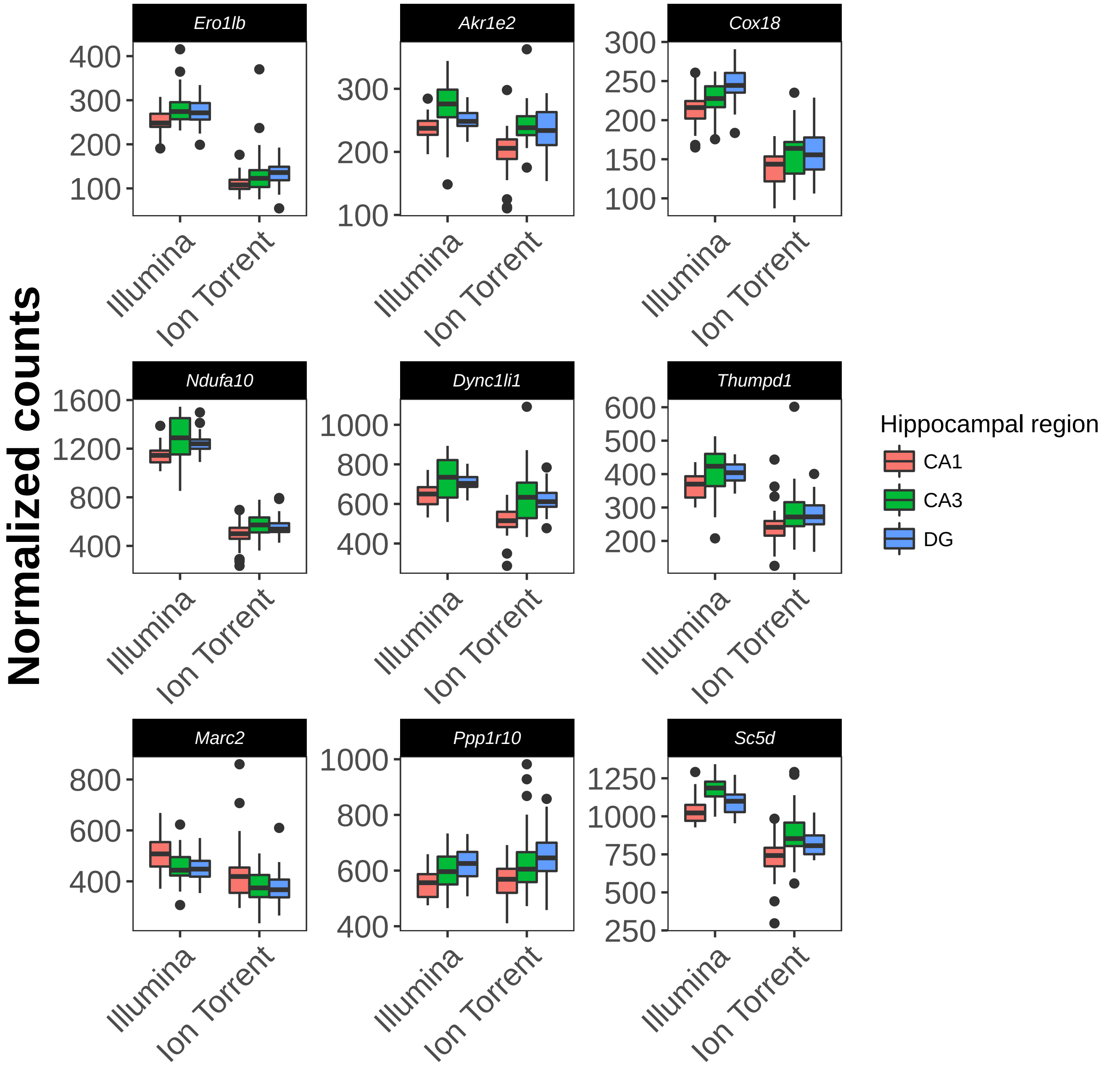

Normalized counts
Ero1lb
Akr1e2
Cox18
Ndufa10
Dync1li1
Thumpd1
Marc2
Ppp1r10
Sc5d
Illumina
Ion Torrent
Hippocampal region
CA1
CA3
DG

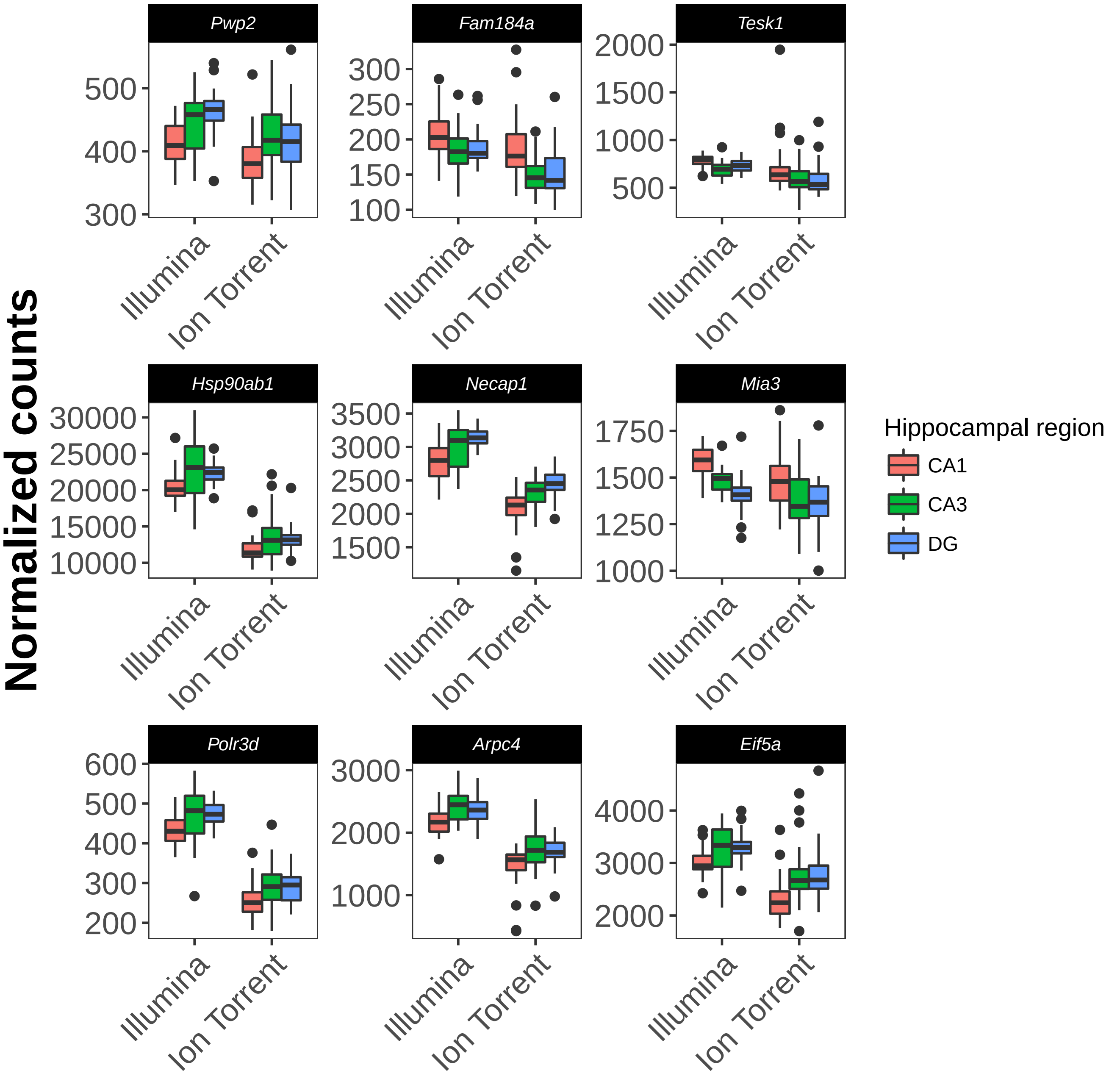
Normalized counts
Pwp2
Fam184a
Tesk1
Hsp90ab1
Necap1
Mia3
Polr3d
Arpc4
Eif5a
Illumina
Ion Torrent
Hippocampal region
CA1
CA3
DG

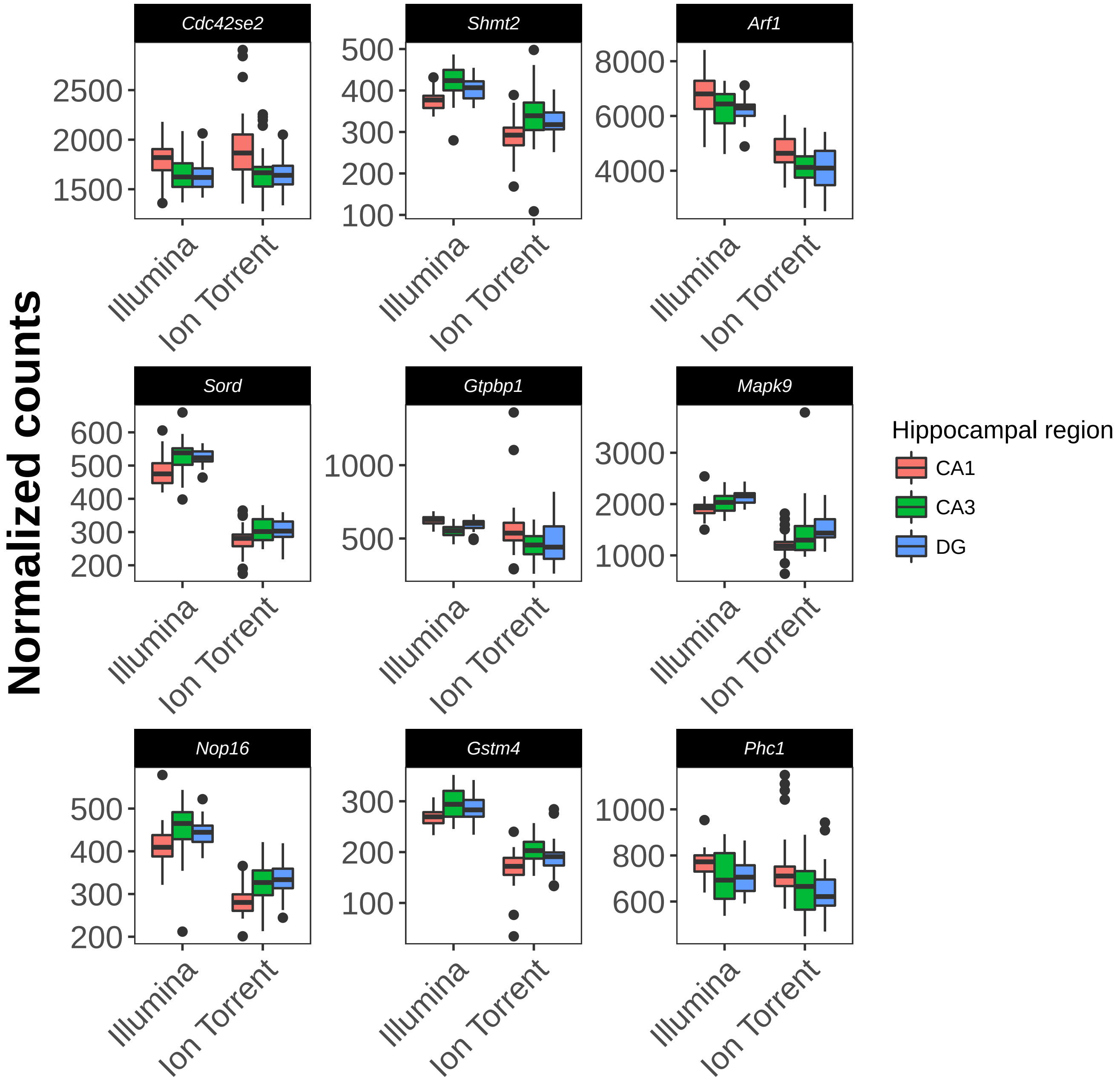

Normalized counts
Cdc42se2
Shmt2
Arf1
Sord
Gtpbp1
Mapk9
Nop16
Gstm4
Phc1
Illumina
Ion Torrent
Hippocampal region
CA1
CA3
DG

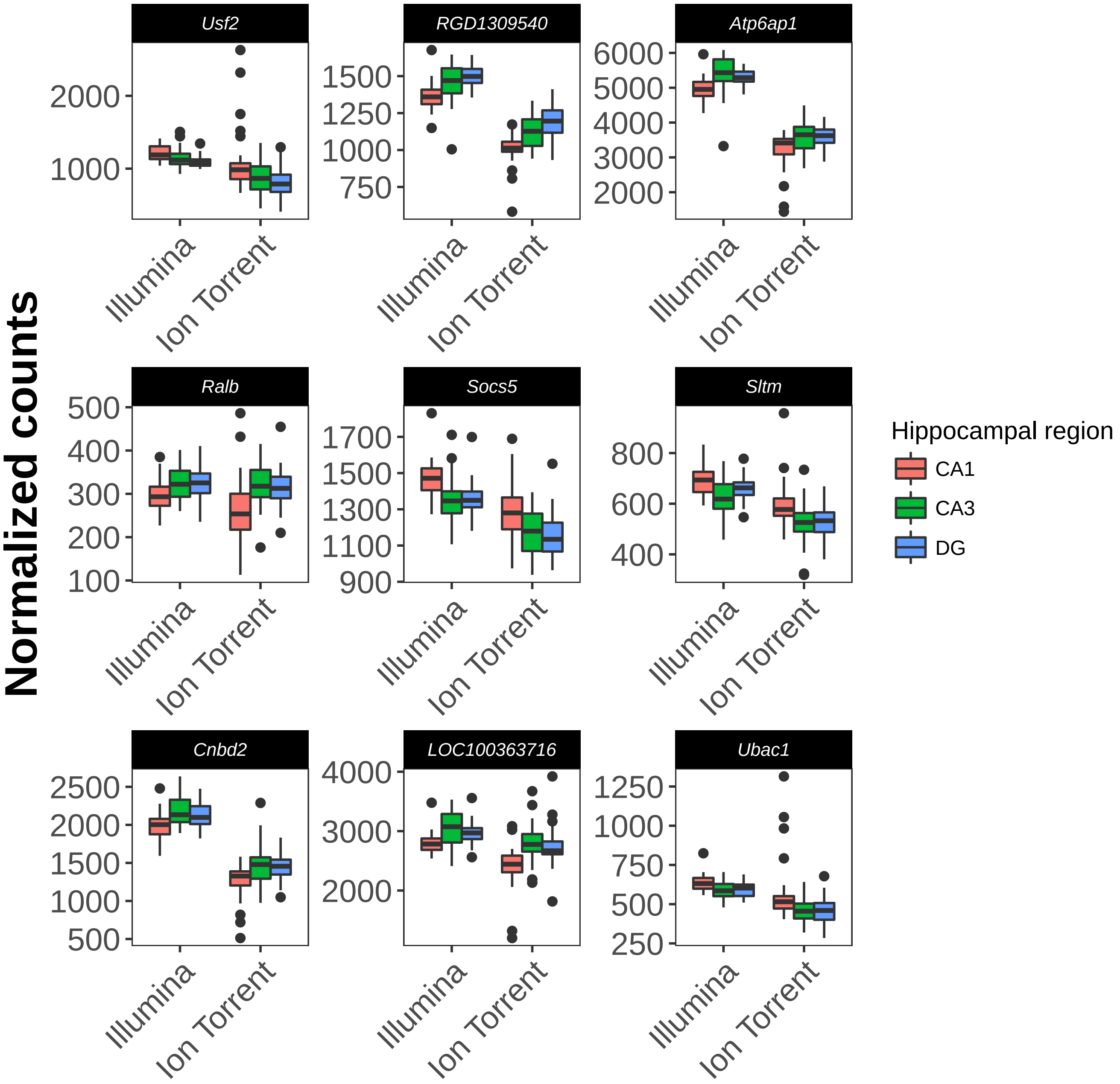

Usf2
RGD1309540
Atp6ap1
Ralb
Socs5
Sltm
Cnbd2
LOC100363716
Ubac1
Normalized counts
Illumina
Ion Torrent
Hippocampal region
CA1
CA3
DG

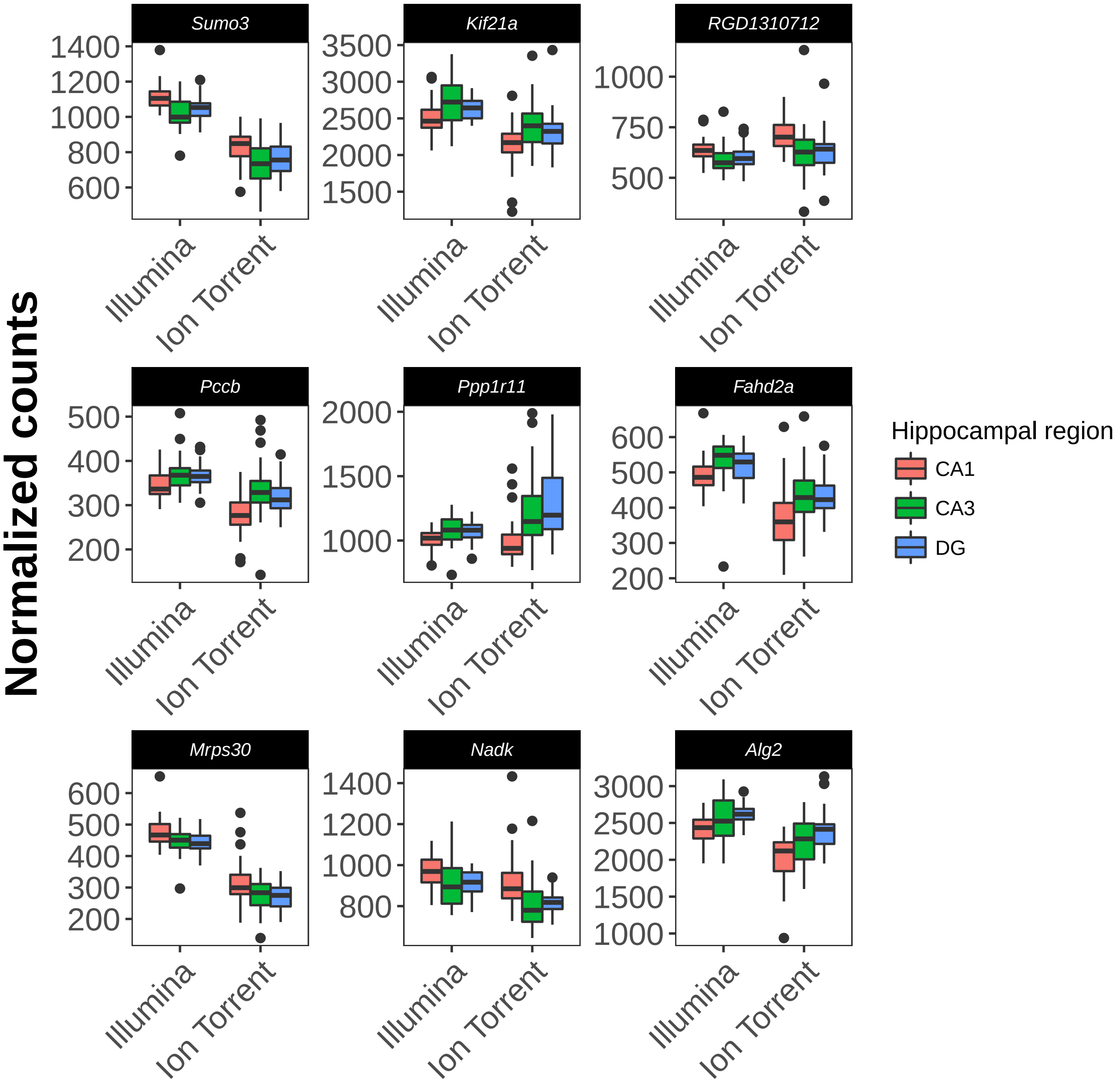

Sumo3
Kif21a
RGD1310712
Pccb
Ppp1r11
Fahd2a
Mrps30
Nadk
Alg2
Normalized counts
Illumina
Ion Torrent
Hippocampal region
CA1
CA3
DG

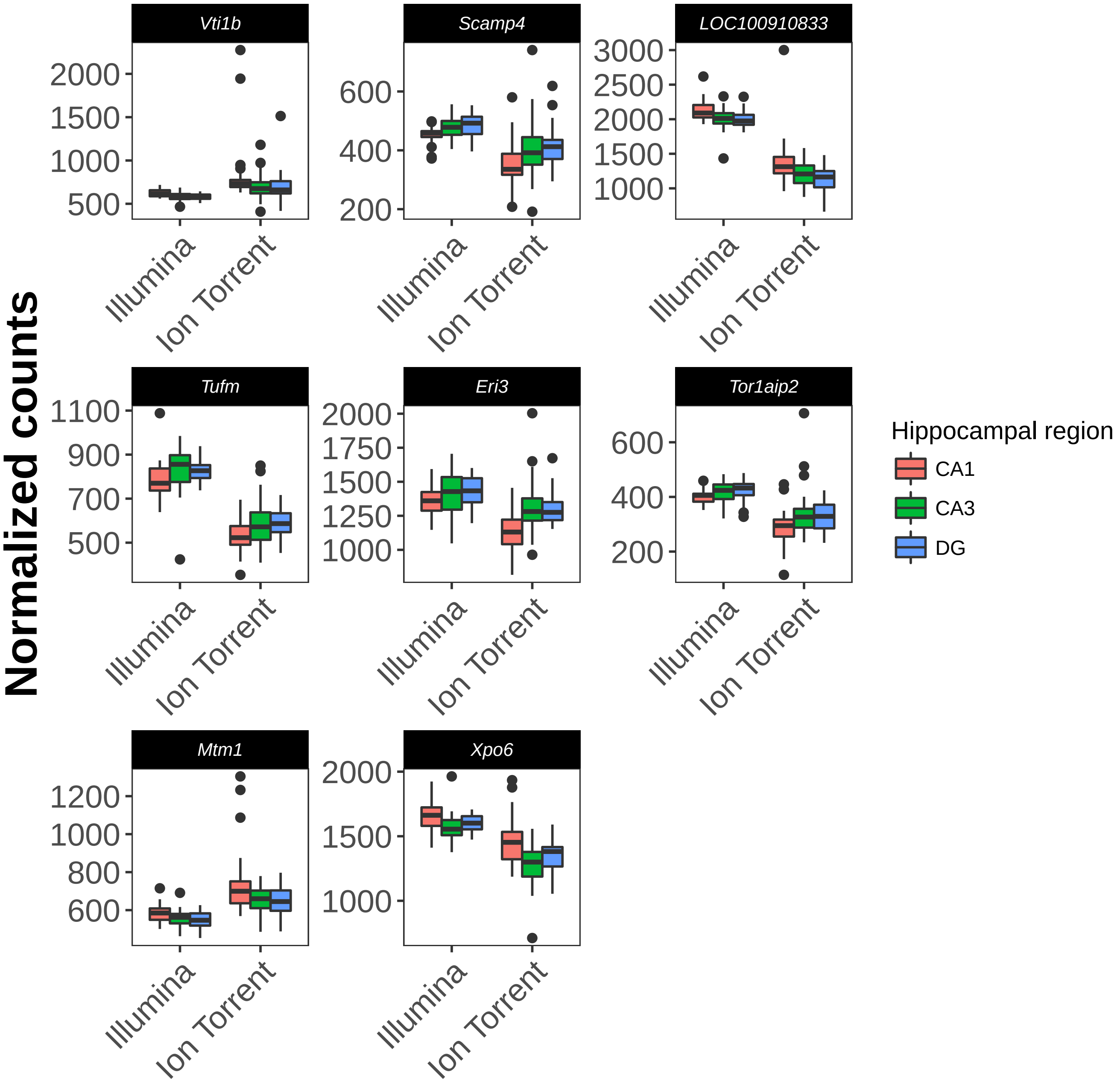
Normalized counts
Vti1b
Scamp4
LOC100910833
Tufm
Eri3
Tor1aip2
Mtm1
Xpo6
Illumina
Ion Torrent
Hippocampal region
CA1
CA3
DG
