# Supplementary material for: Hippocampal Transcriptomic Profiles: Subfield Vulnerability to Age and Cognitive Impairment
Source: Front Aging Neurosci. 2017 Dec 8;9:383. doi: 10.3389/fnagi.2017.00383 (PMC5727020; doi:10.3389/fnagi.2017.00383)
Supplement: Supplementary Figure 5 — Boxplots for the CA3 specific genes reported in Table S5. The normalized counts are from the Illumina experiment, and the genes are ranked according the average Fold Change computed from the pairwise comparisons CA3 vs. CA1 and CA3 vs. DG. [file Image5.PDF]

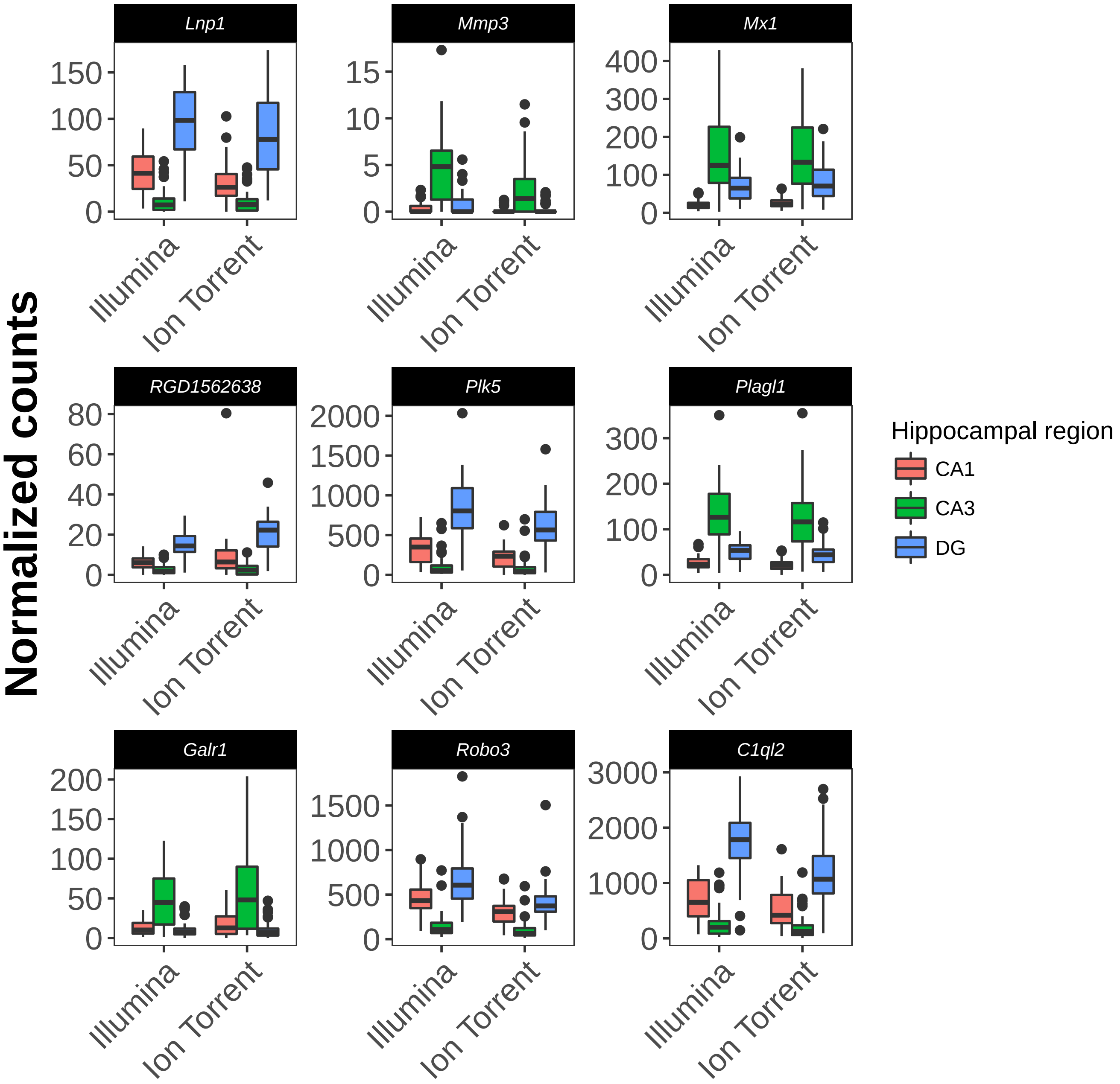
Normalized counts
Lnp1
Mmp3
Mx1
RGD1562638
Plk5
Plagl1
Galr1
Robo3
C1ql2
Illumina
Ion Torrent
Hippocampal region
CA1
CA3
DG

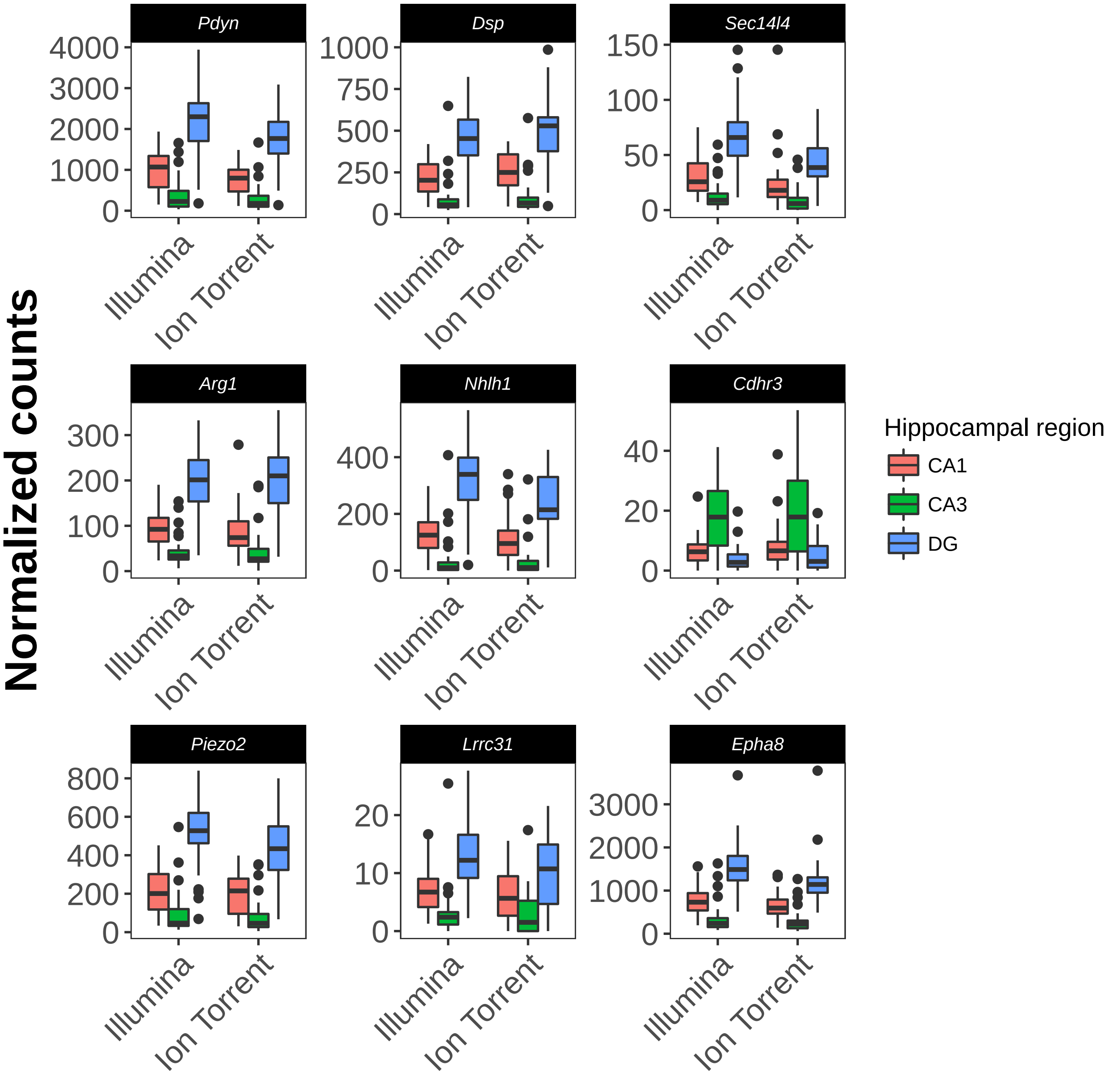

Normalized counts
Pdyn
Dsp
Sec14l4
Arg1
Nhlh1
Cdhr3
Piezo2
Lrrc31
Epha8
Illumina
Ion Torrent
Hippocampal region
CA1
CA3
DG

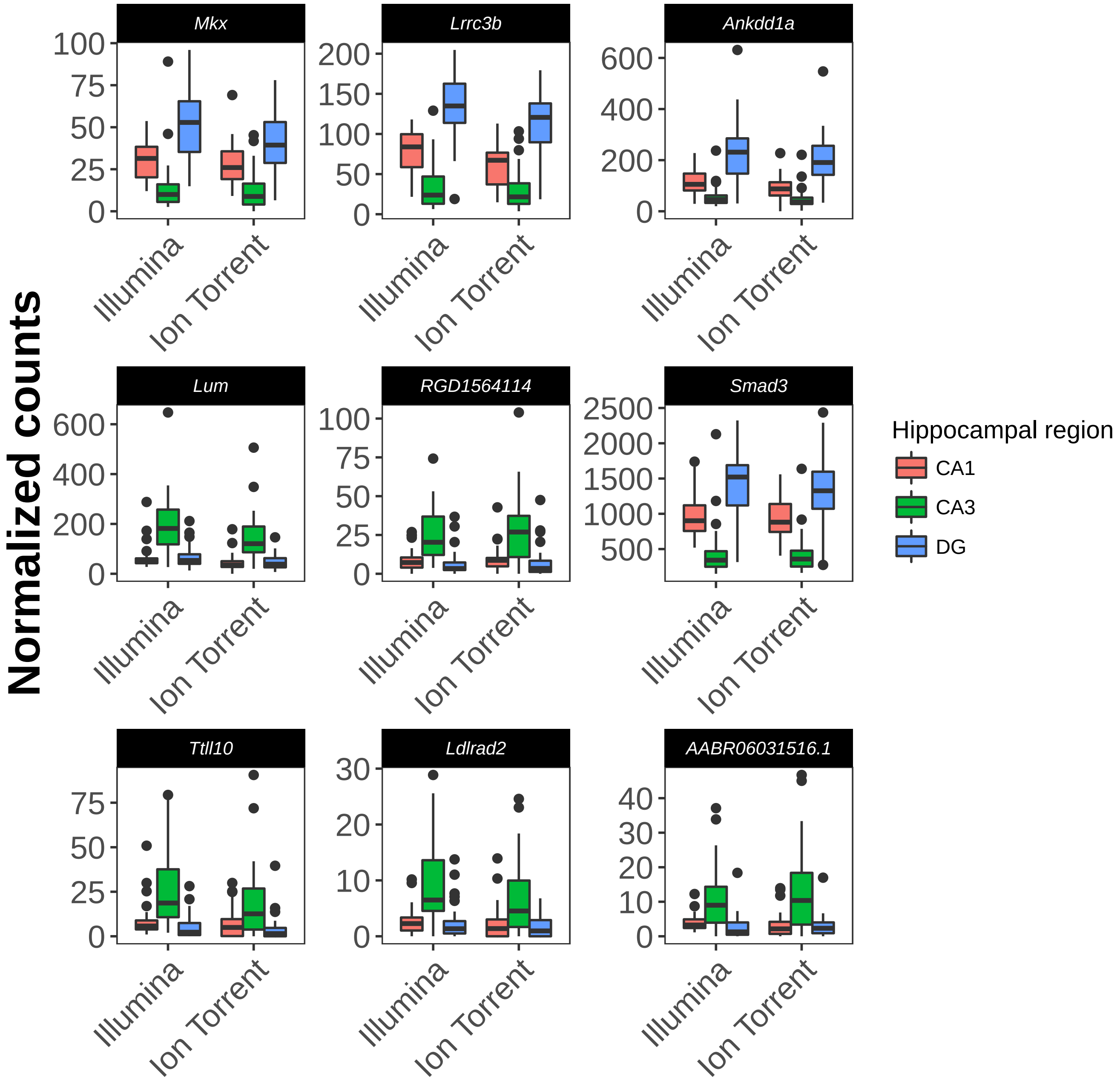
Mkx
Lrrc3b
Ankdd1a
Lum
RGD1564114
Smad3
Ttll10
Ldlrad2
AABR06031516.1
Normalized counts
Illumina
Ion Torrent
Hippocampal region
CA1
CA3
DG

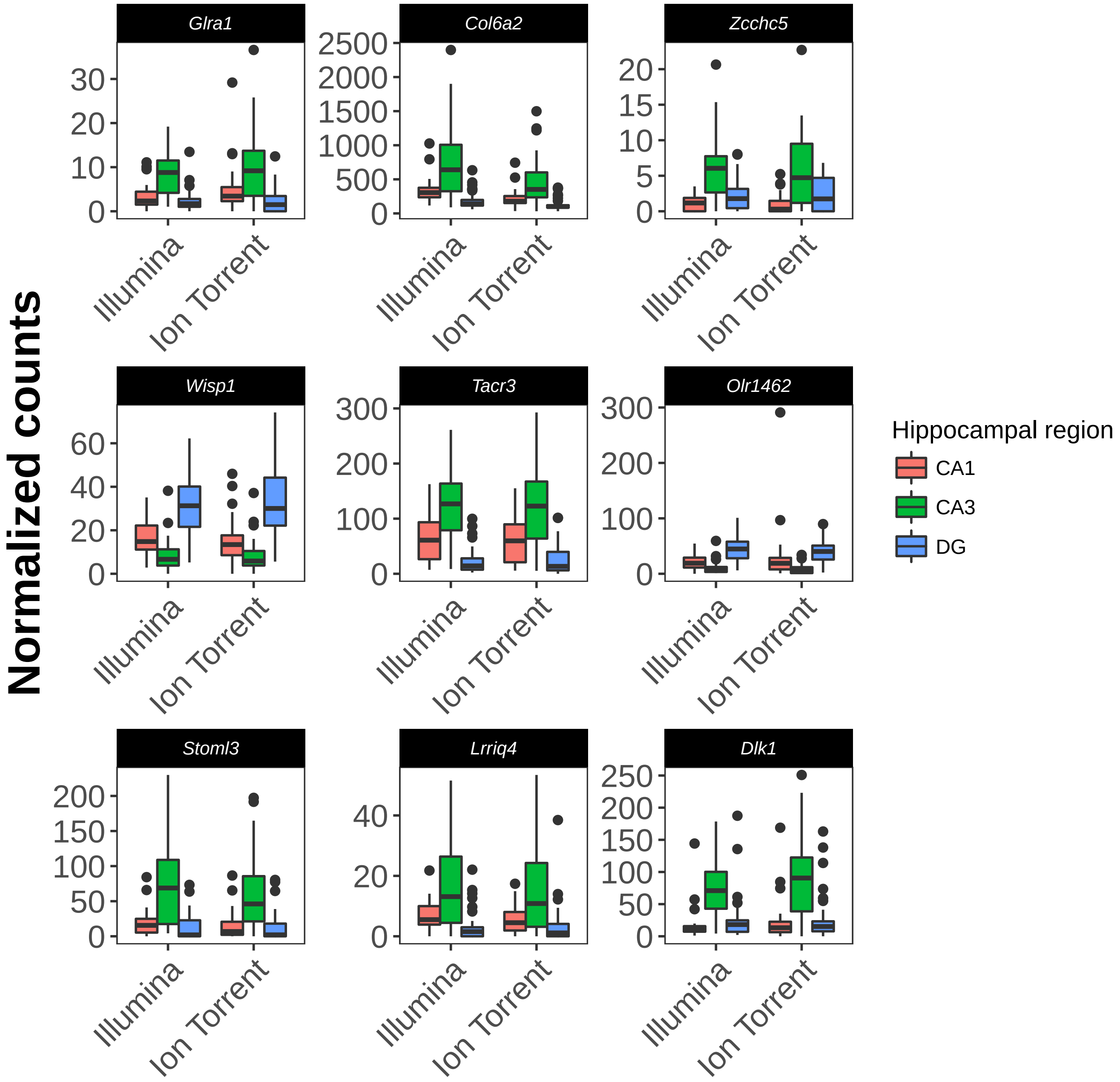

Normalized counts
Glra1
Col6a2
Zcchc5
Wisp1
Tacr3
Olr1462
Stoml3
Lrriq4
Dlk1
Illumina
Ion Torrent
Hippocampal region
CA1
CA3
DG

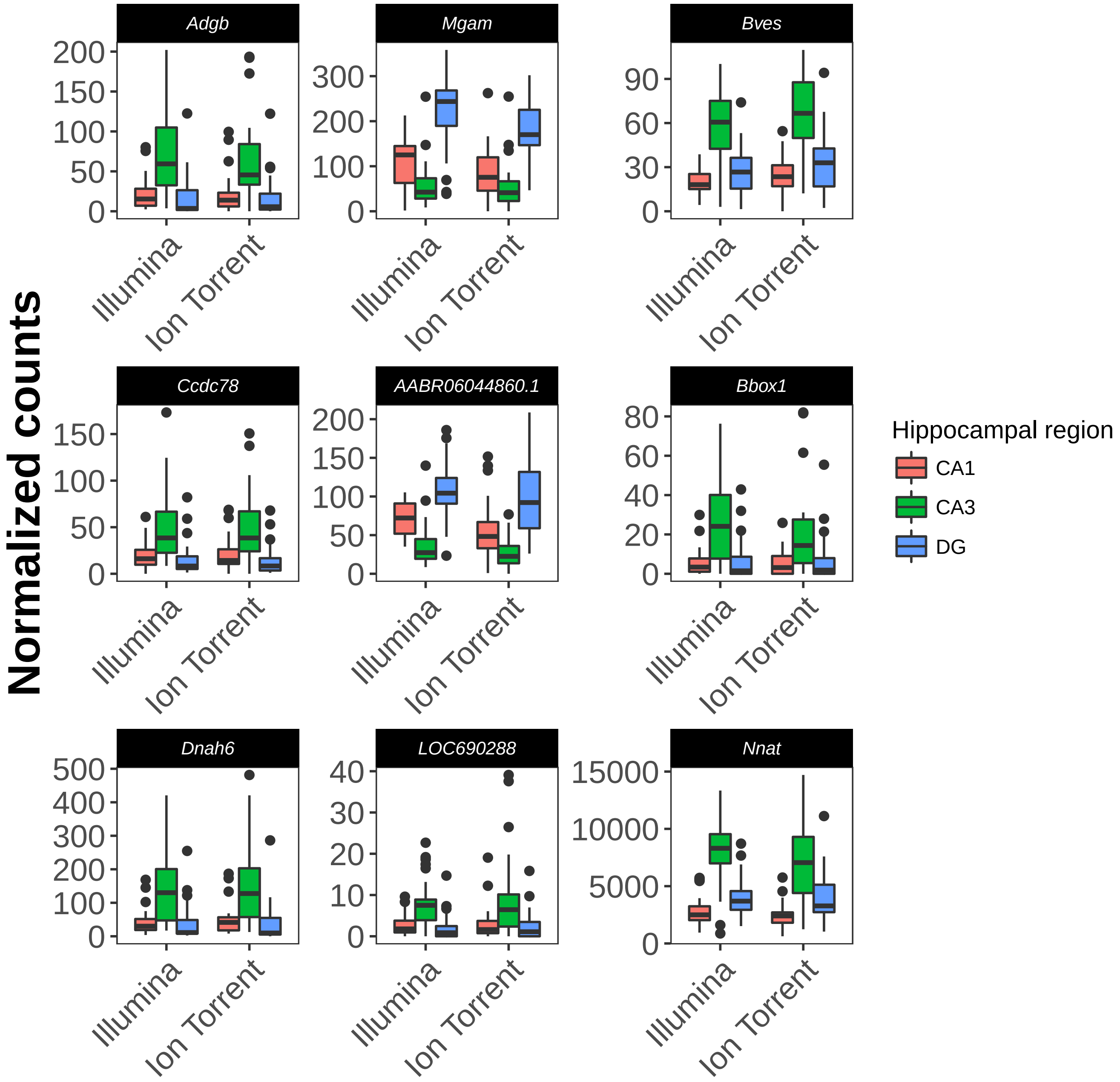

Normalized counts
Adgb
Mgam
Bves
Ccdc78
AABR06044860.1
Bbox1
Dnah6
LOC690288
Nnat
Illumina
Ion Torrent
Hippocampal region
CA1
CA3
DG

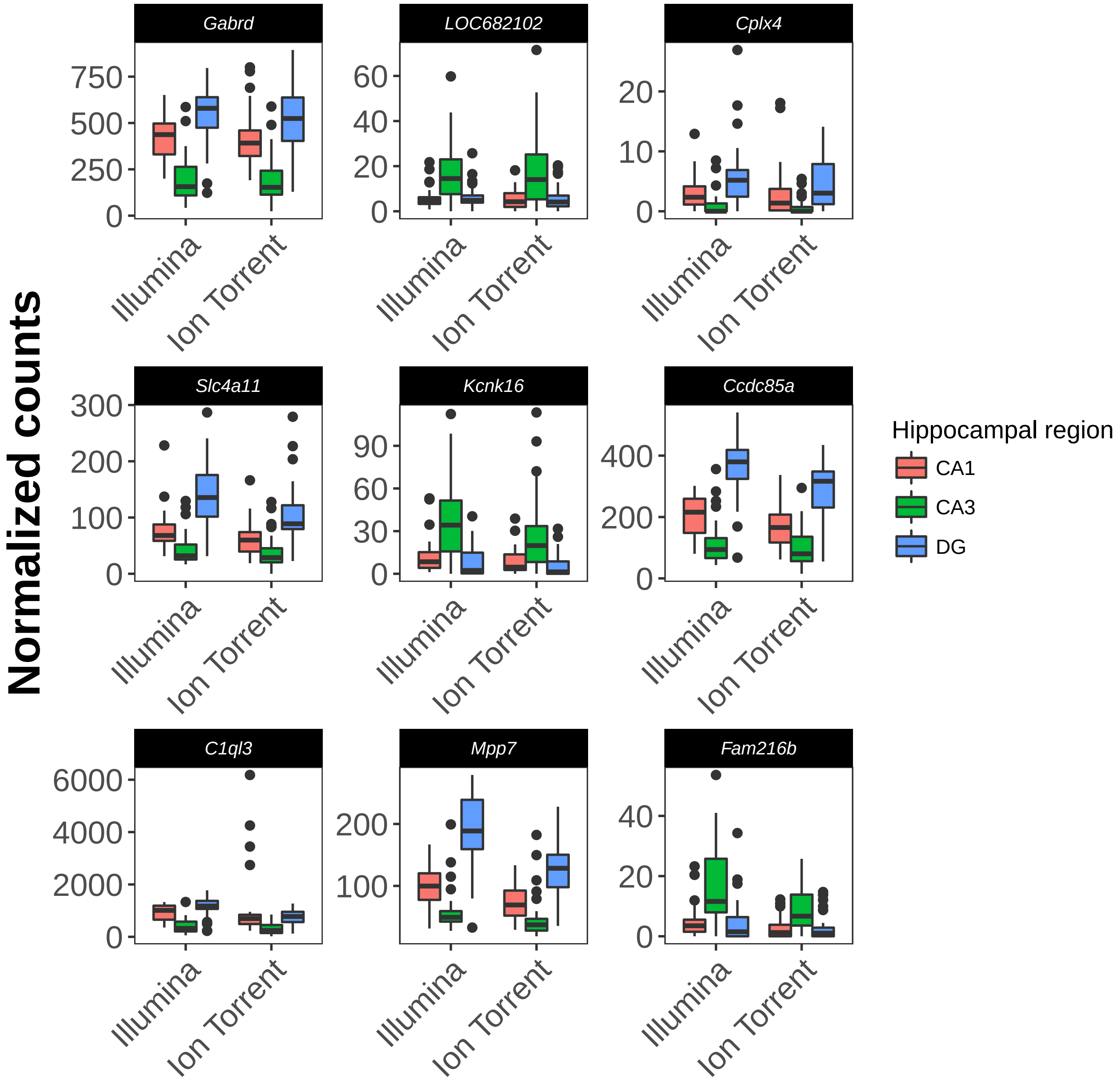
Normalized counts
Gabrd
LOC682102
Cplx4
Slc4a11
Kcnk16
Ccdc85a
C1ql3
Mpp7
Fam216b
Illumina
Ion Torrent
Hippocampal region
CA1
CA3
DG

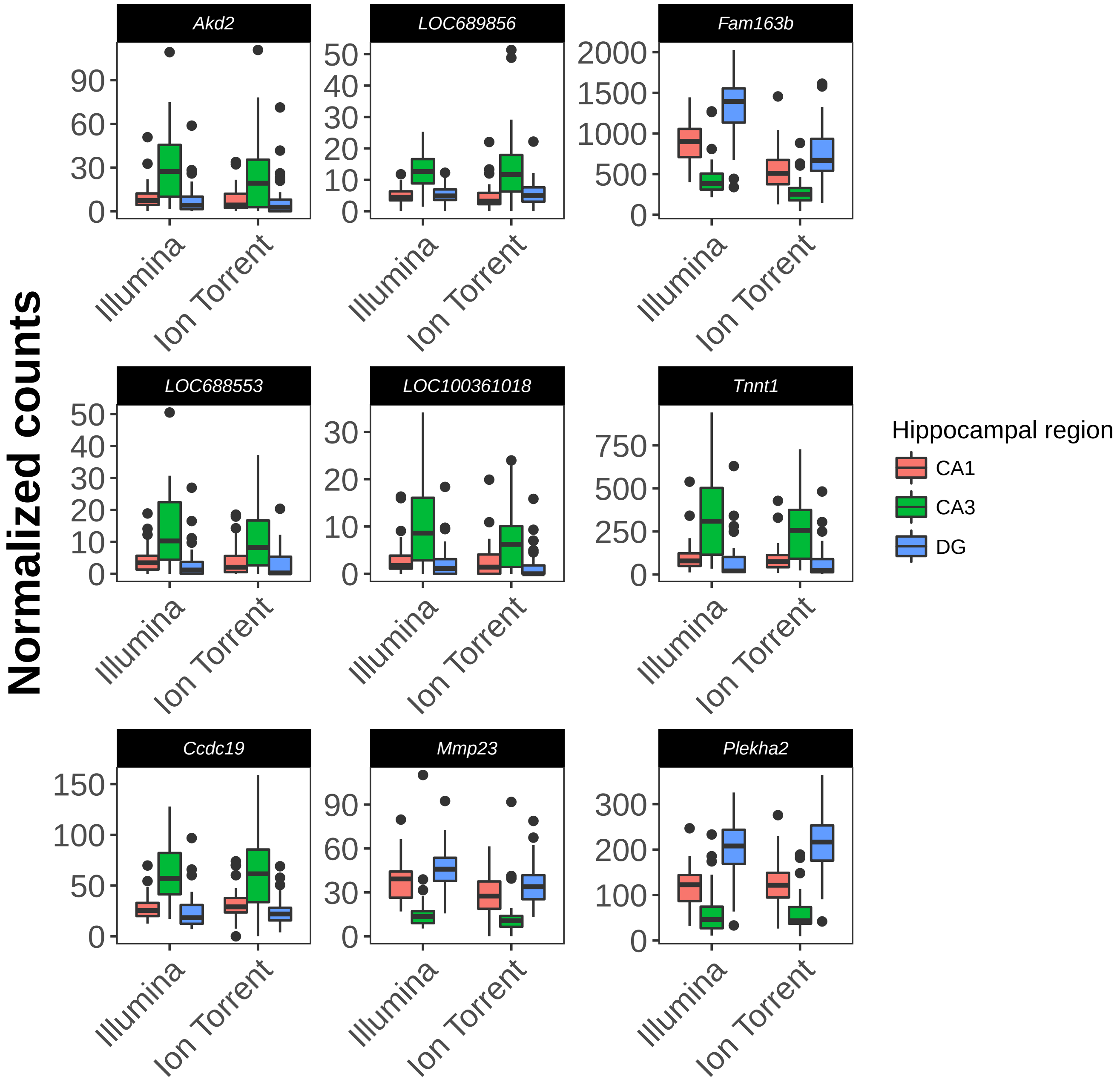

Normalized counts
Akd2
LOC689856
Fam163b
LOC688553
LOC100361018
Tnnt1
Ccdc19
Mmp23
Plekha2
Illumina
Ion Torrent
Hippocampal region
CA1
CA3
DG

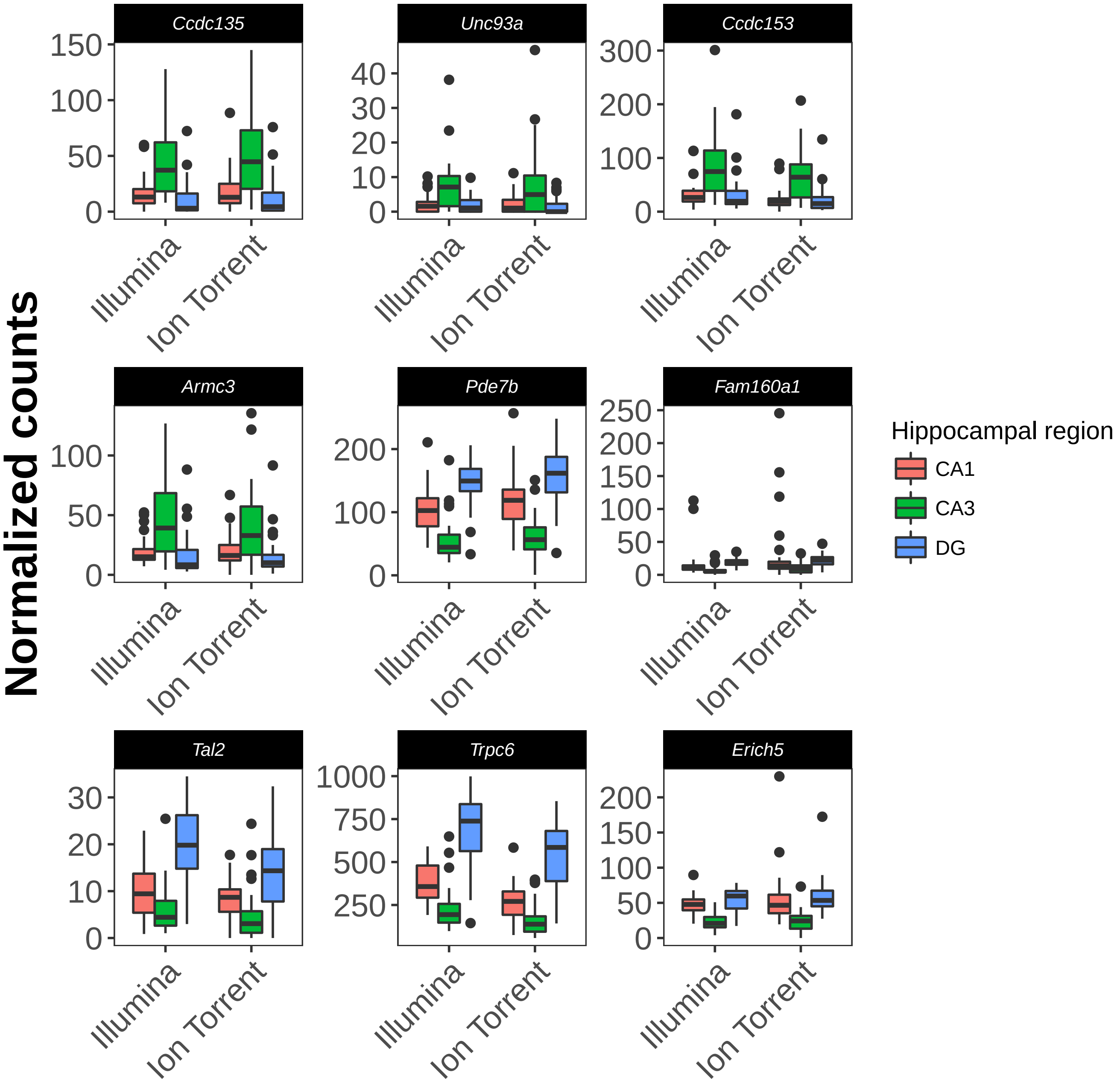

Normalized counts
Ccdc135
Unc93a
Ccdc153
Armc3
Pde7b
Fam160a1
Tal2
Trpc6
Erich5
Illumina
Ion Torrent
Hippocampal region
CA1
CA3
DG

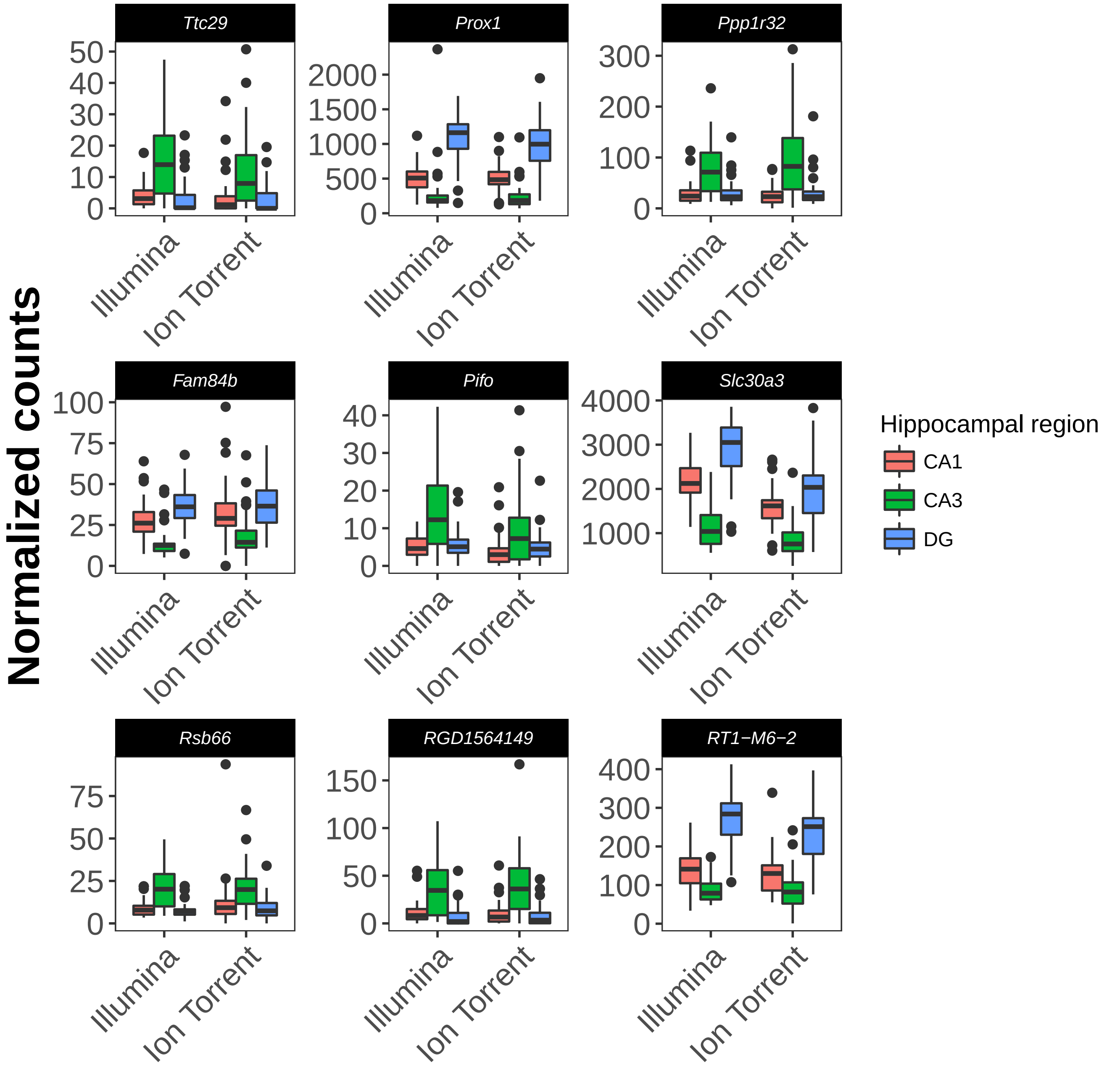

Normalized counts
Ttc29
Prox1
Ppp1r32
Fam84b
Pifo
Slc30a3
Rsb66
RGD1564149
RT1−M6−2
Illumina
Ion Torrent
Hippocampal region
CA1
CA3
DG

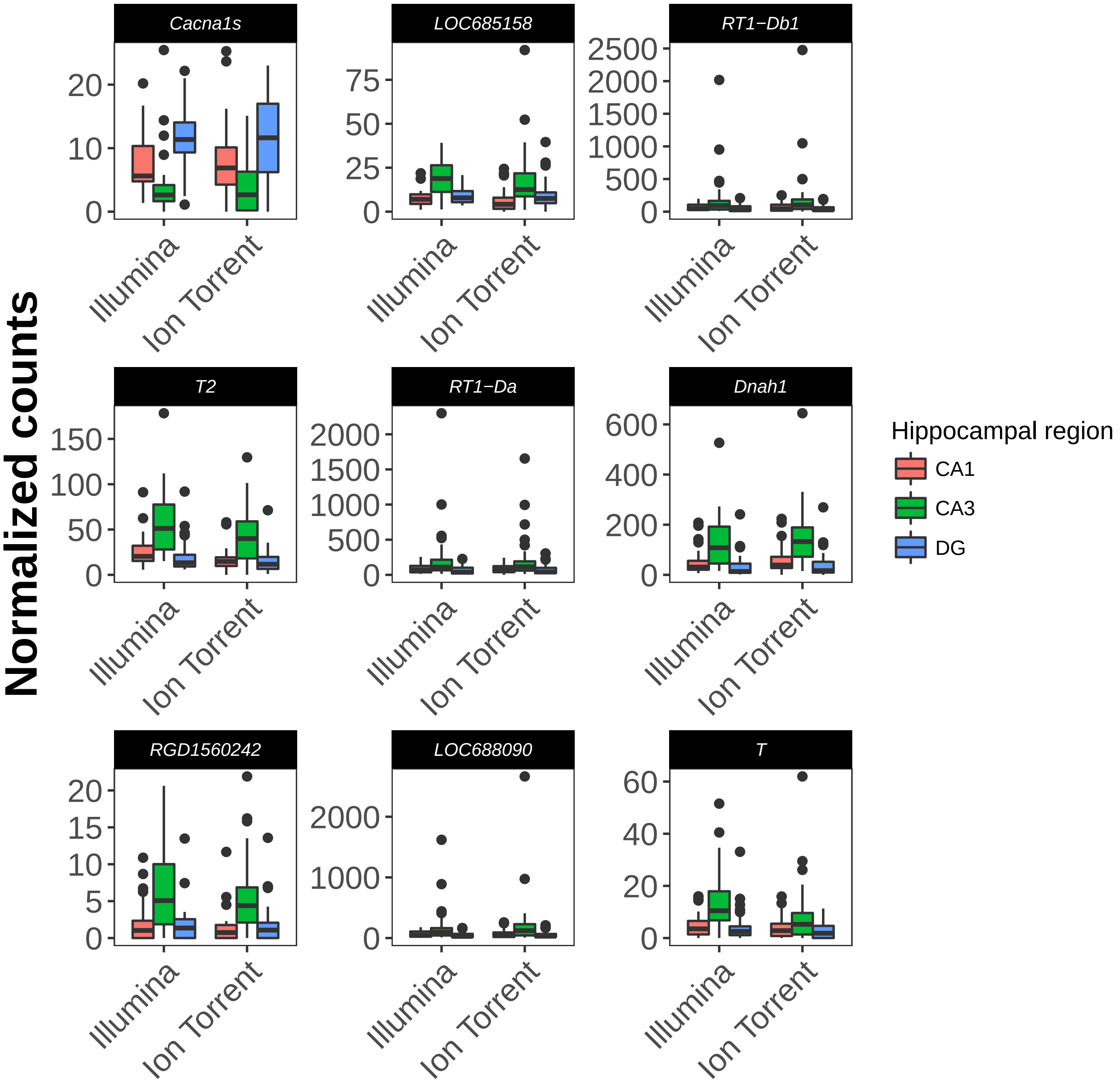

Normalized counts
Cacna1s
LOC685158
RT1−Db1
T2
RT1−Da
Dnah1
RGD1560242
LOC688090
T
Illumina
Ion Torrent
Hippocampal region
CA1
CA3
DG

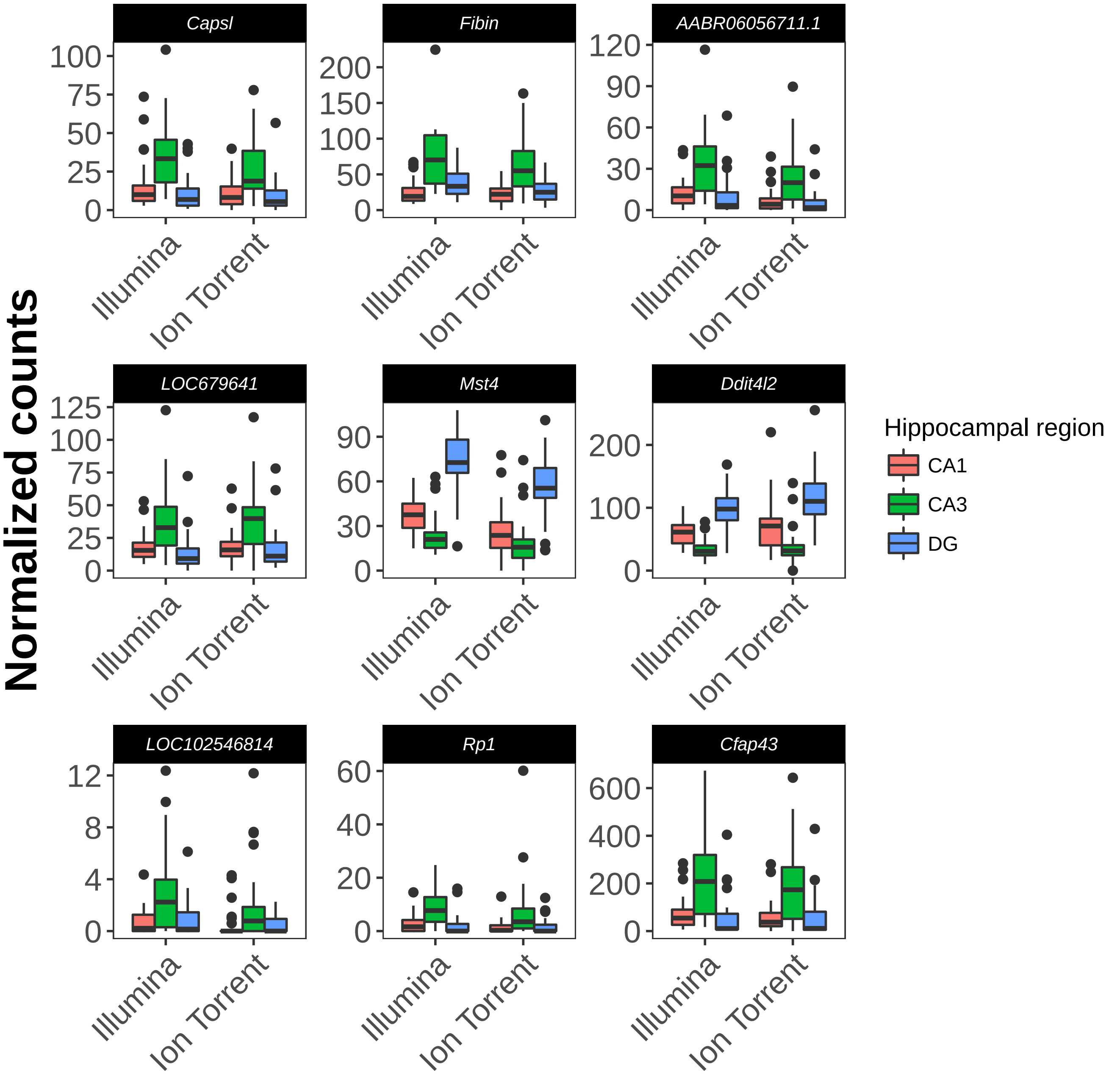
Normalized counts
Capsl
Fibin
AABR06056711.1
LOC679641
Mst4
Ddit4l2
LOC102546814
Rp1
Cfap43
Illumina
Ion Torrent
Hippocampal region
CA1
CA3
DG

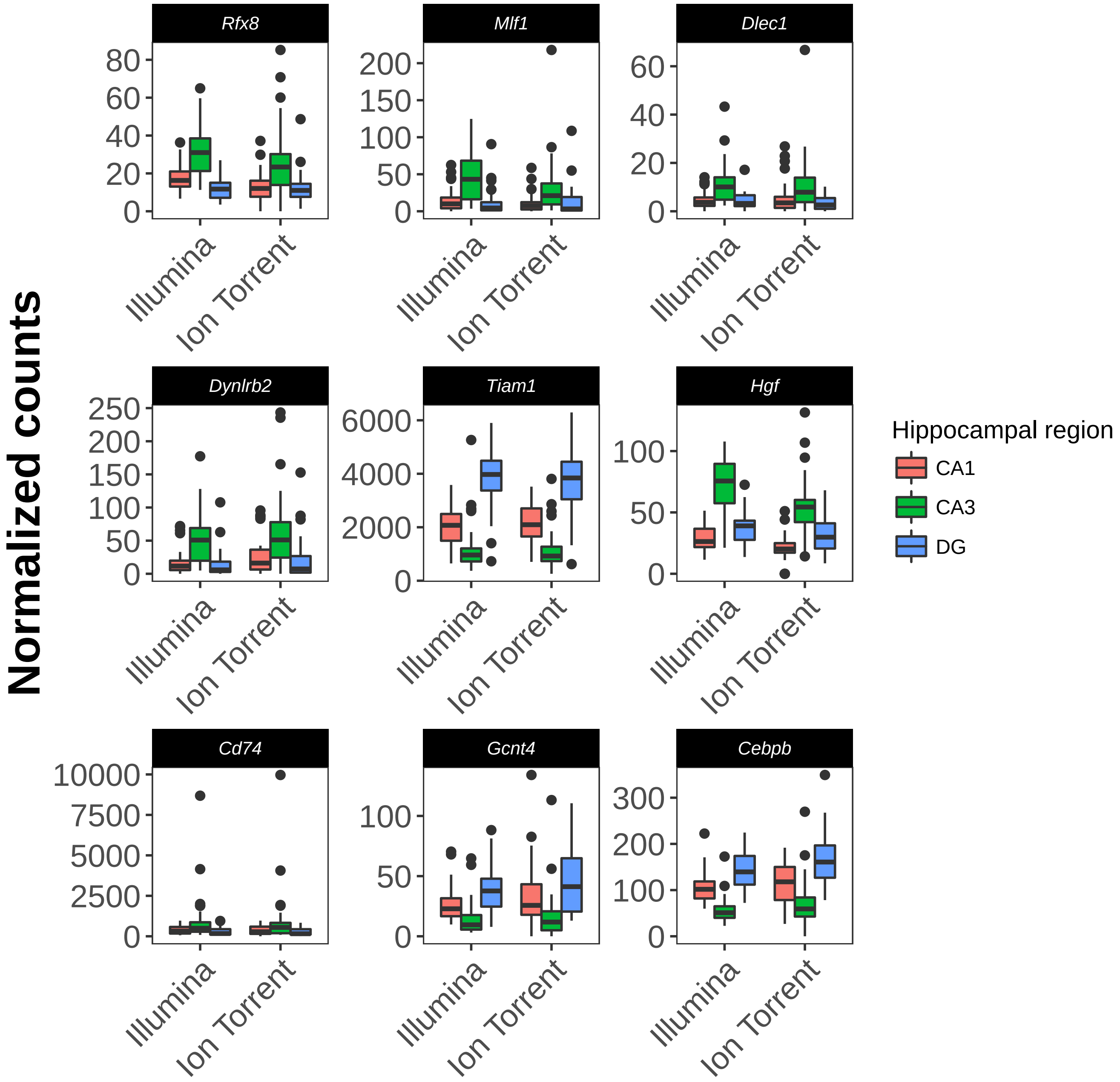

Normalized counts
Rfx8
Mlf1
Dlec1
Dynlrb2
Tiam1
Hgf
Cd74
Gcnt4
Cebpb
Illumina
Ion Torrent
Hippocampal region
CA1
CA3
DG

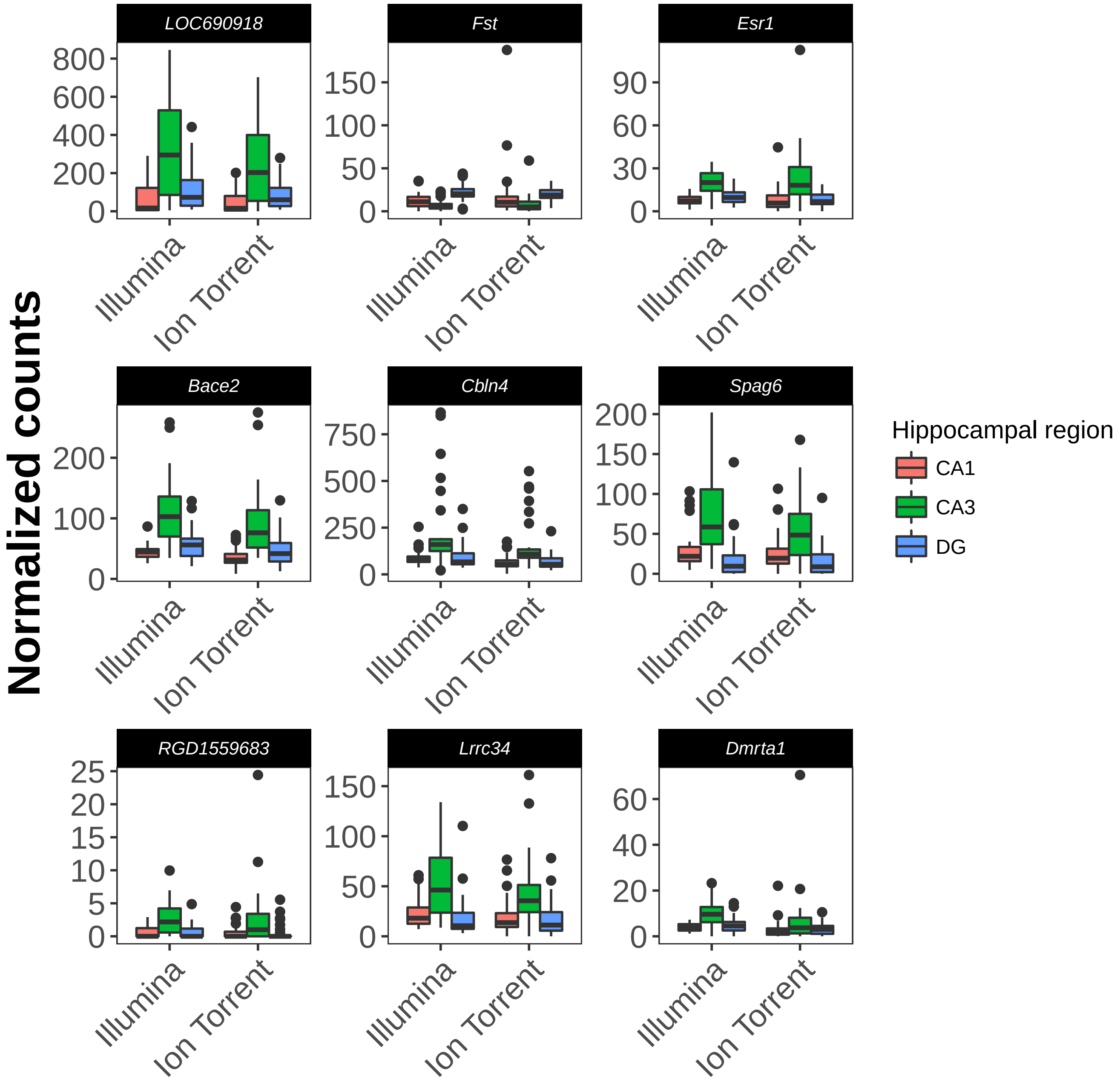

Normalized counts
LOC690918
Fst
Esr1
Bace2
Cbln4
Spag6
RGD1559683
Lrrc34
Dmrta1
Illumina
Ion Torrent
Hippocampal region
CA1
CA3
DG

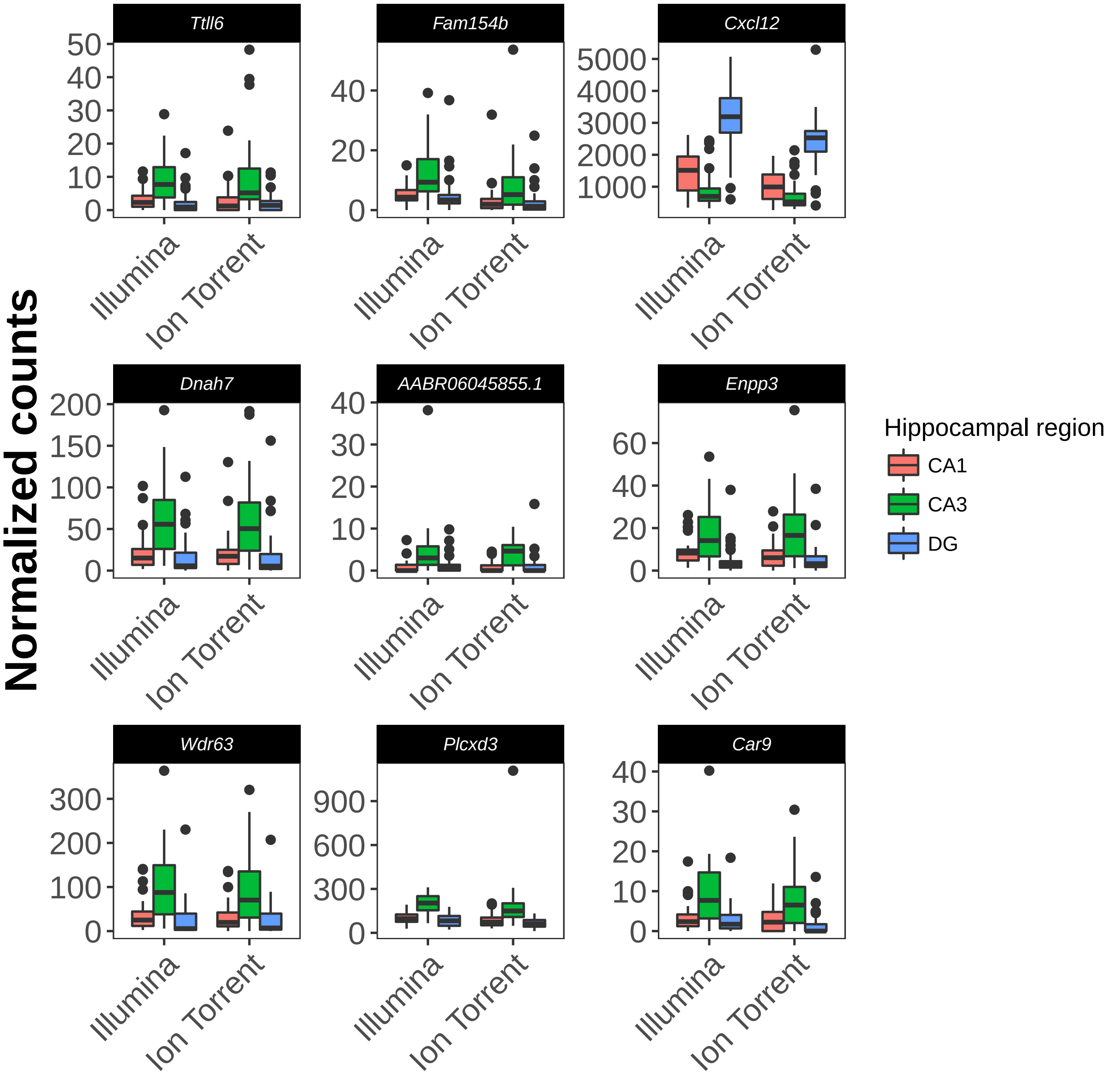

Normalized counts
Ttll6
Fam154b
Cxcl12
Dnah7
AABR06045855.1
Enpp3
Wdr63
Plcxd3
Car9
Illumina
Ion Torrent
Hippocampal region
CA1
CA3
DG

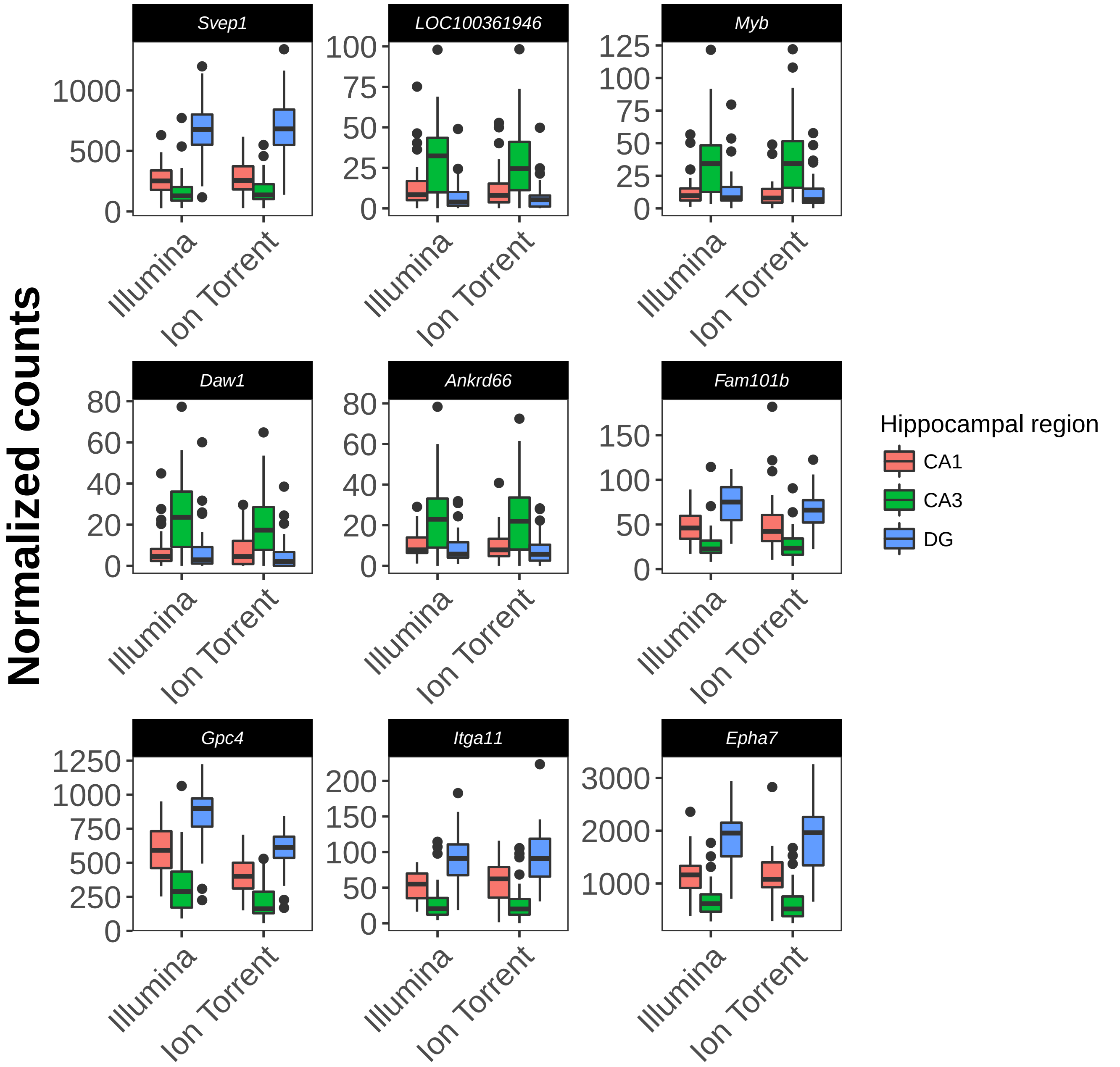

Normalized counts
Svep1
LOC100361946
Myb
Daw1
Ankrd66
Fam101b
Gpc4
Itga11
Epha7
Illumina
Ion Torrent
Hippocampal region
CA1
CA3
DG

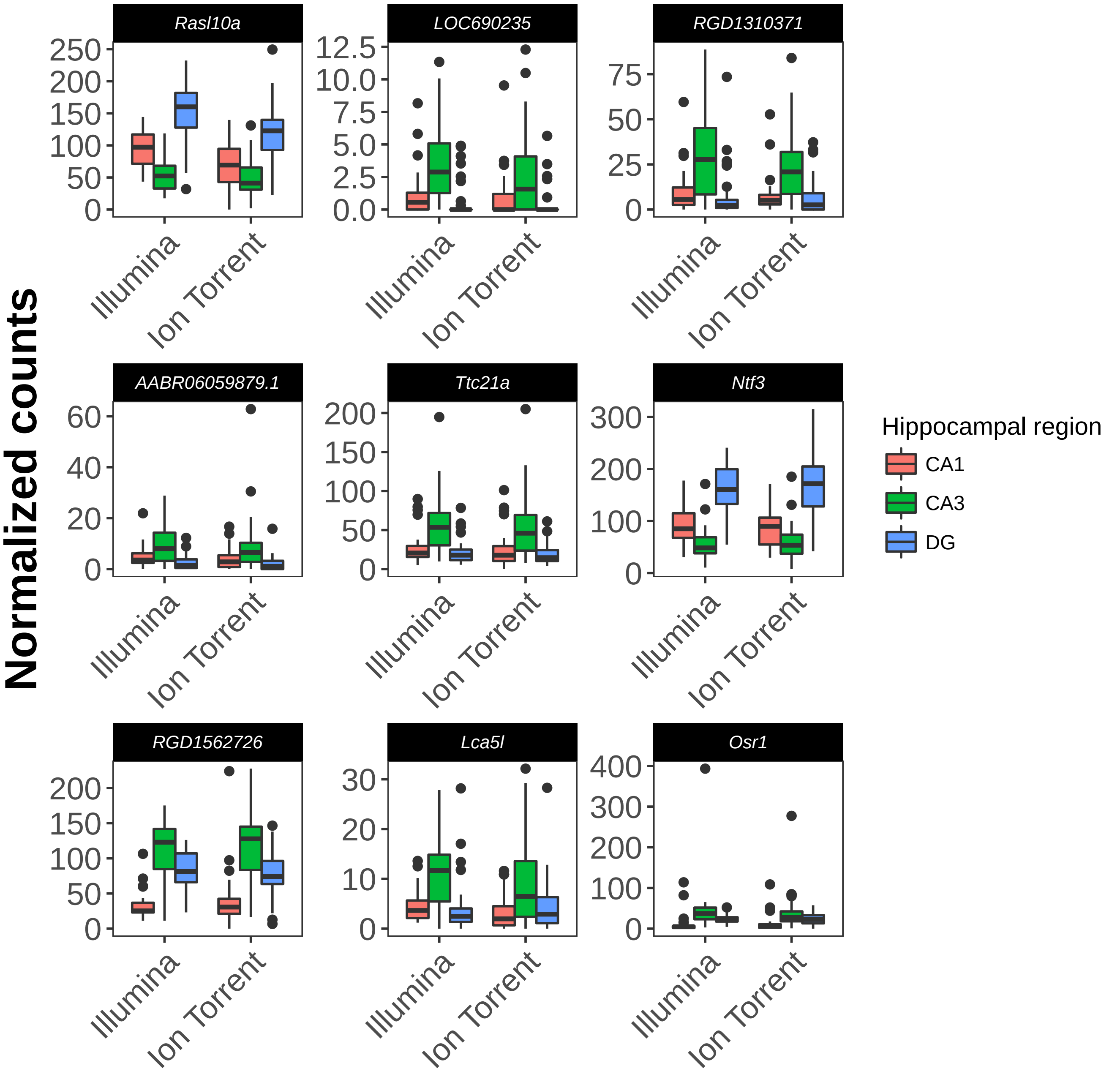
Normalized counts
Rasl10a
LOC690235
RGD1310371
AABR06059879.1
Ttc21a
Ntf3
RGD1562726
Lca5l
Osr1
Illumina
Ion Torrent
Hippocampal region
CA1
CA3
DG

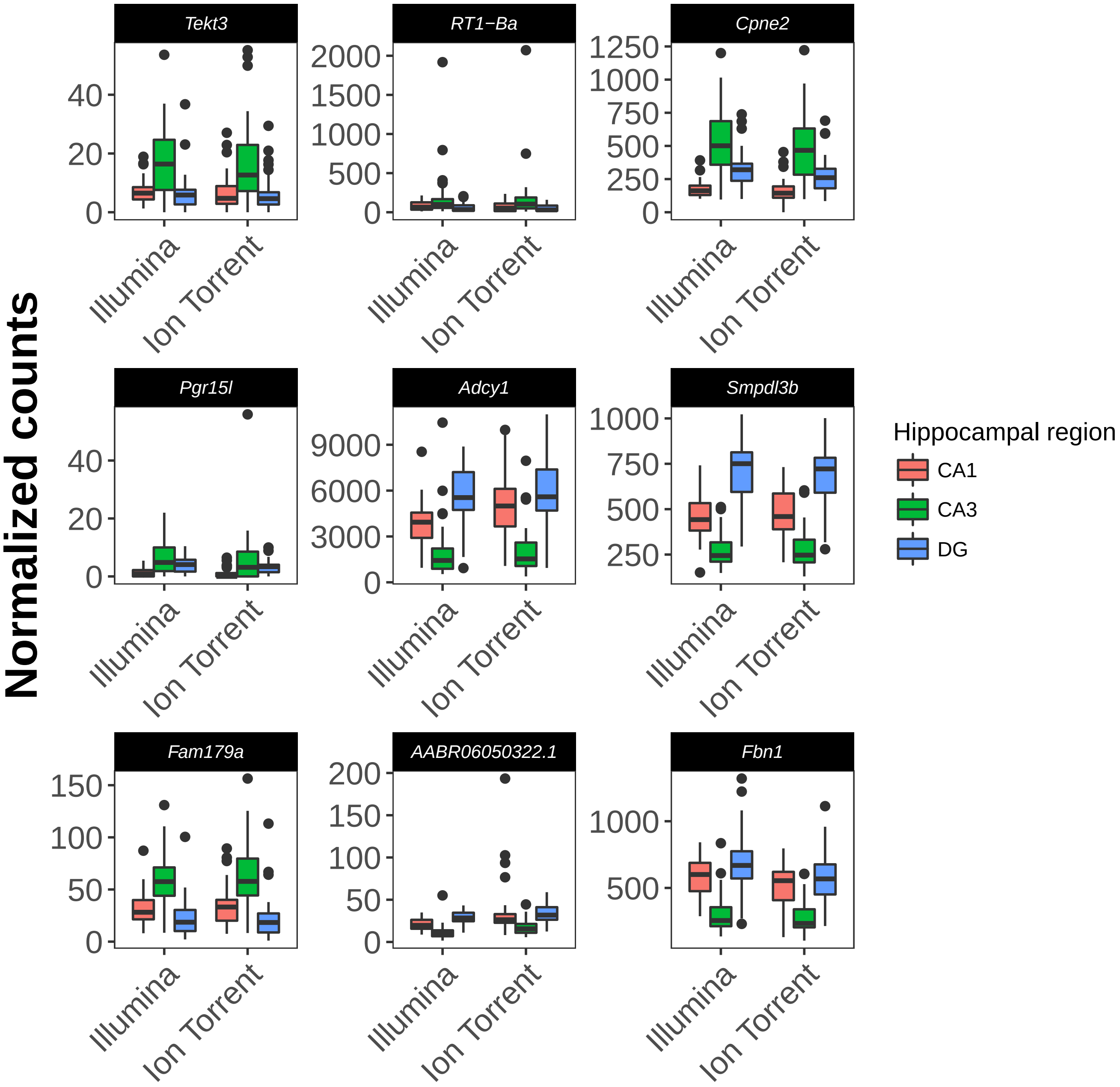

Normalized counts
Tekt3
RT1−Ba
Cpne2
Pgr15l
Adcy1
Smpdl3b
Fam179a
AABR06050322.1
Fbn1
Illumina
Ion Torrent
Hippocampal region
CA1
CA3
DG

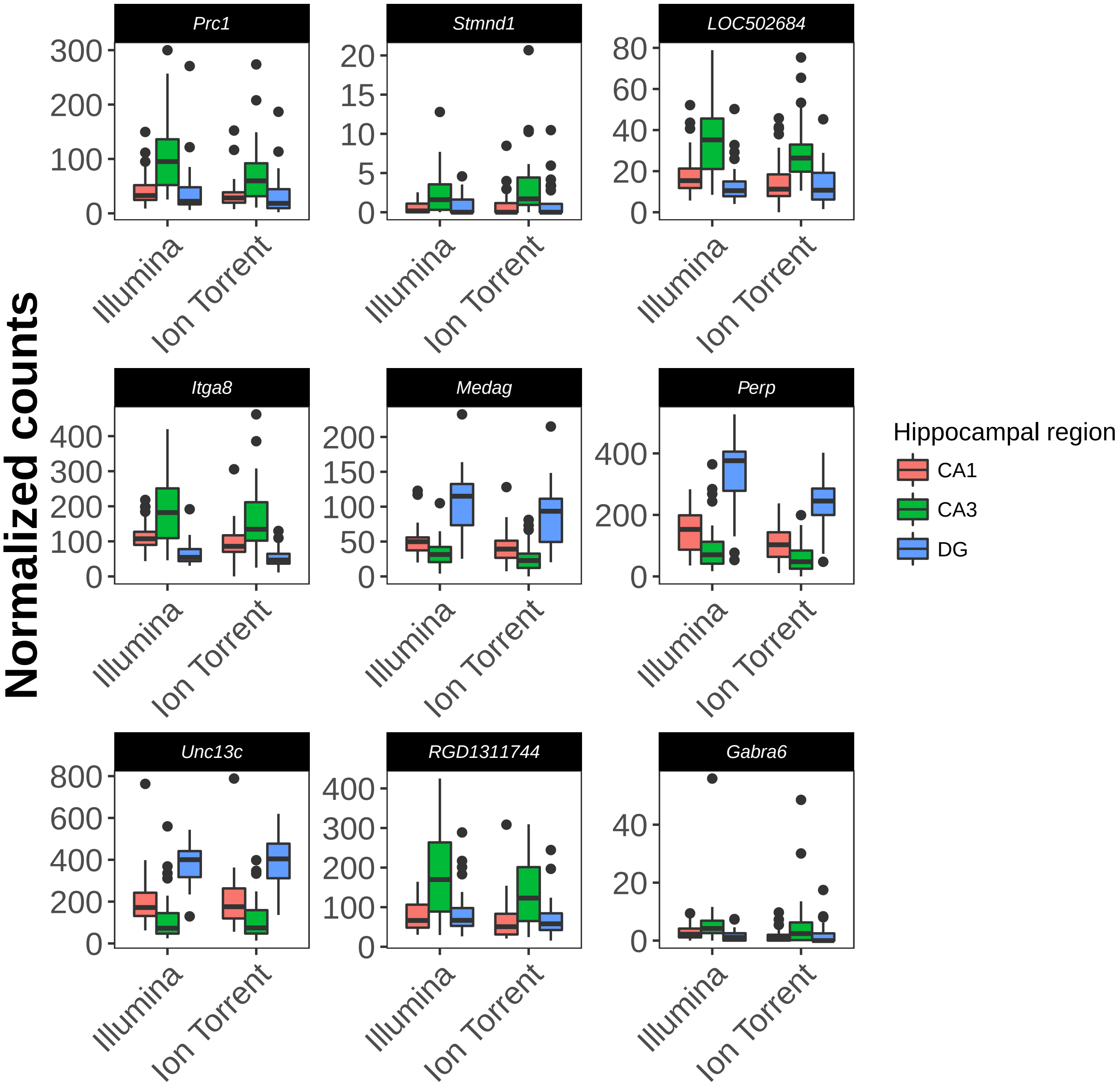

Prc1
Stmnd1
LOC502684
Itga8
Medag
Perp
Unc13c
RGD1311744
Gabra6
Normalized counts
Illumina
Ion Torrent
Hippocampal region
CA1
CA3
DG

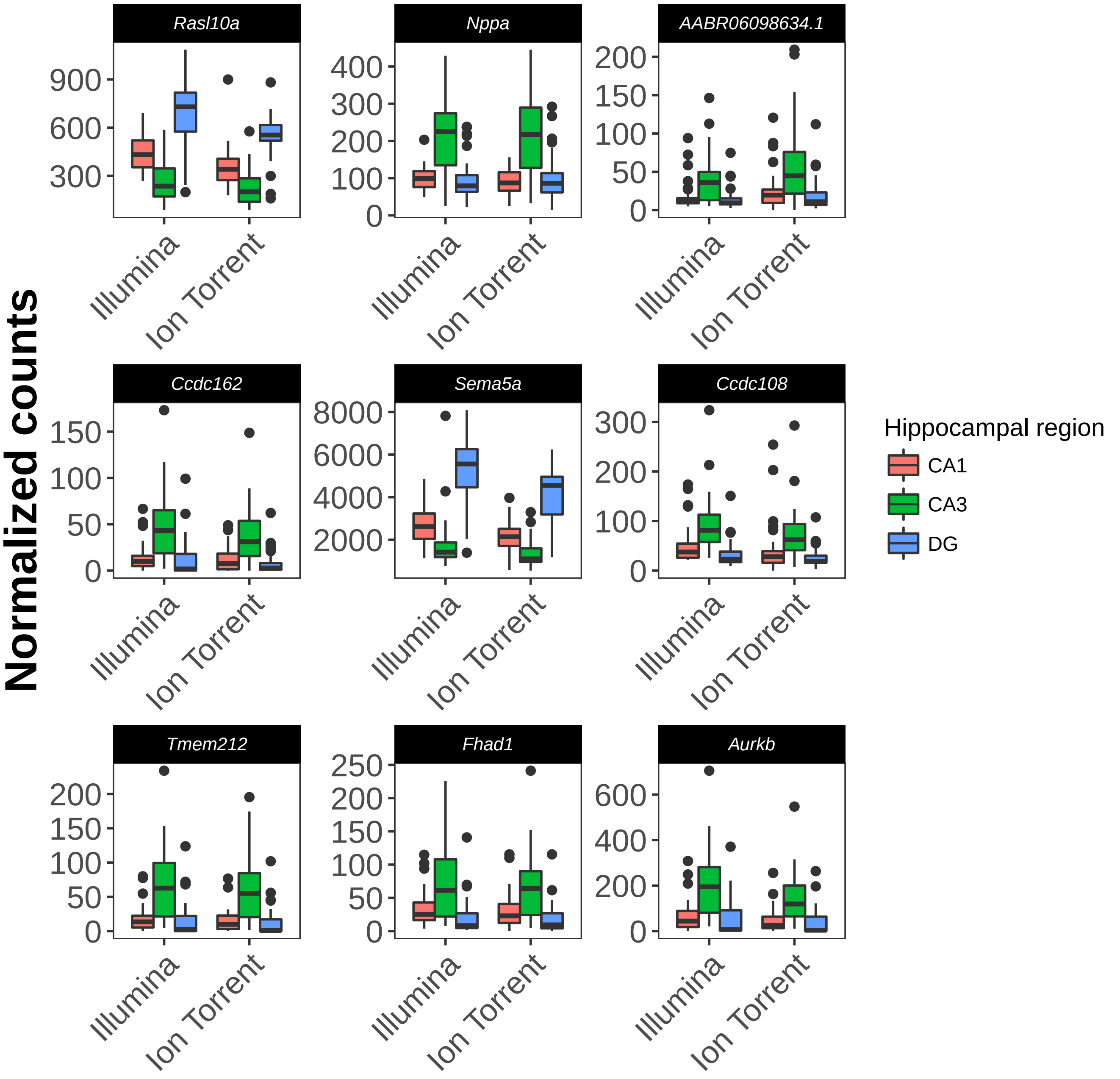
Rasl10a
Nppa
AABR06098634.1
Ccdc162
Sema5a
Ccdc108
Tmem212
Fhad1
Aurkb
Normalized counts
Illumina
Ion Torrent
Hippocampal region
CA1
CA3
DG

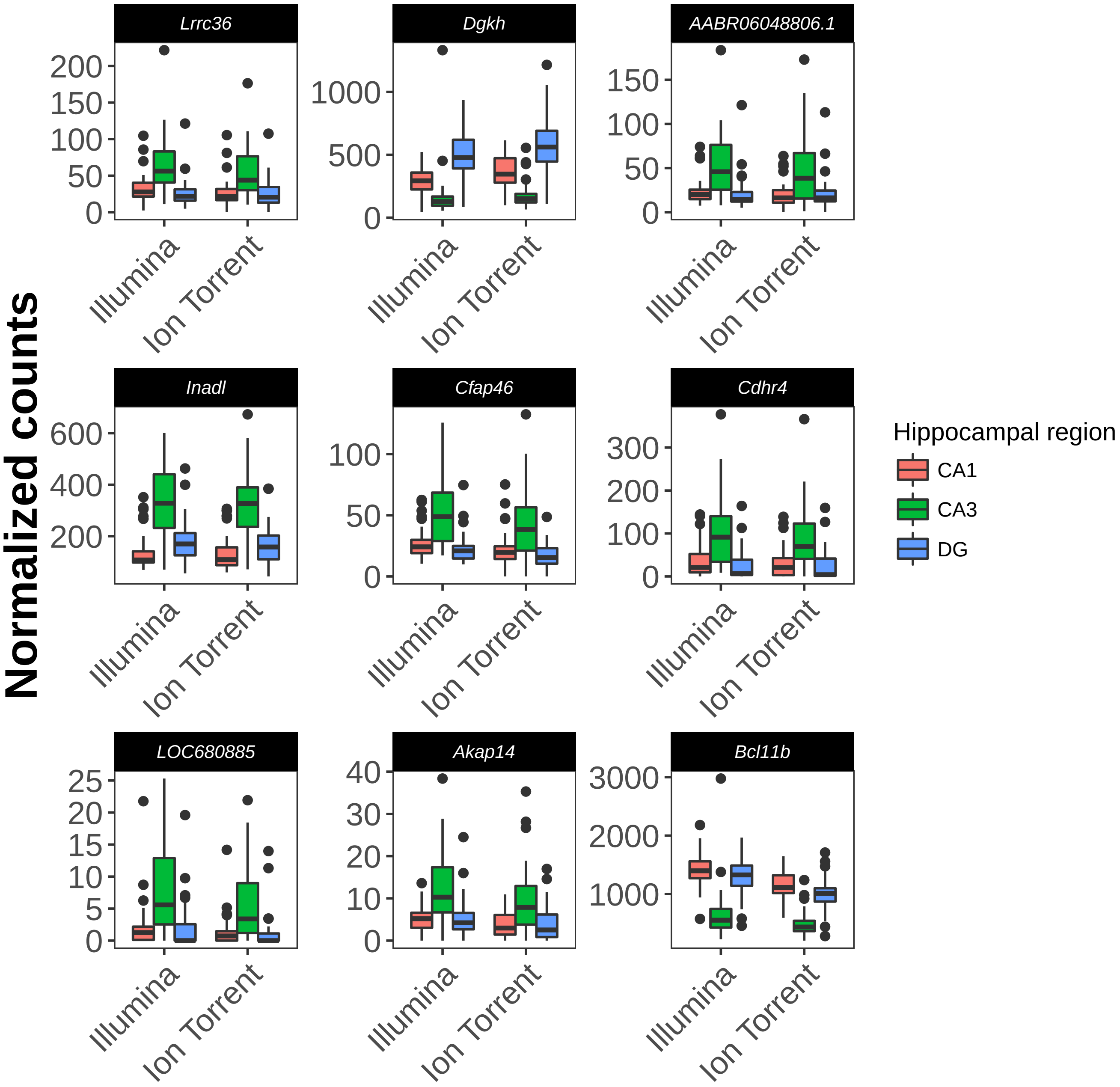

Normalized counts
Lrrc36
Dgkh
AABR06048806.1
Inadl
Cfap46
Cdhr4
LOC680885
Akap14
Bcl11b
Illumina
Ion Torrent
Hippocampal region
CA1
CA3
DG

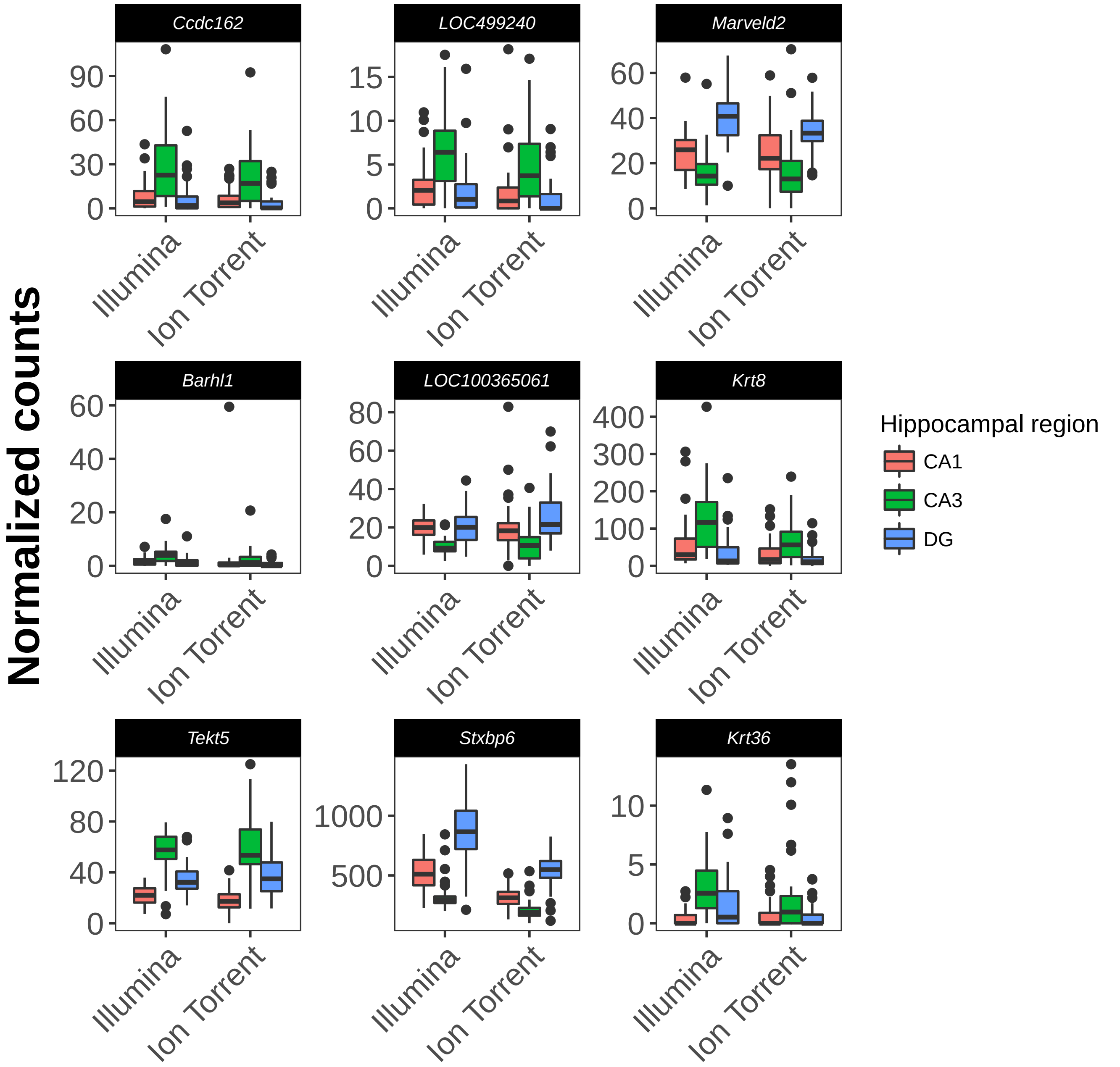
Normalized counts
Ccdc162
LOC499240
Marveld2
Barhl1
LOC100365061
Krt8
Tekt5
Stxbp6
Krt36
Illumina
Ion Torrent
Hippocampal region
CA1
CA3
DG

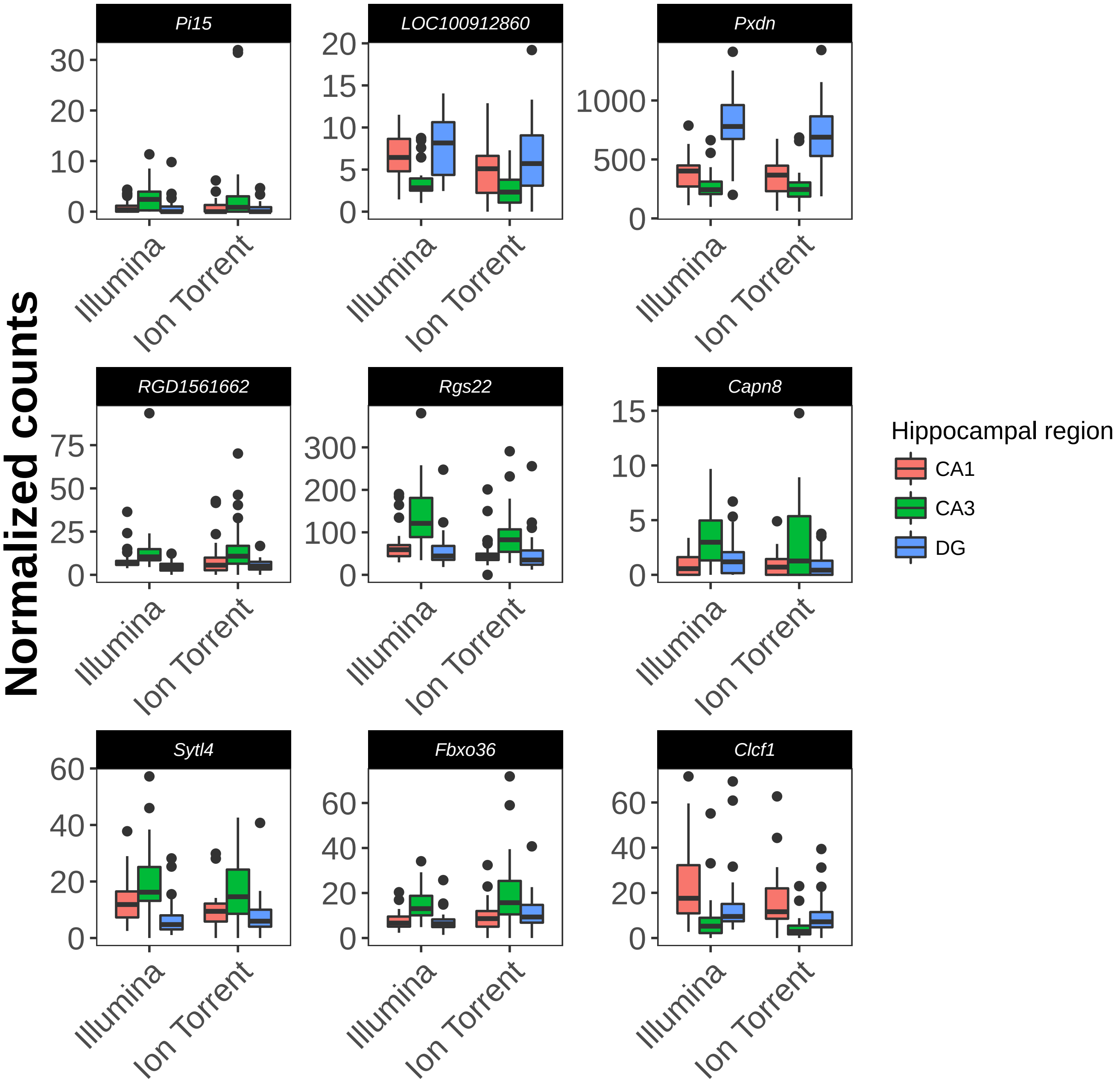

Normalized counts
Pi15
LOC100912860
Pxdn
RGD1561662
Rgs22
Capn8
Sytl4
Fbxo36
Clcf1
Illumina
Ion Torrent
Hippocampal region
CA1
CA3
DG

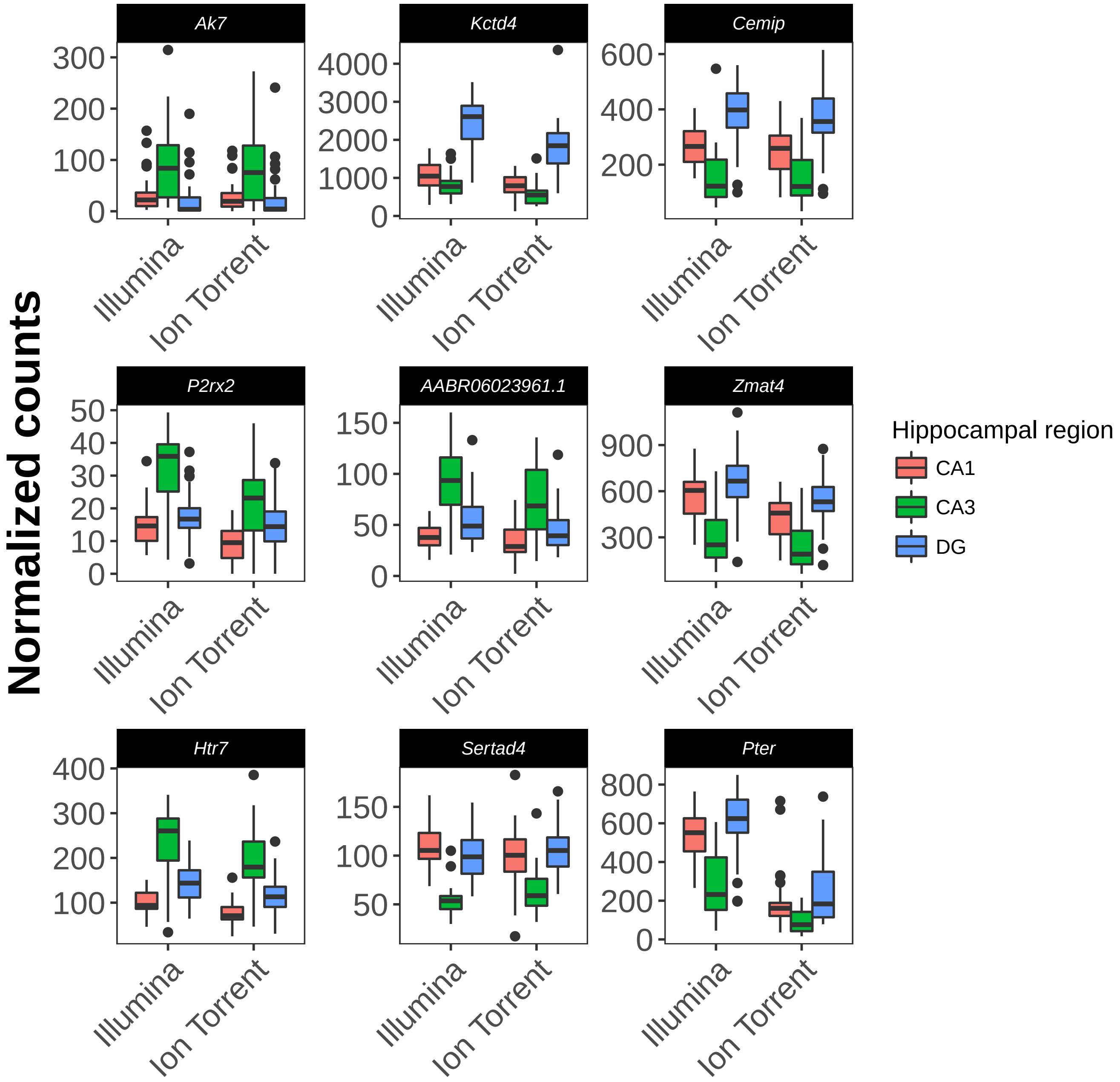
Normalized counts
Ak7
Kctd4
Cemip
P2rx2
AABR06023961.1
Zmat4
Htr7
Sertad4
Pter
Illumina
Ion Torrent
Hippocampal region
CA1
CA3
DG

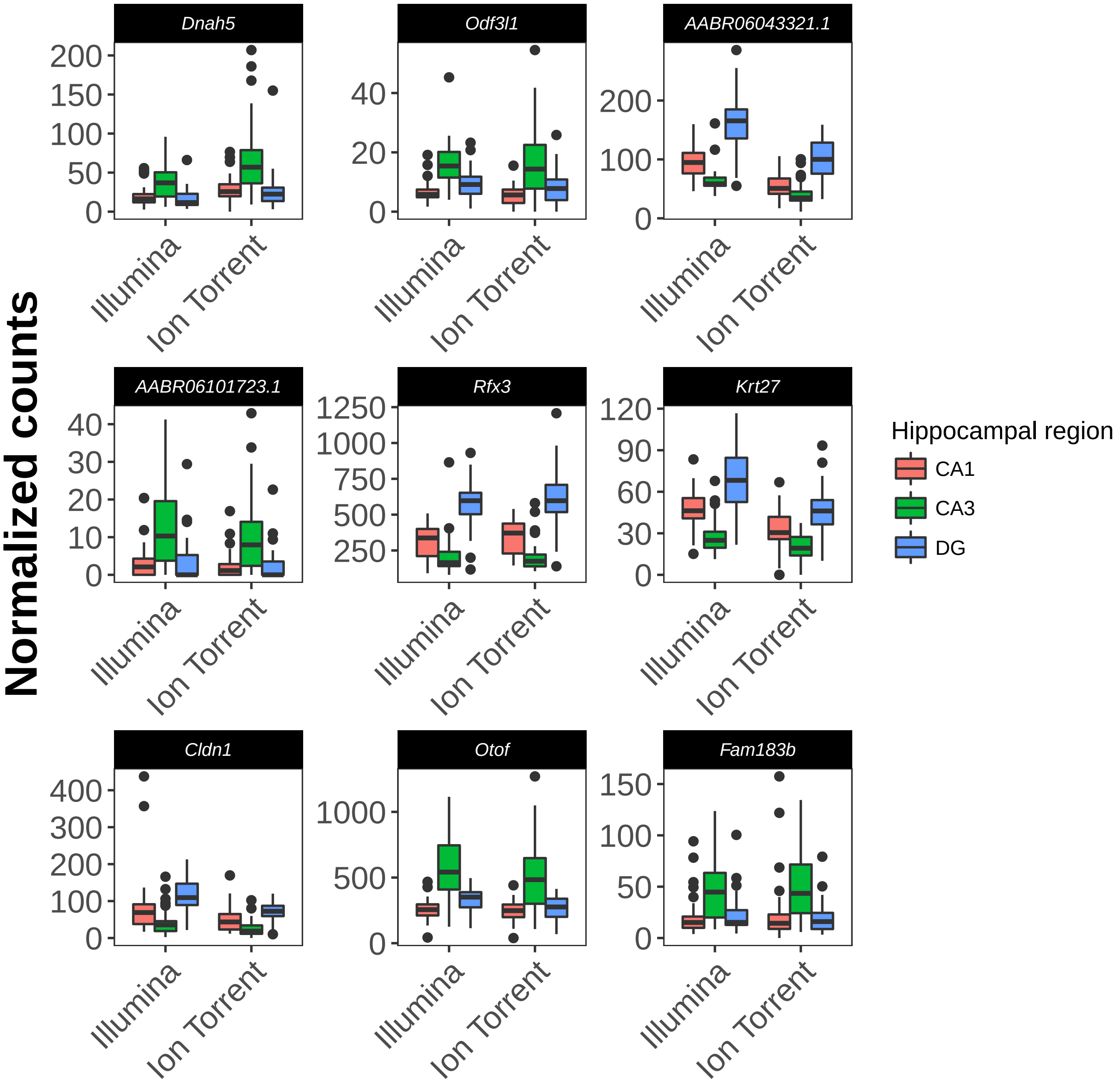
Normalized counts
Dnah5
Odf3l1
AABR06043321.1
AABR06101723.1
Rfx3
Krt27
Cldn1
Otof
Fam183b
Illumina
Ion Torrent
Hippocampal region
CA1
CA3
DG

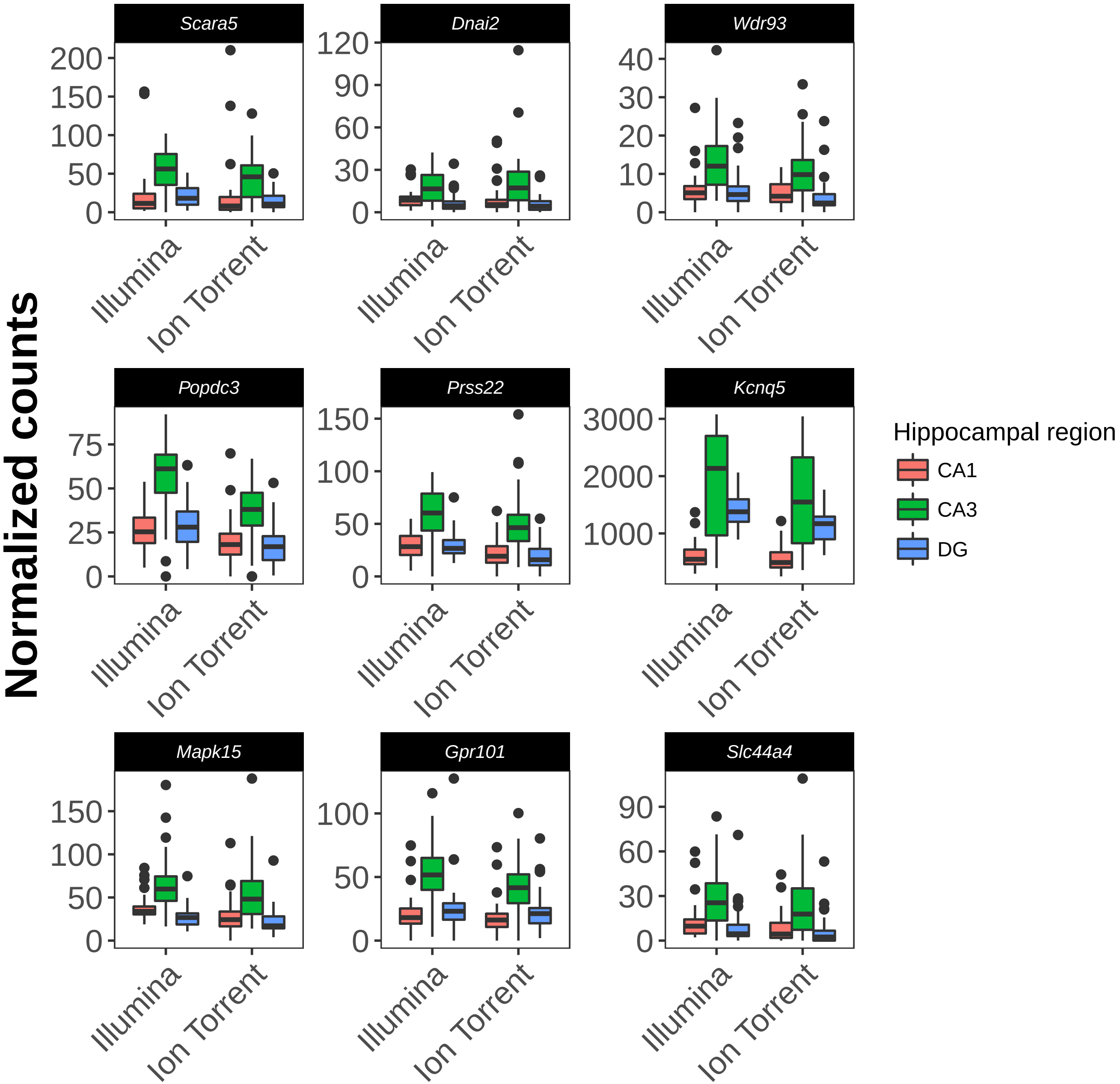
Normalized counts
Scara5
Dnai2
Wdr93
Popdc3
Prss22
Kcnq5
Mapk15
Gpr101
Slc44a4
Illumina
Ion Torrent
Hippocampal region
CA1
CA3
DG

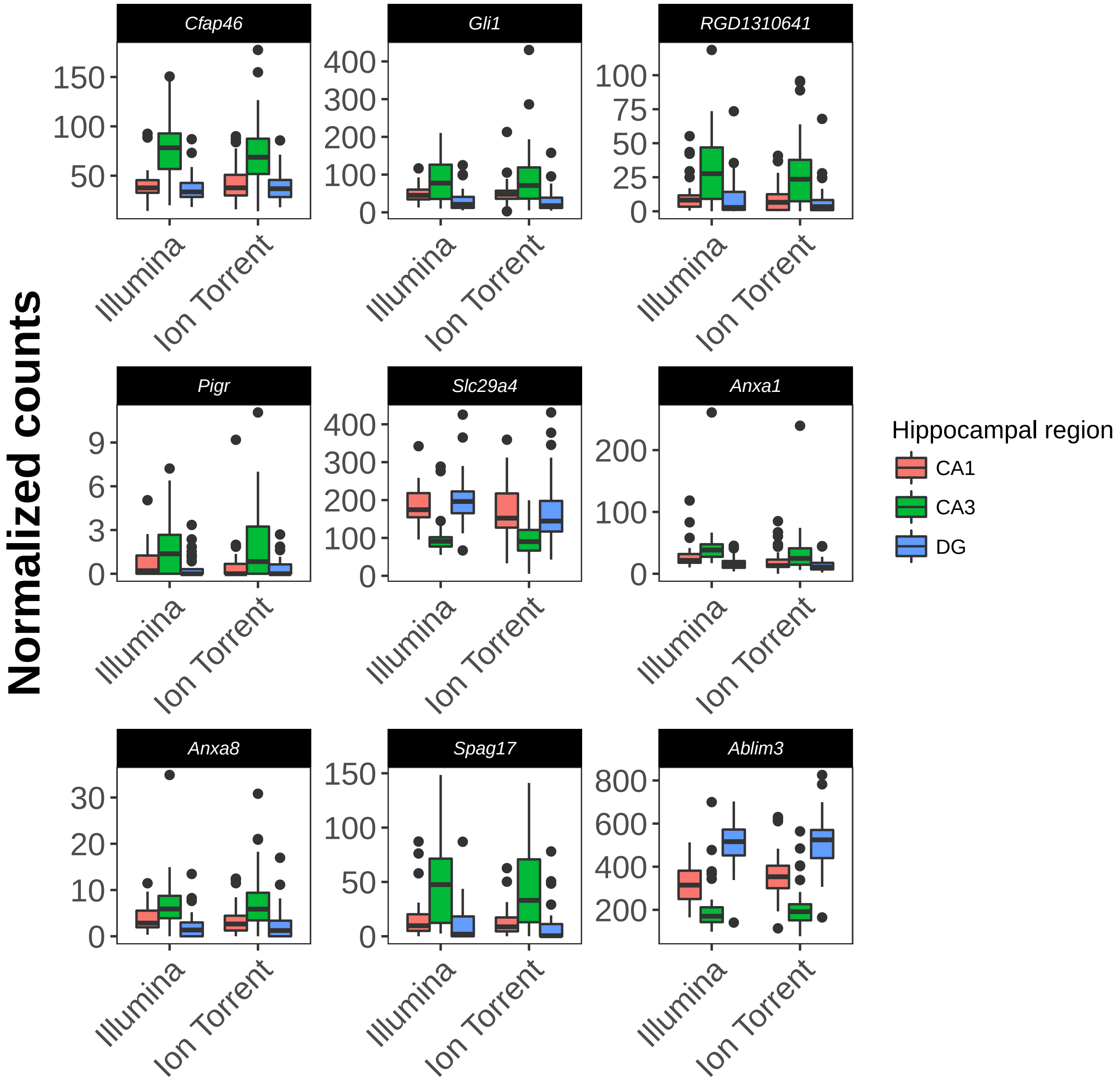

Normalized counts
Cfap46
Gli1
RGD1310641
Pigr
Slc29a4
Anxa1
Anxa8
Spag17
Ablim3
Illumina
Ion Torrent
Hippocampal region
CA1
CA3
DG

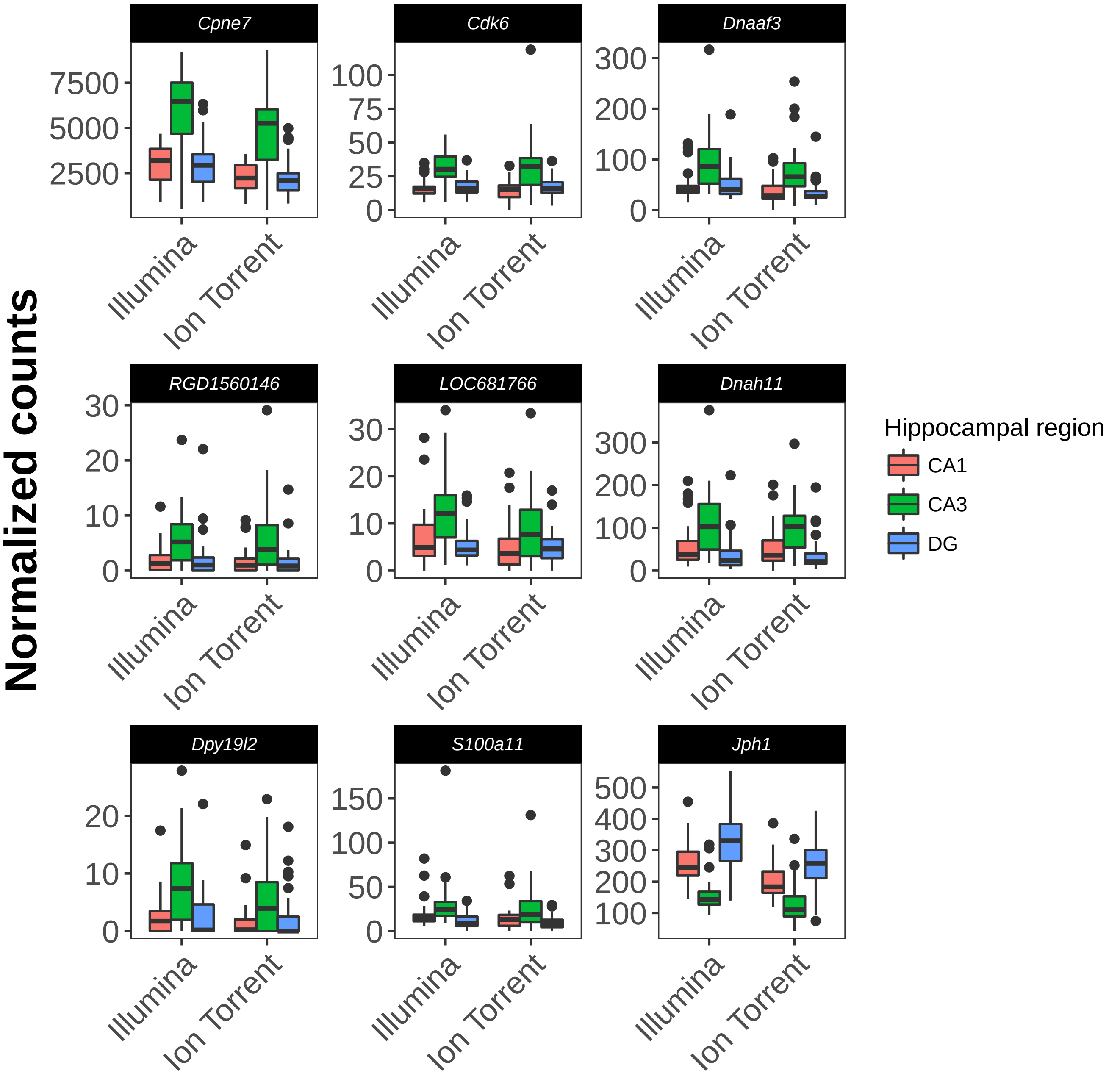

Normalized counts
Cpne7
Cdk6
Dnaaf3
RGD1560146
LOC681766
Dnah11
Dpy19l2
S100a11
Jph1
Illumina
Ion Torrent
Hippocampal region
CA1
CA3
DG

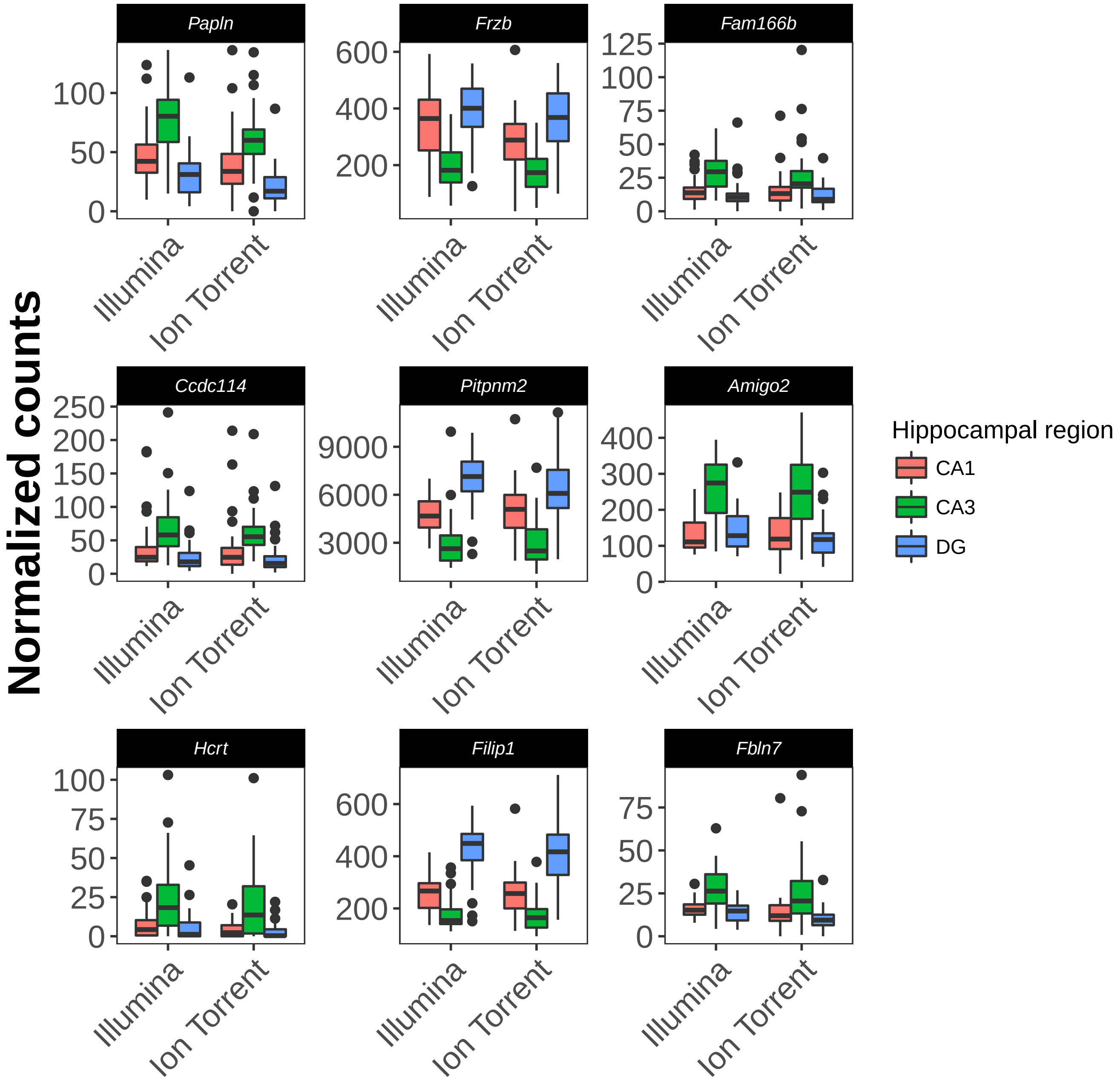

Normalized counts
Papln
Frzb
Fam166b
Ccdc114
Pitpnm2
Amigo2
Hcrt
Filip1
Fbln7
Illumina
Ion Torrent
Hippocampal region
CA1
CA3
DG

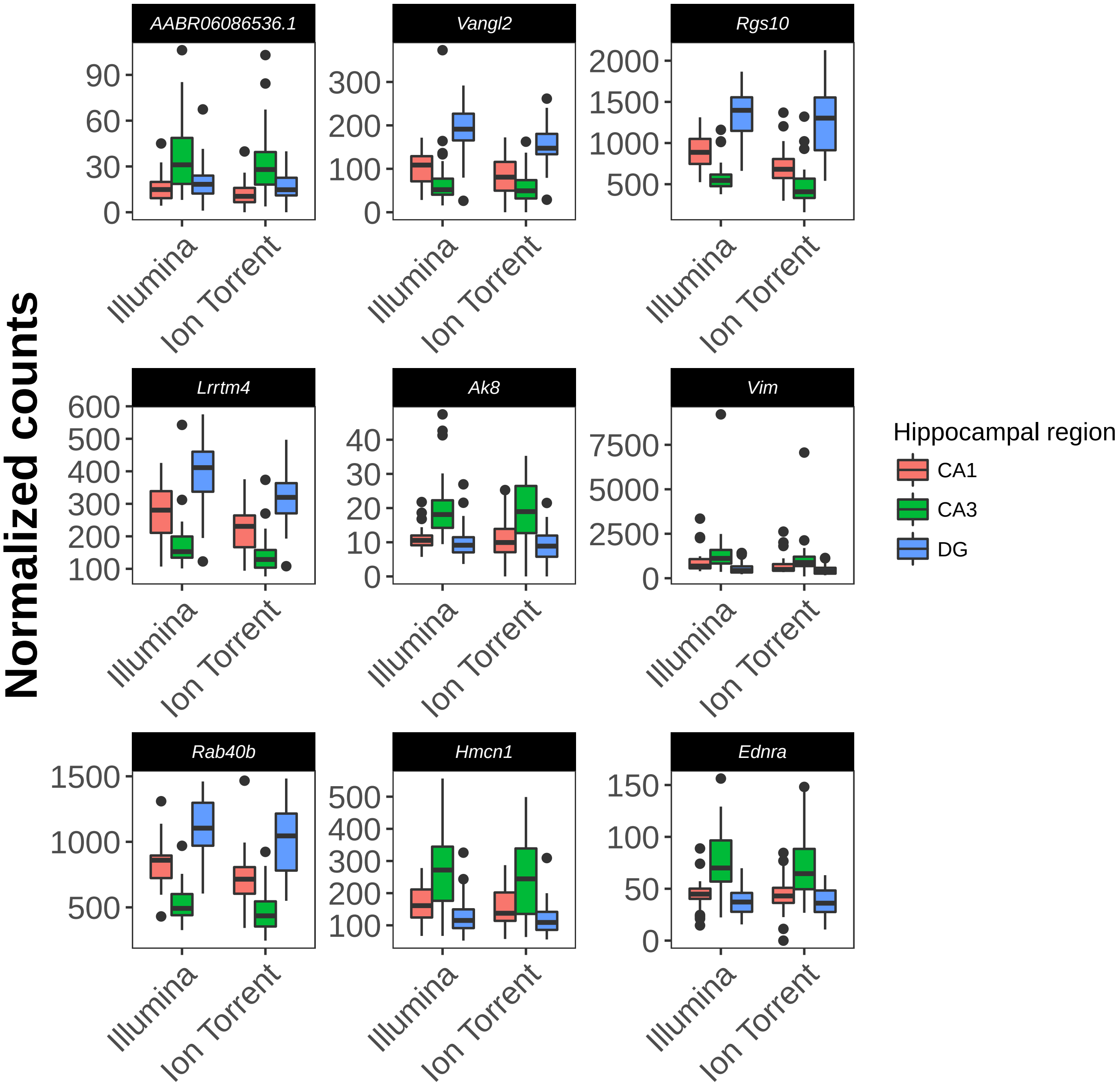

Normalized counts
AABR06086536.1
Vangl2
Rgs10
Lrrtm4
Ak8
Vim
Rab40b
Hmcn1
Ednra
Illumina
Ion Torrent
Hippocampal region
CA1
CA3
DG

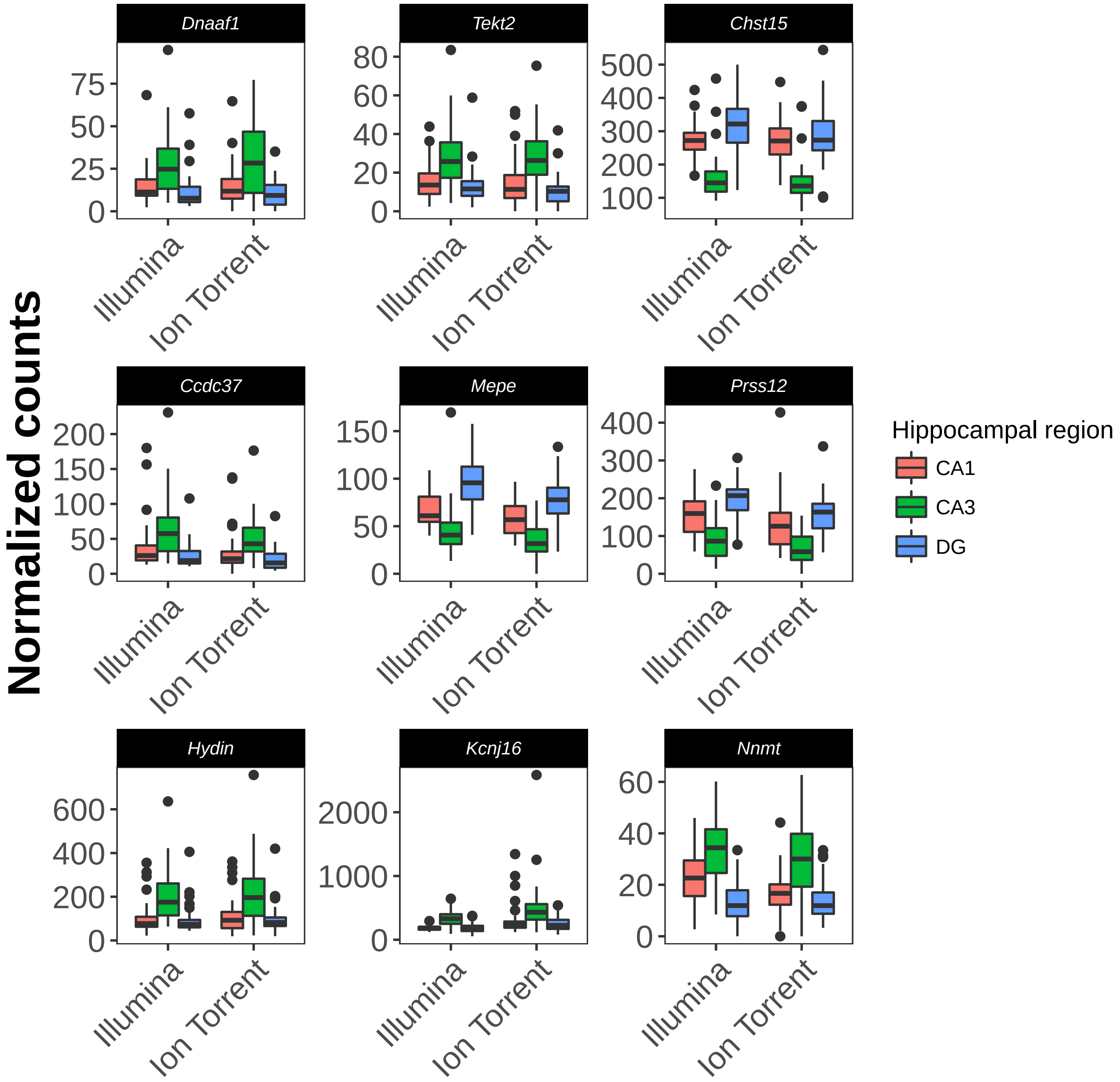
Normalized counts
Dnaaf1
Tekt2
Chst15
Ccdc37
Mepe
Prss12
Hydin
Kcnj16
Nnmt
Illumina
Ion Torrent
Hippocampal region
CA1
CA3
DG

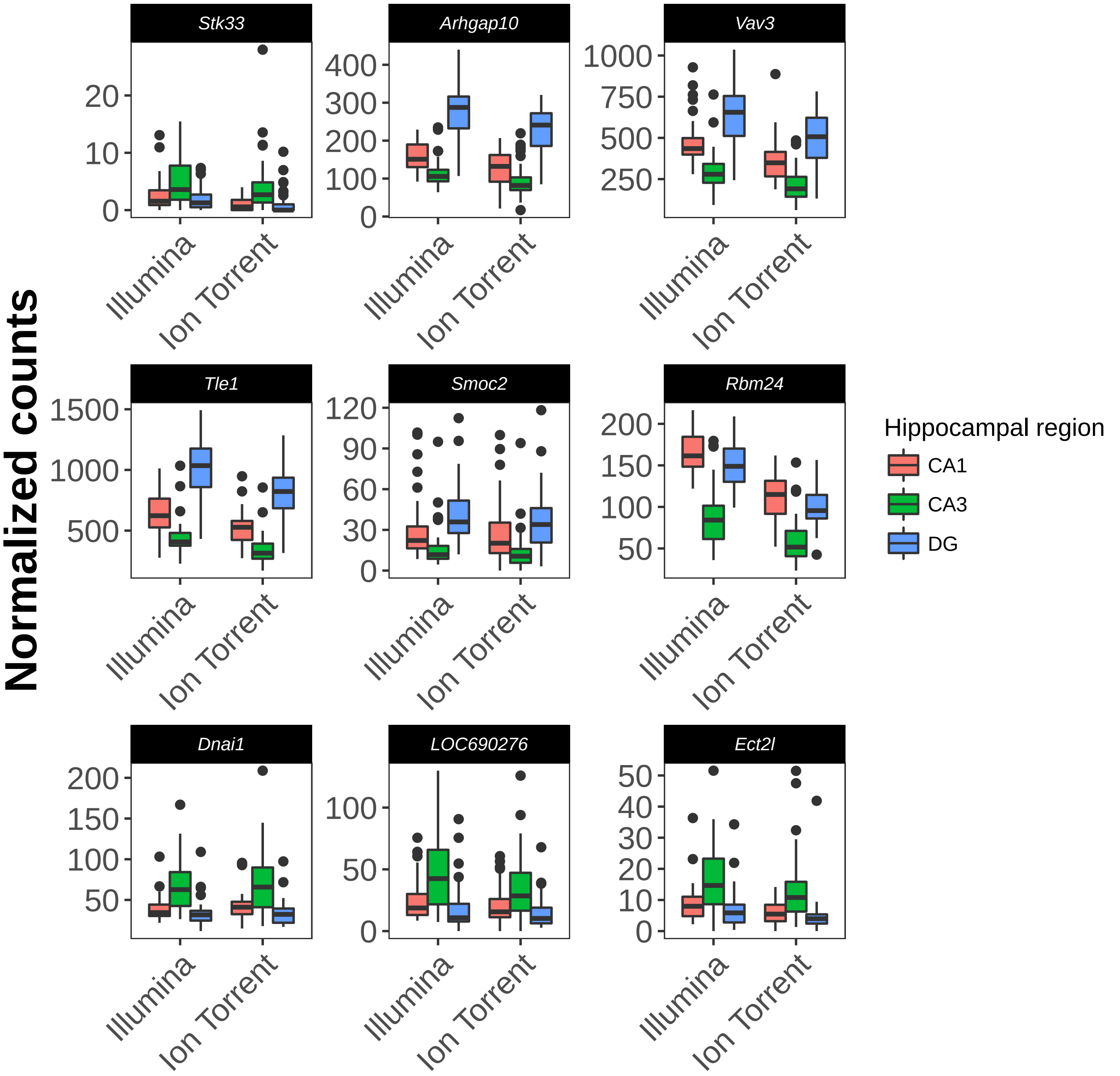

Normalized counts
Stk33
Arhgap10
Vav3
Tle1
Smoc2
Rbm24
Dnai1
LOC690276
Ect2l
Illumina
Ion Torrent
Hippocampal region
CA1
CA3
DG

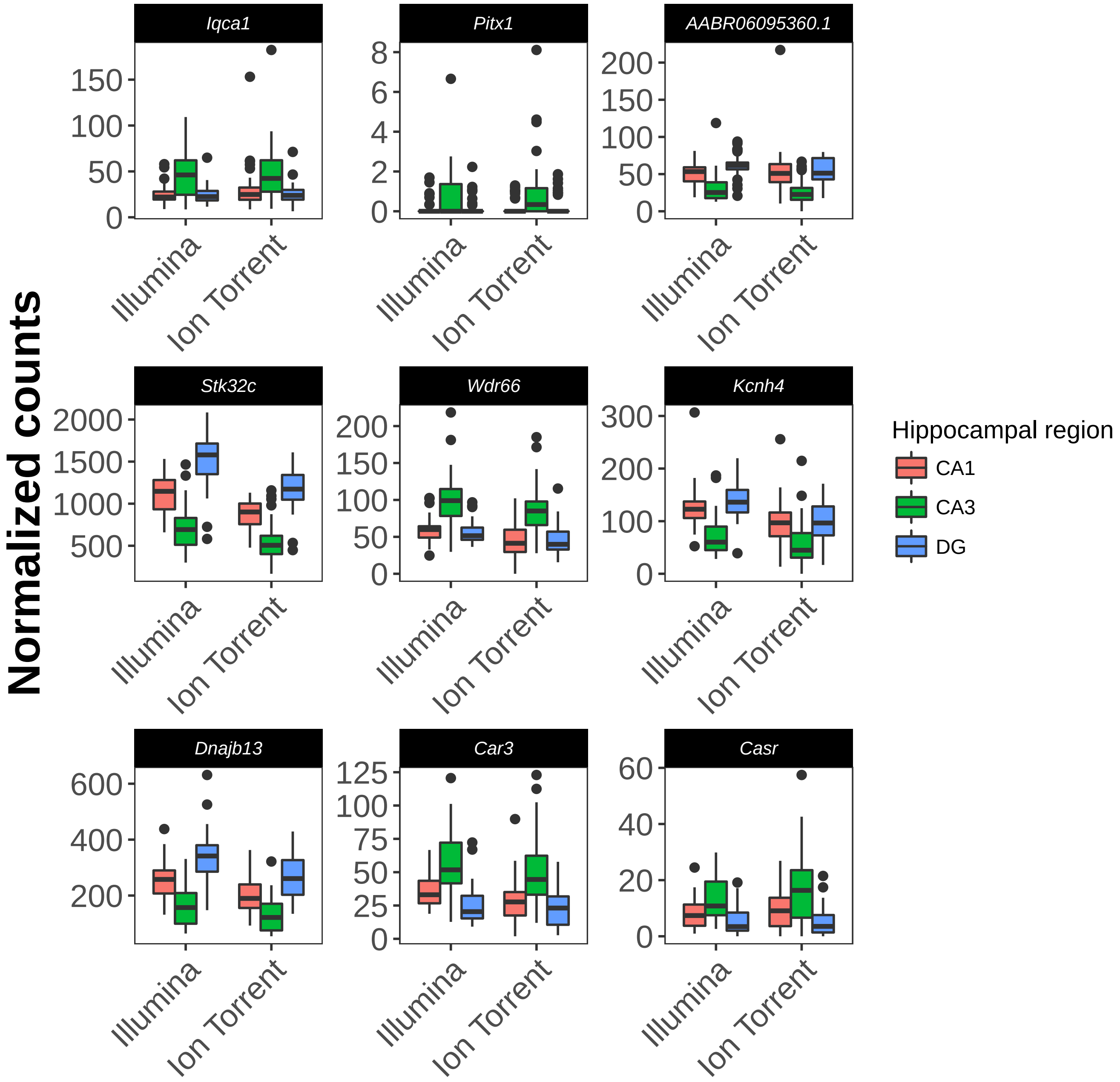

Normalized counts
Iqca1
Pitx1
AABR06095360.1
Stk32c
Wdr66
Kcnh4
Dnajb13
Car3
Casr
Illumina
Ion Torrent
Hippocampal region
CA1
CA3
DG

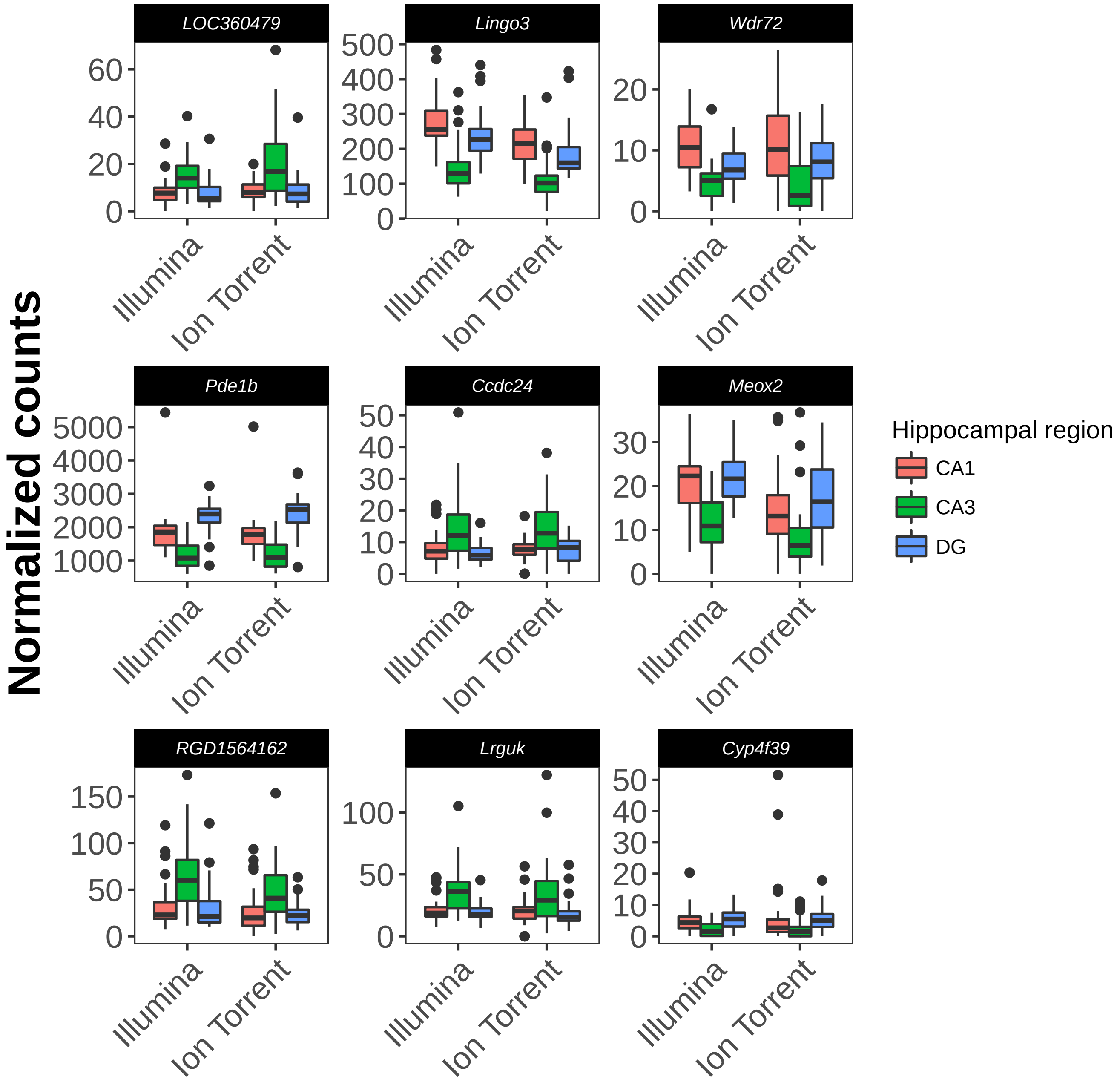

Normalized counts
LOC360479
Lingo3
Wdr72
Pde1b
Ccdc24
Meox2
RGD1564162
Lrguk
Cyp4f39
Illumina
Ion Torrent
Hippocampal region
CA1
CA3
DG

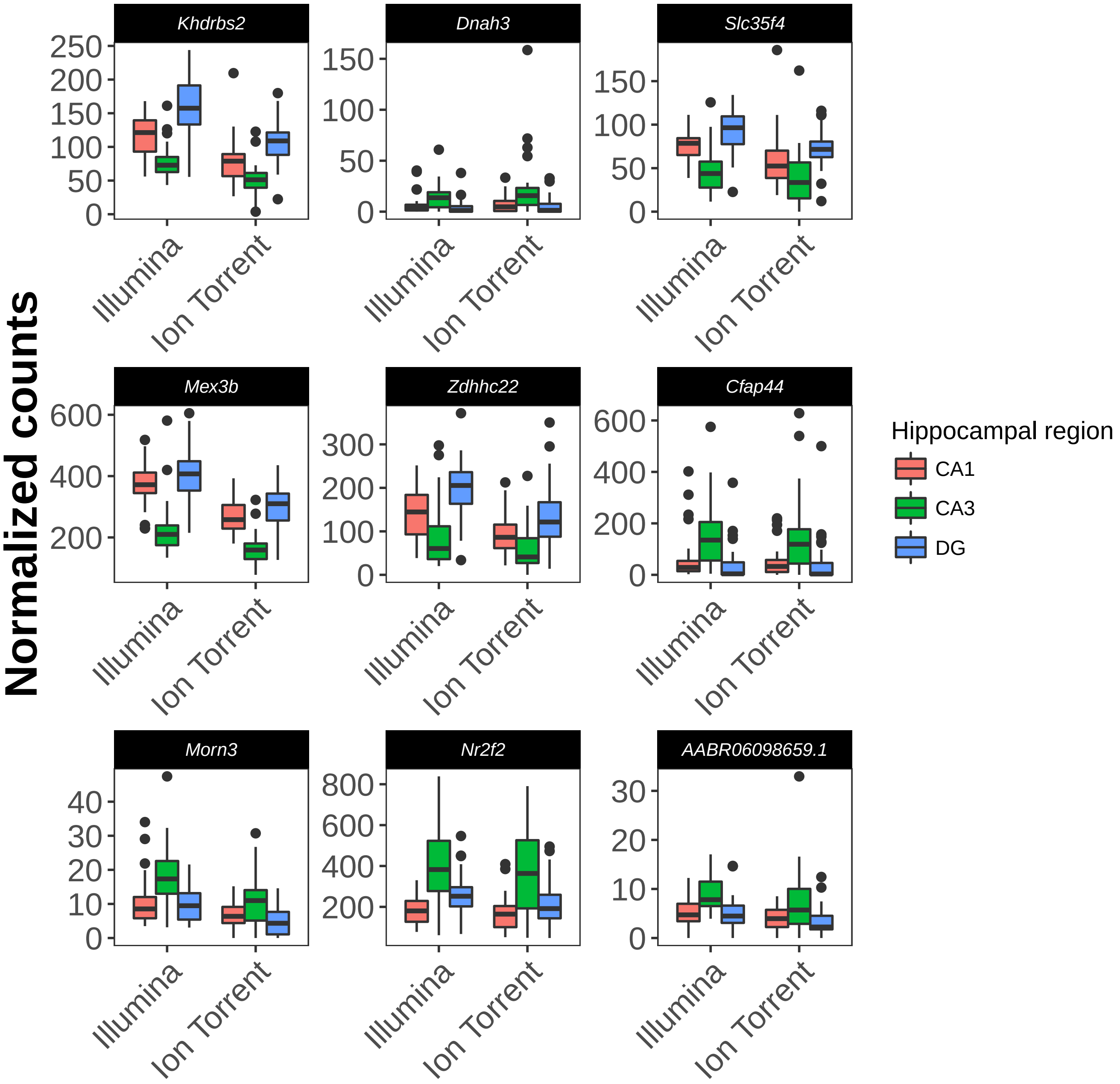
Normalized counts
Khdrbs2
Dnah3
Slc35f4
Mex3b
Zdhhc22
Cfap44
Morn3
Nr2f2
AABR06098659.1
Illumina
Ion Torrent
Hippocampal region
CA1
CA3
DG

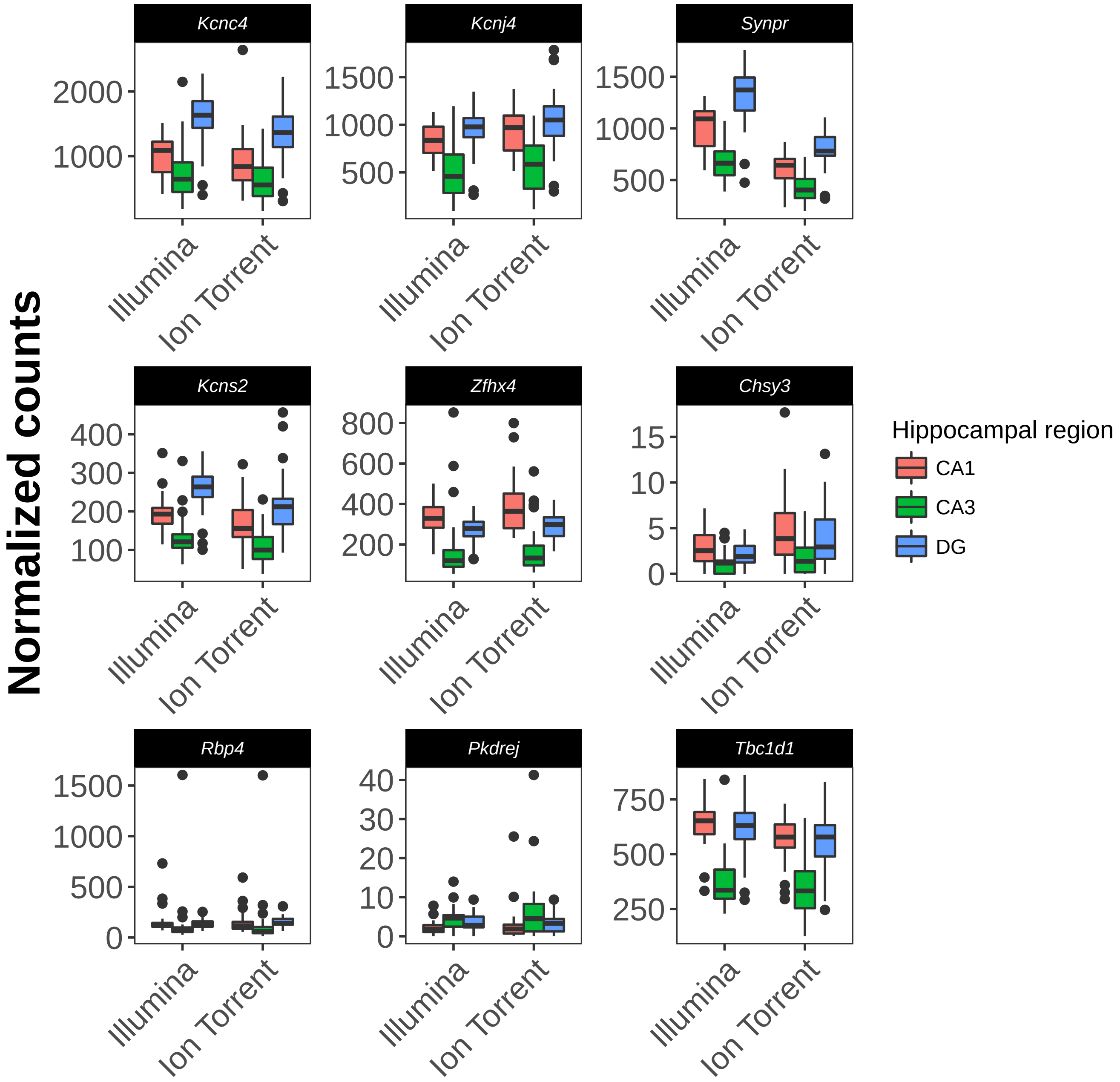
Normalized counts
Kcnc4
Kcnj4
Synpr
Kcns2
Zfhx4
Chsy3
Rbp4
Pkdrej
Tbc1d1
Illumina
Ion Torrent
Hippocampal region
CA1
CA3
DG

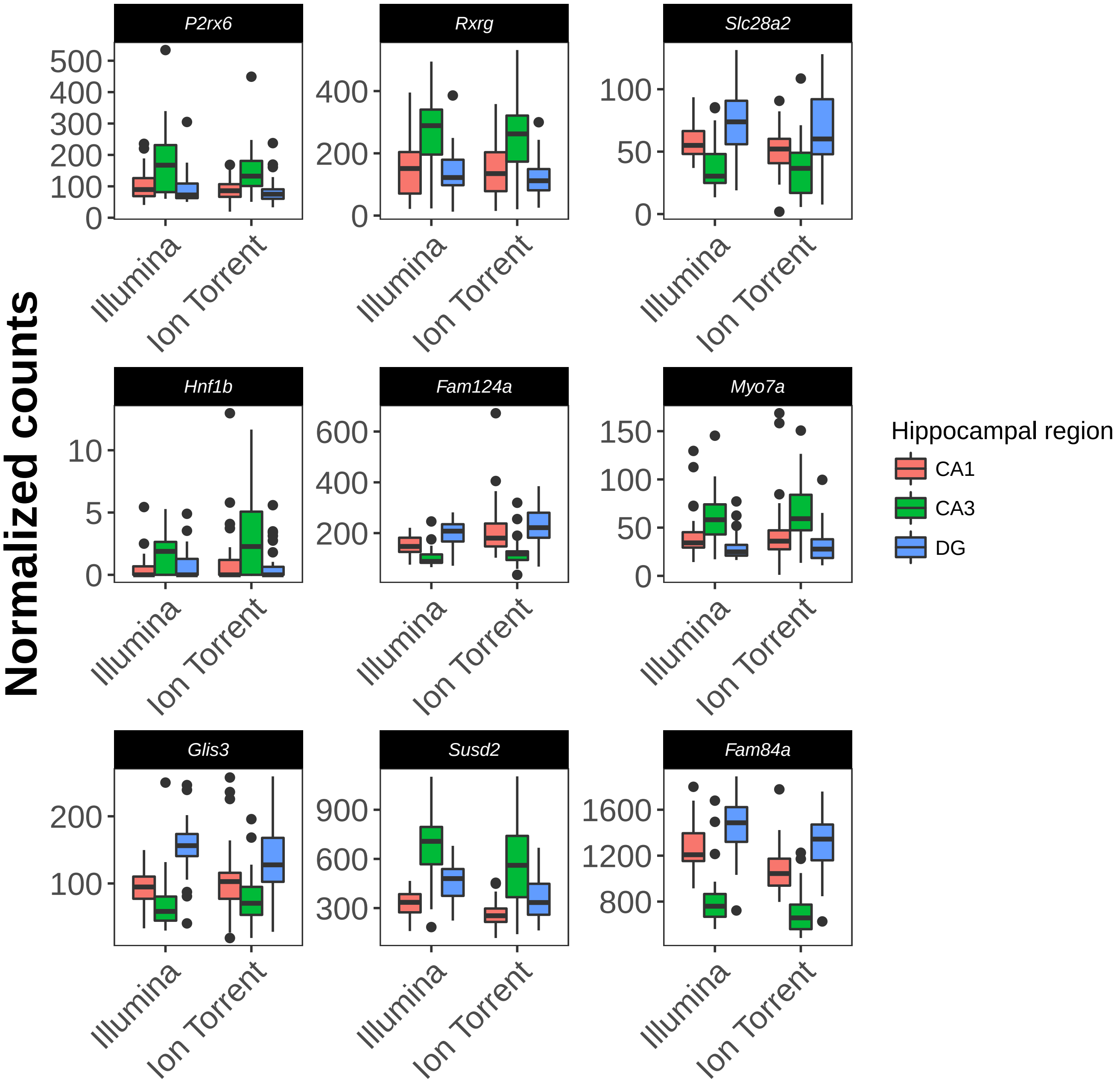
P2rx6
Rxrg
Slc28a2
Hnf1b
Fam124a
Myo7a
Glis3
Susd2
Fam84a
Normalized counts
Illumina
Ion Torrent
Hippocampal region
CA1
CA3
DG

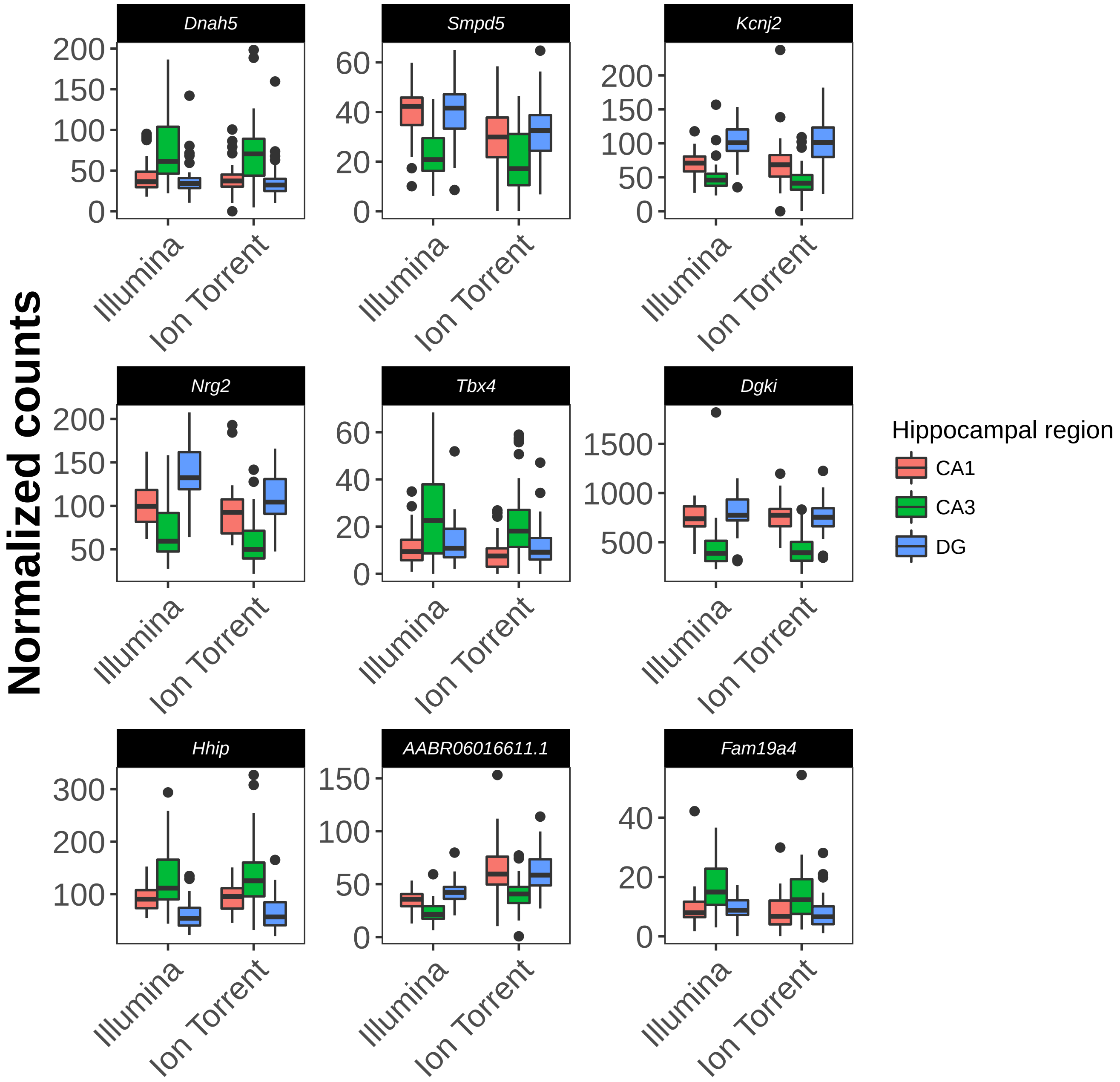

Normalized counts
Dnah5
Smpd5
Kcnj2
Nrg2
Tbx4
Dgki
Hhip
AABR06016611.1
Fam19a4
Illumina
Ion Torrent
Hippocampal region
CA1
CA3
DG

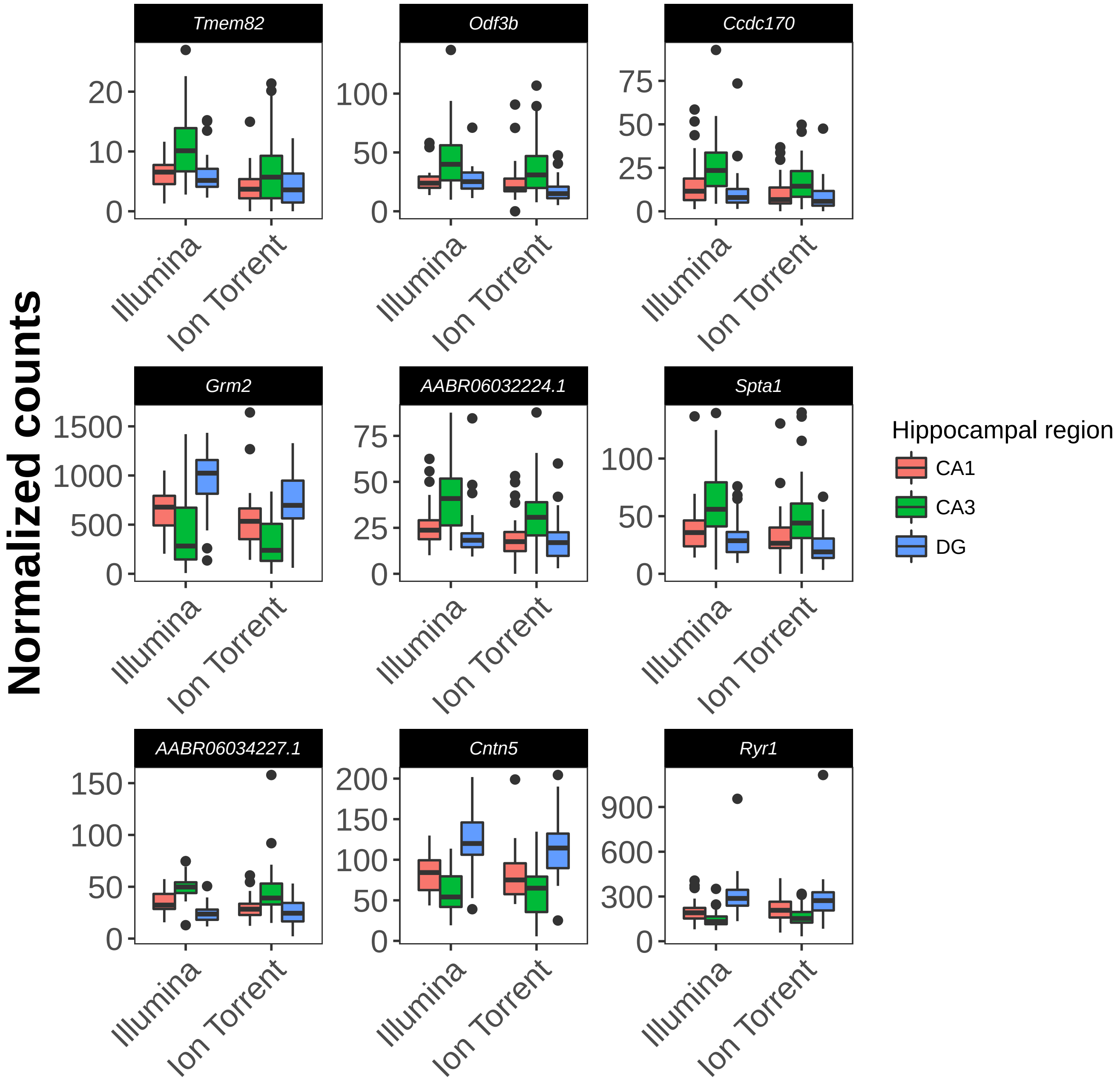

Normalized counts
Tmem82
Odf3b
Ccdc170
Grm2
AABR06032224.1
Spta1
AABR06034227.1
Cntn5
Ryr1
Illumina
Ion Torrent
Hippocampal region
CA1
CA3
DG

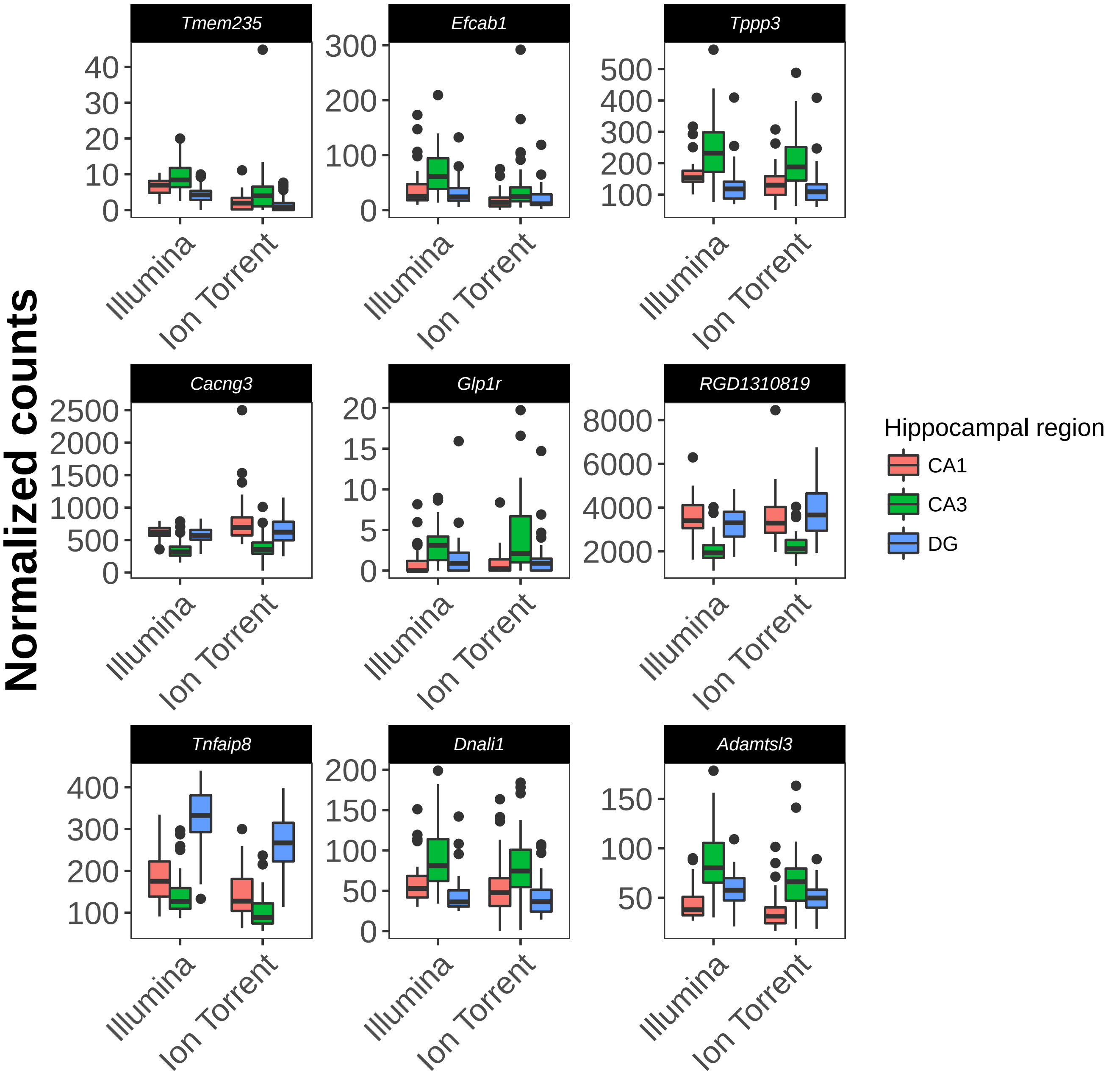
Normalized counts
Tmem235
Efcab1
Tppp3
Cacng3
Glp1r
RGD1310819
Tnfaip8
Dnali1
Adamtsl3
Illumina
Ion Torrent
Hippocampal region
CA1
CA3
DG

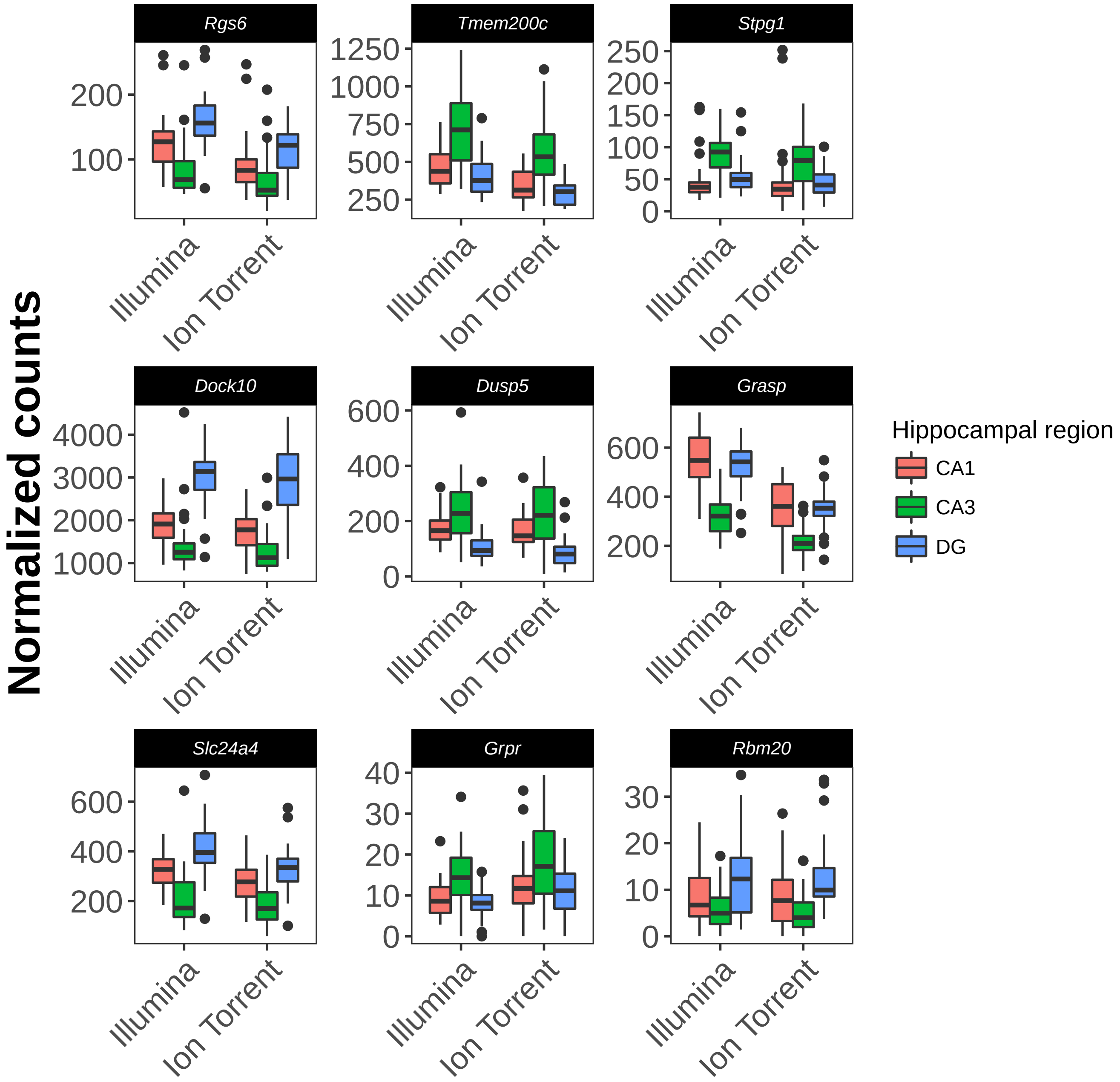

Normalized counts
Rgs6
Tmem200c
Stpg1
Dock10
Dusp5
Grasp
Slc24a4
Grpr
Rbm20
Illumina
Ion Torrent
Hippocampal region
CA1
CA3
DG

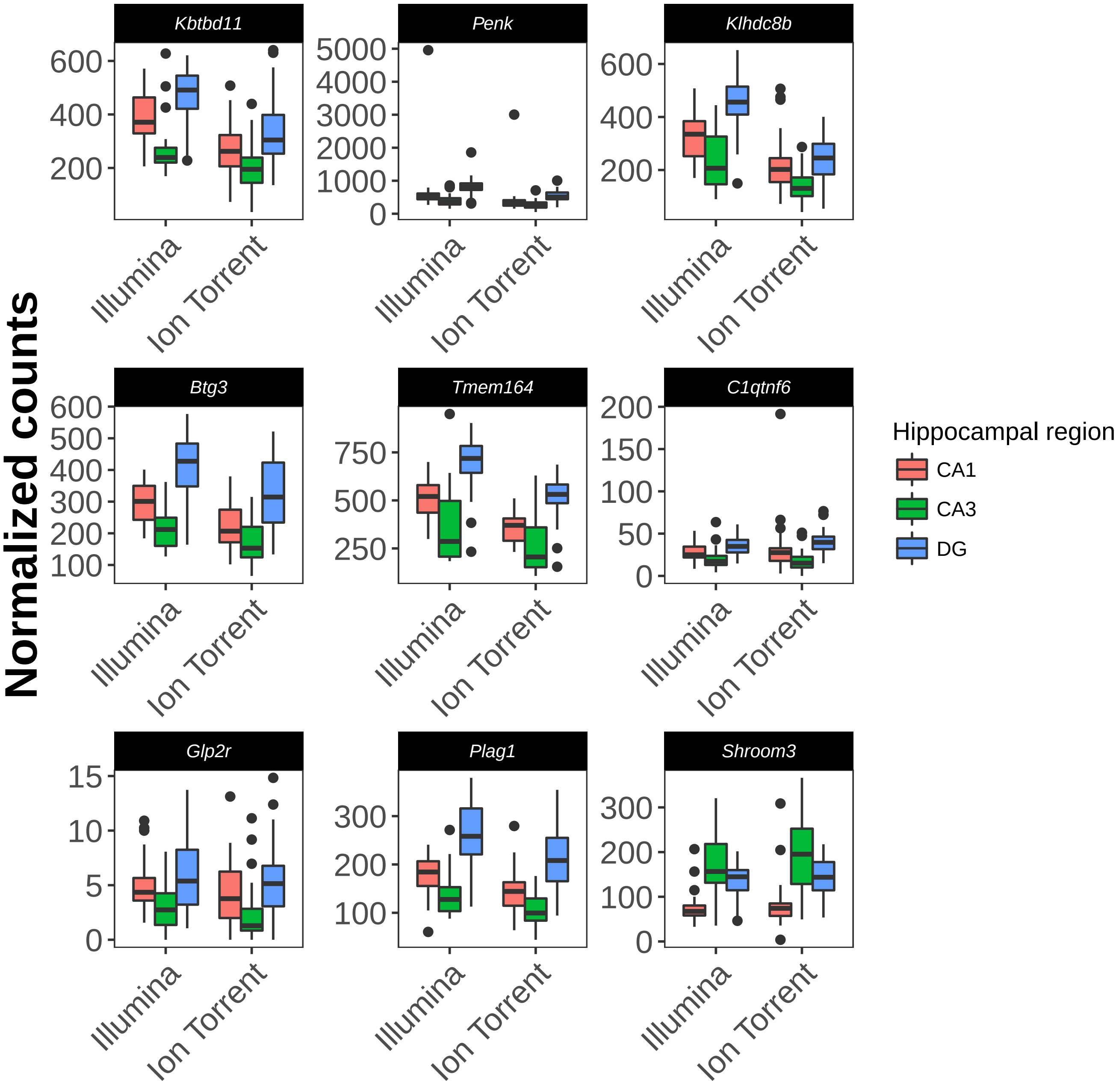
Normalized counts
Kbtbd11
Penk
Klhdc8b
Btg3
Tmem164
C1qtnf6
Glp2r
Plag1
Shroom3
Illumina
Ion Torrent
Hippocampal region
CA1
CA3
DG

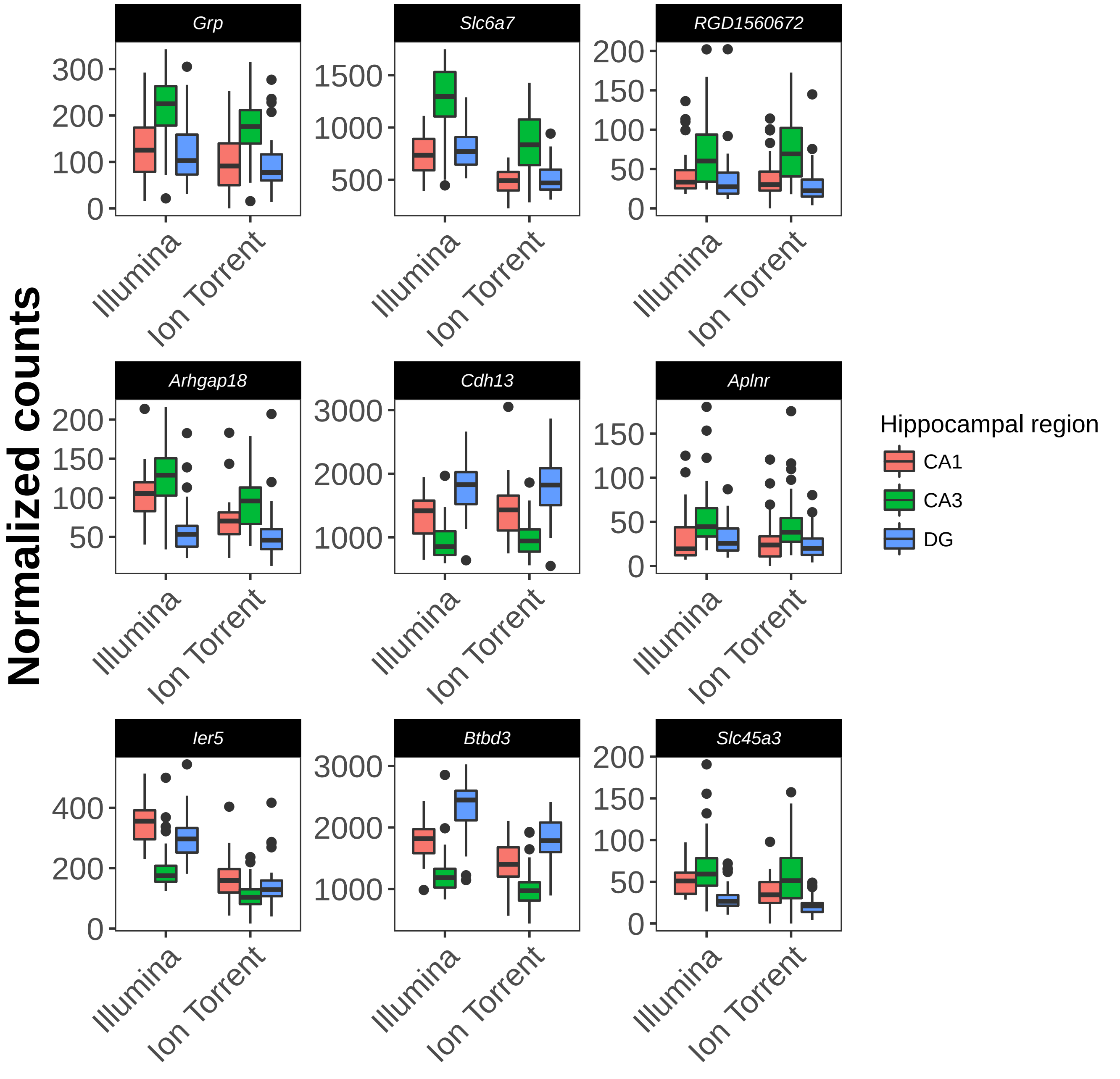
Normalized counts
Grp
Slc6a7
RGD1560672
Arhgap18
Cdh13
Aplnr
Ier5
Btbd3
Slc45a3
Illumina
Ion Torrent
Hippocampal region
CA1
CA3
DG

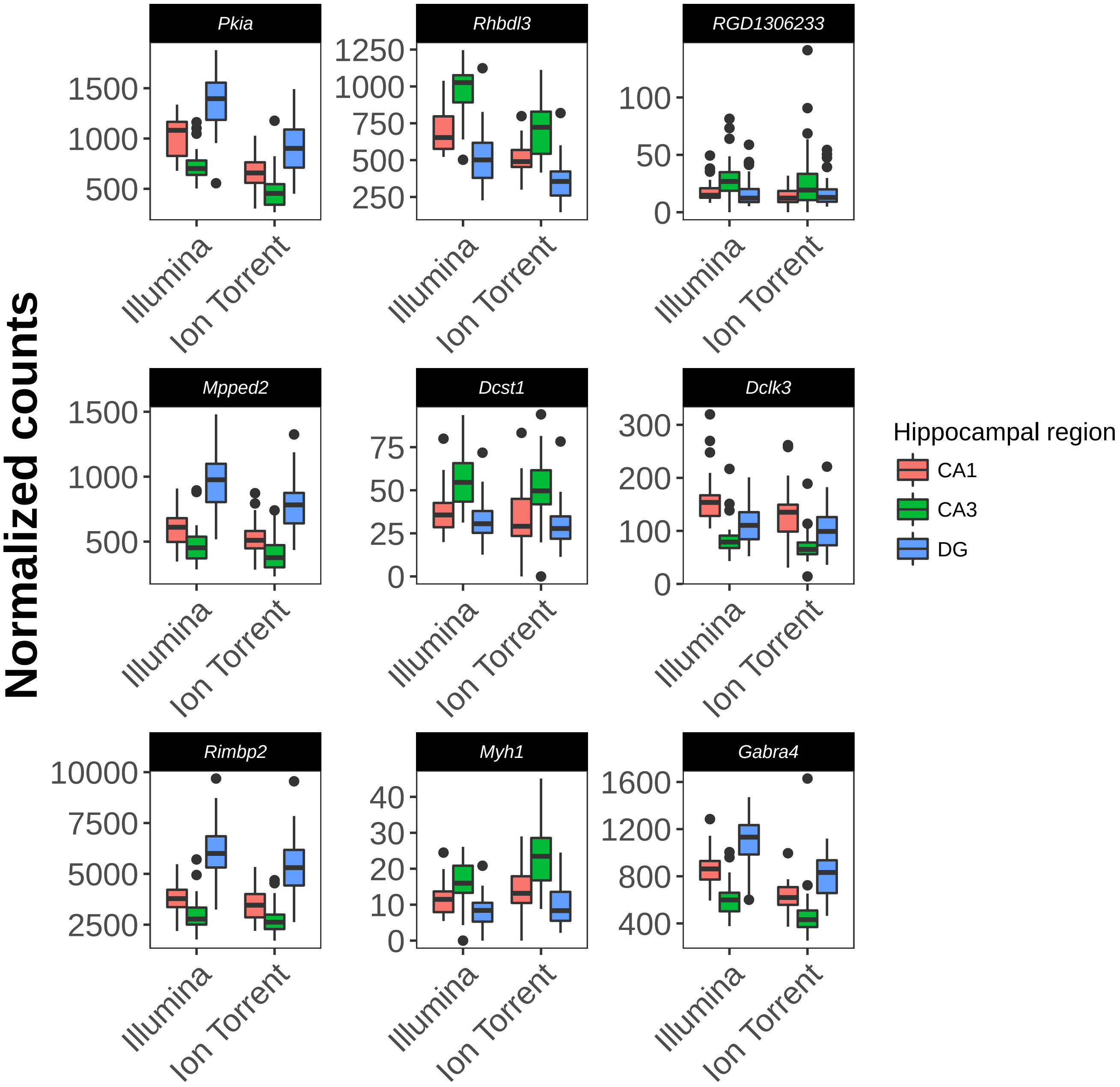
Normalized counts
Pkia
Rhbdl3
RGD1306233
Mpped2
Dcst1
Dclk3
Rimbp2
Myh1
Gabra4
Illumina
Ion Torrent
Hippocampal region
CA1
CA3
DG

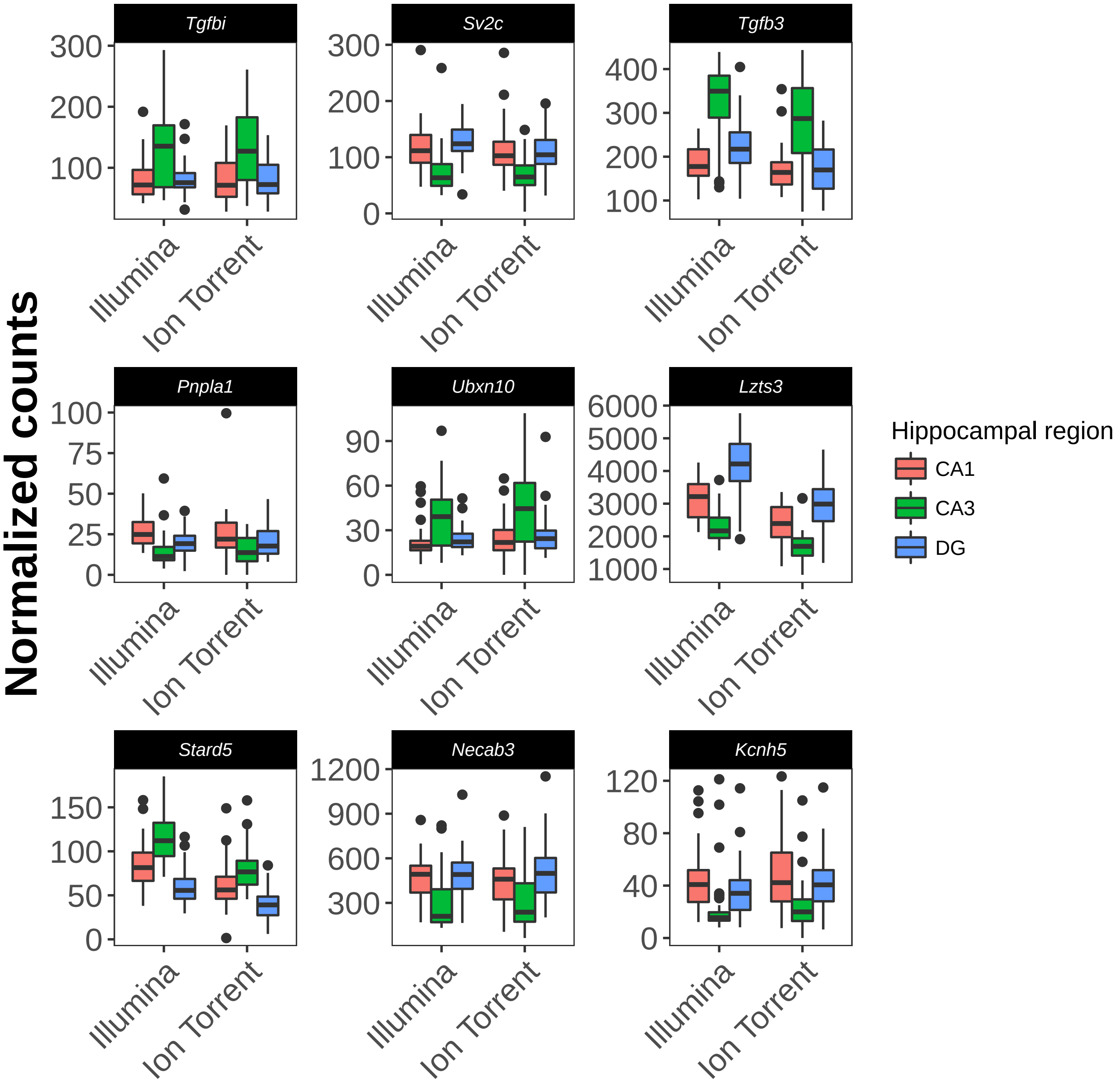
Normalized counts
Tgfbi
Sv2c
Tgfb3
Pnpla1
Ubxn10
Lzts3
Stard5
Necab3
Kcnh5
Illumina
Ion Torrent
Hippocampal region
CA1
CA3
DG

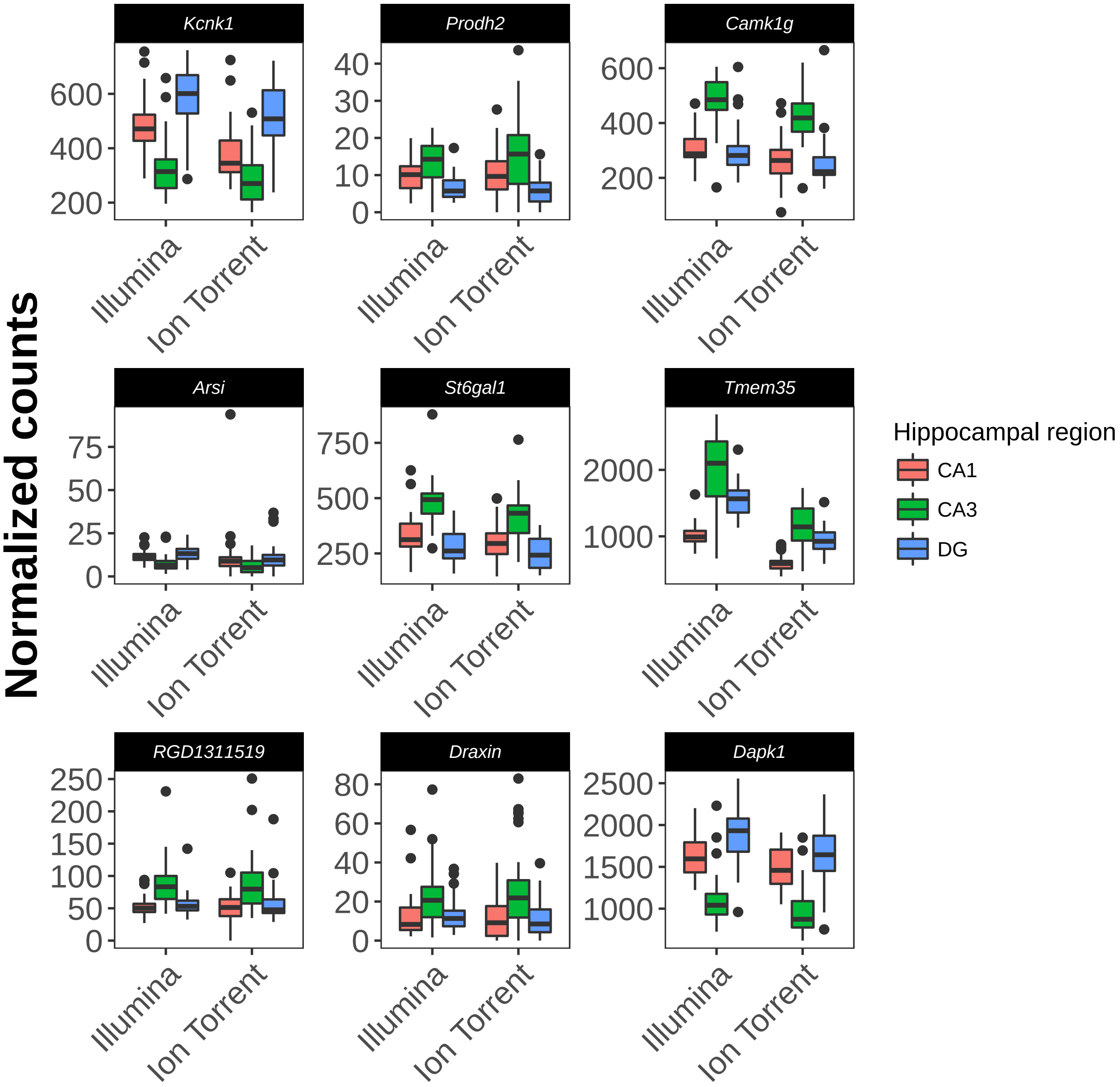
Normalized counts
Kcnk1
Prodh2
Camk1g
Arsi
St6gal1
Tmem35
RGD1311519
Draxin
Dapk1
Illumina
Ion Torrent
Hippocampal region
CA1
CA3
DG

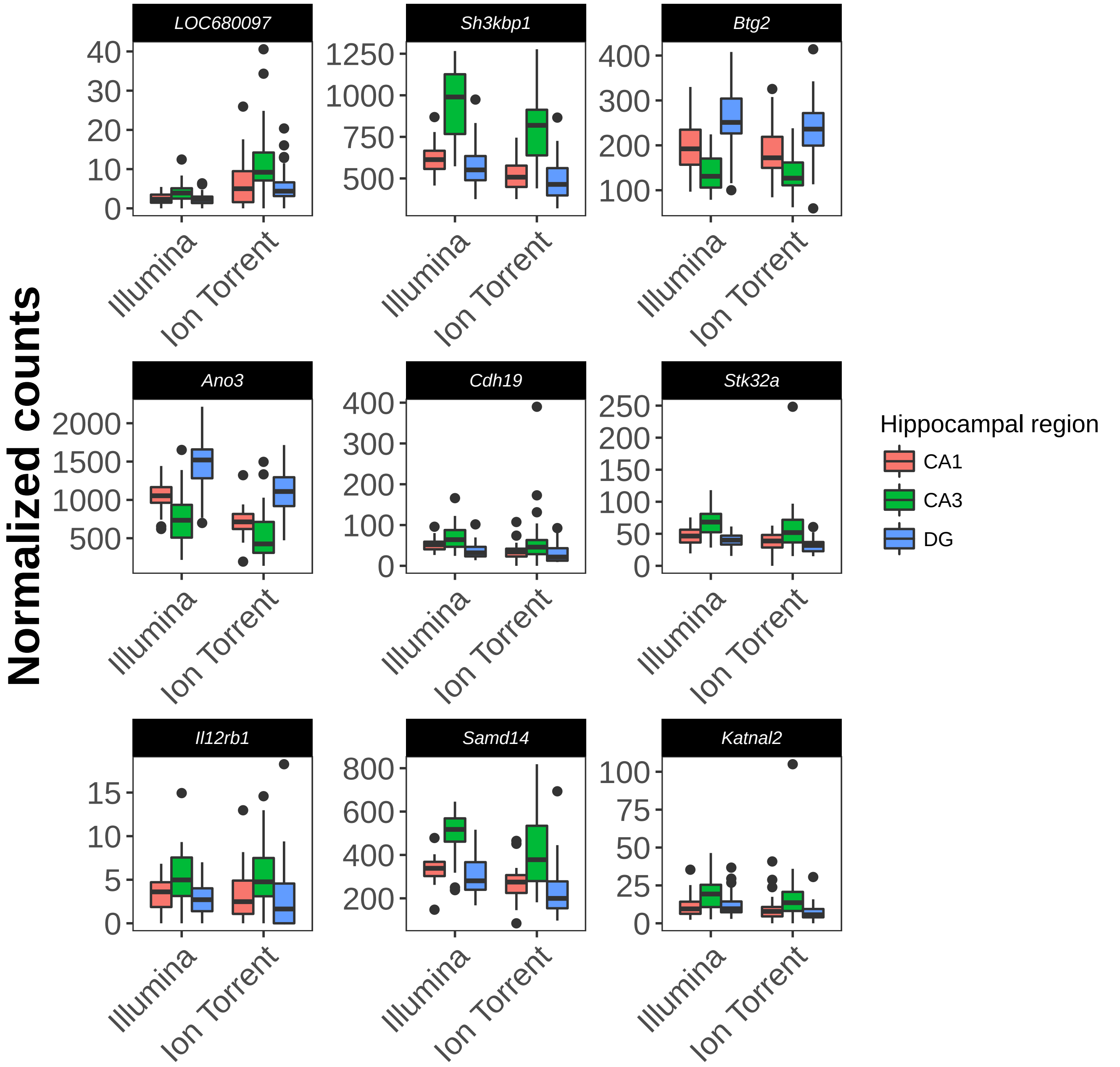

Normalized counts
LOC680097
Sh3kbp1
Btg2
Ano3
Cdh19
Stk32a
Il12rb1
Samd14
Katnal2
Illumina
Ion Torrent
Hippocampal region
CA1
CA3
DG

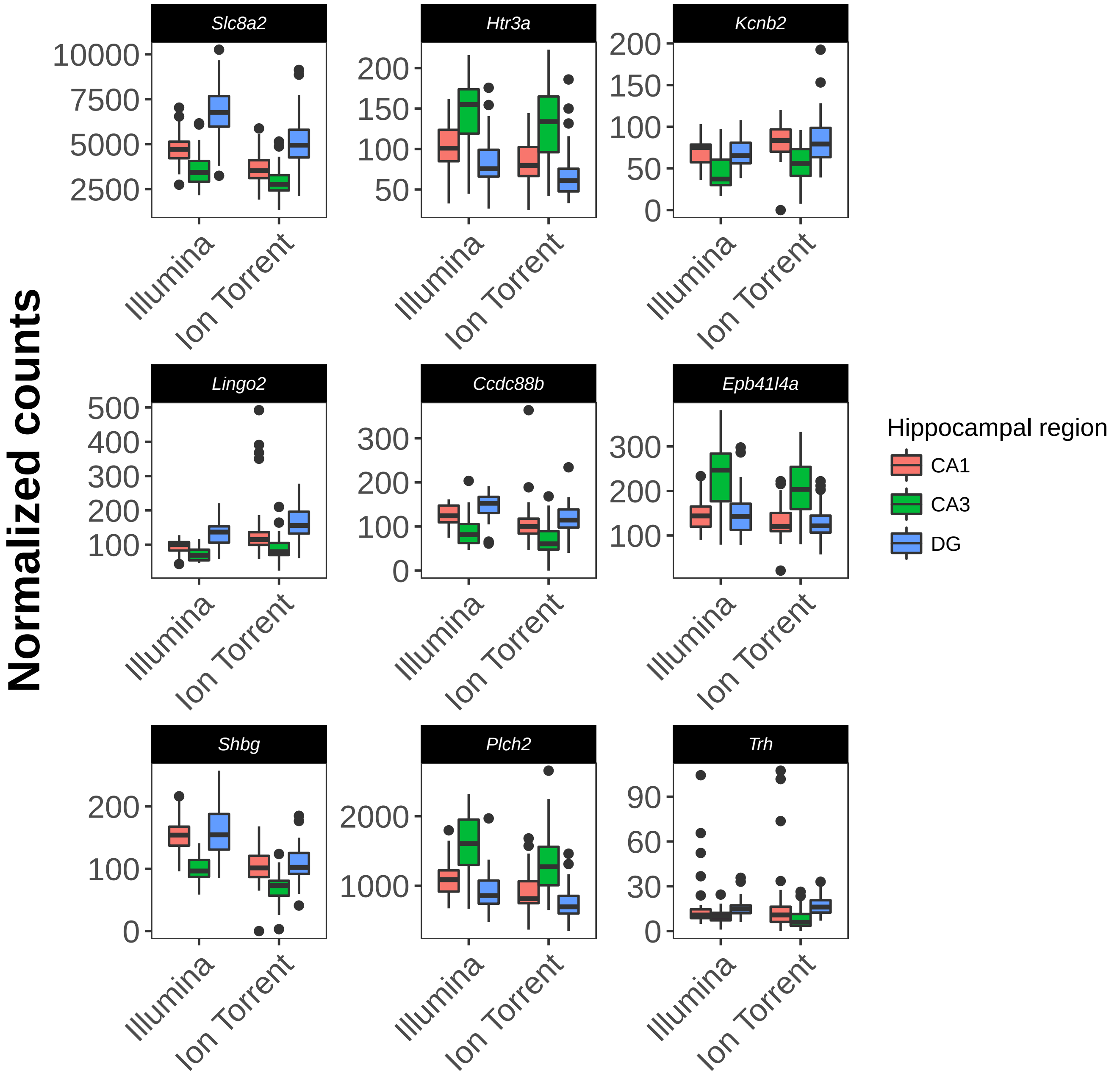

Normalized counts
Slc8a2
Htr3a
Kcnb2
Lingo2
Ccdc88b
Epb41l4a
Shbg
Plch2
Trh
Illumina
Ion Torrent
Hippocampal region
CA1
CA3
DG

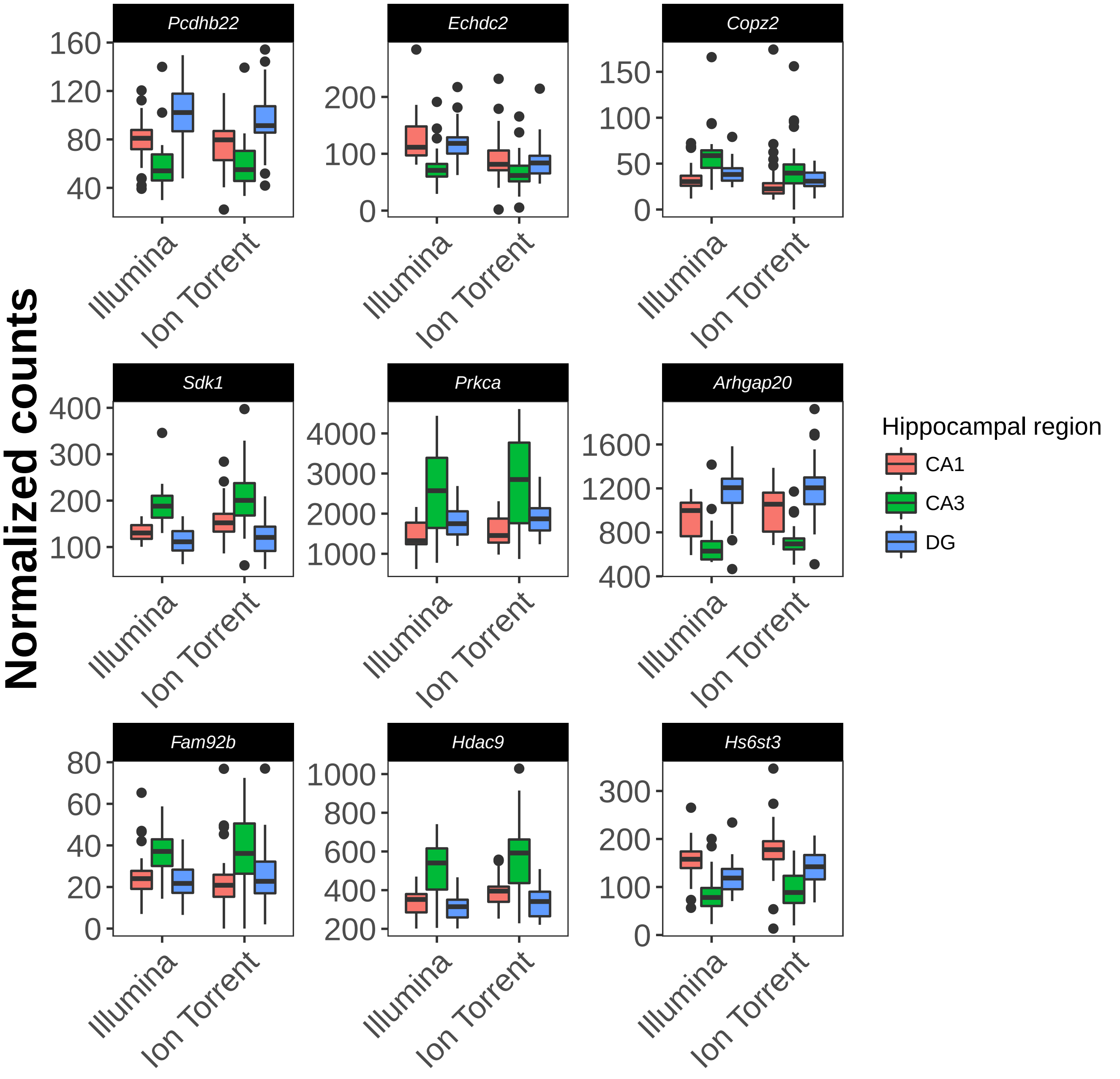

Normalized counts
Pcdhb22
Echdc2
Copz2
Sdk1
Prkca
Arhgap20
Fam92b
Hdac9
Hs6st3
Illumina
Ion Torrent
Hippocampal region
CA1
CA3
DG

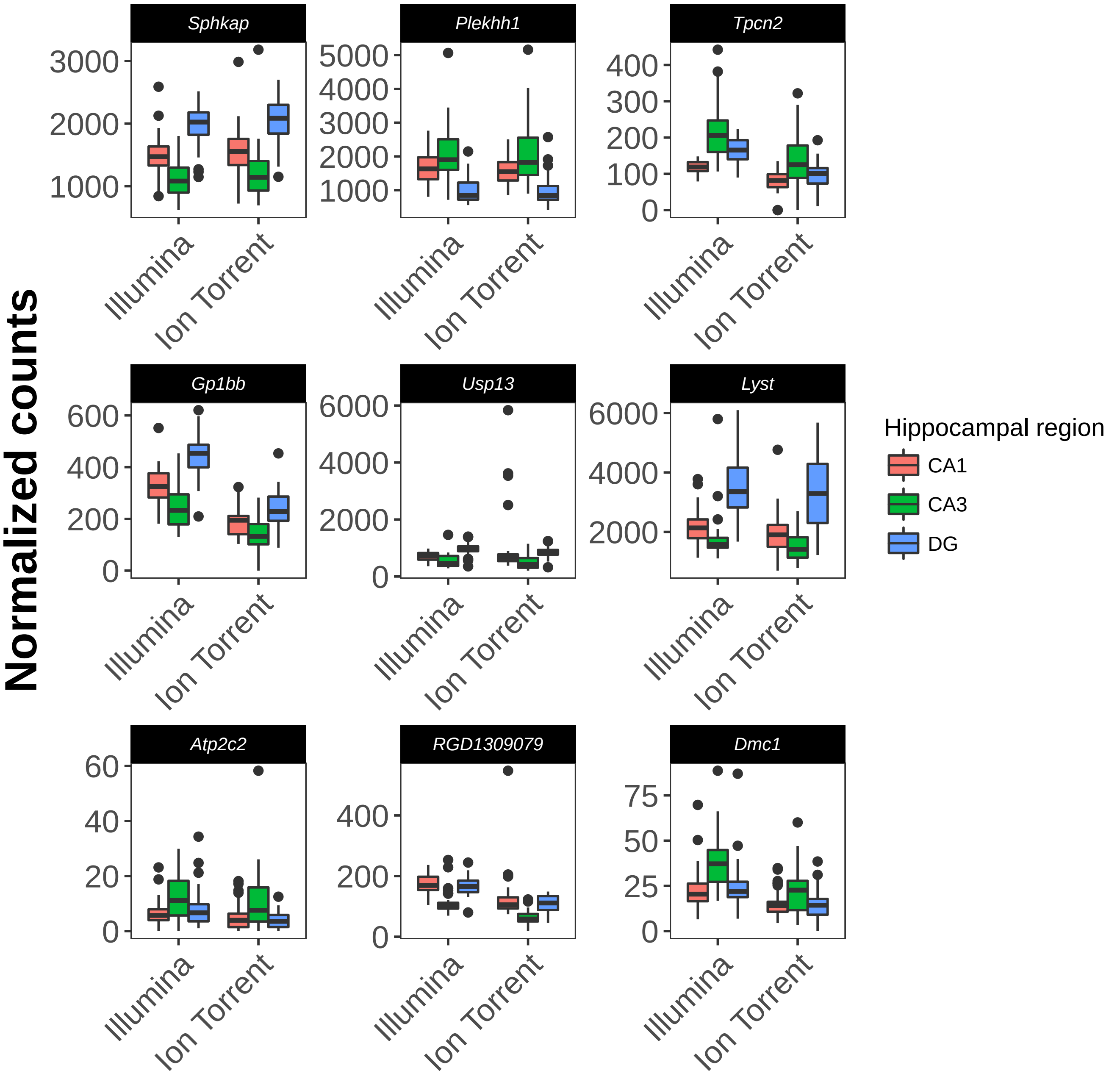
Normalized counts
Sphkap
Plekhh1
Tpcn2
Gp1bb
Usp13
Lyst
Atp2c2
RGD1309079
Dmc1
Illumina
Ion Torrent
Hippocampal region
CA1
CA3
DG

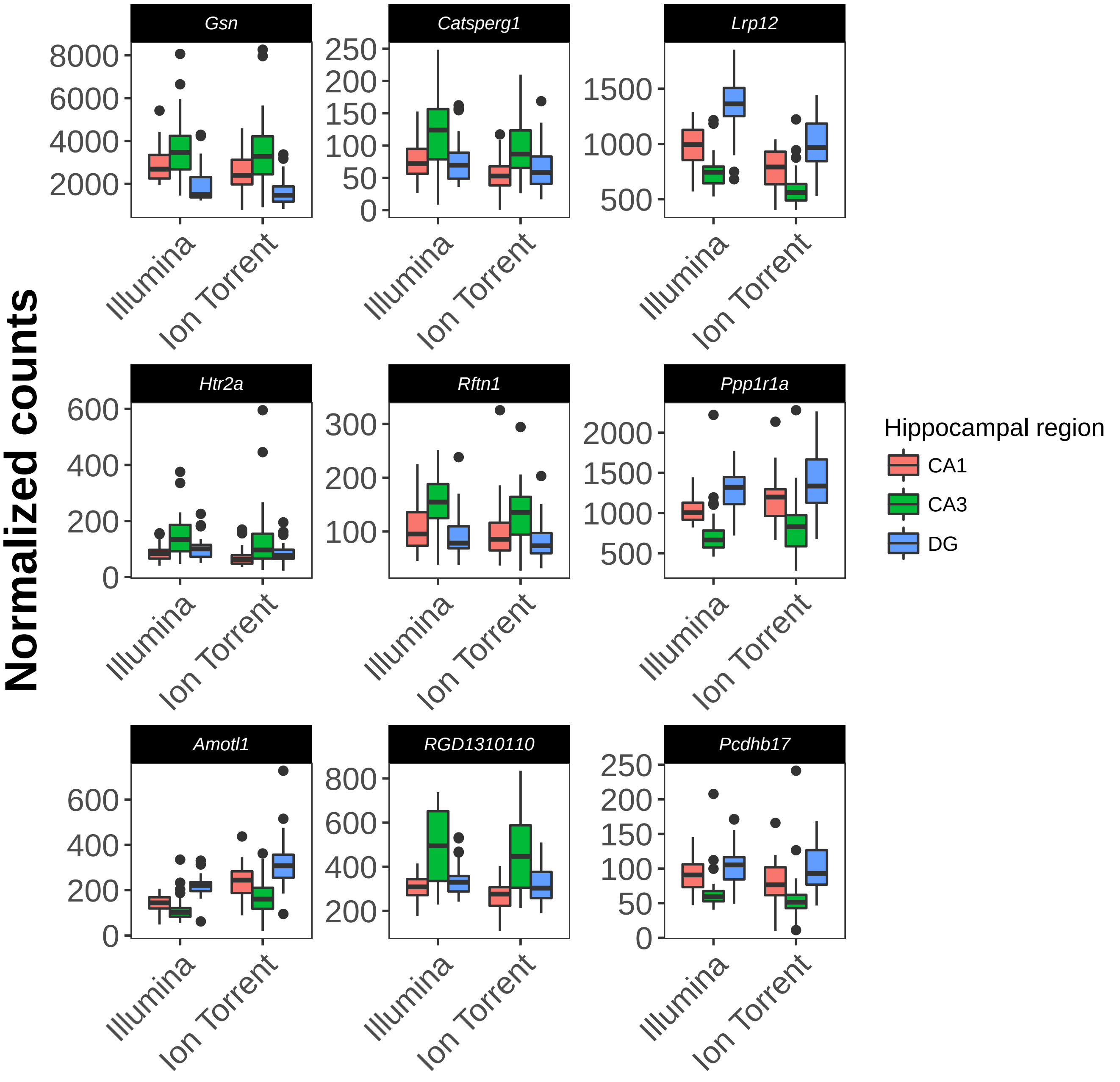

Normalized counts
Gsn
Catsperg1
Lrp12
Htr2a
Rftn1
Ppp1r1a
Amotl1
RGD1310110
Pcdhb17
Illumina
Ion Torrent
Hippocampal region
CA1
CA3
DG

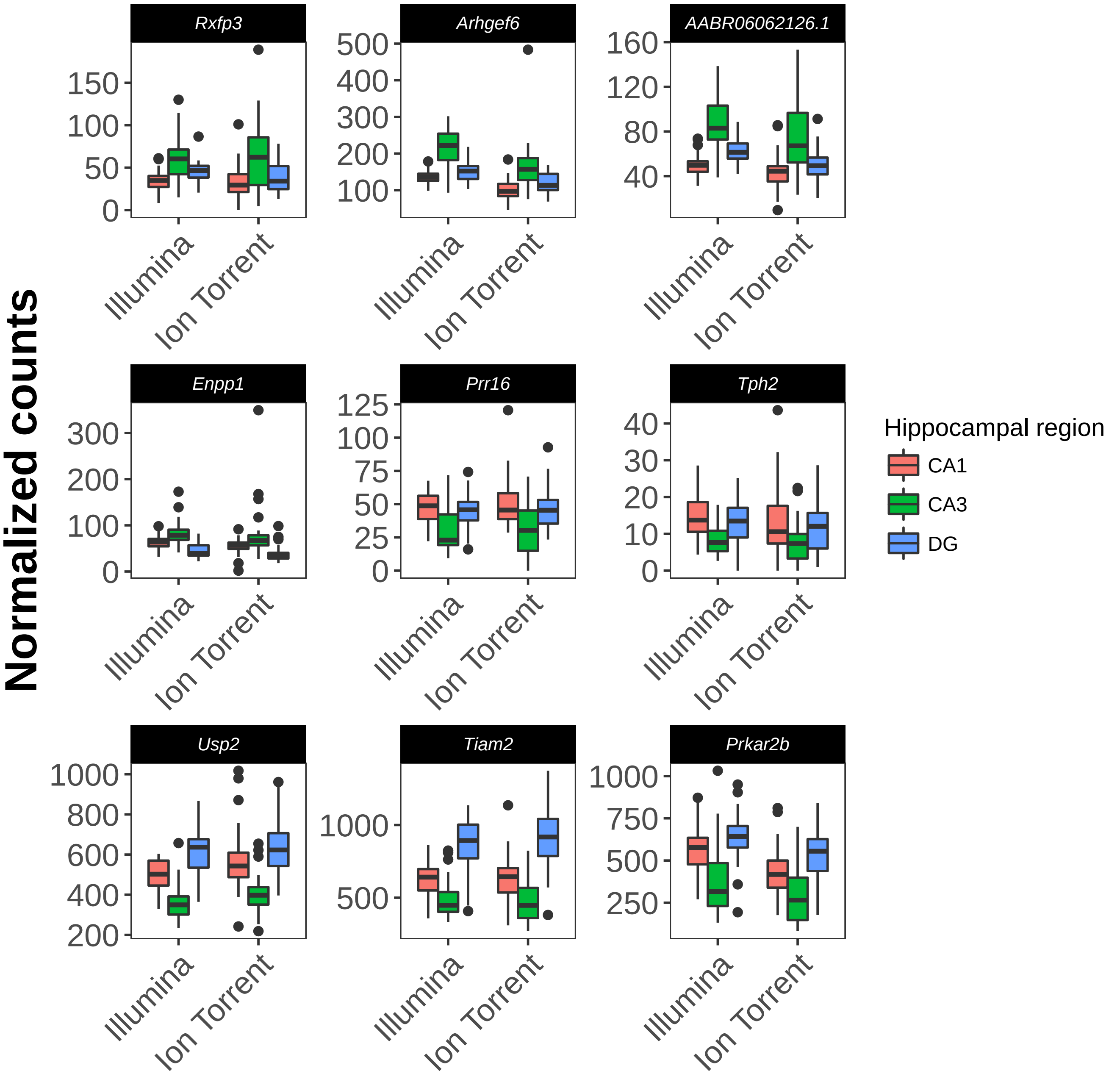

Normalized counts
Rxfp3
Arhgef6
AABR06062126.1
Enpp1
Prr16
Tph2
Usp2
Tiam2
Prkar2b
Illumina
Ion Torrent
Hippocampal region
CA1
CA3
DG

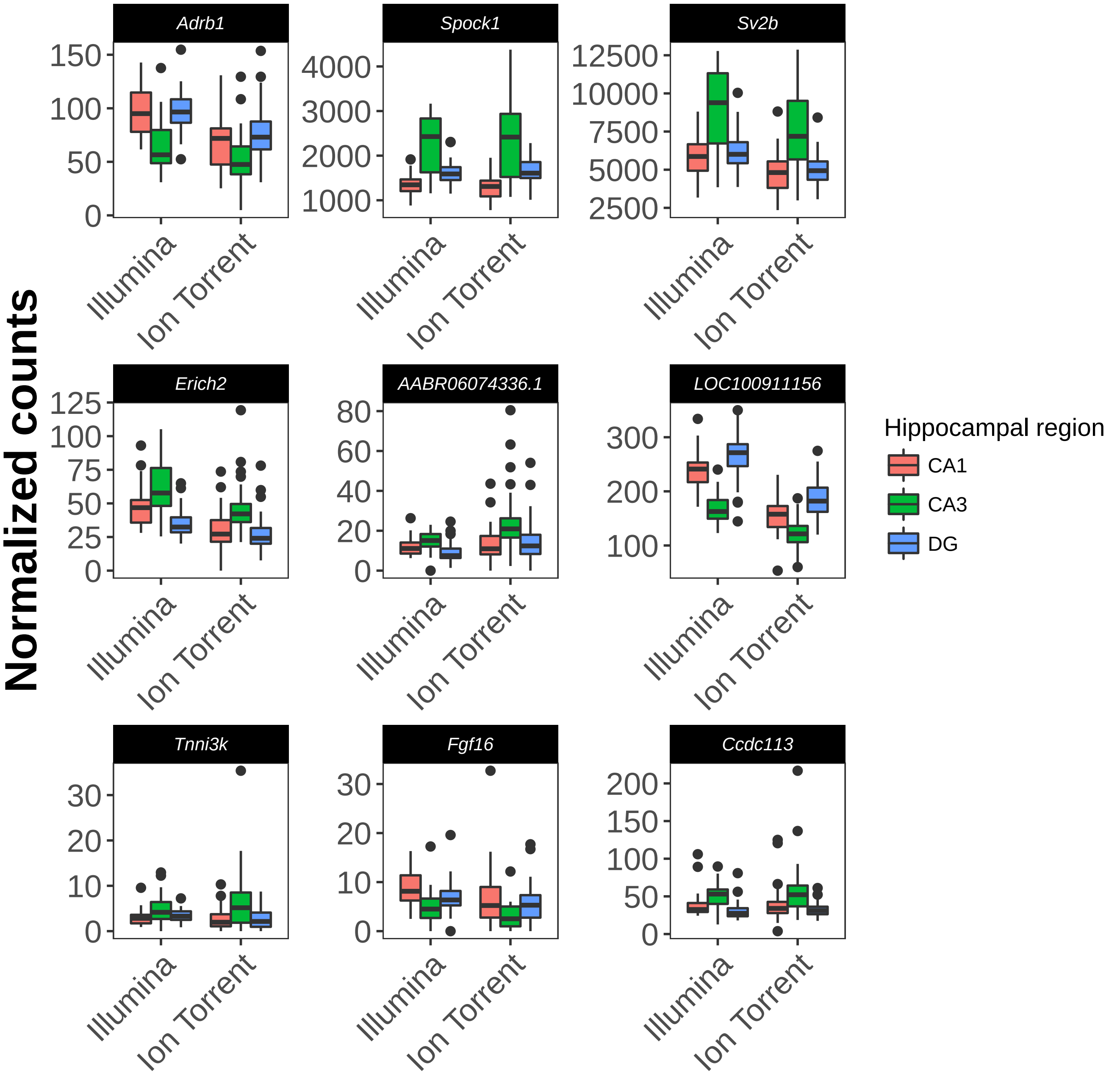
Normalized counts
Adrb1
Spock1
Sv2b
Erich2
AABR06074336.1
LOC100911156
Tnni3k
Fgf16
Ccdc113
Illumina
Ion Torrent
Hippocampal region
CA1
CA3
DG

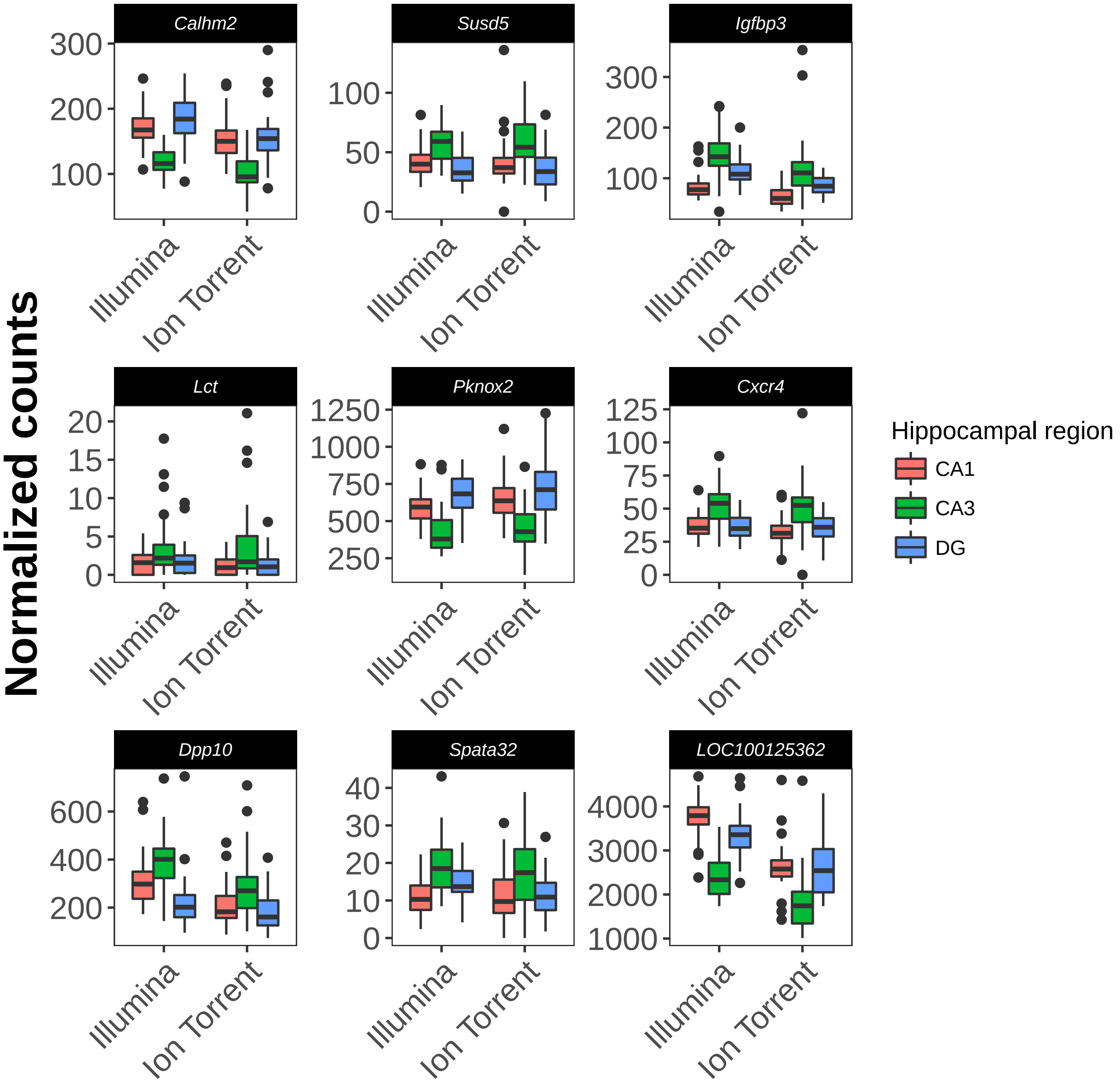
Normalized counts
Calhm2
Susd5
Igfbp3
Lct
Pknox2
Cxcr4
Dpp10
Spata32
LOC100125362
Illumina
Ion Torrent
Hippocampal region
CA1
CA3
DG

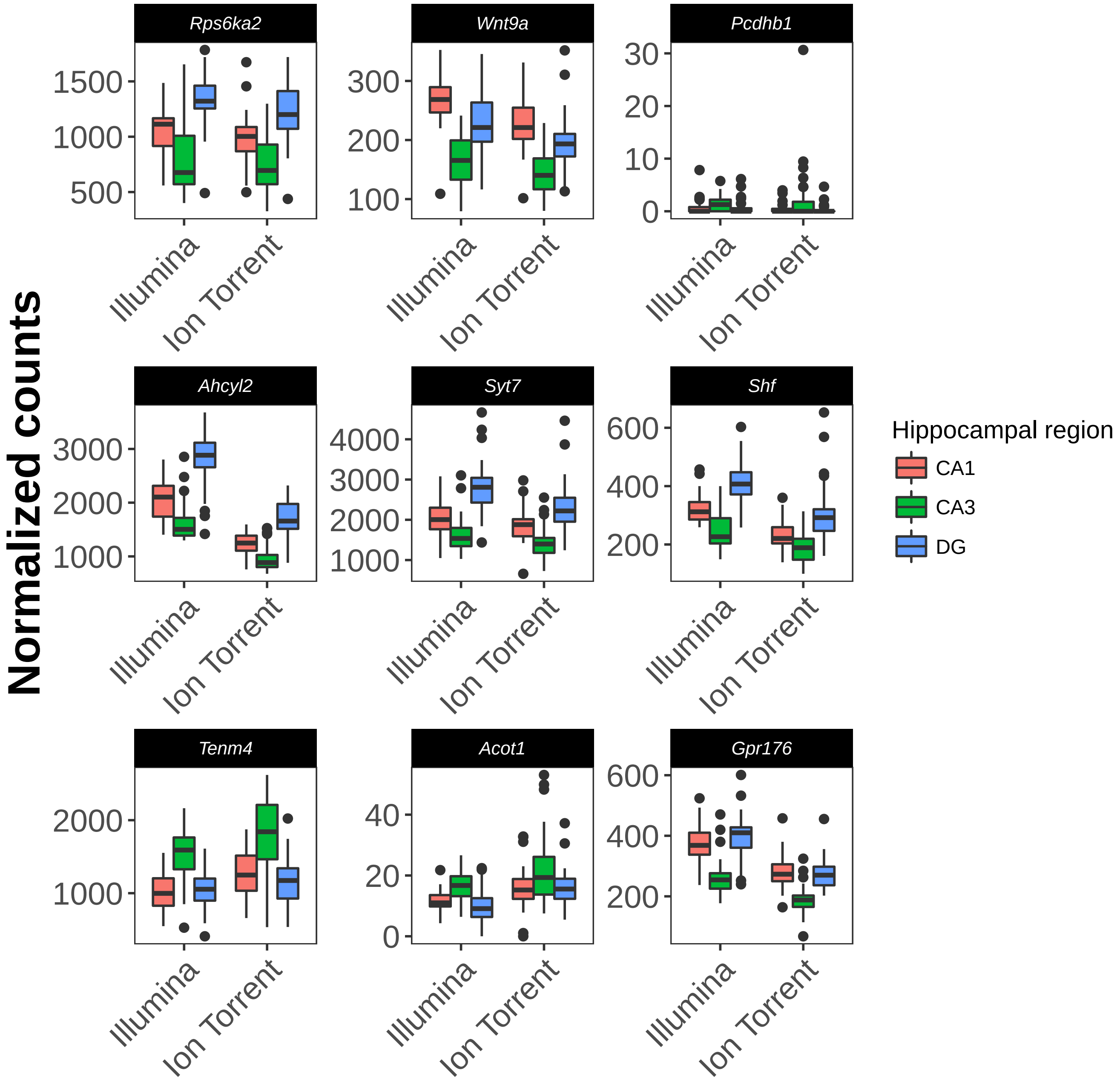
Normalized counts
Rps6ka2
Wnt9a
Pcdhb1
Ahcyl2
Syt7
Shf
Tenm4
Acot1
Gpr176
Illumina
Ion Torrent
Hippocampal region
CA1
CA3
DG

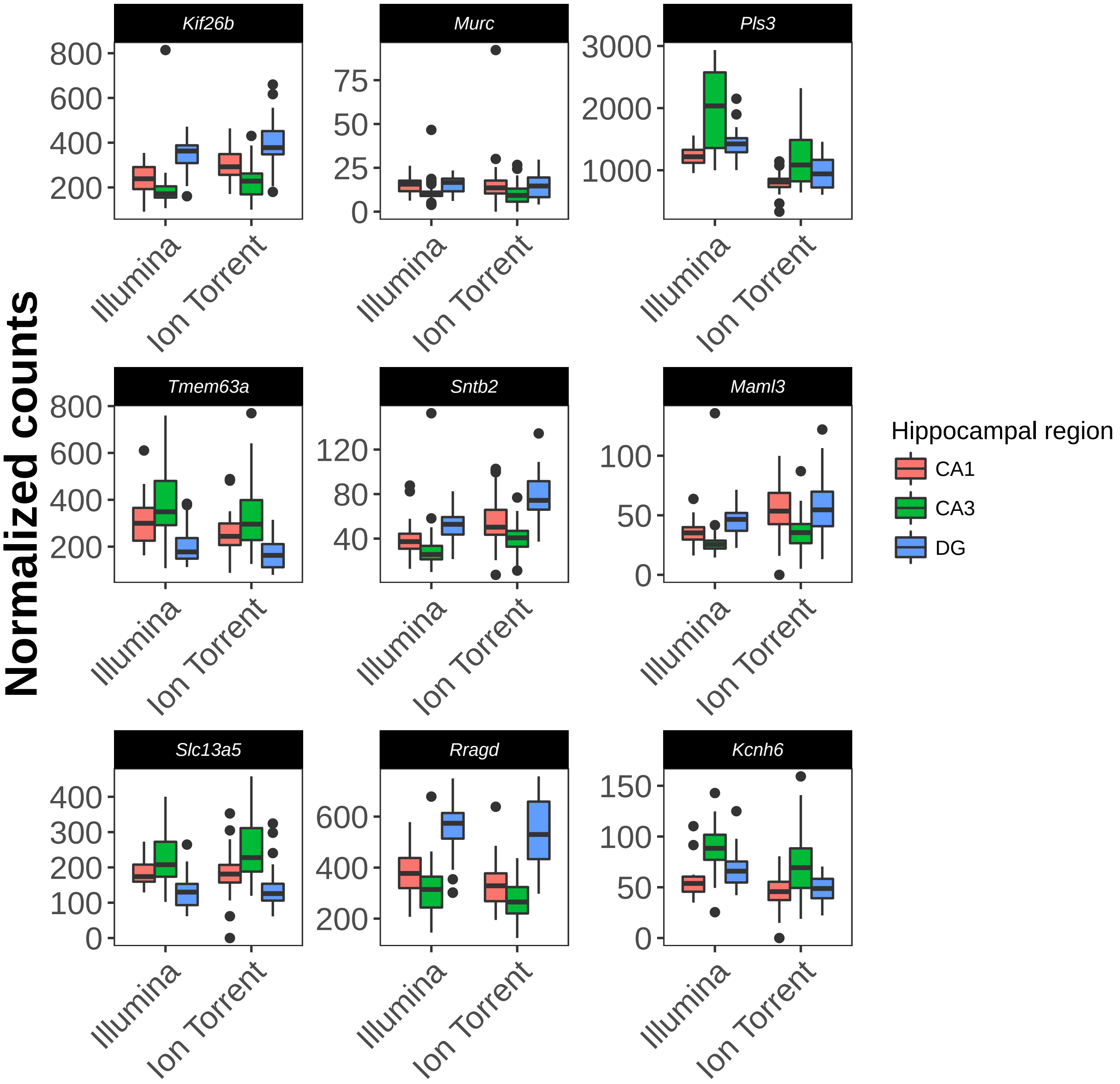

Normalized counts
Kif26b
Murc
Pls3
Tmem63a
Sntb2
Maml3
Slc13a5
Rragd
Kcnh6
Illumina
Ion Torrent
Hippocampal region
CA1
CA3
DG

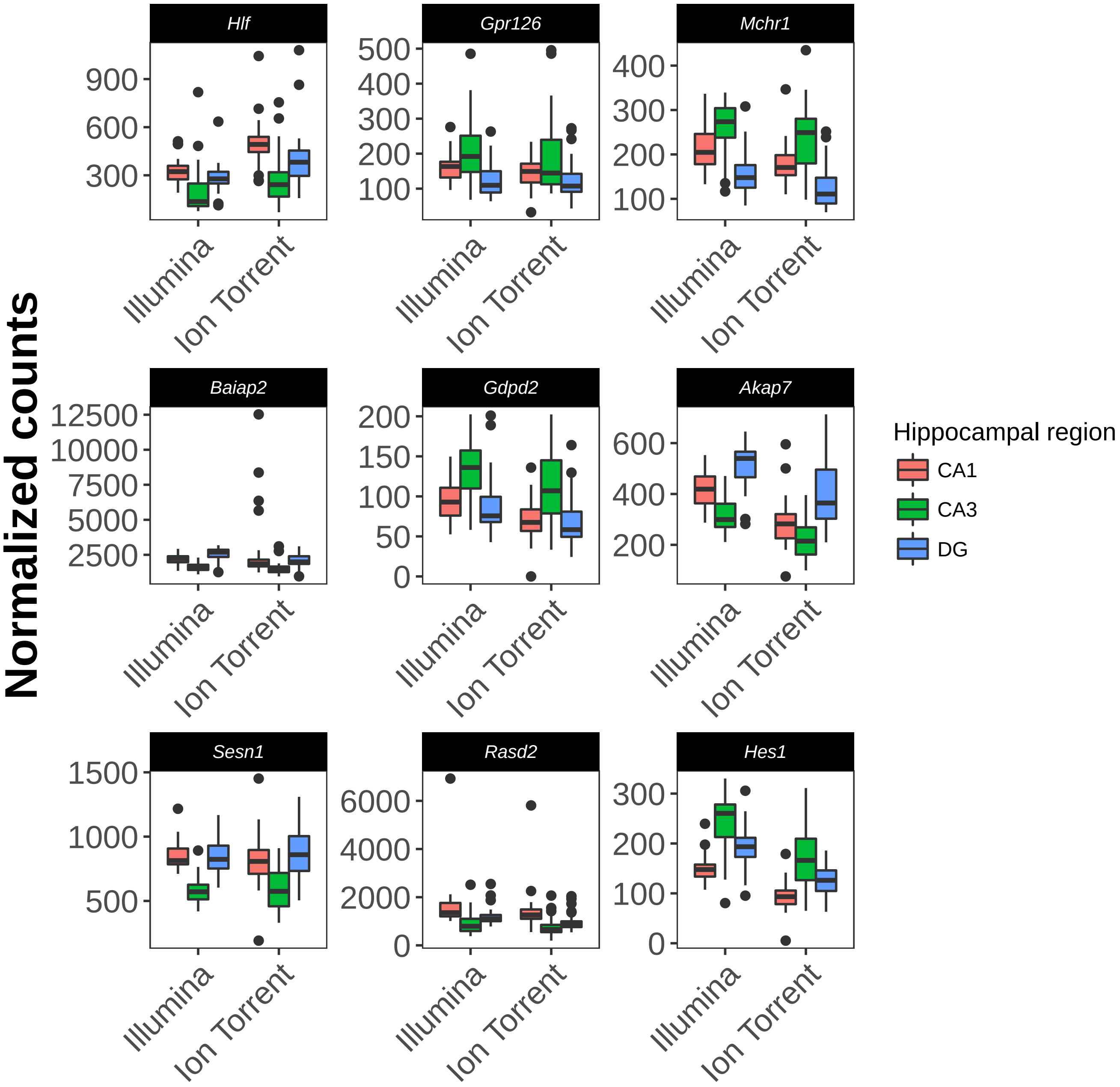

Normalized counts
Hlf
Gpr126
Mchr1
Baiap2
Gdpd2
Akap7
Sesn1
Rasd2
Hes1
Illumina
Ion Torrent
Hippocampal region
CA1
CA3
DG

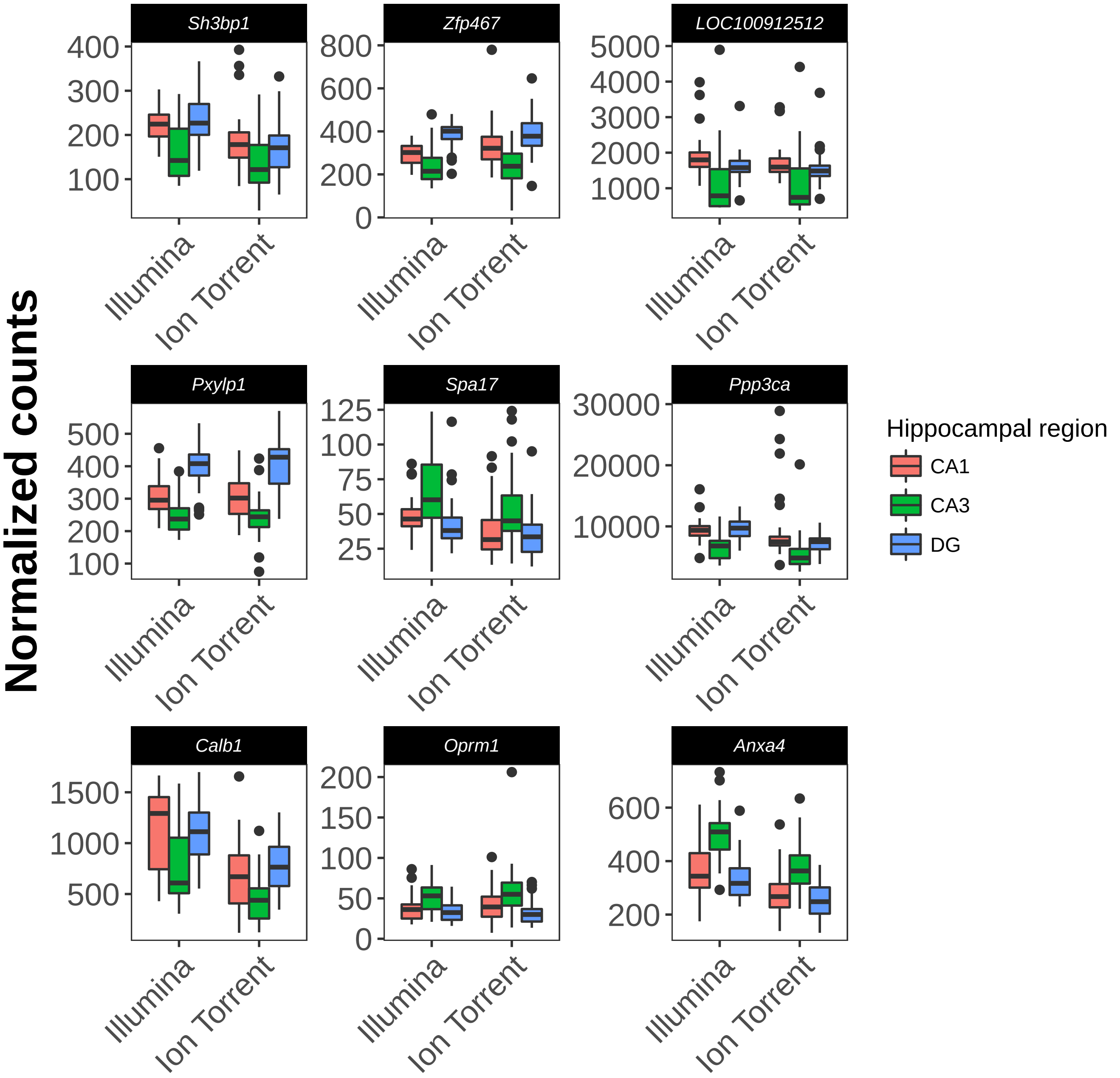

Normalized counts
Sh3bp1
Zfp467
LOC100912512
Pxylp1
Spa17
Ppp3ca
Calb1
Oprm1
Anxa4
Illumina
Ion Torrent
Hippocampal region
CA1
CA3
DG

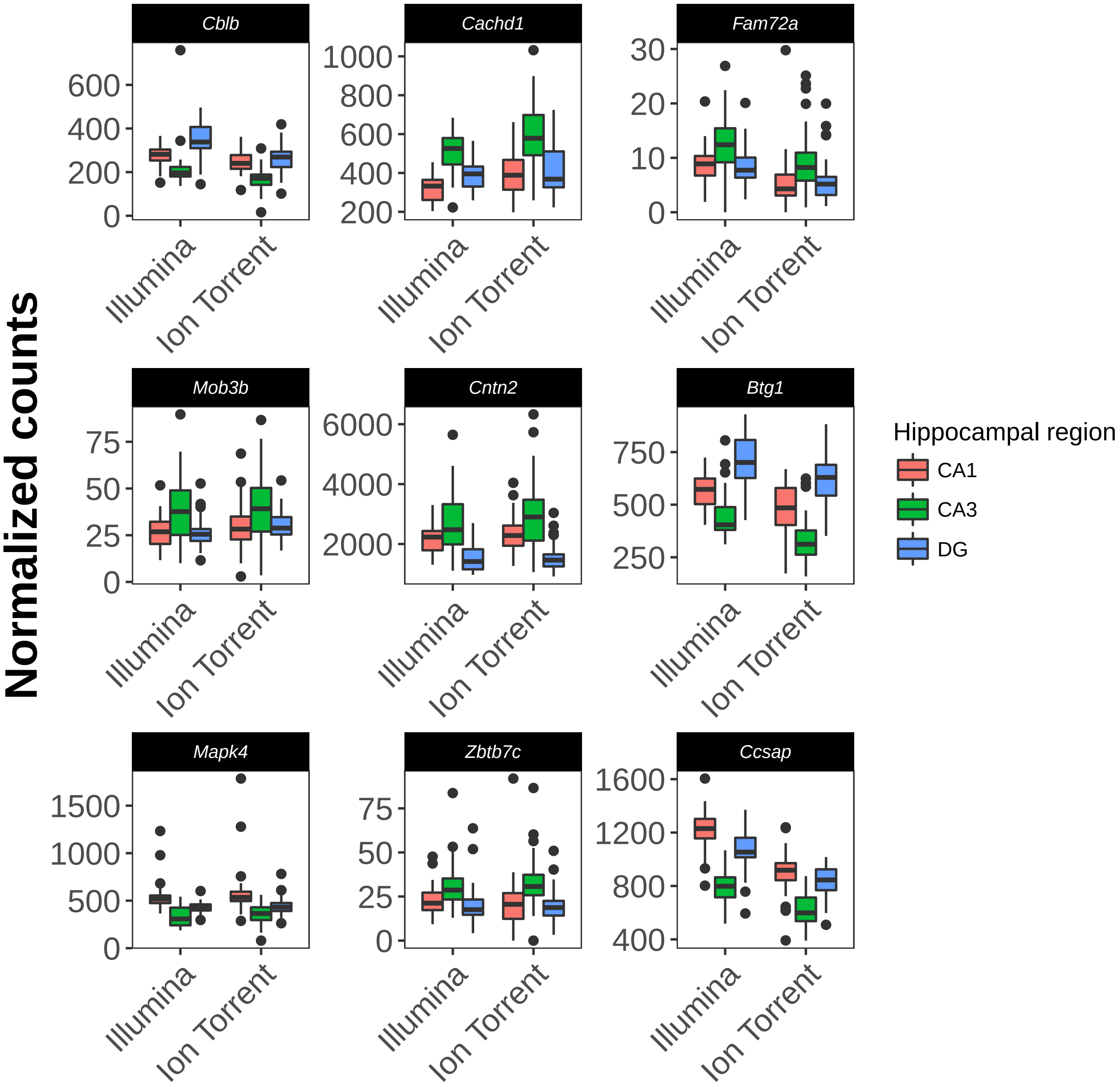

Normalized counts
Cblb
Cachd1
Fam72a
Mob3b
Cntn2
Btg1
Mapk4
Zbtb7c
Ccsap
Illumina
Ion Torrent
Hippocampal region
CA1
CA3
DG

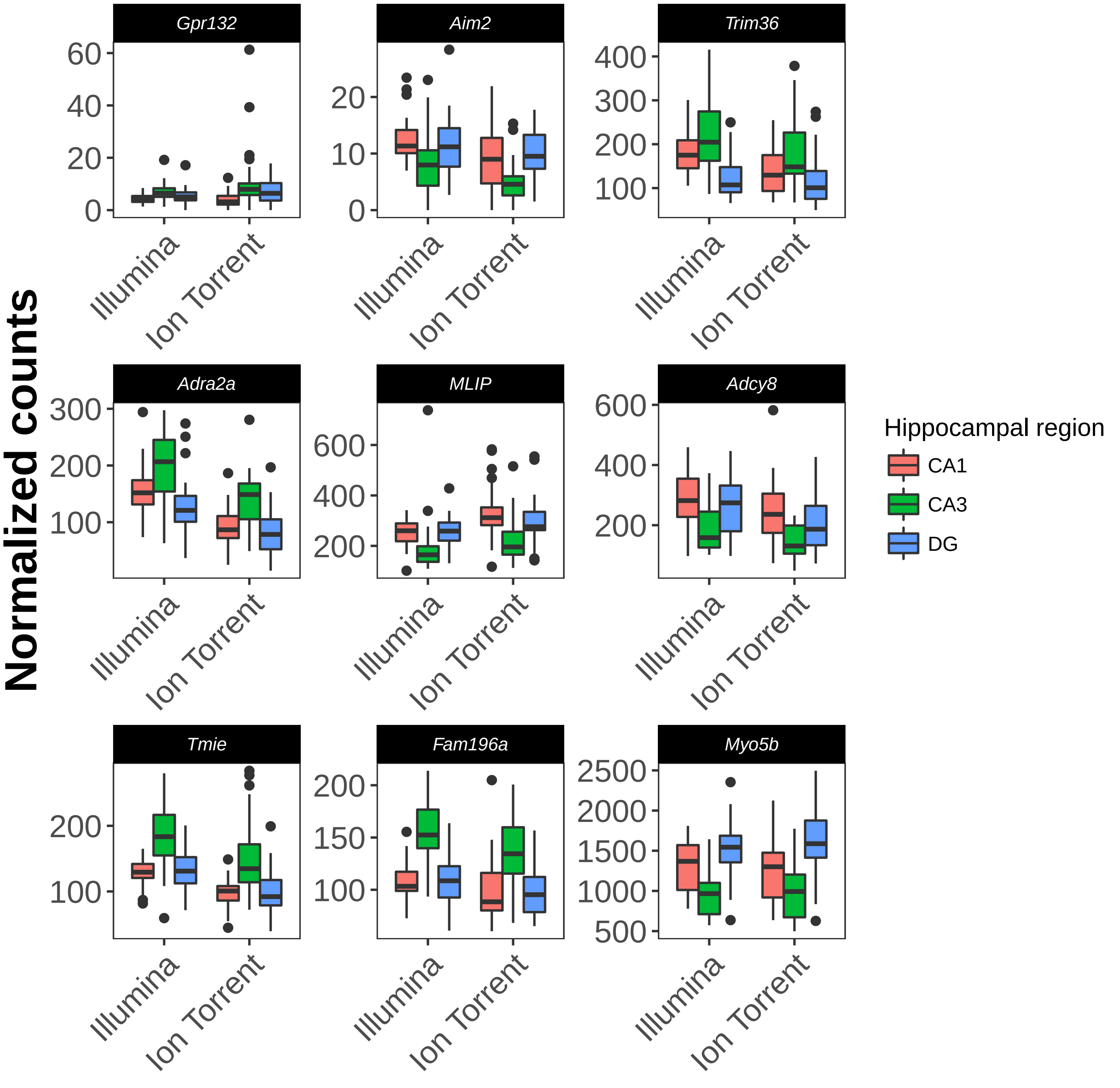
Normalized counts
Gpr132
Aim2
Trim36
Adra2a
MLIP
Adcy8
Tmie
Fam196a
Myo5b
Illumina
Ion Torrent
Hippocampal region
CA1
CA3
DG

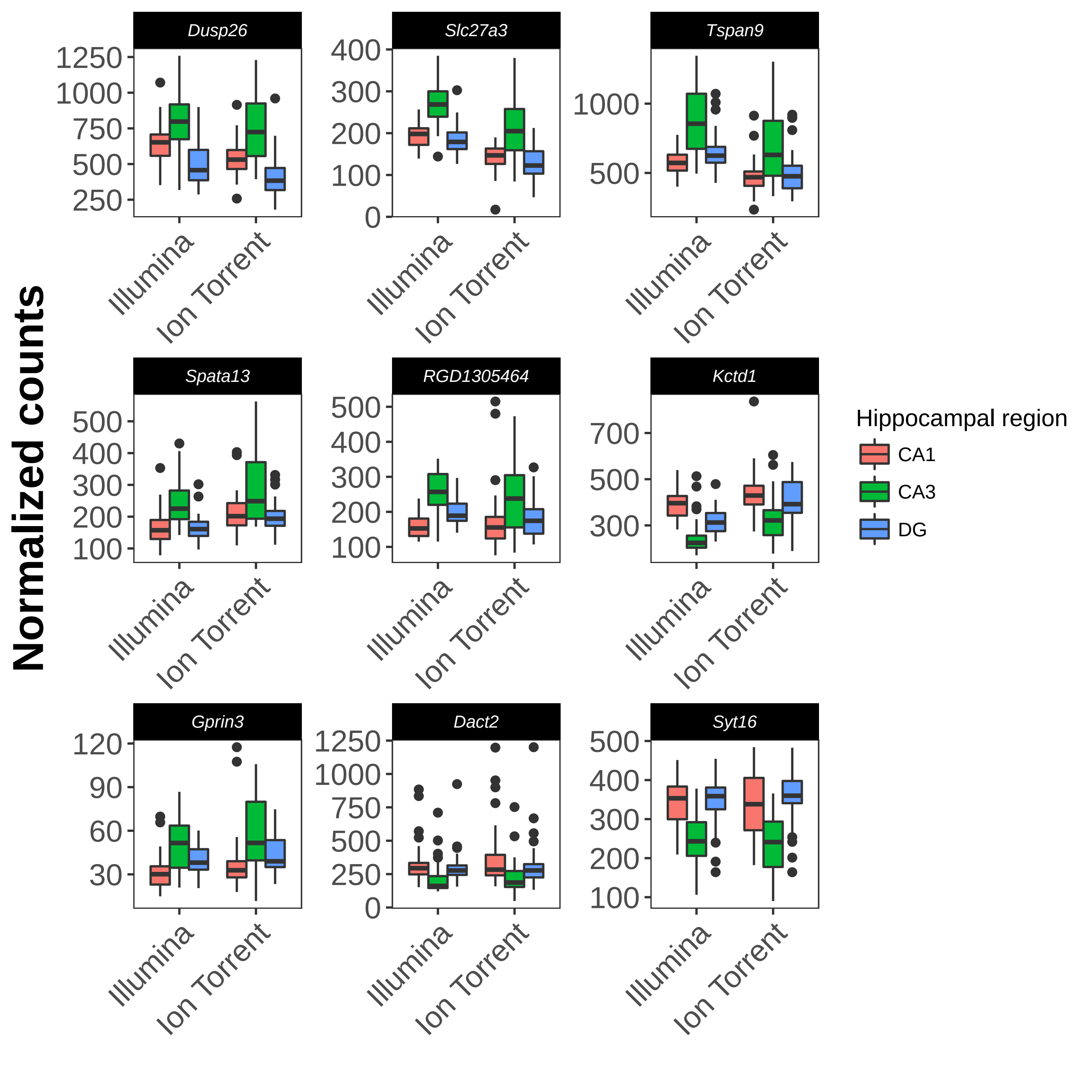
Normalized counts
Dusp26
Slc27a3
Tspan9
Spata13
RGD1305464
Kctd1
Gprin3
Dact2
Syt16
Illumina
Ion Torrent
Hippocampal region
CA1
CA3
DG

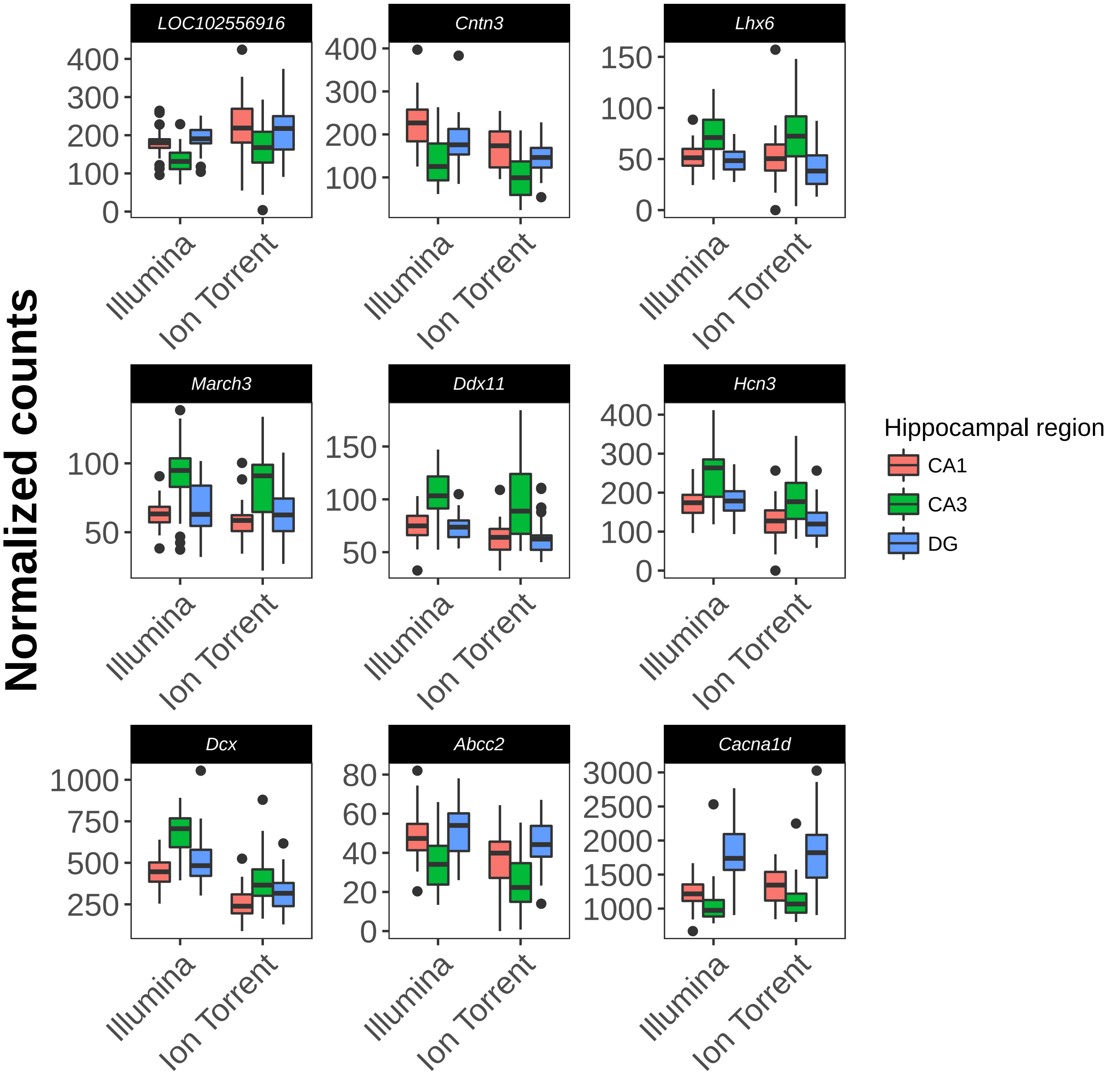

Normalized counts
LOC102556916
Cntn3
Lhx6
March3
Ddx11
Hcn3
Dcx
Abcc2
Cacna1d
Illumina
Ion Torrent
Hippocampal region
CA1
CA3
DG

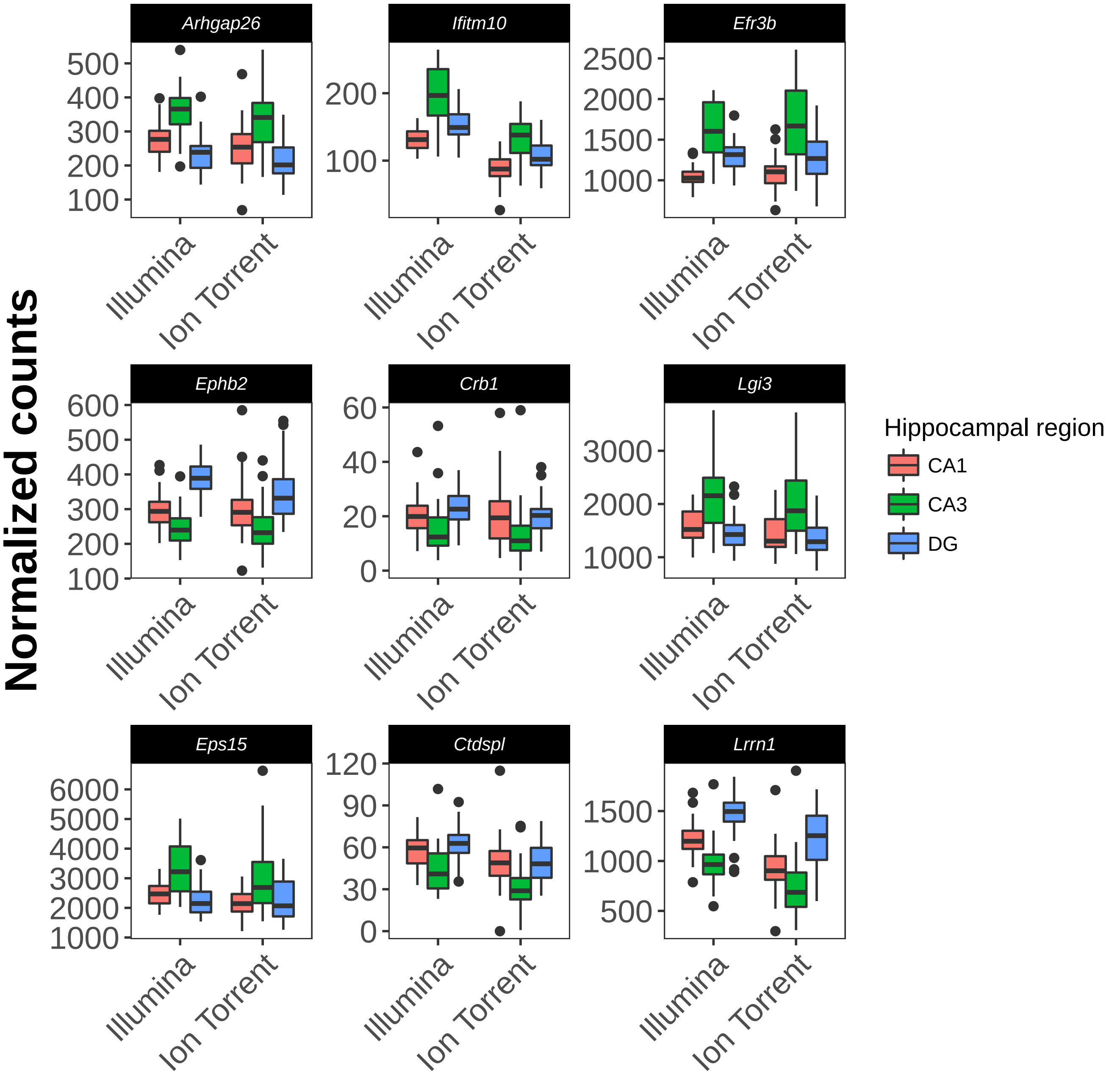

Normalized counts
Arhgap26
Ifitm10
Efr3b
Ephb2
Crb1
Lgi3
Eps15
Ctdspl
Lrrn1
Illumina
Ion Torrent
Hippocampal region
CA1
CA3
DG

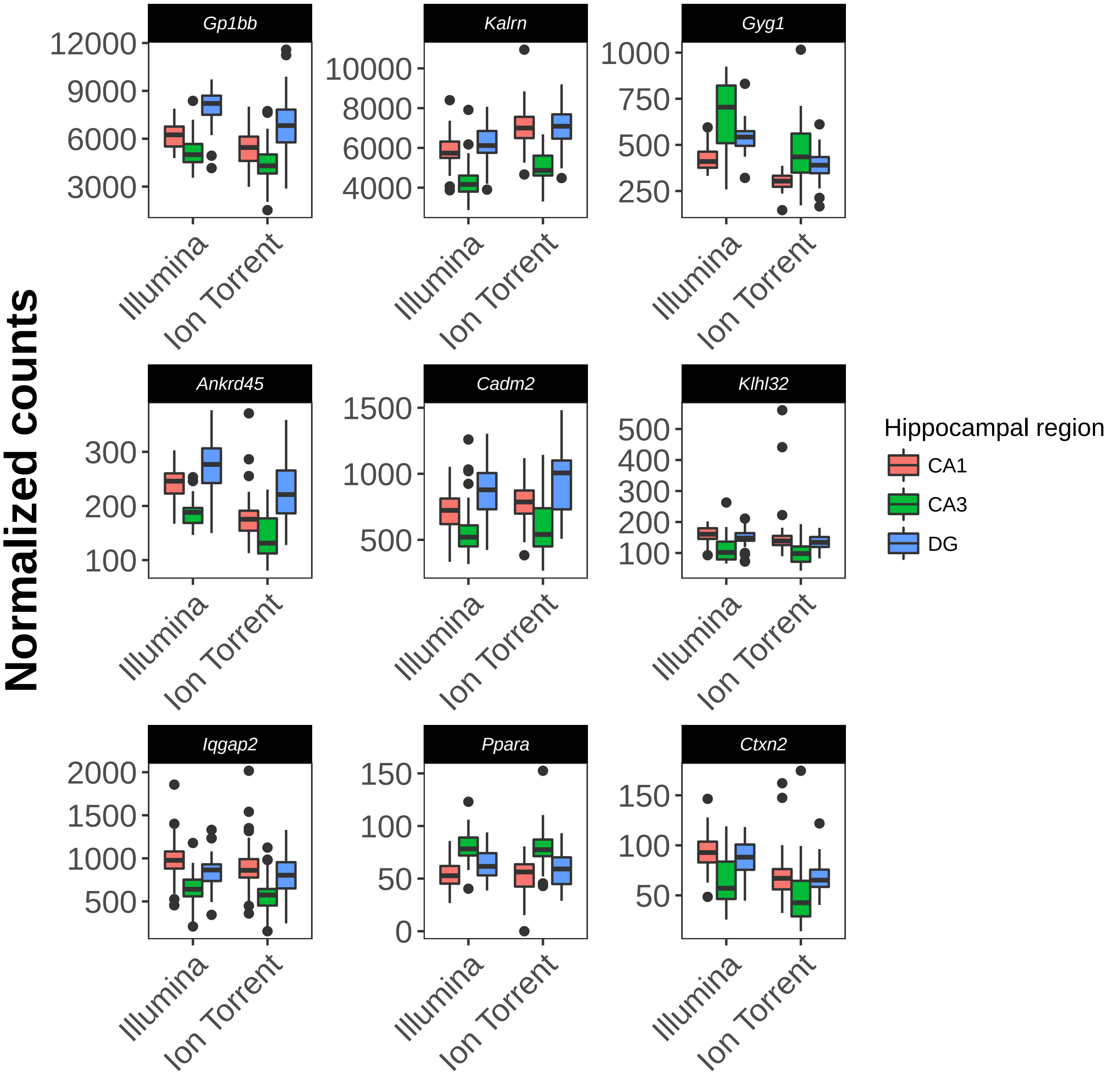
Normalized counts
Gp1bb
Kalrn
Gyg1
Ankrd45
Cadm2
Klhl32
Iqgap2
Ppara
Ctxn2
Illumina
Ion Torrent
Hippocampal region
CA1
CA3
DG

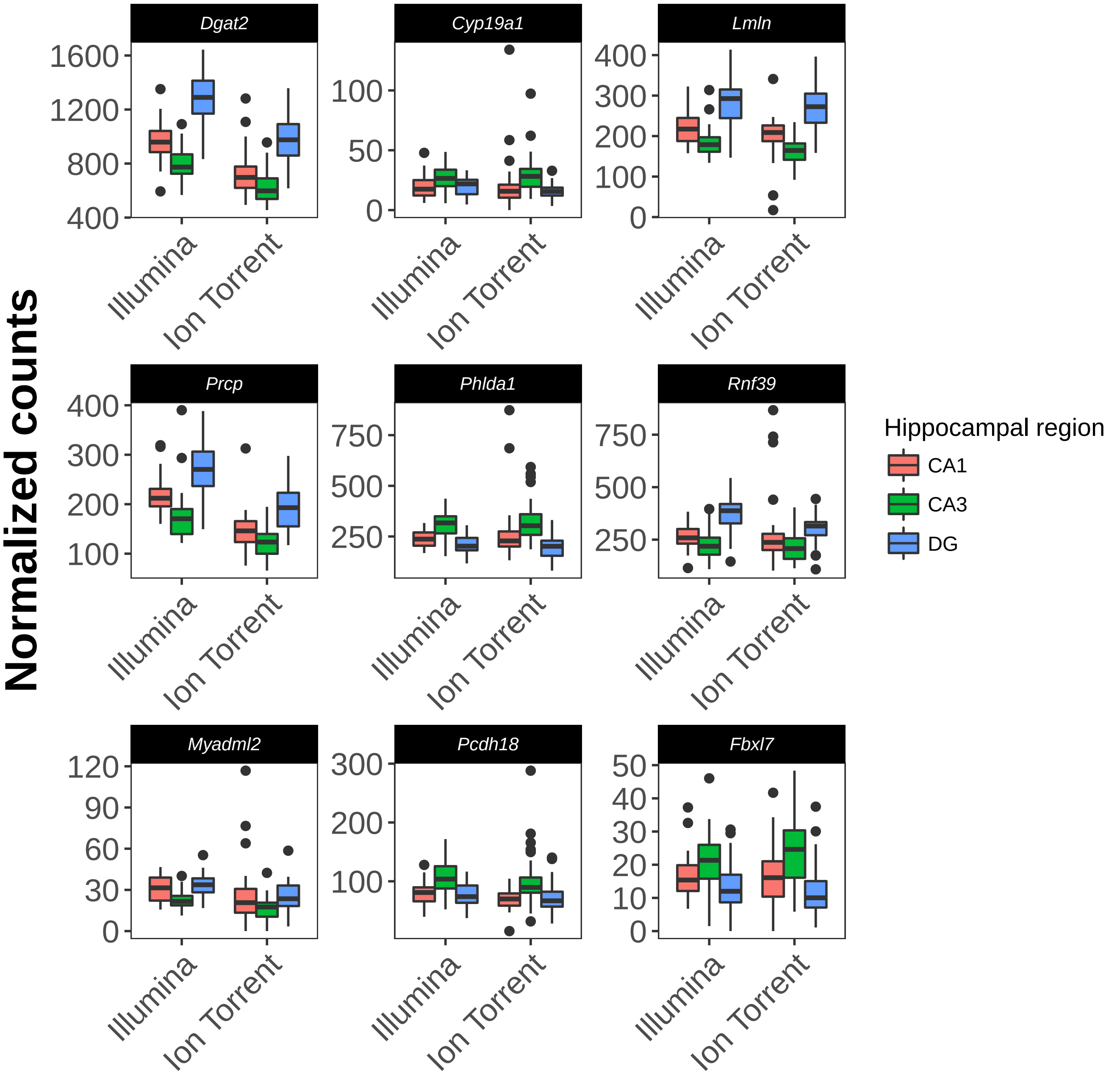
Normalized counts
Dgat2
Cyp19a1
Lmln
Prcp
Phlda1
Rnf39
Myadml2
Pcdh18
Fbxl7
Illumina
Ion Torrent
Hippocampal region
CA1
CA3
DG

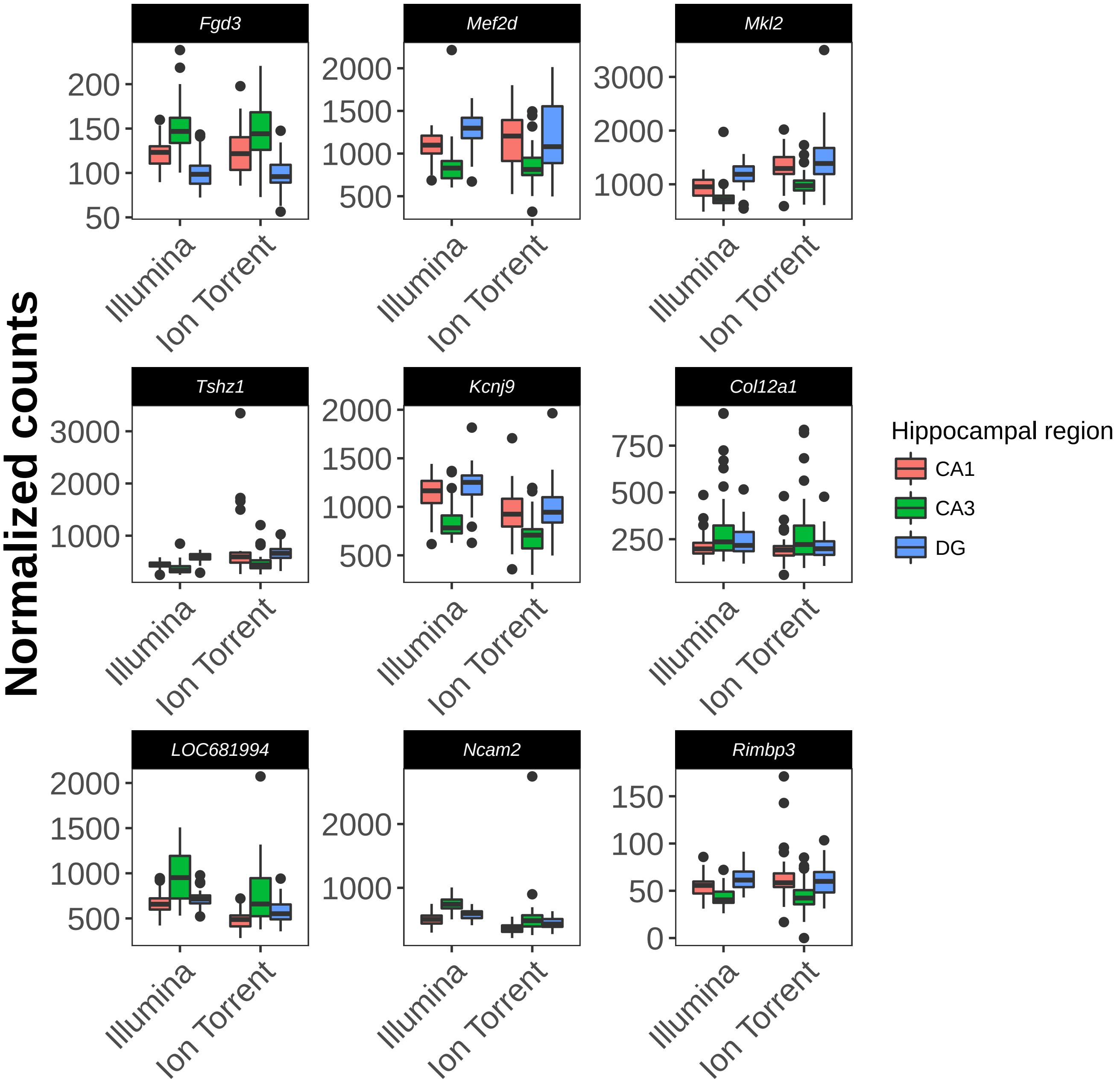

Normalized counts
Fgd3
Mef2d
Mkl2
Tshz1
Kcnj9
Col12a1
LOC681994
Ncam2
Rimbp3
Illumina
Ion Torrent
Hippocampal region
CA1
CA3
DG

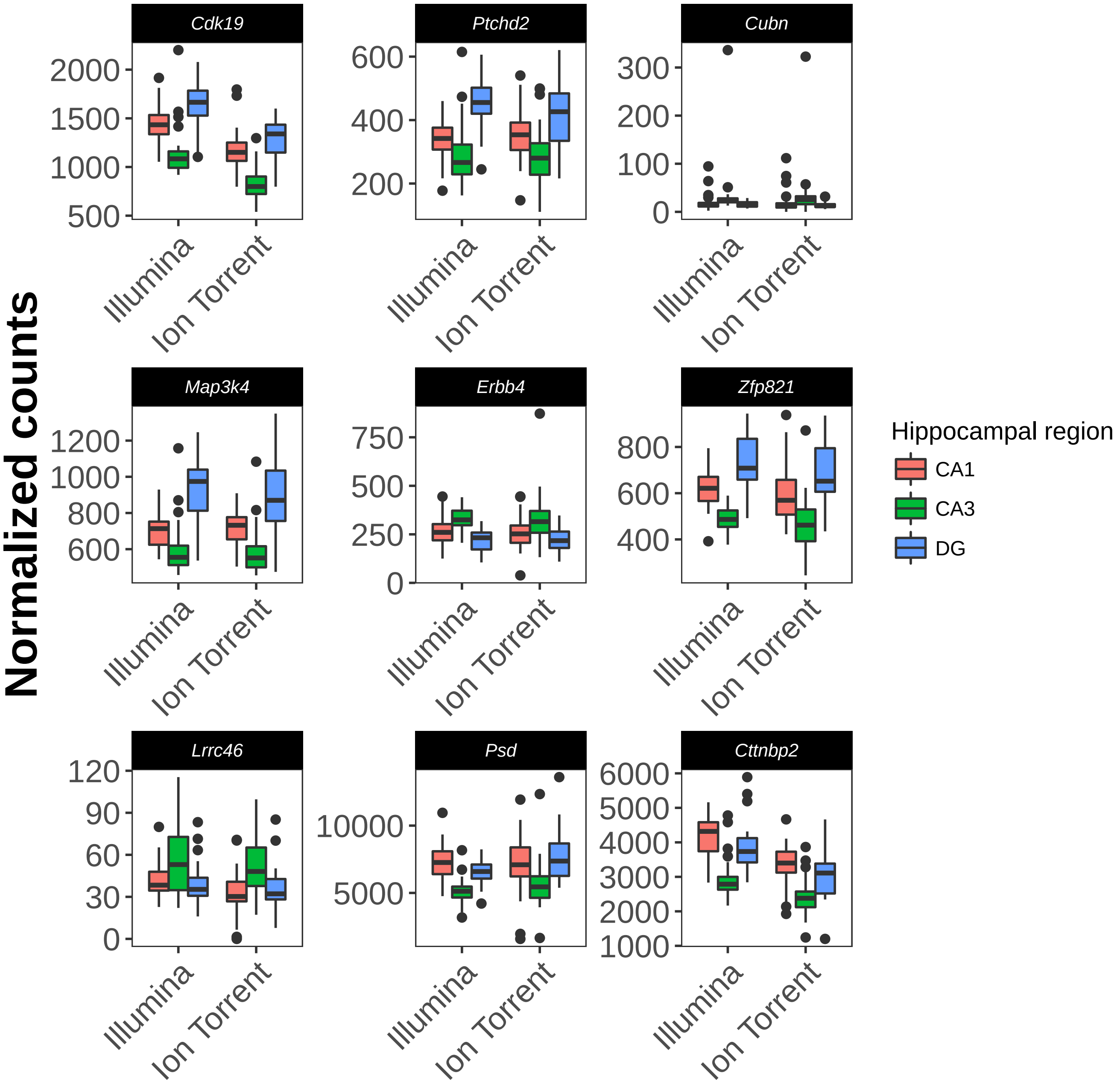
Normalized counts
Cdk19
Ptchd2
Cubn
Map3k4
Erbb4
Zfp821
Lrrc46
Psd
Cttnbp2
Illumina
Ion Torrent
Hippocampal region
CA1
CA3
DG

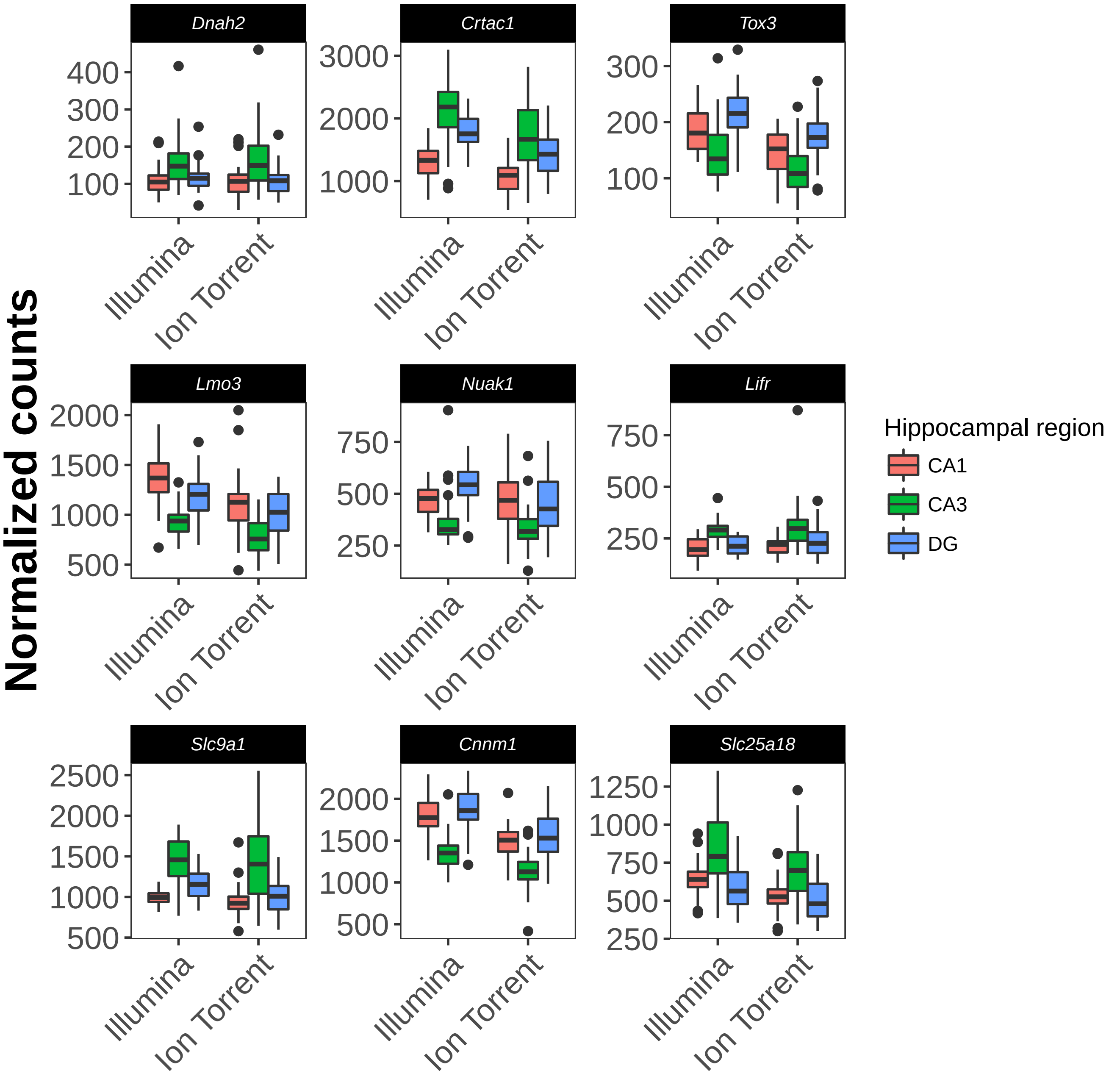

Normalized counts
Dnah2
Crtac1
Tox3
Lmo3
Nuak1
Lifr
Slc9a1
Cnnm1
Slc25a18
Illumina
Ion Torrent
Hippocampal region
CA1
CA3
DG

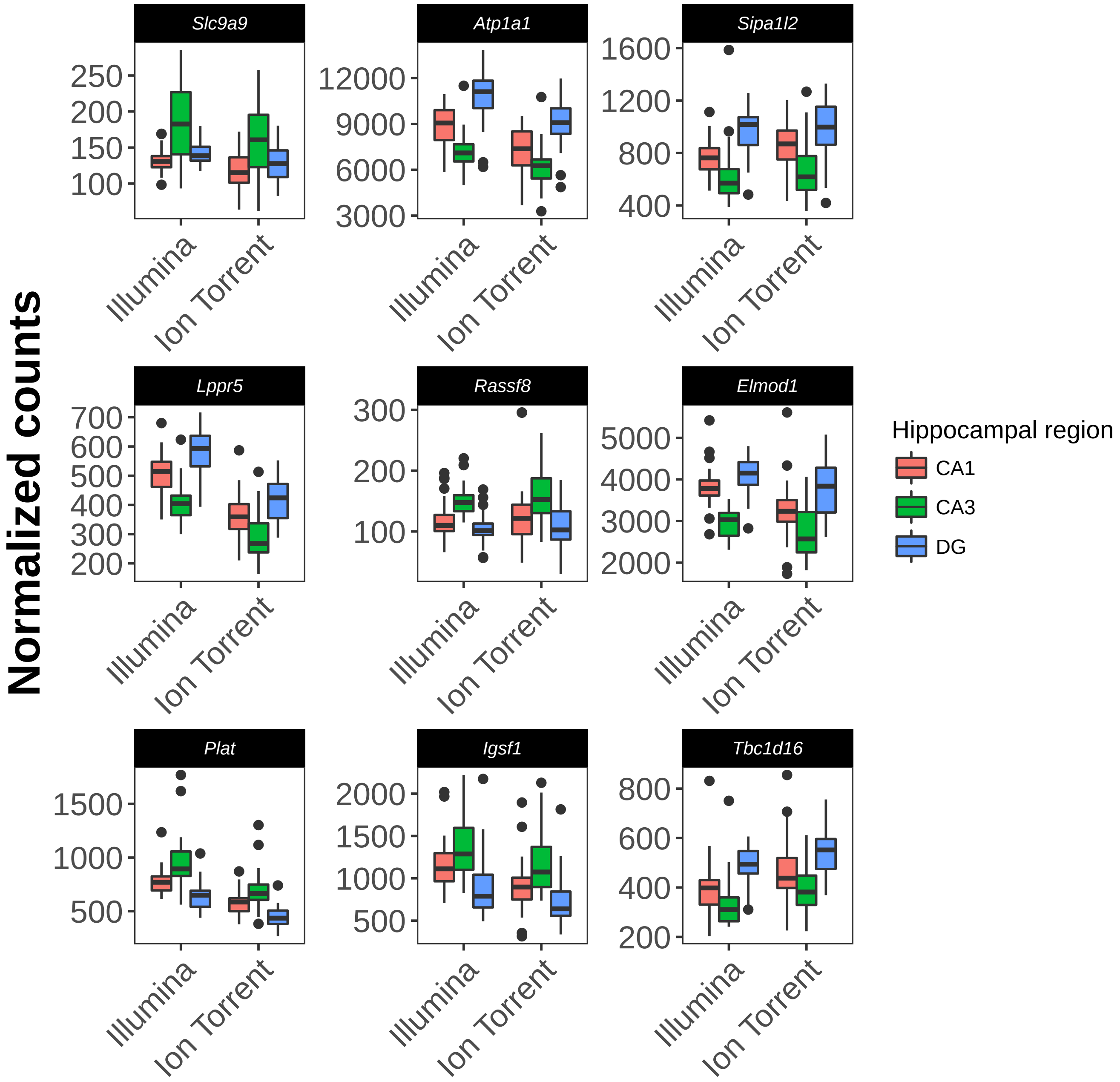
Slc9a9
Atp1a1
Sipa1l2
Lppr5
Rassf8
Elmod1
Plat
Igsf1
Tbc1d16
Illumina
Ion Torrent
Normalized counts
Hippocampal region
CA1
CA3
DG

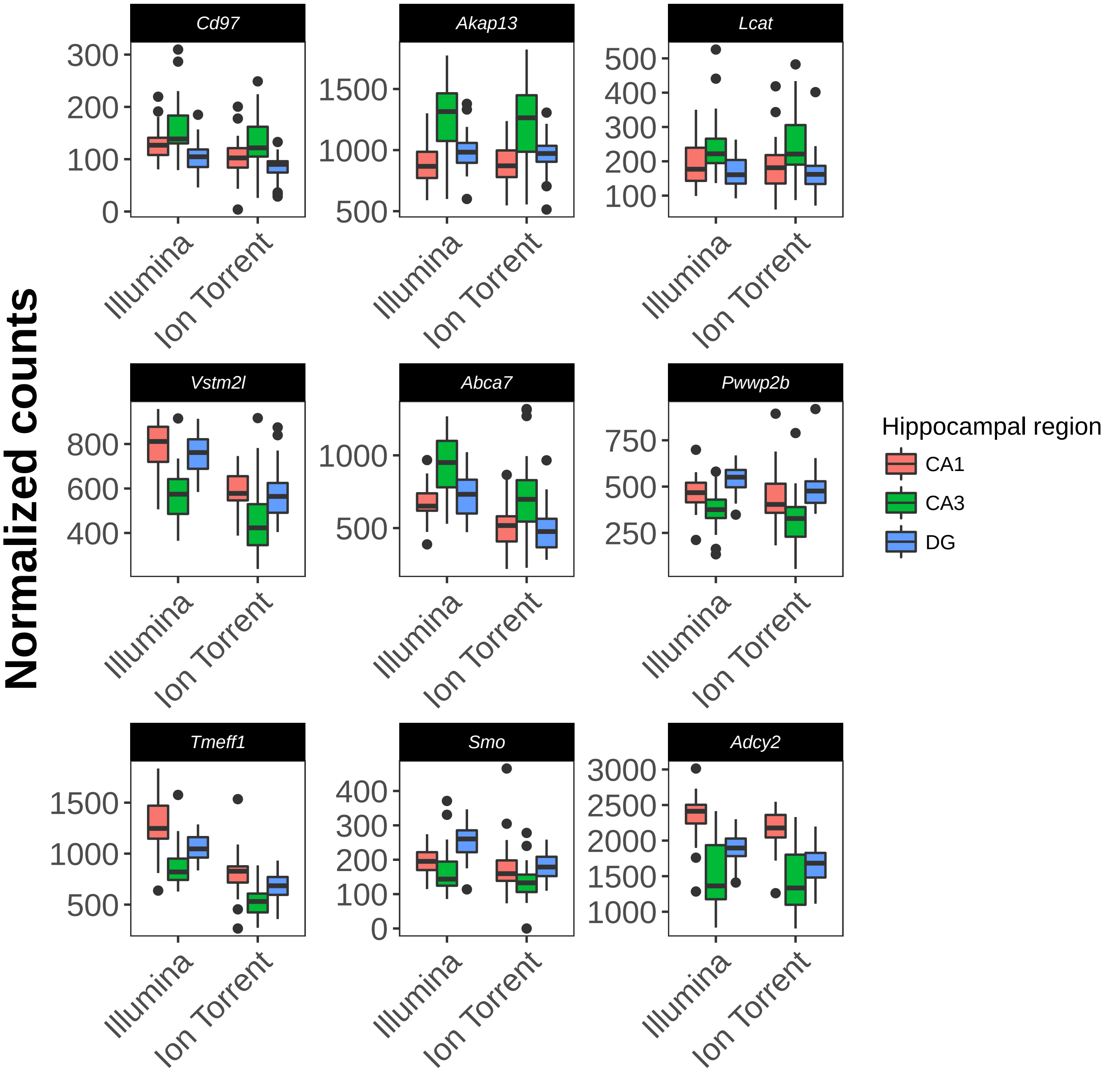

Normalized counts
Cd97
Akap13
Lcat
Vstm2l
Abca7
Pwwp2b
Tmeff1
Smo
Adcy2
Illumina
Ion Torrent
Hippocampal region
CA1
CA3
DG

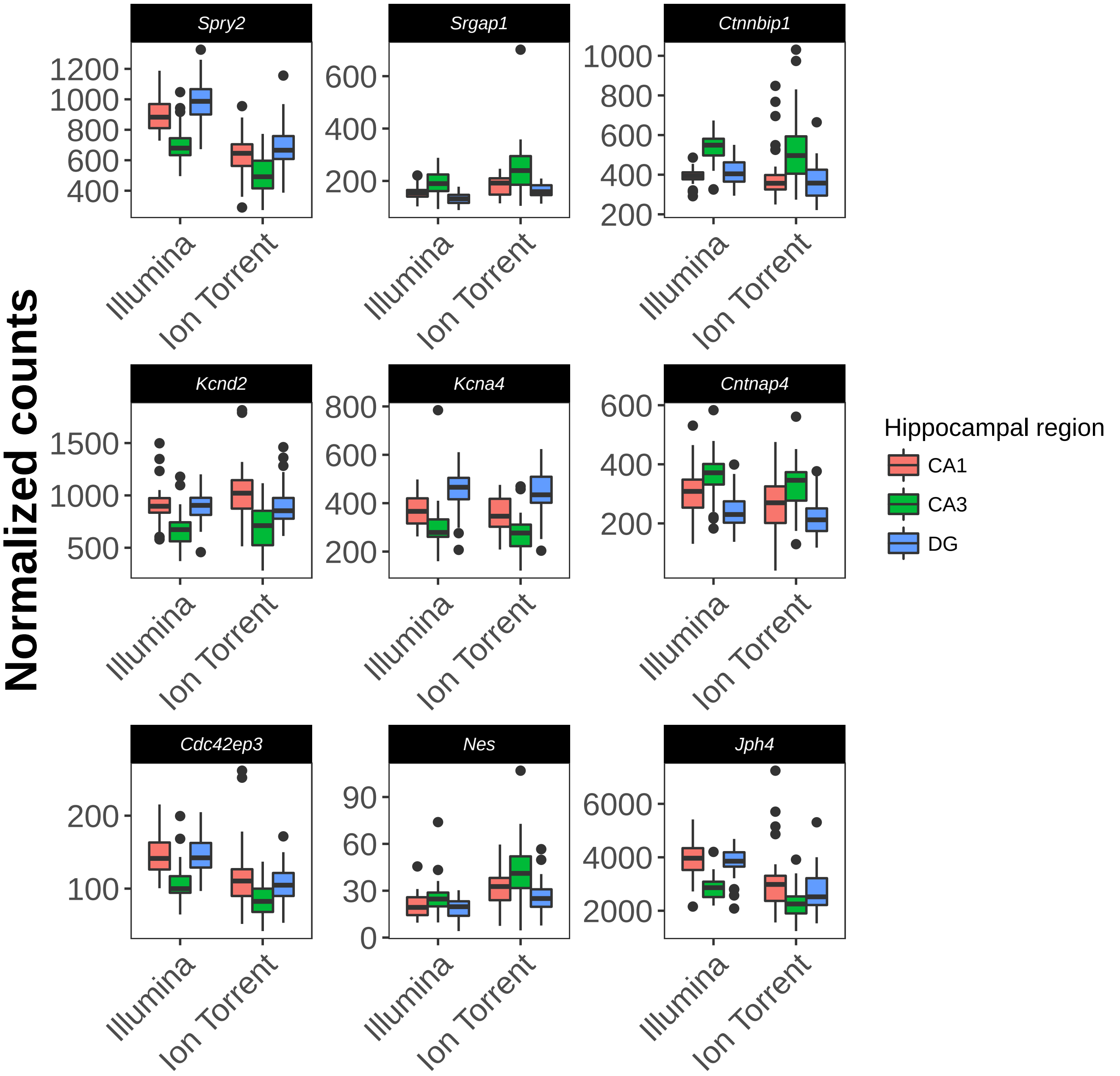

Normalized counts
Spry2
Srgap1
Ctnnbip1
Kcnd2
Kcna4
Cntnap4
Cdc42ep3
Nes
Jph4
Illumina
Ion Torrent
Hippocampal region
CA1
CA3
DG

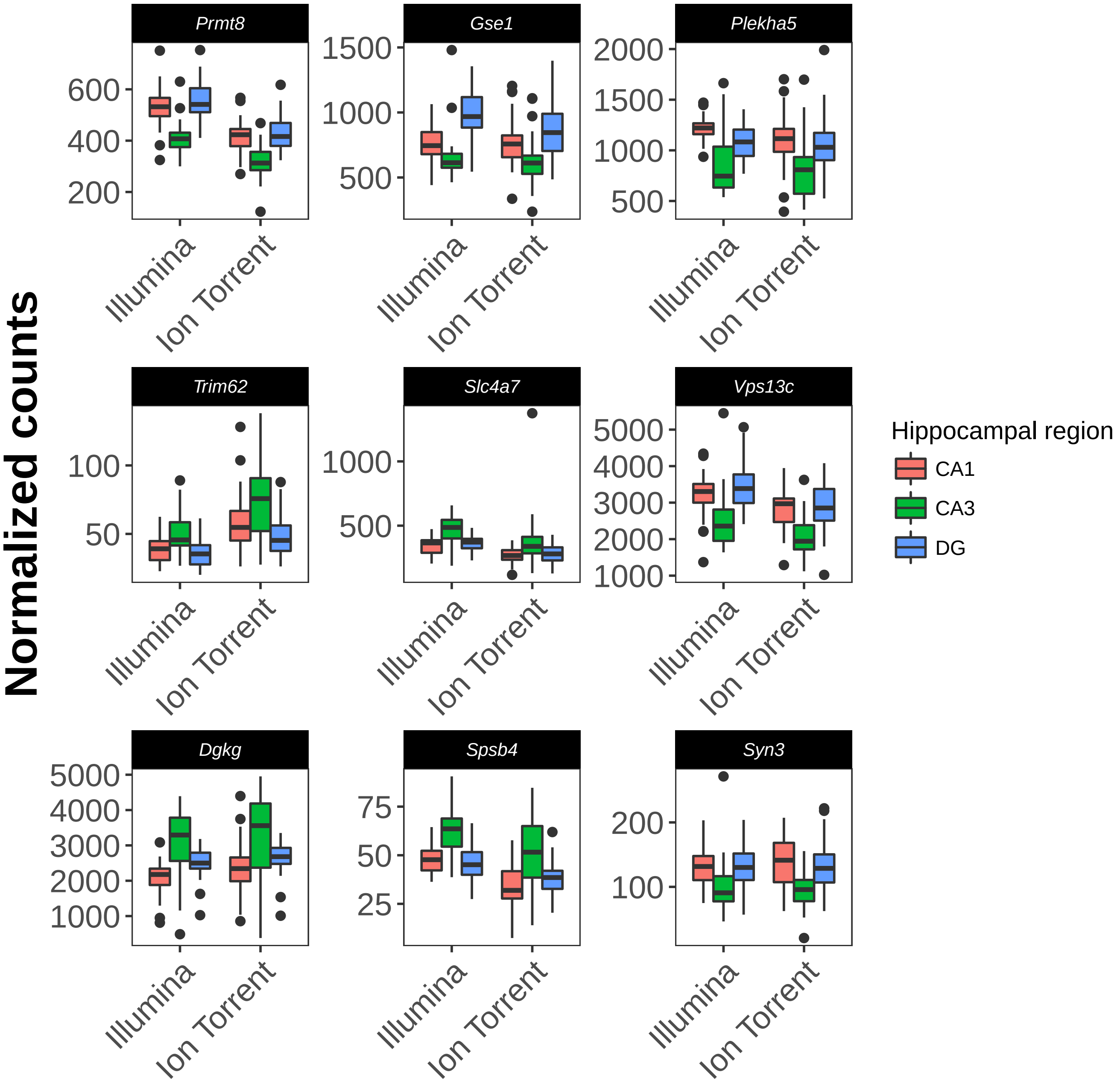

Normalized counts
Prmt8
Gse1
Plekha5
Trim62
Slc4a7
Vps13c
Dgkg
Spsb4
Syn3
Illumina
Ion Torrent
Hippocampal region
CA1
CA3
DG

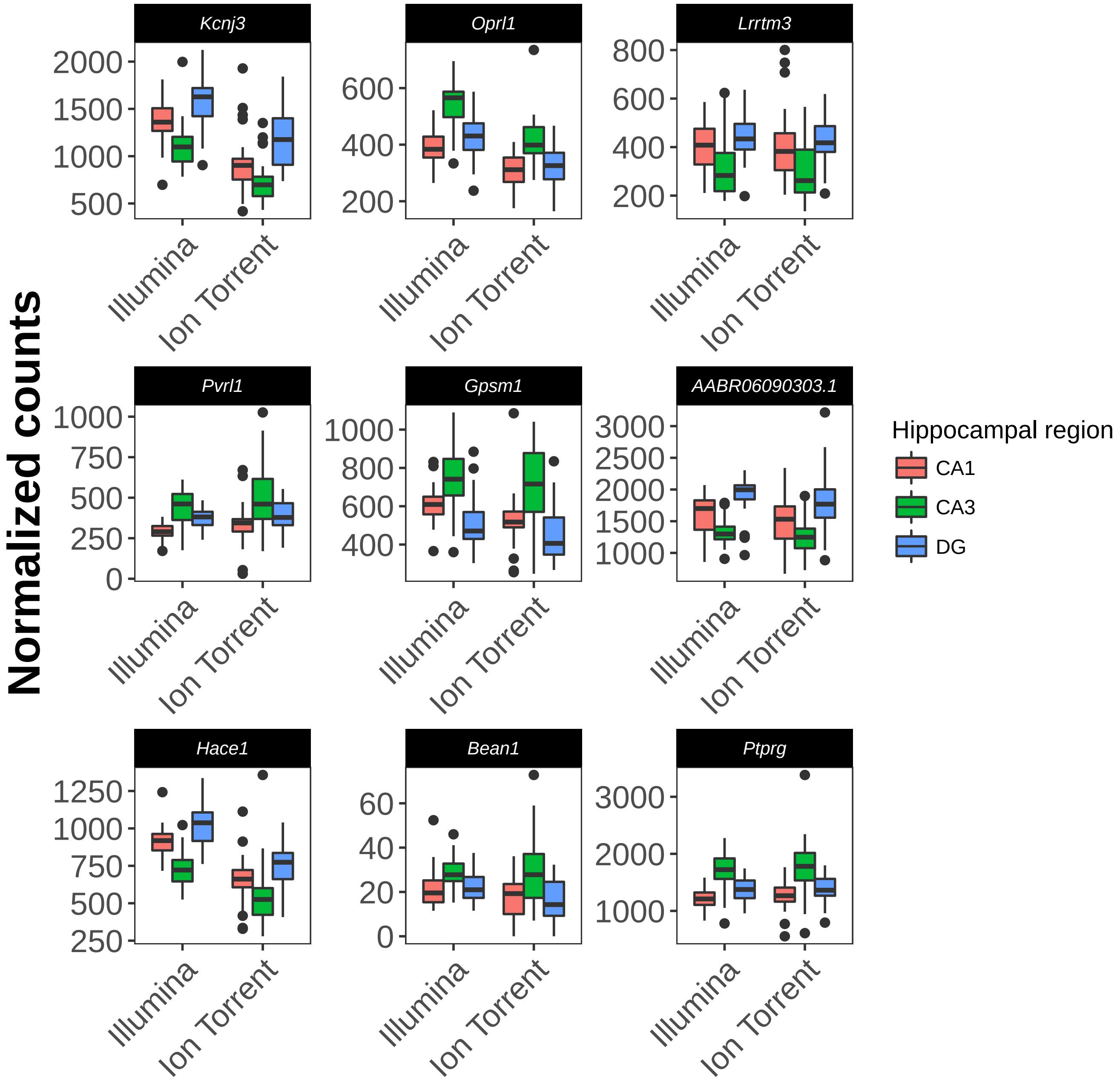

Normalized counts
Kcnj3
Oprl1
Lrrtm3
Pvrl1
Gpsm1
AABR06090303.1
Hace1
Bean1
Ptprg
Illumina
Ion Torrent
Hippocampal region
CA1
CA3
DG

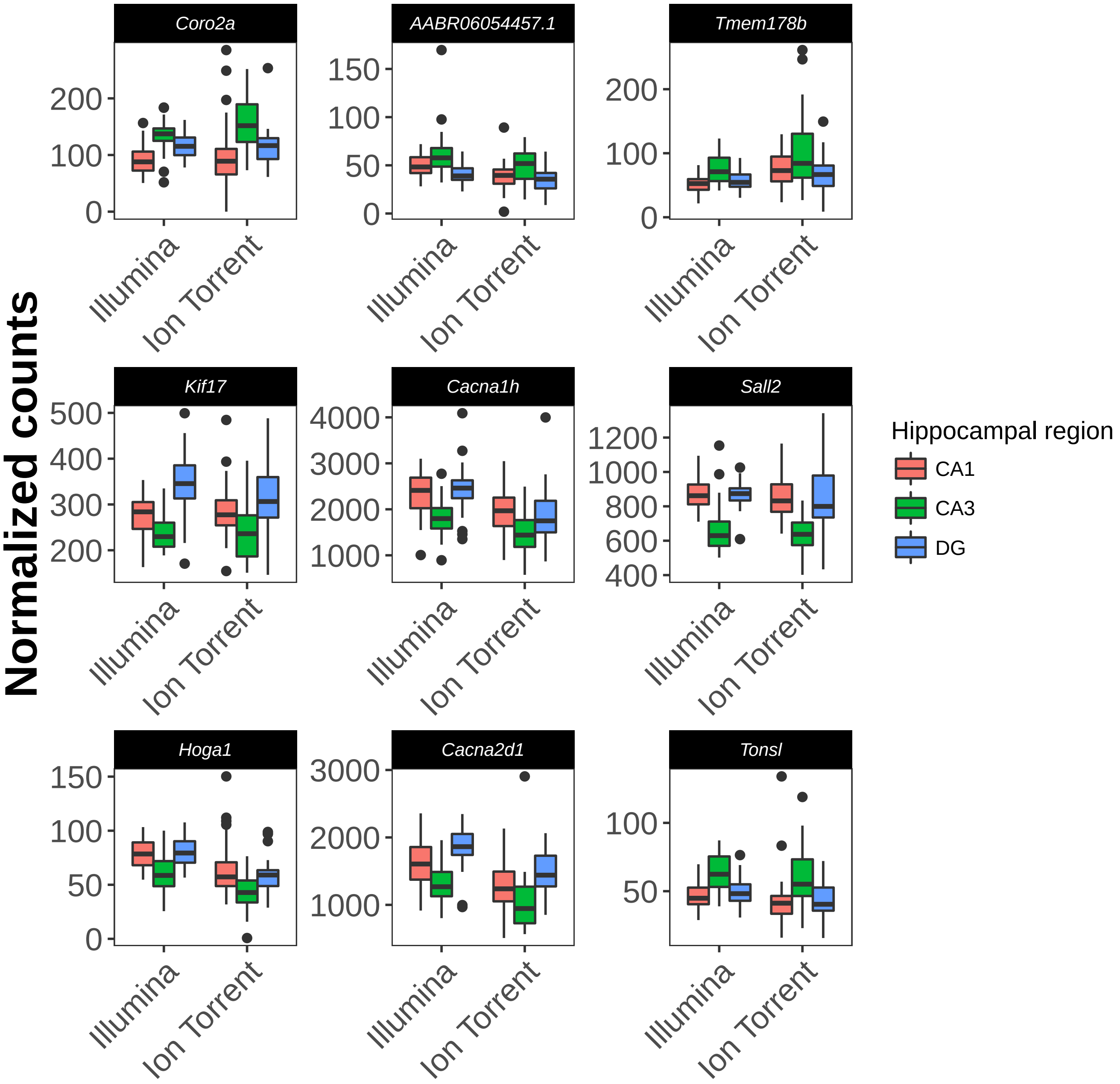

Normalized counts
Coro2a
AABR06054457.1
Tmem178b
Kif17
Cacna1h
Sall2
Hoga1
Cacna2d1
Tonsl
Illumina
Ion Torrent
Hippocampal region
CA1
CA3
DG

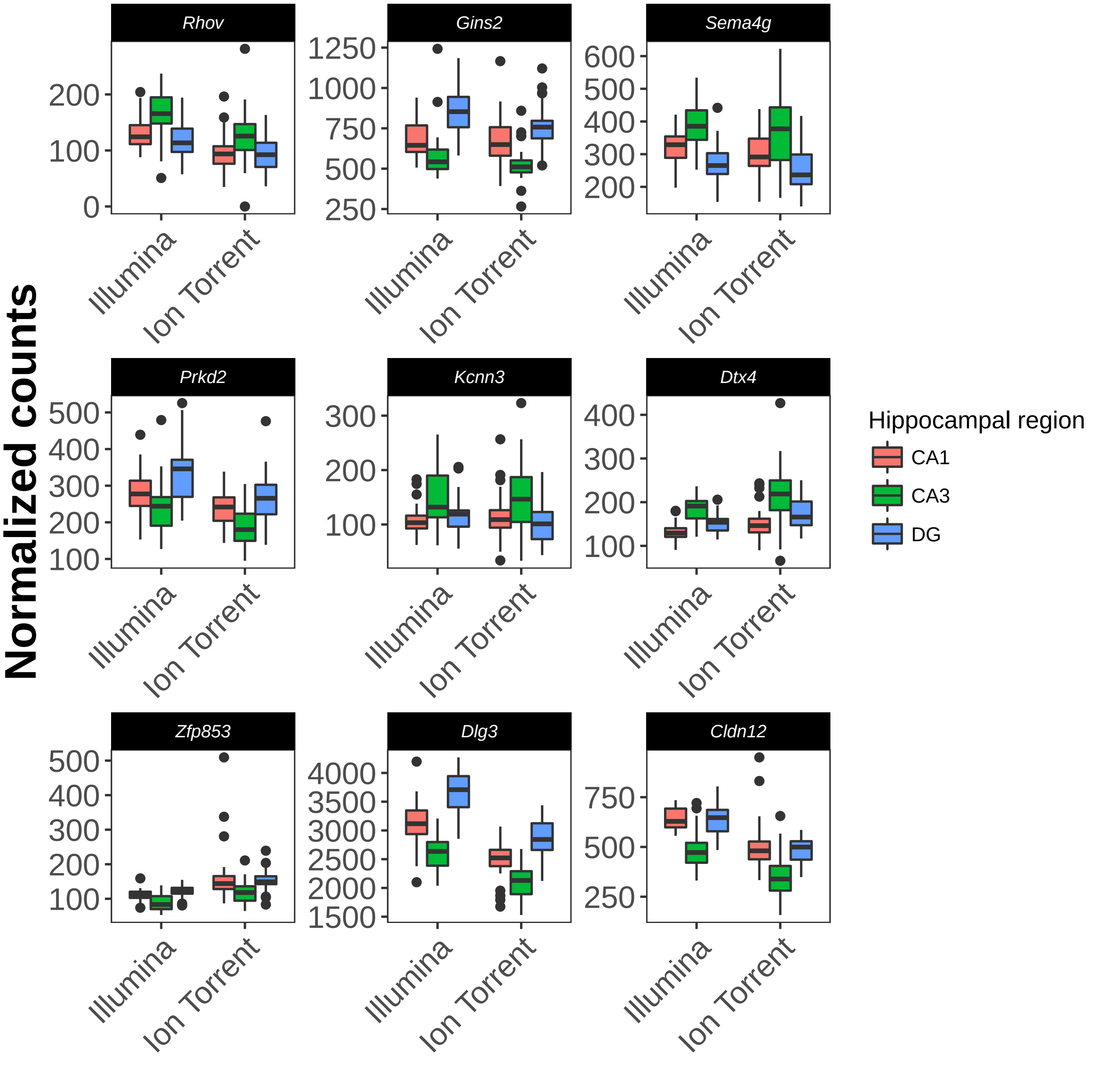

Normalized counts
Rhov
Gins2
Sema4g
Prkd2
Kcnn3
Dtx4
Zfp853
Dlg3
Cldn12
Illumina
Ion Torrent
Hippocampal region
CA1
CA3
DG

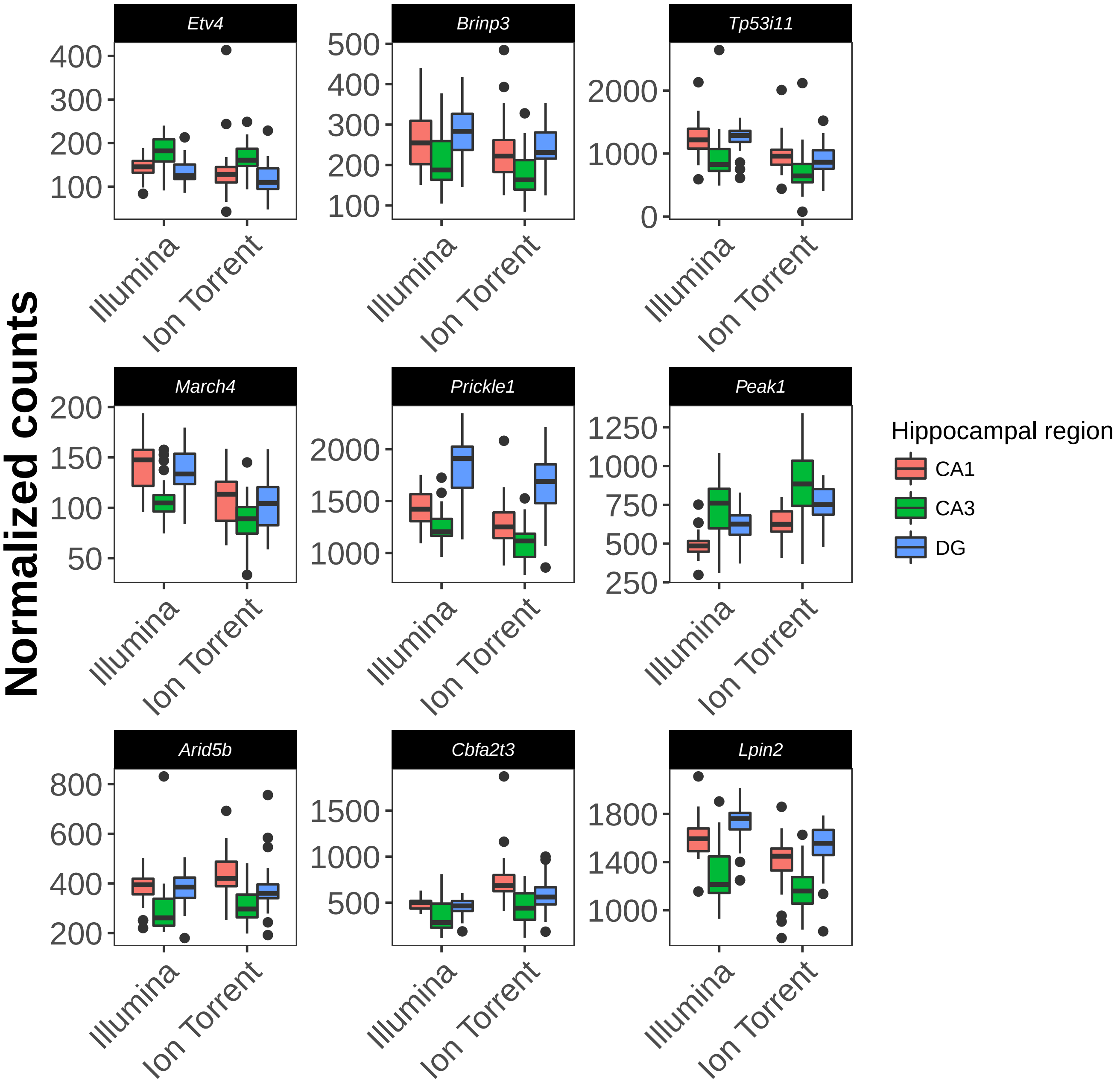

Normalized counts
Etv4
Brinp3
Tp53i11
March4
Prickle1
Peak1
Arid5b
Cbfa2t3
Lpin2
Illumina
Ion Torrent
Hippocampal region
CA1
CA3
DG

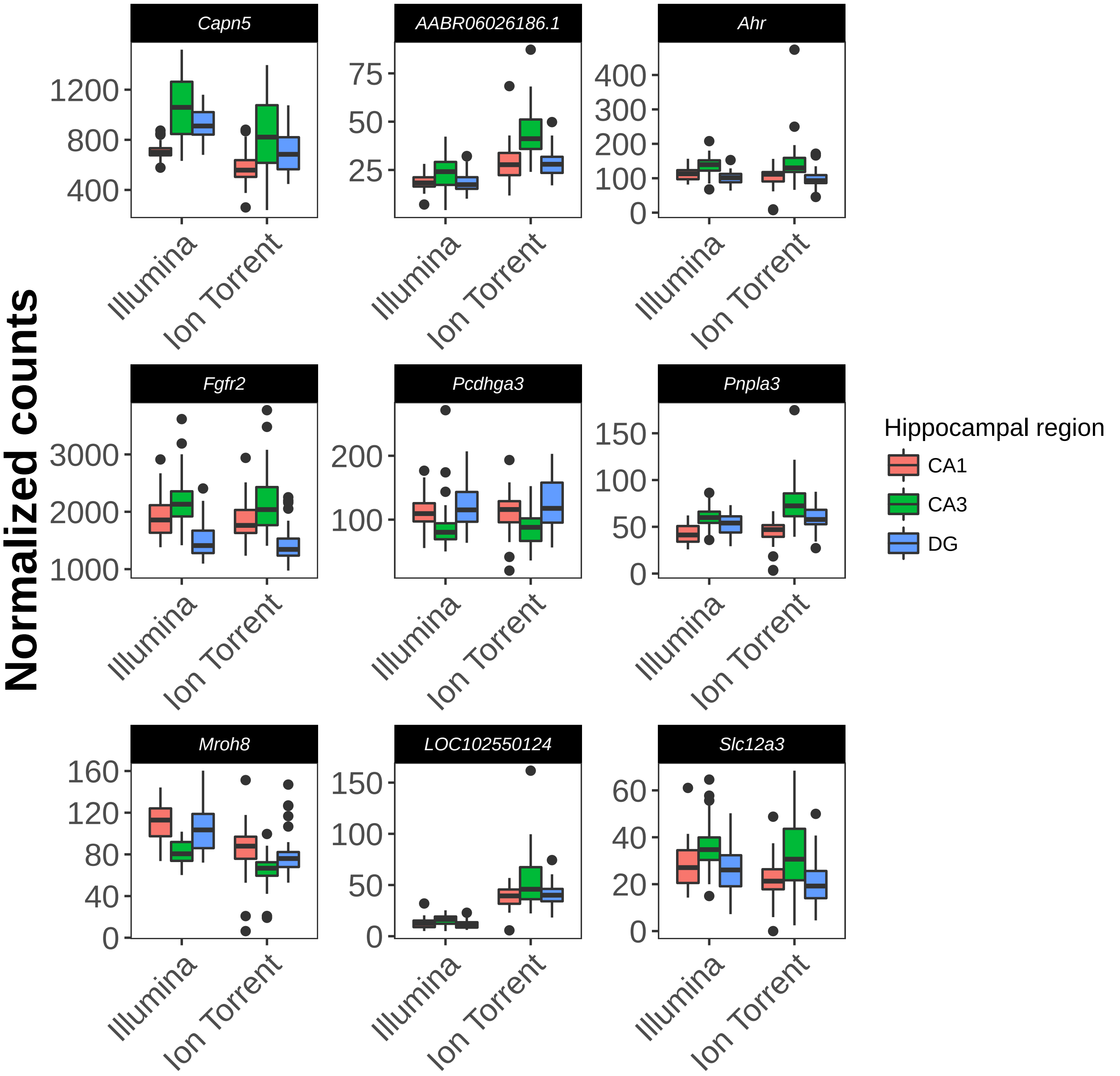

Normalized counts
Capn5
AABR06026186.1
Ahr
Fgfr2
Pcdhga3
Pnpla3
Mroh8
LOC102550124
Slc12a3
Illumina
Ion Torrent
Hippocampal region
CA1
CA3
DG

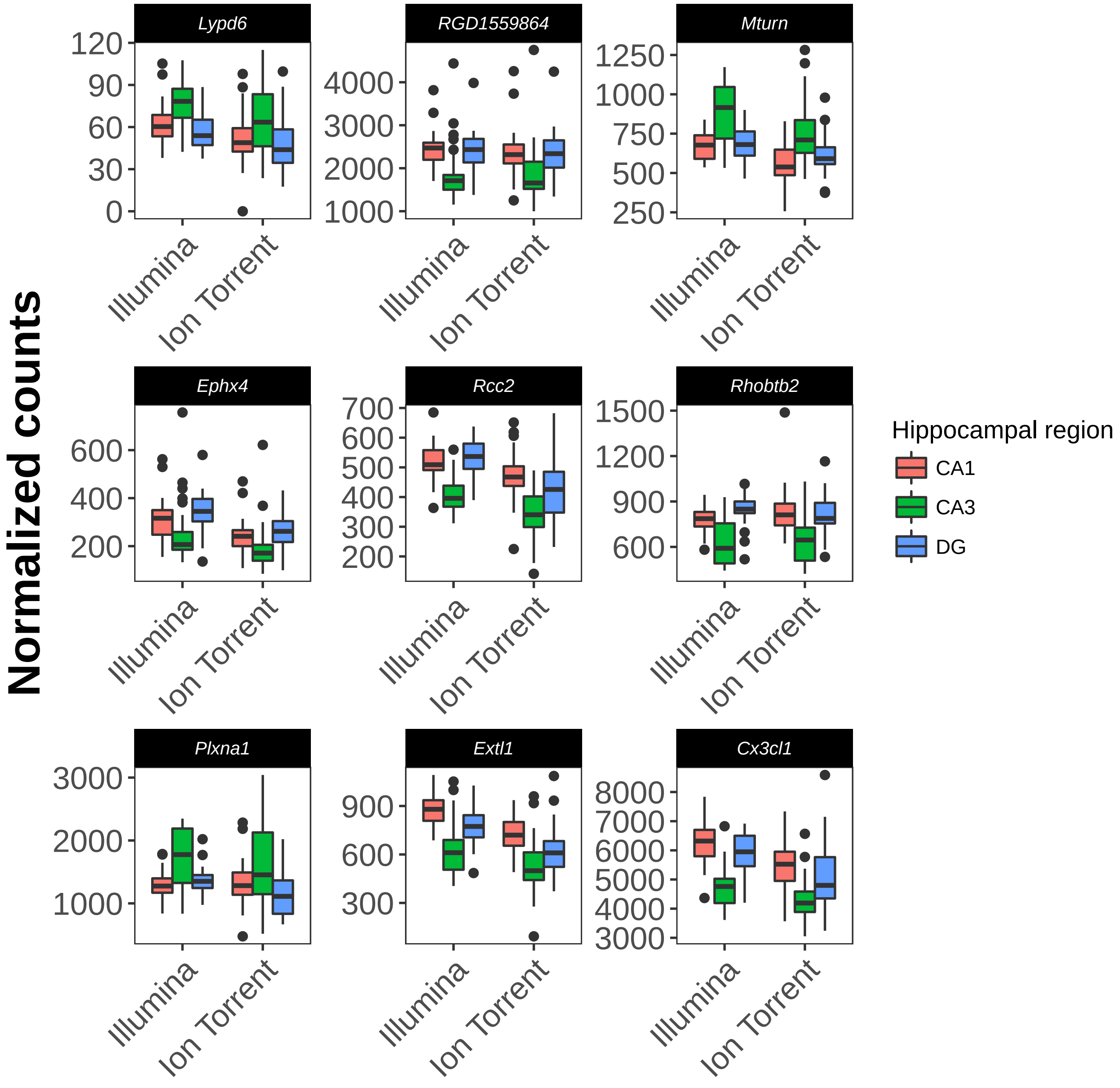

Normalized counts
Lypd6
RGD1559864
Mturn
Ephx4
Rcc2
Rhobtb2
Plxna1
Extl1
Cx3cl1
Illumina
Ion Torrent
Hippocampal region
CA1
CA3
DG

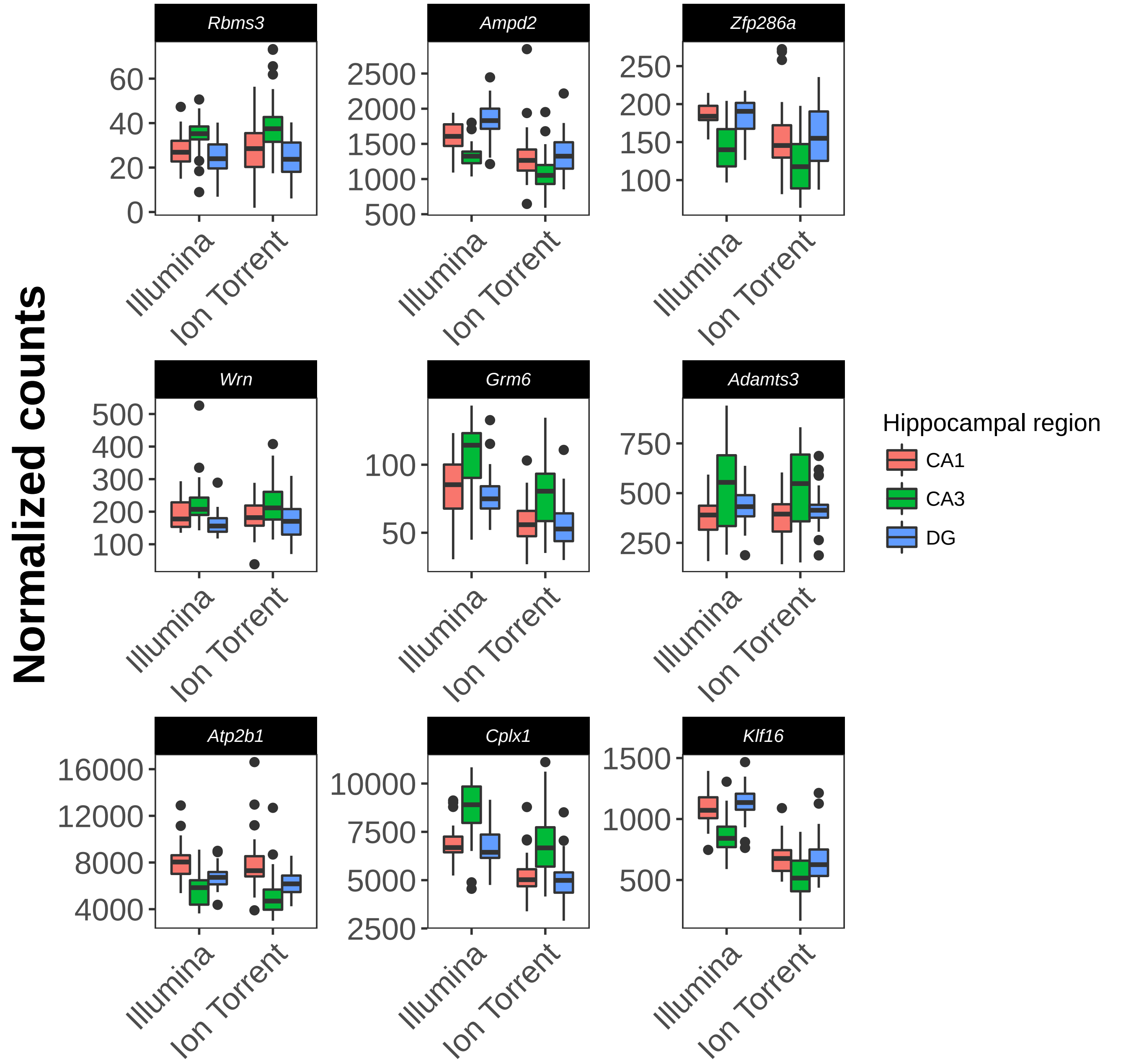
Normalized counts
Rbms3
Ampd2
Zfp286a
Wrn
Grm6
Adamts3
Atp2b1
Cplx1
Klf16
Illumina
Ion Torrent
Hippocampal region
CA1
CA3
DG

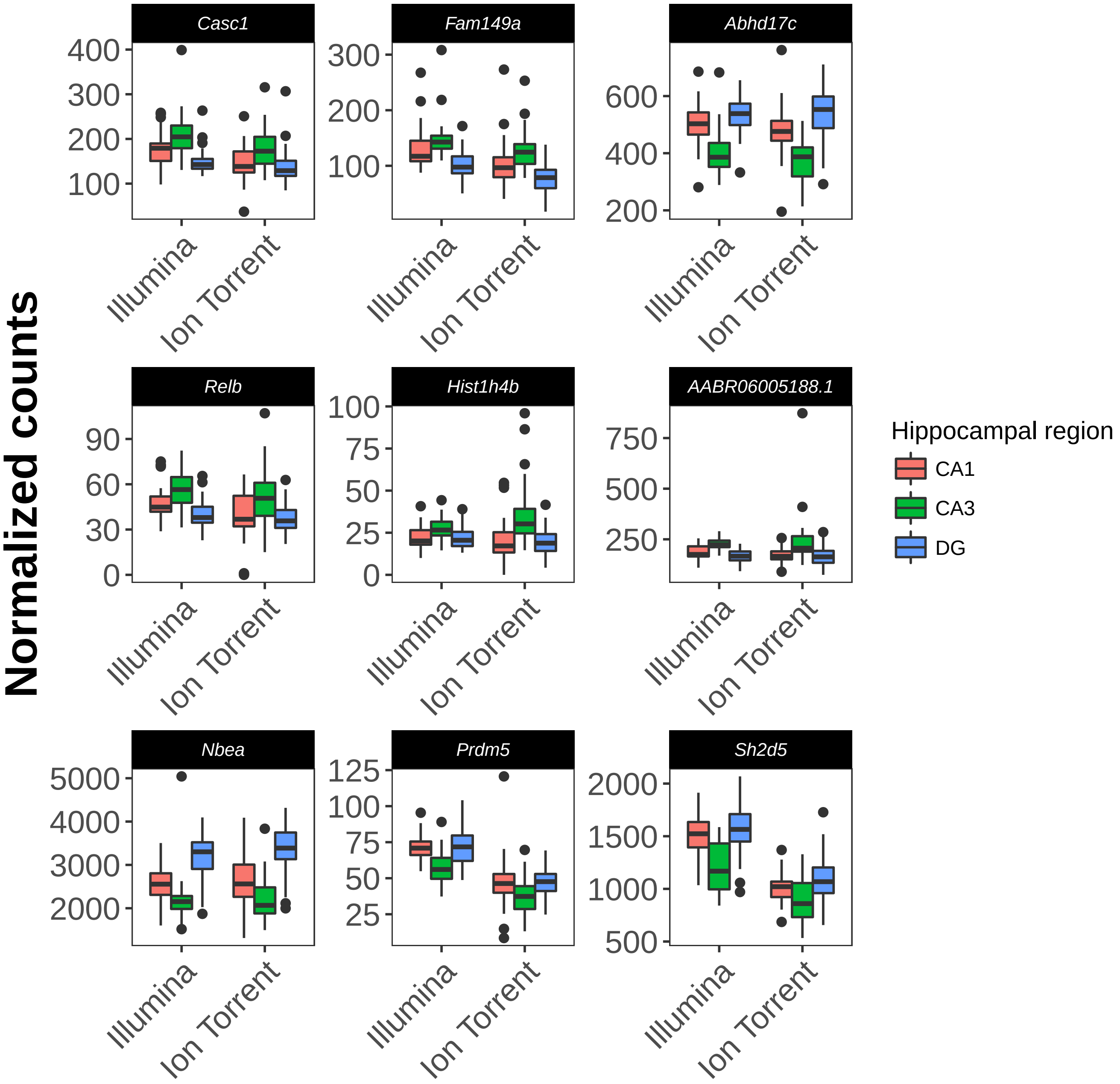
Normalized counts
Casc1
Fam149a
Abhd17c
Relb
Hist1h4b
AABR06005188.1
Nbea
Prdm5
Sh2d5
Illumina
Ion Torrent
Hippocampal region
CA1
CA3
DG

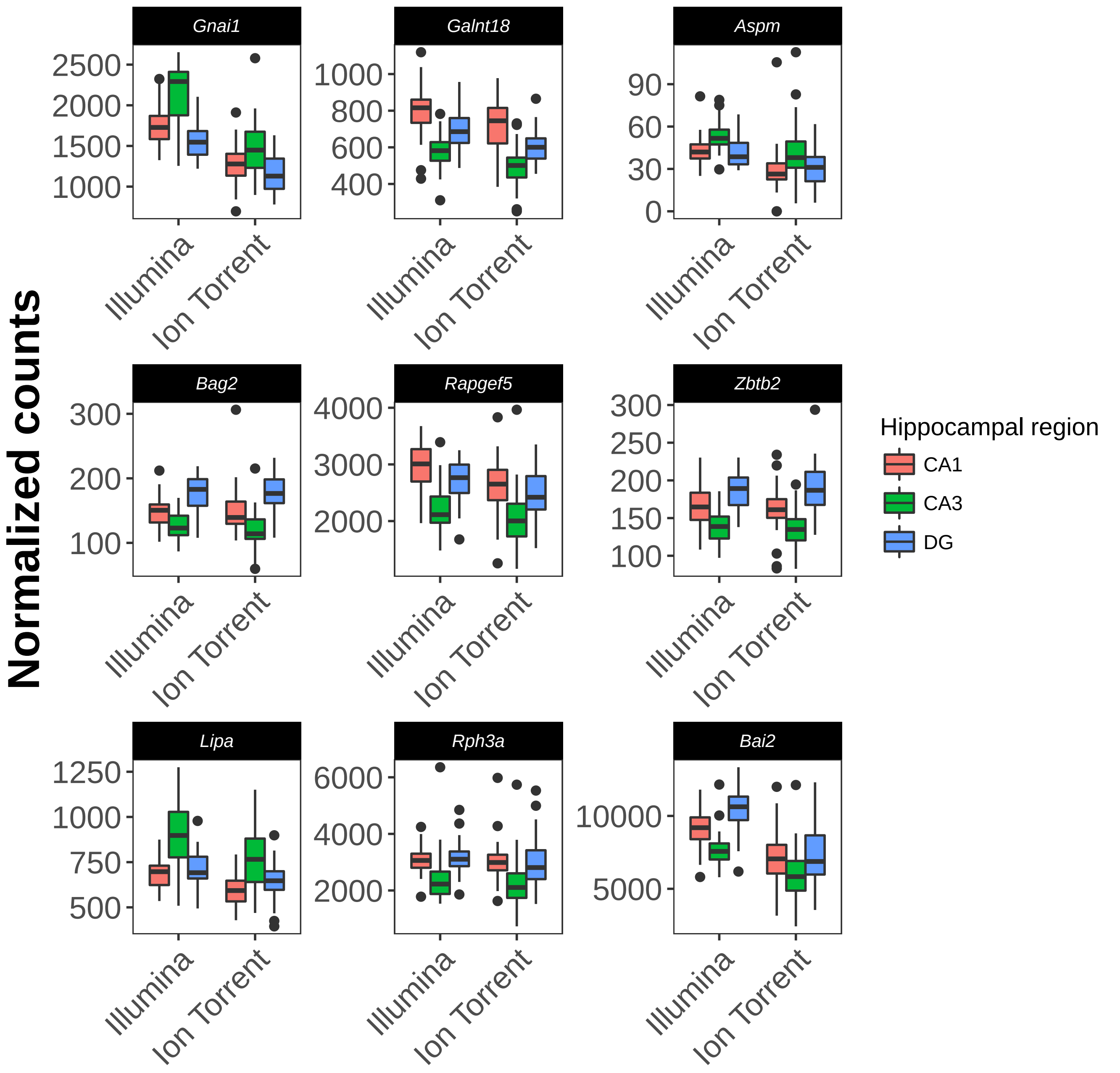
Normalized counts
Gnai1
Galnt18
Aspm
Bag2
Rapgef5
Zbtb2
Lipa
Rph3a
Bai2
Illumina
Ion Torrent
Hippocampal region
CA1
CA3
DG

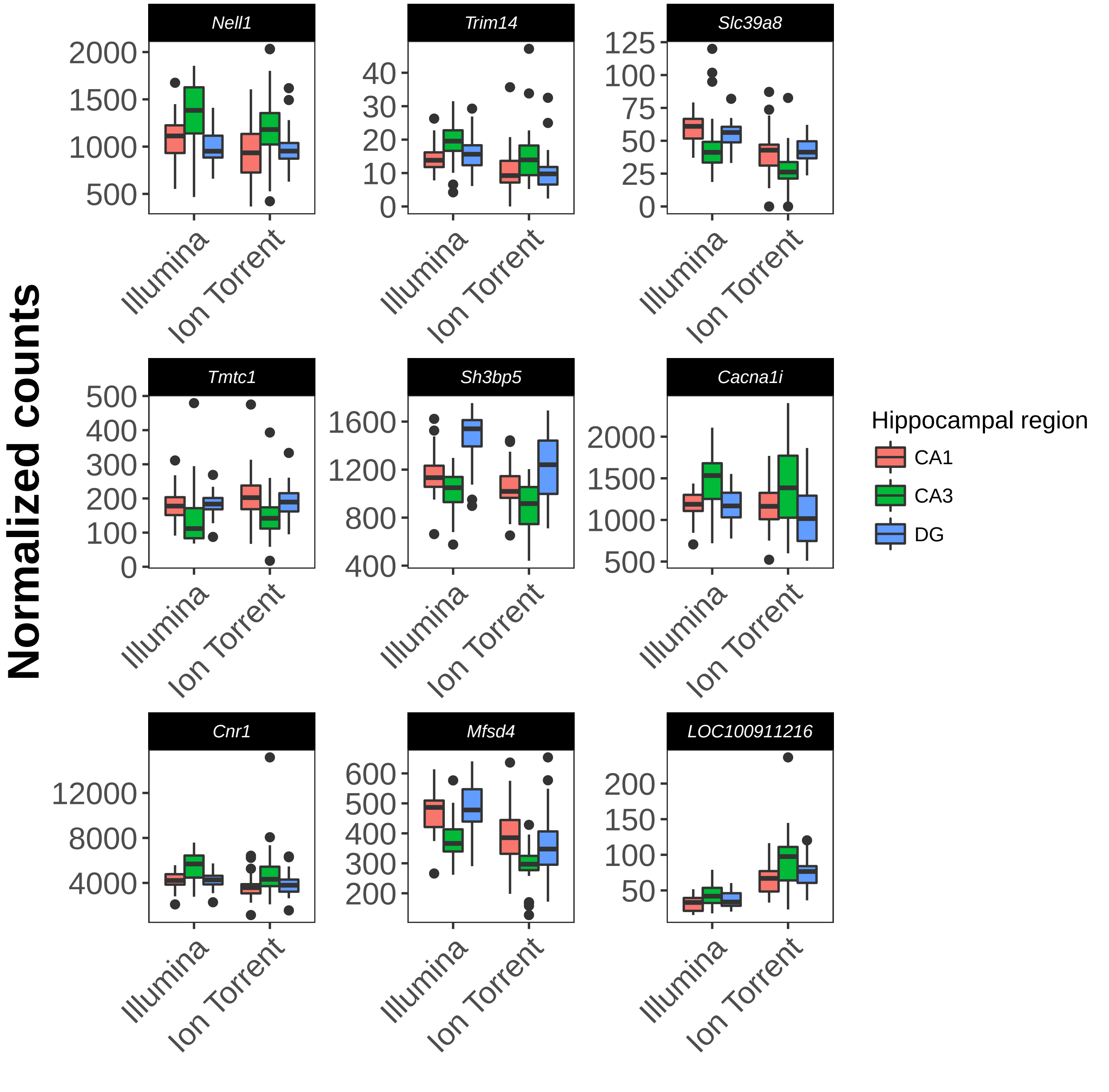

Normalized counts
Nell1
Trim14
Slc39a8
Tmtc1
Sh3bp5
Cacna1i
Cnr1
Mfsd4
LOC100911216
Illumina
Ion Torrent
Hippocampal region
CA1
CA3
DG

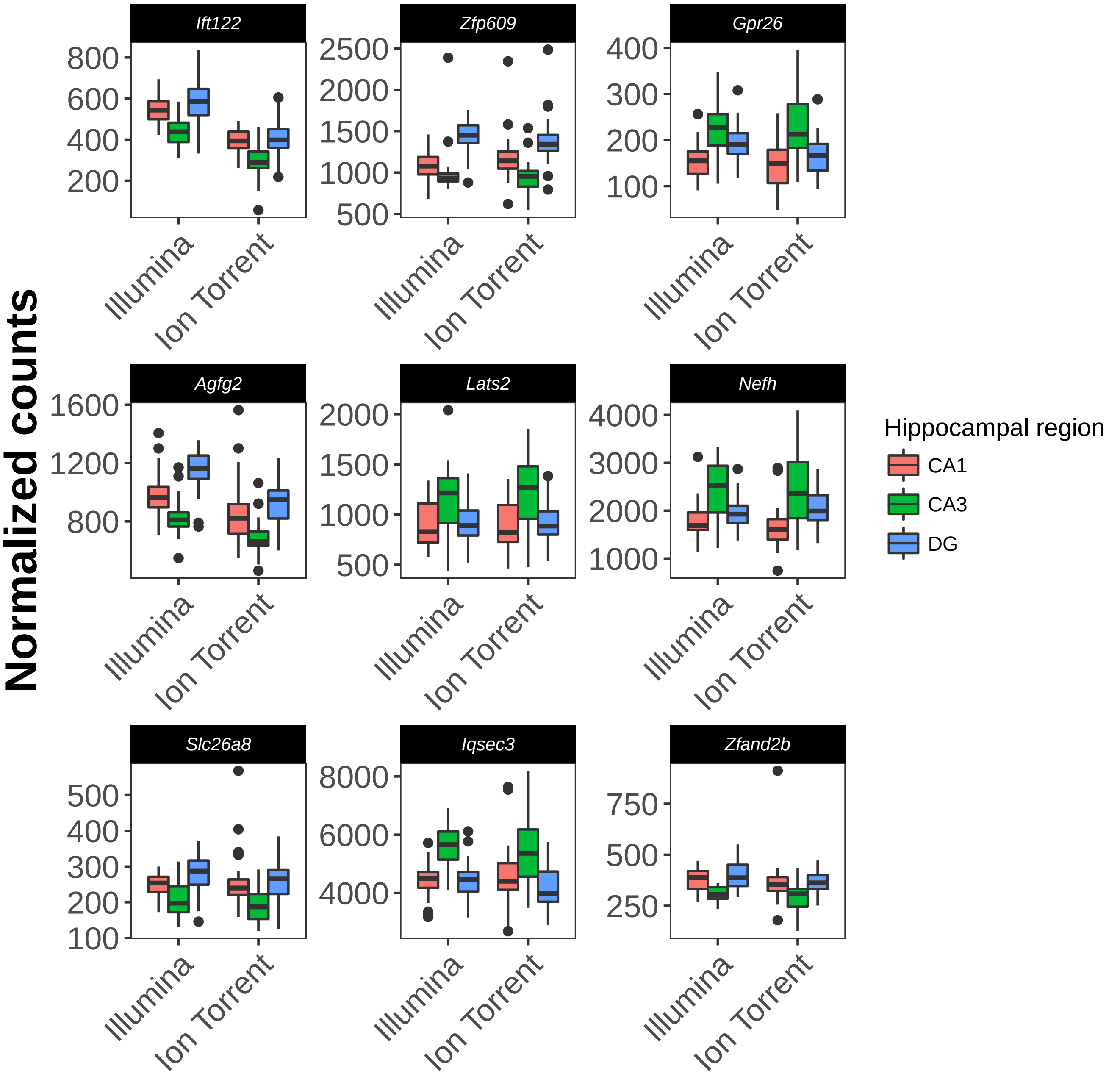

Normalized counts
Ift122
Zfp609
Gpr26
Agfg2
Lats2
Nefh
Slc26a8
Iqsec3
Zfand2b
Illumina
Ion Torrent
800
600
400
200
2500
2000
1500
1000
500
300
100
1600
1200
4000
3000
8000
6000
750
250
Hippocampal region
CA1
CA3
DG

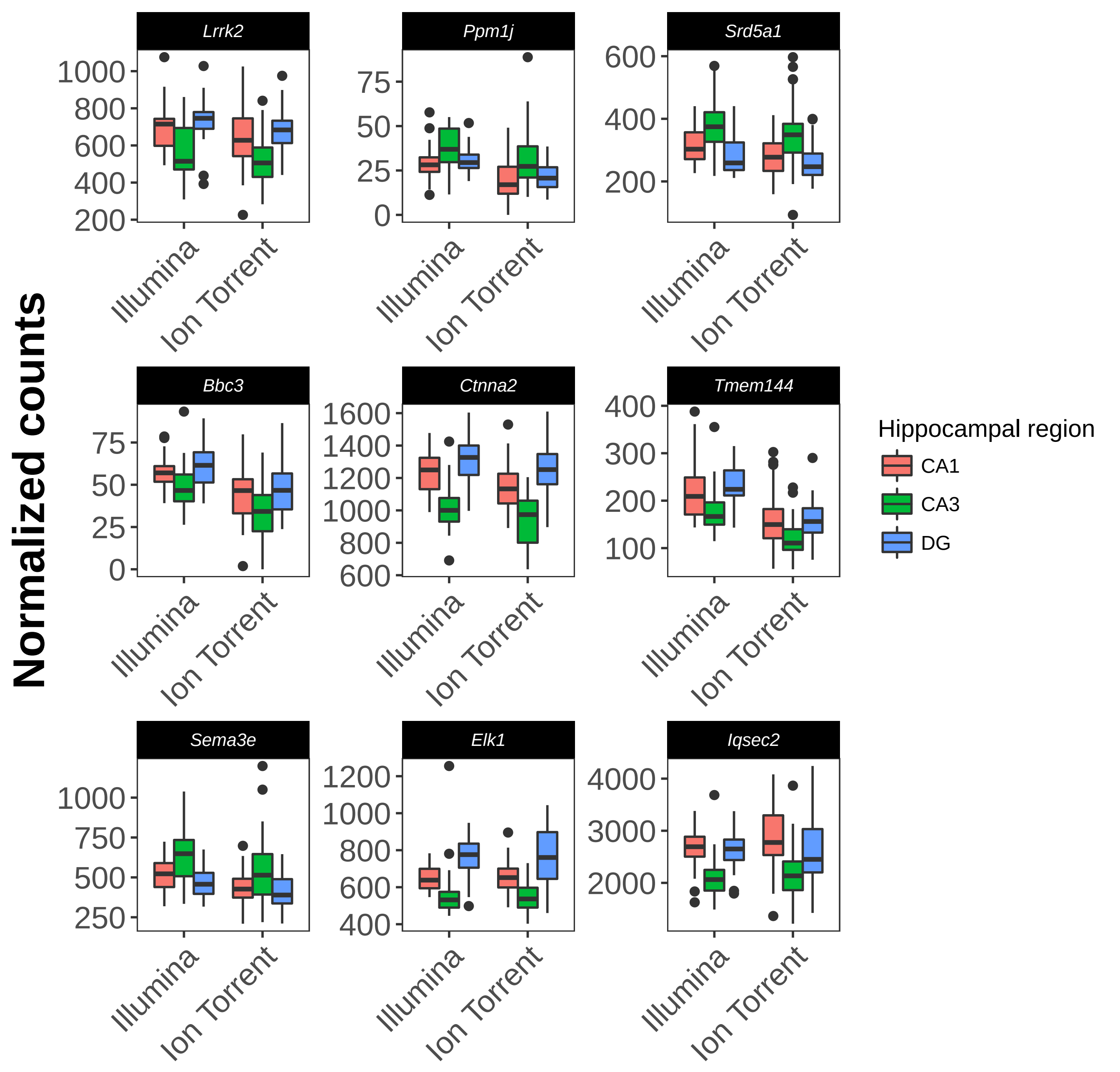

Normalized counts
Lrrk2
Ppm1j
Srd5a1
Bbc3
Ctnna2
Tmem144
Sema3e
Elk1
Iqsec2
Illumina
Ion Torrent
Hippocampal region
CA1
CA3
DG

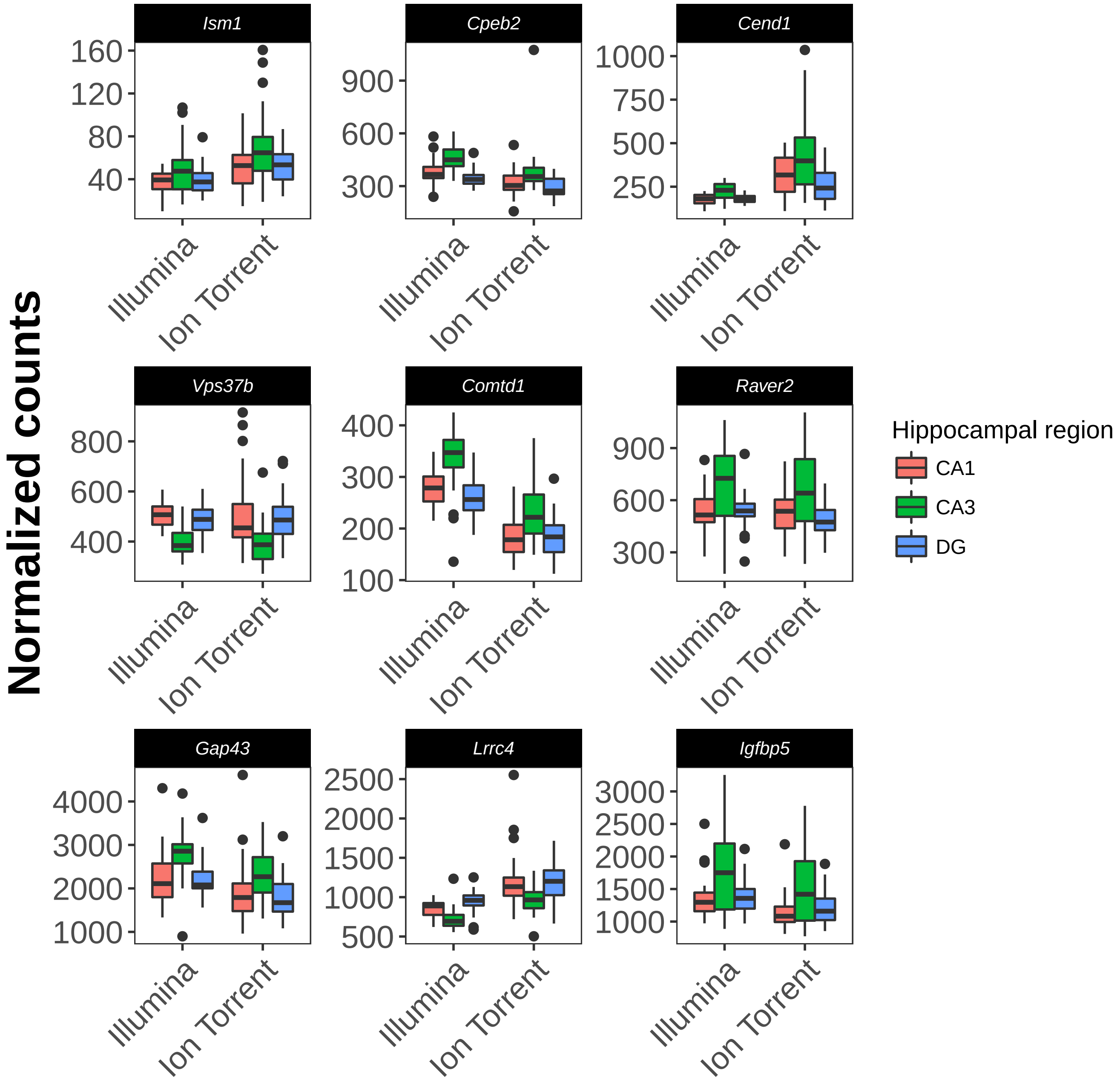

Normalized counts
Ism1
Cpeb2
Cend1
Vps37b
Comtd1
Raver2
Gap43
Lrrc4
Igfbp5
Illumina
Ion Torrent
Hippocampal region
CA1
CA3
DG

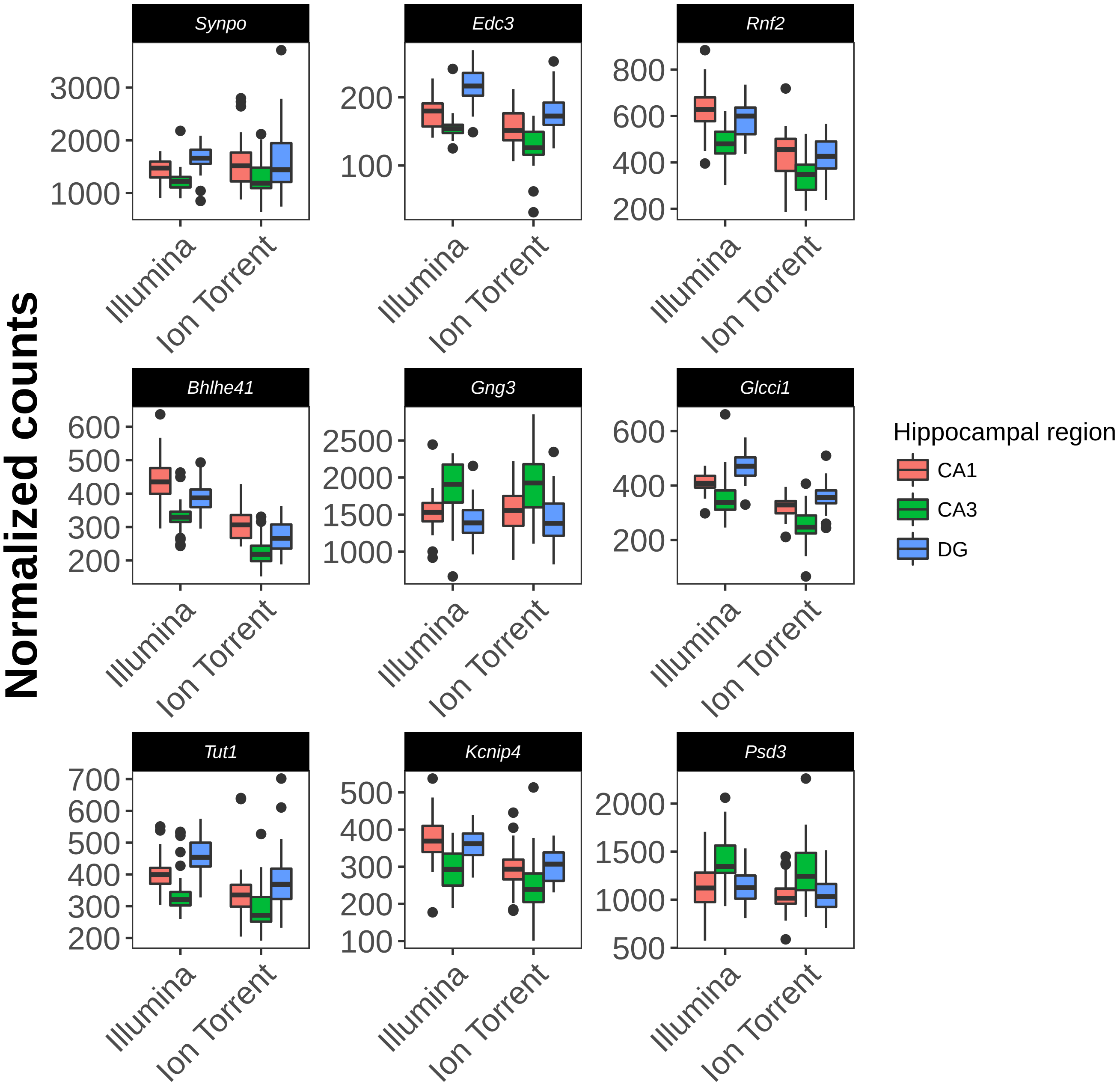

Synpo
Edc3
Rnf2
Bhlhe41
Gng3
Glcci1
Tut1
Kcnip4
Psd3
Normalized counts
Illumina
Ion Torrent
Hippocampal region
CA1
CA3
DG

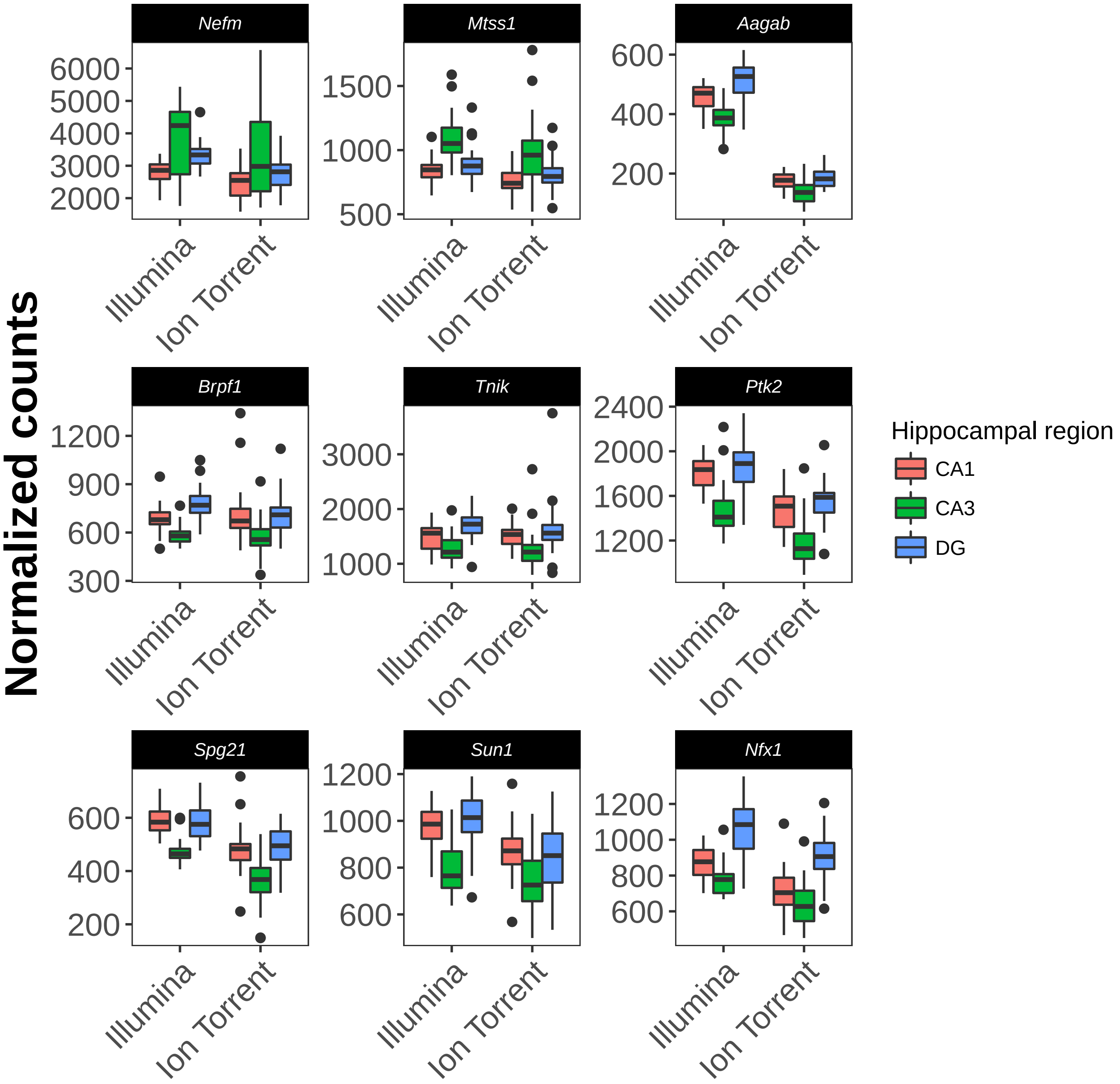
Normalized counts
Nefm
Mtss1
Aagab
Brpf1
Tnik
Ptk2
Spg21
Sun1
Nfx1
Illumina
Ion Torrent
Hippocampal region
CA1
CA3
DG

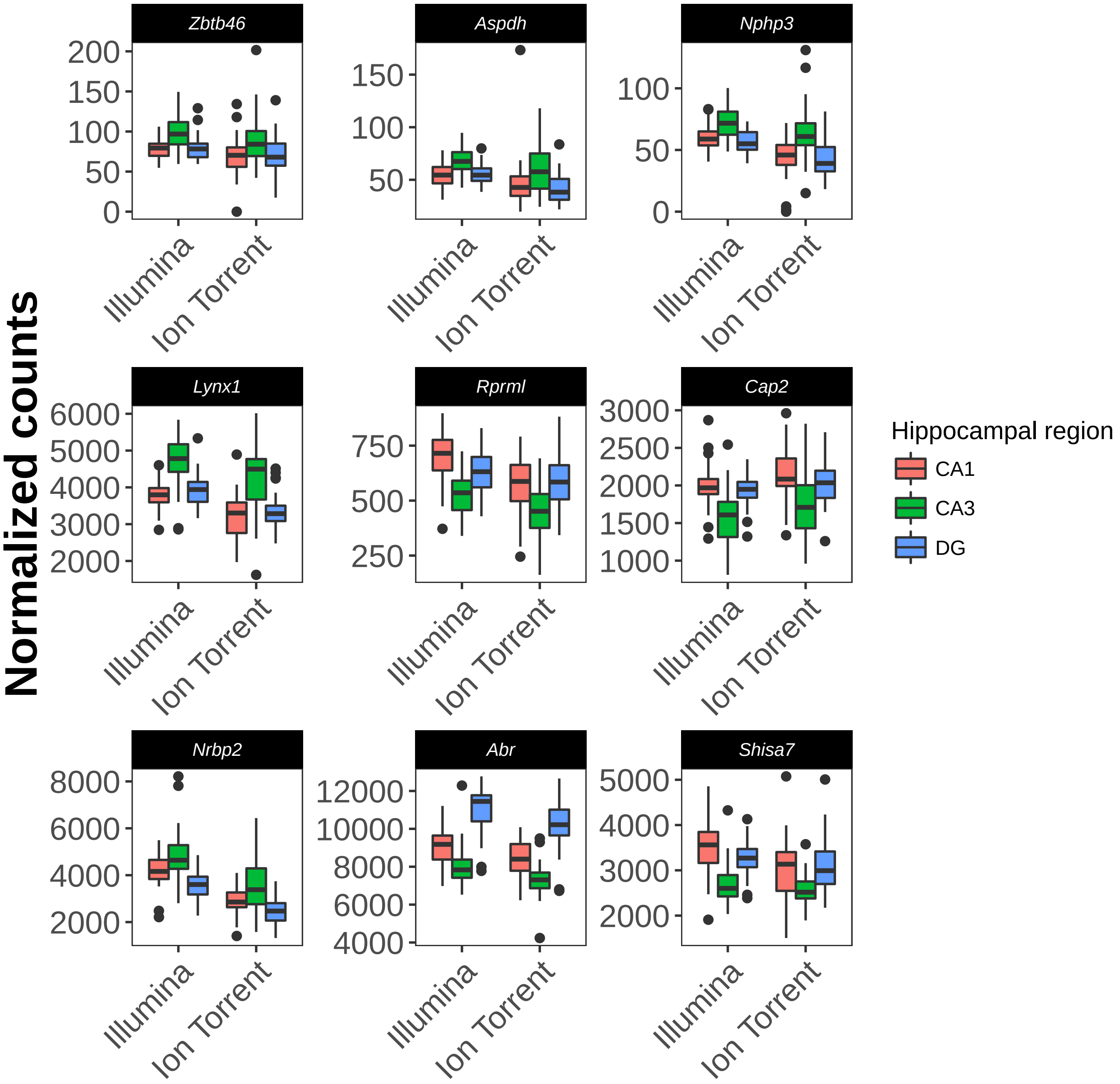
Normalized counts
Zbtb46
Aspdh
Nphp3
Lynx1
Rprml
Cap2
Nrbp2
Abr
Shisa7
Illumina
Ion Torrent
Hippocampal region
CA1
CA3
DG

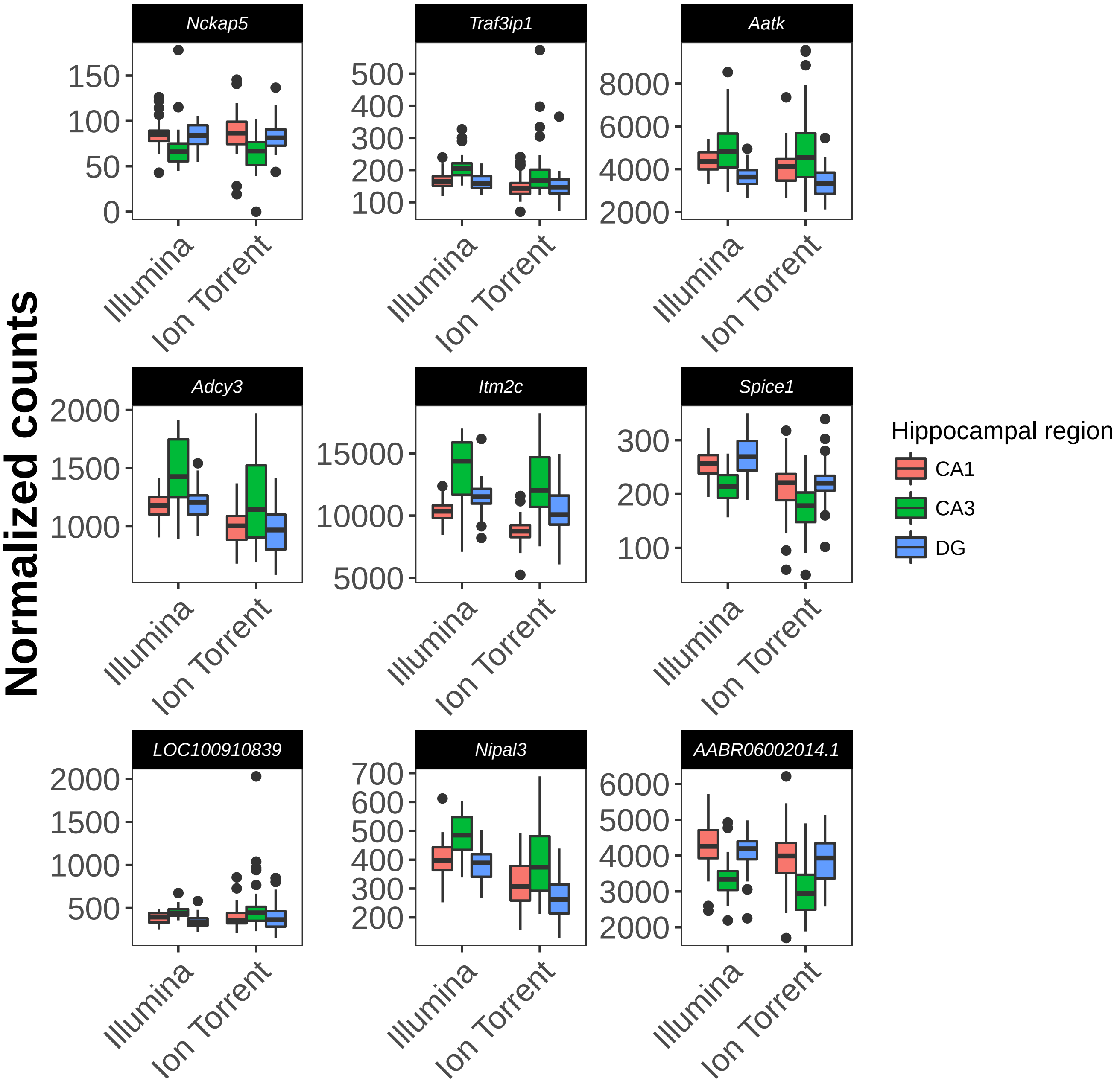

Normalized counts
Nckap5
Traf3ip1
Aatk
Adcy3
Itm2c
Spice1
LOC100910839
Nipal3
AABR06002014.1
Illumina
Ion Torrent
Hippocampal region
CA1
CA3
DG

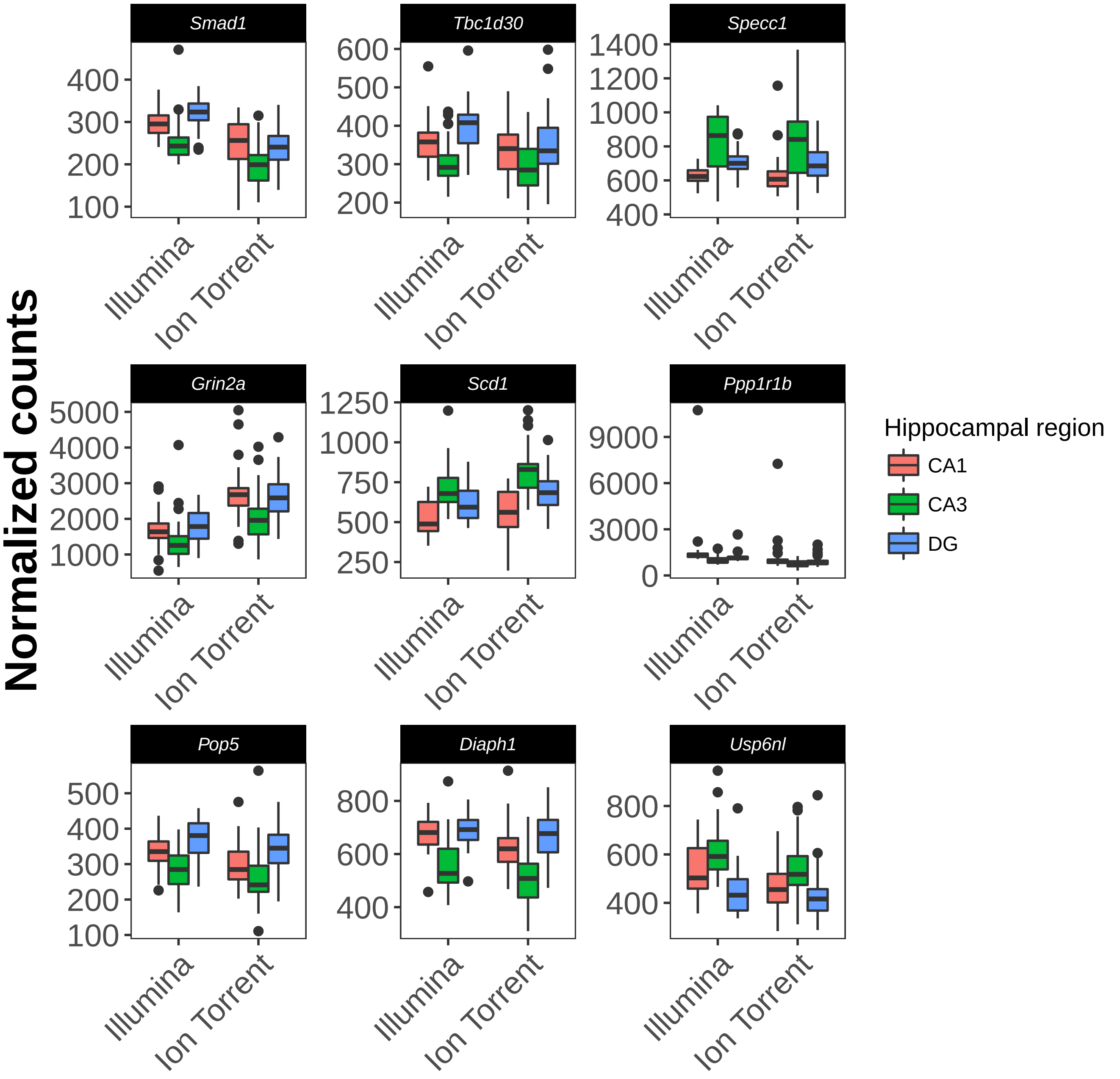

Normalized counts
Smad1
Tbc1d30
Specc1
Grin2a
Scd1
Ppp1r1b
Pop5
Diaph1
Usp6nl
Illumina
Ion Torrent
Hippocampal region
CA1
CA3
DG

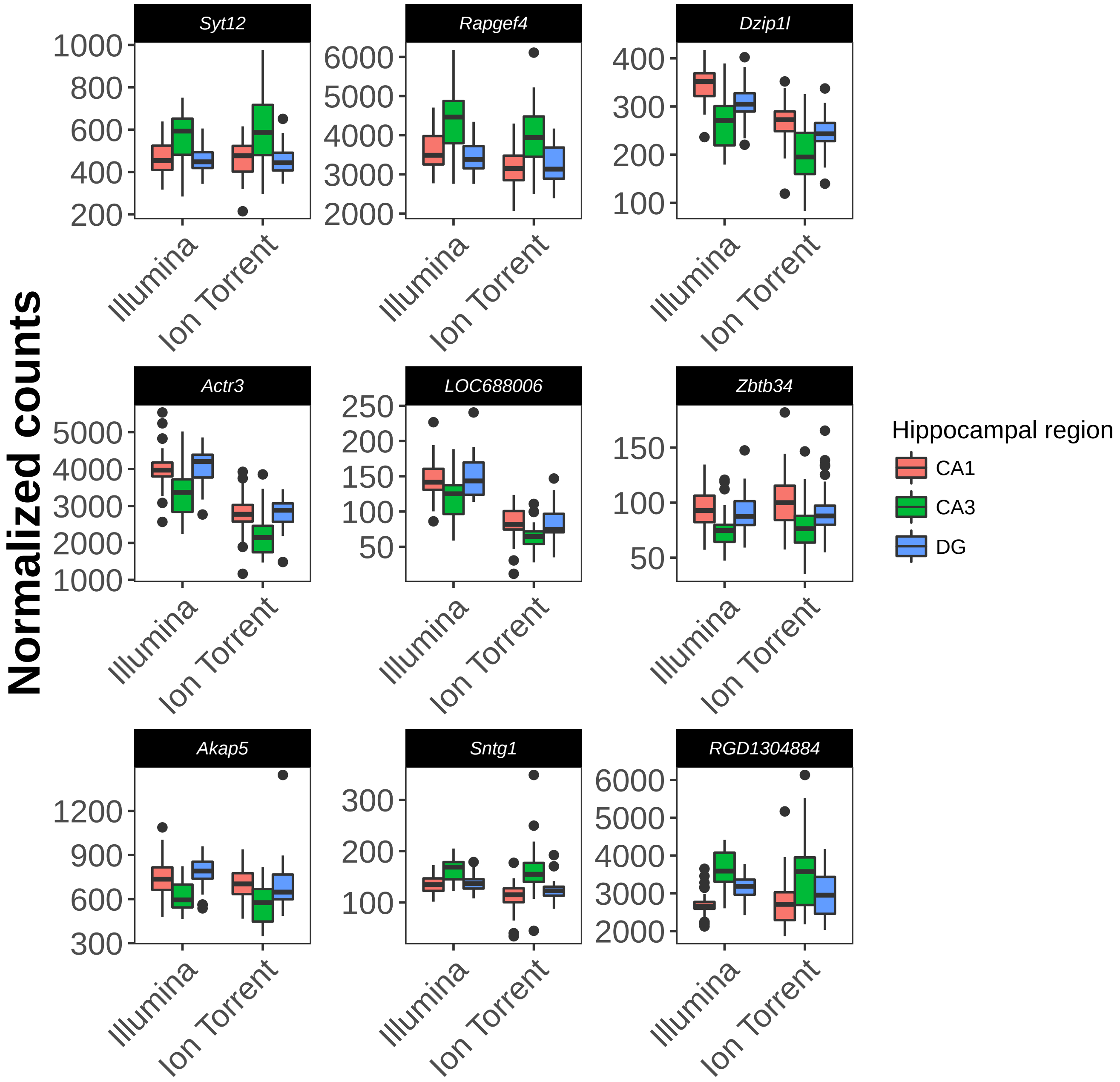

Normalized counts
Syt12
Rapgef4
Dzip1l
Actr3
LOC688006
Zbtb34
Akap5
Sntg1
RGD1304884
Illumina
Ion Torrent
Hippocampal region
CA1
CA3
DG

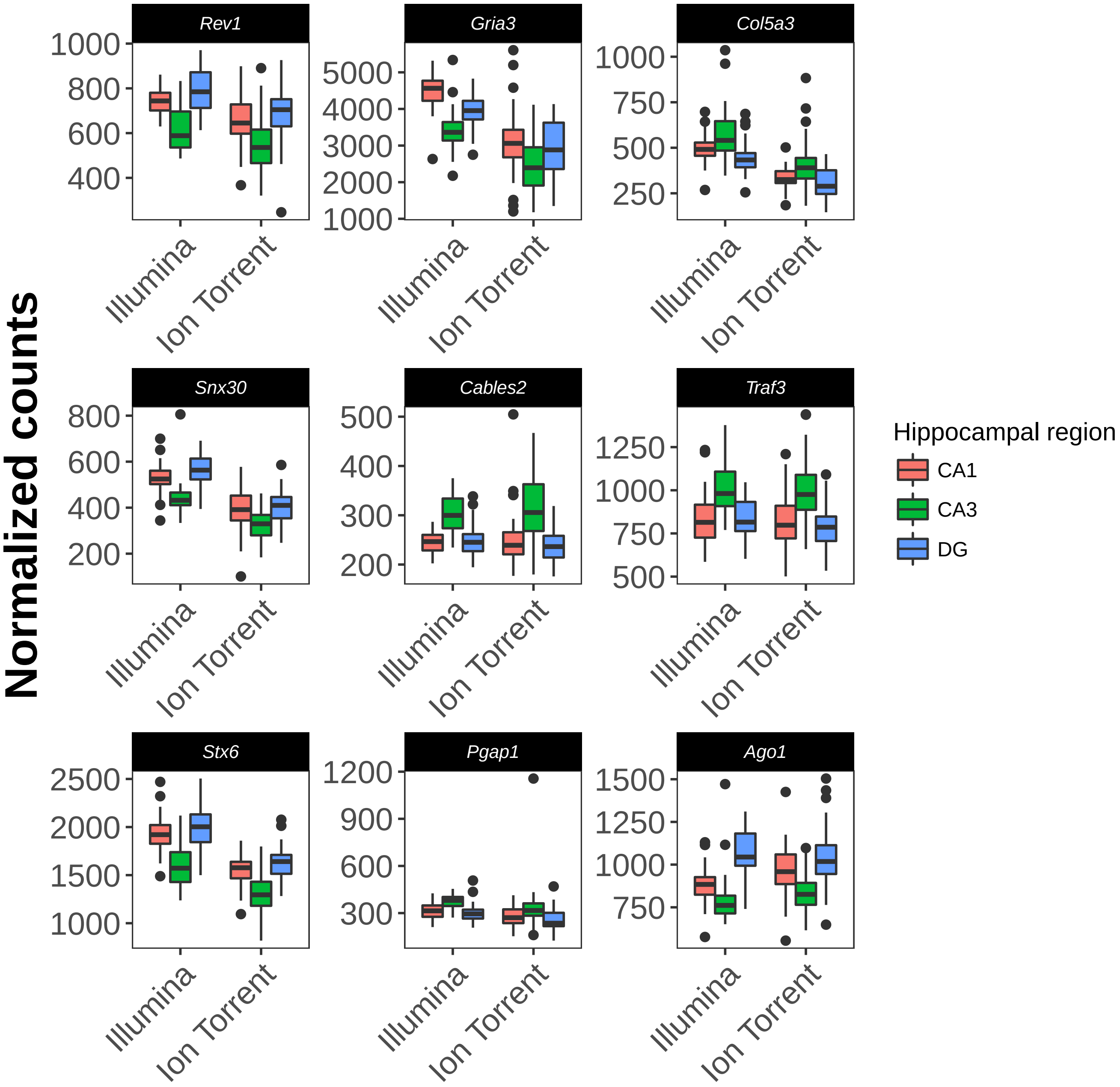

Normalized counts
Rev1
Gria3
Col5a3
Snx30
Cables2
Traf3
Stx6
Pgap1
Ago1
Illumina
Ion Torrent
Hippocampal region
CA1
CA3
DG

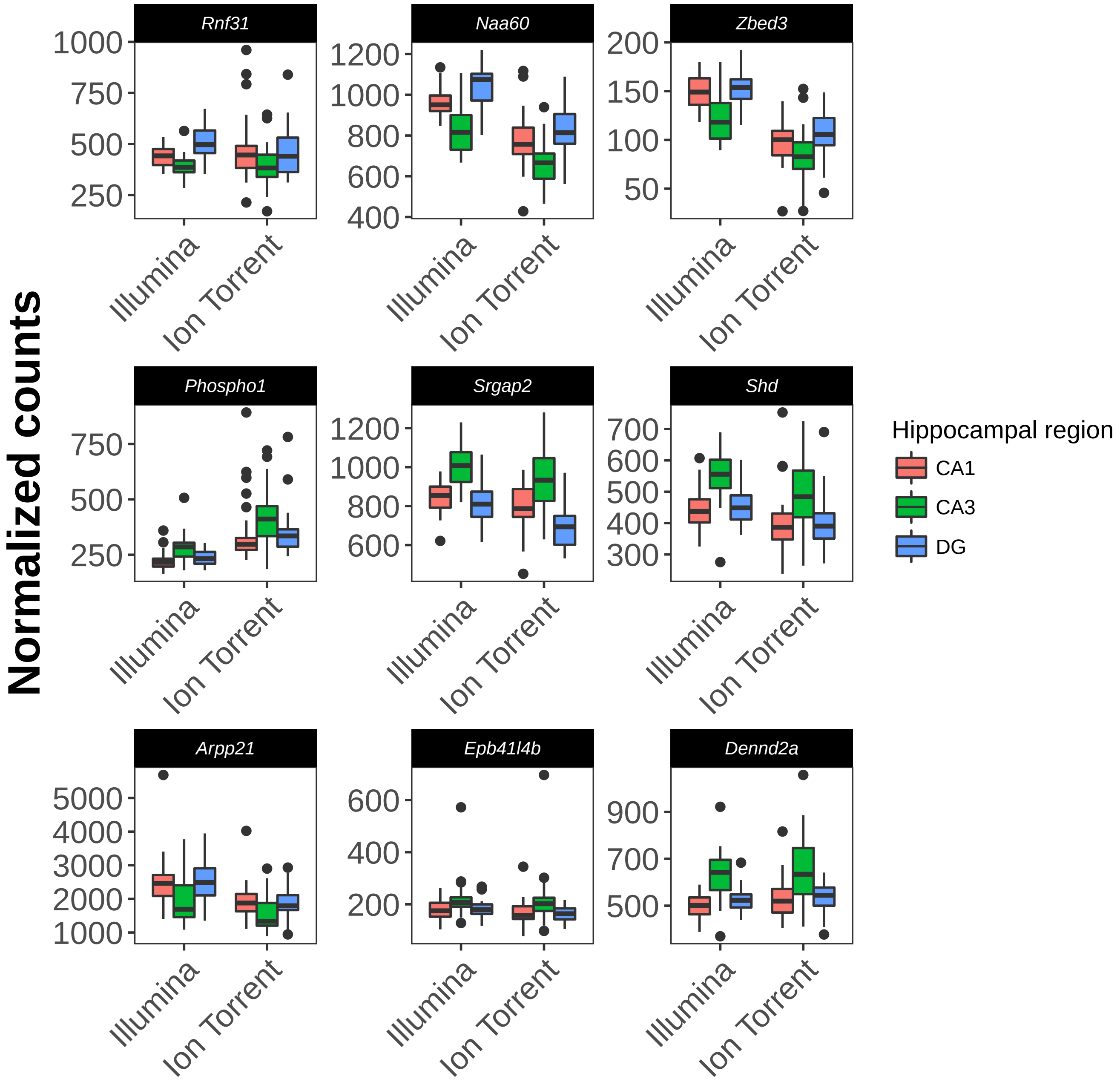
Normalized counts
Rnf31
Naa60
Zbed3
Phospho1
Srgap2
Shd
Arpp21
Epb41l4b
Dennd2a
Illumina
Ion Torrent
Hippocampal region
CA1
CA3
DG

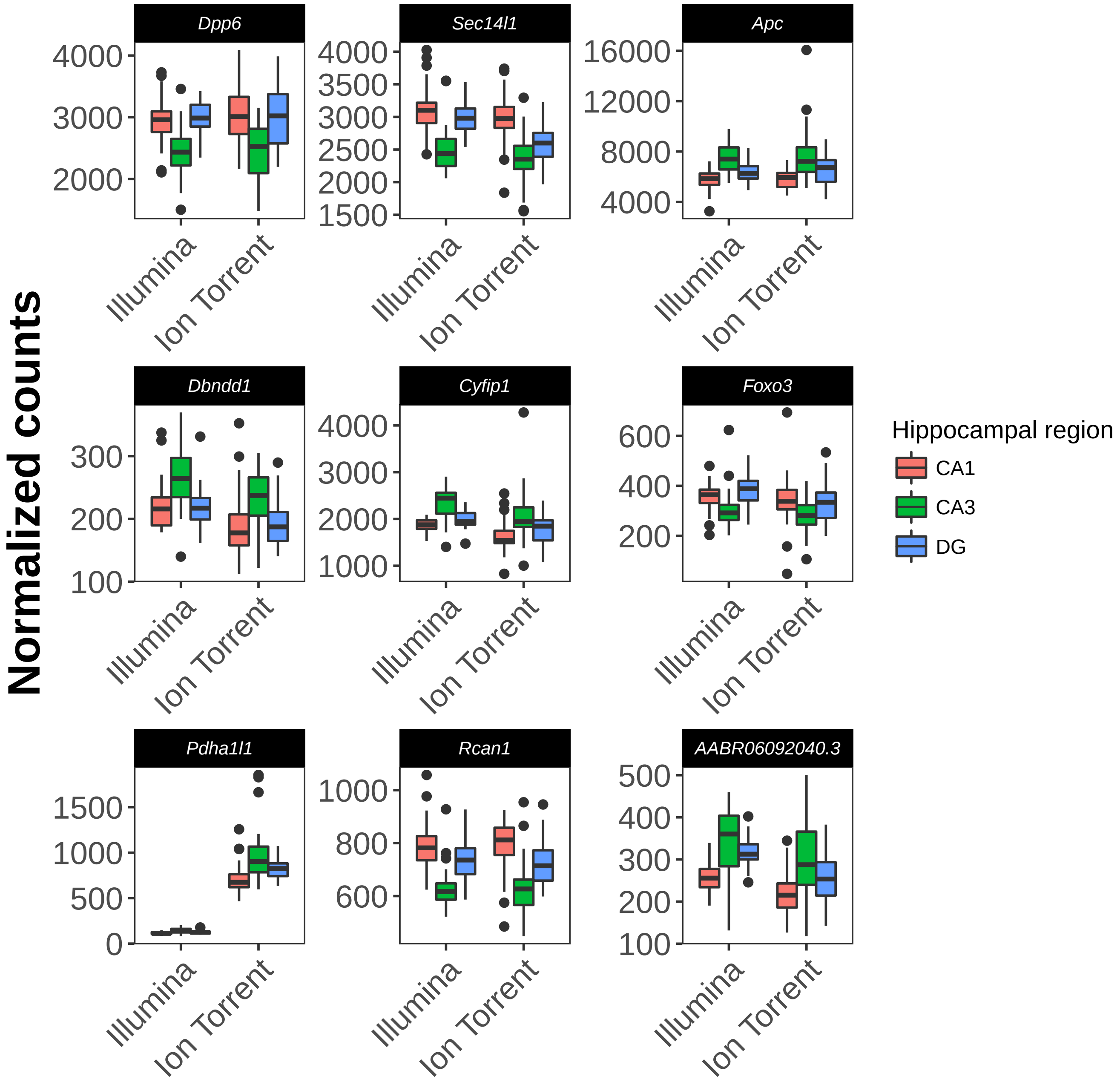

Normalized counts
Dpp6
Sec14l1
Apc
Dbndd1
Cyfip1
Foxo3
Pdha1l1
Rcan1
AABR06092040.3
Illumina
Ion Torrent
Hippocampal region
CA1
CA3
DG

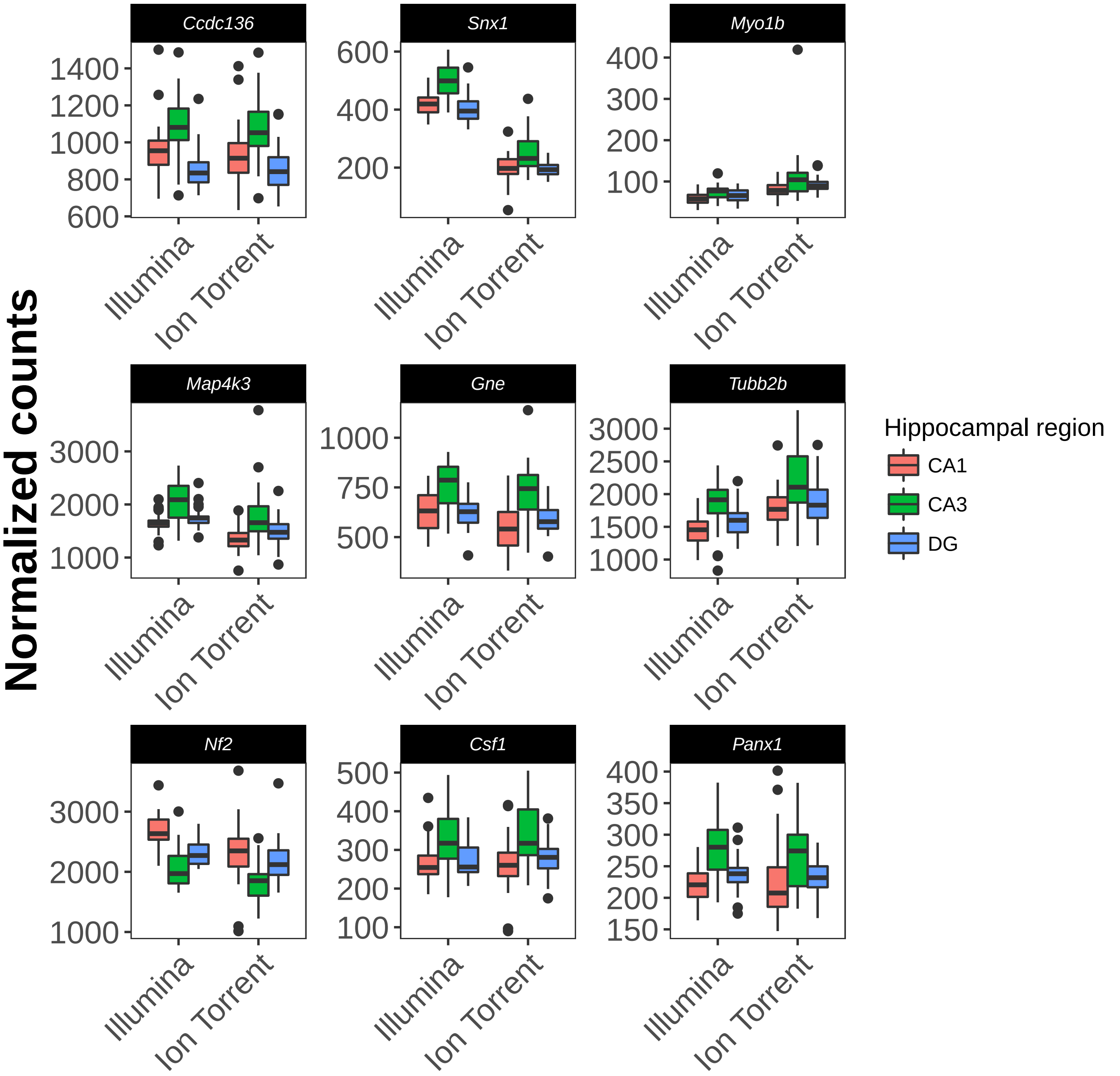
Normalized counts
Ccdc136
Snx1
Myo1b
Map4k3
Gne
Tubb2b
Nf2
Csf1
Panx1
Illumina
Ion Torrent
Hippocampal region
CA1
CA3
DG

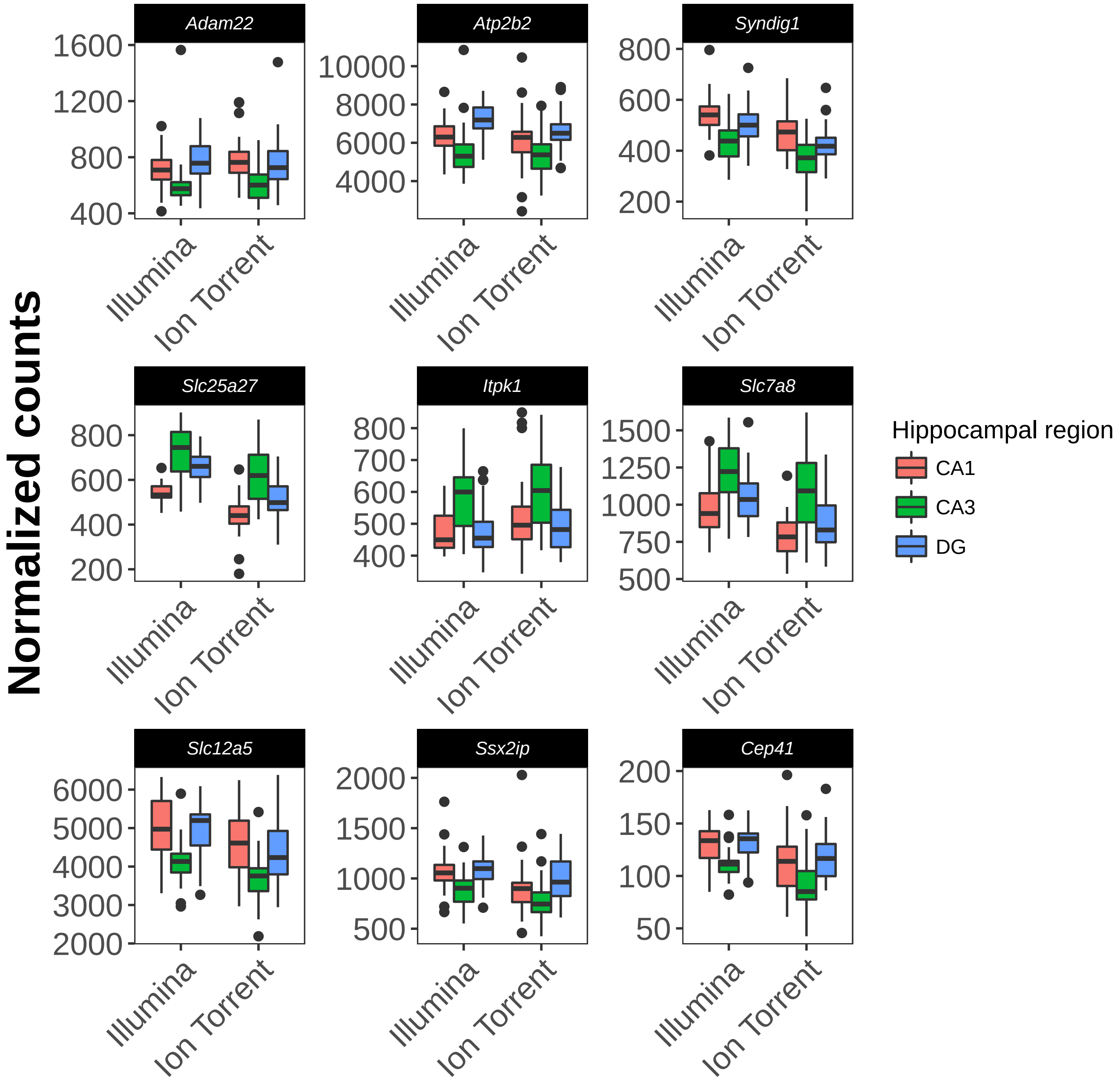
Normalized counts
Adam22
Atp2b2
Syndig1
Slc25a27
Itpk1
Slc7a8
Slc12a5
Ssx2ip
Cep41
Illumina
Ion Torrent
Hippocampal region
CA1
CA3
DG

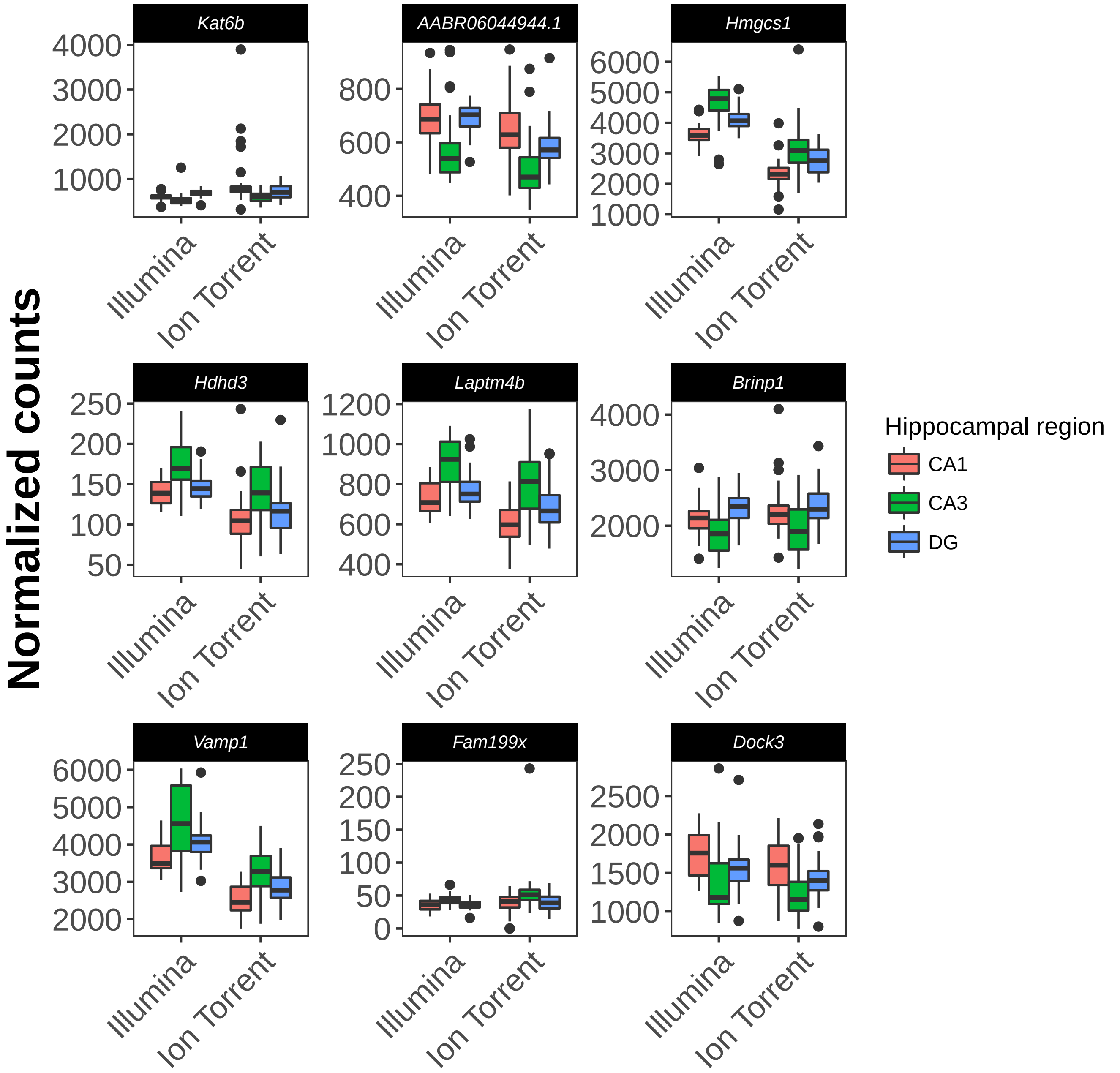
Normalized counts
Kat6b
AABR06044944.1
Hmgcs1
Hdhd3
Laptm4b
Brinp1
Vamp1
Fam199x
Dock3
Illumina
Ion Torrent
Hippocampal region
CA1
CA3
DG

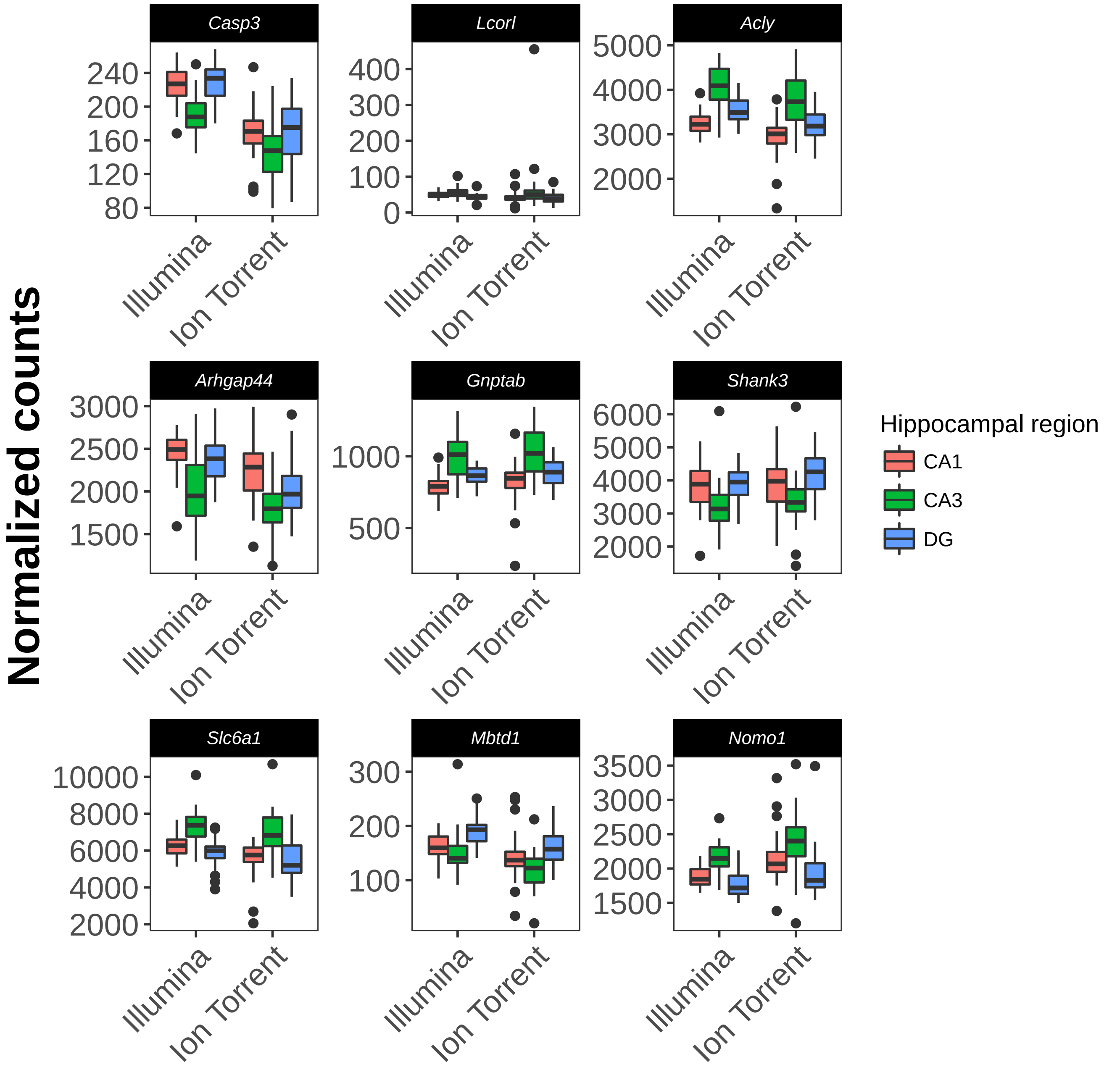

Normalized counts
Casp3
Lcorl
Acly
Arhgap44
Gnptab
Shank3
Slc6a1
Mbtd1
Nomo1
Illumina
Ion Torrent
Hippocampal region
CA1
CA3
DG

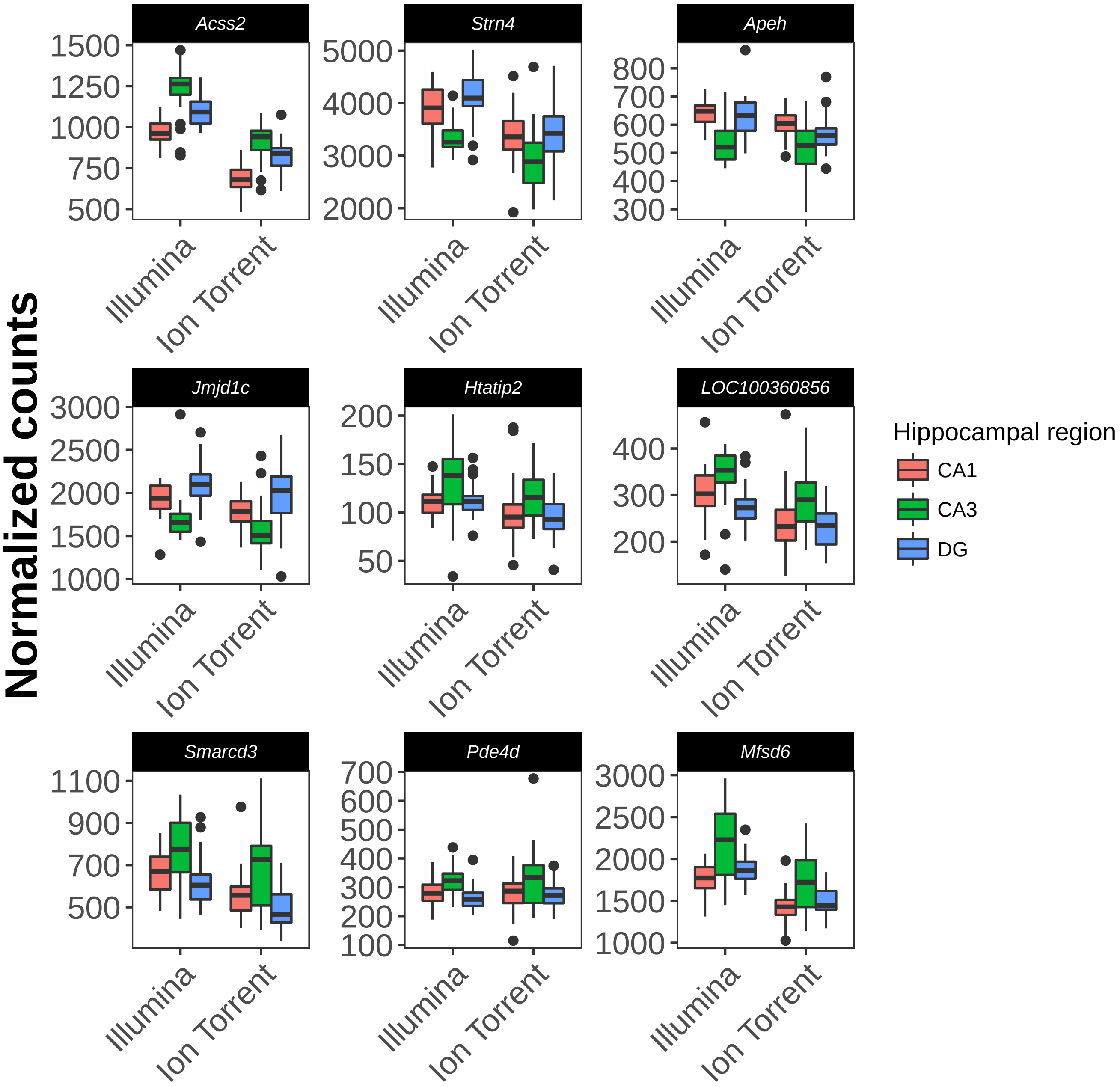

Normalized counts
Acss2
Strn4
Apeh
Jmjd1c
Htatip2
LOC100360856
Smarcd3
Pde4d
Mfsd6
Illumina
Ion Torrent
Hippocampal region
CA1
CA3
DG

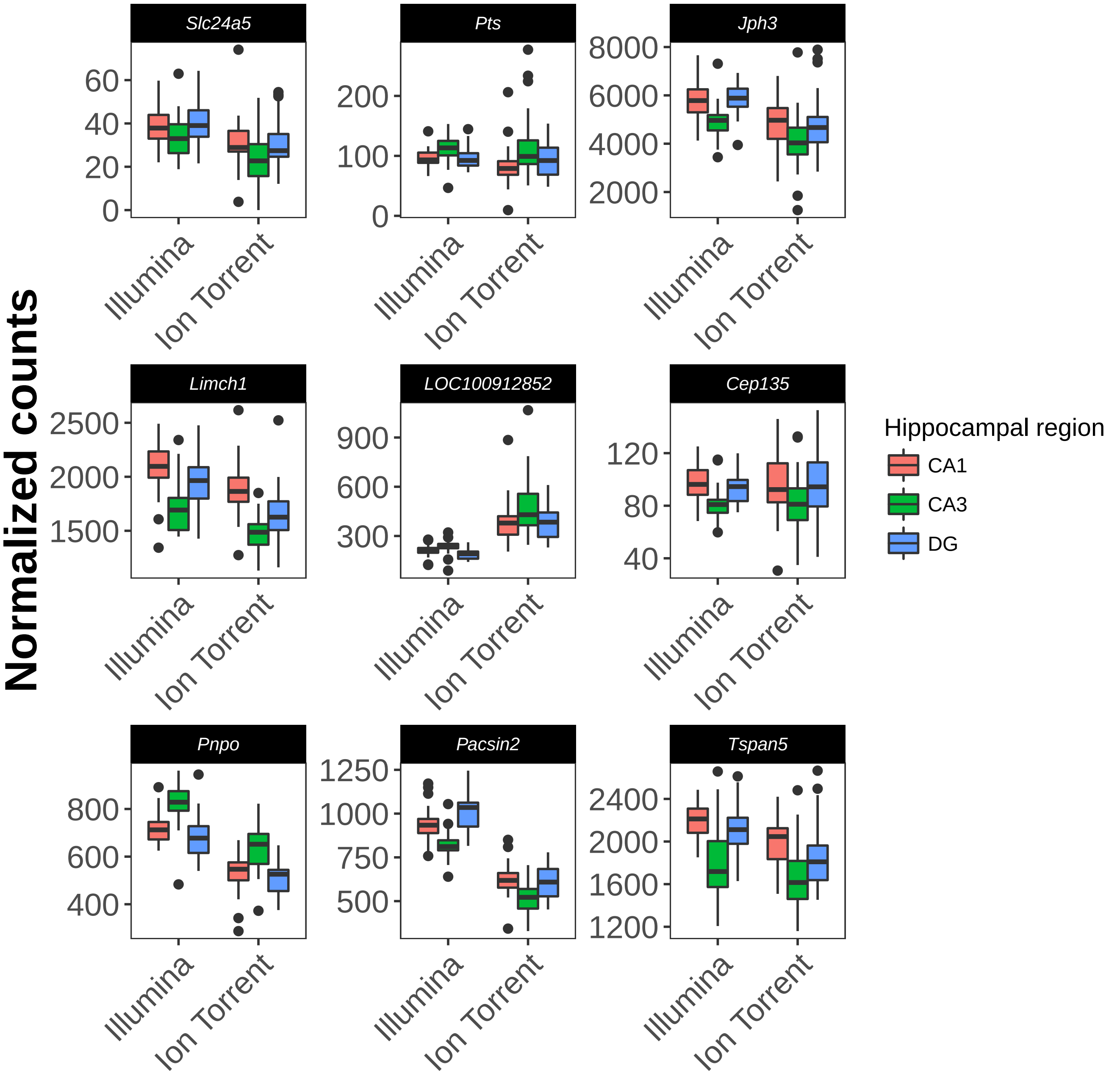
Normalized counts
Slc24a5
Pts
Jph3
Limch1
LOC100912852
Cep135
Pnpo
Pacsin2
Tspan5
Illumina
Ion Torrent
Hippocampal region
CA1
CA3
DG

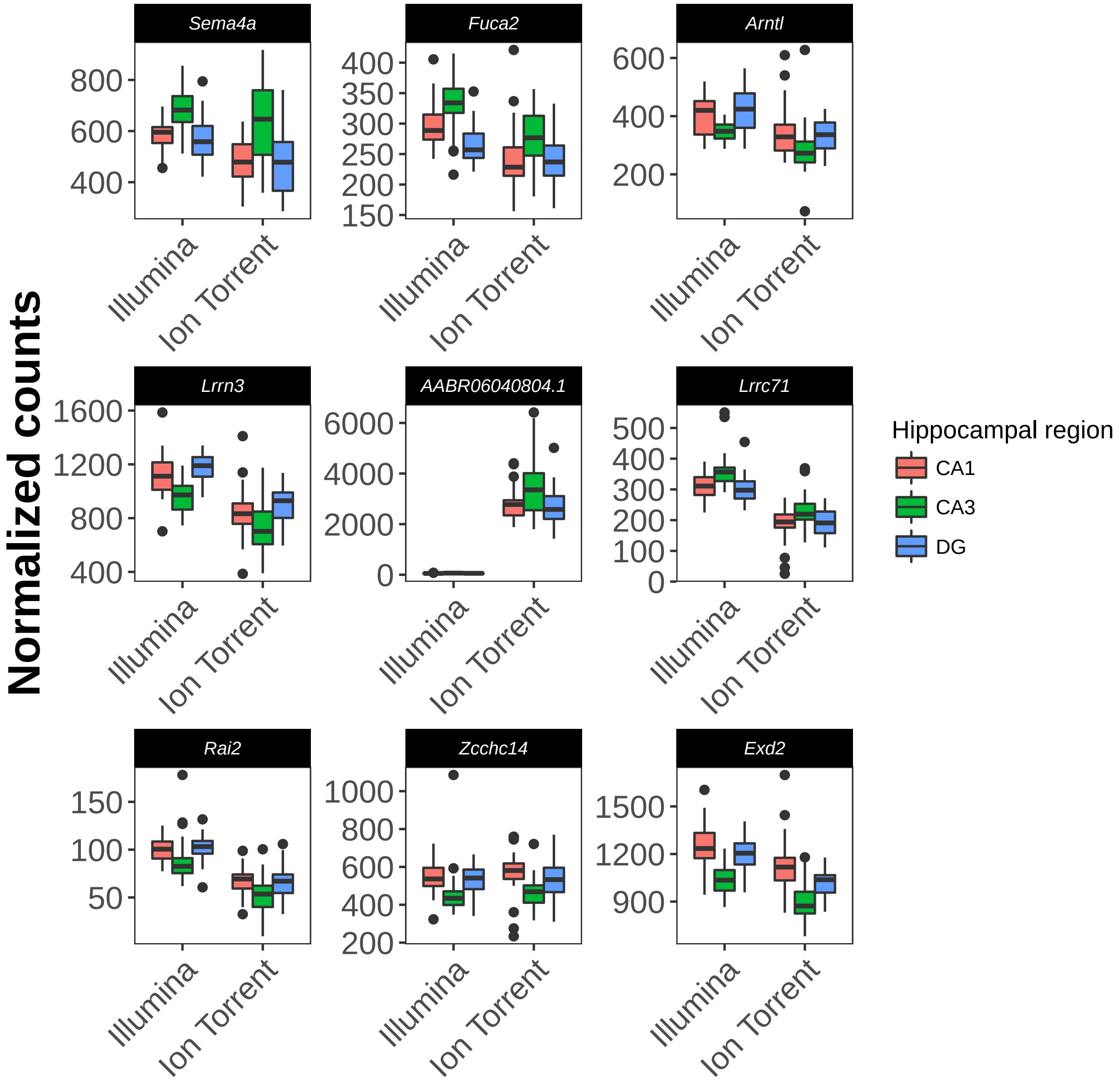

Normalized counts
Sema4a
Fuca2
Arntl
Lrrn3
AABR06040804.1
Lrrc71
Rai2
Zcchc14
Exd2
Illumina
Ion Torrent
Hippocampal region
CA1
CA3
DG

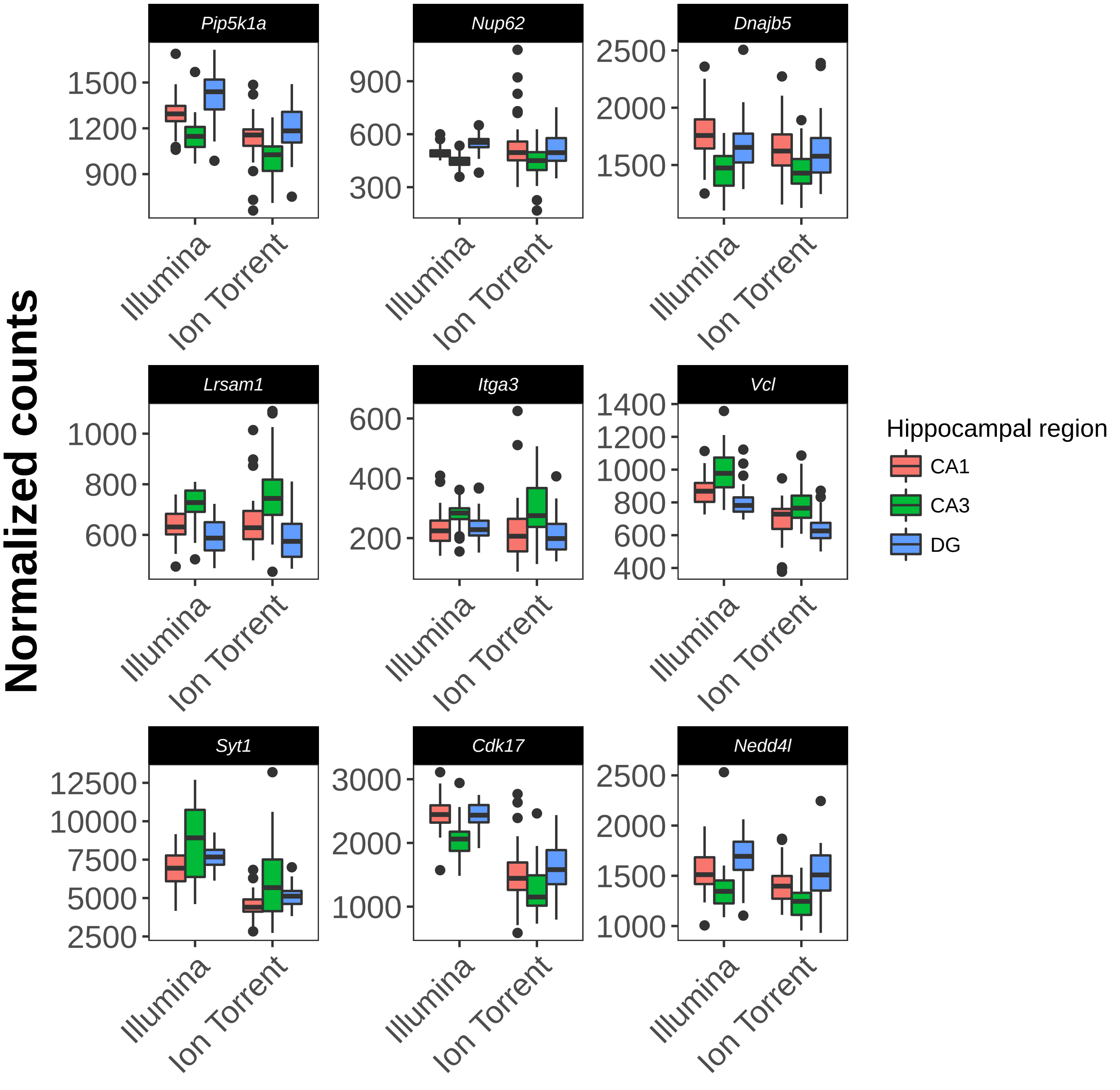

Normalized counts
Pip5k1a
Nup62
Dnajb5
Lrsam1
Itga3
Vcl
Syt1
Cdk17
Nedd4l
Illumina
Ion Torrent
Hippocampal region
CA1
CA3
DG

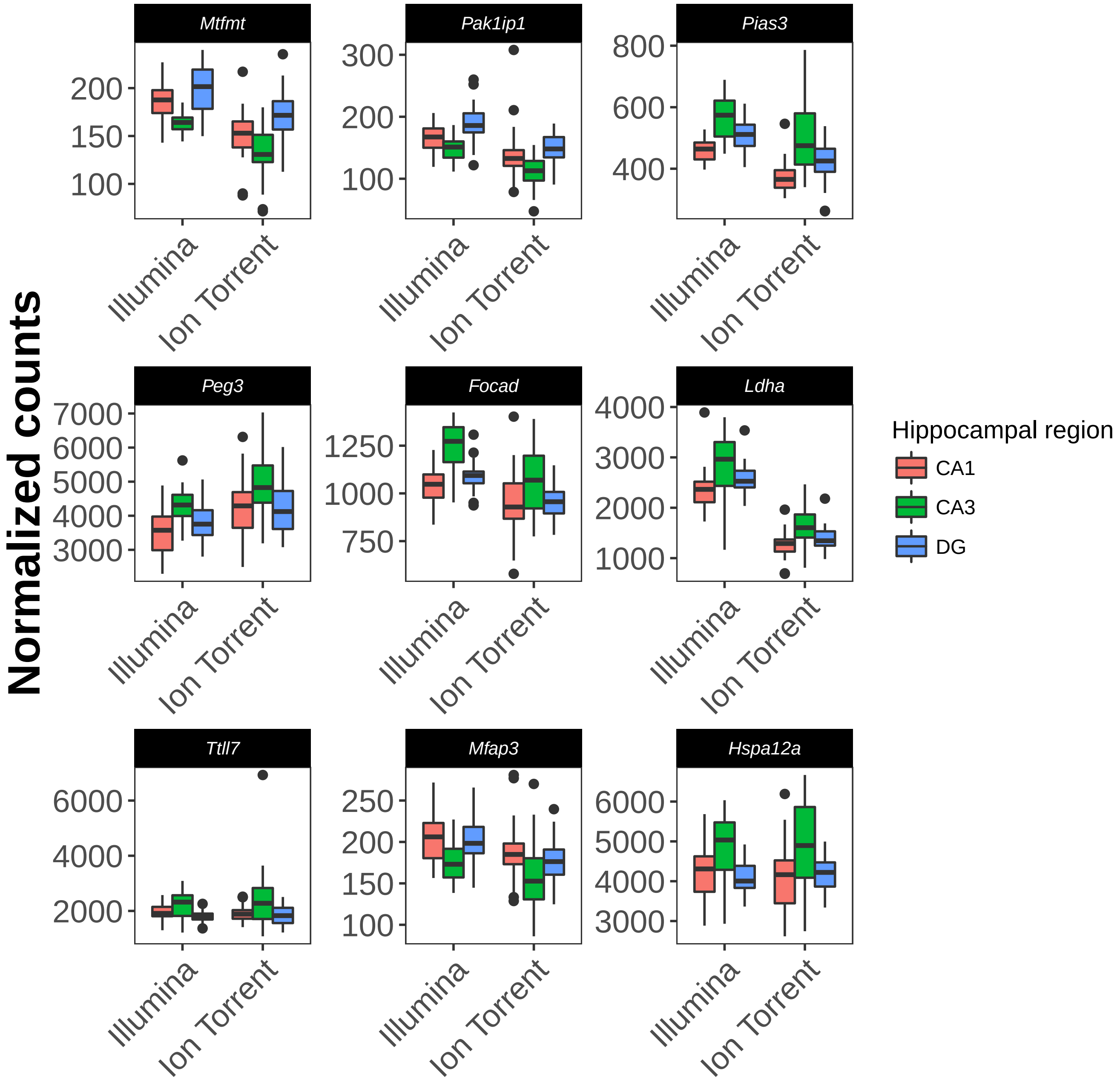

Normalized counts
Mtfmt
Pak1ip1
Pias3
Peg3
Focad
Ldha
Ttll7
Mfap3
Hspa12a
Illumina
Ion Torrent
Hippocampal region
CA1
CA3
DG

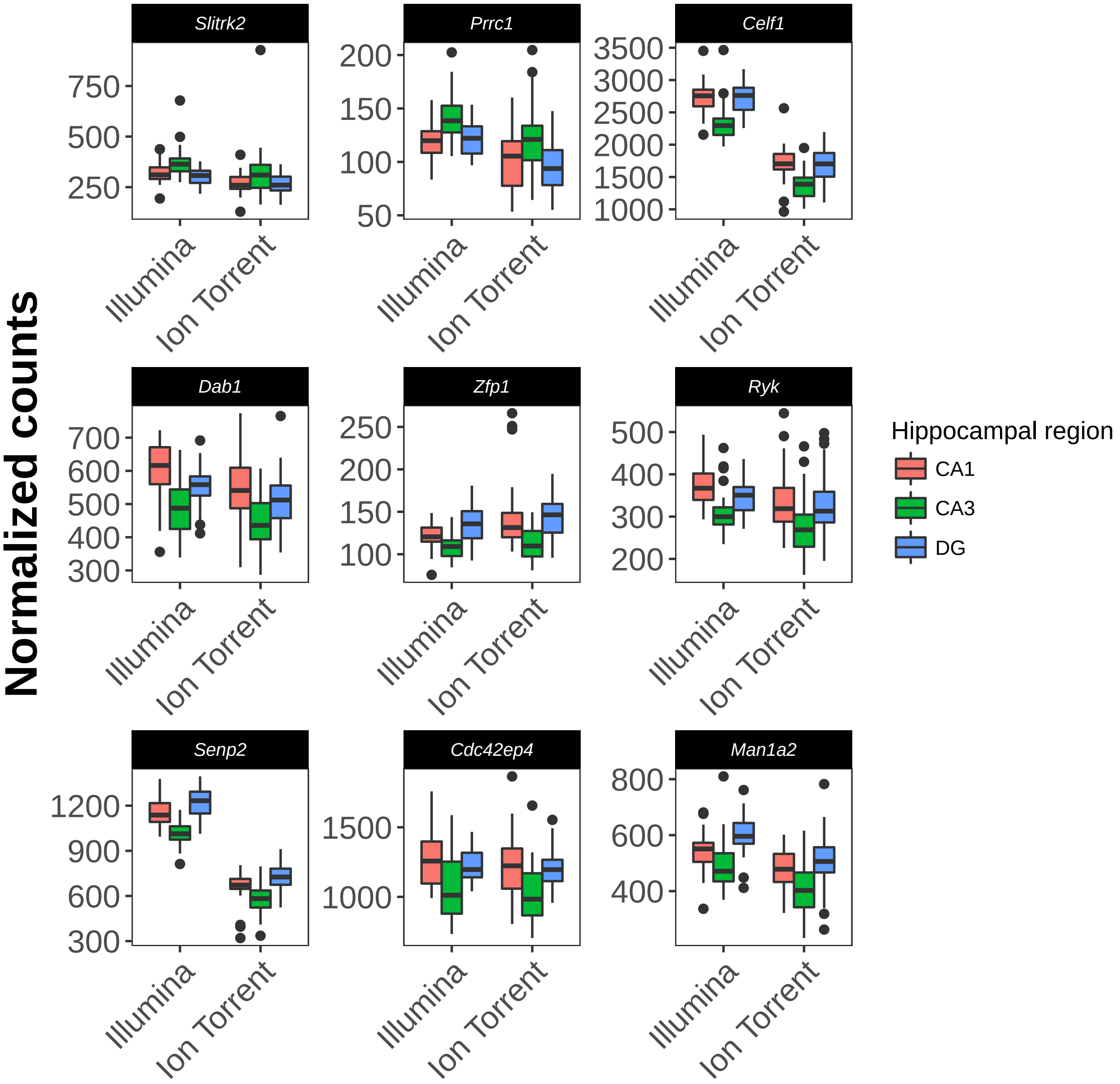

Normalized counts
Slitrk2
Prrc1
Celf1
Dab1
Zfp1
Ryk
Senp2
Cdc42ep4
Man1a2
Illumina
Ion Torrent
Hippocampal region
CA1
CA3
DG

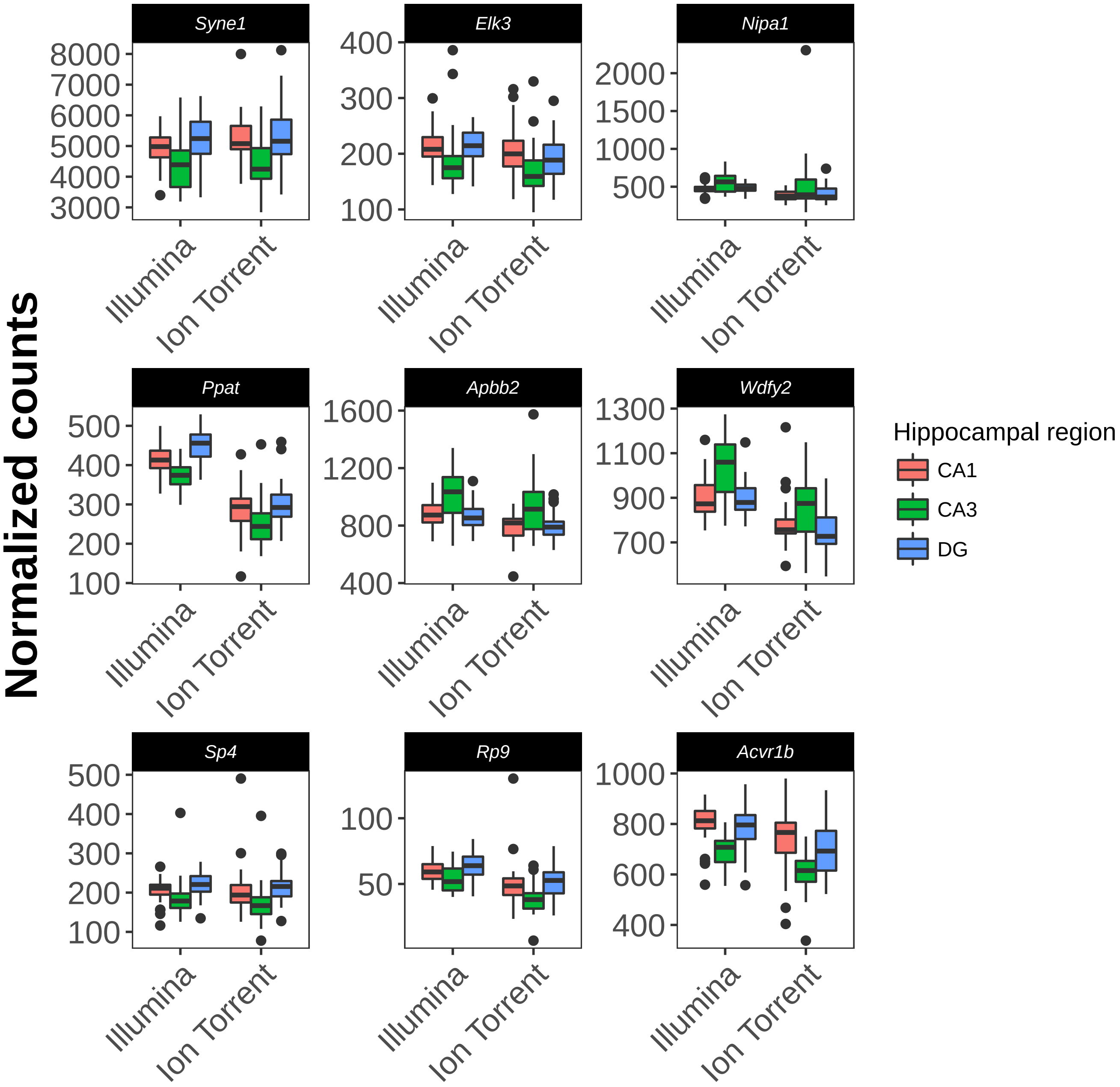

Normalized counts
Syne1
Elk3
Nipa1
Ppat
Apbb2
Wdfy2
Sp4
Rp9
Acvr1b
Illumina
Ion Torrent
Hippocampal region
CA1
CA3
DG

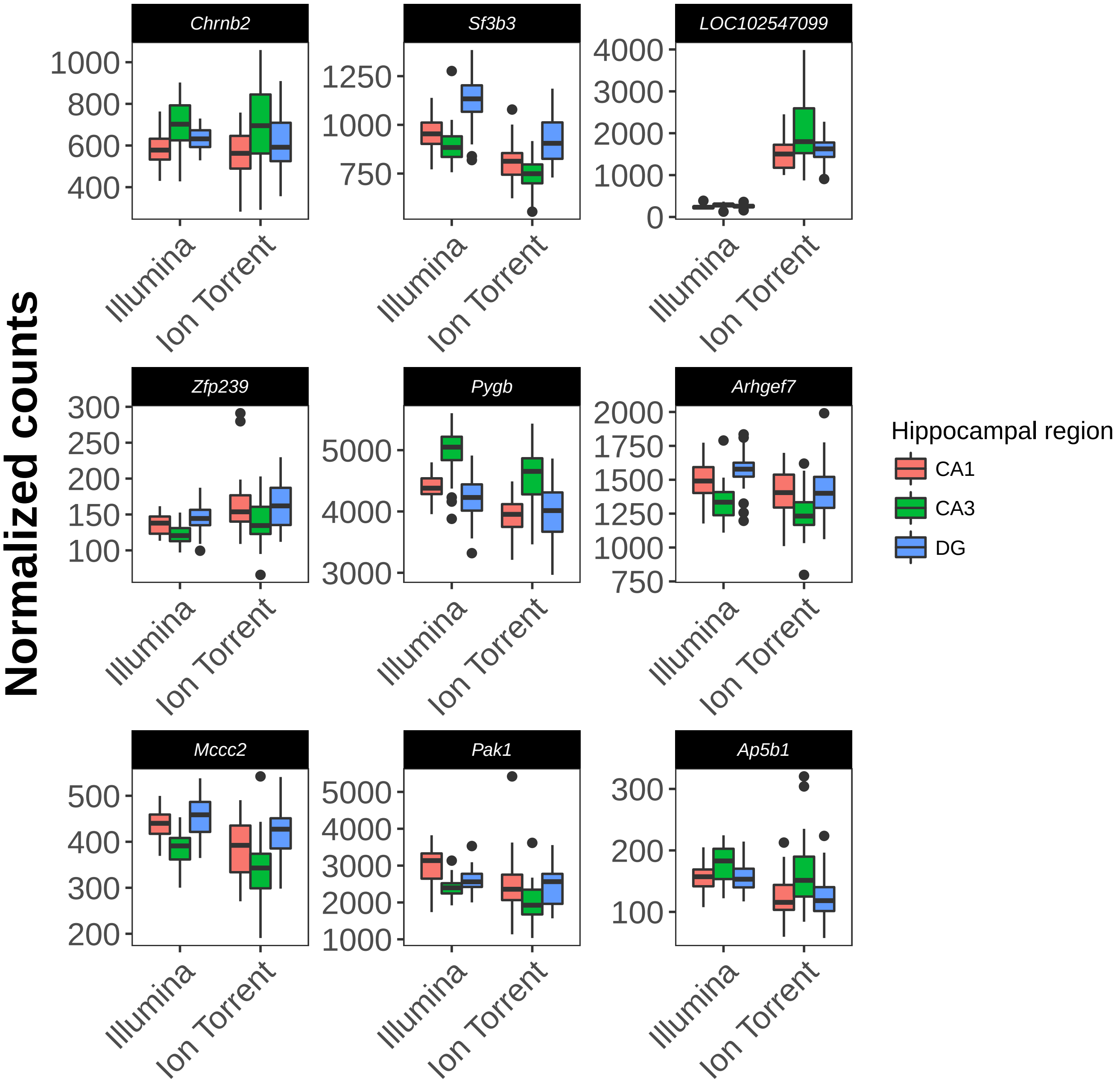

Normalized counts
Chrnb2
Sf3b3
LOC102547099
Zfp239
Pygb
Arhgef7
Mccc2
Pak1
Ap5b1
Illumina
Ion Torrent
Hippocampal region
CA1
CA3
DG

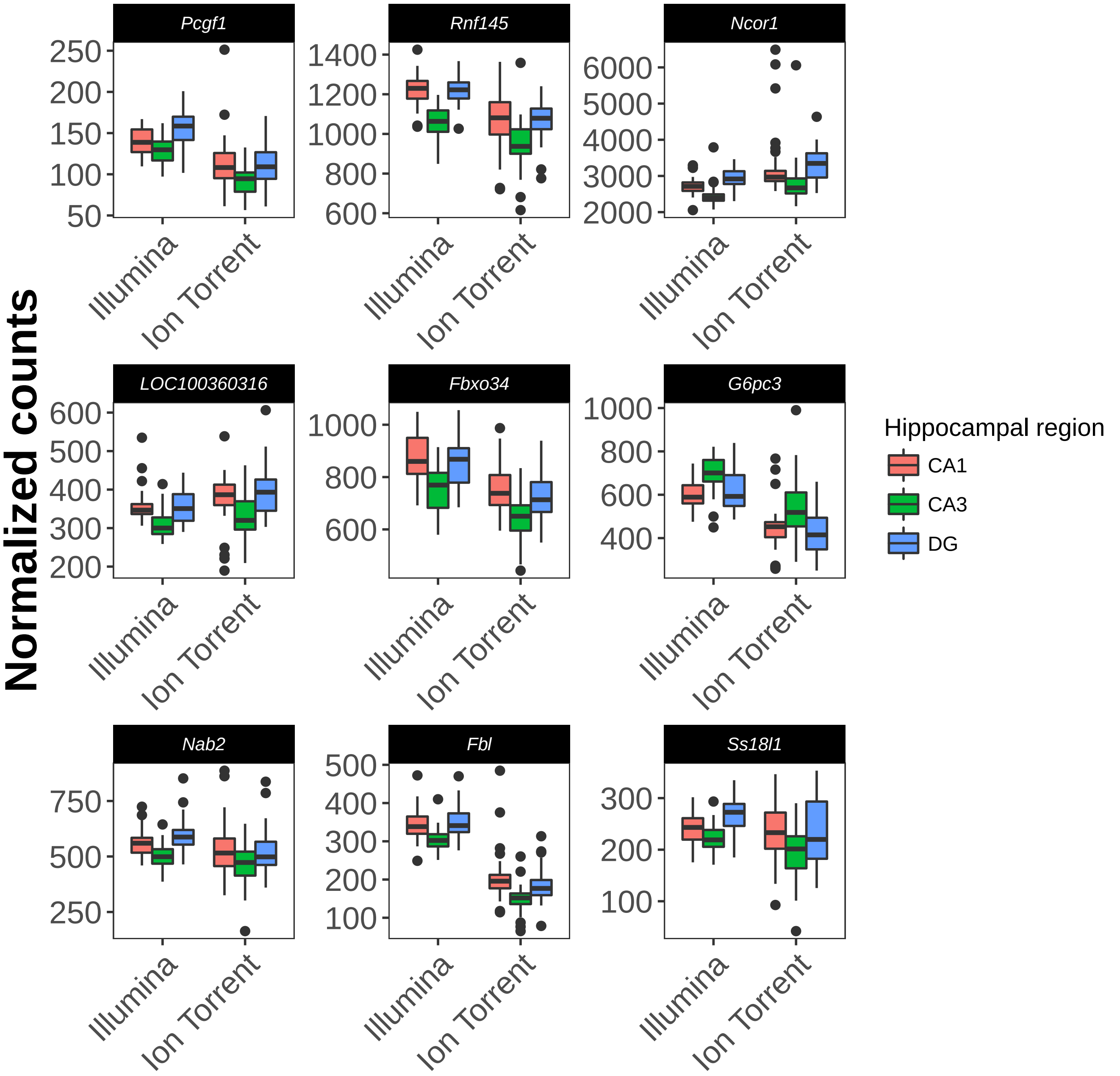

Normalized counts
Pcgf1
Rnf145
Ncor1
LOC100360316
Fbxo34
G6pc3
Nab2
Fbl
Ss18l1
Illumina
Ion Torrent
Hippocampal region
CA1
CA3
DG

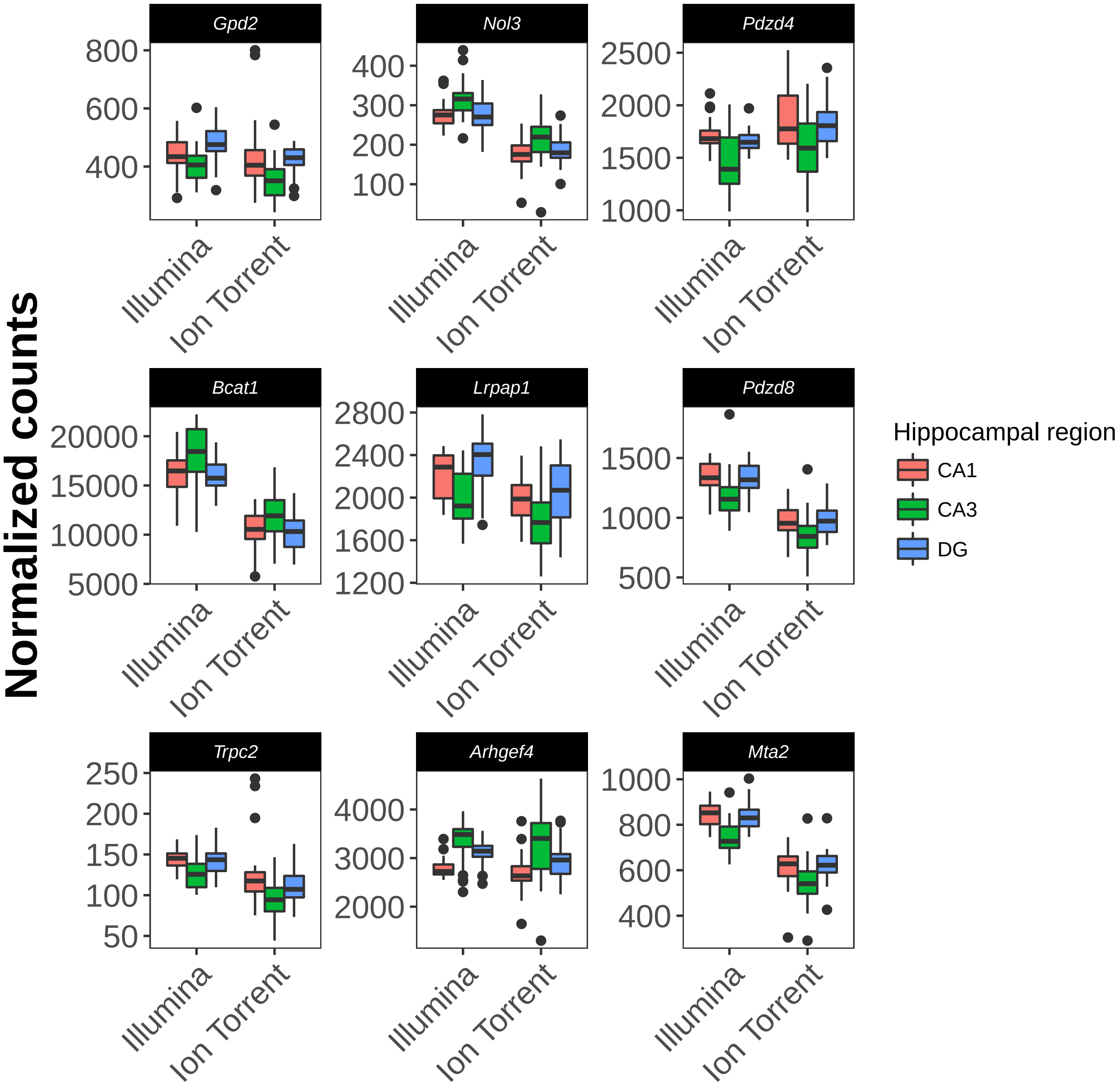

Normalized counts
Gpd2
Nol3
Pdzd4
Bcat1
Lrpap1
Pdzd8
Trpc2
Arhgef4
Mta2
Illumina
Ion Torrent
Hippocampal region
CA1
CA3
DG

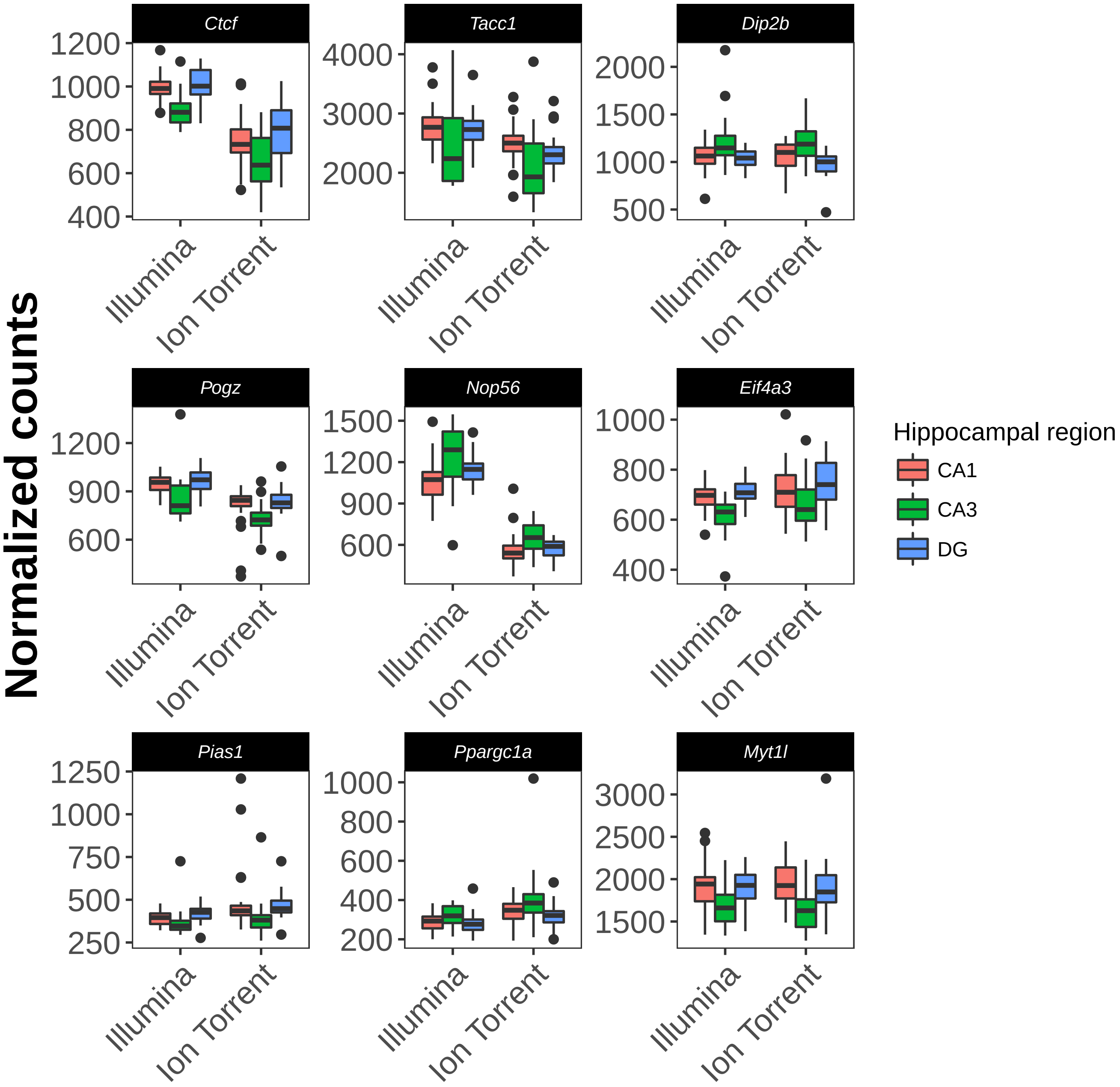
Normalized counts
Ctcf
Tacc1
Dip2b
Pogz
Nop56
Eif4a3
Pias1
Ppargc1a
Myt1l
Illumina
Ion Torrent
Hippocampal region
CA1
CA3
DG

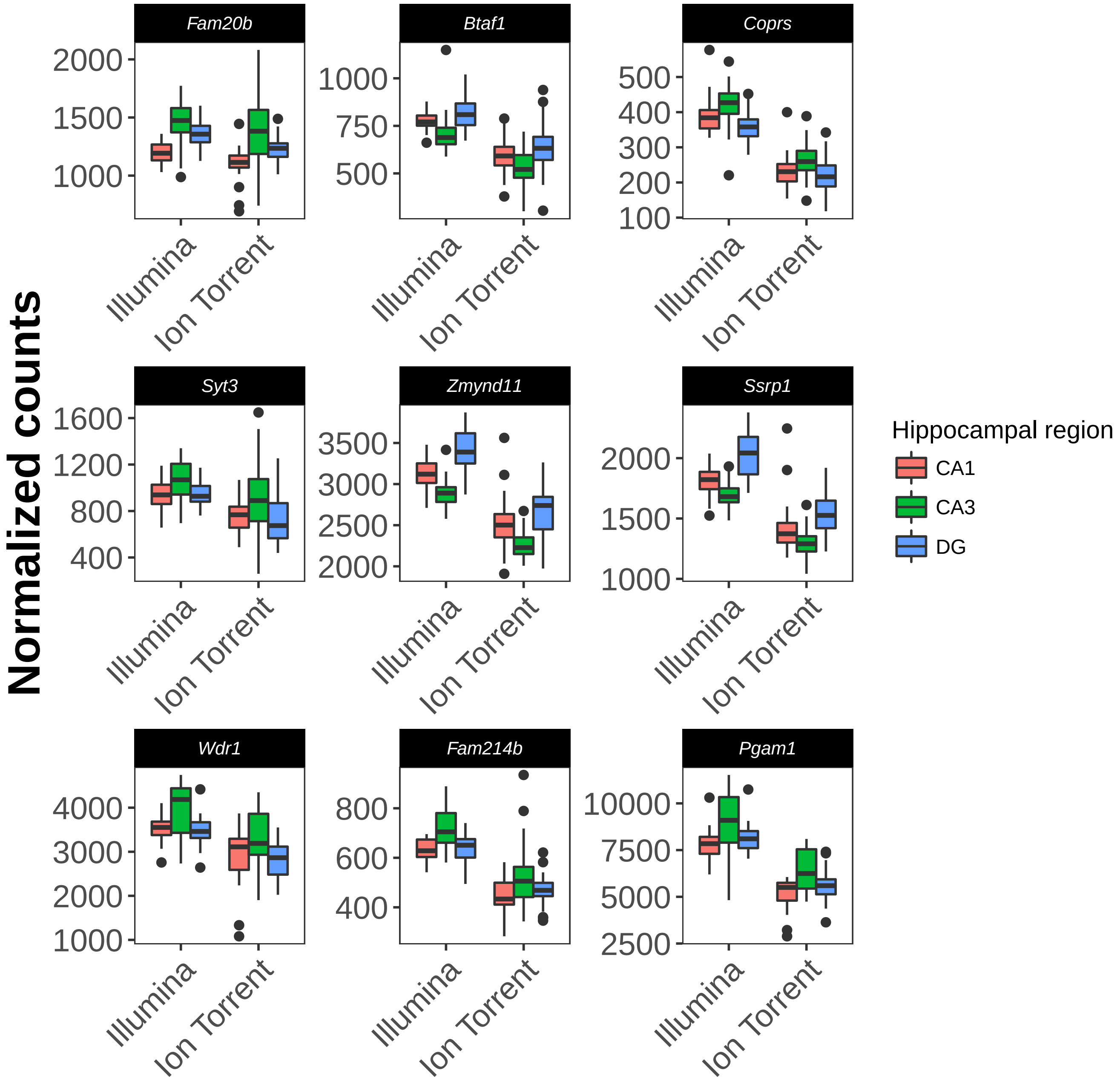

Normalized counts
Fam20b
Btaf1
Coprs
Syt3
Zmynd11
Ssrp1
Wdr1
Fam214b
Pgam1
Illumina
Ion Torrent
Hippocampal region
CA1
CA3
DG

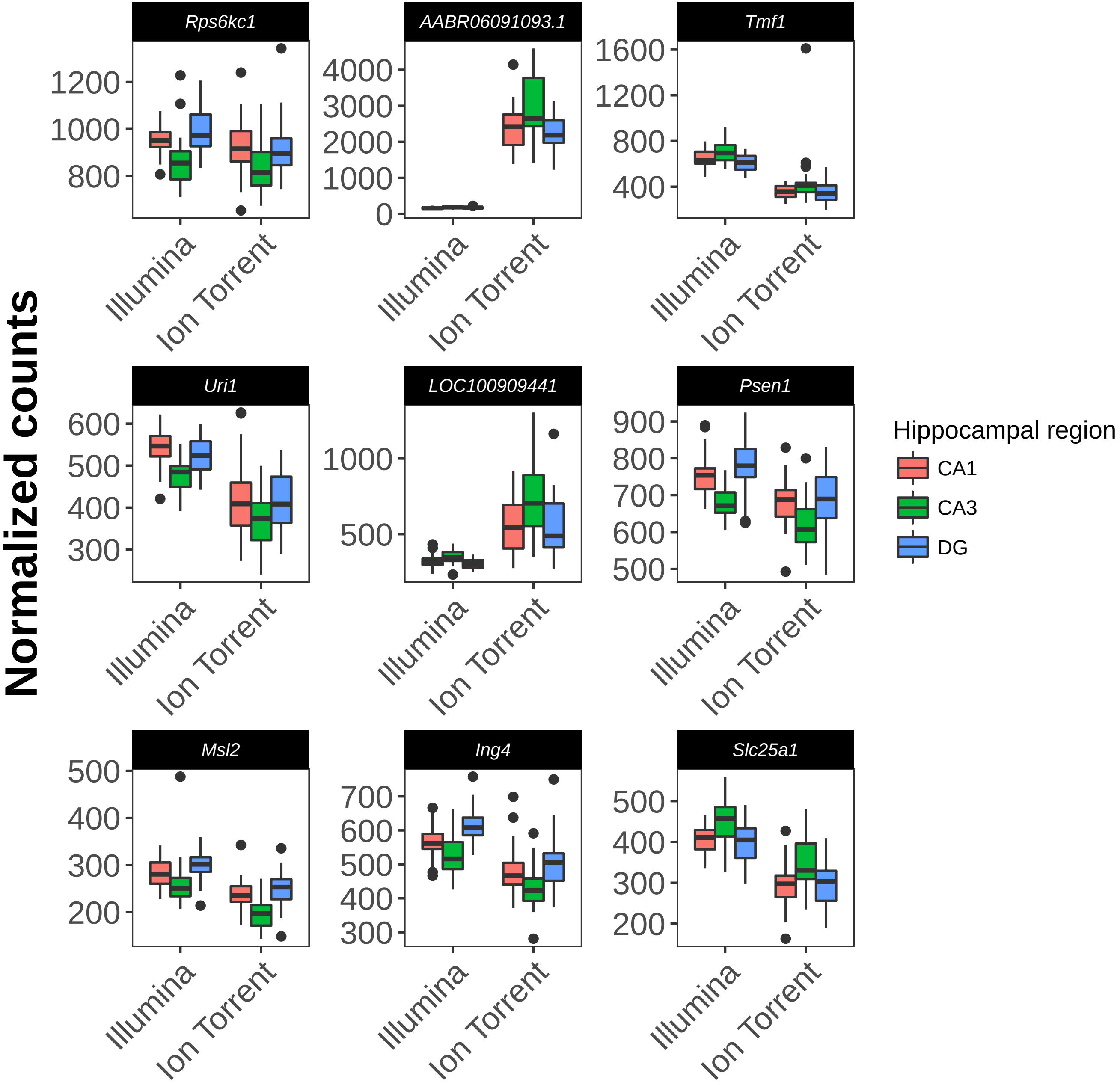

Normalized counts
Rps6kc1
AABR06091093.1
Tmf1
Uri1
LOC100909441
Psen1
Msl2
Ing4
Slc25a1
Illumina
Ion Torrent
Hippocampal region
CA1
CA3
DG

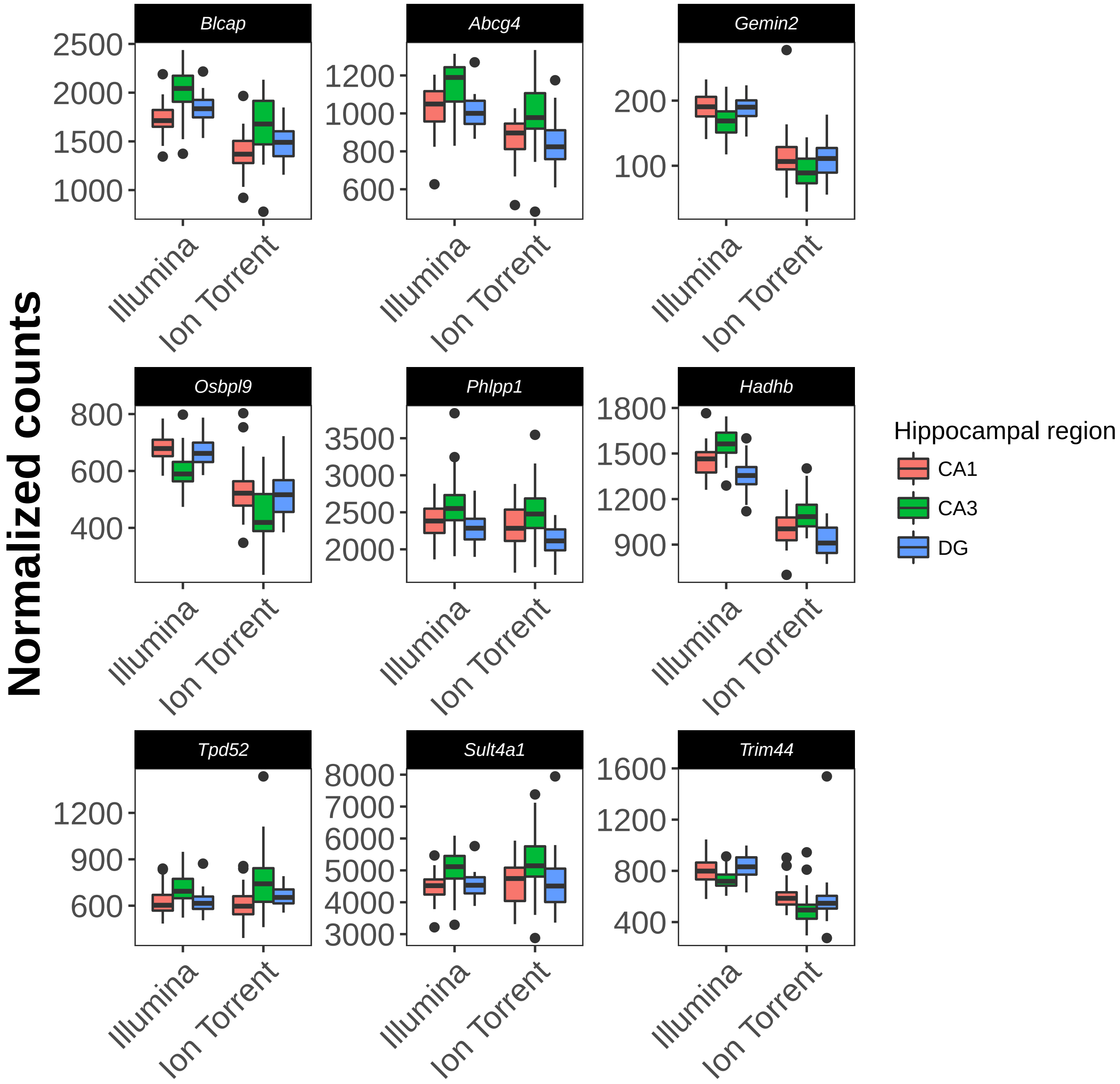

Normalized counts
Blcap
Abcg4
Gemin2
Osbpl9
Phlpp1
Hadhb
Tpd52
Sult4a1
Trim44
Illumina
Ion Torrent
Hippocampal region
CA1
CA3
DG

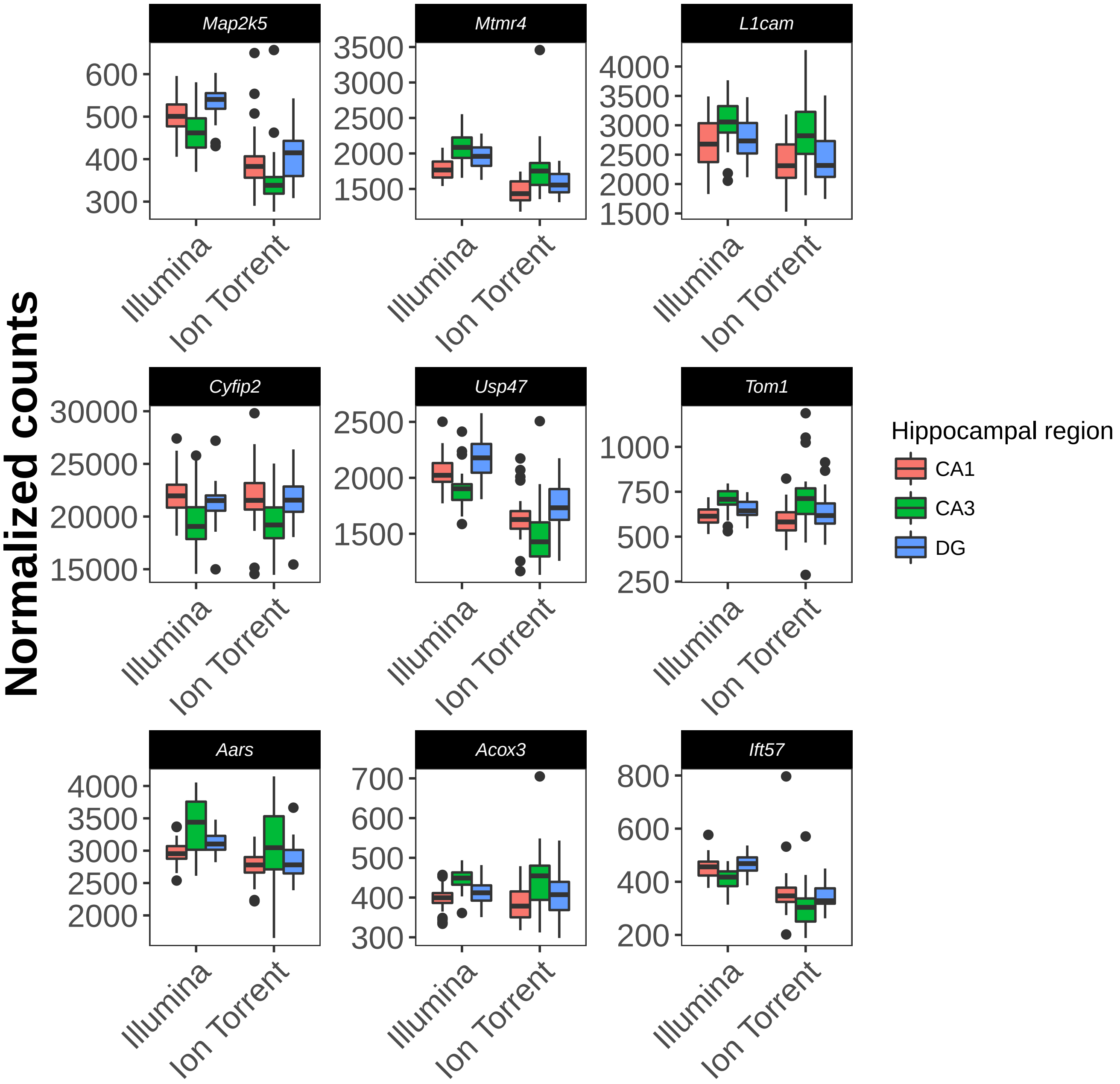
Normalized counts
Map2k5
Mtmr4
L1cam
Cyfip2
Usp47
Tom1
Aars
Acox3
Ift57
Illumina
Ion Torrent
Hippocampal region
CA1
CA3
DG

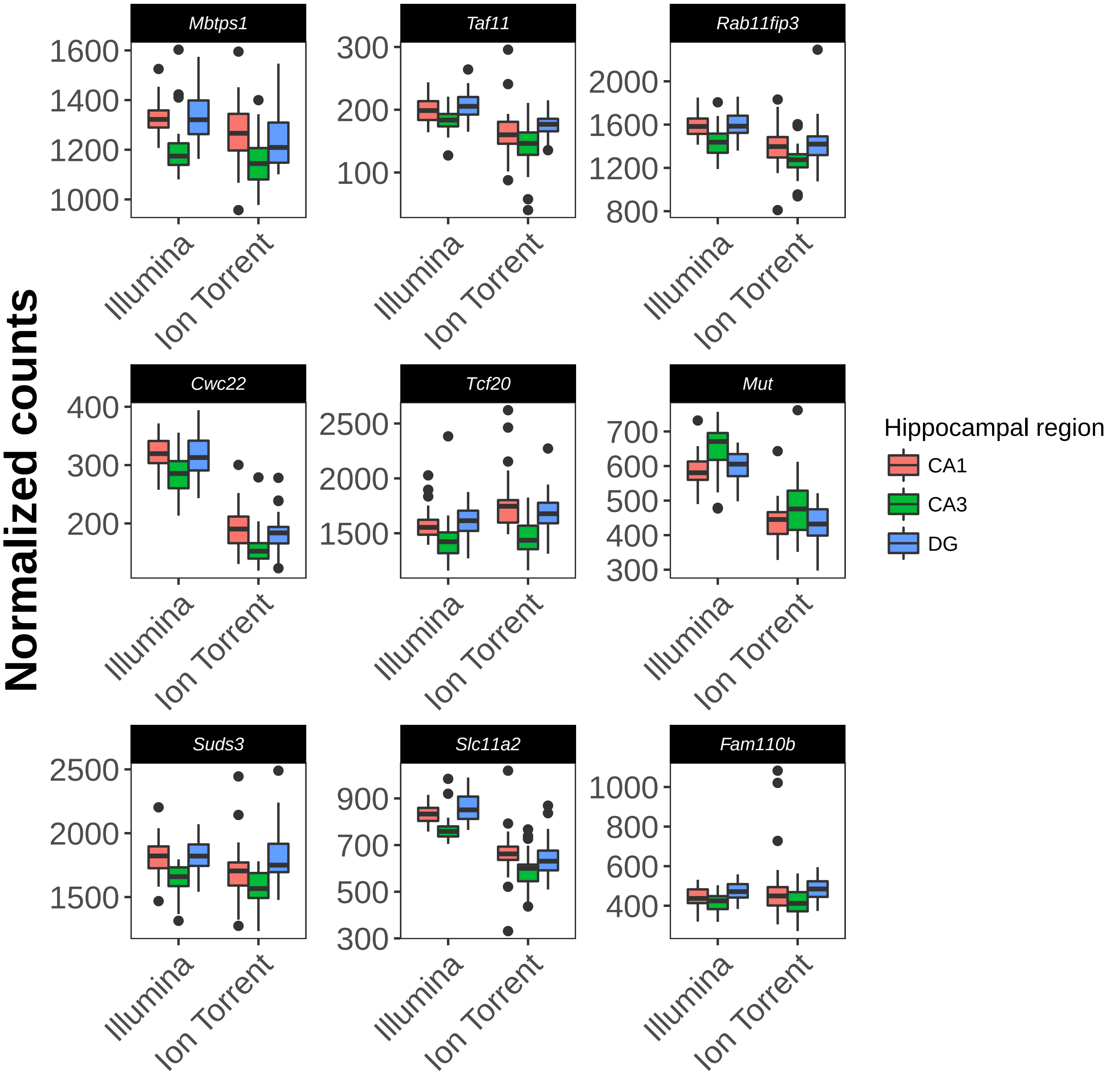

Mbtps1
Taf11
Rab11fip3
Cwc22
Tcf20
Mut
Suds3
Slc11a2
Fam110b
Illumina
Ion Torrent
Normalized counts
Hippocampal region
CA1
CA3
DG

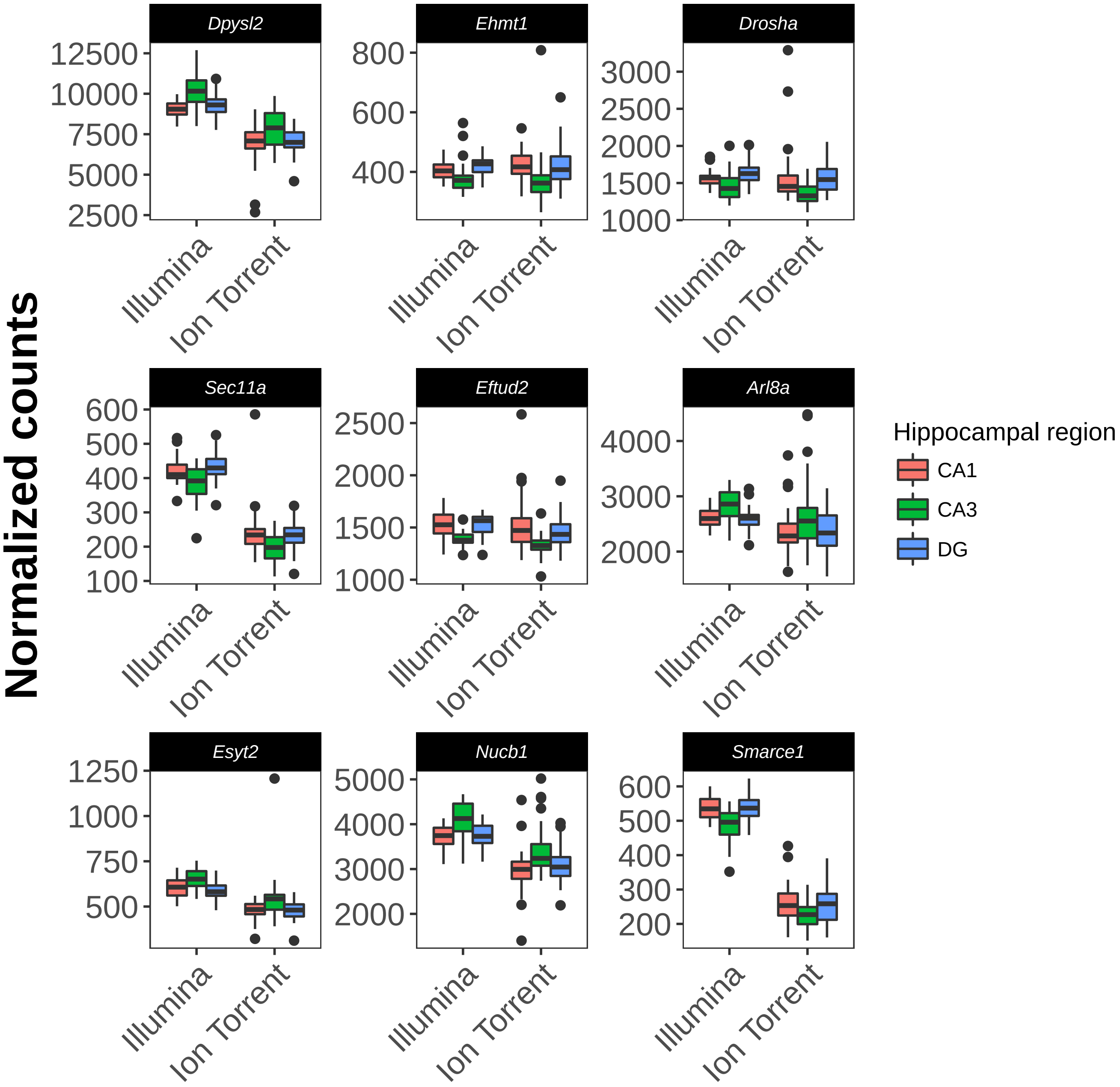
Normalized counts
Dpysl2
Ehmt1
Drosha
Sec11a
Eftud2
Arl8a
Esyt2
Nucb1
Smarce1
Illumina
Ion Torrent
Hippocampal region
CA1
CA3
DG

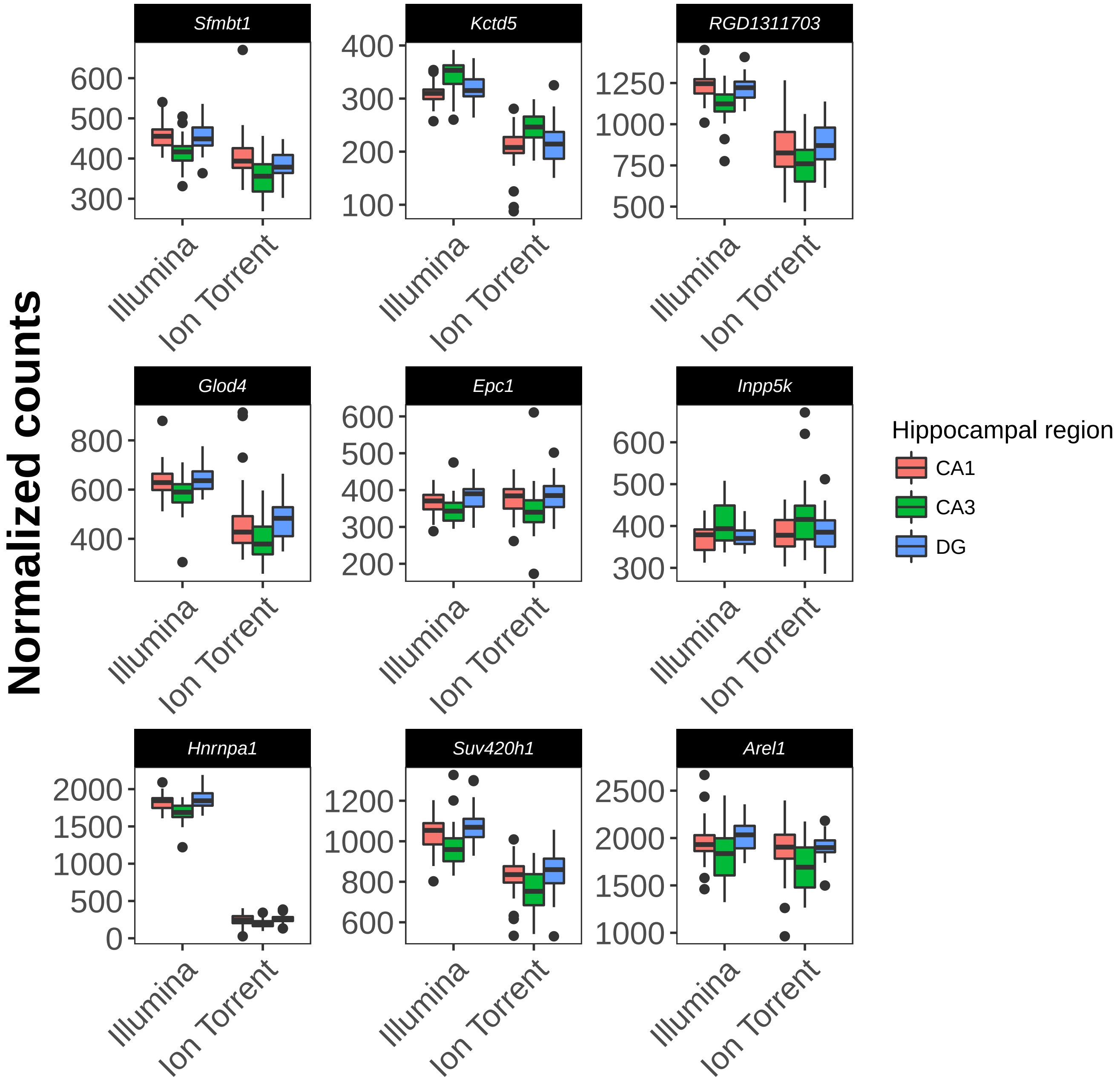

Normalized counts
Sfmbt1
Kctd5
RGD1311703
Glod4
Epc1
Inpp5k
Hnrnpa1
Suv420h1
Arel1
Illumina
Ion Torrent
Hippocampal region
CA1
CA3
DG

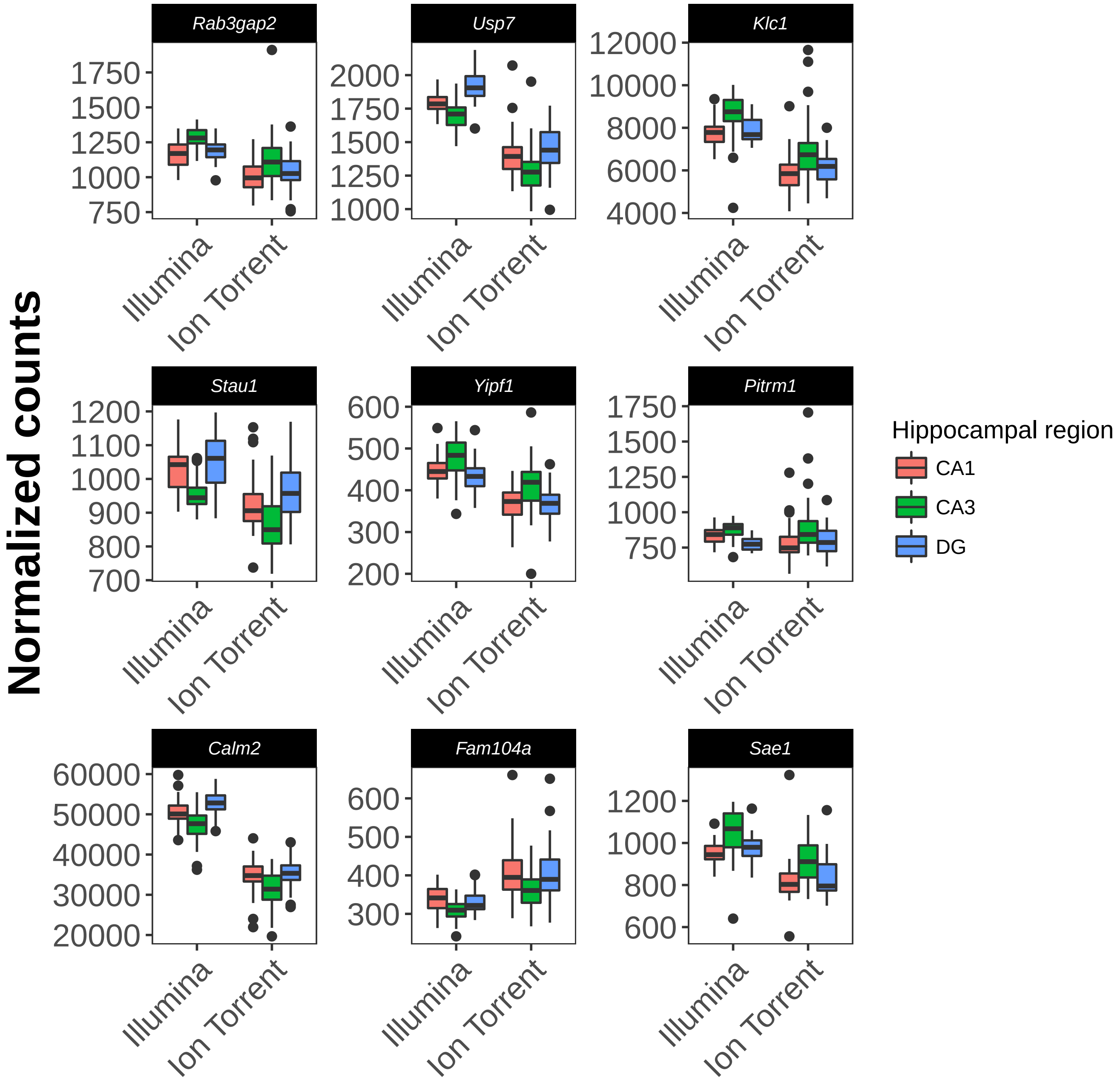

Normalized counts
Rab3gap2
Usp7
Klc1
Stau1
Yipf1
Pitrm1
Calm2
Fam104a
Sae1
Illumina
Ion Torrent
Hippocampal region
CA1
CA3
DG

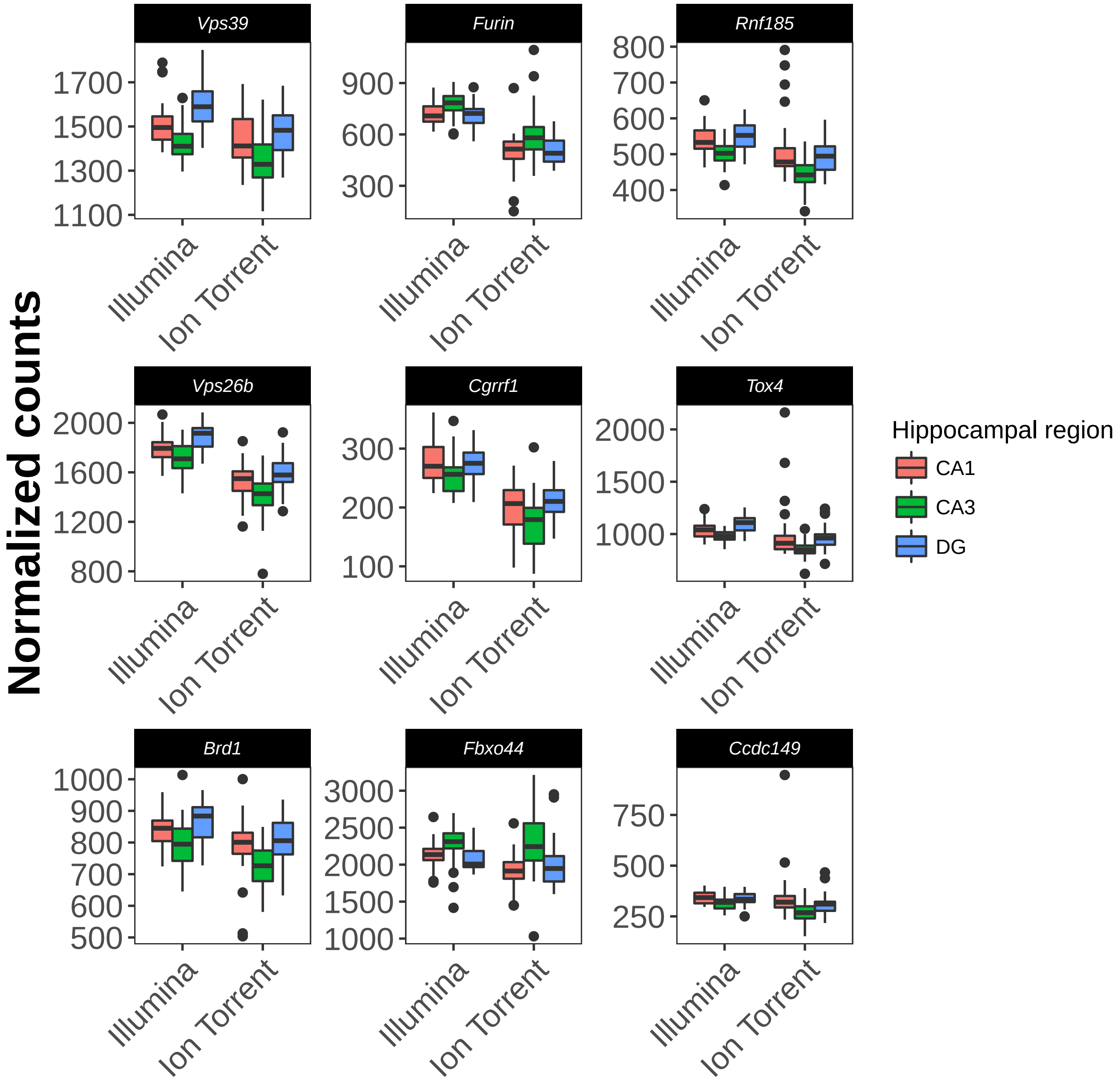

Normalized counts
Vps39
Furin
Rnf185
Vps26b
Cgrrf1
Tox4
Brd1
Fbxo44
Ccdc149
Illumina
Ion Torrent
Hippocampal region
CA1
CA3
DG

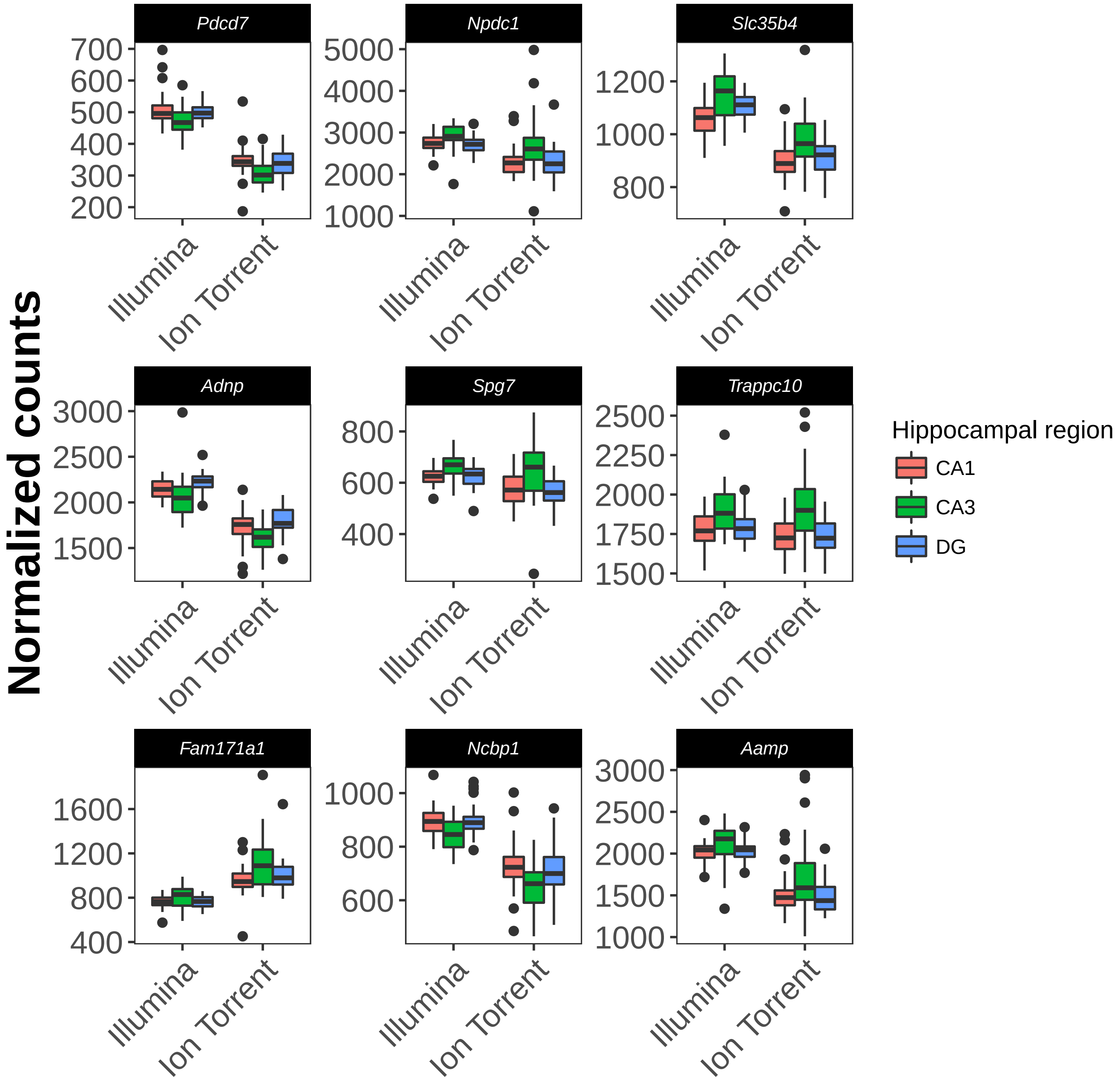

Normalized counts
Pdcd7
Npdc1
Slc35b4
Adnp
Spg7
Trappc10
Fam171a1
Ncbp1
Aamp
Illumina
Ion Torrent
Hippocampal region
CA1
CA3
DG

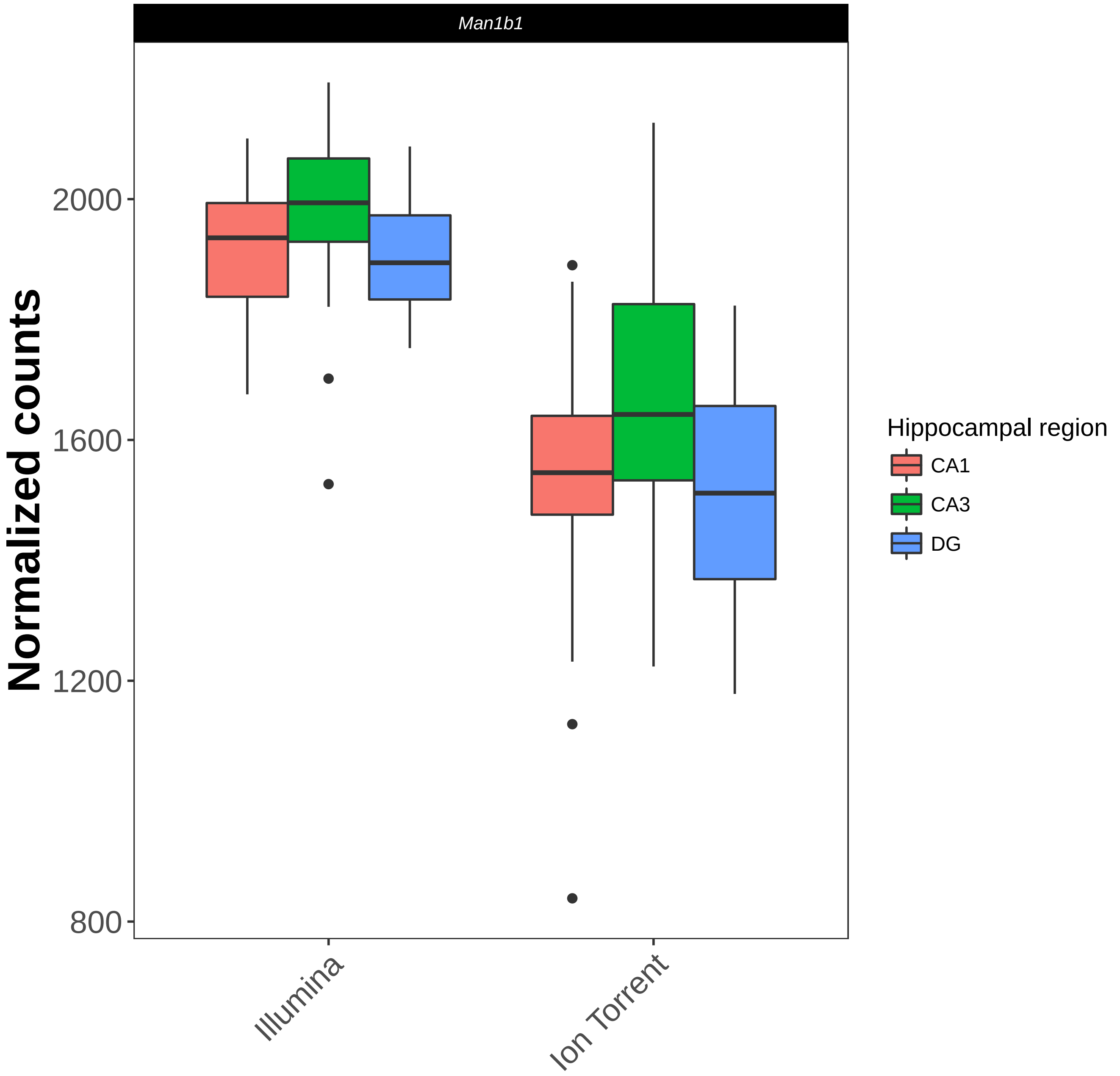

Man1b1
Normalized counts
2000
1600
1200
800
Illumina
Ion Torrent
Hippocampal region
CA1
CA3
DG
