# Supplementary material for: Hippocampal Transcriptomic Profiles: Subfield Vulnerability to Age and Cognitive Impairment
Source: Front Aging Neurosci. 2017 Dec 8;9:383. doi: 10.3389/fnagi.2017.00383 (PMC5727020; doi:10.3389/fnagi.2017.00383)
Supplement: Supplementary Figure 7 — Boxplots for the DG specific genes reported in Table S6. The normalized counts are from the Illumina experiment, and the genes are ranked according the average Fold Change computed from the pairwise comparisons DG vs. CA1 and DG vs. CA3. [file Image7.PDF]

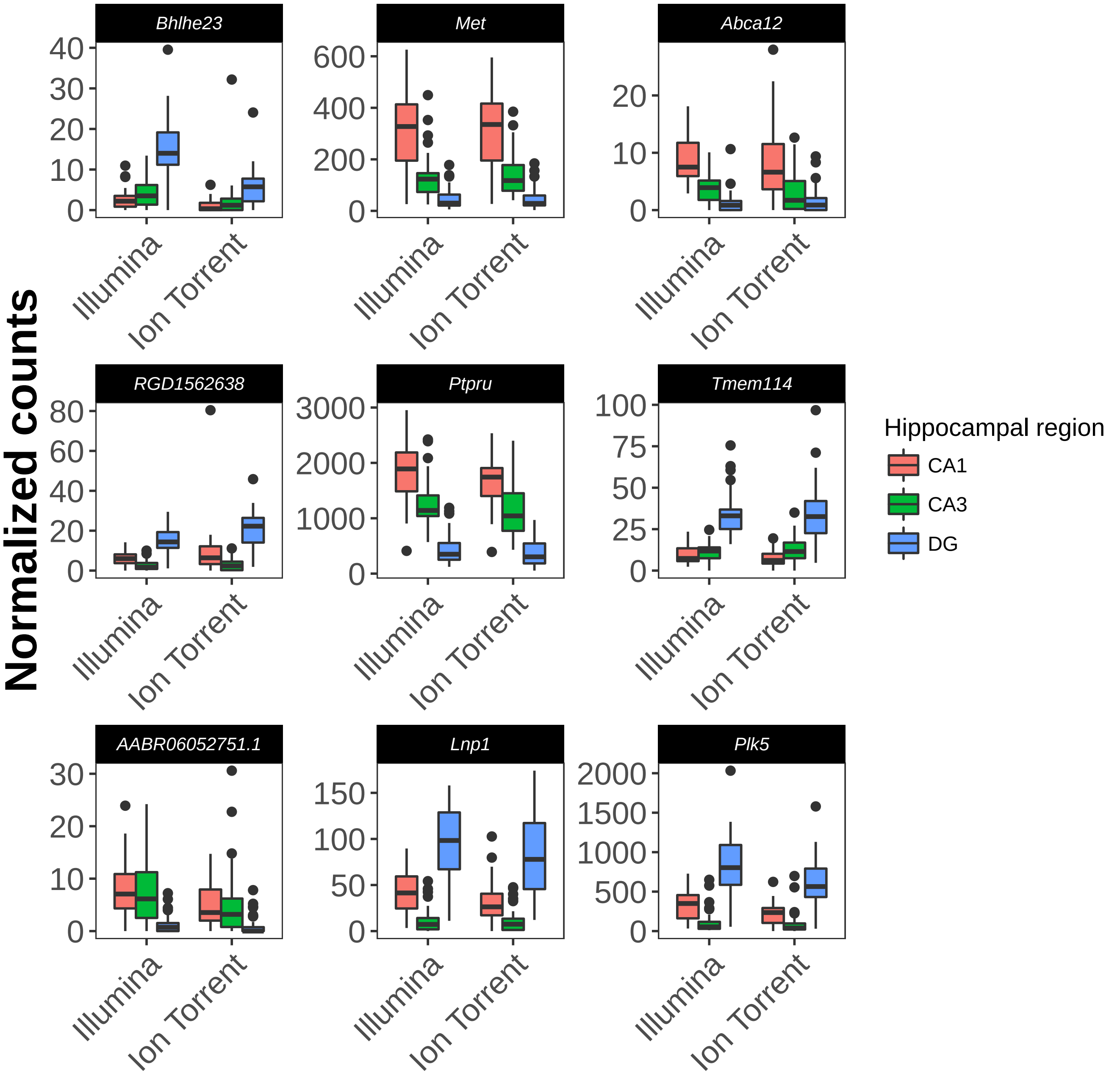

Normalized counts
Bhlhe23
Met
Abca12
RGD1562638
Ptpru
Tmem114
AABR06052751.1
Lnp1
Plk5
Illumina
Ion Torrent
Hippocampal region
CA1
CA3
DG

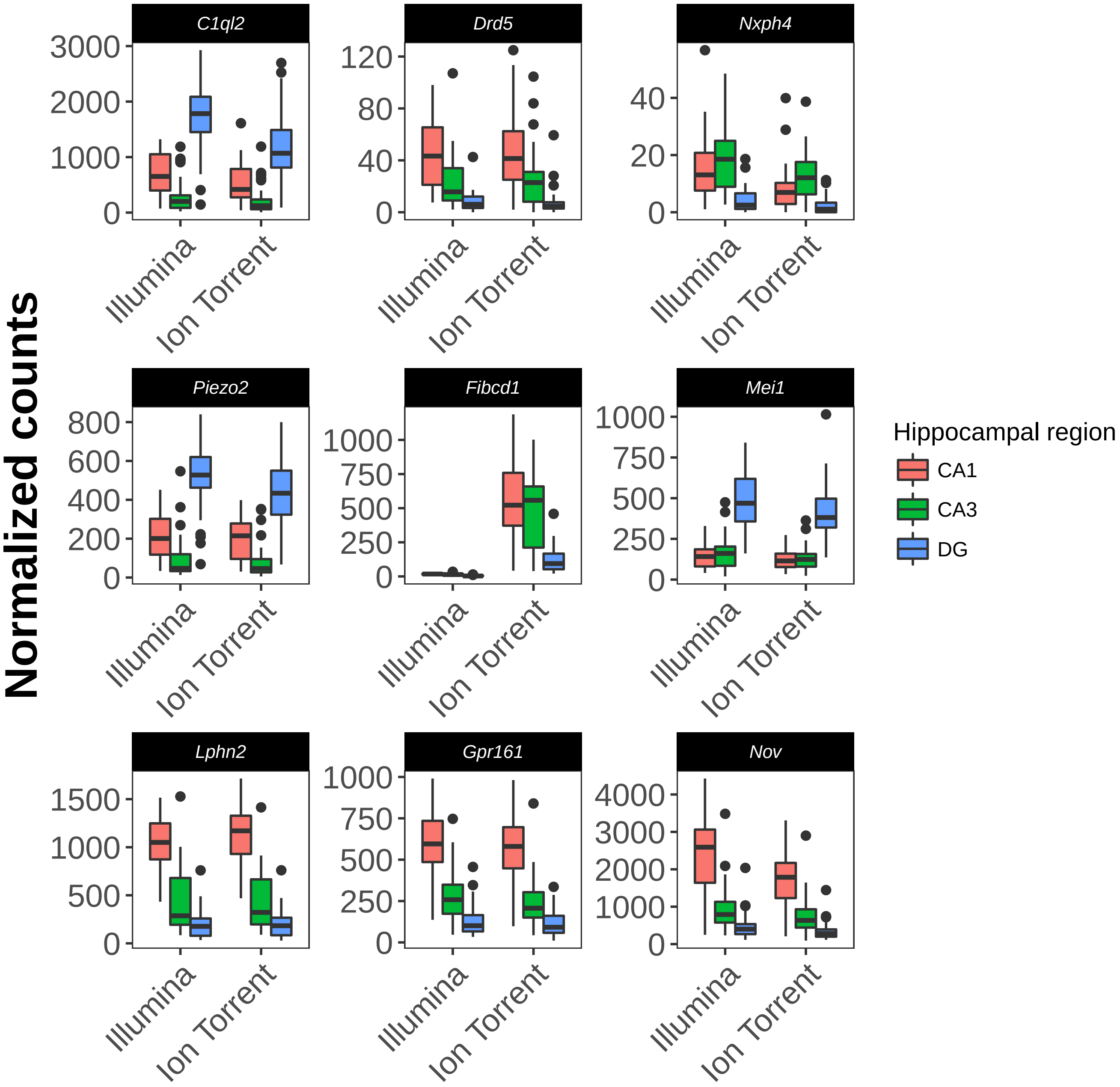
Normalized counts
C1ql2
Drd5
Nxph4
Piezo2
Fibcd1
Mei1
Lphn2
Gpr161
Nov
Illumina
Ion Torrent
Hippocampal region
CA1
CA3
DG

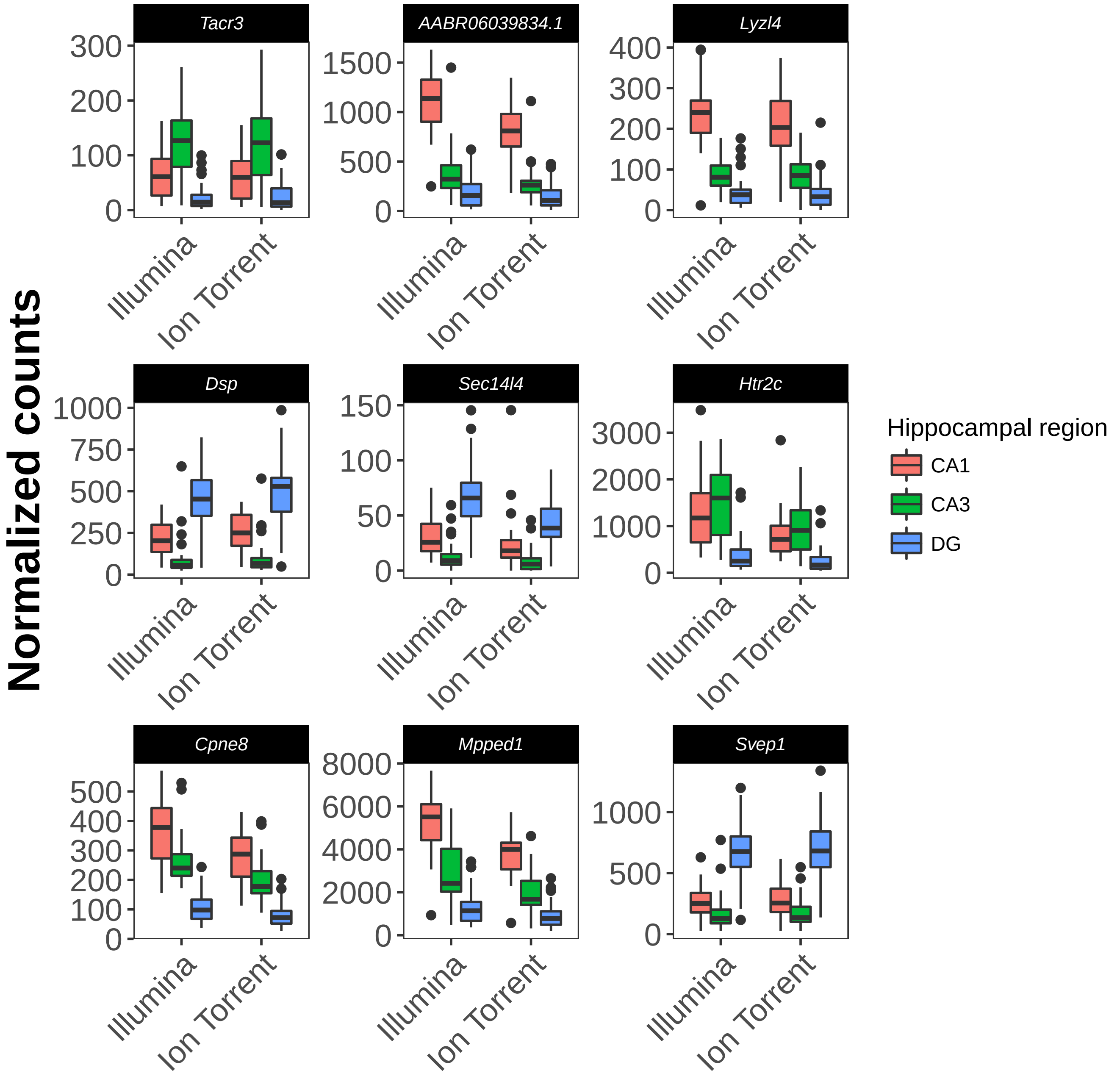
Normalized counts
Tacr3
AABR06039834.1
Lyzl4
Dsp
Sec14l4
Htr2c
Cpne8
Mpped1
Svep1
Illumina
Ion Torrent
Hippocampal region
CA1
CA3
DG

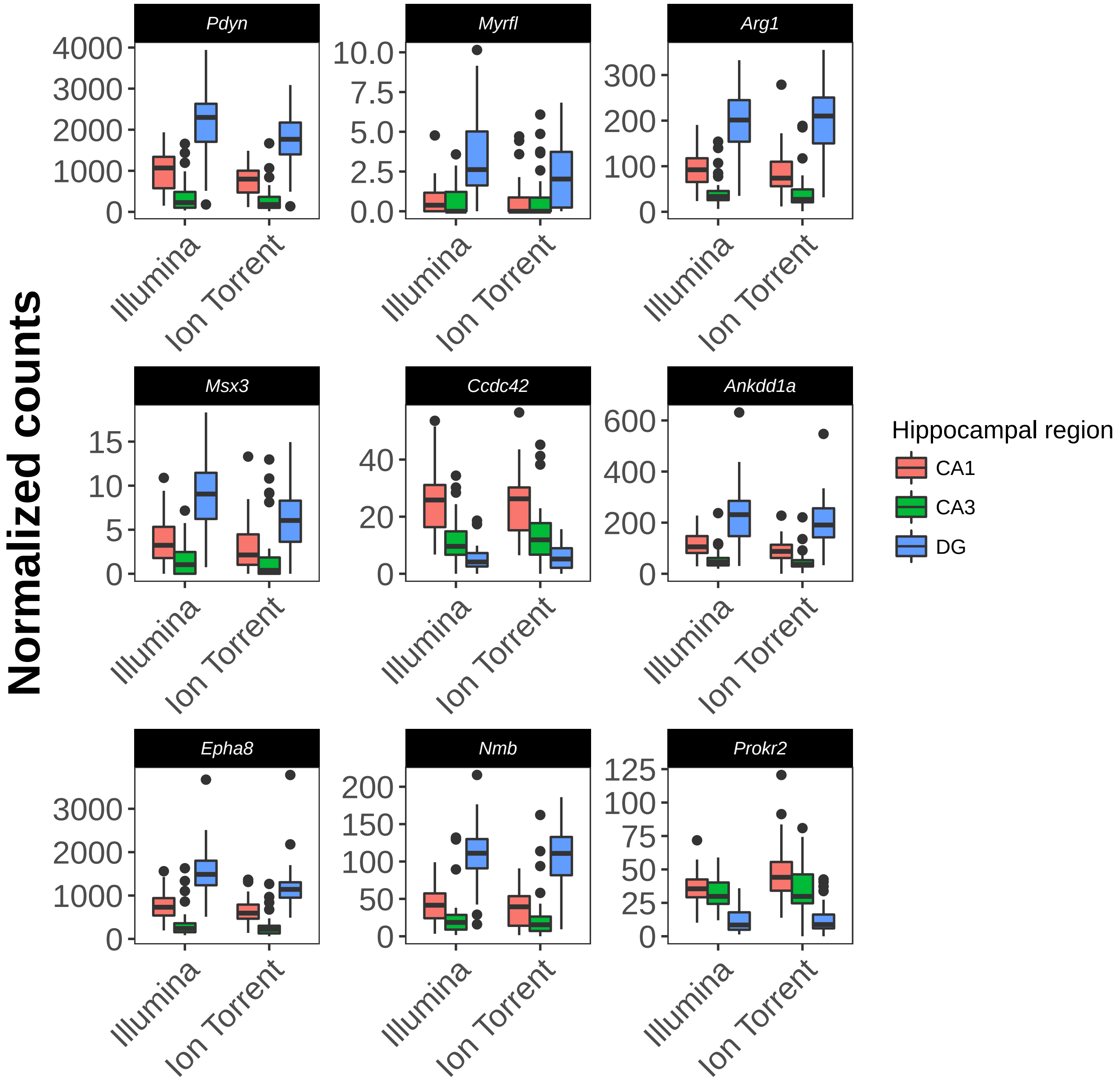
Normalized counts
Pdyn
Myrfl
Arg1
Msx3
Ccdc42
Ankdd1a
Epha8
Nmb
Prokr2
Illumina
Ion Torrent
Hippocampal region
CA1
CA3
DG

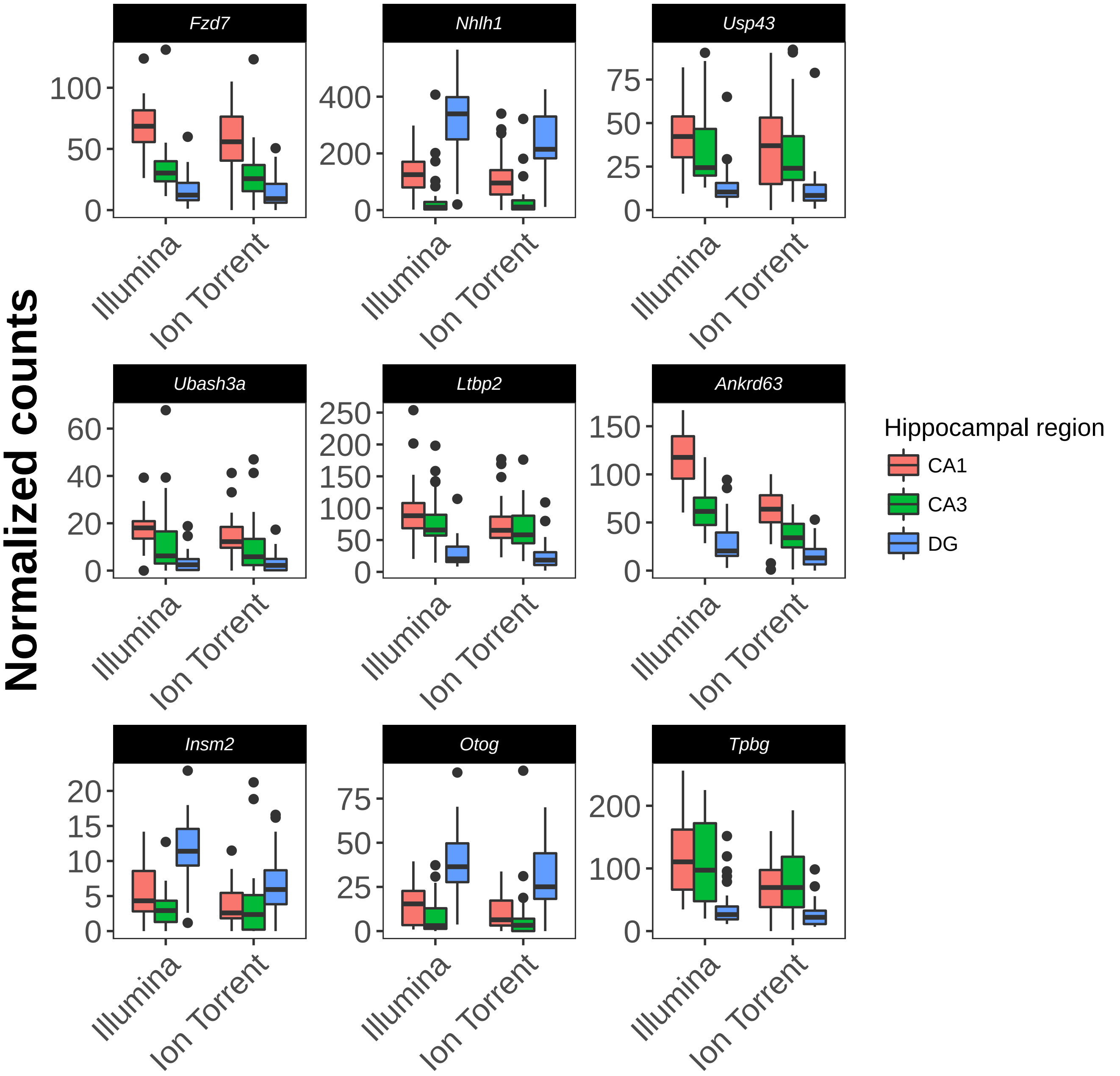
Normalized counts
Fzd7
Nhlh1
Usp43
Ubash3a
Ltbp2
Ankrd63
Insm2
Otog
Tpbg
Illumina
Ion Torrent
Hippocampal region
CA1
CA3
DG

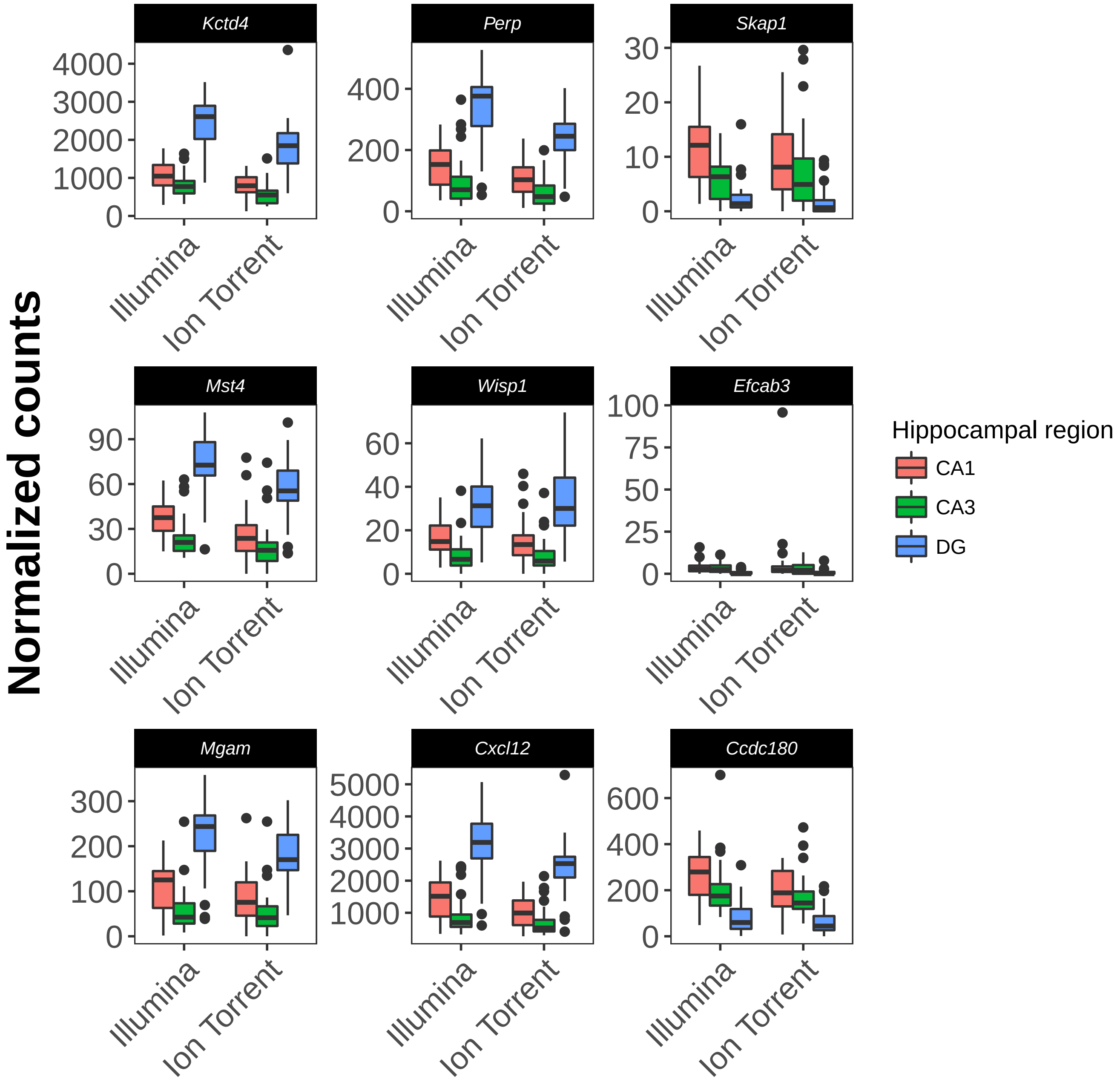
Normalized counts
Kctd4
Perp
Skap1
Mst4
Wisp1
Efcab3
Mgam
Cxcl12
Ccdc180
Illumina
Ion Torrent
Hippocampal region
CA1
CA3
DG

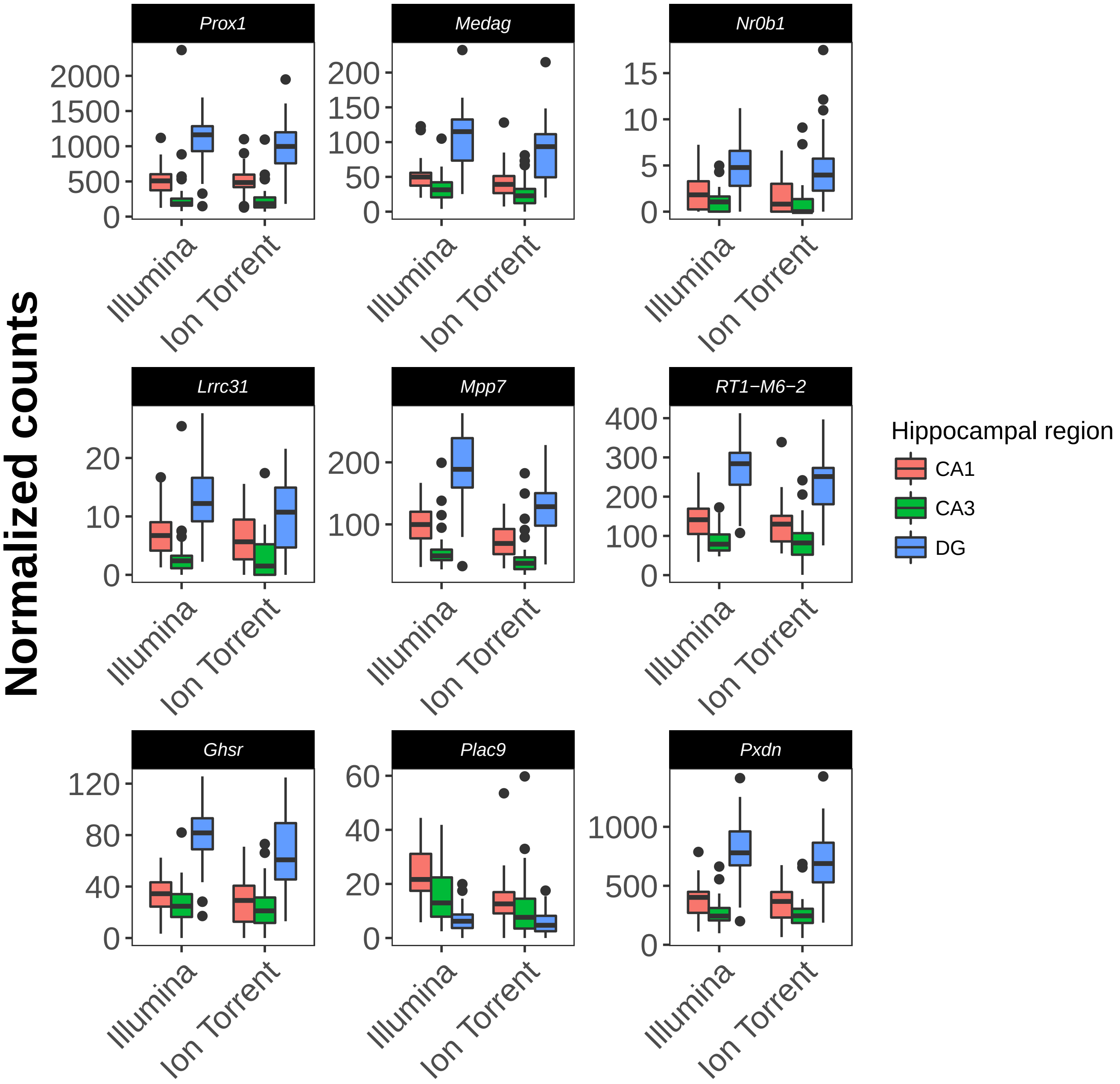

Normalized counts
Prox1
Medag
Nr0b1
Lrrc31
Mpp7
RT1−M6−2
Ghsr
Plac9
Pxdn
Illumina
Ion Torrent
Hippocampal region
CA1
CA3
DG

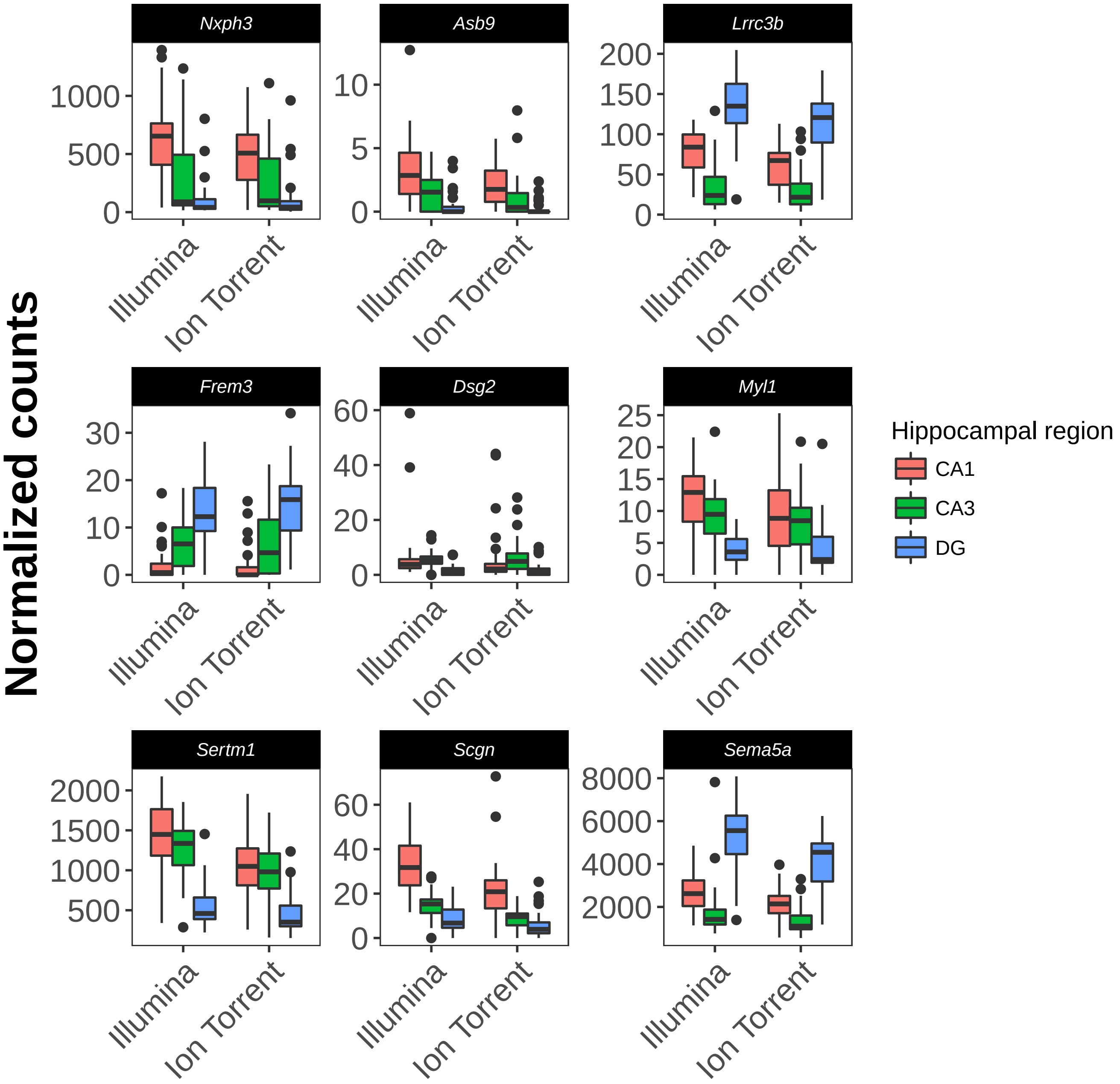
Nxph3
Asb9
Lrrc3b
Frem3
Dsg2
Myl1
Sertm1
Scgn
Sema5a
Normalized counts
Illumina
Ion Torrent
Hippocampal region
CA1
CA3
DG

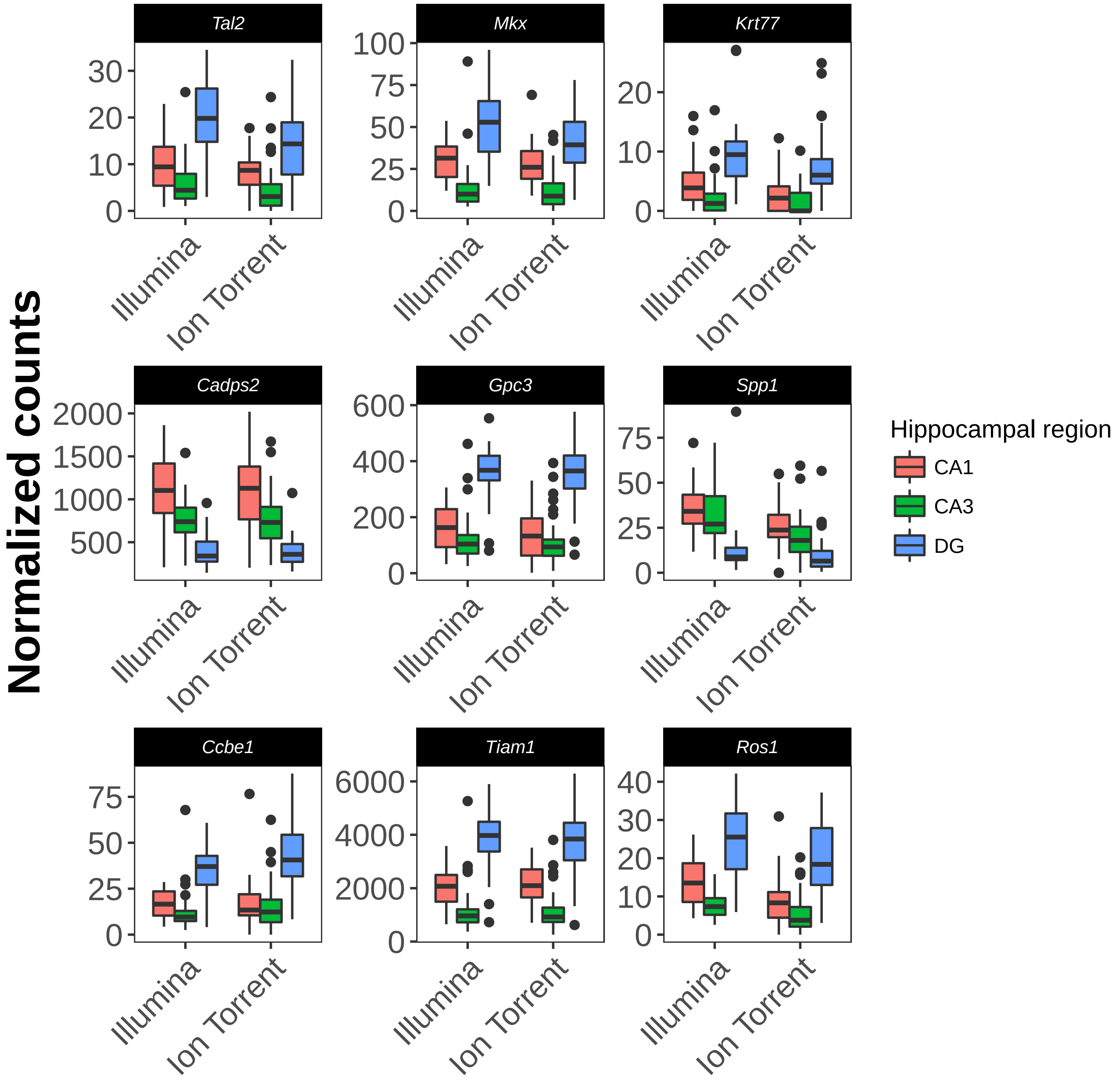

Normalized counts
Tal2
Mkx
Krt77
Cadps2
Gpc3
Spp1
Ccbe1
Tiam1
Ros1
Illumina
Ion Torrent
Hippocampal region
CA1
CA3
DG

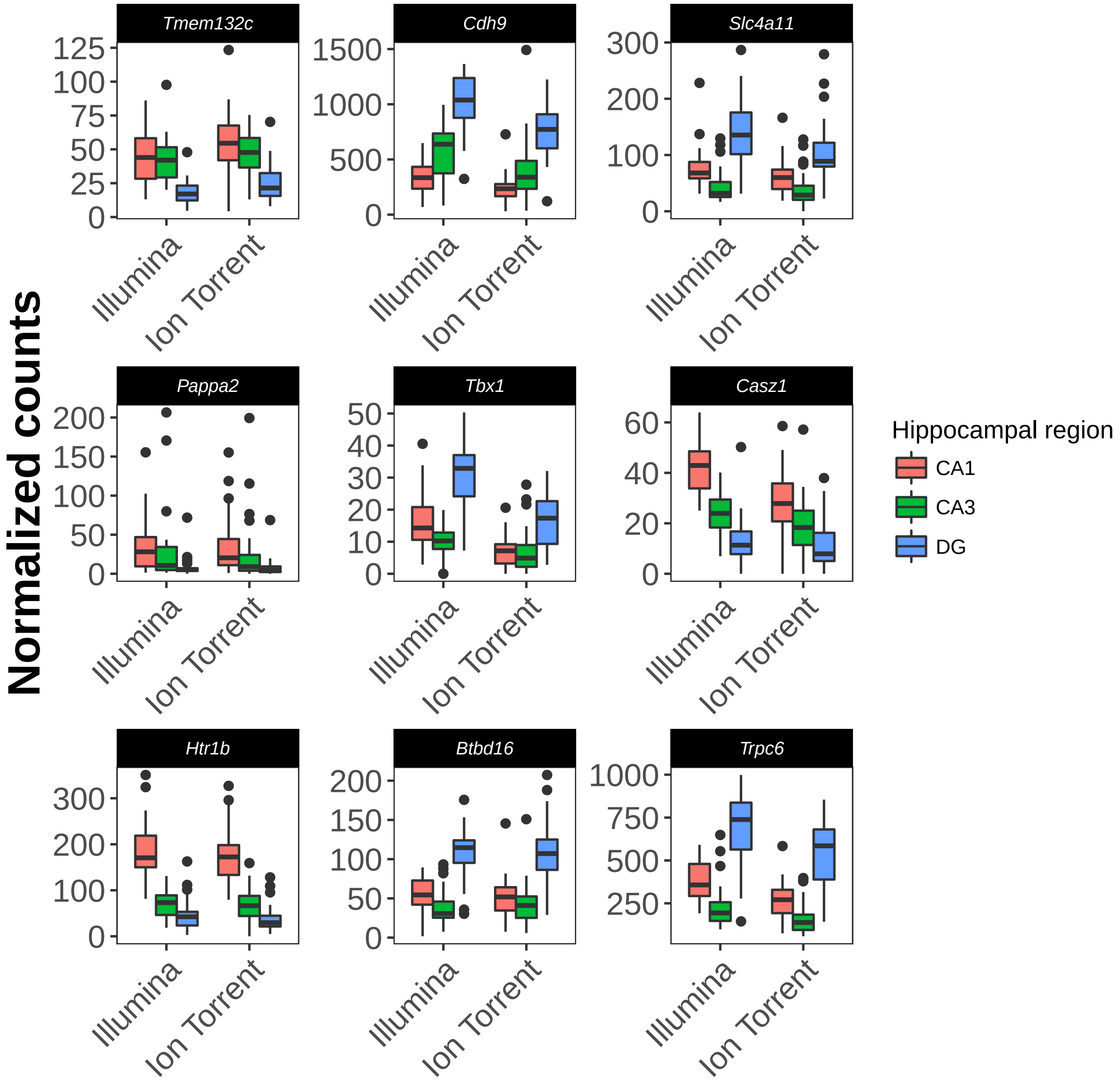
Normalized counts
Tmem132c
Cdh9
Slc4a11
Pappa2
Tbx1
Casz1
Htr1b
Btbd16
Trpc6
Illumina
Ion Torrent
Hippocampal region
CA1
CA3
DG

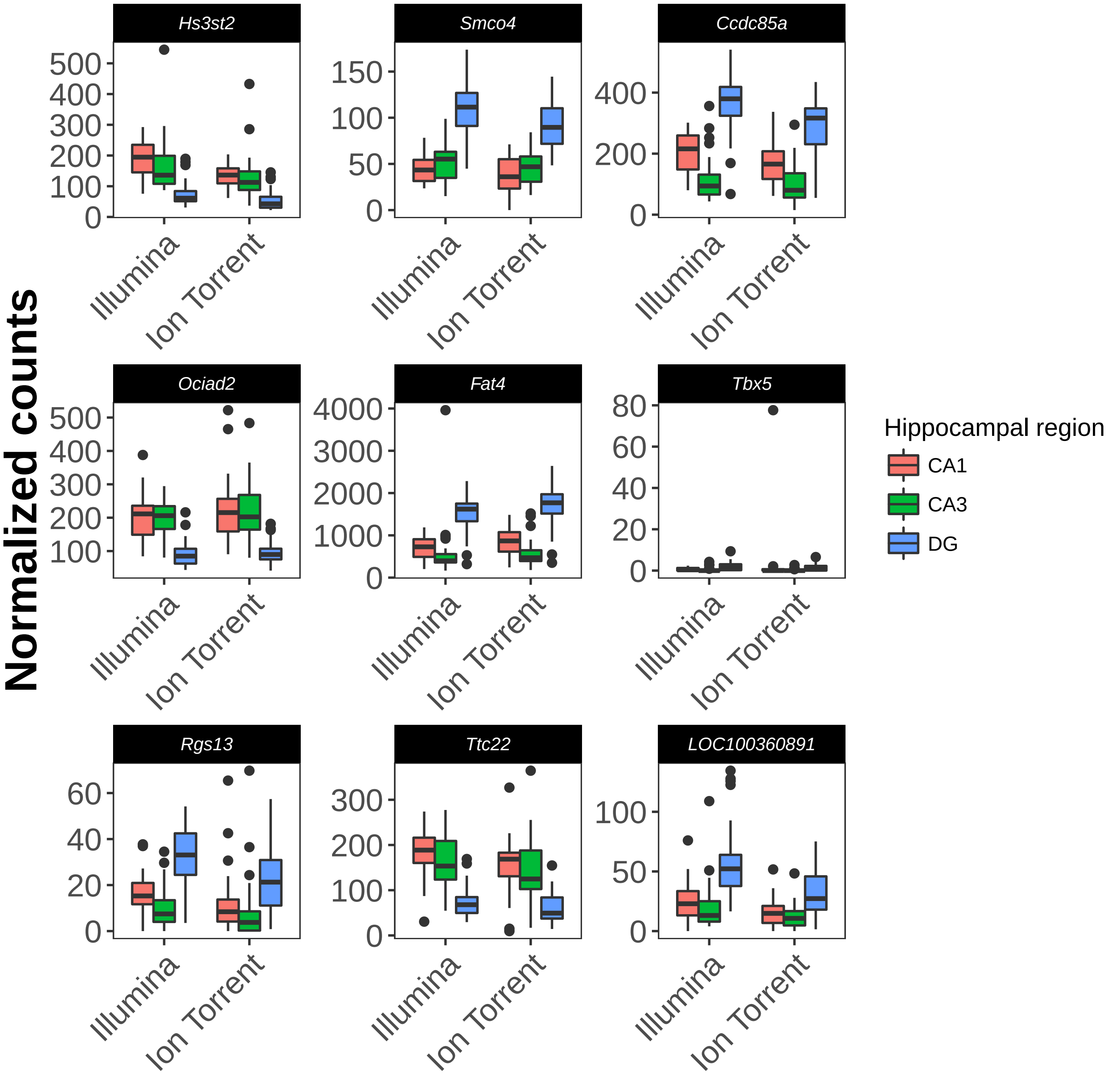

Normalized counts
Hs3st2
Smco4
Ccdc85a
Ociad2
Fat4
Tbx5
Rgs13
Ttc22
LOC100360891
Illumina
Ion Torrent
Hippocampal region
CA1
CA3
DG

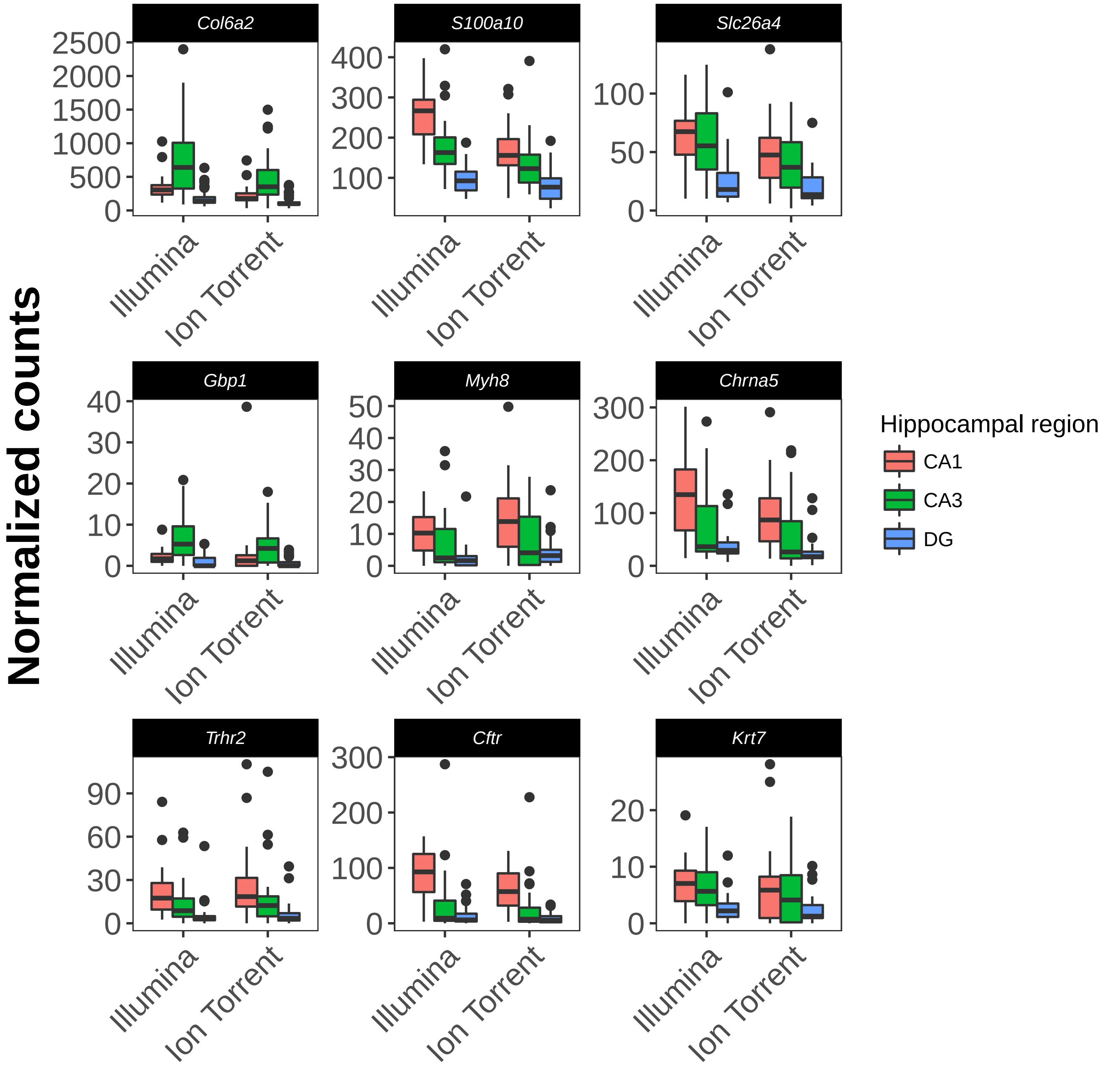
Normalized counts
Col6a2
S100a10
Slc26a4
Gbp1
Myh8
Chrna5
Trhr2
Cftr
Krt7
Illumina
Ion Torrent
Hippocampal region
CA1
CA3
DG

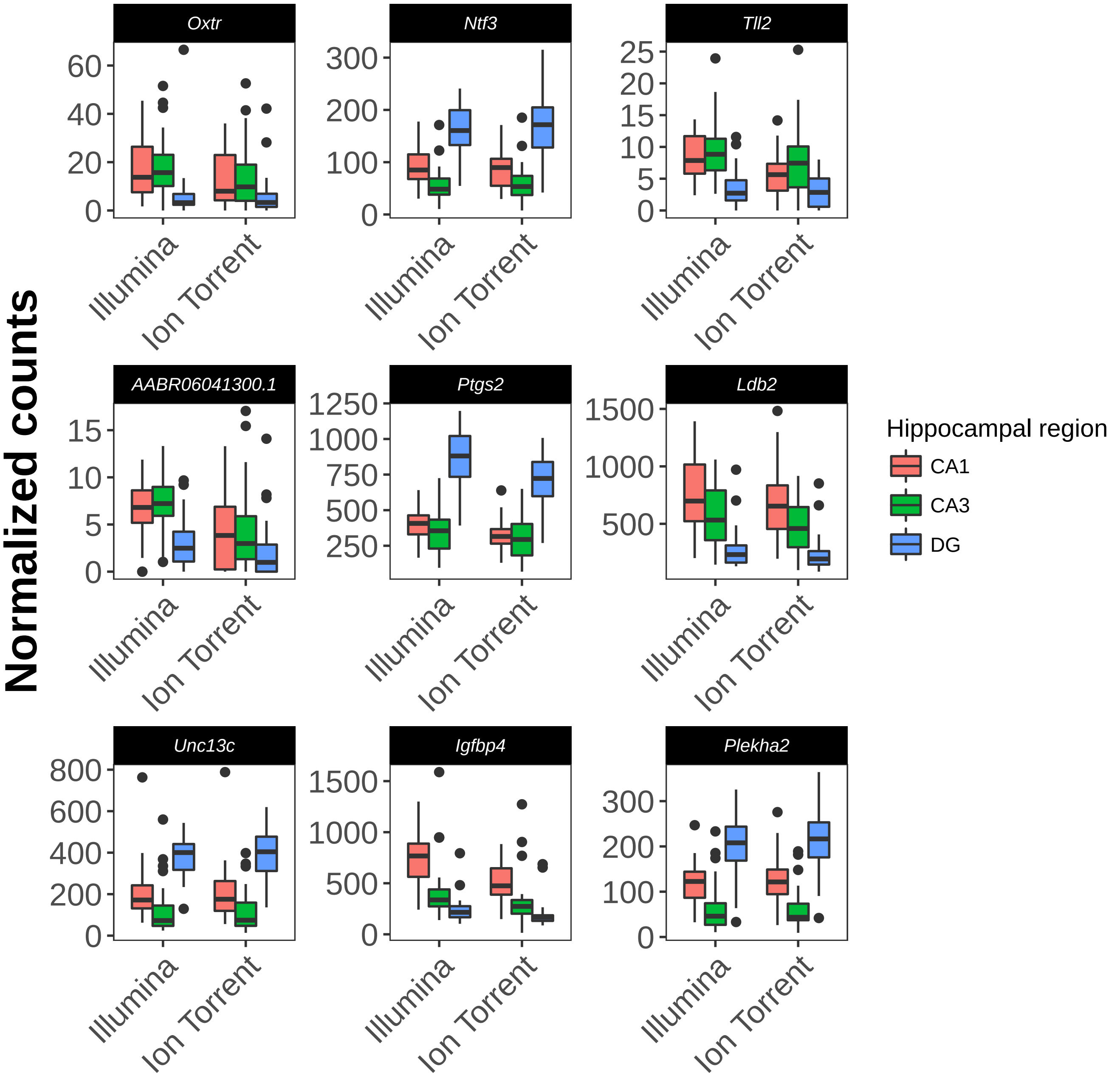

Normalized counts
Oxtr
Ntf3
Tll2
AABR06041300.1
Ptgs2
Ldb2
Unc13c
Igfbp4
Plekha2
Illumina
Ion Torrent
Hippocampal region
CA1
CA3
DG

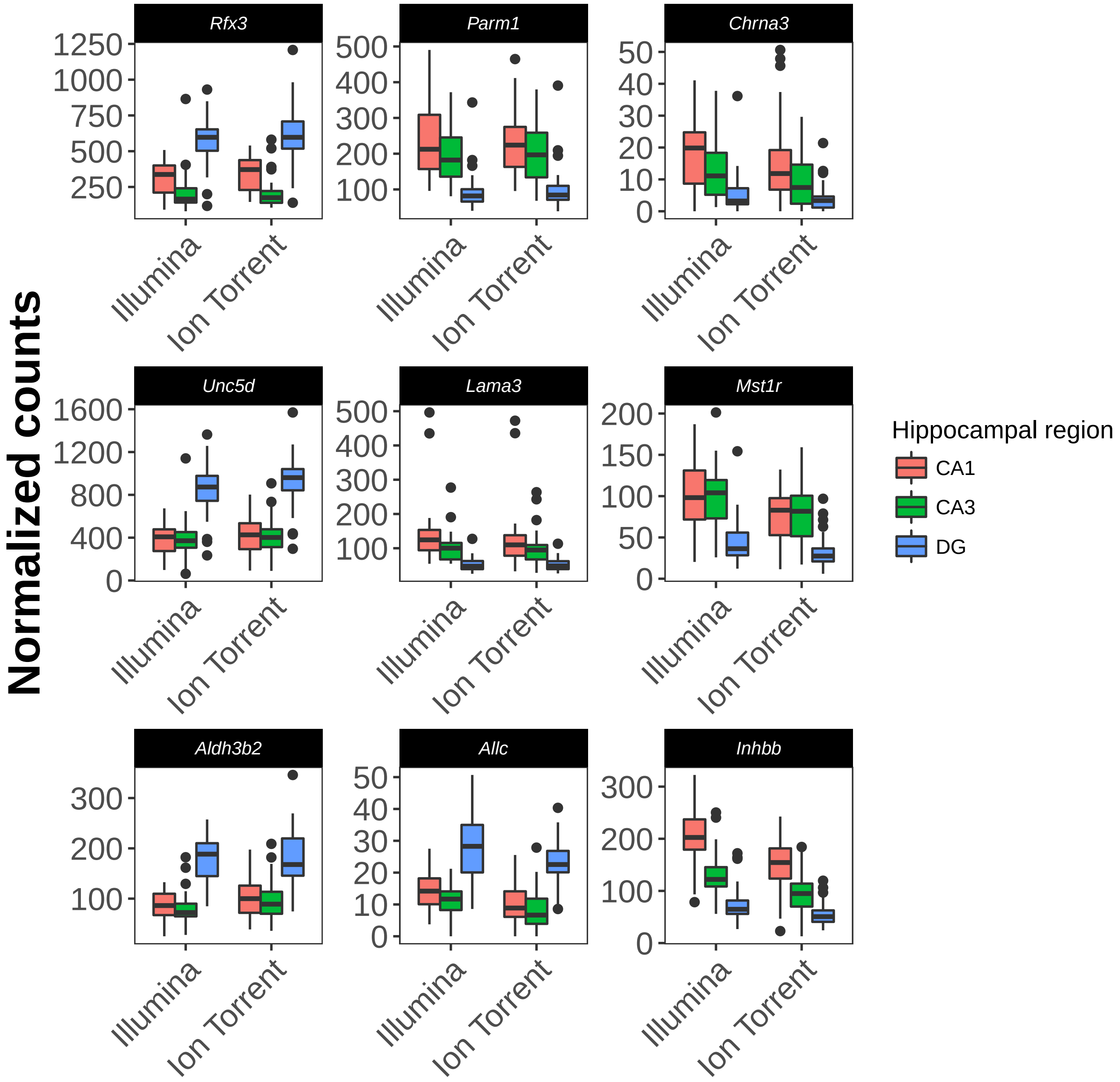
Normalized counts
Rfx3
Parm1
Chrna3
Unc5d
Lama3
Mst1r
Aldh3b2
Allc
Inhbb
Illumina
Ion Torrent
Hippocampal region
CA1
CA3
DG

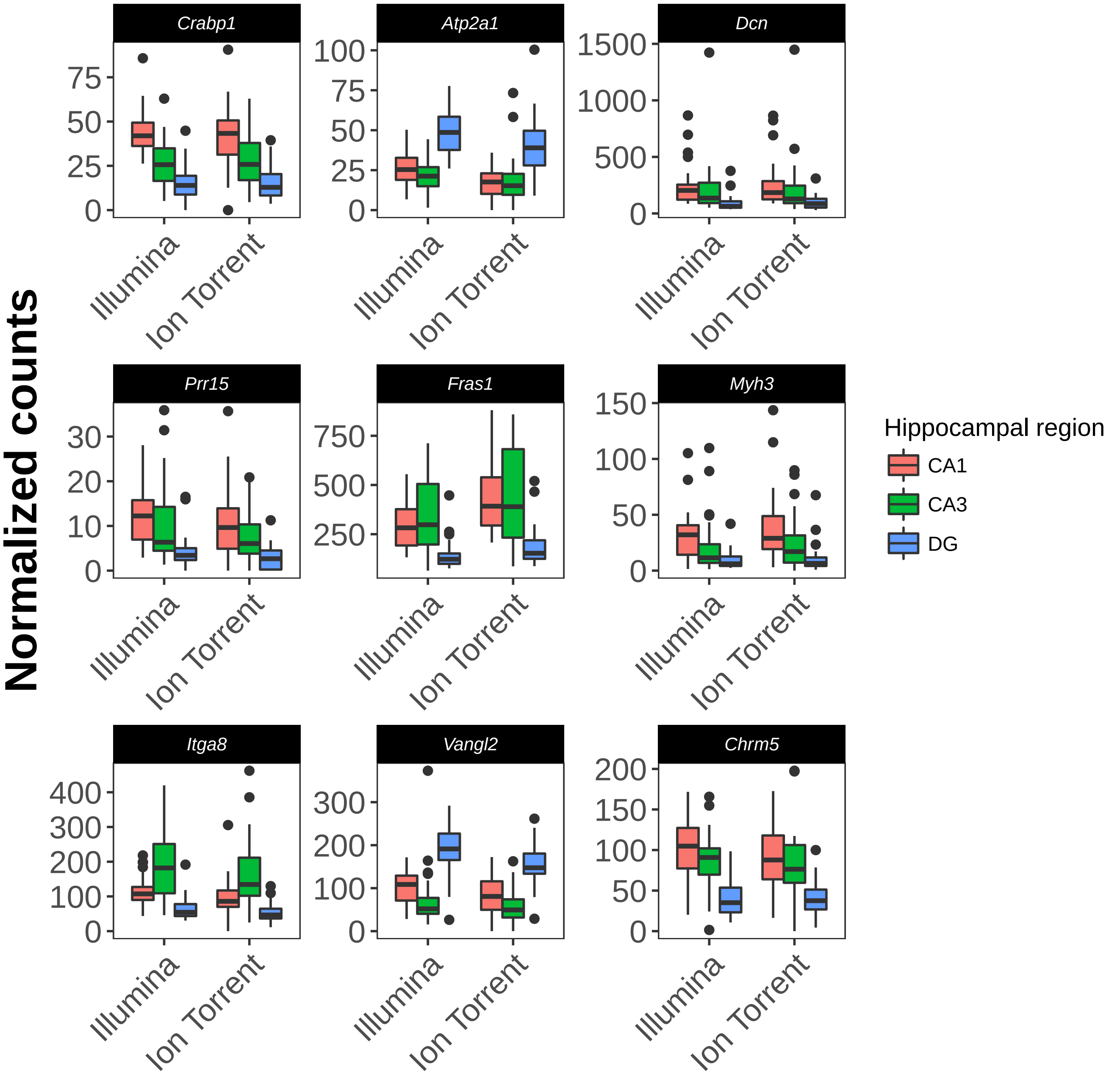
Normalized counts
Crabp1
Atp2a1
Dcn
Prr15
Fras1
Myh3
Itga8
Vangl2
Chrm5
Illumina
Ion Torrent
Hippocampal region
CA1
CA3
DG

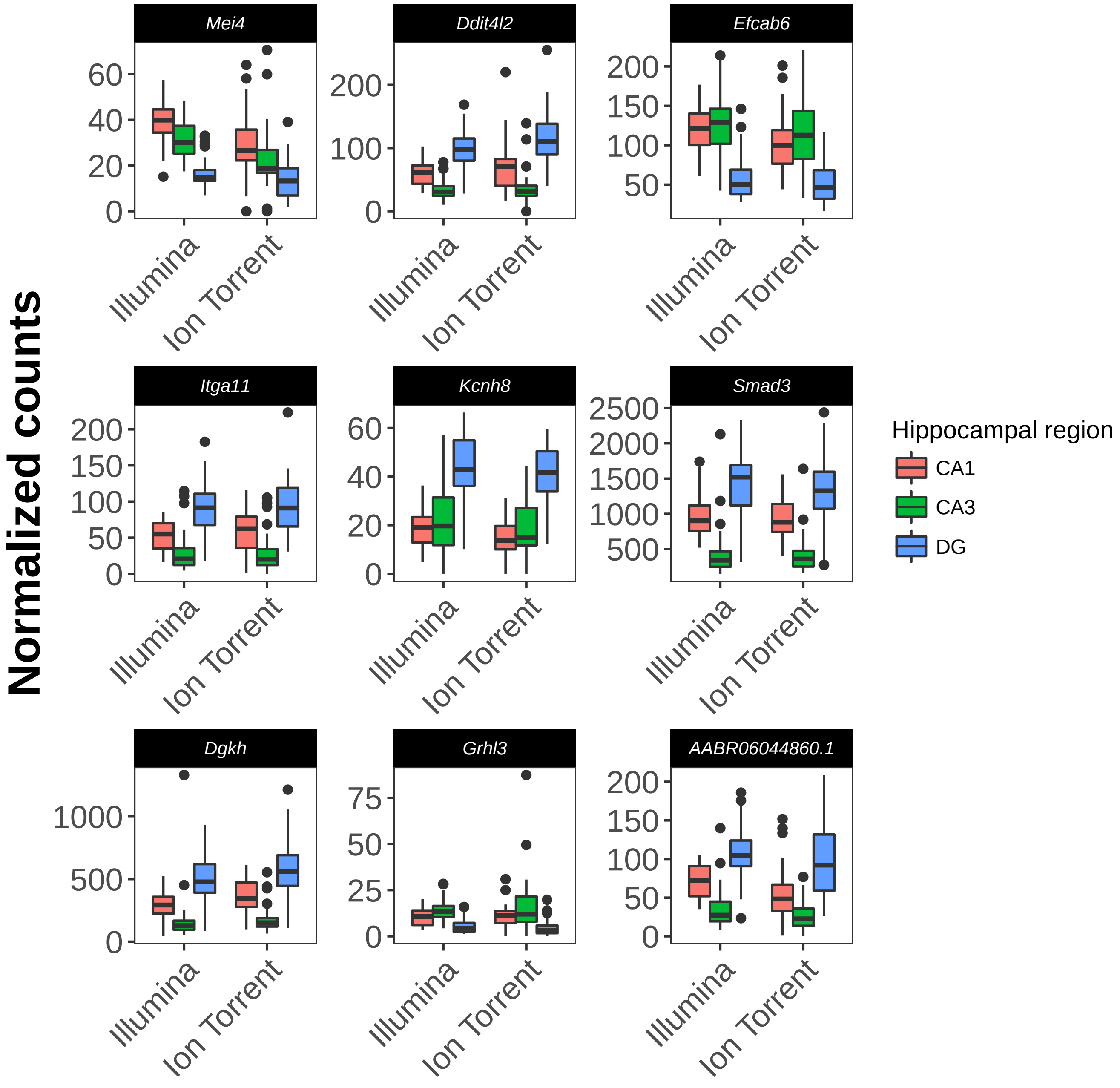

Normalized counts
Mei4
Ddit4l2
Efcab6
Itga11
Kcnh8
Smad3
Dgkh
Grhl3
AABR06044860.1
Illumina
Ion Torrent
Hippocampal region
CA1
CA3
DG

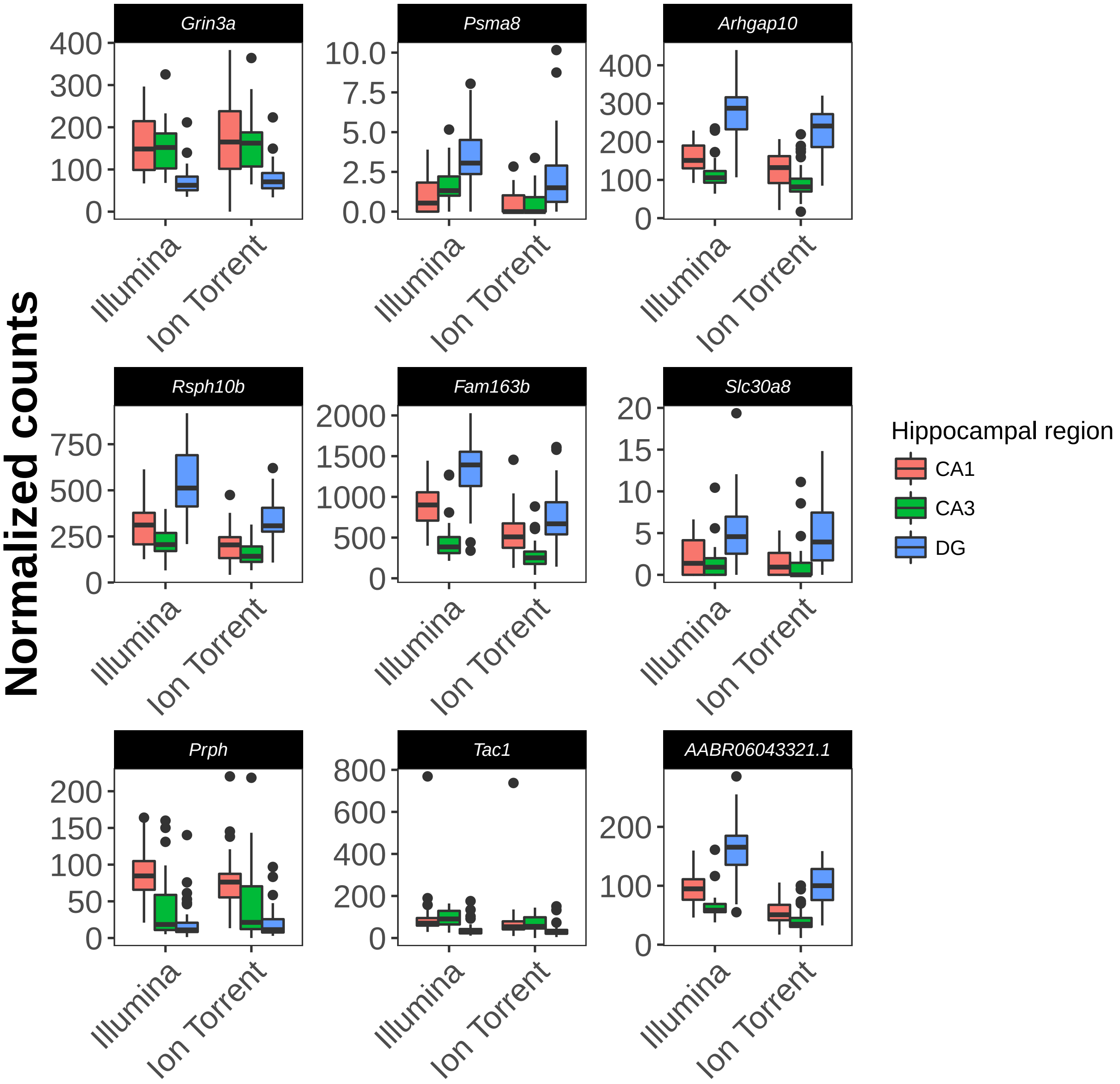
Grin3a
Psma8
Arhgap10
Rsph10b
Fam163b
Slc30a8
Prph
Tac1
AABR06043321.1
Normalized counts
Illumina
Ion Torrent
Hippocampal region
CA1
CA3
DG

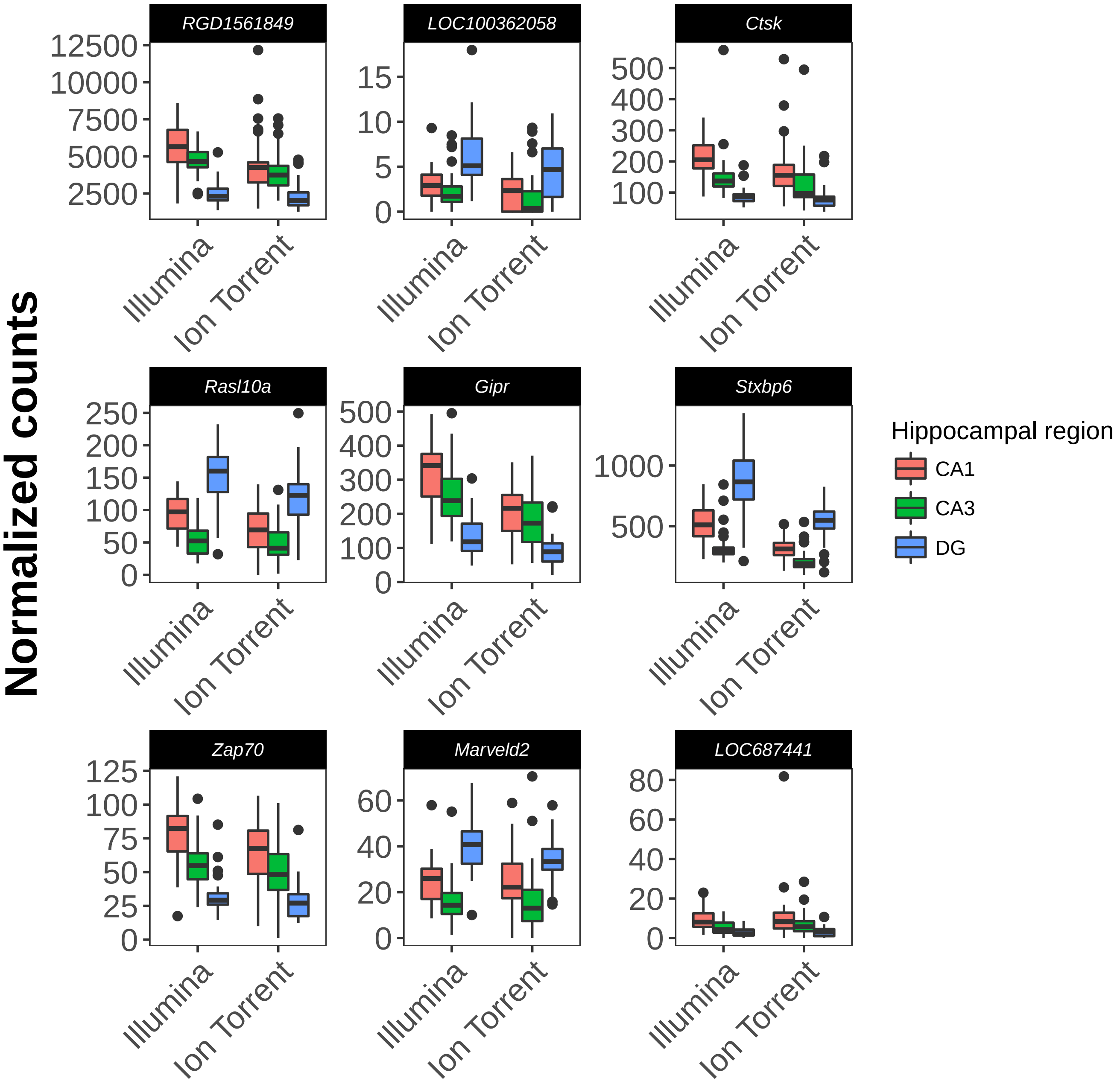
Normalized counts
RGD1561849
LOC100362058
Ctsk
Rasl10a
Gipr
Stxbp6
Zap70
Marveld2
LOC687441
Illumina
Ion Torrent
Hippocampal region
CA1
CA3
DG

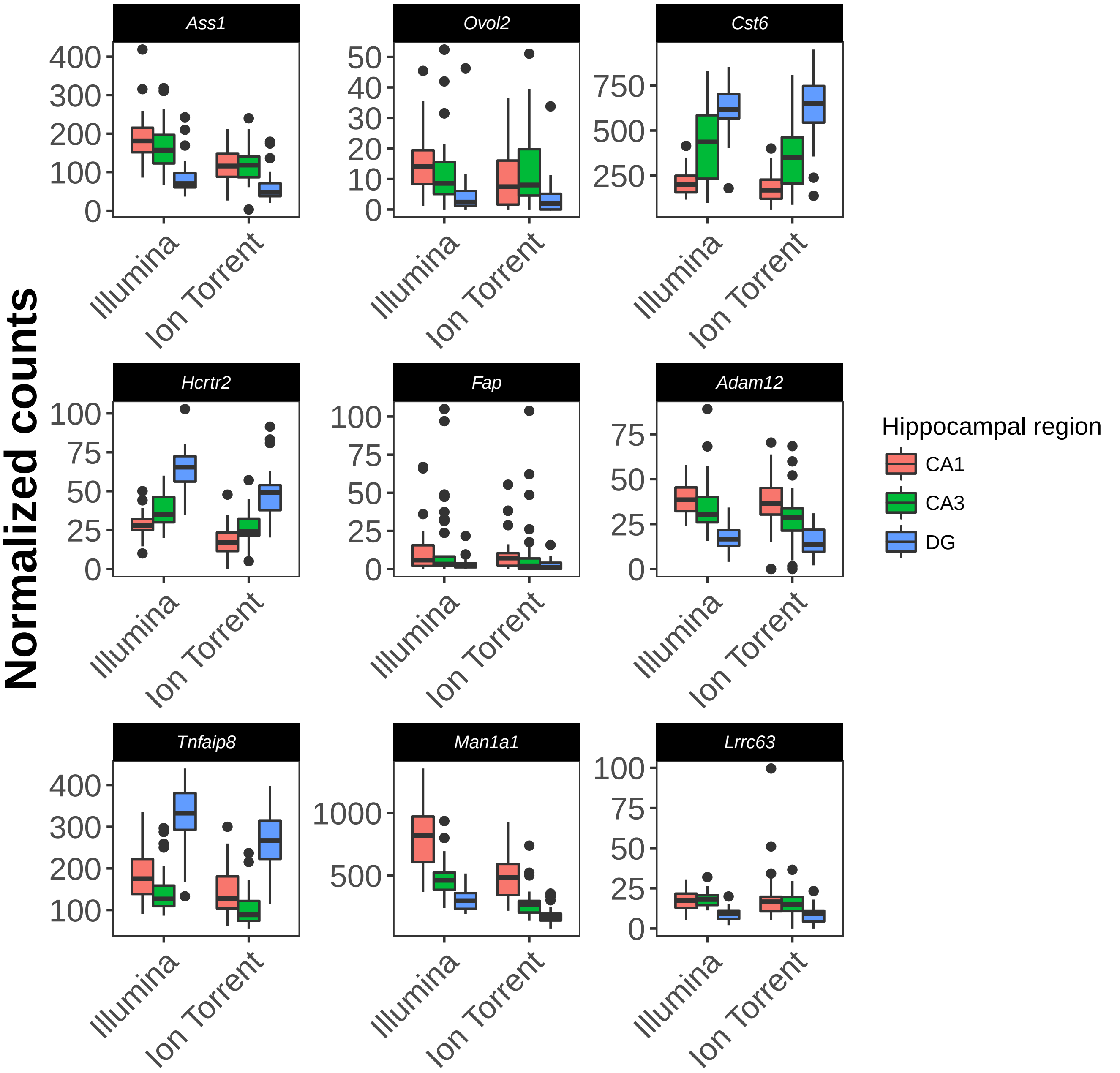

Normalized counts
Ass1
Ovol2
Cst6
Hcrtr2
Fap
Adam12
Tnfaip8
Man1a1
Lrrc63
Illumina
Ion Torrent
Hippocampal region
CA1
CA3
DG

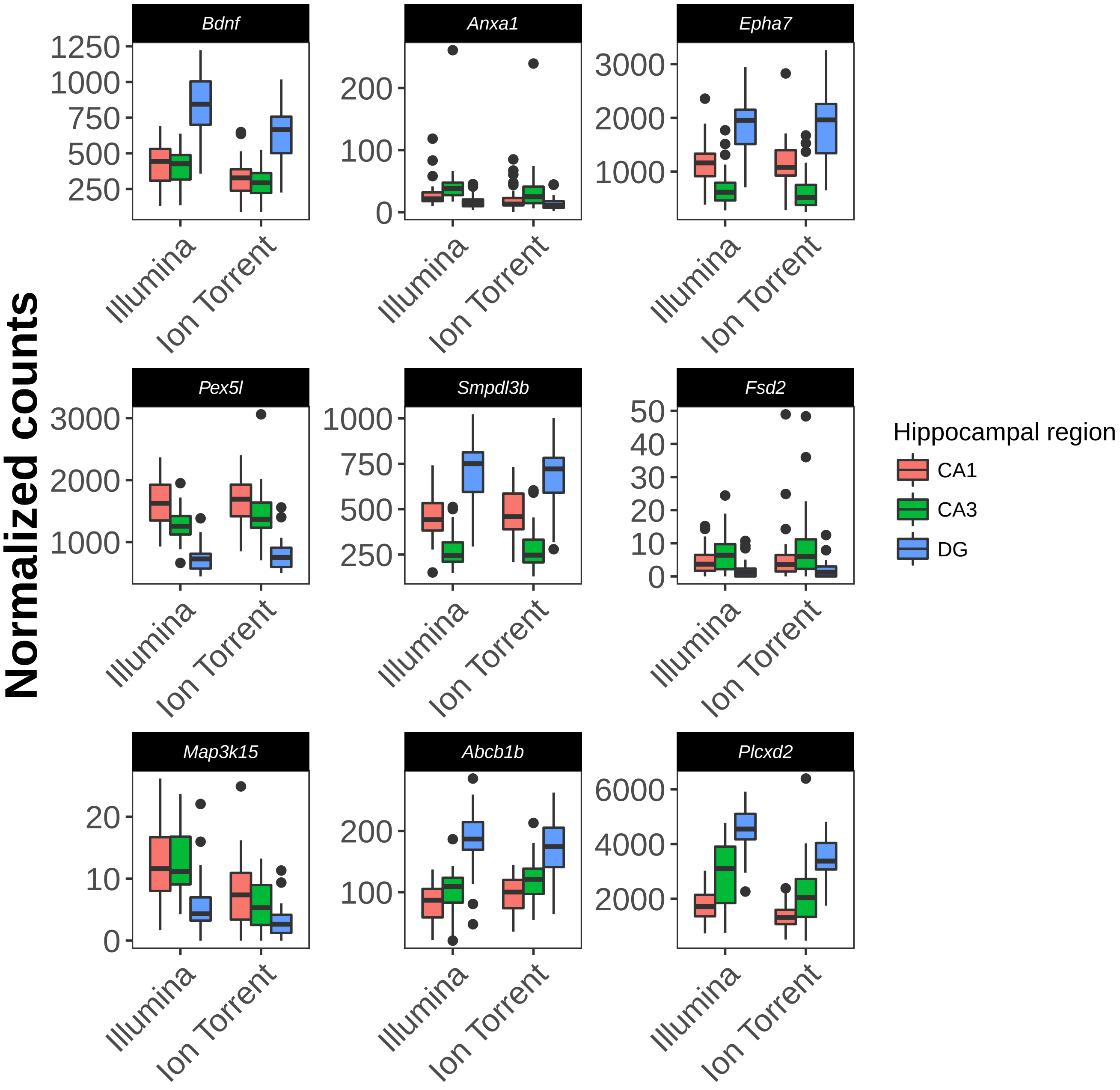
Normalized counts
Bdnf
Anxa1
Epha7
Pex5l
Smpdl3b
Fsd2
Map3k15
Abcb1b
Plcxd2
Illumina
Ion Torrent
Hippocampal region
CA1
CA3
DG

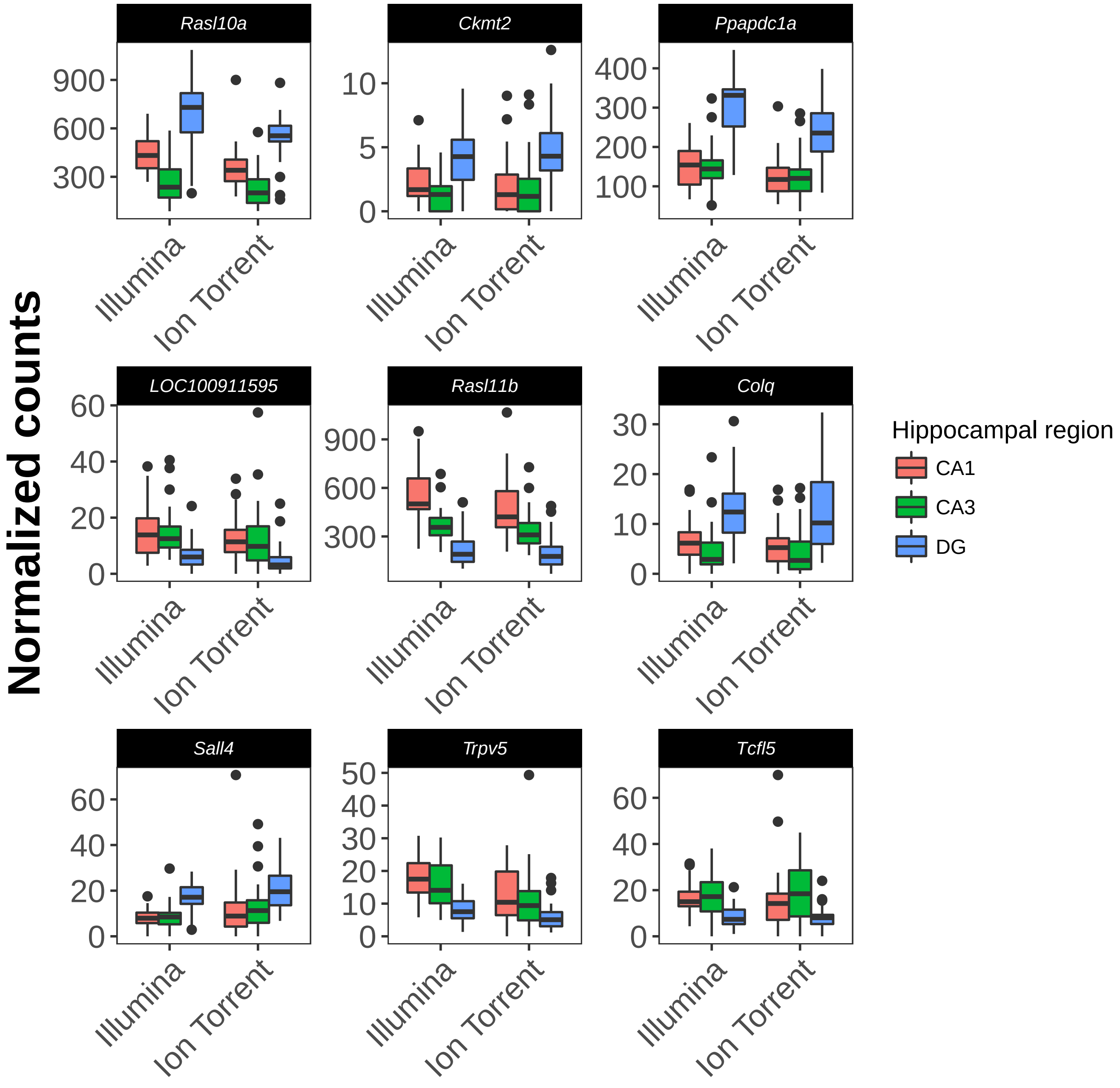
Normalized counts
Rasl10a
Ckmt2
Ppapdc1a
LOC100911595
Rasl11b
Colq
Sall4
Trpv5
Tcfl5
Illumina
Ion Torrent
Hippocampal region
CA1
CA3
DG

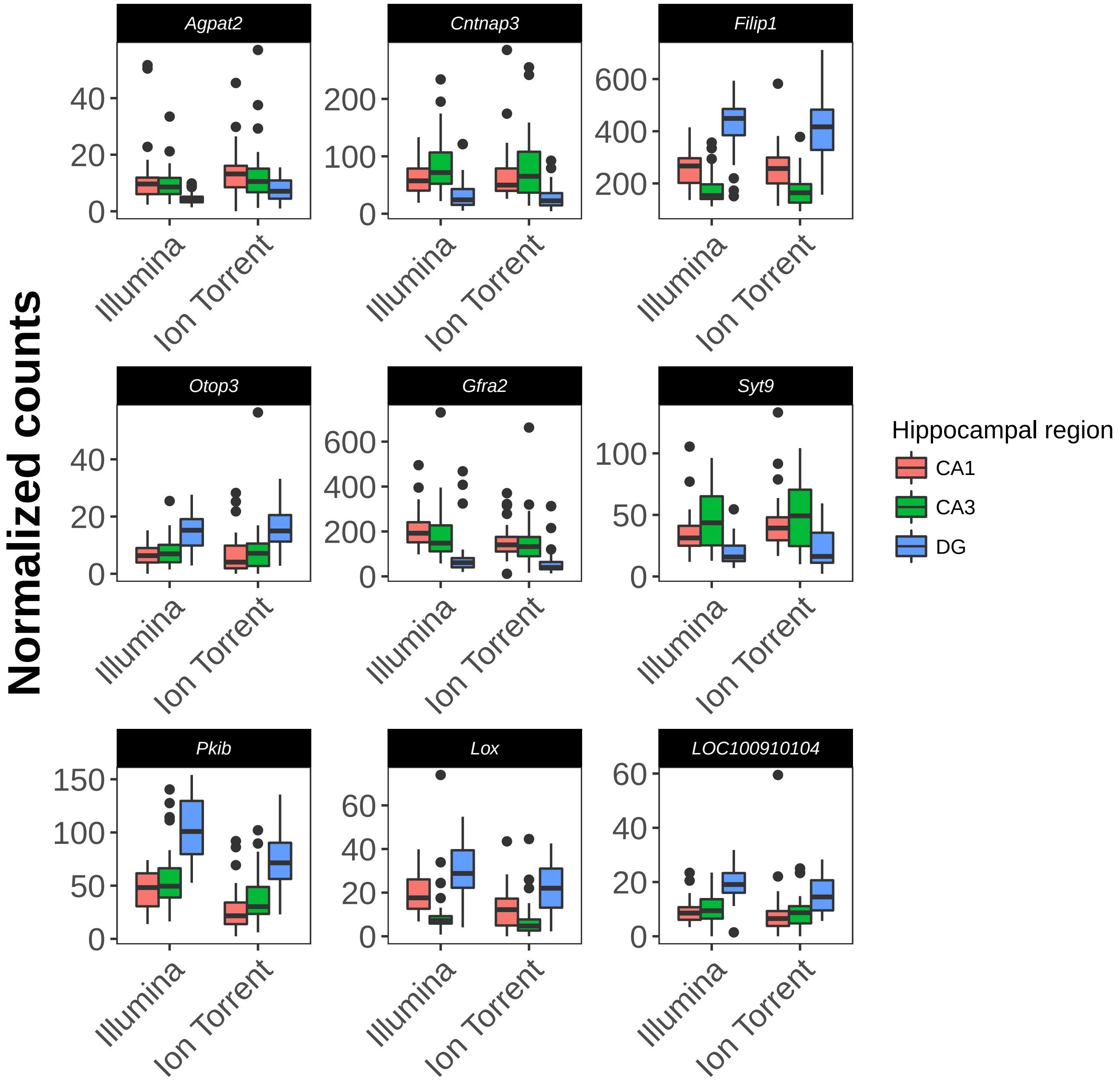

Normalized counts
Agpat2
Cntnap3
Filip1
Otop3
Gfra2
Syt9
Pkib
Lox
LOC100910104
Illumina
Ion Torrent
Hippocampal region
CA1
CA3
DG

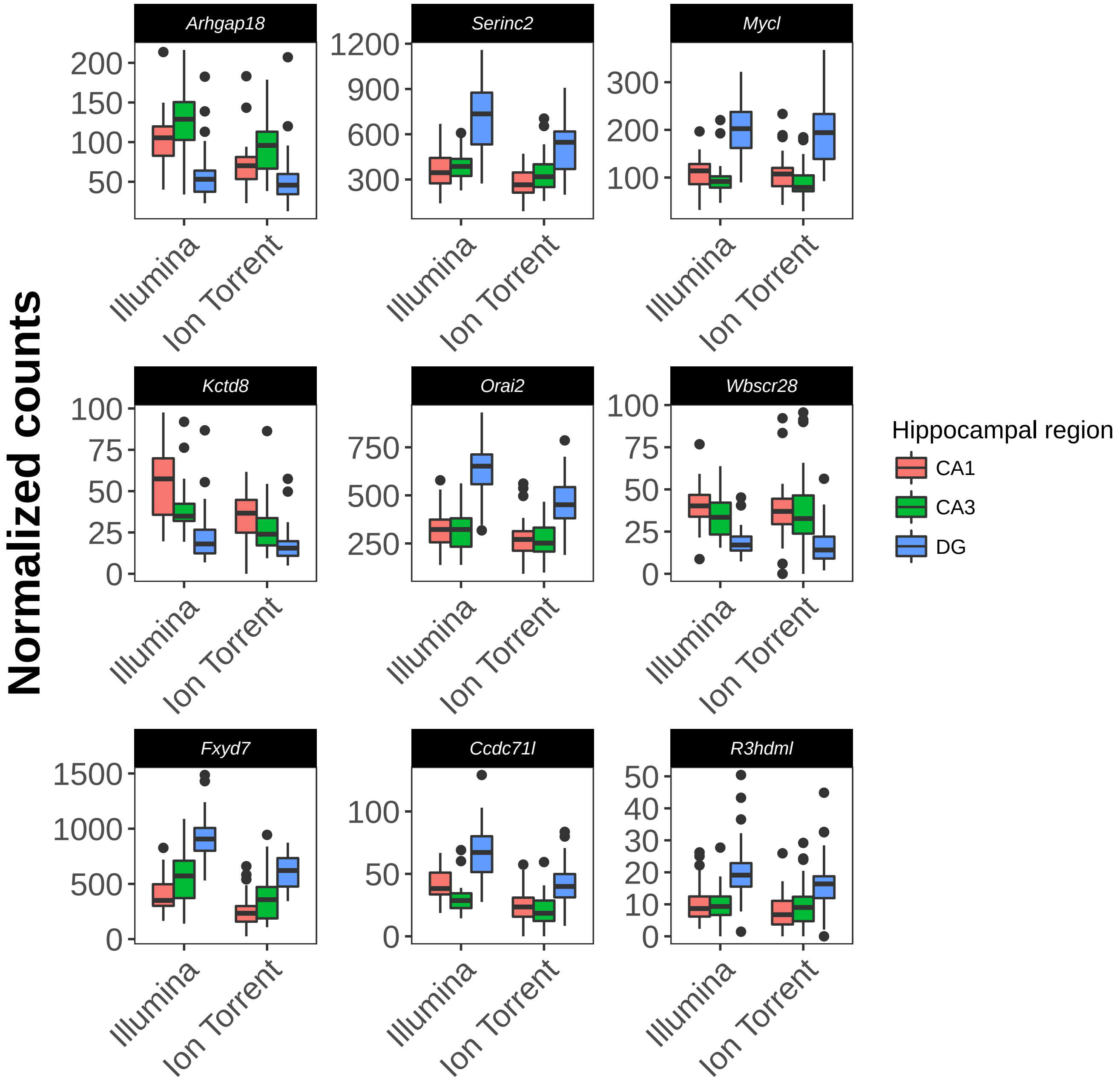
Normalized counts
Arhgap18
Serinc2
Mycl
Kctd8
Orai2
Wbscr28
Fxyd7
Ccdc71l
R3hdml
Illumina
Ion Torrent
Hippocampal region
CA1
CA3
DG

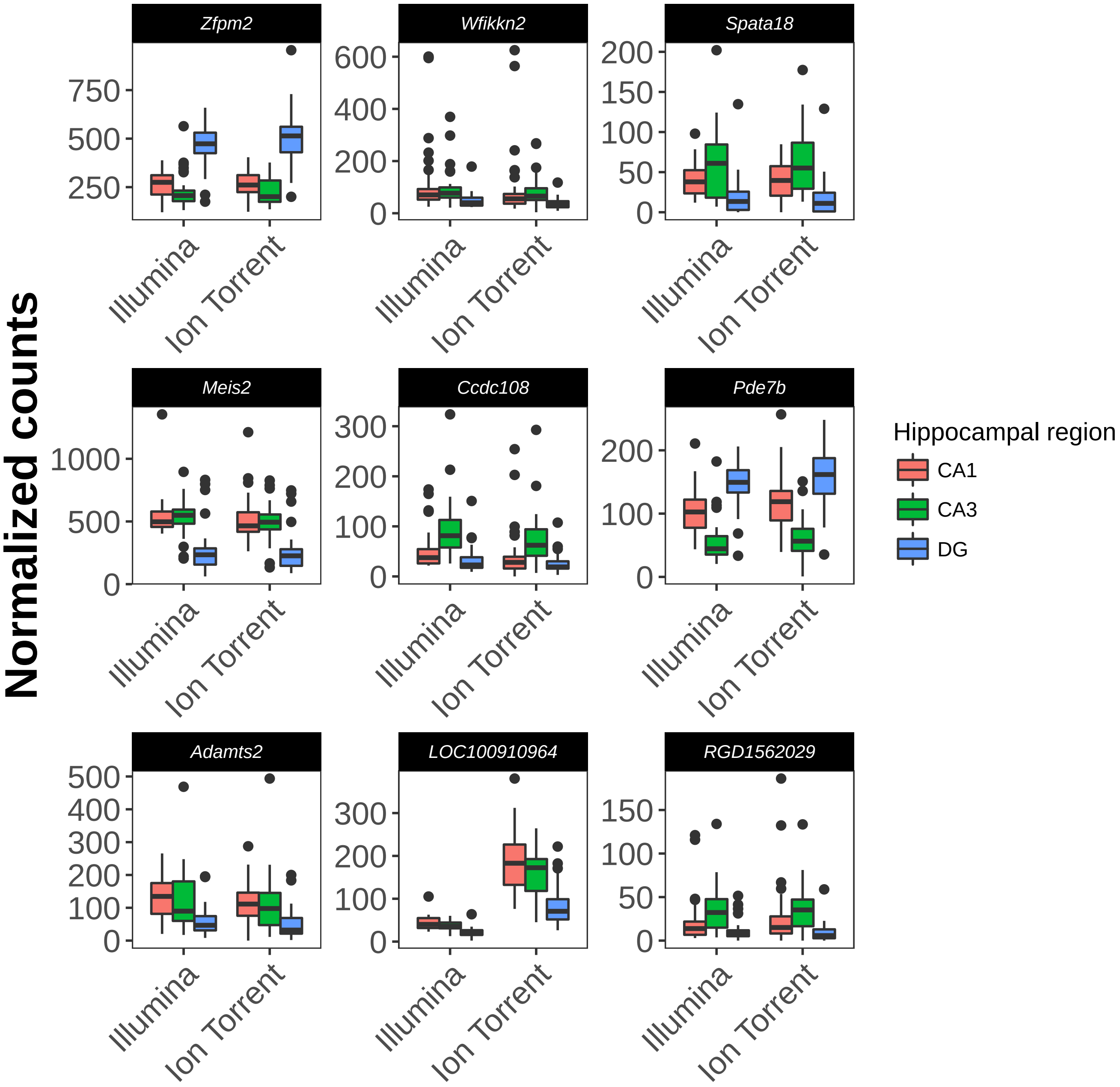
Normalized counts
Zfpm2
Wfikkn2
Spata18
Meis2
Ccdc108
Pde7b
Adamts2
LOC100910964
RGD1562029
Illumina
Ion Torrent
Hippocampal region
CA1
CA3
DG

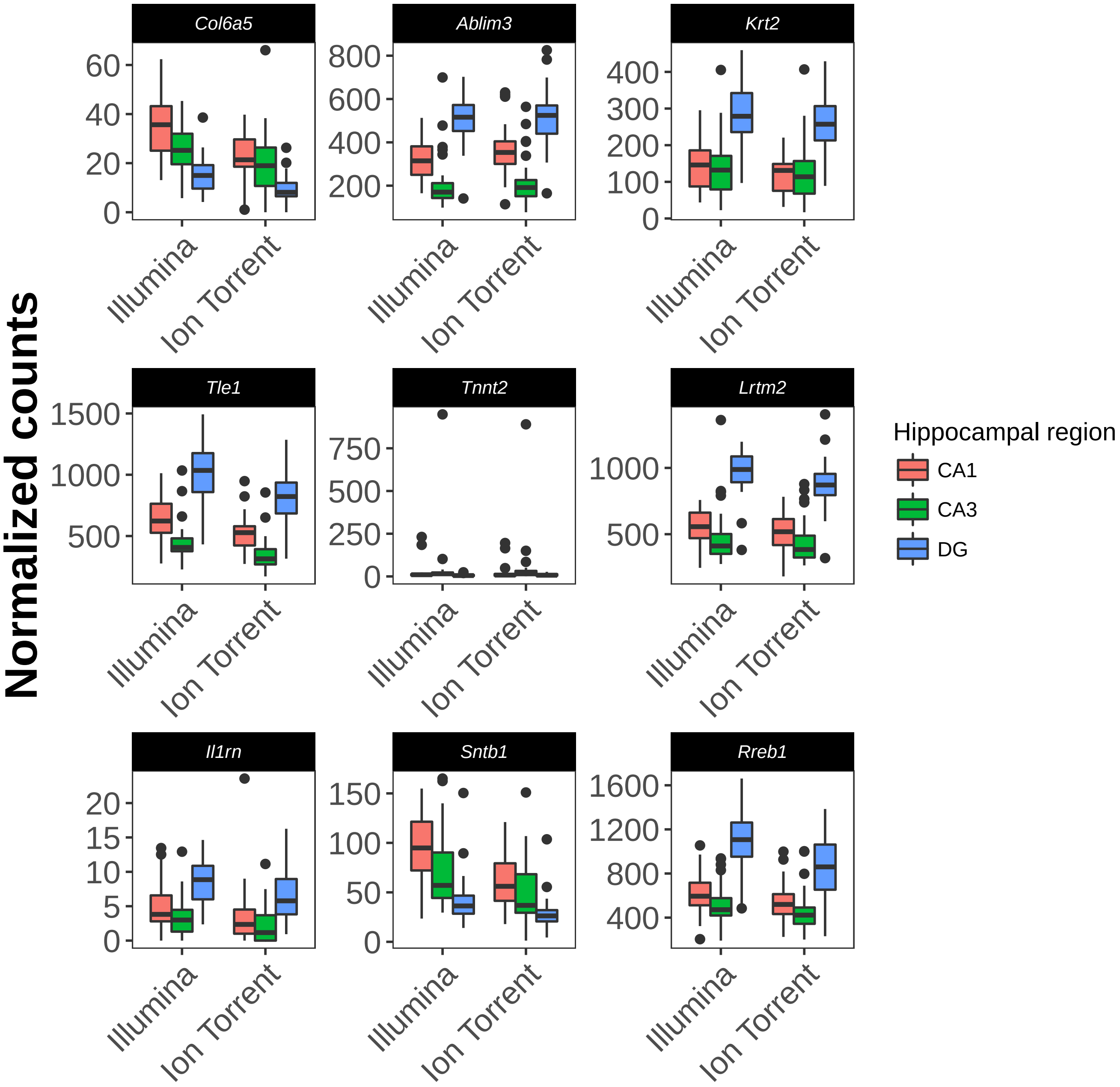
Normalized counts
Col6a5
Ablim3
Krt2
Tle1
Tnnt2
Lrtm2
Il1rn
Sntb1
Rreb1
Illumina
Ion Torrent
Hippocampal region
CA1
CA3
DG

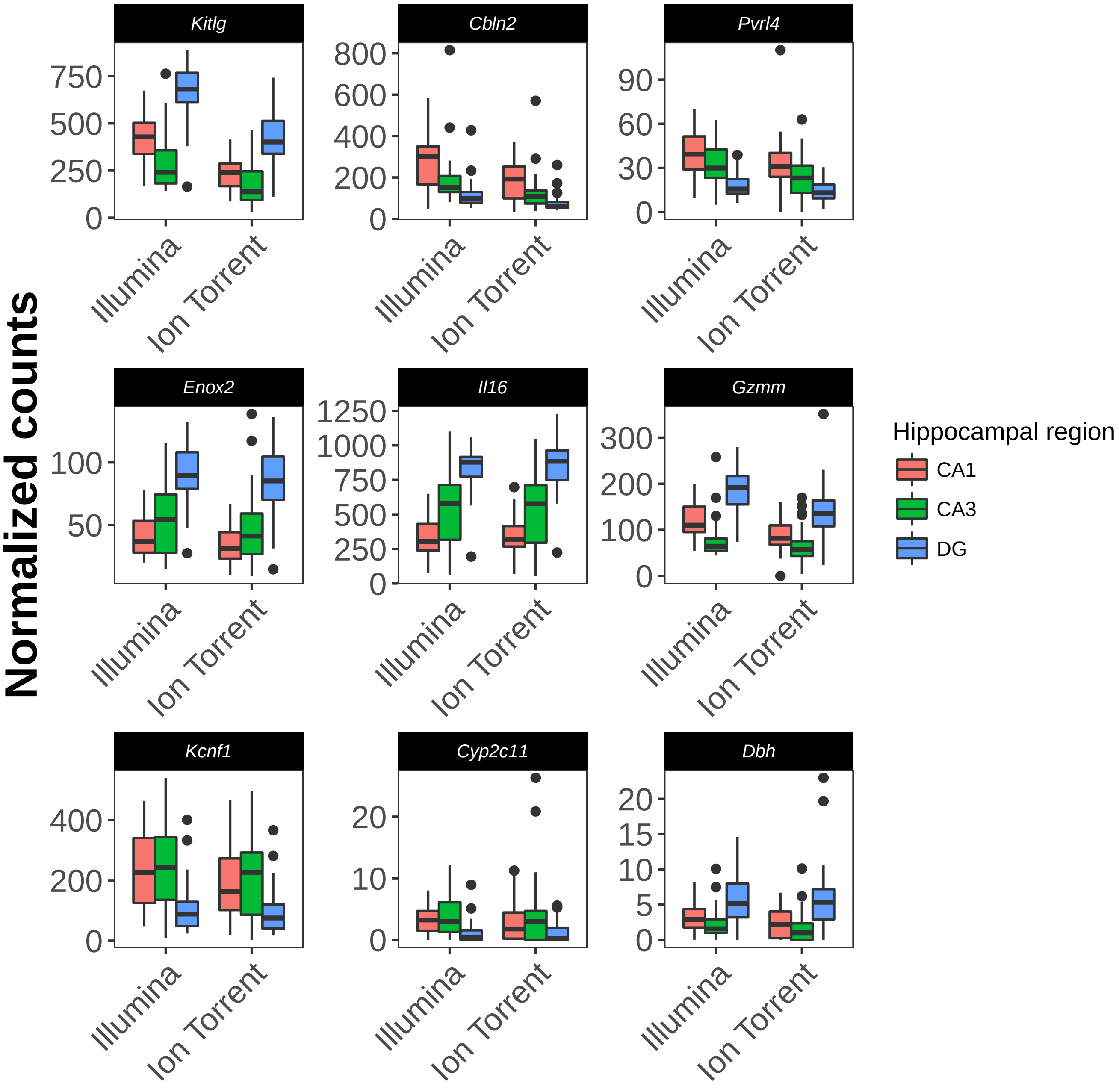
Normalized counts
Kitlg
Cbln2
Pvrl4
Enox2
Il16
Gzmm
Kcnf1
Cyp2c11
Dbh
Illumina
Ion Torrent
Hippocampal region
CA1
CA3
DG

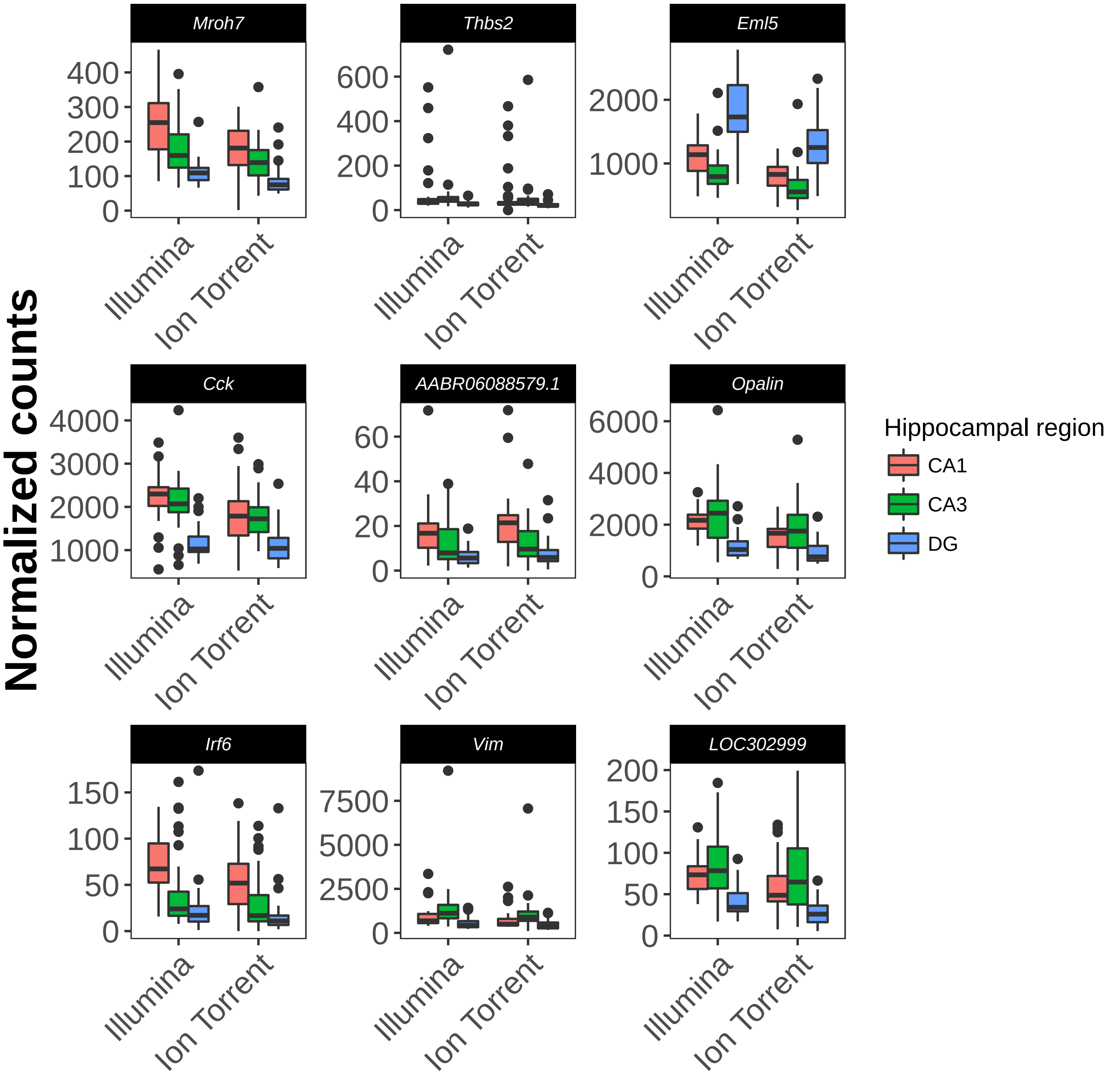

Normalized counts
Mroh7
Thbs2
Eml5
Cck
AABR06088579.1
Opalin
Irf6
Vim
LOC302999
Illumina
Ion Torrent
Hippocampal region
CA1
CA3
DG

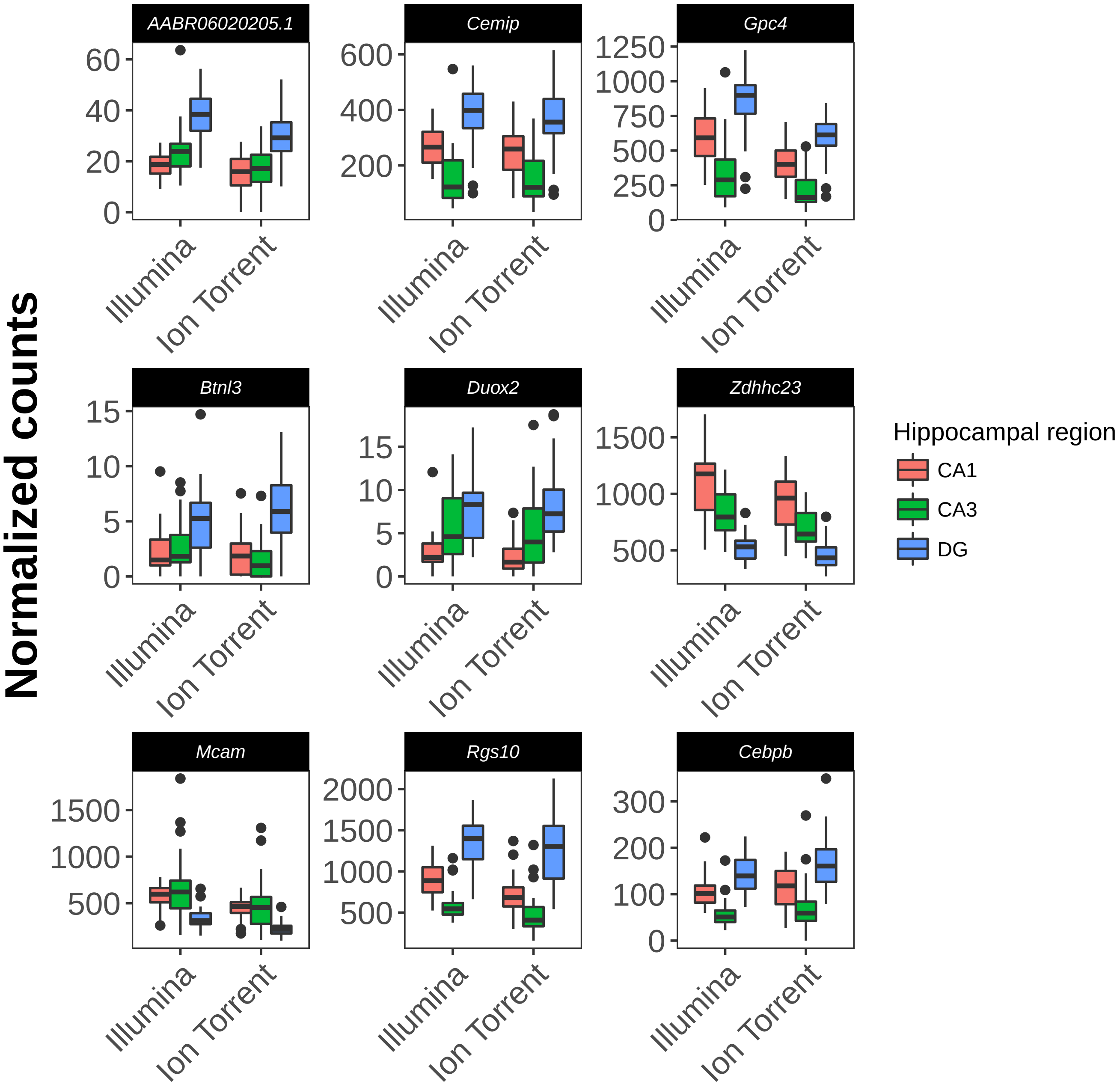
Normalized counts
AABR06020205.1
Cemip
Gpc4
Btnl3
Duox2
Zdhhc23
Mcam
Rgs10
Cebpb
Illumina
Ion Torrent
Hippocampal region
CA1
CA3
DG

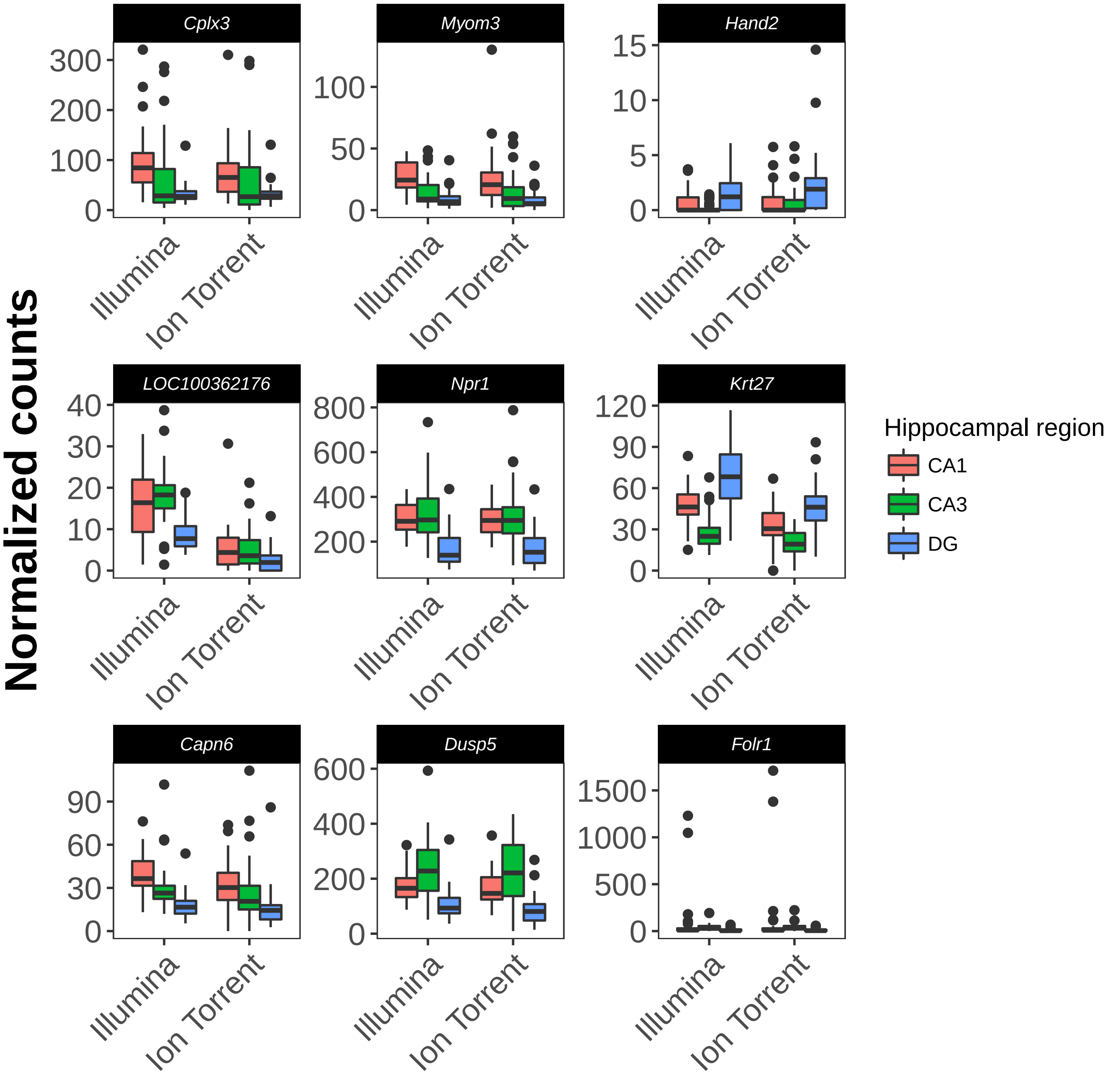

Normalized counts
Cplx3
Myom3
Hand2
LOC100362176
Npr1
Krt27
Capn6
Dusp5
Folr1
Illumina
Ion Torrent
Hippocampal region
CA1
CA3
DG

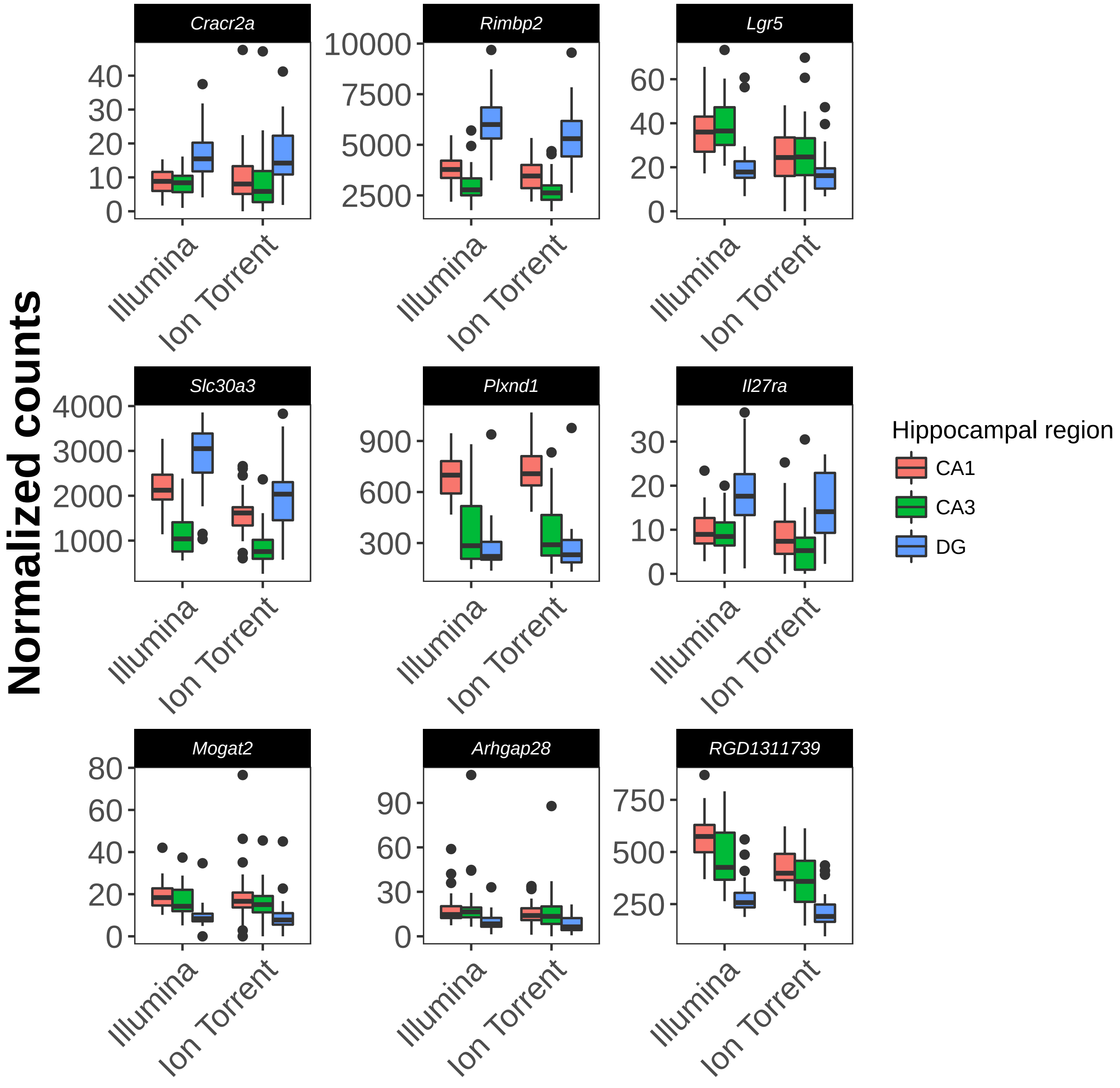

Normalized counts
Cracr2a
Rimbp2
Lgr5
Slc30a3
Plxnd1
Il27ra
Mogat2
Arhgap28
RGD1311739
Illumina
Ion Torrent
Hippocampal region
CA1
CA3
DG

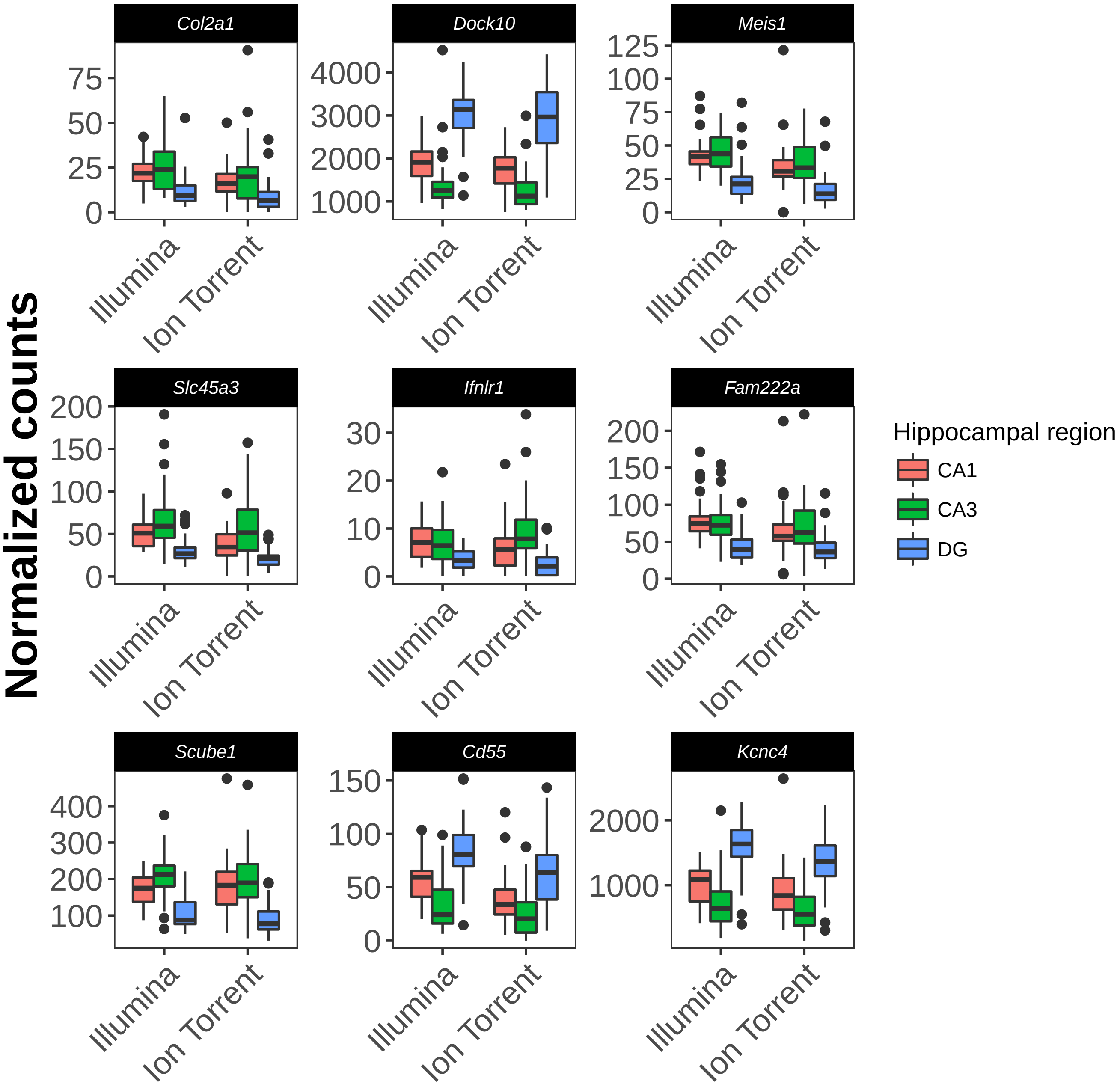
Normalized counts
Col2a1
Dock10
Meis1
Slc45a3
Ifnlr1
Fam222a
Scube1
Cd55
Kcnc4
Illumina
Ion Torrent
Hippocampal region
CA1
CA3
DG

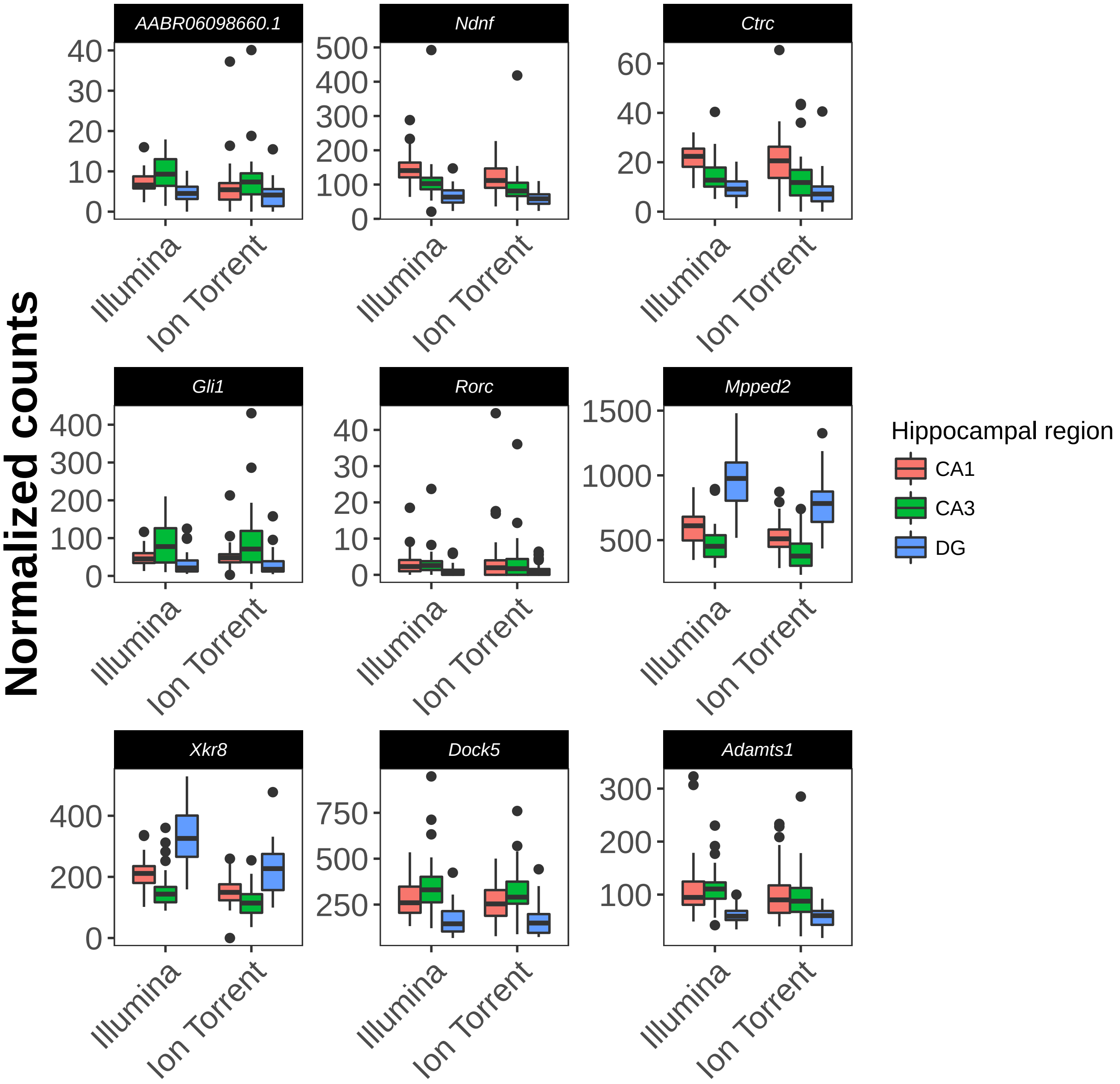

Normalized counts
AABR06098660.1
Ndnf
Ctrc
Gli1
Rorc
Mpped2
Xkr8
Dock5
Adamts1
Illumina
Ion Torrent
Hippocampal region
CA1
CA3
DG

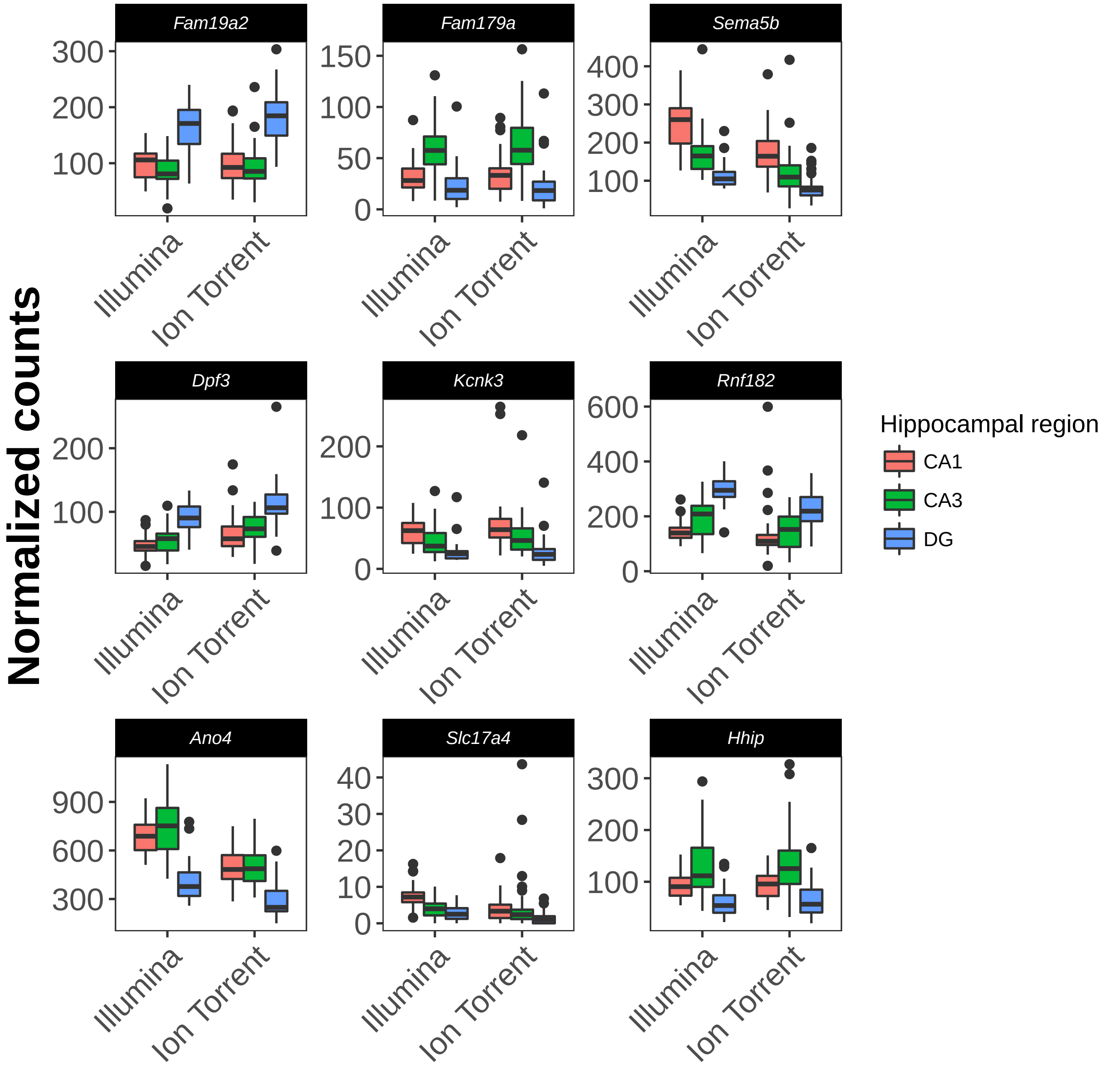
Normalized counts
Fam19a2
Fam179a
Sema5b
Dpf3
Kcnk3
Rnf182
Ano4
Slc17a4
Hhip
Illumina
Ion Torrent
Hippocampal region
CA1
CA3
DG

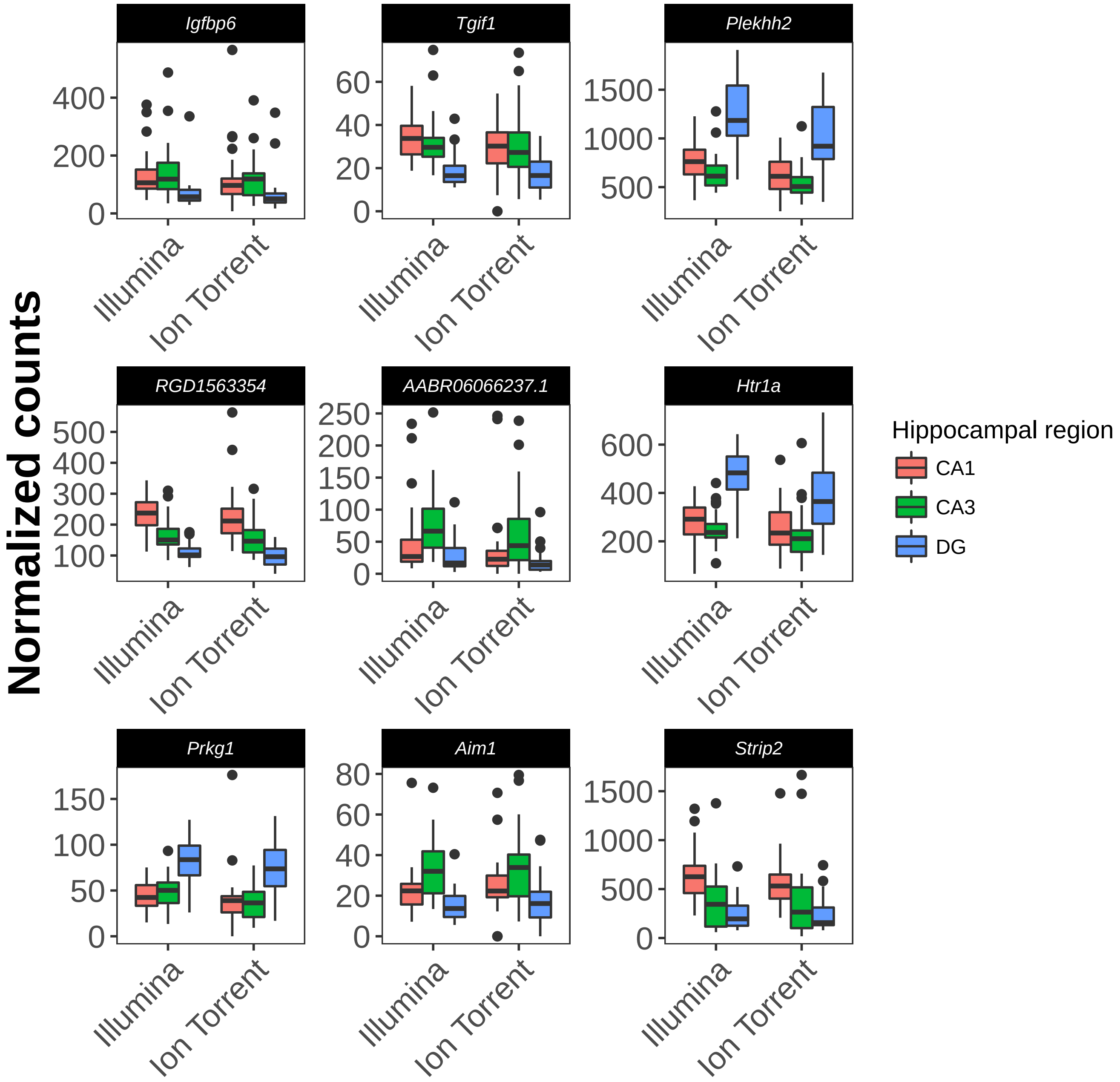
Normalized counts
Igfbp6
Tgif1
Plekhh2
RGD1563354
AABR06066237.1
Htr1a
Prkg1
Aim1
Strip2
Illumina
Ion Torrent
Hippocampal region
CA1
CA3
DG

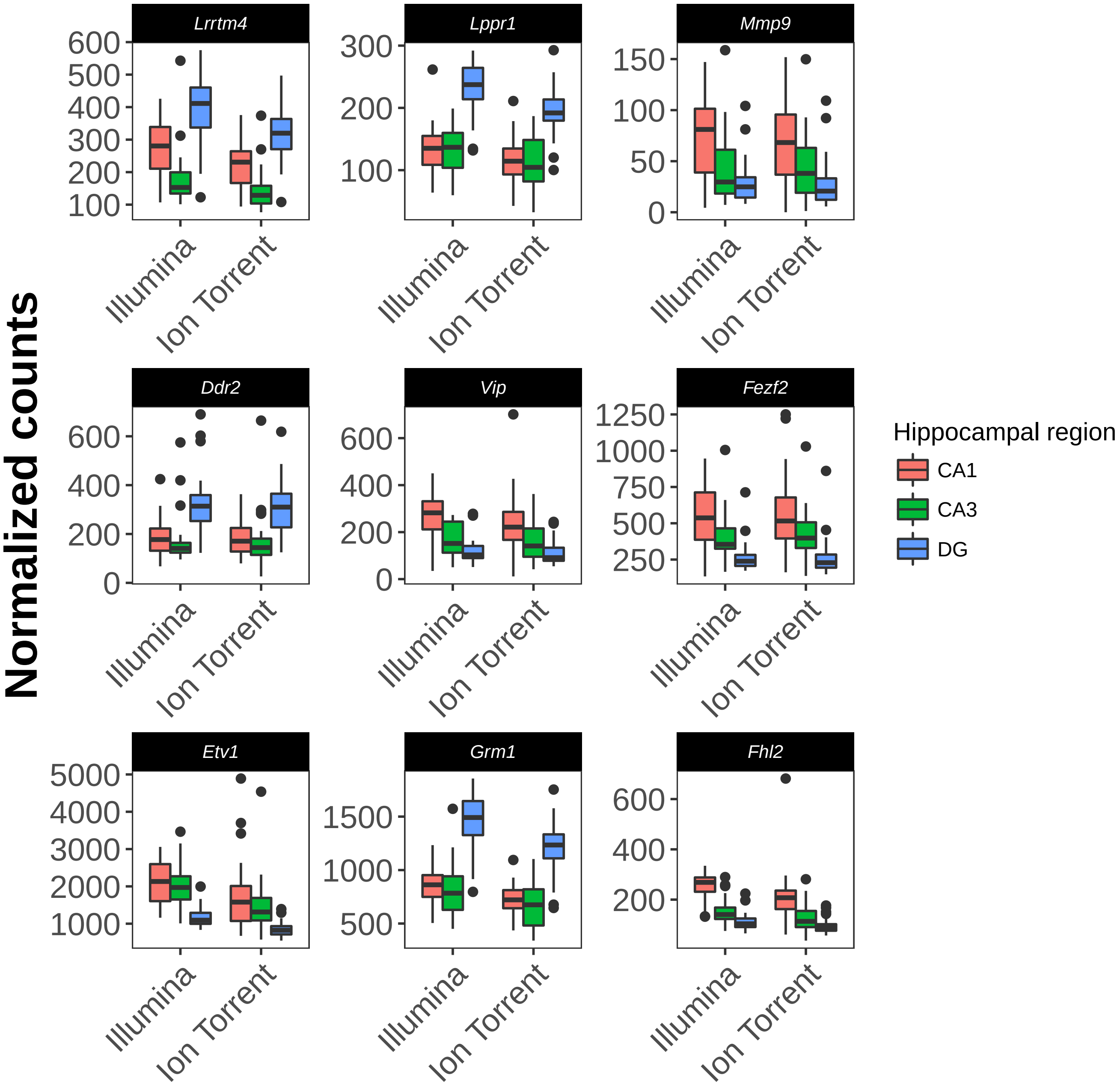

Normalized counts
Lrrtm4
Lppr1
Mmp9
Ddr2
Vip
Fezf2
Etv1
Grm1
Fhl2
Illumina
Ion Torrent
Hippocampal region
CA1
CA3
DG

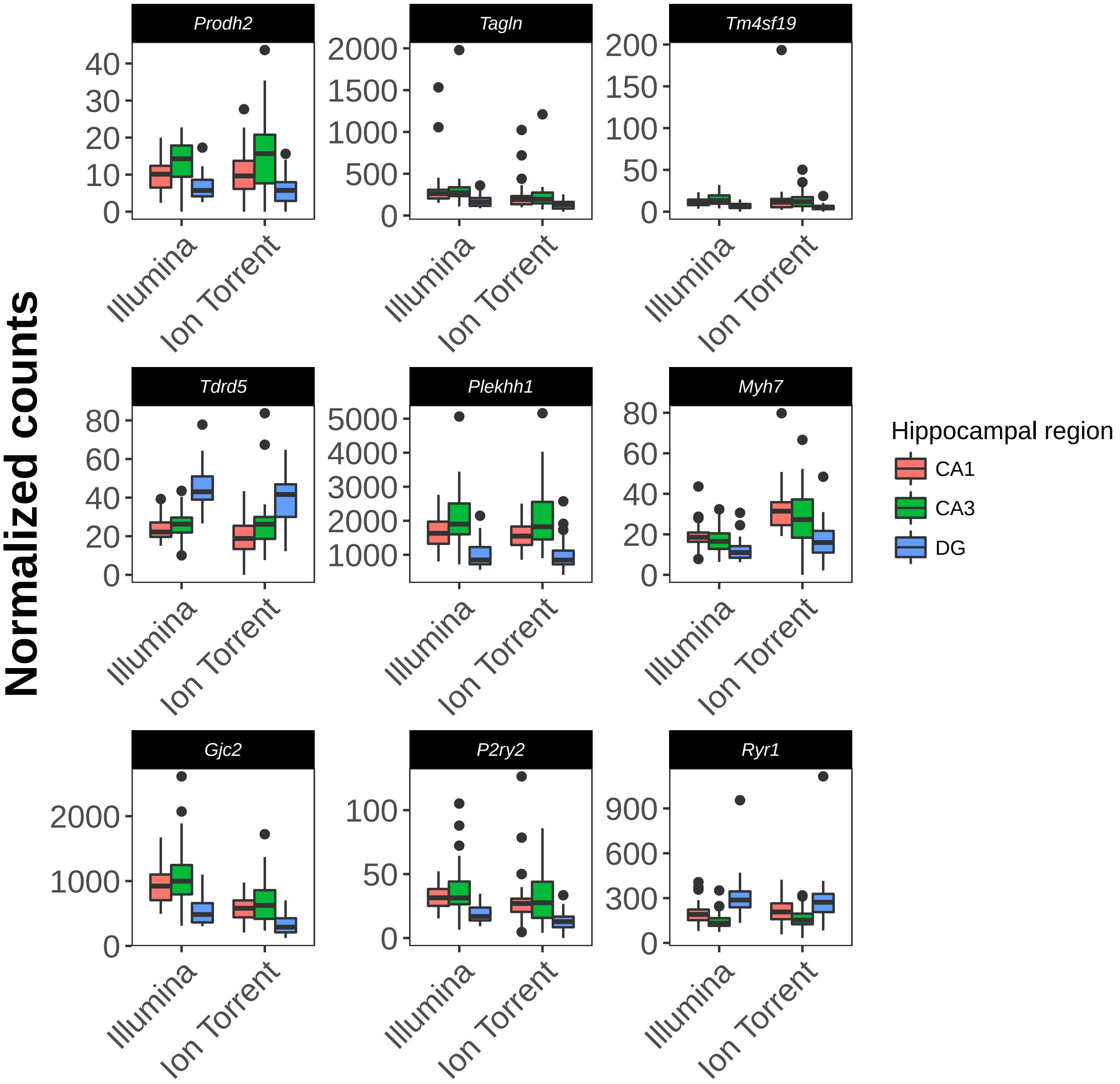

Normalized counts
Prodh2
Tagln
Tm4sf19
Tdrd5
Plekhh1
Myh7
Gjc2
P2ry2
Ryr1
Illumina
Ion Torrent
Hippocampal region
CA1
CA3
DG

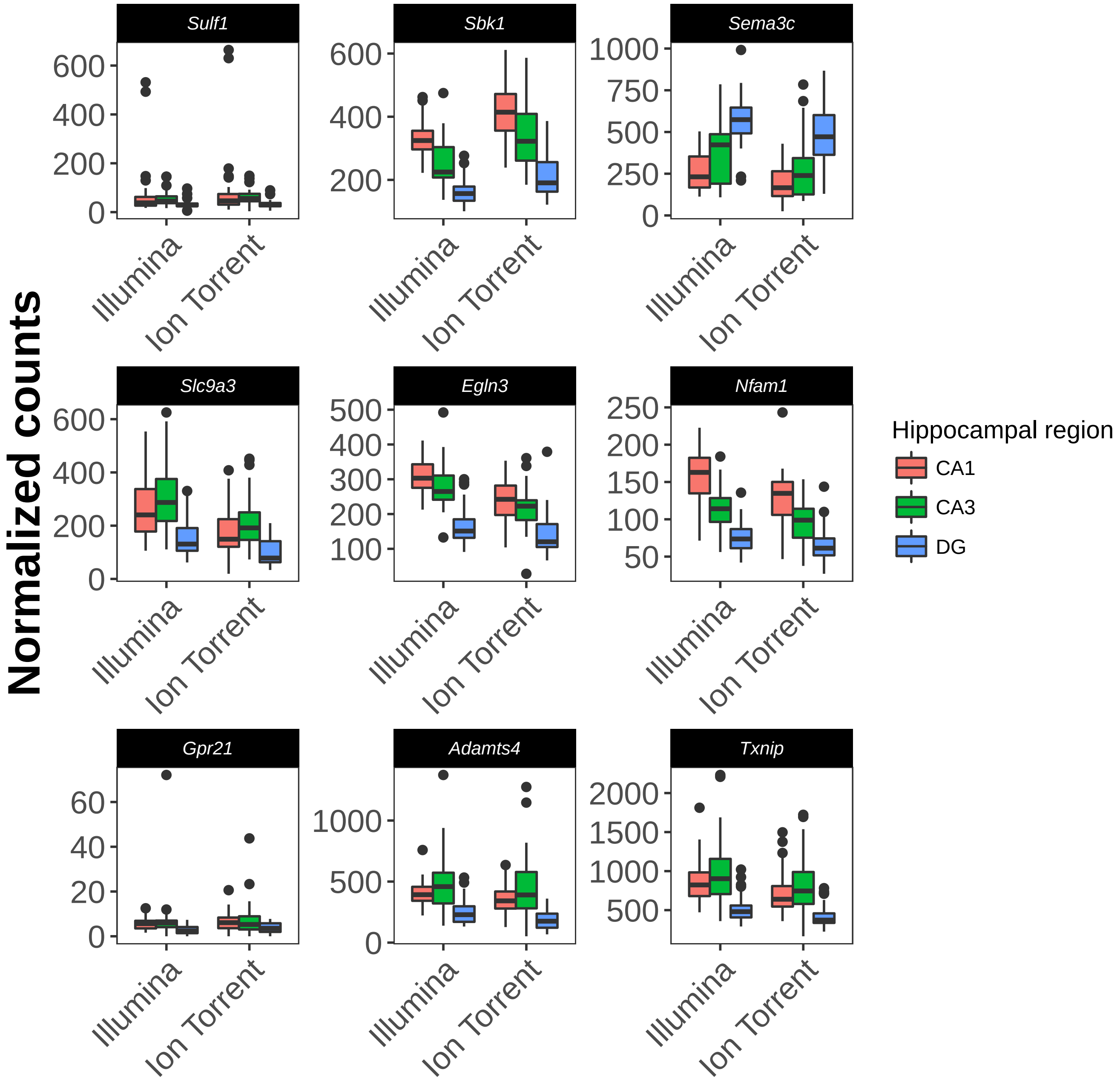
Normalized counts
Sulf1
Sbk1
Sema3c
Slc9a3
Egln3
Nfam1
Gpr21
Adamts4
Txnip
Illumina
Ion Torrent
Hippocampal region
CA1
CA3
DG

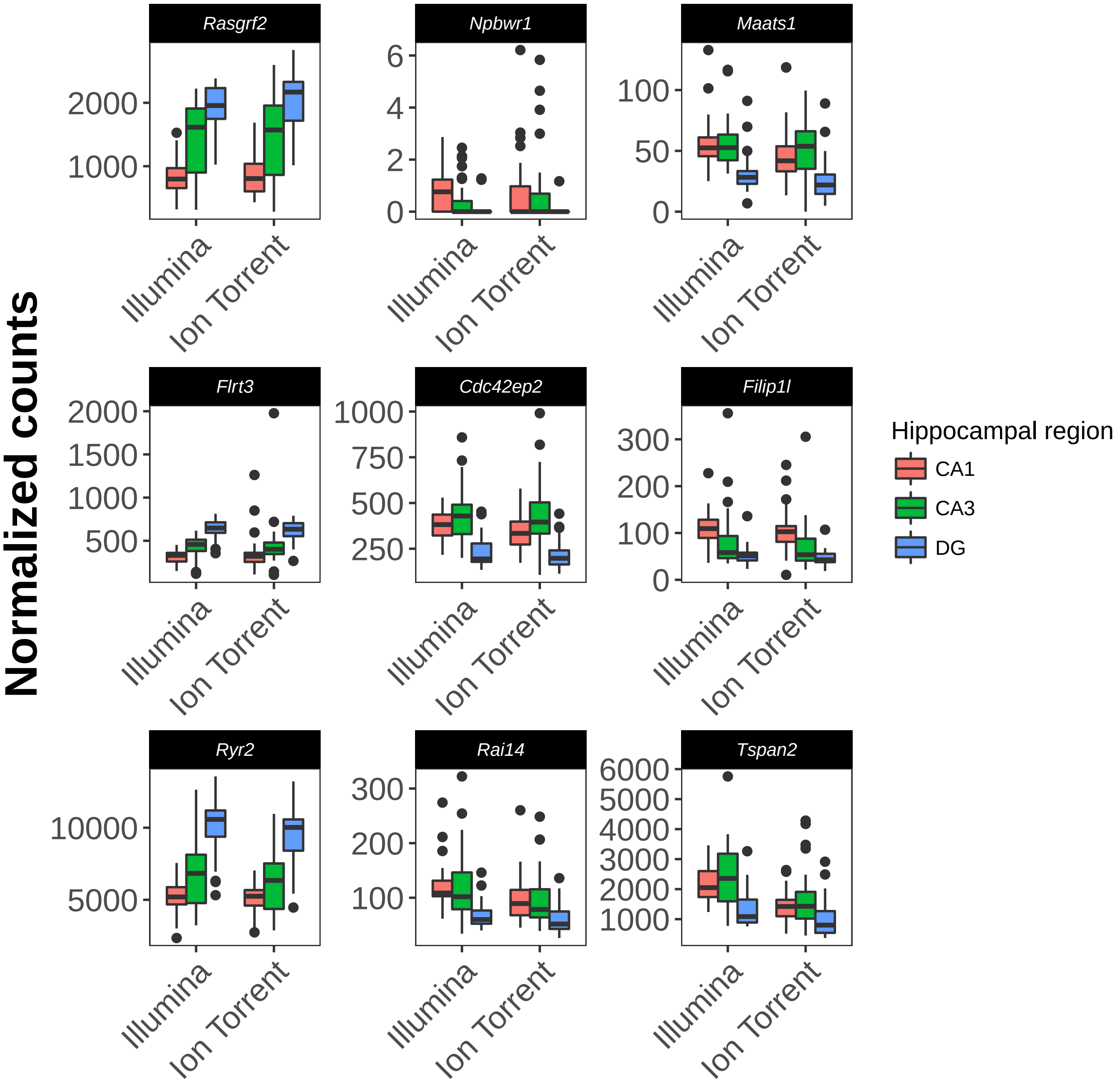
Normalized counts
Rasgrf2
Npbwr1
Maats1
Flrt3
Cdc42ep2
Filip1l
Ryr2
Rai14
Tspan2
Illumina
Ion Torrent
Hippocampal region
CA1
CA3
DG

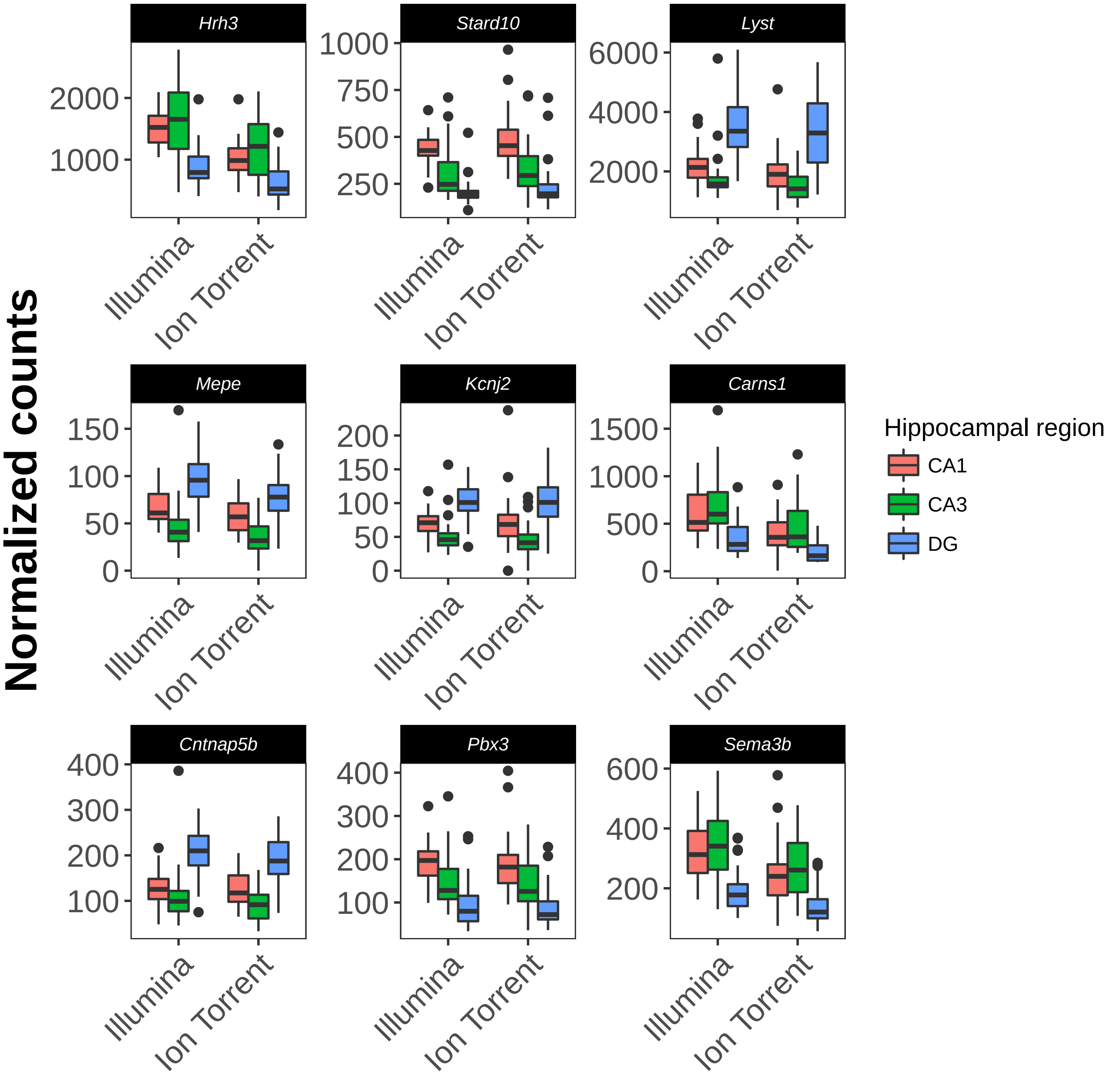

Normalized counts
Hrh3
Stard10
Lyst
Mepe
Kcnj2
Carns1
Cntnap5b
Pbx3
Sema3b
Illumina
Ion Torrent
Hippocampal region
CA1
CA3
DG

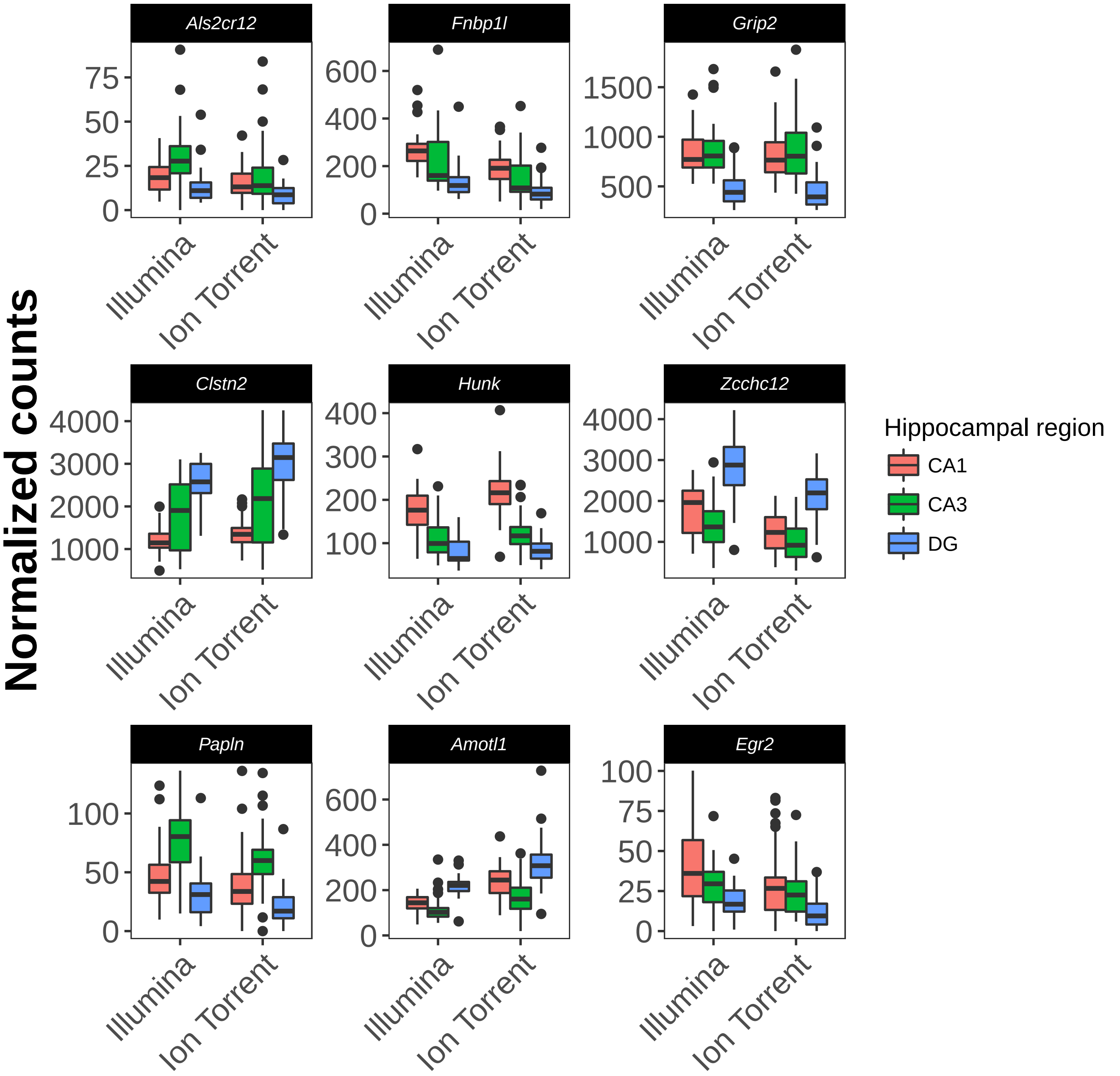
Normalized counts
Als2cr12
Fnbp1l
Grip2
Clstn2
Hunk
Zcchc12
Papln
Amotl1
Egr2
Illumina
Ion Torrent
Hippocampal region
CA1
CA3
DG

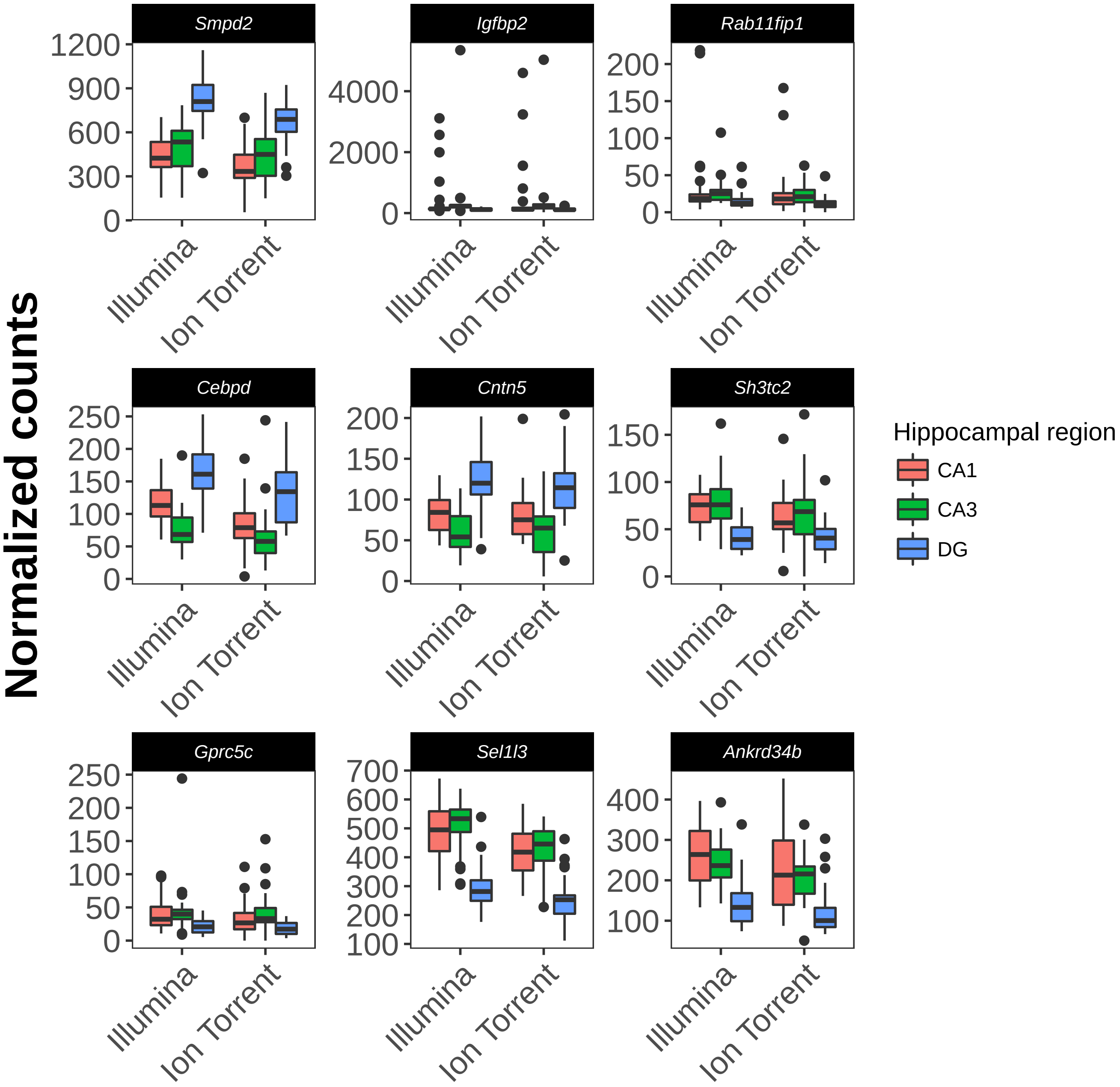
Smpd2
Igfbp2
Rab11fip1
Cebpd
Cntn5
Sh3tc2
Gprc5c
Sel1l3
Ankrd34b
Normalized counts
Illumina
Ion Torrent
Hippocampal region
CA1
CA3
DG

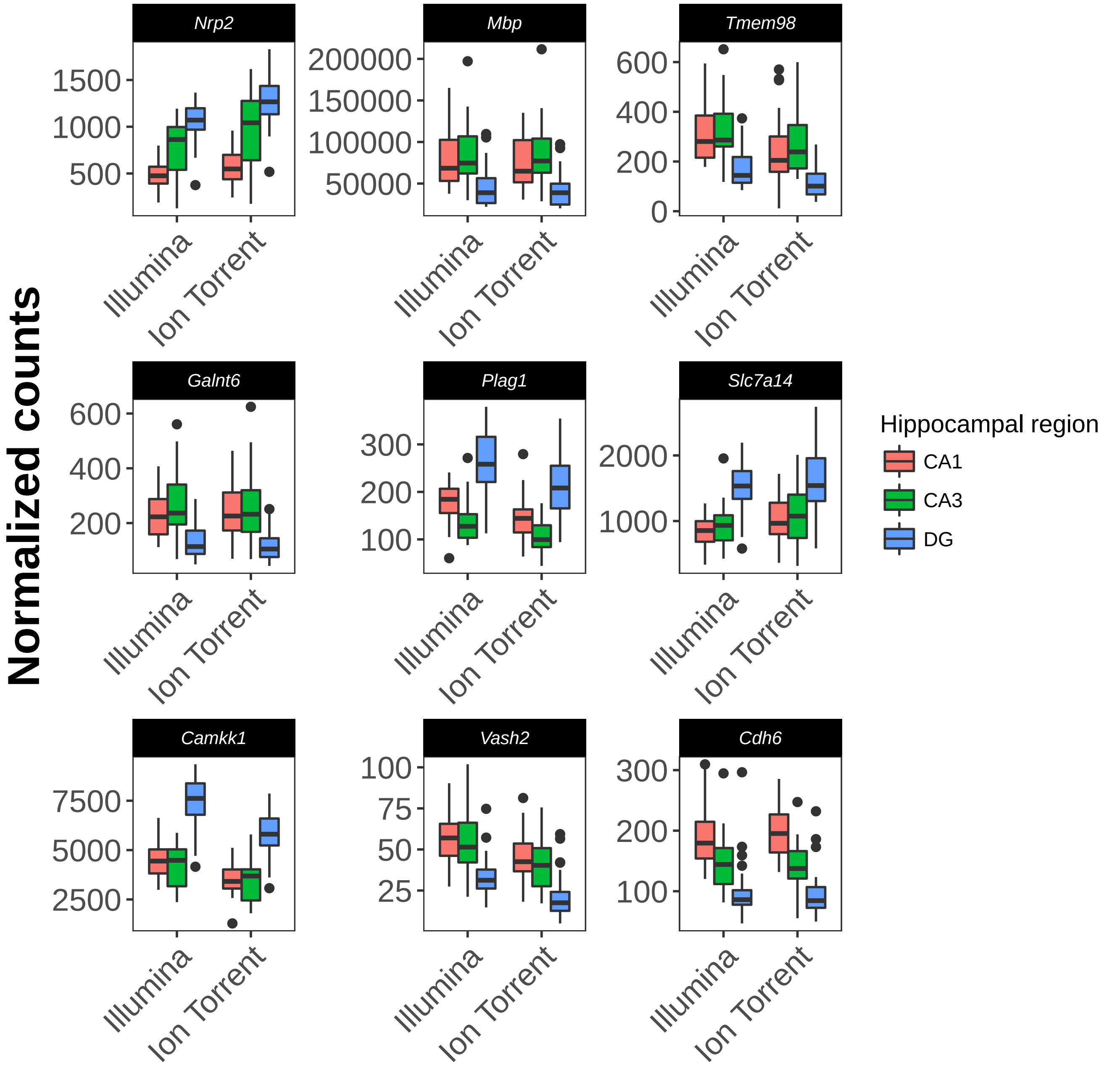

Normalized counts
Nrp2
Mbp
Tmem98
Galnt6
Plag1
Slc7a14
Camkk1
Vash2
Cdh6
Illumina
Ion Torrent
Hippocampal region
CA1
CA3
DG

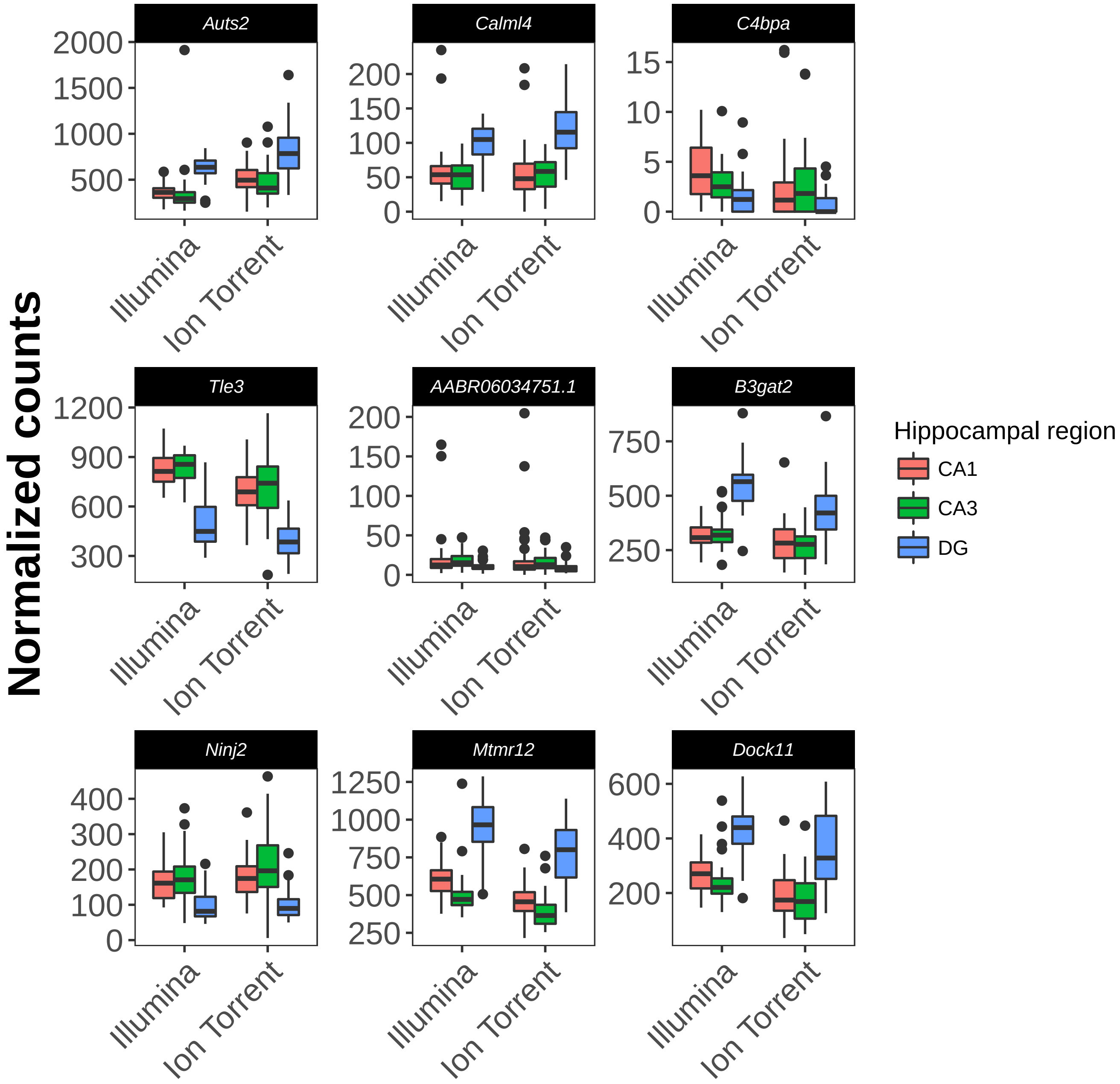

Normalized counts
Auts2
Calml4
C4bpa
Tle3
AABR06034751.1
B3gat2
Ninj2
Mtmr12
Dock11
Illumina
Ion Torrent
Hippocampal region
CA1
CA3
DG

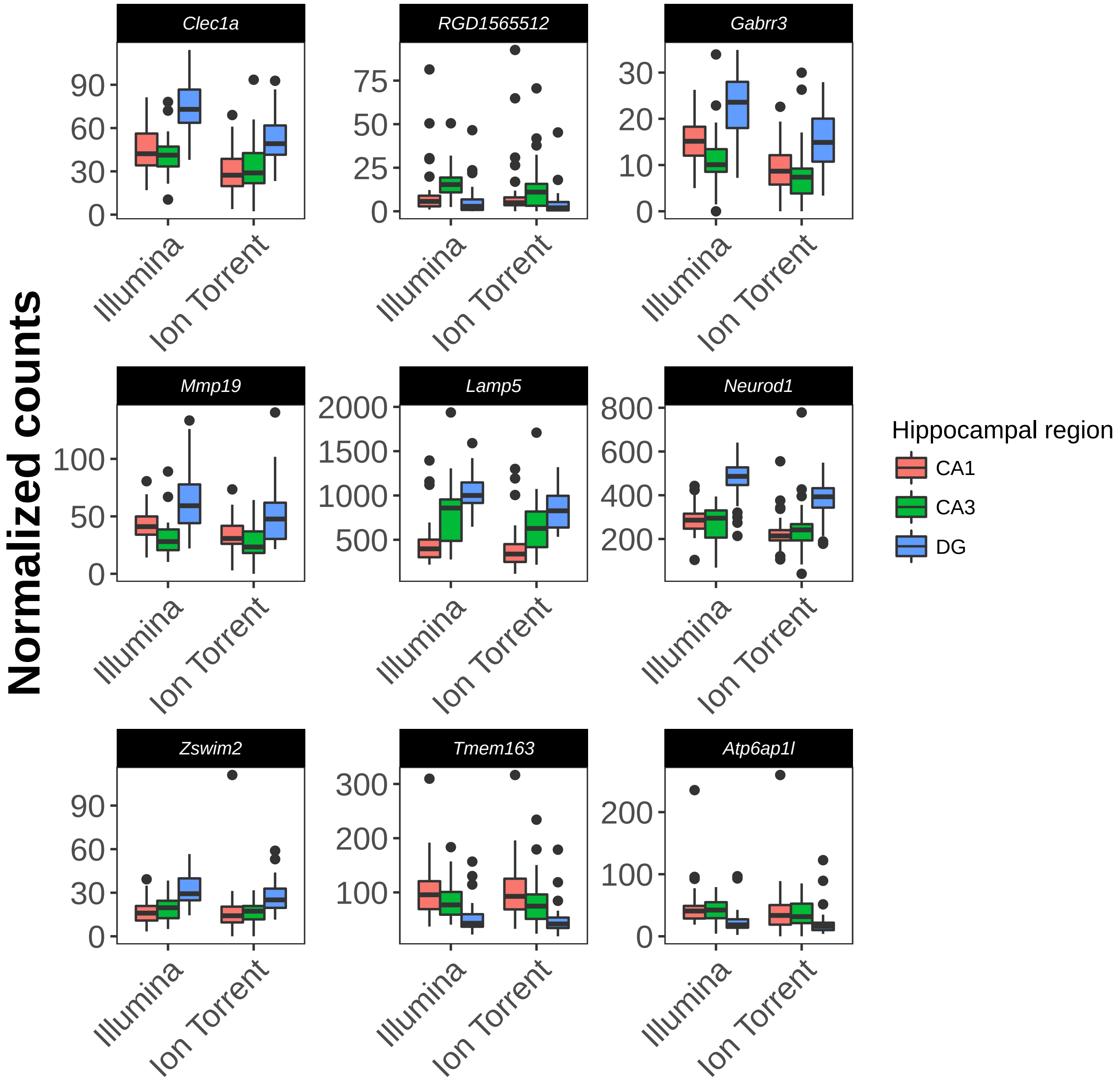
Normalized counts
Clec1a
RGD1565512
Gabrr3
Mmp19
Lamp5
Neurod1
Zswim2
Tmem163
Atp6ap1l
Illumina
Ion Torrent
Hippocampal region
CA1
CA3
DG

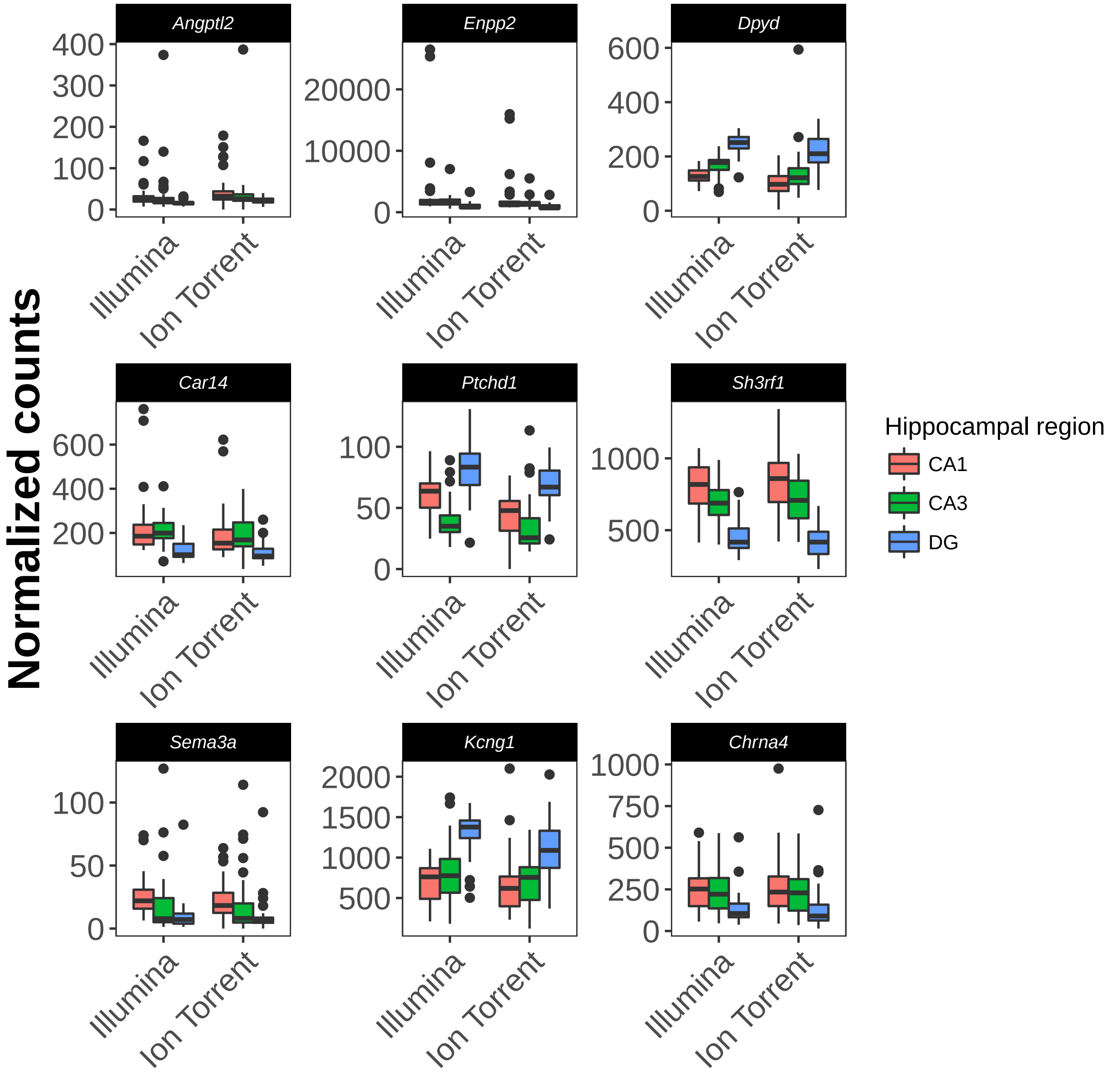

Normalized counts
Angptl2
Enpp2
Dpyd
Car14
Ptchd1
Sh3rf1
Sema3a
Kcng1
Chrna4
Illumina
Ion Torrent
Hippocampal region
CA1
CA3
DG

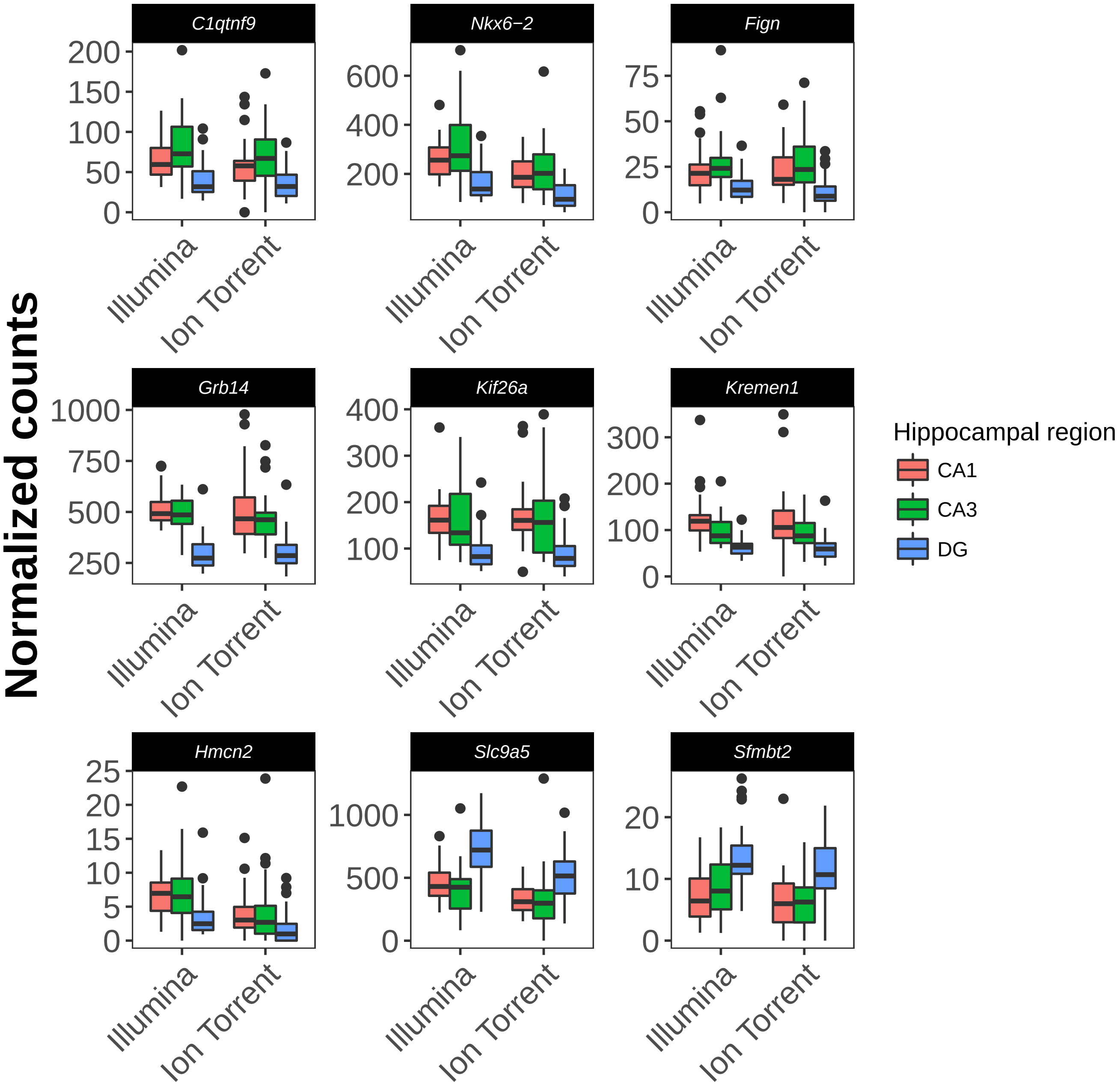

Normalized counts
C1qtnf9
Nkx6−2
Fign
Grb14
Kif26a
Kremen1
Hmcn2
Slc9a5
Sfmbt2
Illumina
Ion Torrent
Hippocampal region
CA1
CA3
DG

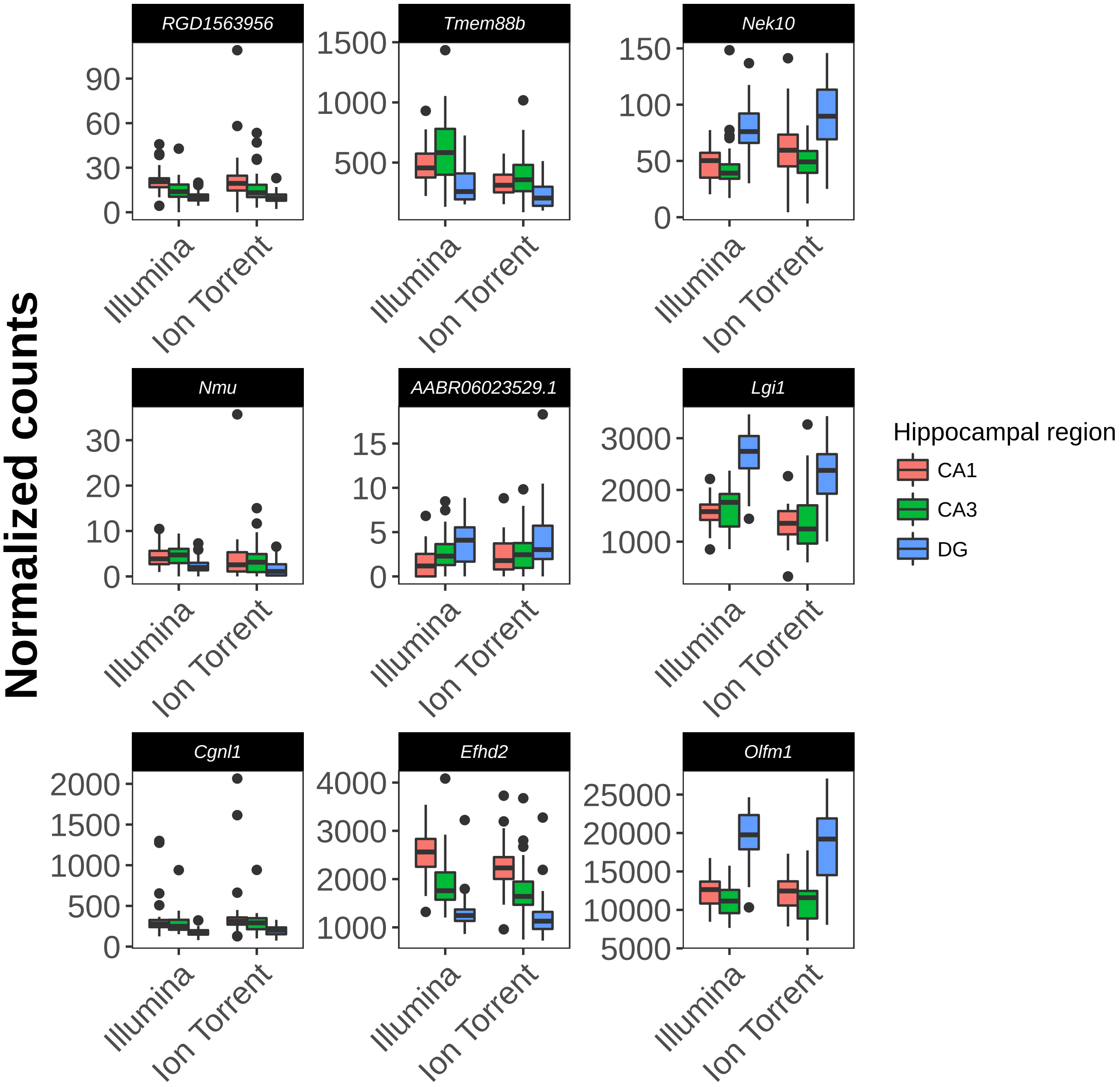

Normalized counts
RGD1563956
Tmem88b
Nek10
Nmu
AABR06023529.1
Lgi1
Cgnl1
Efhd2
Olfm1
Illumina
Ion Torrent
Hippocampal region
CA1
CA3
DG

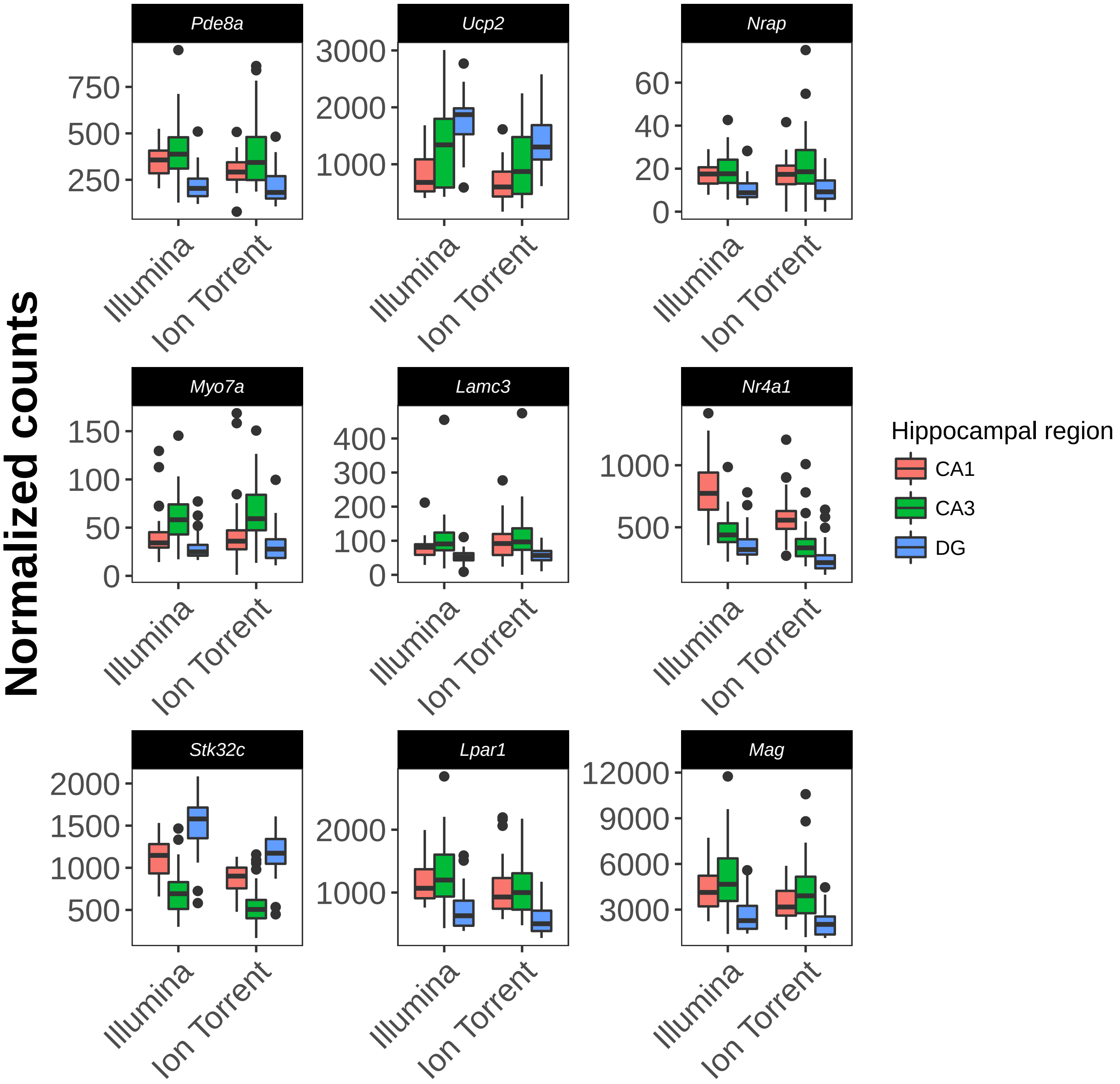
Normalized counts
Pde8a
Ucp2
Nrap
Myo7a
Lamc3
Nr4a1
Stk32c
Lpar1
Mag
Illumina
Ion Torrent
Hippocampal region
CA1
CA3
DG
750
500
250
3000
2000
1000
60
40
20
0
150
100
50
400
300
200
1500
12000
9000
6000

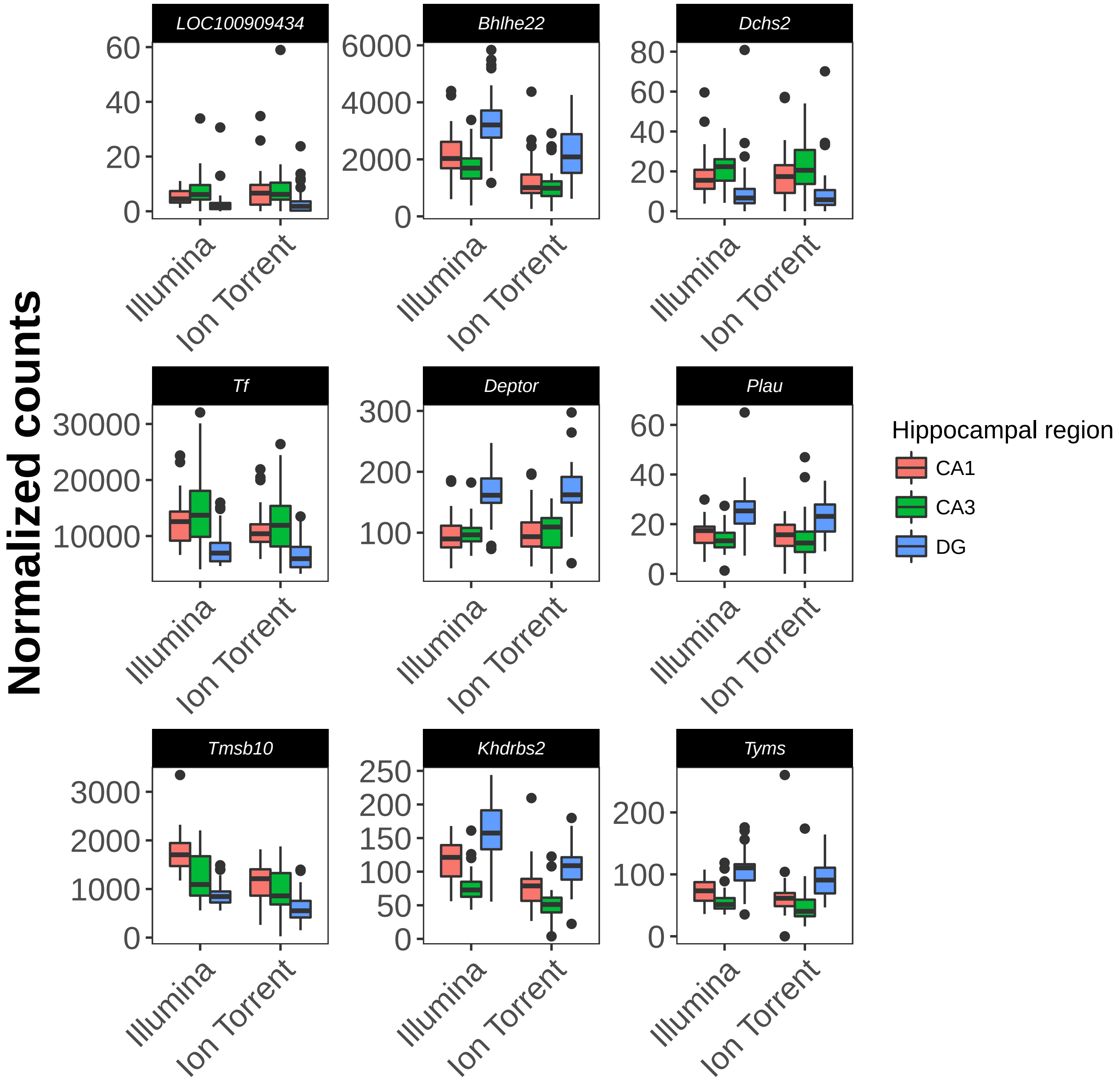

Normalized counts
LOC100909434
Bhlhe22
Dchs2
Tf
Deptor
Plau
Tmsb10
Khdrbs2
Tyms
Illumina
Ion Torrent
Hippocampal region
CA1
CA3
DG

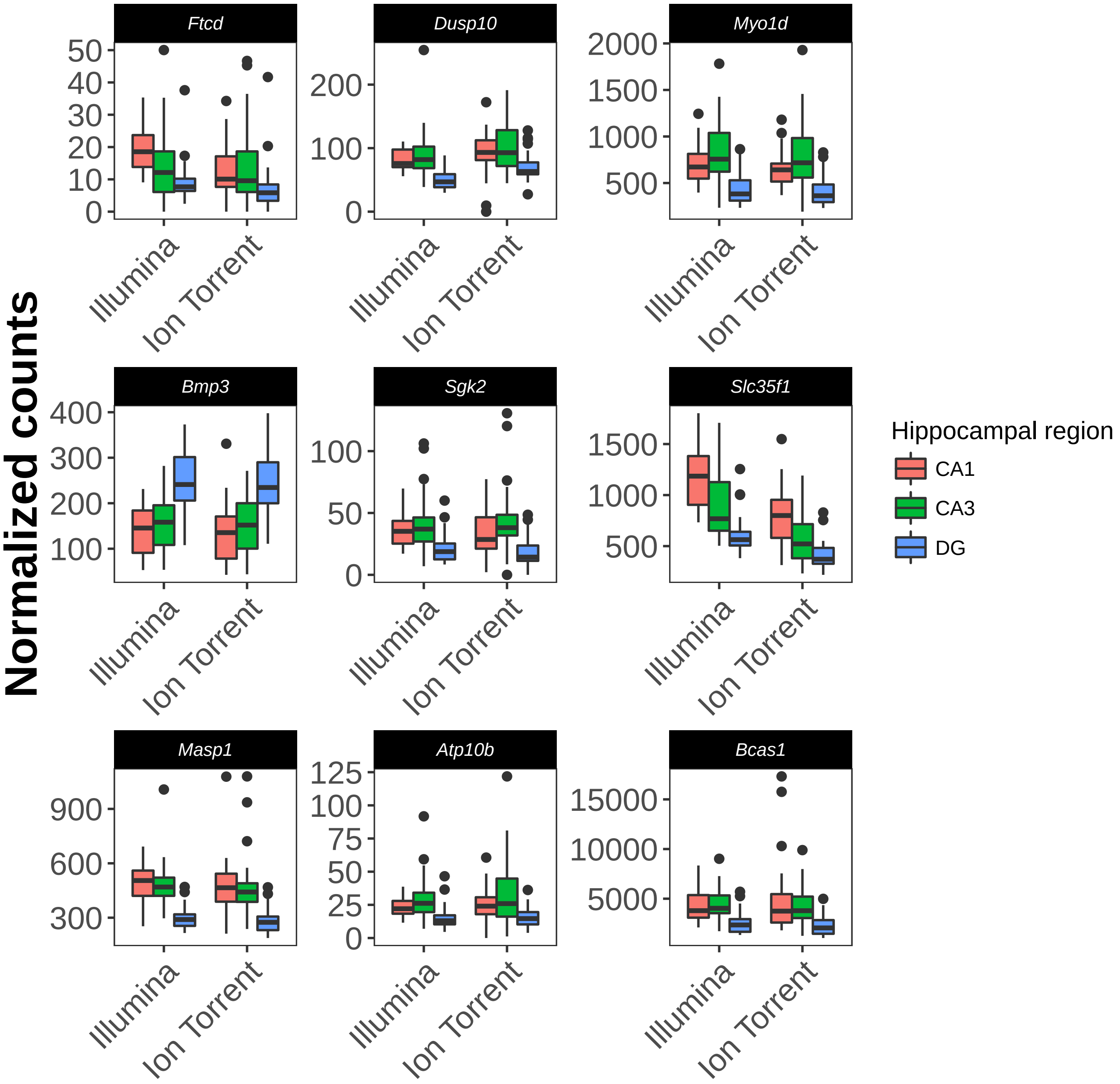
Normalized counts
Ftcd
Dusp10
Myo1d
Bmp3
Sgk2
Slc35f1
Masp1
Atp10b
Bcas1
Illumina
Ion Torrent
Hippocampal region
CA1
CA3
DG

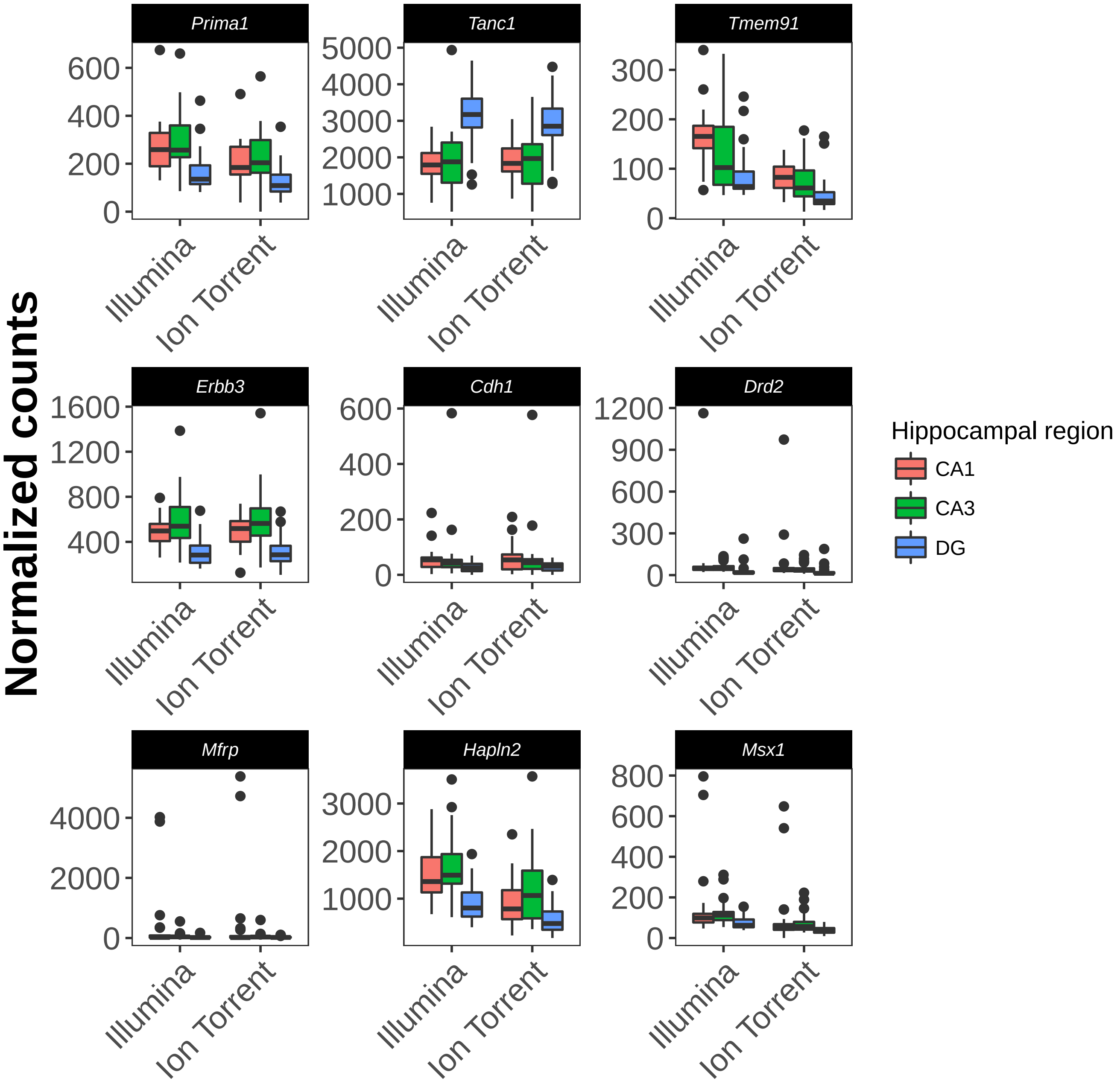
Normalized counts
Prima1
Tanc1
Tmem91
Erbb3
Cdh1
Drd2
Mfrp
Hapln2
Msx1
Illumina
Ion Torrent
Hippocampal region
CA1
CA3
DG

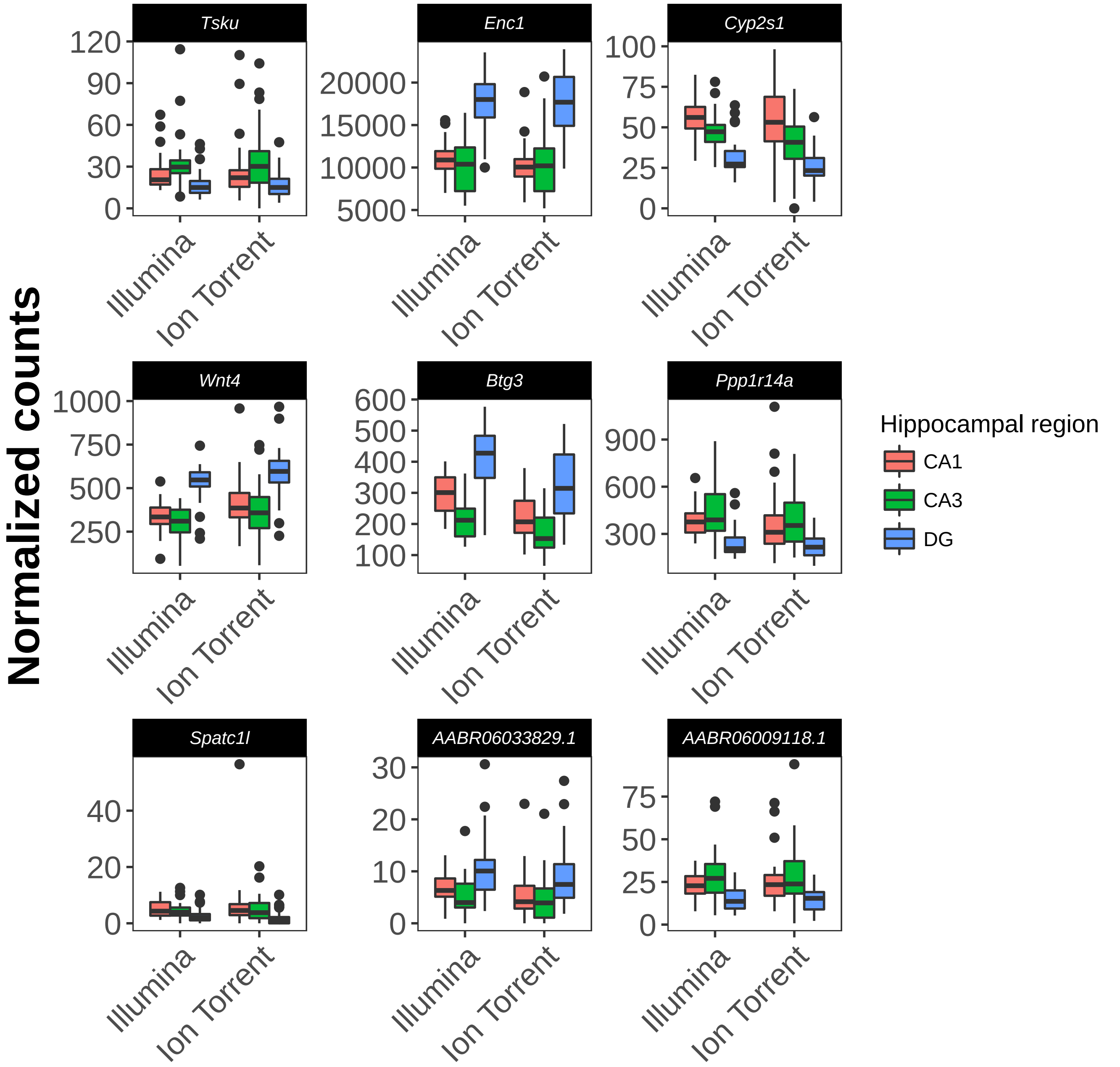

Normalized counts
Tsku
Enc1
Cyp2s1
Wnt4
Btg3
Ppp1r14a
Spatc1l
AABR06033829.1
AABR06009118.1
Illumina
Ion Torrent
Hippocampal region
CA1
CA3
DG

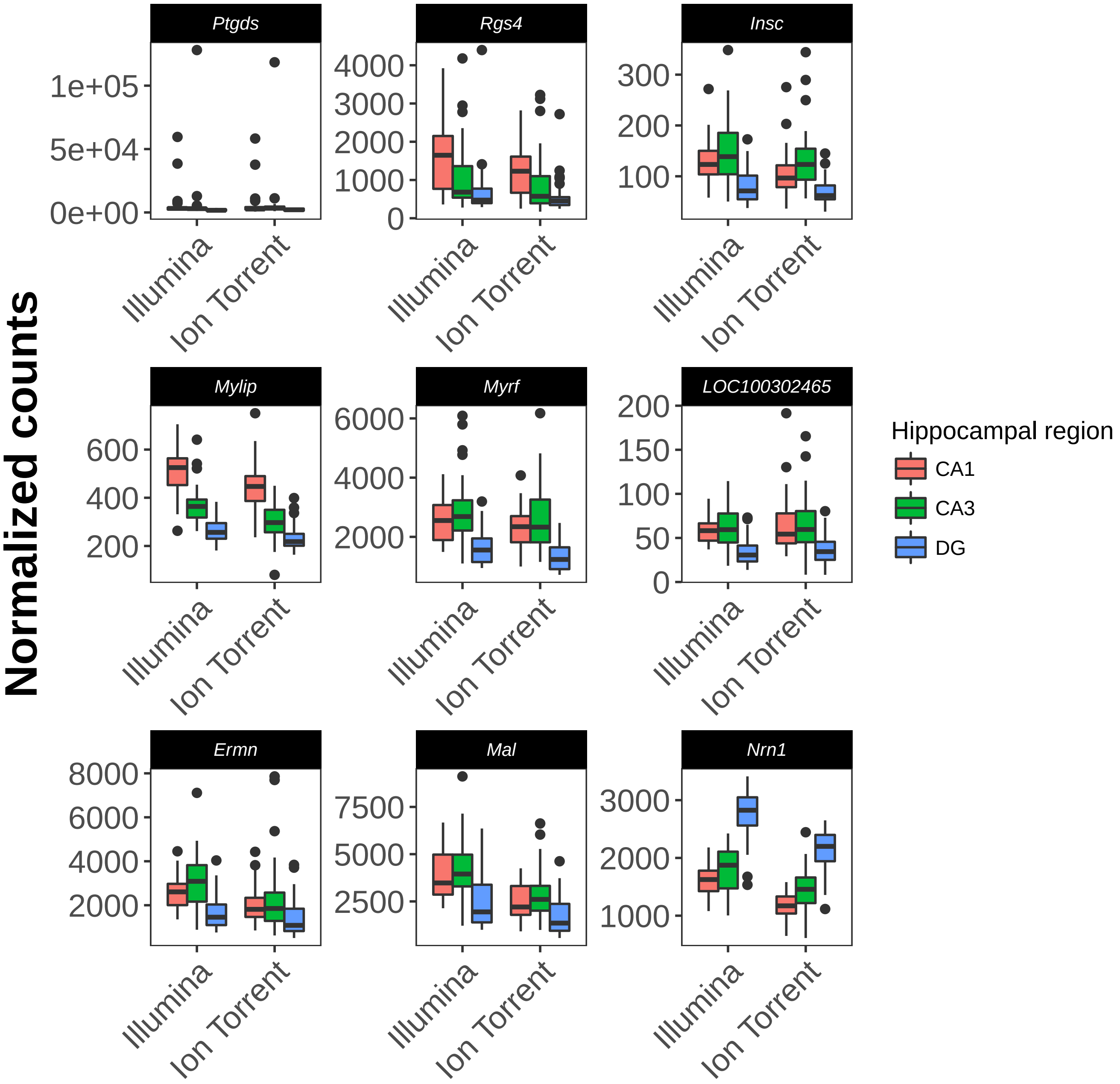
Normalized counts
Ptgds
Rgs4
Insc
Mylip
Myrf
LOC100302465
Ermn
Mal
Nrn1
Illumina
Ion Torrent
Hippocampal region
CA1
CA3
DG

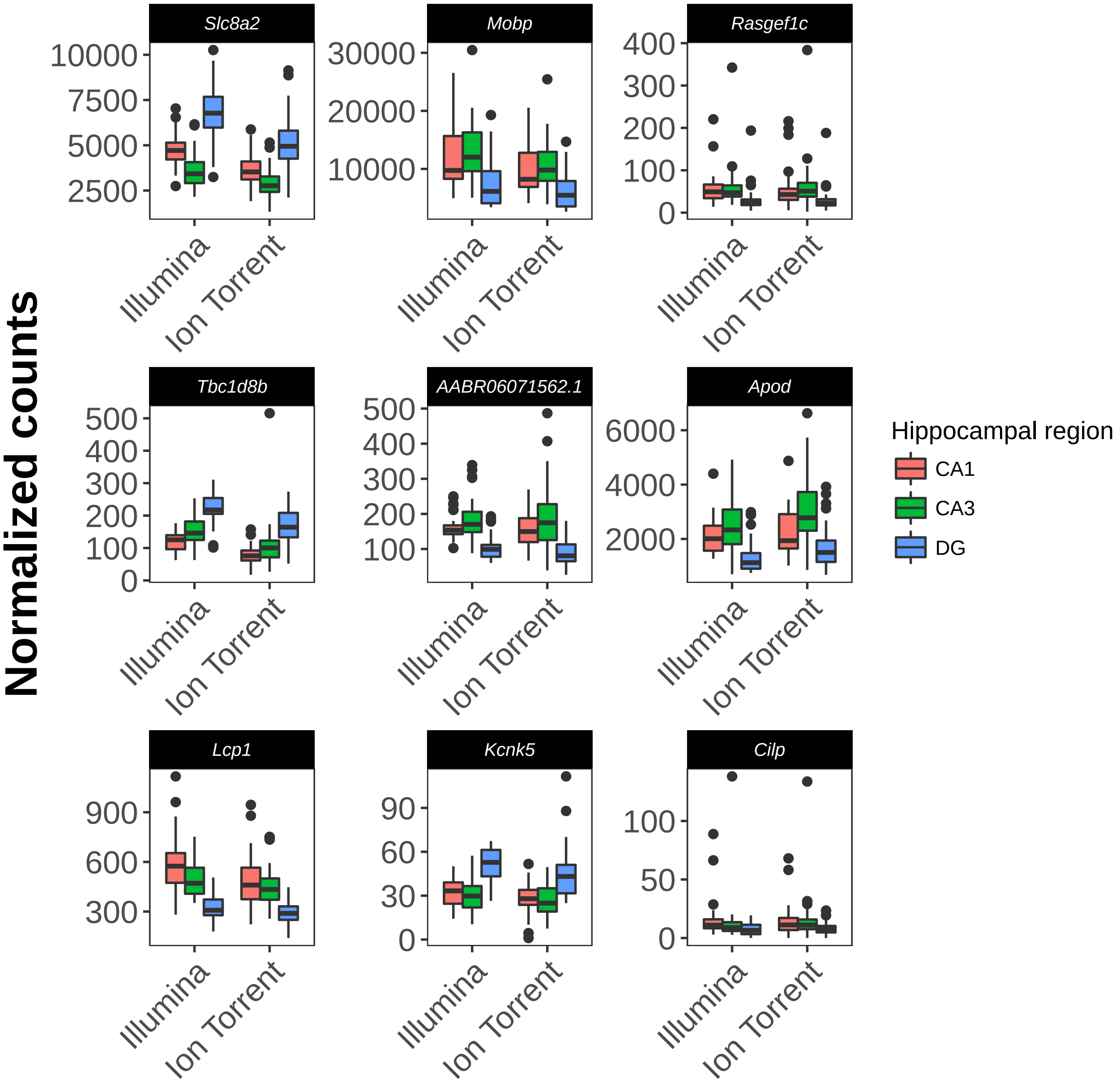
Normalized counts
Slc8a2
Mobp
Rasgef1c
Tbc1d8b
AABR06071562.1
Apod
Lcp1
Kcnk5
Cilp
Illumina
Ion Torrent
Hippocampal region
CA1
CA3
DG

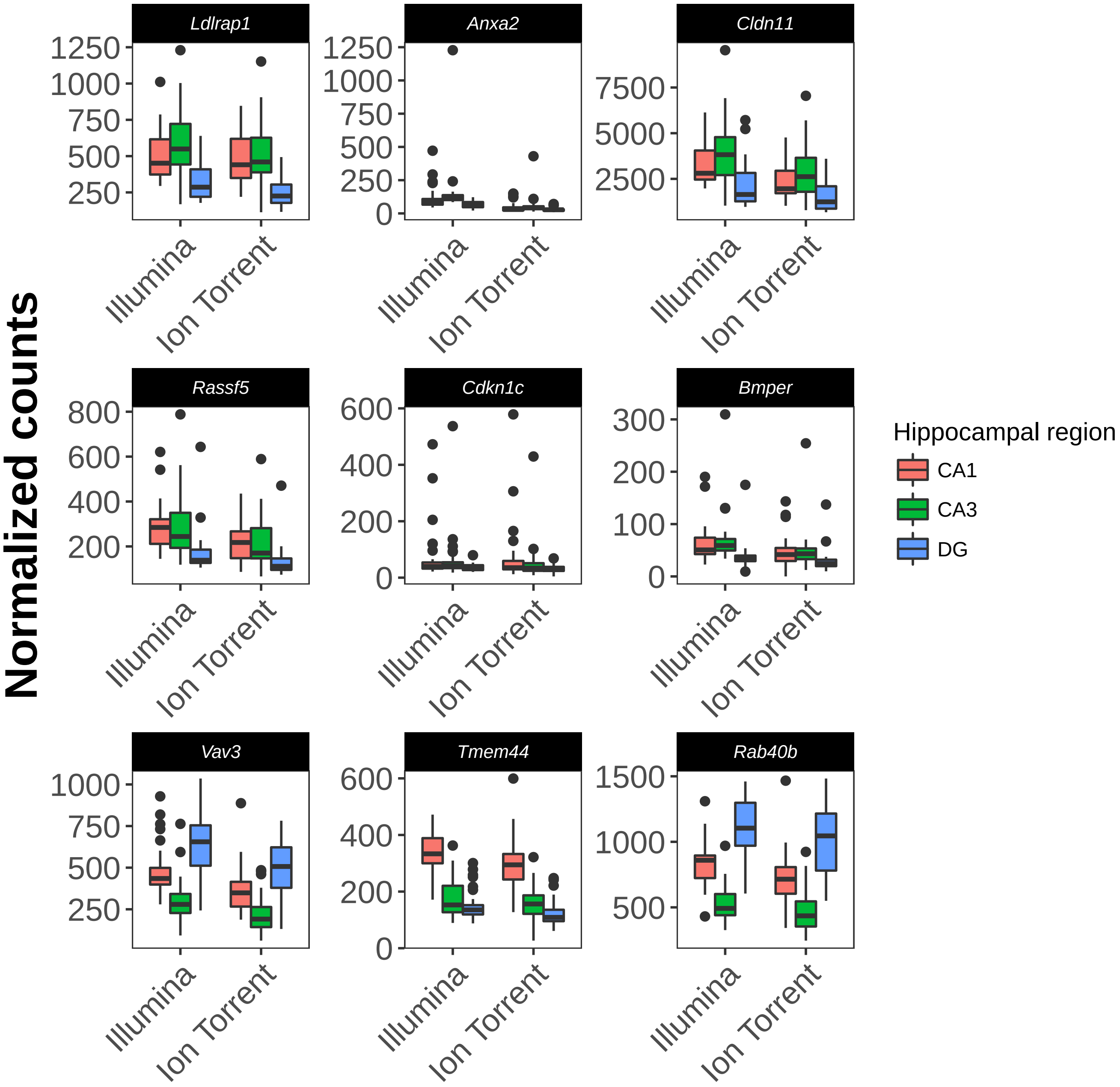

Normalized counts
Ldlrap1
Anxa2
Cldn11
Rassf5
Cdkn1c
Bmper
Vav3
Tmem44
Rab40b
Illumina
Ion Torrent
Hippocampal region
CA1
CA3
DG

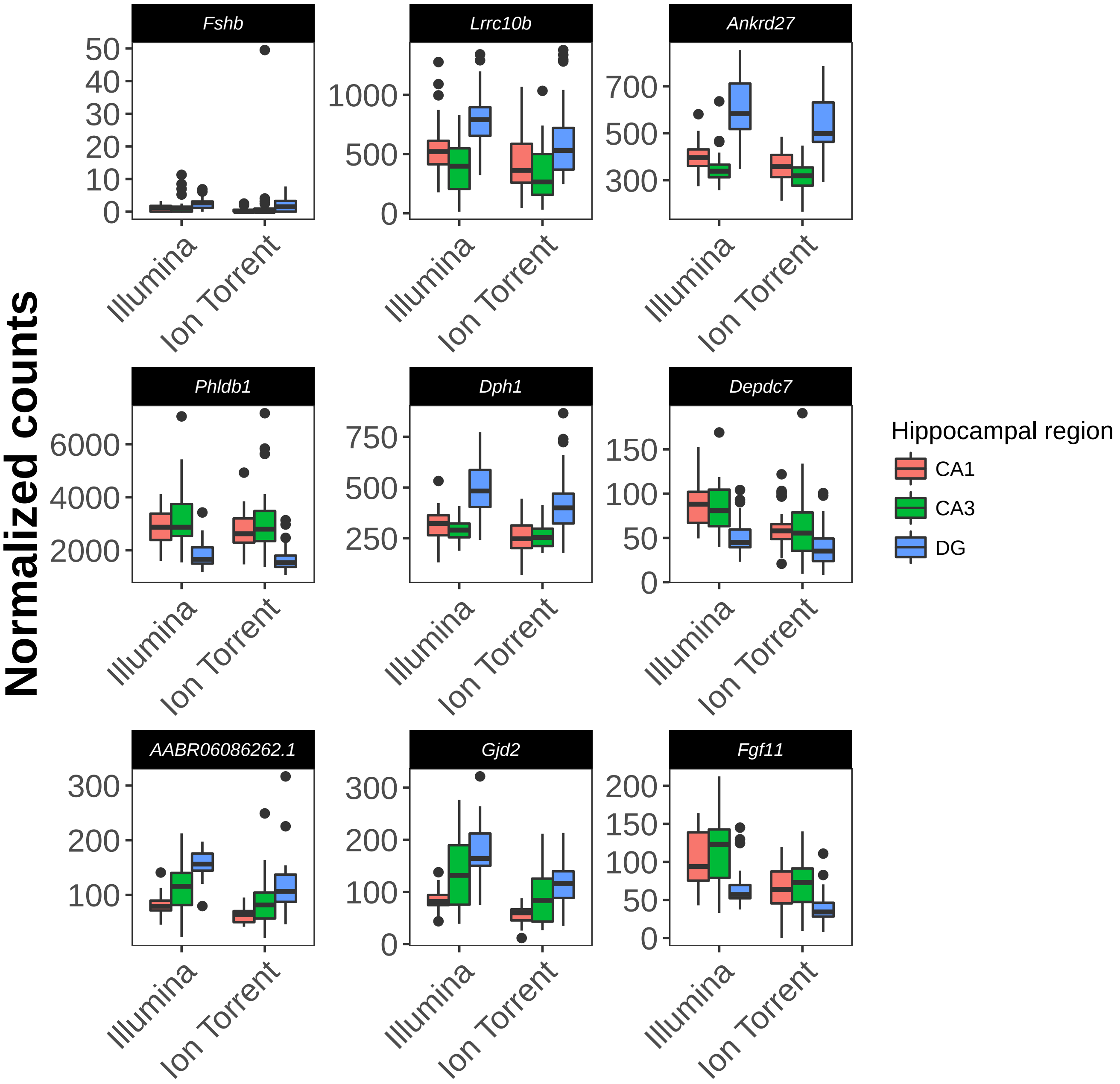

Normalized counts
Fshb
Lrrc10b
Ankrd27
Phldb1
Dph1
Depdc7
AABR06086262.1
Gjd2
Fgf11
Illumina
Ion Torrent
Hippocampal region
CA1
CA3
DG

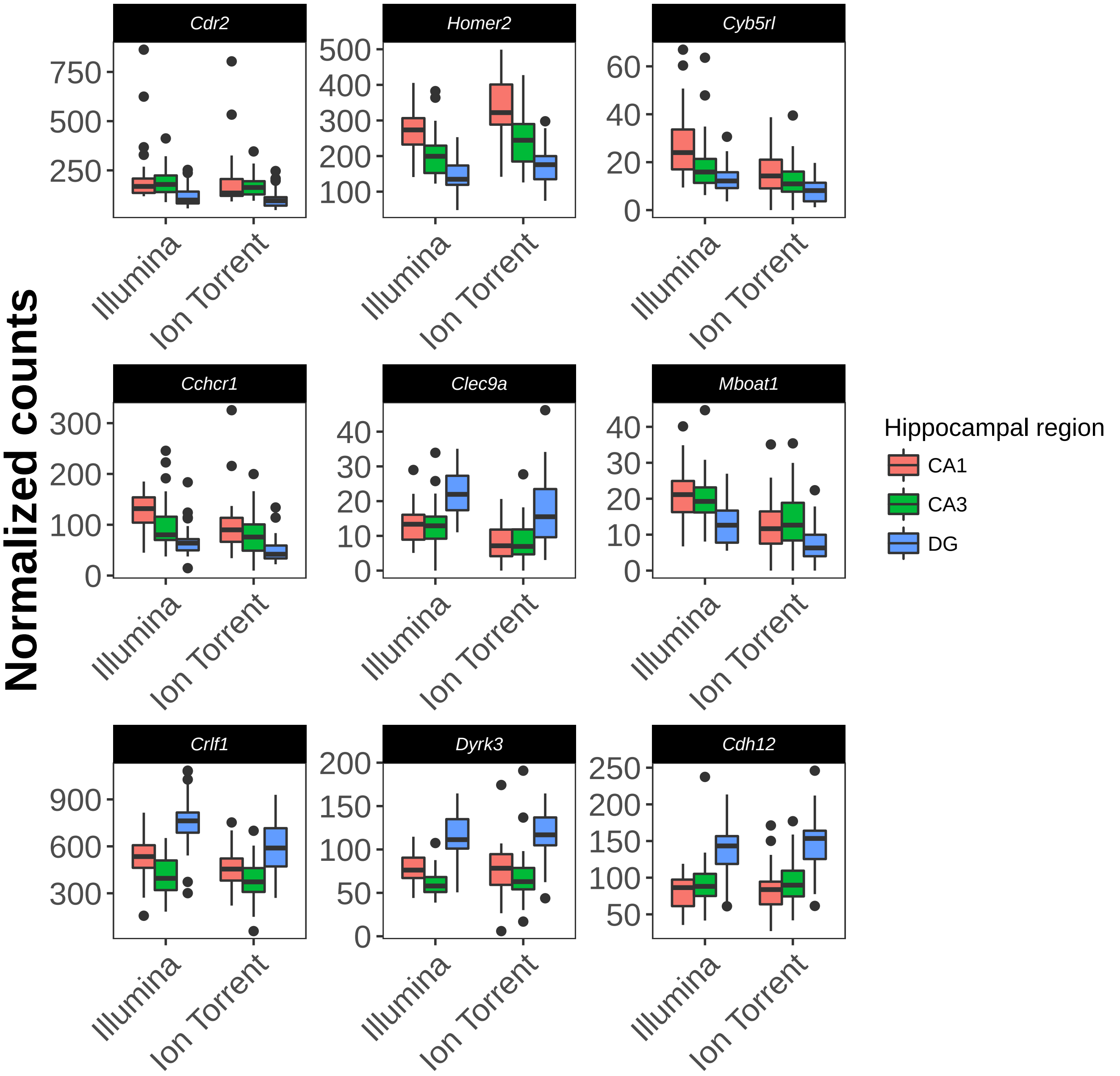
Normalized counts
Cdr2
Homer2
Cyb5rl
Cchcr1
Clec9a
Mboat1
Crlf1
Dyrk3
Cdh12
Illumina
Ion Torrent
Hippocampal region
CA1
CA3
DG

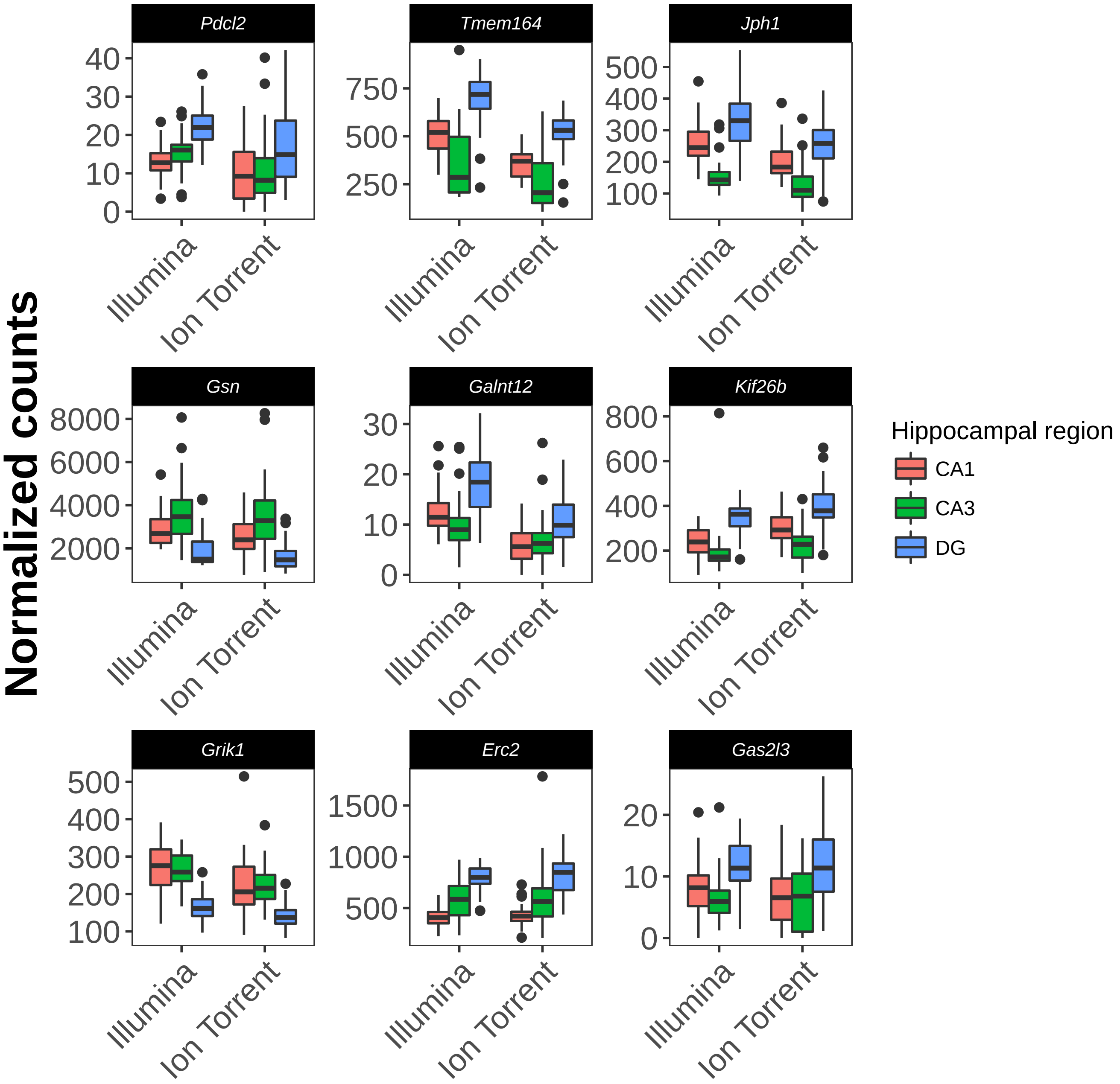

Normalized counts
Pdcl2
Tmem164
Jph1
Gsn
Galnt12
Kif26b
Grik1
Erc2
Gas2l3
Illumina
Ion Torrent
Hippocampal region
CA1
CA3
DG

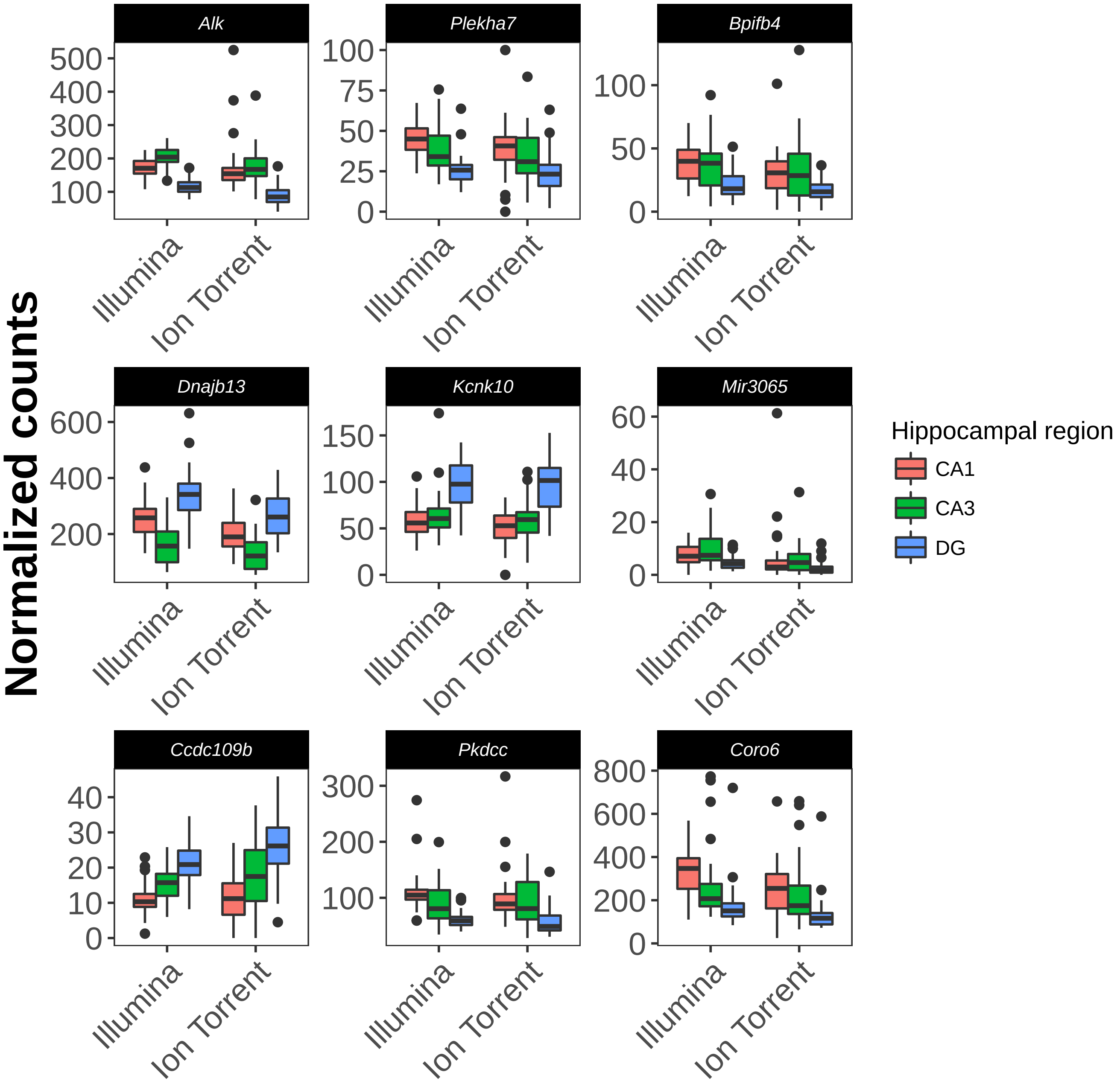
Normalized counts
Alk
Plekha7
Bpifb4
Dnajb13
Kcnk10
Mir3065
Ccdc109b
Pkdcc
Coro6
Illumina
Ion Torrent
Hippocampal region
CA1
CA3
DG

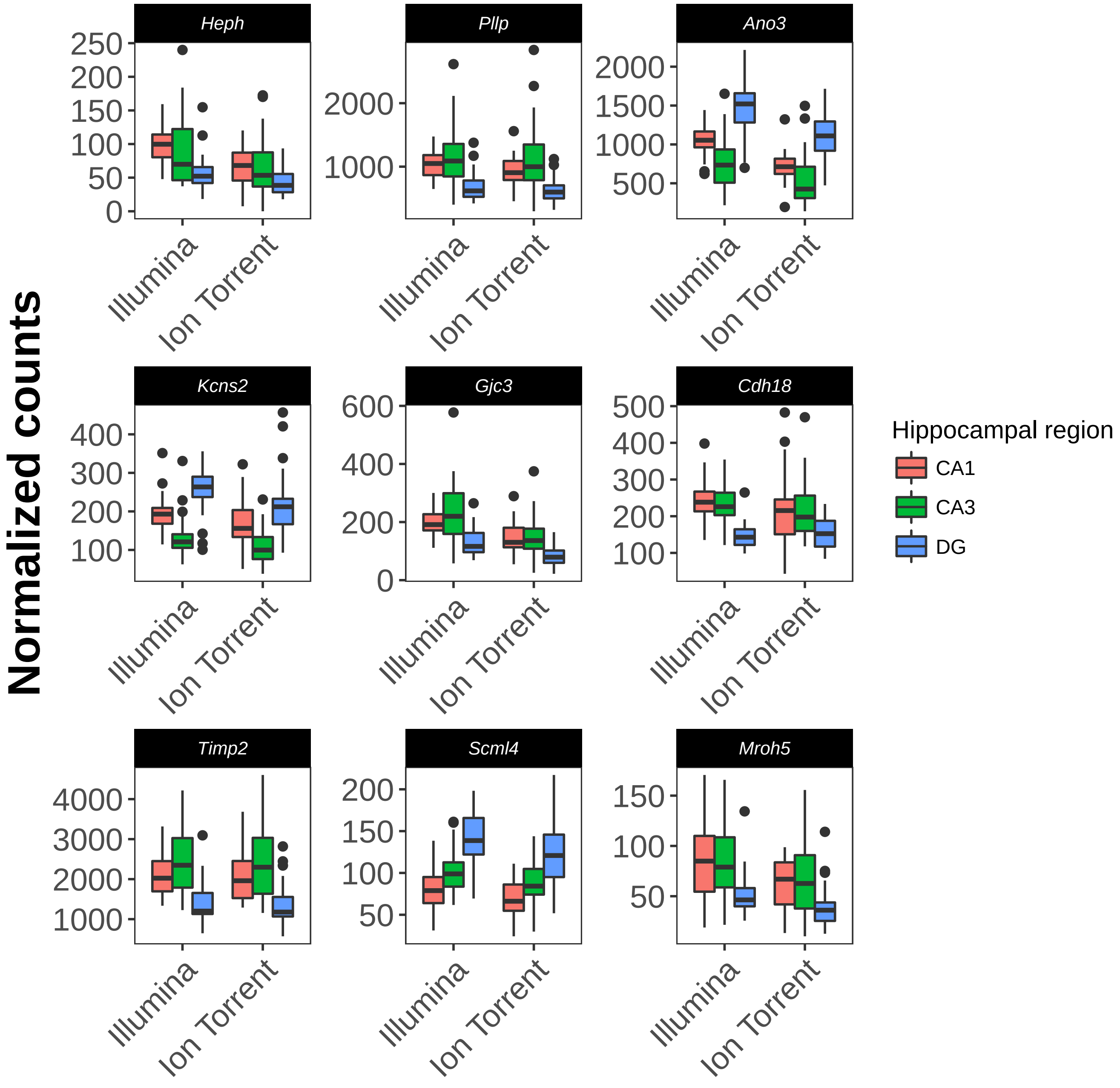
Normalized counts
Heph
Pllp
Ano3
Kcns2
Gjc3
Cdh18
Timp2
Scml4
Mroh5
Illumina
Ion Torrent
Hippocampal region
CA1
CA3
DG

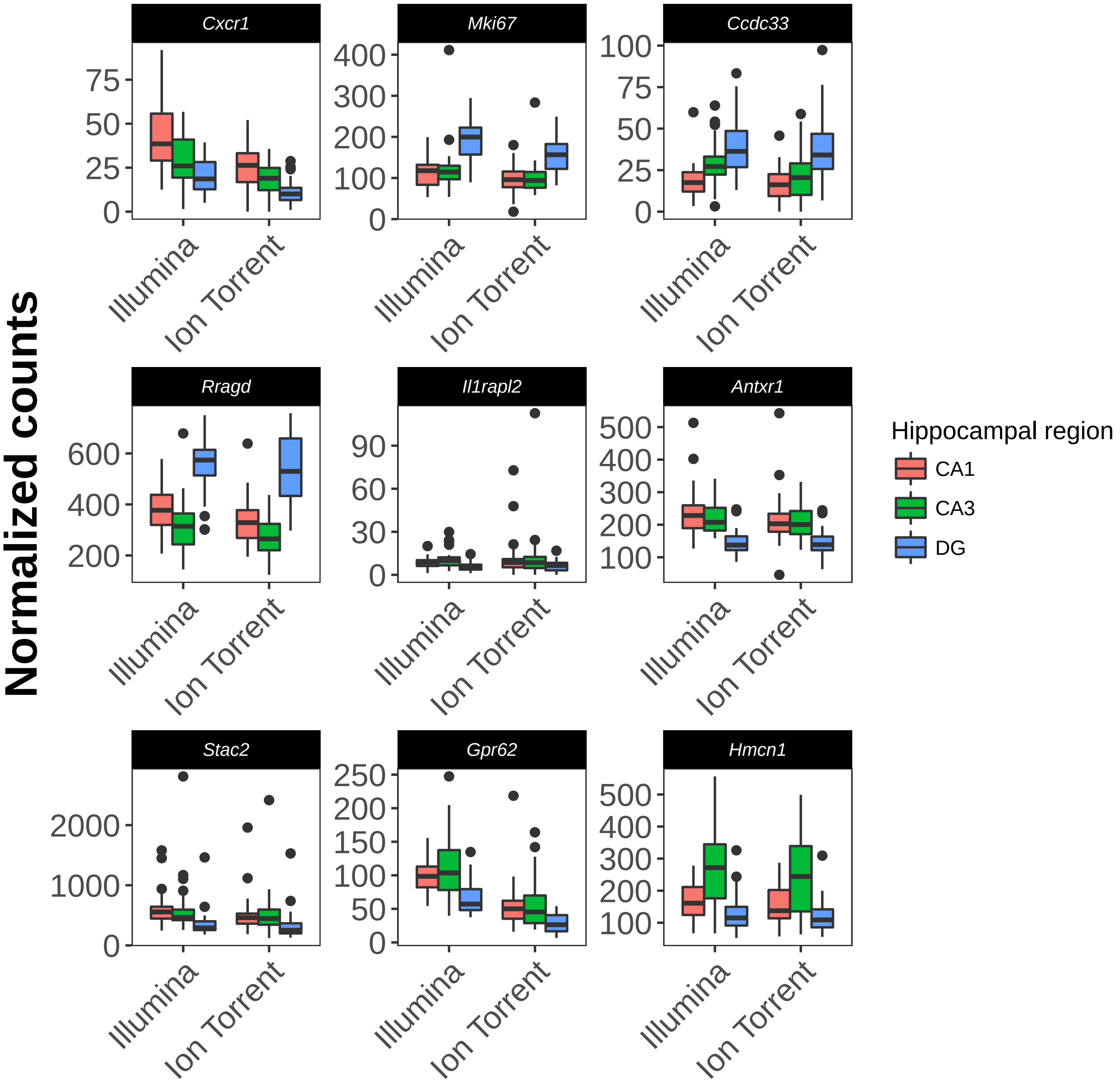

Normalized counts
Cxcr1
Mki67
Ccdc33
Rragd
Il1rapl2
Antxr1
Stac2
Gpr62
Hmcn1
Illumina
Ion Torrent
Hippocampal region
CA1
CA3
DG

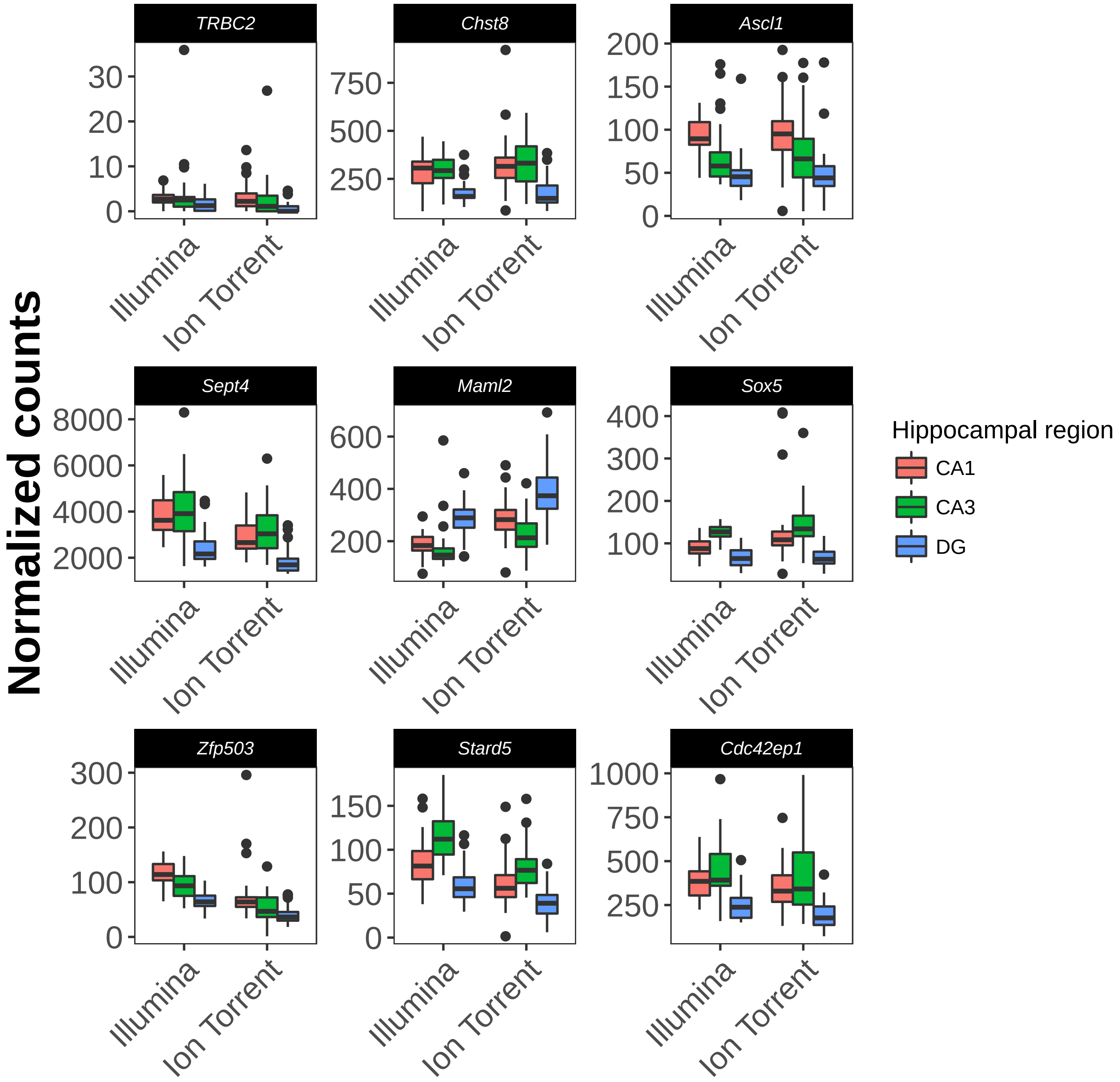
Normalized counts
TRBC2
Chst8
Ascl1
Sept4
Maml2
Sox5
Zfp503
Stard5
Cdc42ep1
Illumina
Ion Torrent
Hippocampal region
CA1
CA3
DG

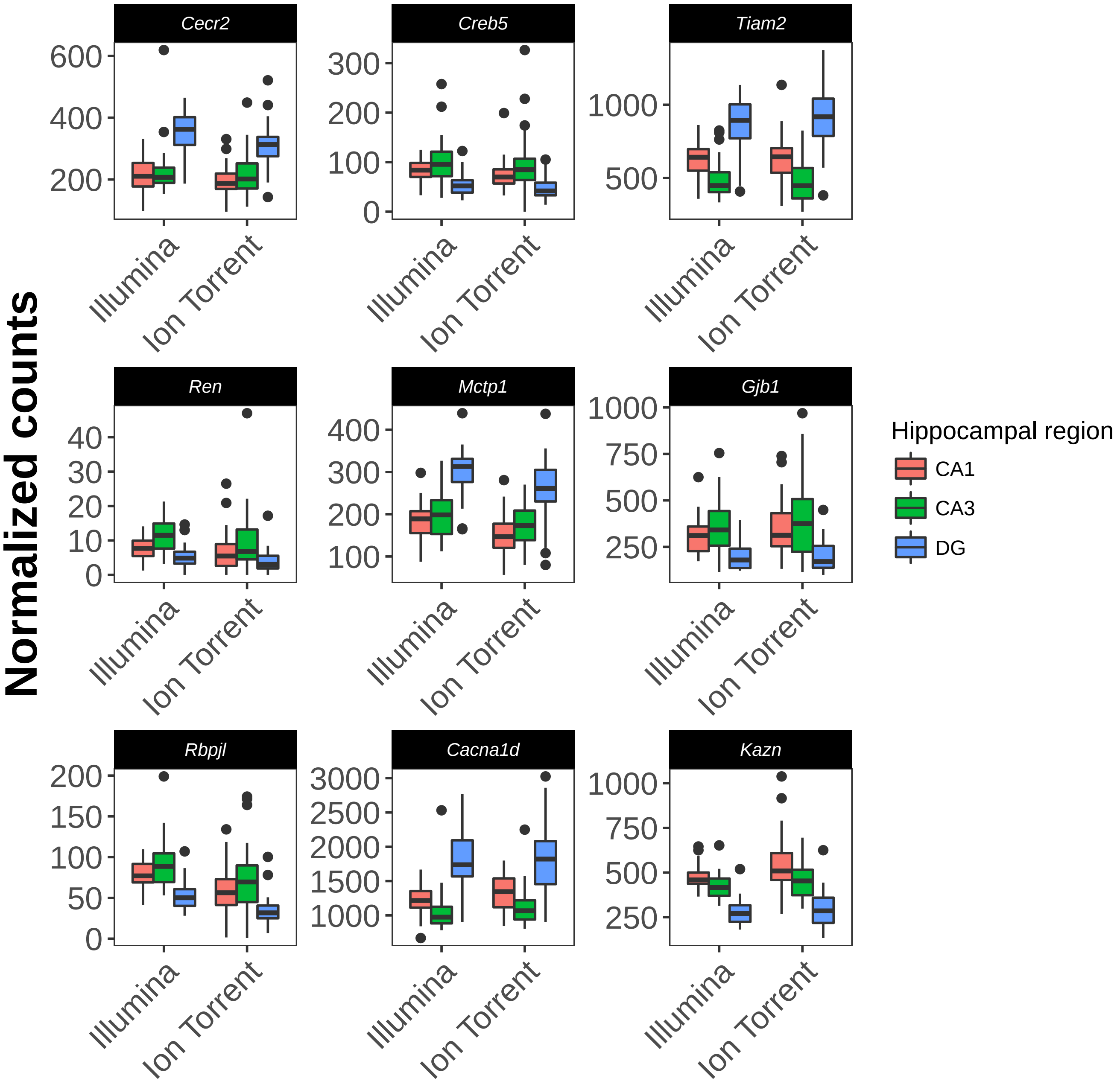

Normalized counts
Cecr2
Creb5
Tiam2
Ren
Mctp1
Gjb1
Rbpjl
Cacna1d
Kazn
Illumina
Ion Torrent
Hippocampal region
CA1
CA3
DG

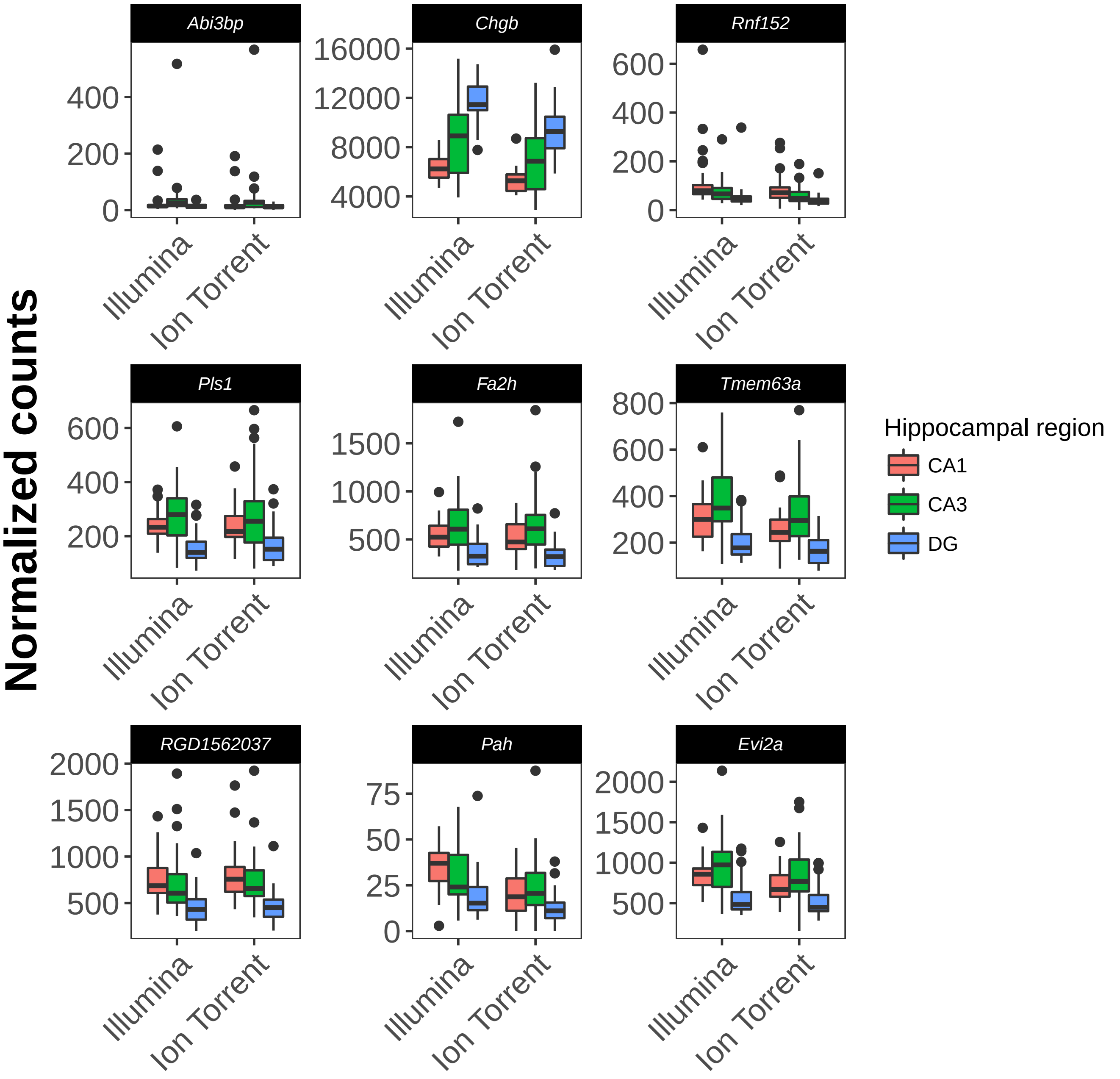

Normalized counts
Abi3bp
Chgb
Rnf152
Pls1
Fa2h
Tmem63a
RGD1562037
Pah
Evi2a
Illumina
Ion Torrent
Hippocampal region
CA1
CA3
DG

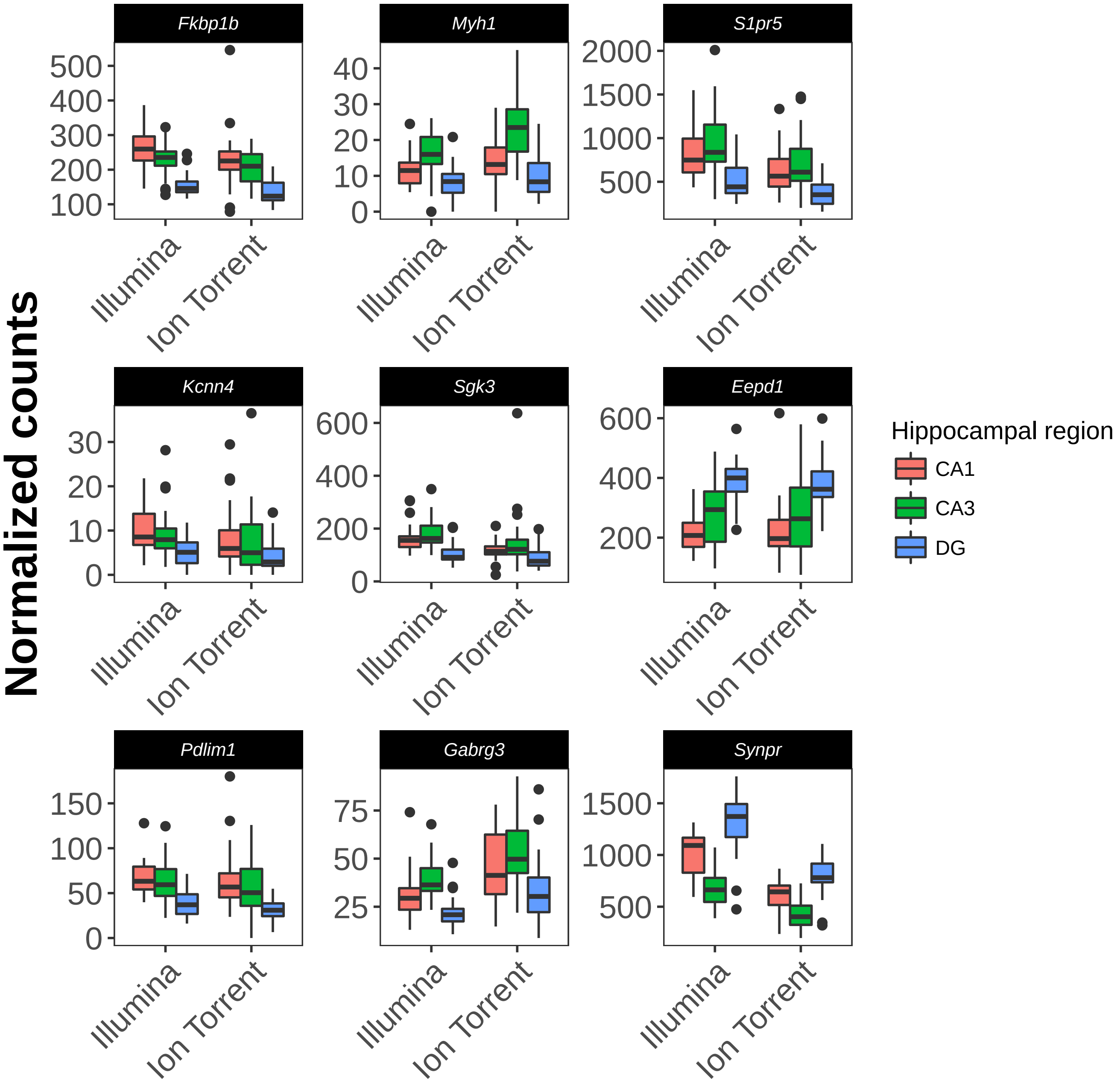
Fkbp1b
Myh1
S1pr5
Kcnn4
Sgk3
Eepd1
Pdlim1
Gabrg3
Synpr
Normalized counts
Illumina
Ion Torrent
Hippocampal region
CA1
CA3
DG

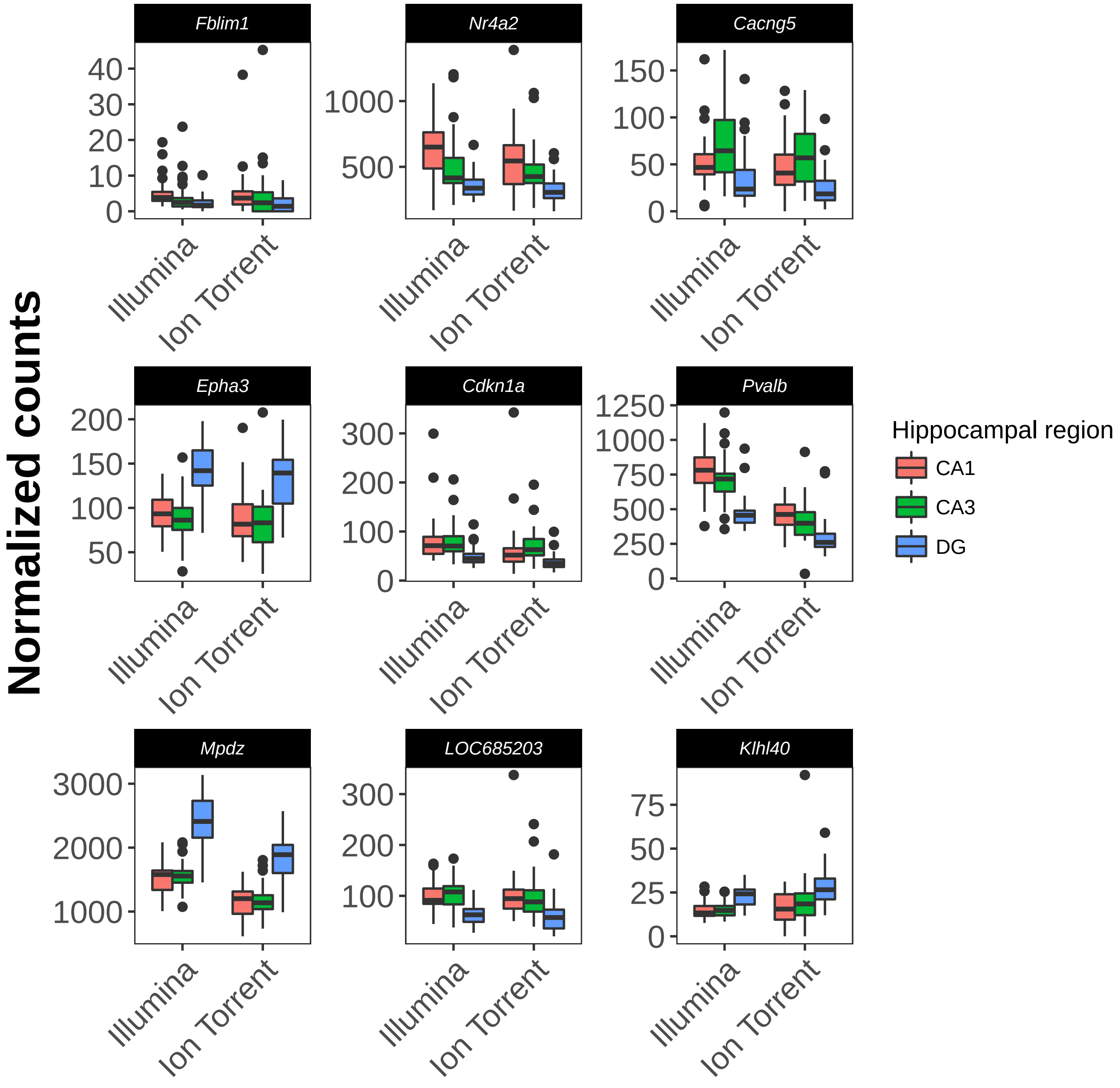

Normalized counts
Fblim1
Nr4a2
Cacng5
Epha3
Cdkn1a
Pvalb
Mpdz
LOC685203
Klhl40
Illumina
Ion Torrent
Hippocampal region
CA1
CA3
DG

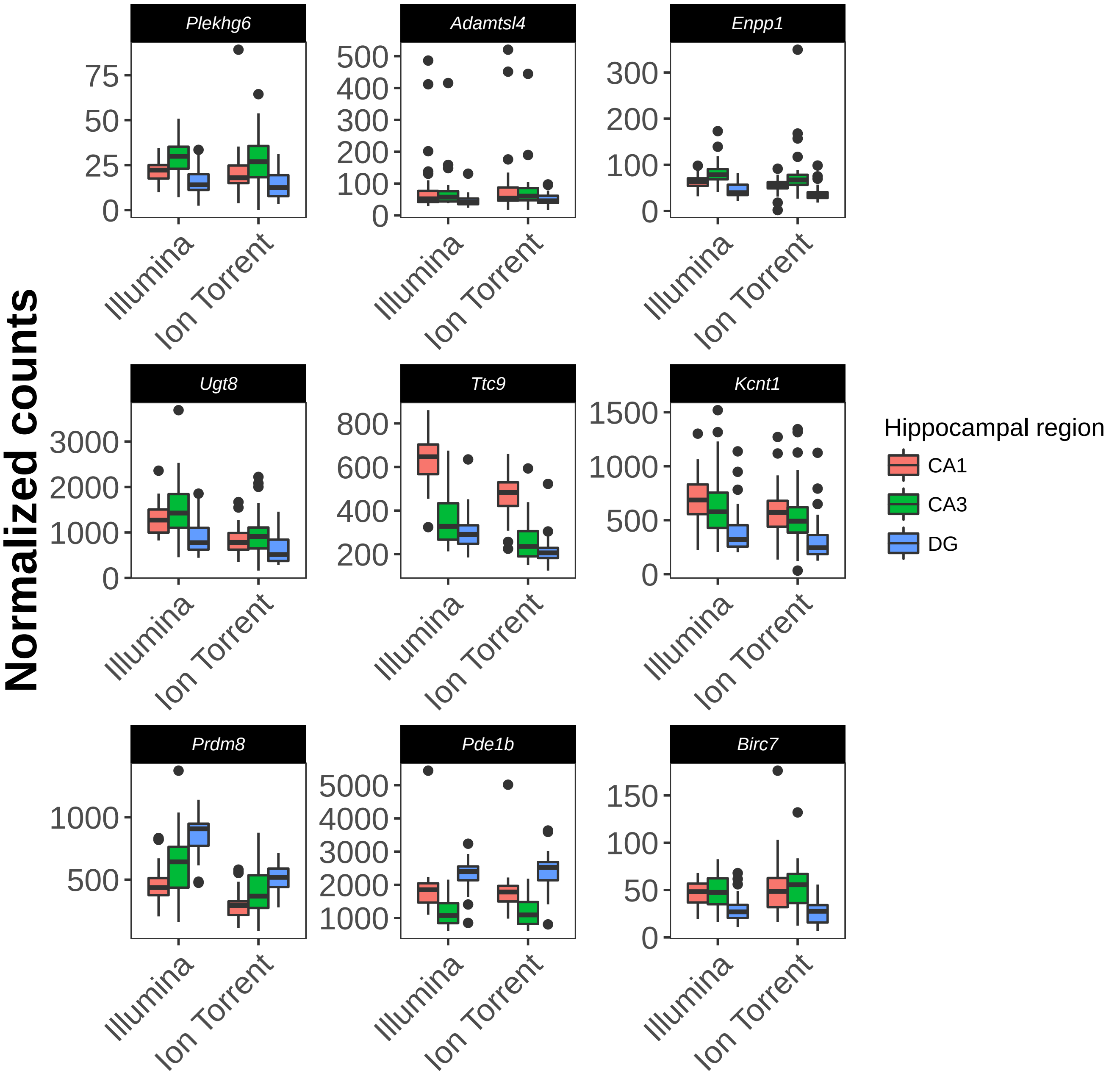
Normalized counts
Plekhg6
Adamtsl4
Enpp1
Ugt8
Ttc9
Kcnt1
Prdm8
Pde1b
Birc7
Illumina
Ion Torrent
Hippocampal region
CA1
CA3
DG

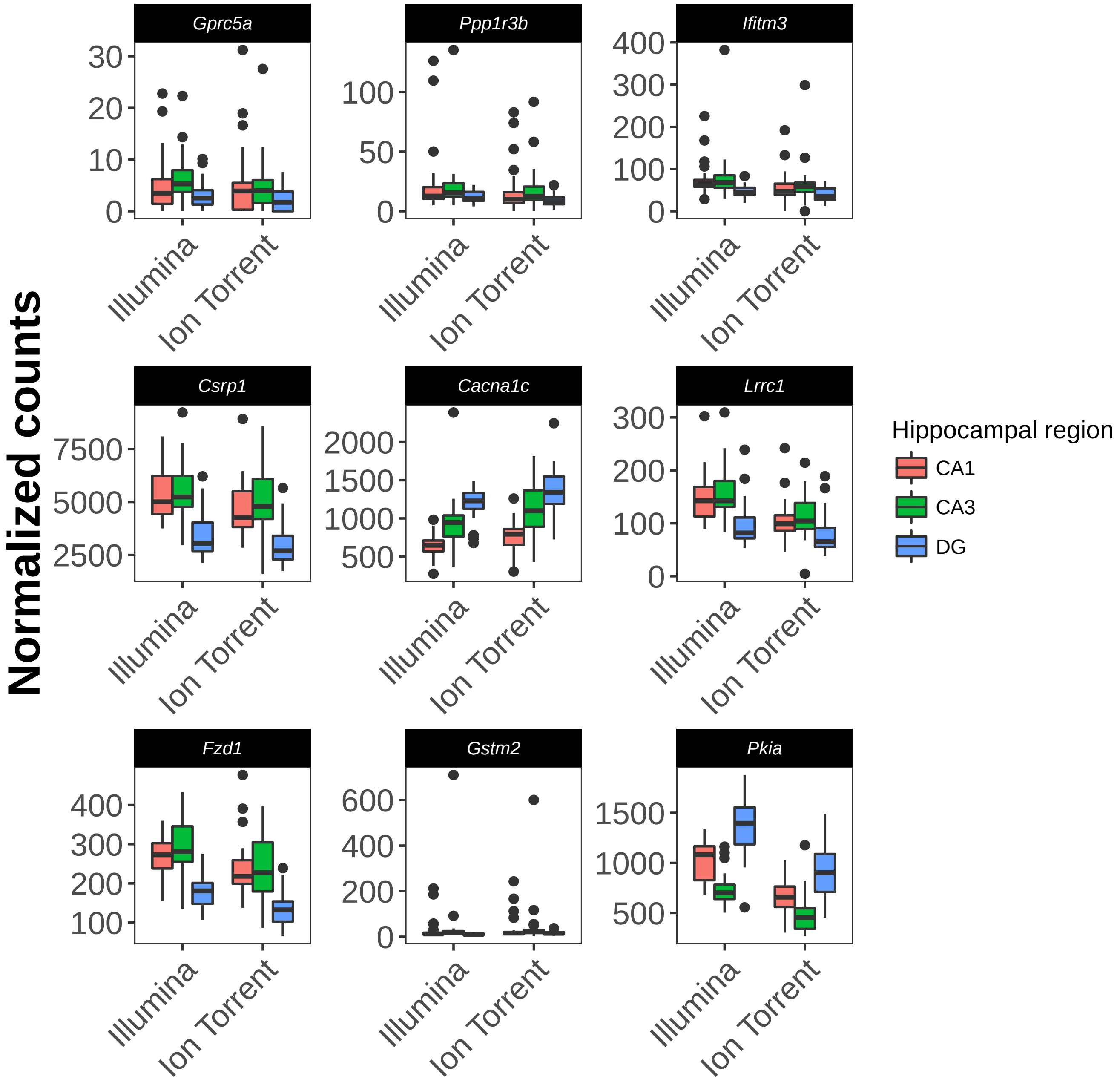

Normalized counts
Gprc5a
Ppp1r3b
Ifitm3
Csrp1
Cacna1c
Lrrc1
Fzd1
Gstm2
Pkia
Illumina
Ion Torrent
Hippocampal region
CA1
CA3
DG

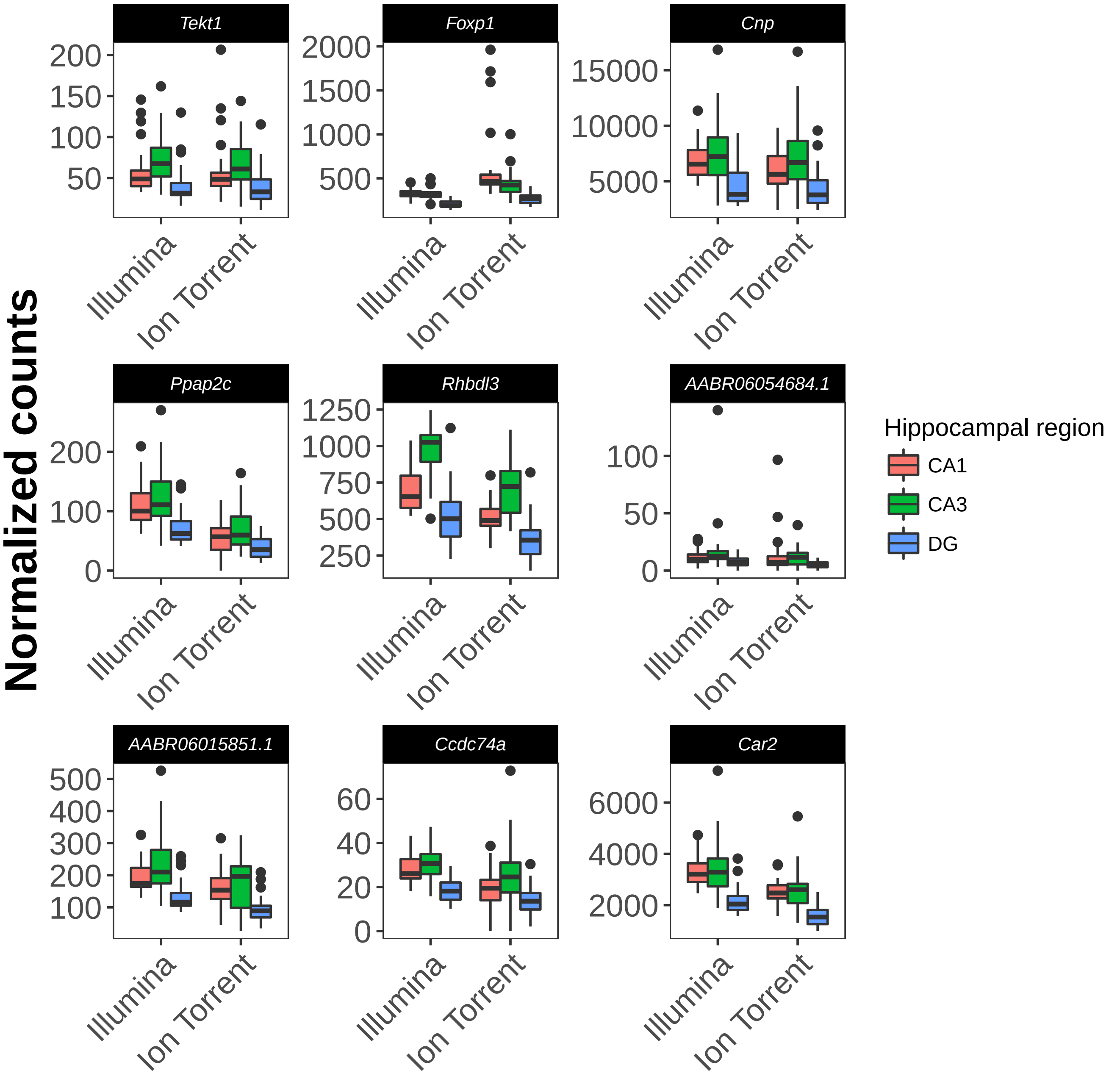

Normalized counts
Tekt1
Foxp1
Cnp
Ppap2c
Rhbdl3
AABR06054684.1
AABR06015851.1
Ccdc74a
Car2
Illumina
Ion Torrent
Hippocampal region
CA1
CA3
DG

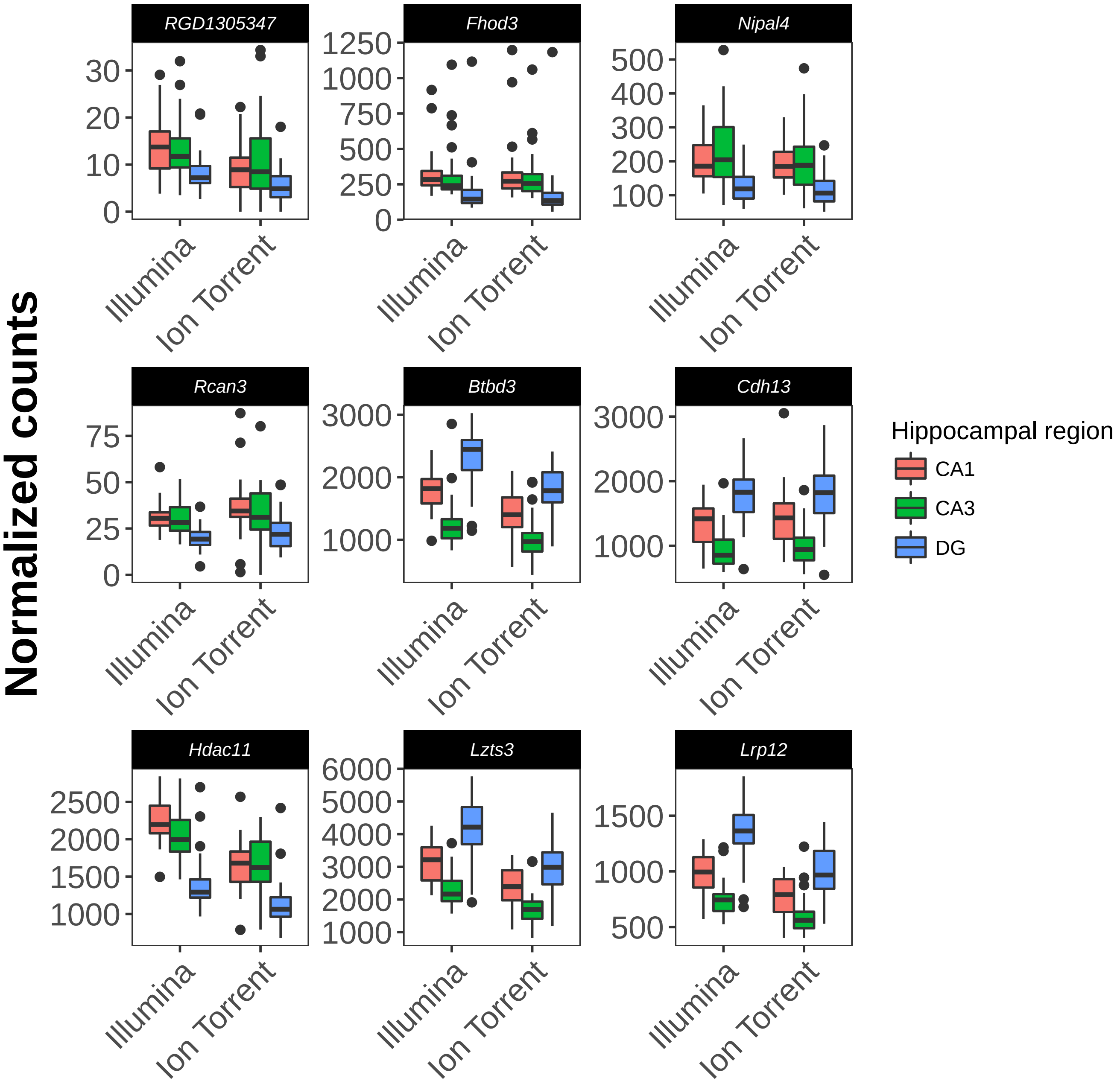
RGD1305347
Fhod3
Nipal4
Rcan3
Btbd3
Cdh13
Hdac11
Lzts3
Lrp12
Illumina
Ion Torrent
Normalized counts
Hippocampal region
CA1
CA3
DG

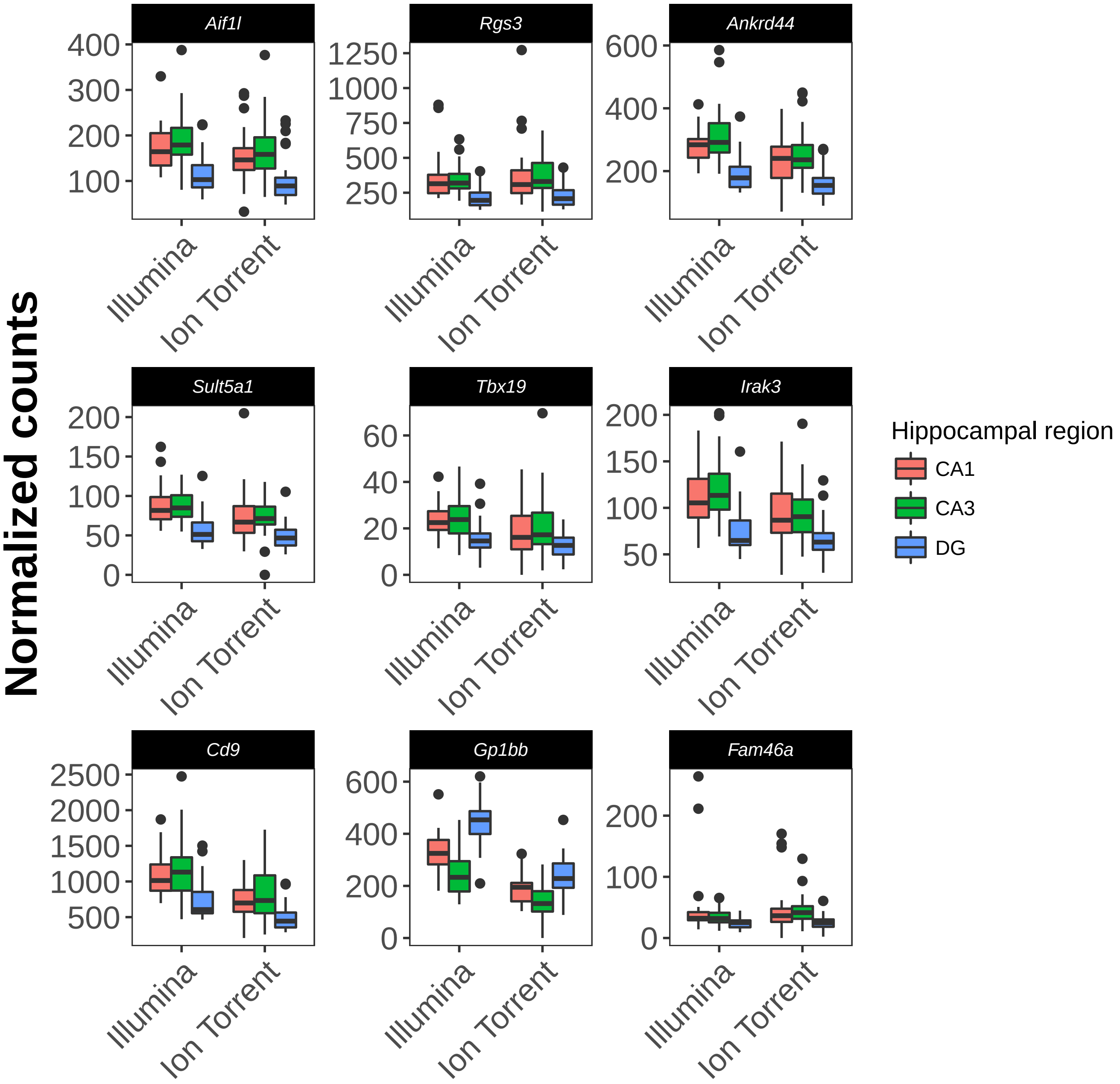
Normalized counts
Aif1l
Rgs3
Ankrd44
Sult5a1
Tbx19
Irak3
Cd9
Gp1bb
Fam46a
Illumina
Ion Torrent
Hippocampal region
CA1
CA3
DG

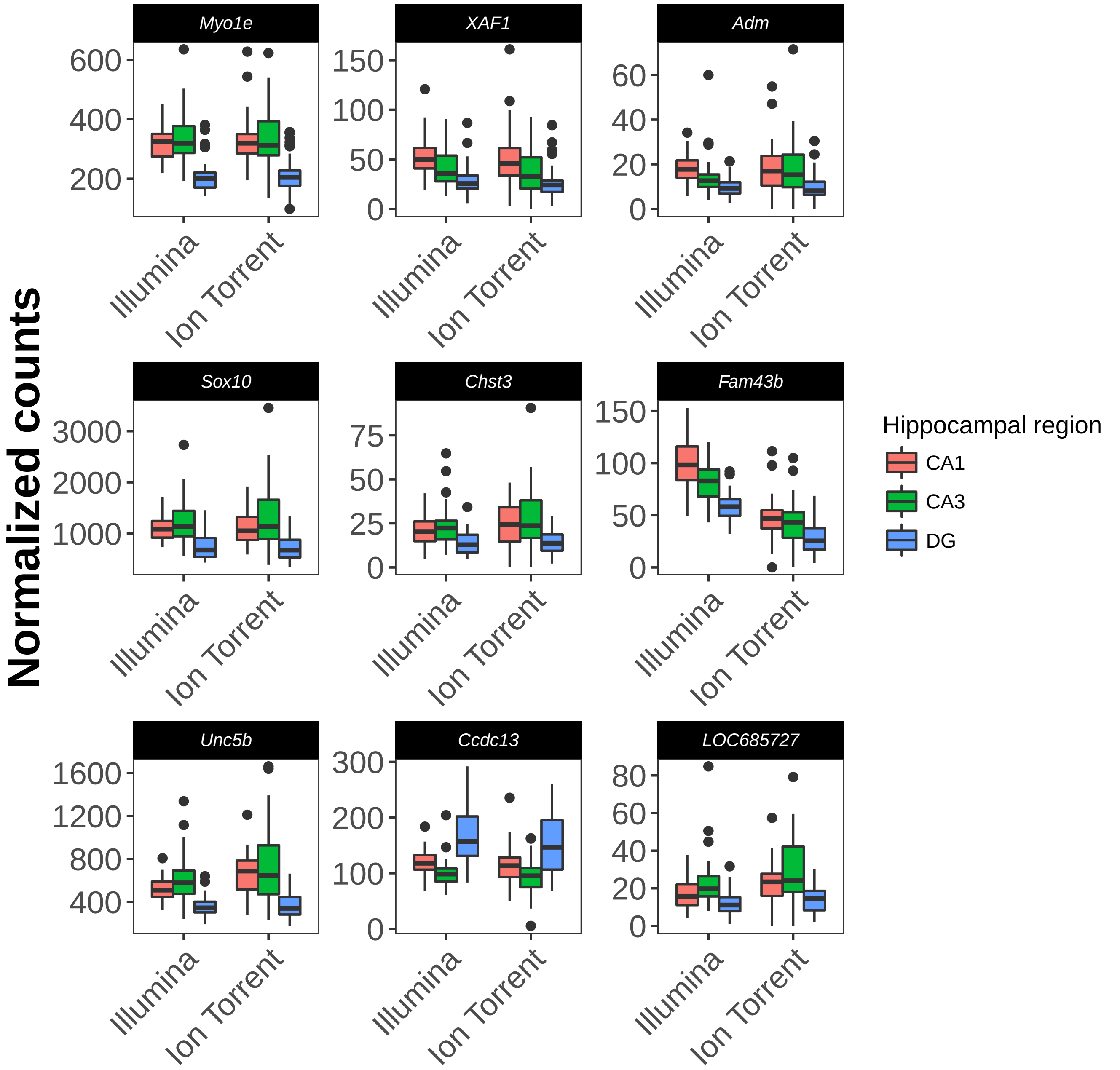

Normalized counts
Myo1e
XAF1
Adm
Sox10
Chst3
Fam43b
Unc5b
Ccdc13
LOC685727
Illumina
Ion Torrent
Hippocampal region
CA1
CA3
DG

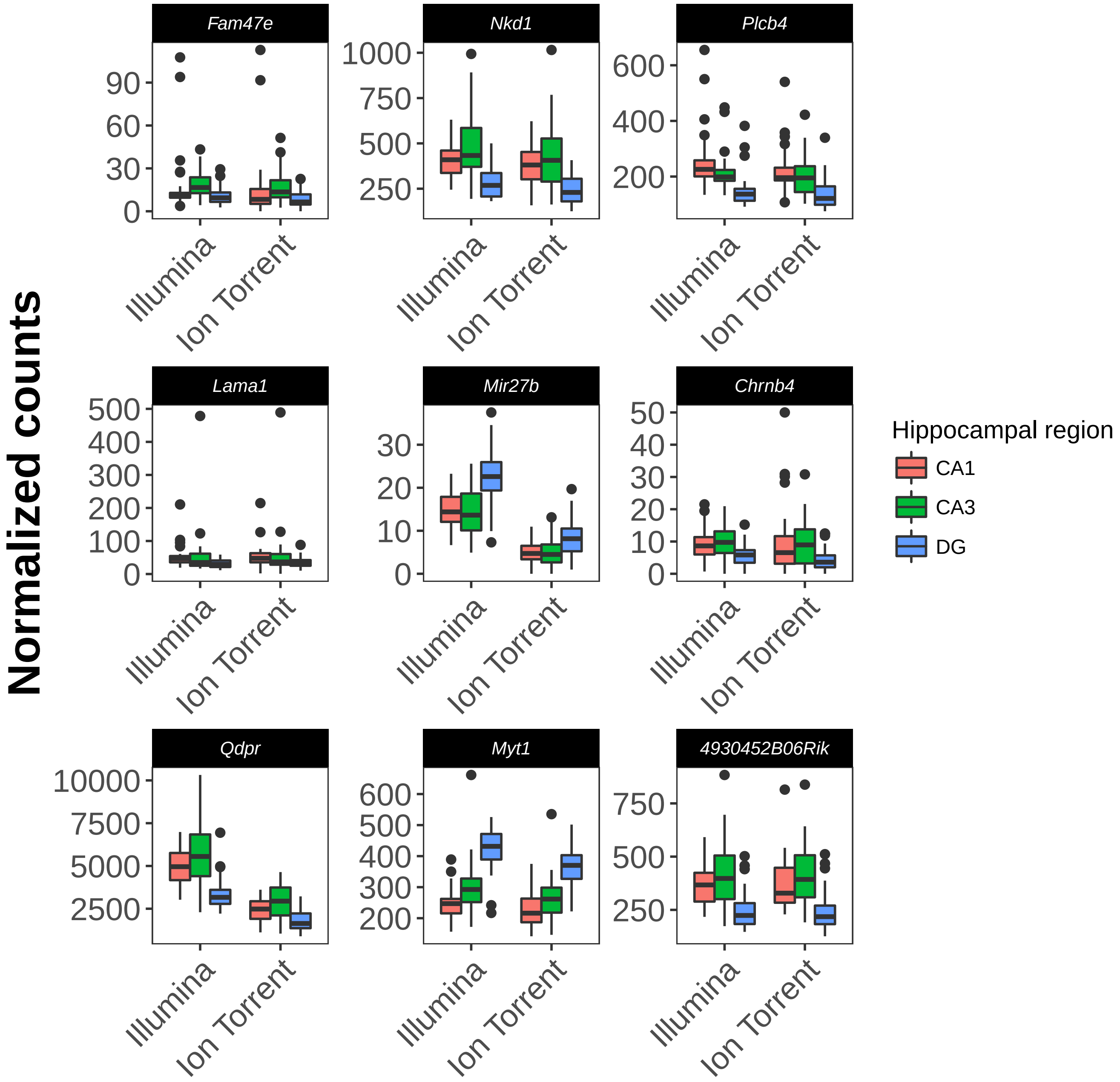
Normalized counts
Fam47e
Nkd1
Plcb4
Lama1
Mir27b
Chrnb4
Qdpr
Myt1
4930452B06Rik
Illumina
Ion Torrent
Hippocampal region
CA1
CA3
DG

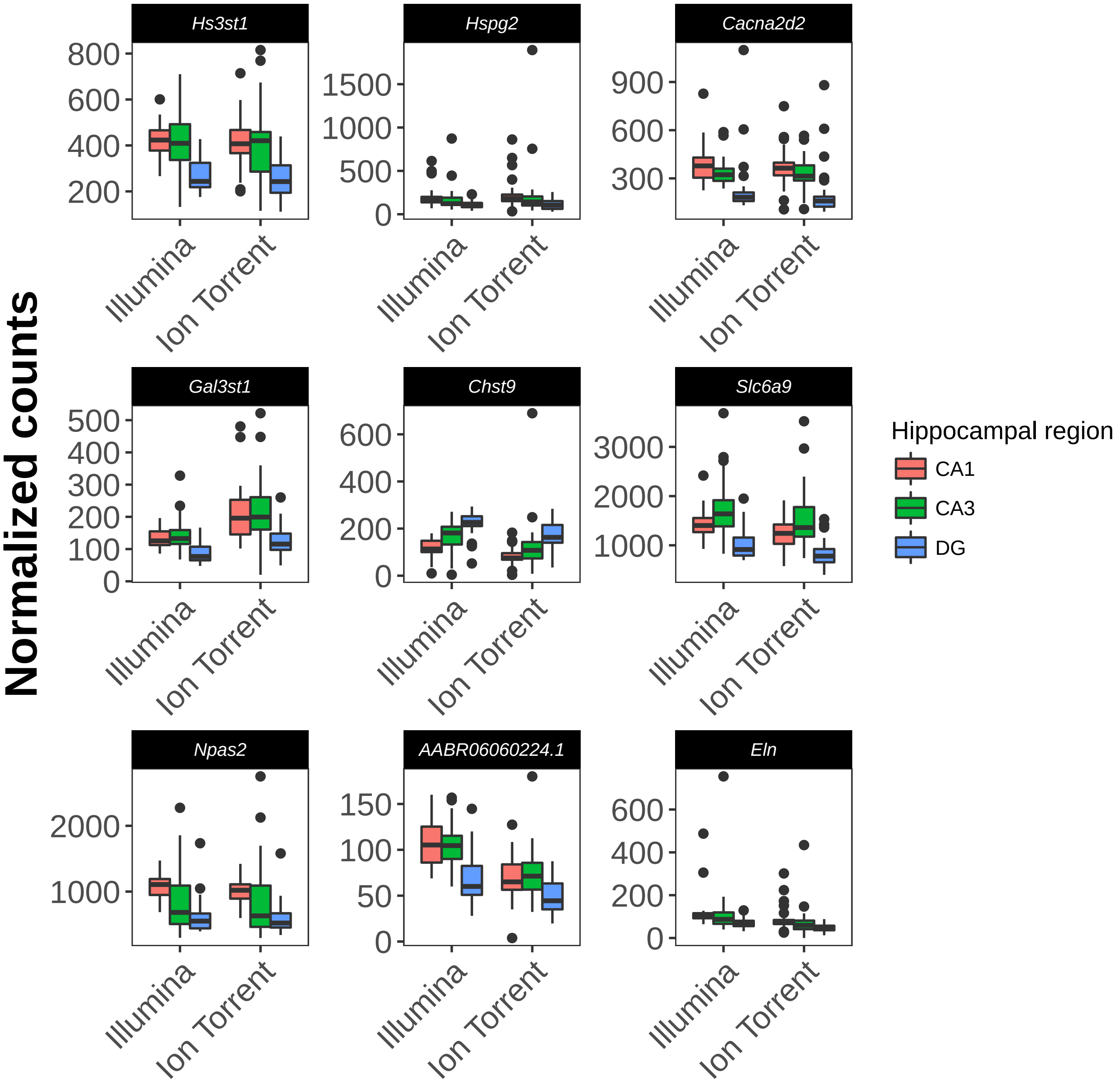
Normalized counts
Hs3st1
Hspg2
Cacna2d2
Gal3st1
Chst9
Slc6a9
Npas2
AABR06060224.1
Eln
Illumina
Ion Torrent
Hippocampal region
CA1
CA3
DG

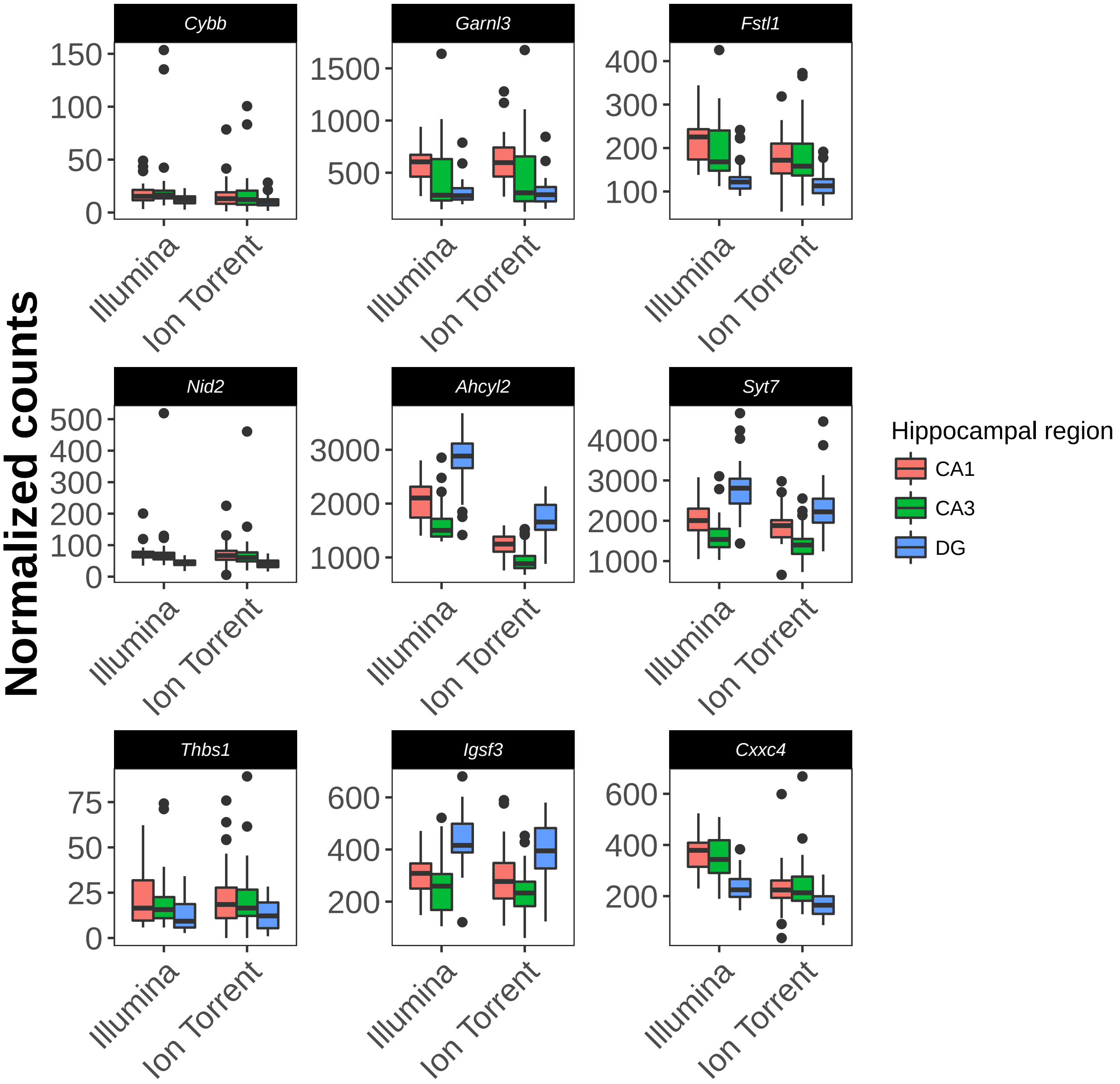
Normalized counts
Cybb
Garnl3
Fstl1
Nid2
Ahcyl2
Syt7
Thbs1
Igsf3
Cxxc4
Illumina
Ion Torrent
Hippocampal region
CA1
CA3
DG

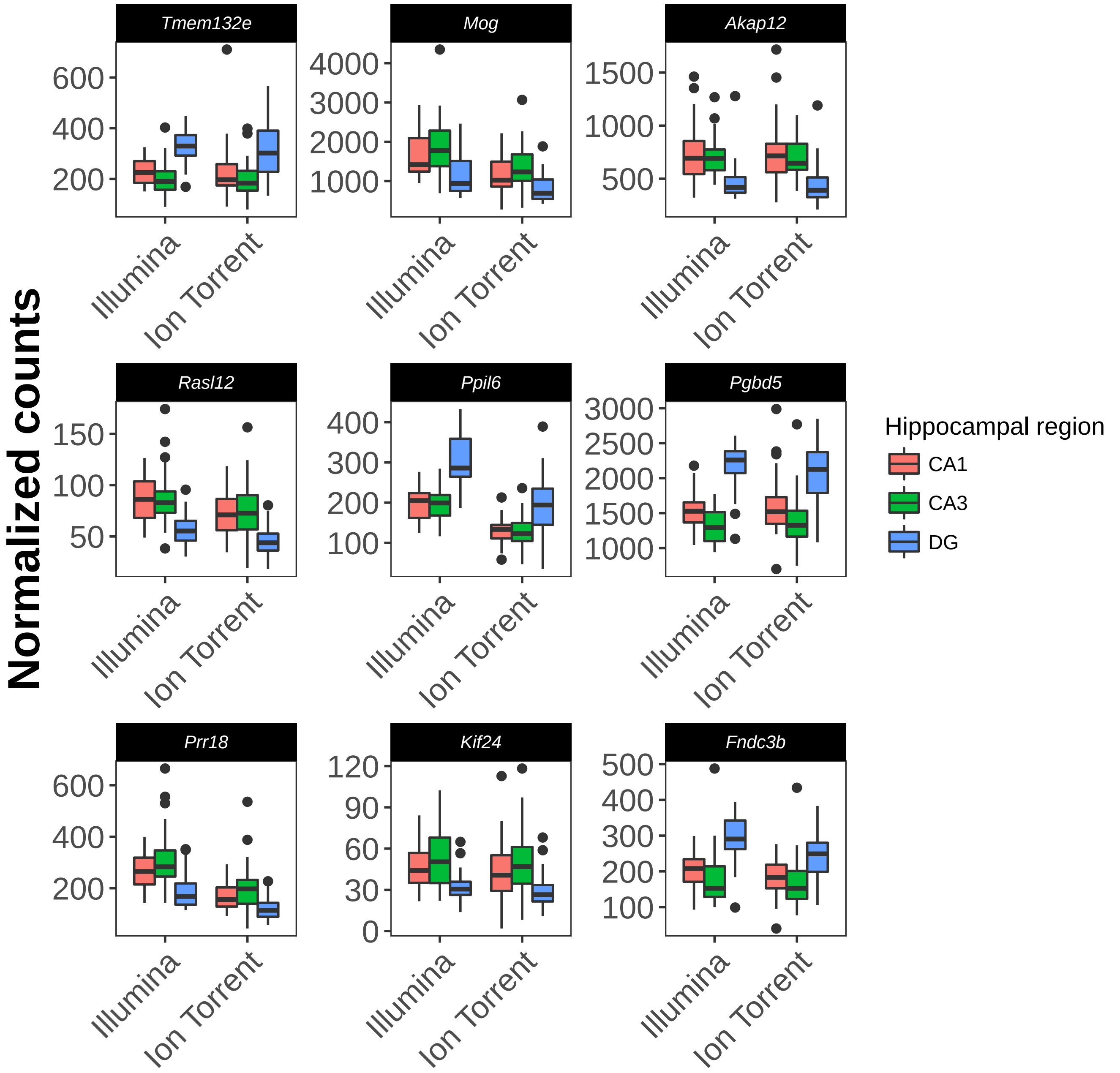
Normalized counts
Tmem132e
Mog
Akap12
Rasl12
Ppil6
Pgbd5
Prr18
Kif24
Fndc3b
Illumina
Ion Torrent
Hippocampal region
CA1
CA3
DG

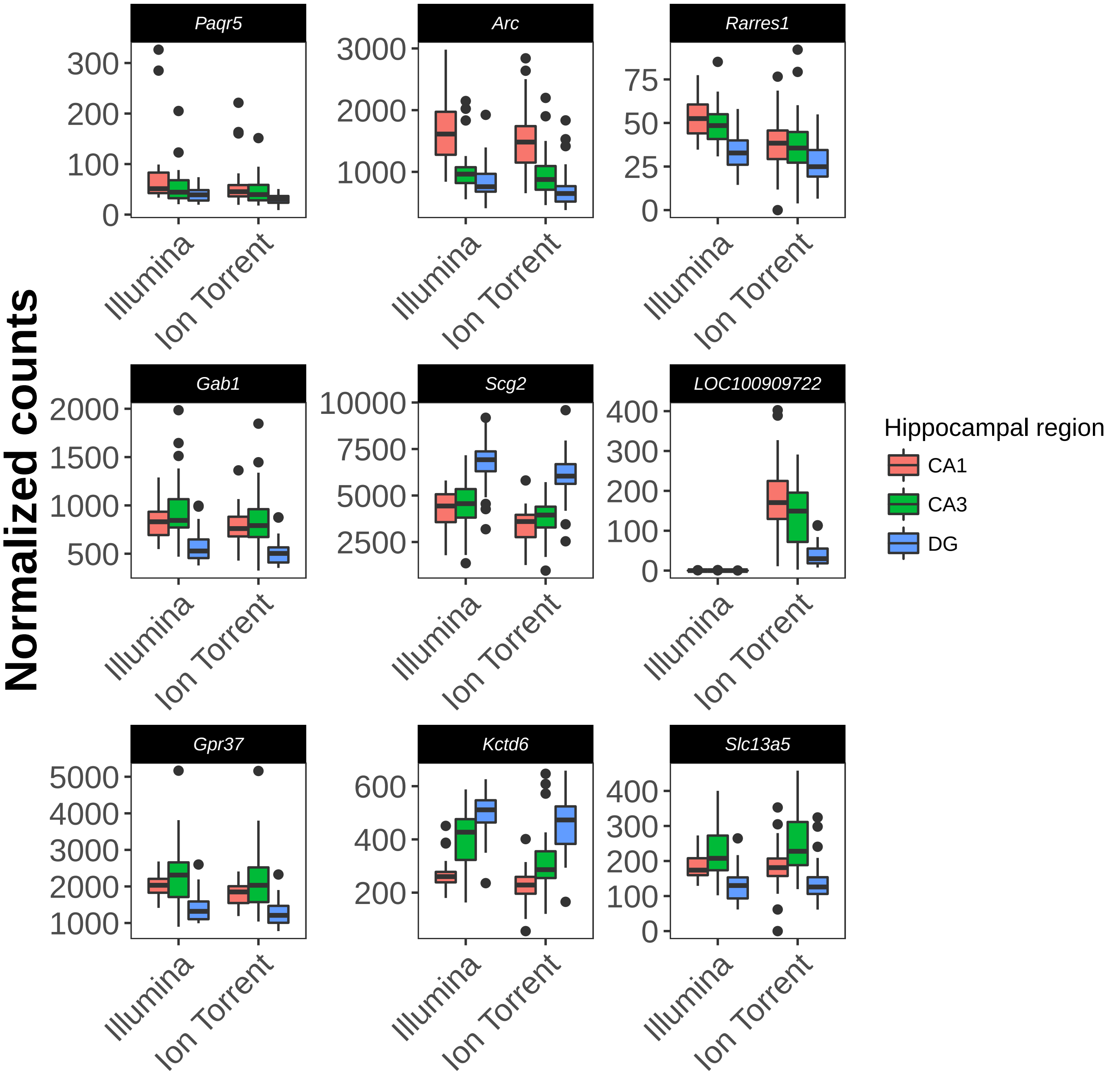

Normalized counts
Paqr5
Arc
Rarres1
Gab1
Scg2
LOC100909722
Gpr37
Kctd6
Slc13a5
Illumina
Ion Torrent
Hippocampal region
CA1
CA3
DG

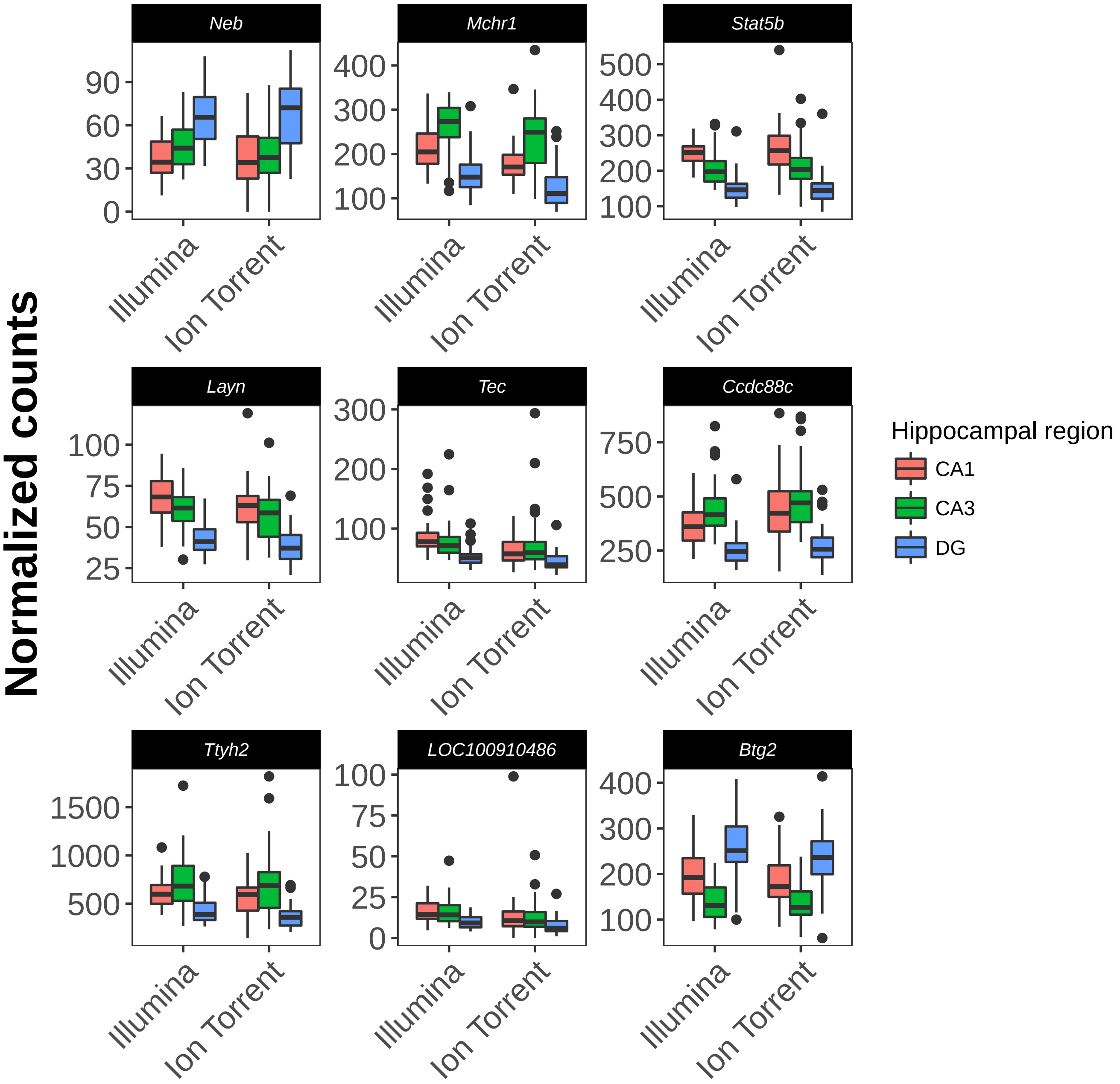
Normalized counts
Neb
Mchr1
Stat5b
Layn
Tec
Ccdc88c
Ttyh2
LOC100910486
Btg2
Illumina
Ion Torrent
Hippocampal region
CA1
CA3
DG

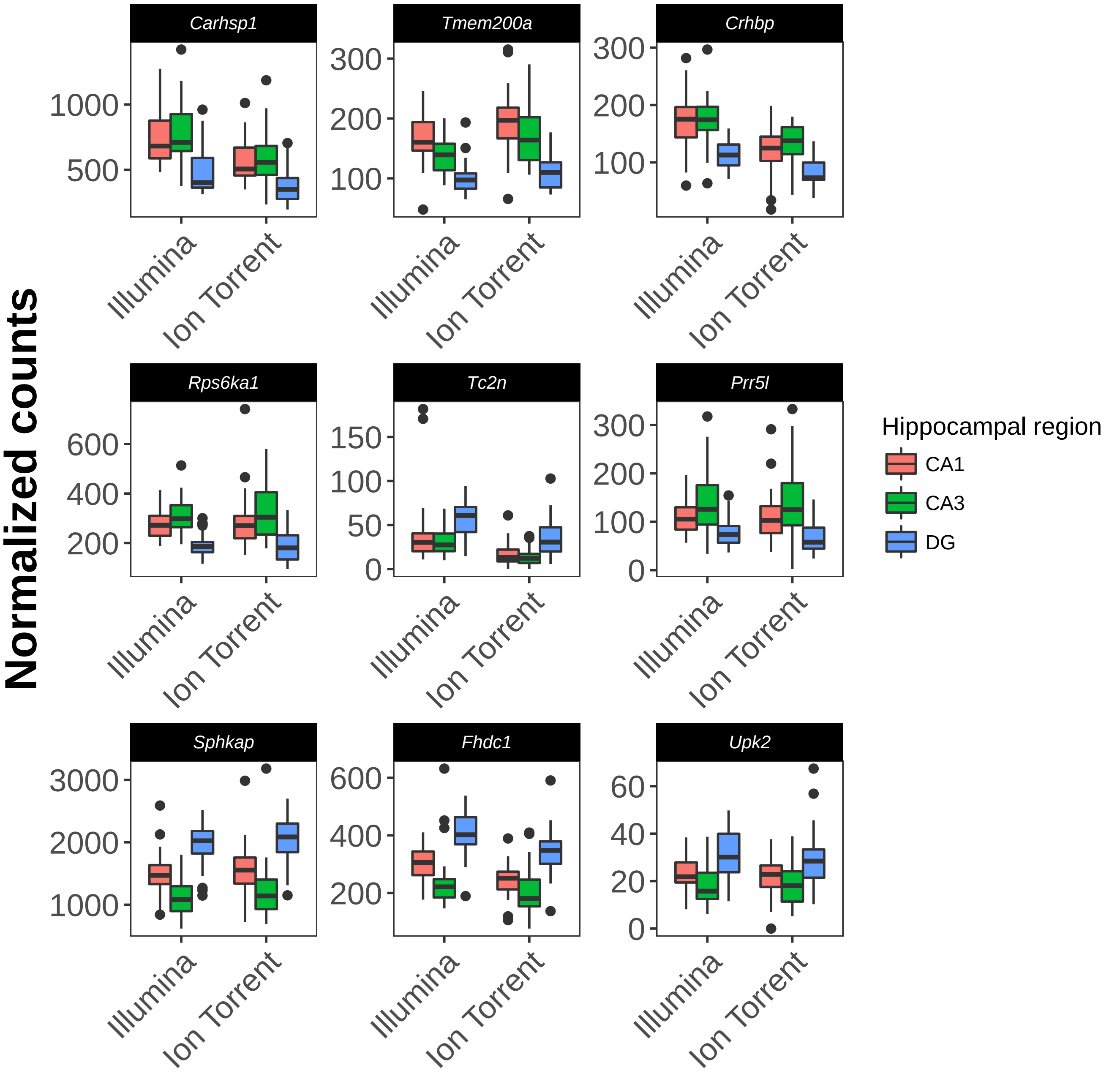

Normalized counts
Carhsp1
Tmem200a
Crhbp
Rps6ka1
Tc2n
Prr5l
Sphkap
Fhdc1
Upk2
Illumina
Ion Torrent
Hippocampal region
CA1
CA3
DG

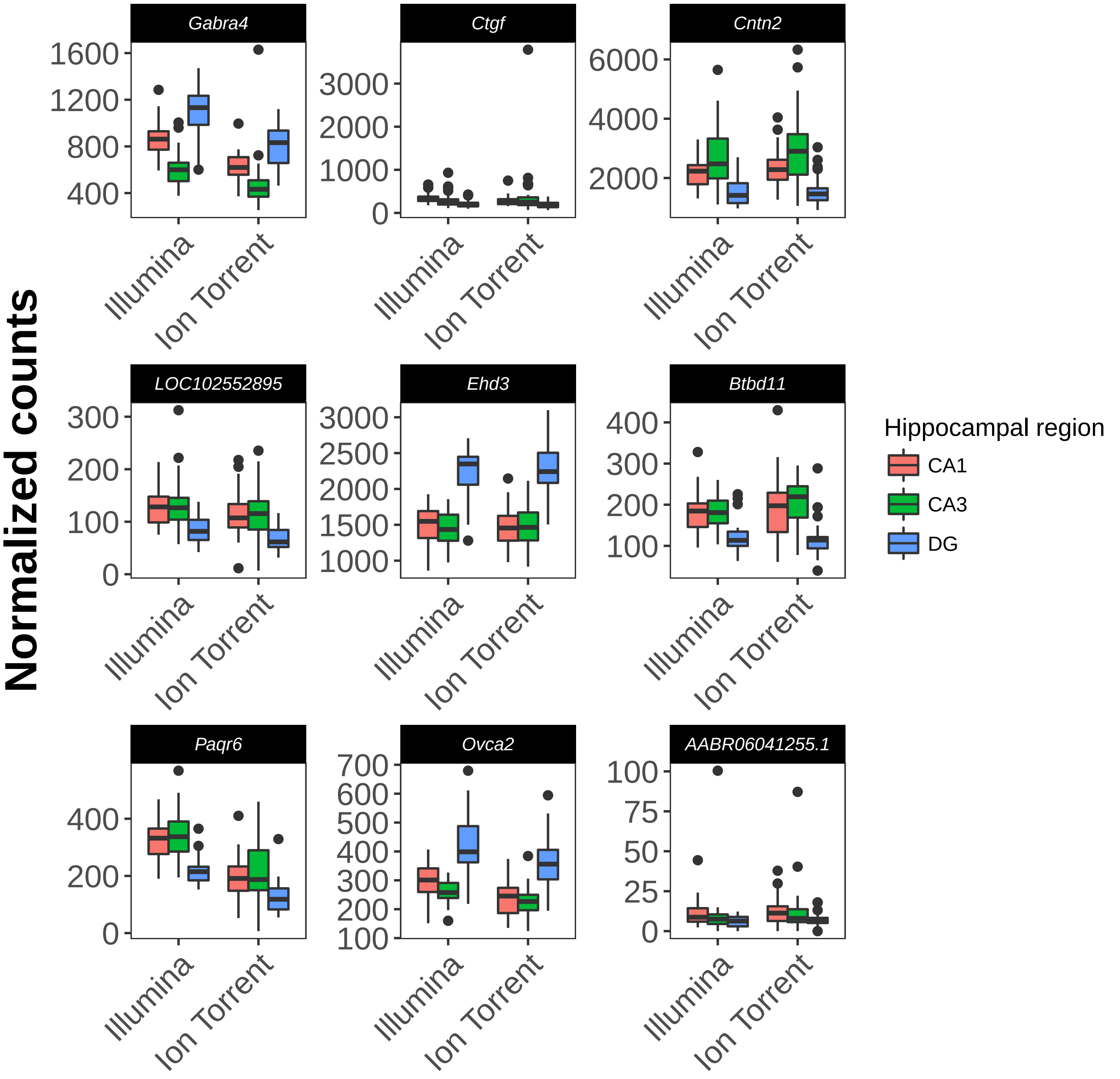

Normalized counts
Gabra4
Ctgf
Cntn2
LOC102552895
Ehd3
Btbd11
Paqr6
Ovca2
AABR06041255.1
Illumina
Ion Torrent
Hippocampal region
CA1
CA3
DG

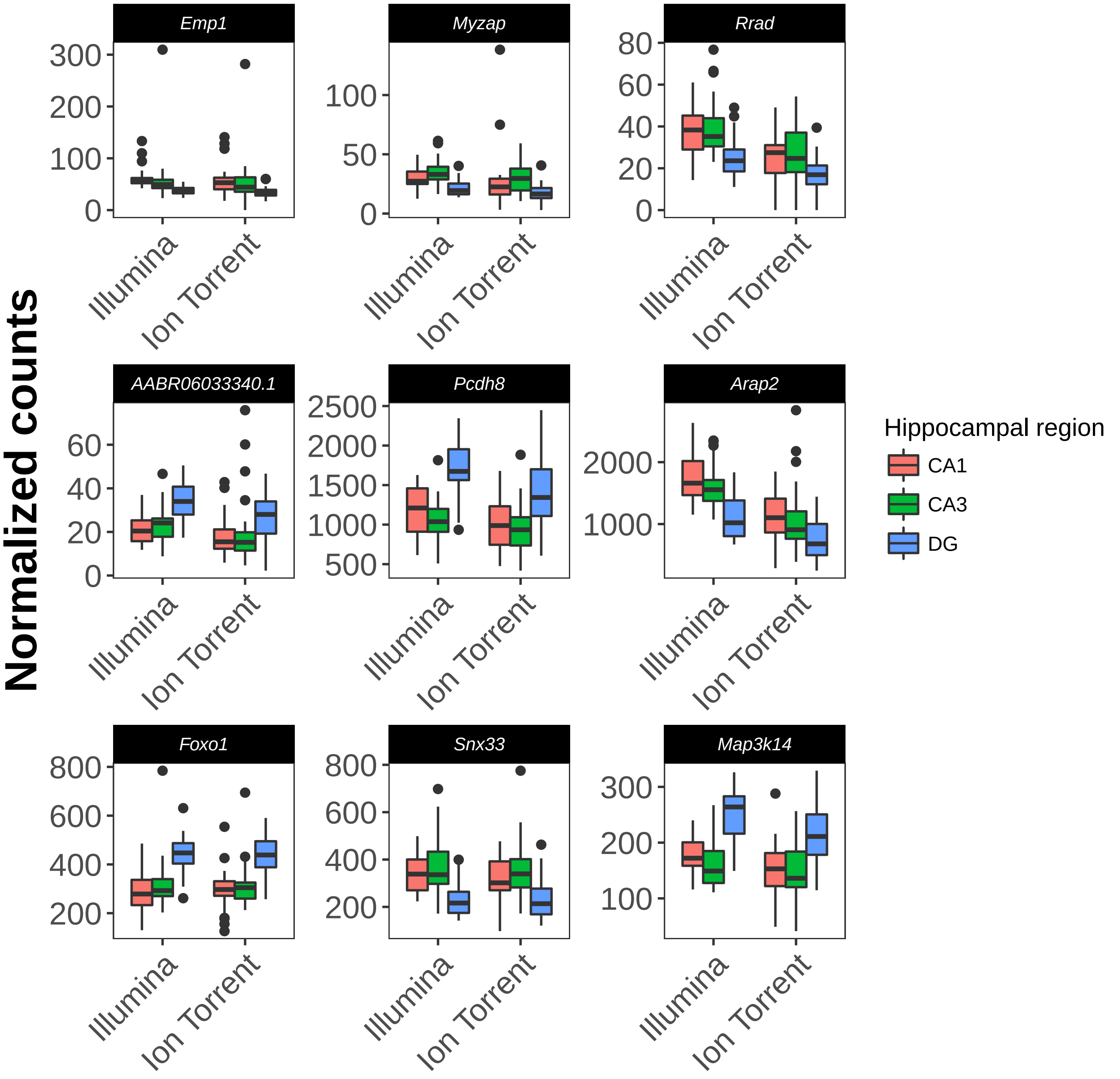
Normalized counts
Emp1
Myzap
Rrad
AABR06033340.1
Pcdh8
Arap2
Foxo1
Snx33
Map3k14
Illumina
Ion Torrent
Hippocampal region
CA1
CA3
DG

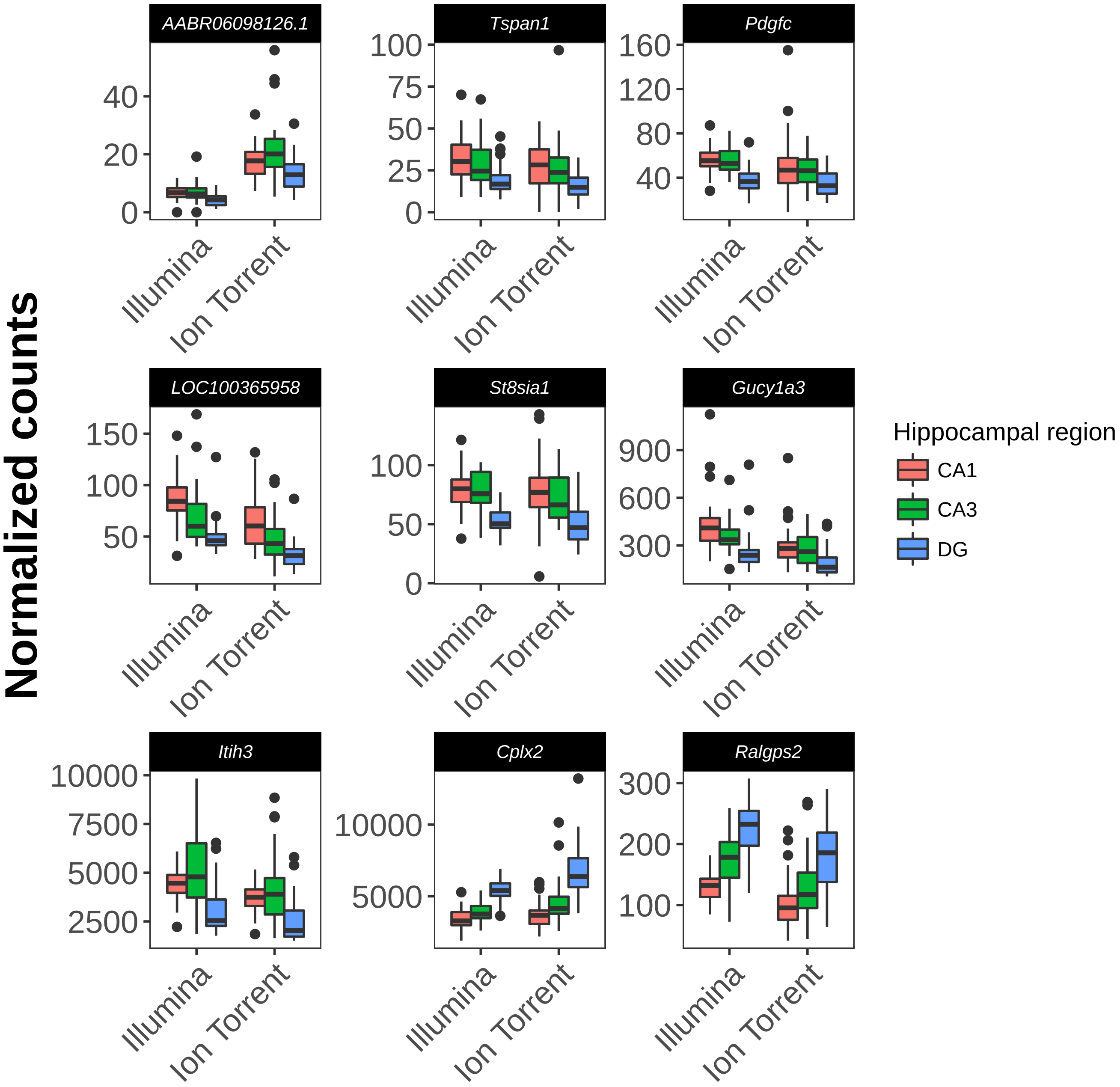

Normalized counts
AABR06098126.1
Tspan1
Pdgfc
LOC100365958
St8sia1
Gucy1a3
Itih3
Cplx2
Ralgps2
Illumina
Ion Torrent
Hippocampal region
CA1
CA3
DG

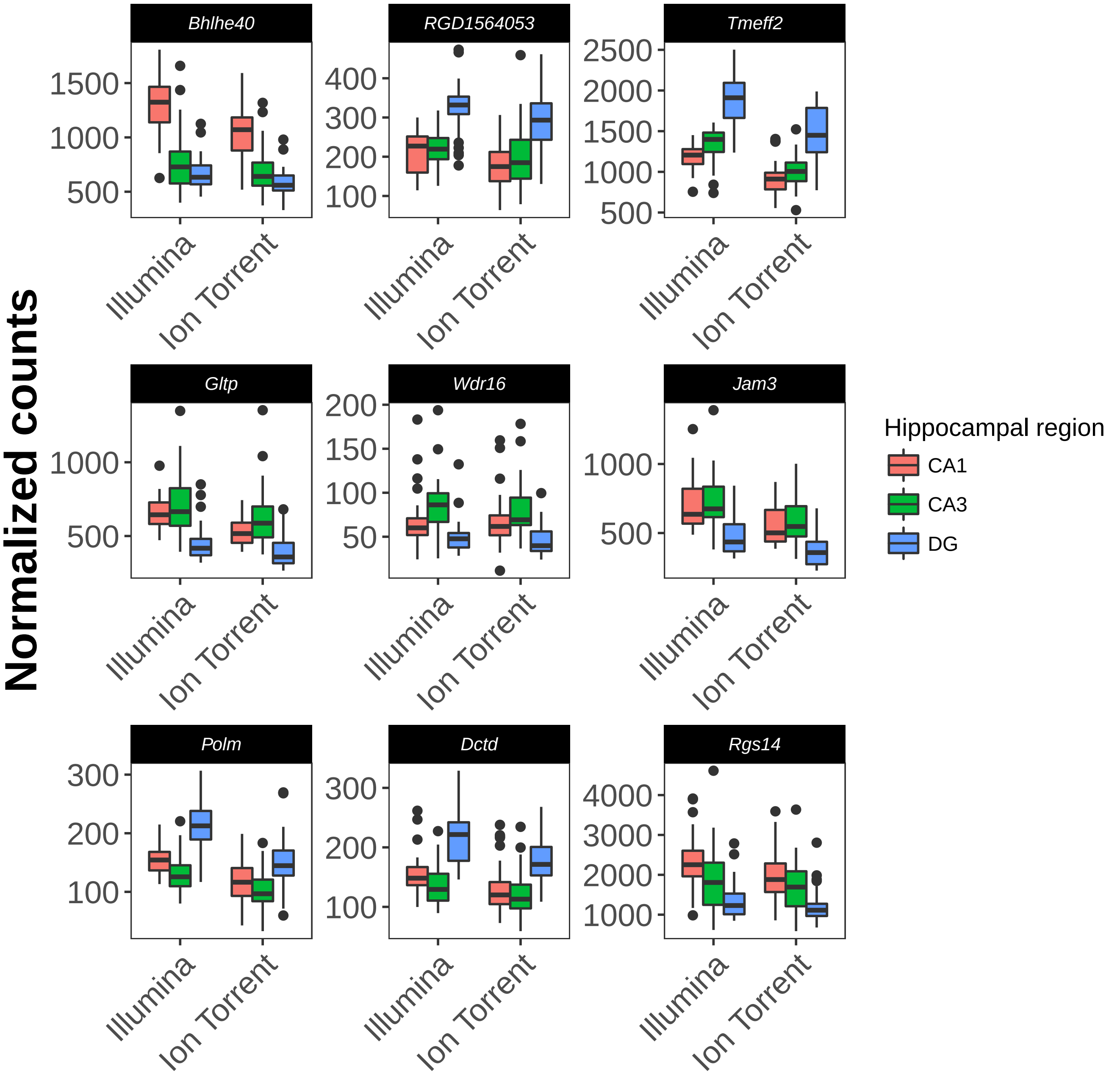
Normalized counts
Bhlhe40
RGD1564053
Tmeff2
Gltp
Wdr16
Jam3
Polm
Dctd
Rgs14
Illumina
Ion Torrent
Hippocampal region
CA1
CA3
DG

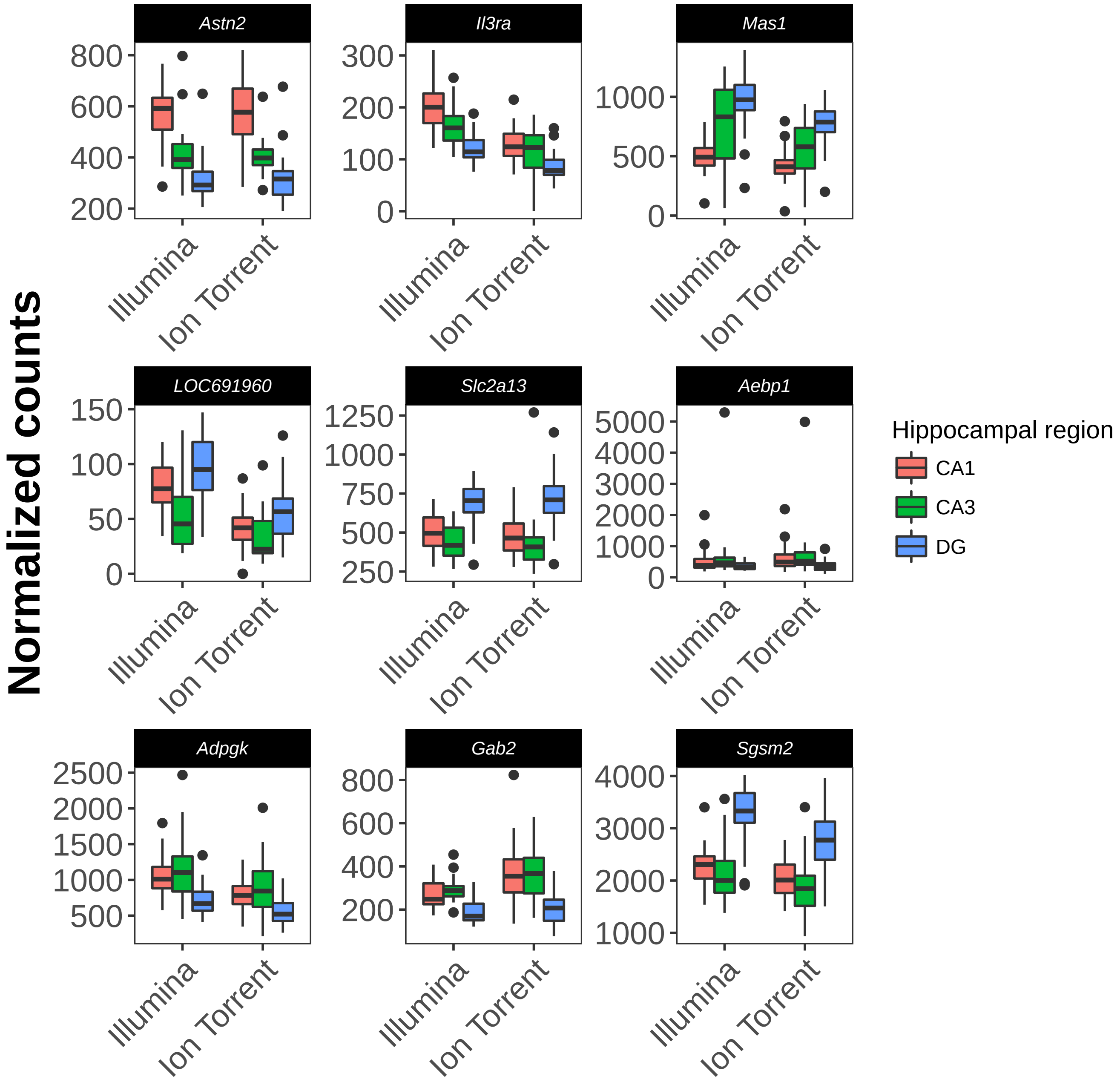

Normalized counts
Astn2
Il3ra
Mas1
LOC691960
Slc2a13
Aebp1
Adpgk
Gab2
Sgsm2
Illumina
Ion Torrent
Hippocampal region
CA1
CA3
DG

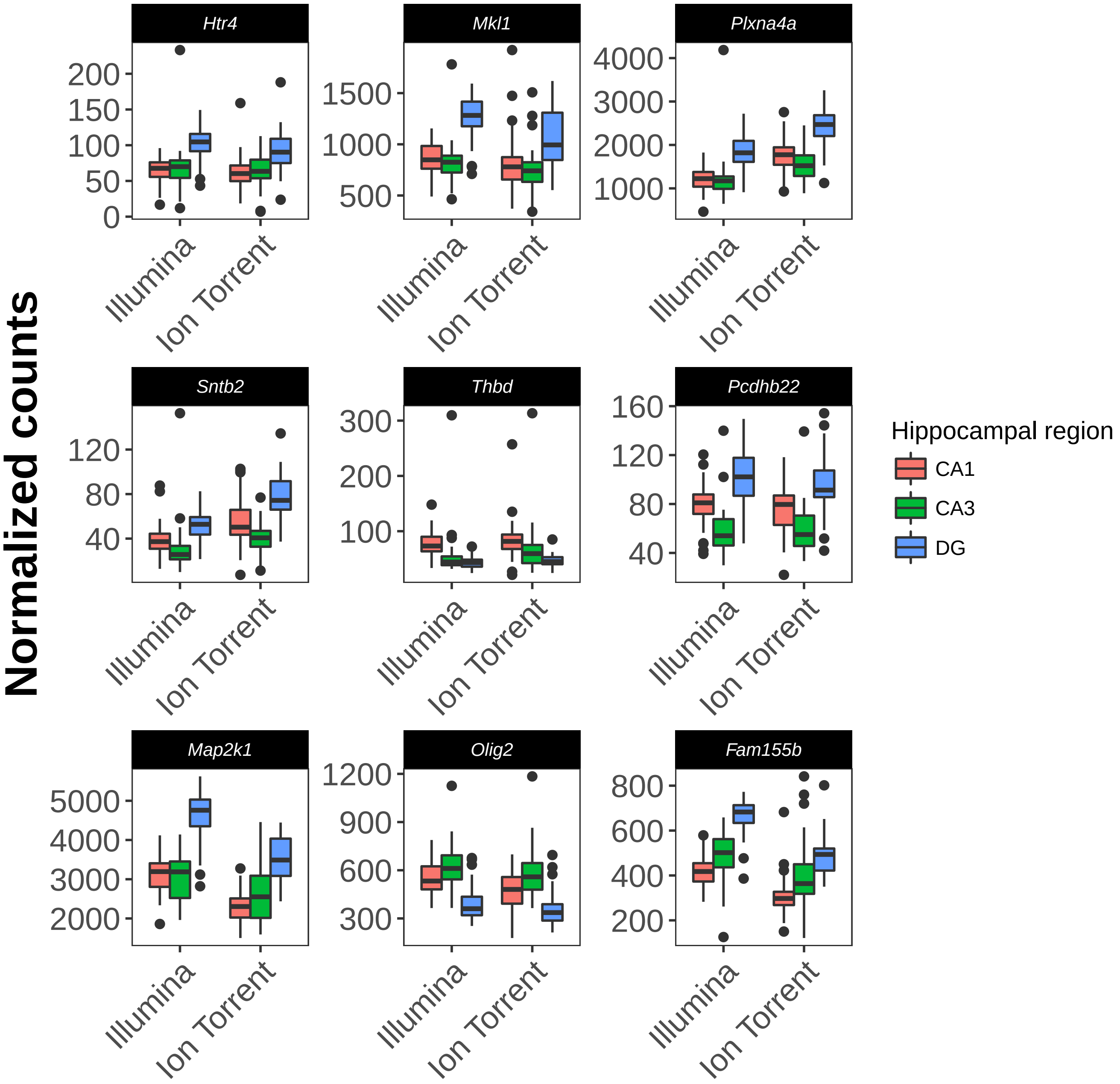
Normalized counts
Htr4
Mkl1
Plxna4a
Sntb2
Thbd
Pcdhb22
Map2k1
Olig2
Fam155b
Illumina
Ion Torrent
Hippocampal region
CA1
CA3
DG

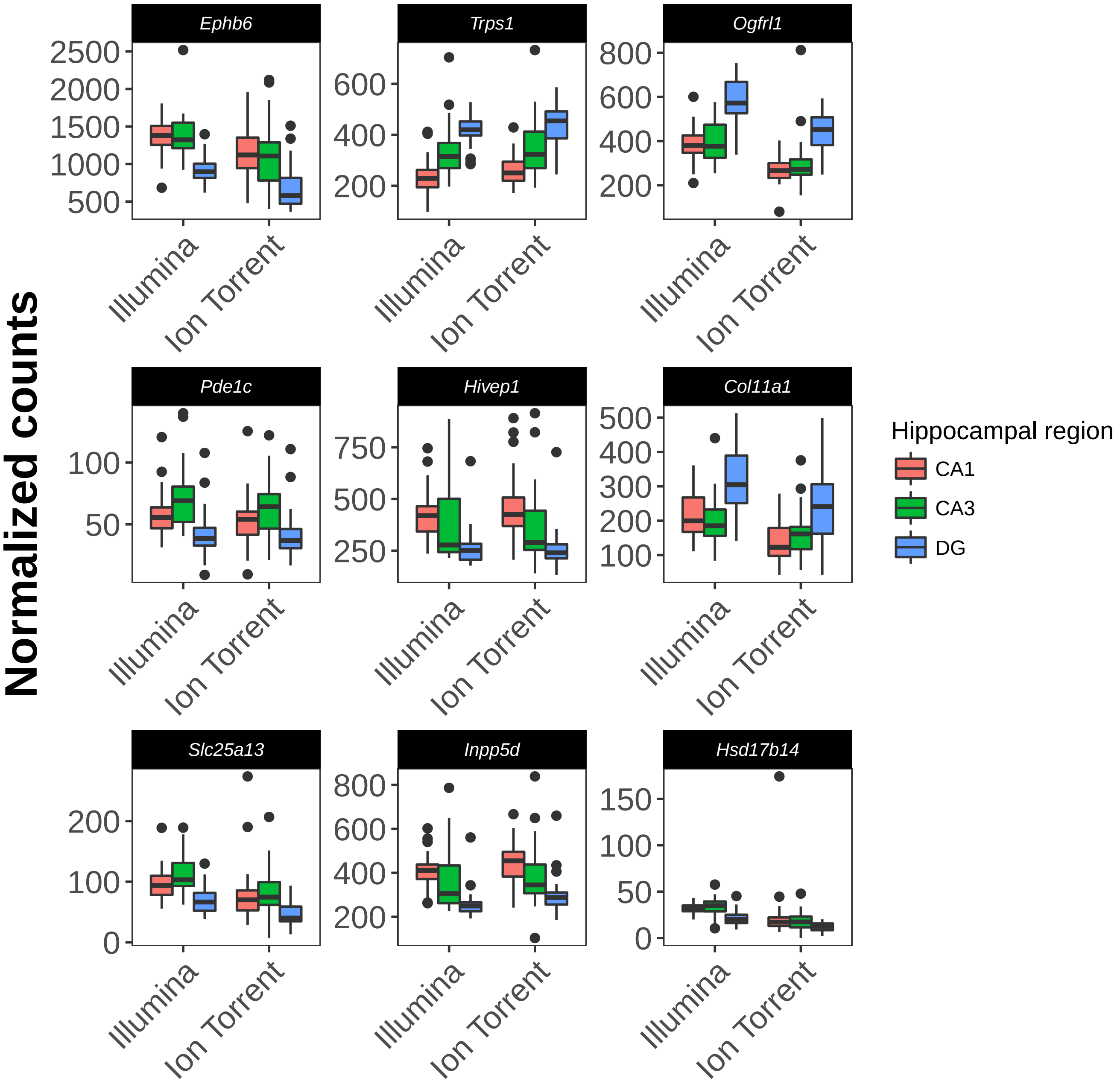
Normalized counts
Ephb6
Trps1
Ogfrl1
Pde1c
Hivep1
Col11a1
Slc25a13
Inpp5d
Hsd17b14
Illumina
Ion Torrent
Hippocampal region
CA1
CA3
DG

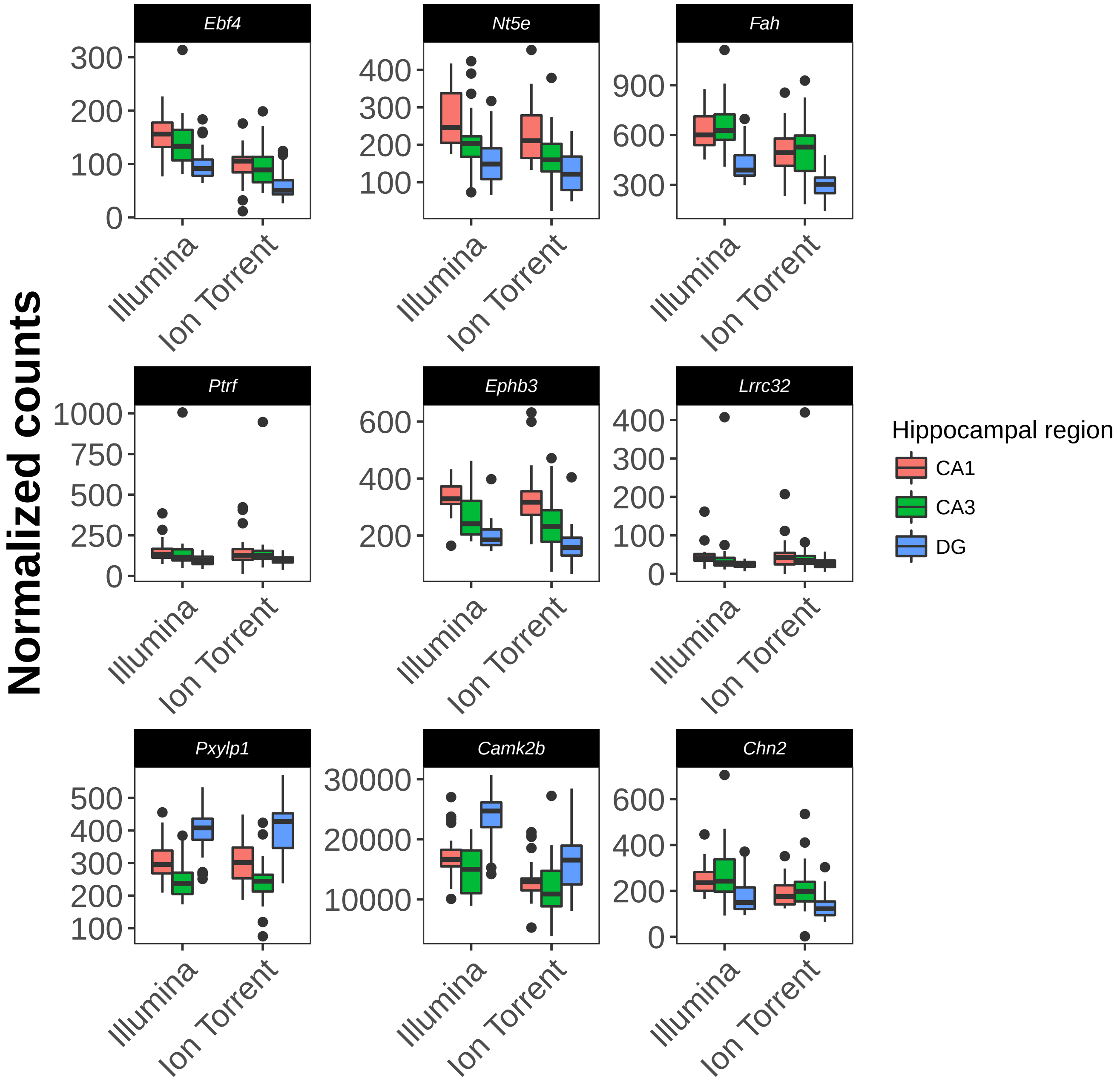
Normalized counts
Ebf4
Nt5e
Fah
Ptrf
Ephb3
Lrrc32
Pxylp1
Camk2b
Chn2
Illumina
Ion Torrent
Hippocampal region
CA1
CA3
DG

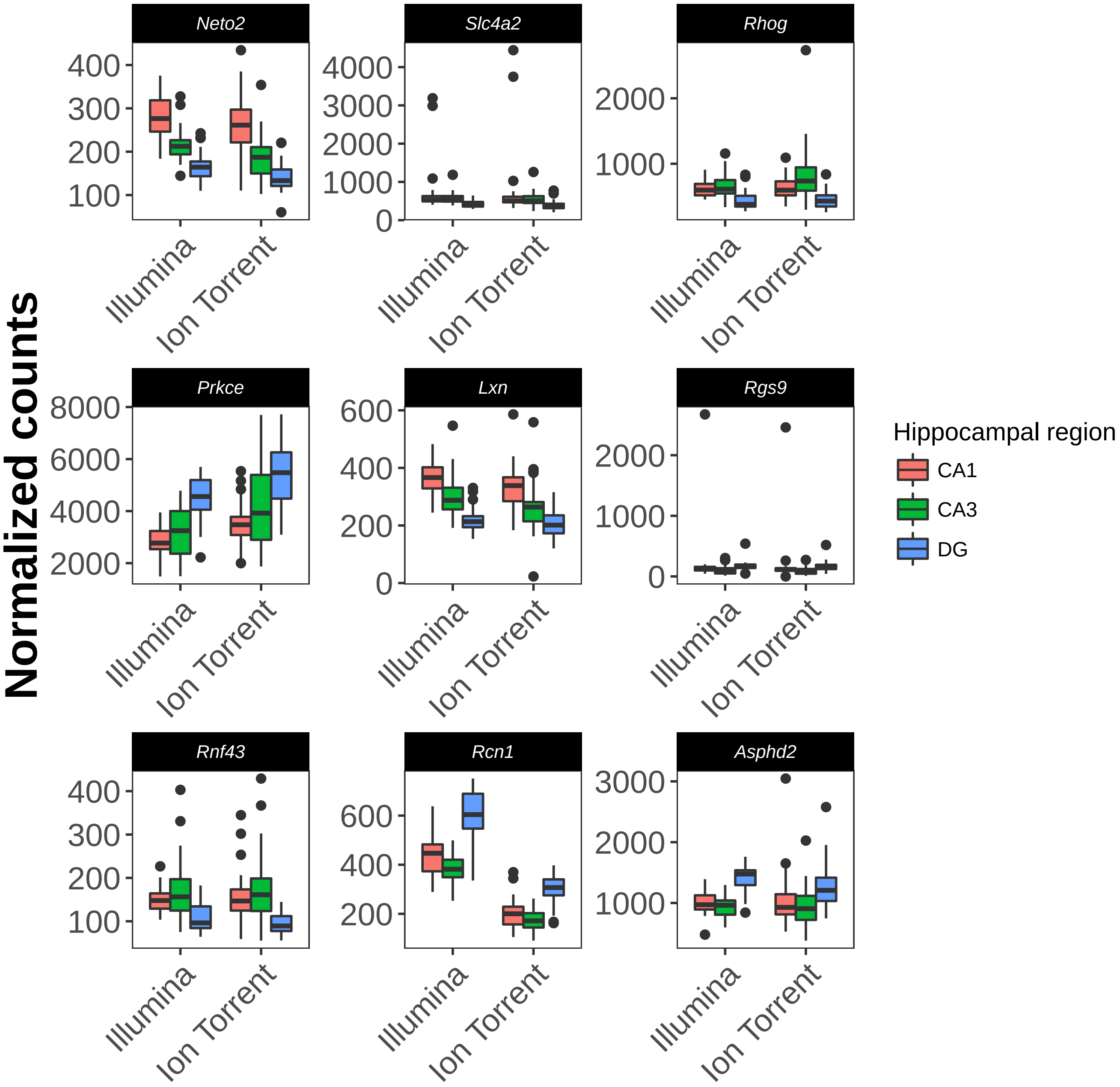

Normalized counts
Neto2
Slc4a2
Rhog
Prkce
Lxn
Rgs9
Rnf43
Rcn1
Asphd2
Illumina
Ion Torrent
Hippocampal region
CA1
CA3
DG

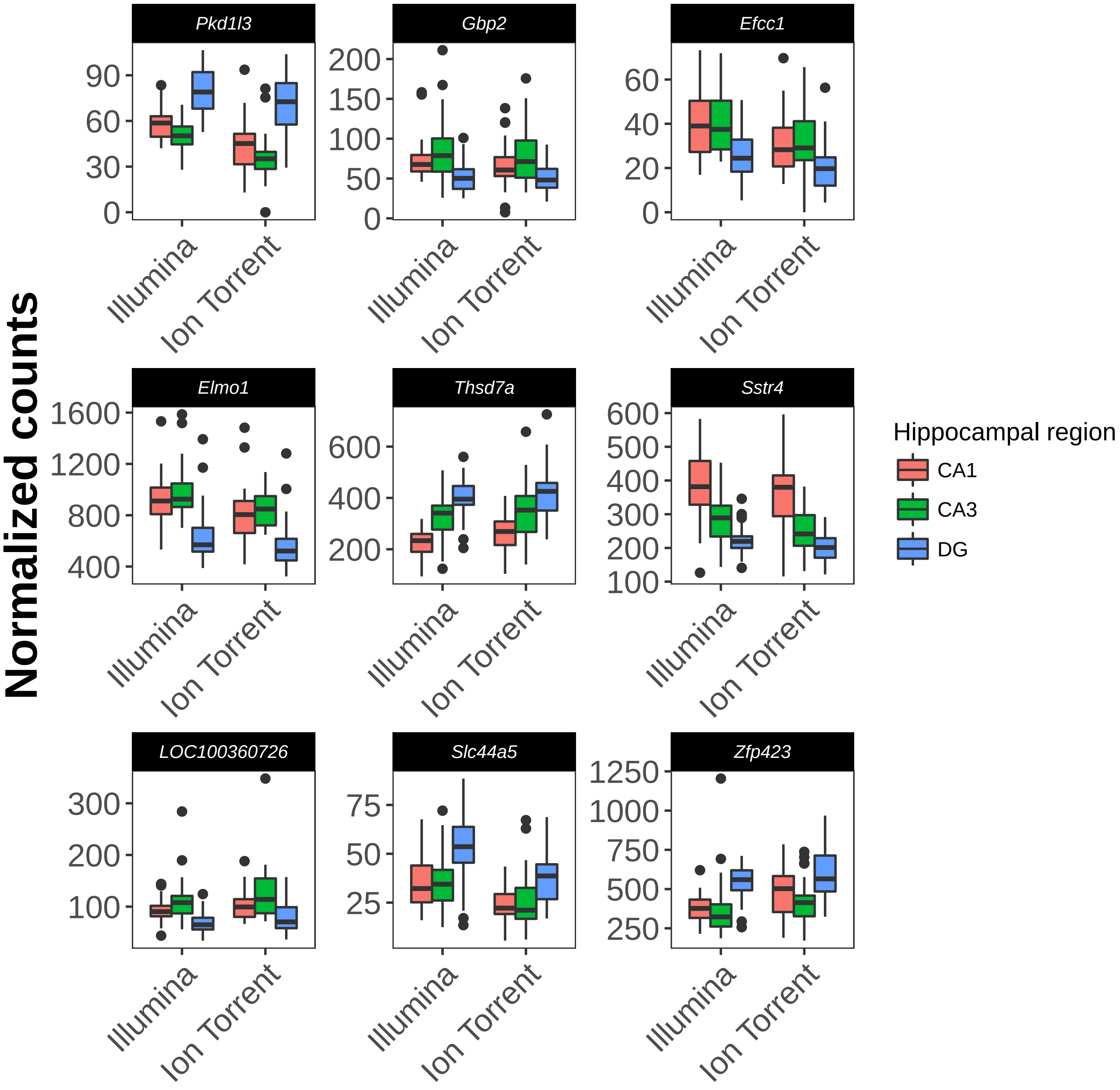

Normalized counts
Pkd1l3
Gbp2
Efcc1
Elmo1
Thsd7a
Sstr4
LOC100360726
Slc44a5
Zfp423
Illumina
Ion Torrent
Hippocampal region
CA1
CA3
DG

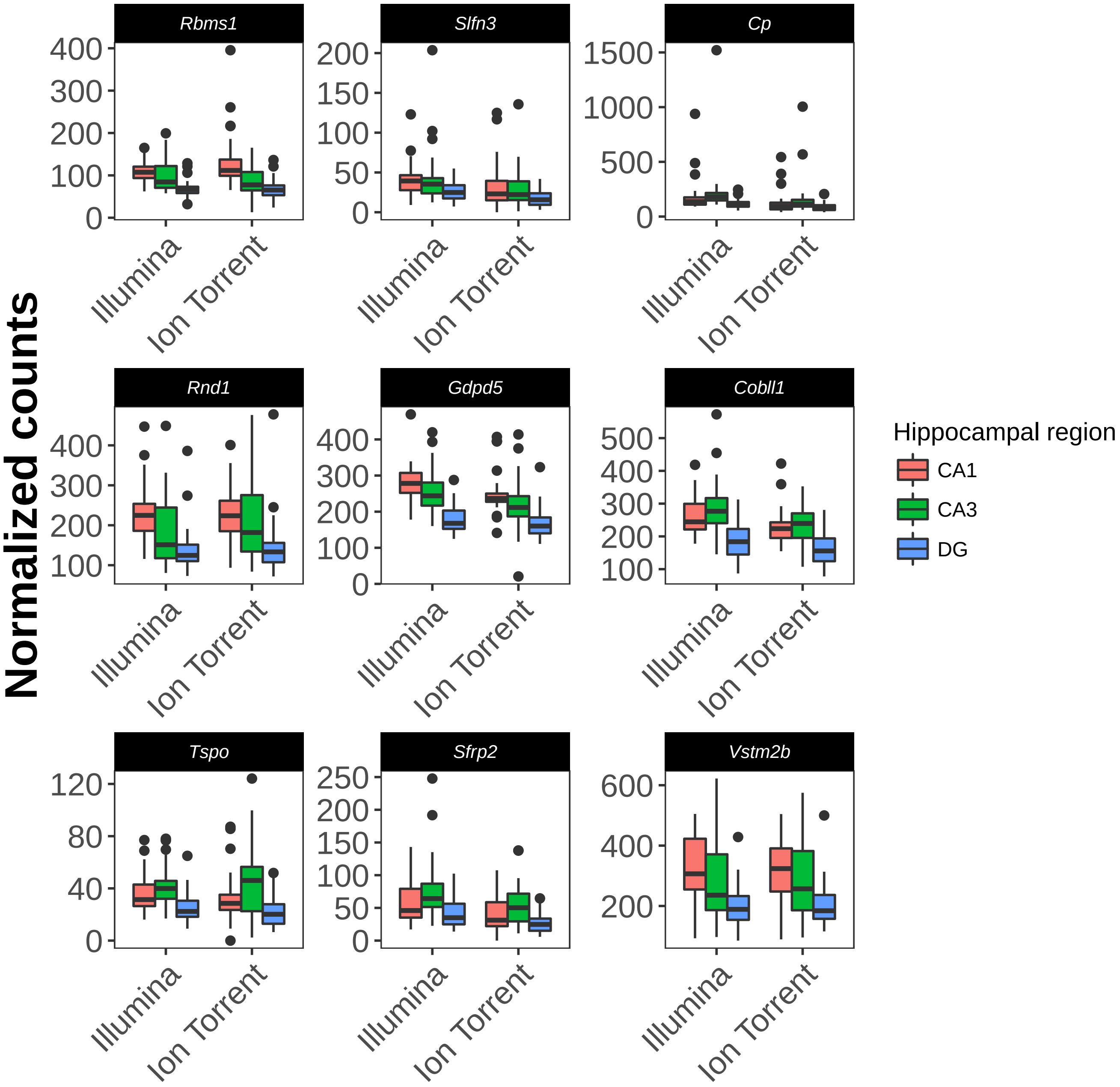

Normalized counts
Rbms1
Slfn3
Cp
Rnd1
Gdpd5
Cobll1
Tspo
Sfrp2
Vstm2b
Illumina
Ion Torrent
Hippocampal region
CA1
CA3
DG

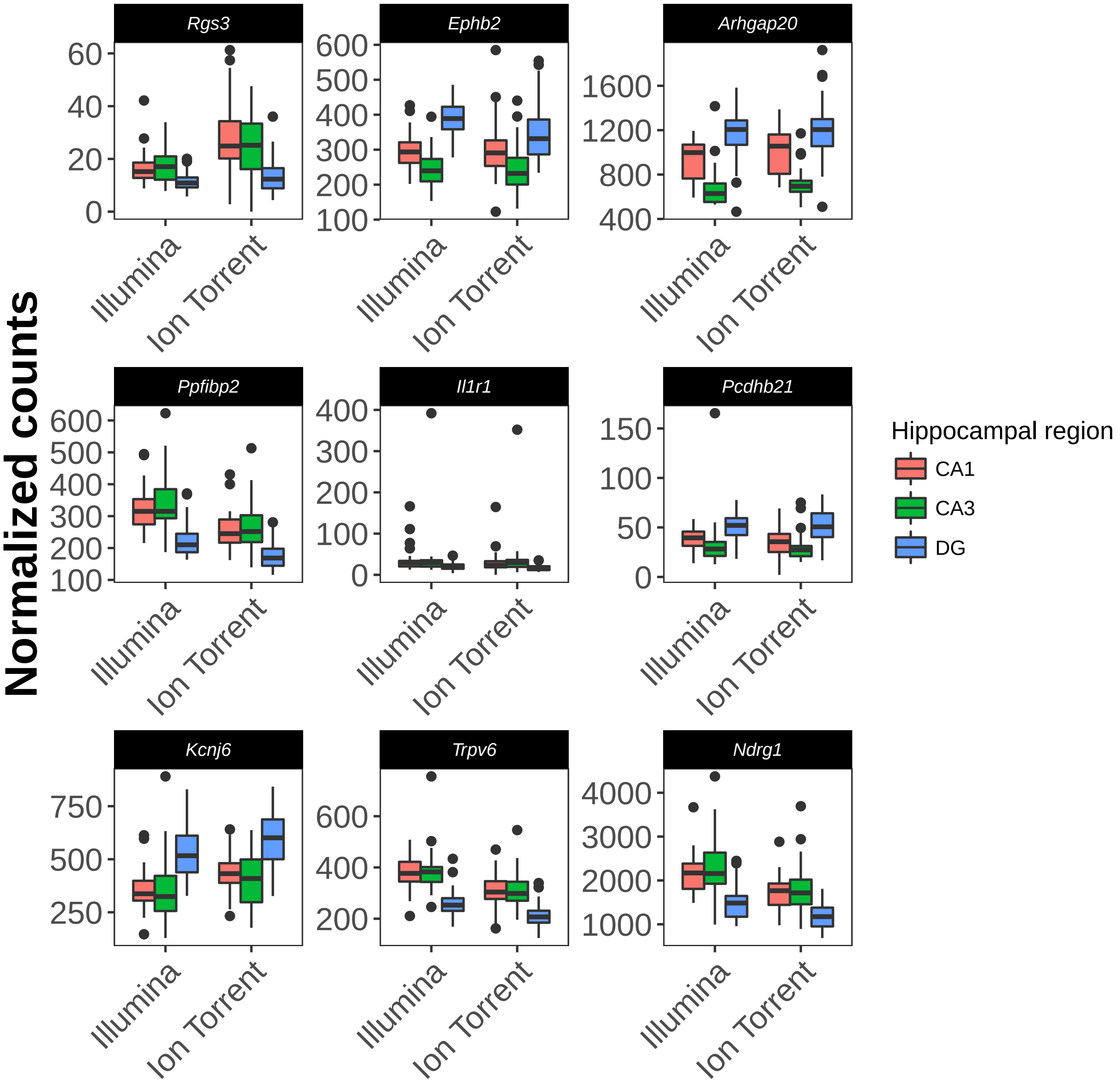

Normalized counts
Rgs3
Ephb2
Arhgap20
Ppfibp2
Il1r1
Pcdhb21
Kcnj6
Trpv6
Ndrg1
Illumina
Ion Torrent
Hippocampal region
CA1
CA3
DG

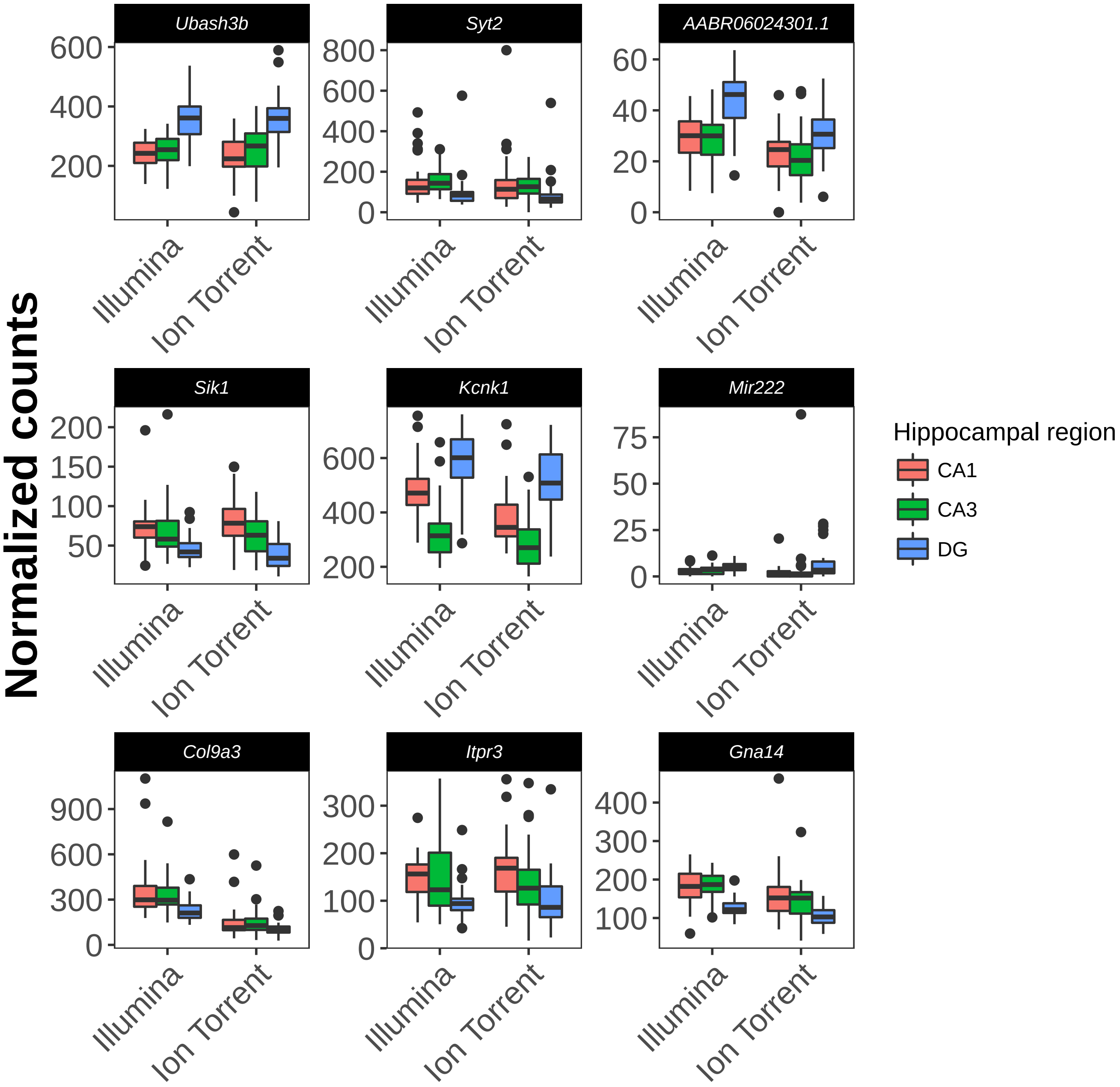
Normalized counts
Ubash3b
Syt2
AABR06024301.1
Sik1
Kcnk1
Mir222
Col9a3
Itpr3
Gna14
Illumina
Ion Torrent
Hippocampal region
CA1
CA3
DG

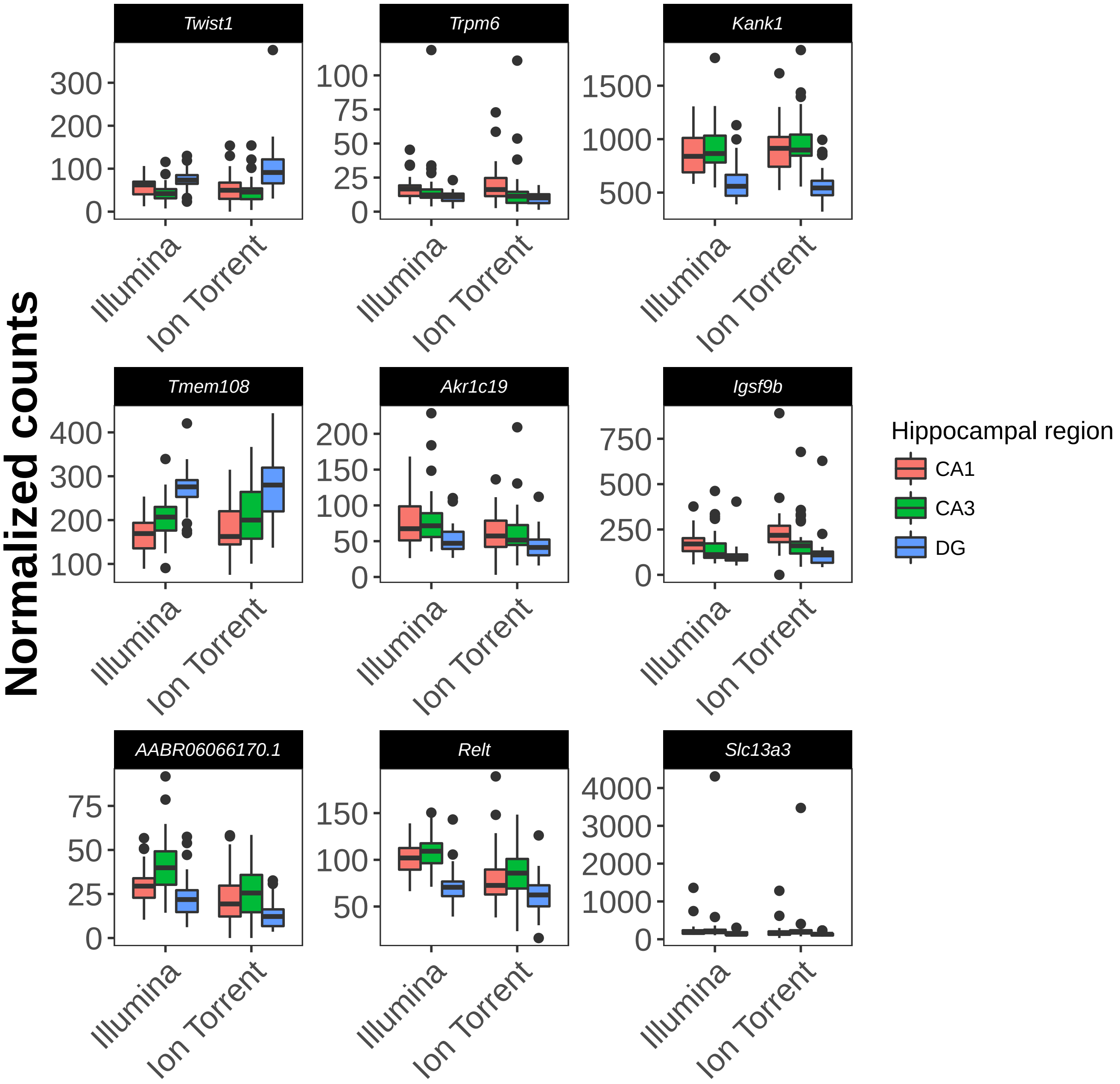

Normalized counts
Twist1
Trpm6
Kank1
Tmem108
Akr1c19
Igsf9b
AABR06066170.1
Relt
Slc13a3
Illumina
Ion Torrent
Hippocampal region
CA1
CA3
DG

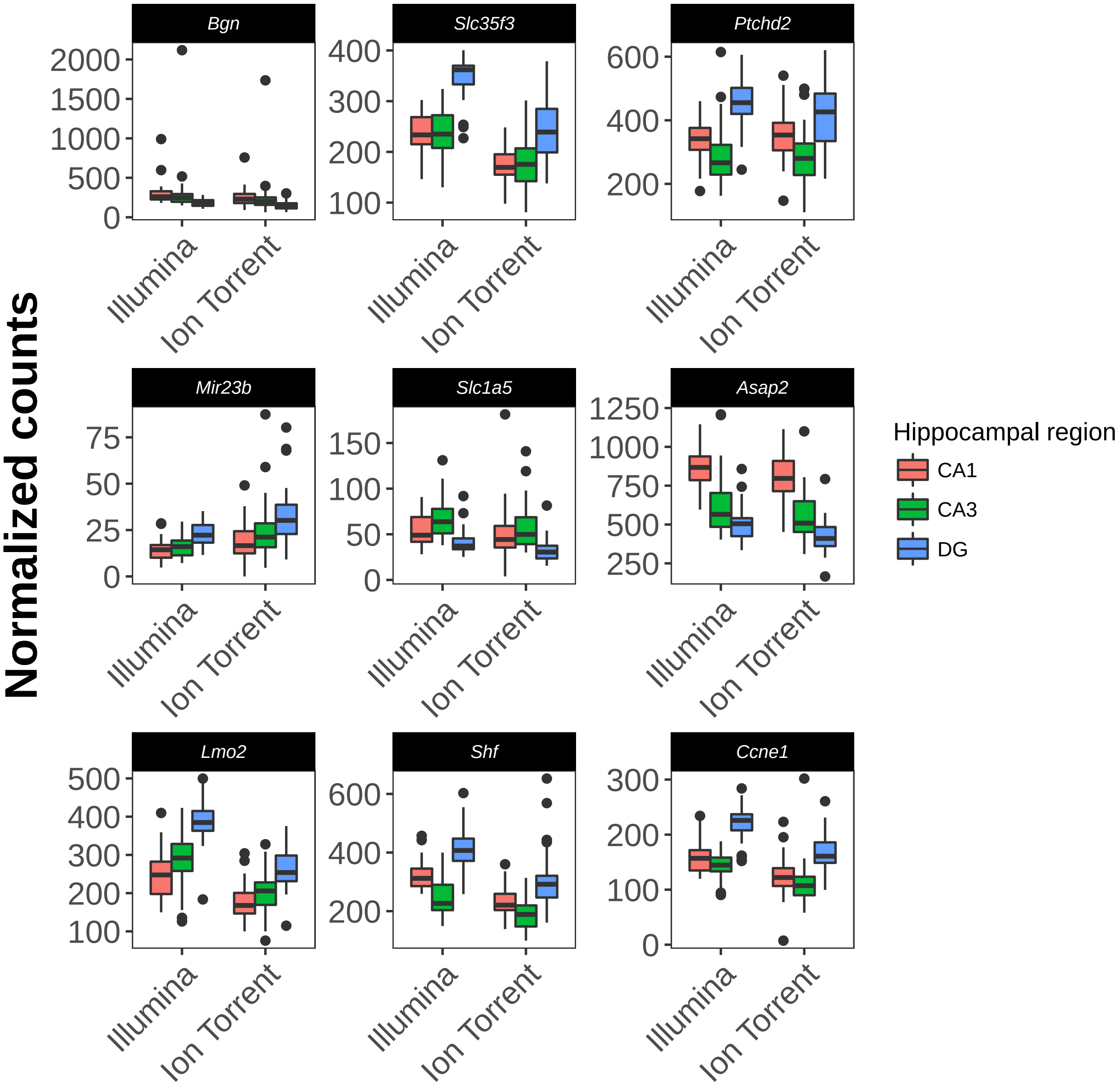
Normalized counts
Bgn
Slc35f3
Ptchd2
Mir23b
Slc1a5
Asap2
Lmo2
Shf
Ccne1
Illumina
Ion Torrent
Hippocampal region
CA1
CA3
DG

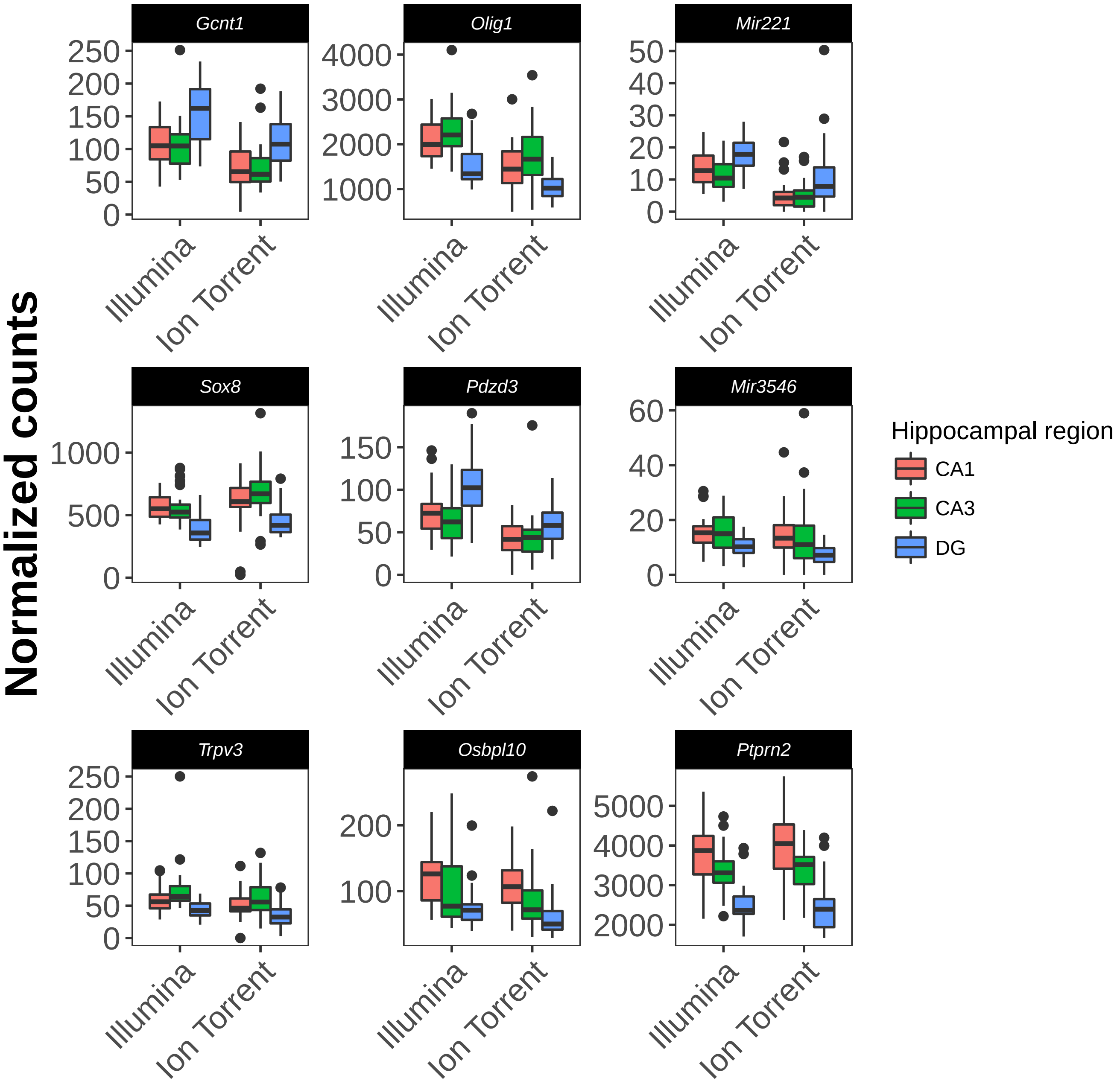
Normalized counts
Gcnt1
Olig1
Mir221
Sox8
Pdzd3
Mir3546
Trpv3
Osbpl10
Ptprn2
Illumina
Ion Torrent
Hippocampal region
CA1
CA3
DG

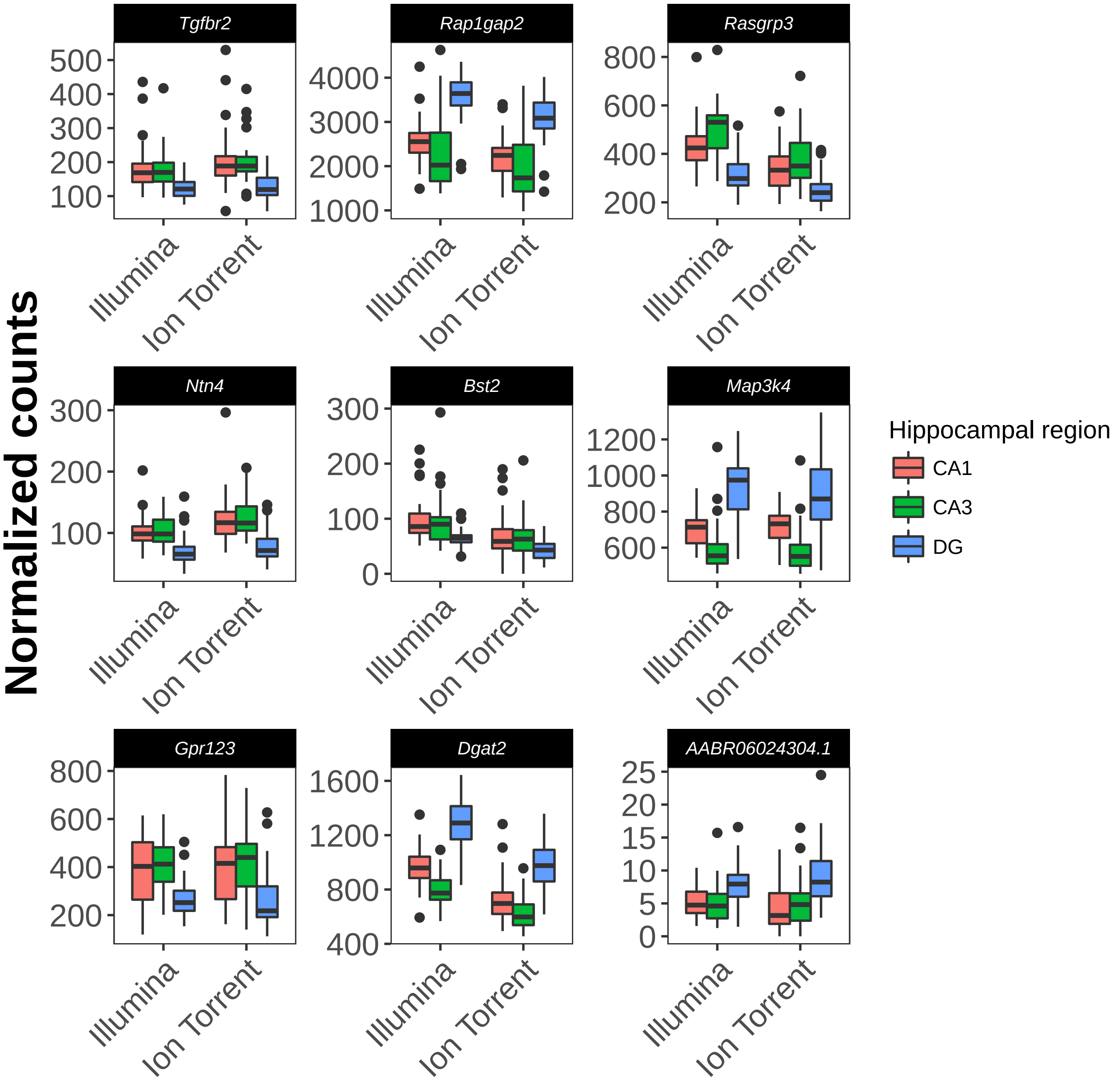
Normalized counts
Tgfbr2
Rap1gap2
Rasgrp3
Ntn4
Bst2
Map3k4
Gpr123
Dgat2
AABR06024304.1
Illumina
Ion Torrent
Hippocampal region
CA1
CA3
DG

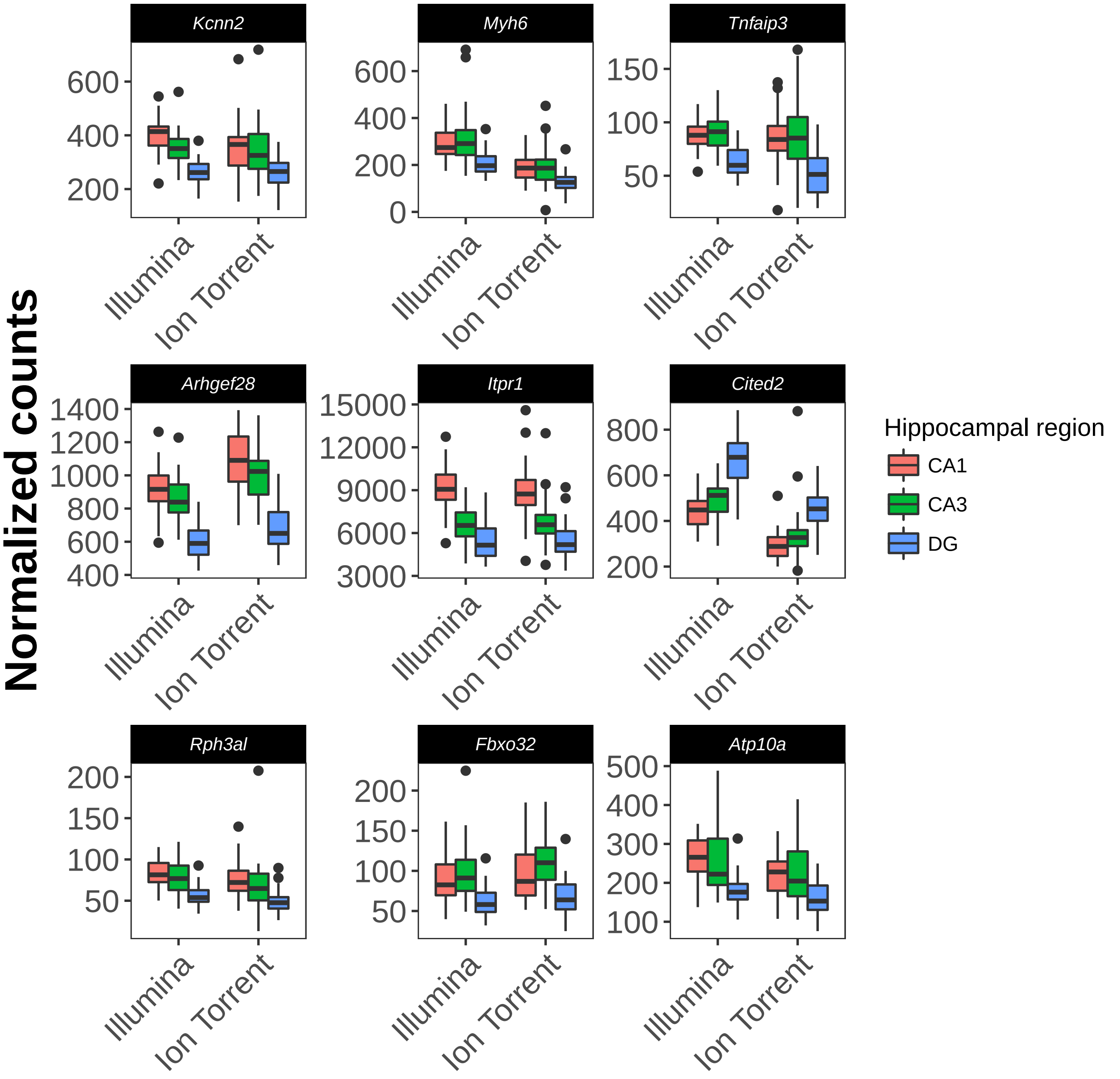
Normalized counts
Kcnn2
Myh6
Tnfaip3
Arhgef28
Itpr1
Cited2
Rph3al
Fbxo32
Atp10a
Illumina
Ion Torrent
Hippocampal region
CA1
CA3
DG

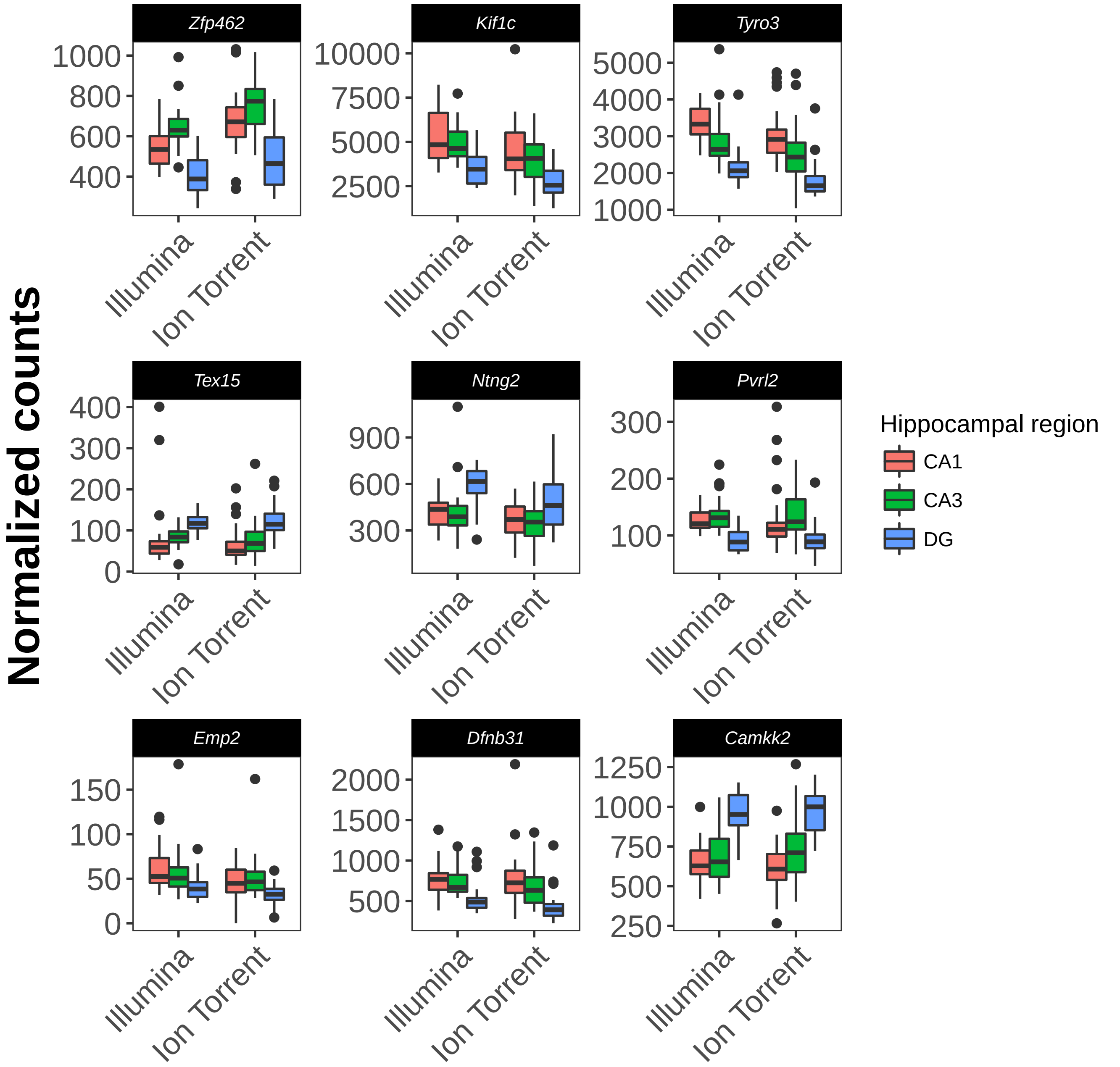
Normalized counts
Zfp462
Kif1c
Tyro3
Tex15
Ntng2
Pvrl2
Emp2
Dfnb31
Camkk2
Illumina
Ion Torrent
Hippocampal region
CA1
CA3
DG

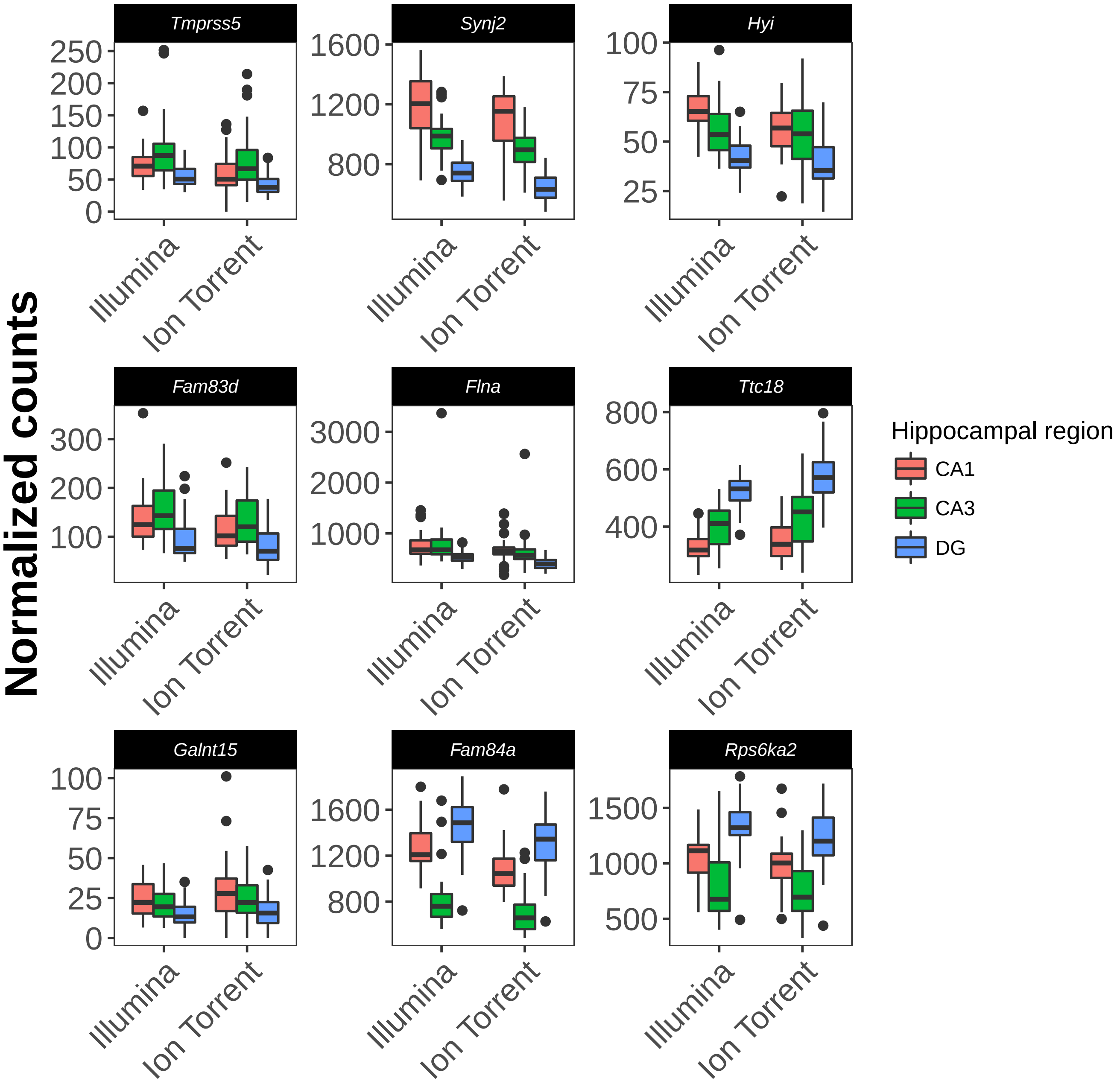
Normalized counts
Tmprss5
Synj2
Hyi
Fam83d
Flna
Ttc18
Galnt15
Fam84a
Rps6ka2
Illumina
Ion Torrent
Hippocampal region
CA1
CA3
DG

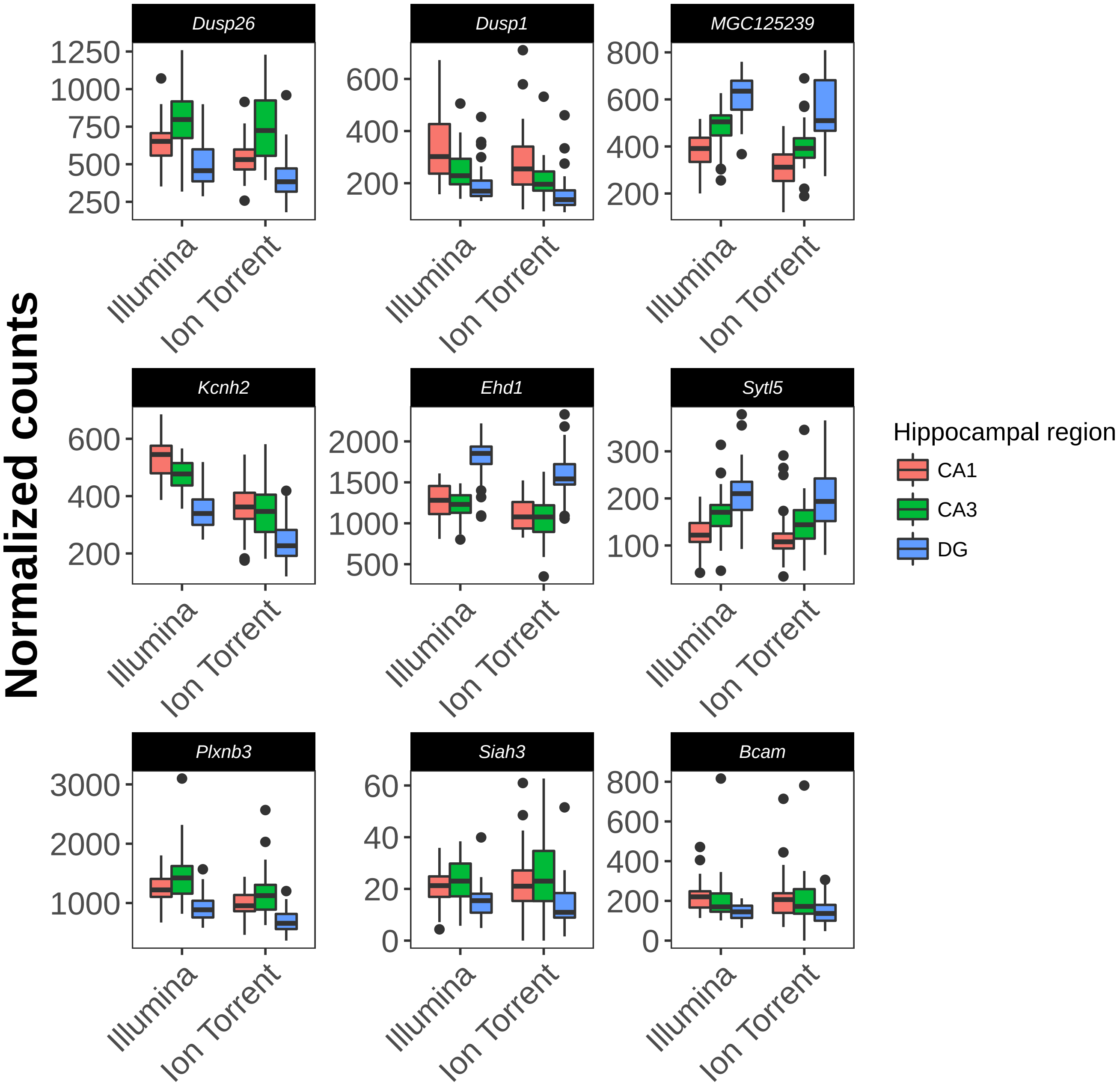
Normalized counts
Dusp26
Dusp1
MGC125239
Kcnh2
Ehd1
Sytl5
Plxnb3
Siah3
Bcam
Illumina
Ion Torrent
Hippocampal region
CA1
CA3
DG

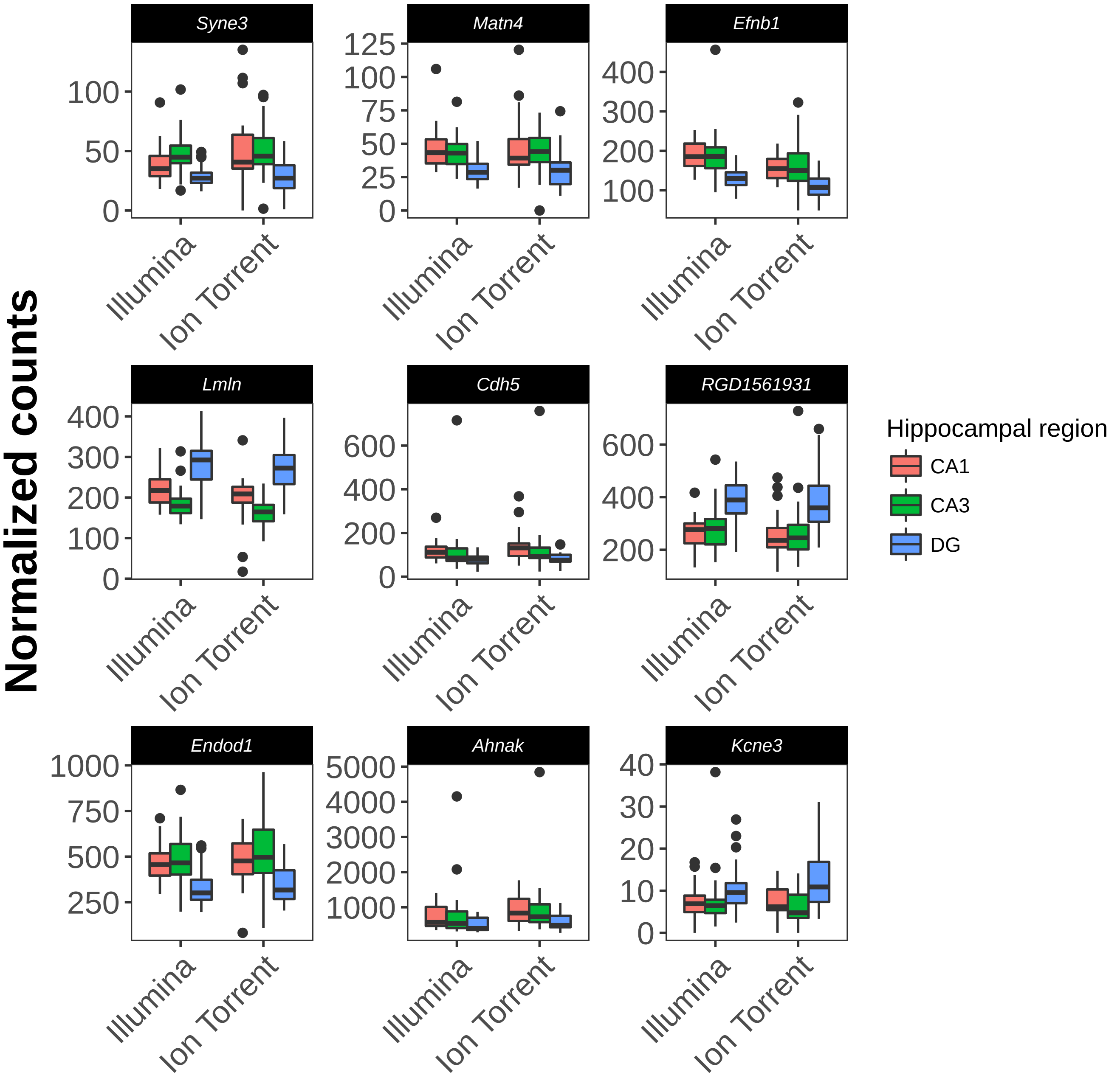

Normalized counts
Syne3
Matn4
Efnb1
Lmln
Cdh5
RGD1561931
Endod1
Ahnak
Kcne3
Illumina
Ion Torrent
Hippocampal region
CA1
CA3
DG

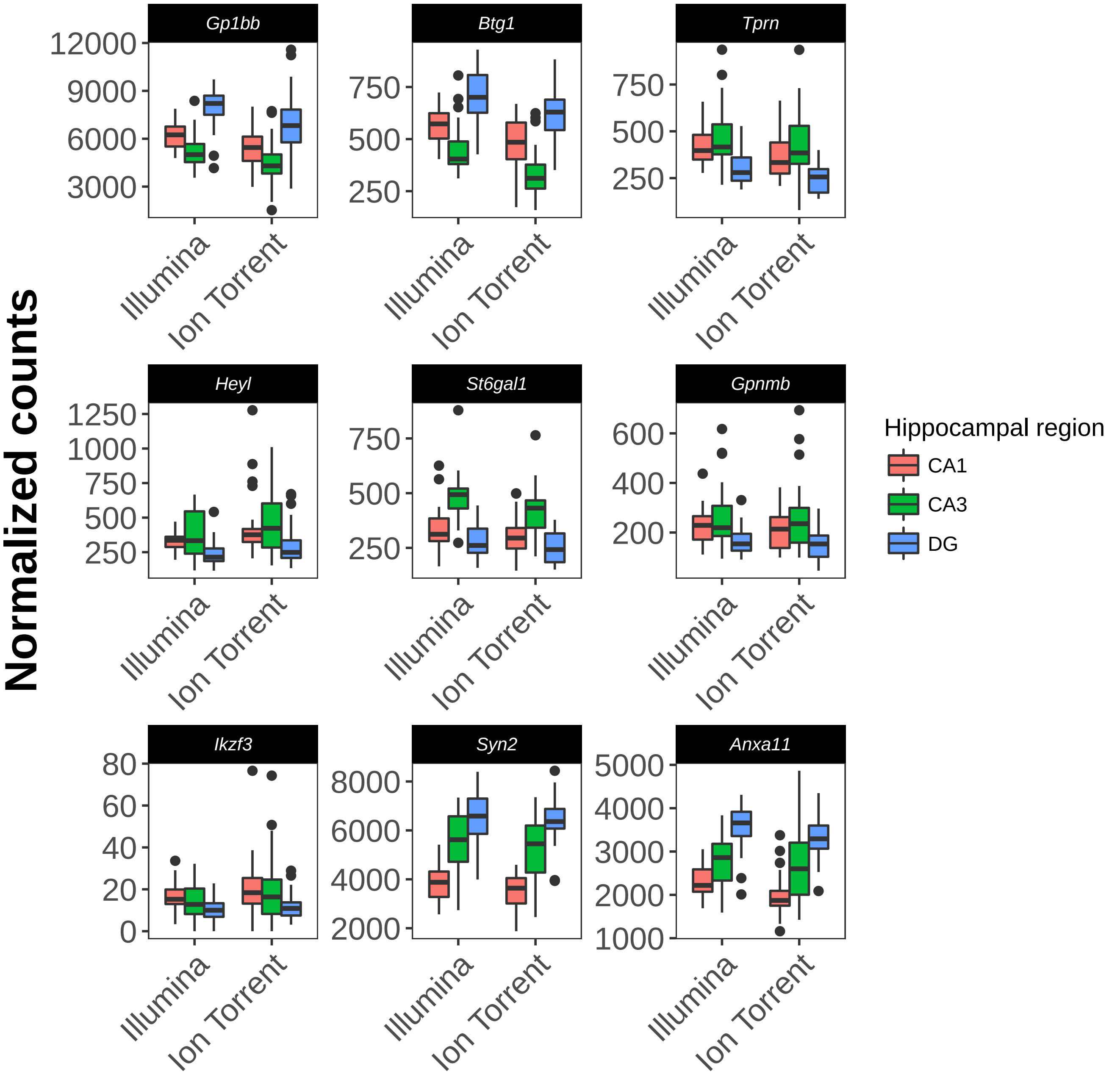

Normalized counts
Gp1bb
Btg1
Tprn
Heyl
St6gal1
Gpnmb
Ikzf3
Syn2
Anxa11
Illumina
Ion Torrent
Hippocampal region
CA1
CA3
DG

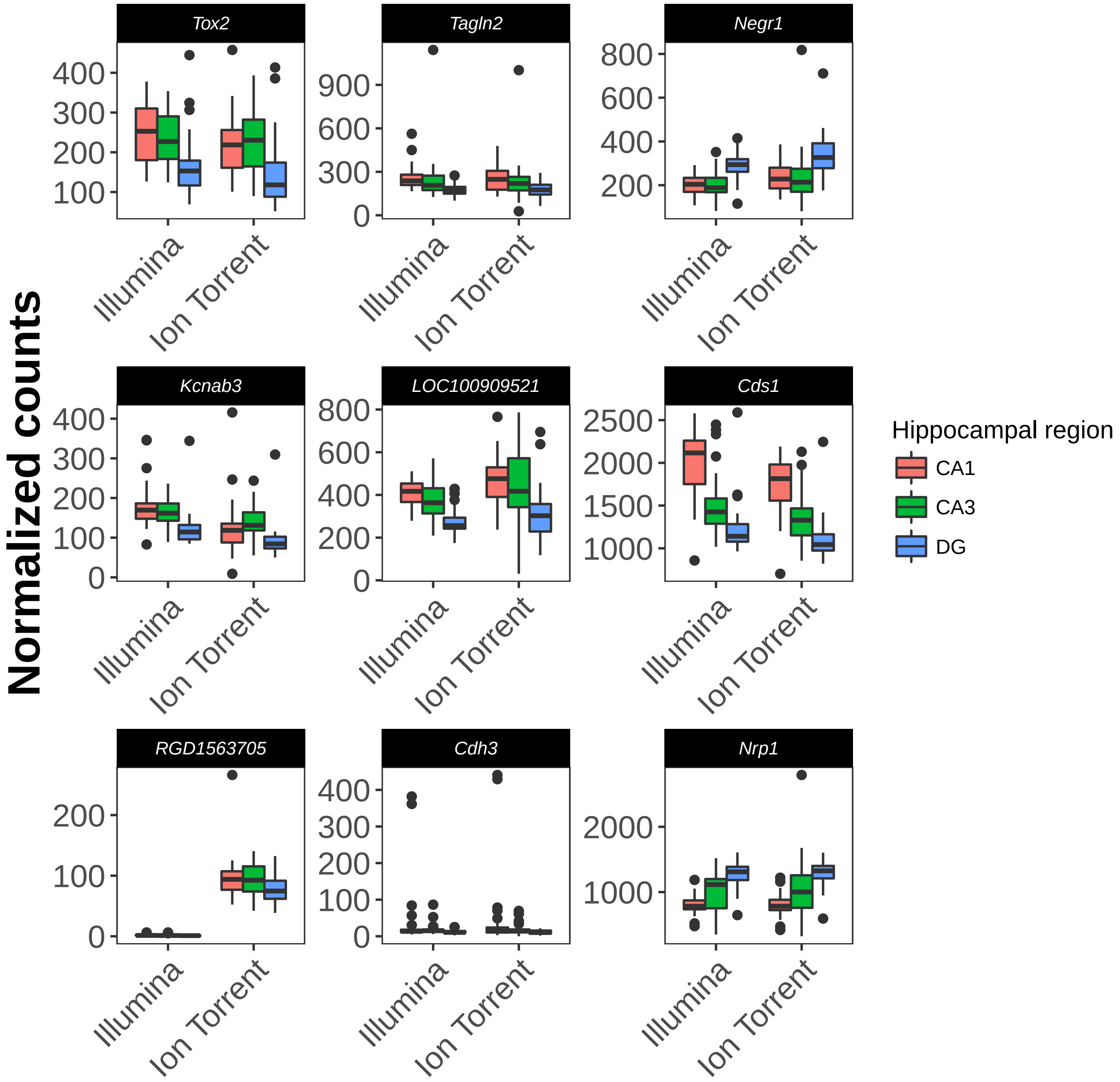

Normalized counts
Tox2
Tagln2
Negr1
Kcnab3
LOC100909521
Cds1
RGD1563705
Cdh3
Nrp1
Illumina
Ion Torrent
Hippocampal region
CA1
CA3
DG

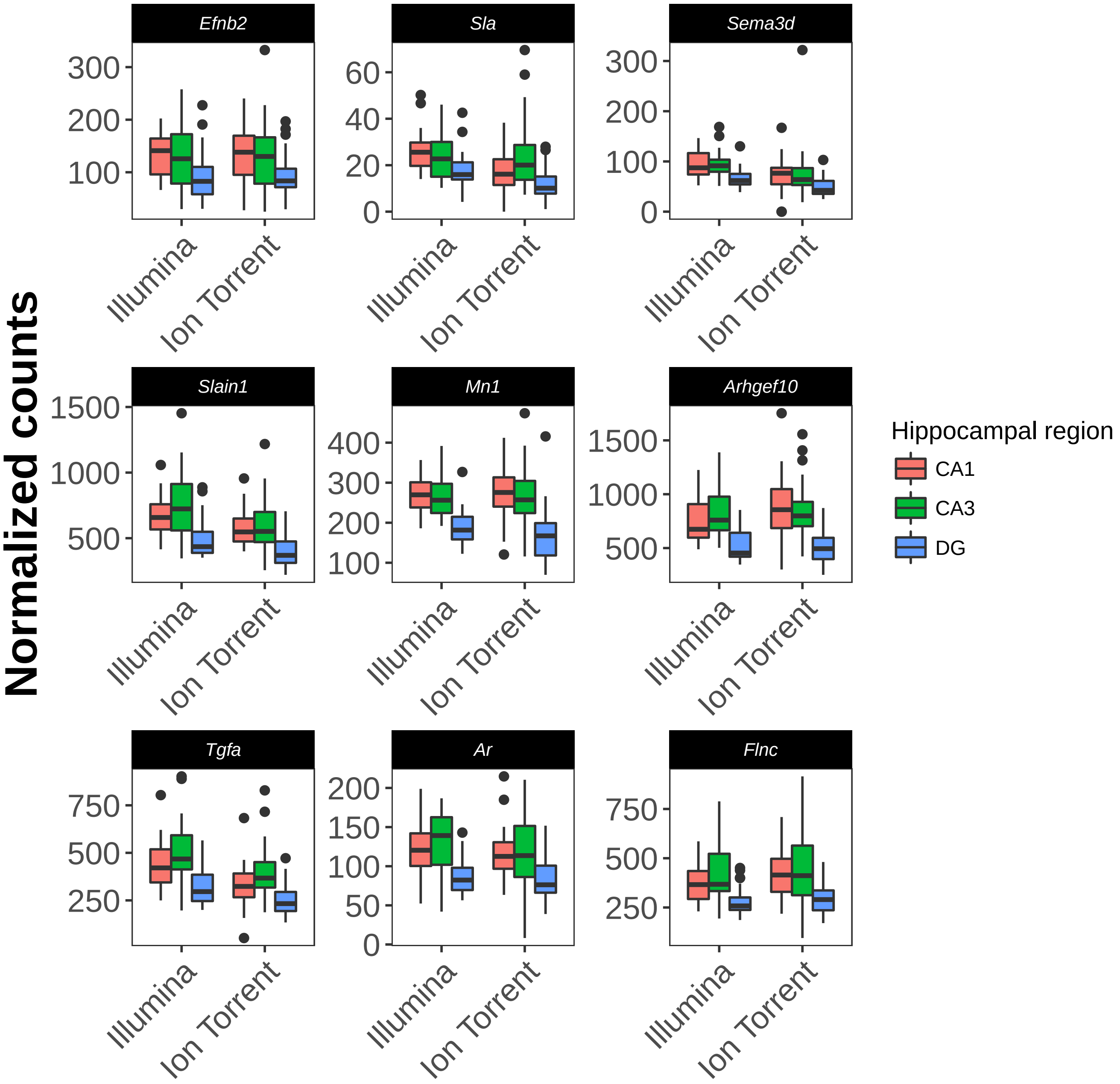

Normalized counts
Efnb2
Sla
Sema3d
Slain1
Mn1
Arhgef10
Tgfa
Ar
Flnc
Illumina
Ion Torrent
Hippocampal region
CA1
CA3
DG

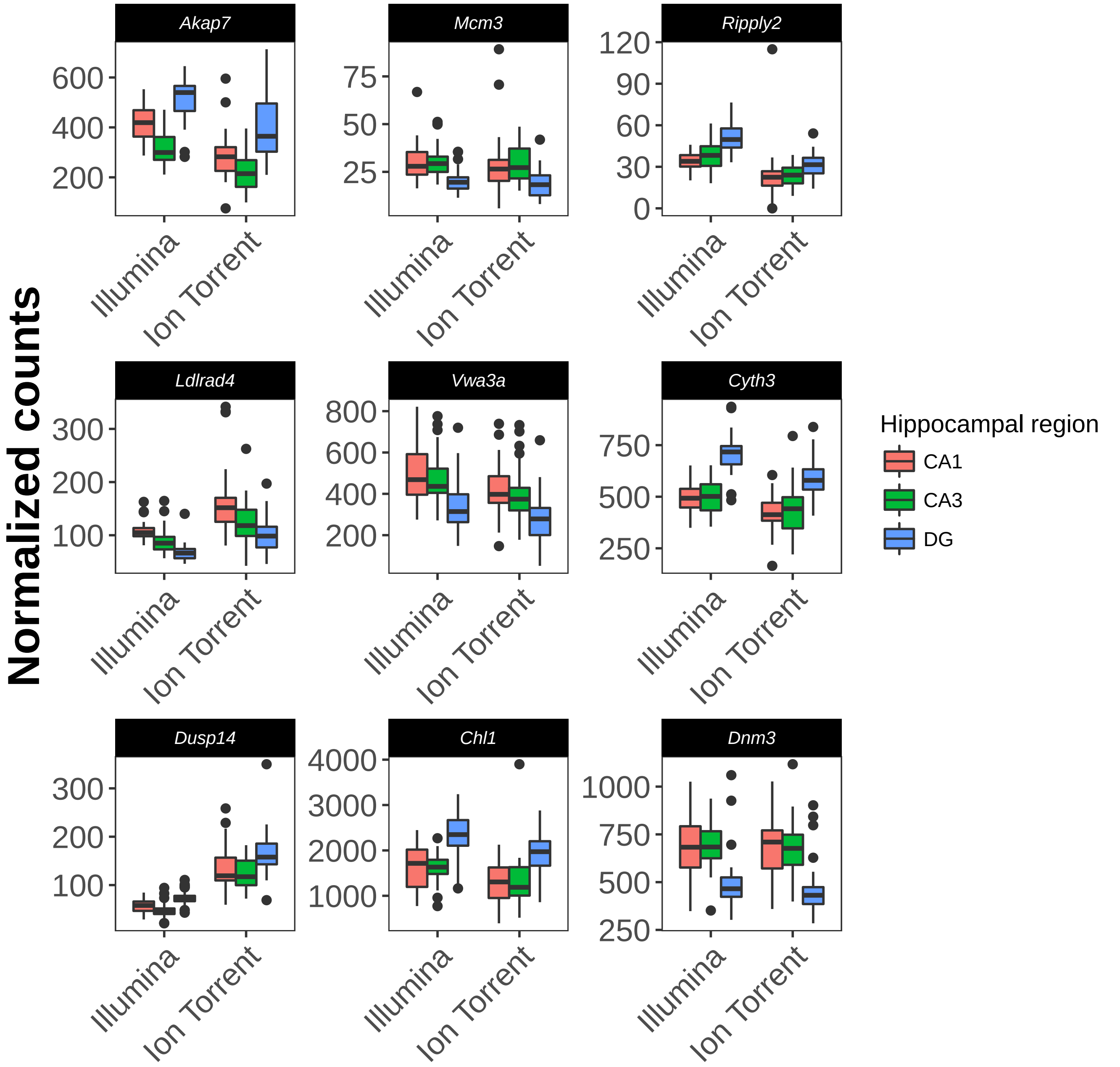

Normalized counts
Akap7
Mcm3
Ripply2
Ldlrad4
Vwa3a
Cyth3
Dusp14
Chl1
Dnm3
Illumina
Ion Torrent
Hippocampal region
CA1
CA3
DG

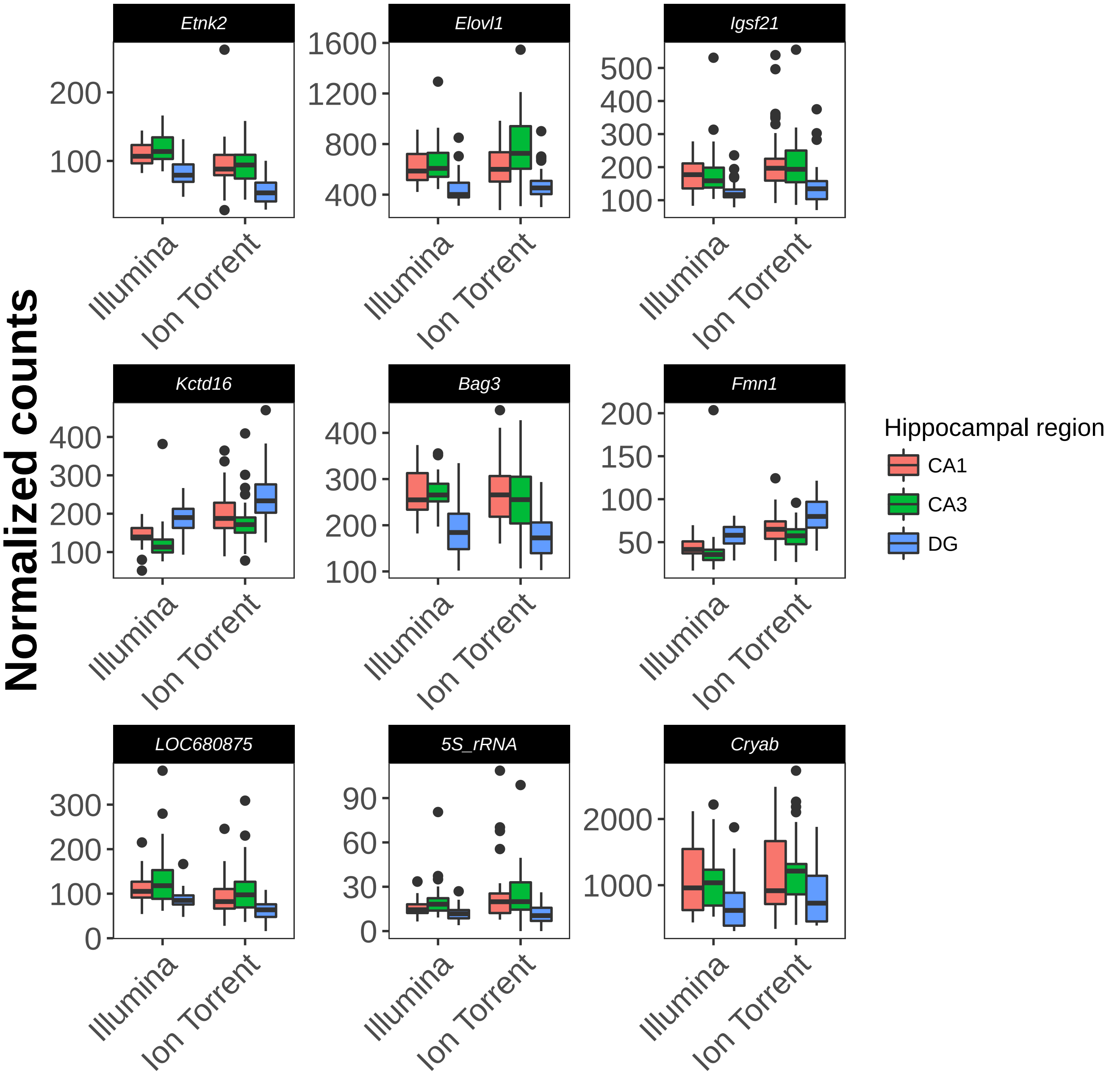
Normalized counts
Etnk2
Elovl1
Igsf21
Kctd16
Bag3
Fmn1
LOC680875
5S_rRNA
Cryab
Illumina
Ion Torrent
Hippocampal region
CA1
CA3
DG

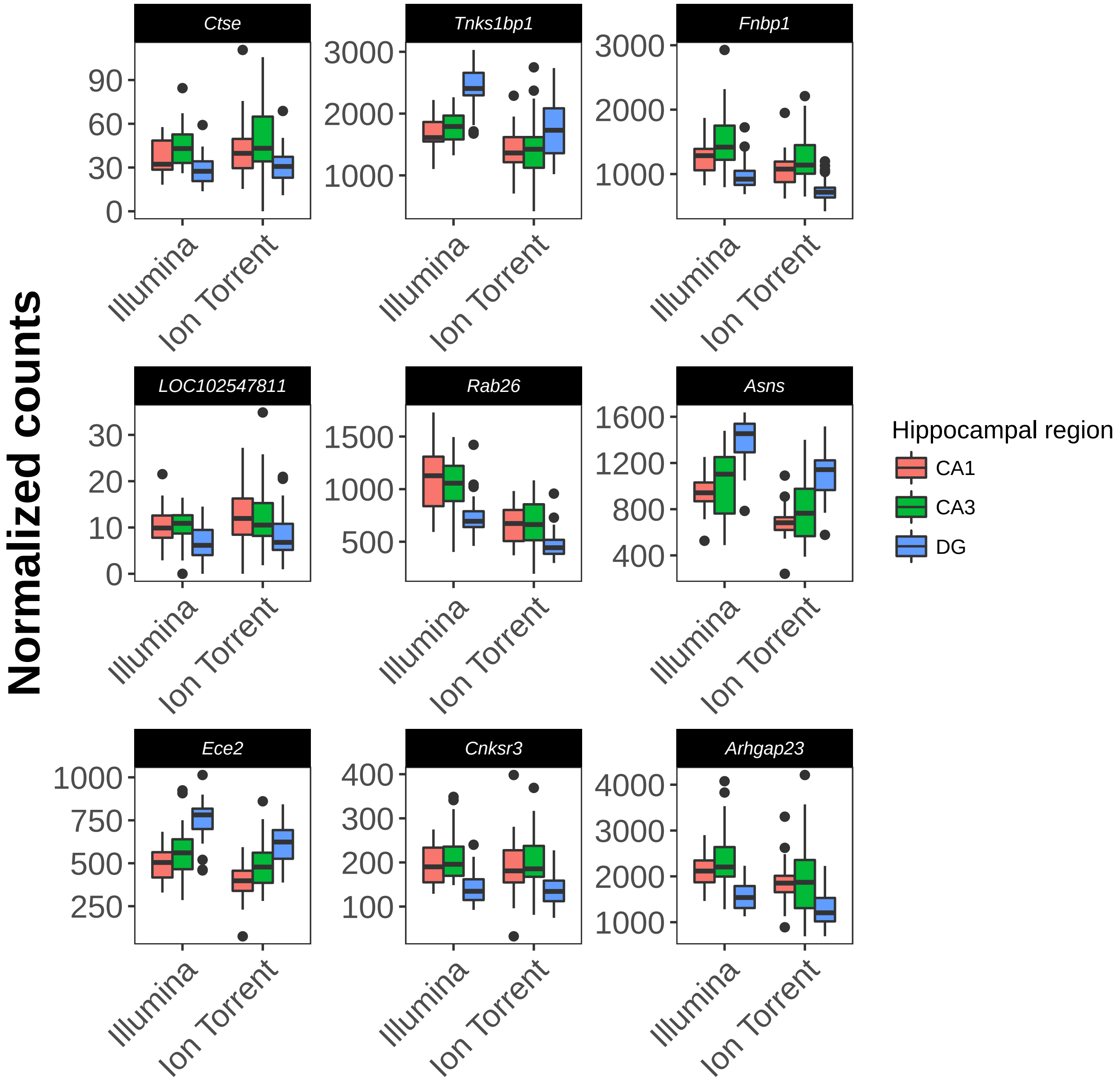
Normalized counts
Ctse
Tnks1bp1
Fnbp1
LOC102547811
Rab26
Asns
Ece2
Cnksr3
Arhgap23
Illumina
Ion Torrent
Hippocampal region
CA1
CA3
DG

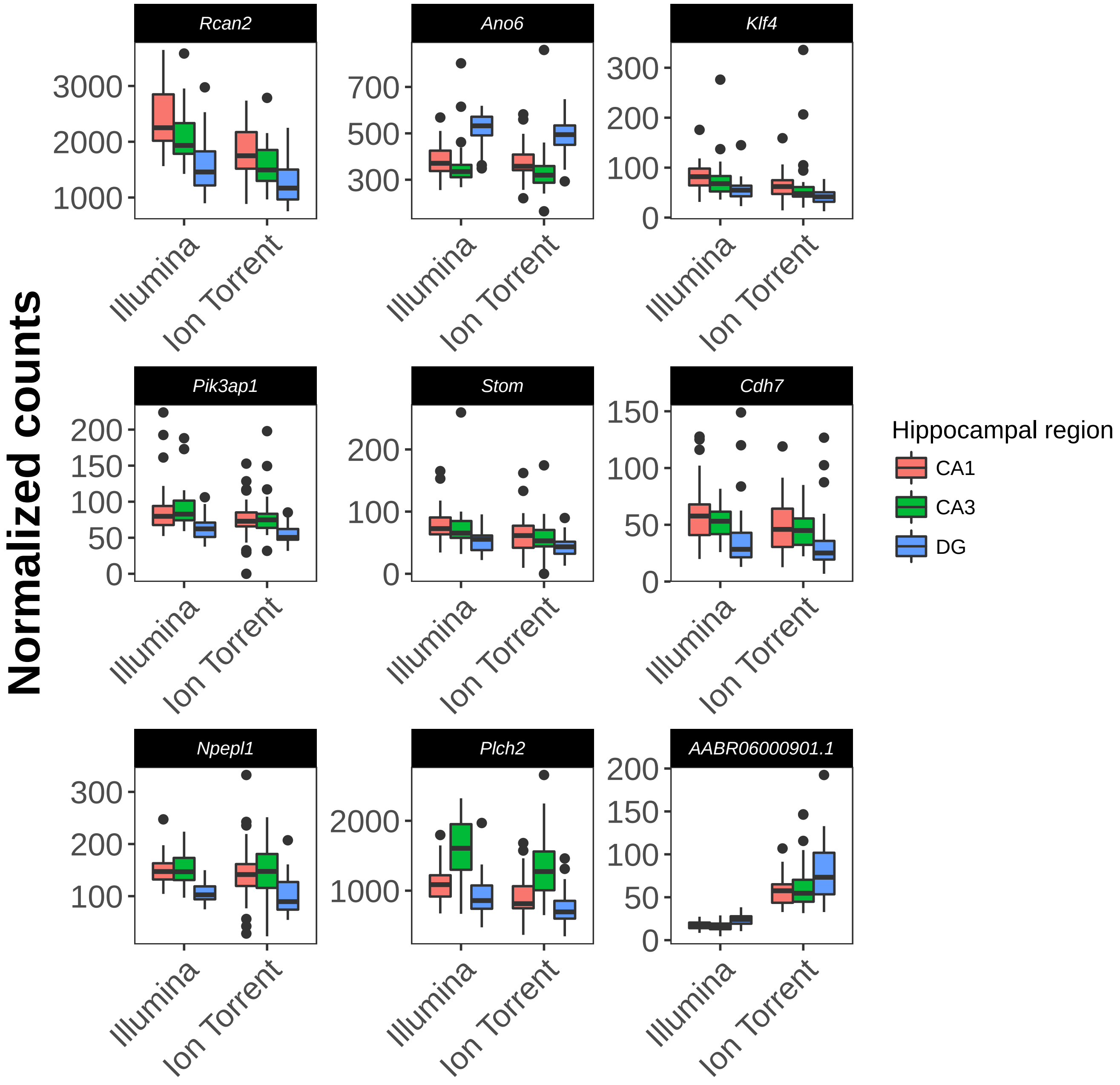

Normalized counts
Rcan2
Ano6
Klf4
Pik3ap1
Stom
Cdh7
Npepl1
Plch2
AABR06000901.1
Illumina
Ion Torrent
Hippocampal region
CA1
CA3
DG

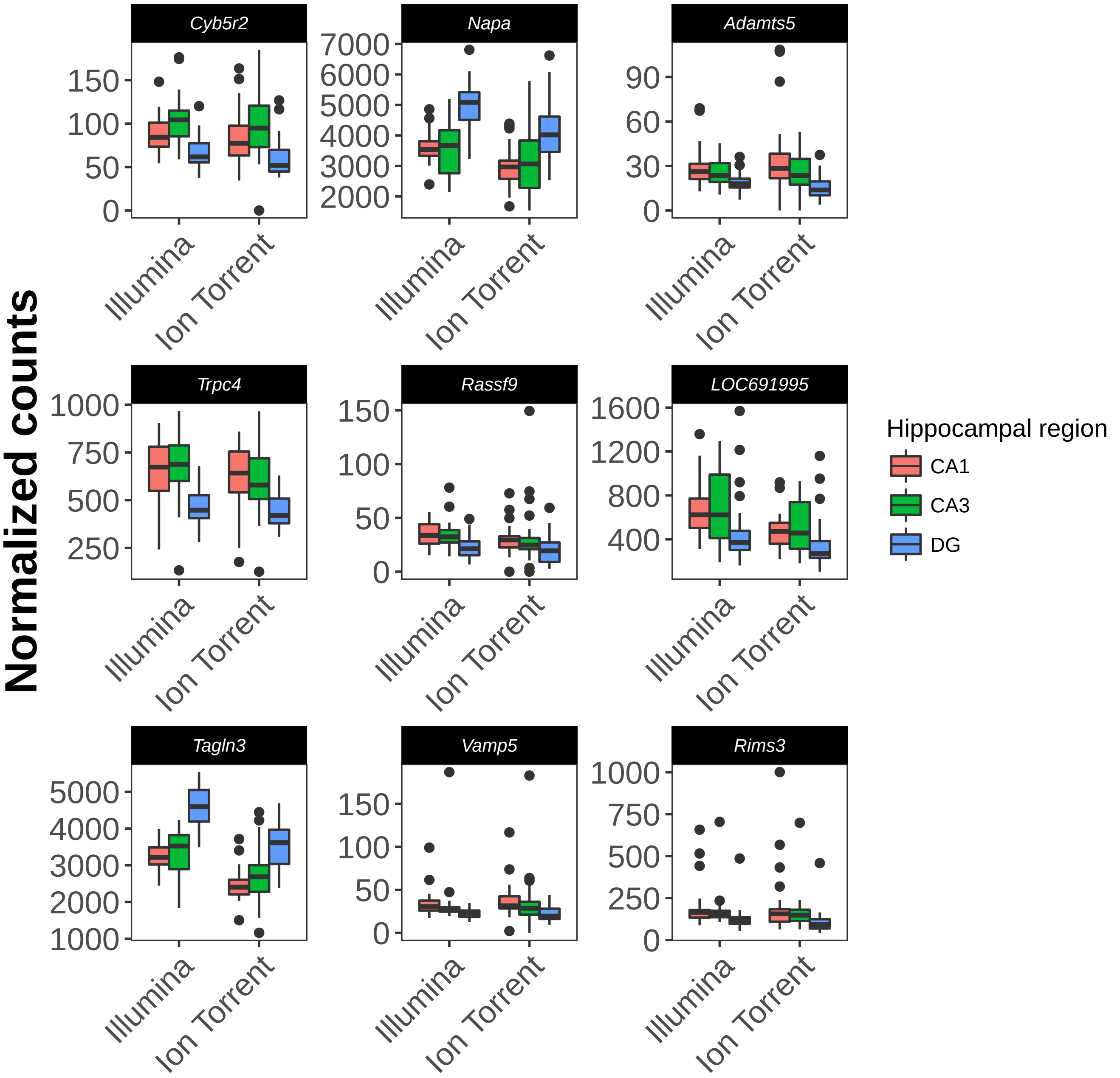
Normalized counts
Cyb5r2
Napa
Adamts5
Trpc4
Rassf9
LOC691995
Tagln3
Vamp5
Rims3
Illumina
Ion Torrent
Hippocampal region
CA1
CA3
DG

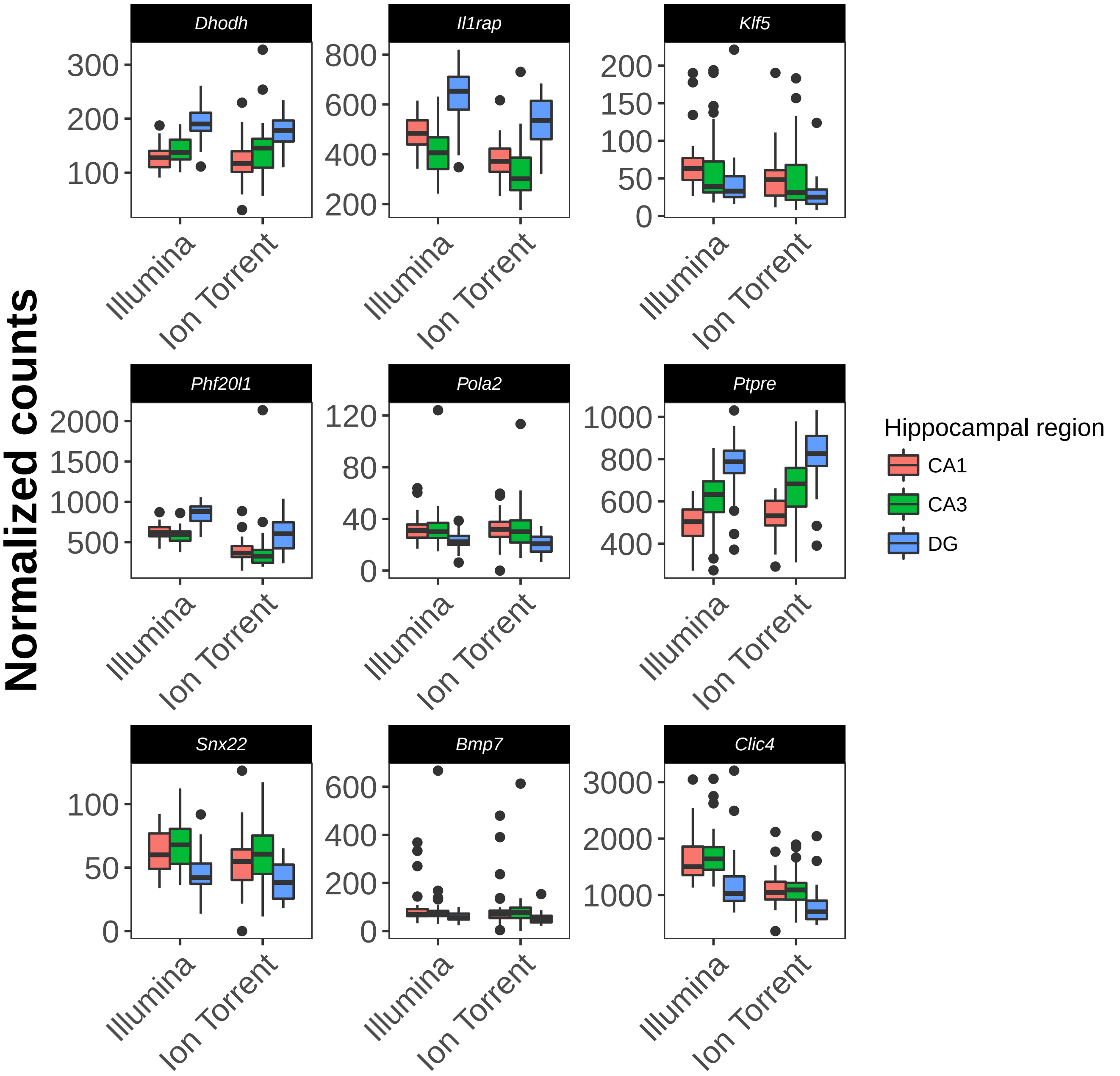
Normalized counts
Dhodh
Il1rap
Klf5
Phf20l1
Pola2
Ptpre
Snx22
Bmp7
Clic4
300
200
100
800
600
400
200
150
50
0
2000
1500
1000
500
120
80
40
3000
Illumina
Ion Torrent
Hippocampal region
CA1
CA3
DG

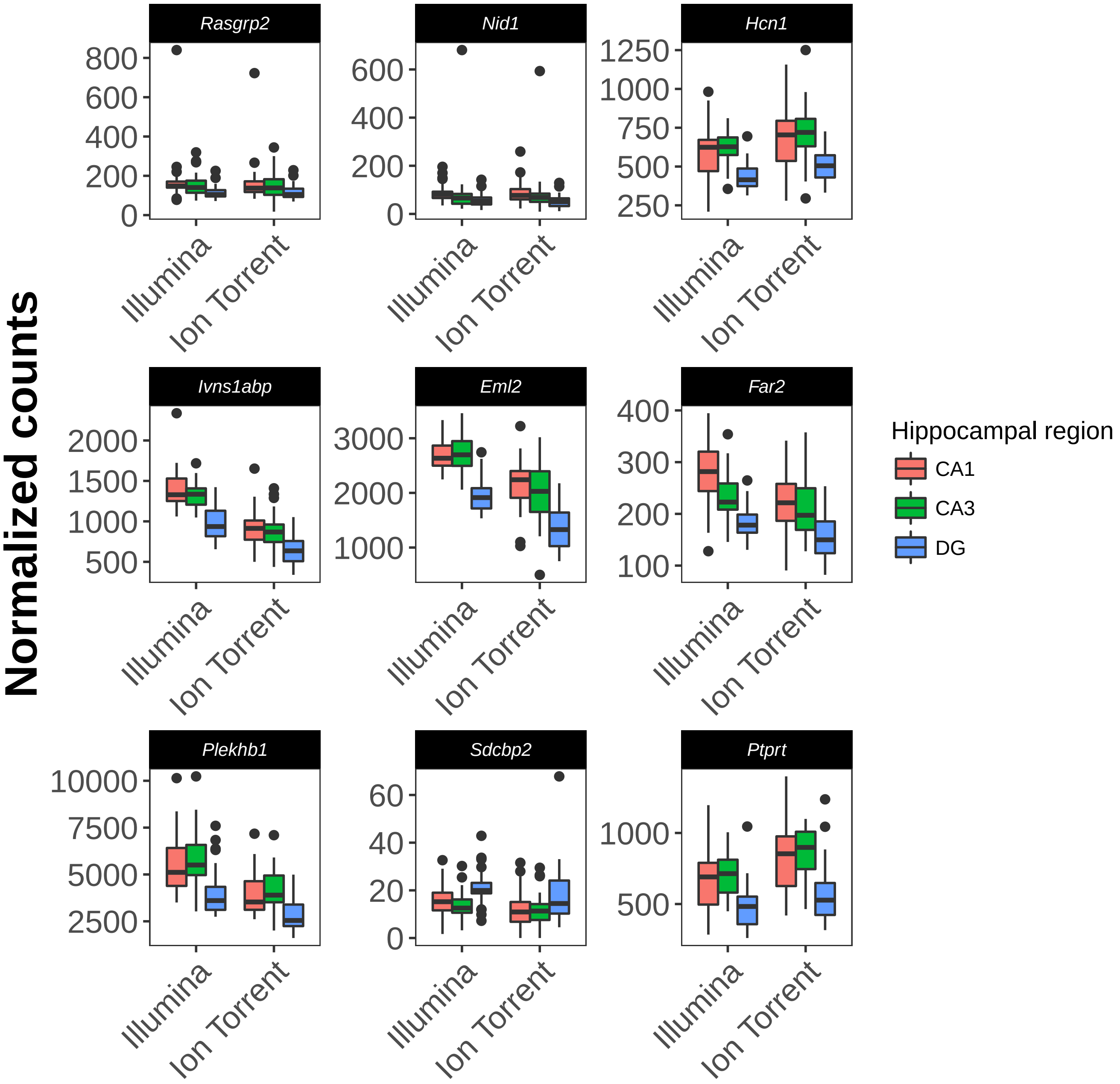
Normalized counts
Rasgrp2
Nid1
Hcn1
Ivns1abp
Eml2
Far2
Plekhb1
Sdcbp2
Ptprt
Illumina
Ion Torrent
Hippocampal region
CA1
CA3
DG

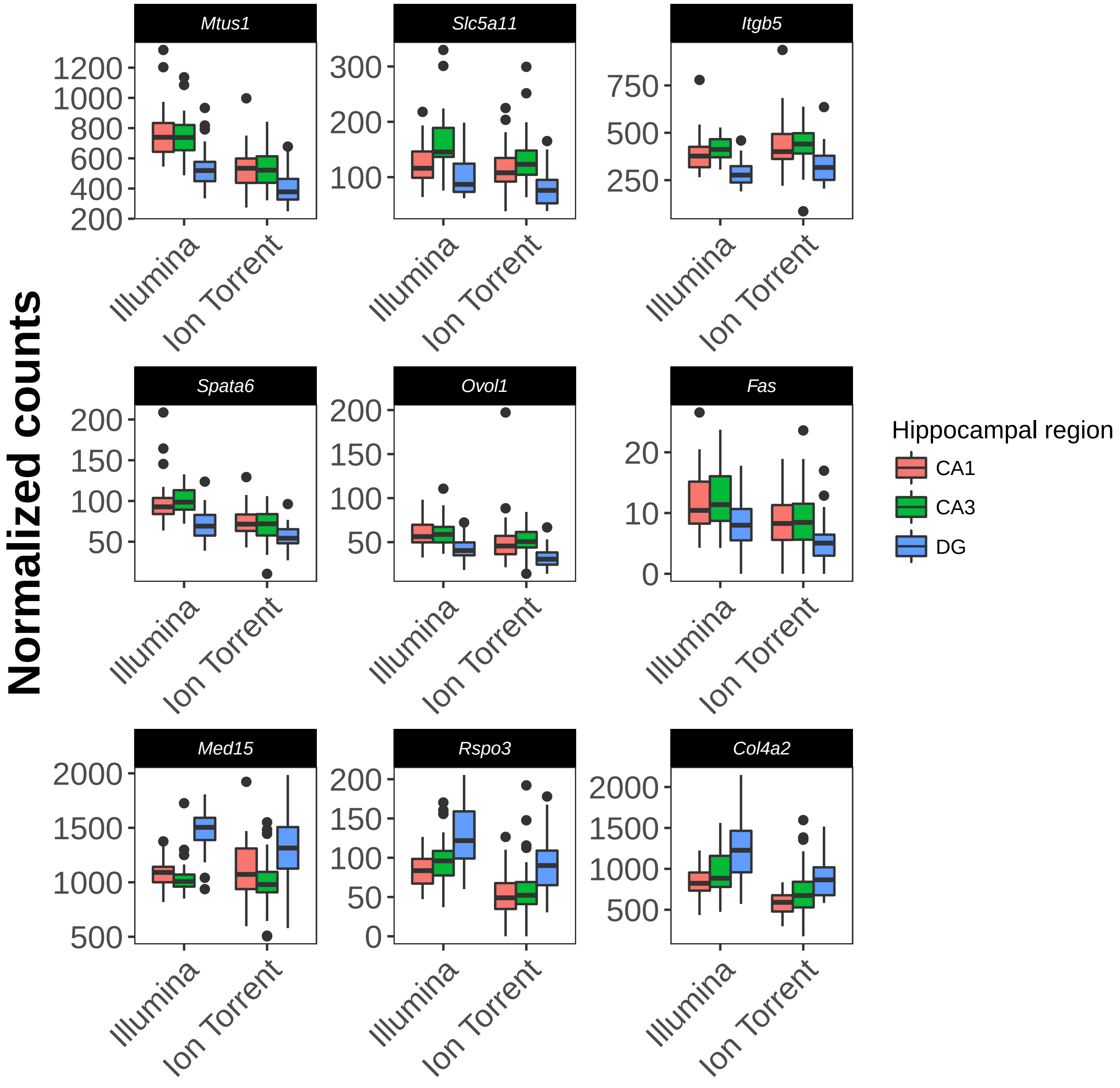

Normalized counts
Mtus1
Slc5a11
Itgb5
Spata6
Ovol1
Fas
Med15
Rspo3
Col4a2
Illumina
Ion Torrent
Hippocampal region
CA1
CA3
DG

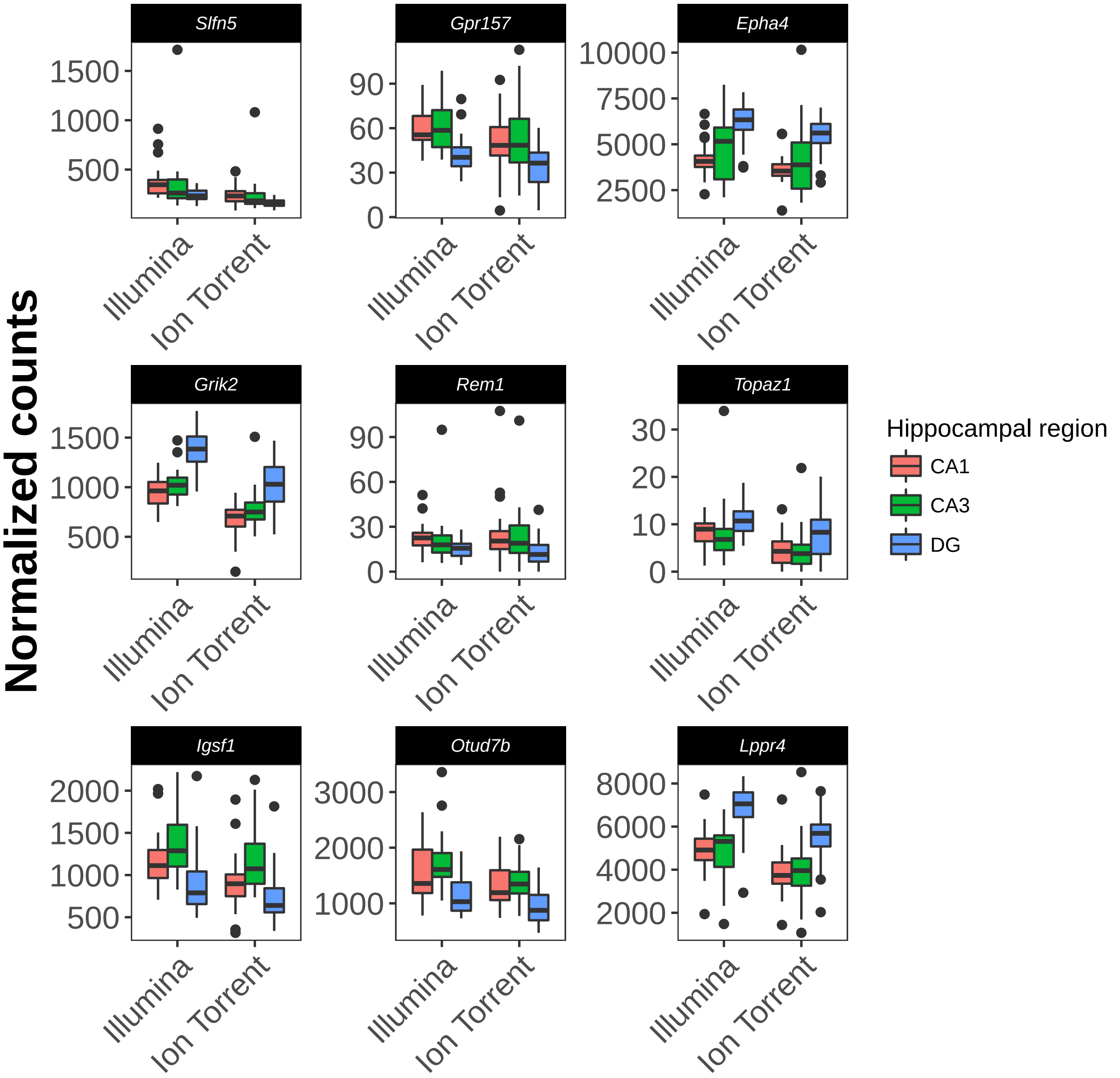

Normalized counts
Slfn5
Gpr157
Epha4
Grik2
Rem1
Topaz1
Igsf1
Otud7b
Lppr4
Illumina
Ion Torrent
Hippocampal region
CA1
CA3
DG

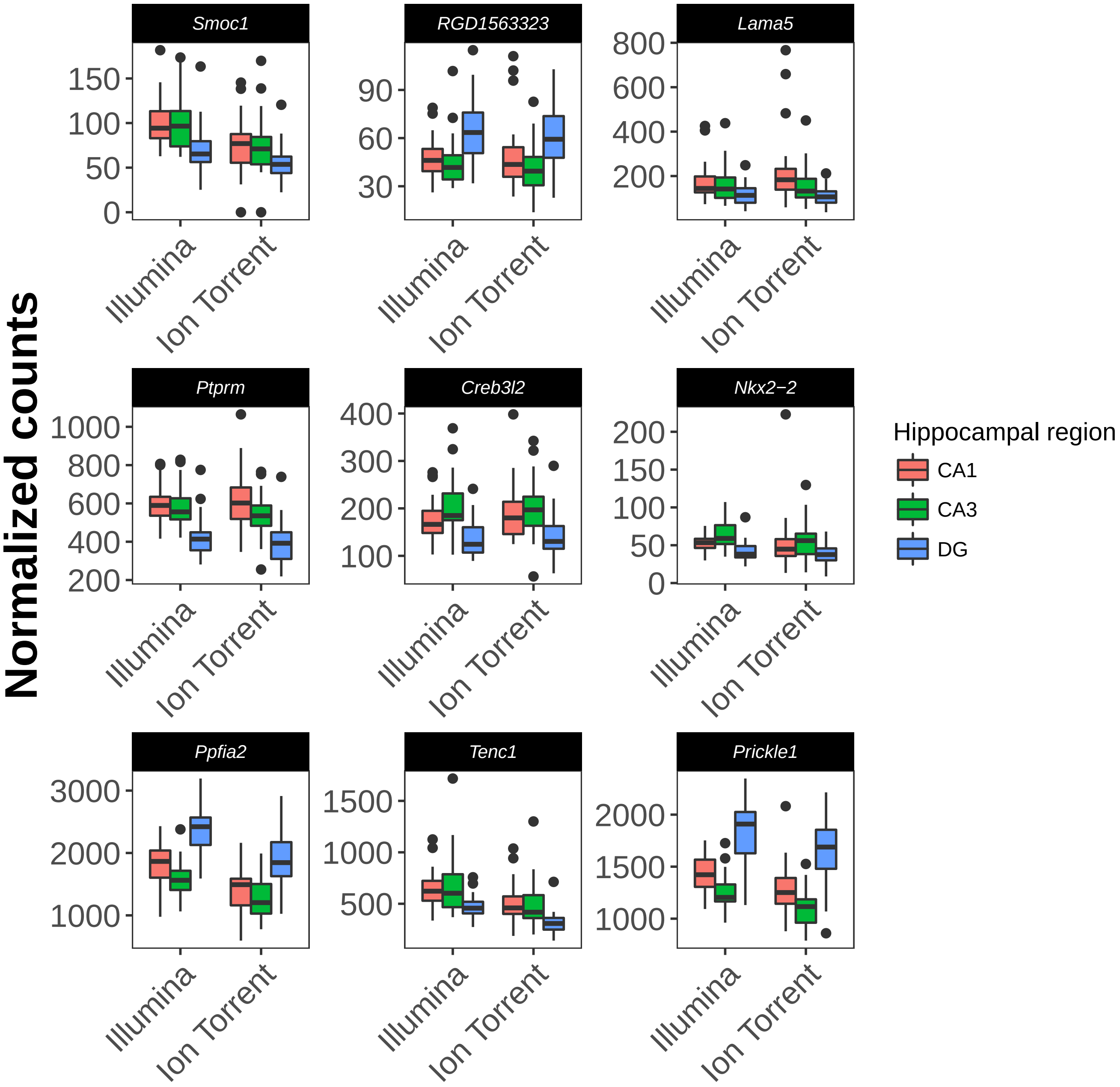
Normalized counts
Smoc1
RGD1563323
Lama5
Ptprm
Creb3l2
Nkx2−2
Ppfia2
Tenc1
Prickle1
Illumina
Ion Torrent
Hippocampal region
CA1
CA3
DG

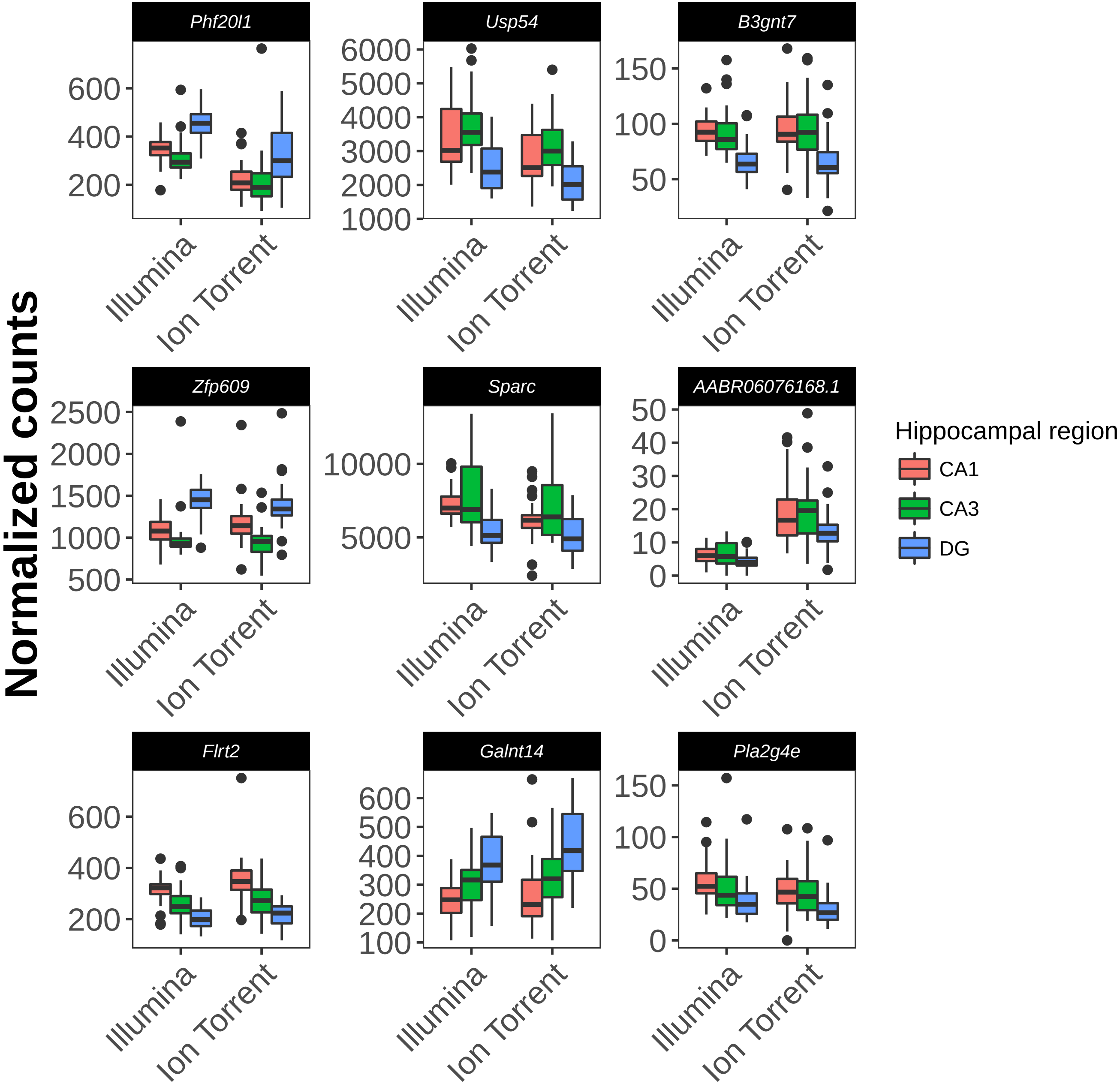
Normalized counts
Phf20l1
Usp54
B3gnt7
Zfp609
Sparc
AABR06076168.1
Flrt2
Galnt14
Pla2g4e
Illumina
Ion Torrent
Hippocampal region
CA1
CA3
DG

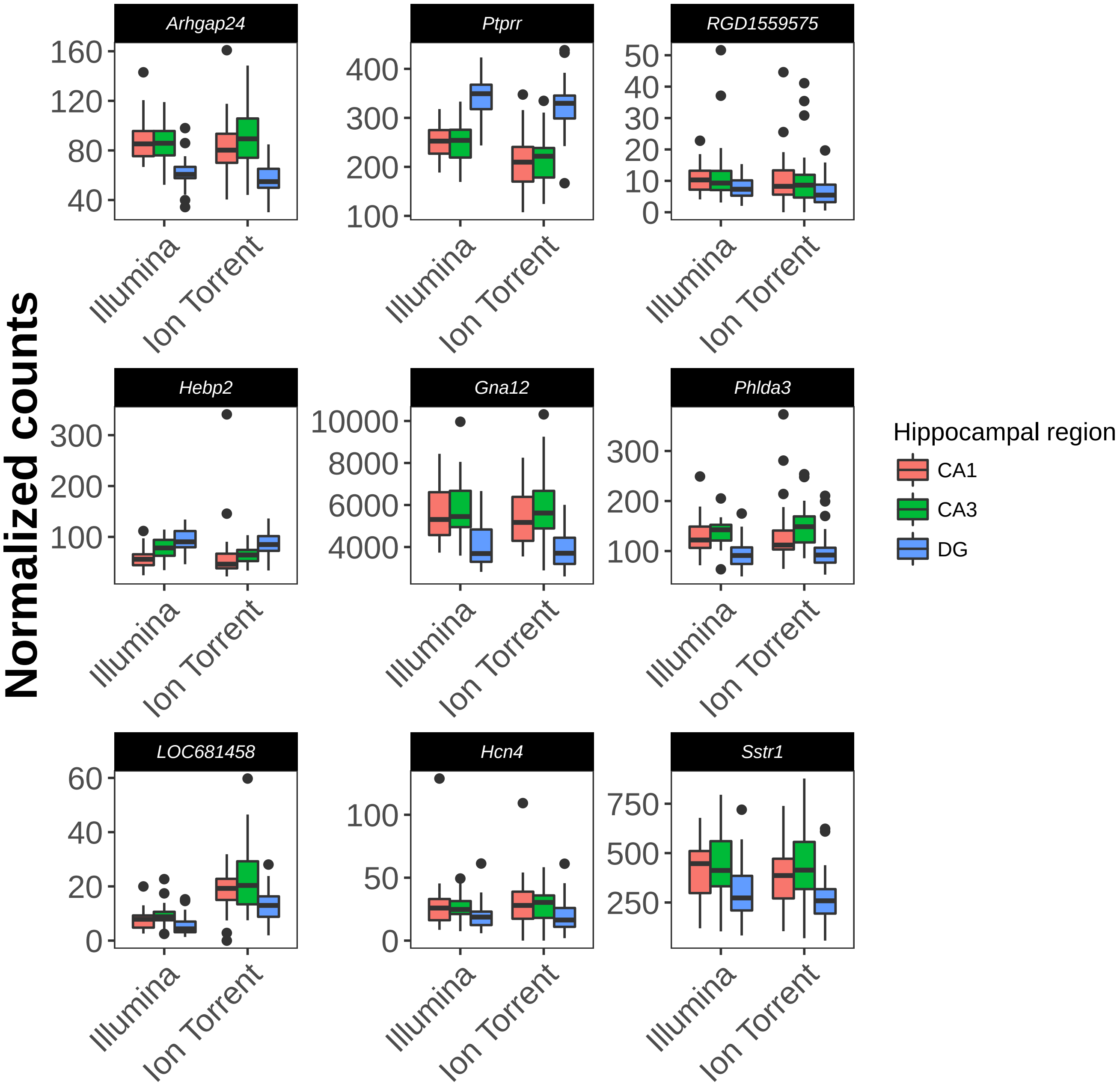
Normalized counts
Arhgap24
Ptprr
RGD1559575
Hebp2
Gna12
Phlda3
LOC681458
Hcn4
Sstr1
Illumina
Ion Torrent
Hippocampal region
CA1
CA3
DG

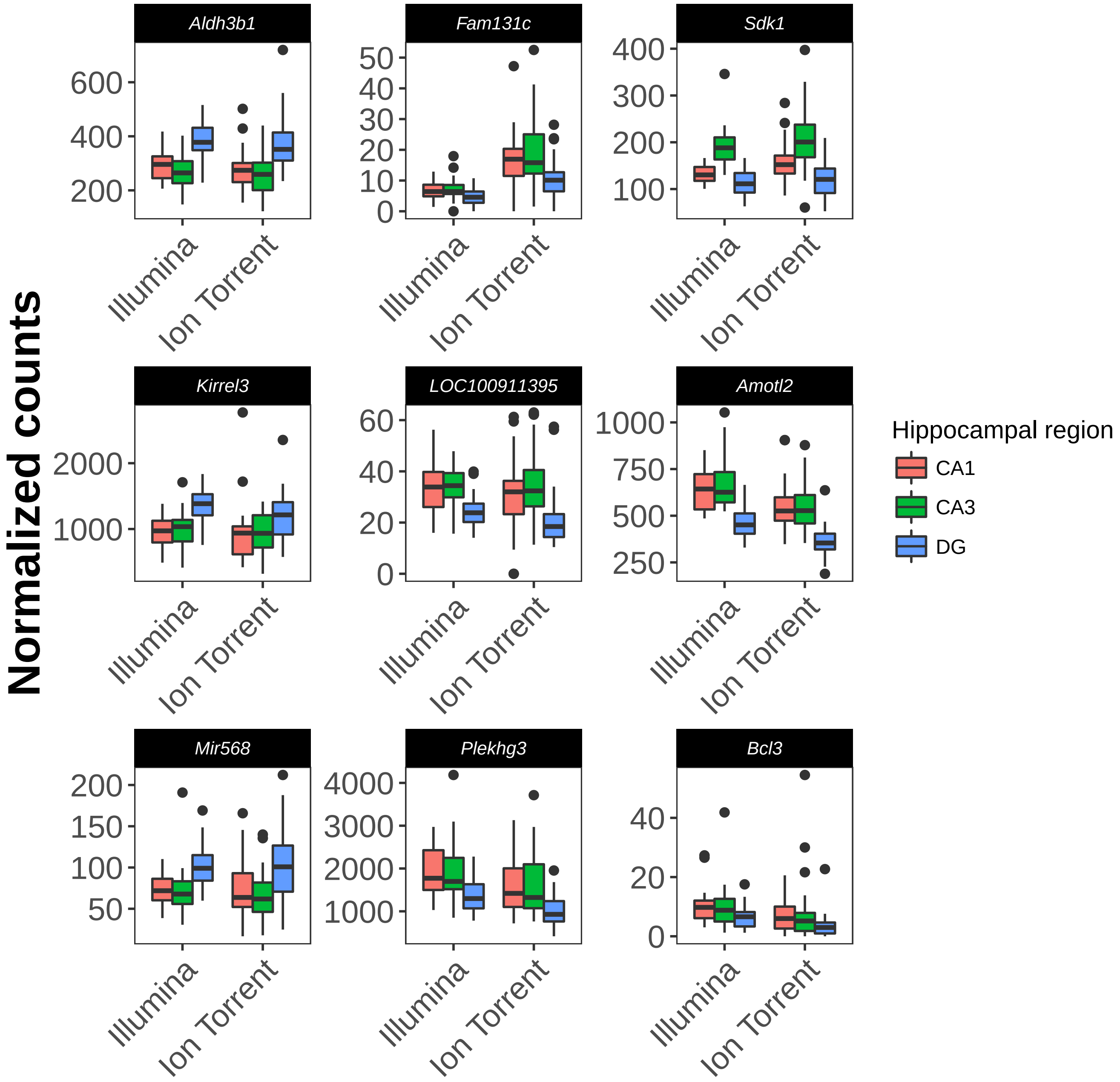

Normalized counts
Aldh3b1
Fam131c
Sdk1
Kirrel3
LOC100911395
Amotl2
Mir568
Plekhg3
Bcl3
Illumina
Ion Torrent
Hippocampal region
CA1
CA3
DG

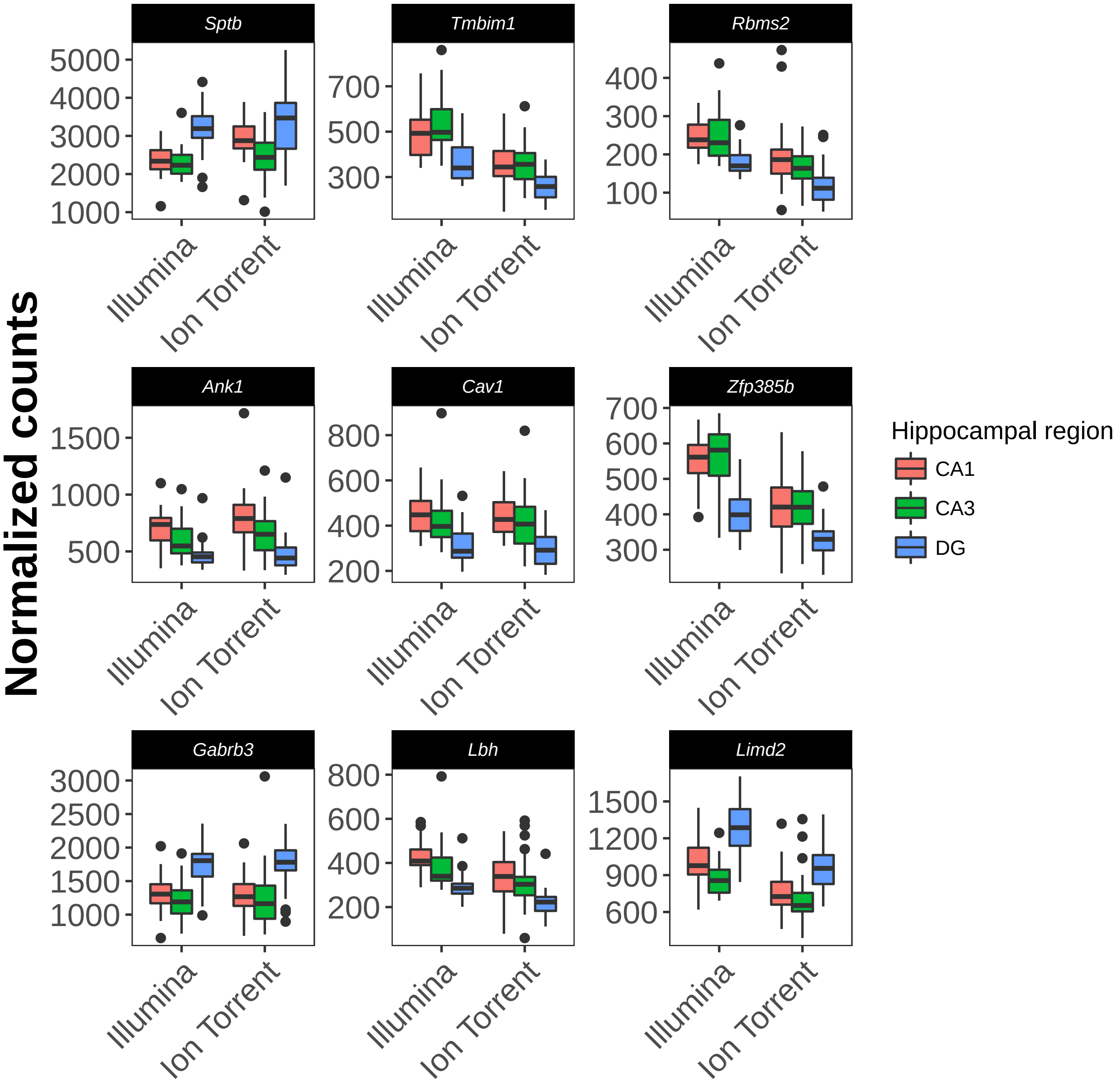
Normalized counts
Sptb
Tmbim1
Rbms2
Ank1
Cav1
Zfp385b
Gabrb3
Lbh
Limd2
Illumina
Ion Torrent
Hippocampal region
CA1
CA3
DG

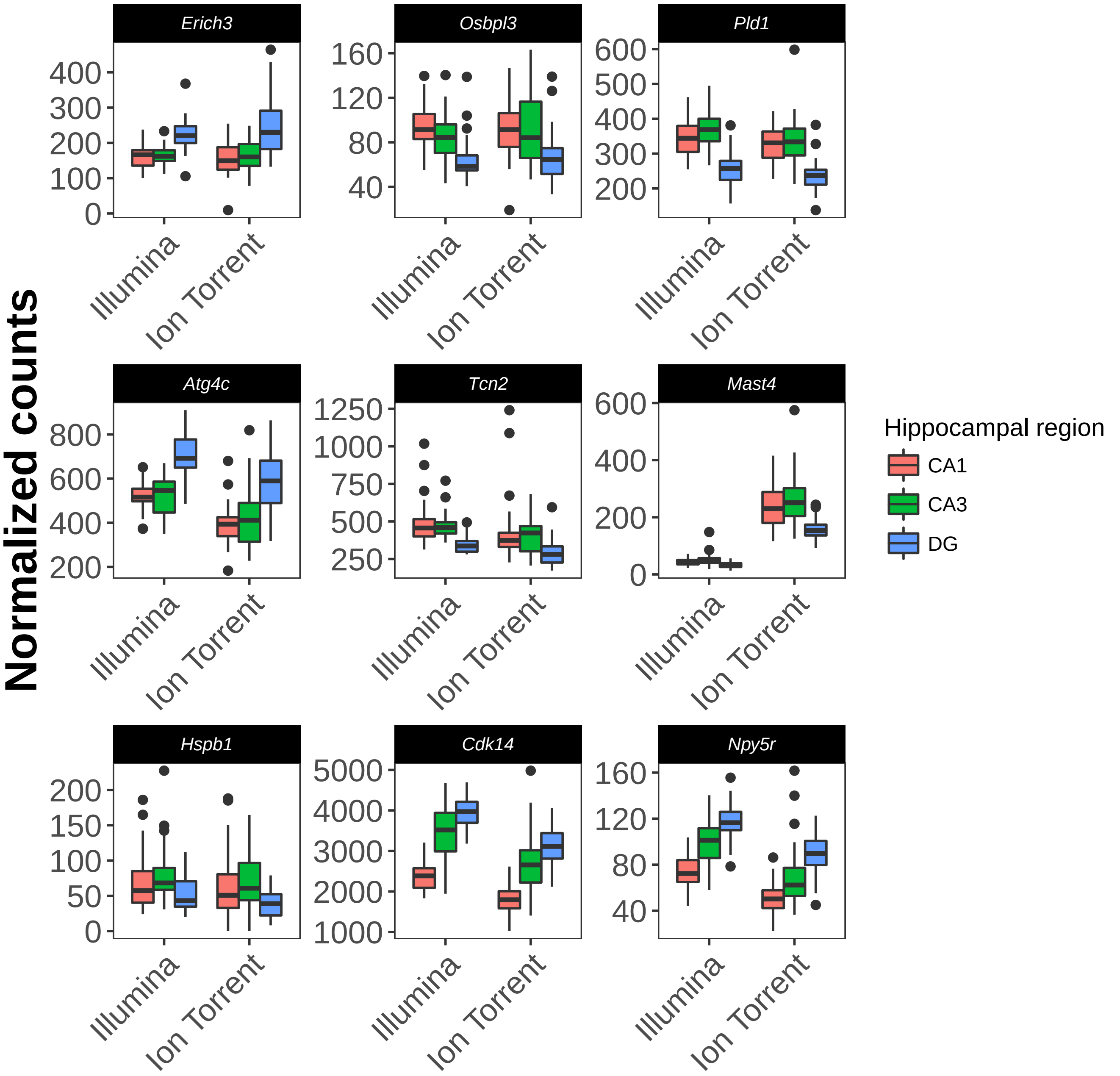
Normalized counts
Erich3
Osbpl3
Pld1
Atg4c
Tcn2
Mast4
Hspb1
Cdk14
Npy5r
Illumina
Ion Torrent
Hippocampal region
CA1
CA3
DG

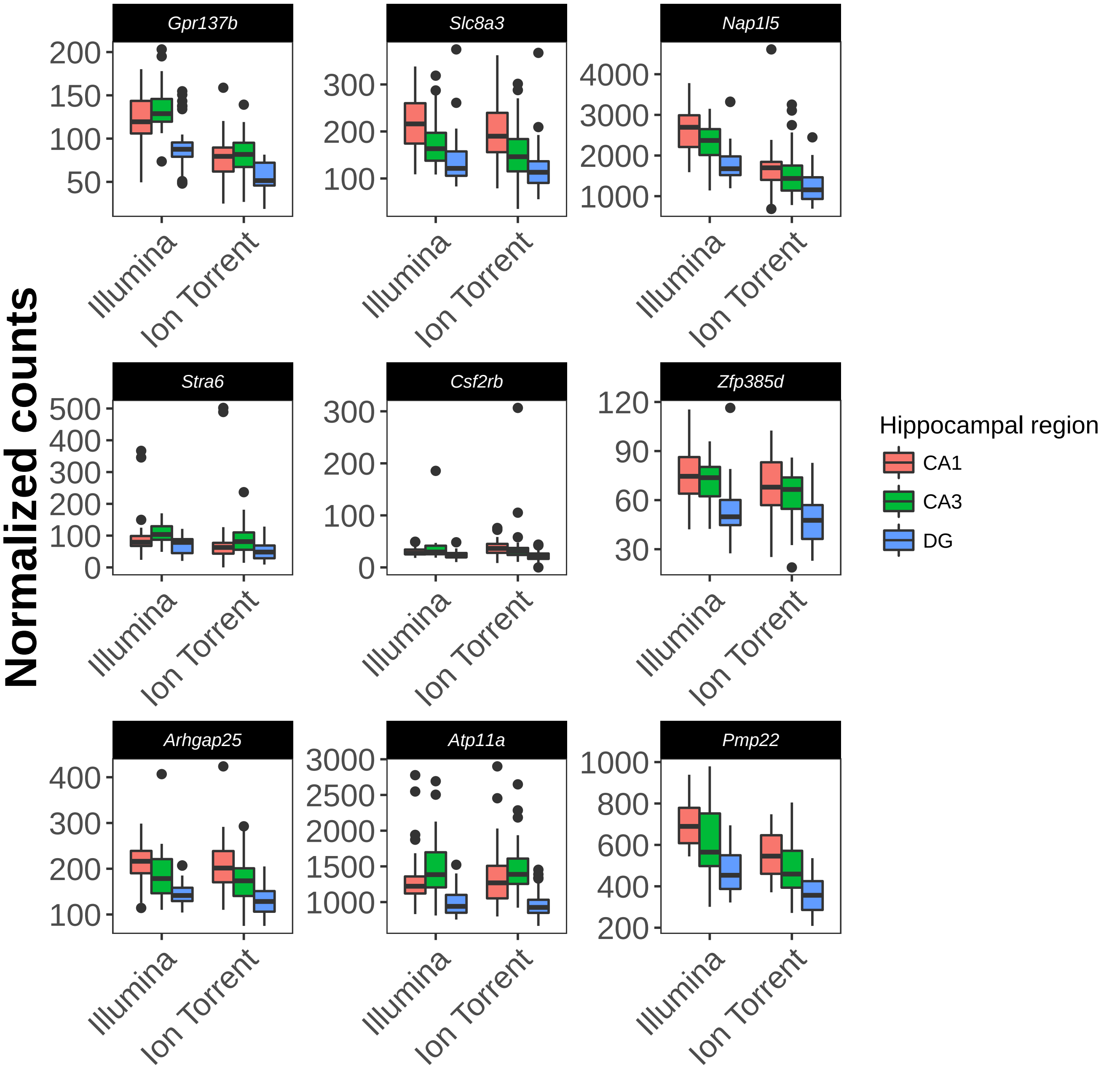

Normalized counts
Gpr137b
Slc8a3
Nap1l5
Stra6
Csf2rb
Zfp385d
Arhgap25
Atp11a
Pmp22
Illumina
Ion Torrent
Hippocampal region
CA1
CA3
DG

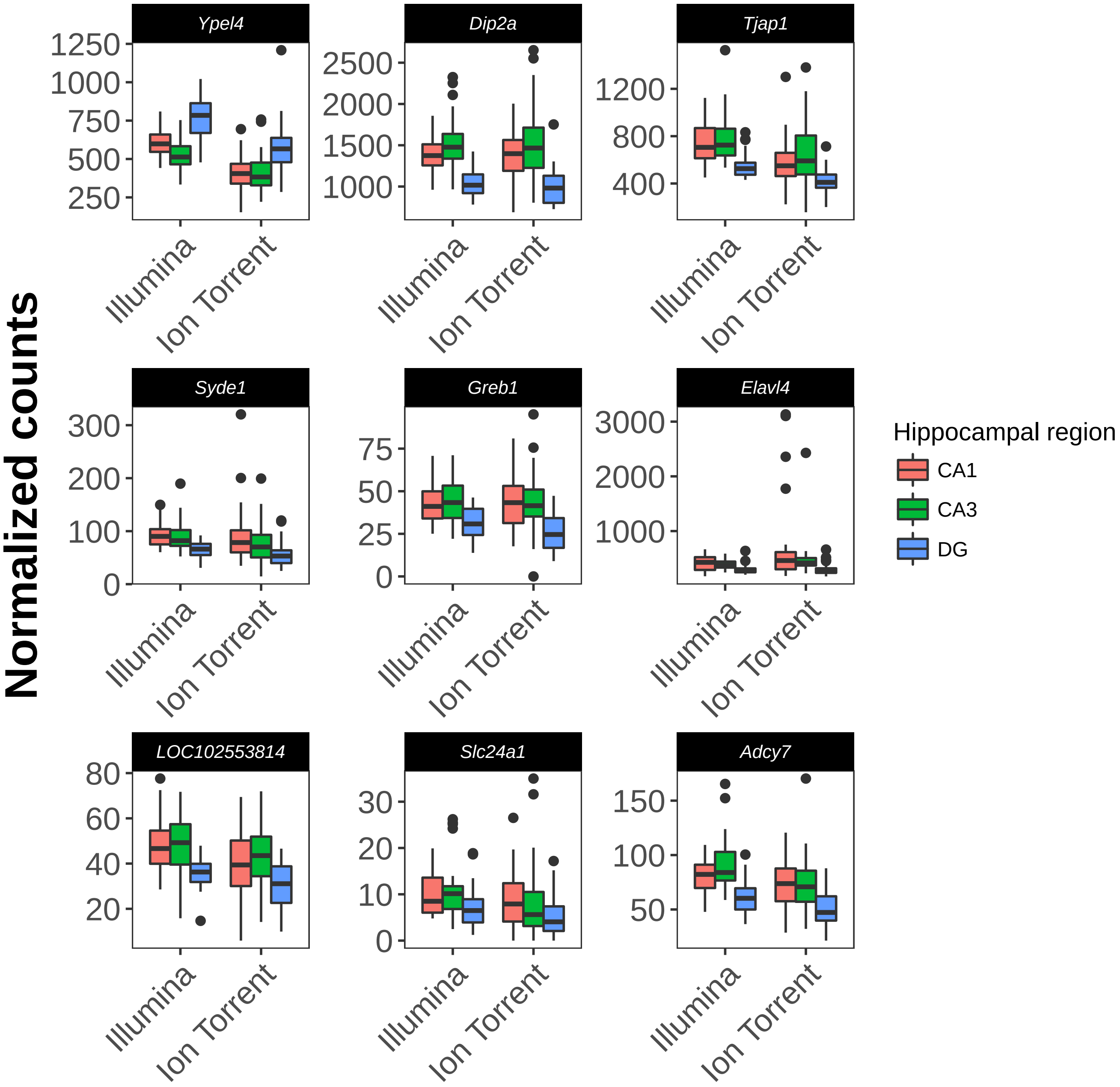
Normalized counts
Ypel4
Dip2a
Tjap1
Syde1
Greb1
Elavl4
LOC102553814
Slc24a1
Adcy7
Illumina
Ion Torrent
Hippocampal region
CA1
CA3
DG

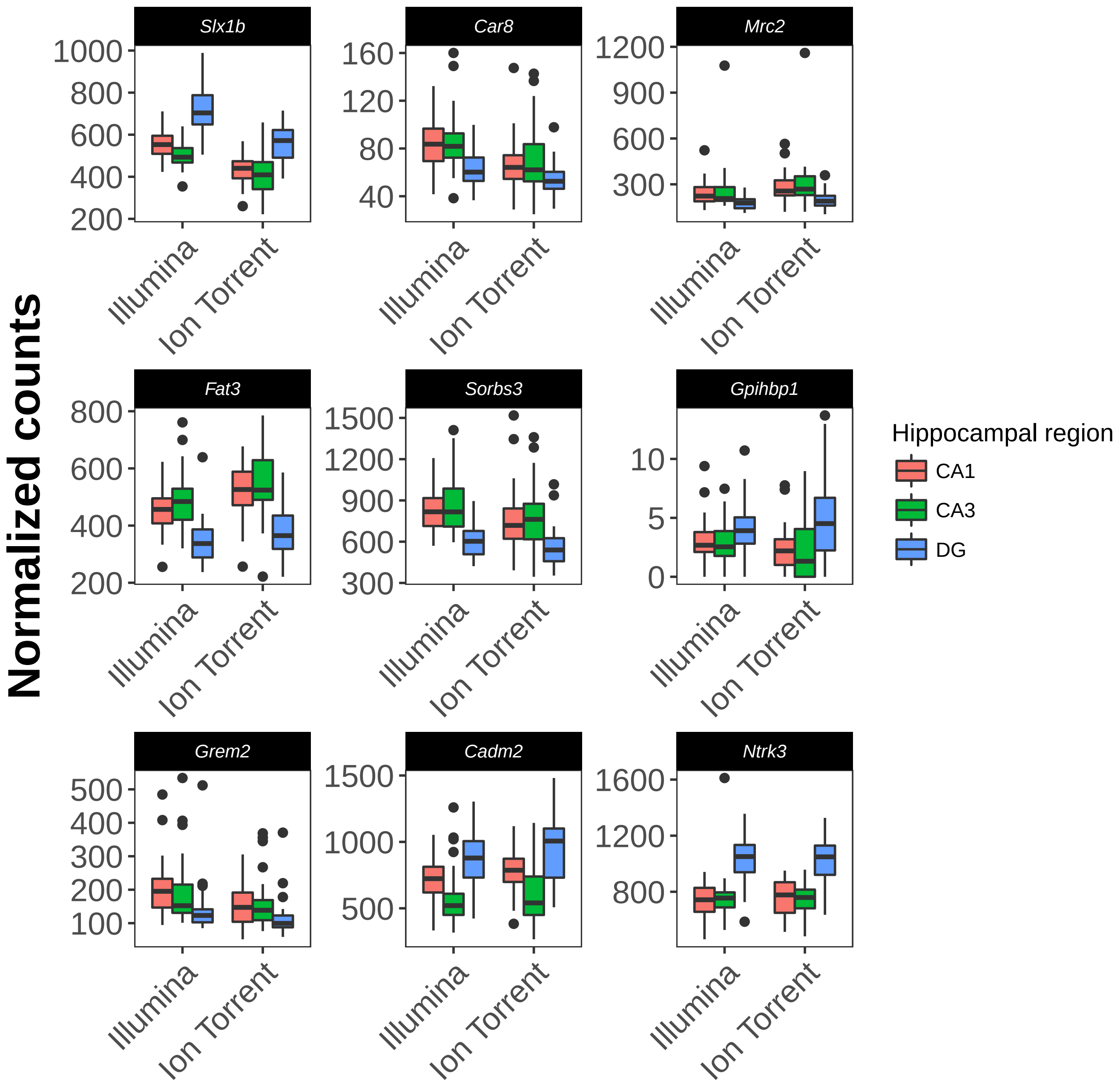
Normalized counts
Slx1b
Car8
Mrc2
Fat3
Sorbs3
Gpihbp1
Grem2
Cadm2
Ntrk3
Illumina
Ion Torrent
Hippocampal region
CA1
CA3
DG

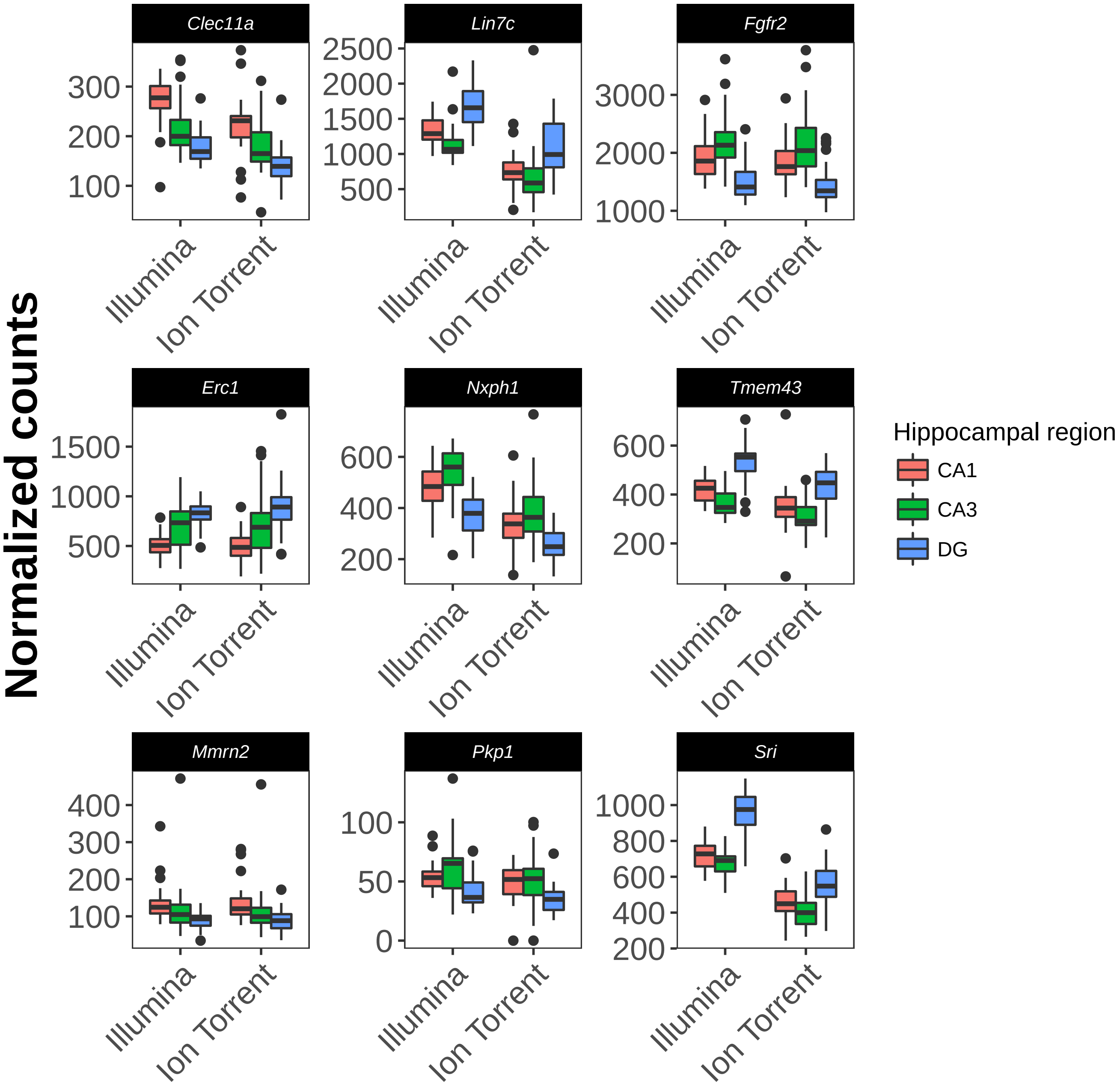

Normalized counts
Clec11a
Lin7c
Fgfr2
Erc1
Nxph1
Tmem43
Mmrn2
Pkp1
Sri
Illumina
Ion Torrent
Hippocampal region
CA1
CA3
DG

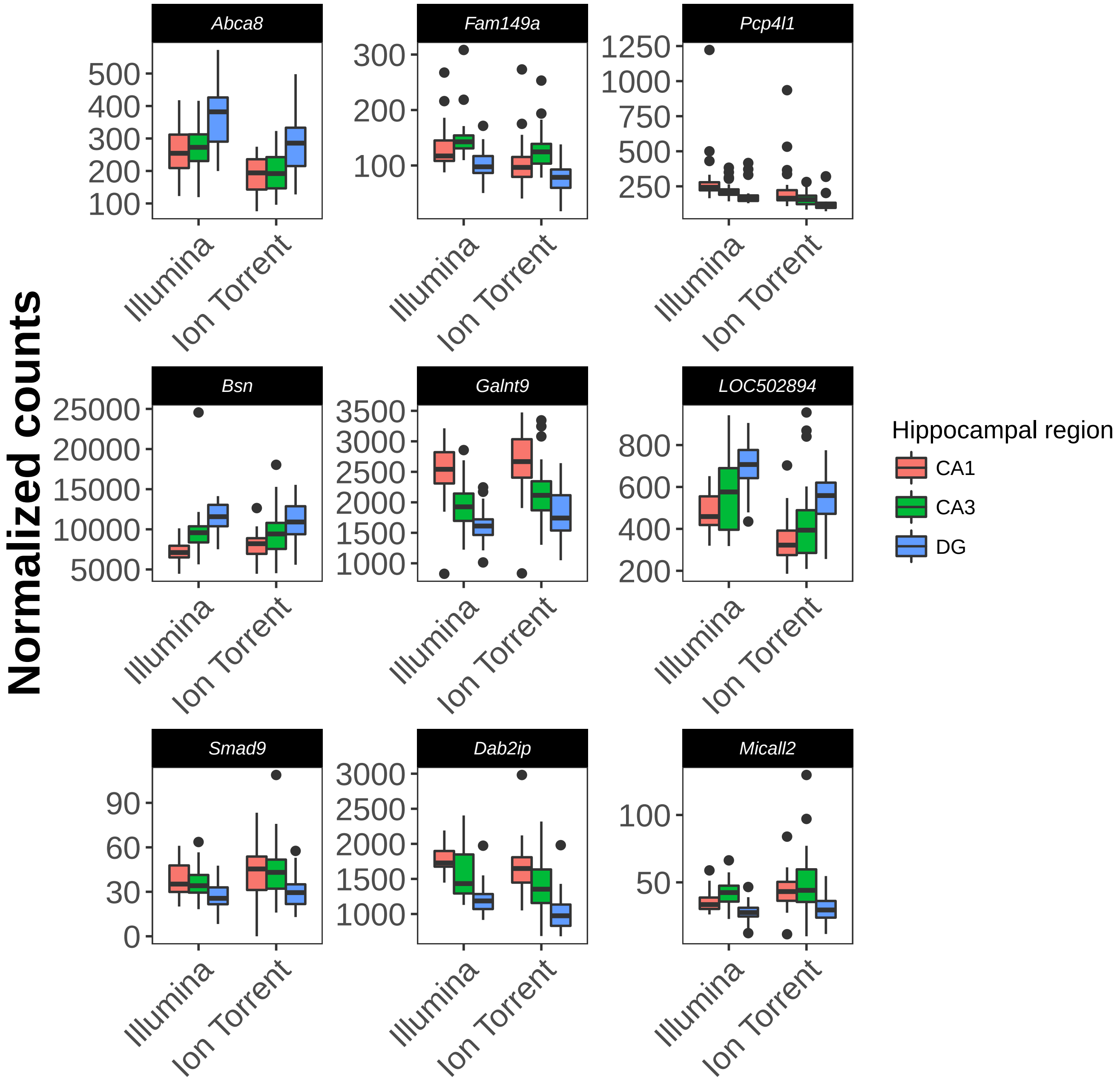

Abca8
Fam149a
Pcp4l1
Bsn
Galnt9
LOC502894
Smad9
Dab2ip
Micall2
Illumina
Ion Torrent
Normalized counts
Hippocampal region
CA1
CA3
DG

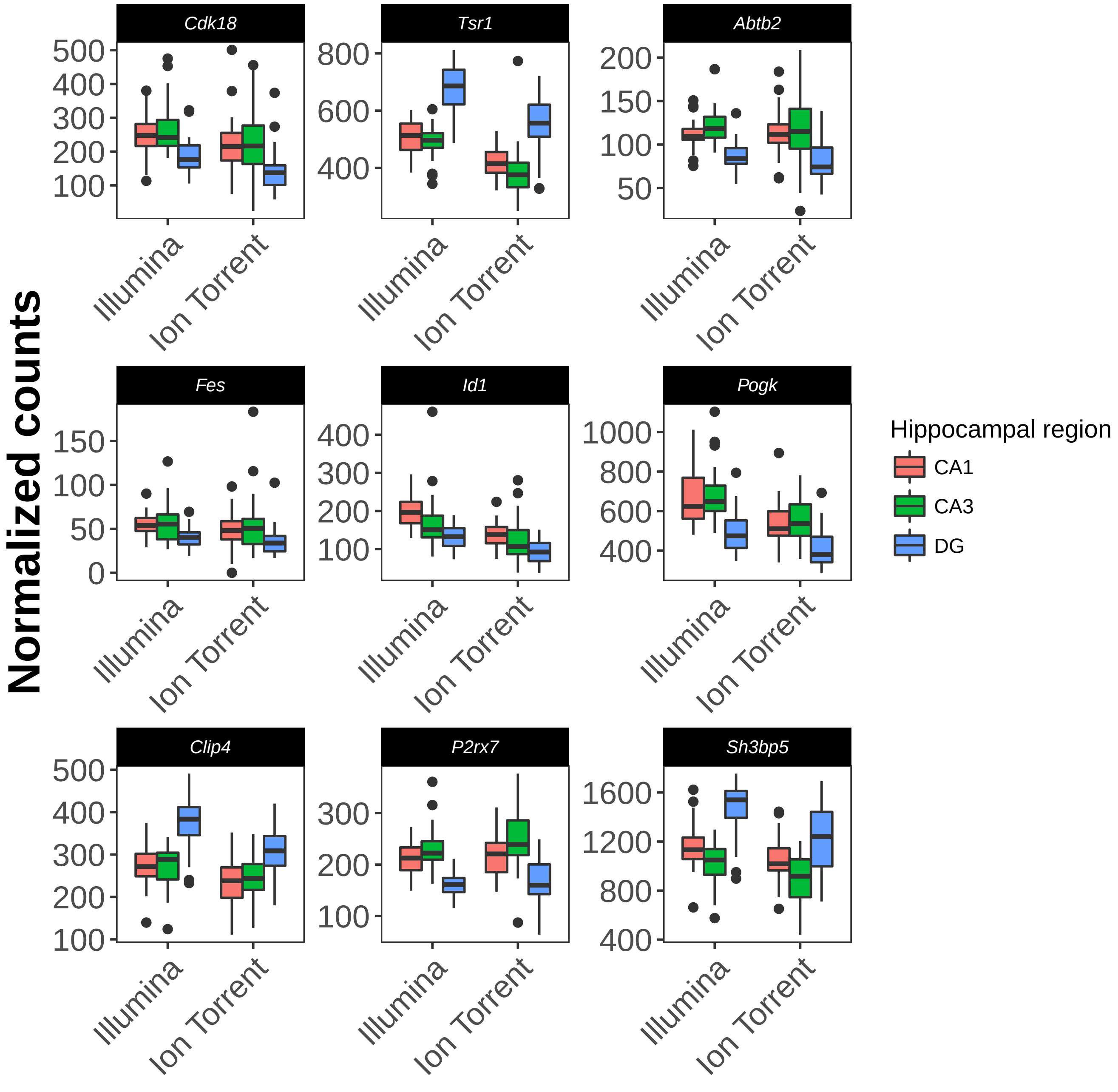
Normalized counts
Cdk18
Tsr1
Abtb2
Fes
Id1
Pogk
Clip4
P2rx7
Sh3bp5
Illumina
Ion Torrent
Hippocampal region
CA1
CA3
DG

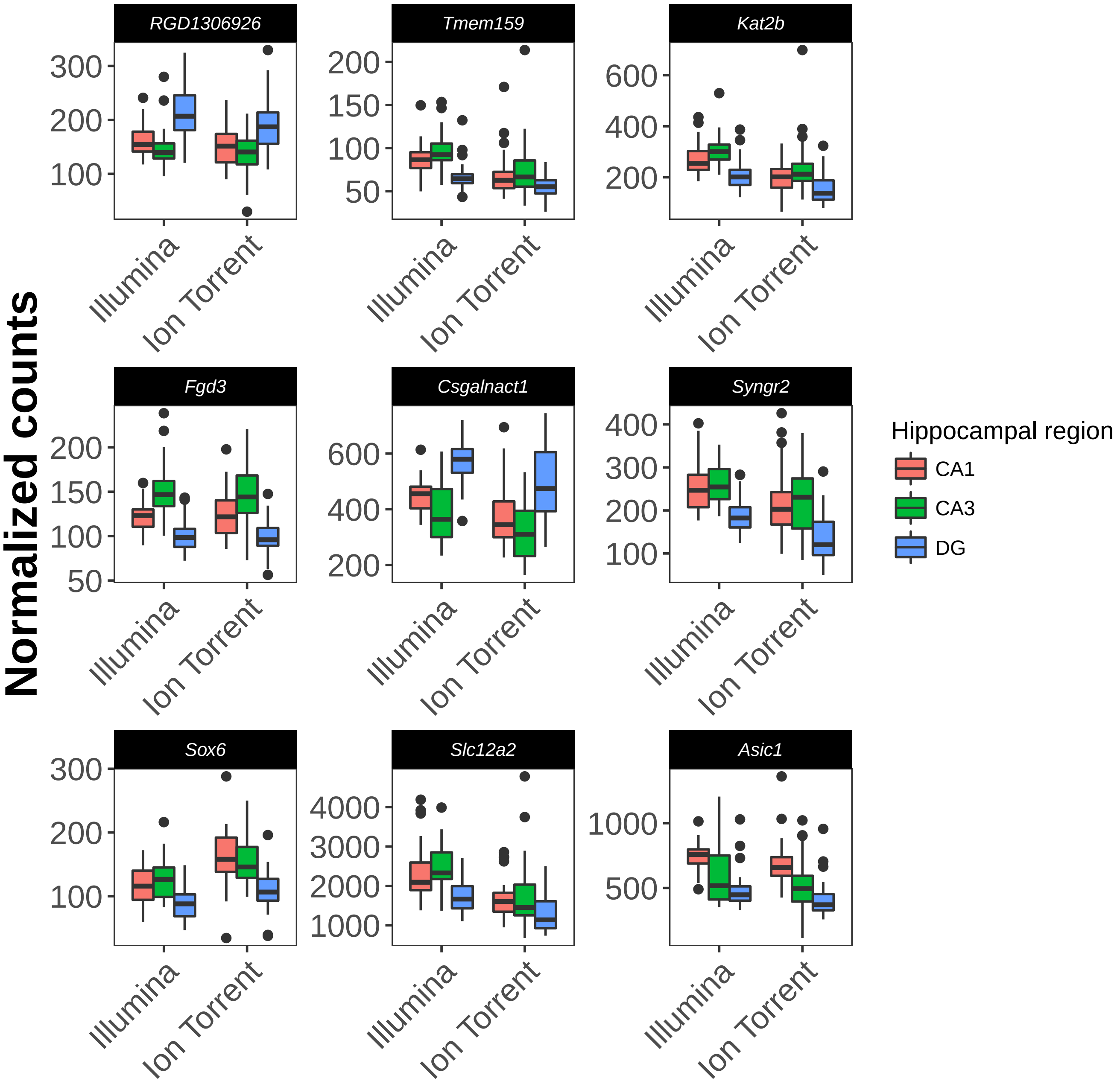

Normalized counts
RGD1306926
Tmem159
Kat2b
Fgd3
Csgalnact1
Syngr2
Sox6
Slc12a2
Asic1
Illumina
Ion Torrent
Hippocampal region
CA1
CA3
DG

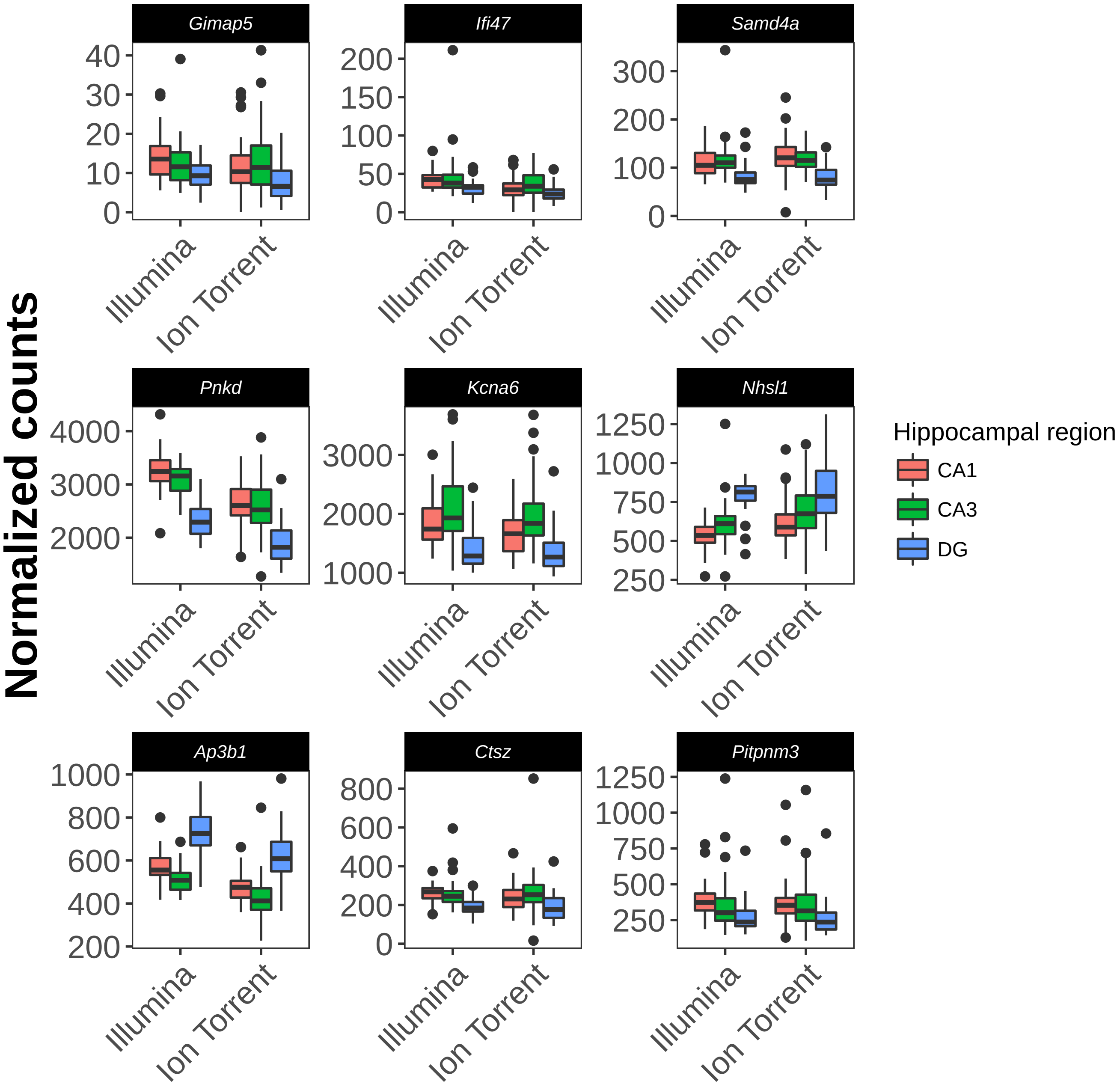
Normalized counts
Gimap5
Ifi47
Samd4a
Pnkd
Kcna6
Nhsl1
Ap3b1
Ctsz
Pitpnm3
Illumina
Ion Torrent
Hippocampal region
CA1
CA3
DG

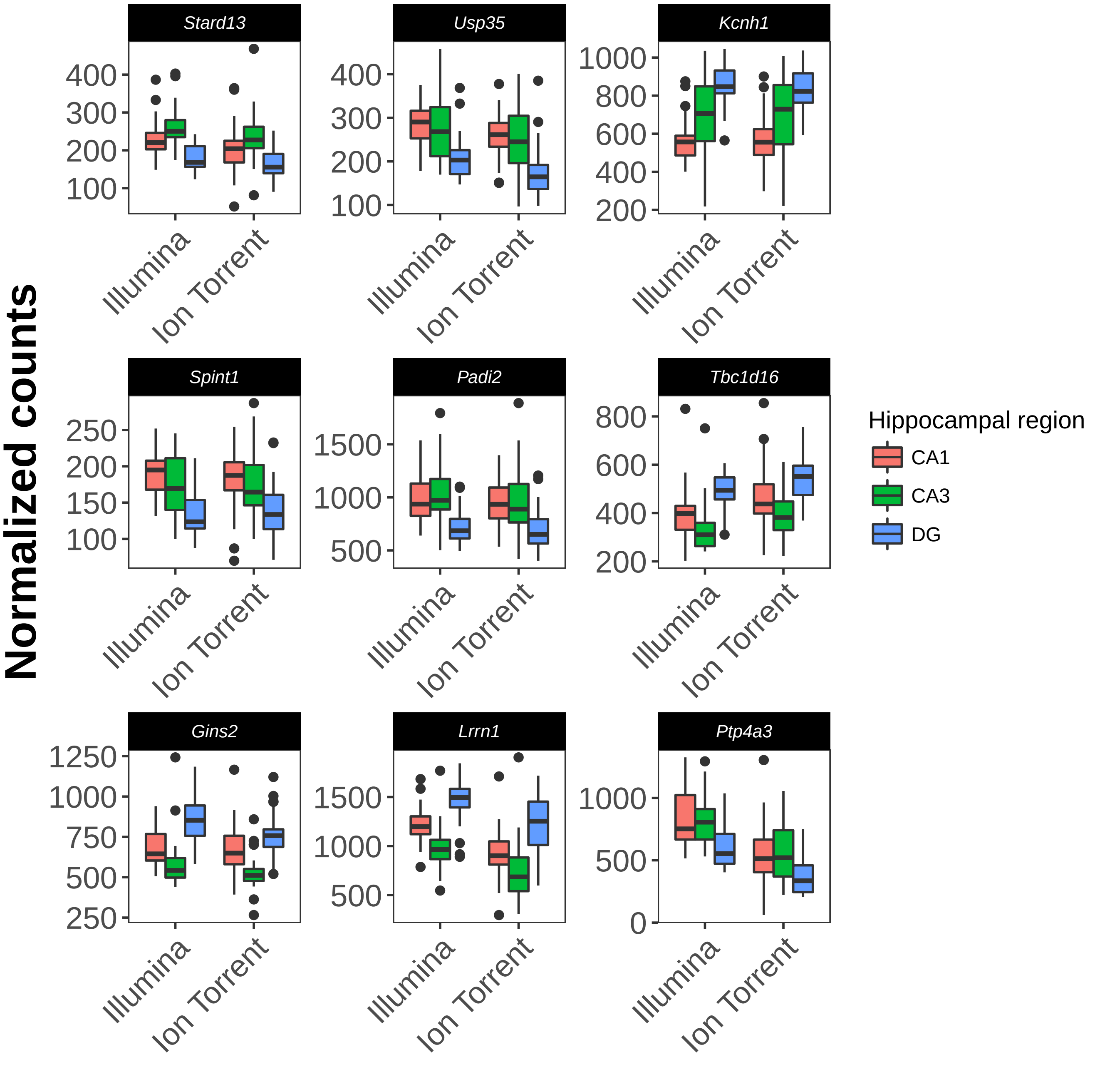
Normalized counts
Stard13
Usp35
Kcnh1
Spint1
Padi2
Tbc1d16
Gins2
Lrrn1
Ptp4a3
Illumina
Ion Torrent
Hippocampal region
CA1
CA3
DG

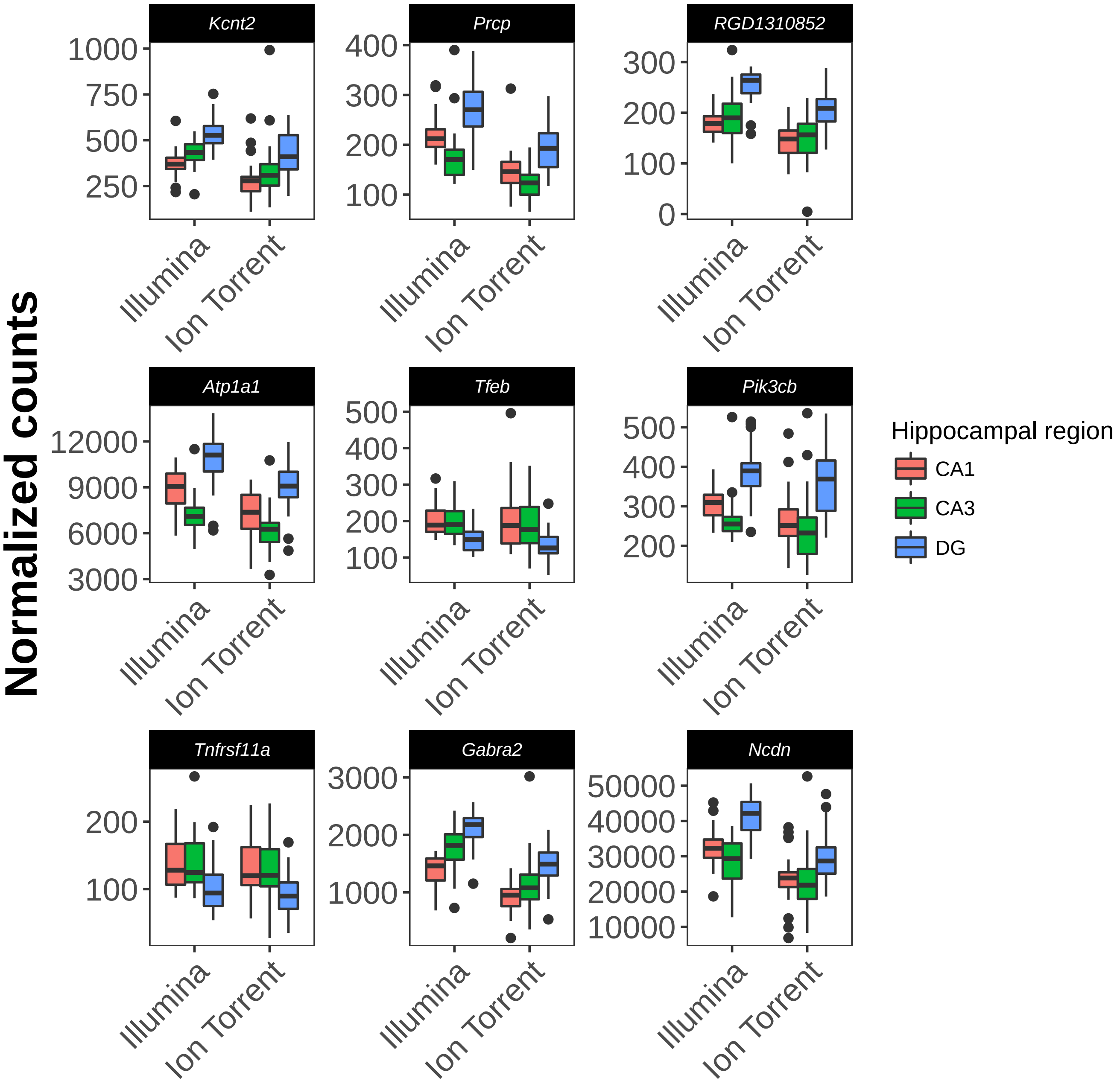
Normalized counts
Kcnt2
Prcp
RGD1310852
Atp1a1
Tfeb
Pik3cb
Tnfrsf11a
Gabra2
Ncdn
Illumina
Ion Torrent
Hippocampal region
CA1
CA3
DG

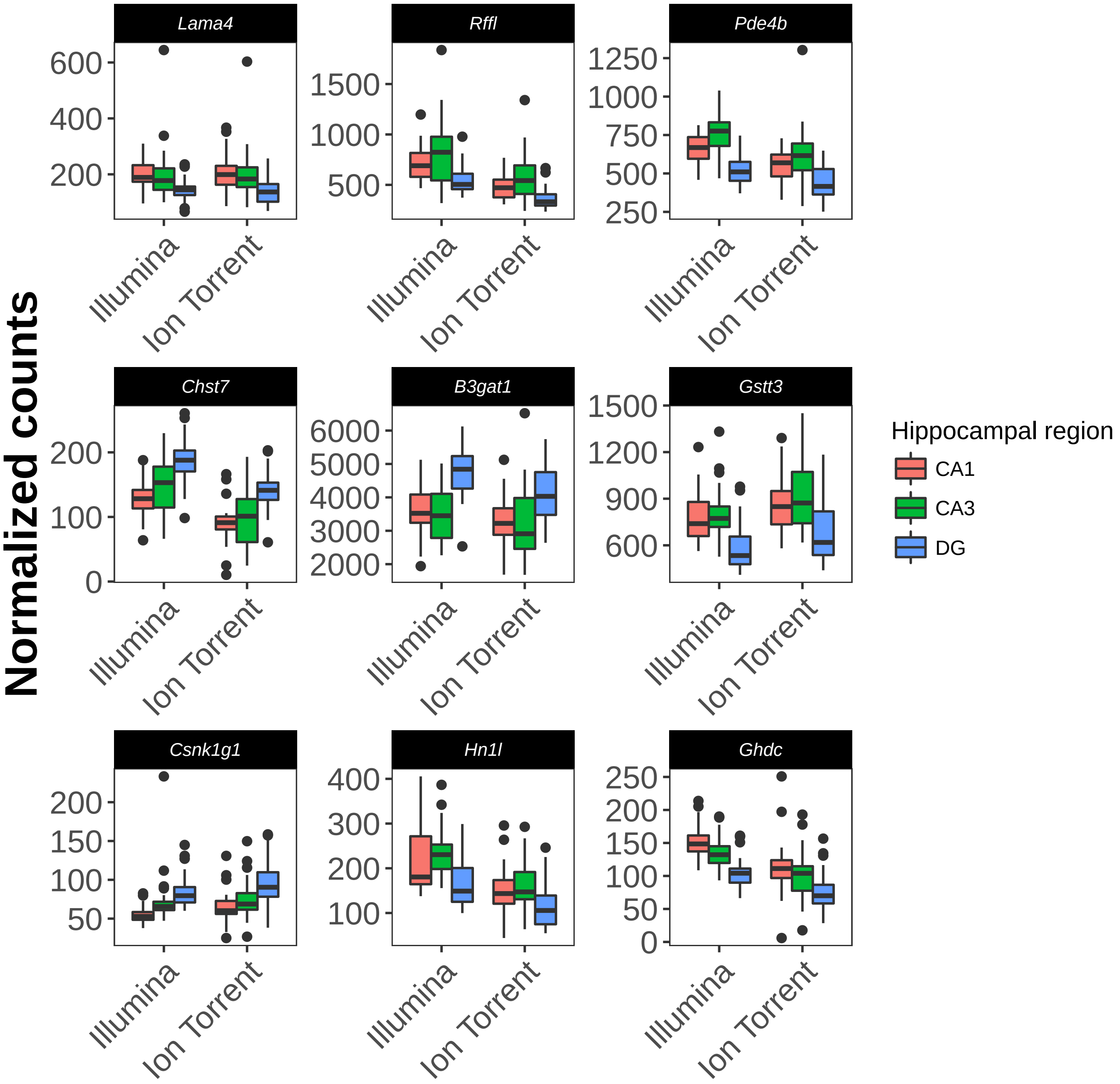

Normalized counts
Lama4
Rffl
Pde4b
Chst7
B3gat1
Gstt3
Csnk1g1
Hn1l
Ghdc
Illumina
Ion Torrent
Hippocampal region
CA1
CA3
DG

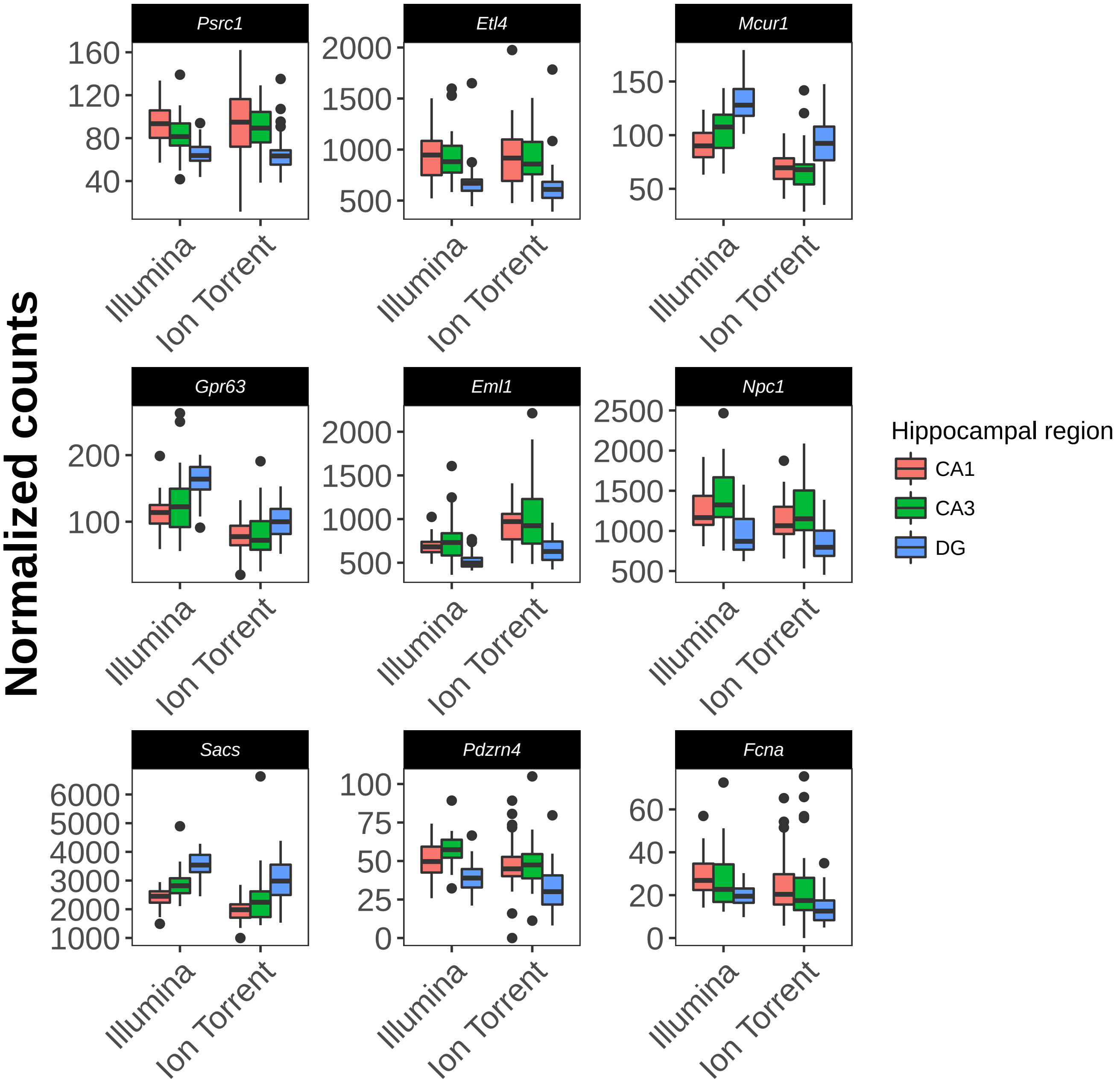

Normalized counts
Psrc1
Etl4
Mcur1
Gpr63
Eml1
Npc1
Sacs
Pdzrn4
Fcna
Illumina
Ion Torrent
Hippocampal region
CA1
CA3
DG

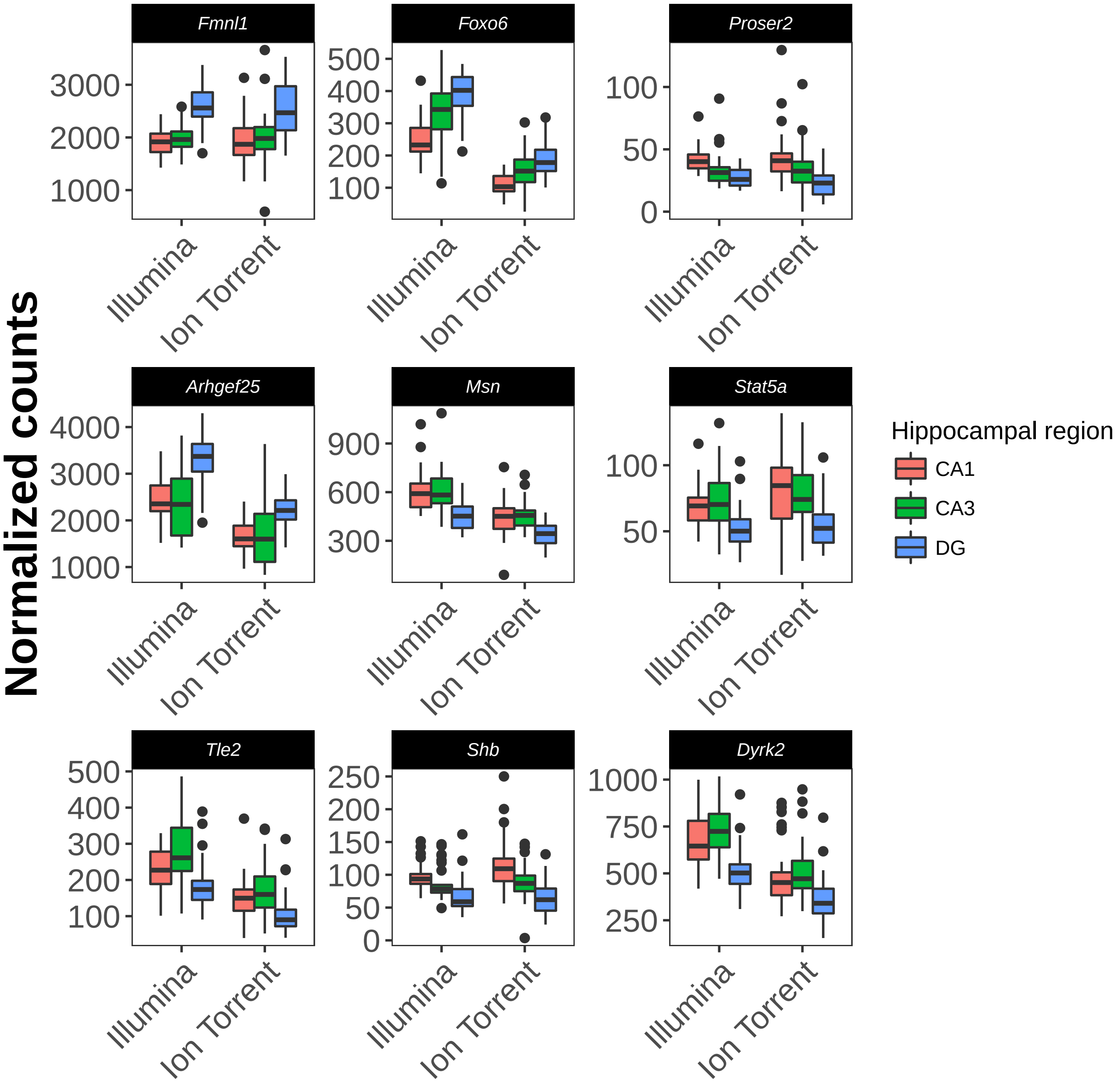
Normalized counts
Fmnl1
Foxo6
Proser2
Arhgef25
Msn
Stat5a
Tle2
Shb
Dyrk2
Illumina
Ion Torrent
Hippocampal region
CA1
CA3
DG

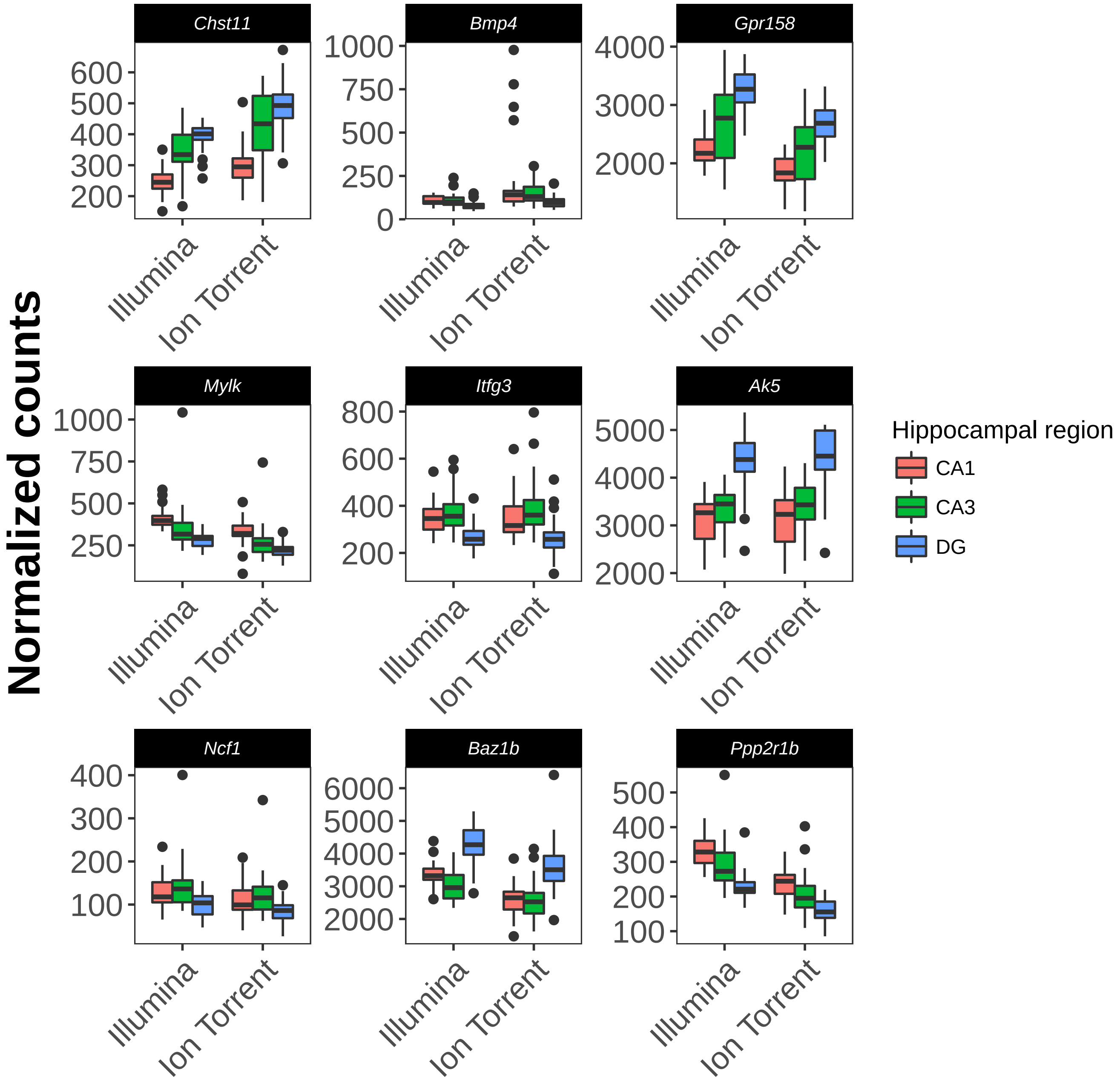

Normalized counts
Chst11
Bmp4
Gpr158
Mylk
Itfg3
Ak5
Ncf1
Baz1b
Ppp2r1b
Illumina
Ion Torrent
Hippocampal region
CA1
CA3
DG

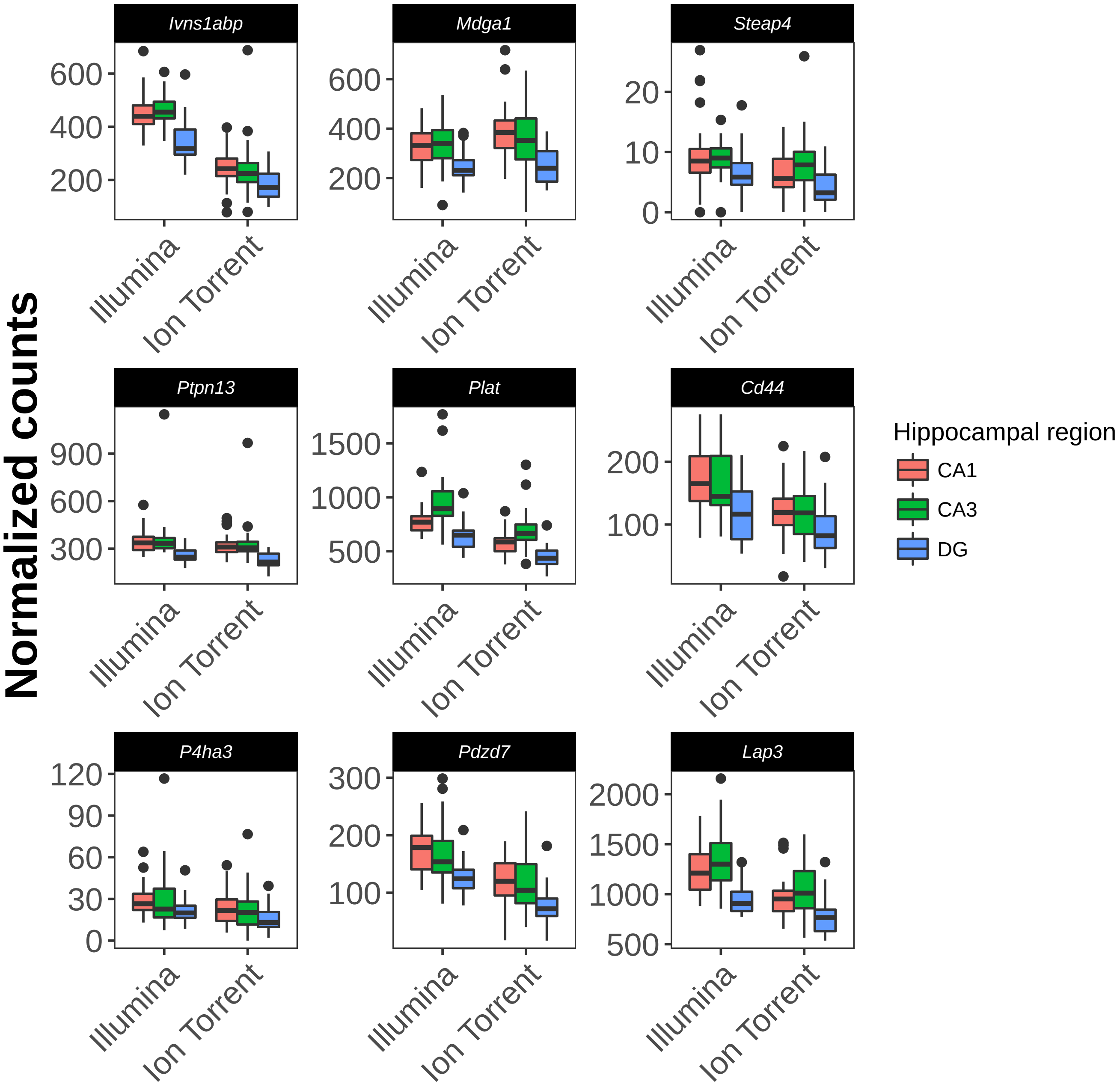
Normalized counts
Ivns1abp
Mdga1
Steap4
Ptpn13
Plat
Cd44
P4ha3
Pdzd7
Lap3
Illumina
Ion Torrent
Hippocampal region
CA1
CA3
DG

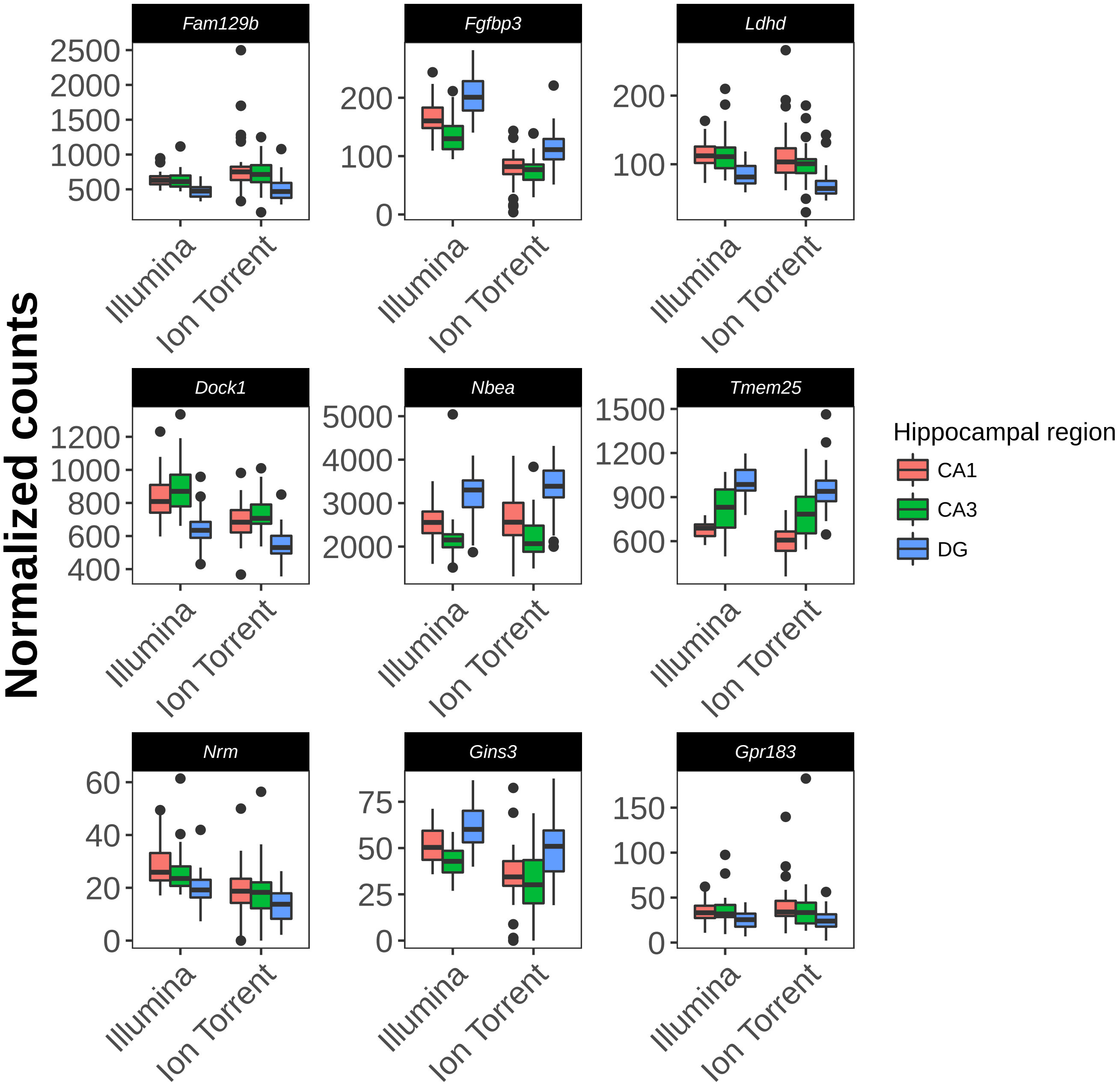
Normalized counts
Fam129b
Fgfbp3
Ldhd
Dock1
Nbea
Tmem25
Nrm
Gins3
Gpr183
Illumina
Ion Torrent
Hippocampal region
CA1
CA3
DG

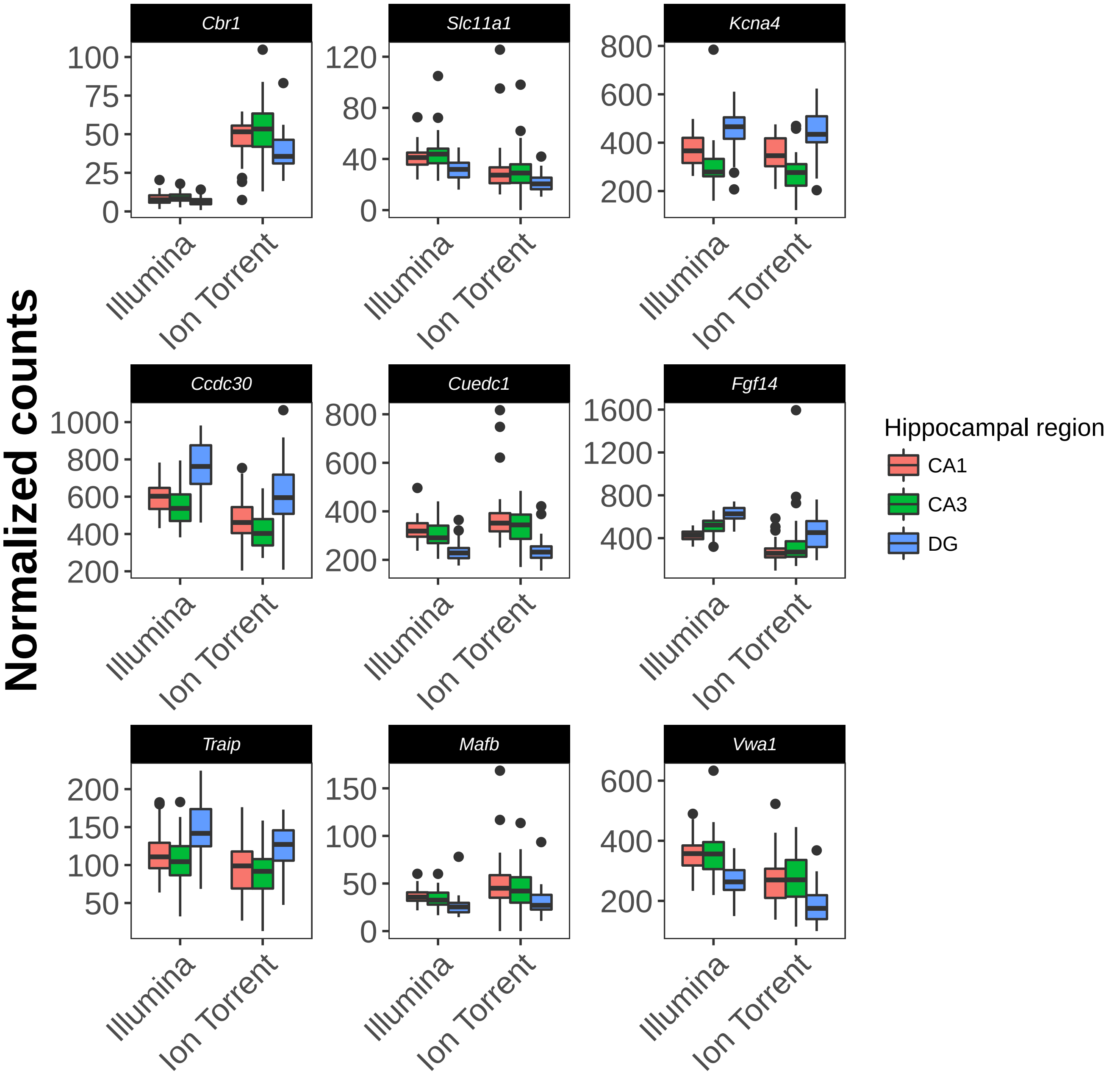
Normalized counts
Cbr1
Slc11a1
Kcna4
Ccdc30
Cuedc1
Fgf14
Traip
Mafb
Vwa1
Illumina
Ion Torrent
Hippocampal region
CA1
CA3
DG

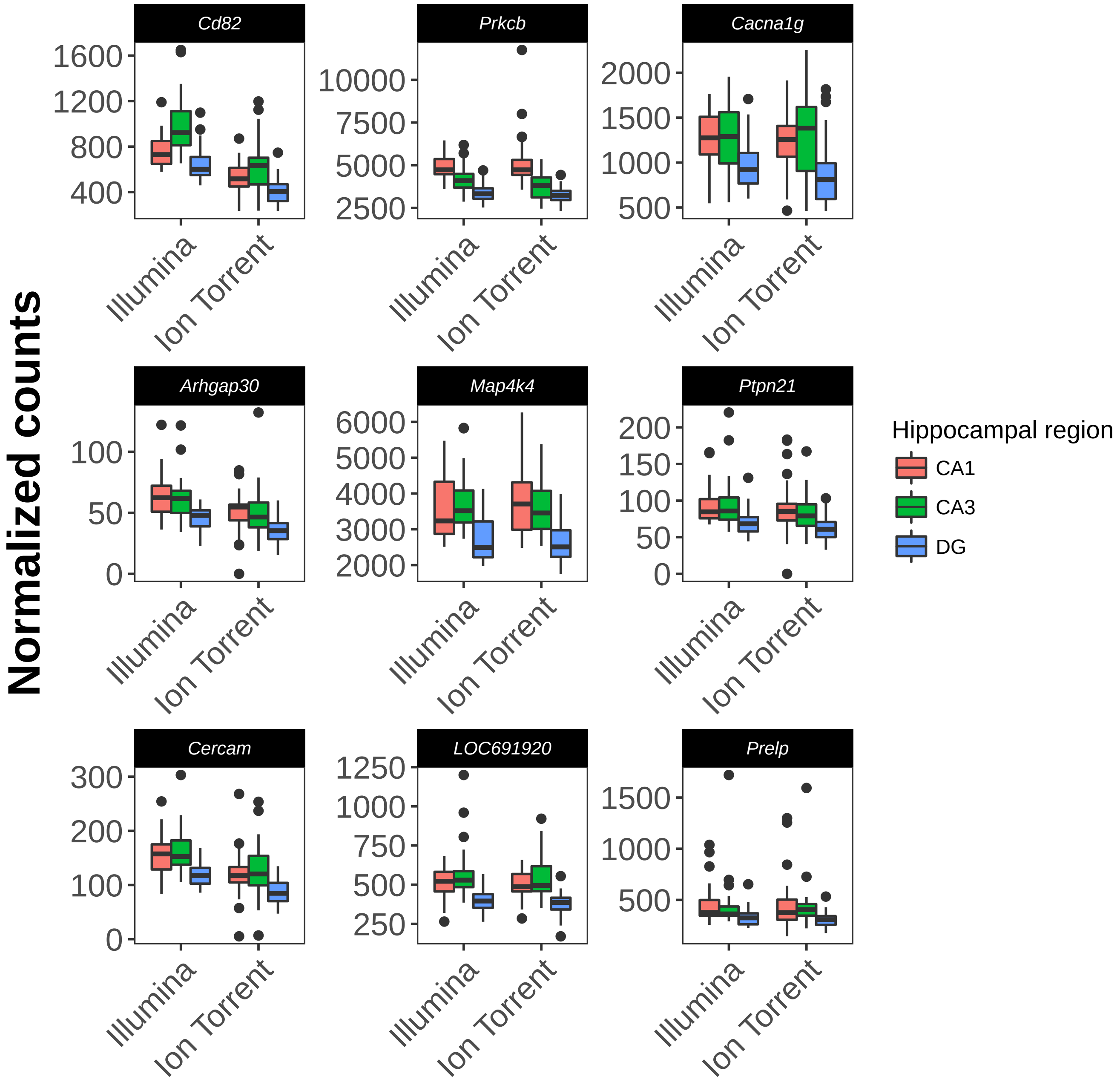
Normalized counts
Cd82
1600
1200
800
400
Prkcb
10000
7500
5000
2500
Cacna1g
2000
1500
1000
500
Arhgap30
100
50
0
Map4k4
6000
5000
4000
3000
2000
Ptpn21
200
150
100
50
0
Cercam
300
200
100
0
LOC691920
1250
1000
750
500
250
Prelp
1500
1000
500
Illumina
Ion Torrent
Hippocampal region
CA1
CA3
DG

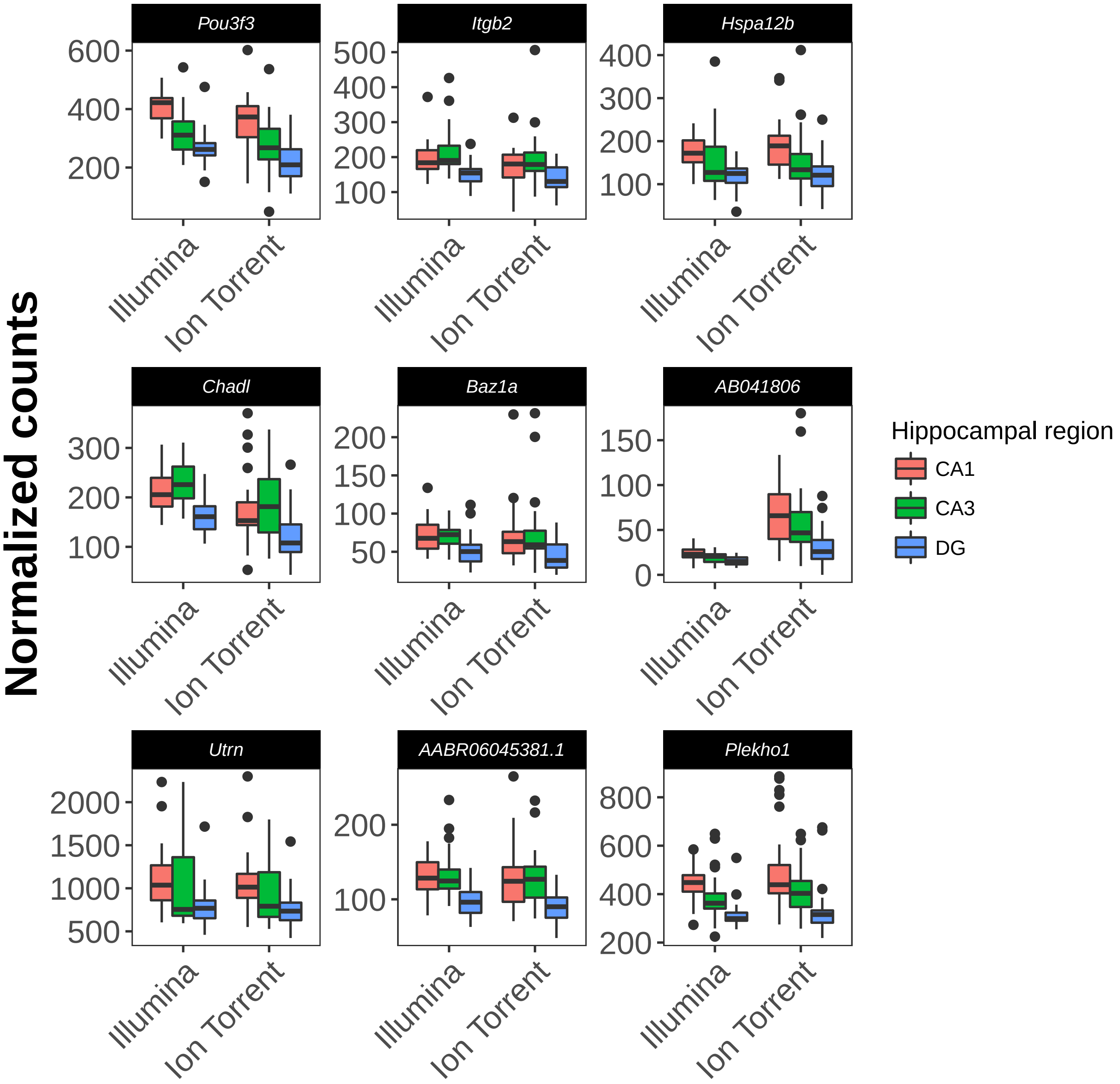

Normalized counts
Pou3f3
Itgb2
Hspa12b
Chadl
Baz1a
AB041806
Utrn
AABR06045381.1
Plekho1
Illumina
Ion Torrent
Hippocampal region
CA1
CA3
DG

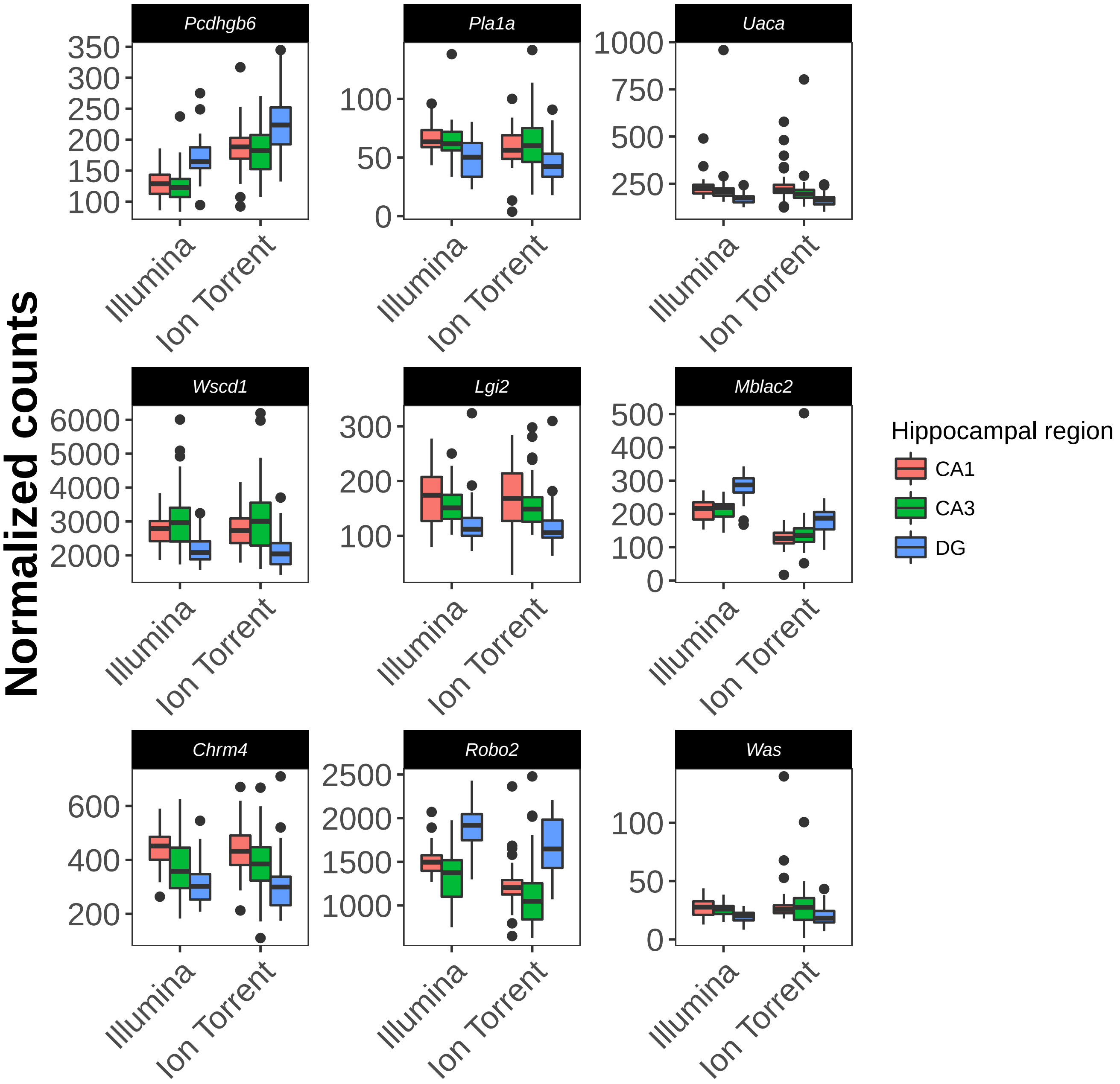

Normalized counts
Pcdhgb6
Pla1a
Uaca
Wscd1
Lgi2
Mblac2
Chrm4
Robo2
Was
Illumina
Ion Torrent
Hippocampal region
CA1
CA3
DG

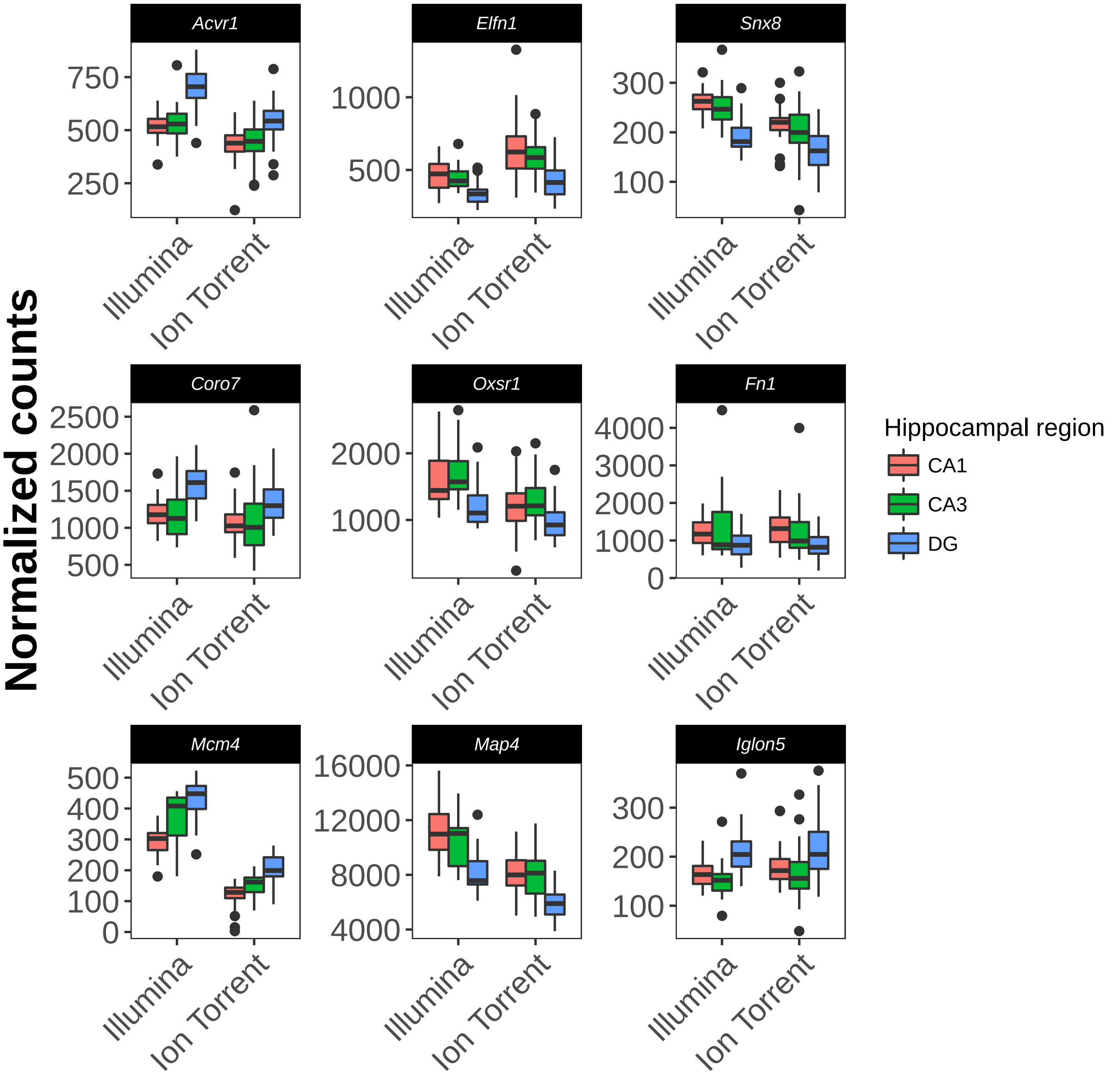

Normalized counts
Acvr1
Elfn1
Snx8
Coro7
Oxsr1
Fn1
Mcm4
Map4
Iglon5
Illumina
Ion Torrent
Hippocampal region
CA1
CA3
DG

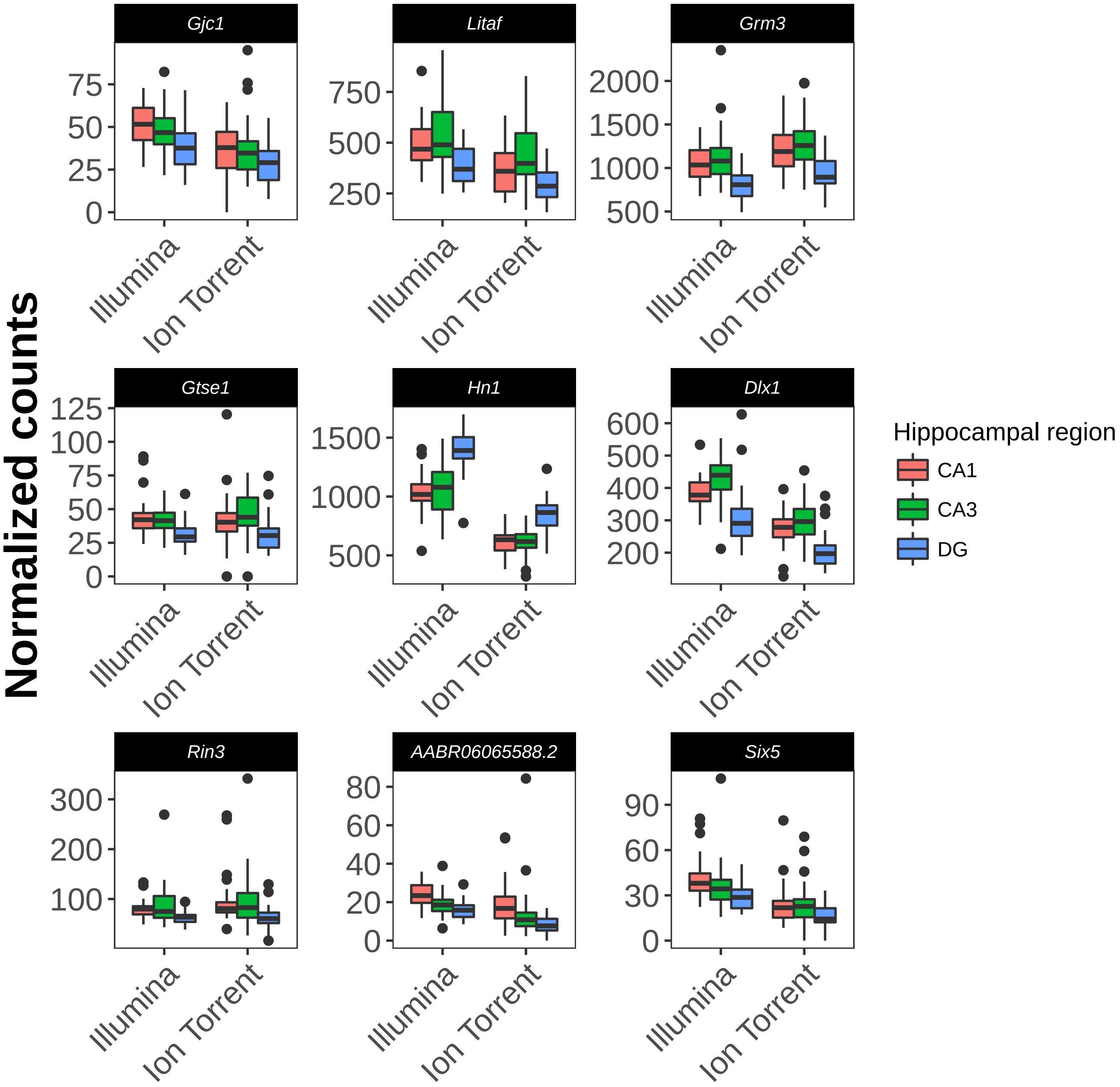

Normalized counts
Gjc1
Litaf
Grm3
Gtse1
Hn1
Dlx1
Rin3
AABR06065588.2
Six5
Illumina
Ion Torrent
Hippocampal region
CA1
CA3
DG

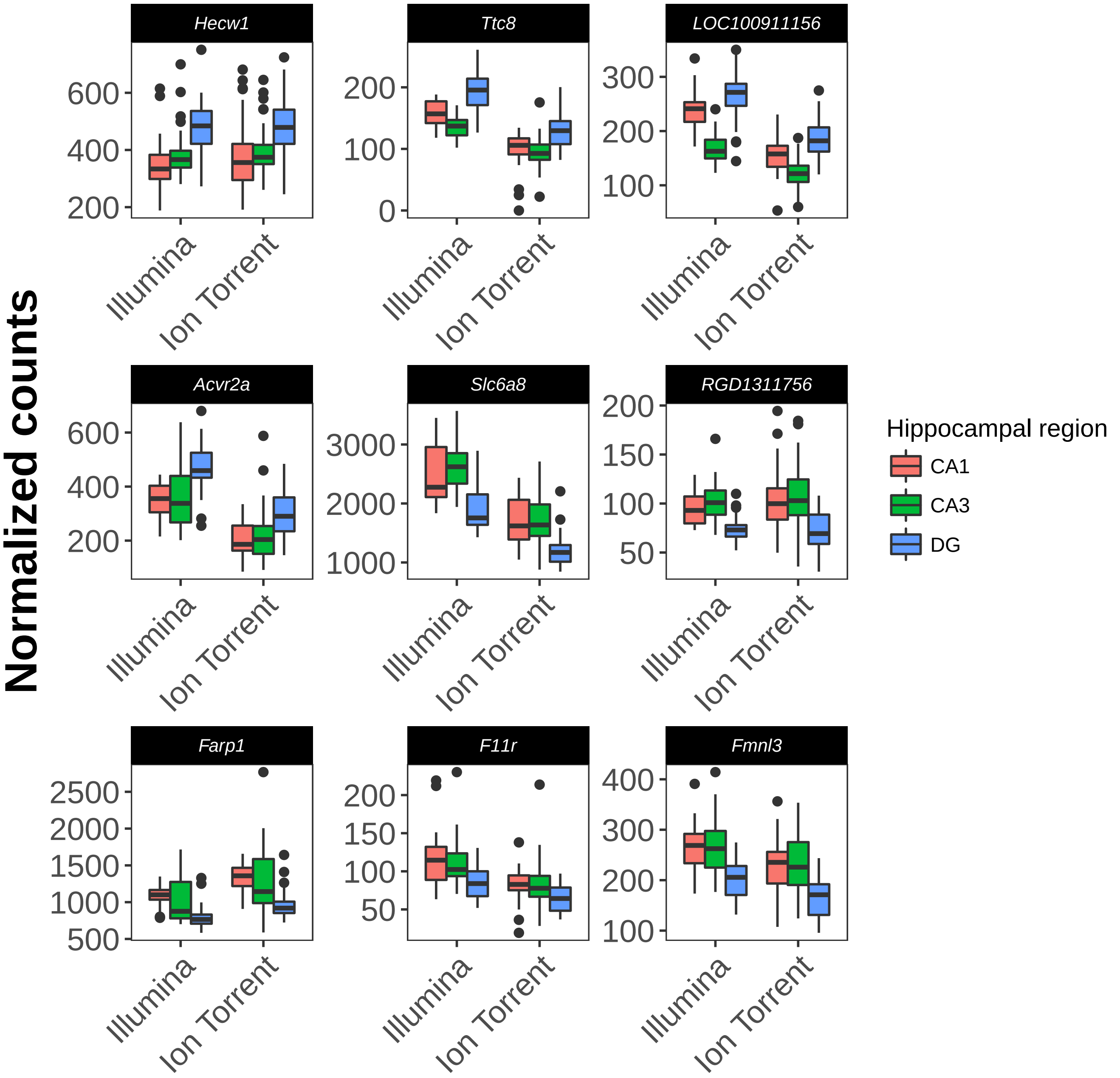
Normalized counts
Hecw1
Ttc8
LOC100911156
Acvr2a
Slc6a8
RGD1311756
Farp1
F11r
Fmnl3
Illumina
Ion Torrent
Hippocampal region
CA1
CA3
DG

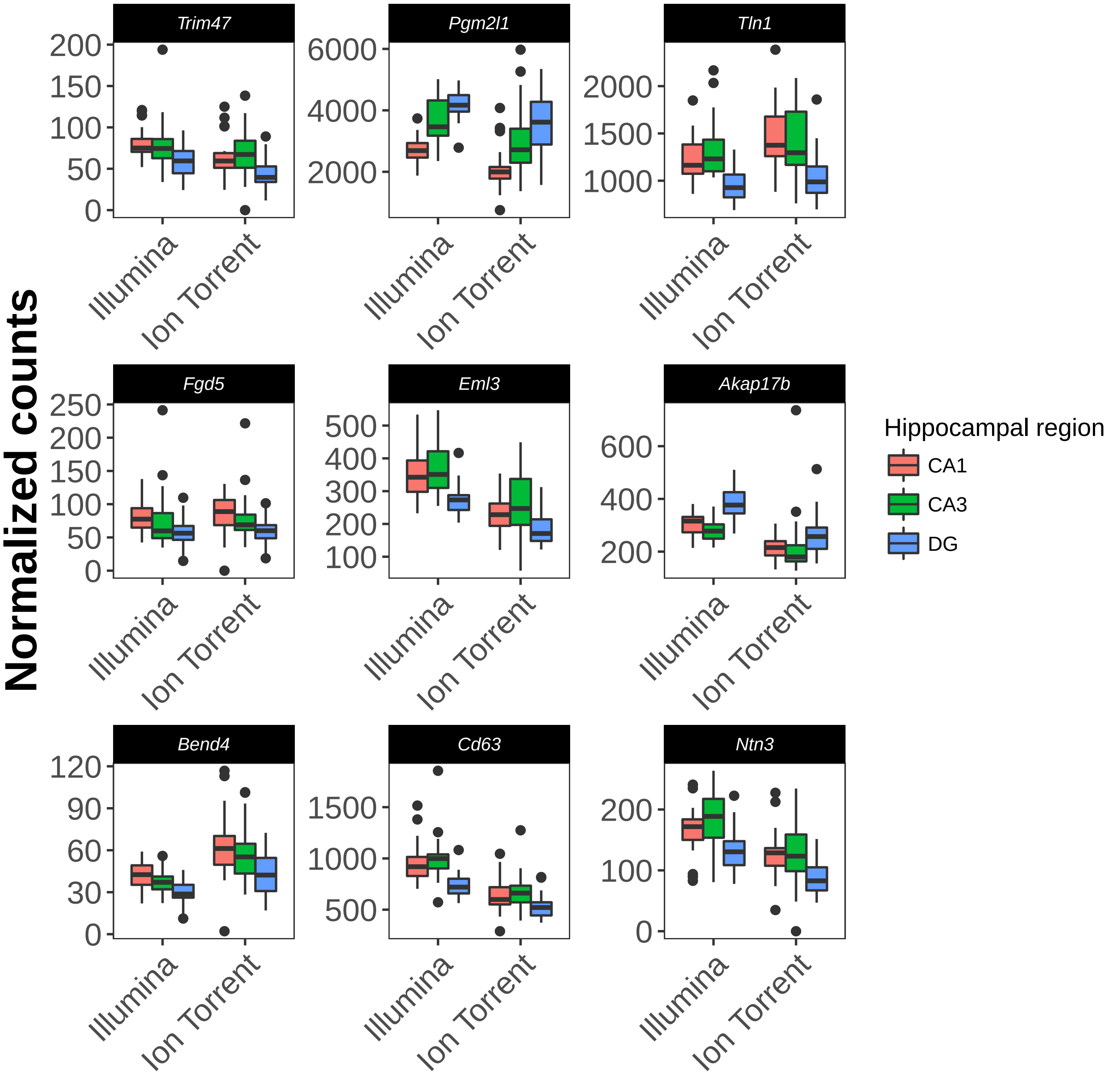
Normalized counts
Trim47
Pgm2l1
Tln1
Fgd5
Eml3
Akap17b
Bend4
Cd63
Ntn3
Illumina
Ion Torrent
Hippocampal region
CA1
CA3
DG

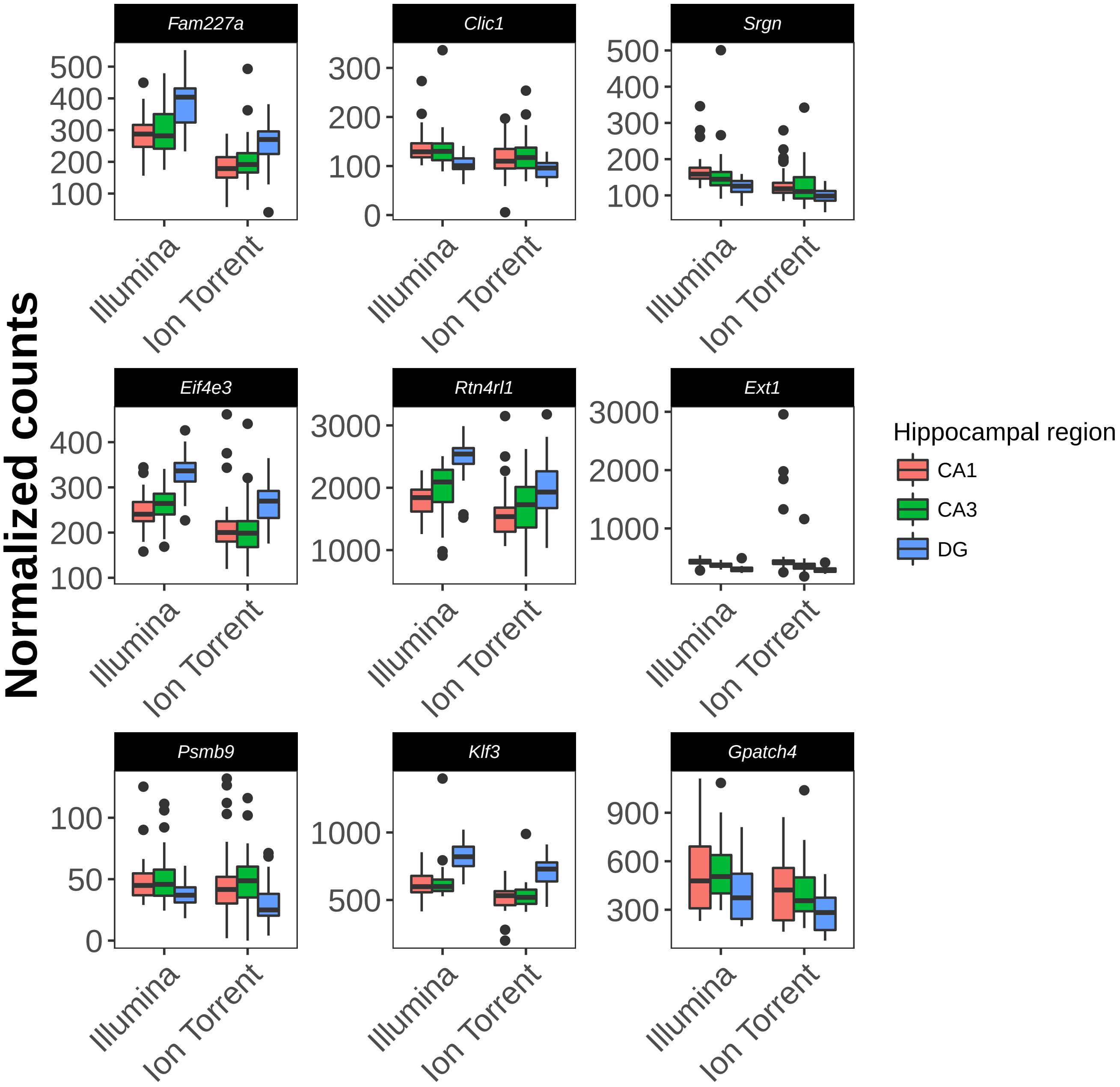
Normalized counts
Fam227a
Clic1
Srgn
Eif4e3
Rtn4rl1
Ext1
Psmb9
Klf3
Gpatch4
Illumina
Ion Torrent
Hippocampal region
CA1
CA3
DG

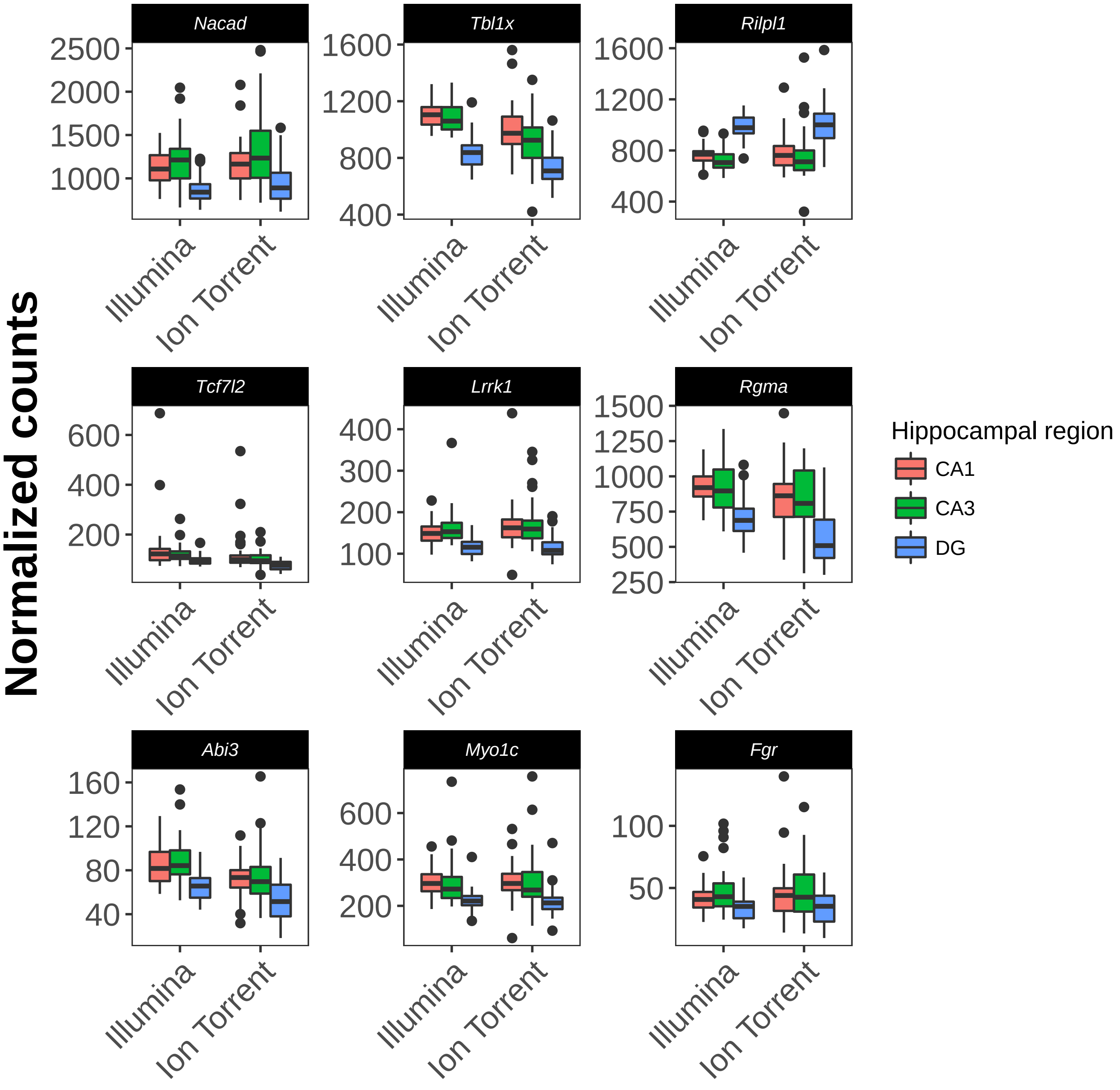
Normalized counts
Nacad
Tbl1x
Rilpl1
Tcf7l2
Lrrk1
Rgma
Abi3
Myo1c
Fgr
Illumina
Ion Torrent
Hippocampal region
CA1
CA3
DG

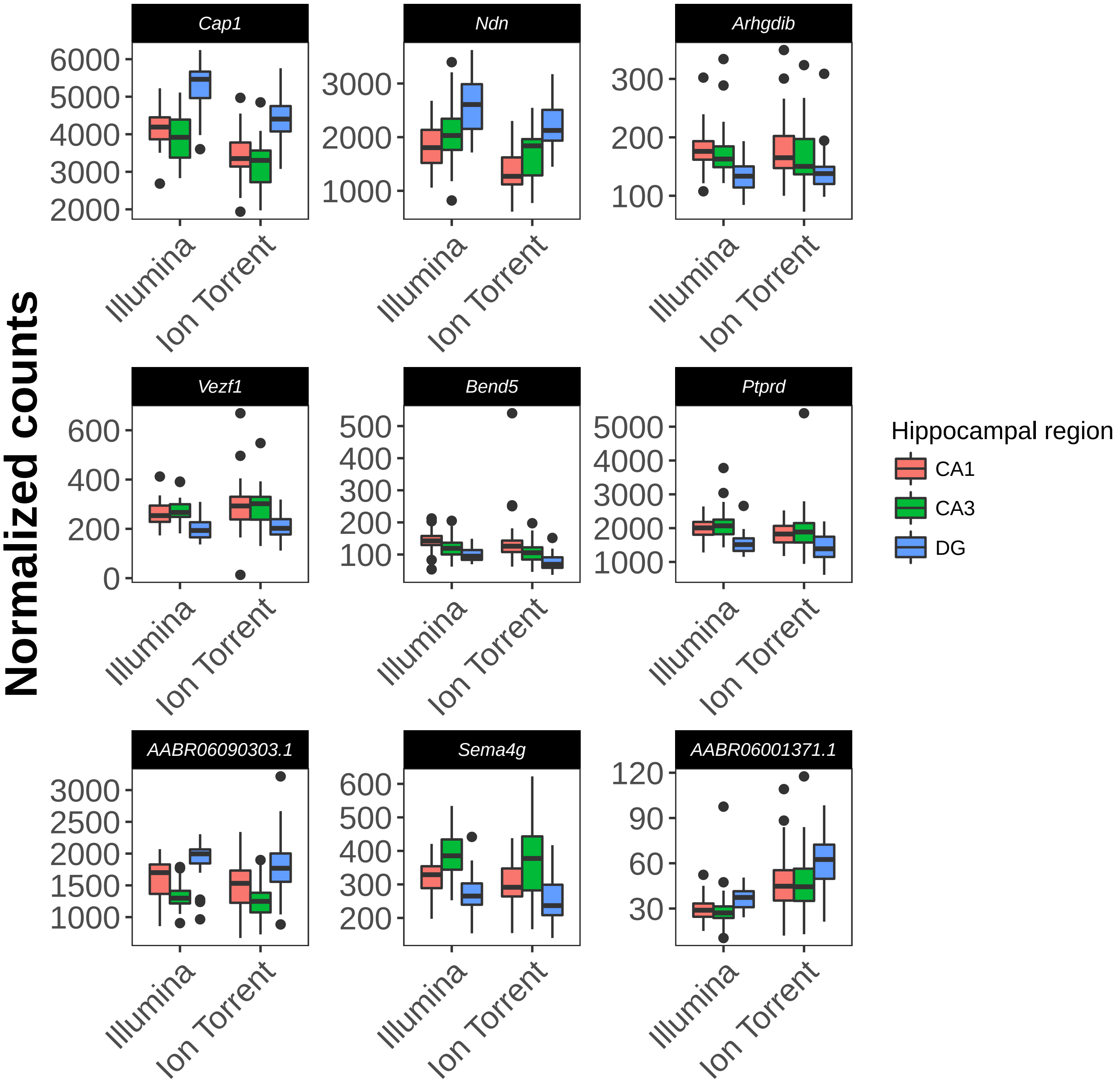

Normalized counts
Cap1
Ndn
Arhgdib
Vezf1
Bend5
Ptprd
AABR06090303.1
Sema4g
AABR06001371.1
Illumina
Ion Torrent
Hippocampal region
CA1
CA3
DG

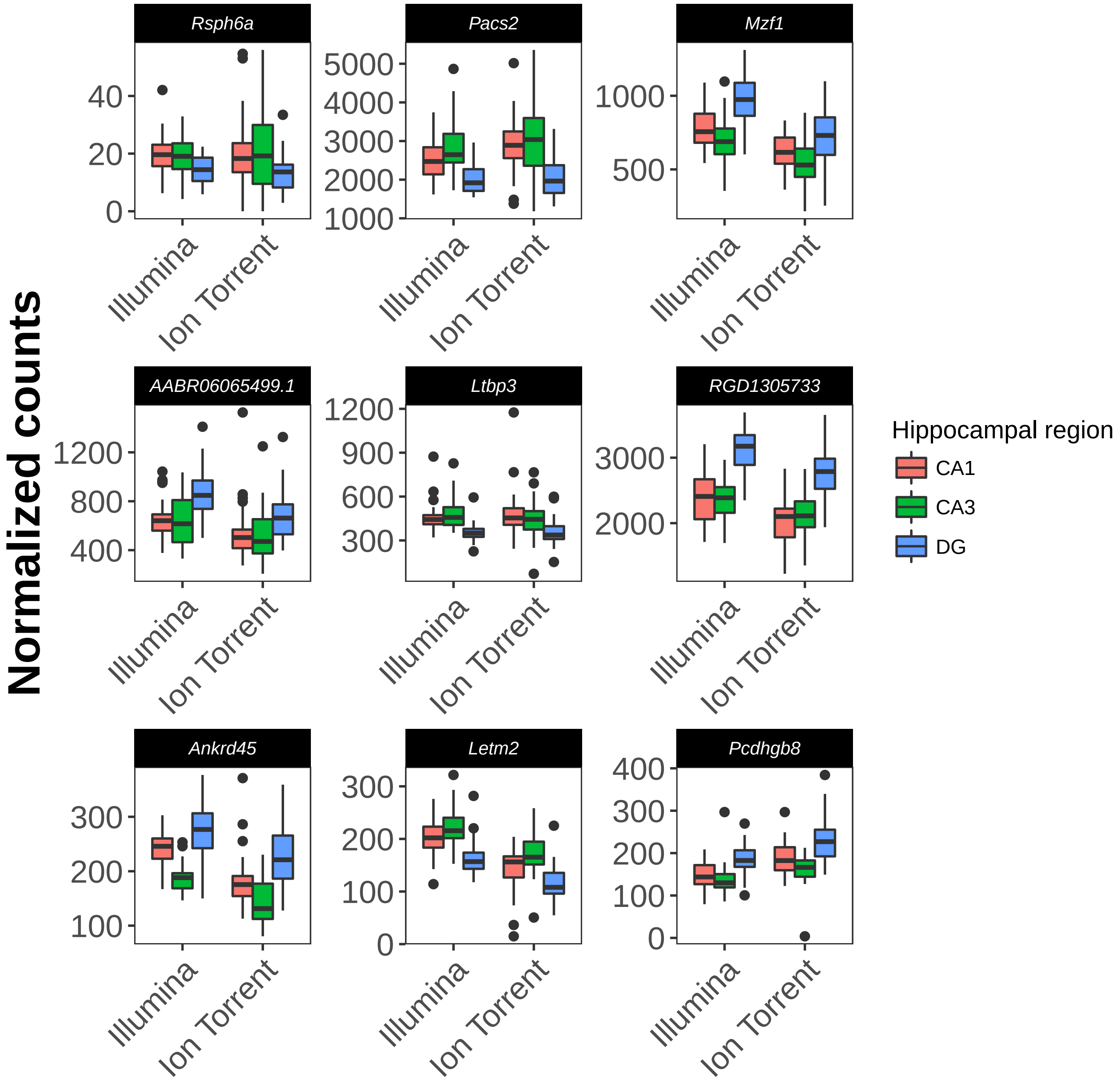
Rsph6a
Pacs2
Mzf1
AABR06065499.1
Ltbp3
RGD1305733
Ankrd45
Letm2
Pcdhgb8
Normalized counts
Illumina
Ion Torrent
Hippocampal region
CA1
CA3
DG

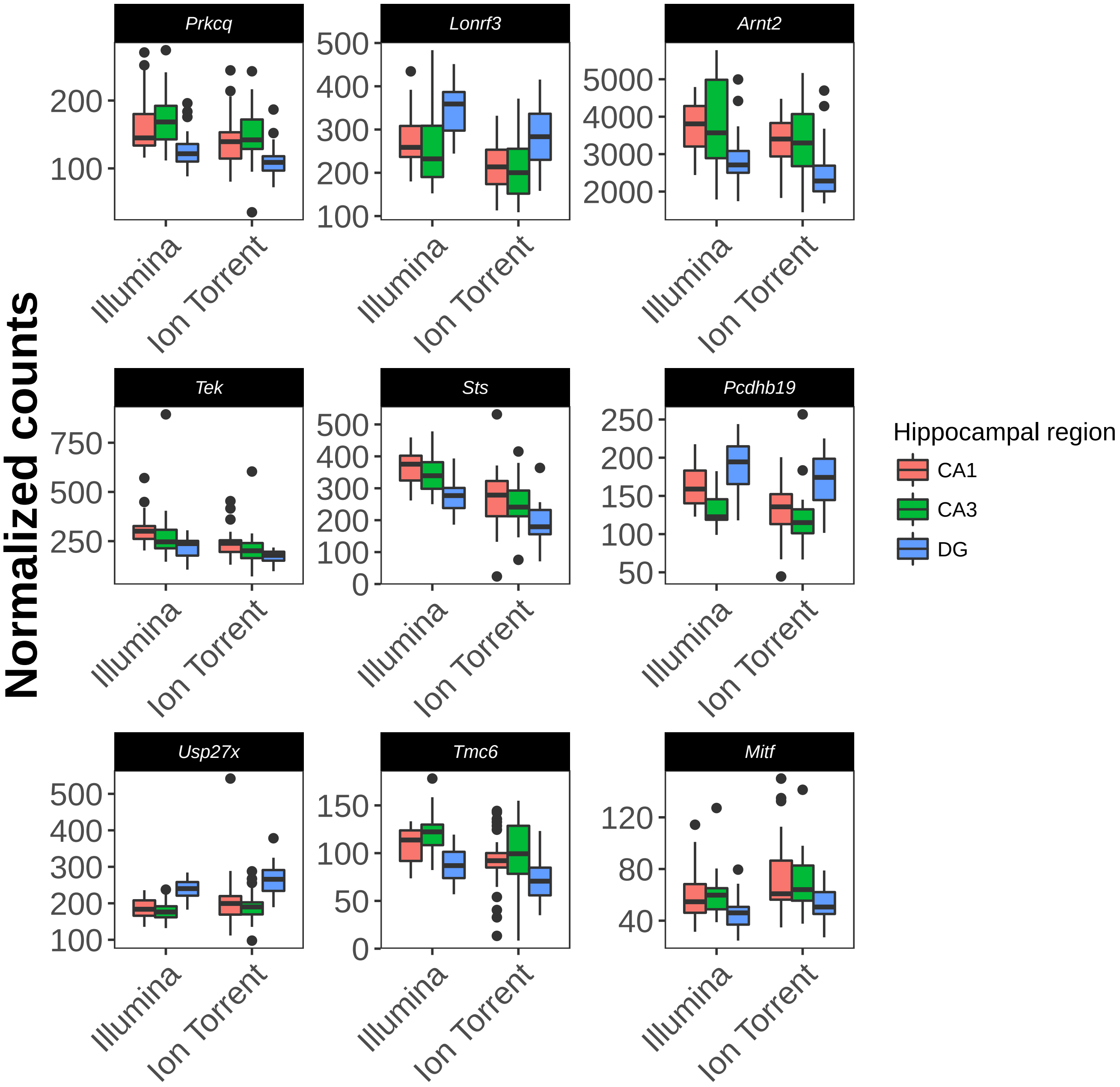
Normalized counts
Prkcq
Lonrf3
Arnt2
Tek
Sts
Pcdhb19
Usp27x
Tmc6
Mitf
Illumina
Ion Torrent
Hippocampal region
CA1
CA3
DG

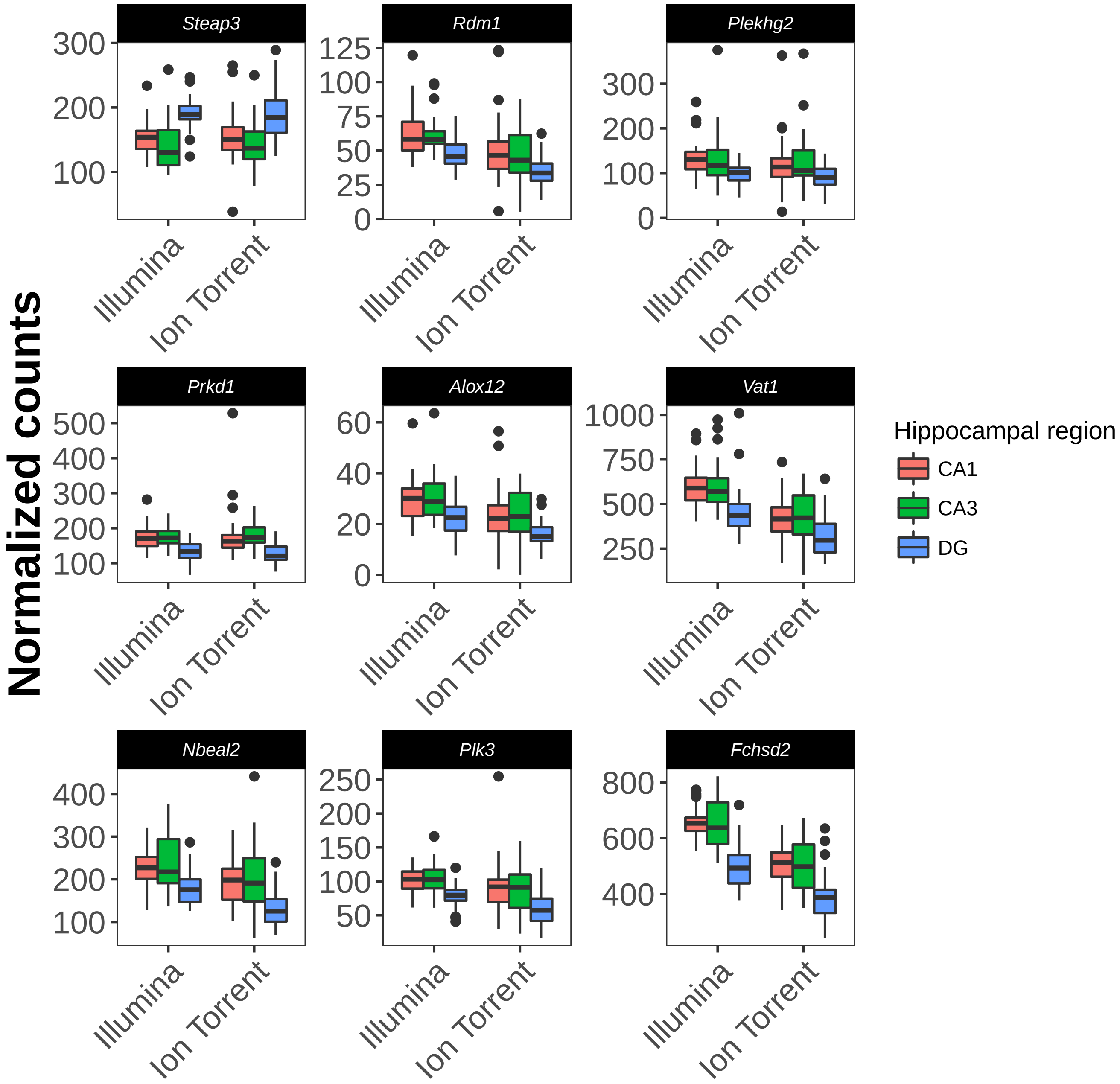
Normalized counts
Steap3
Rdm1
Plekhg2
Prkd1
Alox12
Vat1
Nbeal2
Plk3
Fchsd2
Illumina
Ion Torrent
Hippocampal region
CA1
CA3
DG

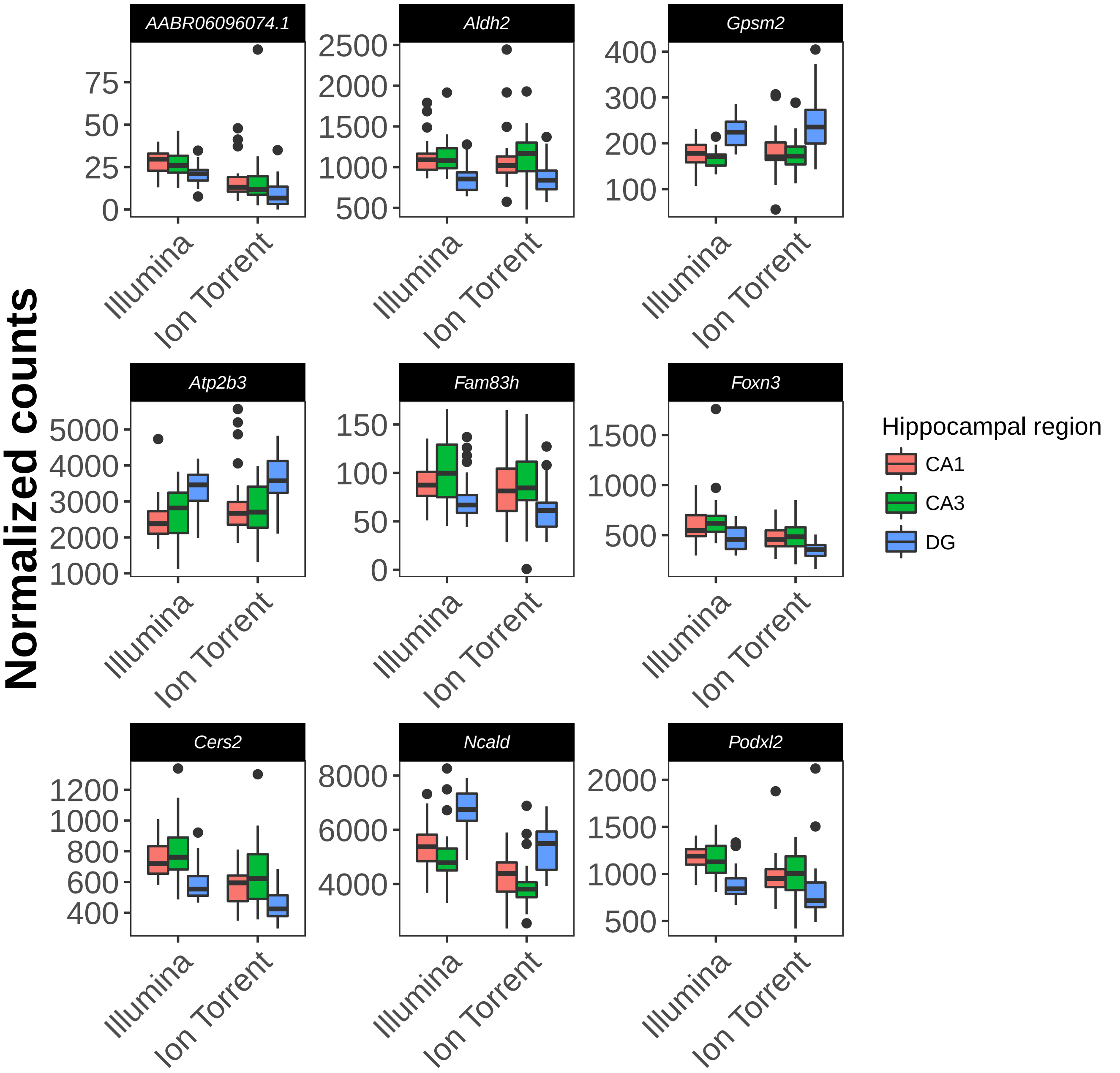
Normalized counts
AABR06096074.1
Aldh2
Gpsm2
Atp2b3
Fam83h
Foxn3
Cers2
Ncald
Podxl2
Illumina
Ion Torrent
Hippocampal region
CA1
CA3
DG

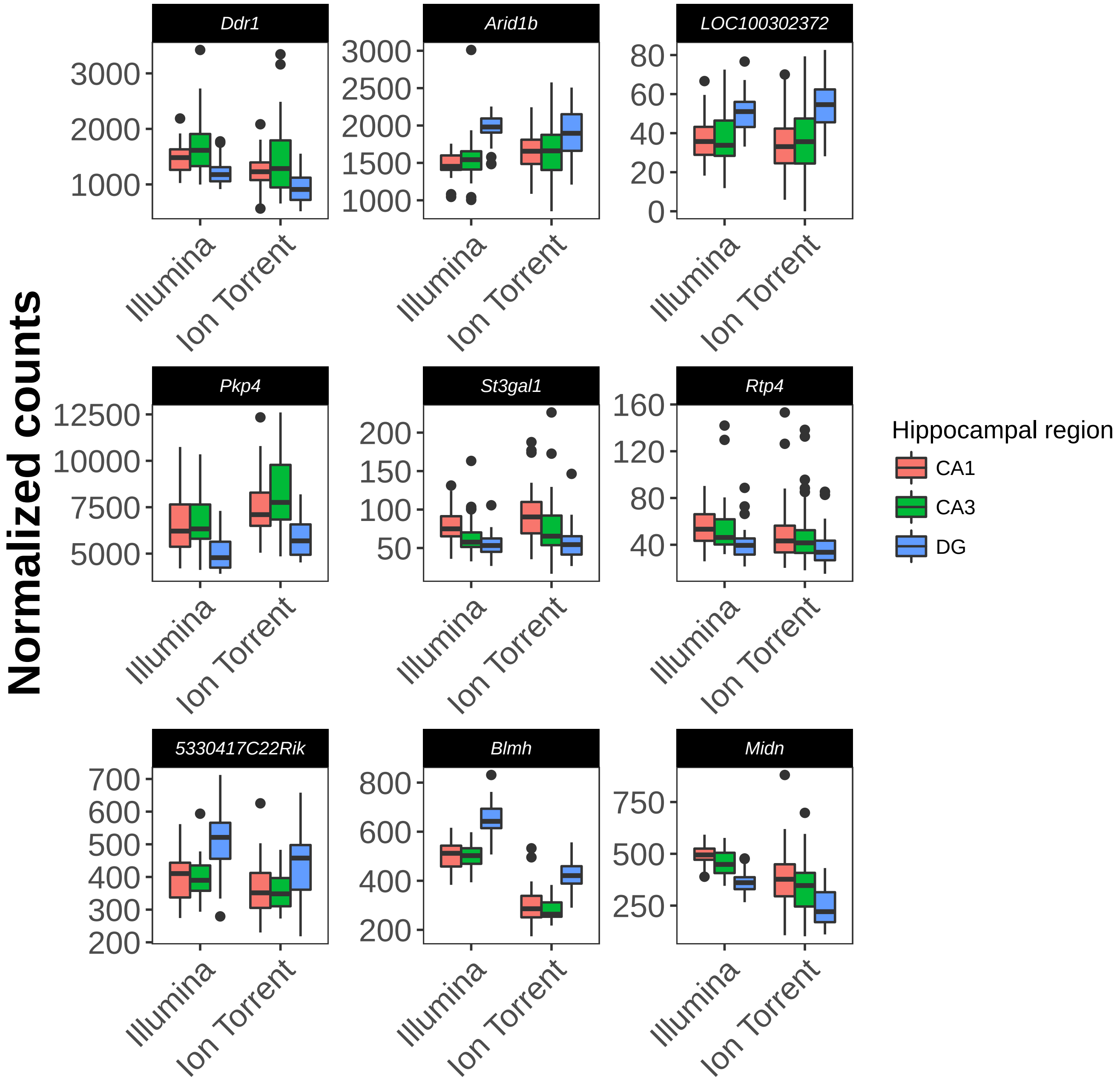

Normalized counts
Ddr1
Arid1b
LOC100302372
Pkp4
St3gal1
Rtp4
5330417C22Rik
Blmh
Midn
Illumina
Ion Torrent
Hippocampal region
CA1
CA3
DG

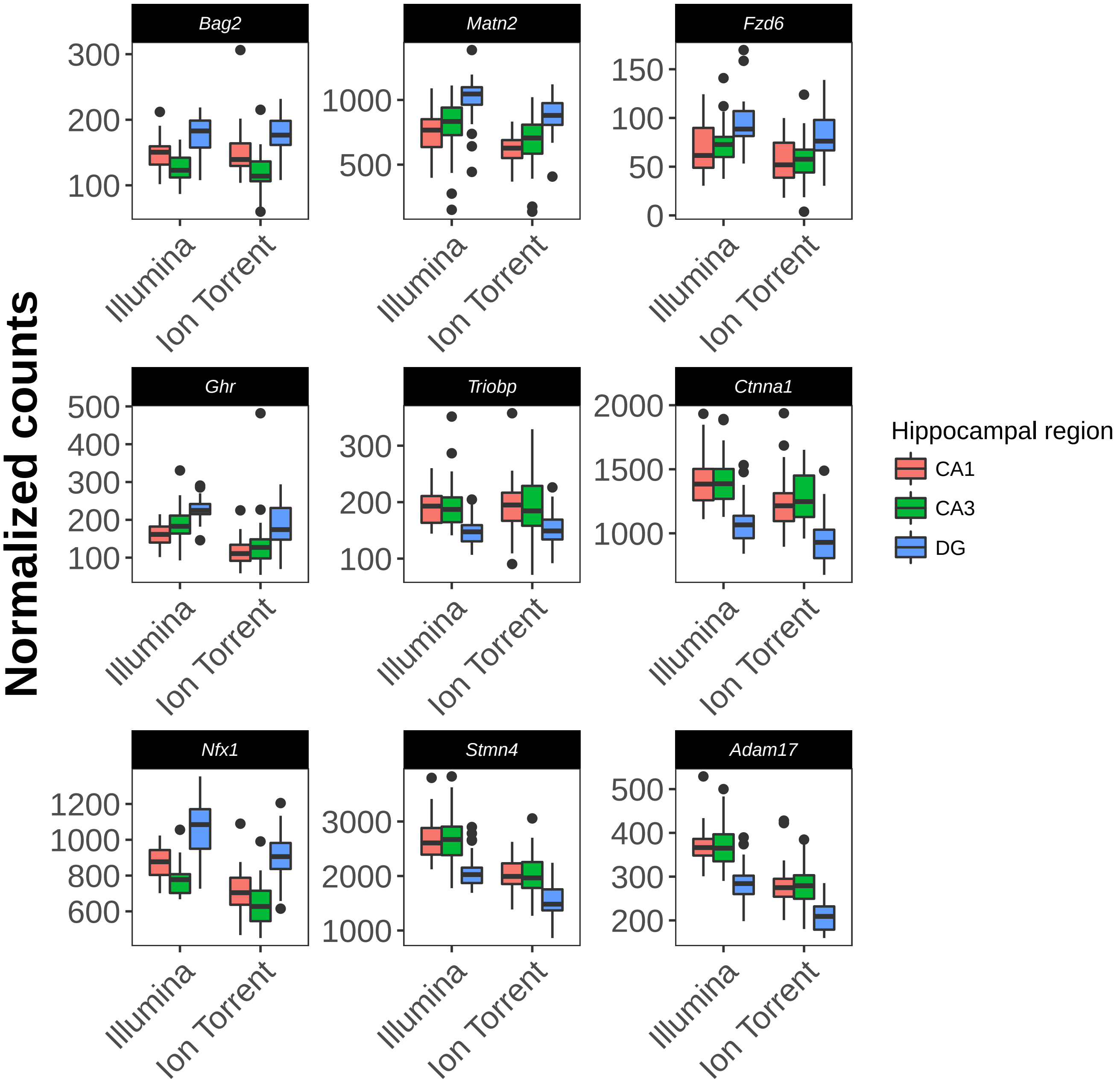
Normalized counts
Bag2
Matn2
Fzd6
Ghr
Triobp
Ctnna1
Nfx1
Stmn4
Adam17
Illumina
Ion Torrent
Hippocampal region
CA1
CA3
DG

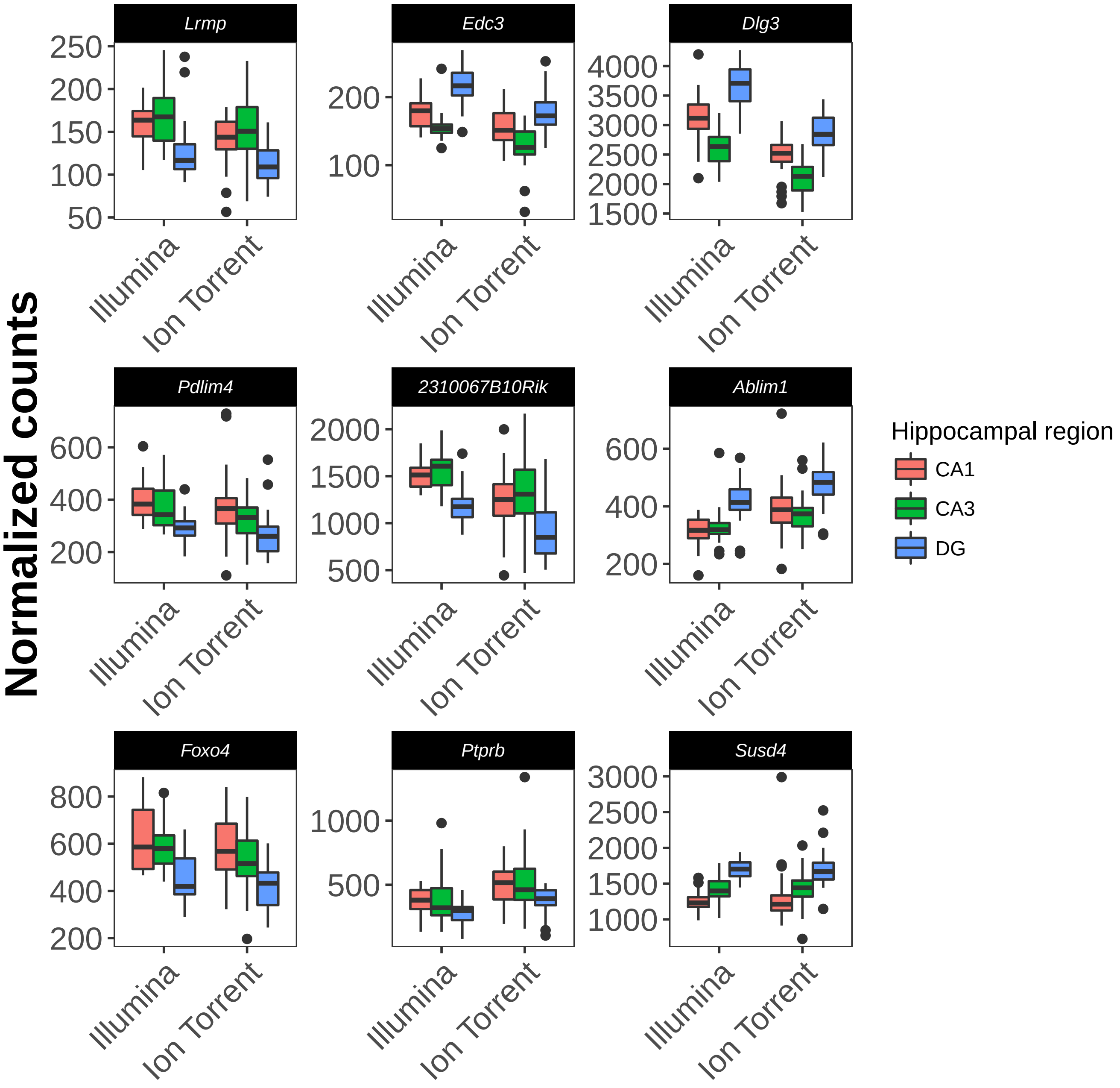
Lrmp
Edc3
Dlg3
Pdlim4
2310067B10Rik
Ablim1
Foxo4
Ptprb
Susd4
Illumina
Ion Torrent
Normalized counts
Hippocampal region
CA1
CA3
DG

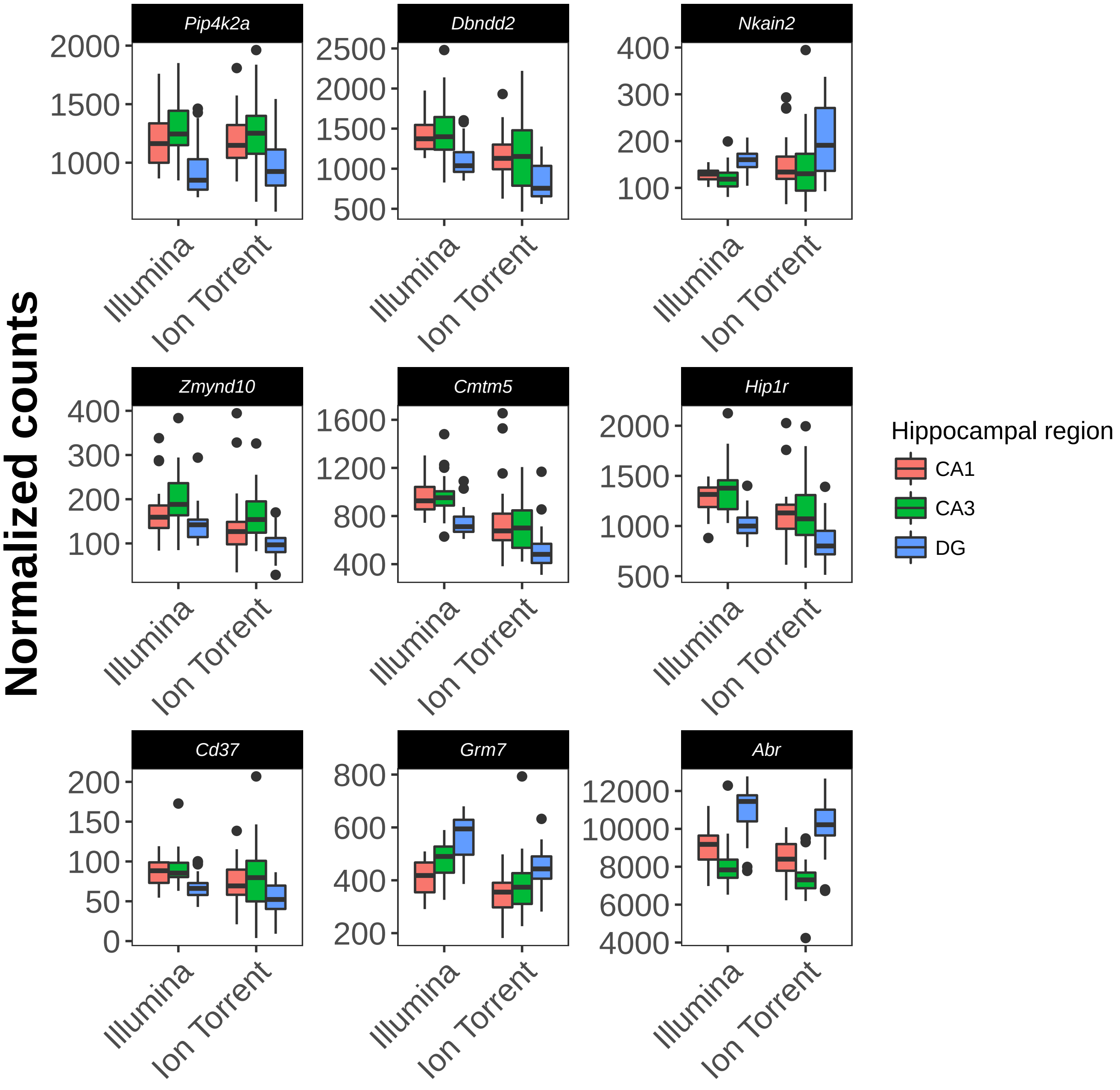
Pip4k2a
Dbndd2
Nkain2
Zmynd10
Cmtm5
Hip1r
Cd37
Grm7
Abr
Normalized counts
Illumina
Ion Torrent
Hippocampal region
CA1
CA3
DG

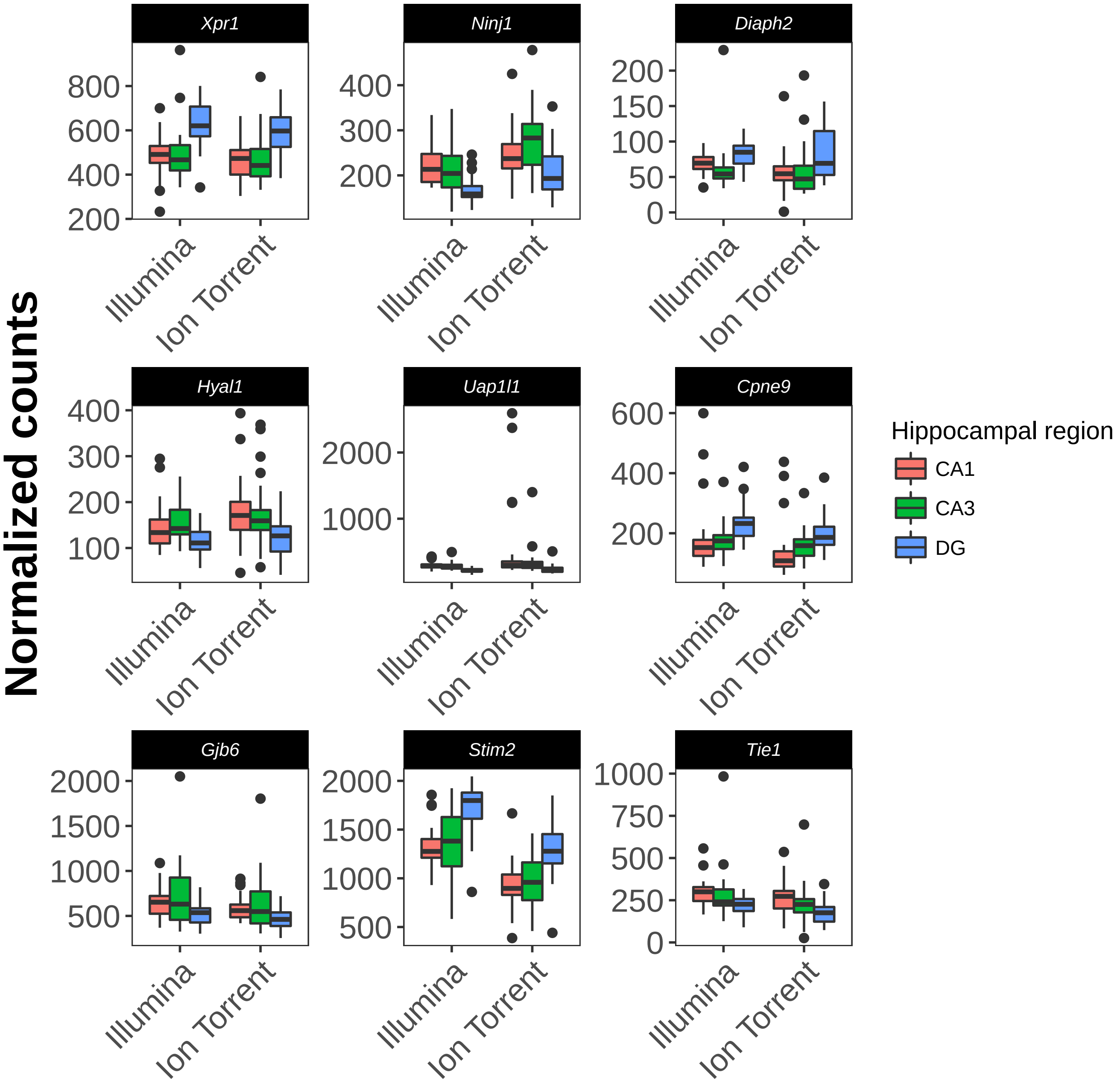

Normalized counts
Xpr1
Ninj1
Diaph2
Hyal1
Uap1l1
Cpne9
Gjb6
Stim2
Tie1
Illumina
Ion Torrent
Hippocampal region
CA1
CA3
DG

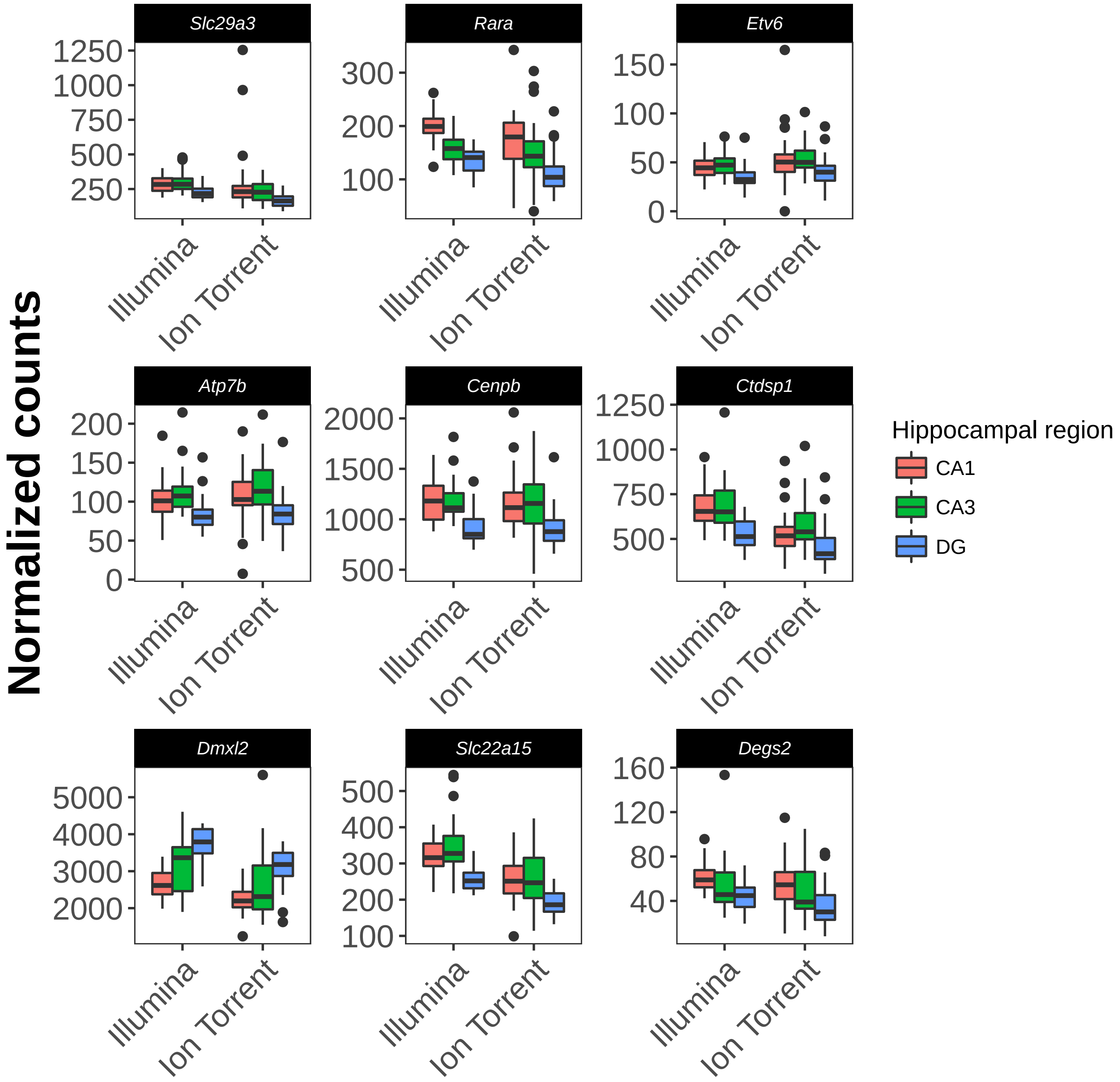
Slc29a3
Rara
Etv6
Atp7b
Cenpb
Ctdsp1
Dmxl2
Slc22a15
Degs2
Normalized counts
Illumina
Ion Torrent
Hippocampal region
CA1
CA3
DG

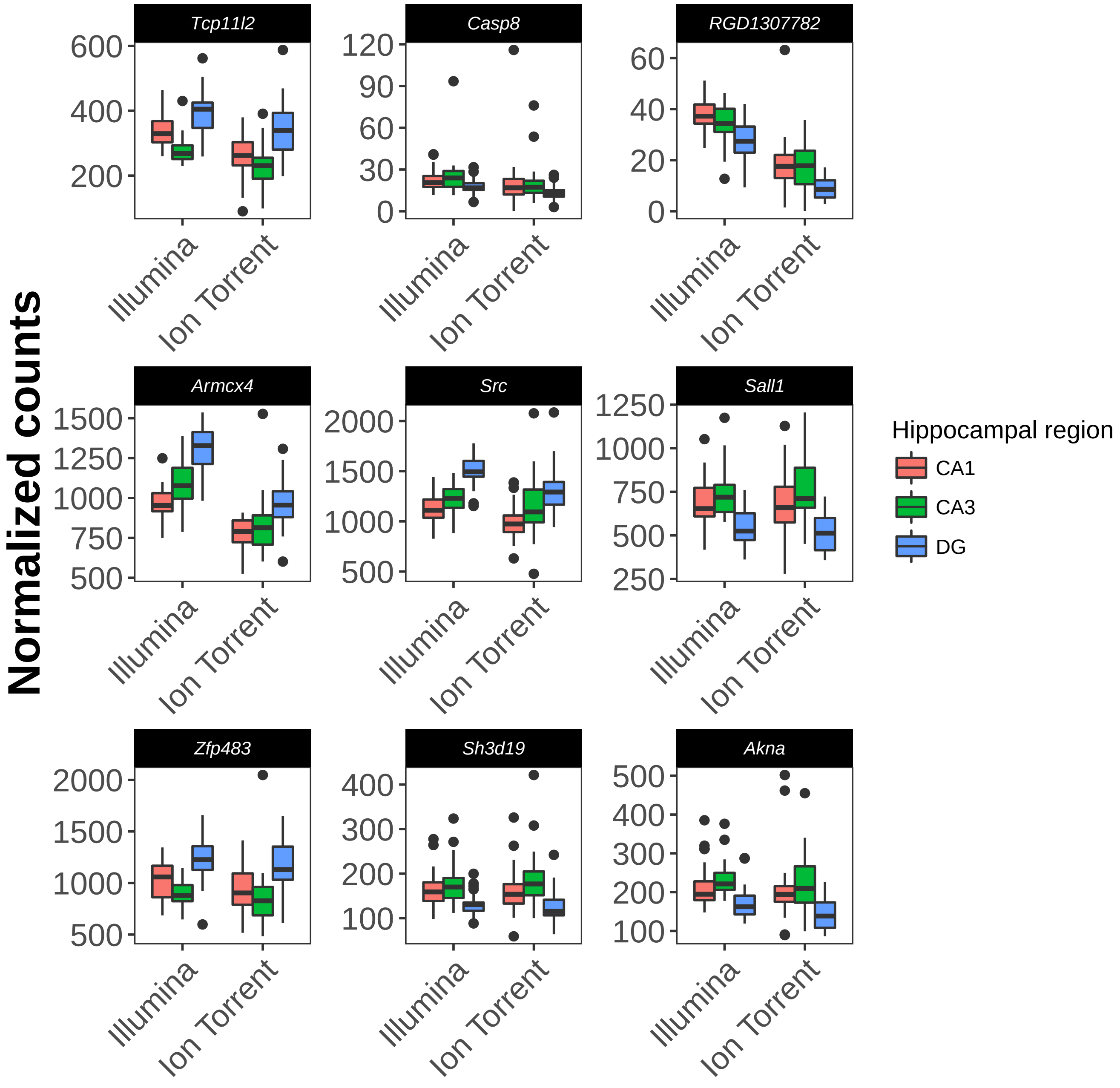

Normalized counts
Tcp11l2
Casp8
RGD1307782
Armcx4
Src
Sall1
Zfp483
Sh3d19
Akna
Illumina
Ion Torrent
Hippocampal region
CA1
CA3
DG

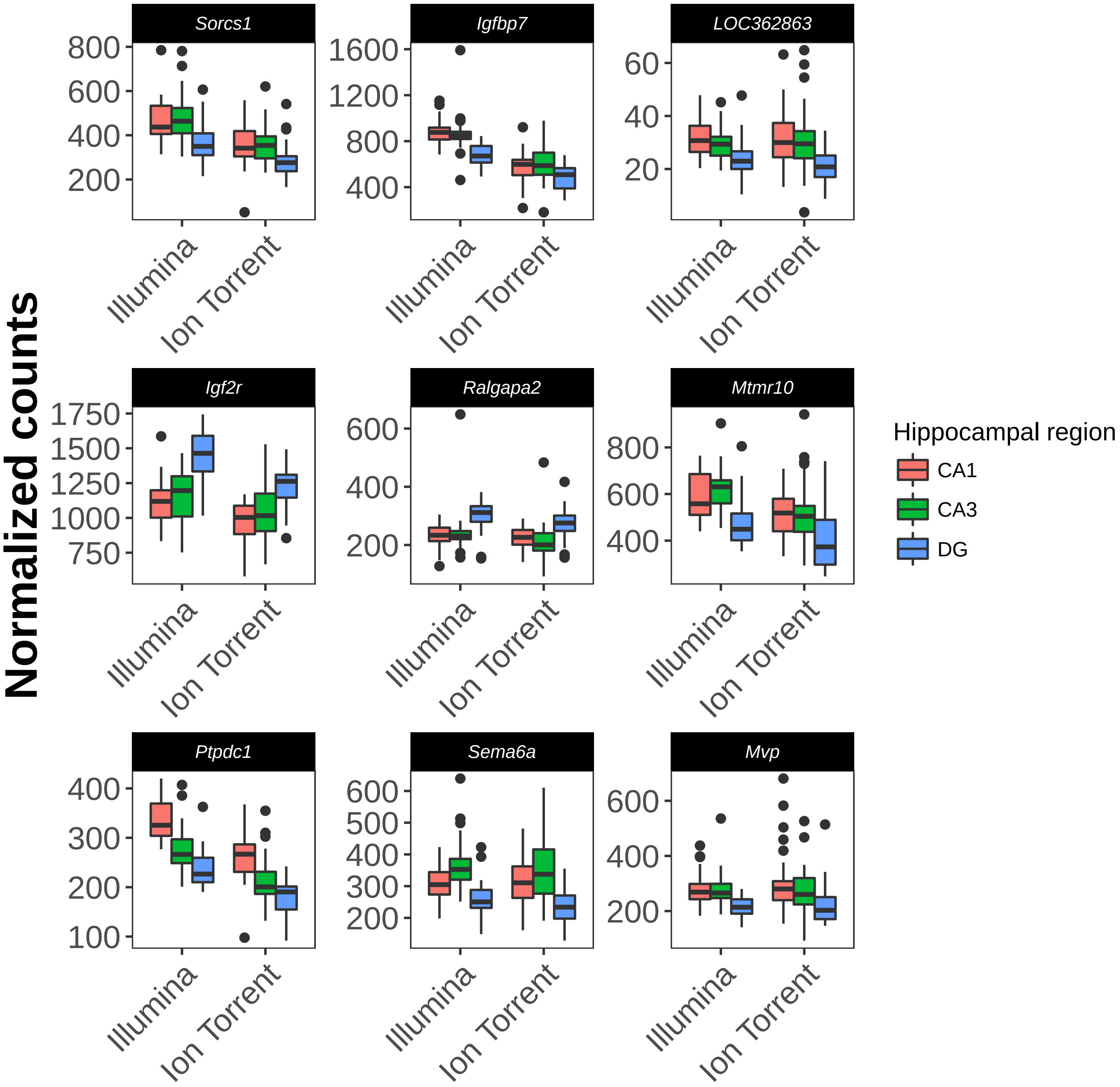

Normalized counts
Sorcs1
Igfbp7
LOC362863
Igf2r
Ralgapa2
Mtmr10
Ptpdc1
Sema6a
Mvp
Illumina
Ion Torrent
Hippocampal region
CA1
CA3
DG

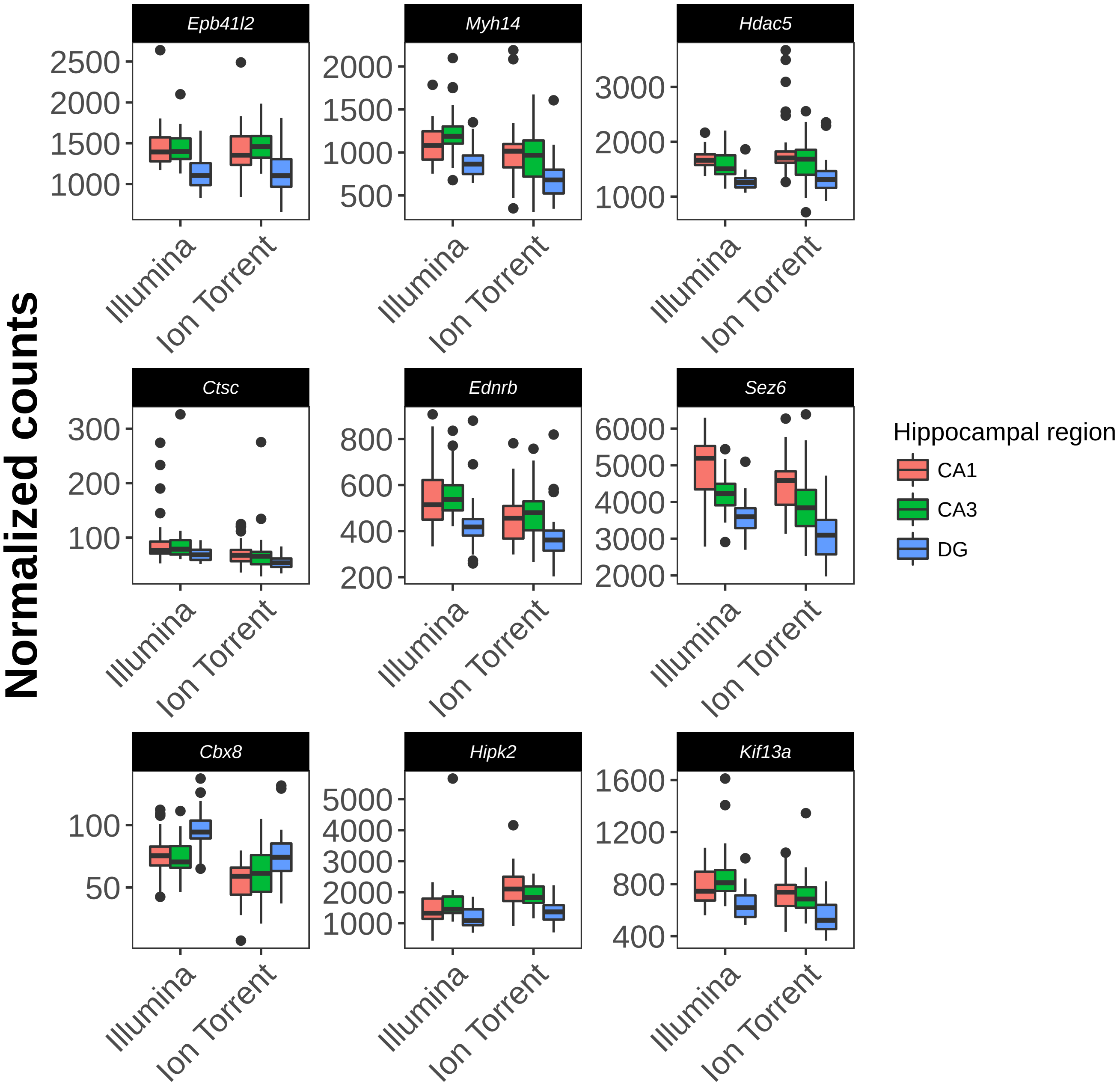
Normalized counts
Epb41l2
Myh14
Hdac5
Ctsc
Ednrb
Sez6
Cbx8
Hipk2
Kif13a
Illumina
Ion Torrent
Hippocampal region
CA1
CA3
DG

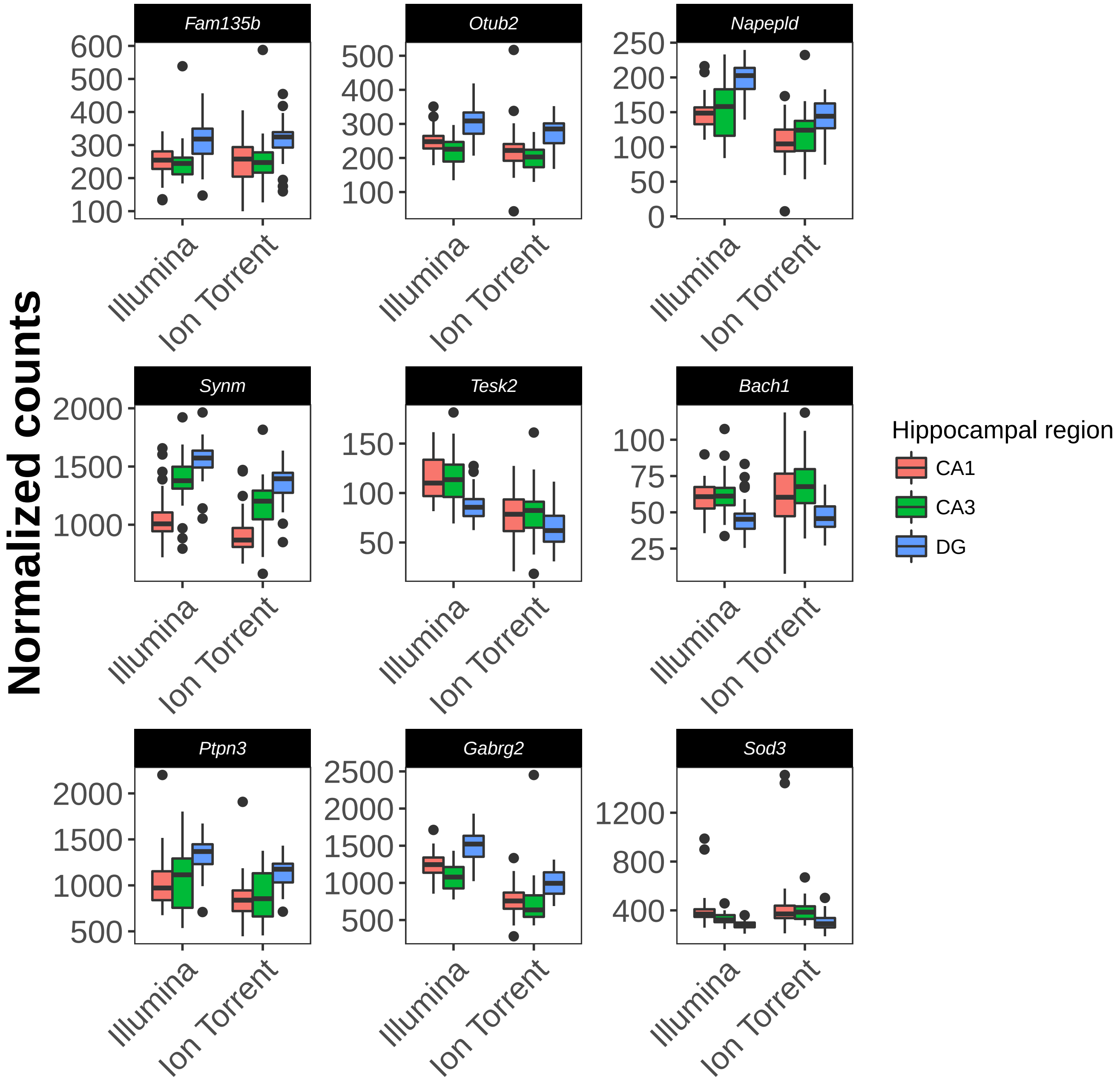

Normalized counts
Fam135b
Otub2
Napepld
Synm
Tesk2
Bach1
Ptpn3
Gabrg2
Sod3
Illumina
Ion Torrent
Hippocampal region
CA1
CA3
DG

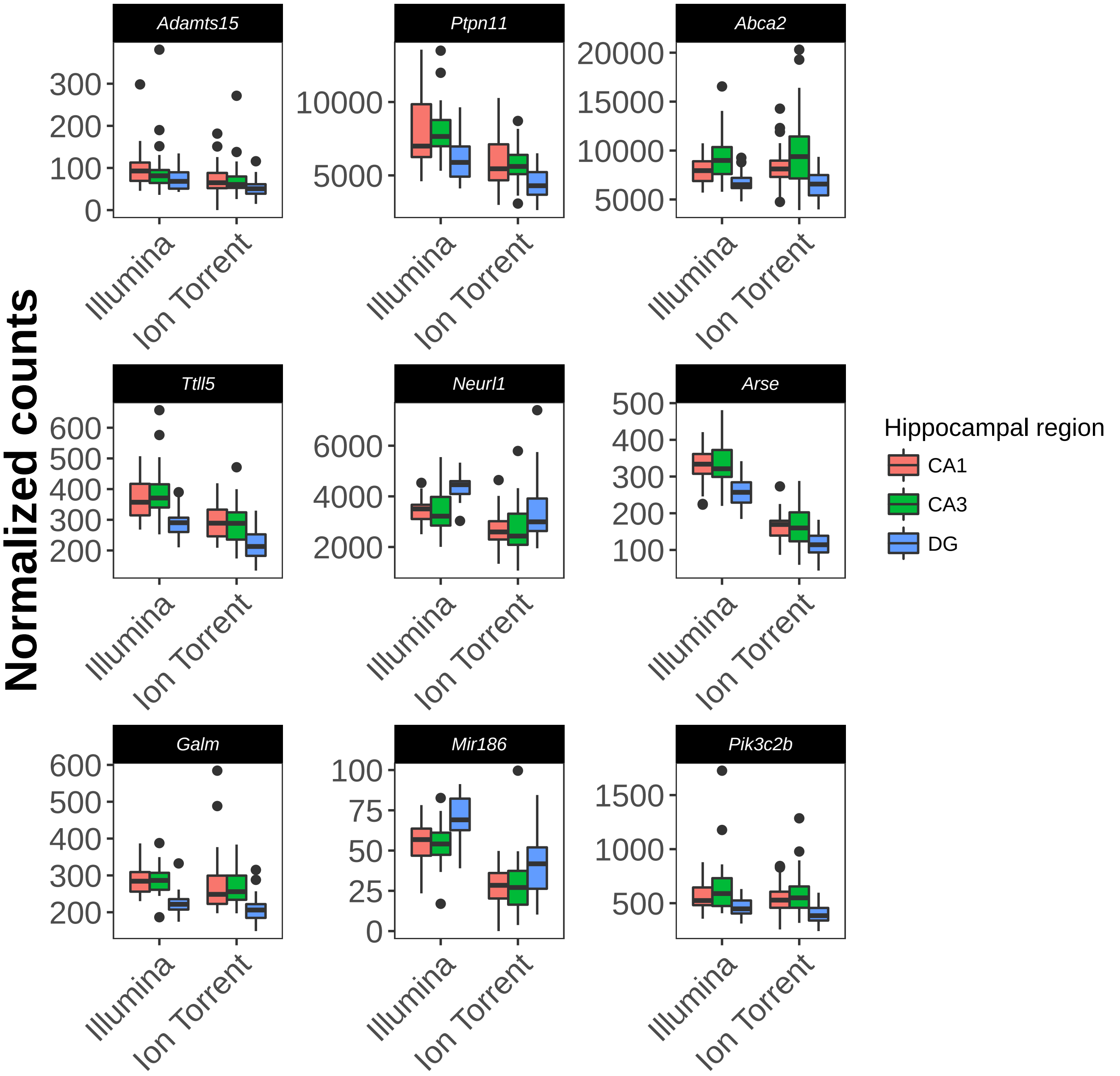

Normalized counts
Adamts15
Ptpn11
Abca2
Ttll5
Neurl1
Arse
Galm
Mir186
Pik3c2b
Illumina
Ion Torrent
Hippocampal region
CA1
CA3
DG

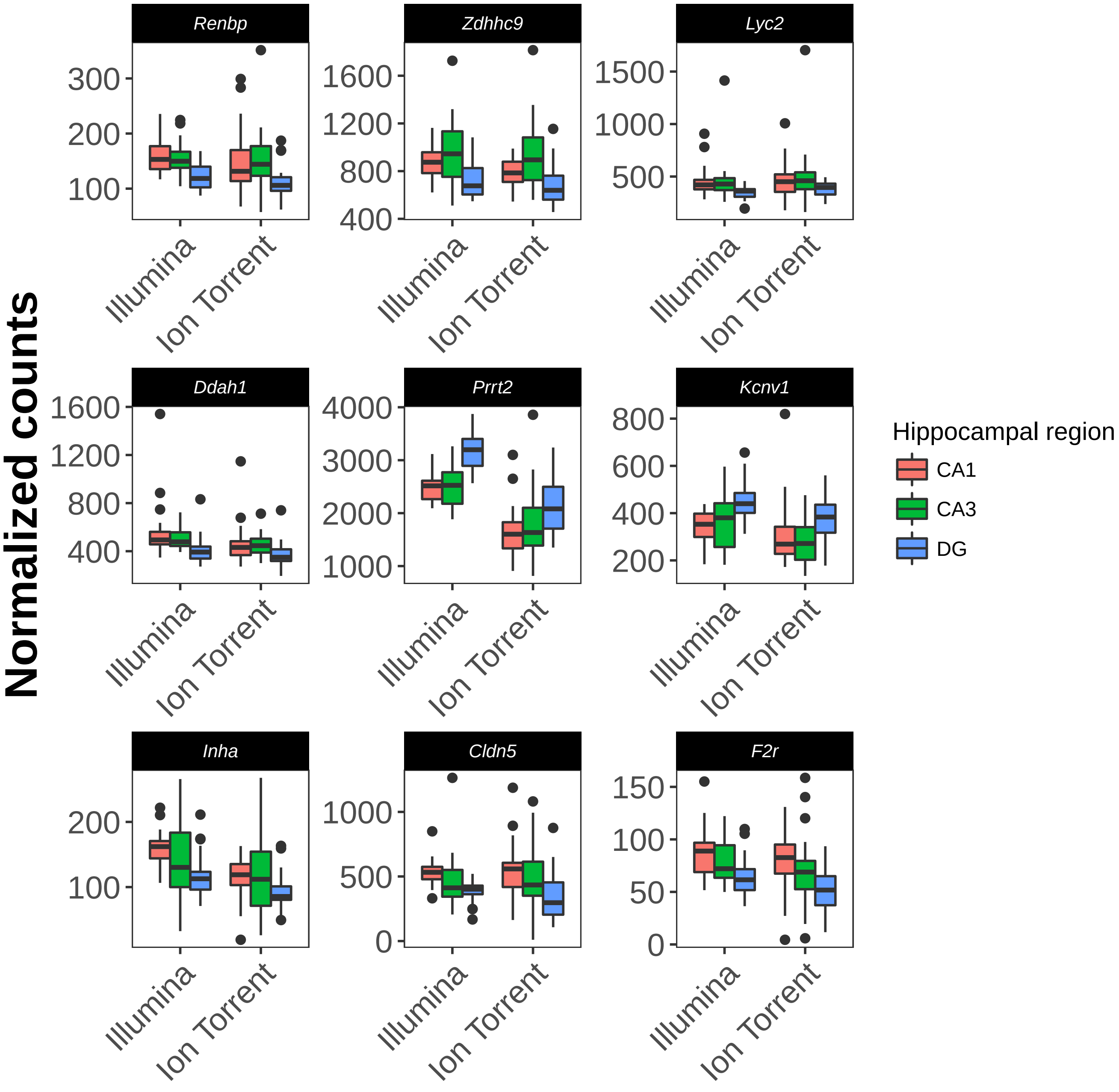
Normalized counts
Renbp
Zdhhc9
Lyc2
Ddah1
Prrt2
Kcnv1
Inha
Cldn5
F2r
Illumina
Ion Torrent
Hippocampal region
CA1
CA3
DG

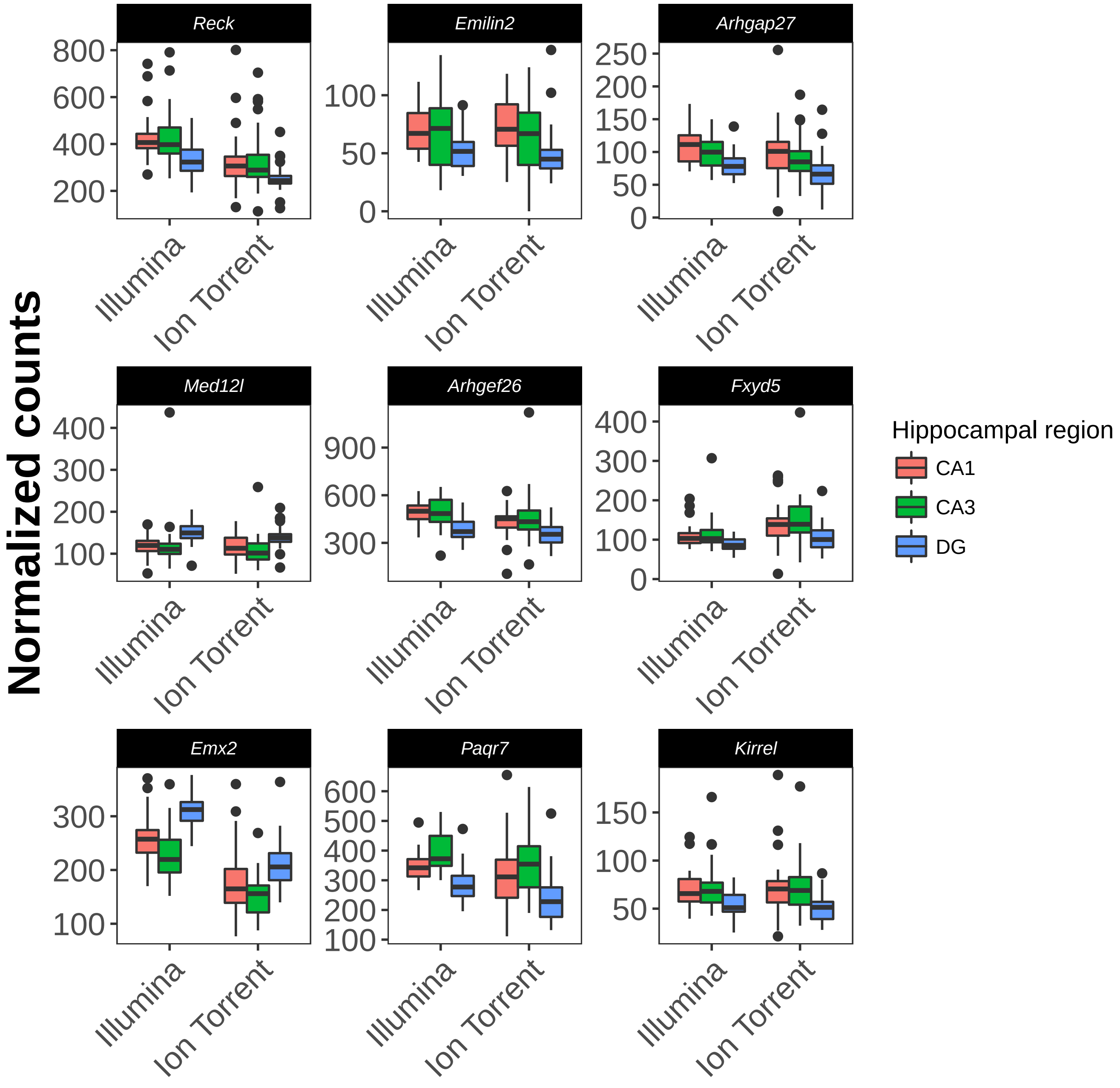

Normalized counts
Reck
Emilin2
Arhgap27
Med12l
Arhgef26
Fxyd5
Emx2
Paqr7
Kirrel
Illumina
Ion Torrent
Hippocampal region
CA1
CA3
DG

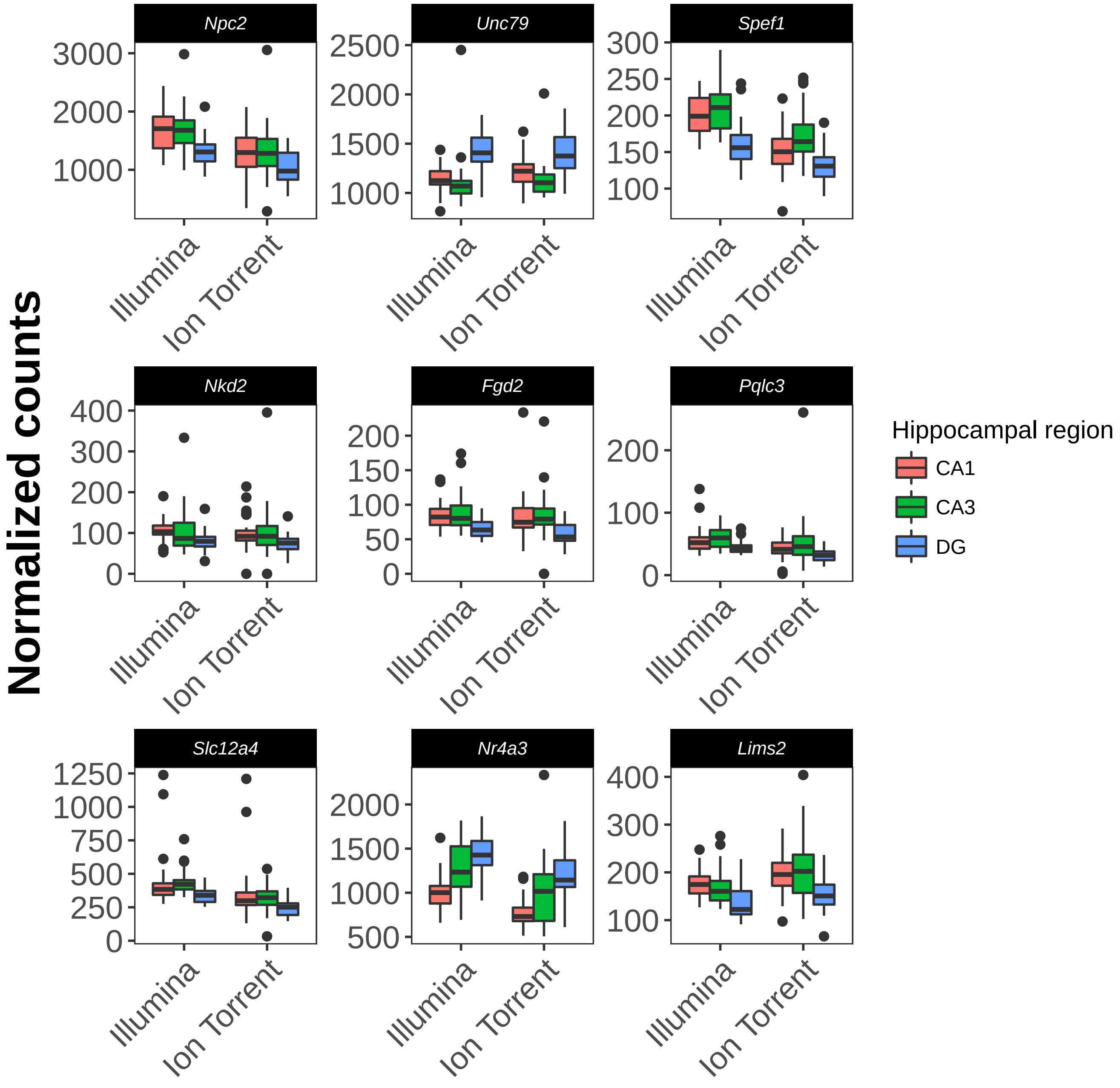
Normalized counts
Npc2
Unc79
Spef1
Nkd2
Fgd2
Pqlc3
Slc12a4
Nr4a3
Lims2
Illumina
Ion Torrent
Hippocampal region
CA1
CA3
DG

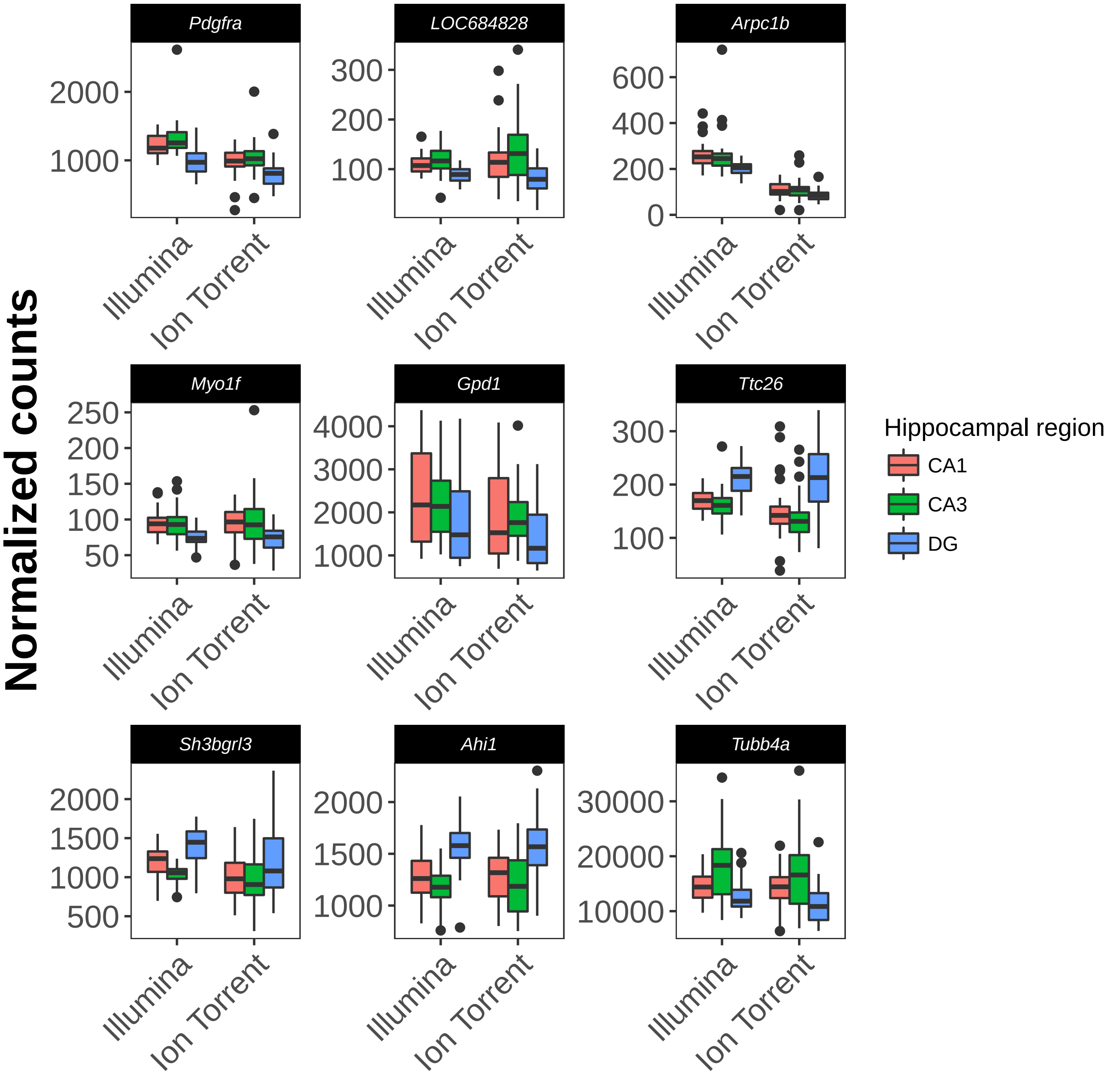
Normalized counts
Pdgfra
LOC684828
Arpc1b
Myo1f
Gpd1
Ttc26
Sh3bgrl3
Ahi1
Tubb4a
Illumina
Ion Torrent
Hippocampal region
CA1
CA3
DG

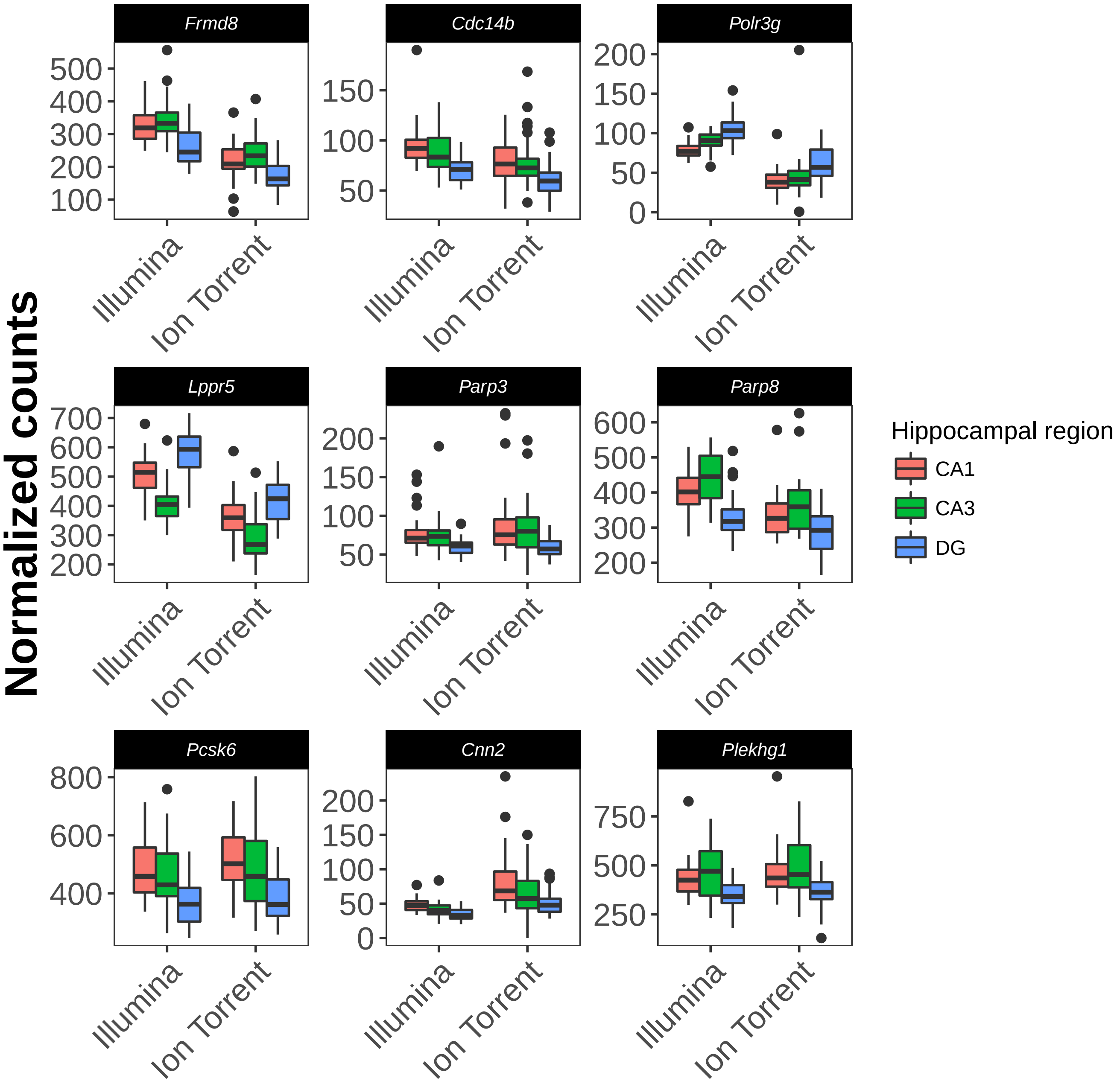
Normalized counts
Frmd8
Cdc14b
Polr3g
Lppr5
Parp3
Parp8
Pcsk6
Cnn2
Plekhg1
Illumina
Ion Torrent
Hippocampal region
CA1
CA3
DG

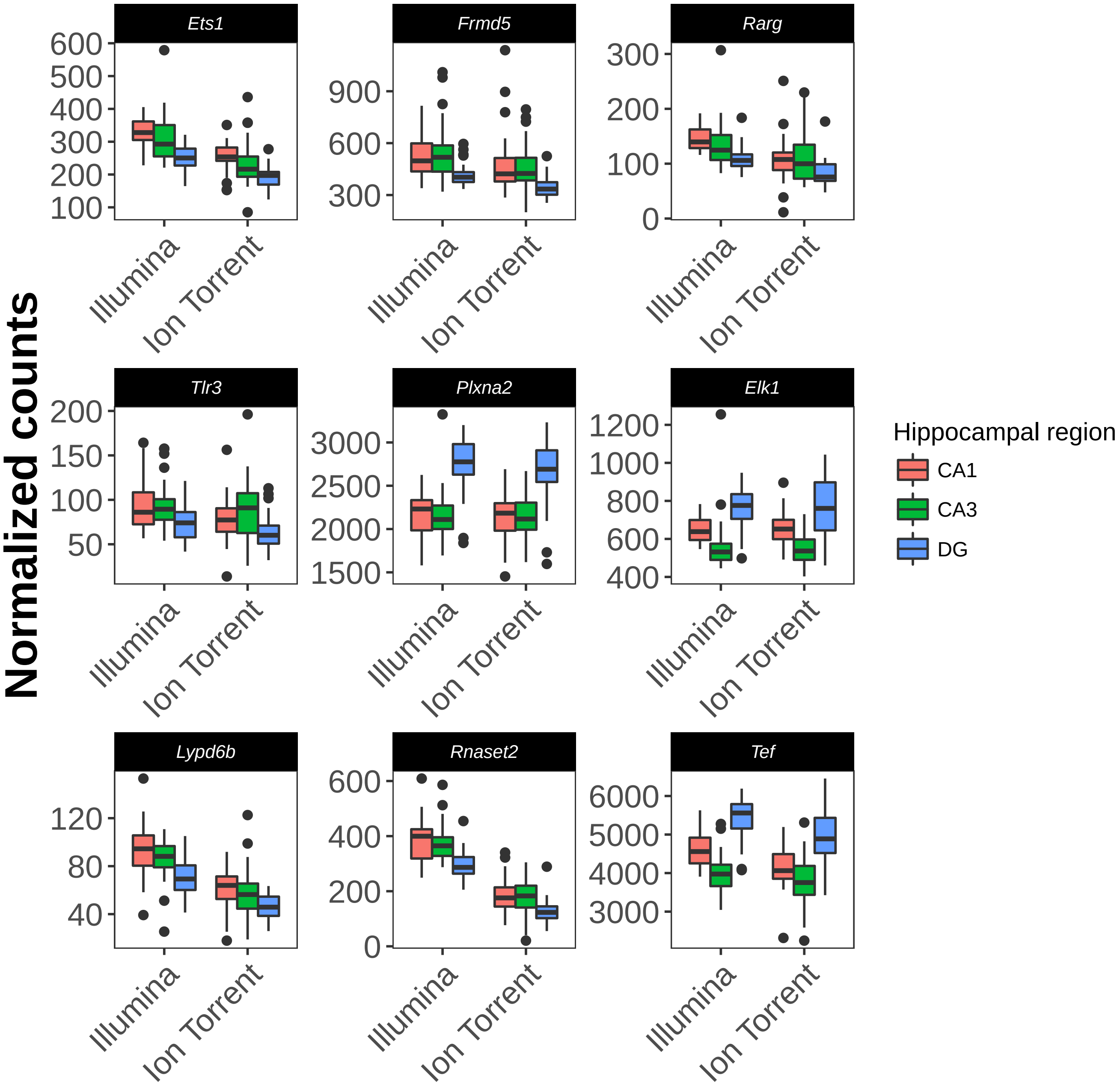
Normalized counts
Ets1
Frmd5
Rarg
Tlr3
Plxna2
Elk1
Lypd6b
Rnaset2
Tef
Illumina
Ion Torrent
Hippocampal region
CA1
CA3
DG

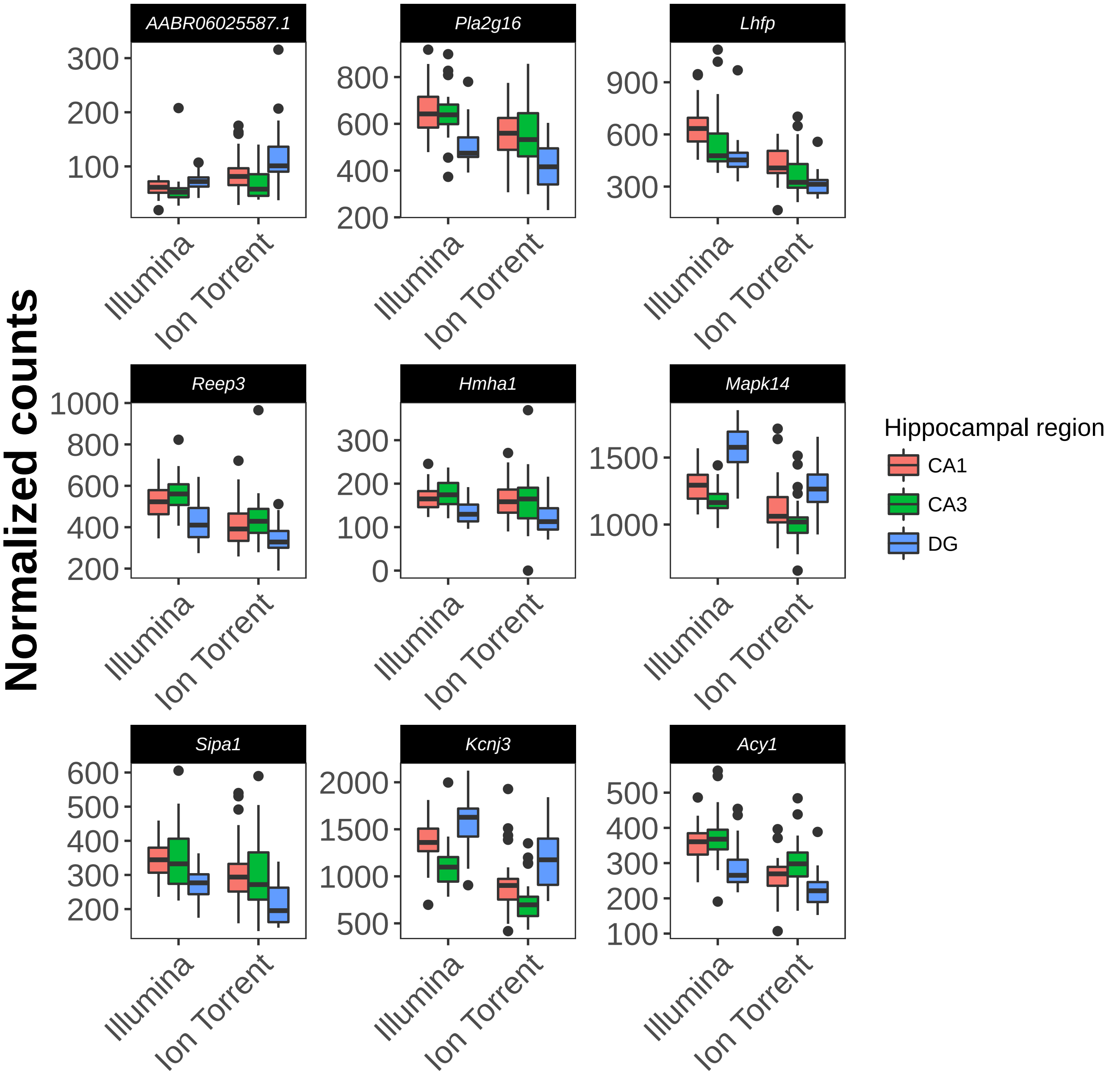
Normalized counts
AABR06025587.1
Pla2g16
Lhfp
Reep3
Hmha1
Mapk14
Sipa1
Kcnj3
Acy1
Illumina
Ion Torrent
Hippocampal region
CA1
CA3
DG

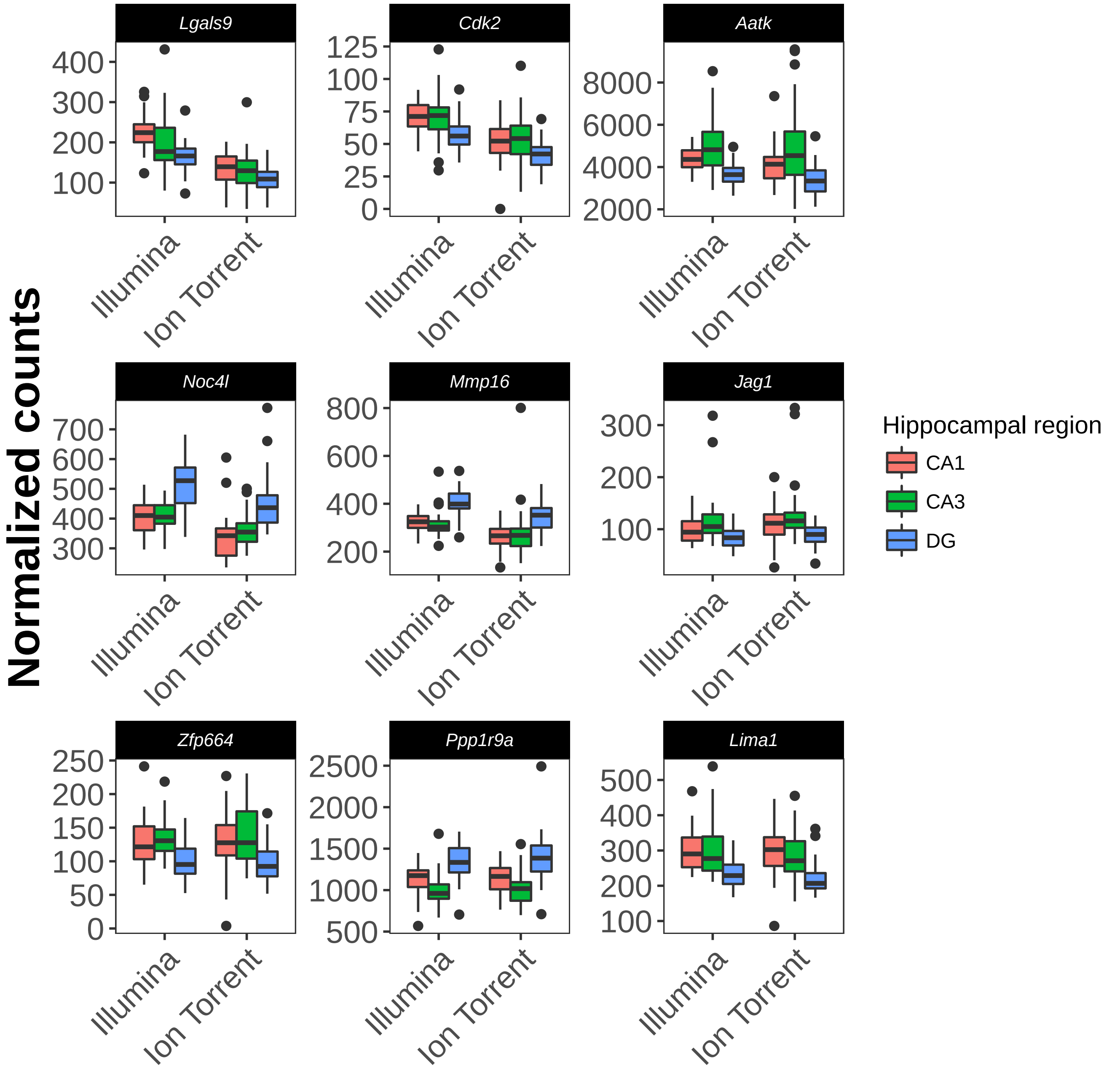
Normalized counts
Lgals9
Cdk2
Aatk
Noc4l
Mmp16
Jag1
Zfp664
Ppp1r9a
Lima1
Illumina
Ion Torrent
Hippocampal region
CA1
CA3
DG

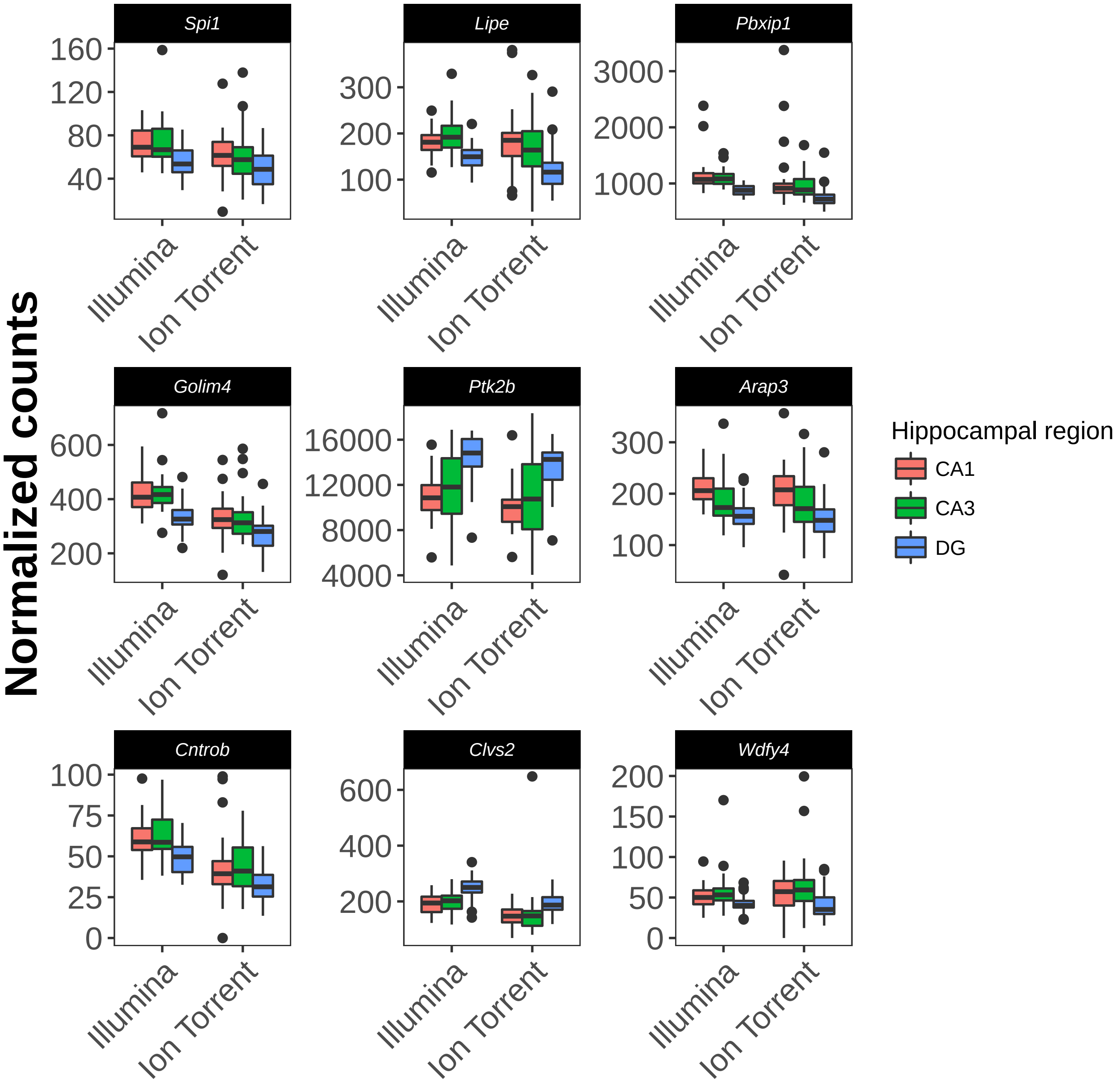
Normalized counts
Spi1
Lipe
Pbxip1
Golim4
Ptk2b
Arap3
Cntrob
Clvs2
Wdfy4
Illumina
Ion Torrent
Hippocampal region
CA1
CA3
DG

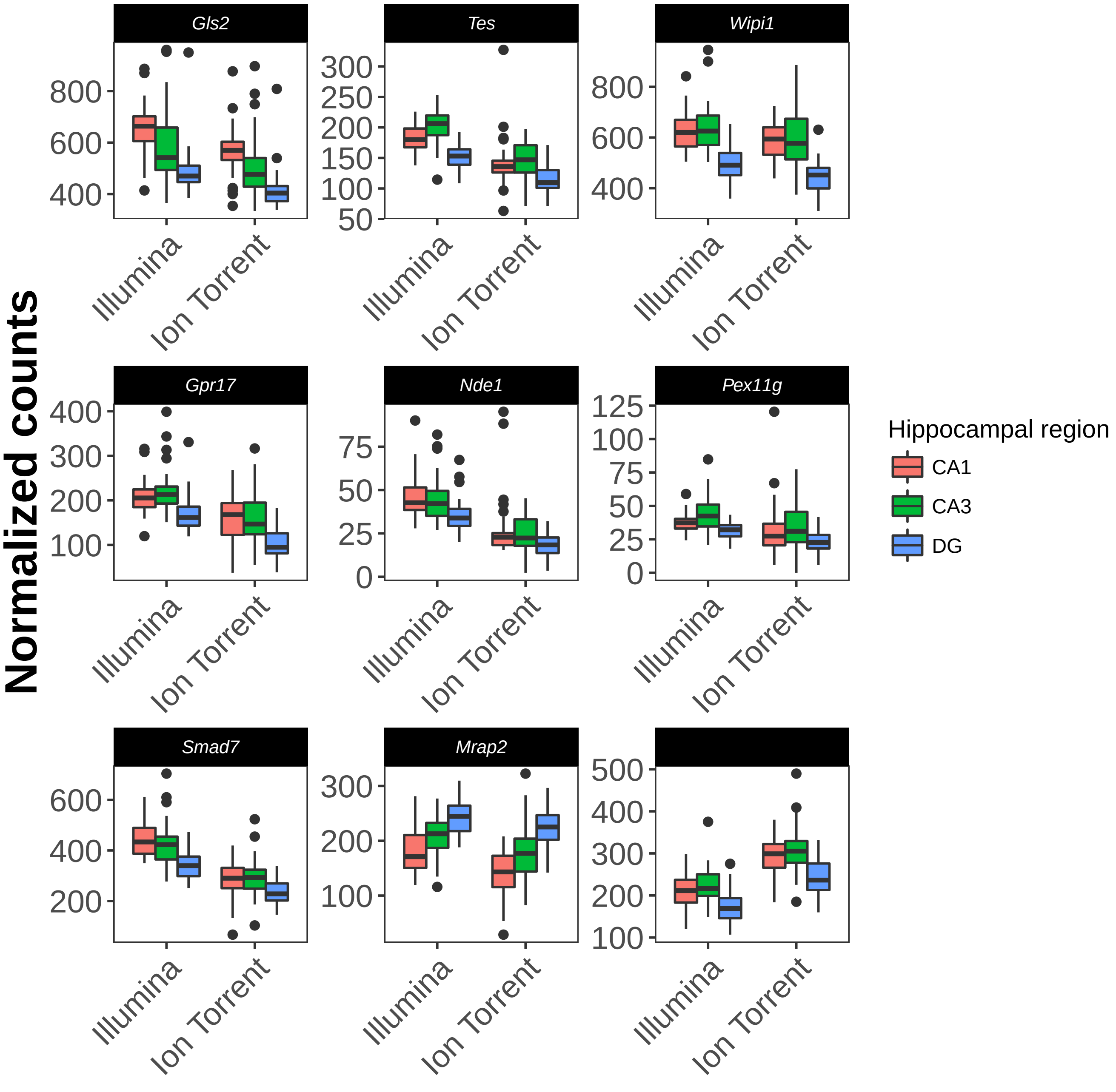

Normalized counts
Gls2
Tes
Wipi1
Gpr17
Nde1
Pex11g
Smad7
Mrap2
Illumina
Ion Torrent
Hippocampal region
CA1
CA3
DG

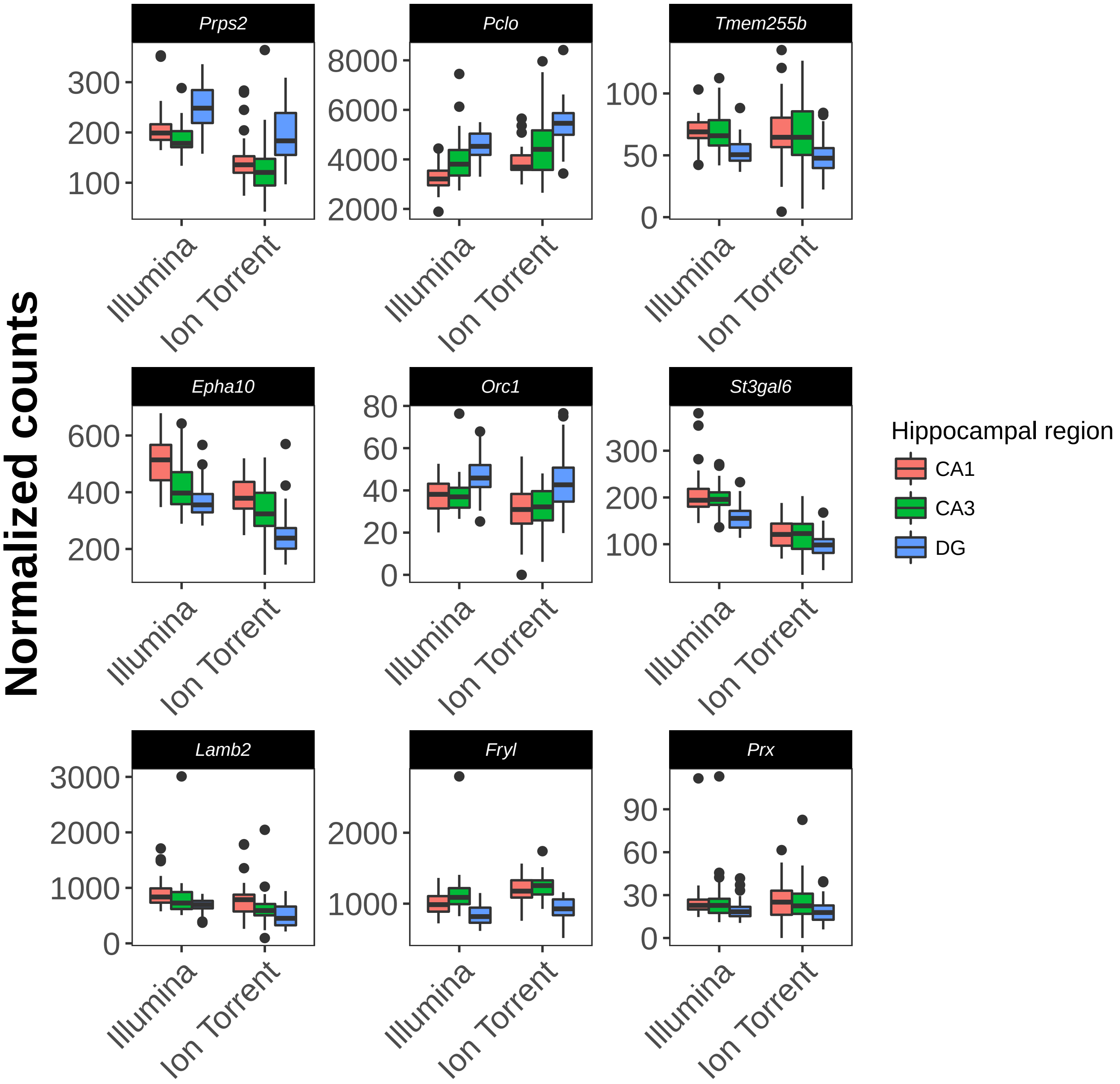
Normalized counts
Prps2
Pclo
Tmem255b
Epha10
Orc1
St3gal6
Lamb2
Fryl
Prx
Illumina
Ion Torrent
Hippocampal region
CA1
CA3
DG

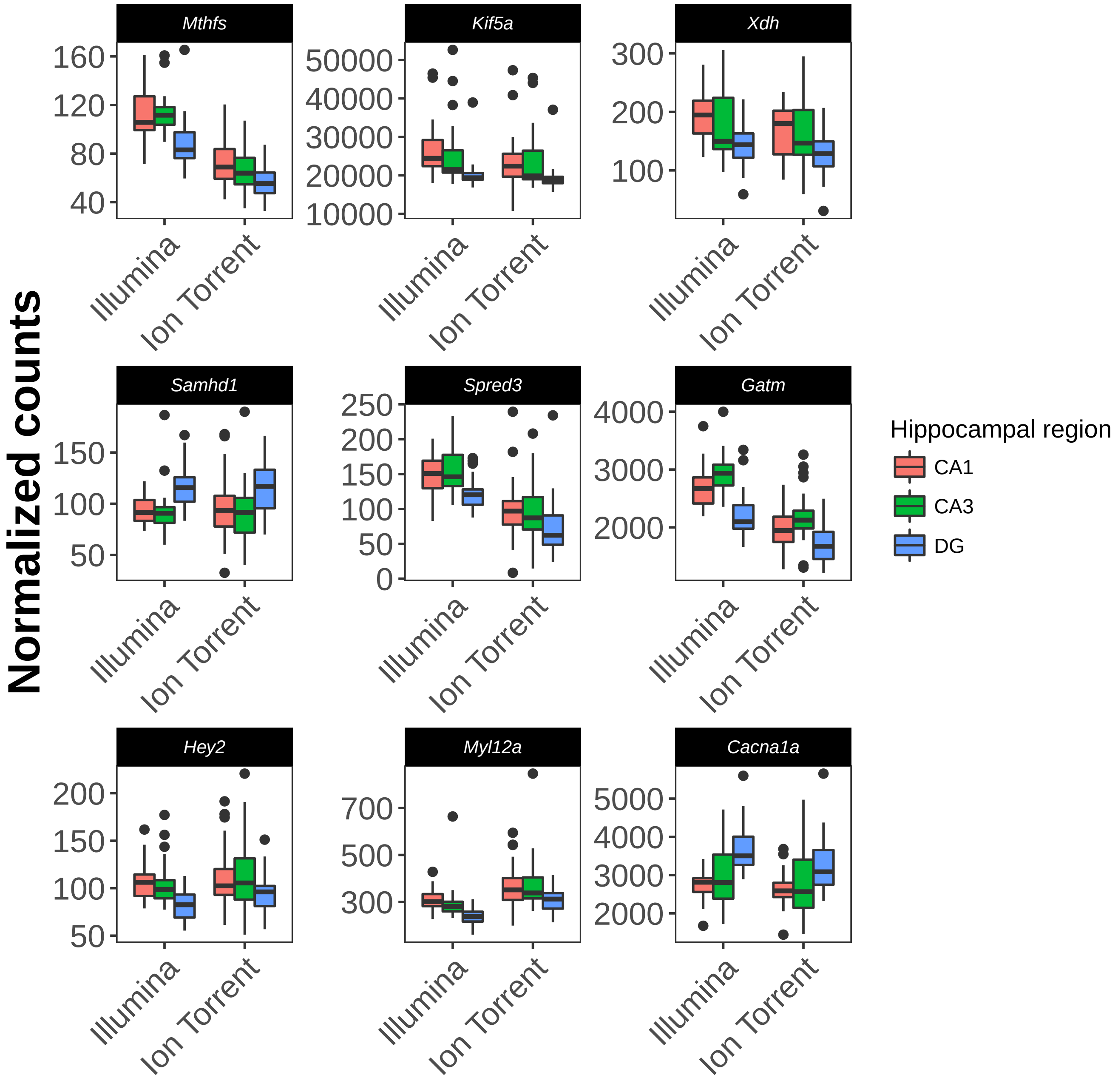

Normalized counts
Mthfs
Kif5a
Xdh
Samhd1
Spred3
Gatm
Hey2
Myl12a
Cacna1a
Illumina
Ion Torrent
Hippocampal region
CA1
CA3
DG

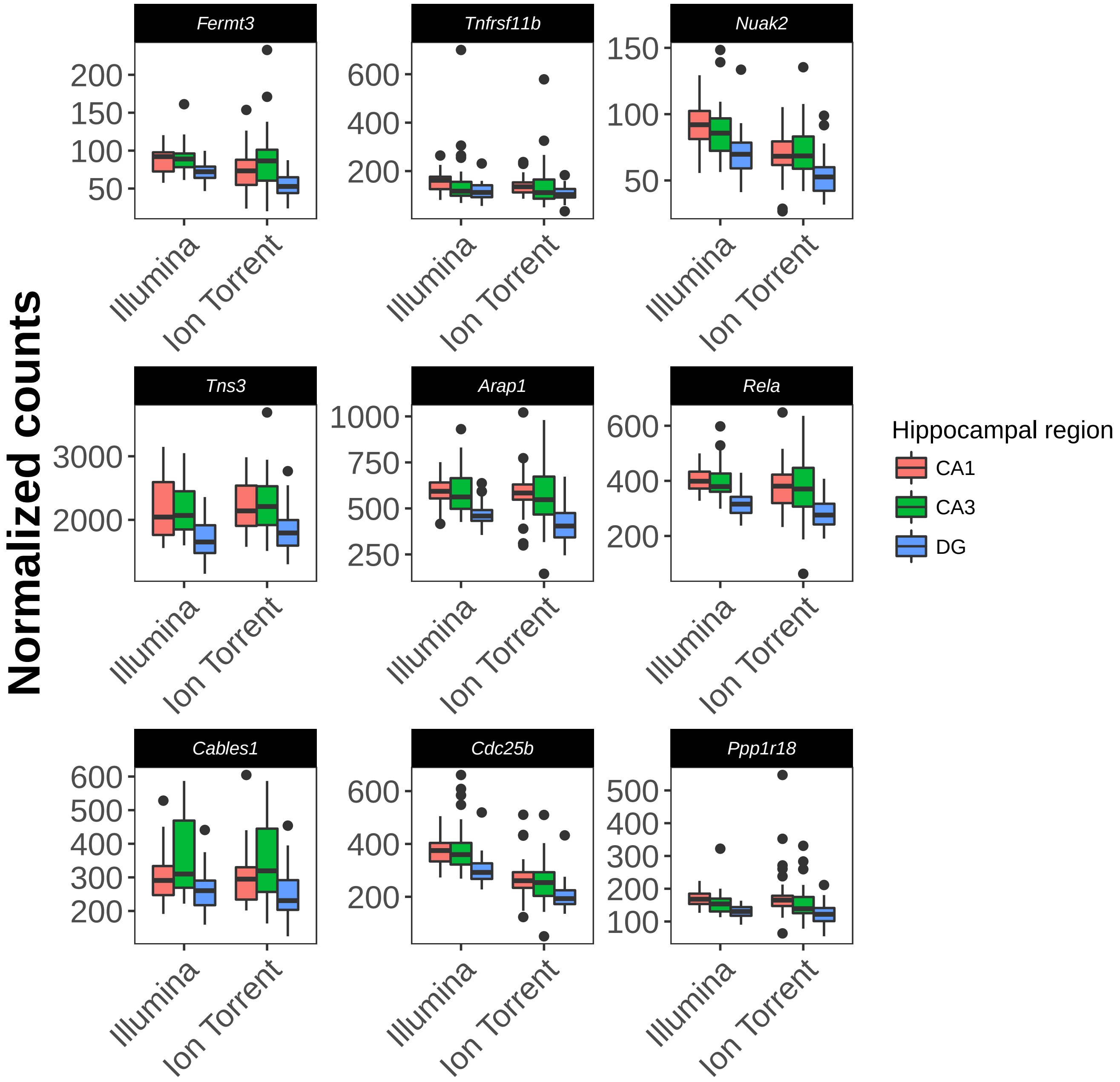
Normalized counts
Fermt3
Tnfrsf11b
Nuak2
Tns3
Arap1
Rela
Cables1
Cdc25b
Ppp1r18
Illumina
Ion Torrent
Hippocampal region
CA1
CA3
DG

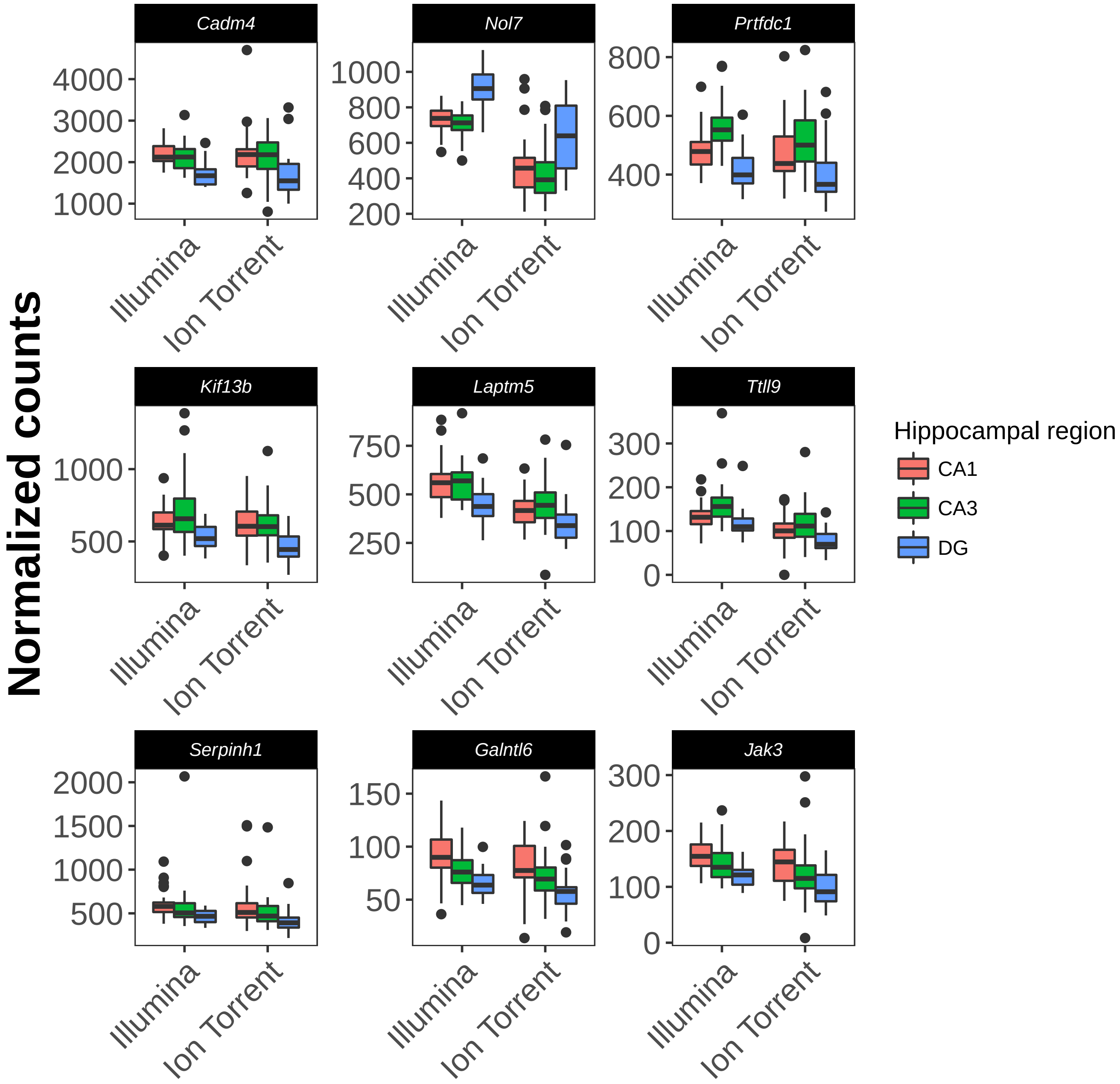
Normalized counts
Cadm4
Nol7
Prtfdc1
Kif13b
Laptm5
Ttll9
Serpinh1
Galntl6
Jak3
Illumina
Ion Torrent
Hippocampal region
CA1
CA3
DG

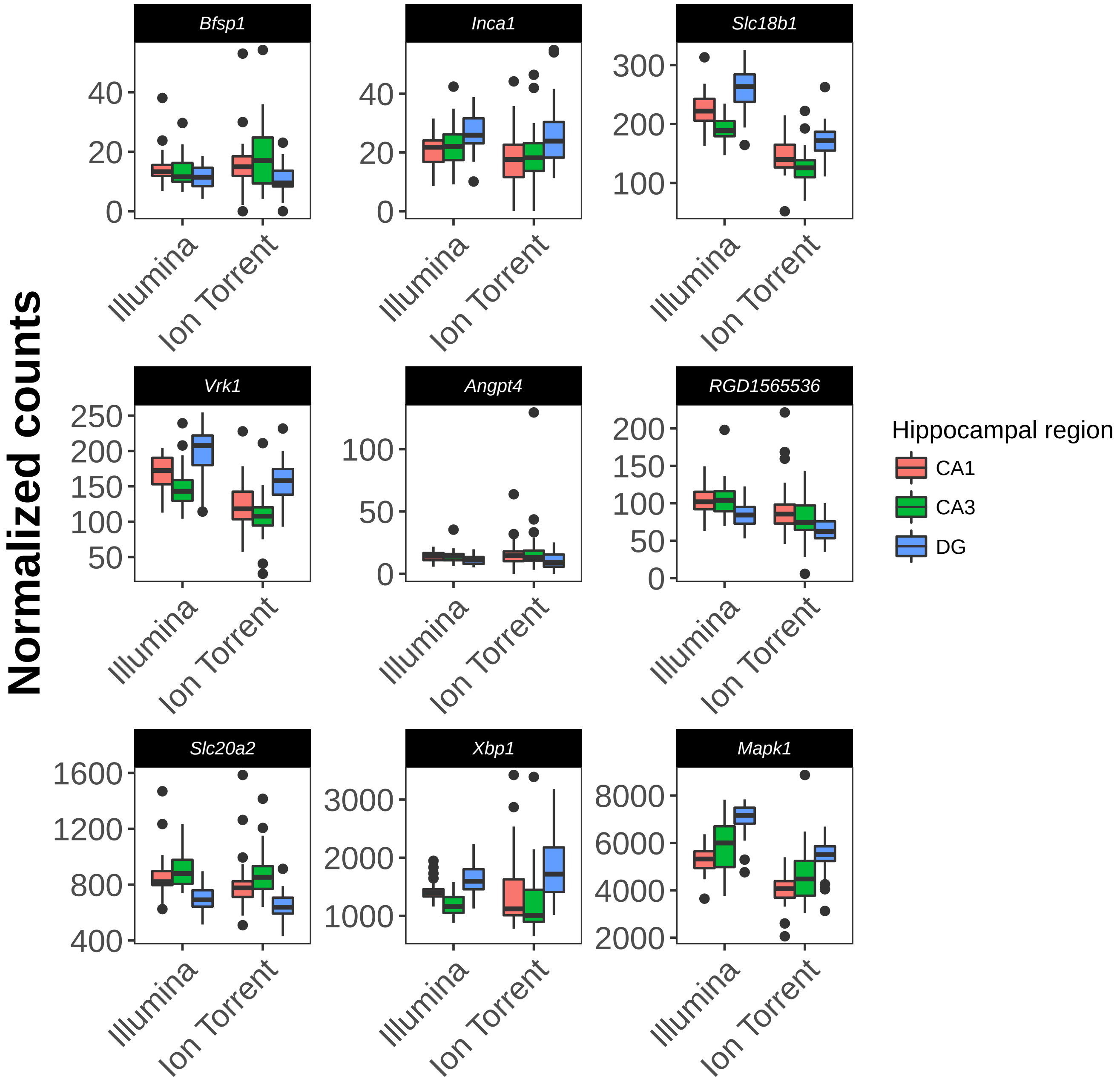

Normalized counts
Bfsp1
Inca1
Slc18b1
Vrk1
Angpt4
RGD1565536
Slc20a2
Xbp1
Mapk1
Illumina
Ion Torrent
Hippocampal region
CA1
CA3
DG

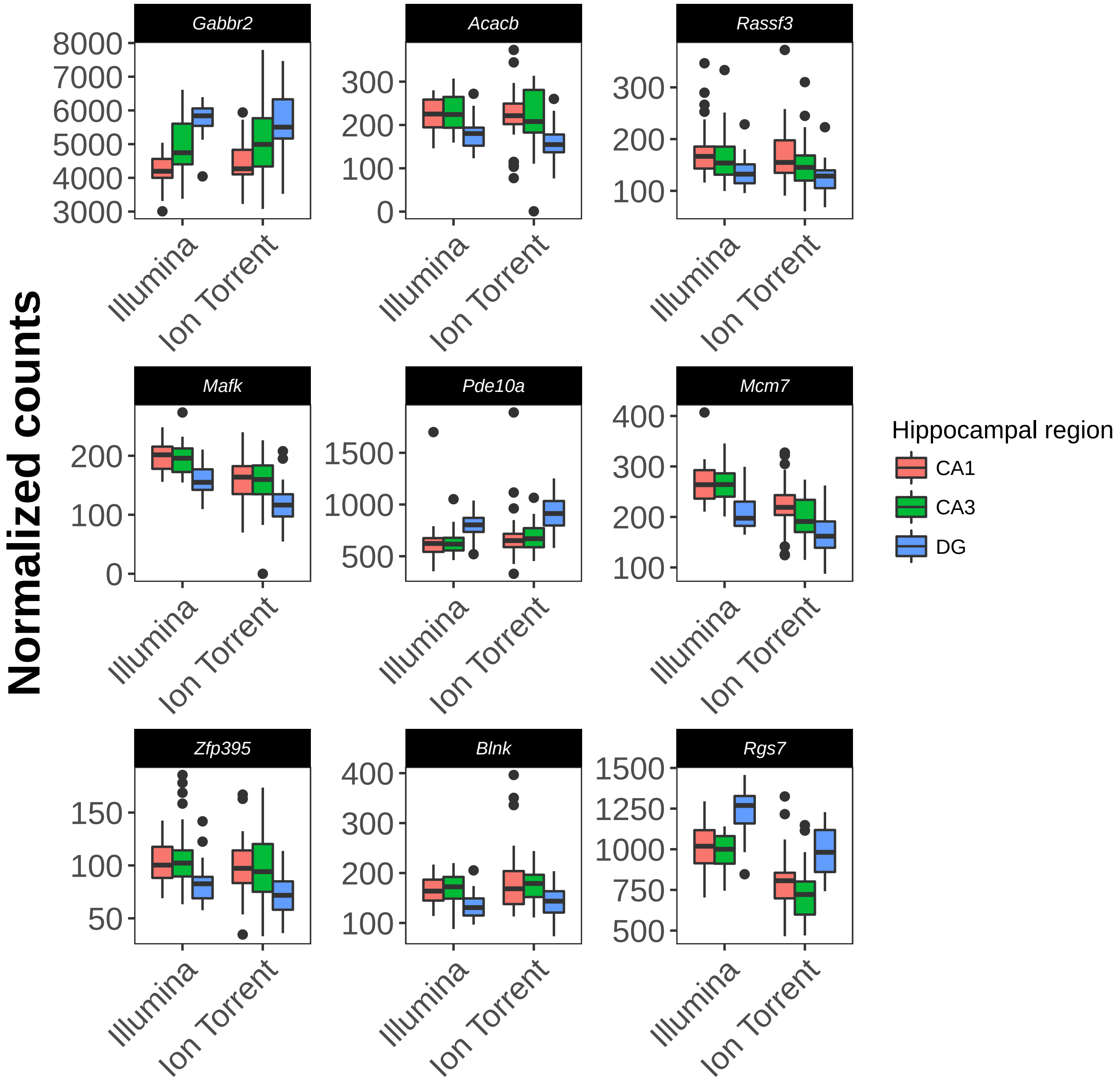

Normalized counts
Gabbr2
Acacb
Rassf3
Mafk
Pde10a
Mcm7
Zfp395
Blnk
Rgs7
Illumina
Ion Torrent
Hippocampal region
CA1
CA3
DG

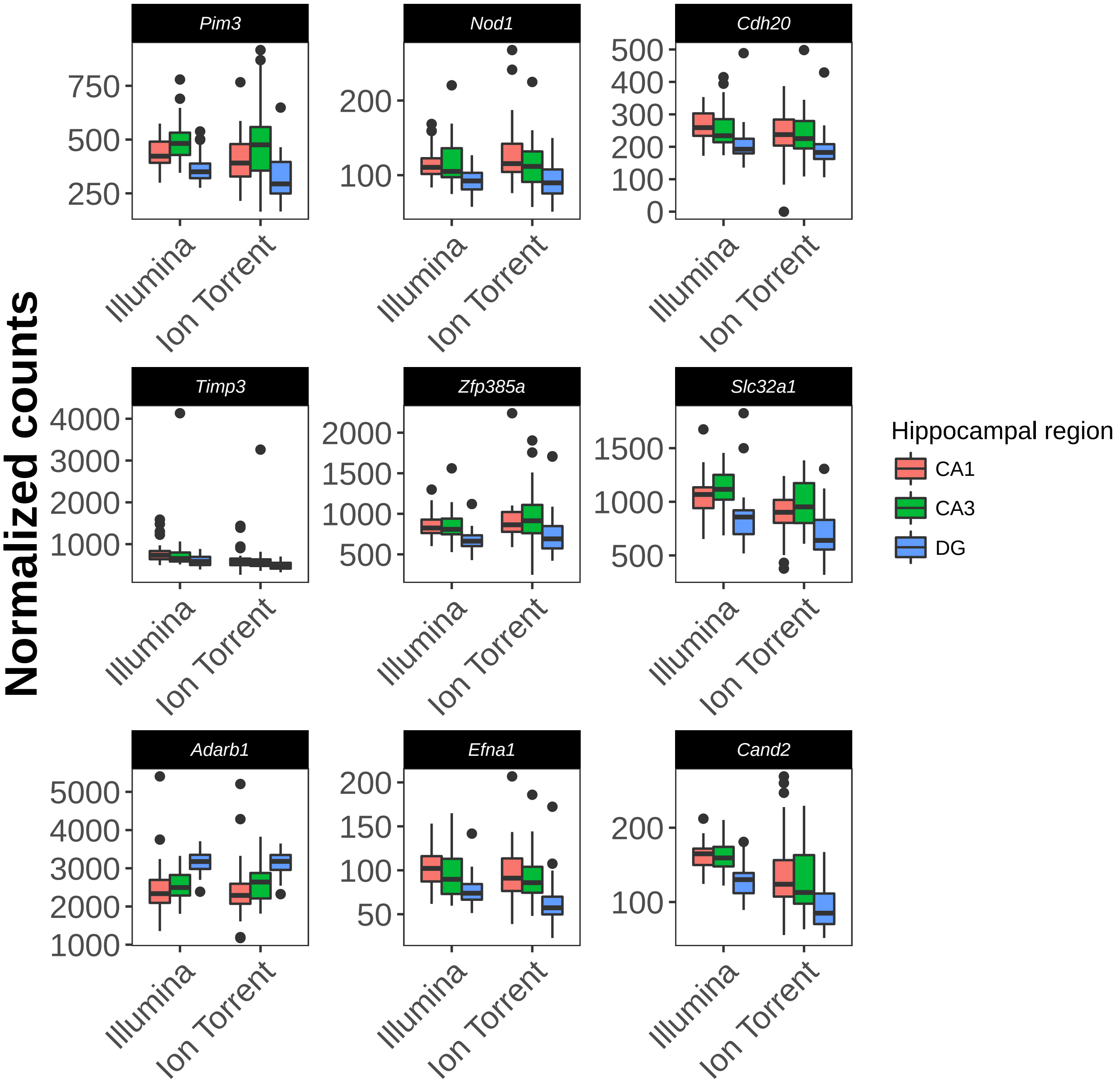
Normalized counts
Pim3
Nod1
Cdh20
Timp3
Zfp385a
Slc32a1
Adarb1
Efna1
Cand2
Illumina
Ion Torrent
Hippocampal region
CA1
CA3
DG

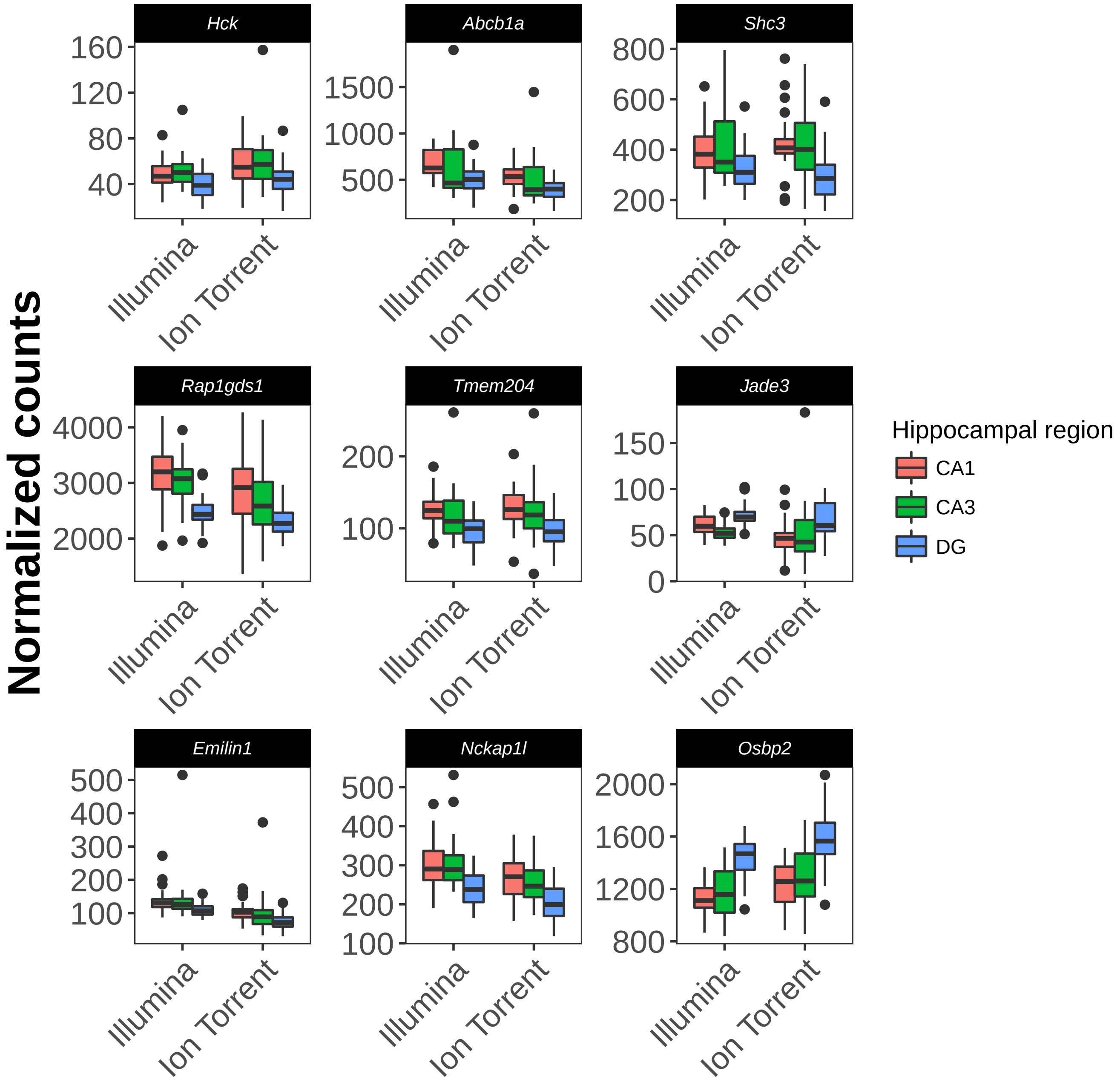

Normalized counts
Hck
160
120
80
40
Abcb1a
1500
1000
500
Shc3
800
600
400
200
Rap1gds1
4000
3000
2000
Tmem204
200
100
Jade3
150
100
50
0
Emilin1
500
400
300
200
100
Nckap1l
500
400
300
200
100
Osbp2
2000
1600
1200
800
Illumina
Ion Torrent
Hippocampal region
CA1
CA3
DG

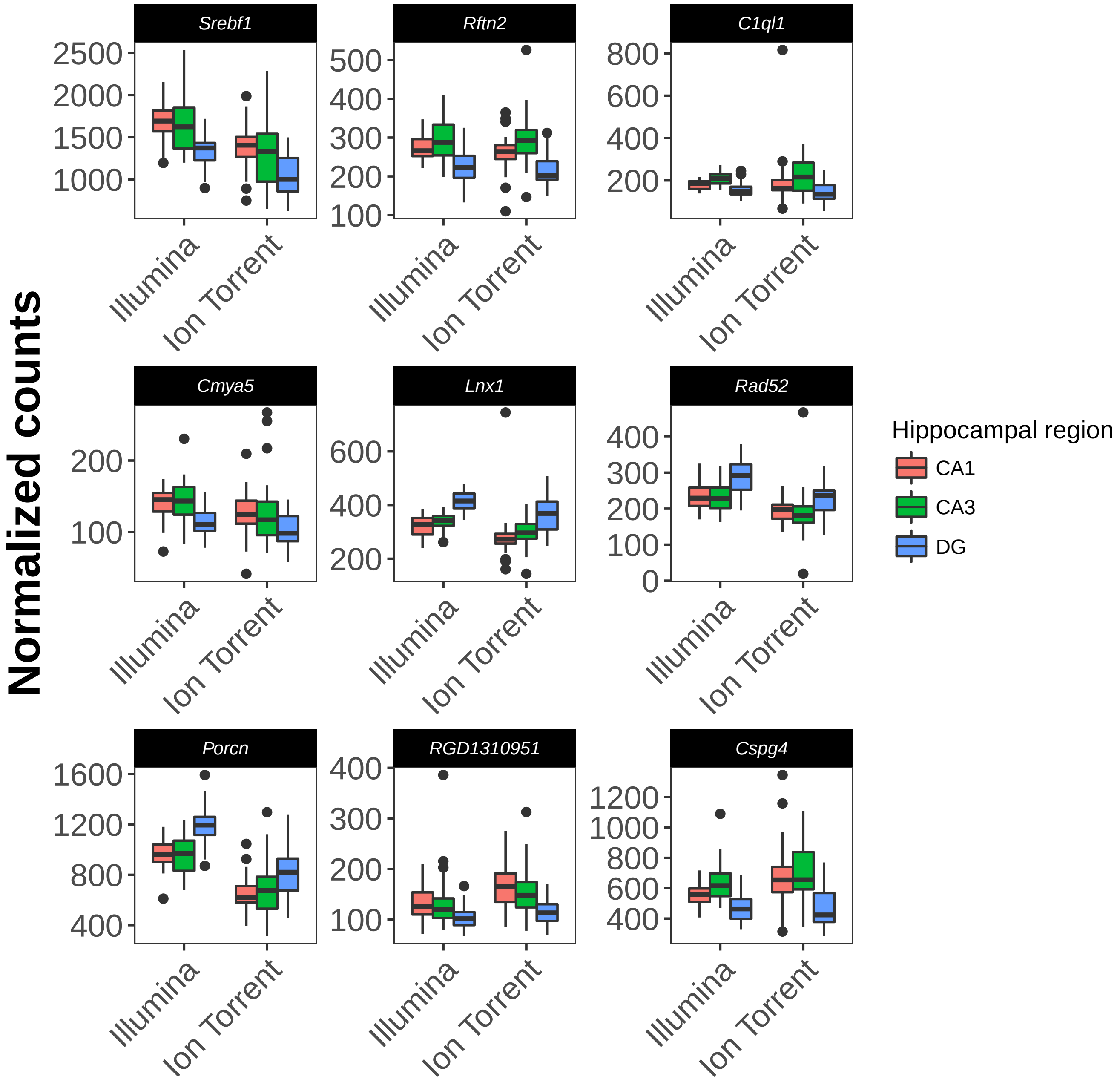
Normalized counts
Srebf1
Rftn2
C1ql1
Cmya5
Lnx1
Rad52
Porcn
RGD1310951
Cspg4
Illumina
Ion Torrent
Hippocampal region
CA1
CA3
DG

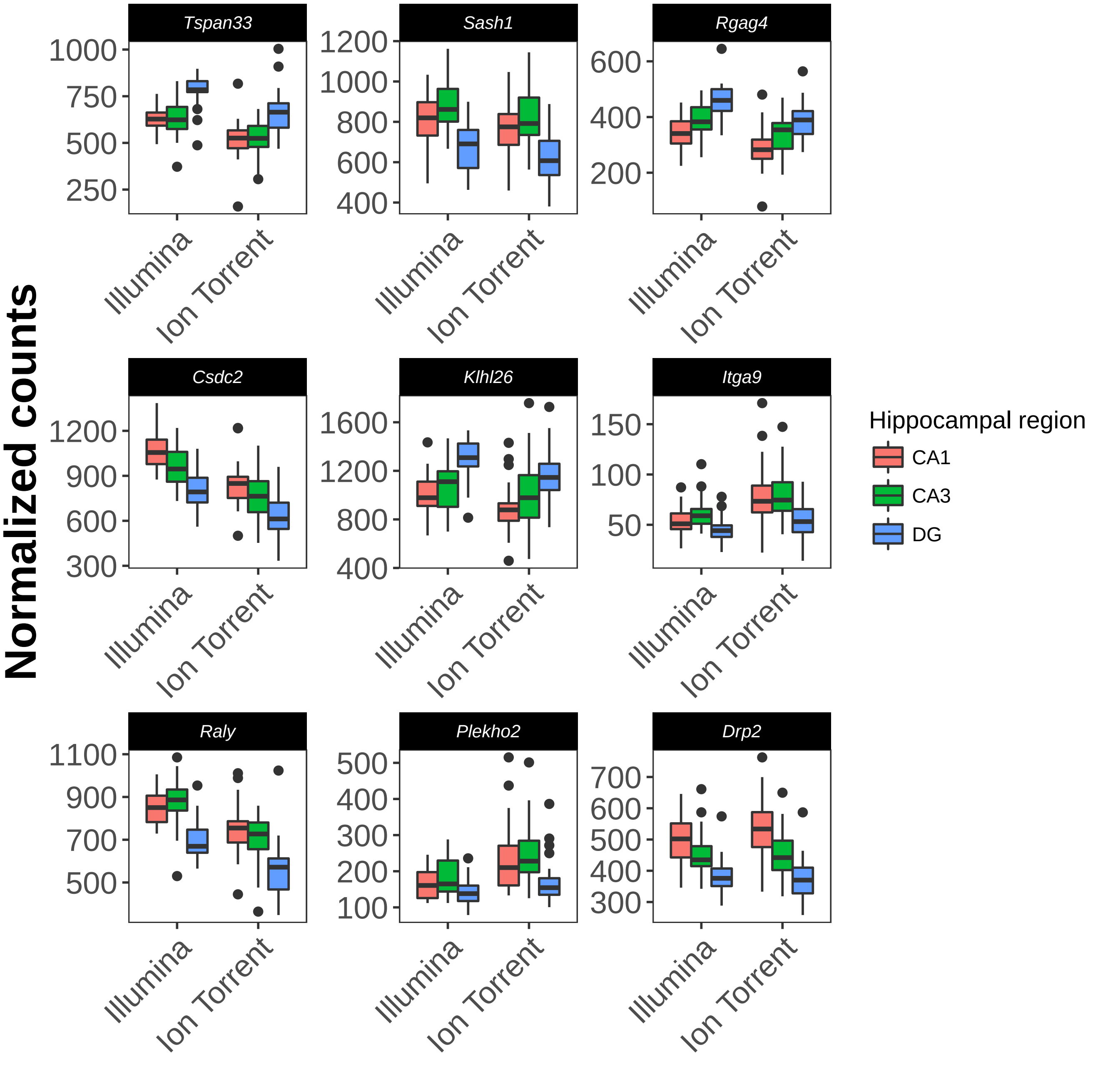

Normalized counts
Tspan33
Sash1
Rgag4
Csdc2
Klhl26
Itga9
Raly
Plekho2
Drp2
Illumina
Ion Torrent
Hippocampal region
CA1
CA3
DG

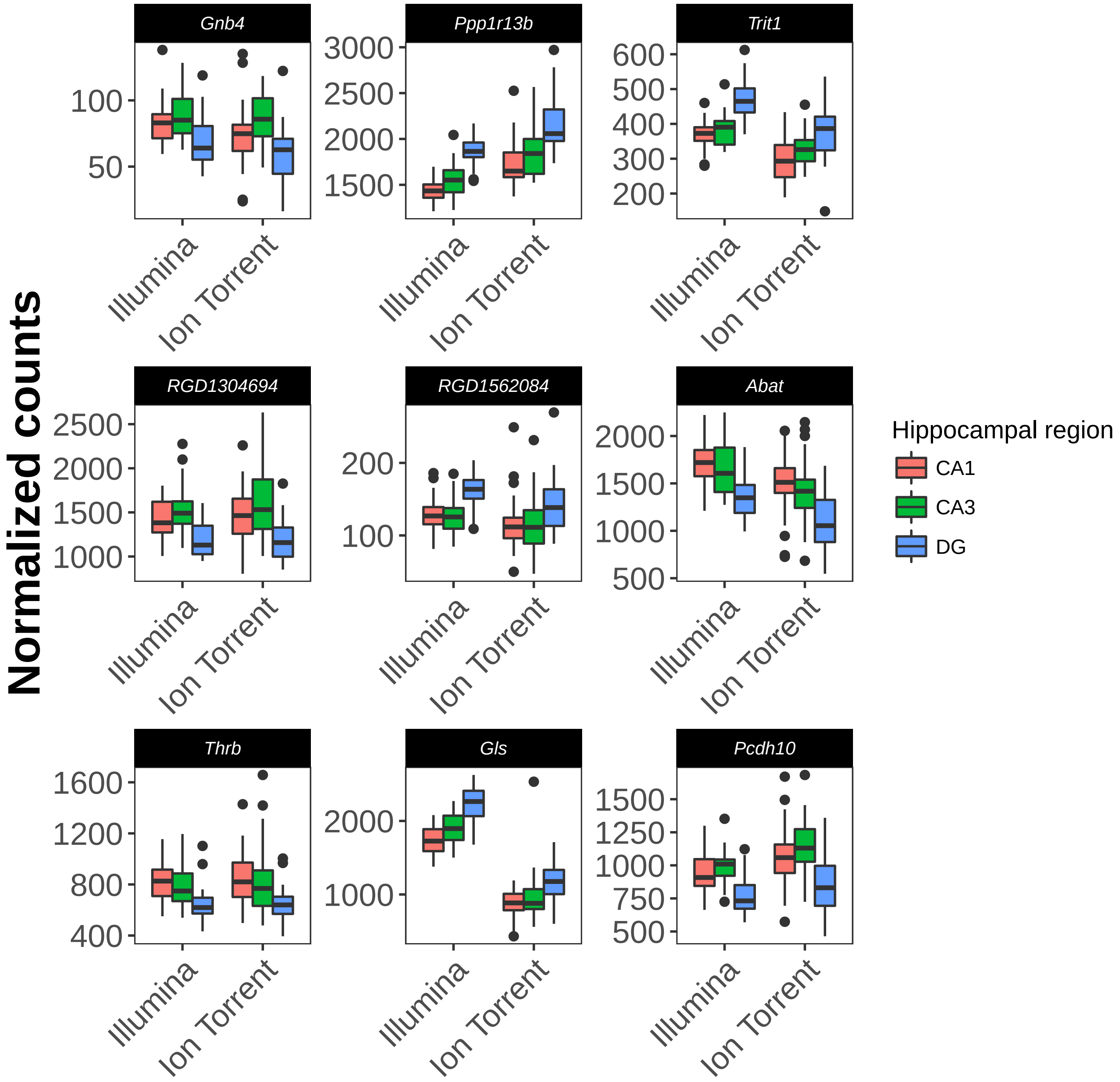
Normalized counts
Gnb4
Ppp1r13b
Trit1
RGD1304694
RGD1562084
Abat
Thrb
Gls
Pcdh10
Illumina
Ion Torrent
Hippocampal region
CA1
CA3
DG

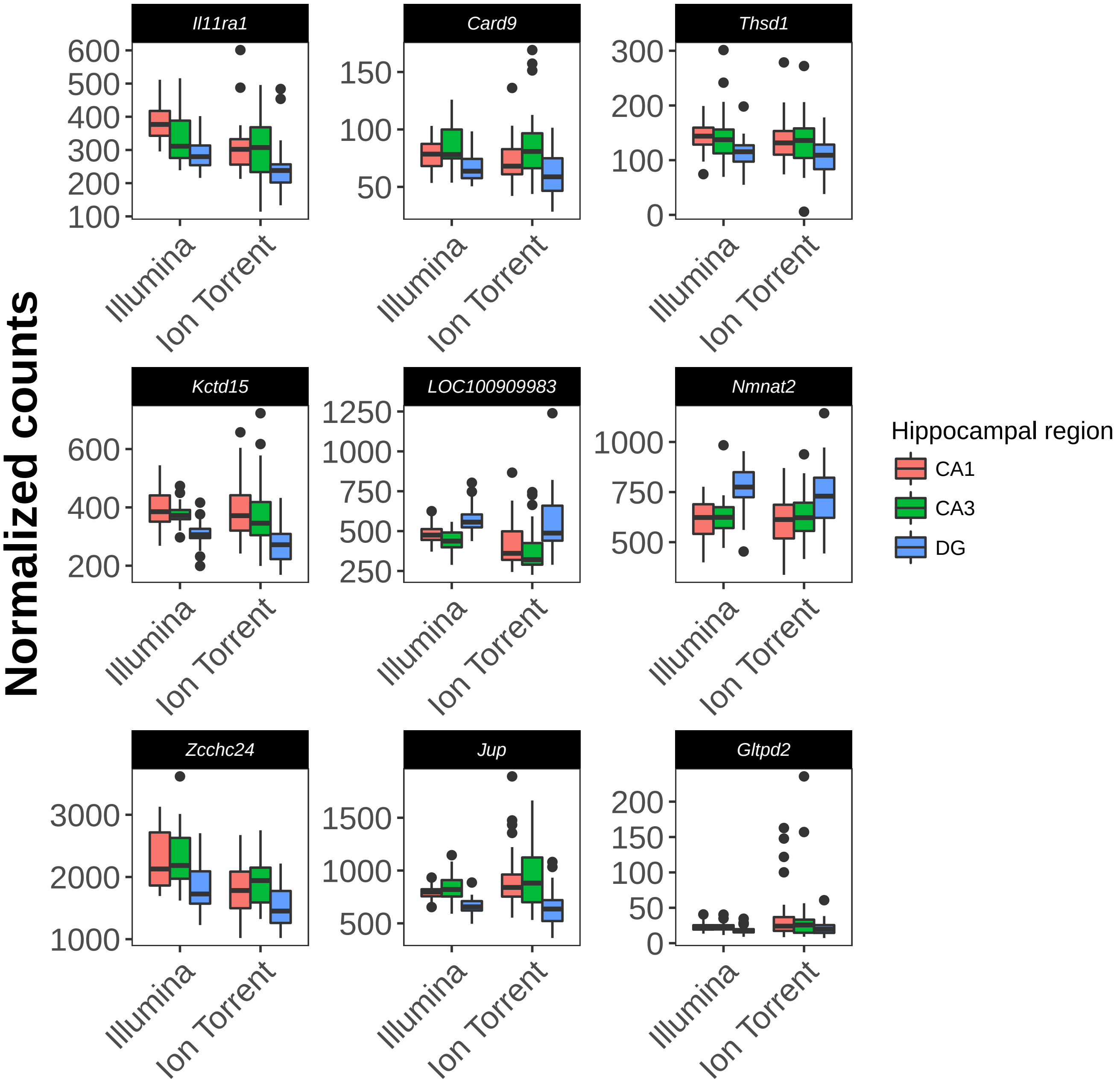
Normalized counts
Il11ra1
Card9
Thsd1
Kctd15
LOC100909983
Nmnat2
Zcchc24
Jup
Gltpd2
Illumina
Ion Torrent
Hippocampal region
CA1
CA3
DG

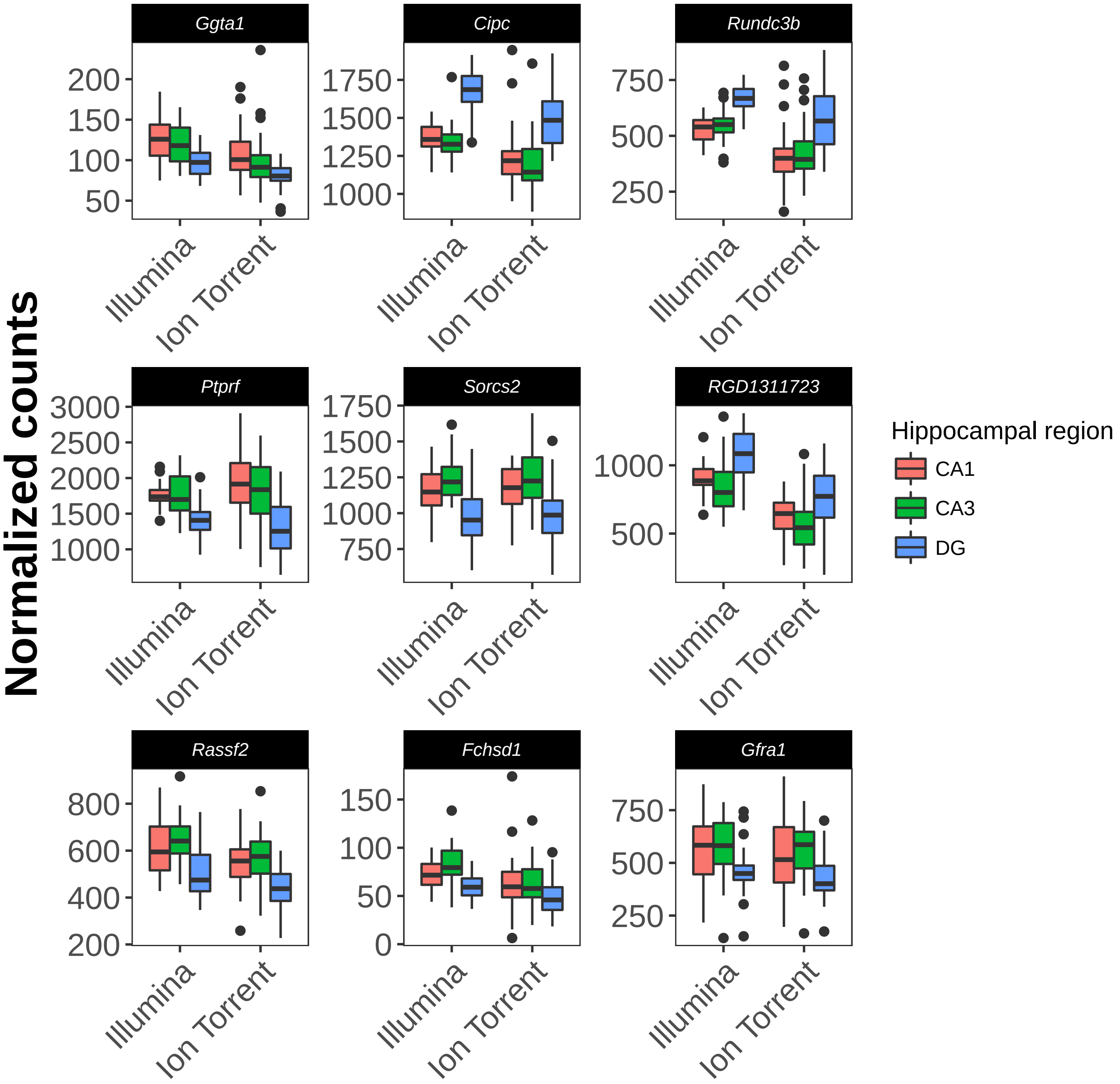

Normalized counts
Ggta1
Cipc
Rundc3b
Ptprf
Sorcs2
RGD1311723
Rassf2
Fchsd1
Gfra1
Illumina
Ion Torrent
Hippocampal region
CA1
CA3
DG

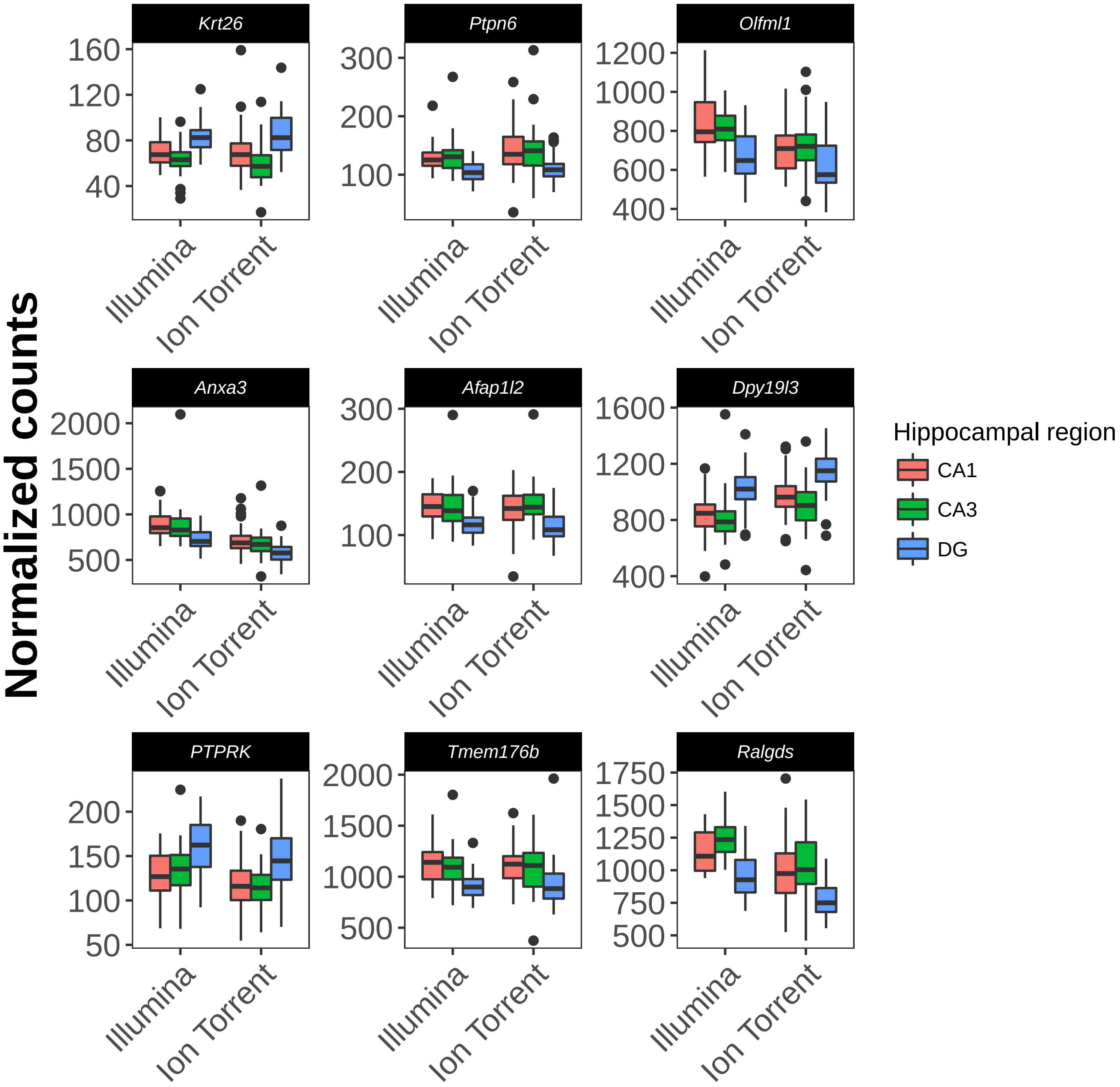

Normalized counts
Krt26
Ptpn6
Olfml1
Anxa3
Afap1l2
Dpy19l3
PTPRK
Tmem176b
Ralgds
Illumina
Ion Torrent
Hippocampal region
CA1
CA3
DG

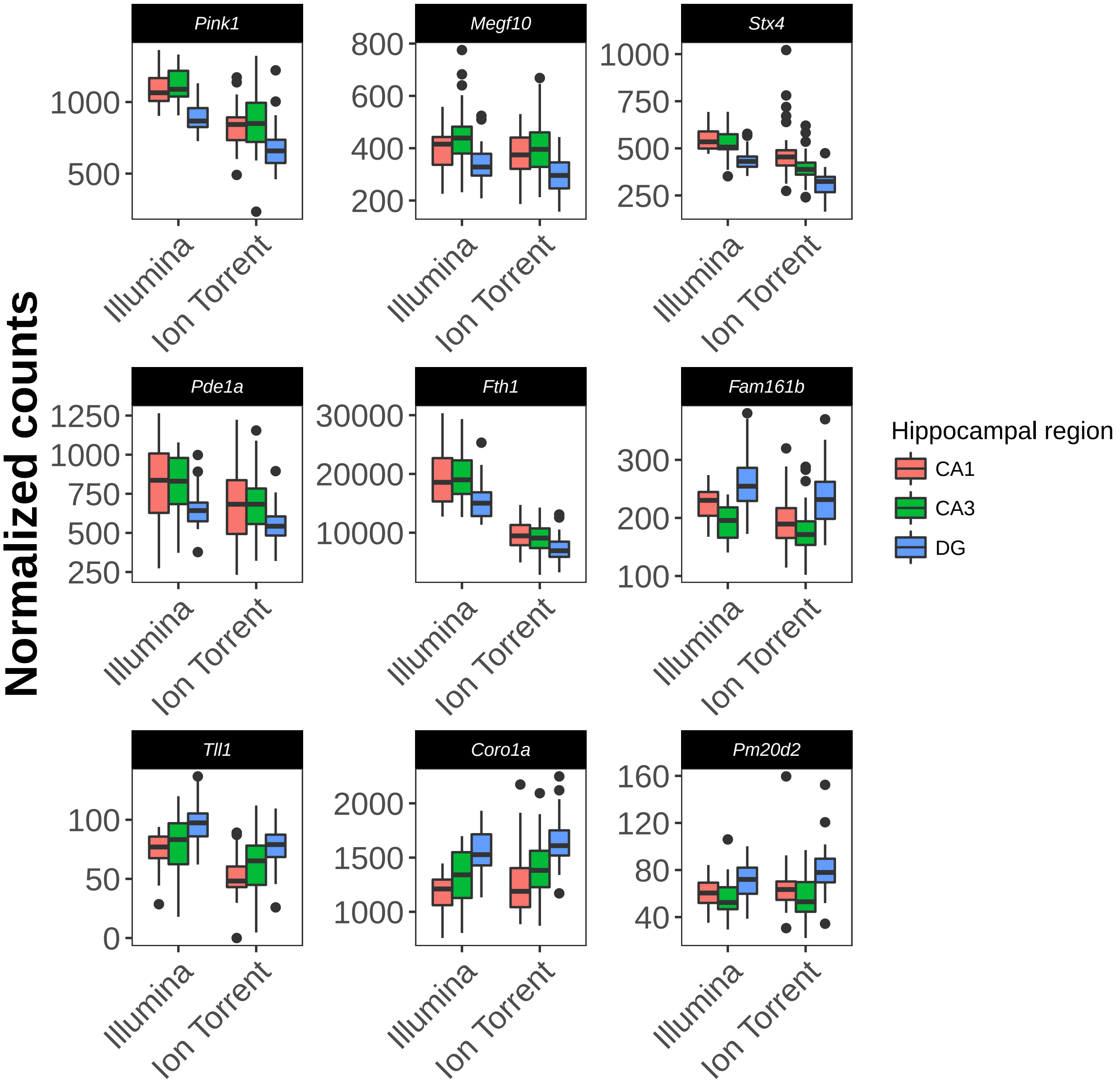

Normalized counts
Pink1
Megf10
Stx4
Pde1a
Fth1
Fam161b
Tll1
Coro1a
Pm20d2
Illumina
Ion Torrent
Hippocampal region
CA1
CA3
DG

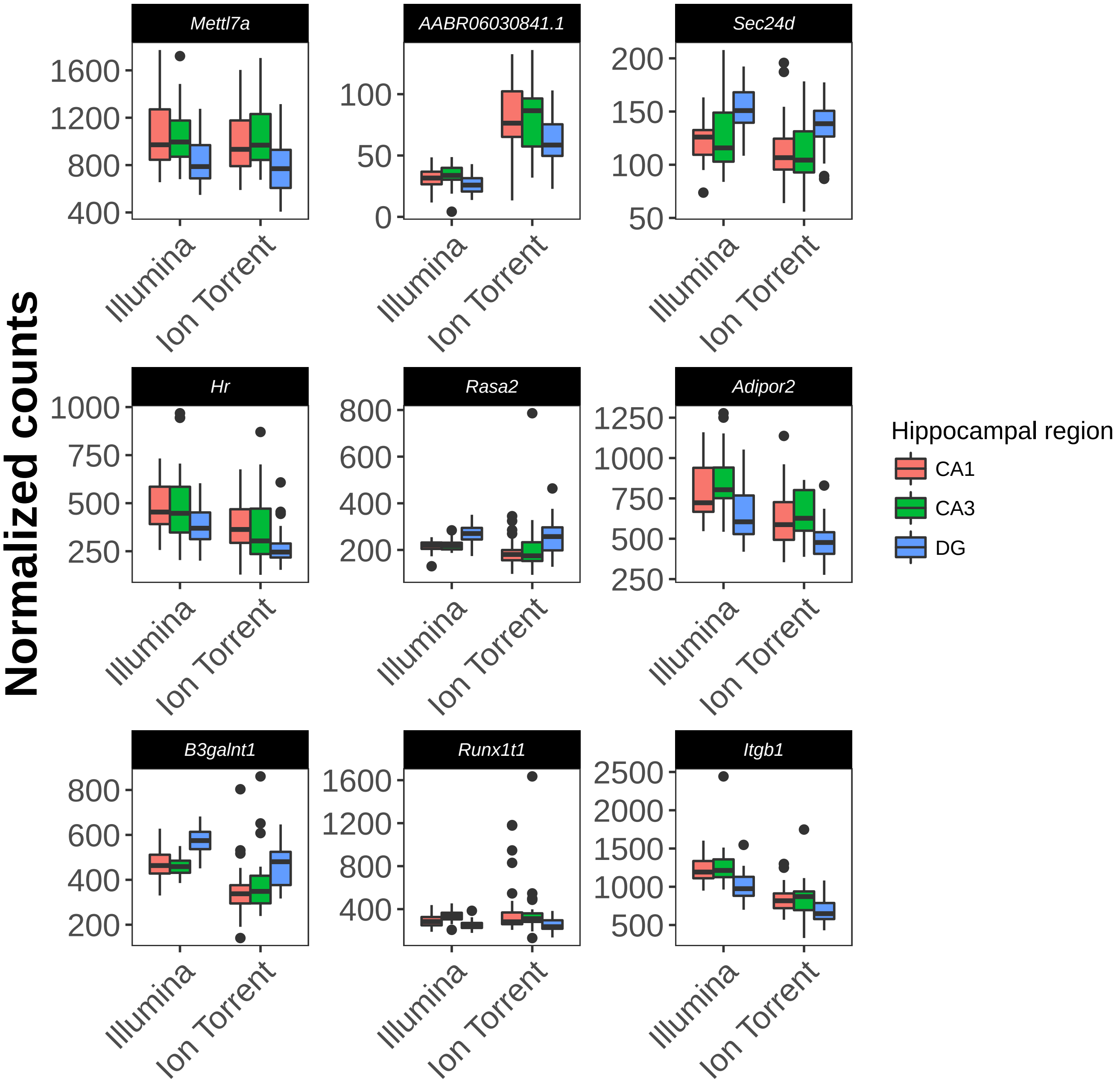
Mettl7a
AABR06030841.1
Sec24d
Hr
Rasa2
Adipor2
B3galnt1
Runx1t1
Itgb1
Normalized counts
Illumina
Ion Torrent
Hippocampal region
CA1
CA3
DG

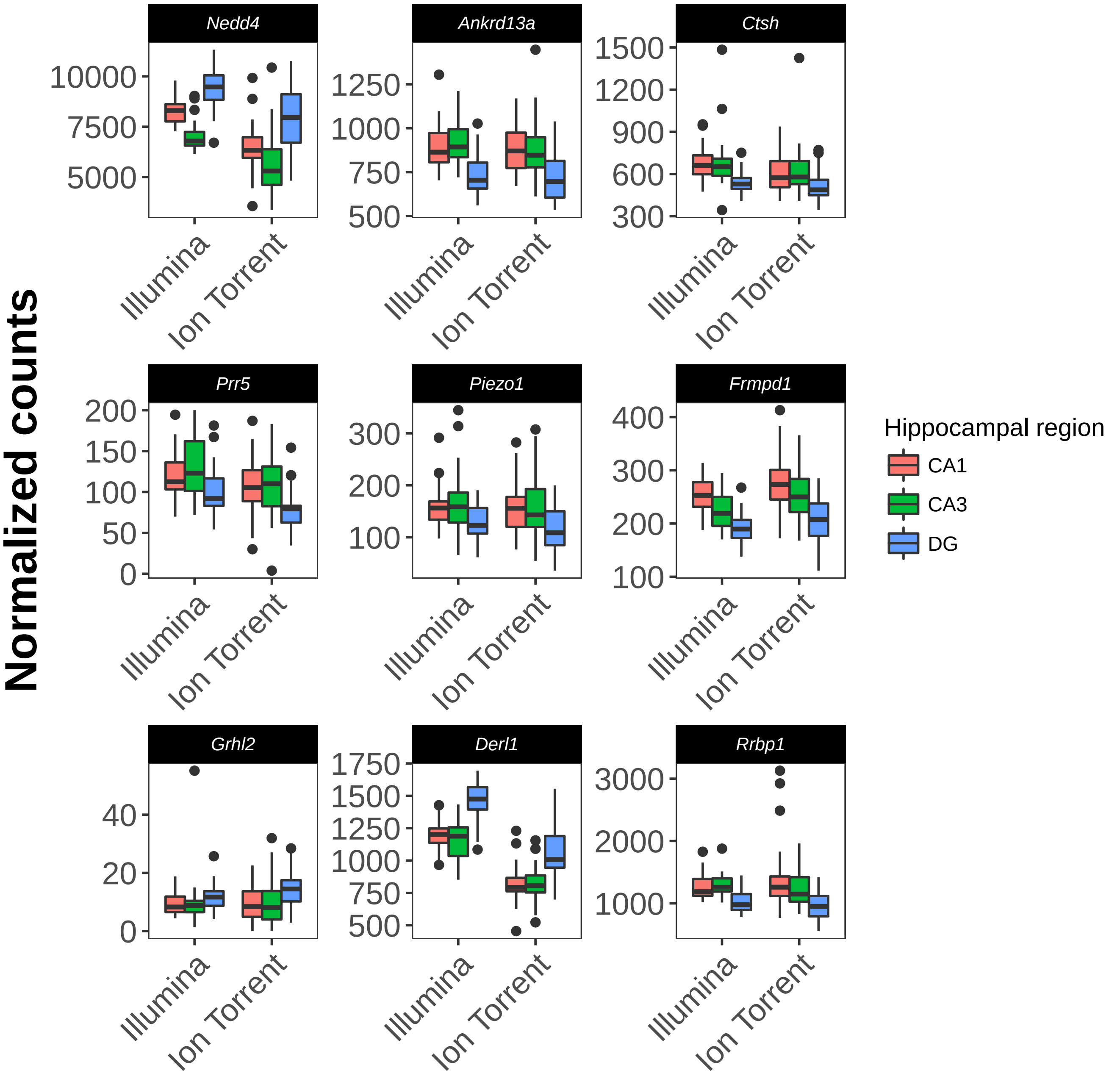
Normalized counts
Nedd4
Ankrd13a
Ctsh
Prr5
Piezo1
Frmpd1
Grhl2
Derl1
Rrbp1
Illumina
Ion Torrent
Hippocampal region
CA1
CA3
DG

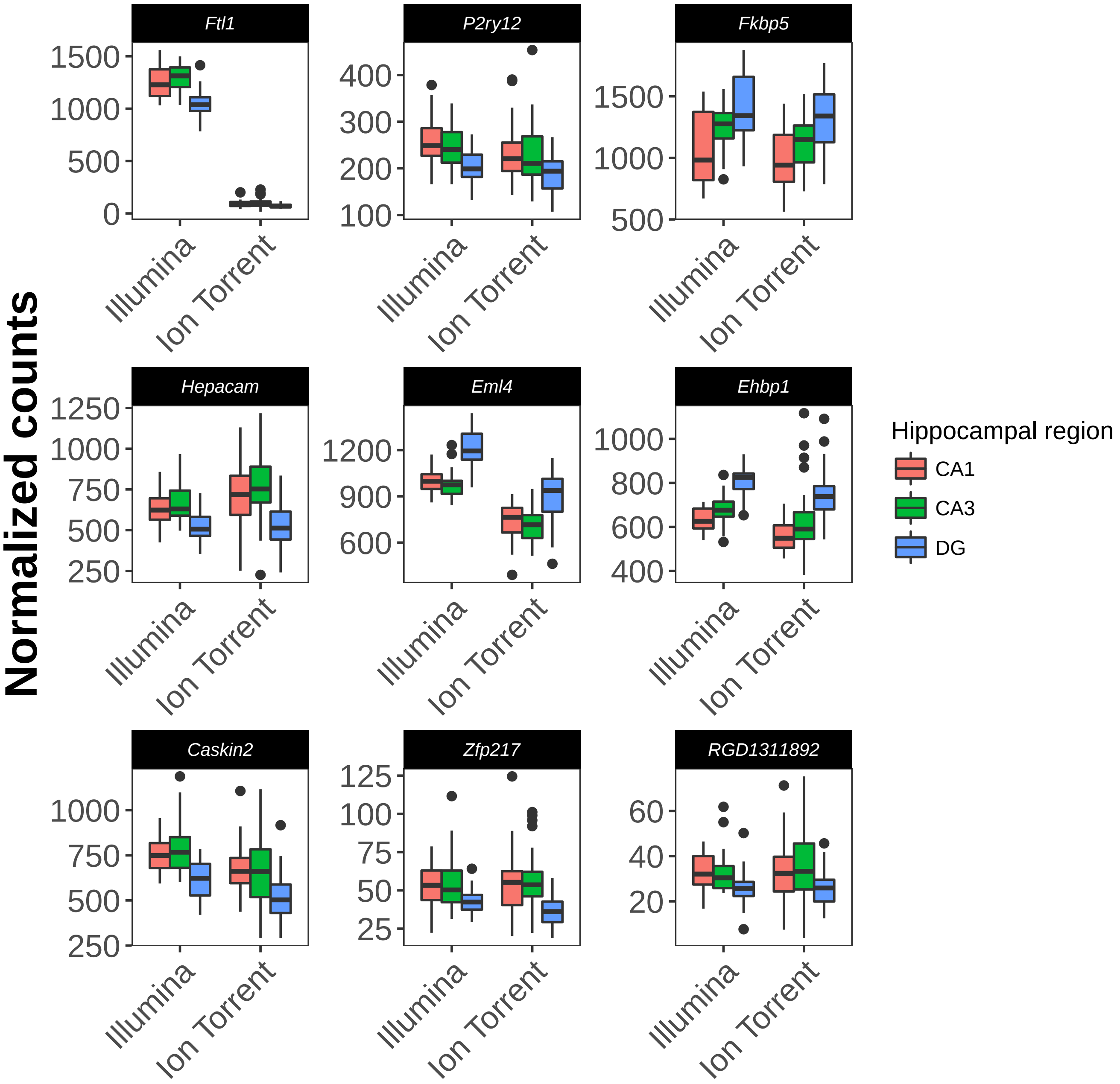

Normalized counts
Ftl1
P2ry12
Fkbp5
Hepacam
Eml4
Ehbp1
Caskin2
Zfp217
RGD1311892
Illumina
Ion Torrent
Hippocampal region
CA1
CA3
DG

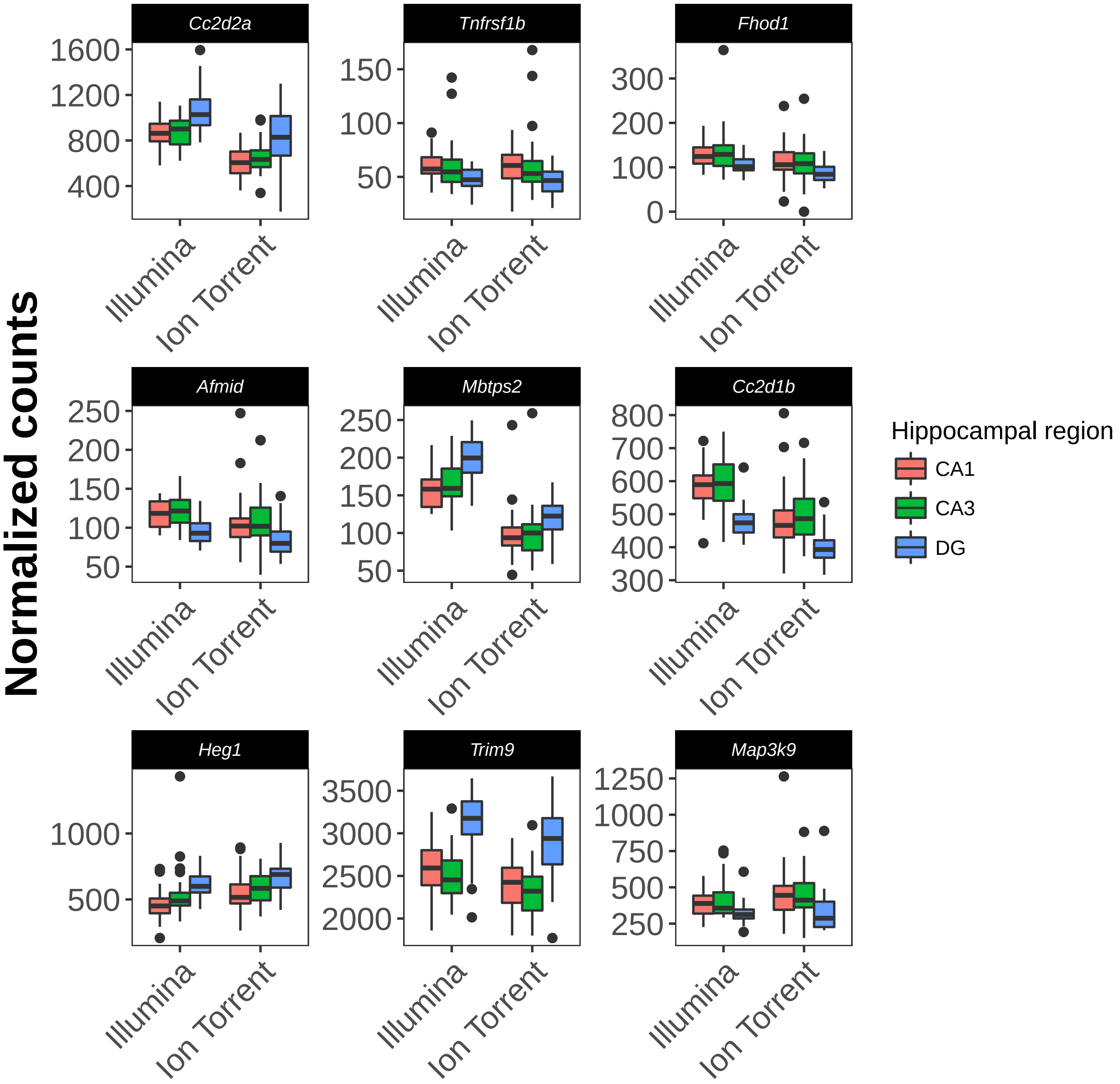

Normalized counts
Cc2d2a
Tnfrsf1b
Fhod1
Afmid
Mbtps2
Cc2d1b
Heg1
Trim9
Map3k9
Illumina
Ion Torrent
Hippocampal region
CA1
CA3
DG

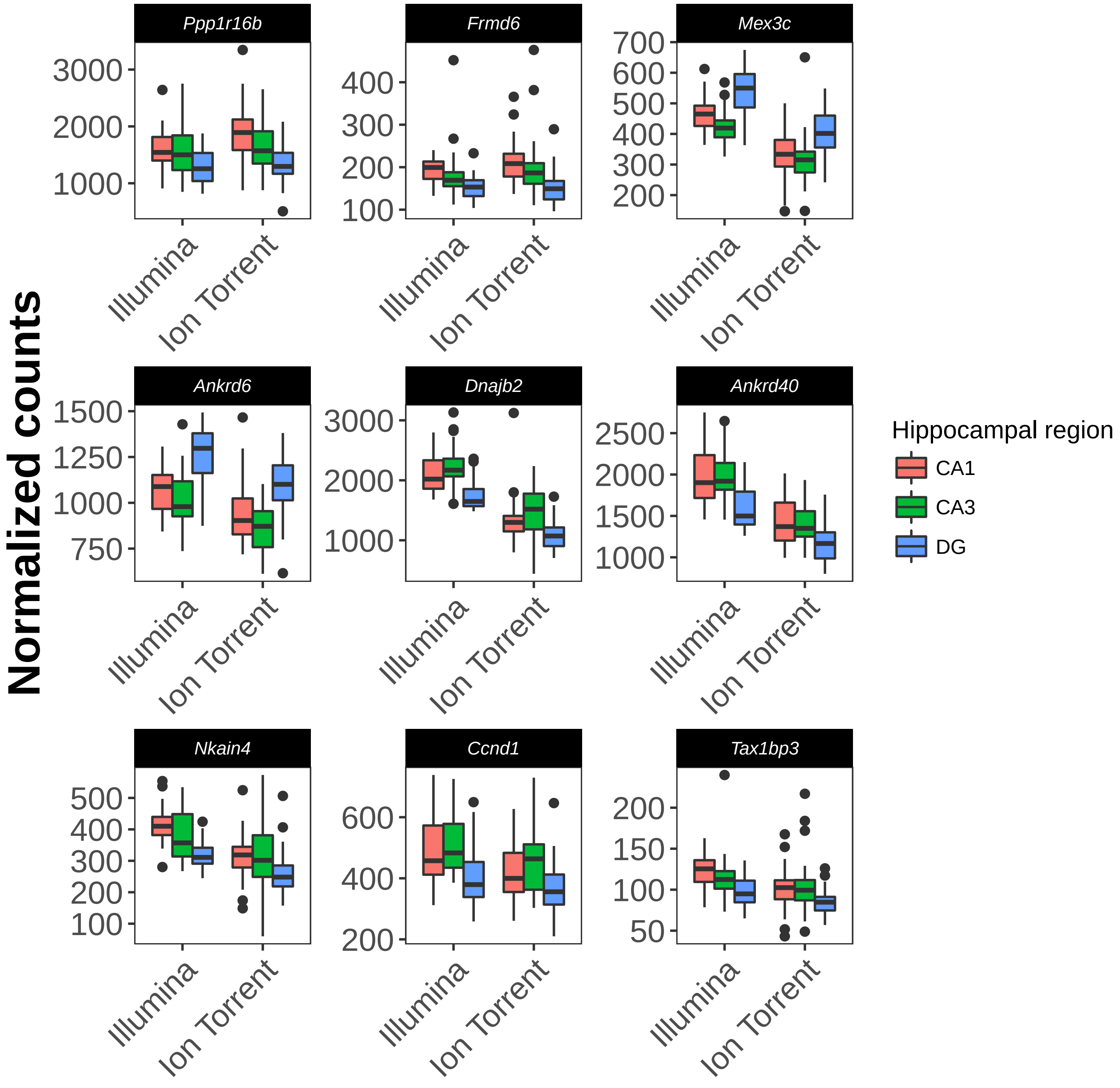
Normalized counts
Ppp1r16b
Frmd6
Mex3c
Ankrd6
Dnajb2
Ankrd40
Nkain4
Ccnd1
Tax1bp3
Illumina
Ion Torrent
Hippocampal region
CA1
CA3
DG

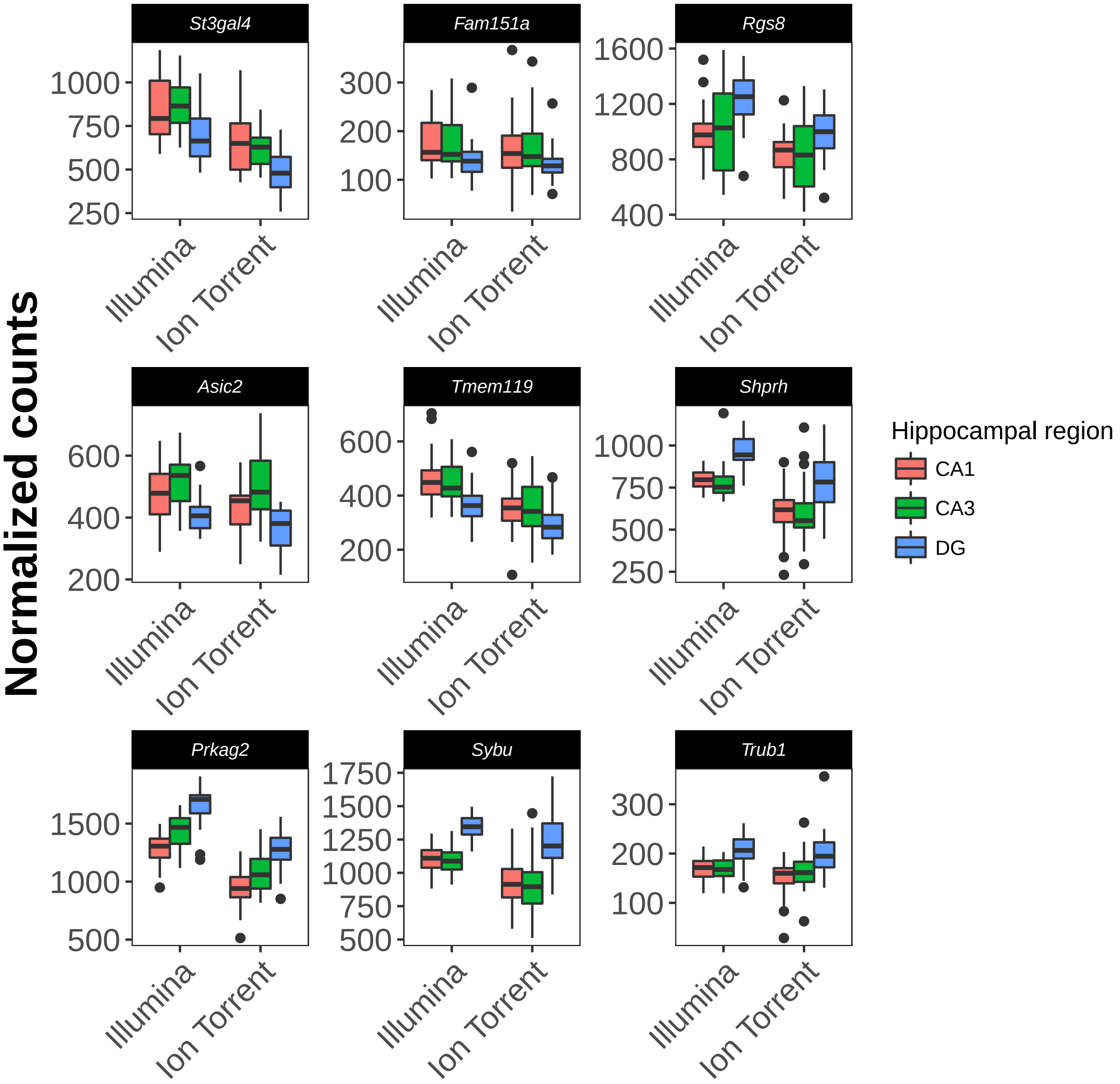

Normalized counts
St3gal4
Fam151a
Rgs8
Asic2
Tmem119
Shprh
Prkag2
Sybu
Trub1
Illumina
Ion Torrent
Hippocampal region
CA1
CA3
DG

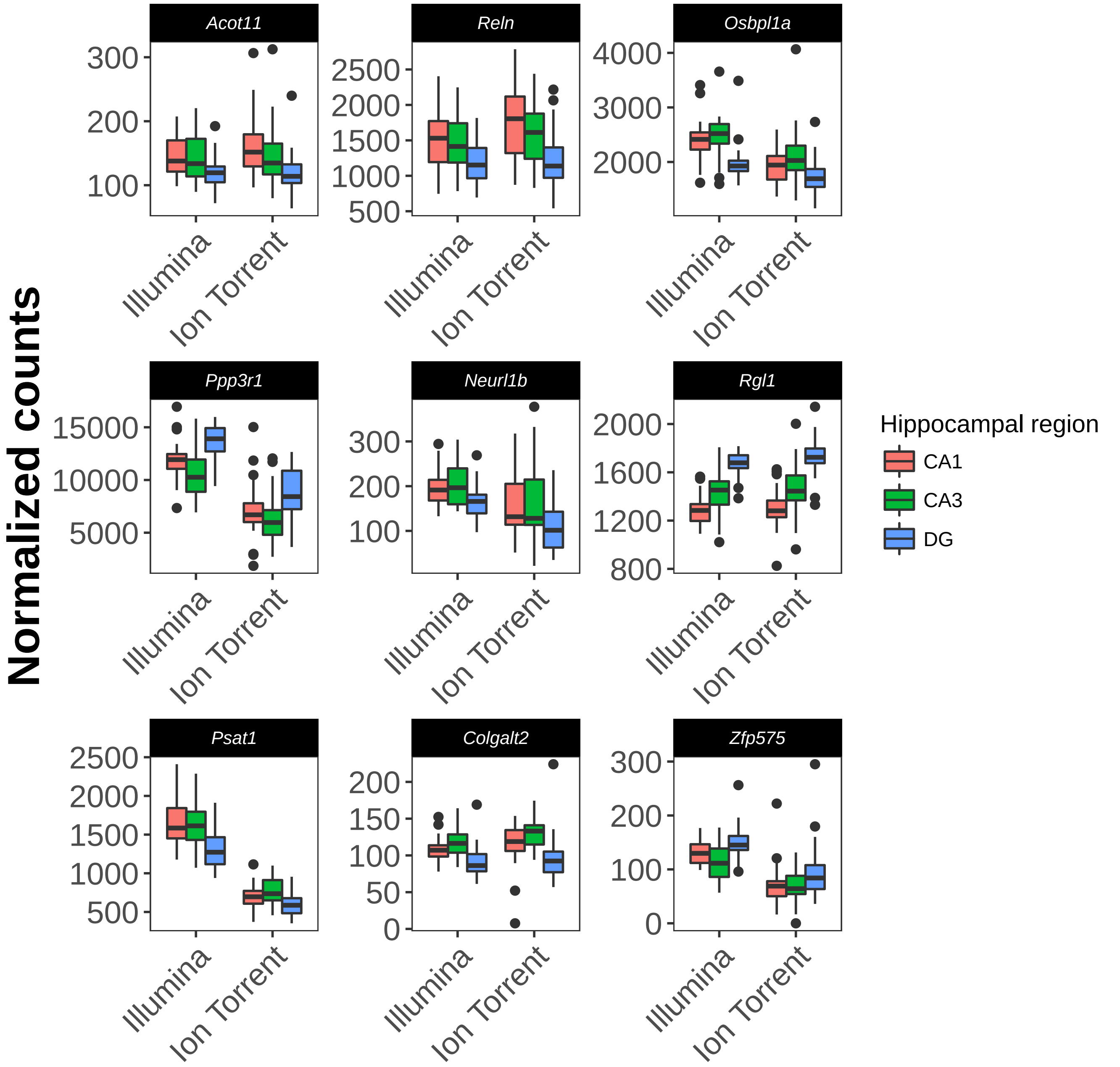
Normalized counts
Acot11
Reln
Osbpl1a
Ppp3r1
Neurl1b
Rgl1
Psat1
Colgalt2
Zfp575
Illumina
Ion Torrent
Hippocampal region
CA1
CA3
DG

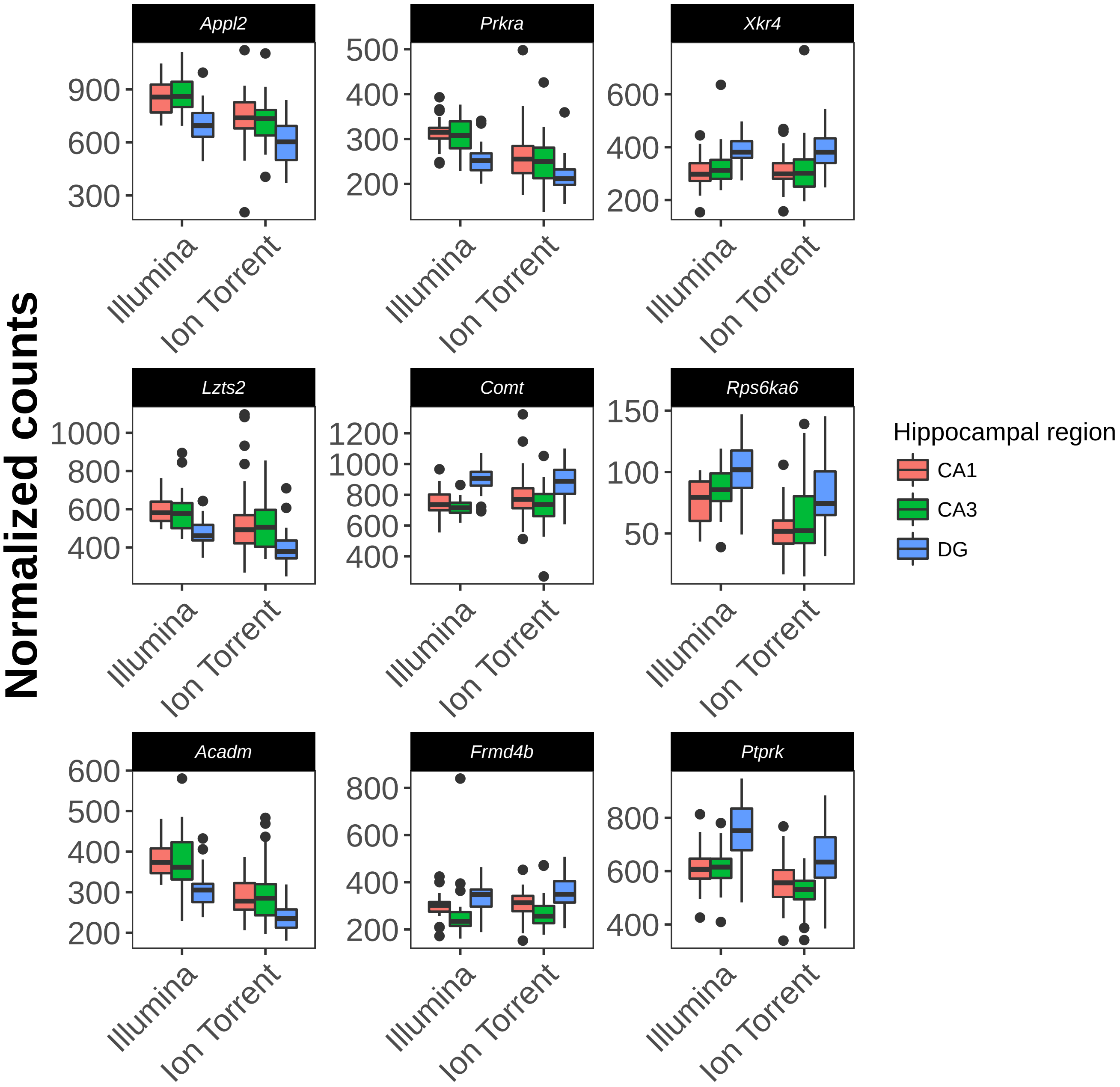
Normalized counts
Appl2
Prkra
Xkr4
Lzts2
Comt
Rps6ka6
Acadm
Frmd4b
Ptprk
Illumina
Ion Torrent
Hippocampal region
CA1
CA3
DG

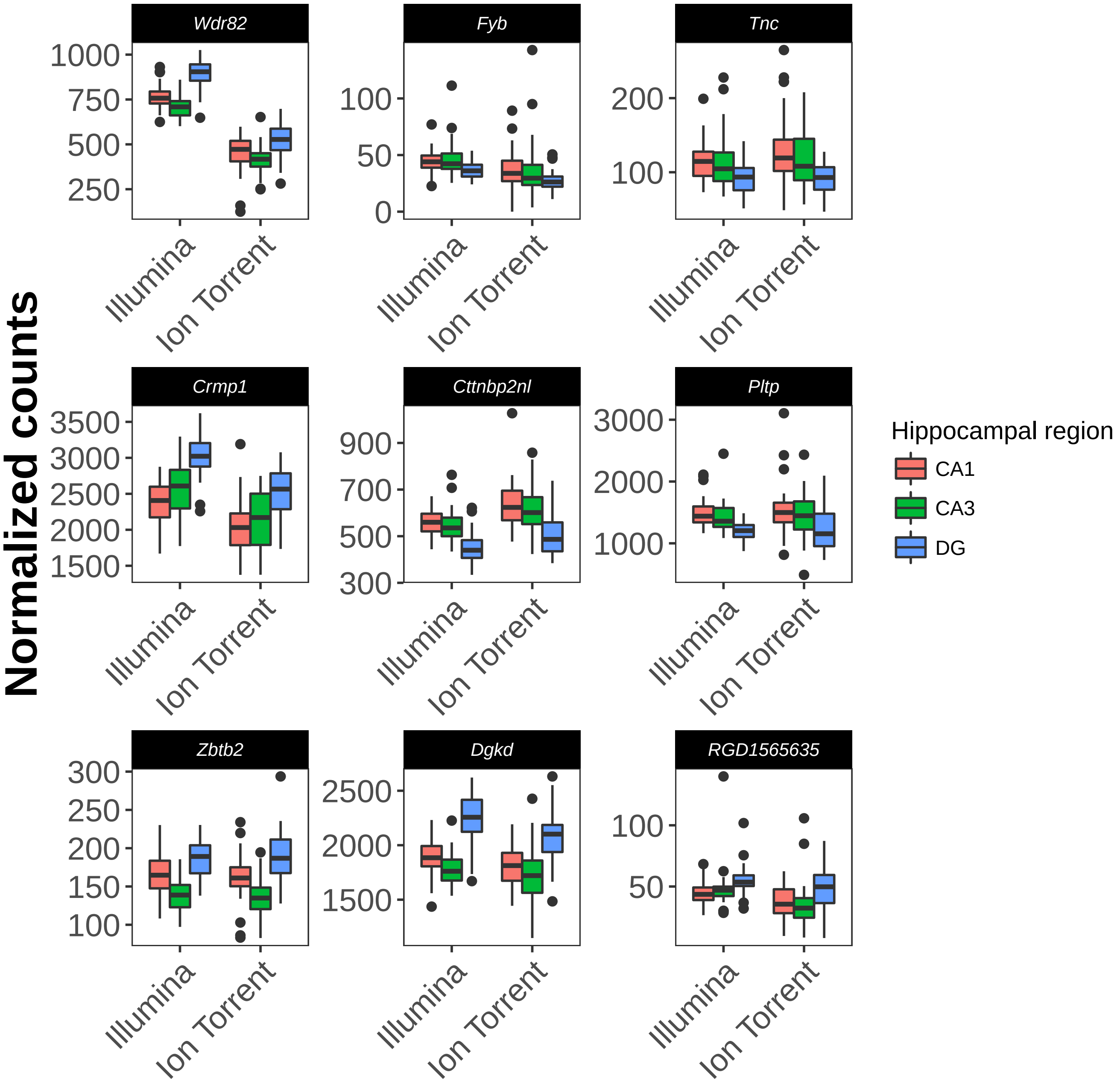
Normalized counts
Wdr82
Fyb
Tnc
Crmp1
Cttnbp2nl
Pltp
Zbtb2
Dgkd
RGD1565635
Illumina
Ion Torrent
Hippocampal region
CA1
CA3
DG

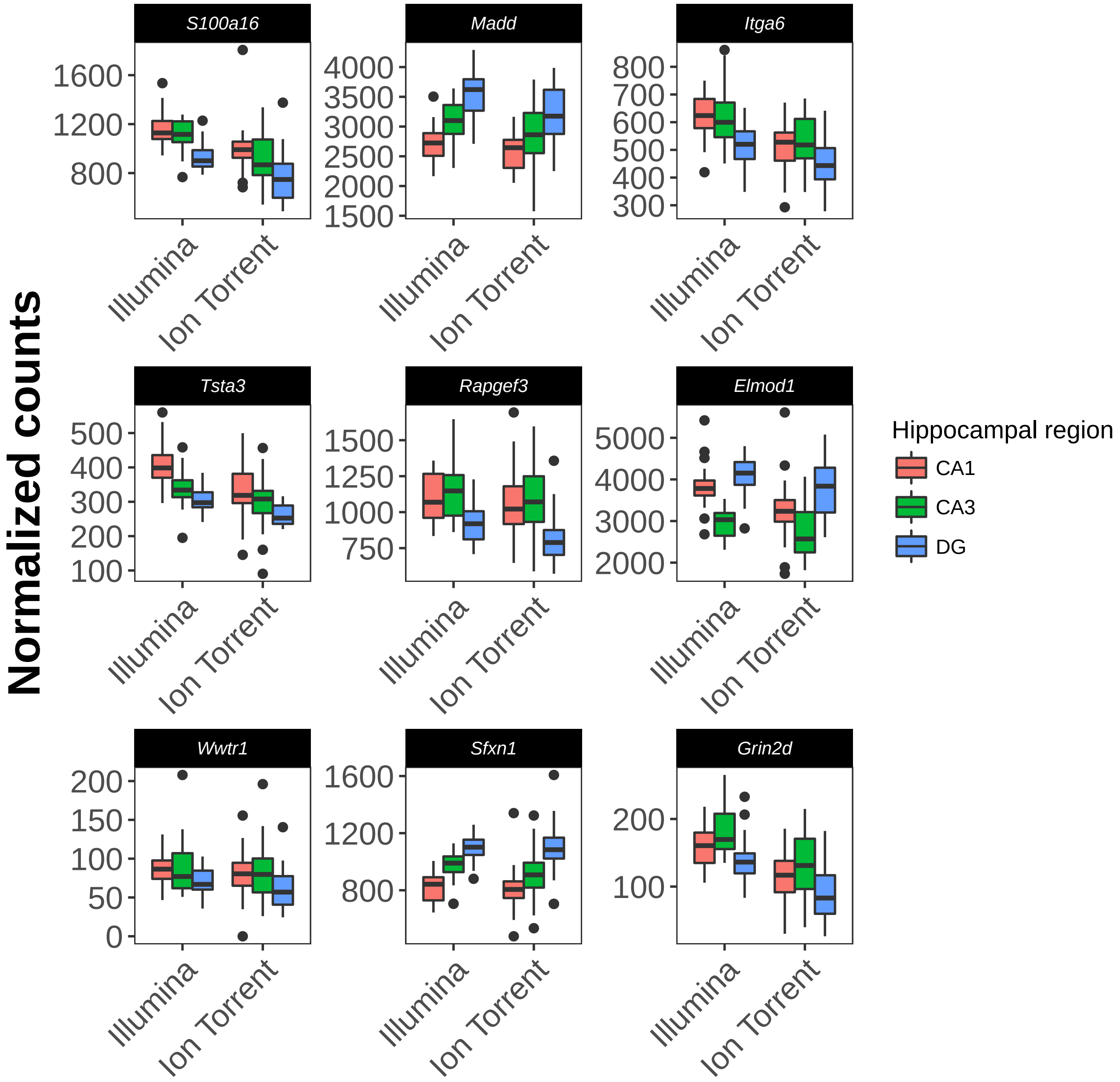

Normalized counts
S100a16
Madd
Itga6
Tsta3
Rapgef3
Elmod1
Wwtr1
Sfxn1
Grin2d
Illumina
Ion Torrent
Hippocampal region
CA1
CA3
DG

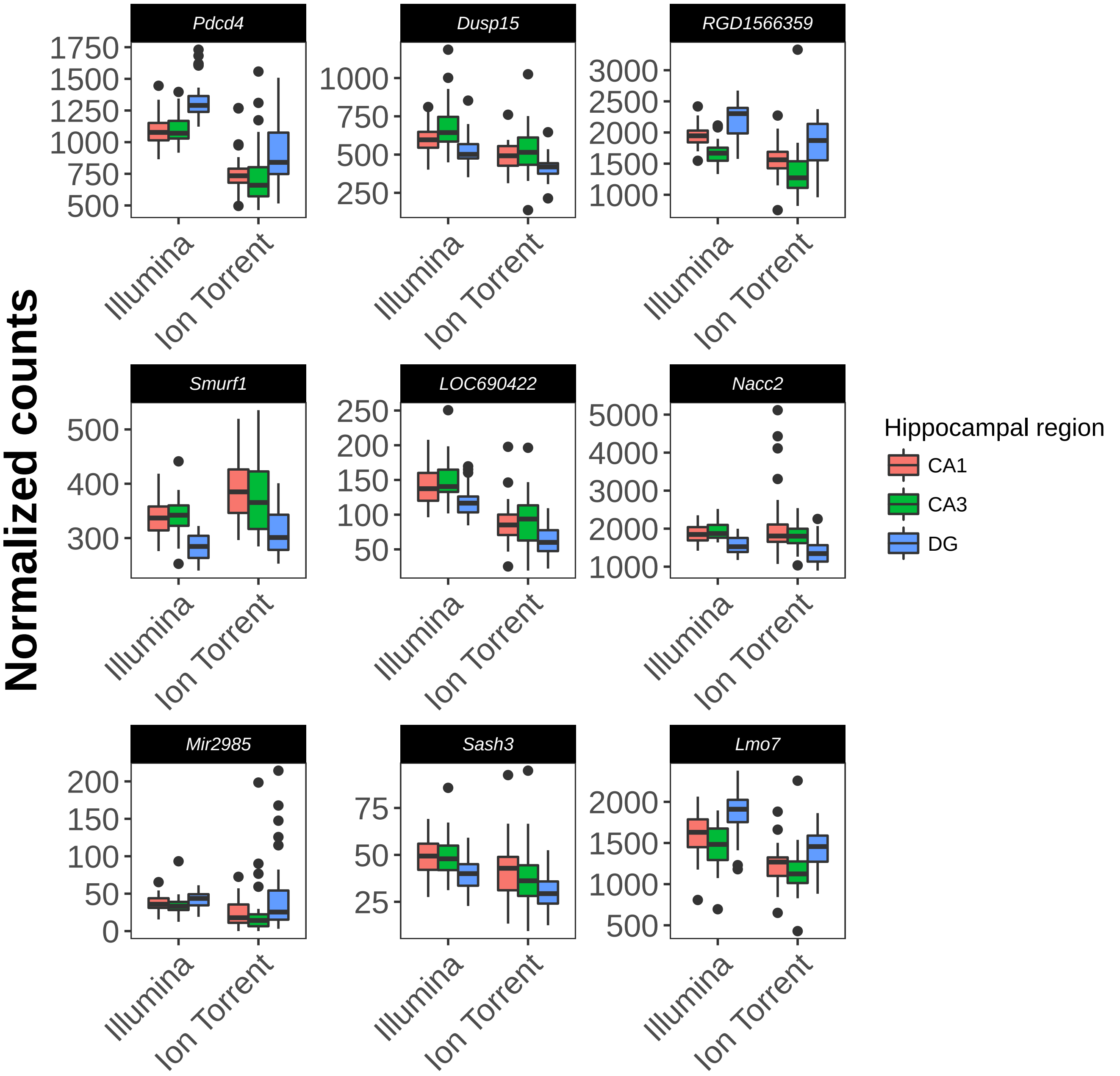

Normalized counts
Pdcd4
Dusp15
RGD1566359
Smurf1
LOC690422
Nacc2
Mir2985
Sash3
Lmo7
Illumina
Ion Torrent
Hippocampal region
CA1
CA3
DG

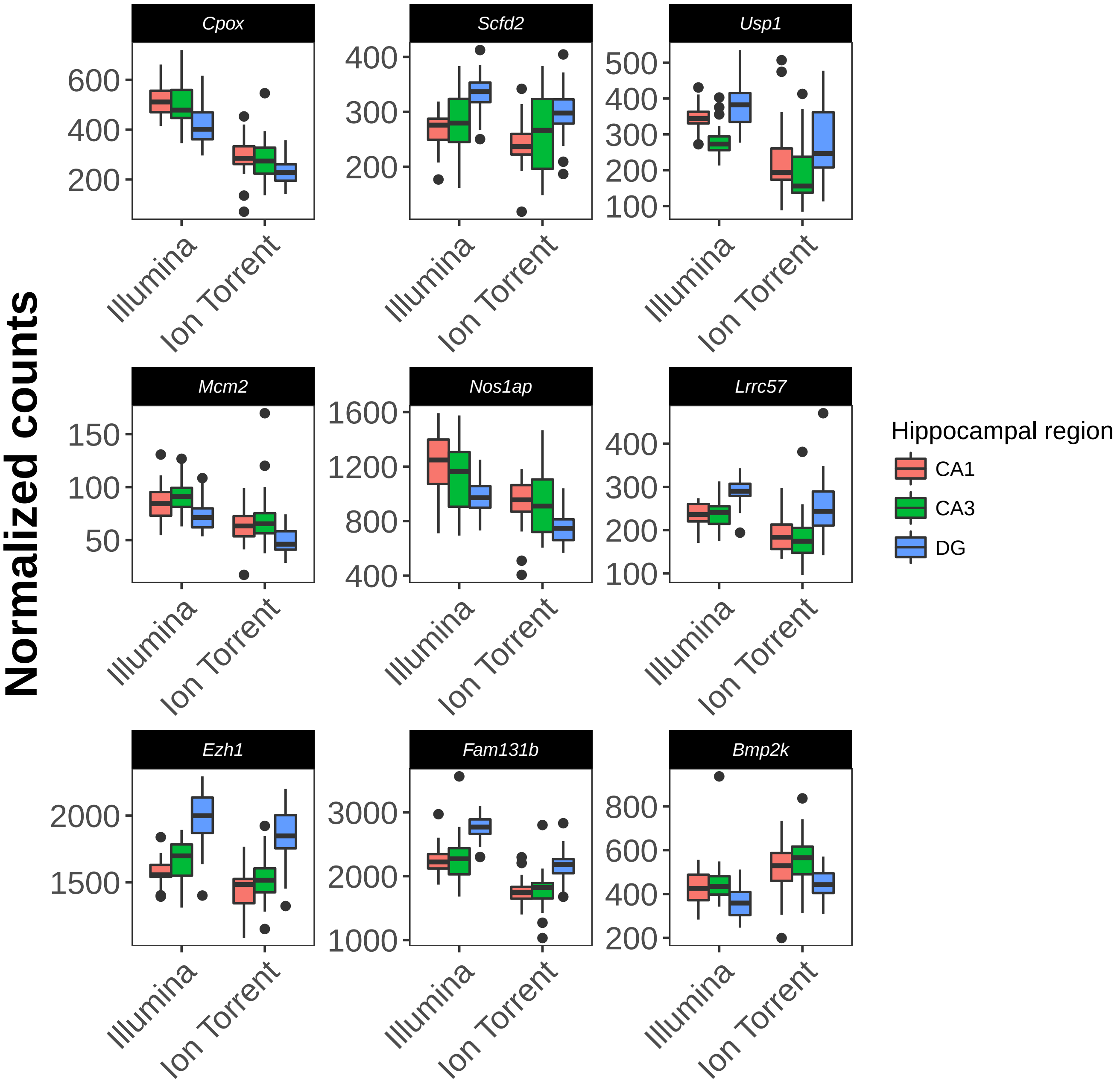

Normalized counts
Cpox
Scfd2
Usp1
Mcm2
Nos1ap
Lrrc57
Ezh1
Fam131b
Bmp2k
Illumina
Ion Torrent
Hippocampal region
CA1
CA3
DG

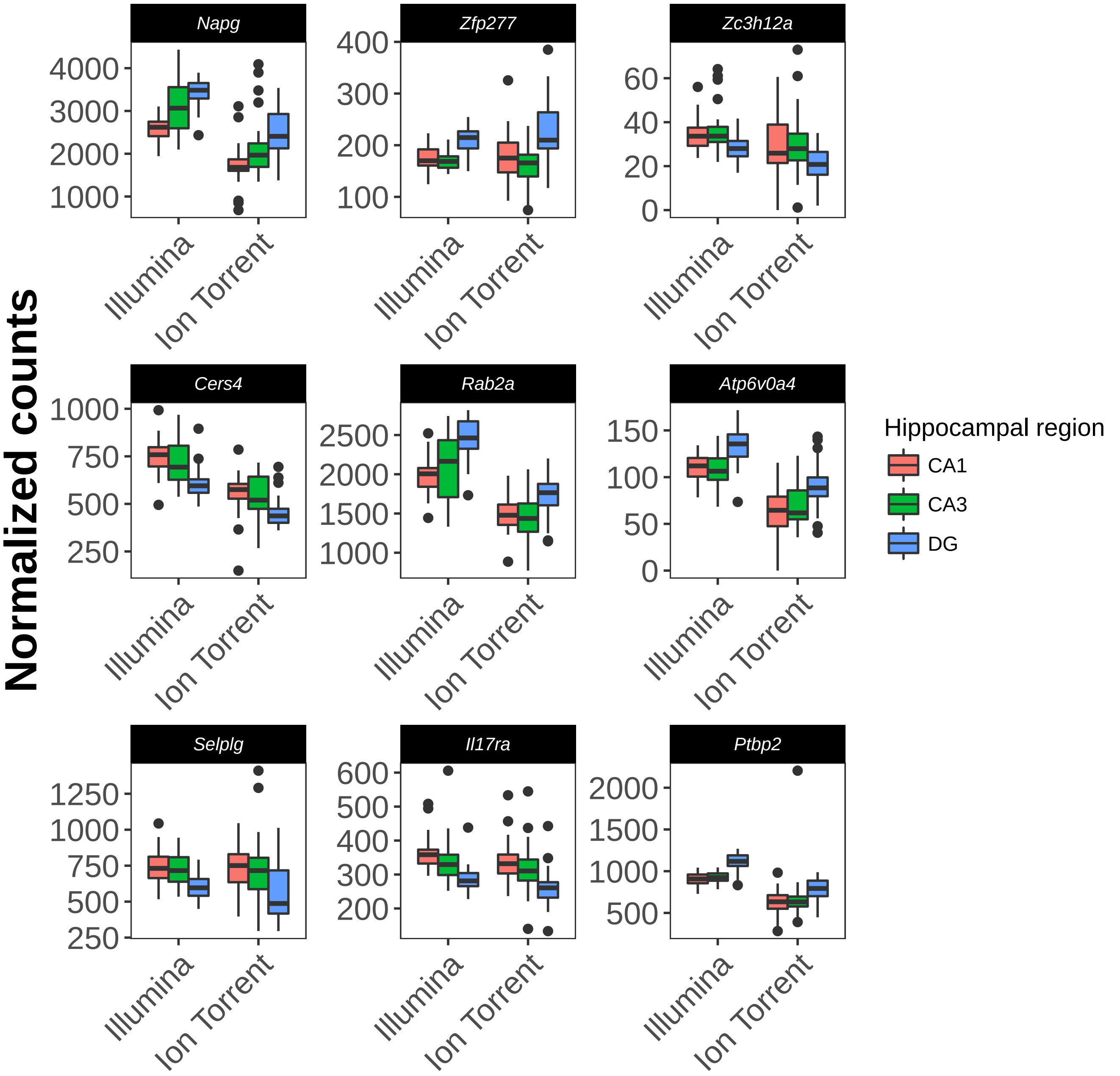

Normalized counts
Napg
Zfp277
Zc3h12a
Cers4
Rab2a
Atp6v0a4
Selplg
Il17ra
Ptbp2
Illumina
Ion Torrent
Hippocampal region
CA1
CA3
DG

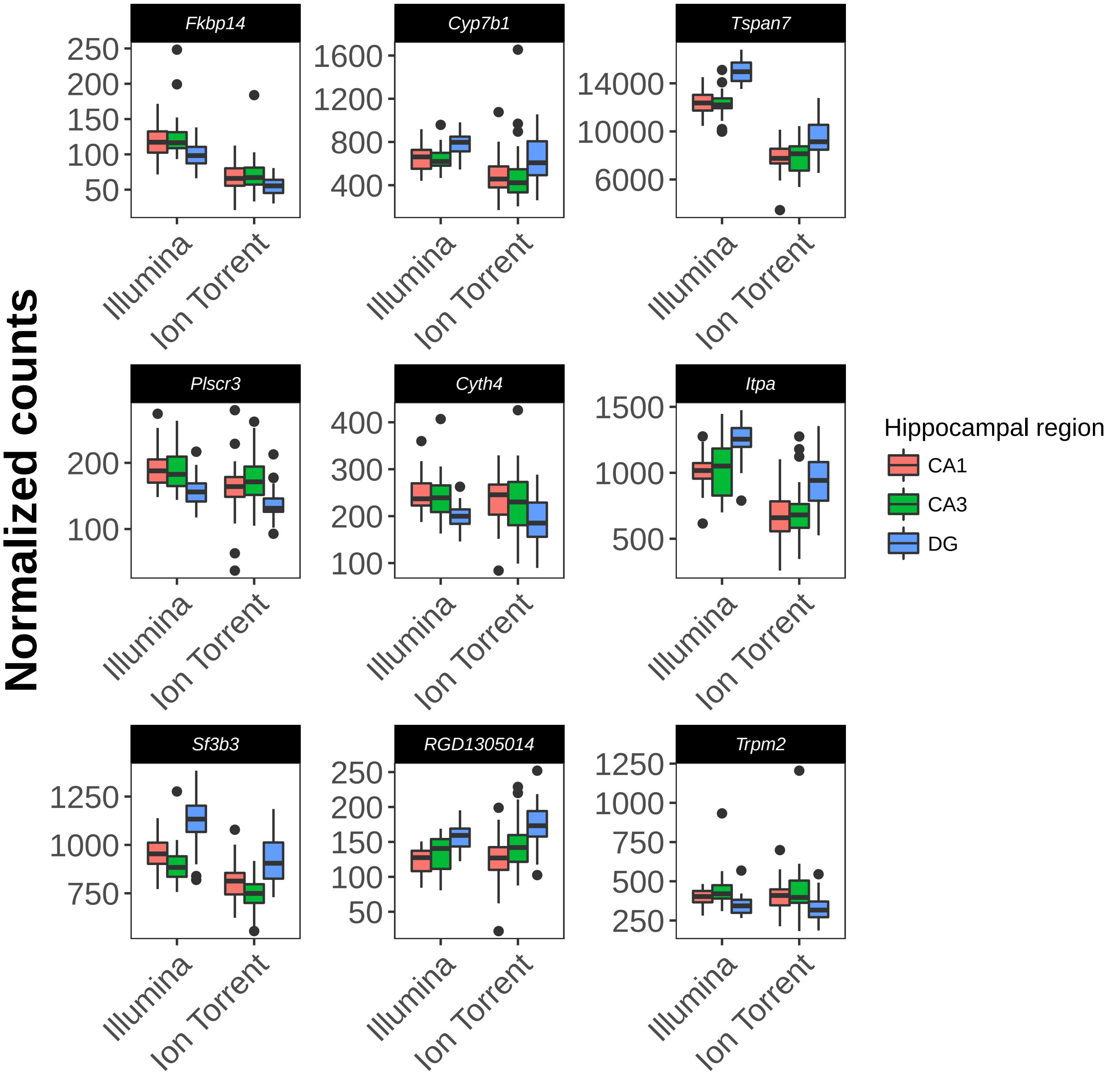
Normalized counts
Fkbp14
Cyp7b1
Tspan7
Plscr3
Cyth4
Itpa
Sf3b3
RGD1305014
Trpm2
Illumina
Ion Torrent
Hippocampal region
CA1
CA3
DG

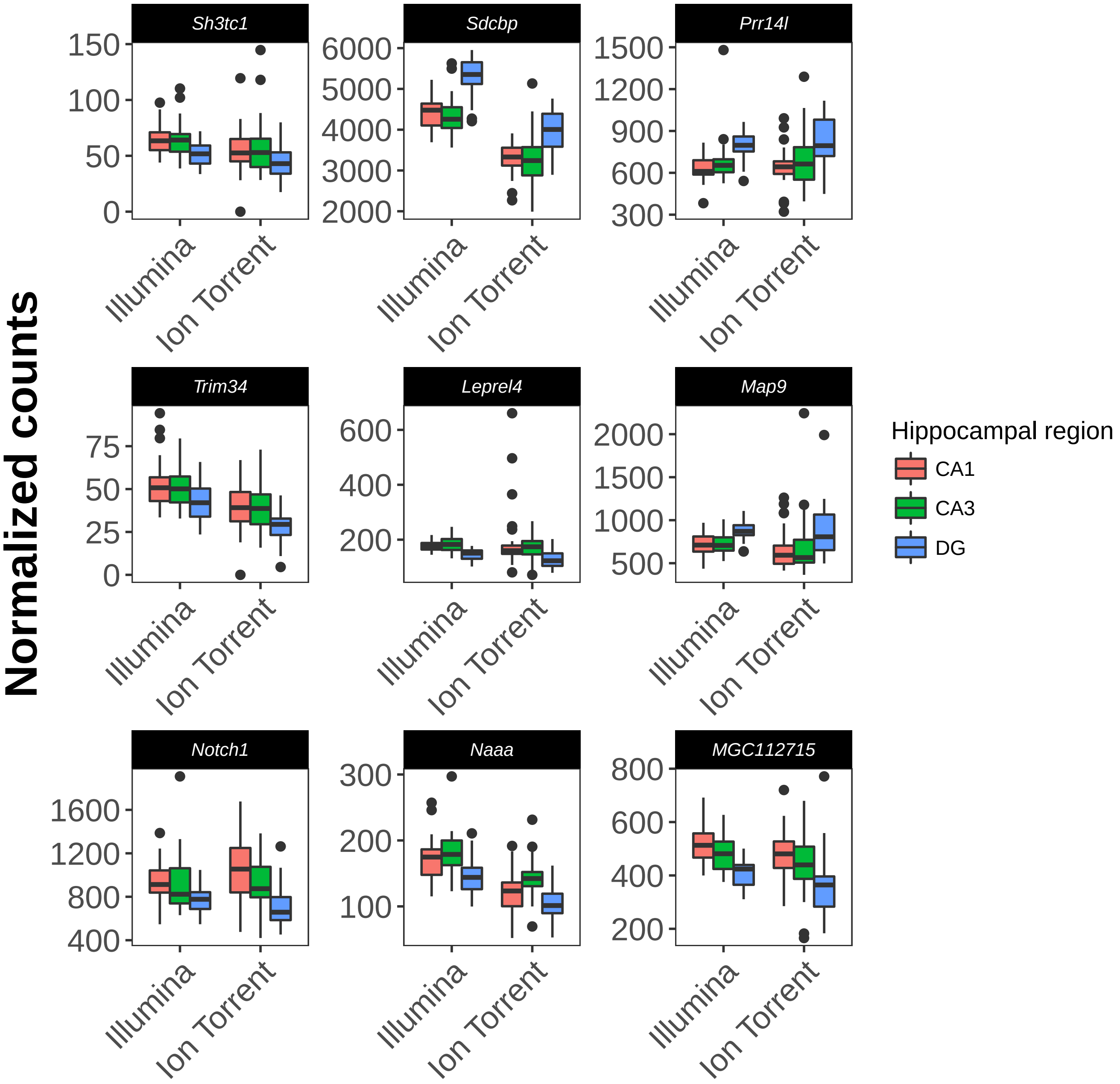

Normalized counts
Sh3tc1
Sdcbp
Prr14l
Trim34
Leprel4
Map9
Notch1
Naaa
MGC112715
Illumina
Ion Torrent
Hippocampal region
CA1
CA3
DG

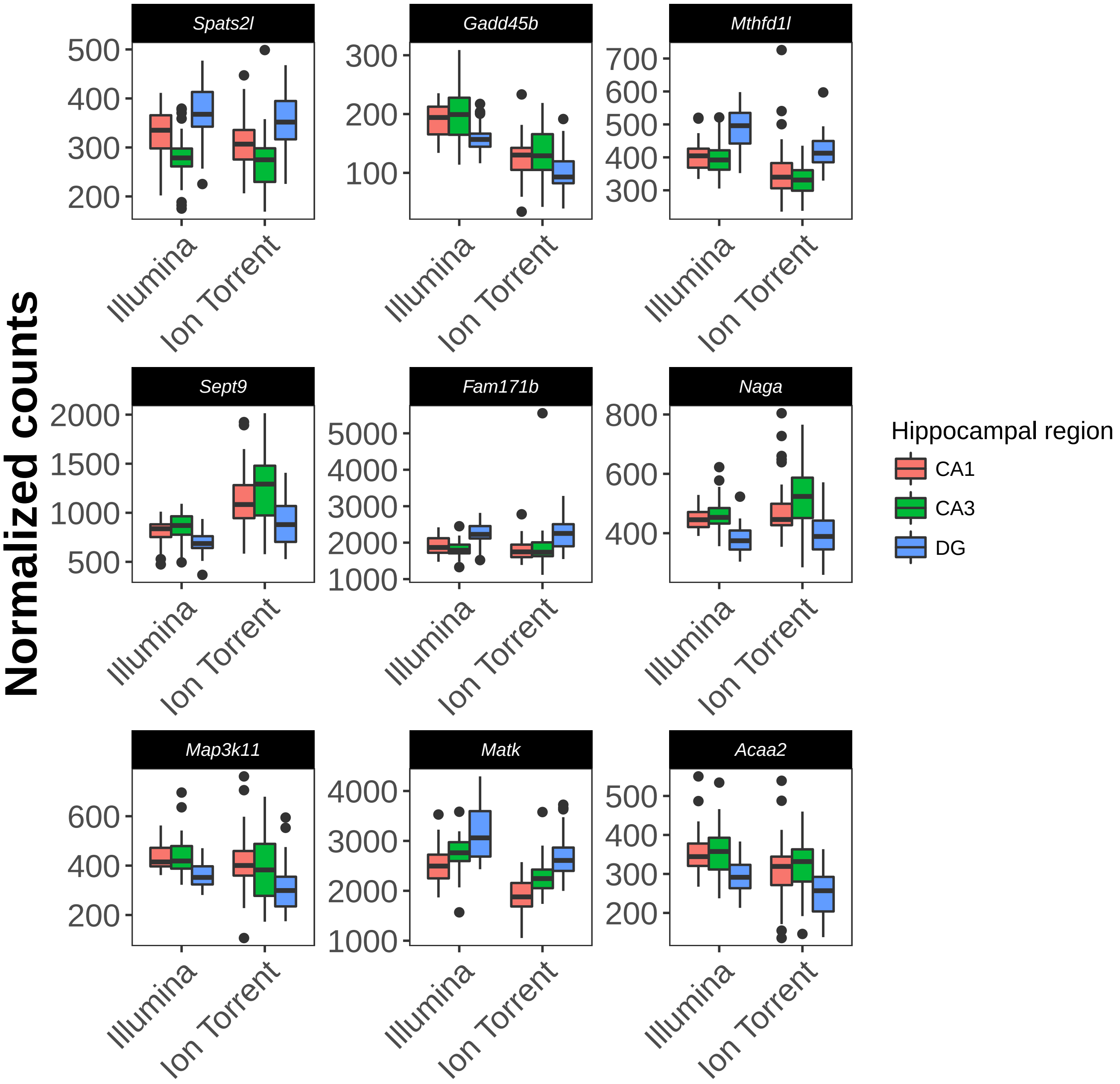
Normalized counts
Spats2l
Gadd45b
Mthfd1l
Sept9
Fam171b
Naga
Map3k11
Matk
Acaa2
Illumina
Ion Torrent
Hippocampal region
CA1
CA3
DG

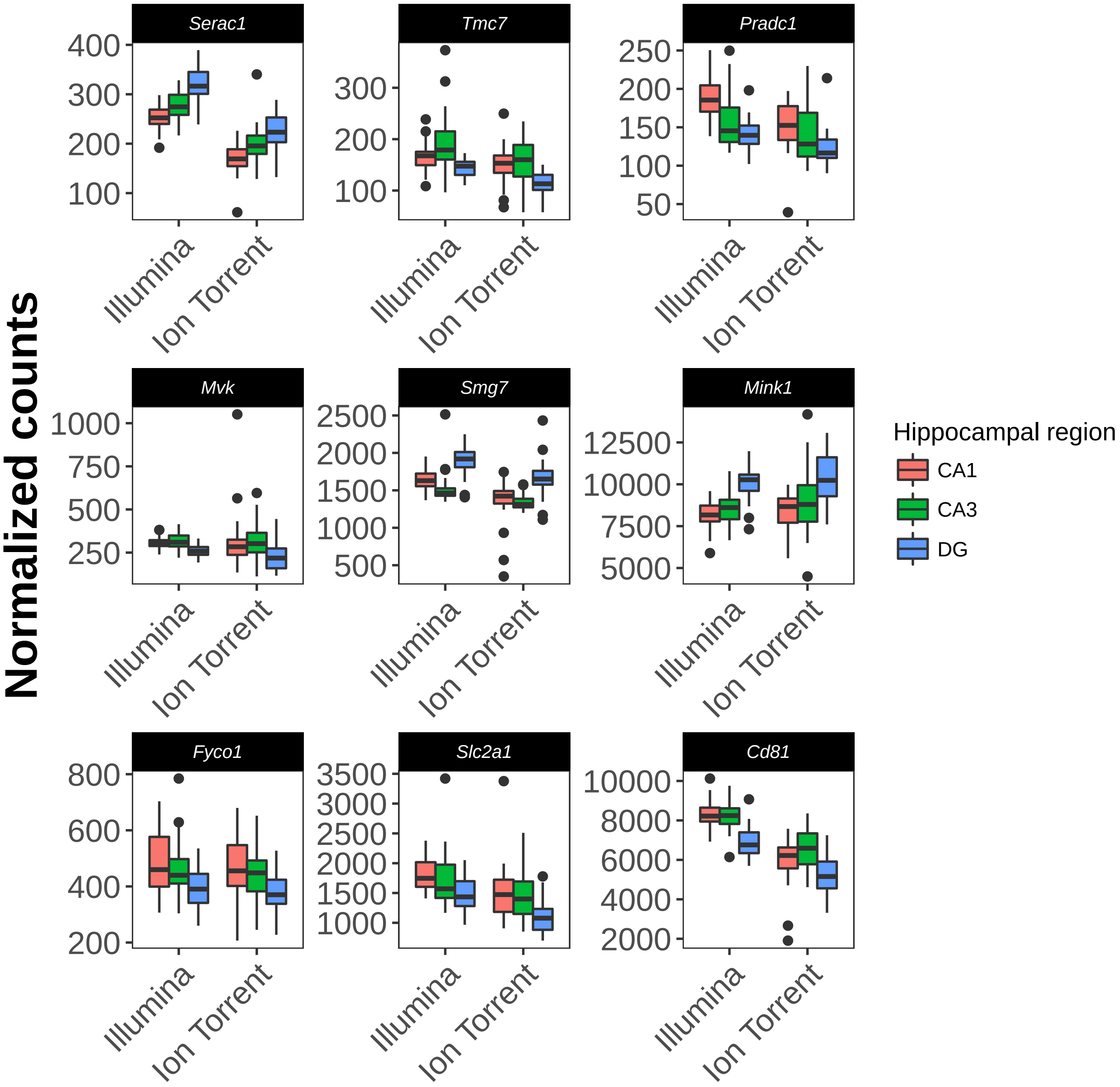
Normalized counts
Serac1
Tmc7
Pradc1
Mvk
Smg7
Mink1
Fyco1
Slc2a1
Cd81
Illumina
Ion Torrent
Hippocampal region
CA1
CA3
DG

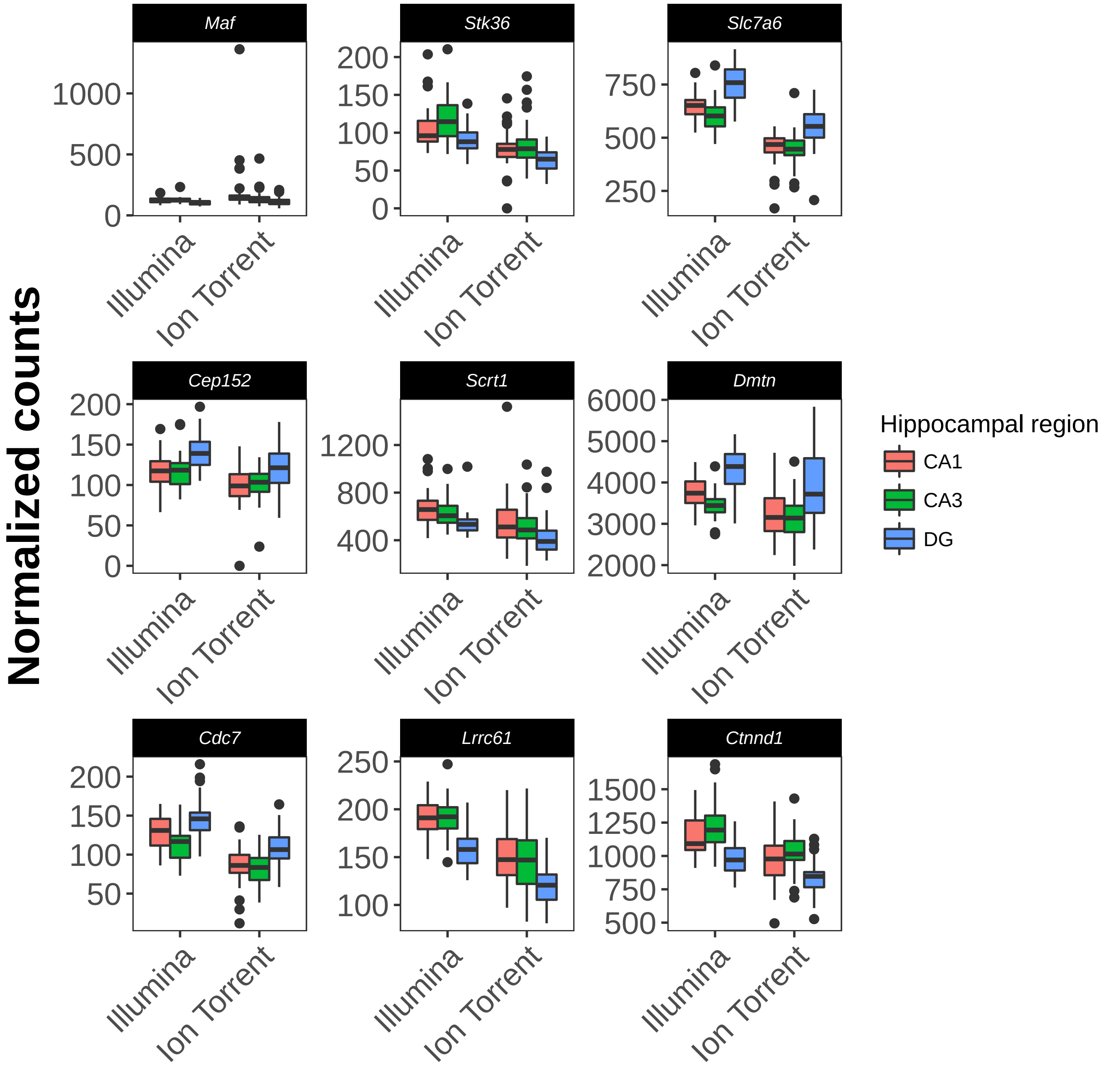

Normalized counts
Maf
Stk36
Slc7a6
Cep152
Scrt1
Dmtn
Cdc7
Lrrc61
Ctnnd1
Illumina
Ion Torrent
Hippocampal region
CA1
CA3
DG

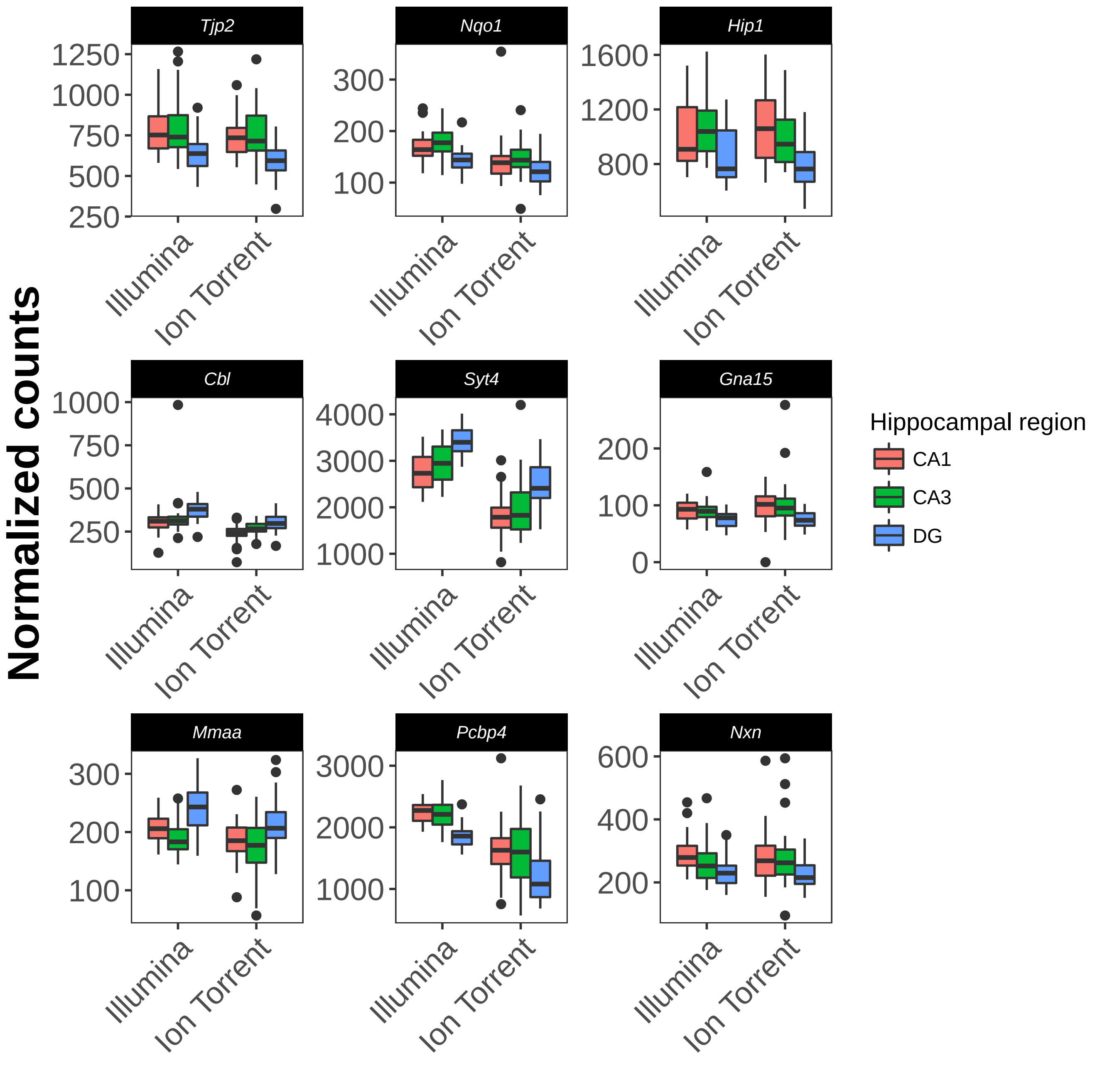
Normalized counts
Tjp2
Nqo1
Hip1
Cbl
Syt4
Gna15
Mmaa
Pcbp4
Nxn
Illumina
Ion Torrent
Hippocampal region
CA1
CA3
DG

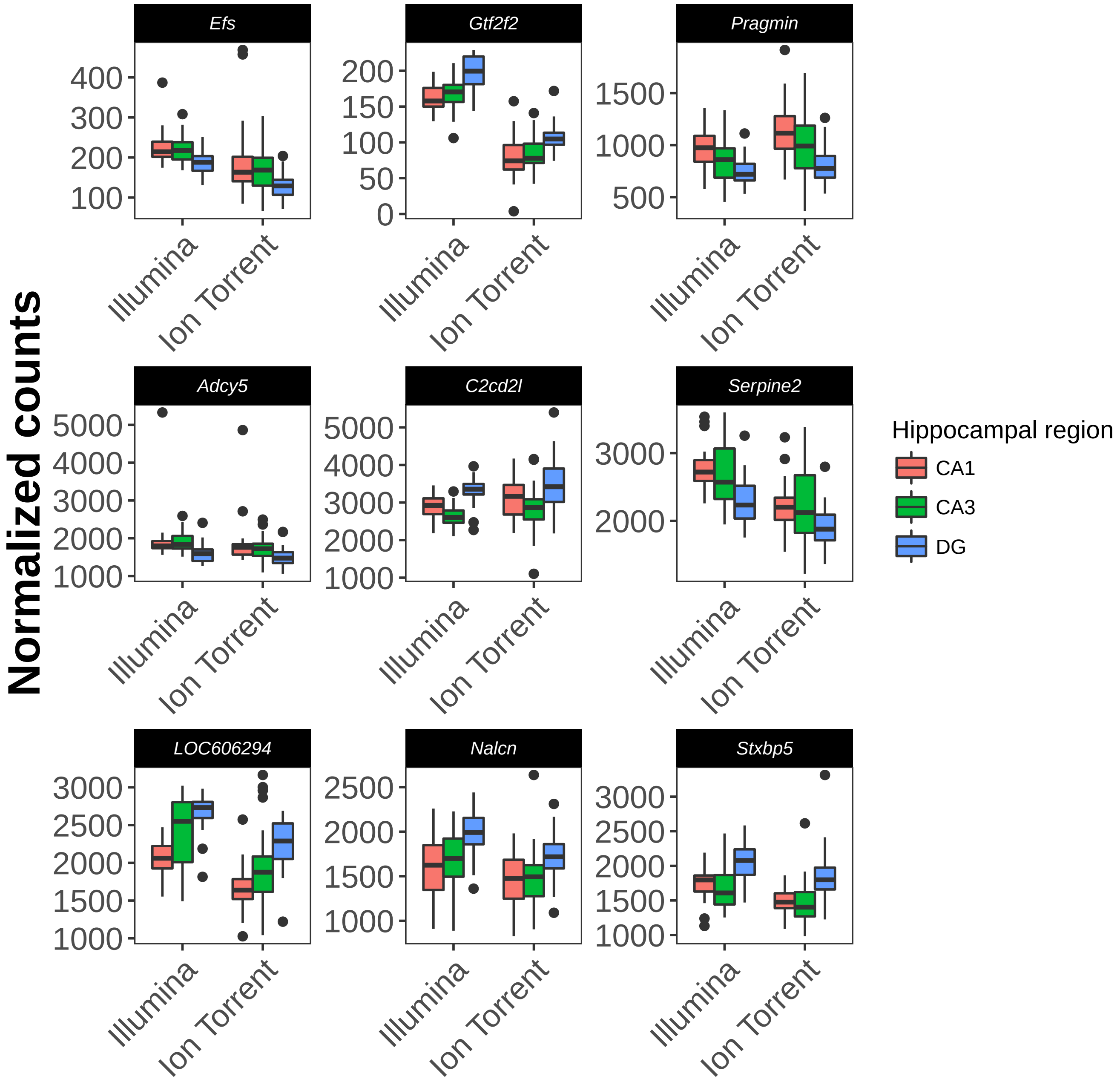

Normalized counts
Efs
Gtf2f2
Pragmin
Adcy5
C2cd2l
Serpine2
LOC606294
Nalcn
Stxbp5
Illumina
Ion Torrent
Hippocampal region
CA1
CA3
DG

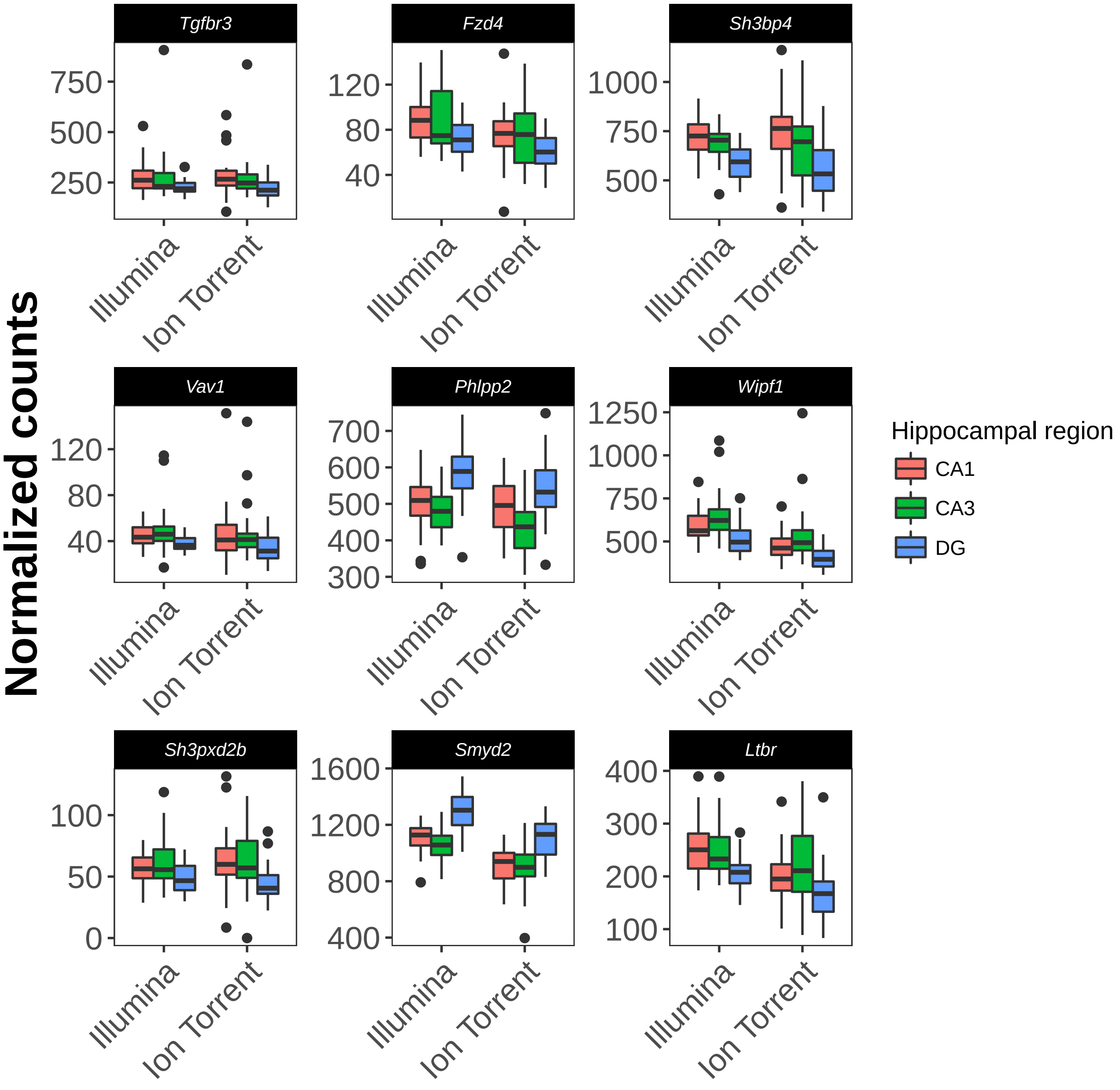

Normalized counts
Tgfbr3
Fzd4
Sh3bp4
Vav1
Phlpp2
Wipf1
Sh3pxd2b
Smyd2
Ltbr
Illumina
Ion Torrent
Hippocampal region
CA1
CA3
DG

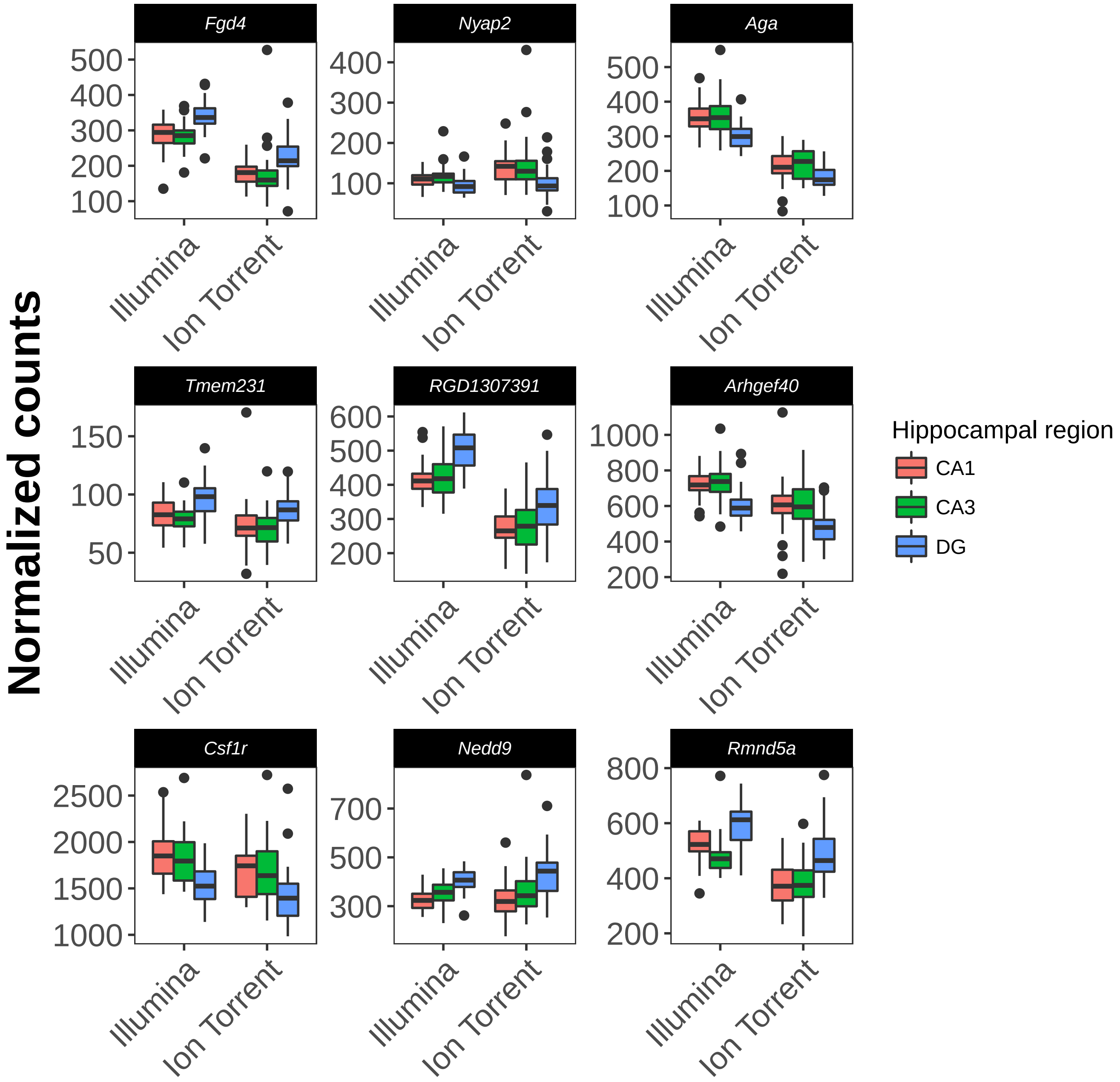

Normalized counts
Fgd4
Nyap2
Aga
Tmem231
RGD1307391
Arhgef40
Csf1r
Nedd9
Rmnd5a
Illumina
Ion Torrent
Hippocampal region
CA1
CA3
DG

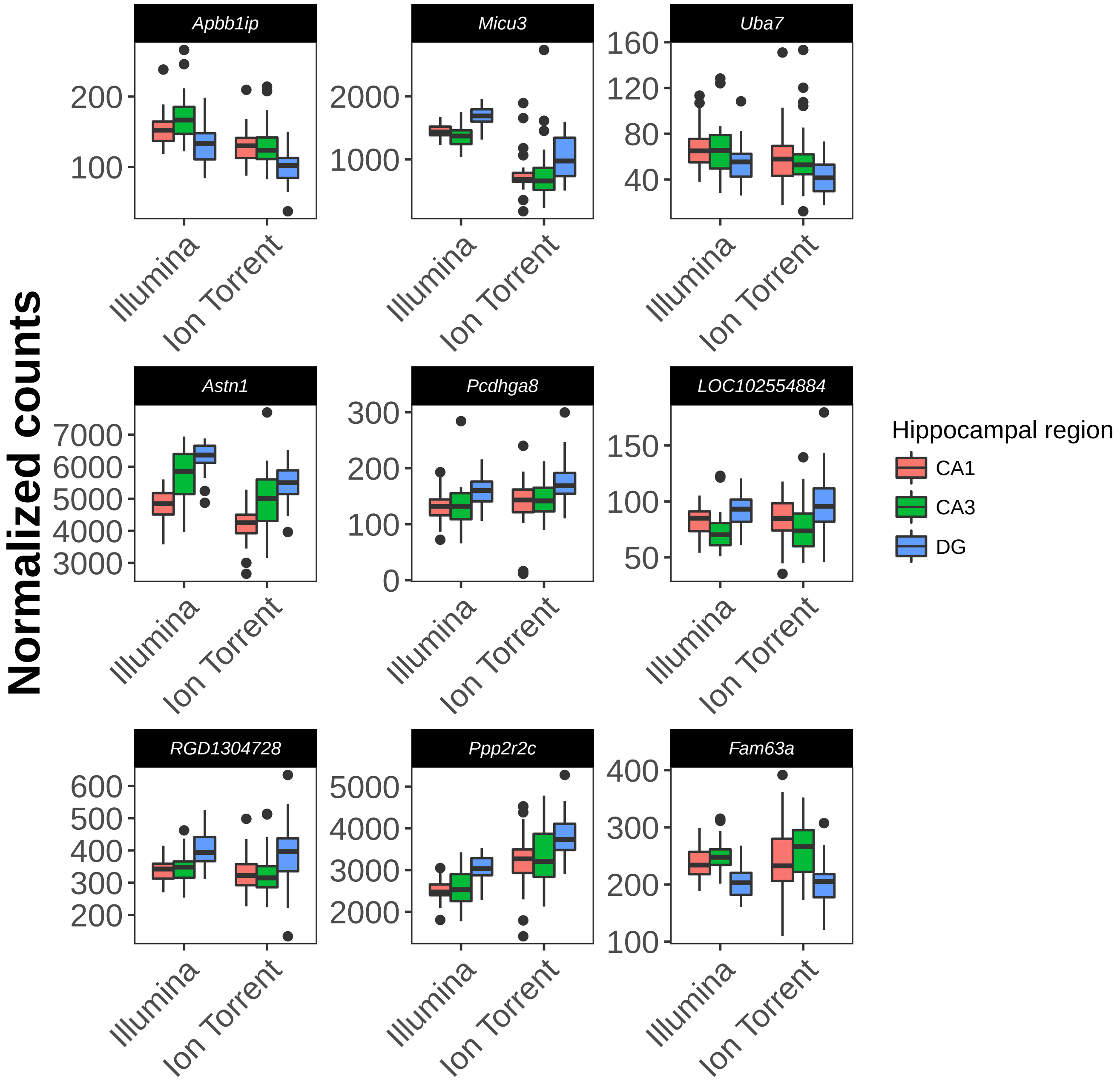

Normalized counts
Apbb1ip
Micu3
Uba7
Astn1
Pcdhga8
LOC102554884
RGD1304728
Ppp2r2c
Fam63a
Illumina
Ion Torrent
Hippocampal region
CA1
CA3
DG

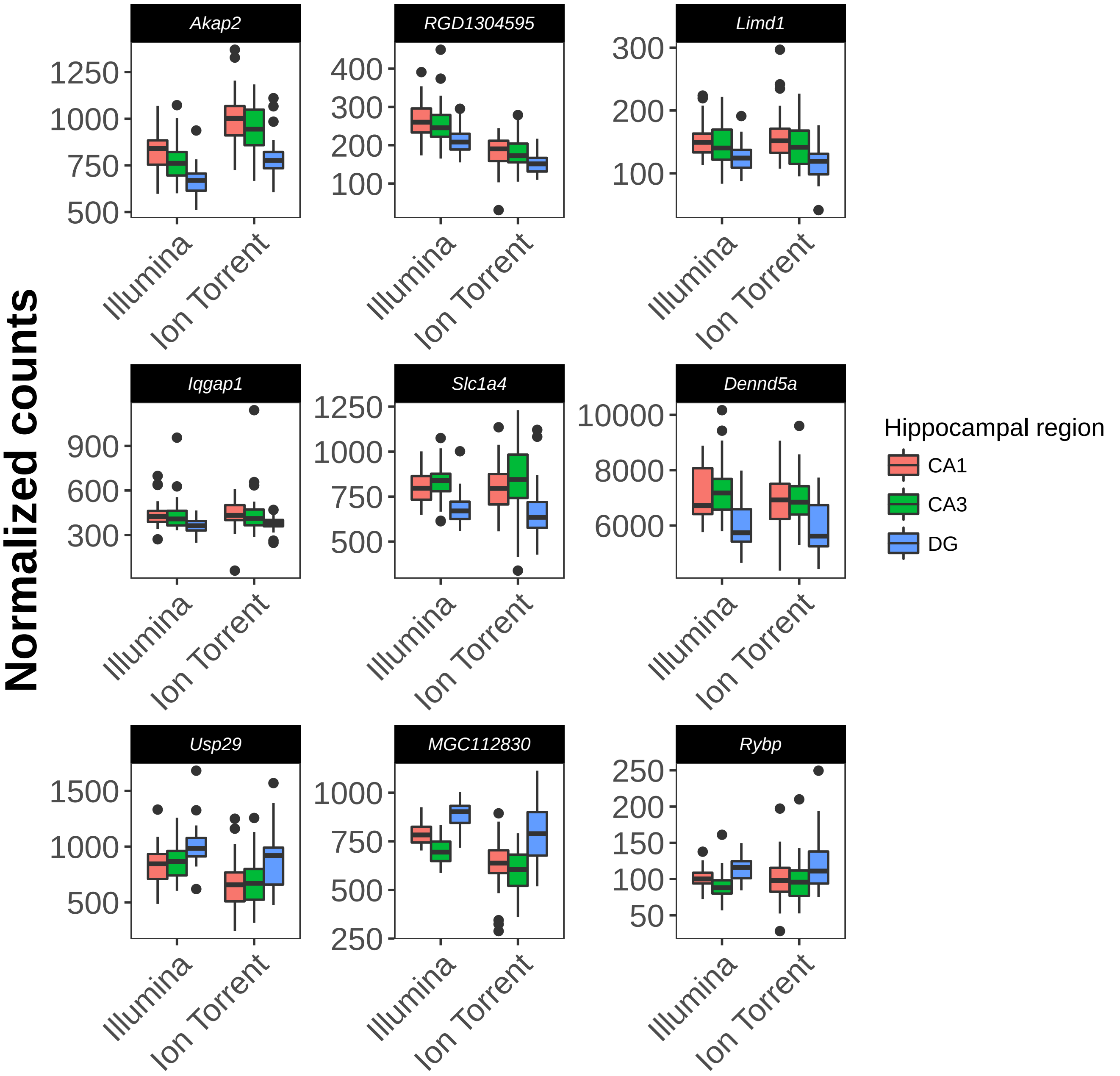

Normalized counts
Akap2
RGD1304595
Limd1
Iqgap1
Slc1a4
Dennd5a
Usp29
MGC112830
Rybp
Illumina
Ion Torrent
Hippocampal region
CA1
CA3
DG

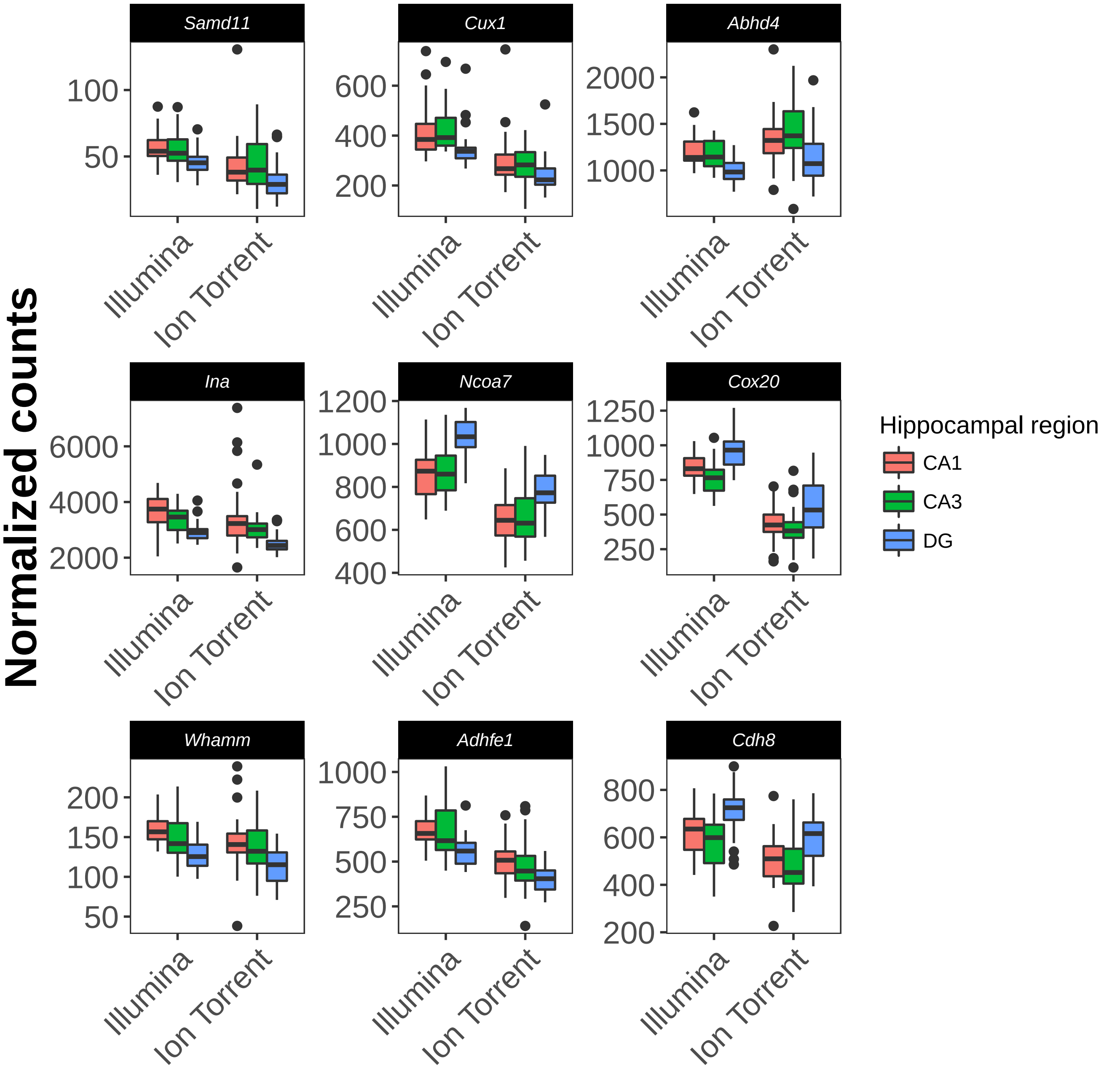
Normalized counts
Samd11
Cux1
Abhd4
Ina
Ncoa7
Cox20
Whamm
Adhfe1
Cdh8
Illumina
Ion Torrent
Hippocampal region
CA1
CA3
DG

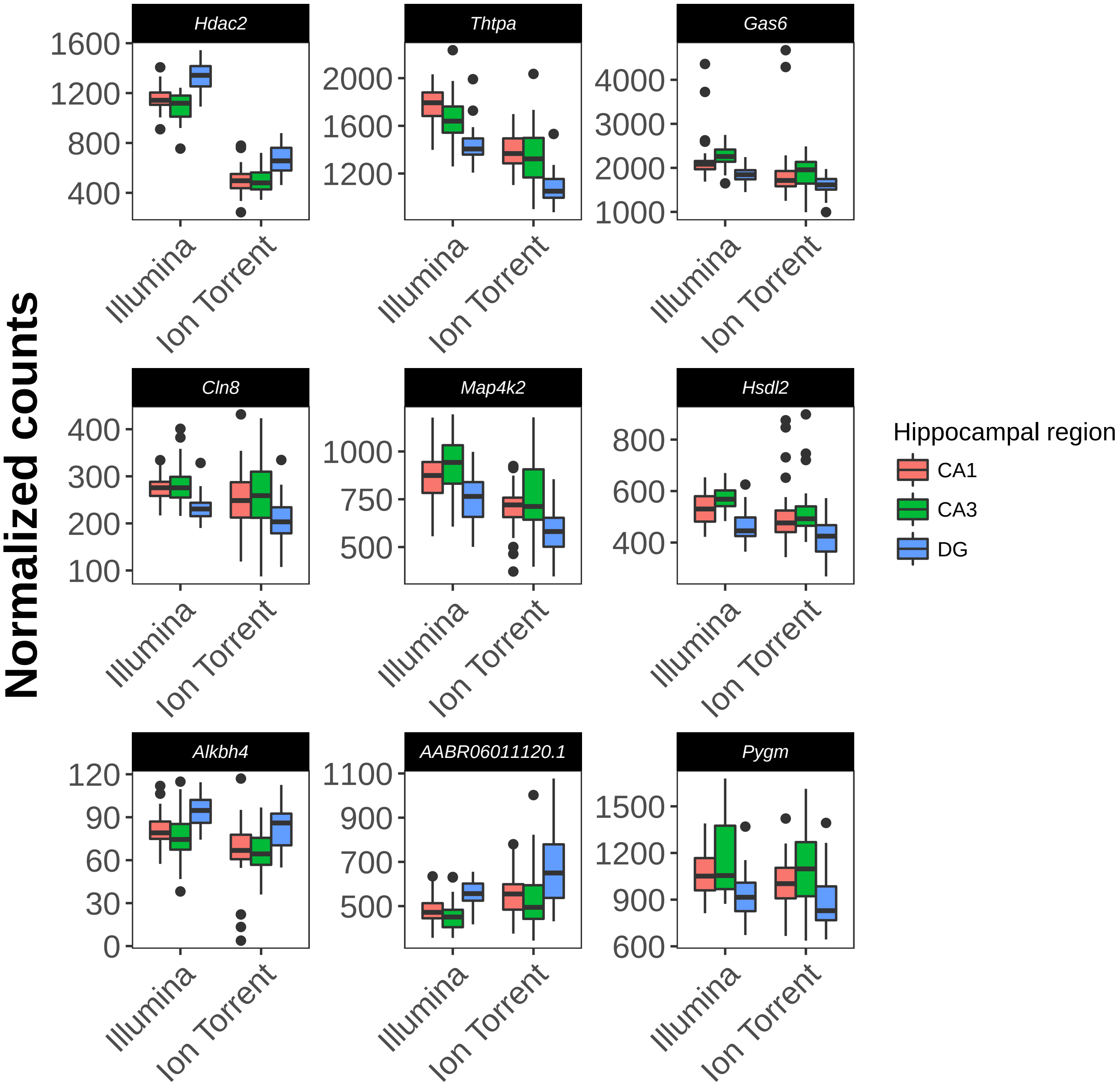

Normalized counts
Hdac2
Thtpa
Gas6
Cln8
Map4k2
Hsdl2
Alkbh4
AABR06011120.1
Pygm
Illumina
Ion Torrent
Hippocampal region
CA1
CA3
DG

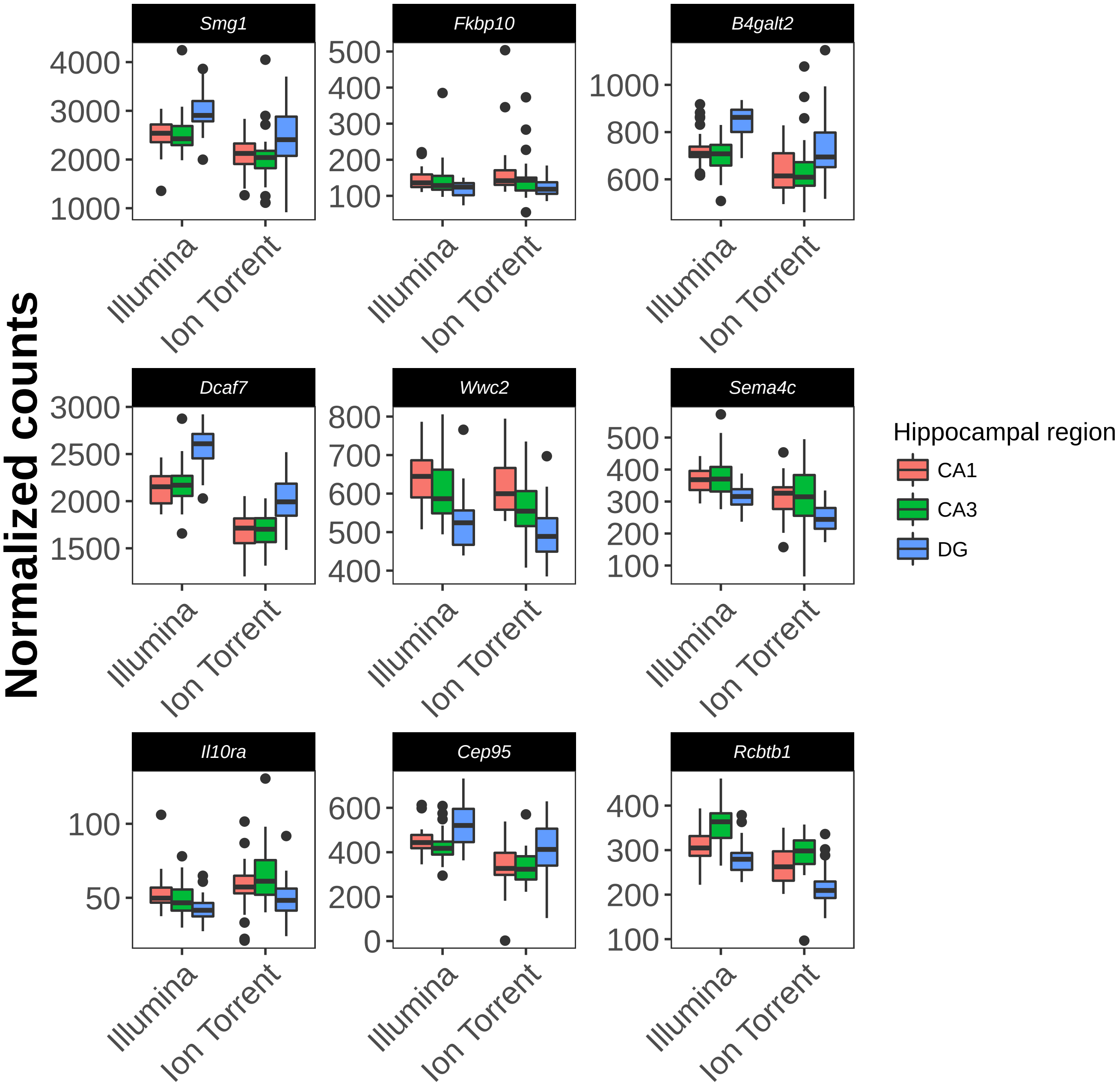
Smg1
Fkbp10
B4galt2
Dcaf7
Wwc2
Sema4c
Il10ra
Cep95
Rcbtb1
Normalized counts
Illumina
Ion Torrent
Hippocampal region
CA1
CA3
DG

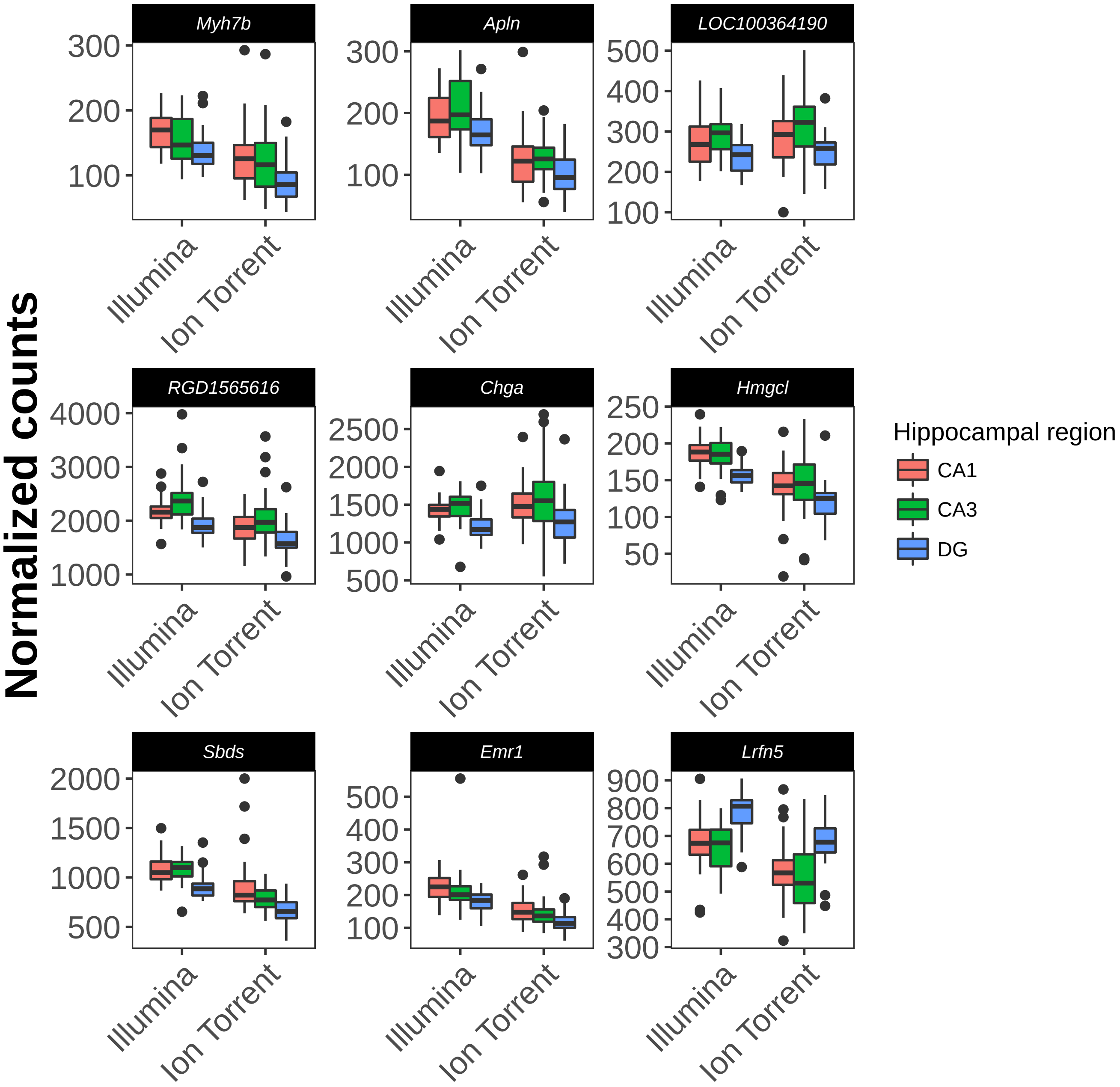

Normalized counts
Myh7b
Apln
LOC100364190
RGD1565616
Chga
Hmgcl
Sbds
Emr1
Lrfn5
Illumina
Ion Torrent
Hippocampal region
CA1
CA3
DG

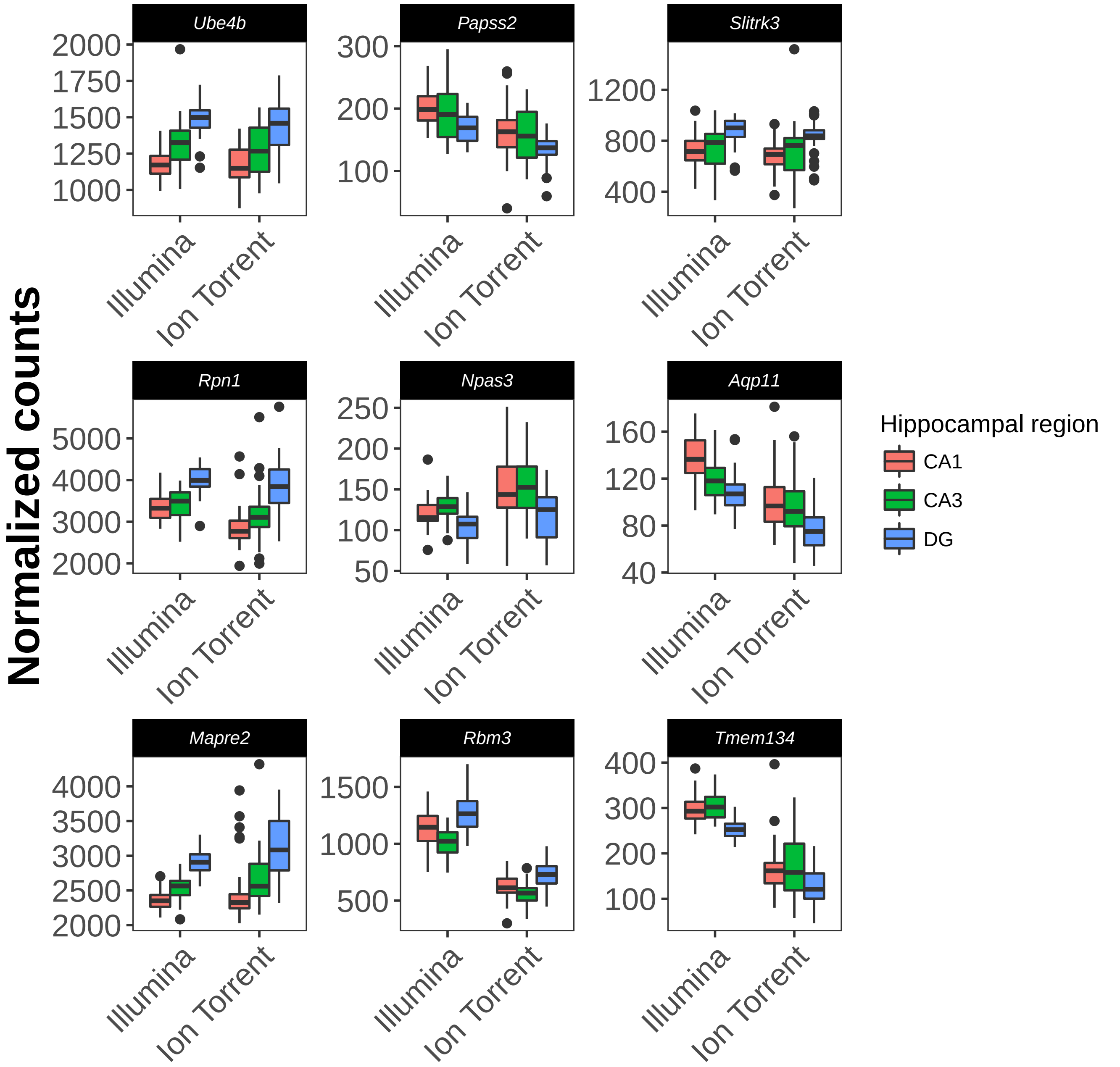
Normalized counts
Ube4b
Papss2
Slitrk3
Rpn1
Npas3
Aqp11
Mapre2
Rbm3
Tmem134
Illumina
Ion Torrent
Hippocampal region
CA1
CA3
DG

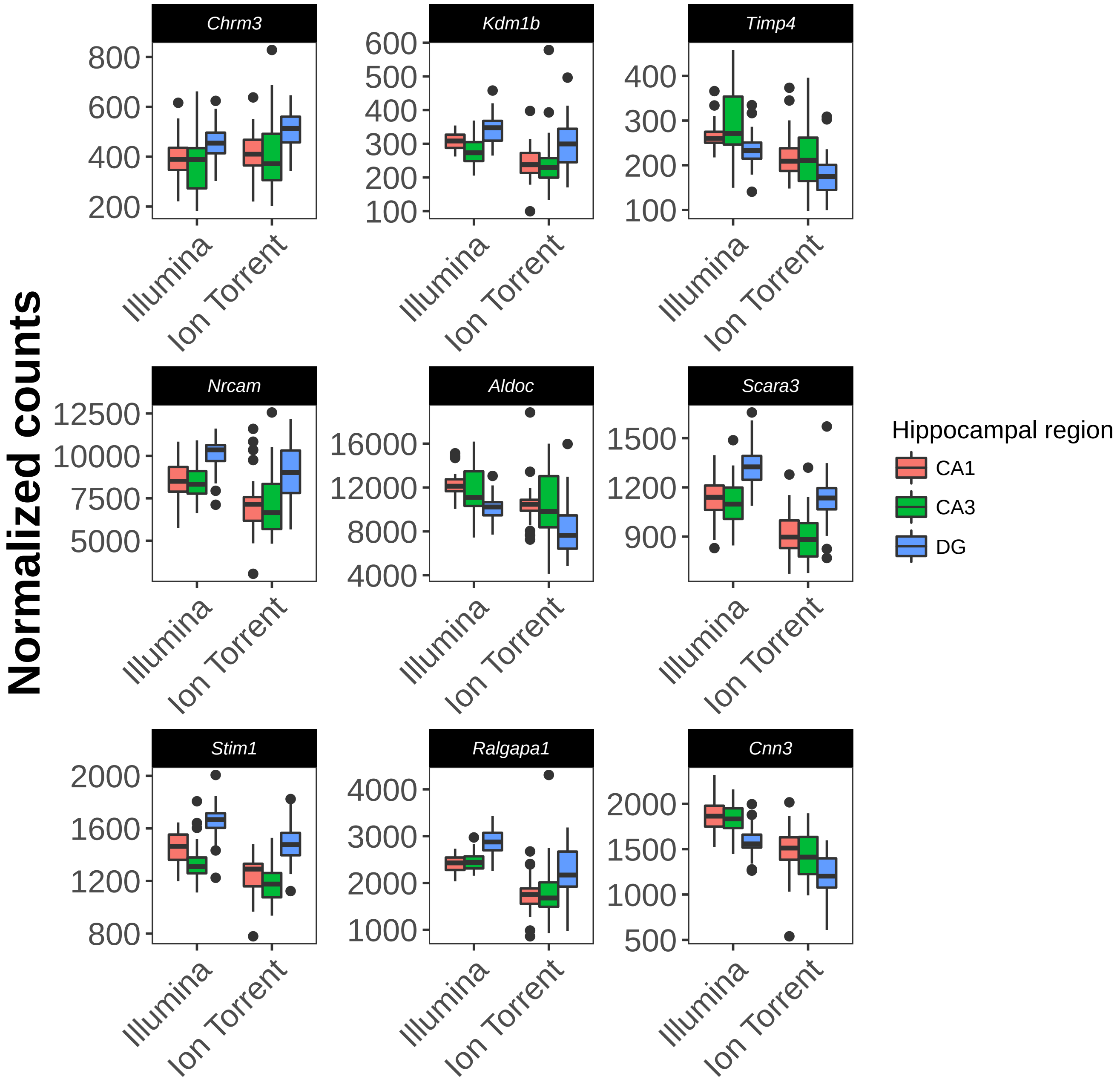
Normalized counts
Chrm3
Kdm1b
Timp4
Nrcam
Aldoc
Scara3
Stim1
Ralgapa1
Cnn3
Illumina
Ion Torrent
Hippocampal region
CA1
CA3
DG

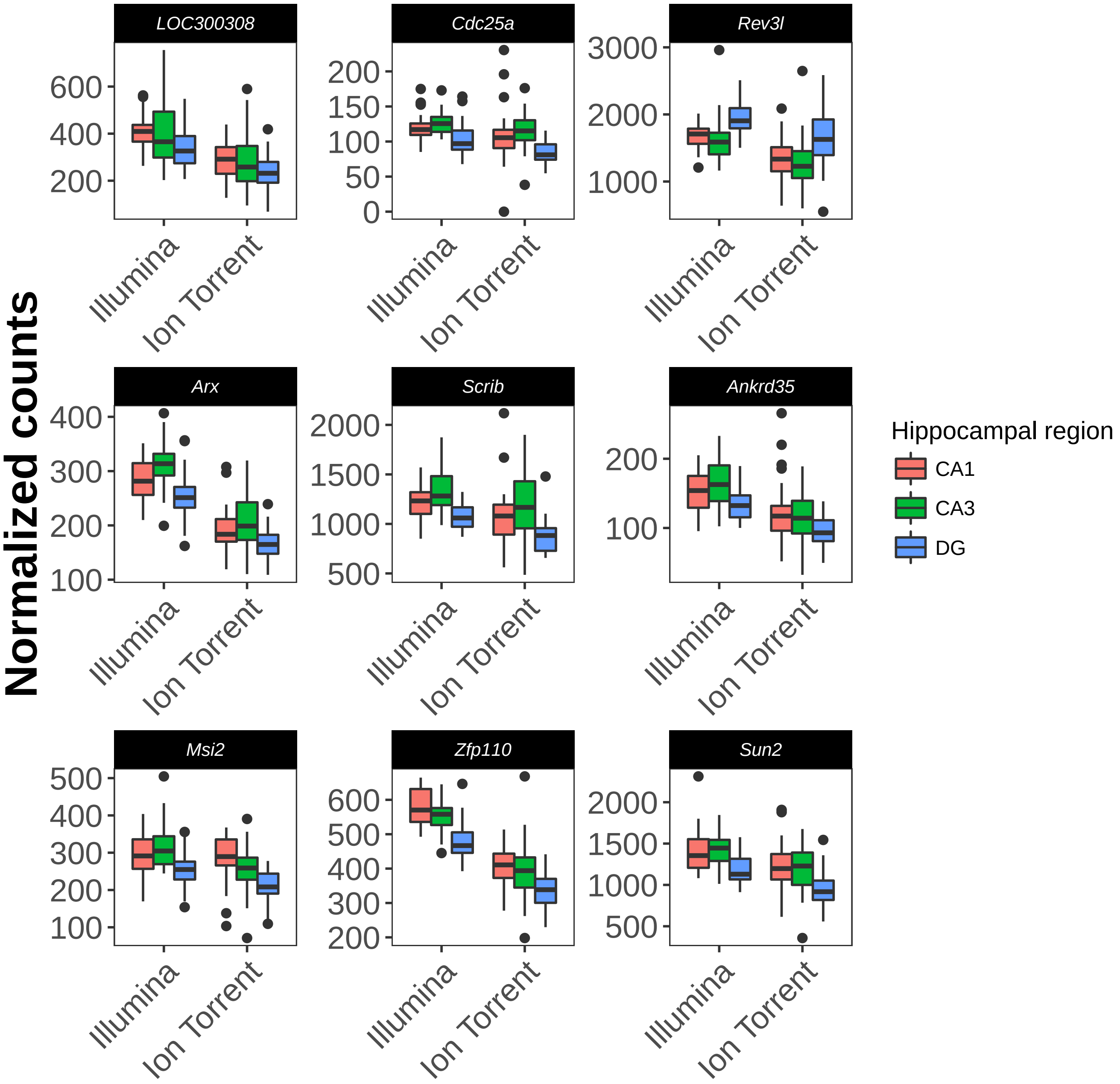

Normalized counts
LOC300308
Cdc25a
Rev3l
Arx
Scrib
Ankrd35
Msi2
Zfp110
Sun2
Illumina
Ion Torrent
Hippocampal region
CA1
CA3
DG

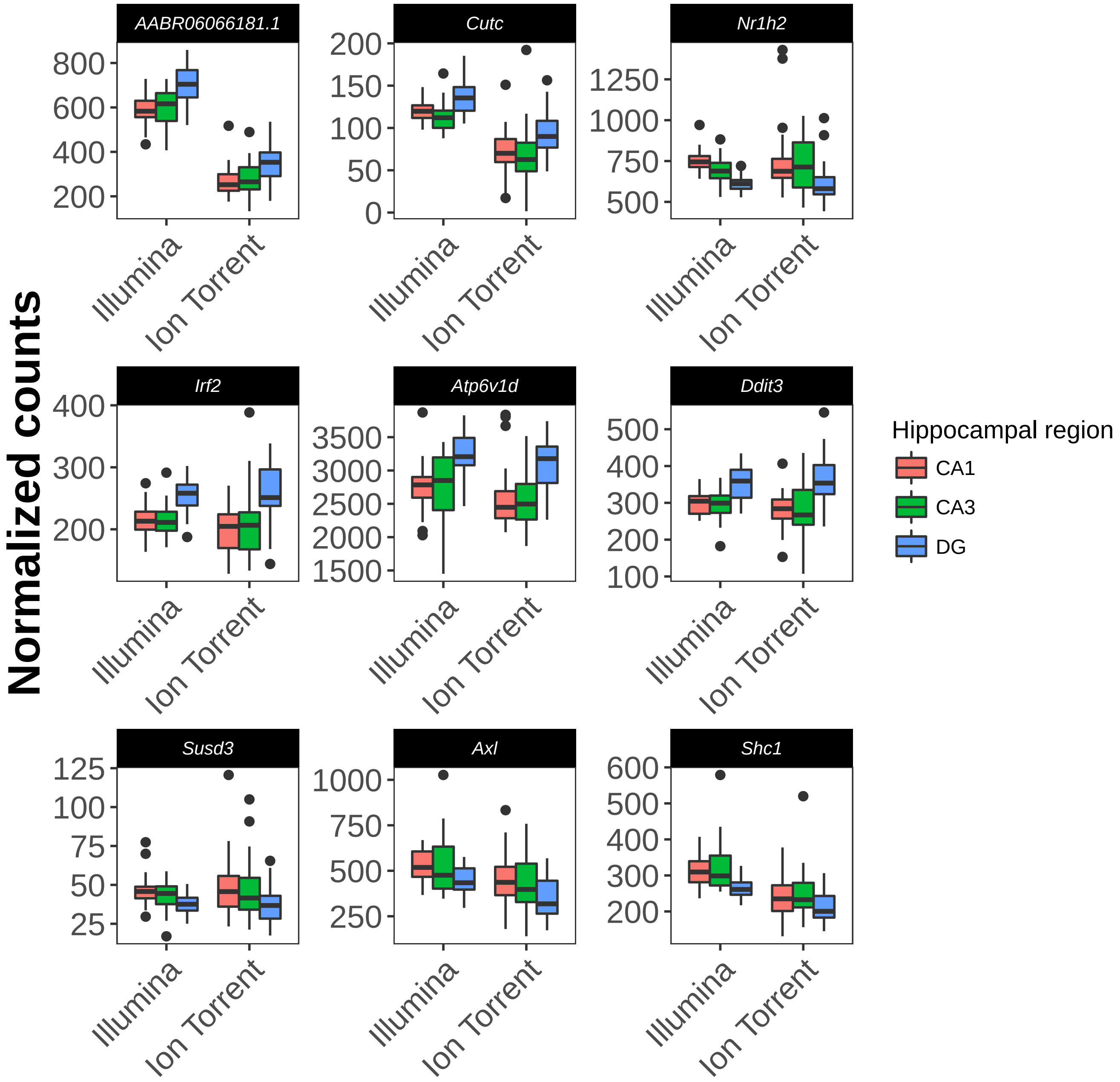

Normalized counts
AABR06066181.1
Cutc
Nr1h2
Irf2
Atp6v1d
Ddit3
Susd3
Axl
Shc1
Illumina
Ion Torrent
Hippocampal region
CA1
CA3
DG

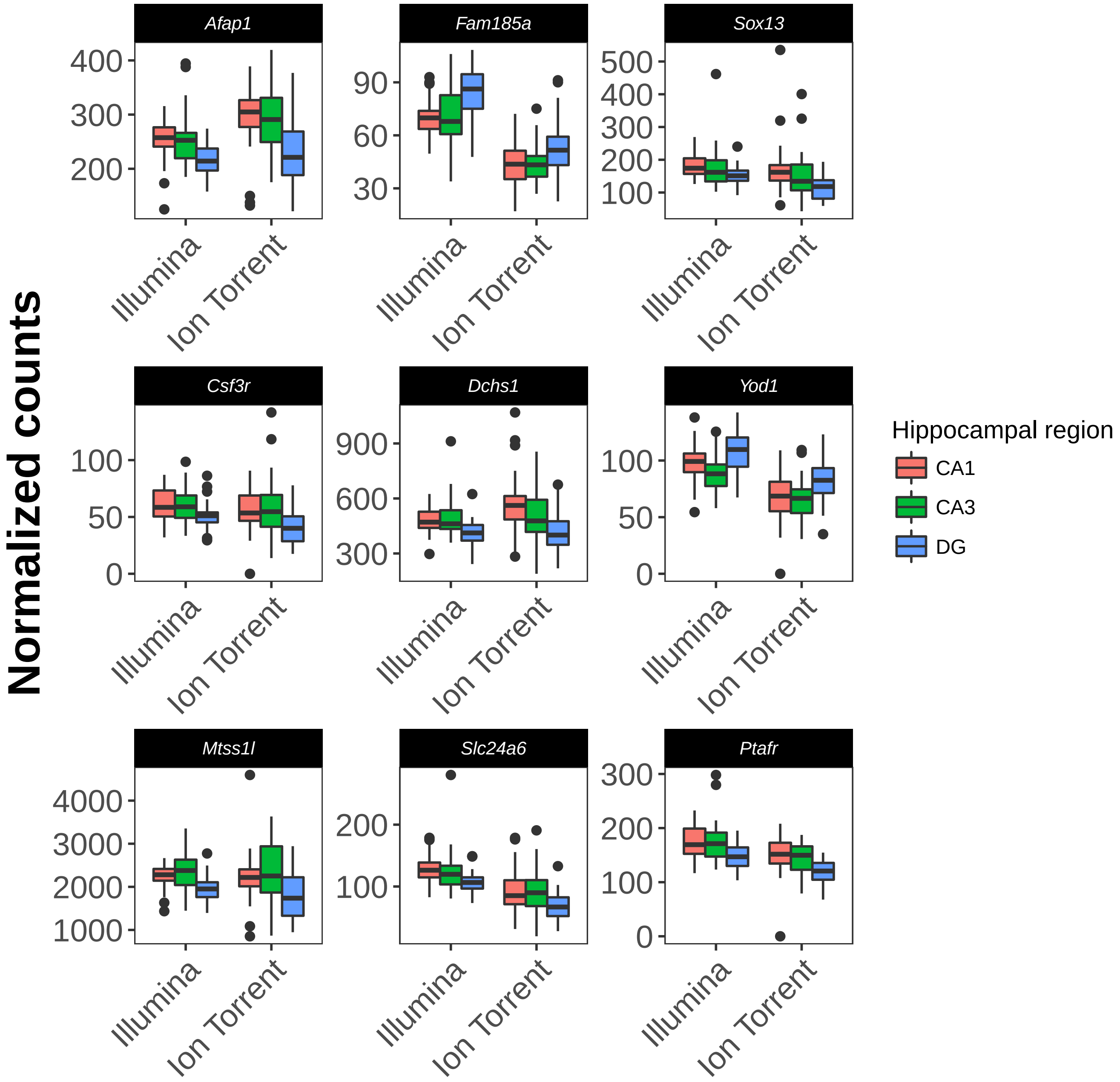
Normalized counts
Afap1
Fam185a
Sox13
Csf3r
Dchs1
Yod1
Mtss1l
Slc24a6
Ptafr
Illumina
Ion Torrent
Hippocampal region
CA1
CA3
DG

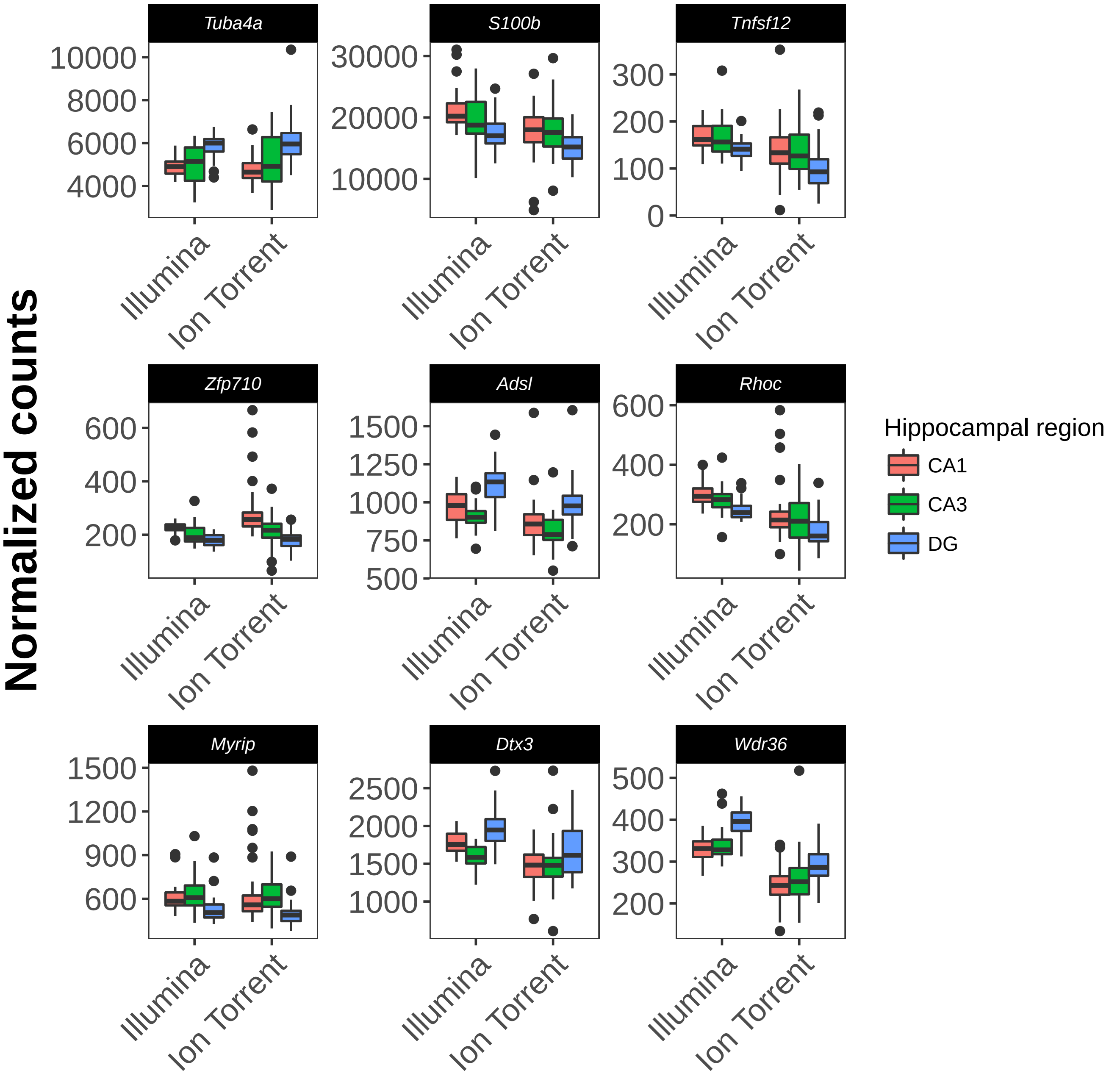

Normalized counts
Tuba4a
S100b
Tnfsf12
Zfp710
Adsl
Rhoc
Myrip
Dtx3
Wdr36
Illumina
Ion Torrent
Hippocampal region
CA1
CA3
DG

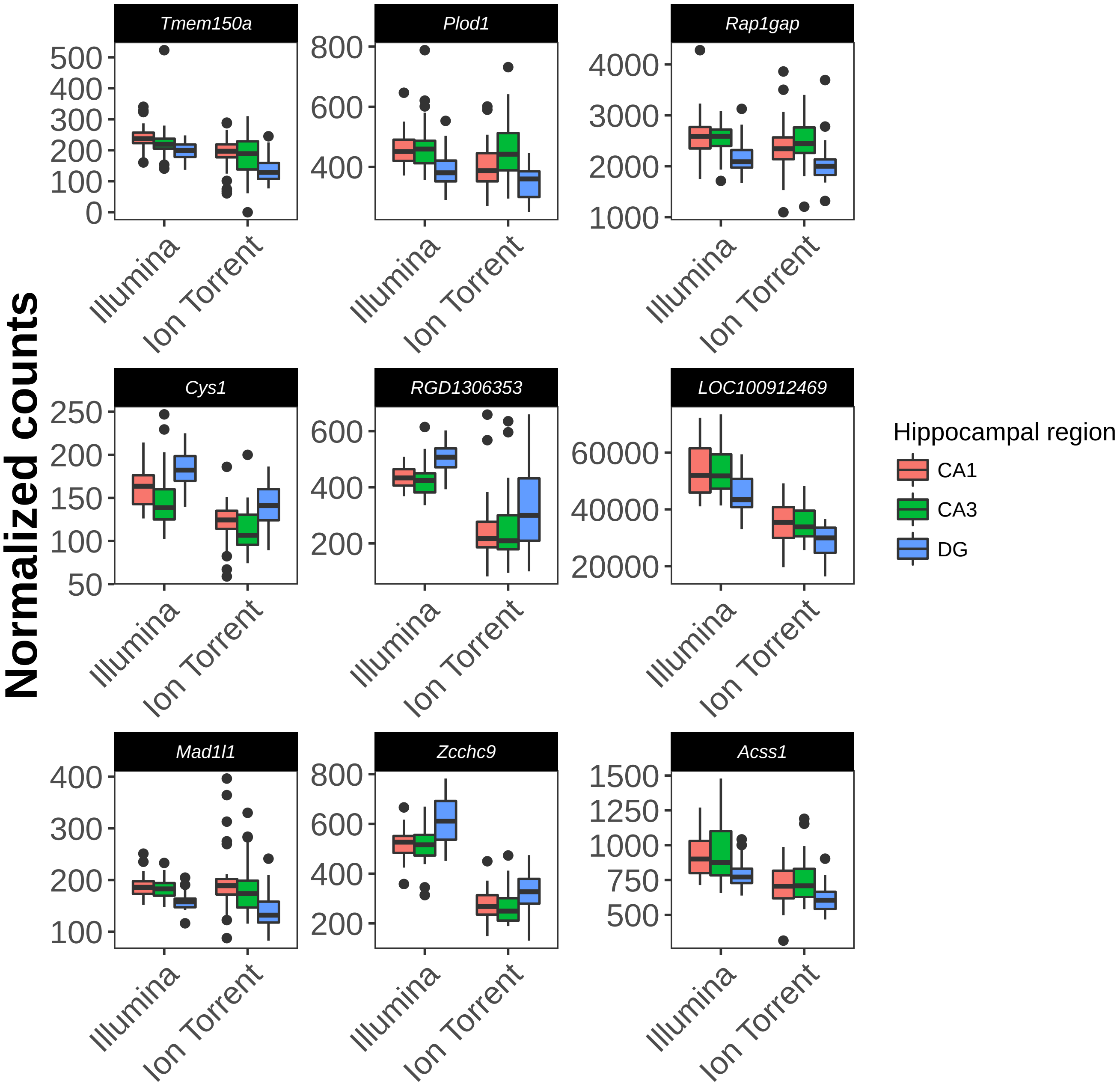
Normalized counts
Tmem150a
Plod1
Rap1gap
Cys1
RGD1306353
LOC100912469
Mad1l1
Zcchc9
Acss1
Illumina
Ion Torrent
Hippocampal region
CA1
CA3
DG

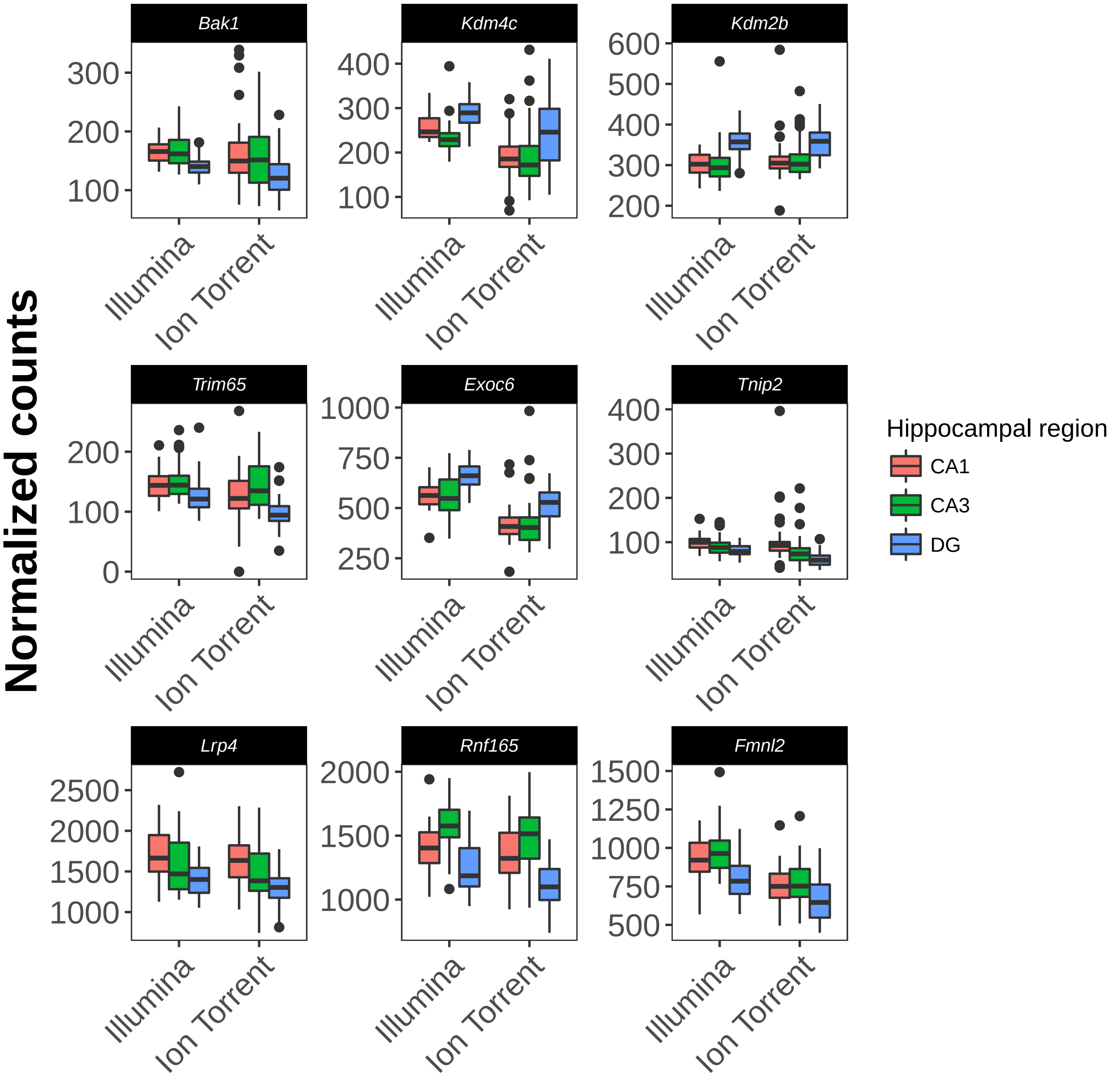
Normalized counts
Bak1
Kdm4c
Kdm2b
Trim65
Exoc6
Tnip2
Lrp4
Rnf165
Fmnl2
Illumina
Ion Torrent
Hippocampal region
CA1
CA3
DG

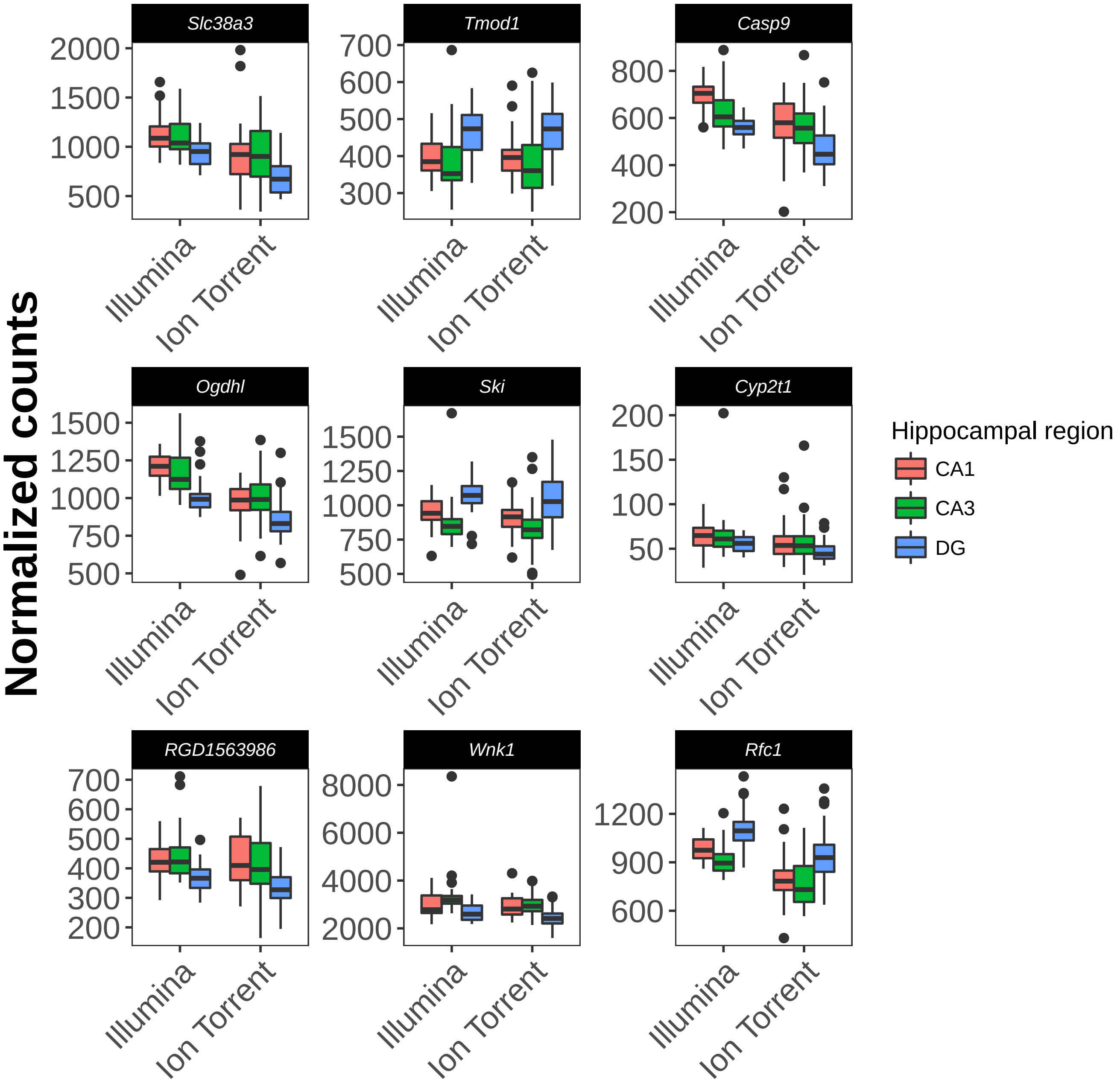

Normalized counts
Slc38a3
Tmod1
Casp9
Ogdhl
Ski
Cyp2t1
RGD1563986
Wnk1
Rfc1
Illumina
Ion Torrent
Hippocampal region
CA1
CA3
DG

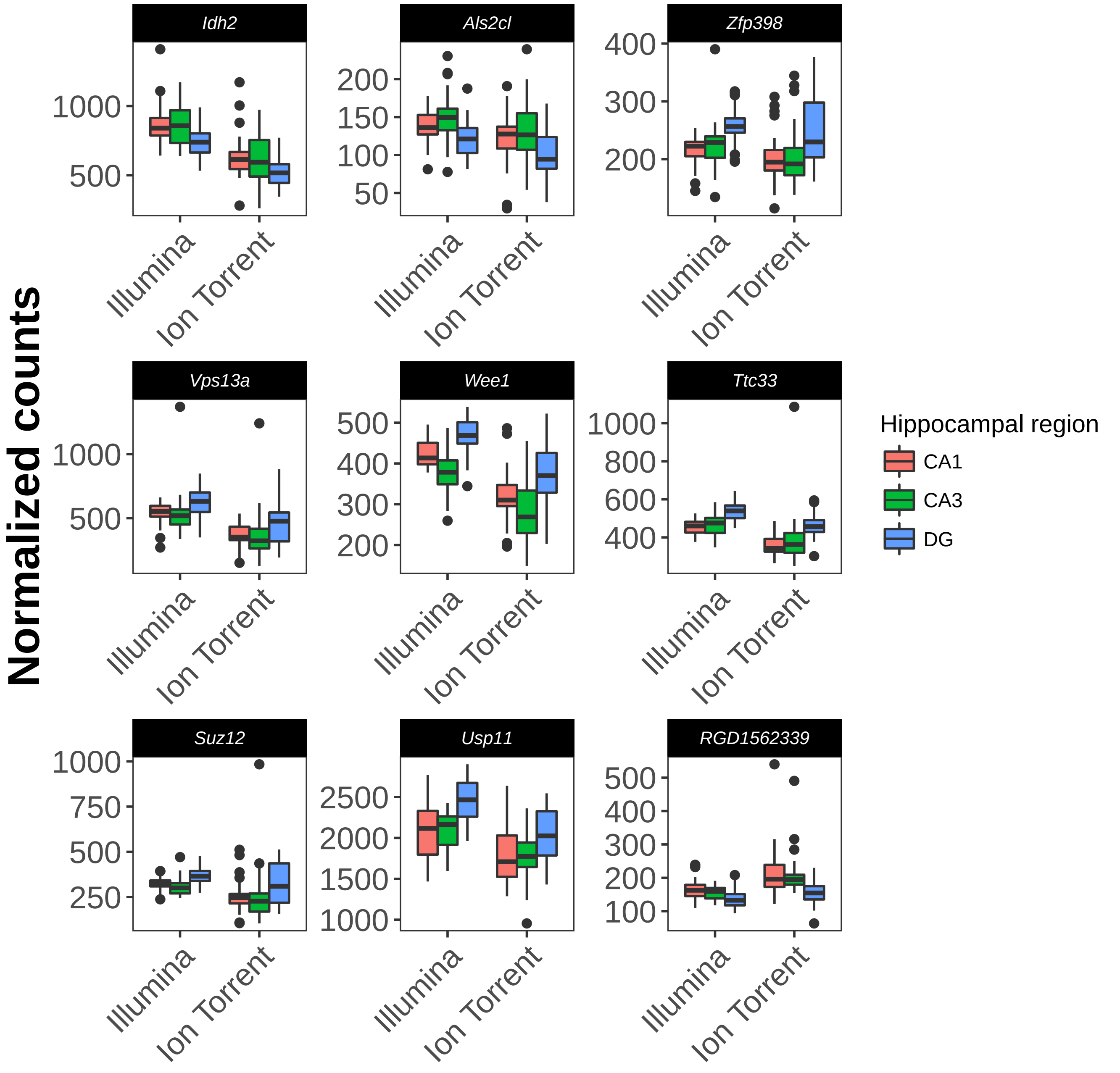
Normalized counts
Idh2
Als2cl
Zfp398
Vps13a
Wee1
Ttc33
Suz12
Usp11
RGD1562339
Illumina
Ion Torrent
Hippocampal region
CA1
CA3
DG

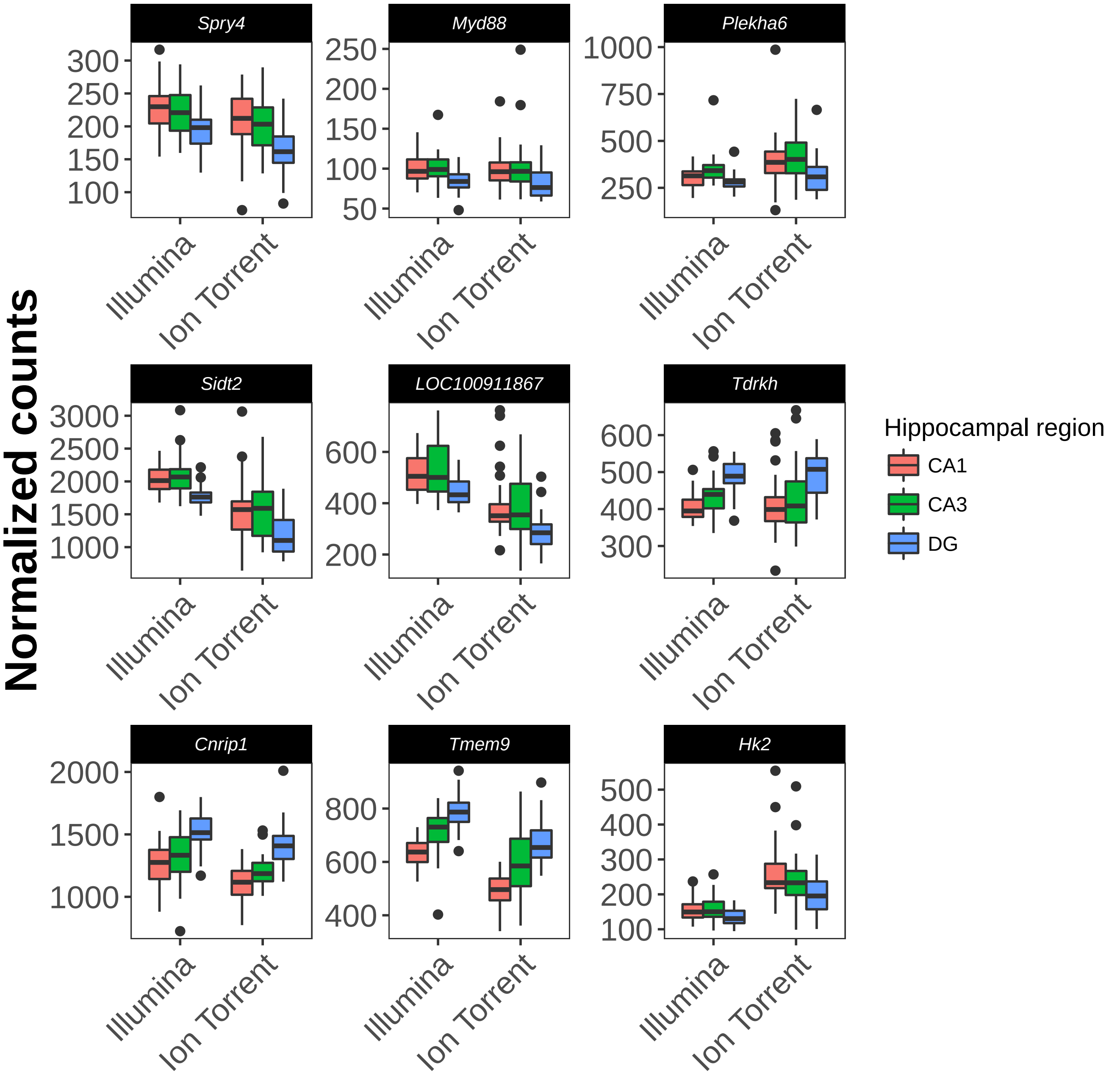
Normalized counts
Spry4
Myd88
Plekha6
Sidt2
LOC100911867
Tdrkh
Cnrip1
Tmem9
Hk2
Illumina
Ion Torrent
Hippocampal region
CA1
CA3
DG

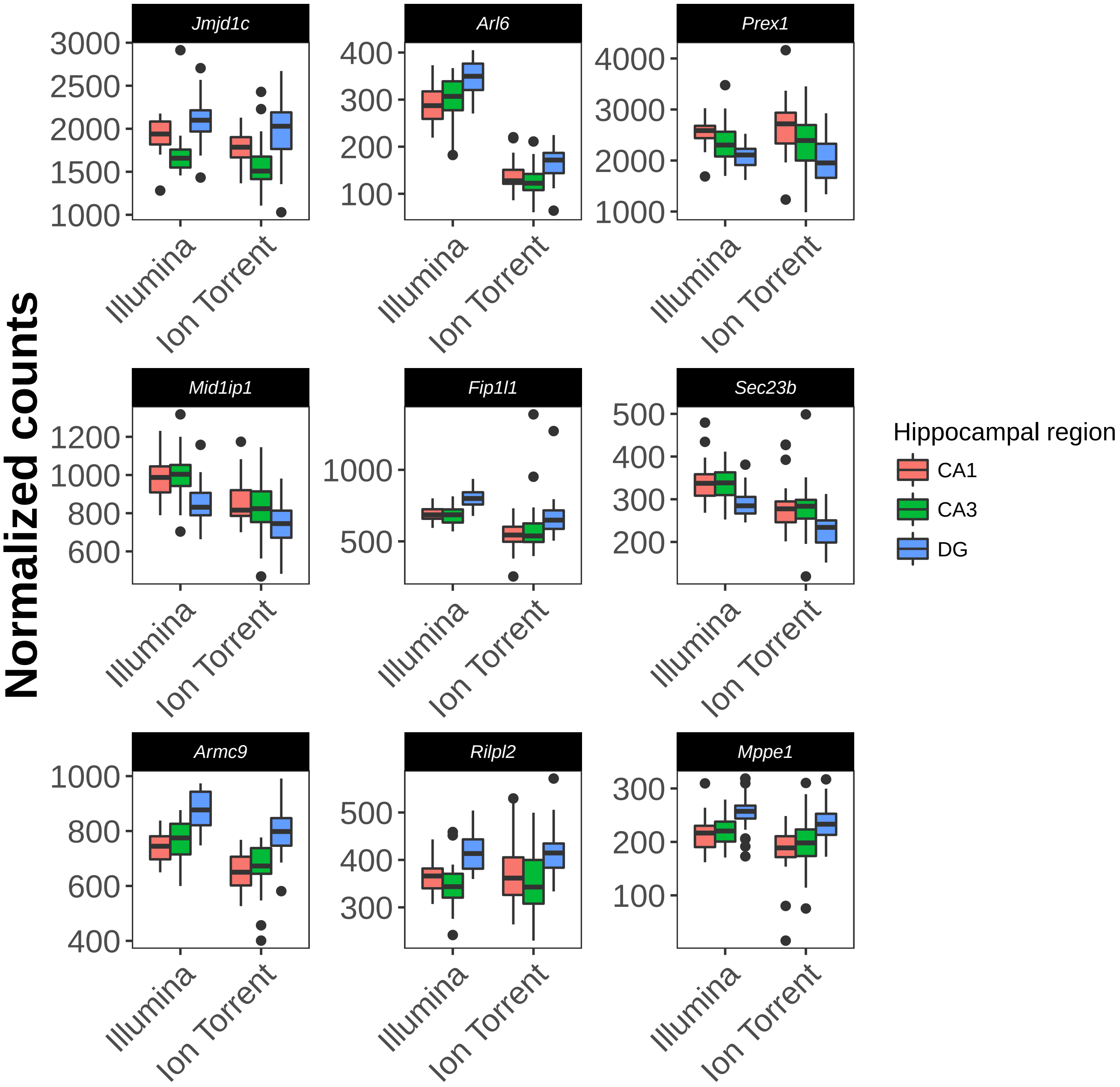

Normalized counts
Jmjd1c
Arl6
Prex1
Mid1ip1
Fip1l1
Sec23b
Armc9
Rilpl2
Mppe1
Illumina
Ion Torrent
Hippocampal region
CA1
CA3
DG

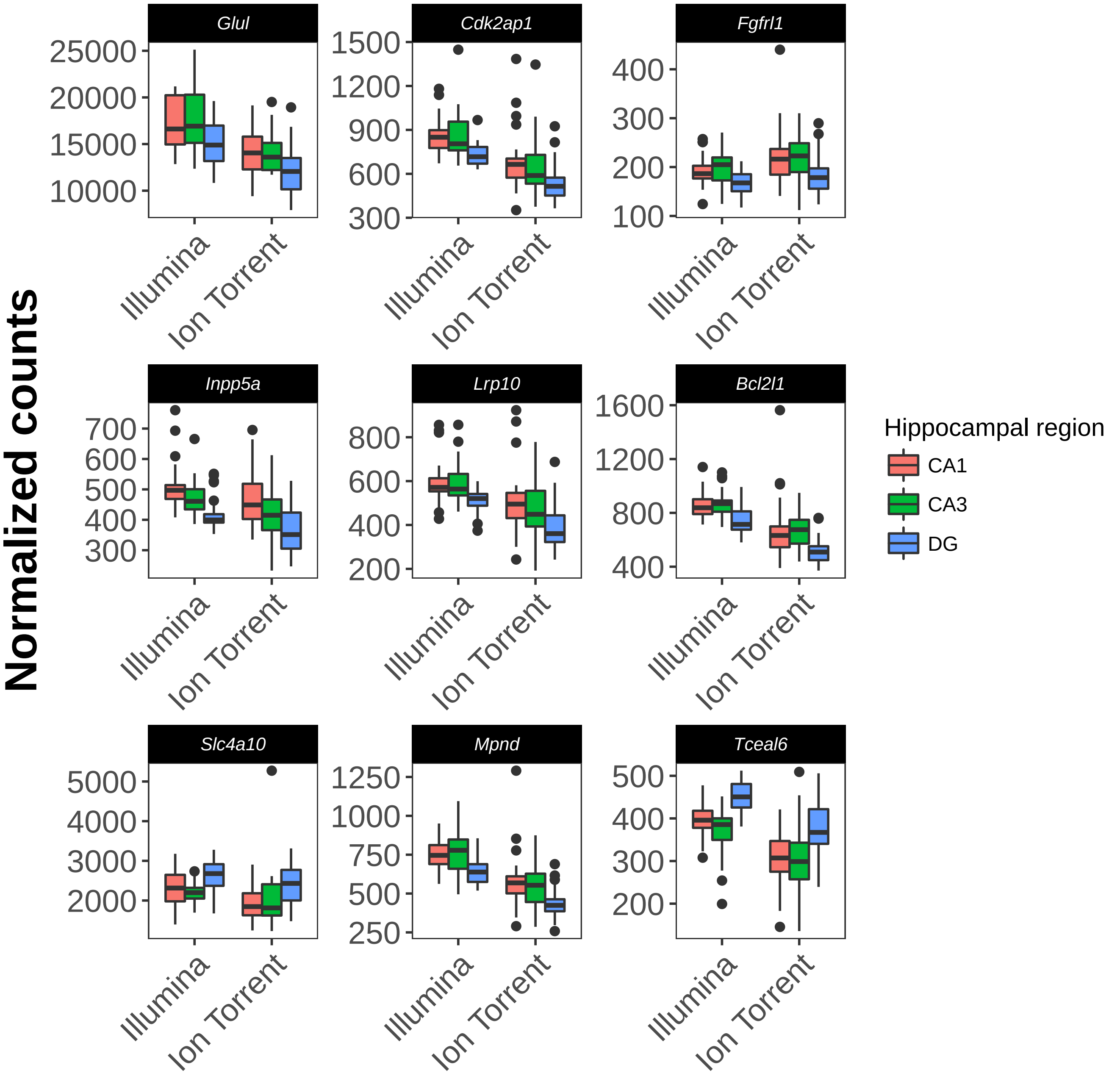
Normalized counts
Glul
Cdk2ap1
Fgfrl1
Inpp5a
Lrp10
Bcl2l1
Slc4a10
Mpnd
Tceal6
Illumina
Ion Torrent
Hippocampal region
CA1
CA3
DG

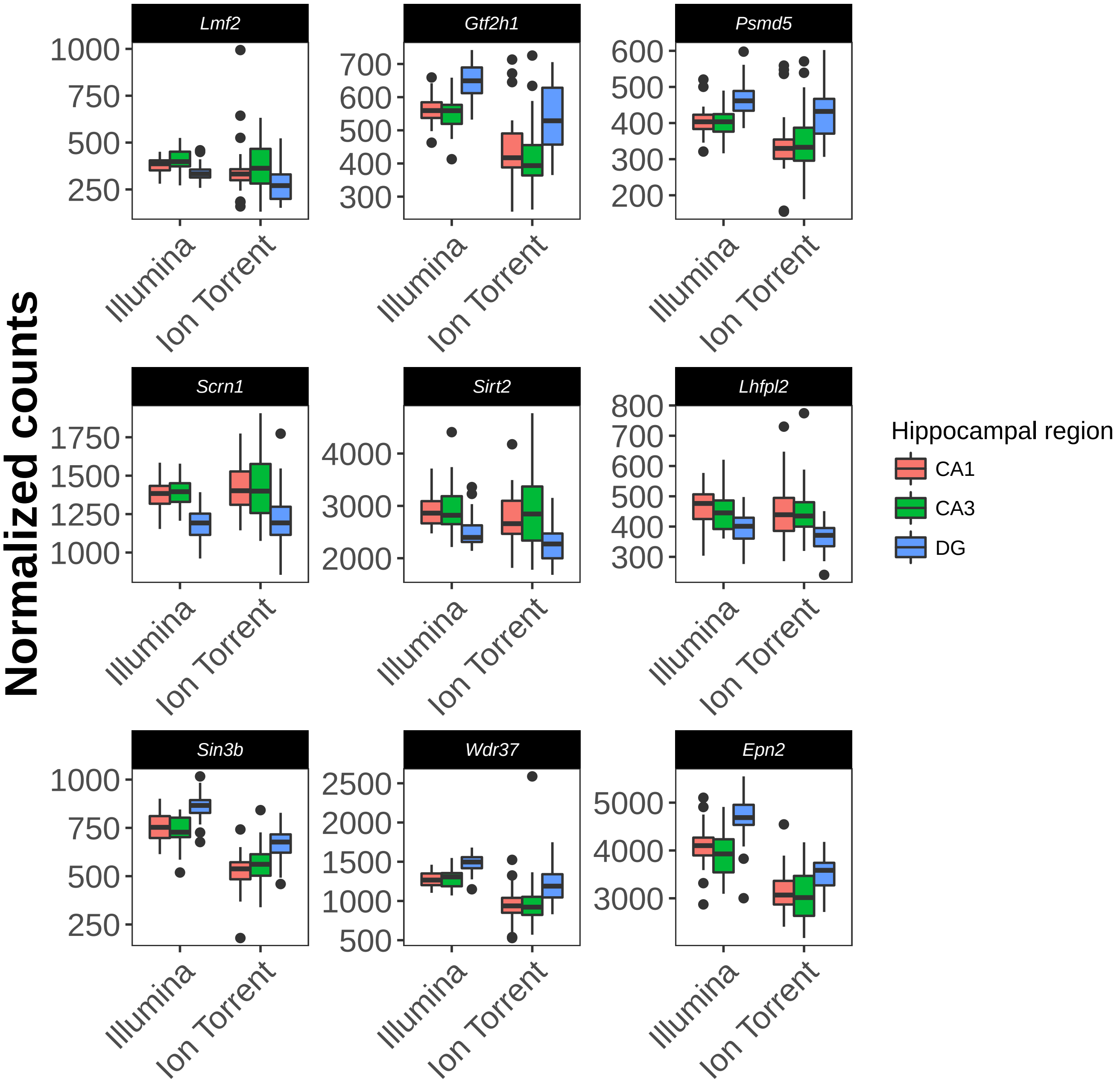
Normalized counts
Lmf2
Gtf2h1
Psmd5
Scrn1
Sirt2
Lhfpl2
Sin3b
Wdr37
Epn2
Illumina
Ion Torrent
Hippocampal region
CA1
CA3
DG

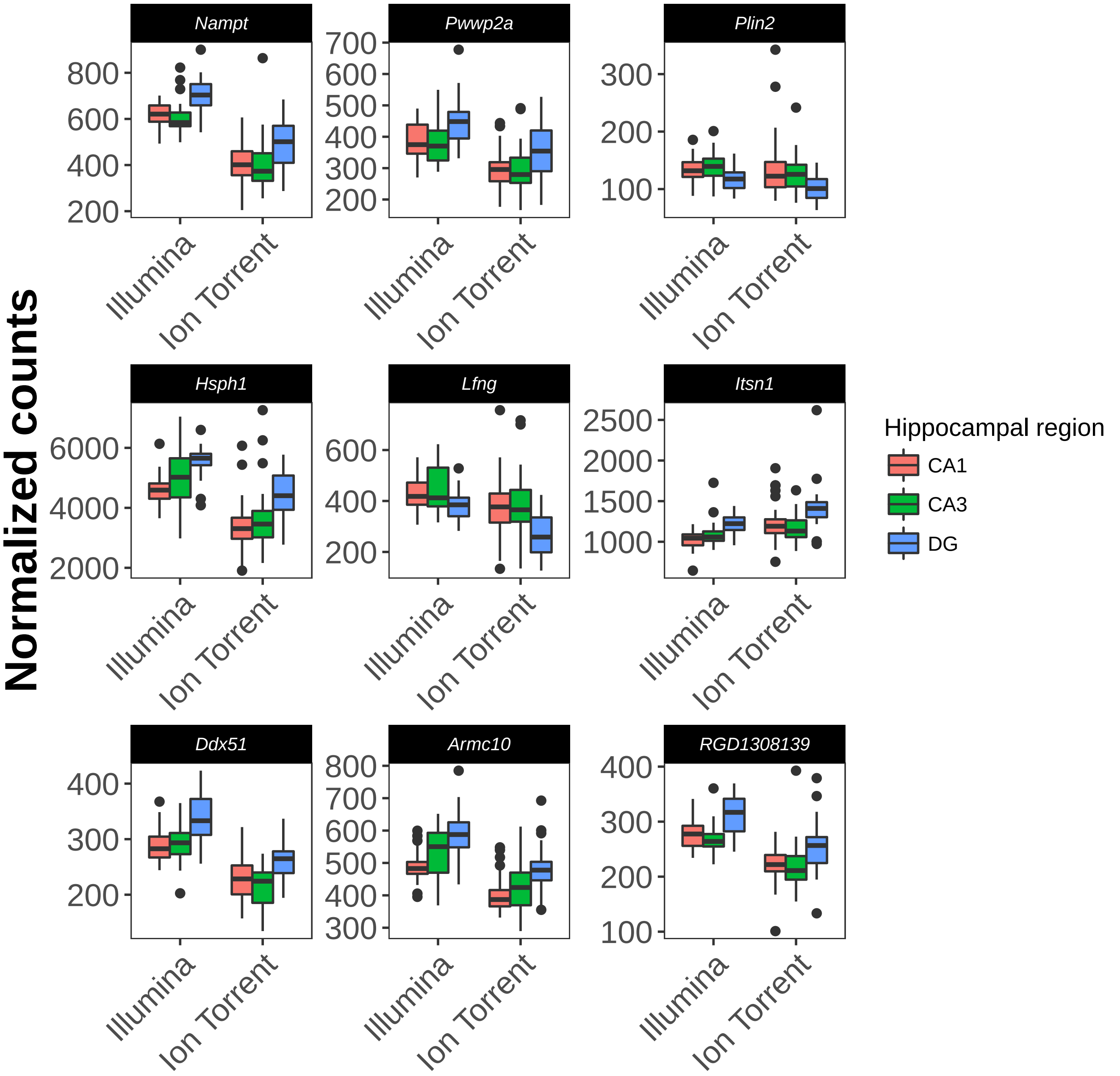

Normalized counts
Nampt
Pwwp2a
Plin2
Hsph1
Lfng
Itsn1
Ddx51
Armc10
RGD1308139
Illumina
Ion Torrent
Hippocampal region
CA1
CA3
DG

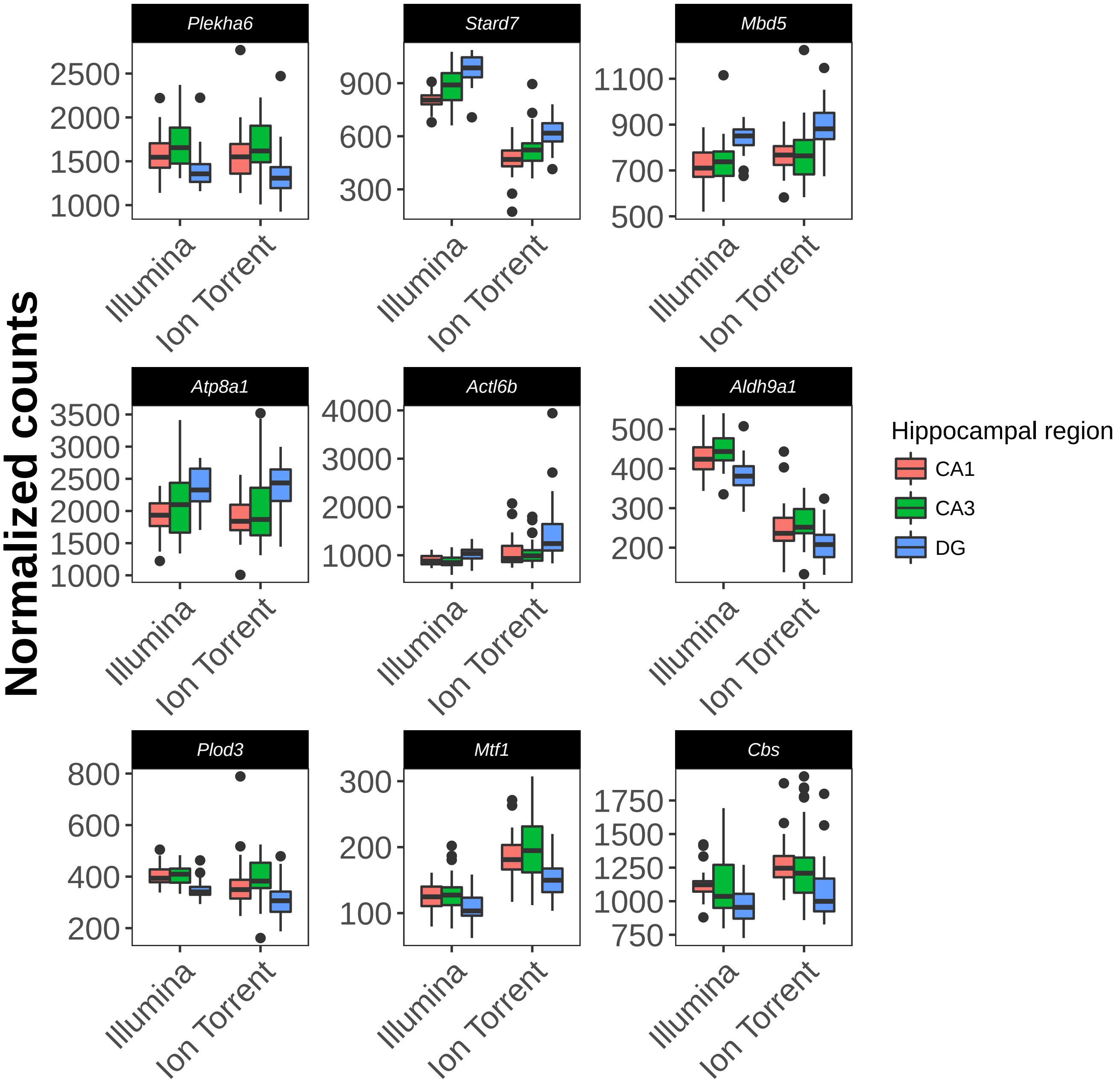
Normalized counts
Plekha6
Stard7
Mbd5
Atp8a1
Actl6b
Aldh9a1
Plod3
Mtf1
Cbs
Illumina
Ion Torrent
Hippocampal region
CA1
CA3
DG

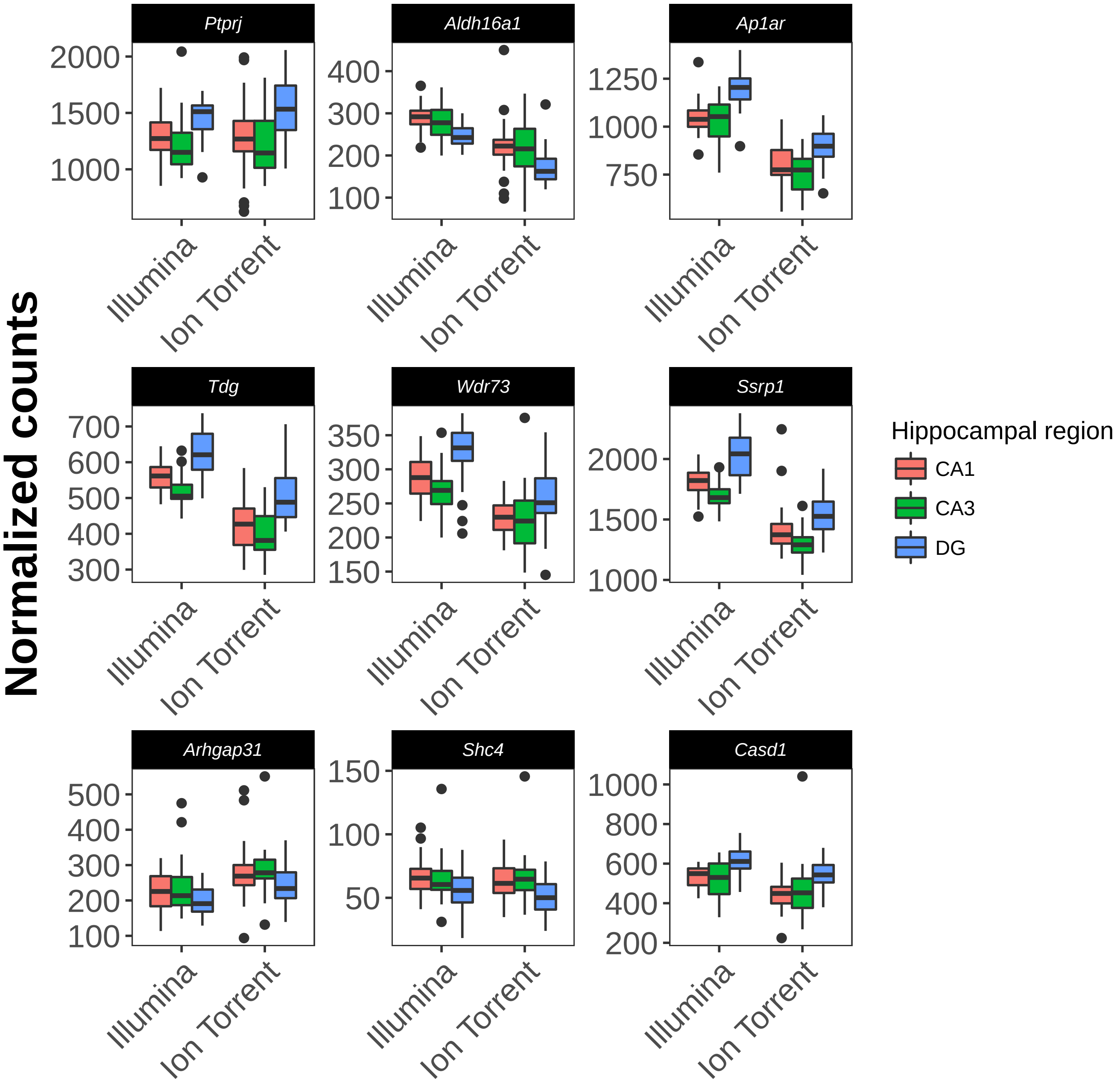

Normalized counts
Ptprj
Aldh16a1
Ap1ar
Tdg
Wdr73
Ssrp1
Arhgap31
Shc4
Casd1
Illumina
Ion Torrent
Hippocampal region
CA1
CA3
DG

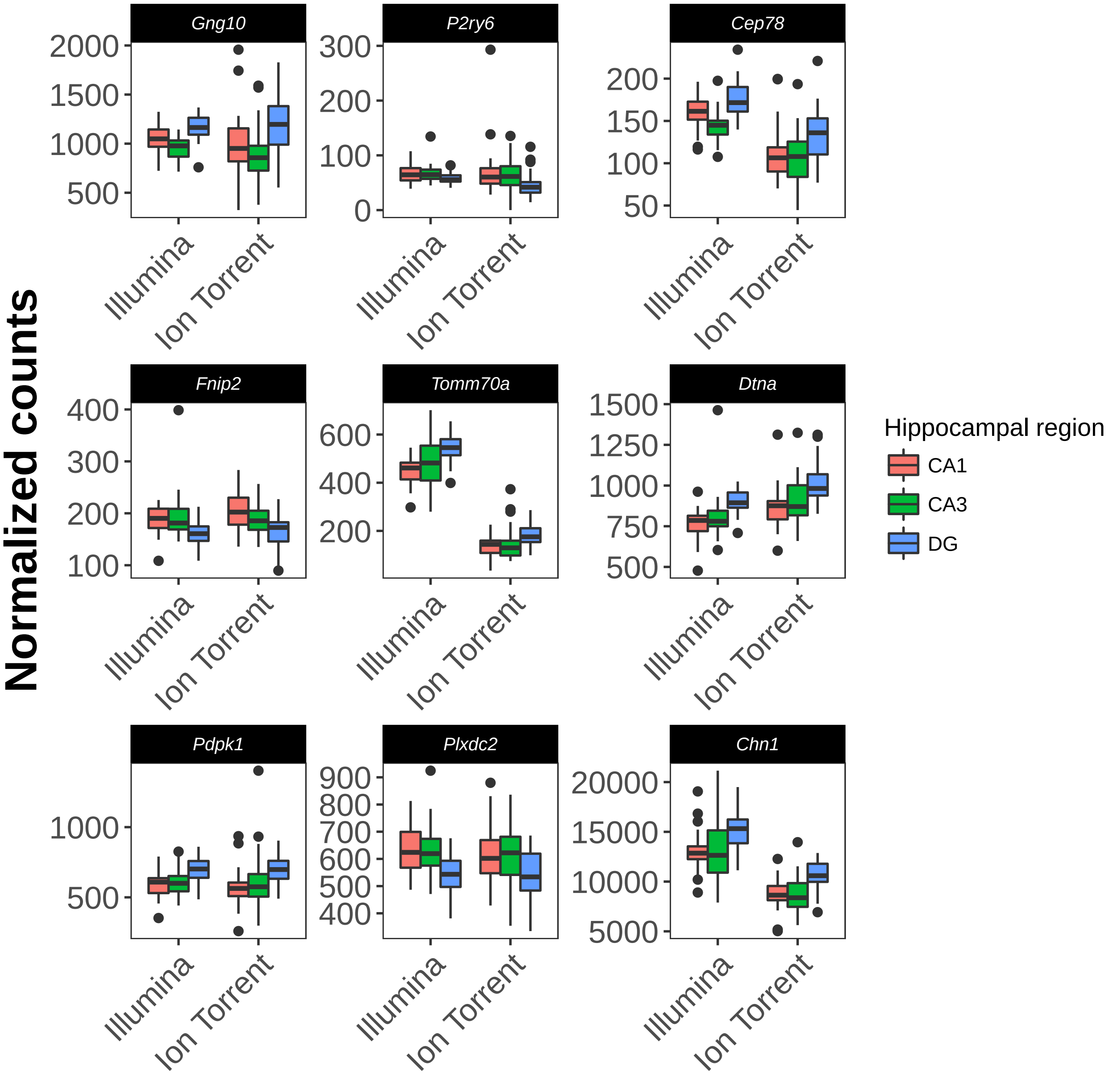
Normalized counts
Gng10
P2ry6
Cep78
Fnip2
Tomm70a
Dtna
Pdpk1
Plxdc2
Chn1
Illumina
Ion Torrent
Hippocampal region
CA1
CA3
DG

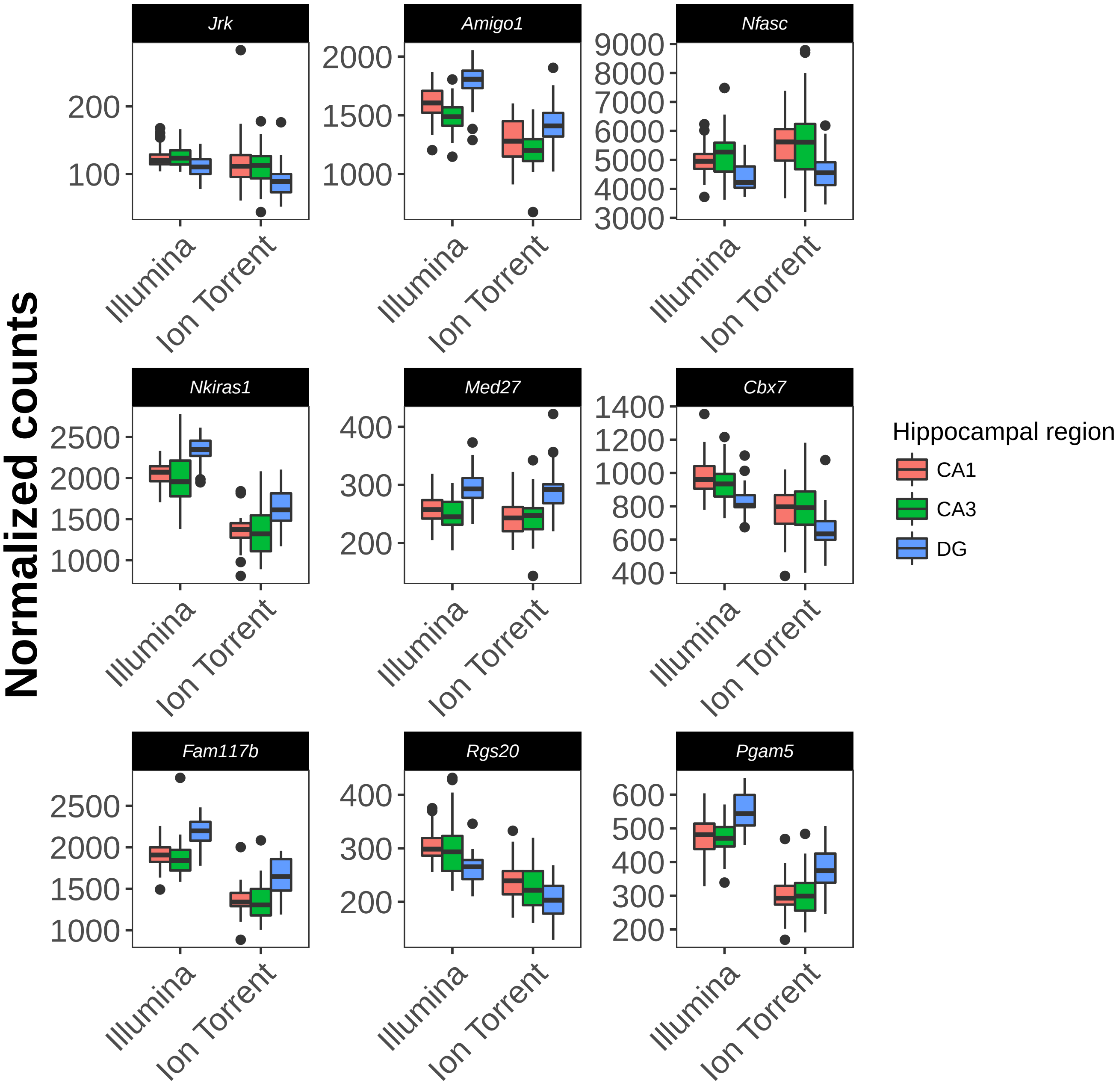
Normalized counts
Jrk
Amigo1
Nfasc
Nkiras1
Med27
Cbx7
Fam117b
Rgs20
Pgam5
Illumina
Ion Torrent
Hippocampal region
CA1
CA3
DG

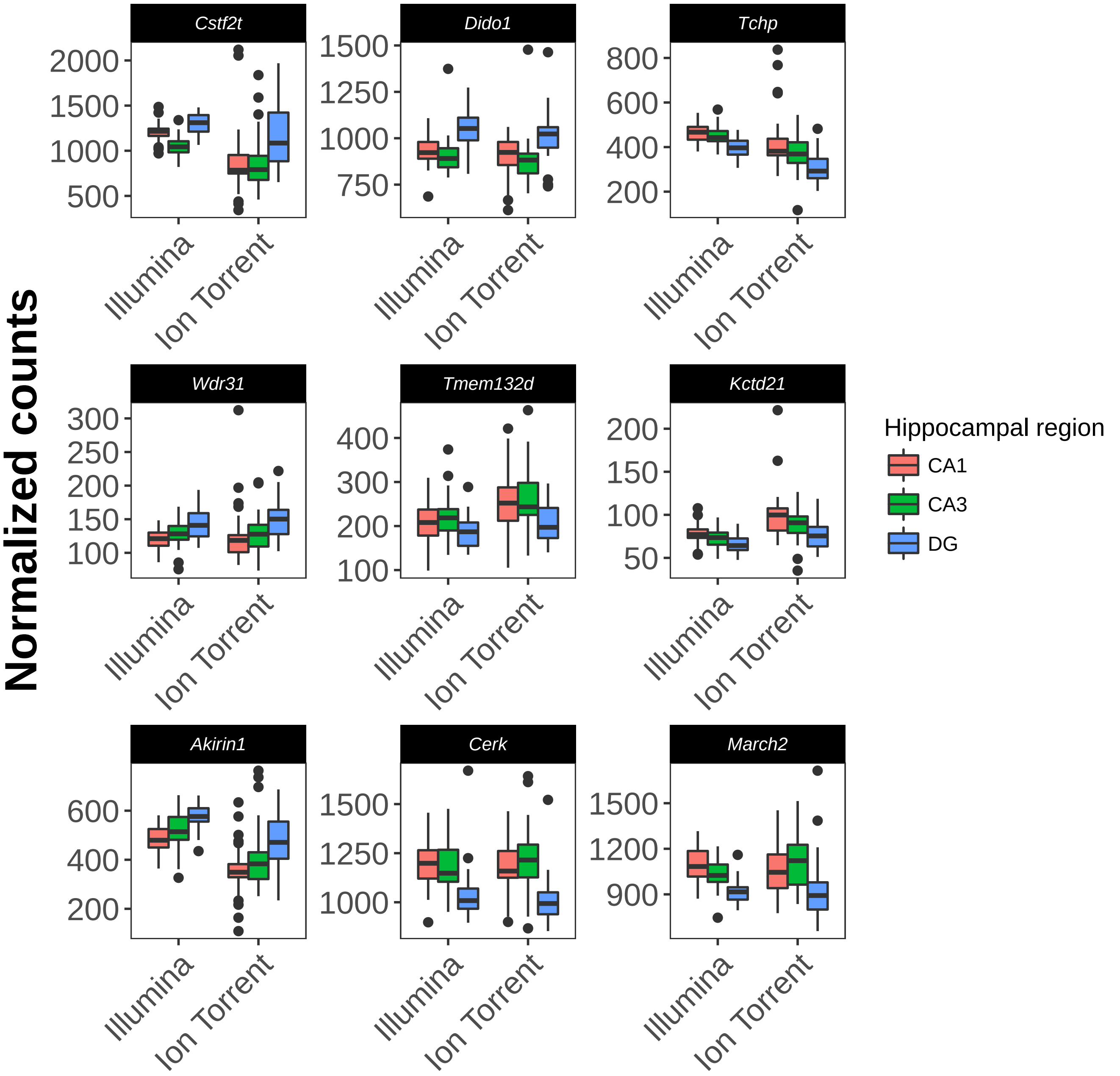
Normalized counts
Cstf2t
Dido1
Tchp
Wdr31
Tmem132d
Kctd21
Akirin1
Cerk
March2
Illumina
Ion Torrent
Hippocampal region
CA1
CA3
DG

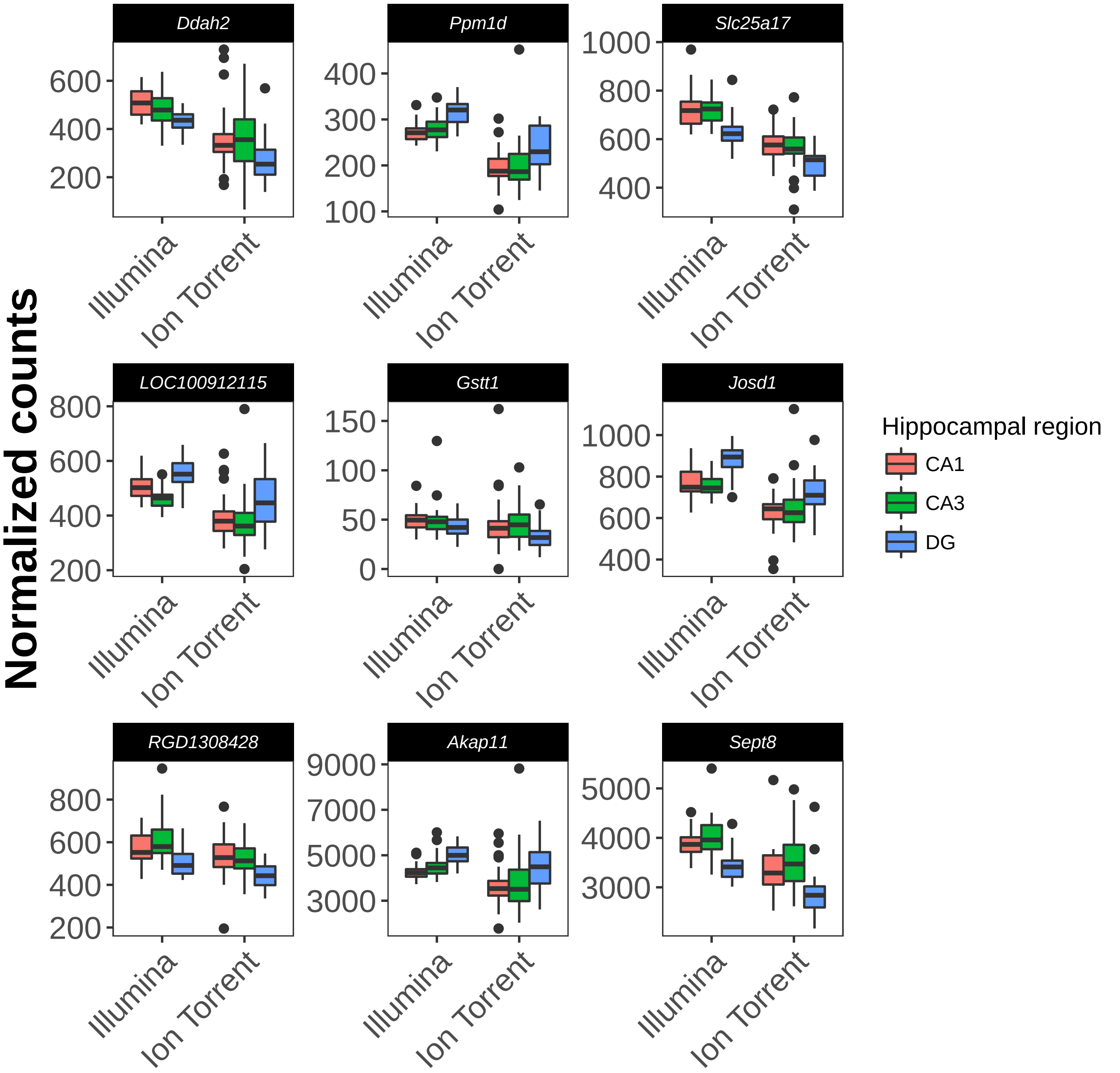
Normalized counts
Ddah2
Ppm1d
Slc25a17
LOC100912115
Gstt1
Josd1
RGD1308428
Akap11
Sept8
Illumina
Ion Torrent
Hippocampal region
CA1
CA3
DG

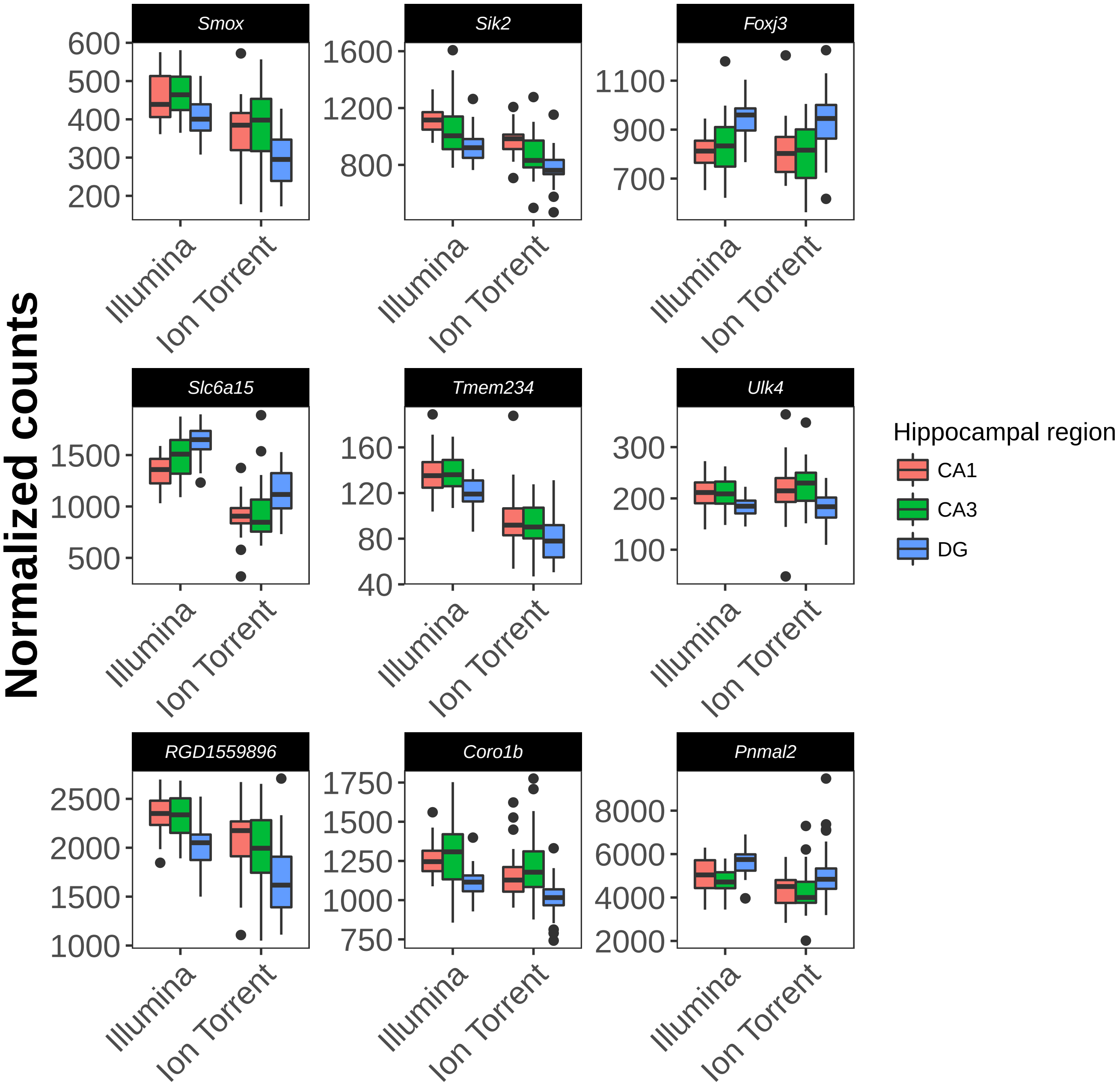
Normalized counts
Smox
Sik2
Foxj3
Slc6a15
Tmem234
Ulk4
RGD1559896
Coro1b
Pnmal2
Illumina
Ion Torrent
Hippocampal region
CA1
CA3
DG

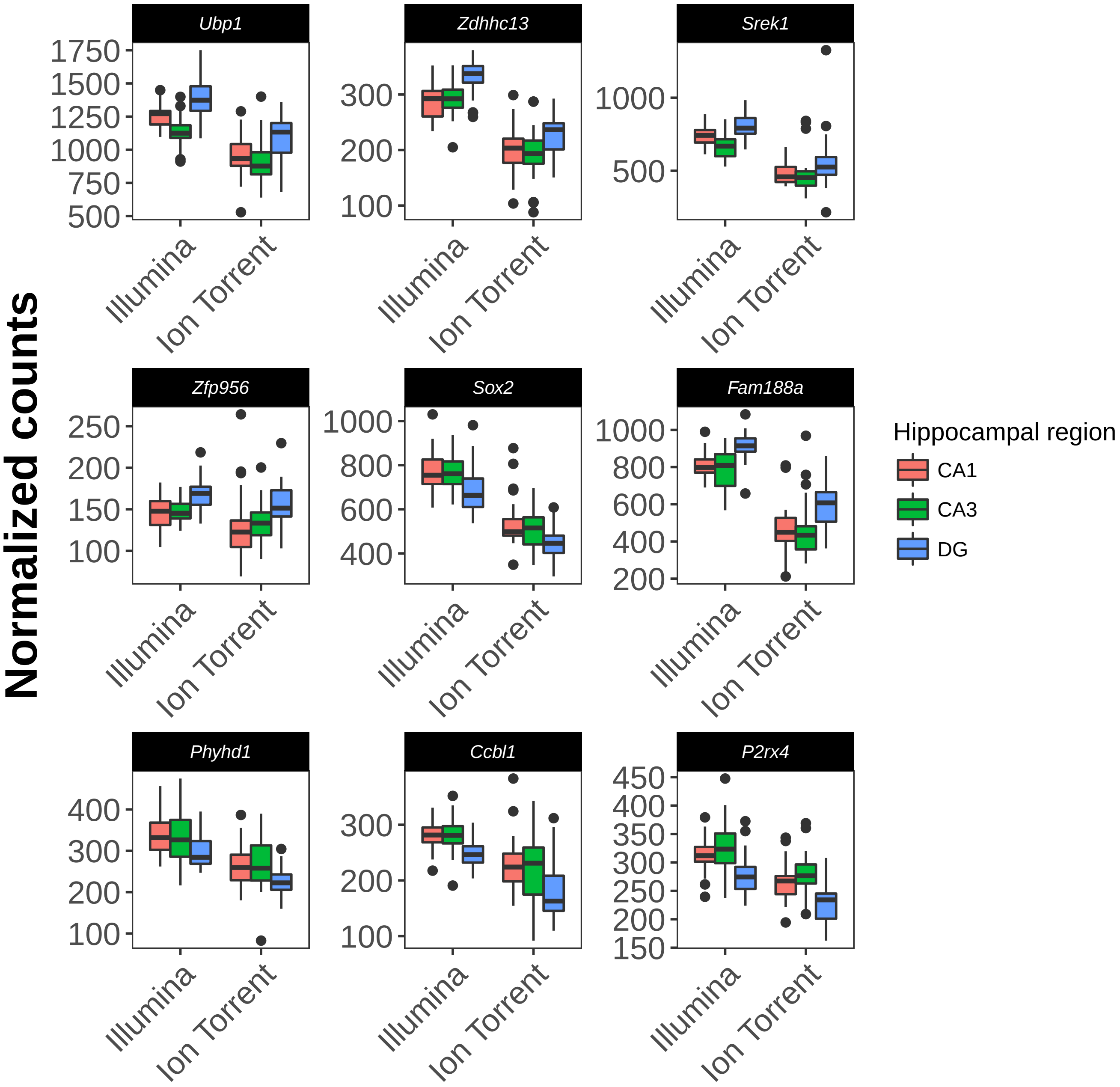
Normalized counts
Ubp1
Zdhhc13
Srek1
Zfp956
Sox2
Fam188a
Phyhd1
Ccbl1
P2rx4
Illumina
Ion Torrent
Hippocampal region
CA1
CA3
DG

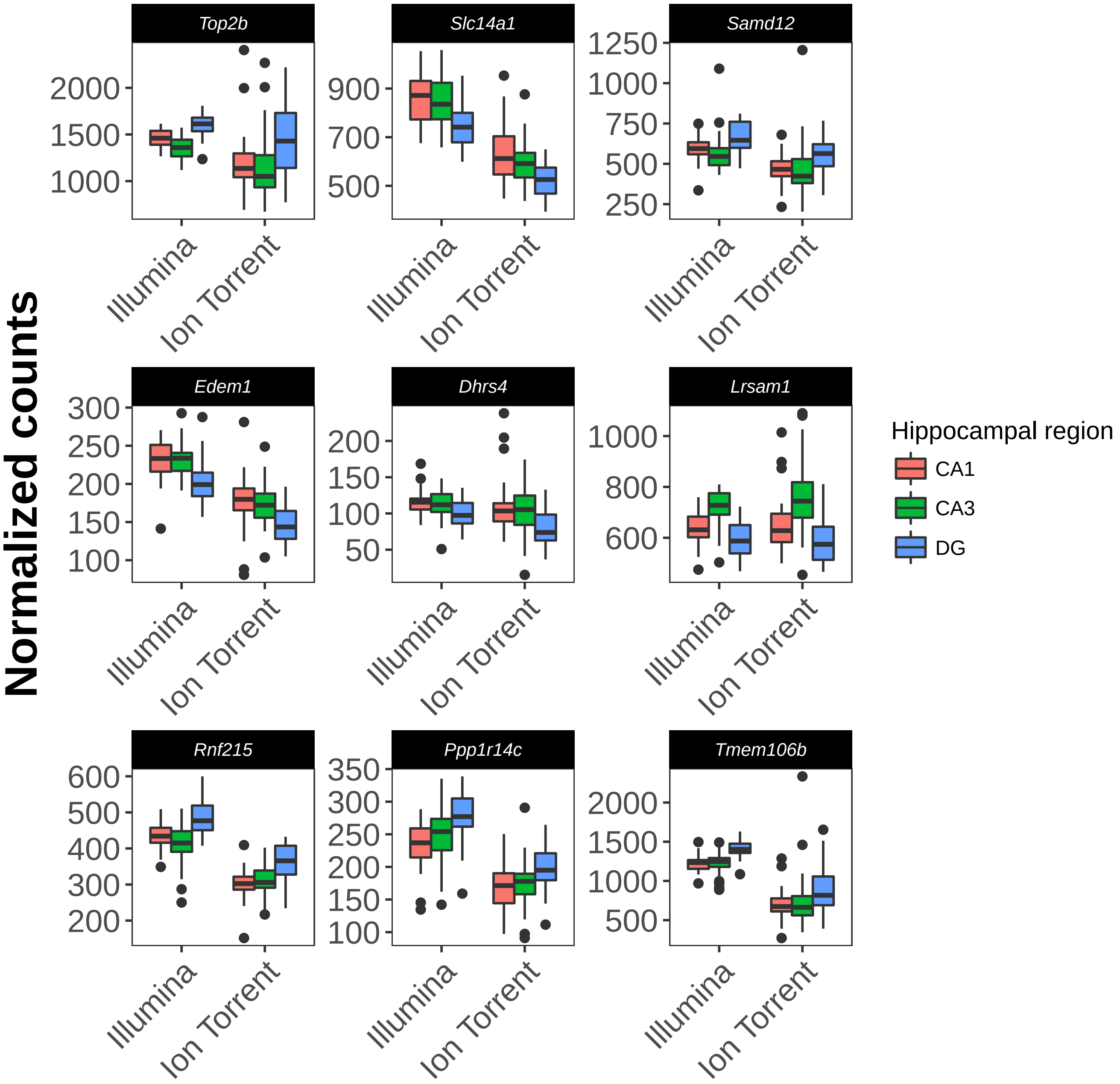
Normalized counts
Top2b
Slc14a1
Samd12
Edem1
Dhrs4
Lrsam1
Rnf215
Ppp1r14c
Tmem106b
Illumina
Ion Torrent
Hippocampal region
CA1
CA3
DG

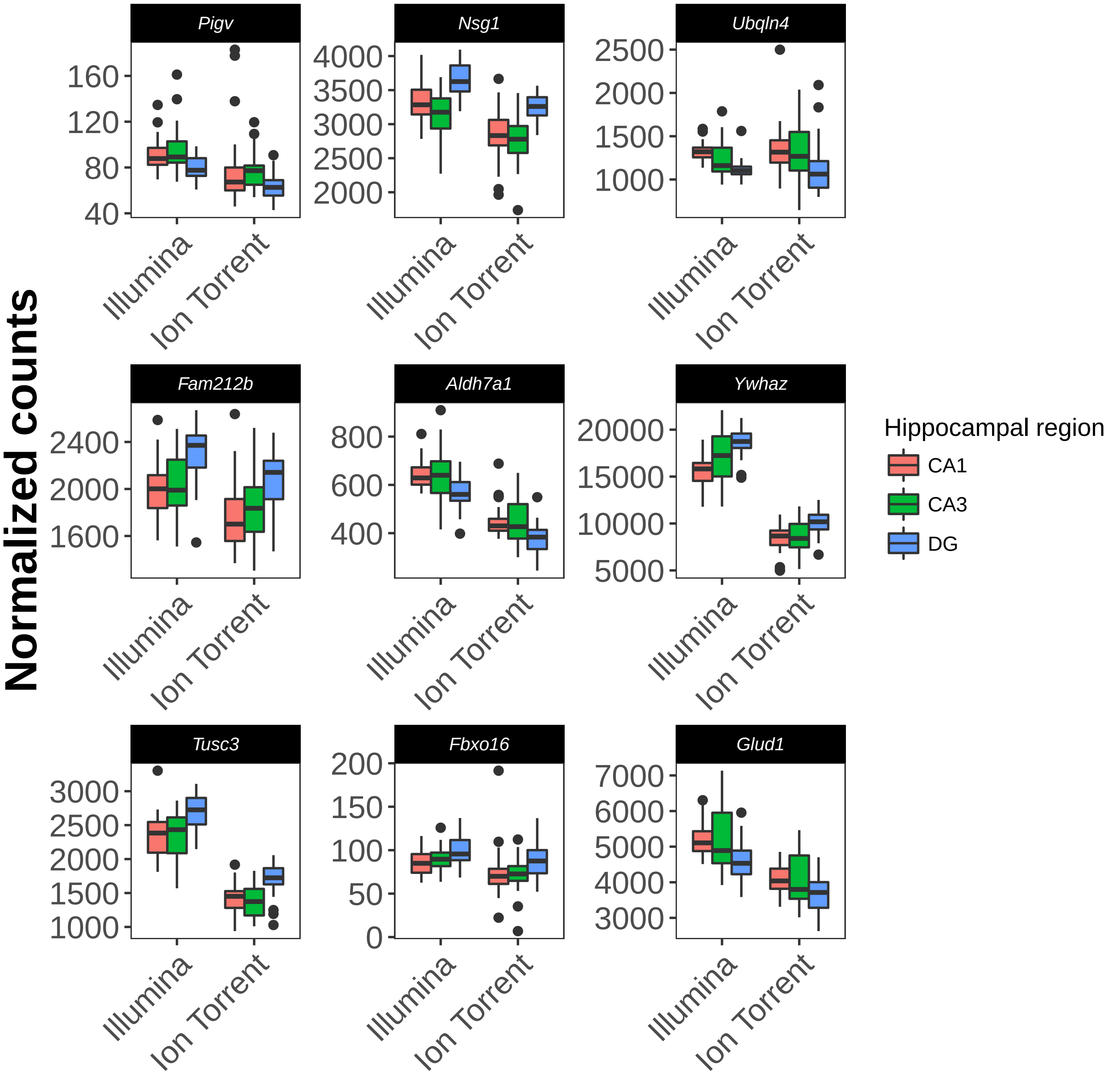

Normalized counts
Pigv
Nsg1
Ubqln4
Fam212b
Aldh7a1
Ywhaz
Tusc3
Fbxo16
Glud1
Illumina
Ion Torrent
Hippocampal region
CA1
CA3
DG

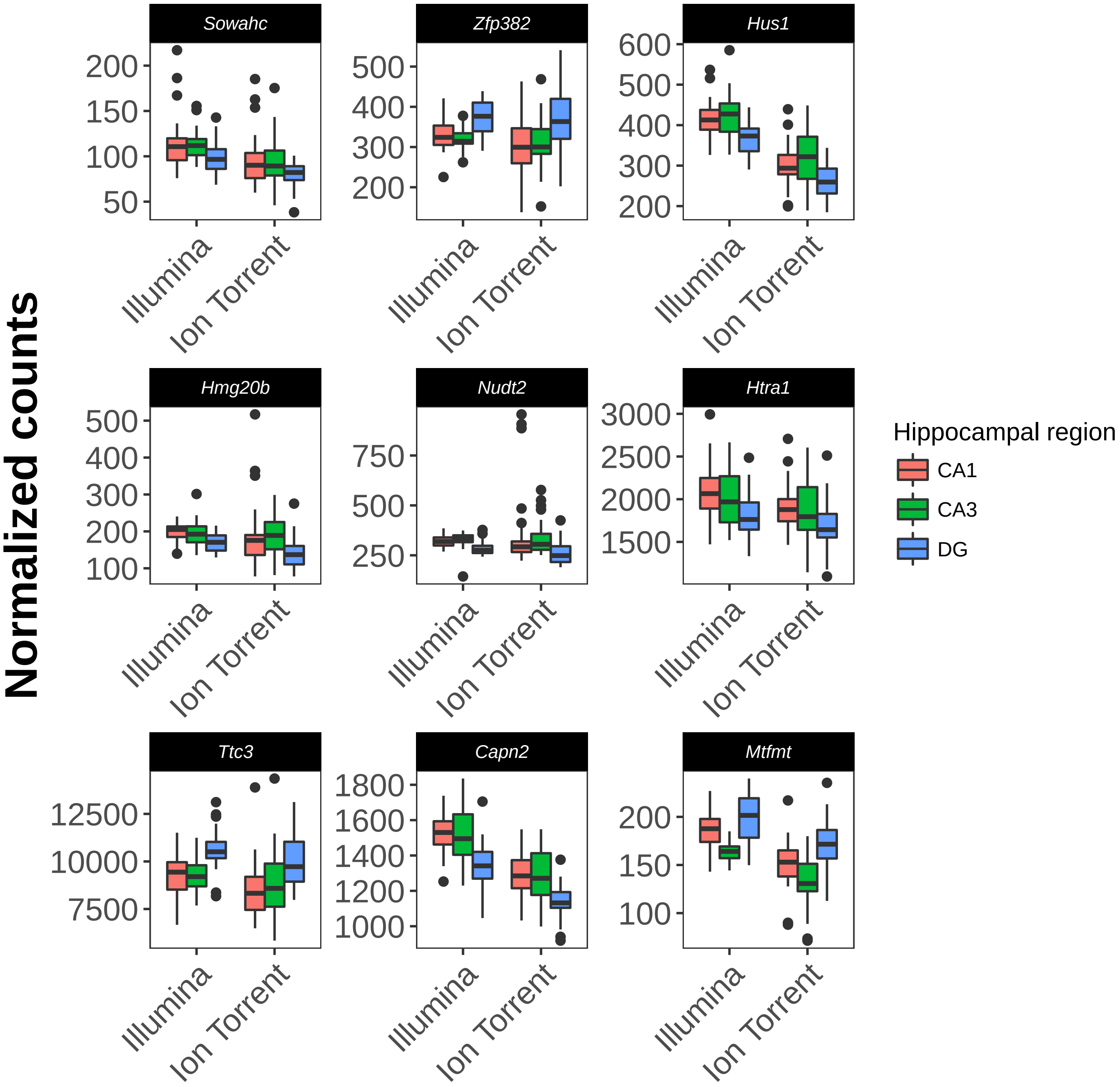
Normalized counts
Sowahc
Zfp382
Hus1
Hmg20b
Nudt2
Htra1
Ttc3
Capn2
Mtfmt
Illumina
Ion Torrent
Hippocampal region
CA1
CA3
DG

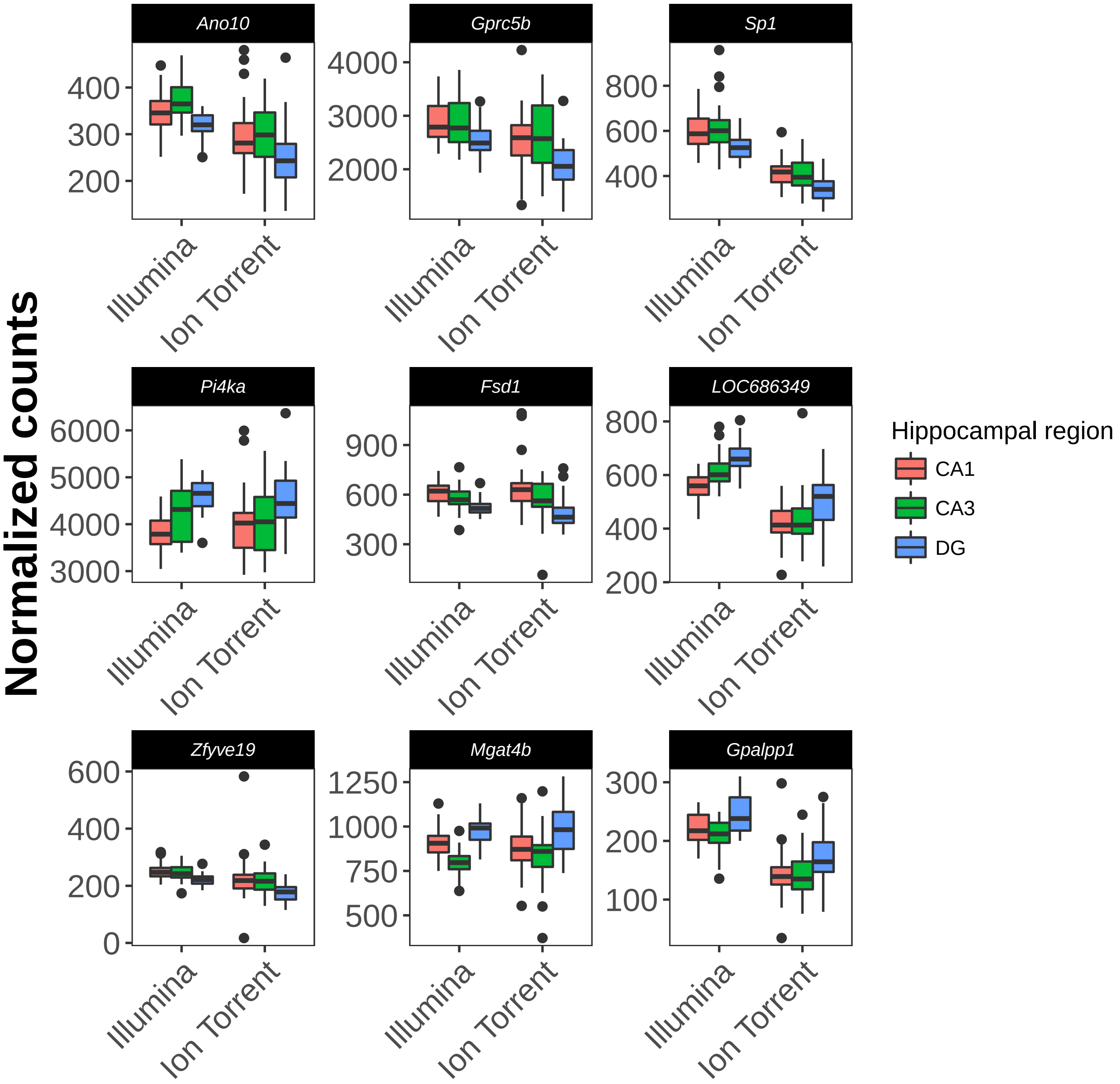
Ano10
Gprc5b
Sp1
Pi4ka
Fsd1
LOC686349
Zfyve19
Mgat4b
Gpalpp1
Normalized counts
Illumina
Ion Torrent
Hippocampal region
CA1
CA3
DG

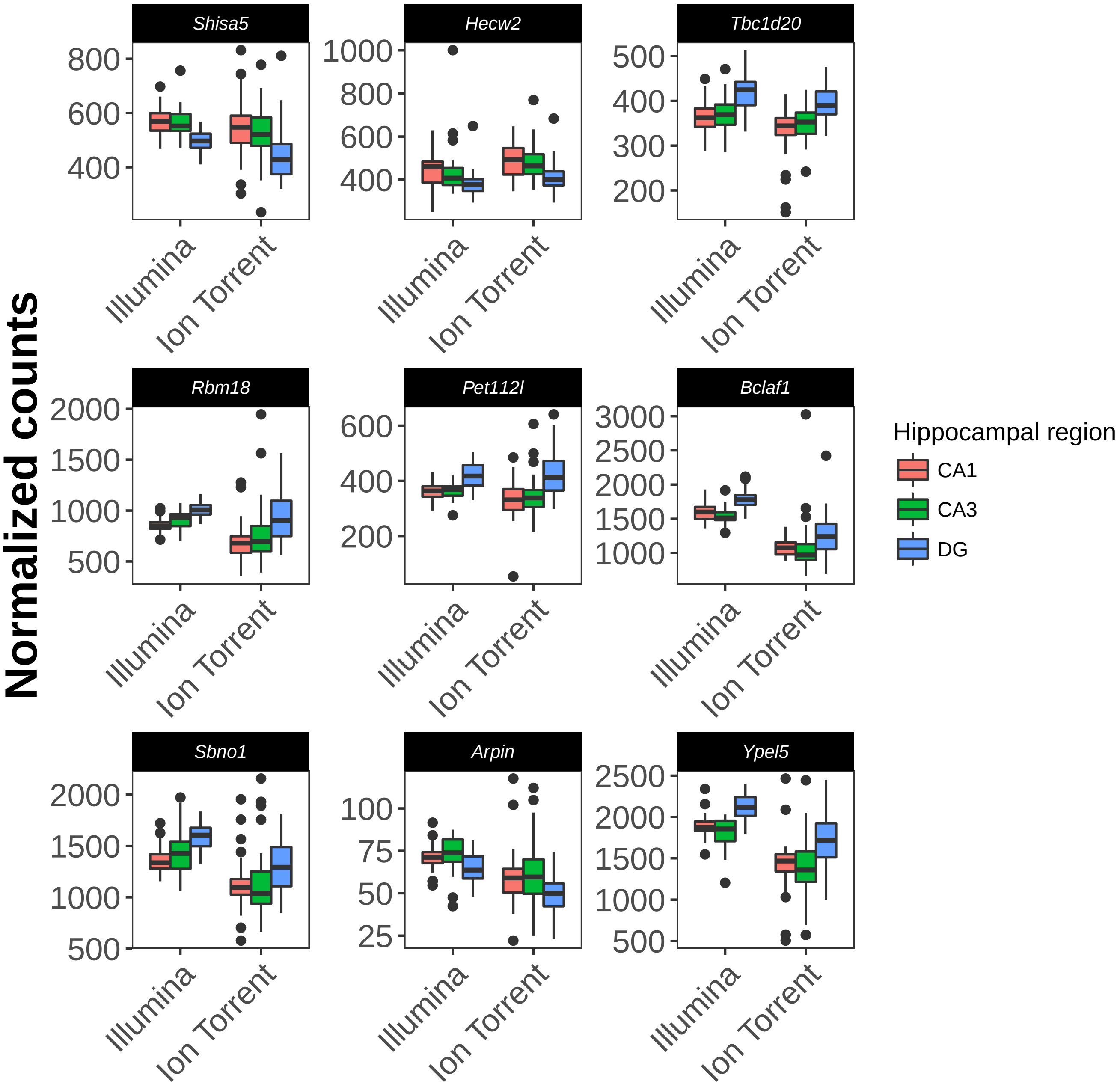

Normalized counts
Shisa5
Hecw2
Tbc1d20
Rbm18
Pet112l
Bclaf1
Sbno1
Arpin
Ypel5
Illumina
Ion Torrent
Hippocampal region
CA1
CA3
DG

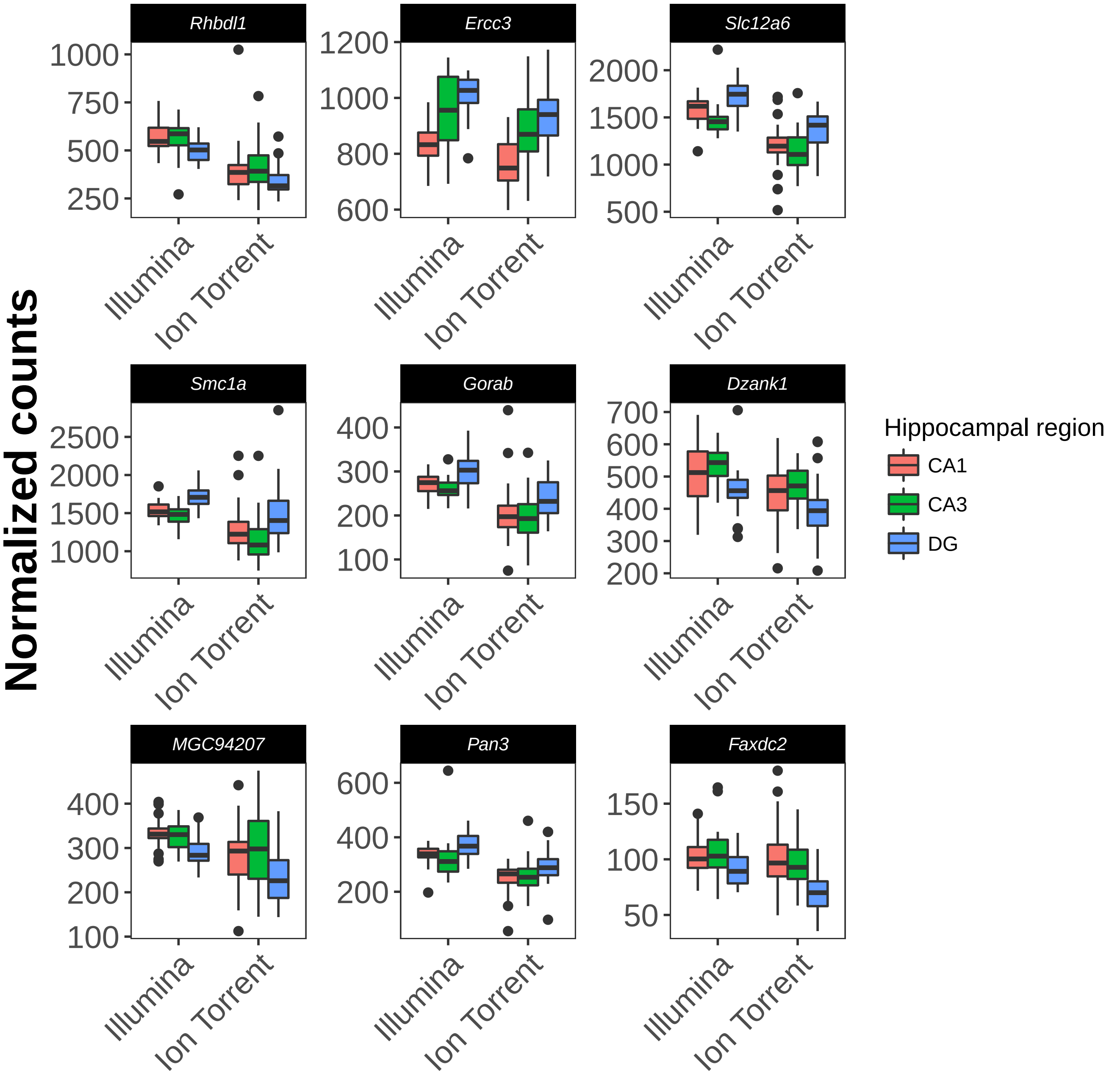

Normalized counts
Rhbdl1
Ercc3
Slc12a6
Smc1a
Gorab
Dzank1
MGC94207
Pan3
Faxdc2
Illumina
Ion Torrent
Hippocampal region
CA1
CA3
DG

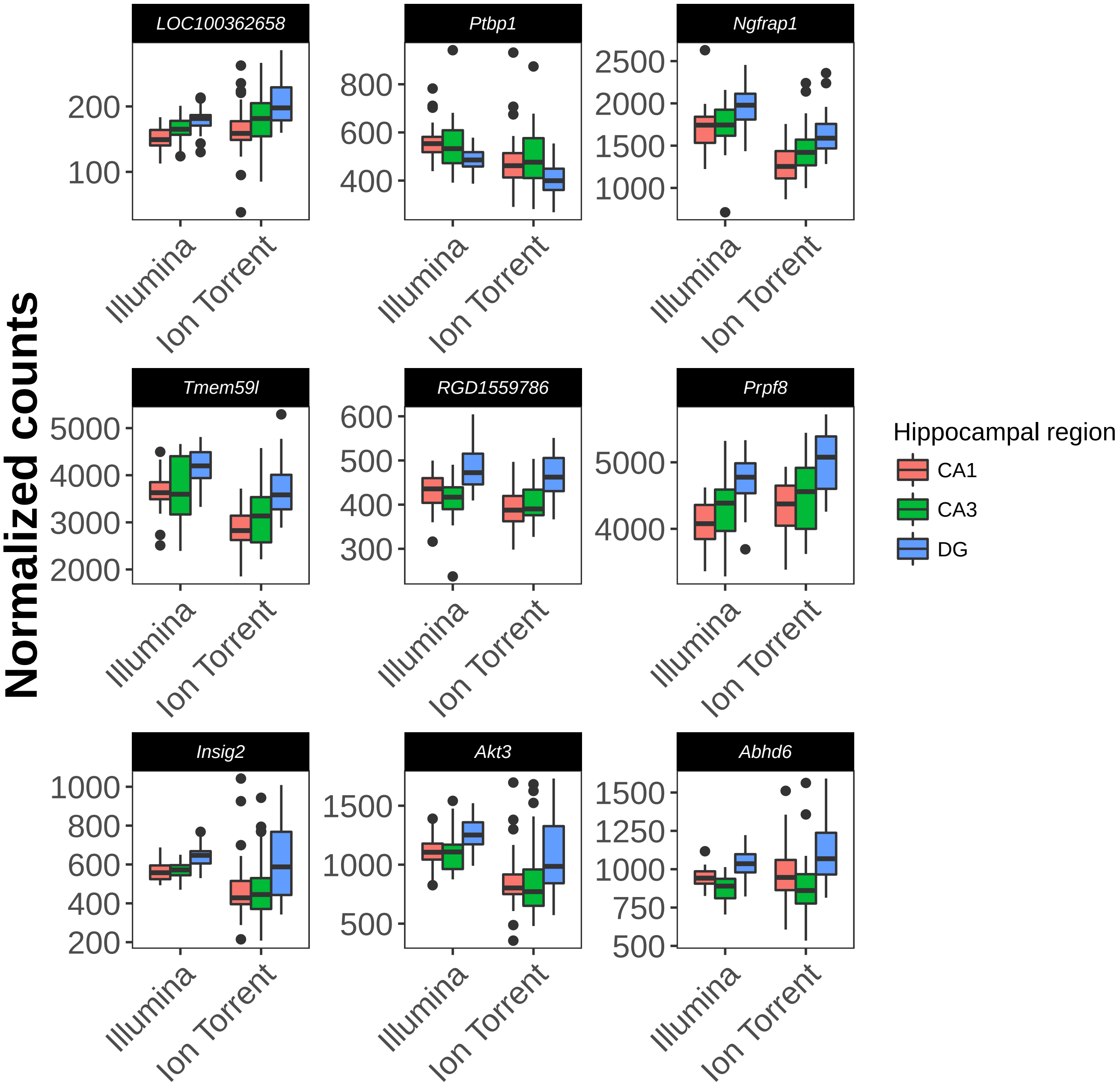
Normalized counts
LOC100362658
Ptbp1
Ngfrap1
Tmem59l
RGD1559786
Prpf8
Insig2
Akt3
Abhd6
Illumina
Ion Torrent
Hippocampal region
CA1
CA3
DG

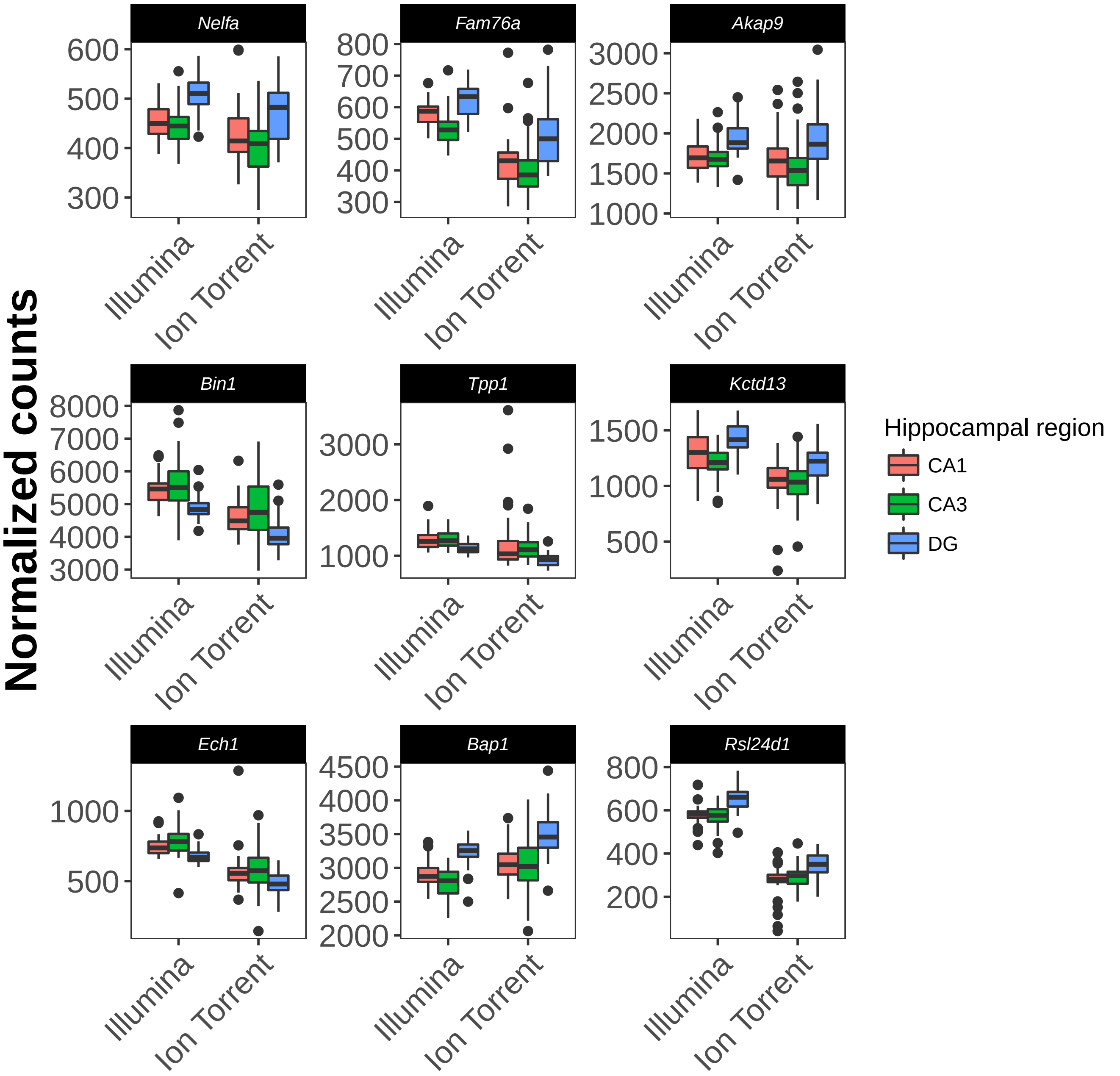

Normalized counts
Nelfa
Fam76a
Akap9
Bin1
Tpp1
Kctd13
Ech1
Bap1
Rsl24d1
Illumina
Ion Torrent
Hippocampal region
CA1
CA3
DG

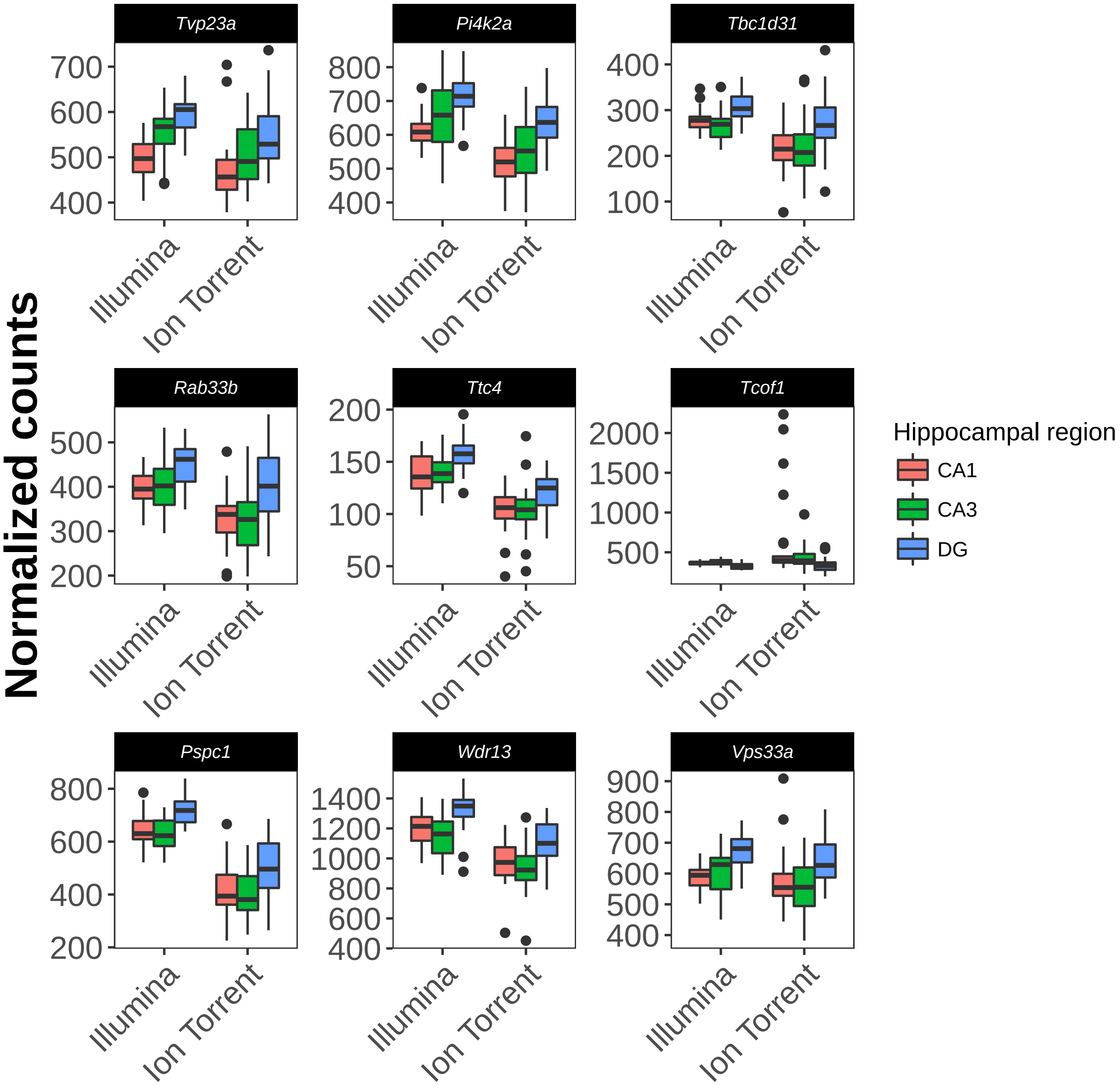

Tvp23a
Pi4k2a
Tbc1d31
Rab33b
Ttc4
Tcof1
Pspc1
Wdr13
Vps33a
Normalized counts
Illumina
Ion Torrent
Hippocampal region
CA1
CA3
DG

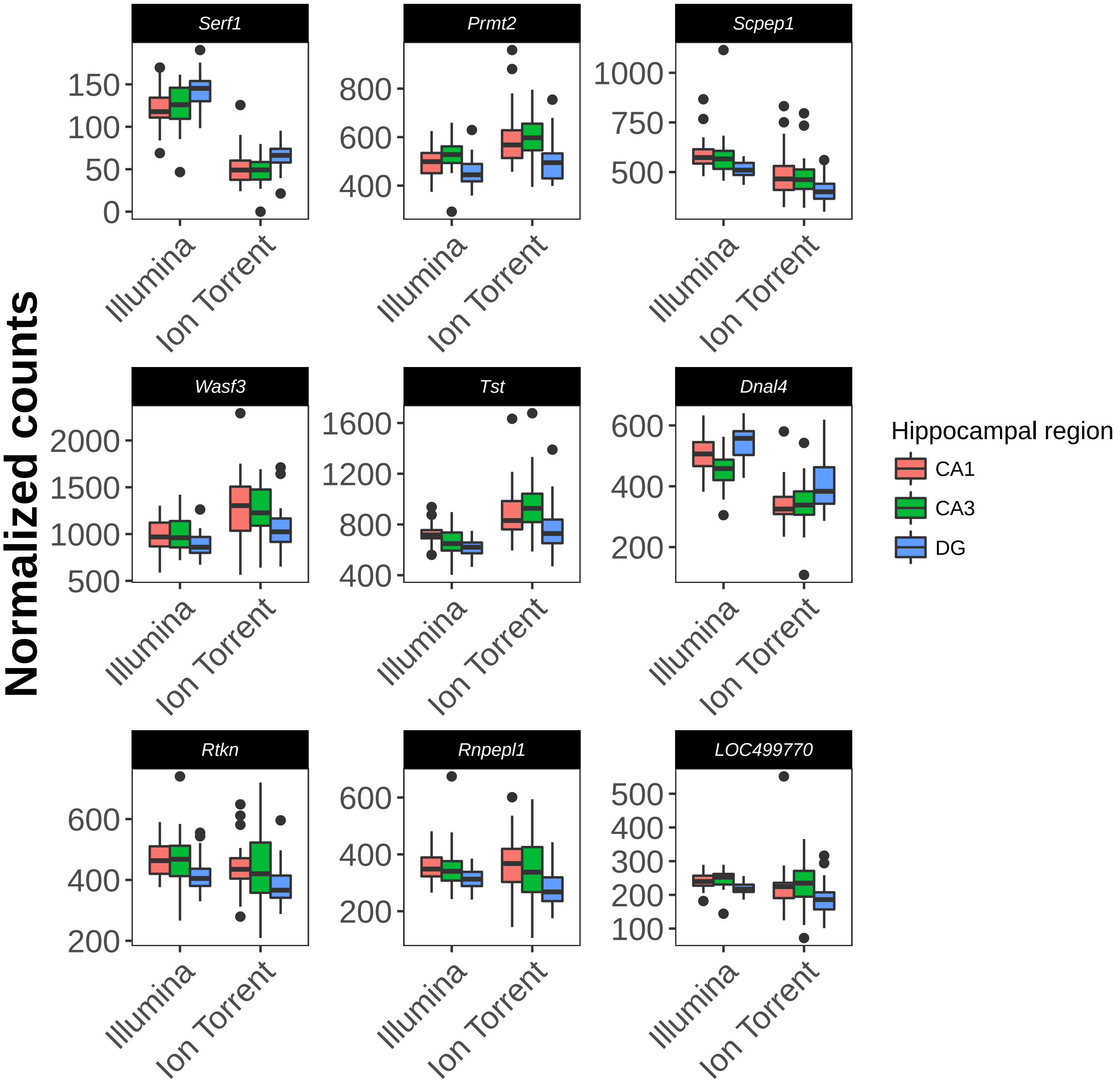

Serf1
Prmt2
Scpep1
Wasf3
Tst
Dnal4
Rtkn
Rnpepl1
LOC499770
Normalized counts
Illumina
Ion Torrent
Hippocampal region
CA1
CA3
DG

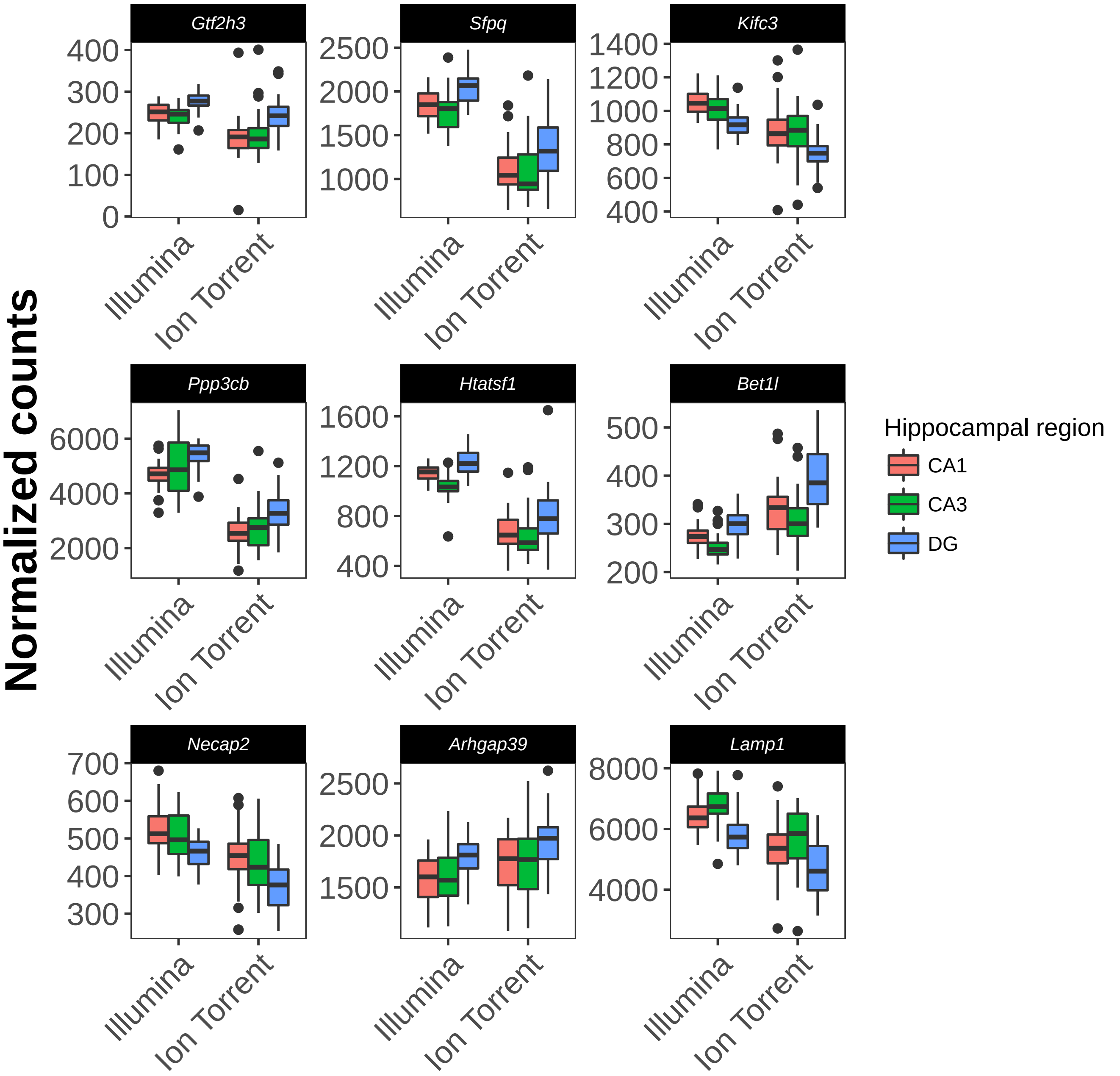
Normalized counts
Gtf2h3
Sfpq
Kifc3
Ppp3cb
Htatsf1
Bet1l
Necap2
Arhgap39
Lamp1
Illumina
Ion Torrent
Hippocampal region
CA1
CA3
DG

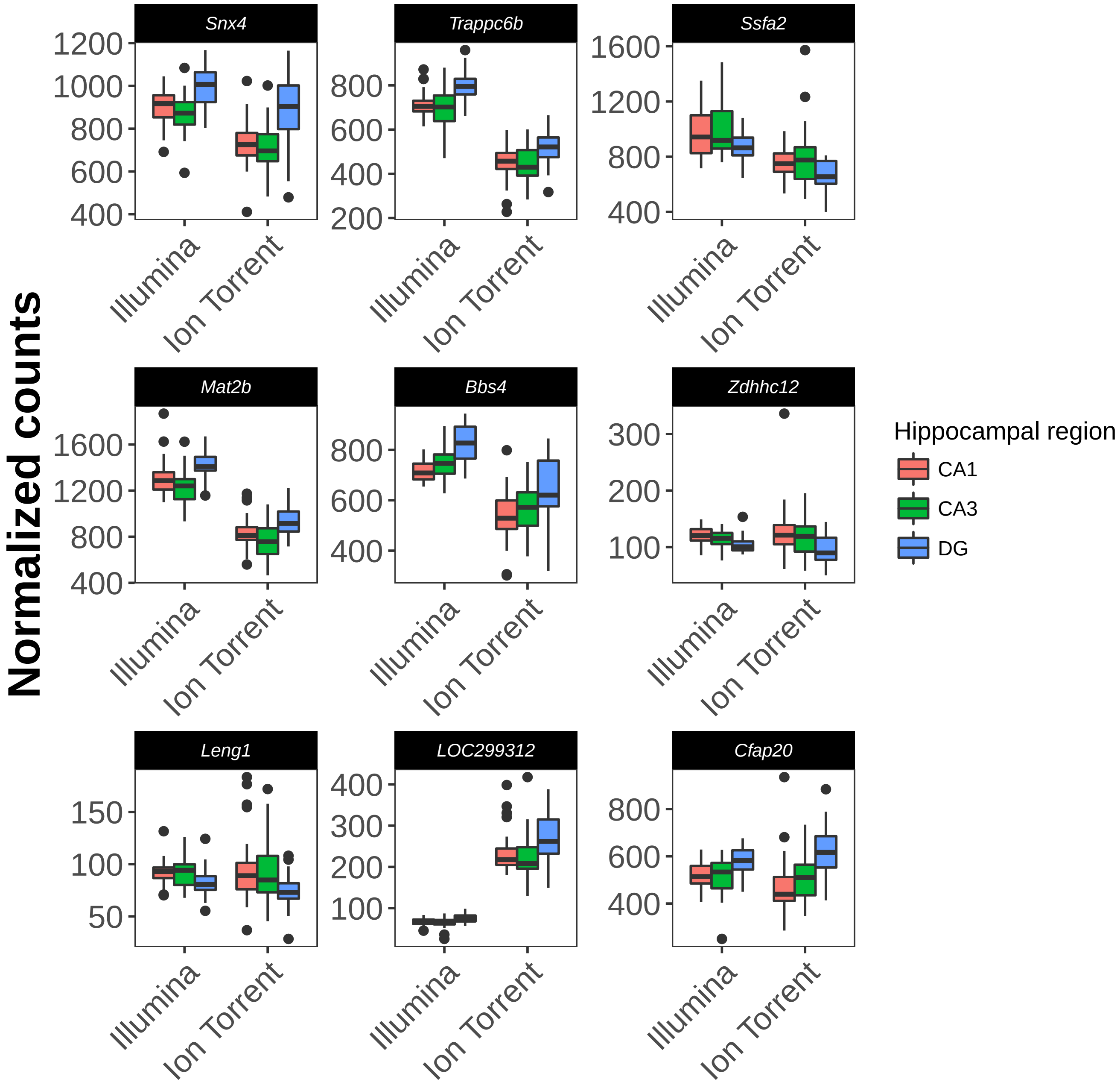

Normalized counts
Snx4
Trappc6b
Ssfa2
Mat2b
Bbs4
Zdhhc12
Leng1
LOC299312
Cfap20
Illumina
Ion Torrent
Hippocampal region
CA1
CA3
DG

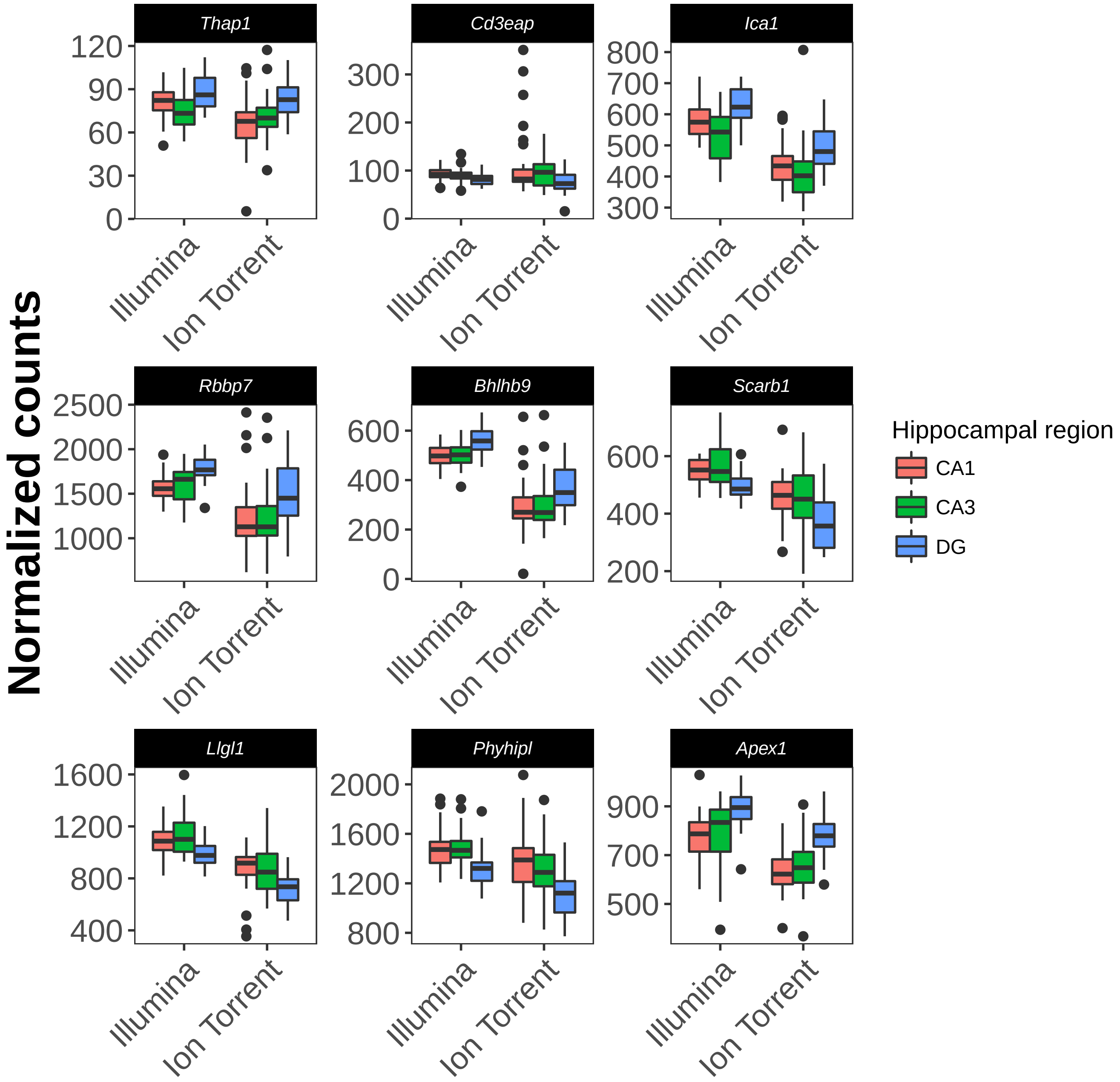
Normalized counts
Thap1
Cd3eap
Ica1
Rbbp7
Bhlhb9
Scarb1
Llgl1
Phyhipl
Apex1
Illumina
Ion Torrent
Hippocampal region
CA1
CA3
DG

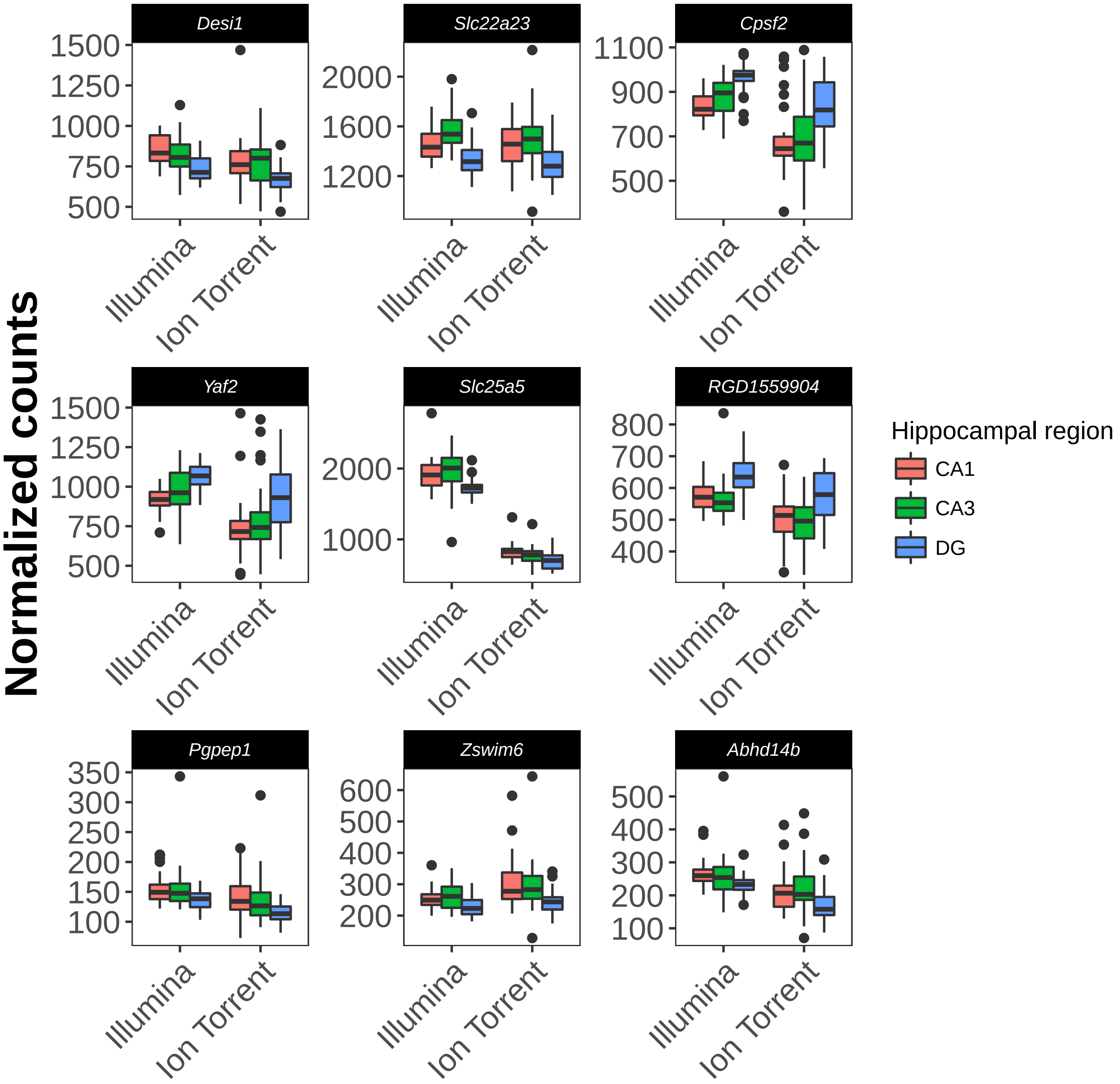

Normalized counts
Desi1
Slc22a23
Cpsf2
Yaf2
Slc25a5
RGD1559904
Pgpep1
Zswim6
Abhd14b
Illumina
Ion Torrent
Hippocampal region
CA1
CA3
DG

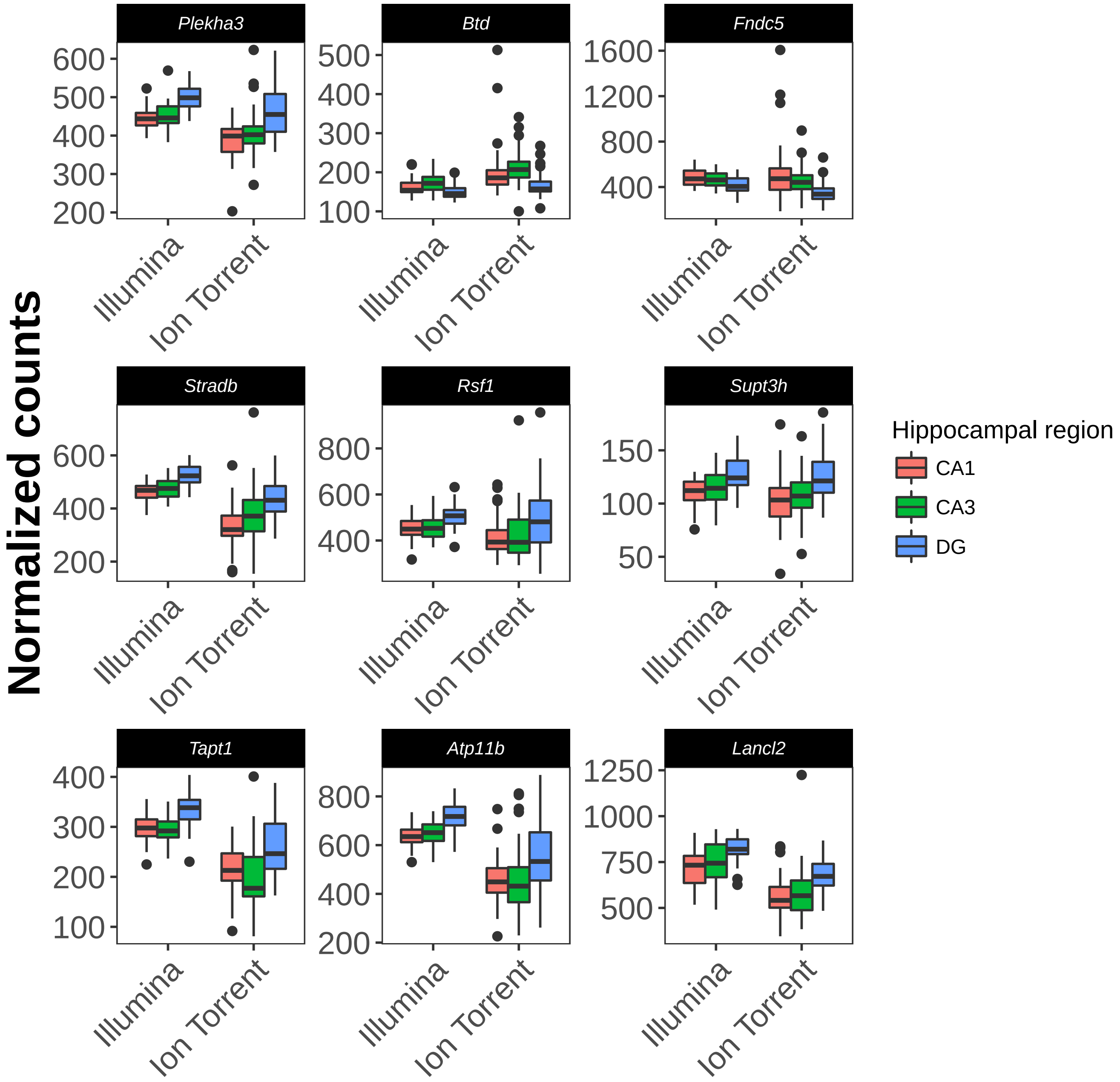
Plekha3
Btd
Fndc5
Stradb
Rsf1
Supt3h
Tapt1
Atp11b
Lancl2
Normalized counts
Illumina
Ion Torrent
Hippocampal region
CA1
CA3
DG

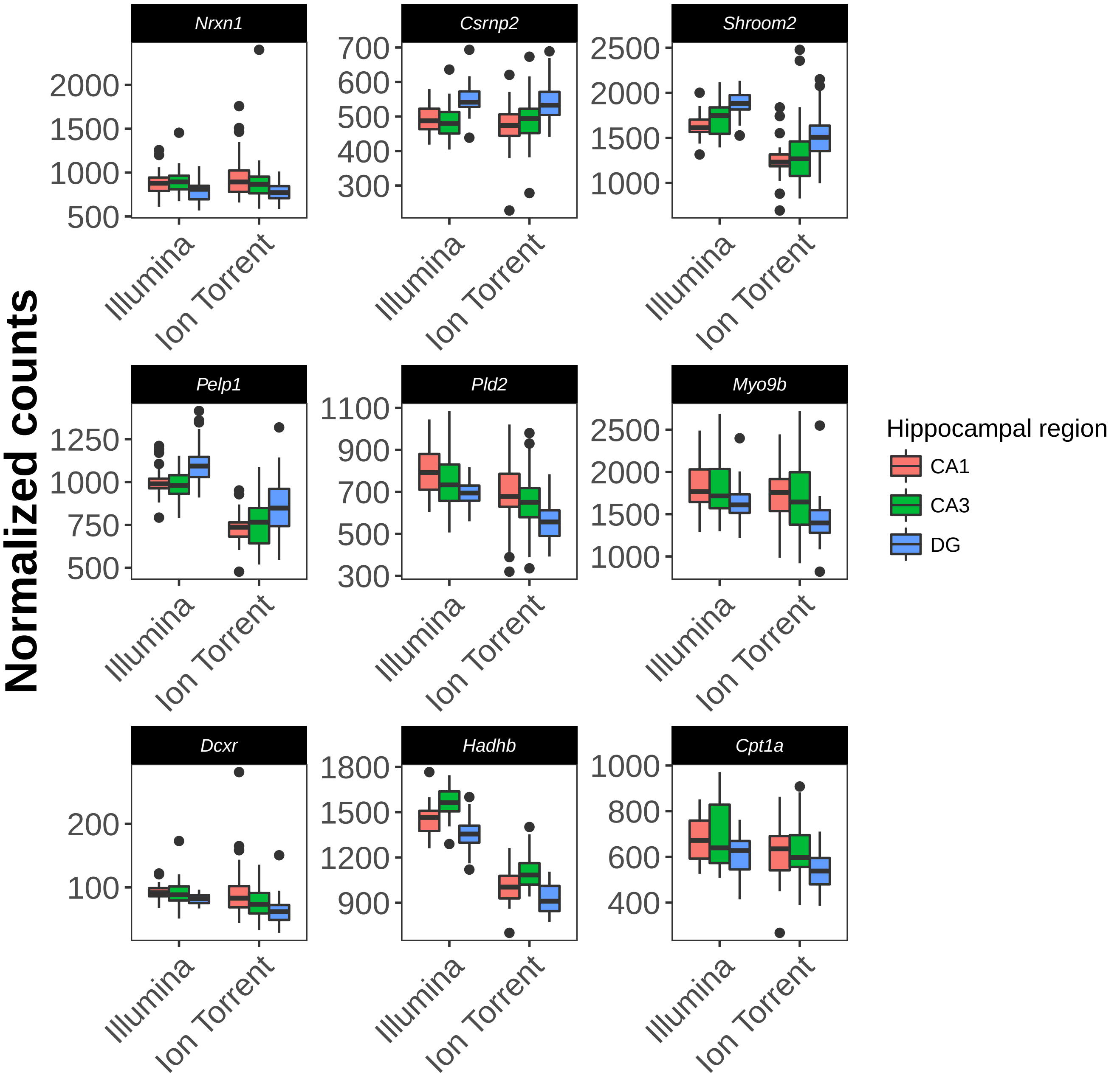

Normalized counts
Nrxn1
Csrnp2
Shroom2
Pelp1
Pld2
Myo9b
Dcxr
Hadhb
Cpt1a
Illumina
Ion Torrent
Hippocampal region
CA1
CA3
DG

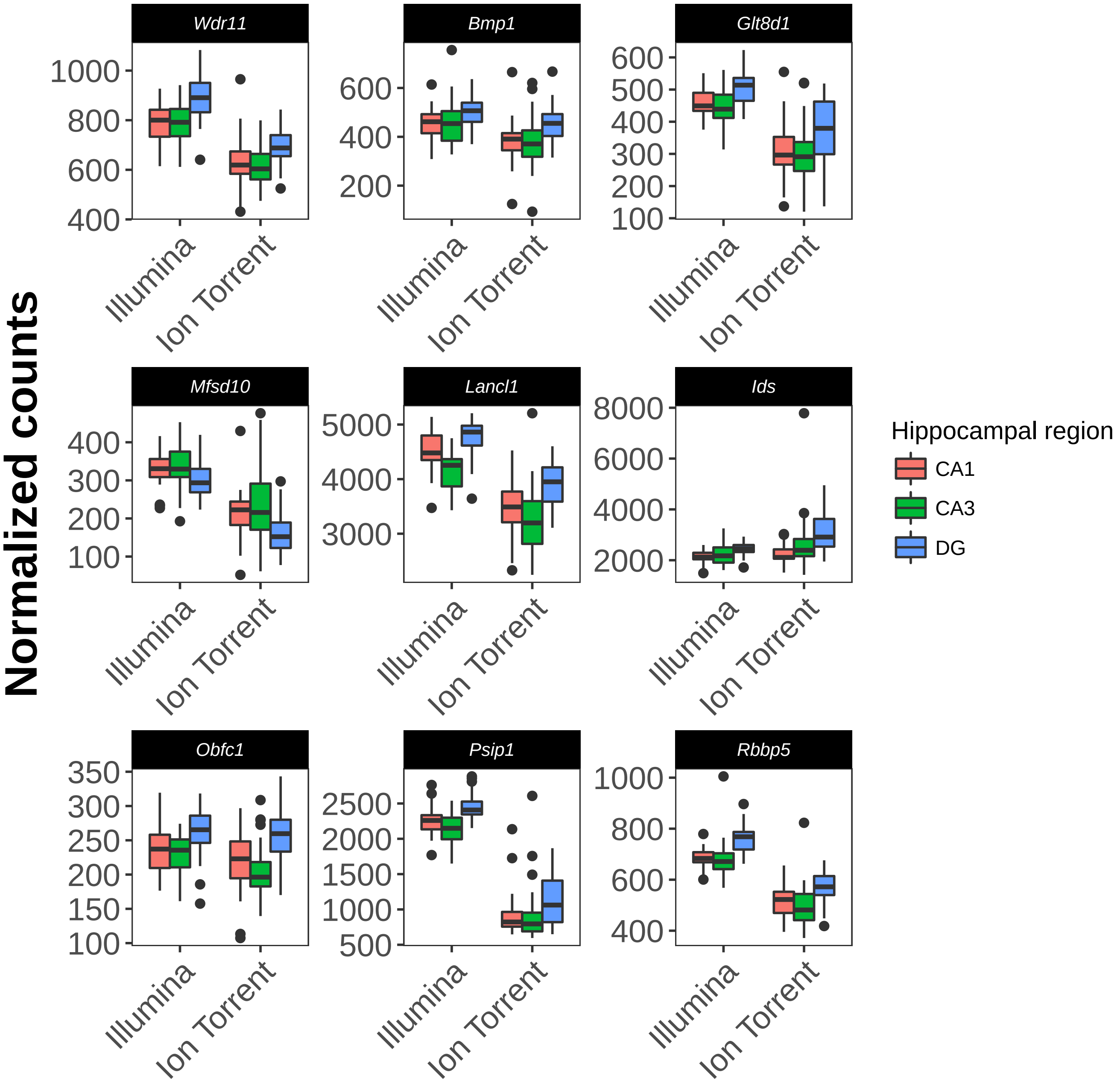
Normalized counts
Wdr11
Bmp1
Glt8d1
Mfsd10
Lancl1
Ids
Obfc1
Psip1
Rbbp5
Illumina
Ion Torrent
Hippocampal region
CA1
CA3
DG

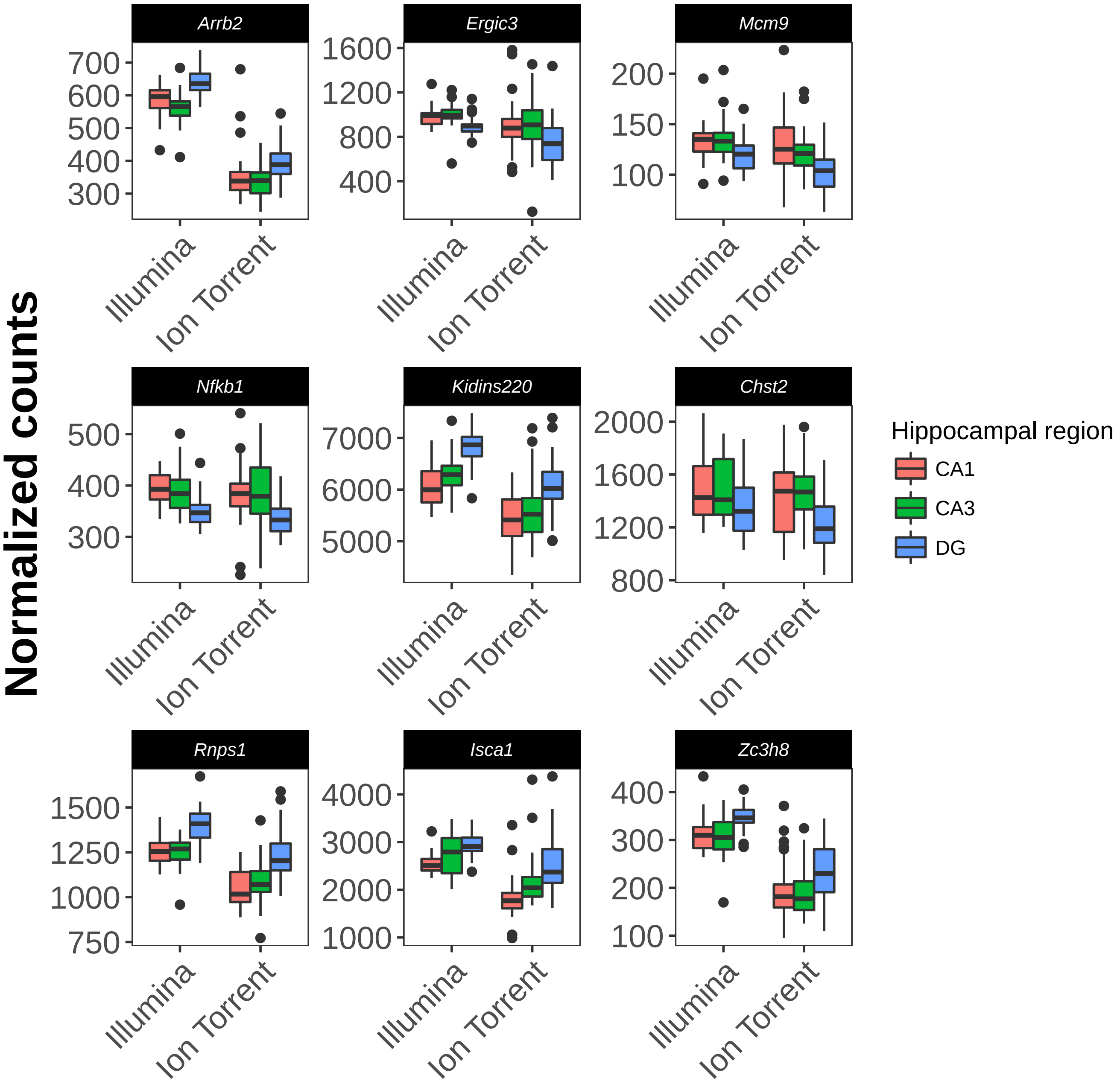

Normalized counts
Arrb2
Ergic3
Mcm9
Nfkb1
Kidins220
Chst2
Rnps1
Isca1
Zc3h8
Illumina
Ion Torrent
Hippocampal region
CA1
CA3
DG

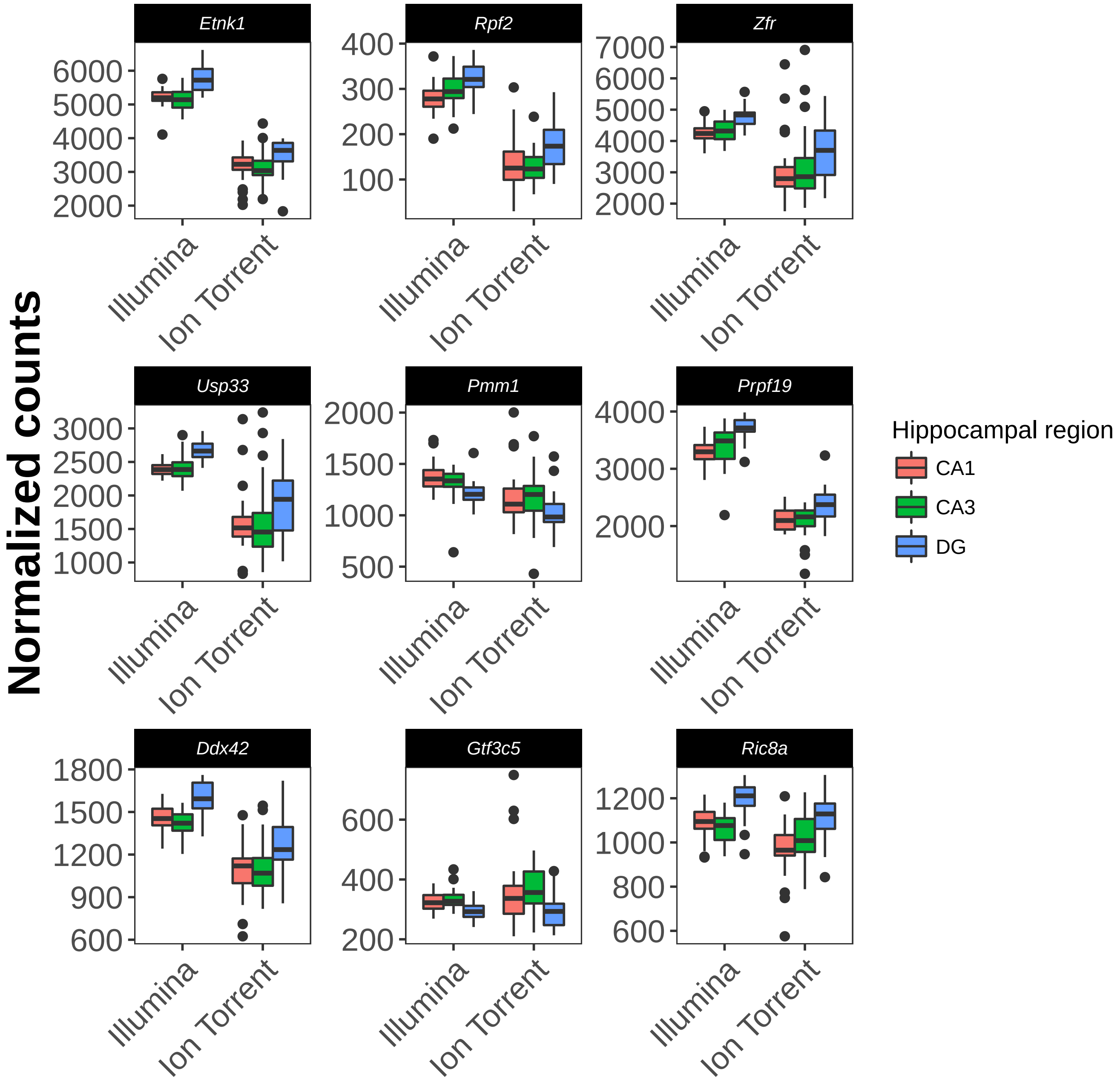
Normalized counts
Etnk1
Rpf2
Zfr
Usp33
Pmm1
Prpf19
Ddx42
Gtf3c5
Ric8a
Illumina
Ion Torrent
Hippocampal region
CA1
CA3
DG

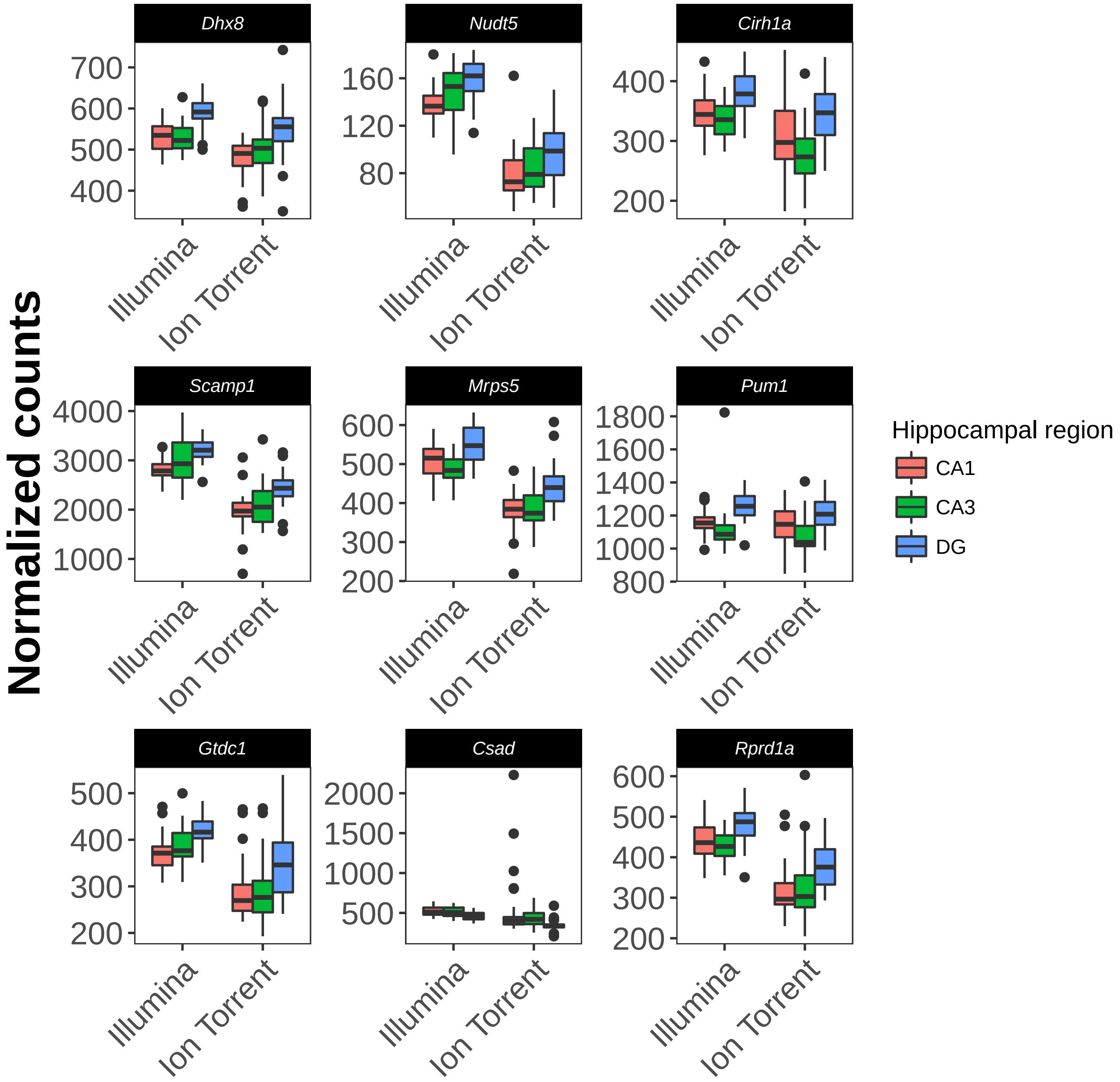
Normalized counts
Dhx8
Nudt5
Cirh1a
Scamp1
Mrps5
Pum1
Gtdc1
Csad
Rprd1a
Illumina
Ion Torrent
Hippocampal region
CA1
CA3
DG

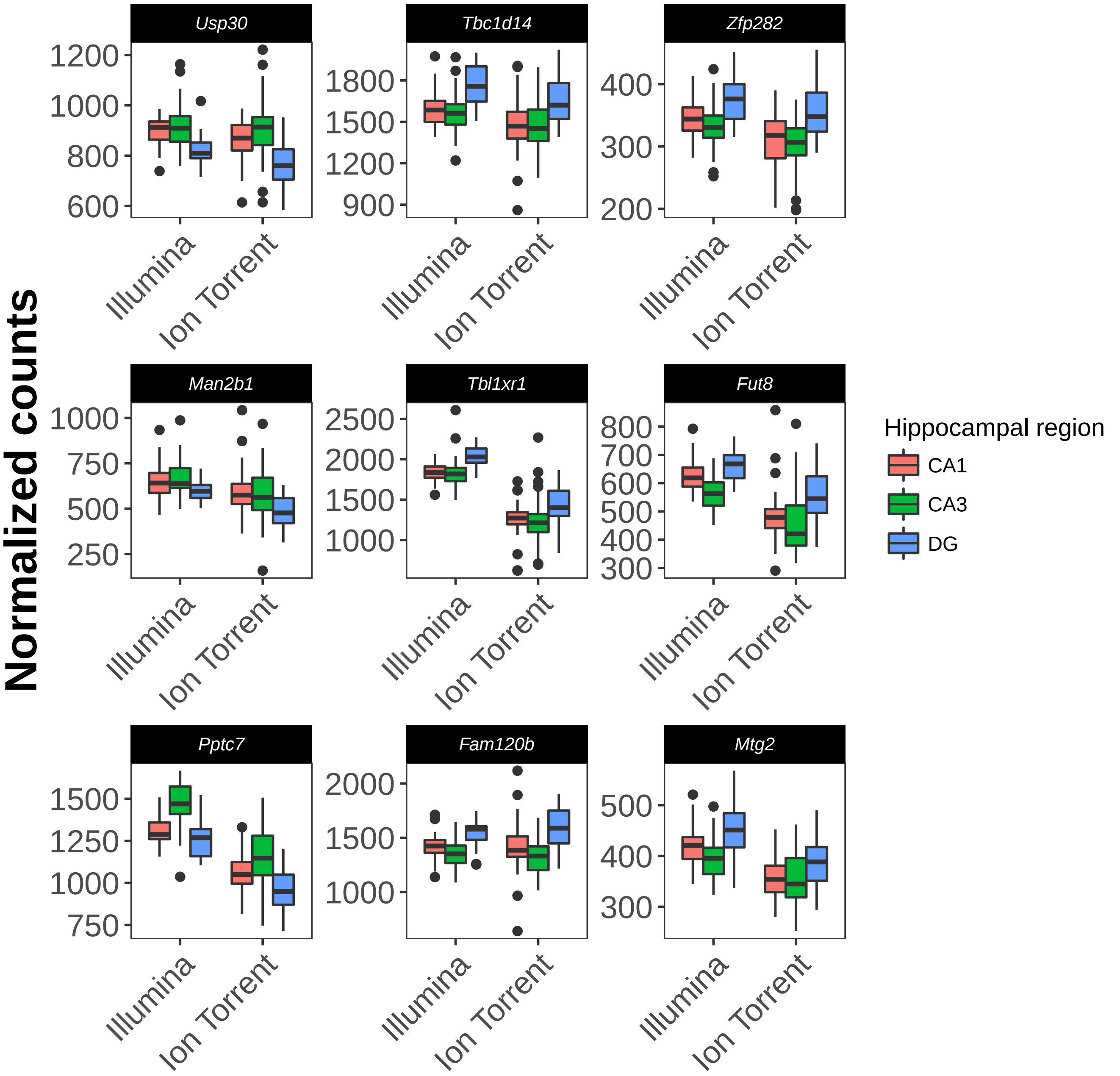

Normalized counts
Usp30
Tbc1d14
Zfp282
Man2b1
Tbl1xr1
Fut8
Pptc7
Fam120b
Mtg2
Illumina
Ion Torrent
Hippocampal region
CA1
CA3
DG

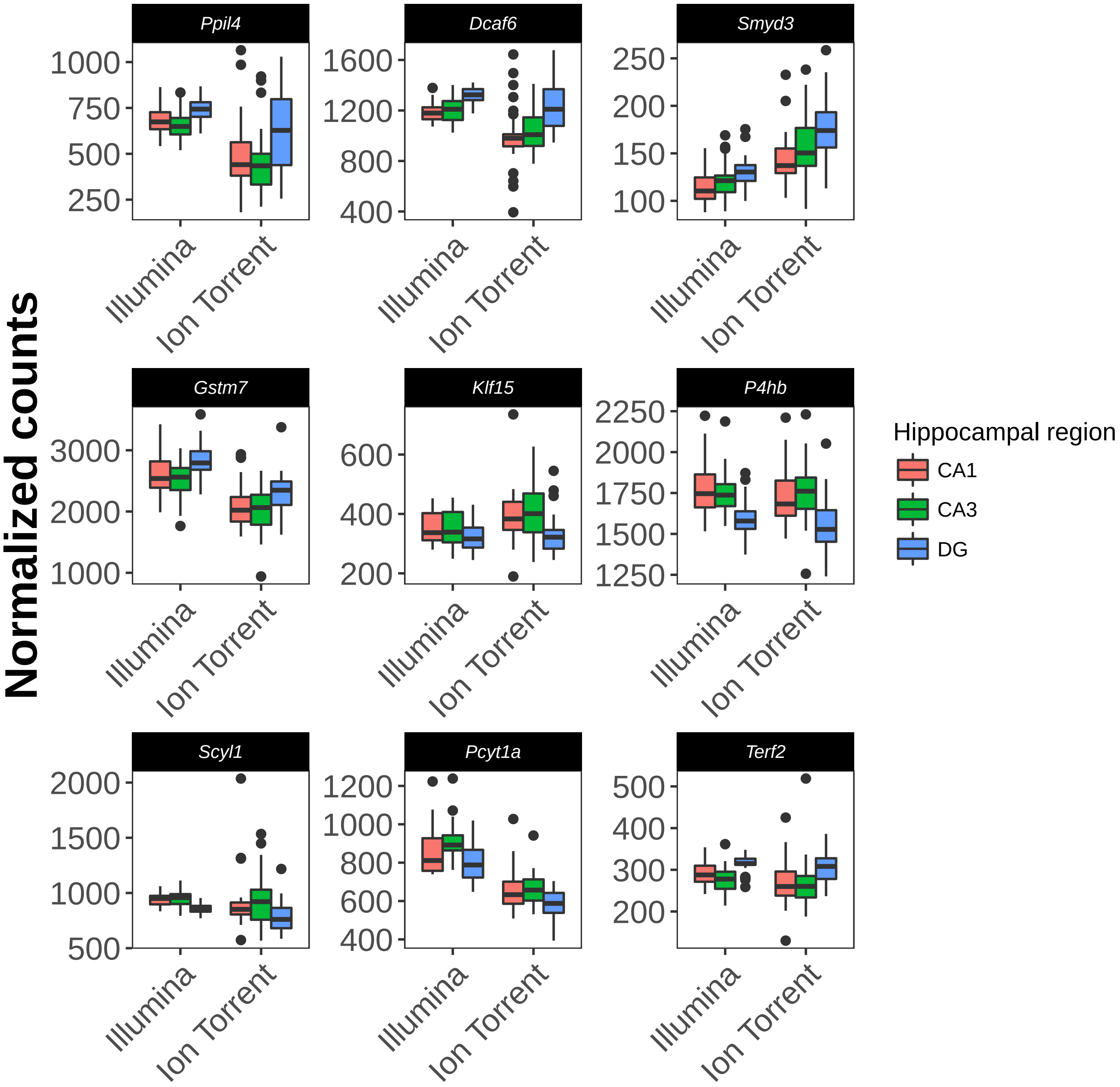
Normalized counts
Ppil4
Dcaf6
Smyd3
Gstm7
Klf15
P4hb
Scyl1
Pcyt1a
Terf2
Illumina
Ion Torrent
Hippocampal region
CA1
CA3
DG

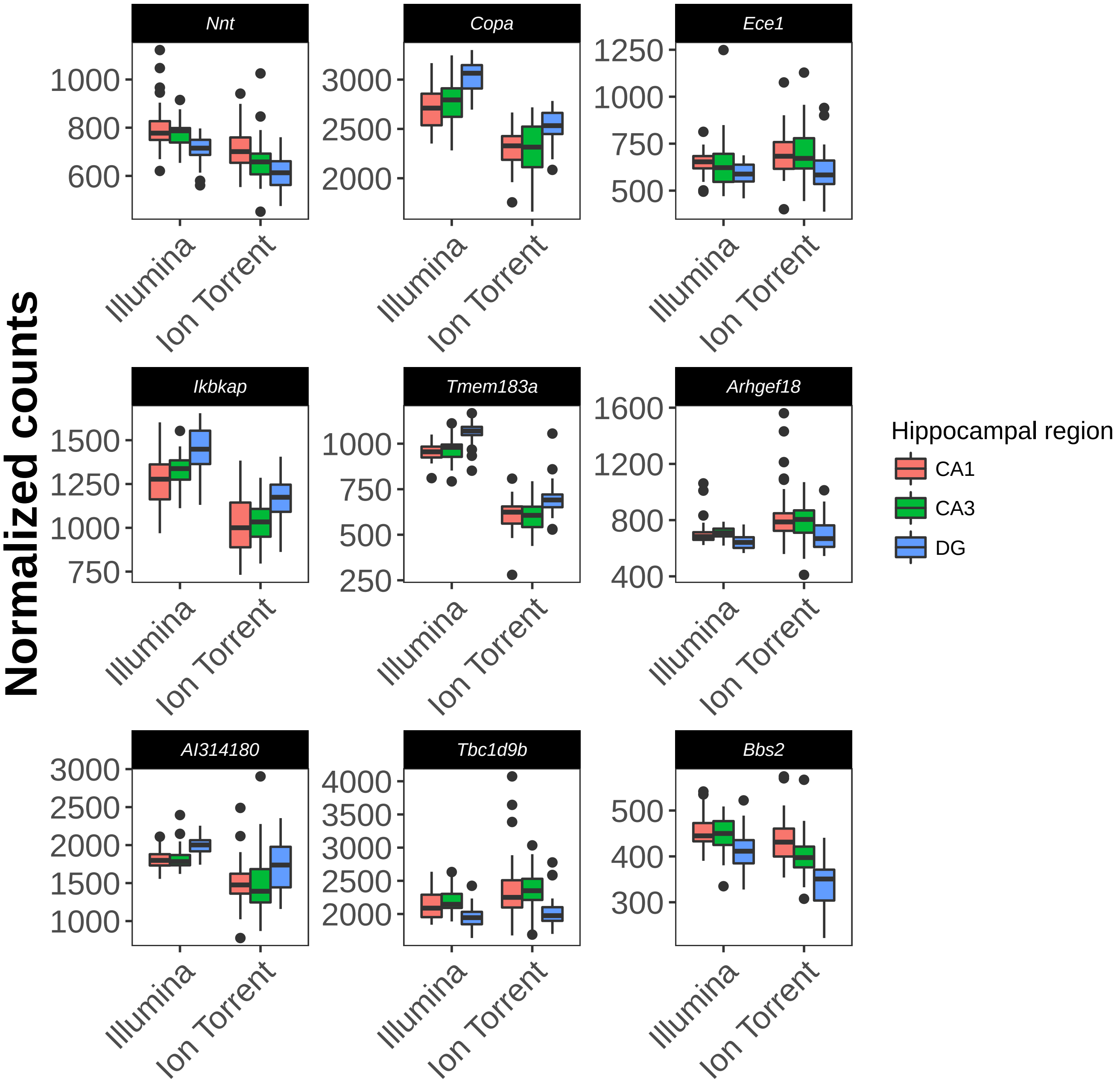
Normalized counts
Nnt
Copa
Ece1
Ikbkap
Tmem183a
Arhgef18
AI314180
Tbc1d9b
Bbs2
Illumina
Ion Torrent
Hippocampal region
CA1
CA3
DG

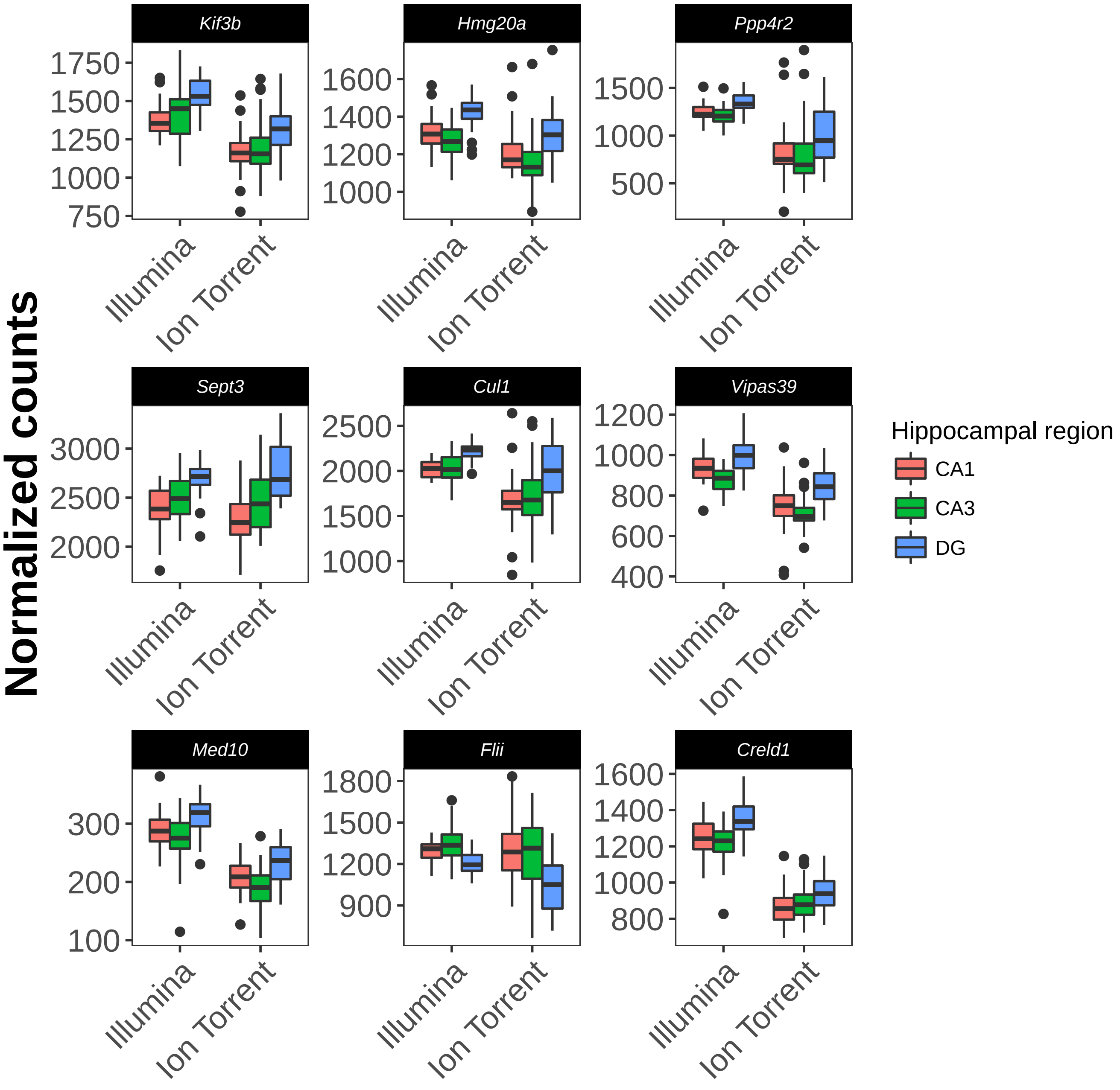

Normalized counts
Kif3b
Hmg20a
Ppp4r2
Sept3
Cul1
Vipas39
Med10
Flii
Creld1
Illumina
Ion Torrent
Hippocampal region
CA1
CA3
DG

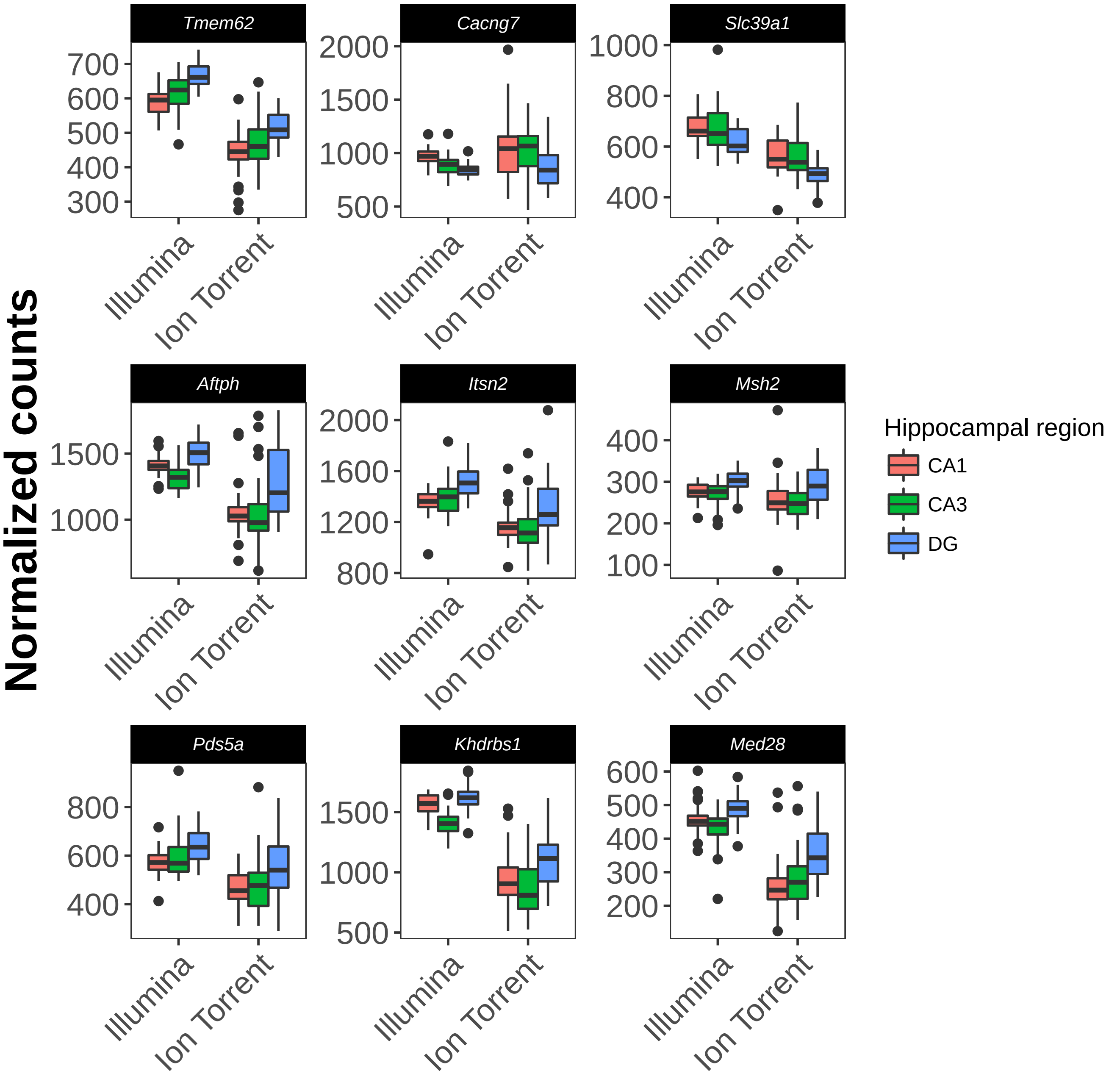
Normalized counts
Tmem62
Cacng7
Slc39a1
Aftph
Itsn2
Msh2
Pds5a
Khdrbs1
Med28
Illumina
Ion Torrent
Hippocampal region
CA1
CA3
DG

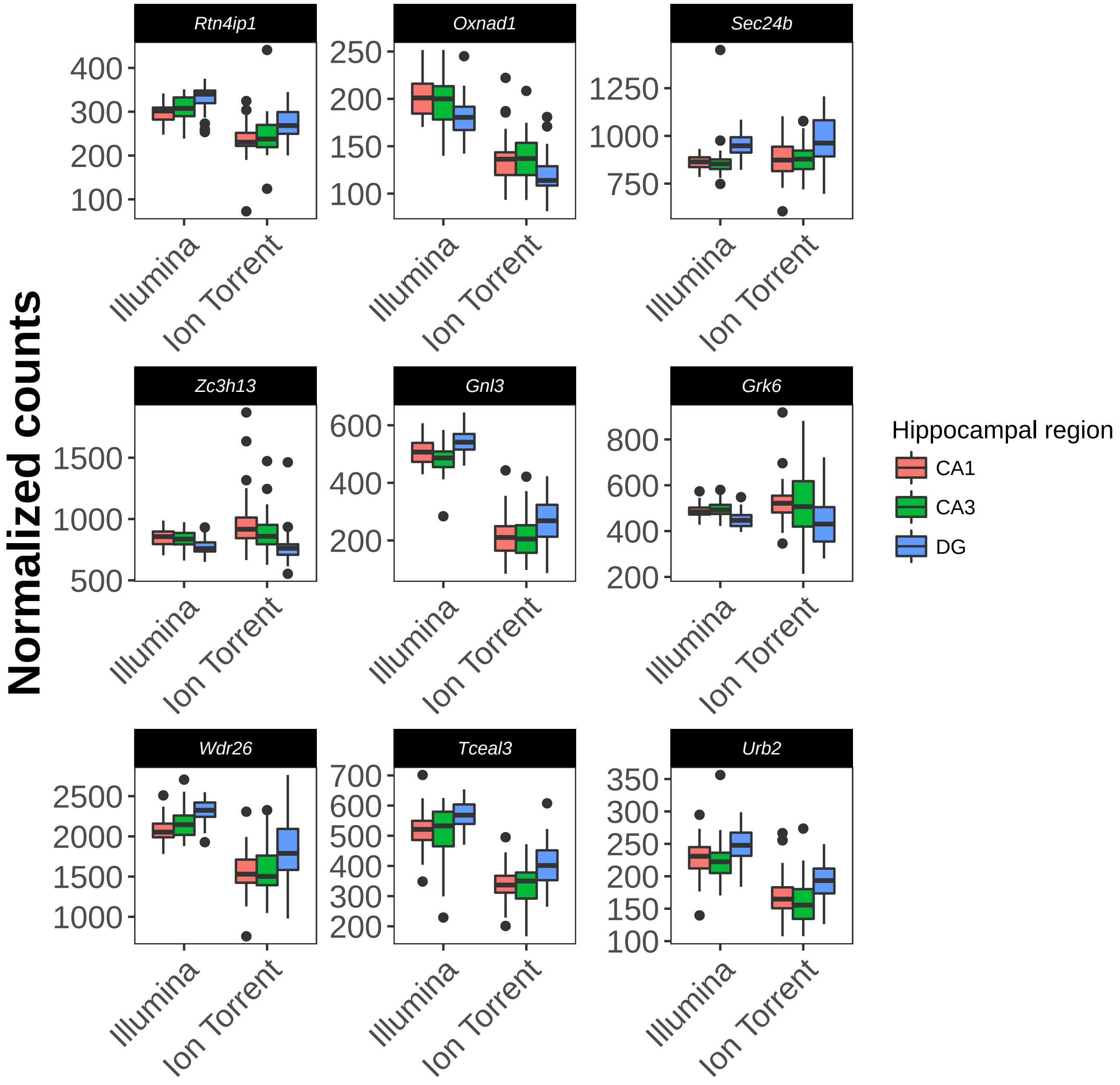
Normalized counts
Rtn4ip1
Oxnad1
Sec24b
Zc3h13
Gnl3
Grk6
Wdr26
Tceal3
Urb2
Illumina
Ion Torrent
Hippocampal region
CA1
CA3
DG

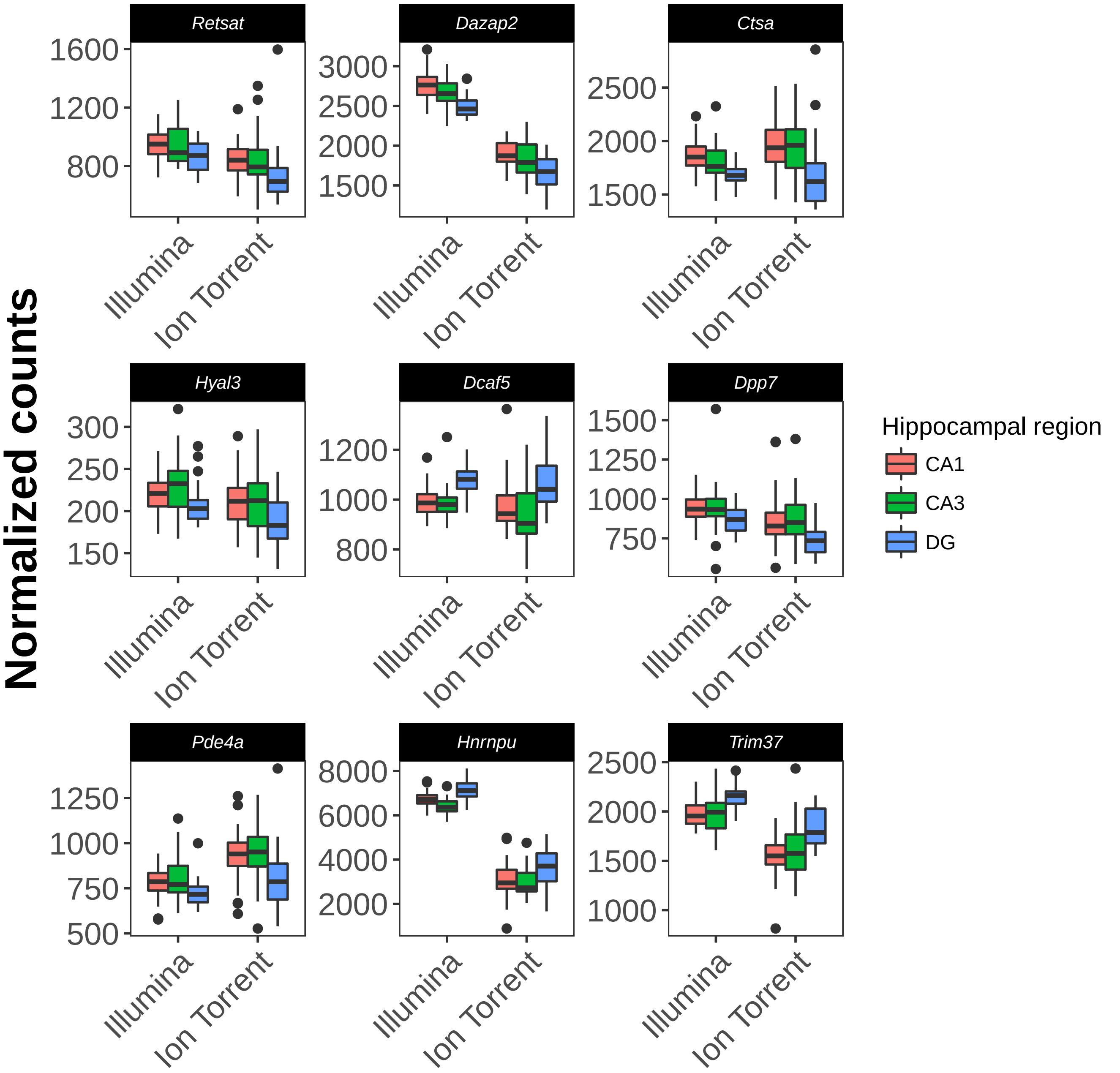
Normalized counts
Retsat
Dazap2
Ctsa
Hyal3
Dcaf5
Dpp7
Pde4a
Hnrnpu
Trim37
Illumina
Ion Torrent
Hippocampal region
CA1
CA3
DG

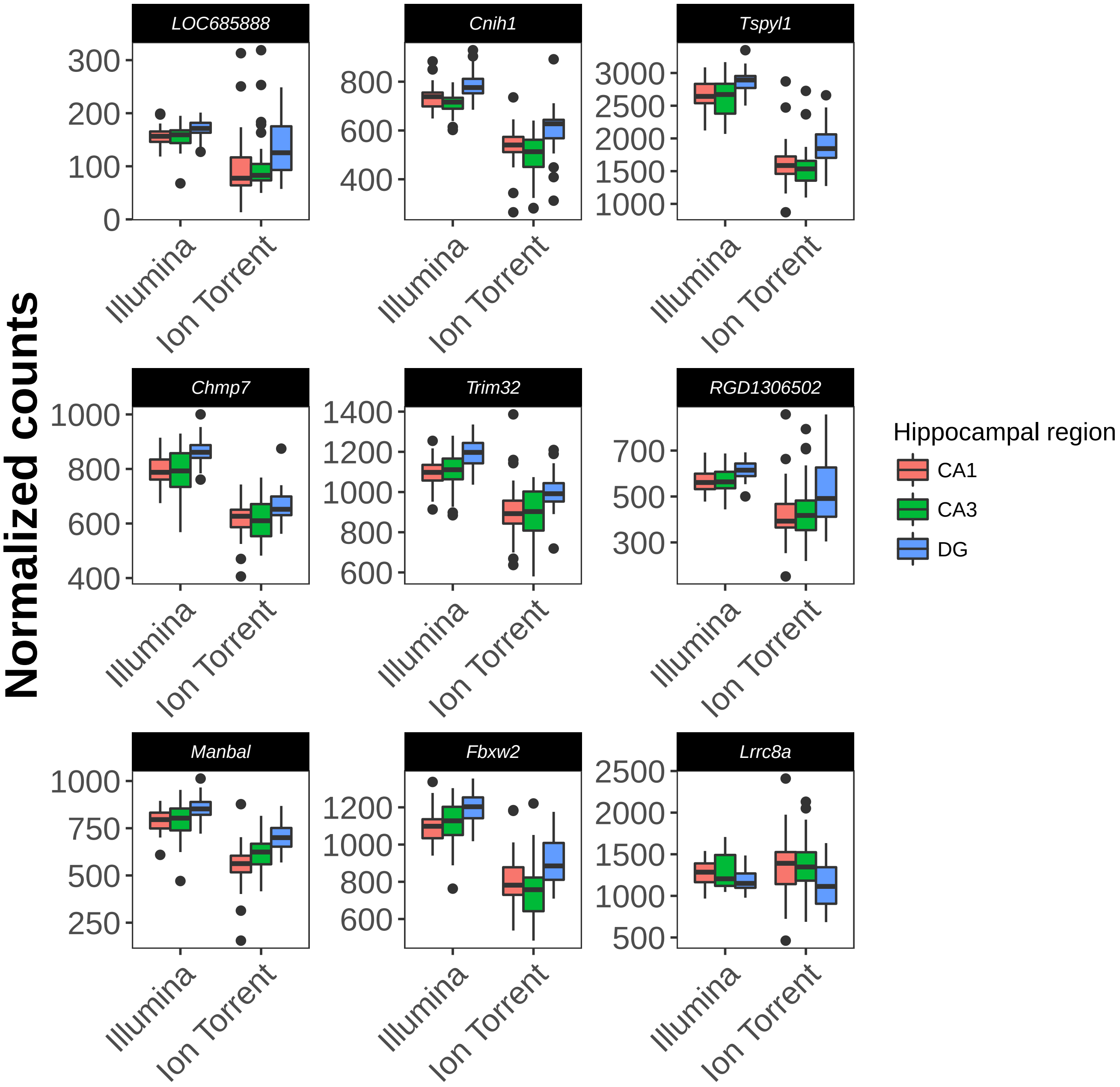

Normalized counts
LOC685888
Cnih1
Tspyl1
Chmp7
Trim32
RGD1306502
Manbal
Fbxw2
Lrrc8a
Illumina
Ion Torrent
Hippocampal region
CA1
CA3
DG

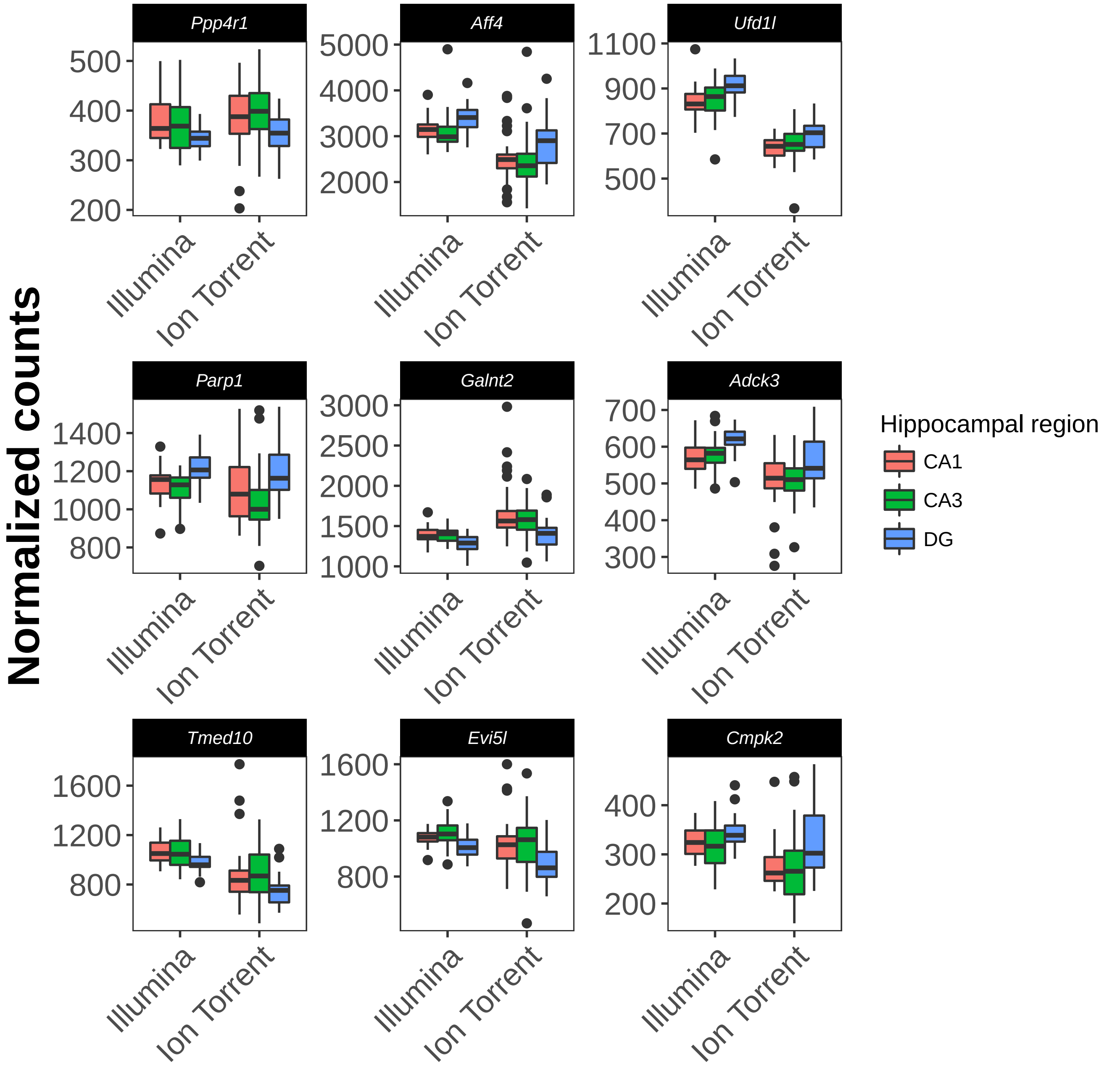
Normalized counts
Ppp4r1
Aff4
Ufd1l
Parp1
Galnt2
Adck3
Tmed10
Evi5l
Cmpk2
500
400
300
200
5000
4000
3000
2000
1100
900
700
500
1400
1200
1000
800
3000
2500
2000
1500
1000
700
600
500
400
300
1600
1200
800
1600
1200
800
400
300
200
Illumina
Ion Torrent
Hippocampal region
CA1
CA3
DG

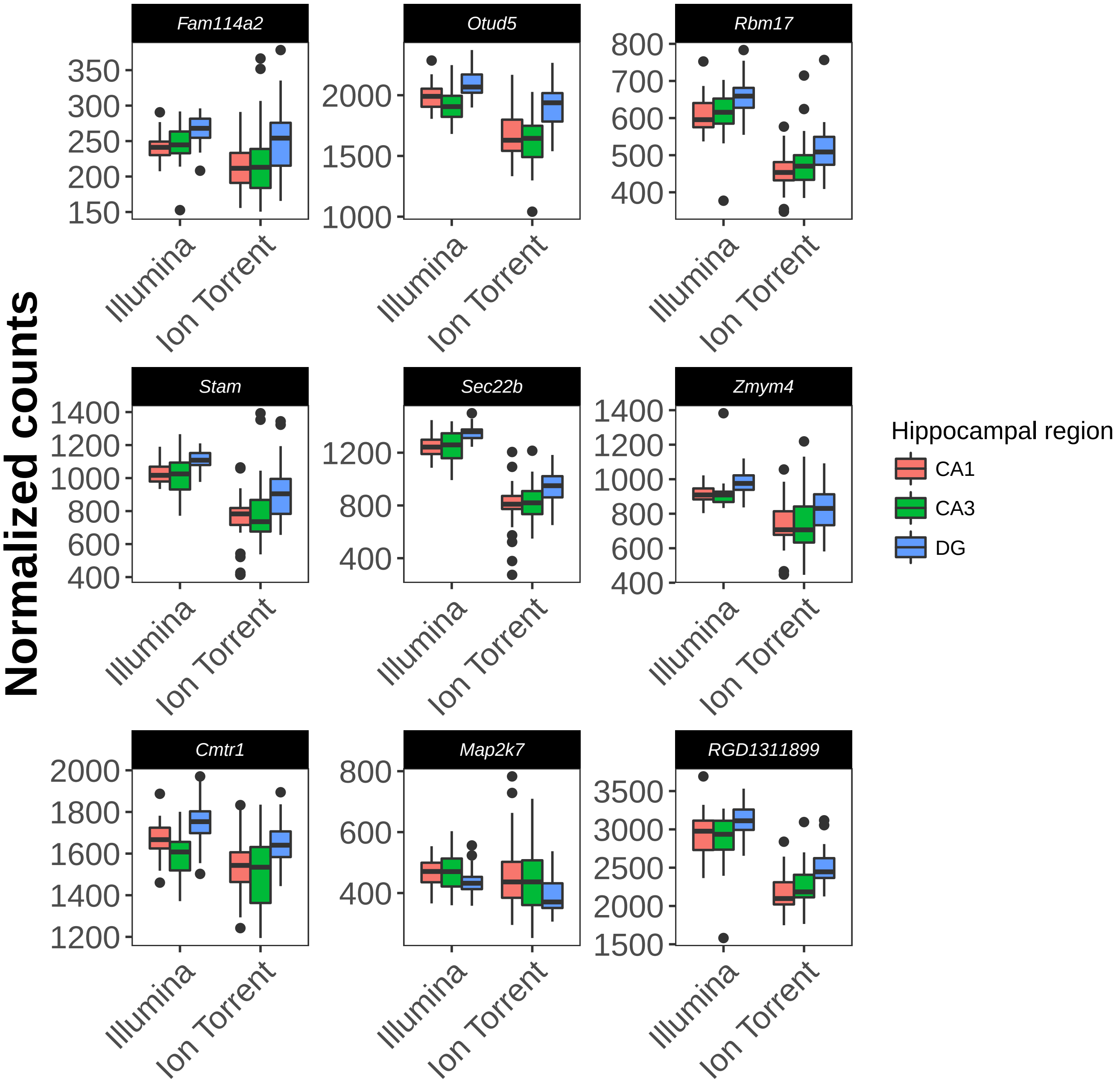
Normalized counts
Fam114a2
Otud5
Rbm17
Stam
Sec22b
Zmym4
Cmtr1
Map2k7
RGD1311899
Illumina
Ion Torrent
Hippocampal region
CA1
CA3
DG

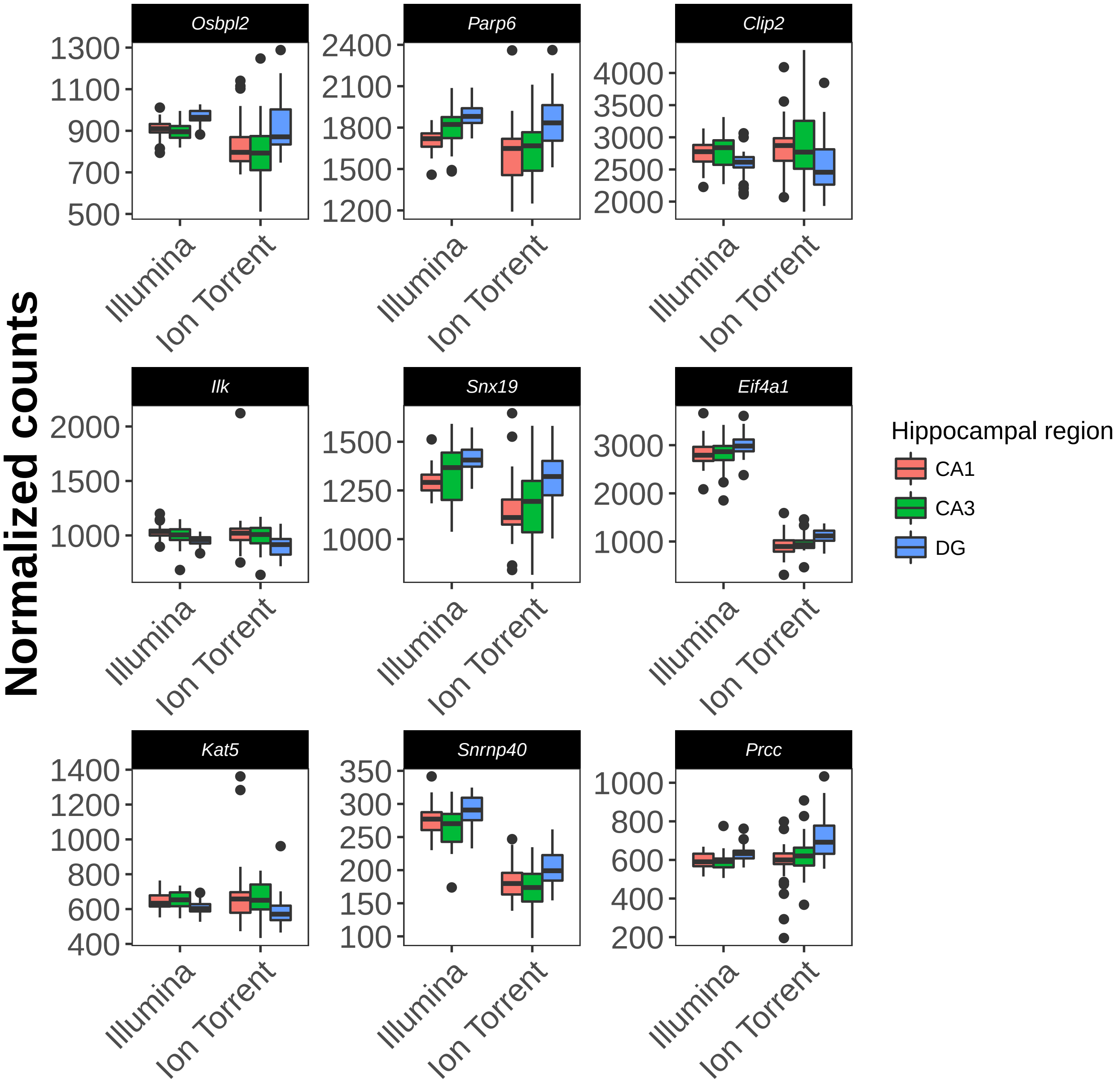

Normalized counts
Osbpl2
Parp6
Clip2
Ilk
Snx19
Eif4a1
Kat5
Snrnp40
Prcc
Illumina
Ion Torrent
Hippocampal region
CA1
CA3
DG

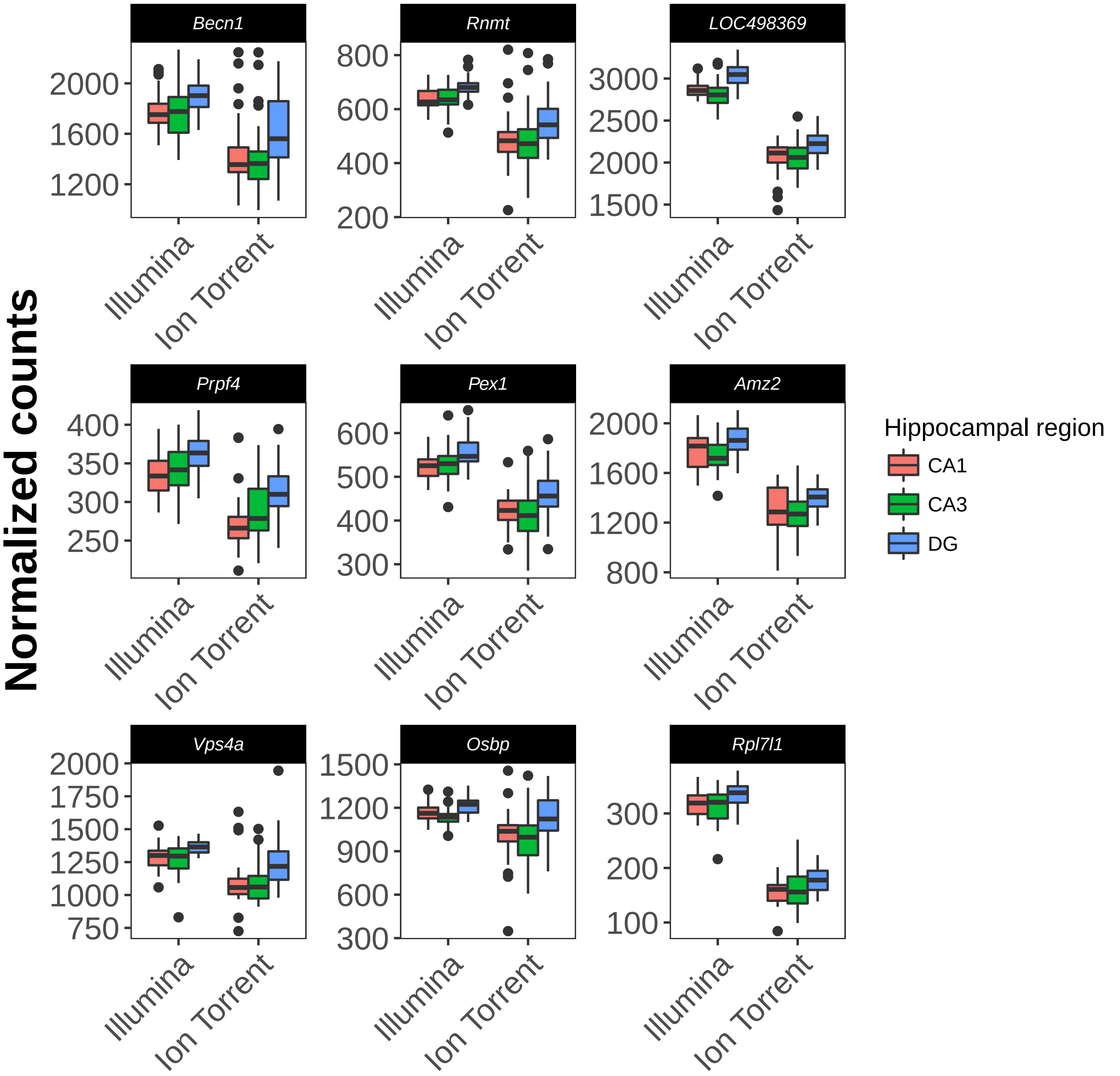
Normalized counts
Becn1
Rnmt
LOC498369
Prpf4
Pex1
Amz2
Vps4a
Osbp
Rpl7l1
Illumina
Ion Torrent
Hippocampal region
CA1
CA3
DG

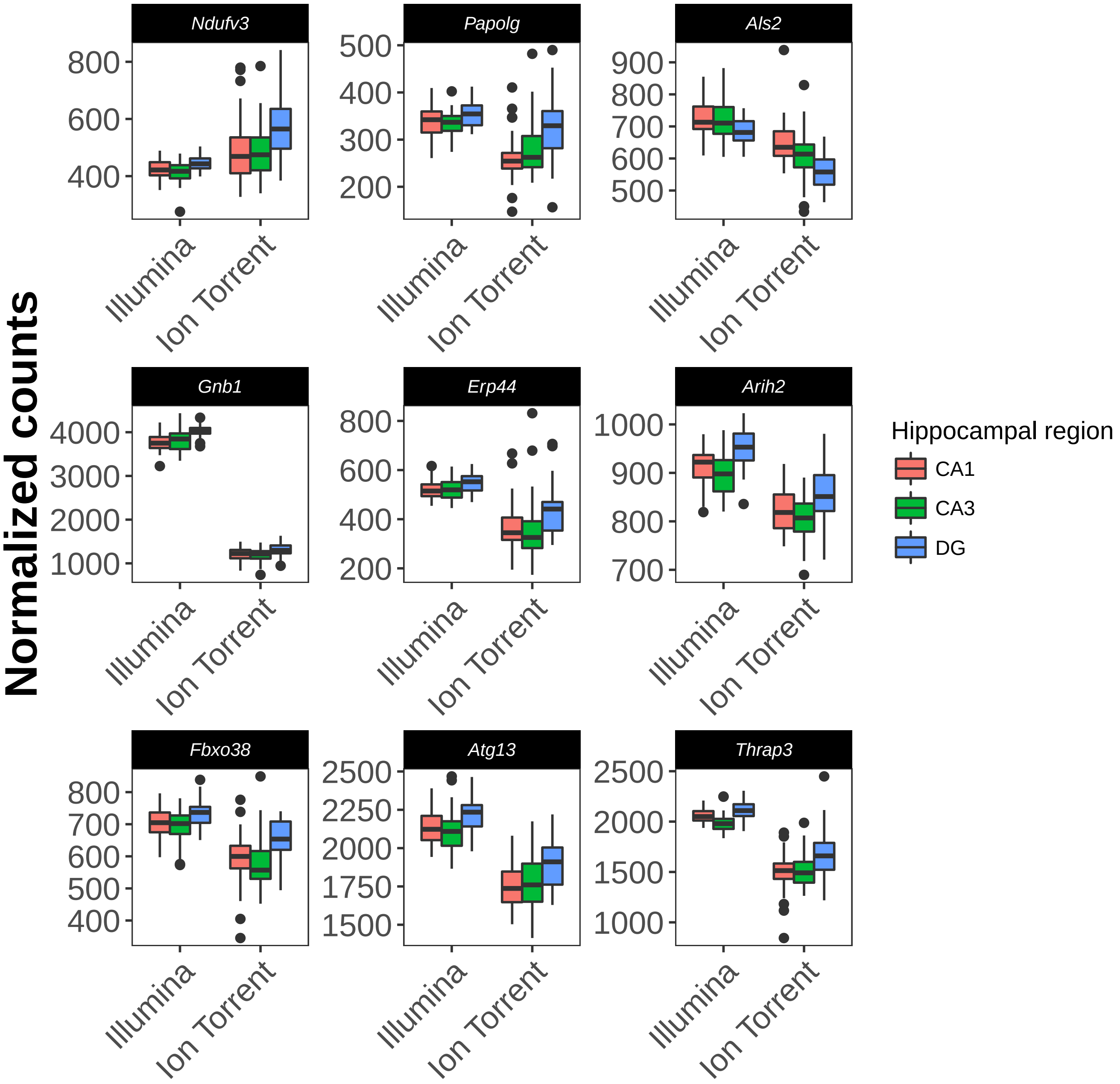
Normalized counts
Ndufv3
Papolg
Als2
Gnb1
Erp44
Arih2
Fbxo38
Atg13
Thrap3
Illumina
Ion Torrent
Hippocampal region
CA1
CA3
DG

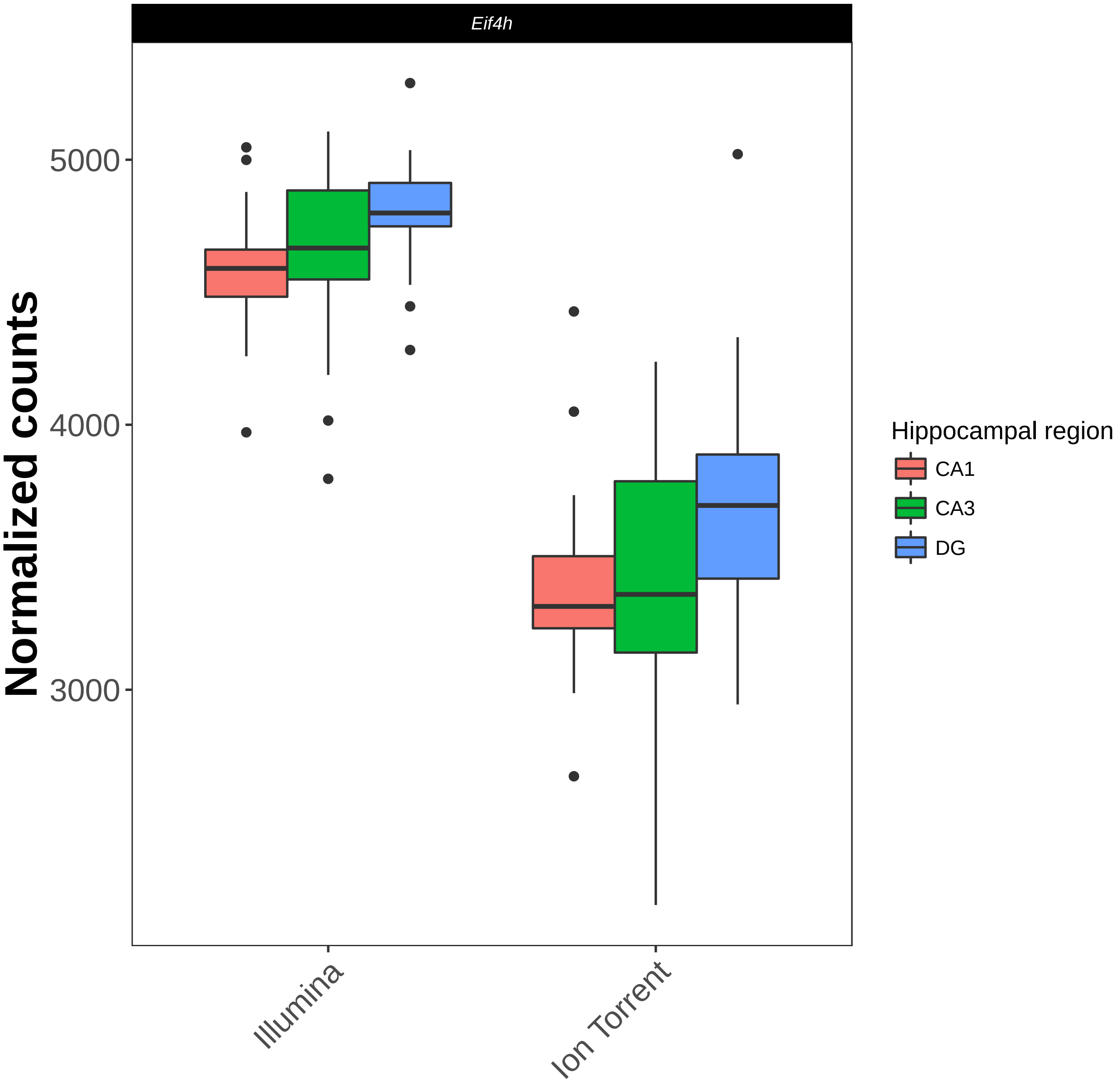

Eif4h
Normalized counts
5000
4000
3000
Illumina
Ion Torrent
Hippocampal region
CA1
CA3
DG
